# Analysis of the Efficacy and Pharmacological Mechanisms of Action of Zhenren Yangzang Decoction on Ulcerative Colitis Using Meta-Analysis and Network Pharmacology

**DOI:** 10.1155/2021/4512755

**Published:** 2021-12-28

**Authors:** Guosheng Xing, Yufeng Zhang, Xinlin Wu, Hua Wang, Yan Liu, Zhen Zhang, Mingxing Hou, Haibing Hua

**Affiliations:** ^1^Nanjing University of Chinese Medicine, Nanjing, Jiangsu 210008, China; ^2^Department of General Surgery, The Affiliated Hospital of Inner Mongolia Medical University, Hohhot, Inner Mongolia 010050, China; ^3^Department of Respiratory Medicine, Jiangyin Hospital of Traditional Chinese Medicine, Jiangyin Hospital Affiliated to Nanjing University of Chinese Medicine, Jiangyin, Jiangsu 214400, China; ^4^Department of Traditional Chinese Medicine, The Affiliated Hospital of Inner Mongolia Medical University, Hohhot, Inner Mongolia 010050, China; ^5^Department of Gastroenterology, Jiangyin Hospital of Traditional Chinese Medicine, Jiangyin Hospital Affiliated to Nanjing University of Chinese Medicine, Jiangyin, Jiangsu 214400, China

## Abstract

**Objective:**

We analyzed the efficacy and pharmacological mechanisms of action of Zhen Ren Yang Zang decoction (ZRYZD) on ulcerative colitis (UC) using meta-analysis and network pharmacology.

**Methods:**

The major databases were searched for randomized controlled trials of ZRYZD for the treatment of UC. Meta-analysis of the efficacy of ZRYZD on UC was conducted using RevMan software. Active compounds and target genes were acquired using the Traditional Chinese Medicine Systems Pharmacology Database and Analysis Platform. UC-related genes were searched using the GeneCards database. Gene Ontology (GO) functional enrichment and Kyoto Encyclopedia of Genes and Genomes (KEGG) pathway enrichment analyses were performed using RGUI. A compound-target network was constructed using Cytoscape software, and a protein-protein interaction network was constructed using the STRING database. Molecular docking simulations of the macromolecular protein targets and their corresponding ligand compounds were performed using the AutoDock tool and AutoDock Vina software.

**Results:**

Meta-analysis revealed that the total effective rate and recovery rate of clinical efficacy were significantly higher in the experimental group than those of the control group. The screening identified 169 active compounds and 277 active target genes for ZRYZD. The 277 active target genes were compared with the 4,798 UC-related genes. This identified 187 active target genes of ZRYZD for UC that correlated with 138 active compounds. GO functional enrichment and KEGG pathway enrichment analyses were performed, and compound-target and protein-protein interaction networks were constructed. The key compounds and key target proteins were then selected. Finally, target protein binding with the corresponding compound was analyzed using molecular docking.

**Conclusion:**

Our findings demonstrate the effectiveness and safety of ZRYZD for the treatment of UC and provide insight into the underlying pharmacological mechanisms of action. Furthermore, key compounds were identified, laying the foundation for future studies on ZRYZD for the treatment of UC.

## 1. Introduction

Ulcerative colitis (UC) is a common chronic intestinal disease of unknown etiology and is associated with multifactorial, multilevel, and nonspecific inflammation [1]. The clinical manifestations of UC include diarrhea, abdominal pain, and stool containing mucus, pus, and/or blood. The incidence of UC is 1.2–20.3 per 100,000 persons per year, and its prevalence is 7.6–246.0 per 100,000 per year [2].

The lesions in UC involve the rectum and sigmoid colon, sometimes throughout the whole colon, mainly invading the colorectal mucosa and submucosa and showing phased and diffuse distribution, resulting in a propensity for relapse [3]. Mesalazine, immunosuppressants, and corticosteroids are clinically used to treat UC; however, these drugs are needed chronically and can cause adverse reactions, and relapse is common after cessation [4, 5]. Traditional Chinese medicine (TCM) has a long history of treating diarrhea and dysentery and is compliant with the concept of individualized treatment [6]. Recently, TCM has been used to treat UC, with positive outcomes [7–9].

Zhen Ren Yang Zang decoction (ZRYZD), first used during the Song Dynasty as the basic prescription for the treatment of diarrhea, primarily consists of yingsuke, roudoukou, hezi, rougui, dangshen, baizhu, danggui, baishao, muxiang, and gancao (scientific names: *Pericarpium Papaveris* (PP), *Semen Myristicae* (SM), *Fructus Chebulae* (FC), *Cortex Cinnamomi* (CC), *Radix Codonopsis* (RC), *Rhizoma Atractylodis Macrocephalae* (RAM), *Radix Angelicae Sinensis* (RAS), *Radix Paeoniae Alba* (RPA), *Radix Aucklandiae* (RA), and *Radix Glycyrrhizae* (RG), respectively) [10]. According to TCM theory, PP, SM, and FC are monarch and minister herbs and are regarded as the main components of ZRYZD.

ZRYZD acts as an intestinal astringent, has antidiarrheal properties, and warms the spleen and kidney. Several clinical studies have reported that the clinical effect of ZRYZD in the treatment of UC is remarkable [10–12]. Previous basic research studies suggest that ZRYZD can ameliorate colonic mucosal dysfunction and that it has a favorable therapeutic action in trinitrobenzene sulfonic acid-induced colitis [13]. Therefore, the clinical efficacy and pharmacology of ZRYZD for the treatment of UC merit further investigation.

In this study, we analyzed the efficacy and pharmacological mechanisms of action of ZRYZD for the treatment of UC using meta-analysis and network pharmacology. First, we screened randomized controlled trials (RCTs) that investigated the clinical efficacy of ZRYZD for UC and performed a meta-analysis to assess clinical efficacy and safety. Next, we identified the active compounds in ZRYZD and its target genes and compared them with UC-related genes to identify the active target genes involved in the therapeutic action of ZRYZD for UC. Subsequently, Gene Ontology (GO) functional enrichment and Kyoto Encyclopedia of Genes and Genomes (KEGG) pathway enrichment analyses were performed. The compound-target, key compound-target, and protein-protein interaction (PPI) networks were constructed, and the key compounds and key target proteins were selected. Finally, target protein binding with the corresponding compound was analyzed using molecular docking analysis.

## 2. Materials and Methods

### 2.1. Screening of RCTs of the Efficacy of ZRYZD in the Treatment of UC

PubMed, the Cochrane Central Register of Controlled Trials, Chinese National Knowledge Infrastructure, Wanfang Data, the Chongqing VIP database, and the Chinese Biomedical Literature database, from the establishment of each database to August 15, 2021, were searched using the terms “Zhen Ren Yang Zang decoction” and “ulcerative colitis.” These terms were searched in titles, abstracts, and the full text. We also checked references and citations in the identified studies manually to include other potentially eligible trials until no additional articles could be identified.

The inclusion criteria included the following: the study was designed as a RCT, the participants had a diagnosis of UC, ZRYZD was used in the experimental group, the control group used conventional therapy without TCM therapy, and there were clear outcome indicators. Exclusion criteria included the following: the outcome data of the study were incomplete and the ZRYZD prescription lacked the main components.

### 2.2. Data Extraction, Quality Assessment, and Meta-Analysis

Two reviewers independently extracted the information from the included studies. The main information included the first author, year of publication, number of patients in each group, methods of intervention in the experimental and control groups, and outcome data.

The Cochrane Reviewers' Handbook of guidelines was used to assess the risk of bias. The following seven criteria were used: random sequence generation; allocation concealment; patient blinding; assessor blinding; incomplete outcome data; selective outcome reporting; other risks of bias [14].

These main data were input into the Cochrane Collaboration's RevMan 5.3 software for meta-analysis to analyze the efficacy of ZRYZD on UC.

### 2.3. Screening of Active Compounds in ZRYZD

The compounds in the ten component herbs (PP, SM, FC, CC, RC, RAM, RAS, RPA, RA, and RG) were obtained using the Traditional Chinese Medicine Systems Pharmacology Database and Analysis Platform (TCMSP) (https://tcmspw.com/tcmsp.php) [15]. TCMSP is a unique systems pharmacology platform of Chinese herbal medicines that captures the relationships between drugs, targets and diseases. Oral bioavailability (OB) and drug-likeness (DL) are commonly used in network pharmacology to define active compounds. OB represents the rate the compound is absorbed into the body, and DL represents the degree to which a compound contains specific functional groups or has physical characteristics similar to existing drugs [16]. We used OB ≥ 30% and DL ≥ 0.18 to screen for the active compounds (the DLs of compounds in CC are generally low, and we, therefore, set DL ≥ 0.10 as the filter criteria) [17].

### 2.4. Screening of the Target Genes of Active Compounds

The corresponding target genes of the active compounds were also retrieved from the TCMSP. Setting the search format as “homo sapiens,” the target genes were imported into the UniProt Knowledgebase, a comprehensive resource for protein sequences and annotation data (https://www.uniprot.org/) [18]. Then, the human official gene symbols were identified and were considered the active target genes of ZRYZD.

### 2.5. Acquisition of UC-Related Genes and Identification of Active Target Genes of ZRYZD Acting on UC

“Ulcerative colitis” was used as the keyword in the GeneCards database (https://www.genecards.org/). The GeneCards database is a searchable, integrative database providing comprehensive, user-friendly information on all annotated and predicted human genes [19], from which the UC-related genes were searched and acquired. Then, the active target genes of ZRYZD were compared with the UC-related genes, and the intersecting genes were defined as the active target genes of ZRYZD acting on UC.

### 2.6. GO Functional Enrichment and KEGG Pathway Enrichment Analyses

The RGUI 3.6.1 and org.Hs.eg.db packages were used to obtain the entrezIDs of the active target genes. Then, RGUI and the clusterProfiler package were used to perform the GO functional enrichment analyses, which included the biological process (BP), molecular function (MF), cellular component (CC) analysis, and the KEGG pathway enrichment analysis [20].

### 2.7. Construction of the Compound-Target Network

Cytoscape 3.6.0 software and its NetworkAnalyzer tool function were used to construct and analyze the compound-target network. Nodes represent compounds and target genes, and edges represent the relationships between them. According to the degree of connection between the compound and the target gene (the more the connections, the higher the degree value), the compounds and target genes in the network were subject to further analysis [21].

### 2.8. Construction of the PPI Network

A PPI network was constructed after introducing the active target genes into the STRING database. The STRING database supports functional discovery in genome-wide experimental datasets (https://string-db.org/) [22]. Defining the research species as “homo sapiens” and the lowest interaction score of 0.4, a PPI network was obtained. Then, the PPI network data were used to perform topology analysis, and the key target proteins of ZRYZD acting on UC were selected according to the degree values of each target protein (the more the connections, the higher the degree value) using Cytoscape 3.6.0 software and its NetworkAnalyzer tool [21].

### 2.9. Verification of Molecular Docking

The binding of the target protein with its corresponding compound was analyzed using molecular docking. The structures of the target proteins were obtained from the RCSB PDB database (https://www.rcsb.org/), and the compounds were obtained from the PubChem database (https://pubchem.ncbi.nlm.nih.gov/). Molecular docking simulations of target proteins with their corresponding compounds were performed using AutoDockTool 1.5.6 and AutoDock Vina software [23, 24].

### 2.10. Statistical Analysis

RevMan 5.3 software was used for meta-analysis, and dichotomous data were expressed as the odds ratio (OR) with 95% confidence interval (CI), and continuous data were expressed as mean difference (MD) with 95% CI. Heterogeneity was assessed with the *Q*-test (*P*-value and *I*^2^), and *P* < 0.10 indicated heterogeneity across studies. Studies with *I*^2^ < 50% were considered to have no heterogeneity, and those with *I*^2^ ≥ 50% were considered to have heterogeneity. If no heterogeneity was detected, the fixed effects model was used as the pooling method; otherwise, the random effects model was used [25, 26]. *P* < 0.05 was considered statistically significant.

Using the bioinformatics tools of the platforms and software mentioned above, some statistical analyses for network pharmacology were performed automatically. In the GO functional enrichment and KEGG pathway enrichment analyses, an adjusted *P* (*q*-value) < 0.05 was considered statistically significant.

## 3. Results

### 3.1. Screened RCTs Investigating the Efficacy of ZRYZD for the Treatment of UC

**Figure 1:**
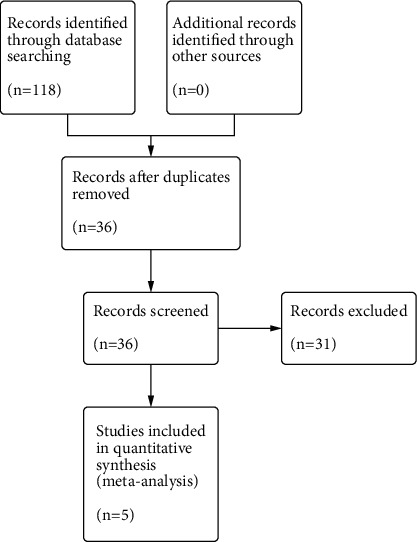
Flowchart of the study selection process.

A total of 118 studies were retrieved through database searching, and 36 studies were retained after removing duplication. According to the inclusion and exclusion criteria, a total of 31 studies were excluded after reading the title, abstract, and full text. Five RCTs [11, 12, 27–29] were included for further evaluation. The literature screening process is shown in Figure 1.

### 3.2. Description of Included RCTs and Assessment of the Methodological Quality

Five eligible RCTs [11, 12, 27–29] were identified. The five RCTs were all conducted in China and included 356 patients. The five studies were all single-center studies. The basic features of the included studies are outlined in Table 1.

One RCT [28] employed the odd and even numbers method of random sequence generation; none of the RCTs introduced allocation concealment; none of the RCTs described blindness; all the RCTs had complete outcome data; and for all studies, we were unable to determine whether they selectively reported data (Table 2, Figures S1 and S2).

Four RCTs [11, 12, 27, 28] assessed the total effective rate of clinical efficacy, four RCTs [11, 12, 27, 28] assessed the recovery rate of clinical efficacy, and one RCT [12] assessed the recovery rate of clinical efficacy. One RCT [28] evaluated the serum cytokines interleukin- (IL-) 6 and IL-8 and tumor necrosis factor- (TNF-) *α*, and one RCT [29] evaluated serum IL-6 and TNF-*α*. Two RCTs [28, 29] analyzed the total syndrome score of TCM, one RCT [28] assessed diarrhea, abdominal pain, mucopurulent bloody stool, and tenesmus score, and one RCT [28] compared Sutherland disease activity indexes. Adverse reactions were mentioned in three studies [11, 28, 29], while the other two studies [12, 27] did not mention whether there were adverse reactions. The main outcomes and results are presented in Table 3.

### 3.3. Meta-Analysis

#### 3.3.1. Clinical Efficacy

**Figure 2:**
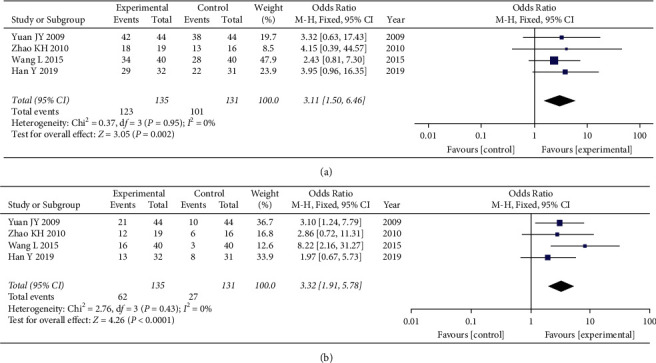
Forest plot of clinical efficacy. (a) The fixed effects model was used to merge OR values, and the pooled OR was 3.11 (95% CI 1.50–6.46, *P*=0.002). The total effective rate of clinical efficacy was statistically significantly higher in the experimental group than that in the control group. (b) The fixed effects model was used to merge OR values, and the pooled OR was 3.32 (95% CI 1.91–5.78, *P* < 0.0001). The recovery rate of clinical efficacy was significantly higher in the experimental group than that in the control group.

The four studies [11, 12, 27, 28] that compared the total effective rate of clinical efficacy included a total of 266 participants—135 in the experimental groups and 131 in the control groups. The four studies showed homogeneity of the data (heterogeneity test, Chi^2^ = 0.37, *P*=0.95, *I*^2^ = 0%). When the fixed effects model was used to merge OR values, the pooled OR was 3.11 (95% CI 1.50–6.46, *Z* = 3.05, *P*=0.002). This indicated that the total effective rate of clinical efficacy was significantly higher in the experimental group than that in the control group (Figure 2(a)).

The four studies [11, 12, 27, 28] that compared the recovery rate of clinical efficacy included a total of 266 participants—135 in the experimental groups and 131 in the control groups. The four studies showed homogeneity (heterogeneity test, Chi^2^ = 2.76, *P*=0.43, *I*^2^ = 0%). When the fixed effects model was used to merge OR values, the pooled OR was 3.32 (95% CI 1.91–5.78, *Z* = 4.26, *P* < 0.0001). This indicated that the recovery rate of clinical efficacy was significantly higher in the experimental group than that in the control group (Figure 2(b)).

#### 3.3.2. Serum Cytokines

The two studies [28, 29] that compared serum IL-6 included a total of 153 participants—77 in the experimental group and 76 in the control group. The two studies showed homogeneity of the data (heterogeneity test, Chi^2^ = 1.53, *P*=0.22, *I*^2^ = 35%). When the fixed effects model was used to merge MD values, the pooled MD was −15.74 [95% CI (−17.95)–(−13.53), *Z* = 13.96, *P* < 0.00001]. This indicated that serum IL-6 was significantly lower in the experimental group than that in the control group (Figure S3A).

The two studies [28, 29] that compared serum TNF-*α* included a total of 153 participants—77 in the experimental group and 76 in the control group. The two studies showed homogeneity (heterogeneity test, Chi^2^ = 0.23, *P*=0.64, *I*^2^ = 0%). When the fixed effects model was used to merge MD values, the pooled MD was −26.21 [95% CI (−29.37)–(−23.05), *Z* = 16.25, *P* < 0.00001]. This indicated that serum TNF-*α* was significantly lower in the experimental group than that in the control group (Figure S3B).

#### 3.3.3. Syndrome Scores of TCM

The two studies [28, 29] that compared the total syndrome score TCM included a total of 153 participants—77 in the experimental group and 76 in the control group. The two studies showed heterogeneity (heterogeneity test, Chi^2^ = 2.45, *P*=0.12, *I*^2^ = 59%). When the random effects model was used to merge MD values, the pooled MD was −2.98 [95% CI (−3.73)–(−2.23), *Z* = 7.81, *P* < 0.00001]. This indicated that the total syndrome score of TCM was significantly lower in the experimental group than that in the control group (Figure S4).

#### 3.3.4. Adverse Reactions

**Figure 3:**
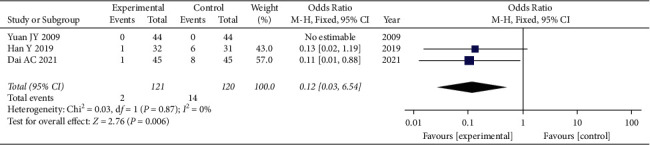
Forest plot of the incidence of adverse reactions. The fixed effects model was used to merge OR values, and the pooled OR was 0.12 (95% CI 0.03–0.54, *P*=0.006). The incidence of adverse reactions was significantly lower in the experimental group than in the control group.

The three studies [11, 28, 29] that compared the incidence of adverse reactions included a total of 241 participants—121 in the experimental group and 120 in the control group. The three studies showed homogeneity of the data (heterogeneity test, Chi^2^ = 0.03, *P*=0.87, *I*^2^ = 0%). When the fixed effects model was used to merge OR values, the pooled OR was 0.12 (95% CI 0.03–0.54, *Z* = 2.76, *P*=0.006). This indicated that the incidence of adverse reactions was significantly lower in the experimental group than in the control group (Figure 3).

### 3.4. Screening of Active Compounds in ZRYZD

A total of 24 compounds were obtained from PP, 64 from SM, 41 from FC, 100 from CC, 134 from RC, 55 from RAM, 125 from RAS, 85 from RPA, 106 from RA, and 280 from RG using the TCMSP (Supplementary File 1). By setting the filter criteria as OB ≥ 30% and DL ≥ 0.18, 11 active compounds from PP, 9 from SM, 8 from FC, 10 from CC (setting DL ≥ 0.10), 21 from RC, 7 from RAM, 2 from RAS, 13 from RPA, 6 from RA, and 92 from RG were obtained. Finally, 169 active compounds in ZRYZD remained after the exclusion of duplicates. The basic information on the active compounds in ZRYZD is shown in Table S1.

### 3.5. Screened Active Target Genes of ZRYZD

The corresponding target genes of the 169 active compounds were also obtained from the TCMSP, in which 19 compounds did not have corresponding targets. Then, the corresponding gene symbols were screened by setting the format as “homo sapiens” from the UniProt Knowledgebase. Finally, 277 active target genes of the 150 active compounds in ZRYZD were identified (Supplementary File 2).

### 3.6. Acquired UC-Related Genes and Identified Active Target Genes of ZRYZD Acting on UC

**Figure 4:**
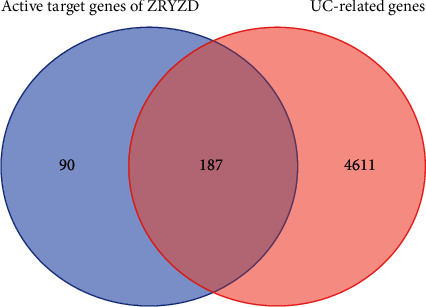
Active target genes of ZRYZD acting on UC. The 277 active target genes of ZRYZD were compared with the 4,798 UC-related genes, and 187 active target genes of ZRYZD acting on UC were identified.

We used “ulcerative colitis” as the keyword to search in the GeneCards database, which retrieved 4,798 UC-related genes (Supplementary File 3). The 277 active target genes of ZRYZD were compared with the 4,798 UC-related genes, which identified 187 active target genes of ZRYZD acting on UC (Figure 4, Table S2).

### 3.7. GO Functional Enrichment and KEGG Pathway Enrichment Analyses

The entrezIDs of the active target genes of ZRYZD acting on UC were obtained using RGUI and org.Hs.eg.db (Table S2). Then, GO functional enrichment and KEGG pathway enrichment analyses were performed using RGUI and clusterProfiler.

**Figure 5:**
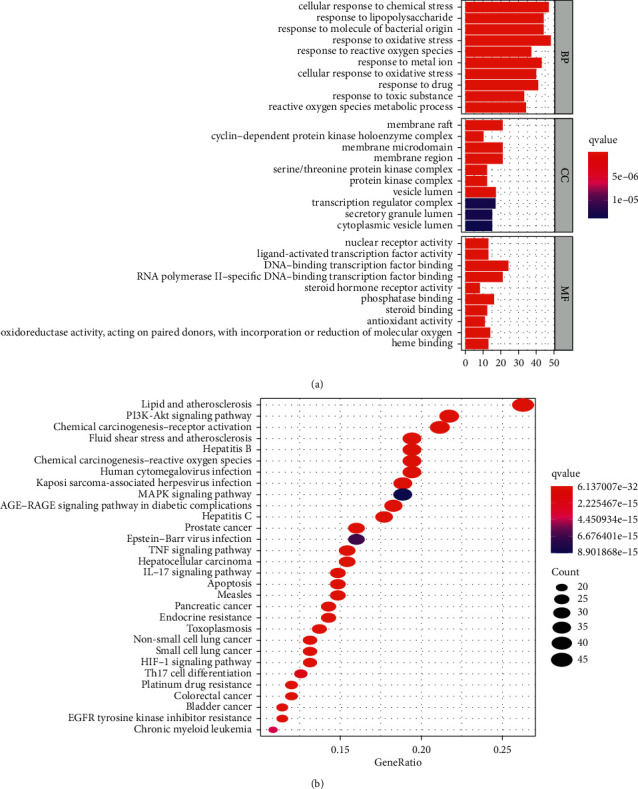
GO functional enrichment and KEGG pathway enrichment. (a) GO functional enrichment of active target genes. The smaller the *q*-value, the more significant the enrichment. (b) KEGG pathway enrichment of active target genes. The smaller the *q*-value and the greater the count, the more significant the enrichment.

The GO BP functional enrichment analysis showed that the active target genes of ZRYZD acting on UC were significantly enriched in cellular response to chemical stress, response to lipopolysaccharides, response to molecules of bacterial origin, response to oxidative stress, response to reactive oxygen species, and other processes. The GO CC functional enrichment analysis showed that the active target genes of ZRYZD acting on UC were significantly enriched in membrane rafts, cyclin-dependent protein kinase holoenzyme complex, membrane microdomains, membrane regions, serine/threonine protein kinase complex, and other functions. The GO MF functional enrichment analysis showed that the active target genes of ZRYZD acting on UC were significantly enriched in nuclear receptor activity, ligand-activated transcription factor activity, DNA-binding transcription factor binding, RNA polymerase II-specific DNA-binding transcription factor binding, steroid hormone receptor activity, and other functions (Supplementary File 4). The top 10 GO functional enrichments ranked by q-value are shown in Figure 5(a).

The KEGG pathway enrichment analysis showed that the active target genes of ZRYZD acting on UC were significantly enriched in lipid and atherosclerosis, receptor for advanced glycation end products (AGE)-receptor for AGE (RAGE) signaling pathway in diabetic complications, fluid shear stress and atherosclerosis, hepatitis B, prostate cancer, chemical carcinogenesis-receptor activation, pancreatic cancer, bladder cancer, IL-17 signaling pathway, hepatitis C, and other pathways (Supplementary File 5). The top 30 KEGG pathway enrichments ranked by count values are shown in Figure 5(b).

### 3.8. Construction of Compound-Target Network

**Figure 6:**
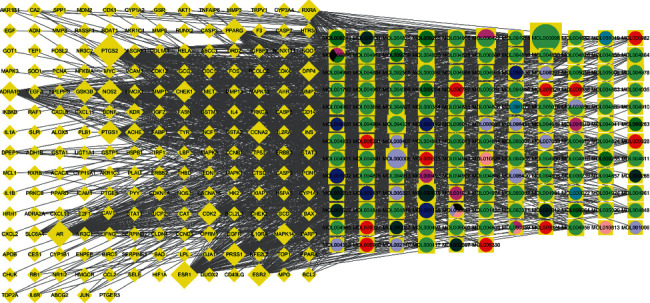
Compound-target network. There were 325 nodes (138 compound nodes and 187 target gene nodes) and 1,418 edges in the network. Circles represent active compounds (different colors represent different compounds), diamonds represent active target genes, and the edges represent links between the nodes. The more the connections between the compound and the target gene, the higher the degree value.

A compound-target network was constructed using Cytoscape software and analyzed using the NetworkAnalyzer tool. As some compounds had no correspondence to an overlapping target gene, the 187 overlapping active target genes correlated with 138 active compounds. There were 325 nodes (138 compound nodes and 187 target gene nodes) and 1,418 edges in the network (Supplementary File 6; Figure 6). Using the NetworkAnalyzer tool, the compounds ranked by the degree in the network are shown in Table S3.

PP, SM, and FC are monarch and minister herbs, which are regarded as the main active herbs in ZRYZD. We selected the compounds in PP, SM, and FC in the network for further analysis, as they can be considered the key compounds in ZRYZD acting on UC. The basic information for the key compounds, ranked by degree, with the 2D structure obtained from the PubChem database, is shown in Table 4.

We organized the data in Table 4, removed the compounds without a structure in the PubChem database, merged the same compounds, and used the most commonly used names in PubChem for the compounds with multiple names. The key compounds included ellipticine, ellagic acid, isoguaiacin, beta-sitosterol, (S)-laudanine, protopine, codeine, papaverine, cheilanthifoline, noscapine, peraksine, myricanone, norswertianin, tetrahydrofuroguaiacin B, narceine, permethrin, galbacin, cryptogenin, and chebulic acid.

**Figure 7:**
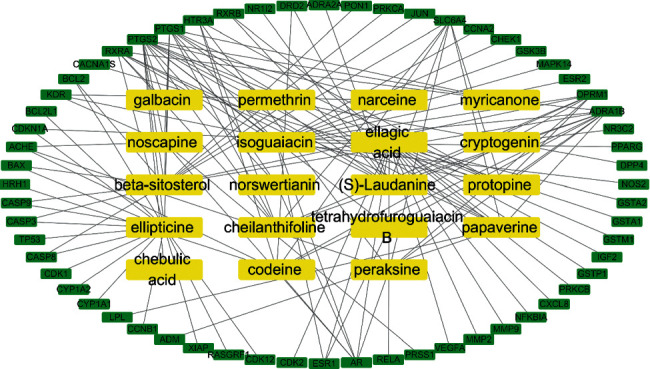
Key compound-target network. There were 79 nodes (19 compound nodes and 60 target gene nodes) and 132 edges in the network. The more the connections between the compound and the target gene, the higher the degree value.

After introducing the key compounds and their 60 corresponding target genes into Cytoscape software, a key compound-target network was constructed. There were 79 nodes (19 compound nodes and 60 target gene nodes) and 132 edges in the network (Supplementary File 7; Figure 7). Using the NetworkAnalyzer tool, the top six target genes, ranked by degree, were PTGS2, PTGS1, ADRA1B, RXRA, OPRM1, and SLC6A4.

### 3.9. Construction of the PPI Network

**Figure 8:**
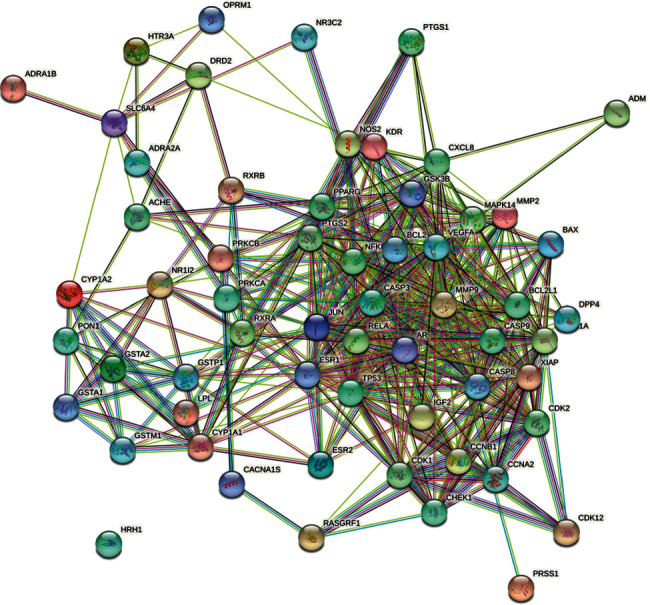
The PPI network. In the network, 23 target proteins had an interaction, and the 90 edges represent the interactions between the proteins, when the lowest interaction score was set to 0.40. The more the connections, the higher the degree value.

The 60 corresponding target genes were mapped into the STRING database, and the PPI network was obtained. In the network, 59 target proteins had interactions, and 456 edges represented the interactions between the proteins when the lowest interaction score was set to 0.40 (Supplementary File 8; Figure 8).

The top 10 target genes ranked by the degree in the PPI network are shown in Table 5; these can be considered the key target proteins of ZRYZD acting on UC.

### 3.10. Molecular Docking Analysis

**Figure 9:**
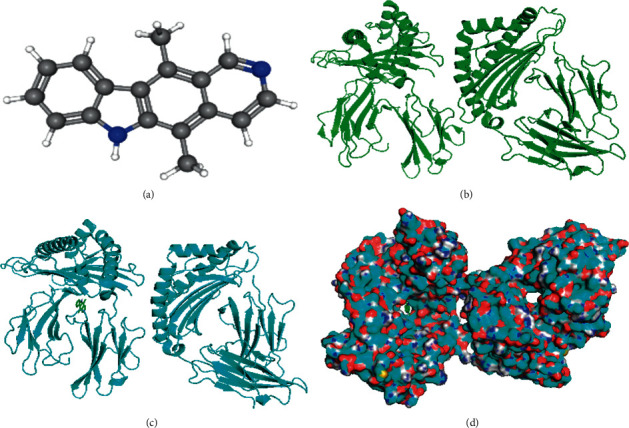
Molecular docking of TP53 and ellipticine. (a) 3D structures of ellipticine from the PubChem database. (b) 3D structures of TP53 from the RCSB PDB database. (c) Molecular docking simulation. (d) Molecular docking simulation displaying the protein surface.

The 3D structures of the compounds were obtained from the PubChem database, and the target proteins from the RCSB PDB database. Molecular docking simulations of the target proteins and their corresponding compounds were performed using AutoDockTool and AutoDock Vina software. The binding of the target proteins with their corresponding compounds was analyzed using molecular docking. The molecular docking simulations of TP53-ellipticine are shown in Figure 9.

## 4. Discussion

According to TCM theory, UC belongs to the category of “dysentery” and is characterized by dampness and heat accumulation, qi and blood disorder, and visceral food accumulation. The disease location of UC is in the intestine, and kidney qi insufficiency, spleen deficiency, endogenous dampness, and heat are considered the primary causes of this disease. Accordingly, TCM theory suggests that the treatment of patients should be based on supplementing the spleen and kidney, invigorating qi and warming yang [30]. From the perspective of modern medicine, the pathogenesis of UC is primarily related to chronic nonspecific inflammation, which is the result of the interaction of the host response, genetic factors, and immune imbalance.

ZRYZD, which consists of PP, SM, FC, CC, RC, RAM, RAS, RPA, RA, and RG as the main components, has the effect of consolidating and astringing the intestine, and nourishing the spleen and kidney. PP, SM and FC, which are considered monarch and minister herbs, can be used as intestinal astringents to stop diarrhea. CC, RC, and RAM can warm the spleen and kidney. RAS, RPA, and RA can regulate qi and blood. RG can replenish qi and reconcile all the other herbs [10].

Several clinical studies have reported that ZRYZD improves clinical outcomes in the treatment of UC. Therefore, we first evaluated the effectiveness and safety of ZRYZD for UC using an evidence-based analytical approach. We screened five RCTs that investigated the efficacy of ZRYZD for UC and performed a meta-analysis. Meta-analysis indicated that the total effective rate and recovery rate of clinical efficacy were statistically significantly higher in the experimental group than those in the control group and that the incidence of adverse reactions was significantly lower in the experimental group than that in the control group. This analysis demonstrates the effectiveness and safety of ZRYZD for UC from the perspective of evidence-based medicine, providing a foundation for further investigation of its pharmacological mechanisms of action. Furthermore, meta-analysis indicated that serum IL-6 and TNF-*α* were significantly lower in the experimental group compared with the control group, suggesting that the therapeutic effectiveness of ZRYZD for UC may be associated with a reduction in inflammation.

Network pharmacology is widely used in the study of TCM. The network pharmacology approach and platform could make the systematic study of herbal medicines achievable and advance pharmacodynamic substance discovery and could also provide a new strategy for translating TCM from an experience-based to an evidence-based medical system [31, 32]. Recently, guidelines for the network pharmacology evaluation method were drafted, allowing many technical and analysis-related problems to be resolved, permitting a more scientific approach for TCM network pharmacology research [33]. Network pharmacology advocates a multicomponent therapeutic approach, which is consistent with the multicomponent, multitarget, and multipathway characteristics of TCM [34, 35]. Hence, we used the network pharmacology approach to investigate the pharmacological mechanisms of action of ZRYZD for UC. In this study, 187 active target genes of ZRYZD acting on UC were identified. The GO BP functional enrichment analysis suggested that the active target genes of ZRYZD acting on UC were significantly enriched in the cellular response to chemical stress, response to lipopolysaccharides, response to molecules of bacterial origin, and other processes. The GO CC functional enrichment analysis revealed that the active target genes of ZRYZD acting on UC were significantly enriched in membrane rafts, cyclin-dependent protein kinase holoenzyme complex, membrane microdomains, and other functions. The GO MF functional enrichment analysis showed that the active target genes of ZRYZD acting on UC were significantly enriched in nuclear receptor activity, ligand-activated transcription factor activity, DNA-binding transcription factor binding, and other functions. These functions are closely related to inflammation and immune regulation, which are in turn closely related to the etiopathogenesis of UC [36–38].

The KEGG pathway enrichment analysis showed that many pathways were closely related to the pathogenesis of UC. The primary pathways included lipid and atherosclerosis, AGE-RAGE signaling pathway in diabetic complications, fluid shear stress and atherosclerosis, hepatitis B, prostate cancer, chemical carcinogenesis-receptor activation, pancreatic cancer, bladder cancer, IL-17 signaling pathway, and hepatitis C. Some of these pathways have been reported to be closely related to UC. IL-17 is upregulated in inflamed mucosa from UC patients, and IL-17 levels in peripheral blood mononuclear cells are correlated with disease severity in UC patients [39]. IL-17 is produced mainly by *T* helper 17 cells and is considered to be a key pathophysiological mediator and plays an important role in regulating the intestinal immune response [40]. In UC, AGE and IL-17 are highly expressed and participate in nuclear factor- (NF-) *κ*B pathway activation [41, 42]. These observations suggest that ZRYZD may ameliorate UC via multiple pathways related to inflammation and the immune response. These pathways and relevant target genes are worthy of further study.

A network of a compound-target network was constructed, and the compounds of ZRYZD acting on UC were identified. The 187 overlapping active target genes correlated with 138 active compounds. We found that these 138 compounds correspond to each herb in ZRYZD, and we can argue that every herb in ZRYZD plays a role in acting on UC. We also found that the importance of active compounds in PP, SM, and FC in the compound-target network is obvious. These findings are in agreement with TCM theory. PP, SM, and FC are monarch and minister herbs and are regarded as the main active herbs in ZRYZD. Therefore, we selected the compounds in PP, SM, and FC in the network for further study, and we constructed a key compound-target network to identify the key compounds. The key compounds were ellipticine, ellagic acid, isoguaiacin, beta-sitosterol, (S)-laudanine, protopine, codeine, papaverine, cheilanthifoline, noscapine, peraksine, myricanone, norswertianin, tetrahydrofuroguaiacin B, narceine, permethrin, galbacin, cryptogenin, and chebulic acid. *Ficus carica* aqueous extract containing ellagic acid can ameliorate UC-associated acute functional gastrointestinal disorder in rats [43]; A study verified the ethnomedical use of *Cornus mas* L. for the treatment of UC, in which ellagic acid was identified in extracts and its amount quantified [44]. A study provided evidence for the therapeutic effectiveness of *Canna x generalis* L.H. Bailey rhizome extract for the treatment of UC and discovered beta-sitosterol as one of the major identified constituents [45], which was shown to improve experimental colitis in mice by targeting pathogenic bacteria [46]. Prostaglandin synthetase activity in rectal biopsy specimens from patients with UC has been shown to fall on treatment with sulfasalazine, local steroids, and codeine phosphate [47]. Papaverine adjuvant can treat microcirculatory disturbance in severe UC complicated with cytomegalovirus infection [48]. These studies reveal an effect on the regulation of inflammation and immune function. Therefore, the action of ZRYZD on UC could be the result of the interaction of multiple compounds. However, there are only a few studies on the effect of individual constituent compounds on UC, which therefore requires further investigation.

The PPI network showed that the action of ZRYZD on UC was related to multiple targets. The key target proteins were TP53, VEGFA, JUN, CASP3, ESR1, PTGS2, MMP9, PPARG, BCL2L1, and CASP8. A previous study showed that alterations in p53 may be an early biomarker of a progressor colon and that p53 is upregulated early in UC-related carcinogenesis [49]. In UC patients, p53 enhances VEGF expression and subsequent production of proinflammatory TNF-*α* and IL-6 [50]. *Berberis lycium* fruit extract can attenuate oxidative/inflammatory stress and promote mucosal healing by downregulating NF-*κ*B/c-Jun/MAPK signaling and augmenting splenic Treg proliferation in a murine model of dextran sulfate sodium-induced UC [51]. Analysis of biopsies from UC patients and normal controls demonstrates that disease-associated occludin downregulation is accompanied by and correlated with reduced caspase-3 expression [52]. Inactivation through methylation of the putative tumor suppressor gene ESR1 may not be associated with colorectal carcinogenesis in UC [53]. Amentoflavone inhibits PTGS2 expression and modulates cytokine profile and NF-*κ*B signal transduction pathways in rats with UC [54]. In patients with active UC, MMP2, MMP9, and inflammatory factors are significantly increased [55]. Gliclazide attenuates acetic acid-induced colitis via the modulation of PPARG, NF-*κ*B, and MAPK signaling pathways [56]. HSPA6 is a UC susceptibility factor that is induced by cigarette smoke and protects intestinal epithelial cells by stabilizing antiapoptotic Bcl-XL [57]. Cyclosporine upregulates transforming growth factor-*β* in colonic tissue and inhibits caspase-8 activity in epithelial cells [58]. These studies demonstrate the relationship between these genes and UC, facilitating the further exploration of the therapeutic mechanisms of action.

Molecular docking was also performed to analyze specific interactions between key compounds and their protein targets, which could improve the robustness of the network model. The preliminary molecular docking results showed that the key active compounds in ZRYZD had high binding activities with their corresponding protein targets. These active compounds may mediate the therapeutic action of ZRYZD for UC via related signaling pathways. Compounds related to the corresponding target proteins can also be investigated in future studies.

The pharmacological mechanisms of action of ZRYZD for UC were investigated using a network pharmacology approach, and the binding of the target to the corresponding compound was analyzed using molecular docking. However, there are some limitations to using these approaches. First, the active compounds and target genes of ZRYZD were searched using the TCMSP database. The screening criteria and definition of the active compounds were fixed, and the UC-related genes were obtained from the GeneCards database. Although these databases are currently relatively comprehensive, some compounds and target genes may have been omitted. In addition, not all the compounds that enter the circulation may contribute to the efficacy of ZRYZD. Second, while the GO functional enrichment and KEGG pathway enrichment analyses were performed, and a PPI network was constructed to investigate the target genes and pathways of ZRYZD acting on UC, the potential target genes and pathways require further study using empirical analyses. Third, only preliminary molecular docking analyses were conducted in this study, and more in-depth analyses of the molecular docking of small-molecule compounds and macromolecular protein targets are needed.

## 5. Conclusion

The effectiveness and safety of ZRYZD for the treatment of UC were evaluated with an evidence-based approach. Using network pharmacology, we investigated the relationships between the active compounds, target genes, and signaling pathways, which revealed the involvement of multiple compounds, multiple targets, and multiple pathways. Finally, key compounds and their predicted target proteins were used for molecular docking analyses, which provided further evidence that these compounds may be important mediators of the therapeutic action of ZRYZD against UC.

## Figures and Tables

**Table 1:** Summary of RCTs of ZRYZD for UC.

Study year[ref]	Country	Sample size (experimental/control)	Mean age (years) (experimental/control)	Experimental	Control	Duration
Yuan JY, 2009 [11]	China	88 (44/44)	35.4/33.6	ZRYZD	SASP	6 months
Zhao KH, 2010 [12]	China	35 (19/16)	39.8 ± 14.0/40.2 ± 15.0	ZRYZD	SASP	6 months
Wang L, 2015 [27]	China	80 (40/40)	33.4/34.6	ZRYZD	SASP	4 weeks
Han Y, 2019 [28]	China	63 (32/31)	38.7 ± 7.9/36.6 ± 9.2	ZRYZD	Mesalazine bowel-soluble tablets	6 weeks
Dai AC, 2021 [29]	China	90 (45/45)	39.8 ± 3.16/39.91 ± 3.22	ZRYZD	Mesalazine bowel-soluble tablets	6 weeks

RCT: randomized controlled trial; ZRYZD: Zhen Ren Yang Zang decoction; UC: ulcerative colitis; SASP: sulfasalazine.

**Table 2:** Risk of bias in the five included RCTs.

Study year[ref]	Random sequence generation	Allocation concealment	Blinding of patient	Blinding of assessor	Incomplete outcome data	Selective reporting	Other bias
Yuan JY, 2009 [11]	U	U	H	H	L	U	L
Zhao KH, 2010 [12]	U	U	H	H	L	U	L
Wang L, 2015 [27]	U	U	H	H	L	U	L
Han Y, 2019 [28]	H	U	H	H	L	U	L
Dai AC, 2021 [29]	U	U	H	H	L	U	L

RCT: randomized controlled trial; L: low risk of bias; H: high risk of bias; U: unclear (uncertain risk of bias).

**Table 3:** Main outcomes in the included RCTs.

Study year [ref]	Main outcomes	Main results (effect size)	Adverse events
Yuan JY, 2009 [11]	(1) Clinical efficacy		No adverse reactions
	Total effective rate	OR, 3.32 [0.63, 17.43]	
	Recovery rate	OR, 3.10 [1.24, 7.79]	
	Recurrence rate	OR, 0.18 [0.05, 0.61]	

Zhao KH, 2010 [12]	(1) Clinical efficacy		n.r.
	Total effective rate	OR, 4.15 [0.39, 44.57]	
	Recovery rate	OR, 2.86 [0.72, 11.31]	

Wang L, 2015 [27]	(1) Clinical efficacy		n.r.
	Total effective rate	OR, 2.43 [0.81, 7.30]	
	Recovery rate	OR, 8.22 [2.16, 31.27]	

Han Y, 2019 [28]	(1) Clinical efficacy		Experimental: nausea (*n* = 3) Control: nausea (*n* = 3) somnolence (*n* = 1) vomit (*n* = 2)
	Total effective rate	OR, 3.95 [0.96, 16.35]	
	Recovery rate	OR, 1.97 [0.67, 5.73]	
	(2) Syndrome score of TCM		
	Total score	MD, −3.45 [−4.30, −2.60]	
	Diarrhea score	MD, −0.71 [−0.84, −0.58]	
	Abdominal pain score	MD, −0.73 [−0.85, −0.61]	
	Mucopurulent bloody stool score	MD, −1.24 [−1.55, −0.93]	
	Tenesmus score	MD, −0.77 [−1.03, −0.51]	
	(3) Sutherland disease activity indexes	MD, −1.32 [−1.69, −0.95]	
	(4) Serum cytokines		
	IL-6	MD, −14.14 [−17.50, −10.78]	
	IL-8	MD, −48.60 [−52.96, −44.24]	
	TNF-*α*	MD, −27.06 [−31.80, −22.32]	

Dai AC 2021 [29]	(1) Syndrome score of TCM		Experimental: nausea (*n* = 1) Control: nausea (*n* = 3) somnolence (*n* = 2) vomit (*n* = 3)
	Total score	MD, −2.67 [−3.16, −2.18]	
	(2) Serum cytokines		
	IL-6	MD, −16.96 [−19.89, −14.03]	
	TNF-*α*	MD, −25.52 [−29.76, −21.28]	

RCT: randomized controlled trial; TCM: traditional Chinese medicine; IL: interleukin; TNF: tumor necrosis factor; OR: odds ratio; MD: mean difference; n.r.: not reported.

**Table 4:** Key compounds in ZRYZD acting on UC.

Compound name	Compound ID	Pubchem CID	Molecular formula	2D structure (from PubChem)	Degree	Herb
Ellipticine	MOL009135	3213	C_17_H_14_N_2_	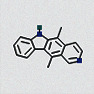	18	FC
Ellagic acid	MOL001002	5281855	C_14_H_6_O_8_	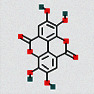	16	FC
Isoguaiacin	MOL009243	10314441	C_20_H_24_O_4_	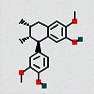	14	SM
Threo-austrobailignan-5	MOL009265	N/A	N/A	N/A	13	SM
Beta-sitosterol	MOL000358	222284	C_29_H_50_O	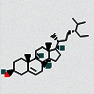	13	SM
5-[[(1S)-6,7-Dimethoxy-2-methyl-3,4-dihydro-1H-isoquinolin-1-yl]methyl]-2-methoxyphenol ((S)-Laudanine)	MOL009328	821396	C_20_H_25_NO_4_	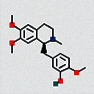	10	PP
5-[(2S,3S)-7-Methoxy-3-methyl-5-[(E)-prop-1-enyl]-2,3-dihydrobenzofuran-2-yl]-1,3-benzodioxole	MOL009255	N/A	N/A	N/A	10	SM
(R)-(6-Methoxy-4-quinolyl)-[(2R,4R,5S)-5-vinylquinuclidin-2-yl]methanol	MOL009137	N/A	N/A	N/A	10	FC
Fumarine (protopine)	MOL000787	4970	C_20_H_19_NO_5_	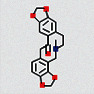	8	PP
Codeine	MOL006982	5284371	C_18_H_21_NO_3_	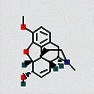	8	PP
Papaverine	MOL006980	4680	C_20_H_21_NO_4_	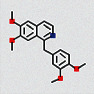	8	PP
Cheilanthifoline	MOL009149	5117621	C_19_H_19_NO_4_	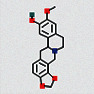	7	FC
Noscapine	MOL009330	275196	C_22_H_23_NO_7_	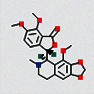	6	PP
Peraksine	MOL009136	78146432	C_19_H_22_N_2_O_2_	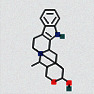	6	FC
Noskapin (noscapine)	MOL009327	275196	C_22_H_23_NO_7_	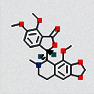	5	PP
Saucernetindiol	MOL009263	N/A	N/A	N/A	5	SM
Erythroculine	MOL009335	N/A	N/A	N/A	4	PP
Myricanone	MOL009331	161748	C_21_H_24_O_5_	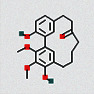	4	PP
Norswertianin	MOL009338	5281658	C_13_H_8_O_6_	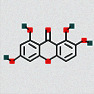	3	PP
Tetrahydrofuroguaiacin B	MOL009264	13870572	C_20_H_24_O_5_	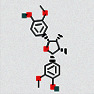	3	SM
Narcein (narceine)	MOL009329	8564	C_23_H_27_NO_8_	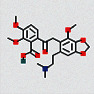	2	PP
Kudos (permethrin)	MOL009259	40326	C_21_H_20_Cl_2_O_3_	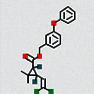	2	SM
Galbacin	MOL009254	234441	C_20_H_20_O_5_	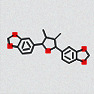	2	SM
Cryptogenin	MOL009324	21117640	C_27_H_42_O_4_	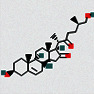	1	PP
Chebulic acid	MOL006826	71308174	C_14_H_12_O_11_	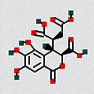	1	FC

ZRYZD: Zhen Ren Yang Zang decoction; UC: ulcerative colitis; PP: *pericarpium papaveris*; SM: *semen myristicae*; FC: *fructus chebulae.*

**Table 5:** Key target proteins of ZRYZD acting on UC.

Key target	Entry	Entry name	Protein names	Degree
TP53	P04637	P53_HUMAN	Cellular tumor antigen p53	37
VEGFA	P15692	VEGFA_HUMAN	Vascular endothelial growth factor A	35
JUN	P05412	JUN_HUMAN	Transcription factor AP-1	35
CASP3	P42574	CASP3_HUMAN	Caspase-3	34
ESR1	P03372	ESR1_HUMAN	Estrogen receptor	33
PTGS2	P35354	PGH2_HUMAN	Prostaglandin G/H synthase 2	30
MMP9	P14780	MMP9_HUMAN	Matrix metalloproteinase-9	30
PPARG	P37231	PPARG_HUMAN	Peroxisome proliferator-activated receptor gamma	28
BCL2L1	Q07817	B2CL1_HUMAN	Bcl-2-like protein 1	27
CASP8	Q14790	CASP8_HUMAN	Caspase-8	27

## Data Availability

The data used to support this study are included in the supplementary files.

## Supplementary Materials

Supplementary Materials Figure S1: Risk of bias graph. Figure S2: risk of bias summary. Figure S3: forest plot of comparison of serum cytokines. Figure S4: forest plot of comparison of the total syndrome score of TCM. Table S1: basic information on the active compounds in ZRYZD. Table S2: gene symbols and entrezID of active target genes. Table S3: compounds ranked by the degree in the network. Supplementary File 1: compounds of ZRYZD from TCMSP. Supplementary File 2: corresponding target genes of ZRYZD. Supplementary File 3: UC-related target genes. Supplementary File 4: GO functional enrichment analysis. Supplementary File 5: KEGG pathway enrichment analysis. Supplementary File 6: data of compound-target networks. Supplementary File 7: data of key compound-target networks. Supplementary File 8: data of PPI network.
