# Supplementary material for: Analysis of the Efficacy and Pharmacological Mechanisms of Action of Zhenren Yangzang Decoction on Ulcerative Colitis Using Meta-Analysis and Network Pharmacology
Source: Evid Based Complement Alternat Med. 2021 Dec 28;2021:4512755. doi: 10.1155/2021/4512755 (PMC8727130; doi:10.1155/2021/4512755)
Supplement: Supplementary Materials — Figure S1: Risk of bias graph. Figure S2: risk of bias summary. Figure S3: forest plot of comparison of serum cytokines. Figure S4: forest plot of comparison of the total syndrome score of TCM. Table S1: basic information on the active compounds in ZRYZD. Table S2: gene symbols and entrezID of active target genes. Table S3: compounds ranked by the degree in the network. Supplementary File 1: compounds of ZRYZD from TCMSP. Supplementary File 2: corresponding target genes of ZRYZD. Supplementary File 3: UC-related target genes. Supplementary File 4: GO functional enrichment analysis. Supplementary File 5: KEGG pathway enrichment analysis. Supplementary File 6: data of compound-target networks. Supplementary File 7: data of key compound-target networks. Supplementary File 8: data of PPI network. [file 4512755.f1.zip › 4512755.f1/Supplementary Figures.pdf]

Figure S1

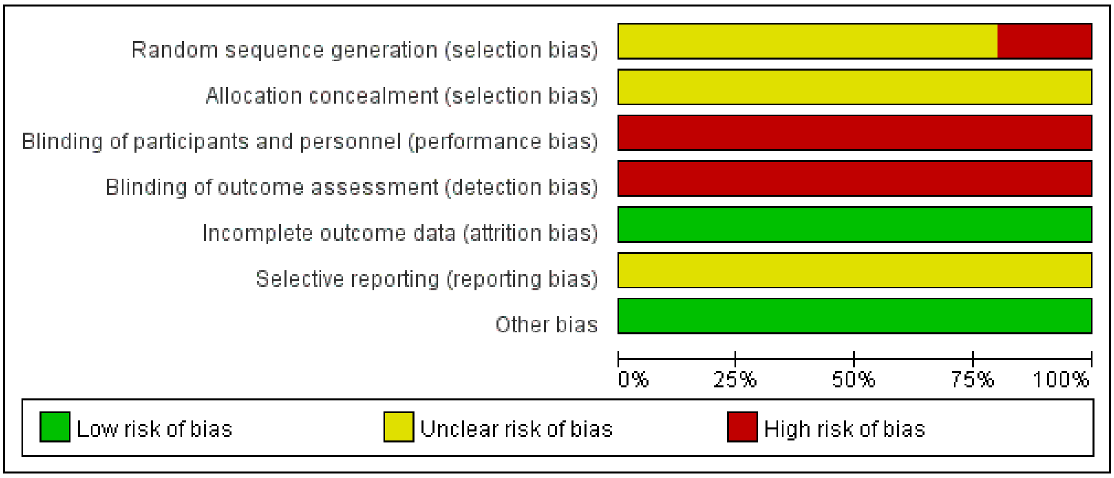

Figure S1. Risk of bias graph.

Review authors' judgements about each risk of bias item presented as percentages across all included studies.

Figure S2

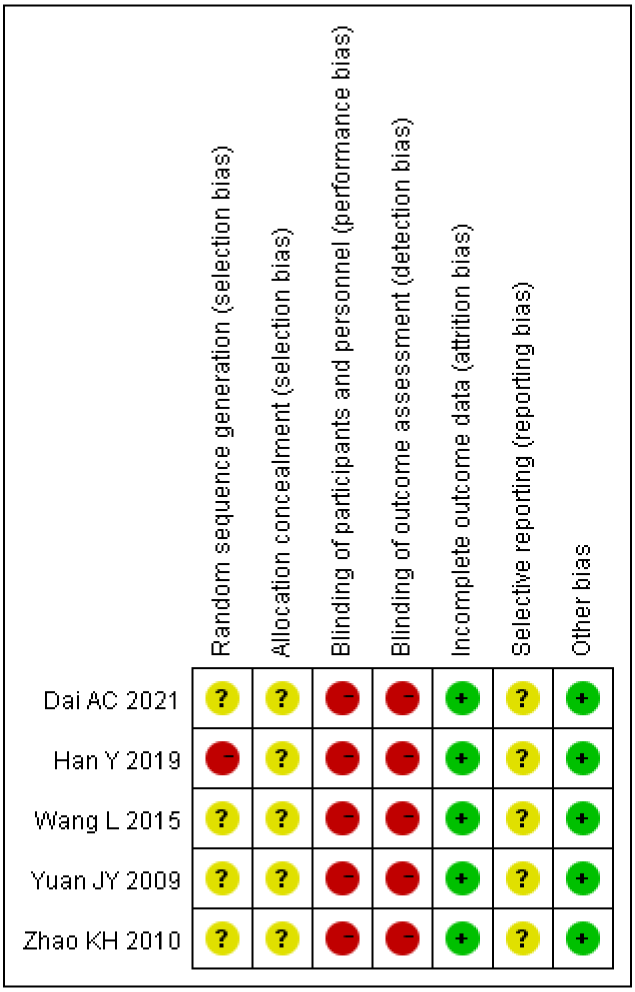

Figure S2. Risk of bias summary.

Review authors' judgements about each risk of bias item for each included study.

Figure S3

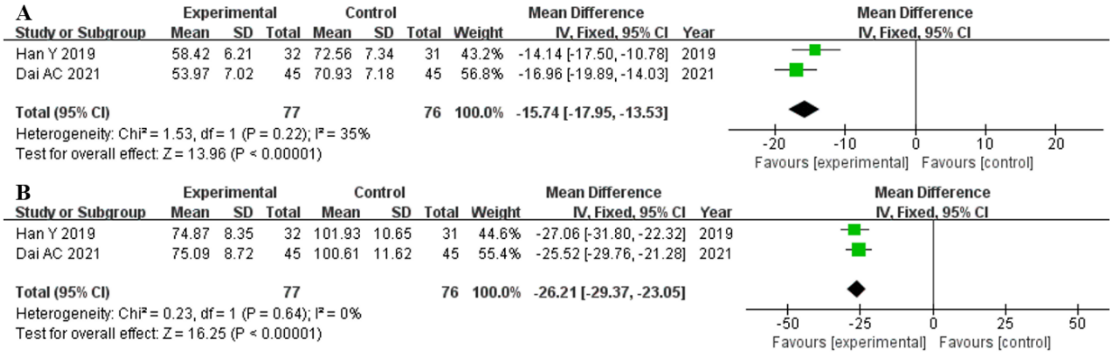

Figure S3. Forest plot of comparison of serum cytokines.

(A) Serum IL-6 was significantly lower in the experimental group compared with the control group; (B) Serum TNF-α was significantly lower in the experimental group compared with the control group.

Figure S4

Study or Subgroup	Experimental Mean	Experimental SD	Experimental Total	Control Mean	Control SD	Control Total	Weight	Mean Difference IV, Random, 95% CI	Year
Han Y 2019	3.93	1.15	32	7.38	2.12	31	39.8%	-3.45 [-4.30, -2.60]	2019
Dai AC 2021	3.84	1.1	45	6.51	1.25	45	60.2%	-2.67 [-3.16, -2.18]	2021
**Total (95% CI)**			**77**			**76**	**100.0%**	**-2.98 [-3.73, -2.23]**	

Heterogeneity: Tau² = 0.18; Chi² = 2.45, df = 1 (P = 0.12); I² = 59%

Test for overall effect: Z = 7.81 (P < 0.00001)

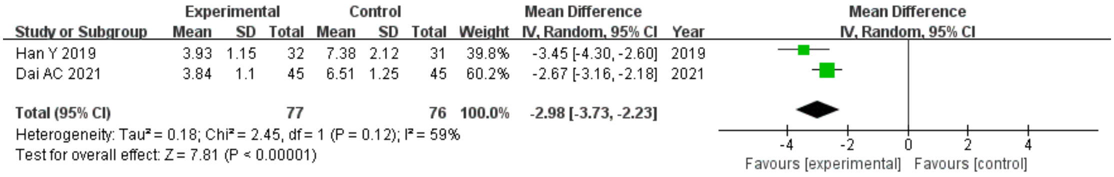

Figure S4. Forest plot of comparison of the total syndrome score of TCM.

The total syndrome score of TCM was significantly lower in the experimental group compared with the control group.
