# Supplementary material for: Analysis of the Efficacy and Pharmacological Mechanisms of Action of Zhenren Yangzang Decoction on Ulcerative Colitis Using Meta-Analysis and Network Pharmacology
Source: Evid Based Complement Alternat Med. 2021 Dec 28;2021:4512755. doi: 10.1155/2021/4512755 (PMC8727130; doi:10.1155/2021/4512755)
Supplement: Supplementary Materials — Figure S1: Risk of bias graph. Figure S2: risk of bias summary. Figure S3: forest plot of comparison of serum cytokines. Figure S4: forest plot of comparison of the total syndrome score of TCM. Table S1: basic information on the active compounds in ZRYZD. Table S2: gene symbols and entrezID of active target genes. Table S3: compounds ranked by the degree in the network. Supplementary File 1: compounds of ZRYZD from TCMSP. Supplementary File 2: corresponding target genes of ZRYZD. Supplementary File 3: UC-related target genes. Supplementary File 4: GO functional enrichment analysis. Supplementary File 5: KEGG pathway enrichment analysis. Supplementary File 6: data of compound-target networks. Supplementary File 7: data of key compound-target networks. Supplementary File 8: data of PPI network. [file 4512755.f1.zip › 4512755.f1/Supplementary File 1 Compounds of ZRYZD from TCMSP (1).pdf]

Supplementary File 1 Compounds of ZRYZD from TCMSP

Pericarpium Papaveris (PP)

Mol ID	Molecule Name	MW	AlogP	Hdon	Hacc	OB (%)	Caco-2	BBB	DL	FASA-	HL
MOL003848	Insularine	620.8	6.97	0	8	14.31	1.05	0.48	0.05	0.18	
MOL000045	atractylenolide iii	248.35	2.93	1	3	68.11	0.75	0.63	0.17	0	7.17
MOL005570	inositol	180.18	-3.06	6	6	15.55	-2.05	-4.57	0.05	0.28	
MOL006635	oxysophocarpine	262.39	-0.86	0	3	0.3	1.04	1.28	0.29	0.03	
MOL006979	morphine	285.37	1.39	2	4	24.69	0.56	0.33	0.51	0.28	
MOL006980	papaverine	339.42	3.5	0	5	64.04	1.22	0.57	0.38	0.18	4.14
MOL006982	codeine	299.4	1.64	1	4	45.48	0.87	0.87	0.56	0.21	10.06
MOL000787	Fumarine	353.4	2.95	0	6	59.26	0.56	-0.13	0.83	0.3	23.46
MOL009324	Cryptogenin	430.69	3.51	2	4	35.11	-0.03	-0.92	0.81	0.23	4.28
MOL009325	Sanleng acid	330.52	3.67	4	5	13.37	-0.69	-1.59	0.2	0.25	
MOL009326	Thellungianin F	218.32	4.14	0	2	27.55	1.32	1.33	0.07	0.38	
MOL009327	Noskapin	413.46	3.01	0	8	40.66	0.78	0.35	0.88	0.2	14.4
MOL009328	5-[[(1S)-6,7-dimethoxy-2-methyl-3,4-dihydro-1H-isoquinolin-1-yl]methyl]-2-methoxyphenol	343.46	3.6	1	5	51.55	0.98	0.78	0.37	0.21	3.55
MOL009329	Narcein	445.51	2.99	1	9	48.18	0.26	-0.04	0.64	0.22	24.99
MOL009330	Noscapine	413.46	3.01	0	8	53.29	0.89	0.36	0.88	0.21	13.55
MOL009331	Palaudine	325.39	3.25	1	5	68.27	0.94	0.42	0.34	0.22	4
MOL009332	Dihydrosecurinine	219.31	1.34	0	3	55.36	0.85	0.87	0.17	0.25	9.65
MOL009333	(S)-Reticuline	329.43	3.34	2	5	3.22	0.91	0.64	0.33	0.25	
MOL009334	salutaridine	329.43	2.15	1	5	17.5	0.58	0.11	0.33	0.25	
MOL009335	Erythroculine	343.46	2.31	0	5	63.36	0.57	0.04	0.53	0.18	9.8
MOL009336	Narcotoline	399.43	2.76	1	8	14.93	0.48	0.13	0.85	0.25	
MOL009337	Nardol	222.41	3.83	1	1	18.93	1.29	1.41	0.09	0.27	
MOL009338	Norswertianin	260.21	1.9	4	6	92.14	0.35	-0.46	0.22	0.4	15.95
MOL009339	Papaverrubine B	385.45	2.51	1	7	10.3	0.9	0.41	0.87	0.19	

Semen Myristicae (SM)

Mol ID	Molecule Name	MW	AlogP	Hdon	Hacc	OB (%)	Caco-2	BBB	DL	FASA-	HL
MOL000105	protocatechuic acid	154.13	0.9	3	4	25.37	0.1	-0.17	0.04	0.43	
MOL000125	(-)-alpha-Pinene	136.26	2.87	0	0	46.25	1.85	2.3	0.05	0.25	11.42
MOL000126	(-)-nopinene	136.26	2.93	0	0	44.84	1.8	2.12	0.05	0.27	11.32
MOL000130	CAM	152.26	1.94	0	1	67.17	1.29	1.71	0.05	0.27	11.34
MOL001308	oleic acid	282.52	6.84	1	2	33.13	1.14	0.93	0.14	0.2	5.39
MOL001393	myristic acid	228.42	5.46	1	2	21.18	1.07	0.99	0.07	0.19	
MOL001789	isoliquiritigenin	256.27	2.9	3	4	85.32	0.44	-0.41	0.15	0.46	17.66
MOL001867	Isovanillin	152.16	1.31	1	3	31.01	0.75	0.5	0.03	0.31	4.72
MOL000198	(R)-linalool	154.28	2.74	1	1	39.8	1.33	1.36	0.02	0.32	6.48

MOL000199	Safrol	162.2	2.61	0	2	45.34	1.44	1.29	0.05	0.38	5
MOL000200	(S)-(+)-alpha-Phellandrene	136.26	3.25	0	0	27.9	1.87	2.2	0.02	0.29	
MOL000206	isoeugenol	164.22	2.5	1	2	70.1	1.38	1.28	0.04	0.33	0.65
MOL000207	Methyleugenol	178.25	2.81	0	2	73.36	1.47	1.41	0.04	0.27	2.92
MOL000234	L-Limonen	136.26	3.5	0	0	38.09	1.83	2.13	0.02	0.29	11.64
MOL002399	TMPEA	211.29	1.21	2	4	47.54	0.64	0	0.06	0.15	3.52
MOL000261	Myristicin	192.23	2.59	0	3	17.99	1.35	1.14	0.07	0.3	
MOL000269	Elemicin	208.28	2.79	0	3	21.94	1.41	1.28	0.06	0.2	
MOL003521	Isohomogenol	178.25	2.75	0	2	32.61	1.49	1.33	0.04	0.28	2.73
MOL000357	Sitogluside	576.95	6.34	4	6	20.63	-0.14	-0.93	0.62	0.23	
MOL000358	beta-sitosterol	414.79	8.08	1	1	36.91	1.32	0.99	0.75	0.23	5.36
MOL000485	TMH	136.26	2.87	0	0	46.25	1.82	2.18	0.05	0	11.42
MOL005315	(R)-()-Citronellal	154.28	3.02	0	1	50.78	1.37	1.61	0.02	0.28	5.35
MOL000669	(S)-camphor	152.26	1.94	0	1	21.68	1.28	1.74	0.05	0.28	
MOL000675	oleic acid	282.52	6.84	1	2	33.13	1.17	0.78	0.14	0.2	4.99
MOL000676	DBP	278.38	4.2	0	4	64.54	0.8	0.56	0.13	0.34	5.41
MOL006866	Isosafrole	162.2	2.55	0	2	38.01	1.48	1.35	0.05	0.39	4.76
MOL000698	(R)-(-)-alpha-Phellandrene	136.26	3.25	0	0	27.51	1.86	2.17	0.02	0.3	
MOL000774	(-)-Citronellal	154.28	3.02	0	1	35.71	1.34	1.62	0.02	0.27	6.57
MOL007920	meso-1,4-Bis-(4-hydroxy-3-methoxyphenyl)-2,3-dimethylbutane	330.46	5.11	2	4	31.32	1.1	0.49	0.26	0.27	0.68
MOL000905	()-beta-Pinene	136.26	2.93	0	0	44.77	1.85	2.29	0.05	0.28	11.32
MOL009198	(+)-Anwulignan	328.44	5.16	1	4	23.61	1.13	0.52	0.32	0.28	
MOL000922	(R)-p-Menth-1-en-4-ol	154.28	2.55	1	1	32.16	1.33	1.52	0.03	0.26	11.39
MOL009237	2,3-dihydro-7-methoxy-2-(3-methoxy-4,5-methylenedioxyphenyl)-3-methyl-5-(E)-propenyl-benzofuran	354.43	4.51	0	5	7.95	1.22	0.49	0.5	0.26	
MOL009238	Methoxyeugenol	194.25	2.54	1	3	65.28	1.36	1.35	0.05	0.24	3.1
MOL009239	4-[(1R,2S)-2-(4-allyl-2,6-dimethoxyphenoxy)-1-hydroxypropyl]-2-methoxyphenol	374.47	3.96	2	6	9.43	0.7	0.14	0.35	0.28	
MOL009240	4-[(2R,3R,4S,5S)-5-(1,3-benzodioxol-5-yl)-3,4-dimethyl-2-tetrahydrofuranyl]-2-methoxyphenol	342.42	3.86	1	5	8.53	0.9	0.47	0.41	0.3	
MOL009241	2,6-dimethoxy-4-[(2S,3S)-7-methoxy-3-methyl-5-[(E)-prop-1-enyl]-2,3-dihydrobenzofuran-2-yl]phenol	356.45	4.46	1	5	28.06	1.26	0.73	0.38	0.24	
MOL009242	4-[(2R,3S,4S,5S)-5-(1,3-benzodioxol-5-yl)-3,4-dimethyl-2-tetrahydrofuranyl]-2-methoxyphenol	342.42	3.86	1	5	8.53	0.91	0.4	0.41	0.3	
MOL009243	Isoguaiacin	328.44	4.56	2	4	48.78	1.06	0.51	0.31	0.23	-0.92
MOL009244	Machilin D	344.44	3.92	2	5	10.56	0.77	0.21	0.29	0.31	

MOL009245	Myristargenol A	344.44	4.13	2	5	8.92	0.61	-0.17	0.34	0.31	
MOL009246	Myristargenol B	346.46	4.08	3	5	5	0.78	0.08	0.28	0.26	
MOL009247	Phenol, 4,4'-((2R,3R,4S,5S)-tetrahydro-3,4-dimethyl-2,5-furandiyl)bis(2-methoxy-, rel-	344.44	3.81	2	5	4.09	0.77	0.16	0.32	0.26	
MOL009248	Licarin A	326.42	4.47	1	4	9.75	1.29	0.76	0.31	0.28	
MOL009249	[(R)-cyano-[3-(phenoxy)phenyl]methyl] (1S,3R)-3-(2,2-dibromovinyl)-2,2-dimethyl-cyclopropane-1-carboxylate	505.22	5.63	0	4	16.22	1.1	0.56	0.43	0.55	
MOL009250	OKO	220.98	0.47	0	4	21.74	1.16	1.18	0.02	0.5	
MOL009251	4-[(2R,3S)-4-(1,3-benzodioxol-5-yl)-2,3-dimethylbutyl]-2-methoxyphenol	328.44	5.16	1	4	23.61	1.08	0.77	0.32	0.29	
MOL009252	(1R,4S,5S)-1-isopropyl-4-methyl-4-bicyclo[3.1.0]hexanol	154.28	1.91	1	1	95.36	1.2	1.45	0.05	0.28	11.21
MOL009253	4-[(2S,3S,4R,5R)-5-(4-hydroxy-3-methoxyphenyl)-3,4-dimethyloxolan-2-yl]-2,6-dimethoxyphenol	374.47	3.79	2	6	3.71	0.81	0.08	0.4	0.22	
MOL009254	galbacin	340.4	3.91	0	5	61	0.92	0.23	0.53	0.34	13.4
MOL009255	5-[(2S,3S)-7-methoxy-3-methyl-5-[(E)-prop-1-enyl]-2,3-dihydrobenzofuran-2-yl]-1,3-benzodioxole	324.4	4.53	0	4	53.11	1.28	0.72	0.4	0.33	12.02
MOL009256	MALABARICONE B	342.47	5.66	3	4	1.04	0.68	0.02	0.35	0.34	
MOL009257	MALABARICONE C	358.47	5.39	4	5	1.26	0.58	-0.22	0.4	0.3	
MOL009258	diethyleniminophosphoryl-(4-methylphenyl)amine	237.27	1.9	1	4	97.66	0.99	0.86	0.09	0.24	23.95
MOL009259	Kudos	391.31	5.44	0	3	45.06	1.32	0.79	0.38	0.49	4.27
MOL009260	IPMC	209.27	2.3	1	4	66.39	1.01	1.14	0.06	0.29	0.06
MOL009261	Propranolol, L-	259.38	2.54	2	3	57.99	1.03	0.73	0.15	0.33	4.73
MOL009262	S-Atenolol	266.38	0.67	4	5	44.08	0.16	-0.35	0.12	0.28	3.8
MOL009263	saucernetindiol	344.44	3.81	2	5	41.85	0.83	0.14	0.32	0.26	1.68
MOL009264	tetrahydrofuroguaiacin B	344.44	3.81	2	5	62.86	0.88	0.23	0.32	0.26	2.67
MOL009265	threo-austrobailignan-5	326.42	4.31	2	4	49.49	1	0.42	0.32	0.27	-0.05
MOL009266	trans-carane-cis 4-ol	154.28	1.95	1	1	66.73	1.3	1.51	0.05	0.24	10.92
MOL009267	4-[(2R,3S,4S,5S)-5-(4-hydroxy-3-methoxyphenyl)-3,4-dimethyloxolan-2-yl]-2-methoxyphenol	344.44	3.81	2	5	4.09	0.83	0.33	0.32	0.29	
MOL009268	2',4'-Dimethoxypropiophenone	194.25	2.2	0	3	33.36	1.27	1.07	0.05	0.21	16.79

Fructus Chebulae (FC)

Mol ID	Molecule Name	MW	AlogP	Hdon	Hacc	OB (%)	Caco-2	BBB	DL	FASA-	HL
MOL001002	ellagic acid	302.2	1.48	4	8	43.06	-0.44	-1.41	0.43	0.43	-1.04
MOL000131	EIC	280.5	6.39	1	2	41.9	1.16	0.9	0.14	0.25	7.5

MOL000219	BOX	121.12	0.76	0	2	31.55	0.54	0.84	0.02	0.11	12.07
MOL002275	Sennoside C	848.82	0.1	12	19	3.99	-3.54	-4.8	0.09	0.34	
MOL002276	Sennoside E_qt	524.5	3.91	6	9	50.69	-0.74	-1.56	0.61	0.37	33.6
MOL002285	1-O-Galloyl-glycerol	244.22	-0.17	5	7	63.21	-0.81	-1.71	0.1	0.34	5.5
MOL002850	butylated hydroxytoluene	220.39	4.85	1	1	40.02	1.75	1.8	0.07	0.3	10.36
MOL003069	quinic acid	191.18	-3.07	4	6	55.92	-1.79	-4.38	0.06	0.1	11.01
MOL004665	Ethyl geranate	196.32	3.74	0	2	64.07	1.41	1.43	0.04	0.29	7.16
MOL005074	SKM	174.17	-1.18	4	5	46.24	-1.16	-1.56	0.04	0.32	11.18
MOL005079	corilagin	634.49	0.9	11	18	3.01	-1.79	-2.58	0.44	0.37	
MOL000513	3,4,5-trihydroxybenzoic acid	170.13	0.63	4	5	31.69	-0.09	-0.54	0.04	0.41	11.78
MOL000515	Melissic acid	452.9	12.75	1	2	13.22	1.31	0.9	0.49	0.18	
MOL005508	Glucosol	472.78	5.5	3	4	15.86	-0.08	-0.75	0.74	0.25	
MOL005559	Maslinic acid	472.78	5.46	3	4	15.54	0.1	-0.55	0.74	0.25	
MOL006376	7-Dehydrosigmasterol	414.79	8.08	1	1	37.42	1.34	0.92	0.75	0.22	4.82
MOL006789	Gallic acid-3-O-(6'-O-galloyl)glucoside	484.4	-0.03	9	14	2.81	-1.99	-3.03	0.67	0.38	
MOL006826	chebulic acid	356.26	-0.26	6	11	72	-1.4	-1.75	0.32	0.4	3.44
MOL000069	palmitic acid	256.48	6.37	1	2	19.3	1.09	1	0.1	0	
MOL007254	Arjunolic acid	488.78	4.36	4	5	23.22	-0.32	-1.01	0.72	0.24	
MOL000749	Linoleic	280.5	6.39	1	2	41.9	1.23	0.81	0.14	0.23	5.27
MOL008319	α-Santalol	220.39	3.03	1	1	48.27	1.37	1.5	0.13	0.24	2.54
MOL009092	Pentagalloylglucose	940.72	3.69	15	26	3.01	-3.08	-4.17	0.21	0.45	
MOL009132	3-Dehydroshikimate	172.15	-0.93	3	5	46.09	-1.25	-2.06	0.04	0.38	11.52
MOL009133	Tannic acid (Corilagin)	636.51	1.21	11	18	3.01	-2	-3.29	0.54	0.39	
MOL009134	D-Altro-3-heptulose	210.21	-3.2	6	7	1.21	-1.99	-4.87	0.05	0.29	
MOL009135	ellipticine	246.33	4.05	1	1	30.82	1.6	0.97	0.28	0.29	9.73
MOL009136	Peraksine	310.43	2.48	2	3	82.58	0.51	0.07	0.78	0.26	7.11
MOL009137	(R)-(6-methoxy-4-quinolyl)-[(2R,4R,5S)-5-vinylquinuclidin-2-	324.46	2.73	1	4	55.88	0.62	0.21	0.4	0.25	3.84
MOL009138	Teresautalic acid	166.24	1.42	1	2	41.42	0.94	1.26	0.09	0.31	11.6
MOL009139	(2Z)-2-[(E)-3-cyano-3-[(2S,3R,4S,5S,6R)-3,4,5-trihydroxy-6-(hydroxymethyl)oxan-2-yl]oxyprop-2-enylidene]butanedioic acid	359.32	-2.32	6	11	1.7	-1.94	-2.43	0.28	0.37	
MOL009140	Triglochinin_qt	197.16	-0.23	3	6	49.04	-0.84	-1.27	0.05	0.48	4.12
MOL009141	(1S,4aR,6aR,6aS,6bR,8aR,9R,10R,11R,12aR,14bS)-1,10,11-trihydroxy-2,2,6a,6b,9,12a-hexamethyl-9-methylol-1,3,4,5,6,6a,7,8,8a,10,11,12,13,14b-tetradecahydropicene-4a-carboxylic acid	504.78	3.33	5	6	14.45	-0.71	-1.47	0.69	0.24	
MOL009142	beta-Glucogallin	332.29	-0.69	6	10	18.88	-1.18	-1.75	0.25	0.32	
MOL009143	chebulagic acid	954.7	1.52	13	27	3.01	-3.48	-4.07	0.03	0.39	
MOL009144	chebulinic acid	956.72	1.83	13	27	33.48	-3.24	-4.28	0.13	0.42	6.92

MOL009145	chebupentol	490.8	3.12	5	5	5.71	-0.5	-1.34	0.71	0.22	
MOL009146	punicalagin	1,084.75	3	17	30	18.17	-3.44	-4.46	0	0.38	
MOL009147	2,6-Dimethylheptadecane	268.59	8.63	0	0	3.81	1.84	1.93	0.1	0.18	
MOL009148	Catharanthamine	809.06	4.56	2	12	12.53	0.26	-0.31	0.02	0.2	
MOL009149	Cheilanthifoline	325.39	3.15	1	5	46.51	0.91	0.52	0.72	0.25	6.05

Cortex Cinnamomi (CC)

Mol ID	Molecule Name	MW	AlogP	Hdon	Hacc	OB (%)	Caco-2	BBB	DL	FASA-	HL
MOL001123	muurolene	204.39	4.75	0	0	19.5	1.84	2.16	0.08	0.25	
MOL000117	Cymol	134.24	3.51	0	0	27.2	1.86	2.1	0.02	0.34	
MOL000118	(L)-alpha-Terpineol	154.28	2.42	1	1	48.8	1.39	1.72	0.03	0.27	11.35
MOL000119	ZINC02040970	222.41	4.56	1	1	40.43	1.44	1.31	0.06	0.3	4.73
MOL001201	(1R,5R,7S)-4,7-dimethyl-7-(4-methylpent-3-enyl)bicyclo[3.1.1]hept-3-	204.39	4.7	0	0	16.23	1.86	1.96	0.09	0.27	
MOL000122	1,8-cineole	154.28	2.15	0	1	39.73	1.57	2.06	0.05	0.24	11.29
MOL001237	o-Acetyltoluene	134.19	2.06	0	1	38.96	1.47	1.66	0.02	0.43	25.18
MOL000125	(-)-alpha-Pinene	136.26	2.87	0	0	46.25	1.85	2.3	0.05	0.25	11.42
MOL000126	(-)-nopinene	136.26	2.93	0	0	44.84	1.8	2.12	0.05	0.27	11.32
MOL000128	NERYLACETATE	196.32	3.31	0	2	25.94	1.28	1.28	0.04	0.27	
MOL000130	CAM	152.26	1.94	0	1	67.17	1.29	1.71	0.05	0.27	11.34
MOL001300	PEL	122.18	1.55	1	1	44.03	1.11	1.13	0.02	0.37	-2.41
MOL000131	EIC	280.5	6.39	1	2	41.9	1.16	0.9	0.14	0.25	7.5
MOL001393	myristic acid	228.42	5.46	1	2	21.18	1.07	0.99	0.07	0.19	
MOL001578	Hypnon	120.16	1.57	0	1	48.19	1.36	1.54	0.02	0.45	25.2
MOL001579	germacrene	208.43	5.58	0	0	15.06	1.82	2.06	0.06	0.22	
MOL001599	α-cubebol	208.38	3.28	1	1	64.81	1.32	1.43	0.09	0.26	7.24
MOL001600	copaene	204.39	4.17	0	0	29.47	1.81	2.04	0.12	0.22	
MOL001602	1,4-cadinadiene	204.39	4.75	0	0	16.73	1.85	2.11	0.08	0.24	
MOL001619	UPL	268.59	9.04	0	0	8.52	1.83	1.7	0.11	0.16	
MOL000166	ZINC01609418	222.41	4.31	1	1	21.62	1.41	1.37	0.07	0.26	
MOL000170	guaiene	204.39	5.13	0	0	28.21	1.83	2.03	0.07	0.23	
MOL000172	Furol	96.09	0.99	0	2	34.35	1.08	1.51	0.01	0.2	4.53
MOL001739	zoomaric acid	254.46	5.92	1	2	35.78	1.18	0.88	0.1	0.24	5.29
MOL001853	2-Acetyl-6-methoxynaphthalene	200.25	2.46	0	2	17.46	1.27	0.96	0.08	0.39	
MOL001862	Cadalin	198.33	4.91	0	0	12.96	1.95	1.93	0.08	0.38	
MOL000019	D-Camphene	136.26	2.93	0	0	34.98	1.81	2.19	0.04	0	11.29
MOL000193	(Z)-caryophyllene	204.39	4.75	0	0	30.29	1.82	2.15	0.09	0.28	8
MOL000197	Myrcene	136.26	3.69	0	0	24.96	1.84	1.98	0.02	0.37	
MOL000198	(R)-linalool	154.28	2.74	1	1	39.8	1.33	1.36	0.02	0.32	6.48
MOL000201	p-Ocimene	136.26	3.63	0	0	15.06	1.85	1.99	0.02	0.39	
MOL000202	Moslene	136.26	3.45	0	0	33.02	1.88	2.05	0.02	0.27	11.08
MOL000208	()-Aromadendrene	204.39	4.22	0	0	55.74	1.81	2.06	0.1	0.26	11.84

MOL002085	alpha-Cubebene	204.39	4.17	0	0	16.73	1.83	2.1	0.11	0.25	
MOL000219	BOX	121.12	0.76	0	2	31.55	0.54	0.84	0.02	0.11	12.07
MOL002225	Styrone	134.19	1.69	1	1	38.35	1.14	1.09	0.02	0.41	4.38
MOL002295	cinnamic acid	148.17	1.9	1	2	19.68	0.91	0.96	0.03	0.47	
MOL000234	L-Limonen	136.26	3.5	0	0	38.09	1.83	2.13	0.02	0.29	11.64
MOL000024	alpha-humulene	204.39	5.04	0	0	22.98	1.88	2.08	0.06	0	
MOL002458	ZINC01850974	154.28	2.11	1	1	78.86	1.31	1.6	0.05	0.23	11.19
MOL000247	(Z,Z)-farnesol	222.41	4.76	1	1	41.14	1.25	1.15	0.06	0.28	6.29
MOL000249	Methylcinnamate	162.2	2.15	0	2	18.42	1.3	1.3	0.04	0.37	
MOL002496	Sulcatone	126.22	1.79	0	1	26.36	1.35	1.52	0.01	0.31	
MOL000250	cis-Cinnamaldehyde	132.17	1.95	0	1	27.21	1.33	1.45	0.02	0.47	
MOL002502	copaene	204.39	4.17	0	0	24.08	1.81	2.06	0.12	0.23	
MOL002526	3691-11-0	204.39	4.99	0	0	23.66	1.86	2.05	0.07	0.25	
MOL000254	eugenol	164.22	2.55	1	2	56.24	1.35	1.32	0.04	0.32	0.92
MOL002541	WLN: VH2R	134.19	1.97	0	1	19.01	1.35	1.61	0.02	0.39	
MOL000266	beta-Cubebene	204.39	4.22	0	0	32.81	1.83	2.02	0.11	0.25	6.59
MOL002697	junipene	204.39	4.18	0	0	44.07	1.82	2.14	0.11	0.25	12.2
MOL002836	anisaldehyde	136.16	1.57	0	2	21.54	1.12	1.15	0.02	0.33	
MOL002841	l-alpha-Fenchone	152.26	2.36	0	1	72.64	1.35	1.79	0.05	0.29	11.25
MOL002972	(4S)-1-methyl-4-(6-methylhepta-1,5-dien-2-yl)cyclohexene	204.39	5.33	0	0	20.3	1.89	1.99	0.06	0.28	
MOL003032	BZM	212.26	3.27	0	2	18.64	1.36	1.18	0.09	0.47	
MOL003047	[(1S)-endo]-(-)-Borneol	154.28	1.98	1	1	83.54	1.22	1.43	0.05	0.25	11.33
MOL000035	beta-Selinene	204.39	4.81	0	0	24.39	1.83	2.12	0.08	0	
MOL003521	Isohomogenol	178.25	2.75	0	2	32.61	1.49	1.33	0.04	0.28	2.73
MOL003522	()-Sativene	204.39	4.22	0	0	37.41	1.78	1.99	0.1	0.24	14.2
MOL003523	hexadec-11-enal	238.46	5.97	0	1	31.71	1.5	1.47	0.08	0.2	6.62
MOL003524	Benzenepropanol	136.21	2	1	1	36.57	1.18	1.15	0.02	0.33	4.09
MOL003525	Pyruvophenone	148.17	1.31	0	2	35.93	0.92	0.93	0.03	0.43	11.18
MOL003526	Cinnamyl acetate	176.23	2.07	0	2	21.15	1.29	1.29	0.04	0.38	
MOL003527	Tyranton	116.18	-0.08	1	2	58.34	0.49	0.37	0.01	0.31	11.21
MOL003528	METHYLBENZOFURAN	132.17	2.28	0	1	54.35	1.74	1.78	0.03	0.21	13.14
MOL003529	m-Methylacetophenone	134.19	2.06	0	1	40.63	1.39	1.44	0.02	0.43	24.94
MOL003530	O-METHOXYCINNAMALDEHYDE	162.2	1.93	0	2	26.52	1.19	1.18	0.04	0.36	
MOL003531	3-methoxycinnamaldehyde	162.2	1.93	0	2	54.65	1.1	0.99	0.04	0.37	4.92
MOL003532	(1R)-1-(4-methylphenyl)ethanol	136.21	2.09	1	1	23.64	1.23	1.34	0.02	0.36	
MOL003533	Bicyclo[4.4.0]dec-1-ene,2-isopropy-5-methyl-9-methylene-	204.39	4.99	0	0	19.78	1.86	2.12	0.08	0.25	
MOL003534	CADINENE	204.39	4.75	0	0	17.12	1.88	2.06	0.08	0.25	
MOL003535	1,1,6-trimethyl-2H-naphthalene	172.29	3.84	0	0	24.94	1.88	1.99	0.06	0.34	
MOL003536	T-Cadinol	222.41	3.78	1	1	28.59	1.41	1.47	0.09	0.24	
MOL003537	T-Muurolol	222.41	3.78	1	1	30.41	1.36	1.44	0.09	0.23	7.11

MOL003538	()-Ledene	204.39	4.36	0	0	51.84	1.86	2.16	0.1	0.23	11.79
MOL000431	coumarin	146.15	1.9	0	2	29.17	1.2	1.3	0.04	0	
MOL000475	anethole	148.22	2.77	0	1	32.49	1.75	1.81	0.03	0	1.68
MOL000479	Farnesene	204.39	5.52	0	0	17.42	1.95	2.21	0.05	0	
MOL000489	(1S,4aR,8aR)-1-isopropyl-7-methyl-4-methylene-2,3,4a,5,6,8a-hexahydro-1H-naphthalene	204.39	4.8	0	0	19.8	1.86	2.03	0.08	0	
MOL002003	(-)-Caryophyllene oxide	220.39	3.52	0	1	32.67	1.58	1.76	0.13	0.28	6.51
MOL000057	DIBP	278.38	3.92	0	4	49.63	0.85	0.68	0.13	0	3.94
MOL000608	()-Terpinen-4-ol	154.28	2.55	1	1	81.41	1.36	1.66	0.03	0.25	10.81
MOL000612	(-)-alpha-cedrene	204.39	4.12	0	0	55.56	1.81	2.16	0.1	0.24	4.82
MOL000615	delta-amorphene	204.39	4.94	0	0	17.95	1.85	1.99	0.08	0.24	
MOL000666	hexanal	100.18	1.85	0	1	55.71	1.25	1.52	0.01	0.23	10.96
MOL000675	oleic acid	282.52	6.84	1	2	33.13	1.17	0.78	0.14	0.2	4.99
MOL000677	(1R,4R)-4-isopropyl-1,6-	202.37	5.04	0	0	17.47	1.86	1.98	0.08	0.29	
MOL000069	palmitic acid	256.48	6.37	1	2	19.3	1.09	1	0.1	0	
MOL000698	(R)-(-)-alpha-Phellandrene	136.26	3.25	0	0	27.51	1.86	2.17	0.02	0.3	
MOL000699	m-Cymol	134.24	3.51	0	0	48.85	1.88	2.1	0.02	0.34	11.33
MOL000704	styrene	104.16	2.38	0	0	29.55	1.85	2	0.01	0.5	
MOL000708	WLN: VHR	106.13	1.59	0	1	32.63	1.32	1.66	0.01	0.44	12.07
MOL000860	stearic acid	284.54	7.28	1	2	17.83	1.15	1.22	0.14	0.19	
MOL000864	MYS	212.47	7.22	0	0	13.98	1.81	1.92	0.05	0.15	
MOL000868	LFA	282.62	9.5	0	0	8.46	1.83	1.8	0.13	0.13	
MOL000869	Henicosane	296.65	9.95	0	0	8.41	1.84	1.8	0.15	0.13	
MOL000885	Dodekan	170.38	5.85	0	0	17.74	1.79	1.96	0.02	0.16	
MOL000911	Terpilene	136.26	3.45	0	0	33.95	1.84	2.1	0.02	0.27	11.05
MOL000927	α-muurolene	204.39	4.75	0	0	17.24	1.88	2.09	0.08	0.25	
MOL000932	alpha-Farnesene	204.39	5.46	0	0	21.7	1.97	1.89	0.05	0.32	
MOL000991	cinnamaldehyde	132.17	1.95	0	1	31.99	1.35	1.48	0.02	0.48	4.73

Radix Codonopsis (RC)

Mol ID	Molecule Name	MW	AlogP	Hdon	Hacc	OB (%)	Caco-2	BBB	DL	FASA-	HL
MOL001006	poriferasta-7,22E-dien-3beta-ol	412.77	7.64	1	1	42.98	1.45	1.11	0.76	0.21	5.48
MOL001160	2-methoxyfuranodiene	246.38	3.94	0	2	53.58	1.45	1.51	0.13	0.21	-2.5
MOL000125	(-)-alpha-Pinene	136.26	2.87	0	0	46.25	1.85	2.3	0.05	0.25	11.42
MOL001309	6-methylolpyridin-3-ol	125.14	0.24	2	3	47.53	0.34	0.17	0.02	0.29	11.76
MOL000131	EIC	280.5	6.39	1	2	41.9	1.16	0.9	0.14	0.25	7.5
MOL001314	Azelex	188.25	1.87	2	4	16.9	-0.04	-0.72	0.04	0.26	
MOL001392	Methyl myristate	242.45	5.71	0	2	19.68	1.36	1.19	0.08	0.14	
MOL001393	myristic acid	228.42	5.46	1	2	21.18	1.07	0.99	0.07	0.19	
MOL001394	Oktadekan	254.56	8.58	0	0	9.81	1.83	1.83	0.09	0.14	
MOL001396	PENTADECYLIC ACID	242.45	5.91	1	2	20.18	1.08	0.88	0.08	0.18	

MOL001399	TWT	310.68	10.41	0	0	8.37	1.85	1.64	0.18	0.13	
MOL001619	UPL	268.59	9.04	0	0	8.52	1.83	1.7	0.11	0.16	
MOL001620	Pentadecene	210.45	6.82	0	0	17.72	1.84	1.97	0.05	0.21	
MOL001641	METHYL LINOLEATE	294.53	6.64	0	2	41.93	1.44	1.08	0.17	0.21	6.05
MOL001644	Dodecanal	184.36	4.59	0	1	21.52	1.4	1.42	0.03	0.19	
MOL000018	(+/-)-Isoborneol	154.28	1.98	1	1	86.98	1.27	1.6	0.05	0	11.36
MOL001817	Methyl stearate	298.57	7.53	0	2	16.8	1.41	1.29	0.16	0.16	
MOL001819	METHYL PENTADECANOATE	256.48	6.16	0	2	18.82	1.37	1.28	0.1	0.17	
MOL001887	SRT	150.1	-1.5	4	6	45.27	-1.7	-4.06	0.02	0.45	11.63
MOL002046	hexanoic acid	116.18	1.81	1	2	73.08	0.8	0.93	0.01	0.27	10.81
MOL002140	Perlolyrine	264.3	3.2	2	3	65.95	0.88	0.15	0.27	0.21	12.62
MOL002307	20-Hexadecanoylingenol	586.94	7.38	3	6	28.2	0.3	-0.04	0.68	0.24	
MOL002521	beta-Curcumene	204.39	5.33	0	0	4.48	1.92	2.07	0.06	0.3	
MOL002526	3691-11-0	204.39	4.99	0	0	23.66	1.86	2.05	0.07	0.25	
MOL002579	capsaicin	305.46	3.89	2	4	10.31	0.93	0.43	0.2	0.24	
MOL000261	Myristicin	192.23	2.59	0	3	17.99	1.35	1.14	0.07	0.3	
MOL000027	alpha-Curcumene	202.37	5.34	0	0	4.68	1.93	1.99	0.06	0	
MOL002879	Diop	390.62	7.44	0	4	43.59	0.79	0.26	0.39	0.28	3.6
MOL002943	BuOH	74.14	0.97	1	1	22.02	0.94	1.05	0	0.21	
MOL000303	caprylic acid	144.24	2.72	1	2	16.4	0.9	1.02	0.02	0	
MOL003035	stigmasterol-β-glucoside	574.93	5.89	4	6	2.4	-0.23	-1	0.63	0.23	
MOL003036	ZINC03978781	412.77	7.64	1	1	43.83	1.32	0.96	0.76	0	5.79
MOL000305	lauric acid	200.36	4.54	1	2	23.59	1.02	1.1	0.04	0	
MOL003050	nonanoic acid	158.27	3.17	1	2	40.51	0.92	1.08	0.02	0.23	4.15
MOL003177	Syringaldehyde	182.19	1.29	1	4	67.06	0.71	0.4	0.05	0.25	11.47
MOL003304	Hentriacontan	436.95	14.51	0	0	8.07	1.88	1.8	0.51	0.15	
MOL000449	Stigmasterol	412.77	7.64	1	1	43.83	1.44	1	0.76	0.22	5.57
MOL000347	Syrigin	372.41	-0.51	5	9	14.64	-1.01	-1.81	0.32	0.23	
MOL003487	D-Friedoolean-14-en-3-one	424.78	7.26	0	1	12.9	1.48	1.36	0.77	0.25	
MOL003509	Nonanol	144.29	3.25	1	1	33.19	1.17	1.15	0.01	0.18	6.07
MOL000365	syringaresinol	418.48	2.1	2	8	3.29	0.6	-0.03	0.72	0.15	
MOL003766	Shekanin	462.44	0.14	6	11	25.1	-0.89	-1.85	0.79	0.28	
MOL003767	tectorigenin	300.28	2.05	3	6	28.41	0.52	-0.37	0.27	0.28	
MOL003895	5-Methoxymethyl furfural	140.15	1.08	0	3	28.23	0.6	0.87	0.02	0.16	
MOL003896	7-Methoxy-2-methyl isoflavone	266.31	3.36	0	3	42.56	1.16	0.56	0.2	0.33	16.89
MOL000394	choline	104.2	-1.57	1	1	0.47	0.86	0.64	0.01	0	
MOL000421	nicotinic acid	123.12	0.28	1	3	47.65	0.34	0.21	0.02	0	11.98
MOL004355	Spinasterol	412.77	7.64	1	1	42.98	1.44	1.04	0.76	0.21	5.32
MOL000044	atractylenolideII	232.35	3.57	0	2	47.5	1.3	1.37	0.15	0	7.21
MOL004492	Chrysanthemaxanthin	584.96	8.24	2	3	38.72	0.51	-0.98	0.58	0.3	17.47
MOL004498	12-O-Nicotinoylisolineolone	469.63	1.32	3	7	20.7	-0.54	-1.19	0.83	0.31	
MOL000045	atractylenolide iii	248.35	2.93	1	3	68.11	0.75	0.63	0.17	0	7.17

MOL004582	Methyl naphthalene	142.21	3.23	0	0	39.01	1.9	1.91	0.04	0.47	11.81
MOL004623	Encecalin	232.3	2.34	0	3	21.36	1.17	1	0.11	0.28	
MOL004652	(2R,3R,4S,5S,6R)-2-[[(3S,5S,9R,10S,13R,14R,17R)-17-[(E,2R,5S)-5-ethyl-6-methylhept-3-en-2-yl]-10,13-dimethyl-2,3,4,5,6,9,11,12,14,15,16,17-dodecahydro-1H-cyclopenta[a]phenanthren-3-yl]oxy]-6-(hydroxymethyl)oxane-3,4,5-triol	574.93	5.89	4	6	21.2	-0.2	-0.99	0.63	0.21	
MOL004664	heptanoic acid	130.21	2.26	1	2	13.38	0.87	0.98	0.01	0.26	
MOL000508	Friedelin	426.8	7.3	0	1	29.16	1.43	1.38	0.76	0	
MOL005270	n-Heptadecanol	256.53	6.9	1	1	12.97	1.31	0.98	0.09	0.15	
MOL005302	7-(beta-Xylosyl)cephalomannine	962.15	1.97	6	18	27.33	-1.67	-2.25	0.17	0.32	
MOL005304	7alpha-L-Rhamnosyl-6-methoxylutcolin	462.44	1.03	6	11	15.03	-0.69	-1.99	0.79	0.29	
MOL005321	Frutinone A	264.24	2.7	0	4	65.9	0.89	0.46	0.34	0.47	19.1
MOL005482	FOA	112.09	0.83	1	3	35.66	0.63	0.74	0.02	0.24	4.43
MOL000006	luteolin	286.25	2.07	4	6	36.16	0.19	-0.84	0.25	0.39	15.94
MOL000617	(14S)-14-methylpalmitic acid	270.51	6.62	1	2	23.12	1.01	0.9	0.11	0.22	
MOL000628	darutoside	574.93	5.89	4	6	21.32	-0.26	-0.96	0.63	0.22	
MOL006322	Friedoolean-14-en-3-yl acetate	468.84	7.68	0	2	9.59	1.36	1.15	0.74	0.24	
MOL006554	Taraxerol	426.8	7.3	1	1	38.4	1.37	1.18	0.77	0.22	2.07
MOL000667	1-hexanol	102.2	1.88	1	1	22.04	1.08	1.21	0.01	0.19	
MOL006774	stigmast-7-enol	414.79	8.08	1	1	37.42	1.39	1.04	0.75	0.22	6.28
MOL006844	Norharman	168.21	2.17	1	1	18.88	1.46	1.49	0.08	0.26	
MOL000069	palmitic acid	256.48	6.37	1	2	19.3	1.09	1	0.1	0	
MOL006988	NSC405997	342.68	7.96	2	2	12.59	0.58	-0.58	0.26	0.15	
MOL007059	3-beta-Hydroxymethyllenetanshiquinone	294.32	3.16	1	4	32.16	0.38	-0.48	0.41	0.36	22.51
MOL000721	Nonadienal	138.23	2.75	0	1	19.03	1.38	1.56	0.02	0.33	
MOL000748	HMF	126.12	0.67	1	3	45.07	0.05	-0.27	0.02	0.22	11.73
MOL007514	methyl icosa-11,14-dienoate	322.59	7.55	0	2	39.67	1.47	1.1	0.23	0.18	5.24
MOL000008	apigenin	270.25	2.33	3	5	23.06	0.43	-0.61	0.21	0.41	
MOL008142	Ricinin	164.18	-0.78	0	4	26.26	0.85	0.71	0.04	0.26	
MOL008284	BUA	88.12	0.89	1	2	21.62	0.69	0.98	0	0.34	
MOL000835	EA-fructofuranoside	208.24	-1.81	4	6	47.33	-1.05	-2.82	0.06	0.22	7.48
MOL008375	(1R)-2,3,4,9-tetrahydro-1H-$b-carboline-1-carboxylic acid	216.26	1.73	3	3	52.9	0.61	0.51	0.13	0.28	9.95
MOL008376	13-Methyl pentadecanoic acid	256.48	6.16	1	2	24.14	1.09	0.85	0.1	0.2	
MOL008377	Galuteolin	448.41	0.16	7	11	2.7	-1.5	-2.47	0.79	0.34	
MOL008378	o-(o-Methoxyphenoxy)phenol	216.25	3.11	1	3	50.75	1.33	1.32	0.09	0.39	7.61
MOL008379	tangshenoside I	678.71	-2.37	9	18	4.93	-2.8	-3.82	0.47	0	
MOL008380	tangshenoside I_qt	354.39	1.29	3	8	23.58	-0.3	-0.73	0.3	0	

MOL008381	tangshenoside III	726.8	-0.27	9	17	8.75	-2.29	-3.46	0.46	0	
MOL008382	tangshenoside III_qt	402.48	3.54	3	7	4.98	0.35	-0.77	0.45	0	
MOL008383	tangshenoside IV	1,033.10	-2.13	13	26	3.01	-4.15	-5.96	0.15	0	
MOL008384	tangshenoside IV_qt	546.62	3.43	4	11	4.28	0.1	-1.35	0.67	0	
MOL008385	T-BUTYLBENZENE	134.24	3.23	0	0	53.06	1.89	2.14	0.02	0.37	11.5
MOL008386	2,6-NONADIENOL	140.25	2.5	1	1	23.77	1.21	1.18	0.02	0.25	
MOL008387	3-METHYLCARBAZOLE	181.25	3.81	1	0	20.14	1.87	1.83	0.09	0.29	
MOL008388	L-Sulforaphane	177.32	1.16	0	2	56.97	0.69	0.64	0.02	0.42	4.21
MOL008389	4-Phenylbicyclo[2,2,2]octan-1-ol	202.32	2.89	1	1	25.07	1.27	1.3	0.09	0.32	
MOL008390	5-Mpe-bis(hobz)phenol	438.55	6.95	3	4	11.33	1	0.07	0.66	0.32	
MOL008391	5alpha-Stigmastan-3,6-dione	428.77	6.66	0	2	33.12	0.9	0.47	0.79	0.25	5.19
MOL008392	6,6'-Dimethoxygossypol	550.7	7.39	4	8	8.93	1.08	0.25	0.86	0.2	
MOL008393	7-(beta-Xylosyl)cephalomannine_qt	830.02	3.21	4	14	38.33	-0.87	-1.59	0.29	0	5.97
MOL008394	Butylcyclohexane	140.3	4.36	0	0	35.68	1.8	2.14	0.02	0.18	3.03
MOL008395	Codonopsine	267.36	1.07	2	5	45.83	-0.04	-0.43	0.13	0.25	-0.72
MOL008396	Coelogin	300.33	3.12	2	5	21.68	0.84	0.11	0.42	0.24	
MOL008397	Daturilin	436.64	4.34	0	4	50.37	0.61	0.06	0.77	0.32	5.73
MOL008398	Ethyl-p-digallate	350.3	2.13	5	9	2.27	-0.58	-1.23	0.31	0.4	
MOL008399	fritillaziebinol	324.98	4.93	1	1	15.01	1.33	1.28	0.34	0.3	
MOL008400	glycitein	284.28	2.32	2	5	50.48	0.56	-0.29	0.24	0.3	16.32
MOL008401	Henicosanoic acid	326.63	8.65	1	2	16.14	1.15	0.69	0.23	0.18	
MOL008402	BHG	264.36	0.14	4	6	15.6	-0.72	-1.12	0.11	0	
MOL008403	Hexyl-beta-D-glucopyranosyl-(1-2)-beta-D-glucopyranoside	426.52	-1.61	7	11	6.51	-2.03	-2.67	0.44	0	
MOL008404	(2S)-2-ammonio-4-[(R)-methylsulfinyl]butyrate	165.24	-1.38	3	4	83.52	-0.93	-2.88	0.02	0.29	11.38
MOL008405	1-Peroxyferolide	338.39	1.44	1	7	17.38	-0.31	-0.86	0.35	0.38	
MOL008406	Spinoside A	716.95	2.91	5	12	39.97	-1.02	-1.76	0.4	0.32	8.24
MOL008407	(8S,9S,10R,13R,14S,17R)-17-[(E,2R,5S)-5-ethyl-6-methylhept-3-en-2-yl]-10,13-dimethyl-1,2,4,7,8,9,11,12,14,15,16,17-dodecahydrocyclopenta[a]phenanthren-	410.75	7.31	0	1	45.4	1.49	1.26	0.76	0.24	5.65
MOL008408	Stigmasteryl ferulate	588.95	9.87	1	4	24.53	1.16	0.3	0.55	0.26	
MOL008409	Tangshenoside II	372.41	-0.33	5	9	19.5	-0.99	-1.65	0.32	0	
MOL008410	Tangshenoside II_qt	210.25	1.57	2	4	51.72	0.65	0.24	0.06	0	5.8
MOL008411	11-Hydroxyrankinidine	356.46	1.04	2	6	40	0.29	-0.19	0.66	0.28	10.8
MOL008412	alpha-Stigmasta-7,22-dien-3-one	410.75	7.31	0	1	11.42	1.44	1.21	0.76	0.23	
MOL008413	Codopiloic acid	127.11	-1.06	1	4	57.5	0.04	0.19	0.02	0.31	44.48
MOL008414	delta22-Stigmasterol	414.79	7.89	1	1	7.04	1.4	1.1	0.76	0.2	
MOL008415	delta7-Stigmastenone-3	412.77	7.76	0	1	9.69	1.42	1.2	0.76	0.23	

MOL008416	delta7-stigmastenol-belta-D-glucopyranoside	578.97	6.59	4	6	19.58	-0.44	-0.99	0.62	0	
MOL008417	ethyl-β-D-fructofuranoside	320.48	1.22	0	6	33.84	0.81	0.5	0.15	0.2	4.46
MOL000860	stearic acid	284.54	7.28	1	2	17.83	1.15	1.22	0.14	0.19	
MOL000867	Heptadekan	240.53	8.13	0	0	8.64	1.84	1.87	0.07	0.14	
MOL000869	Henicosane	296.65	9.95	0	0	8.41	1.84	1.8	0.15	0.13	
MOL000879	methyl palmitate	270.51	6.62	0	2	18.09	1.37	1.18	0.12	0.14	
MOL000885	Dodekan	170.38	5.85	0	0	17.74	1.79	1.96	0.02	0.16	
MOL000890	(+)-alpha-Curcumene	202.37	5.34	0	0	26.56	1.93	2.03	0.06	0.33	
MOL000899	Furanodiene	216.35	4.63	0	1	45.11	1.77	1.95	0.1	0.25	-1.17
MOL000009	luteolin-7-o-glucoside	448.41	0.16	7	11	7.29	-1.23	-2.49	0.78	0.32	
MOL000905	()-beta-Pinene	136.26	2.93	0	0	44.77	1.85	2.29	0.05	0.28	11.32
MOL000095	delta 7-stigmastenol	416.81	8.33	1	1	25.32	1.31	0.98	0.75	0.21	
MOL000998	2-(3,4-dihydroxyphenyl)-5,7-dihydroxy-3-[(2R,3R,4S,5S)-3,4,5-trihydroxytetrahydropyran-2-yl]oxy-chromone	434.38	-0.08	7	11	4.05	-1.17	-1.9	0.7	0.36	

Rhizoma Atractylodis Macrocephalae (RAM)

Mol ID	Molecule Name	MW	AlogP	Hdon	Hacc	OB (%)	Caco-2	BBB	DL	FASA-	HL
MOL000018	(+/-)-Isoborneol	154.28	1.98	1	1	86.98	1.27	1.6	0.05	0	11.36
MOL000019	D-Camphene	136.26	2.93	0	0	34.98	1.81	2.19	0.04	0	11.29
MOL000020	12-senecioyl-2E,8E,10E-atractylentriol	312.39	2.5	0	4	62.4	0.01	-1.37	0.22	0.12	6.07
MOL000021	14-acetyl-12-senecioyl-2E,8E,10E-atractylentriol	355.44	3.21	0	5	60.31	0.33	-1.09	0.31	0.05	5.32
MOL000022	14-acetyl-12-senecioyl-2E,8Z,10E-atractylentriol	356.45	3.54	1	5	63.37	0.42	-1.14	0.3	0	6.43
MOL000023	Hemo-sol	136.26	3.5	0	0	39.84	1.83	2.12	0.02	0	11.68
MOL000024	alpha-humulene	204.39	5.04	0	0	22.98	1.88	2.08	0.06	0	
MOL000025	α-Longipinene	204.39	4.12	0	0	53.26	1.83	2.14	0.12	0	12.06
MOL000026	stigmast-22E-en-3beta-ol	414.79	7.89	1	1	10.39	1.43	1.13	0.75	0	
MOL000027	alpha-Curcumene	202.37	5.34	0	0	4.68	1.93	1.99	0.06	0	
MOL000028	α-Amyrin	426.8	7.35	1	1	39.51	1.42	1.28	0.76	0	3.83
MOL000029	beta-Humulene	204.39	5.09	0	0	26.87	1.82	2.01	0.06	0	
MOL000030	(1R)-2-methyl-1-phenylprop-2-en-1-ol	148.22	2.32	1	1	75.1	1.27	1.38	0.03	0	-2.13
MOL000031	(3S)-3-[(1R)-1,5-dimethylhex-4-enyl]-6-methylenecyclohexene	204.39	5.14	0	0	19.86	1.88	1.99	0.06	0	
MOL000032	beta-Eudesmol	222.41	3.72	1	1	26.09	1.32	1.38	0.1	0	

MOL000033	(3S,8S,9S,10R,13R,14S,17R)-10,13-dimethyl-17-[(2R,5S)-5-propan-2-yloctan-2-yl]-2,3,4,7,8,9,11,12,14,15,16,17-dodecahydro-1H-	428.82	8.54	1	1	36.23	1.45	1.09	0.78	0	5.22
MOL000034	2-[(1R,3S,4S)-3-isopropenyl-4-methyl-4-vinylcyclohexyl]propan-2-ol	222.41	3.7	1	1	19.03	1.37	1.46	0.07	0	
MOL000035	beta-Selinene	204.39	4.81	0	0	24.39	1.83	2.12	0.08	0	
MOL000036	beta-caryophyllene	204.39	4.75	0	0	29.7	1.83	2.07	0.09	0	
MOL000037	γ-elemene	204.39	4.93	0	0	23.79	1.87	2.1	0.06	0	
MOL000038	Akridin	179.23	3.35	0	1	33.71	1.63	1.53	0.1	0	6.82
MOL000039	(1S,2R,4R)-Neoiso-dihydrocarveol	154.28	2.58	1	1	52.4	1.38	1.69	0.03	0	11.46
MOL000040	Scopoletol	192.18	1.62	1	4	27.77	0.71	0.3	0.08	0	
MOL000041	PHA	165.21	0.96	3	3	41.62	0.36	0.22	0.04	0	4.62
MOL000042	LPG	89.11	-0.6	3	3	87.69	-0.34	-0.77	0.01	0	11.58
MOL000043	atractylenolide i	230.33	3.32	0	2	37.37	1.3	1.29	0.15	0	7.1
MOL000044	atractylenolideII	232.35	3.57	0	2	47.5	1.3	1.37	0.15	0	7.21
MOL000045	atractylenolide iii	248.35	2.93	1	3	68.11	0.75	0.63	0.17	0	7.17
MOL000046	atractylone	216.35	4.11	0	1	41.1	1.76	1.85	0.13	0	1.74
MOL000047	juniper camphor	222.41	3.93	1	1	33.3	1.44	1.61	0.1	0	9.27
MOL000048	(5E,9Z)-3,6,10-trimethyl-4,7,8,11-tetrahydrocyclodeca[b]furan	216.35	4.63	0	1	43.17	1.77	1.83	0.1	0	-1.4
MOL000049	3β-acetoxyatractylone	274.39	3.39	0	3	54.07	1.13	1.08	0.22	0	-1.31
MOL000050	GLY	75.08	-0.98	3	3	48.74	-0.56	-1.03	0	0	11.95
MOL000051	Polymannose	180.18	-2.68	5	6	1.76	-1.94	-4.53	0.03	0	
MOL000052	Gulutamine	147.15	-0.92	4	5	6.66	-1.05	-1.97	0.02	0	
MOL000053	Methose	180.18	-2.69	5	6	1.68	-1.8	-4.58	0.03	0	
MOL000054	L-	174.24	-1.11	7	6	47.64	-0.49	-1.04	0.03	0	0.85
MOL000055	L-Lysin	146.22	-0.68	5	4	29.33	-0.66	-1.44	0.02	0	
MOL000056	DTY	181.21	0.69	4	4	57.55	-0.1	-0.49	0.05	0	0.96
MOL000057	DIBP	278.38	3.92	0	4	49.63	0.85	0.68	0.13	0	3.94
MOL000058	2-[(2R,5S,6S)-6,10-dimethylspiro[4.5]dec-9-en-2-yl]propan-2-ol	222.41	3.67	1	1	38.59	1.34	1.42	0.09	0	7.61
MOL000059	uridine	244.23	-2.45	4	8	10.49	-1.14	-1.61	0.11	0	
MOL000060	selina-4(14),7(11)-dien-8-one	218.37	3.81	0	1	32.31	1.42	1.57	0.1	0	7.68
MOL000061	Prolinum	115.15	-0.06	2	3	77.57	0.22	0.29	0.01	0	11.13
MOL000062	biatractylolide	462.68	6.68	0	4	17.45	0.83	0.37	0.81	0	
MOL000063	ATRACTYLODES	462.68	6.68	0	4	14.6	0.88	0.6	0.81	0	
MOL000064	D-Serin	105.11	-1.49	4	4	83.59	-0.94	-1.75	0.01	0	11.51
MOL000065	ASI	133.12	-1.25	4	5	79.74	-1.02	-1.53	0.02	0	11.38
MOL000066	alloaromadedrene	204.39	4.22	0	0	53.46	1.83	2.1	0.1	0	12.51

MOL000067	L-Valin	117.17	0.24	3	3	53.33	0.04	-0.14	0.01	0	11.34
MOL000068	L-Ile	131.2	0.7	3	3	59.05	0.06	-0.11	0.02	0	11.21
MOL000069	palmitic acid	256.48	6.37	1	2	19.3	1.09	1	0.1	0	
MOL000070	Ethyl pivaloylacetate	172.25	1.69	0	3	40.52	0.82	0.83	0.03	0	1.92
MOL000071	Istidina	155.18	-1.01	4	4	53.18	-0.25	-0.4	0.03	0	-5.72
MOL000072	8β-ethoxy atractylenolide Ⅲ	276.41	3.68	0	3	35.95	1.08	1.12	0.21	0	8.34

Radix Angelicae Sinensis (RAS)

Mol ID	Molecule Name	MW	AlogP	Hdon	Hacc	OB (%)	Caco-2	BBB	DL	FASA-	HL
MOL000116	Nonanal	142.27	3.22	0	1	40.28	1.31	1.5	0.02	0.21	6.35
MOL000117	Cymol	134.24	3.51	0	0	27.2	1.86	2.1	0.02	0.34	
MOL000121	Decanal	156.3	3.68	0	1	29.81	1.34	1.44	0.02	0.2	
MOL001212	Loxanol V	214.44	5.53	1	1	14.19	1.27	0.98	0.05	0.14	
MOL001224	Tridecylene	182.39	5.91	0	0	17.69	1.83	2.01	0.03	0.2	
MOL000125	(-)-alpha-Pinene	136.26	2.87	0	0	46.25	1.85	2.3	0.05	0.25	11.42
MOL001273	80-57-9	150.24	1.94	0	1	50.63	1.27	1.59	0.06	0.34	11.56
MOL001302	Usaf hc-1	202.28	2.33	2	4	16.23	-0.01	-0.64	0.05	0.25	
MOL001306	o-Acetyl-p-cresol	150.19	1.79	1	2	24.96	1.02	0.88	0.03	0.39	
MOL001314	Azelex	188.25	1.87	2	4	16.9	-0.04	-0.72	0.04	0.26	
MOL001388	(+)-Ledol	222.41	3.2	1	1	16.96	1.43	1.69	0.12	0.21	
MOL001578	Hypnon	120.16	1.57	0	1	48.19	1.36	1.54	0.02	0.45	25.2
MOL000162	beta-Chamigrene	204.39	4.71	0	0	31.99	1.82	2.07	0.08	0	8.59
MOL001640	NON	172.3	3.63	1	2	26.74	0.96	1.06	0.03	0.22	
MOL001788	adenine	135.15	-0.58	3	4	62.81	-0.3	-0.63	0.03	0	13.33
MOL000197	Myrcene	136.26	3.69	0	0	24.96	1.84	1.98	0.02	0.37	
MOL000199	Safrol	162.2	2.61	0	2	45.34	1.44	1.29	0.05	0.38	5
MOL000201	p-Ocimene	136.26	3.63	0	0	15.06	1.85	1.99	0.02	0.39	
MOL000202	Moslene	136.26	3.45	0	0	33.02	1.88	2.05	0.02	0.27	11.08
MOL002029	()-Cuparene	202.37	4.72	0	0	38.26	1.88	2.15	0.07	0.3	-2.24
MOL002033	cis-Thujopsene	204.39	4.08	0	0	56.43	1.84	2.24	0.12	0.25	-1.45
MOL000206	isoeugenol	164.22	2.5	1	2	70.1	1.38	1.28	0.04	0.33	0.65
MOL002098	3-Butylidene-7-hydroxyphthalide	204.24	2.74	1	3	62.68	1	0.9	0.08	0.37	4.66
MOL002102	Levistolid A	380.52	4.97	0	4	2.15	0.94	0.45	0.82	0.27	
MOL002110	Allocymene	136.26	3.58	0	0	14.89	1.85	1.91	0.02	0.38	
MOL002111	BdPh	188.24	3	0	2	42.44	1.32	1.27	0.07	0.39	5.64
MOL002143	senkyunolide-C	204.24	2.74	1	3	46.8	0.87	0.5	0.08	0.39	5.8
MOL002144	senkyunolide-D	222.26	1.8	1	4	79.13	0.12	-0.07	0.1	0.37	5.37
MOL002145	senkyunolide-E	204.24	1.9	1	3	34.4	0.55	0.06	0.08	0.39	7.18
MOL002180	4-Octanone	128.24	2.46	0	1	19.37	1.37	1.55	0.01	0.22	
MOL002184	(6R)-6-butylcyclohepta-1,4-diene	150.29	3.93	0	0	31.69	1.85	2.18	0.02	0.25	5.7
MOL002201	cis-ligustilide	190.26	2.94	0	2	51.3	1.3	1.24	0.07	0.34	5.53
MOL000023	Hemo-sol	136.26	3.5	0	0	39.84	1.83	2.12	0.02	0	11.68

MOL000232	()-alpha-Terpineol	154.28	2.42	1	1	46.3	1.28	1.4	0.03	0.26	10.75
MOL002480	Methylbutenol	86.15	0.84	1	1	54.58	1.12	1.32	0.01	0.33	11.47
MOL000259	o-Thymol	150.24	3.24	1	1	43.28	1.58	1.71	0.03	0.33	11.29
MOL000270	CHEBI:7	136.26	2.87	0	0	45.2	1.84	2.15	0.04	0.27	11.44
MOL002830	PCR	108.15	2.05	1	1	51.99	1.56	1.88	0.01	0.41	11.87
MOL002972	(4S)-1-methyl-4-(6-methylhepta-1,5-dien-2-yl)cyclohexene	204.39	5.33	0	0	20.3	1.89	1.99	0.06	0.28	
MOL002983	Guasol	124.15	1.55	1	2	51.6	1.28	1.48	0.02	0.32	11.92
MOL002998	IPH	94.12	1.56	1	1	36.05	1.5	1.86	0.01	0.44	12.07
MOL000346	succinic acid	118.1	-0.41	2	4	29.62	-0.44	-0.71	0.01	0.42	
MOL000035	beta-Selinene	204.39	4.81	0	0	24.39	1.83	2.12	0.08	0	
MOL003534	CADINENE	204.39	4.75	0	0	17.12	1.88	2.06	0.08	0.25	
MOL000357	Sitogluside	576.95	6.34	4	6	20.63	-0.14	-0.93	0.62	0.23	
MOL000358	beta-sitosterol	414.79	8.08	1	1	36.91	1.32	0.99	0.75	0.23	5.36
MOL003587	Acoradiene	204.39	4.75	0	0	36.73	1.85	2.11	0.07	0.28	-0.84
MOL000360	FER	194.2	1.62	2	4	39.56	0.47	-0.03	0.06	0.34	2.38
MOL000383	D-Galacturonic acid, homopolymer	194.16	-2.47	5	7	29.75	-2	-4.71	0.04	0	
MOL000389	FERULIC ACID (CIS)	194.2	1.62	2	4	54.97	0.53	0.36	0.06	0	2.58
MOL000040	Scopoletol	192.18	1.62	1	4	27.77	0.71	0.3	0.08	0	
MOL000421	nicotinic acid	123.12	0.28	1	3	47.65	0.34	0.21	0.02	0	11.98
MOL004474	Maruzen M	122.18	2.51	1	1	48.44	1.57	1.81	0.02	0.37	11.63
MOL004479	o-cresol	108.15	2.05	1	1	62.45	1.57	1.9	0.02	0.4	11.96
MOL004613	6,7,3',8'-diligustilide	380.52	5.17	0	4	9.83	0.79	0.48	0.7	0.32	
MOL004723	beta-Terpinene	136.26	3.5	0	0	42.29	1.85	2.12	0.02	0.29	11.21
MOL004734	Butal	72.12	0.94	0	1	68.66	1.18	1.55	0	0.27	11.61
MOL000478	Eucarvone	150.24	2.07	0	1	53.14	1.35	1.65	0.03	0	11.45
MOL000479	Farnesene	204.39	5.52	0	0	17.42	1.95	2.21	0.05	0	
MOL004791	Ethol	242.5	6.45	1	1	13.32	1.31	1.07	0.08	0.15	
MOL000489	(1S,4aR,8aR)-1-isopropyl-7-methyl-4-methylene-2,3,4a,5,6,8a-hexahydro-1H-naphthalene	204.39	4.8	0	0	19.8	1.86	2.03	0.08	0	
MOL005125	ANN	152.16	1.42	1	3	29.69	0.69	0.51	0.03	0.36	
MOL005449	h-Met-h	149.24	-0.27	3	3	70.87	0.06	-0.17	0.01	0.37	11.69
MOL000449	Stigmasterol	412.77	7.64	1	1	43.83	1.44	1	0.76	0.22	5.57
MOL005589	3,4-DIMETHYLBENZALDEHYDE	134.19	2.56	0	1	39.99	1.4	1.51	0.02	0.39	11.76
MOL005590	ETHYLBENZALDEHYDE	134.19	2.53	0	1	40.95	1.4	1.62	0.02	0.37	4.59
MOL005608	TMHYDROP	152.21	2.75	2	2	54.42	1.21	1.04	0.03	0.35	11.67
MOL000635	vanillin	152.16	1.31	1	3	52	0.68	0.41	0.03	0.33	11.79
MOL006440	bicycloelemene	204.39	4.89	0	0	20.89	1.88	2.07	0.08	0.25	
MOL006869	WLN: QR CQ DV1	152.16	1.03	2	3	36.49	0.67	0.46	0.03	0.4	25.1
MOL000069	palmitic acid	256.48	6.37	1	2	19.3	1.09	1	0.1	0	
MOL000705	WLN: VH6	114.21	2.31	0	1	19.59	1.29	1.49	0.01	0.23	

MOL007745	WLN: QVR BVQ	166.14	1.04	2	4	17.74	-0.05	-0.23	0.04	0.46	
MOL008244	(1R,2S,4R)-1-ethyl-1-methyl-2,4-bis(1-methylethyl)cyclohexane	210.45	5.57	0	0	15.01	1.8	2.2	0.06	0.24	
MOL008245	DODECENE	168.36	5.46	0	0	17.74	1.8	2.05	0.02	0.22	
MOL008246	phosphatdic acid	228.11	-1.55	2	8	19.32	-1.22	-1.66	0.05	0.42	
MOL008247	L-beta,gamma-Dimyristoyl-alpha-	635.97	9.49	3	9	20.69	-0.43	-1.8	0.47	0.21	
MOL008248	phosphatidylinositol	390.27	-3.85	6	13	4.63	-2.34	-2.96	0.29	0.34	
MOL008249	phosphatidylinositol_qt	228.11	-1.55	2	8	12.66	-1.15	-1.77	0.05	0	
MOL008250	ESEN	148.12	1.3	0	3	47.31	0.56	0.3	0.04	0.54	13.73
MOL008251	sedanolide	194.3	3.37	0	2	62.46	1.24	1.4	0.07	0.28	5.05
MOL008252	senkyunolide	192.28	3.19	0	2	68.28	1.28	1.4	0.07	0.31	5.49
MOL008253	sphingomyelin	493.73	3.18	3	7	0.31	-0.46	-1.31	0.51	0.19	
MOL008254	Isotetandrine	622.82	7.22	0	8	10.42	0.95	0.28	0.1	0.21	
MOL008255	α-acoradiene	204.39	4.51	0	0	40.98	1.82	2.09	0.07	0.25	-0.9
MOL008256	InChI=1/C15H24/c1-10-7-8-15-9-12(10)14(3,4)13(15)6-5-11(15)2/h7,11-13H,5-6,8-9H2,1-4H	204.39	4.12	0	0	55.56	1.79	2.05	0.1	0.25	4.82
MOL008257	α-copaene	204.39	4.17	0	0	29.33	1.83	2.04	0.12	0.23	
MOL008258	(1R,4R,5S)-4-isopropenyl-1,8-dimethylspiro[4.5]dec-8-ene	204.39	4.75	0	0	40.65	1.83	2.1	0.07	0.26	-0.18
MOL008259	2,6-di(phenyl)thiopyran-4-thione	280.43	4.62	0	0	69.13	1.74	1.29	0.15	0.58	6.76
MOL008260	o-Xylenol	122.18	2.54	1	1	53.13	1.62	1.88	0.02	0.37	11.92
MOL008261	2,4,6-trimethyl-Octane	156.35	4.78	0	0	29.14	1.81	2.2	0.02	0.25	
MOL008262	Mesitaldehyde	148.22	3.05	0	1	37.8	1.54	1.58	0.03	0.37	11.63
MOL008263	Isoxylaldehyde	134.19	2.56	0	1	38.85	1.39	1.66	0.02	0.4	11.74
MOL008264	(E)-octadec-3-ene	252.54	8.14	0	0	19.5	1.86	2.07	0.09	0.19	
MOL008265	2-valerylbenzoic acid	206.26	2.75	1	3	78.26	0.61	0.45	0.06	0.36	17.78
MOL008266	(Z)-2-Hexenyl hexanoate	198.34	3.99	0	2	19.39	1.26	1.26	0.04	0.23	
MOL008267	2-Methylhexadecanoic acid	270.51	6.83	1	2	20.23	1.07	0.87	0.11	0.2	
MOL008268	cis-Isoeugenol	164.22	2.5	1	2	20.73	1.43	1.39	0.04	0.32	
MOL008269	2-methyl-5-decanone	310.63	7.99	0	1	20.4	1.42	1.32	0.11	0.25	
MOL008270	2-methyldodecan-5-one	198.39	4.54	0	1	13.16	1.43	1.39	0.04	0.2	
MOL008271	3,7-dimethyl-nonane	156.35	4.98	0	0	16.97	1.78	2.05	0.02	0.22	
MOL008272	1,1,5-trimethyl-2-formylcyclohexa-2,5-diene-4-one	164.22	1.64	0	2	48.94	0.82	0.65	0.04	0.36	11.76
MOL008273	4-Methyl-6-hepten-3-one	126.22	2.07	0	1	78.38	1.39	1.7	0.01	0.32	11.01
MOL008274	6-Ethylresorcinol	138.18	2.24	2	2	46.45	1.13	1.07	0.03	0.37	11.56
MOL008275	5-Indolol	133.16	1.86	2	1	63.14	1.38	1.44	0.03	0.19	5.74
MOL008276	Undecanol-6	172.35	4.15	1	1	25.77	1.19	1.14	0.02	0.2	
MOL008277	7,10-PENTADECADIYNOIC ACID	234.37	5.27	1	2	41.5	1.32	0.57	0.09	0.27	6.38

MOL008278	4-chloro-N-[1-methyl-5-[[1-methyl-5-[[1-methyl-5-(2-morpholinoethylcarbamoyl)pyrrol-3-yl]carbamoyl]pyrrol-3-yl]carbamoyl]pyrrol-3-yl]-5-[2-(2-pyridyl)ethylamino]isothiazole-3-carboxamide	762.38	2.93	5	13	7.18	-0.21	-1.98	0.31	0.17	
MOL008279	Amyl ketone	170.33	3.83	0	1	18.65	1.31	1.48	0.02	0.21	
MOL008280	Isoamylbenzene	148.27	3.94	0	0	35.69	1.84	2.05	0.03	0.34	5.89
MOL008281	(Z)-2-[[(Z)-2-methylbut-2-enoyl]oxymethyl]but-2-enoic acid	198.24	2.15	1	4	77.1	0.49	0.32	0.04	0.31	3.97
MOL008282	Tropone	106.13	1.14	0	1	47.41	1.25	1.52	0.01	0.5	12.06
MOL008283	aromadendrene	204.39	4.22	0	0	18.21	1.83	2.02	0.1	0.24	
MOL008284	BUA	88.12	0.89	1	2	21.62	0.69	0.98	0	0.34	
MOL008285	(3S)-3-butyl-3H-isobenzofuran-1-one	190.26	3.25	0	2	55.05	1.3	1.37	0.07	0.37	5.65
MOL008286	()-Camphoric acid	200.26	1.48	2	4	99.13	0.1	0.13	0.07	0.34	12.15
MOL008287	(3E)-3-butylidene-7-hydroxy-2-benzofuran-1-one	204.24	2.74	1	3	42.17	1.03	0.94	0.08	0.38	4.75
MOL008288	Coniferyl ferulate	356.4	3.64	2	6	4.54	0.71	-0.16	0.39	0.33	
MOL008290	lecithin	678.06	8.77	0	8	0.31	0.16	-1.1	0.4	0.02	
MOL008291	2,4-Xylylaldehyde	134.19	2.56	0	1	39.33	1.42	1.71	0.03	0.41	11.73
MOL008292	m-Ethylphenol	122.18	2.51	1	1	51.3	1.55	1.81	0.02	0.38	11.7
MOL008293	1,5,5,6-tetramethyl-1,3-Cyclohexadiene	136.26	3	0	0	39.22	1.82	2.18	0.03	0.29	11.69
MOL000885	Dodekan	170.38	5.85	0	0	17.74	1.79	1.96	0.02	0.16	
MOL000967	bergamotene	204.39	4.75	0	0	28.51	1.86	2.06	0.09	0.29	
MOL000974	cuminal	148.22	2.78	0	1	38.29	1.39	1.61	0.03	0.36	1.94

Radix Paeoniae Alba (RPA)

Mol ID	Molecule Name	MW	AlogP	Hdon	Hacc	OB (%)	Caco-2	BBB	DL	FASA-	HL
MOL000106	PYG	126.12	1.03	3	3	22.98	0.69	0.79	0.02	0.4	
MOL000211	Mairin	456.78	6.52	2	3	55.38	0.73	0.22	0.78	0.26	8.87
MOL000219	BOX	121.12	0.76	0	2	31.55	0.54	0.84	0.02	0.11	12.07
MOL000263	oleanolic acid	456.78	6.42	2	3	29.02	0.59	0.07	0.76	0.25	
MOL000357	Sitogluside	576.95	6.34	4	6	20.63	-0.14	-0.93	0.62	0.23	
MOL000358	beta-sitosterol	414.79	8.08	1	1	36.91	1.32	0.99	0.75	0.23	5.36
MOL000359	sitosterol	414.79	8.08	1	1	36.91	1.32	0.87	0.75	0.22	5.37
MOL000422	kaempferol	286.25	1.77	4	6	41.88	0.26	-0.55	0.24	0	14.74
MOL000492	(+)-catechin	290.29	1.92	5	6	54.83	-0.03	-0.73	0.24	0	0.61
MOL000513	3,4,5-trihydroxybenzoic acid	170.13	0.63	4	5	31.69	-0.09	-0.54	0.04	0.41	11.78
MOL000551	Hederagenol	472.78	5.33	3	4	22.42	0.1	-0.51	0.74	0.23	
MOL000561	Astragalin	448.41	-0.32	7	11	14.03	-1.34	-1.97	0.74	0.34	
MOL000610	TRD	184.41	6.3	0	0	17.89	1.78	1.96	0.03	0.19	

MOL000612	(-)-alpha-cedrene	204.39	4.12	0	0	55.56	1.81	2.16	0.1	0.24	4.82
MOL000676	DBP	278.38	4.2	0	4	64.54	0.8	0.56	0.13	0.34	5.41
MOL000775	EEE	88.12	0.37	0	2	45.02	1.07	1.26	0	0.21	11.64
MOL000842	sucrose	342.34	-4.31	8	11	7.17	-2.89	-6.67	0.23	0.2	
MOL000860	stearic acid	284.54	7.28	1	2	17.83	1.15	1.22	0.14	0.19	
MOL000867	Heptadekan	240.53	8.13	0	0	8.64	1.84	1.87	0.07	0.14	
MOL000868	LFA	282.62	9.5	0	0	8.46	1.83	1.8	0.13	0.13	
MOL000869	Henicosane	296.65	9.95	0	0	8.41	1.84	1.8	0.15	0.13	
MOL000874	paeonol	166.19	1.29	1	3	28.79	0.93	0.84	0.04	0.32	
MOL000875	Cedrol	222.41	3.16	1	1	16.23	1.35	1.46	0.12	0.24	
MOL000876	(6R,10R)-6,10,14-trimethylpentadecan-2-one	268.54	6.2	0	1	23.3	1.41	1.35	0.1	0.23	
MOL001218	Pisol	186.38	4.62	1	1	18.5	1.23	1.06	0.03	0.14	
MOL001219	Satol	268.54	6.91	1	1	27.27	1.34	0.97	0.11	0.17	
MOL001246	(1R)-()-Nopinone	138.23	1.52	0	1	57.86	1.23	1.6	0.05	0.28	11.1
MOL001393	myristic acid	228.42	5.46	1	2	21.18	1.07	0.99	0.07	0.19	
MOL001396	PENTADECYLIC ACID	242.45	5.91	1	2	20.18	1.08	0.88	0.08	0.18	
MOL001402	Octacosane	394.86	13.15	0	0	8.15	1.91	1.46	0.37	0.12	
MOL001644	Dodecanal	184.36	4.59	0	1	21.52	1.4	1.42	0.03	0.19	
MOL001801	salicylic acid	138.13	1.17	2	3	32.13	0.63	0.63	0.03	0.43	12
MOL001888	2,2-dimethylcyclohexanol	128.24	2.09	1	1	82.54	1.22	1.6	0.02	0.2	11.06
MOL001889	Methyl linolelaidate	294.53	6.64	0	2	41.93	1.46	1.11	0.17	0.2	5.78
MOL001890	octadec-9-ene	252.54	8.14	0	0	19.5	1.87	1.84	0.09	0.19	
MOL001891	9-methylenefluorene	178.24	3.55	0	0	26.87	1.95	1.78	0.09	0.54	
MOL001892	Dipropyl phthalate	250.32	3.29	0	4	66.3	0.78	0.71	0.1	0.35	5.7
MOL001893	BU3	90.14	-0.14	2	2	34.87	0.19	-0.01	0.01	0.28	11.25
MOL001894	Bicetyl	450.98	14.97	0	0	8.03	1.96	1.71	0.46	0.14	
MOL001895	Dibutylphenol	206.36	4.36	1	1	38.9	1.73	1.87	0.06	0.3	10.76
MOL001896	bicyclo[3.1.1]hept-2-ene-2-methanol, 6,6-dimethyl-	152.26	1.78	1	1	49.79	1.23	1.34	0.06	0.21	11.54
MOL001897	()-trans-Myrtanol	154.28	1.97	1	1	49.66	1.17	1.37	0.06	0.21	11.13
MOL001898	Acetyl oxide	102.1	-0.19	0	3	45.13	0.65	0.77	0.01	0.23	11.84
MOL001899	ZINC02169908	268.54	6.2	0	1	23.3	1.42	1.24	0.1	0.23	
MOL001900	Pulchinenoside A_qt	472.78	5.43	3	4	16.91	0.12	-0.58	0.77	0.25	
MOL001901	24-Methylenecycloartanol	440.83	7.86	1	1	10.4	1.42	1.13	0.79	0.25	
MOL001902	3β,23-dihydroxy-oleana-11,13(18)-dien-28-oic acid	470.76	5.08	3	4	21.53	0.1	-0.62	0.75	0.24	
MOL001903	3β-hydroxy-11-oxo-olean-12-en-28-oic acid	470.76	5.49	2	4	13.49	0.21	-0.2	0.74	0.27	
MOL001904	2 - methyl - 3 - (2 - propenyl) - phenol	148.22	3.06	1	1	52.06	1.64	1.81	0.03	0.39	4.7
MOL001905	3β-hydroxy-oleana-11,13(18)-dien-28-oic acid?	454.76	6.17	2	3	17.11	0.55	-0.13	0.76	0.26	

MOL001906	Methylgallate	184.16	0.88	3	5	30.91	0.26	0.06	0.05	0.32	5.67
MOL001907	Progallin A	198.19	1.23	3	5	25.61	0.33	0.11	0.06	0.34	
MOL001908	(Z)-(1S,5R)-beta-pinen-10-yl-beta-vicianoside	446.55	-1.28	6	10	5.74	-1.56	-1.89	0.67	0.23	
MOL001909	(Z)-(1S,5R)-beta-pinen-10-yl-beta-vicianoside_qt	152.26	2.05	1	1	50.32	1.52	1.93	0.06	0.22	11.47
MOL001910	11alpha,12alpha-epoxy-3beta-23-dihydroxy-30-norolean-20-en-28,12beta-olide	470.71	3.91	2	5	64.77	0.09	-0.59	0.38	0.27	2.62
MOL001911	albiflorin R1	480.51	-1.91	5	11	21.29	-1.53	-1.98	0.82	0.35	
MOL001912	albiflorin R1_qt	318.35	-0.17	2	6	26.18	-0.46	-0.85	0.34	0.4	
MOL001913	propyl (2R)-2-hydroxypropanoate	132.18	0.81	1	3	25.5	0.44	0.35	0.01	0.29	
MOL001914	gallotannin	1701.27	8.18	25	46	7.36	-5.47	-7.15	0.03	0.49	
MOL001915	paeonoside	610.57	-2.22	10	16	3.47	-2.71	-3.73	0.71	0.29	
MOL001917	(3S,3aR,5S,6S,7aR)-5,6-dihydroxy-3,6-dimethyl-3,3a,4,5,7,7a-hexahydrobenzofuran-2-one	200.26	-0.08	2	4	96.64	-0.16	-0.69	0.07	0.29	13.6
MOL001918	paeoniflorgenone	318.35	0.79	1	6	87.59	-0.09	-0.56	0.37	0.37	7.45
MOL001919	(3S,5R,8R,9R,10S,14S)-3,17-dihydroxy-4,4,8,10,14-pentamethyl-2,3,5,6,7,9-hexahydro-1H-cyclopenta[a]phenanthrene-15,16-dione	358.52	2.69	2	4	43.56	0	-0.41	0.53	0.35	4.34
MOL001921	Lactiflorin	462.49	-0.57	3	10	49.12	-1.13	-1.76	0.8	0.34	7.26
MOL001922	2-Hexyl-1-decanol	242.5	6.31	1	1	17.08	1.29	1.03	0.07	0.17	
MOL001923	[(3S,3aR,6S,7aR)-6-hydroxy-6-methyl-2,5-dioxo-3a,4,7,7a-tetrahydro-3H-benzofuran-3-yl]methyl benzoate	318.35	0.84	1	6	17.84	-0.17	-0.66	0.3	0.41	
MOL001924	paeoniflorin	480.51	-1.28	5	11	53.87	-1.47	-1.86	0.79	0.34	13.88
MOL001925	paeoniflorin_qt	318.35	0.46	2	6	68.18	-0.34	-0.73	0.4	0.39	8.81
MOL001926	1,2,3,6-tetra-O-galloylglucose	788.62	-0.25	17	22	3.01	-2.97	-4.03	0.34	0.39	
MOL001927	albiflorin	480.51	-1.33	5	11	12.09	-1.54	-2.19	0.77	0.36	
MOL001928	albiflorin_qt	318.35	0.42	2	6	66.64	-0.49	-0.88	0.33	0.41	6.54
MOL001929	alexandrin	576.95	6.34	4	6	20.63	-0.29	-0.98	0.62	0.24	
MOL001930	benzoyl paeoniflorin	584.62	0.81	4	12	31.27	-0.69	-1.24	0.75	0.29	-1.85
MOL001931	4-Chlorobutyric acid	122.56	0.87	1	2	85.82	0.74	0.85	0.01	0.44	11.6
MOL001932	galloylpaeoniflorin	632.62	-0.04	7	15	3.03	-1.77	-2.78	0.42	0.36	
MOL001933	oxypaeoniflorin	496.51	-1.55	6	12	21.88	-1.88	-2.41	0.78	0.34	
MOL001934	(3R,3aR,6S,7aR)-6-hydroxy-3,6-dimethyl-3a,4,7,7a-tetrahydro-3H-benzofuran-2,5-dione	198.24	-0.12	1	4	104.94	-0.08	-0.32	0.08	0.35	13.69

MOL001935	(3aR,6S,7aR)-6-hydroxy-6-methyl-3-methylene-3a,4,7,7a-tetrahydrobenzofuran-2,5-dione	196.22	-0.1	1	4	97.79	-0.01	-0.13	0.08	0.39	13.98
MOL001936	cis-5-Octen-1-ol	128.24	2.35	1	1	31.84	1.16	1.15	0.01	0.23	5.4
MOL001937	24253-30-3	98.16	1.16	0	1	74.2	1.33	1.57	0.01	0.32	11.36
MOL004480	acetic acid	60.06	-0.23	1	2	47.87	0.42	0.72	0	0.29	12.21
MOL005089	Oxypaeoniflorin	496.51	-1.55	6	12	8.38	-1.62	-2.33	0.78	0.33	
MOL007465	10-Methylnonadecane	282.62	9.29	0	0	10.28	1.84	1.79	0.12	0.16	
MOL008707	Ethylisobutyrate	116.18	1.5	0	2	83.67	1.24	1.49	0.01	0.27	11.14
MOL009092	Pentagalloylglucose	940.72	3.69	15	26	3.01	-3.08	-4.17	0.21	0.45	

Radix Aucklandiae (RA)

Mol ID	Molecule Name	MW	AlogP	Hdon	Hacc	OB (%)	Caco-2	BBB	DL	FASA-	HL
MOL001050	CRS	108.15	2.05	1	1	53.95	1.51	1.78	0.01	0.41	11.91
MOL010536	(3aS,6aR,9aR,9bR)-3,6,9-trimethylene-3a,4,5,6a,7,8,9a,9b-octahydroazuleno[5,4-d]furan-2-one	230.33	3.28	0	2	61.2	1.29	1.4	0.14	0.37	3.17
MOL010812	(Z)6,(Z)9-Pentadecadien-1-ol	224.43	5.1	1	1	17.53	1.34	1.03	0.06	0.2	
MOL010813	Benzo[a]carbazole	217.28	4.23	1	0	35.22	1.87	1.65	0.22	0.4	11.43
MOL010814	2-(4a,8-dimethyl-1,2,3,4,4a,5,6,7-octahydro-napthalen-2-yl)-prop-2en-1-ol	220.39	3.86	1	1	28.72	1.25	1.19	0.09	0.18	
MOL010815	2,4-Hexadiene, 3,4-dimethyl-, (E,Z)-	110.22	3.11	0	0	51.82	1.84	2.06	0.01	0.33	11.66
MOL010816	7-methylocta-3,4-diene	124.25	3.67	0	0	27.17	1.88	2.02	0.01	0.37	
MOL010817	7-methyl-4-(1-methylethylidene)-bicyclo[5.3.1]undec-1-en-8-ol	218.37	3.62	0	1	58.36	1.62	1.75	0.1	0.3	9.29
MOL010818	(3aS,5R,8aS,9aS)-5,8a-dimethyl-3-methylene-5,6,7,8,9,9a-hexahydro-3aH-benzo[f]benzofuran-2-one	232.35	3.31	0	2	53.29	1.3	1.42	0.15	0.3	7.93
MOL010819	Ambrosin	246.33	2.53	0	3	68.54	0.64	0.8	0.17	0.39	2.8
MOL010820	Methyl betulinate	470.81	6.77	1	3	16.81	0.96	0.55	0.76	0.25	
MOL010821	Caryophyleine-(I3)	204.39	4.7	0	0	35.37	1.84	2.04	0.09	0.26	7.44
MOL010822	ZINC04026824	220.39	3.08	1	1	68.97	1.23	1.39	0.12	0.24	4.96
MOL010823	ETA	61.1	-1.19	3	2	57.26	-0.17	-1.58	0	0.15	11.74
MOL010824	2-[(2R,4aR,8aS)-4a-methyl-8-methylene-decalin-2-yl]acrylic acid	234.37	3.93	1	2	30.83	1.02	1.03	0.11	0.31	0.76
MOL010825	Costunolide	232.35	4.02	0	2	29.07	1.28	1.42	0.11	0.36	
MOL010826	costuslactone	232.35	4.02	0	2	60.48	1.3	1.44	0.11	0.35	5.25
MOL010827	1,2-Dimethylcyclohexene	110.22	3.19	0	0	45.06	1.82	2.09	0.01	0.22	11.38
MOL010828	cynaropicrin	346.41	1.6	2	6	67.5	-0.1	-0.83	0.38	0.34	1.38
MOL010829	4-[(1R)-2,6,6-trimethyl-1-cyclohex-2-enyl]butan-2-one	194.35	3.04	0	1	22.54	1.38	1.49	0.05	0.23	
MOL010830	dihydrocostus lactone	234.37	4.01	0	2	62.3	1.27	1.38	0.11	0.32	4.71

MOL010831	Ermanthin	230.33	3.23	0	2	61.55	1.28	1.43	0.14	0.37	2.5
MOL010832	Eremophilene	204.39	4.75	0	0	37.31	1.83	2.14	0.08	0.25	7.47
MOL010833	eudesma-5,11(13)-dien-8,12-olide	232.35	3.31	0	2	52.71	1.27	1.4	0.15	0.31	7.53
MOL010834	Aceteugenol	206.26	2.59	0	3	33.87	1.15	1.12	0.06	0.27	1.35
MOL010835	humulane-1,6-dien-3-ol	220.44	5.54	0	0	25.76	1.85	2.04	0.07	0.25	
MOL010836	Isoalantolactone	232.35	3.37	0	2	53.43	1.28	1.42	0.15	0.33	7.95
MOL010837	isodihydrocostunolide	236.34	3.35	0	3	43.44	0.73	0.78	0.12	0.34	5.87
MOL010838	3-Epizaluzanin C	246.33	2.18	1	3	48.97	0.54	0.12	0.16	0.35	-0.34
MOL010839	lappadilactone	494.68	4.27	1	6	38.56	-0.12	-0.62	0.73	0.35	9.35
MOL010840	(3aS,6E,10Z,11aR)-6-methyl-3-methylidene-10-[[(2R,3R,4S,5S,6R)-3,4,5-trihydroxy-6-(hydroxymethyl)oxan-2-yl]oxymethyl]-3a,4,5,8,9,11a-	410.51	1.18	4	8	12.11	-0.72	-1.19	0.6	0.29	
MOL010841	picriside B_qt	218.32	3.58	0	2	70.68	1.25	1.38	0.1	0.36	3.91
MOL010842	Naphthol[1,2-b]furan-2(3H)-one,decahydro-6-hydroxy-5a-methyl-3,9-bis(methylene)-,[3as-(3aalpha,5abeta,6beta,9aalpha,9bbeta)]	248.35	2.33	1	3	40.37	0.57	0.17	0.17	0.34	6.89
MOL010843	Balchanin	248.35	2.28	1	3	36.48	0.59	0.2	0.17	0.32	6.93
MOL010844	spathulenol	220.39	3.01	1	1	80.32	1.28	1.33	0.12	0.27	12.1
MOL000117	Cymol	134.24	3.51	0	0	27.2	1.86	2.1	0.02	0.34	
MOL002085	alpha-Cubebene	204.39	4.17	0	0	16.73	1.83	2.1	0.11	0.25	
MOL000118	(L)-alpha-Terpineol	154.28	2.42	1	1	48.8	1.39	1.72	0.03	0.27	11.35
MOL001201	(1R,5R,7S)-4,7-dimethyl-7-(4-methylpent-3-enyl)bicyclo[3.1.1]hept-3-	204.39	4.7	0	0	16.23	1.86	1.96	0.09	0.27	
MOL000125	(-)-alpha-Pinene	136.26	2.87	0	0	46.25	1.85	2.3	0.05	0.25	11.42
MOL000131	EIC	280.5	6.39	1	2	41.9	1.16	0.9	0.14	0.25	7.5
MOL001390	49070_FLUKA	222.41	3.2	1	1	85.51	1.29	1.46	0.12	0.24	12.06
MOL001393	myristic acid	228.42	5.46	1	2	21.18	1.07	0.99	0.07	0.19	
MOL001551	Trochol	442.8	6.31	2	2	15.48	0.84	0.35	0.78	0.24	
MOL001620	Pentadecene	210.45	6.82	0	0	17.72	1.84	1.97	0.05	0.21	
MOL001729	Crysophanol	254.25	2.76	2	4	18.64	0.62	-0.2	0.21	0.44	
MOL001732	IFP	92.11	-1.41	3	3	72.87	-0.97	-2.63	0.01	0.2	11.45
MOL000175	cyperene	204.39	4.32	0	0	51.1	1.81	2.13	0.11	0.23	12.57
MOL000019	D-Camphene	136.26	2.93	0	0	34.98	1.81	2.19	0.04	0	11.29
MOL000193	(Z)-caryophyllene	204.39	4.75	0	0	30.29	1.82	2.15	0.09	0.28	8
MOL000197	Myrcene	136.26	3.69	0	0	24.96	1.84	1.98	0.02	0.37	
MOL000198	(R)-linalool	154.28	2.74	1	1	39.8	1.33	1.36	0.02	0.32	6.48
MOL000200	(S)-(+)-alpha-Phellandrene	136.26	3.25	0	0	27.9	1.87	2.2	0.02	0.29	
MOL000202	Moslene	136.26	3.45	0	0	33.02	1.88	2.05	0.02	0.27	11.08
MOL002028	(+)-beta-Phellandrene	136.26	3.31	0	0	40.3	1.83	2.2	0.02	0.3	11.28

MOL000204	-cis-.beta.-Elemene diastereomer	204.39	4.79	0	0	28.62	1.85	2.09	0.06	0.33	
MOL000208	()-Aromadendrene	204.39	4.22	0	0	55.74	1.81	2.06	0.1	0.26	11.84
MOL002092	Antioxidant No. 33	206.36	4.36	1	1	26.74	1.68	1.81	0.06	0.28	
MOL000211	Mairin	456.78	6.52	2	3	55.38	0.73	0.22	0.78	0.26	8.87
MOL002117	β-sesquiphellandrene	204.39	5.14	0	0	23.68	1.89	2.02	0.06	0.3	
MOL002122	(Z)-Ligustilide	188.24	3	0	2	53.72	1.3	1.25	0.07	0.38	5.61
MOL002138	p-Cymen-8-ol	150.24	2.29	1	1	32.26	1.33	1.35	0.03	0.35	11.04
MOL002186	Aromadendrene oxide 2	220.39	3.08	0	1	65.1	1.56	1.82	0.14	0.2	1.94
MOL001955	Heriguard	354.34	-0.42	6	9	11.93	-1.03	-1.71	0.33	0.37	
MOL002361	Terragon	148.22	2.82	0	1	36.59	1.72	1.83	0.03	0.34	1.79
MOL000024	alpha-humulene	204.39	5.04	0	0	22.98	1.88	2.08	0.06	0	
MOL000244	()-Borneol	154.28	1.98	1	1	81.8	1.22	1.47	0.05	0.24	11.36
MOL002504	Peruviol	222.41	4.56	1	1	29.61	1.47	1.44	0.06	0.29	
MOL000259	o-Thymol	150.24	3.24	1	1	43.28	1.58	1.71	0.03	0.33	11.29
MOL000263	oleanolic acid	456.78	6.42	2	3	29.02	0.59	0.07	0.76	0.25	
MOL000264	Tereben	136.26	3.64	0	0	29.62	1.86	2.11	0.02	0.28	
MOL000267	beta-Citronellol	156.3	3.05	1	1	38.89	1.2	1.14	0.02	0.24	5.21
MOL002676	10482-53-8	232.45	6.4	0	0	44.06	1.87	1.98	0.08	0.27	10.6
MOL002697	junipene	204.39	4.18	0	0	44.07	1.82	2.14	0.11	0.25	12.2
MOL000027	alpha-Curcumene	202.37	5.34	0	0	4.68	1.93	1.99	0.06	0	
MOL002850	butylated hydroxytoluene	220.39	4.85	1	1	40.02	1.75	1.8	0.07	0.3	10.36
MOL003028	Eudesmol	224.43	4.1	1	1	35.38	1.28	1.41	0.09	0.23	5.82
MOL003180	widdrene	204.39	4.08	0	0	53.81	1.85	2.19	0.12	0.25	-1.56
MOL000032	beta-Eudesmol	222.41	3.72	1	1	26.09	1.32	1.38	0.1	0	
MOL000034	2-[(1R,3S,4S)-3-isopropenyl-4-methyl-4-vinylcyclohexyl]propan-2-ol	222.41	3.7	1	1	19.03	1.37	1.46	0.07	0	
MOL000347	Syrigin	372.41	-0.51	5	9	14.64	-1.01	-1.81	0.32	0.23	
MOL000348	4-[(Z)-3-hydroxyprop-1-enyl]-2,6-dimethoxyphenol	210.25	1.39	2	4	49.15	0.56	0.03	0.06	0.2	3.88
MOL003484	PEY	178.24	3.65	0	0	25.7	1.88	1.79	0.1	0.52	
MOL002363	beta-Ionone	192.33	3.22	0	1	20.63	1.41	1.47	0.05	0.3	
MOL003541	()-alpha-Longipinene	204.39	4.12	0	0	57.47	1.81	2.05	0.12	0	12.13
MOL000359	sitosterol	414.79	8.08	1	1	36.91	1.32	0.87	0.75	0.22	5.37
MOL000388	gamma-aminobutyric acid	103.14	-0.62	3	3	24.09	-0.26	-0.57	0.01	0	
MOL000449	Stigmasterol	412.77	7.64	1	1	43.83	1.44	1	0.76	0.22	5.57
MOL000475	anethole	148.22	2.77	0	1	32.49	1.75	1.81	0.03	0	1.68
MOL000479	Farnesene	204.39	5.52	0	0	17.42	1.95	2.21	0.05	0	
MOL002003	(-)-Caryophyllene oxide	220.39	3.52	0	1	32.67	1.58	1.76	0.13	0.28	6.51
MOL000612	(-)-alpha-cedrene	204.39	4.12	0	0	55.56	1.81	2.16	0.1	0.24	4.82
MOL000066	alloaromadedrene	204.39	4.22	0	0	53.46	1.83	2.1	0.1	0	12.51
MOL000666	hexanal	100.18	1.85	0	1	55.71	1.25	1.52	0.01	0.23	10.96
MOL000668	PENTYLFURAN	138.23	3.12	0	1	54.59	1.72	1.94	0.02	0.1	-1.96

MOL000677	(1R,4R)-4-isopropyl-1,6-	202.37	5.04	0	0	17.47	1.86	1.98	0.08	0.29	
MOL000716	trans-2-nonenal	140.25	3.2	0	1	19.18	1.37	1.59	0.02	0.27	
MOL000724	Geranylacetone	194.35	3.62	0	1	18.66	1.5	1.55	0.04	0.29	
MOL007460	Cyclooctadiene	108.2	2.76	0	0	42.82	1.81	2.2	0.01	0.27	11.38
MOL007914	473-08-5	218.37	3.81	0	1	35.53	1.42	1.52	0.1	0.3	5.07
MOL000875	Cedrol	222.41	3.16	1	1	16.23	1.35	1.46	0.12	0.24	
MOL008941	(E)-9-Isopropyl-6-methyl-5,9-decadiene-2-one	208.38	3.93	0	1	60.25	1.45	1.44	0.05	0.31	4.61
MOL000922	(R)-p-Menth-1-en-4-ol	154.28	2.55	1	1	32.16	1.33	1.52	0.03	0.26	11.39
MOL009489	trans-4-(Isopropyl)-1-methylcyclohex-2-en-1-ol	154.28	2.36	1	1	47.06	1.24	1.5	0.03	0.27	11.03
MOL000968	beta-Bisabolene	204.39	5.33	0	0	29.59	1.88	2.13	0.06	0.29	
MOL000991	cinnamaldehyde	132.17	1.95	0	1	31.99	1.35	1.48	0.02	0.48	4.73

Radix Glycyrrhizae (RG)

Mol ID	Molecule Name	MW	AlogP	Hdon	Hacc	OB (%)	Caco-2	BBB	DL	FASA-	HL
MOL000105	protocatechuic acid	154.13	0.9	3	4	25.37	0.1	-0.17	0.04	0.43	
MOL001097	o-xylene	106.18	2.8	0	0	45.55	1.85	2.08	0.01	0.41	11.86
MOL001098	m-xylene	106.18	2.8	0	0	47.43	1.83	2.02	0.01	0.39	11.82
MOL001099	p-xylene	106.18	2.8	0	0	48.74	1.83	2.06	0.01	0.39	11.71
MOL000118	(L)-alpha-Terpineol	154.28	2.42	1	1	48.8	1.39	1.72	0.03	0.27	11.35
MOL000012	Arachic acid	312.6	8.19	1	2	16.66	1.18	1.09	0.19	0.18	
MOL001484	Inermine	284.28	2.44	1	5	75.18	0.89	0.4	0.54	0.3	11.72
MOL001543	Vicenin-2	594.57	-2.45	11	15	3.42	-3.15	-3.87	0.78	0.32	
MOL001599	α-cubebol	208.38	3.28	1	1	64.81	1.32	1.43	0.09	0.26	7.24
MOL001696	Morusin	420.49	4.94	3	6	11.52	0.51	-0.22	0.76	0.31	
MOL001737	ICO	161.17	1.73	2	2	33.86	0.82	0.63	0.05	0.3	-7.7
MOL001789	isoliquiritigenin	256.27	2.9	3	4	85.32	0.44	-0.41	0.15	0.46	17.66
MOL001792	DFV	256.27	2.57	2	4	32.76	0.51	-0.29	0.18	0.42	17.89
MOL001850	Izoforon	138.23	2.06	0	1	44.98	1.28	1.66	0.03	0.32	11.4
MOL000211	Mairin	456.78	6.52	2	3	55.38	0.73	0.22	0.78	0.26	8.87
MOL002137	OCT	114.26	4.02	0	0	29.72	1.78	2.02	0.01	0.19	
MOL002166	ISOHEPTANE	100.23	3.36	0	0	59.94	1.81	2.25	0.01	0.22	10.67
MOL002198	Heptan	100.23	3.57	0	0	41.8	1.77	2.04	0	0.2	10.36
MOL002311	Glycyrol	366.39	4.85	2	6	90.78	0.71	-0.2	0.67	0.28	9.85
MOL000239	Jaranol	314.31	2.09	2	6	50.83	0.61	-0.22	0.29	0.29	15.5
MOL002547	21987_FLUKA	136.26	2.68	0	0	40.92	1.84	2.14	0.04	0.26	11.22
MOL002565	Medicarpin	270.3	2.66	1	4	49.22	1	0.53	0.34	0.31	8.46
MOL000263	oleanolic acid	456.78	6.42	2	3	29.02	0.59	0.07	0.76	0.25	
MOL002678	EB	106.18	2.77	0	0	49.38	1.83	2.15	0.01	0.38	11.76
MOL002693	nicotiflorin	594.57	-1.18	9	15	3.64	-1.77	-2.55	0.73	0.3	
MOL002844	Pinocembrin	256.27	2.57	2	4	64.72	0.61	0.12	0.18	0.43	17.96

MOL002850	butylated hydroxytoluene	220.39	4.85	1	1	40.02	1.75	1.8	0.07	0.3	10.36
MOL002943	BuOH	74.14	0.97	1	1	22.02	0.94	1.05	0	0.21	
MOL003218	Neouralenol	370.38	3.36	5	7	12.76	0.24	-0.75	0.46	0.37	
MOL000354	isorhamnetin	316.28	1.76	4	7	49.6	0.31	-0.54	0.31	0.32	14.34
MOL000359	sitosterol	414.79	8.08	1	1	36.91	1.32	0.87	0.75	0.22	5.37
MOL003656	Lupiwighteone	338.38	3.92	3	5	51.64	0.68	-0.23	0.37	0.36	15.63
MOL003662	7,4'-Dihydroxyflavone	254.25	2.6	2	4	19.18	0.56	-0.16	0.18	0.43	
MOL003686	Narcissoside	624.6	-1.19	9	16	5.09	-2.14	-2.74	0.65	0	
MOL003896	7-Methoxy-2-methyl isoflavone	266.31	3.36	0	3	42.56	1.16	0.56	0.2	0.33	16.89
MOL000392	formononetin	268.28	2.58	1	4	69.67	0.78	0.02	0.21	0	17.04
MOL003985	2-Caren-10-al	150.24	2.04	0	1	44.74	1.37	1.63	0.05	0.28	11.47
MOL000040	Scopoletol	192.18	1.62	1	4	27.77	0.71	0.3	0.08	0	
MOL000415	rutin	610.57	-1.45	10	16	3.2	-1.93	-2.75	0.68	0	
MOL000417	Calycosin	284.28	2.32	2	5	47.75	0.52	-0.43	0.24	0	17.1
MOL000422	kaempferol	286.25	1.77	4	6	41.88	0.26	-0.55	0.24	0	14.74
MOL004328	naringenin	272.27	2.3	3	5	59.29	0.28	-0.37	0.21	0.4	16.98
MOL000437	Hirsutrin	464.41	-0.59	8	12	1.86	-1.66	-2.31	0.77	0	
MOL000445	8-Prenylwighteone	406.51	5.78	3	5	23.22	0.93	0	0.54	0.33	
MOL004589	Methylheptane	114.26	3.82	0	0	28.65	1.79	2.12	0.01	0.21	
MOL000467	Castanin	298.31	2.57	1	5	23.54	0.77	-0.1	0.27	0	
MOL004723	beta-Terpinene	136.26	3.5	0	0	42.29	1.85	2.12	0.02	0.29	11.21
MOL000475	anethole	148.22	2.77	0	1	32.49	1.75	1.81	0.03	0	1.68
MOL004801	2',7-Dihydroxy-4'-methoxyisoflavan-7-O-β-d-glucopyranoside	434.48	0.59	5	9	10.46	-1.02	-1.82	0.73	0.31	
MOL004802	(E)-1-butoxyhex-2-ene	156.3	3.31	0	1	41.72	1.5	1.56	0.02	0	6.68
MOL004803	3-Hydroxyglabrol	408.53	5.73	3	5	4.73	0.48	-0.31	0.58	0.33	
MOL004804	18beta-glycyrrhetinic acid	470.76	5.49	2	4	22.05	0.1	-0.53	0.74	0	
MOL004805	(2S)-2-[4-hydroxy-3-(3-methylbut-2-enyl)phenyl]-8,8-dimethyl-2,3-dihydropyrano[2,3-f]chromen-4-one	390.51	5.48	1	4	31.79	1	0.25	0.72	0.35	14.82
MOL004806	euchrenone	406.56	6.35	1	4	30.29	1.09	0.39	0.57	0	15.89
MOL004807	glucuronic acid	194.16	-2.31	5	7	46.18	-2.05	-4.58	0.06	0	11.13
MOL004808	glyasperin B	370.43	4.02	3	6	65.22	0.47	-0.09	0.44	0	16.1
MOL004809	glyasperin E	444.51	6.41	2	6	4.12	0.69	-0.41	0.75	0	
MOL004810	glyasperin F	354.38	2.97	3	6	75.84	0.43	-0.15	0.54	0	15.64
MOL004811	Glyasperin C	356.45	4.73	3	5	45.56	0.71	0.07	0.4	0	3.13
MOL004812	glyasperins D	370.48	4.99	2	5	29.91	0.89	0.25	0.43	0	
MOL004813	glyasperins Z	340.45	5	2	4	4.17	1.09	0.53	0.36	0	
MOL004814	Isotrifoliol	298.26	2.99	2	6	31.94	0.53	-0.25	0.42	0	7.91
MOL004815	(E)-1-(2,4-dihydroxyphenyl)-3-(2,2-dimethylchromen-6-yl)prop-2-en-1-one	322.38	3.96	2	4	39.62	0.66	-0.12	0.35	0	16.16

MOL004816	(2R)-1-[2,4-dihydroxy-5-(3-methylbut-2-enyl)phenyl]-2-hydroxy-3-[4-hydroxy-3-(3-methylbut-2-enyl)phenyl]propan-1-	410.55	5.96	4	5	1.06	0.33	-0.35	0.48	0	
MOL004817	kanzonols K	436.54	5.76	3	6	0.97	0.76	0.05	0.66	0	
MOL004818	kanzonols L	490.64	7.04	3	6	0.98	0.7	0.11	0.78	0	
MOL004819	kanzonols T	440.53	4.41	5	7	17.87	0.05	-0.66	0.67	0	
MOL004820	kanzonols W	336.36	3.63	2	5	50.48	0.63	0.04	0.52	0	0.15
MOL004821	kanzonols X	394.55	6.61	3	4	7.56	1.1	0.34	0.56	0	
MOL004822	(E)-1-(2,4-dihydroxyphenyl)-3-[4-hydroxy-3-(3-methylbut-2-enyl)phenyl]prop-2-en-1-one	324.4	4.76	3	4	1.04	0.55	-0.22	0.27	0	
MOL004823	licoagropin	320.46	5.77	0	2	27.14	1.63	1.08	0.51	0	
MOL004824	(2S)-6-(2,4-dihydroxyphenyl)-2-(2-hydroxypropan-2-yl)-4-methoxy-2,3-dihydrofuro[3,2-g]chromen-7-one	384.41	2.96	3	7	60.25	0	-0.76	0.63	0	4.31
MOL004825	glyinflanin A	408.53	6.05	4	5	1.06	0.69	0.03	0.48	0.38	
MOL005812	naringin	580.59	-0.46	8	14	6.92	-1.99	-3	0.78	0.31	
MOL004827	Semilicoisoflavone B	352.36	2.85	3	6	48.78	0.45	-0.33	0.55	0	17.02
MOL004828	Glepidotin A	338.38	3.9	3	5	44.72	0.79	0.06	0.35	0	16.09
MOL004829	Glepidotin B	340.4	3.88	3	5	64.46	0.46	-0.09	0.34	0	15.98
MOL004830	Octadiene	110.22	3.19	0	0	34.53	1.81	2.07	0.01	0	3.75
MOL004831	(E)-1-[2,4-dihydroxy-3-(3-methylbut-2-enyl)phenyl]-3-[4-hydroxy-3-(3-methylbut-2-enyl)phenyl]prop-2-en-1-	392.53	6.61	3	4	1.02	0.81	0.01	0.45	0.37	
MOL004832	WLN: 4OVR	178.25	3.01	0	2	48.41	1.31	1.42	0.04	0	7.28
MOL004833	Phaseolinisoflavan	324.4	3.95	2	4	32.01	1.01	0.46	0.45	0	2.66
MOL004834	3-(2-hydroxy-4-methoxyphenyl)-2H-chromen-7-ol	270.3	2.96	2	4	4.66	0.89	0.16	0.21	0	
MOL004835	Glypallichalcone	284.33	3.4	1	4	61.6	0.76	0.23	0.19	0	17.01
MOL004836	echinatin	270.3	3.15	2	4	66.58	0.38	-0.18	0.17	0	19.56
MOL004837	Karenzu DK2	224.27	3.16	0	2	62.26	0.94	0.64	0.1	0	33.72
MOL004838	8-(6-hydroxy-2-benzofuranyl)-2,2-dimethyl-5-chromenol	308.35	4.2	2	4	58.44	1	0.34	0.38	0.34	8.71
MOL004839	(1S,2S)-1,2-dimethylcyclopentane	98.21	2.79	0	0	41.78	1.78	2.26	0.01	0	10.93
MOL004840	Liconeolignan	354.43	5.23	2	5	4.41	1	0.04	0.4	0	
MOL004841	Licochalcone B	286.3	2.88	3	5	76.76	0.47	-0.46	0.19	0	17.02
MOL004842	licochalcone C	338.43	5.01	2	4	4.44	0.63	0.05	0.29	0	
MOL004843	licochalconeD	354.43	4.74	3	5	1.01	0.47	0.06	0.34	0	
MOL004844	glabrol	392.53	6.28	2	4	4.25	0.84	0.06	0.54	0	
MOL004845	apioglycyrrhizin	779.03	2.54	7	14	17.8	-1.91	-2.67	0.14	0	
MOL004846	apioglycyrrhizin_qt	470.76	5.49	2	4	23.73	0.1	-0.56	0.74	0	
MOL004847	2,2-DIMETHYLPENTANE	100.23	3.11	0	0	55.33	1.79	2.11	0.01	0	11

MOL004848	licochalcone G	354.43	4.35	3	5	49.25	0.64	-0.04	0.32	0.35	15.75
MOL004849	3-(2,4-dihydroxyphenyl)-8-(1,1-dimethylprop-2-enyl)-7-hydroxy-5-methoxy-coumarin	368.41	4.03	3	6	59.62	0.4	-0.23	0.43	0	0.69
MOL004850	liquoric acid	484.74	4.05	2	5	25.44	-0.01	-0.52	0.55	0	
MOL004851	Licoflavone	322.38	4.46	2	4	18.75	0.82	-0.31	0.33	0	
MOL004852	7-hydroxy-2-[4-hydroxy-3-(3-methylbut-2-enyl)phenyl]-6-(3-methylbut-2-enyl)chromone	390.51	6.32	2	4	4.44	0.88	-0.17	0.56	0	
MOL004853	Licoflavonol	354.38	3.63	4	6	8.75	0.49	-0.3	0.4	0	
MOL004385	Yinyanghuo D	338.38	4.19	3	5	13.99	0.61	-0.53	0.38	0	
MOL004855	Licoricone	382.44	4.16	2	6	63.58	0.53	-0.14	0.47	0	16.37
MOL004856	Gancaonin A	352.41	4.17	2	5	51.08	0.8	0.13	0.4	0	16.82
MOL004857	Gancaonin B	368.41	3.91	3	6	48.79	0.58	-0.1	0.45	0	16.49
MOL004858	Gancaonin C	354.38	2.83	4	6	2.87	0.14	-0.87	0.42	0	
MOL004859	2,3-dimethylhexane	114.26	3.61	0	0	46.24	1.78	2.16	0.01	0	11.01
MOL000486	Prunetin	284.28	2.32	2	5	5.41	0.65	-0.25	0.24	0	
MOL004860	licorice glycoside E	693.71	1.59	7	14	32.89	-2.06	-2.8	0.27	0.31	25.39
MOL004861	Gancaonin D	384.41	2.81	4	7	2.72	-0.11	-0.93	0.51	0	
MOL004862	(2R)-2-[3,4-dihydroxy-5-(3-methylbut-2-enyl)phenyl]-5,7-dihydroxy-8-(3-methylbut-2-enyl)chroman-4-one	424.53	5.74	4	6	1.21	0.51	-0.35	0.63	0	
MOL004863	3-(3,4-dihydroxyphenyl)-5,7-dihydroxy-8-(3-methylbut-2-enyl)chromone	354.38	3.65	4	6	66.37	0.52	-0.13	0.41	0	15.81
MOL004864	5,7-dihydroxy-3-(4-methoxyphenyl)-8-(3-methylbut-2-enyl)chromone	352.41	4.17	2	5	30.49	0.9	0.21	0.41	0	14.99
MOL004865	5,7-dihydroxy-3-(2-hydroxy-4-methoxy-phenyl)-6-(3-methylbut-2-	368.41	3.91	3	6	2.47	0.58	-0.14	0.45	0	
MOL004866	2-(3,4-dihydroxyphenyl)-5,7-dihydroxy-6-(3-methylbut-2-enyl)chromone	354.38	3.92	4	6	44.15	0.48	-0.28	0.41	0	16.77
MOL004867	Gancaonin P	370.38	3.36	5	7	1.41	0.27	-0.4	0.45	0	
MOL004868	Gancaonin Q	406.51	6.05	3	5	8.98	0.86	-0.01	0.6	0	
MOL004869	Gancaonin R	382.54	6.9	4	4	1.26	1.03	0.25	0.37	0	
MOL004870	Gancaonin S	382.54	6.9	4	4	1.26	0.98	0.18	0.38	0	
MOL004871	(3S)-2,3-dimethylpentane	100.23	3.16	0	0	35.57	1.78	2.23	0.01	0	11.19
MOL004872	gancaonin T	398.54	5.45	4	5	1.04	0.5	-0.43	0.53	0	
MOL004873	Gancaonin U	380.52	6.59	4	4	14.53	1.08	0.6	0.53	0	
MOL004874	Gancaonin V	312.39	4.74	4	4	1.24	0.73	0.04	0.34	0	
MOL004875	3-[4,6-dihydroxy-2-methoxy-3-(3-methylbut-2-enyl)phenyl]-7-hydroxy-chromone	368.41	3.91	3	6	2.47	0.43	-0.3	0.44	0	
MOL004876	Glycyram	823.04	2.42	8	16	19.62	-2.66	-2.86	0.11	0	

MOL004877	Licoricidin	424.58	6.59	3	5	0.99	0.96	0.3	0.62	0	
MOL004878	Glycycoumarin	368.41	4.42	3	6	23.56	0.52	-0.24	0.44	0	
MOL004879	Glycyrin	382.44	4.67	2	6	52.61	0.59	-0.13	0.47	0	1.31
MOL004880	5,6,7,8-Tetrahydro-2,4-	161.27	2.97	0	1	49.77	1.64	1.7	0.05	0	-2.84
MOL004881	(E)-1-[2,4-dihydroxy-3-(3-methylbut-2-enyl)phenyl]-3-(2,4-dihydroxyphenyl)prop-2-en-1-one	340.4	4.49	4	5	1.36	0.5	-0.22	0.3	0.38	
MOL004882	Licocoumarone	340.4	4.98	3	5	33.21	0.84	0.06	0.36	0	9.66
MOL004883	Licoisoflavone	354.38	3.65	4	6	41.61	0.37	-0.27	0.42	0	16.09
MOL004884	Licoisoflavone B	352.36	2.85	3	6	38.93	0.46	-0.18	0.55	0	15.73
MOL004885	licoisoflavanone	354.38	2.97	3	6	52.47	0.39	-0.22	0.54	0	15.67
MOL004886	licorice-saponin C2	807.04	3.1	8	15	59.66	-2.28	-2.89	0.11	0	3.56
MOL004887	licorice-saponin C2_qt	454.76	6.17	2	3	17.33	0.48	-0.3	0.76	0	
MOL004888	licorice-saponin F3	983.18	1.32	10	21	17.68	-2.82	-3.47	0.03	0	
MOL004889	licorice-saponin F3_qt	454.76	5.93	1	3	27.53	0.74	0.07	0.64	0	
MOL004890	(4S)-2,4-dimethylhexane	114.26	3.61	0	0	37.13	1.77	2.19	0.01	0	10.95
MOL004891	shinpterocarpin	322.38	3.46	1	4	80.3	1.1	0.68	0.73	0.32	6.5
MOL004892	licorice-saponin G2	839.04	1.33	9	17	6.39	-2.01	-2.84	0.11	0	
MOL004893	licorice-saponin G2_qt	486.76	4.4	3	5	22.78	-0.27	-0.74	0.72	0	
MOL004894	licorice-saponin H2	823.04	2.42	8	16	44.37	-2.08	-2.83	0.11	0	5.09
MOL004895	licorice-saponin H2_qt	470.76	5.49	2	4	22.91	0.01	-0.48	0.74	0	
MOL004896	licorice-saponin J2	825.06	2.26	9	16	6.25	-2.25	-2.84	0.11	0	
MOL004897	licorice-saponin J2_qt	472.78	5.33	3	4	28.3	0.04	-0.82	0.74	0	
MOL004898	(E)-3-[3,4-dihydroxy-5-(3-methylbut-2-enyl)phenyl]-1-(2,4-dihydroxyphenyl)prop-2-en-1-one	340.4	4.49	4	5	46.27	0.41	-0.4	0.31	0.43	15.24
MOL004899	licorice-saponin B2	809.06	3.35	8	15	58.55	-2.4	-2.96	0.11	0.3	4.24
MOL004900	licorice-saponin K2	823.04	2.01	9	16	7.82	-2.64	-3.25	0.11	0	
MOL004901	licorice-saponin K2_qt	470.76	5.08	3	4	27.79	0.05	-0.67	0.75	0	
MOL004902	glycyrrhetol	456.78	5.28	2	3	14.66	0.36	-0.26	0.75	0	
MOL004903	liquiritin	418.43	0.66	5	9	65.69	-1.06	-1.93	0.74	0	17.96
MOL004904	licopyranocoumarin	384.41	3.04	3	7	80.36	0.13	-0.62	0.65	0	0.08
MOL004905	3,22-Dihydroxy-11-oxo-delta(12)-oleanene-27-alpha-methoxycarbonyl-29-oic acid	512.75	4.37	1	6	34.32	-0.06	-0.75	0.55	0	3.56
MOL004906	Hispaglabridin B	390.51	5	1	4	22.94	1.18	0.62	0.88	0.31	
MOL004907	Glyzaglabrin	298.26	2.1	2	6	61.07	0.34	-0.2	0.35	0	21.2
MOL004908	Glabridin	324.4	3.95	2	4	53.25	0.97	0.36	0.47	0	0.03
MOL004909	glabrolide	468.74	5	1	4	17.46	0.29	-0.48	0.61	0	
MOL004910	Glabranin	324.4	4.42	2	4	52.9	0.97	0.31	0.31	0	16.24
MOL004911	Glabrene	322.38	3.77	2	4	46.27	0.99	0.04	0.44	0	3.63
MOL004912	Glabrone	336.36	3.12	2	5	52.51	0.59	-0.11	0.5	0	16.09

MOL004913	1,3-dihydroxy-9-methoxy-6-benzofurano[3,2-c]chromenone	298.26	2.99	2	6	48.14	0.48	-0.19	0.43	0	8.87
MOL004914	1,3-dihydroxy-8,9-dimethoxy-6-benzofurano[3,2-c]chromenone	328.29	2.98	2	7	62.9	0.4	-0.34	0.53	0	9.32
MOL004915	Eurycarpin A	338.38	3.92	3	5	43.28	0.43	-0.06	0.37	0	17.1
MOL004916	2-methyl-5-propyl -nonane	184.41	5.89	0	0	15.28	1.81	2.03	0.03	0	
MOL004917	glycyroside	562.57	-0.73	6	13	37.25	-1.58	-2.56	0.79	0	14.62
MOL004918	HEX	86.2	3.11	0	0	52.5	1.78	2.14	0	0	10.8
MOL004919	Sextone B	98.21	2.99	0	0	56.2	1.77	2.14	0.01	0	10.85
MOL004920	Methylcyclopentane	84.18	2.53	0	0	55.78	1.79	2.22	0.01	0	10.97
MOL004921	Docosyl caffeate	488.83	11.16	2	4	3.14	1.01	0.31	0.59	0	
MOL004922	2-methyl-6-ethyl decane	184.41	5.89	0	0	5.5	1.81	2	0.03	0	
MOL000391	Ononin	430.44	0.68	4	9	11.52	-0.74	-1.67	0.78	0	
MOL004924	(-)-Medicocarpin	432.46	0.75	4	9	40.99	-0.6	-1.34	0.95	0	13.2
MOL004925	vitexin	432.41	-0.06	7	10	3.05	-1.52	-2.41	0.71	0	
MOL004926	4H-1-Benzopyran-4-one, 2-(4-(beta-D-glucopyranosyloxy)phenyl)-2,3-dihydro-5,7-dihydroxy-, (2S)-	434.43	0.39	6	10	14.03	-1.13	-2.08	0.78	0	
MOL004927	Hispaglabridin A	392.53	5.81	2	4	14.6	1.12	0.43	0.73	0	
MOL004928	violanthin	578.57	-1.56	10	14	4.17	-2.01	-2.77	0.81	0	
MOL004929	Pentadecanol	228.47	5.99	1	1	13.73	1.3	1.06	0.06	0	
MOL004930	Uralenol	370.38	3.36	5	7	8.55	0.35	-0.57	0.46	0	
MOL004931	Uralenol-3-methylether	384.41	3.43	4	7	1.41	0.39	-0.62	0.49	0	
MOL004932	glycyrrhizin	823.04	2.42	8	16	9.06	-2.23	-3.13	0.11	0	
MOL004933	uralsaponin B	823.04	2.42	8	16	7.92	-2.6	-3.17	0.11	0	
MOL004934	Isohexane	86.2	2.9	0	0	56.13	1.77	2.08	0	0	11.03
MOL004935	Sigmoidin-B	356.4	3.89	4	6	34.88	0.42	-0.41	0.41	0	14.49
MOL004936	Uralene	384.41	3.43	4	7	11.7	0.63	-0.22	0.49	0	
MOL004937	uralenneoside	286.26	-0.49	5	8	24.96	-1.01	-1.61	0.17	0	
MOL004938	schaftoside	596.54	-1.5	10	16	7.88	-2.46	-3.48	0.75	0	
MOL004939	Nortangeretin	302.25	1.8	5	7	17.9	0.24	-0.68	0.27	0	
MOL004940	neoliquiritin	418.43	0.66	5	9	13.01	-1.08	-1.95	0.71	0	
MOL004941	(2R)-7-hydroxy-2-(4-hydroxyphenyl)chroman-4-one	256.27	2.57	2	4	71.12	0.41	-0.25	0.18	0	18.09
MOL004942	(E)-dodec-2-ene	168.36	5.4	0	0	17.74	1.83	2	0.02	0	
MOL004943	neoisoliquiritin	418.43	1	6	9	21.18	-1.41	-2.05	0.58	0	
MOL004944	Cyclobutanol, 1-ethyl-	100.18	1.32	1	1	93.23	1.13	1.35	0.02	0	11.08
MOL004945	(2S)-7-hydroxy-2-(4-hydroxyphenyl)-8-(3-methylbut-2-enyl)chroman-4-one	324.4	4.42	2	4	36.57	0.72	-0.04	0.32	0	17.95
MOL004946	2-Tetradecanone	212.42	4.99	0	1	17.71	1.46	1.6	0.05	0	
MOL004947	Isoviolanthin	578.57	-1.56	10	14	18.79	-2.43	-3.14	0.81	0	
MOL004948	Isoglycyrol	366.39	4.36	1	6	44.7	0.91	0.05	0.84	0	6.69

MOL004949	Isolicoflavonol	354.38	3.63	4	6	45.17	0.54	-0.42	0.42	0	15.55
MOL004950	isoglycycoumarin	368.41	3.92	2	6	22.09	0.55	-0.16	0.6	0	
MOL004951	Isoliquiritin	418.43	1	6	9	8.61	-1.36	-1.93	0.6	0	
MOL004952	licuraside	550.56	-0.41	8	13	5.25	-1.92	-3	0.77	0	
MOL004953	Liquiritin apioside	550.56	-0.75	7	13	29.23	-1.88	-2.69	0.82	0.31	
MOL004954	isograbrol	392.53	6.28	2	4	11.04	0.87	-0.07	0.5	0	
MOL004955	isoglabrolide	468.74	5.15	1	4	14.77	0.32	-0.16	0.62	0	
MOL004956	Isoononin	430.44	0.68	4	9	8.29	-1	-1.57	0.79	0	
MOL004957	HMO	268.28	2.58	1	4	38.37	0.79	0.25	0.21	0	16.56
MOL004958	Isoschaftoside	564.54	-1.94	10	14	17.38	-2.62	-3.36	0.83	0	
MOL004959	1-Methoxyphaseollidin	354.43	4.25	2	5	69.98	1.01	0.48	0.64	0	9.53
MOL004960	22β-acetylglabric acid	528.8	4.77	2	6	17.76	-0.21	-0.98	0.64	0	
MOL004961	Quercetin der.	330.31	1.82	3	7	46.45	0.39	-0.44	0.33	0	16.61
MOL004962	24-Hydroxy-11-deoxyglycyrrhetic acid	458.75	5.13	3	4	17.57	0.26	-0.36	0.76	0	
MOL004963	24-Hydroxyglycyrrhetic acid	486.76	4.4	3	5	24.17	-0.1	-0.8	0.72	0	
MOL004964	(Z)-1-(2,4-dihydroxyphenyl)-3-phenylprop-2-en-1-one	240.27	3.17	2	3	73.18	0.57	0.14	0.12	0	20.35
MOL004965	3'(γ,γ-dimethylallyl)-kievitone	424.53	5.63	4	6	1.21	0.51	-0.15	0.63	0	
MOL004966	3'-Hydroxy-4'-O-Methylglabridin	354.43	3.93	2	5	43.71	1	0.73	0.57	0	-0.61
MOL004967	3,3-Dimethylpentane	100.23	3.11	0	0	41.97	1.75	2.1	0.01	0	11.09
MOL004968	3,4,3',4'-Tetrahydroxy-2-	288.32	3.19	4	5	1.33	0.64	0.01	0.2	0	
MOL004969	2-Ethyl-p-xylene	134.24	3.75	0	0	20.6	1.89	2.03	0.02	0	
MOL000497	licochalcone a	338.43	4.62	2	4	40.79	0.82	-0.21	0.29	0	16.2
MOL004970	3-methylheptane	114.26	3.82	0	0	36.61	1.79	2.16	0.01	0	10.56
MOL004971	3-methylhexane	100.23	3.36	0	0	38.19	1.78	2.15	0.01	0	10.9
MOL004972	3-Methylpentane	86.2	2.9	0	0	35.77	1.76	2.14	0	0	11.1
MOL004973	3-Ethylpentane	100.23	3.36	0	0	35.74	1.79	2.16	0.01	0	11.5
MOL004974	3'-Methoxyglabridin	354.43	3.93	2	5	46.16	0.94	0.47	0.57	0	0.52
MOL004975	3β-formylglabrolide	496.75	5.33	0	5	16.36	0.26	-0.5	0.55	0	
MOL004976	Daidzein dimethyl ether	282.31	2.83	0	4	24.29	0.98	0.17	0.24	0	
MOL004977	1-Methoxyficifolinol	422.56	6.1	2	5	14.61	1.09	0.2	0.86	0	
MOL004978	2-[(3R)-8,8-dimethyl-3,4-dihydro-2H-pyrano[6,5-f]chromen-3-yl]-5-methoxyphenol	338.43	4.2	1	4	36.21	1.12	0.61	0.52	0	-0.13
MOL004979	4,2',4',alpha-	274.29	2.24	4	5	2.45	0.1	-0.4	0.16	0	
MOL004980	Inflacoumarin A	322.38	4.7	2	4	39.71	0.73	-0.24	0.33	0	2.31
MOL004981	1-(5-hydroxy-2,2-dimethylchromen-6-yl)-3-(4-hydroxyphenyl)prop-2-en-1-one	322.38	3.96	2	4	5.2	0.86	-0.01	0.34	0	
MOL004982	2,6,10-trimethyl-dodecane	144.14	-1.02	2	4	37.8	0.08	0.04	0.03	0	11.49
MOL004983	5,6,7,8-Tetrahydro-4-methylquinoline	147.24	2.69	0	1	59.18	1.63	1.79	0.04	0	11.39
MOL005015	Licoriisoflavan A	438.61	6.84	2	5	3.68	1.1	0.28	0.66	0	
MOL004985	icos-5-enoic acid	310.58	7.75	1	2	30.7	1.22	1.09	0.2	0	5.28

MOL004986	6″-O-acetylliquiritin	444.47	2.33	3	9	6.26	-0.48	-1.24	0.82	0	
MOL004987	11-deoxyglycyrrhetic acid	456.78	6.42	2	3	16.21	0.51	-0.22	0.76	0	
MOL004988	Kanzonol F	420.54	5.3	1	5	32.47	1.18	0.56	0.89	0.28	9.98
MOL004989	6-prenylated eriodictyol	356.4	3.89	4	6	39.22	0.4	-0.29	0.41	0	16.52
MOL004990	7,2',4'-trihydroxy − 5-methoxy-3 − arylcoumarin	300.28	2.56	3	6	83.71	0.24	-0.59	0.27	0	0.99
MOL004991	7-Acetoxy-2-methylisoflavone	294.32	3.15	0	4	38.92	0.74	0.16	0.26	0	17.49
MOL004992	7-hydroxy-2-methyl-3-phenyl-chromone	252.28	3.11	1	3	25.8	1	0.4	0.18	0	
MOL004993	8-prenylated eriodictyol	356.4	3.89	4	6	53.79	0.43	-0.44	0.4	0	15.7
MOL004994	12-methyltetradecanoate	256.48	5.96	0	2	17.36	1.35	1.15	0.09	0	
MOL004995	Kanzonol H	424.58	6.1	2	5	16.92	0.96	0.38	0.8	0.27	
MOL004996	gadelaidic acid	310.58	7.75	1	2	30.7	1.2	0.94	0.2	0	5.25
MOL004997	Araboglycyrrhizin	779.03	2.72	7	14	17.73	-2.46	-2.73	0.14	0	
MOL004998	Araboglycyrrhizin_qt	470.76	5.49	2	4	17.71	0.11	-0.52	0.74	0	
MOL004999	Artonin E	436.49	4.67	4	7	11.38	0.34	-0.33	0.8	0	
MOL000500	Vestitol	272.32	3.15	2	4	74.66	0.86	0.3	0.21	0	3
MOL005000	Gancaonin G	352.41	4.17	2	5	60.44	0.78	0.23	0.39	0	16.13
MOL005001	Gancaonin H	420.49	4.71	3	6	50.1	0.6	-0.14	0.78	0	16.64
MOL005002	beta-Glycyrrhetinic acid	470.76	5.49	2	4	17.41	0.19	-0.4	0.74	0	
MOL005003	Licoagrocarpin	338.43	4.51	1	4	58.81	1.23	0.61	0.58	0.27	9.45
MOL005004	Gancaonin I	354.43	5.23	2	5	21.9	0.93	0.37	0.39	0	
MOL005005	Glyasperin A	422.51	5.48	4	6	2.46	0.6	-0.26	0.63	0	
MOL005006	Glyasperins K	368.46	4.54	1	5	10.15	0.74	0.22	0.44	0	
MOL005007	Glyasperins M	368.41	3.22	2	6	72.67	0.49	-0.04	0.59	0	15.57
MOL005008	Glycyrrhiza flavonol A	370.38	2.17	4	7	41.28	-0.09	-0.81	0.6	0	13.71
MOL005009	Corylifolinin	324.4	4.76	3	4	1.04	0.81	-0.03	0.27	0	
MOL005010	Kanzonol E	388.49	5.51	1	4	5.77	0.98	0.06	0.71	0	
MOL005011	Kanzonol Z	406.51	4.93	2	5	21.77	0.5	-0.18	0.76	0	
MOL005012	Licoagroisoflavone	336.36	3.48	2	5	57.28	0.71	0.09	0.49	0	19.64
MOL005013	18α-hydroxyglycyrrhetic acid	486.76	4.55	3	5	41.16	-0.29	-0.78	0.71	0	4.96
MOL005014	Licorice glycoside A	726.74	1.81	8	16	5.95	-2.37	-3.26	0.35	0.32	
MOL005016	Odoratin	314.31	2.3	2	6	49.95	0.42	-0.24	0.3	0	16.35
MOL005017	Phaseol	336.36	4.87	2	5	78.77	0.76	-0.06	0.58	0	9.64
MOL005018	Xambioona	388.49	4.68	0	4	54.85	1.09	0.52	0.87	0	14.5
MOL005019	(2R)-7-hydroxy-2-[4-hydroxy-3-(3-methylbut-2-enyl)phenyl]chroman-4-one	324.4	4.42	2	4	5.99	0.74	-0.06	0.33	0	
MOL005020	dehydroglyasperins C	340.4	4.3	4	5	53.82	0.68	-0.12	0.37	0	2.75
MOL005021	Mipax	194.2	1.54	0	4	57.4	0.64	0.63	0.06	0	5.56
MOL000511	ursolic acid	456.78	6.47	2	3	16.77	0.67	0.07	0.75	0.26	
MOL000561	Astragalin	448.41	-0.32	7	11	14.03	-1.34	-1.97	0.74	0.34	
MOL000057	DIBP	278.38	3.92	0	4	49.63	0.85	0.68	0.13	0	3.94
MOL000668	PENTYLFURAN	138.23	3.12	0	1	54.59	1.72	1.94	0.02	0.1	-1.96

MOL000671	()-Menthol	156.3	2.78	1	1	59.33	1.27	1.42	0.03	0.22	11.03
MOL000676	DBP	278.38	4.2	0	4	64.54	0.8	0.56	0.13	0.34	5.41
MOL000703	2-heptanone	114.21	1.79	0	1	46.56	1.31	1.57	0.01	0.25	10.58
MOL000705	WLN: VH6	114.21	2.31	0	1	19.59	1.29	1.49	0.01	0.23	
MOL000098	quercetin	302.25	1.5	5	7	46.43	0.05	-0.77	0.28	0.38	14.4
