# Supplementary material for: Analysis of the Efficacy and Pharmacological Mechanisms of Action of Zhenren Yangzang Decoction on Ulcerative Colitis Using Meta-Analysis and Network Pharmacology
Source: Evid Based Complement Alternat Med. 2021 Dec 28;2021:4512755. doi: 10.1155/2021/4512755 (PMC8727130; doi:10.1155/2021/4512755)
Supplement: Supplementary Materials — Figure S1: Risk of bias graph. Figure S2: risk of bias summary. Figure S3: forest plot of comparison of serum cytokines. Figure S4: forest plot of comparison of the total syndrome score of TCM. Table S1: basic information on the active compounds in ZRYZD. Table S2: gene symbols and entrezID of active target genes. Table S3: compounds ranked by the degree in the network. Supplementary File 1: compounds of ZRYZD from TCMSP. Supplementary File 2: corresponding target genes of ZRYZD. Supplementary File 3: UC-related target genes. Supplementary File 4: GO functional enrichment analysis. Supplementary File 5: KEGG pathway enrichment analysis. Supplementary File 6: data of compound-target networks. Supplementary File 7: data of key compound-target networks. Supplementary File 8: data of PPI network. [file 4512755.f1.zip › 4512755.f1/Supplementary File 2 Corresponding target genes of ZRYZD (1).pdf]

Supplementary File 2 Corresponding target genes of ZRYZD

Corresponding target genes of active compounds

Herb	MolId	MolName	Target
Radix Paeoniae Alba (RPA)	MOL001918	paeoniflorgenone	Gamma-aminobutyric acid receptor subunit alpha-1
Radix Paeoniae Alba (RPA)	MOL001919	(3S,5R,8R,9R,10S,14S)-3,17-dihydroxy-4,4,8,10,14-pentamethyl-2,3,5,6,7,9-hexahydro-1H-cyclopenta[a]phenanthrene-15,16-dione	Progesterone receptor
Radix Paeoniae Alba (RPA)	MOL001919	(3S,5R,8R,9R,10S,14S)-3,17-dihydroxy-4,4,8,10,14-pentamethyl-2,3,5,6,7,9-hexahydro-1H-cyclopenta[a]phenanthrene-15,16-dione	Mineralocorticoid receptor
Radix Paeoniae Alba (RPA)	MOL001924	paeoniflorin	Tumor necrosis factor
Radix Paeoniae Alba (RPA)	MOL001924	paeoniflorin	Interleukin-6
Radix Paeoniae Alba (RPA)	MOL001924	paeoniflorin	Monocyte differentiation antigen CD14
Radix Paeoniae Alba (RPA)	MOL001924	paeoniflorin	Lipopolysaccharide-binding protein
Radix Paeoniae Alba (RPA)	MOL000211	Mairin	Progesterone receptor
Radix Paeoniae Alba (RPA)	MOL000358	beta-sitosterol	Progesterone receptor
Radix Paeoniae Alba (RPA)	MOL000358	beta-sitosterol	Nuclear receptor coactivator 2
Radix Paeoniae Alba (RPA)	MOL000358	beta-sitosterol	Prostaglandin G/H synthase 1
Radix Paeoniae Alba (RPA)	MOL000358	beta-sitosterol	Prostaglandin G/H synthase 2
Radix Paeoniae Alba (RPA)	MOL000358	beta-sitosterol	Heat shock protein HSP 90
Radix Paeoniae Alba (RPA)	MOL000358	beta-sitosterol	Phosphatidylinositol-4,5-bisphosphate 3-kinase catalytic subunit, gamma
Radix Paeoniae Alba (RPA)	MOL000358	beta-sitosterol	Potassium voltage-gated channel subfamily H member 2
Radix Paeoniae Alba (RPA)	MOL000358	beta-sitosterol	mRNA of PKA Catalytic Subunit C-
Radix Paeoniae Alba (RPA)	MOL000358	beta-sitosterol	Dopamine D1 receptor
Radix Paeoniae Alba (RPA)	MOL000358	beta-sitosterol	Muscarinic acetylcholine receptor M3
Radix Paeoniae Alba (RPA)	MOL000358	beta-sitosterol	Muscarinic acetylcholine receptor M1
Radix Paeoniae Alba (RPA)	MOL000358	beta-sitosterol	Sodium channel protein type 5 subunit alpha
Radix Paeoniae Alba (RPA)	MOL000358	beta-sitosterol	Gamma-aminobutyric-acid receptor alpha-2 subunit
Radix Paeoniae Alba (RPA)	MOL000358	beta-sitosterol	Muscarinic acetylcholine receptor M4
Radix Paeoniae Alba (RPA)	MOL000358	beta-sitosterol	CGMP-inhibited 3',5'-cyclic phosphodiesterase A
Radix Paeoniae Alba (RPA)	MOL000358	beta-sitosterol	5-hydroxytryptamine 2A receptor
Radix Paeoniae Alba (RPA)	MOL000358	beta-sitosterol	Gamma-aminobutyric-acid receptor alpha-5 subunit
Radix Paeoniae Alba (RPA)	MOL000358	beta-sitosterol	Alpha-1A adrenergic receptor
Radix Paeoniae Alba (RPA)	MOL000358	beta-sitosterol	Gamma-aminobutyric-acid receptor alpha-3 subunit
Radix Paeoniae Alba (RPA)	MOL000358	beta-sitosterol	Muscarinic acetylcholine receptor M2
Radix Paeoniae Alba (RPA)	MOL000358	beta-sitosterol	Alpha-1B adrenergic receptor
Radix Paeoniae Alba (RPA)	MOL000358	beta-sitosterol	Beta-2 adrenergic receptor
Radix Paeoniae Alba (RPA)	MOL000358	beta-sitosterol	Neuronal acetylcholine receptor subunit alpha-2
Radix Paeoniae Alba (RPA)	MOL000358	beta-sitosterol	Sodium-dependent serotonin transporter
Radix Paeoniae Alba (RPA)	MOL000358	beta-sitosterol	Mu-type opioid receptor
Radix Paeoniae Alba (RPA)	MOL000358	beta-sitosterol	Gamma-aminobutyric acid receptor subunit alpha-1
Radix Paeoniae Alba (RPA)	MOL000358	beta-sitosterol	Neuronal acetylcholine receptor protein, alpha-7 chain
Radix Paeoniae Alba (RPA)	MOL000358	beta-sitosterol	Cytochrome P450-cam
Radix Paeoniae Alba (RPA)	MOL000358	beta-sitosterol	Apoptosis regulator Bcl-2
Radix Paeoniae Alba (RPA)	MOL000358	beta-sitosterol	Apoptosis regulator BAX

Radix Paeoniae Alba (RPA)	MOL000358	beta-sitosterol	Caspase-9
Radix Paeoniae Alba (RPA)	MOL000358	beta-sitosterol	Transcription factor AP-1
Radix Paeoniae Alba (RPA)	MOL000358	beta-sitosterol	Caspase-3
Radix Paeoniae Alba (RPA)	MOL000358	beta-sitosterol	Caspase-8
Radix Paeoniae Alba (RPA)	MOL000358	beta-sitosterol	Protein kinase C alpha type
Radix Paeoniae Alba (RPA)	MOL000358	beta-sitosterol	Transforming growth factor beta-1
Radix Paeoniae Alba (RPA)	MOL000358	beta-sitosterol	Serum paraoxonase/arylesterase 1
Radix Paeoniae Alba (RPA)	MOL000358	beta-sitosterol	Microtubule-associated protein 2
Radix Paeoniae Alba (RPA)	MOL000359	sitosterol	Progesterone receptor
Radix Paeoniae Alba (RPA)	MOL000359	sitosterol	Nuclear receptor coactivator 2
Radix Paeoniae Alba (RPA)	MOL000359	sitosterol	Mineralocorticoid receptor
Radix Paeoniae Alba (RPA)	MOL000422	kaempferol	Nitric oxide synthase, inducible
Radix Paeoniae Alba (RPA)	MOL000422	kaempferol	Prostaglandin G/H synthase 1
Radix Paeoniae Alba (RPA)	MOL000422	kaempferol	Androgen receptor
Radix Paeoniae Alba (RPA)	MOL000422	kaempferol	Peroxisome proliferator activated receptor gamma
Radix Paeoniae Alba (RPA)	MOL000422	kaempferol	Prostaglandin G/H synthase 2
Radix Paeoniae Alba (RPA)	MOL000422	kaempferol	Heat shock protein HSP 90
Radix Paeoniae Alba (RPA)	MOL000422	kaempferol	Phosphatidylinositol-4,5-bisphosphate 3-kinase catalytic subunit, gamma
Radix Paeoniae Alba (RPA)	MOL000422	kaempferol	mRNA of PKA Catalytic Subunit C-
Radix Paeoniae Alba (RPA)	MOL000422	kaempferol	Nuclear receptor coactivator 2
Radix Paeoniae Alba (RPA)	MOL000422	kaempferol	Dipeptidyl peptidase IV
Radix Paeoniae Alba (RPA)	MOL000422	kaempferol	Trypsin-1
Radix Paeoniae Alba (RPA)	MOL000422	kaempferol	Progesterone receptor
Radix Paeoniae Alba (RPA)	MOL000422	kaempferol	Thrombin
Radix Paeoniae Alba (RPA)	MOL000422	kaempferol	Muscarinic acetylcholine receptor M1
Radix Paeoniae Alba (RPA)	MOL000422	kaempferol	Nitric-oxide synthase, endothelial
Radix Paeoniae Alba (RPA)	MOL000422	kaempferol	Gamma-aminobutyric-acid receptor alpha-2 subunit
Radix Paeoniae Alba (RPA)	MOL000422	kaempferol	Acetylcholinesterase
Radix Paeoniae Alba (RPA)	MOL000422	kaempferol	Sodium-dependent noradrenaline transporter
Radix Paeoniae Alba (RPA)	MOL000422	kaempferol	Muscarinic acetylcholine receptor M2
Radix Paeoniae Alba (RPA)	MOL000422	kaempferol	Alpha-1B adrenergic receptor
Radix Paeoniae Alba (RPA)	MOL000422	kaempferol	Gamma-aminobutyric acid receptor subunit alpha-1
Radix Paeoniae Alba (RPA)	MOL000422	kaempferol	DNA topoisomerase II
Radix Paeoniae Alba (RPA)	MOL000422	kaempferol	Coagulation factor VII
Radix Paeoniae Alba (RPA)	MOL000422	kaempferol	Calmodulin
Radix Paeoniae Alba (RPA)	MOL000422	kaempferol	Transcription factor p65
Radix Paeoniae Alba (RPA)	MOL000422	kaempferol	Inhibitor of nuclear factor kappa-B kinase subunit beta
Radix Paeoniae Alba (RPA)	MOL000422	kaempferol	RAC-alpha serine/threonine-protein kinase
Radix Paeoniae Alba (RPA)	MOL000422	kaempferol	Apoptosis regulator Bcl-2
Radix Paeoniae Alba (RPA)	MOL000422	kaempferol	Apoptosis regulator BAX
Radix Paeoniae Alba (RPA)	MOL000422	kaempferol	Tumor necrosis factor
Radix Paeoniae Alba (RPA)	MOL000422	kaempferol	Transcription factor AP-1
Radix Paeoniae Alba (RPA)	MOL000422	kaempferol	Activator of 90 kDa heat shock protein ATPase homolog 1
Radix Paeoniae Alba (RPA)	MOL000422	kaempferol	Caspase-3
Radix Paeoniae Alba (RPA)	MOL000422	kaempferol	Mitogen-activated protein kinase 8
Radix Paeoniae Alba (RPA)	MOL000422	kaempferol	Xanthine dehydrogenase/oxidase
Radix Paeoniae Alba (RPA)	MOL000422	kaempferol	Interstitial collagenase
Radix Paeoniae Alba (RPA)	MOL000422	kaempferol	Signal transducer and activator of transcription 1-alpha/beta
Radix Paeoniae Alba (RPA)	MOL000422	kaempferol	Cell division control protein 2 homolog
Radix Paeoniae Alba (RPA)	MOL000422	kaempferol	Peroxisome proliferator-activated receptor gamma
Radix Paeoniae Alba (RPA)	MOL000422	kaempferol	Heme oxygenase 1
Radix Paeoniae Alba (RPA)	MOL000422	kaempferol	Cytochrome P450 3A4
Radix Paeoniae Alba (RPA)	MOL000422	kaempferol	Cytochrome P450 1A2

Radix Paeoniae Alba (RPA)	MOL000422	kaempferol	Cytochrome P450 1A1
Radix Paeoniae Alba (RPA)	MOL000422	kaempferol	Intercellular adhesion molecule 1
Radix Paeoniae Alba (RPA)	MOL000422	kaempferol	E-selectin
Radix Paeoniae Alba (RPA)	MOL000422	kaempferol	Vascular cell adhesion protein 1
Radix Paeoniae Alba (RPA)	MOL000422	kaempferol	Nuclear receptor subfamily 1 group I member 2
Radix Paeoniae Alba (RPA)	MOL000422	kaempferol	Cytochrome P450 1B1
Radix Paeoniae Alba (RPA)	MOL000422	kaempferol	Arachidonate 5-lipoxygenase
Radix Paeoniae Alba (RPA)	MOL000422	kaempferol	Hyaluronan synthase 2
Radix Paeoniae Alba (RPA)	MOL000422	kaempferol	Glutathione S-transferase P
Radix Paeoniae Alba (RPA)	MOL000422	kaempferol	Aryl hydrocarbon receptor
Radix Paeoniae Alba (RPA)	MOL000422	kaempferol	26S proteasome non-ATPase regulatory subunit 3
Radix Paeoniae Alba (RPA)	MOL000422	kaempferol	Solute carrier family 2, facilitated glucose transporter member 4
Radix Paeoniae Alba (RPA)	MOL000422	kaempferol	Nuclear receptor subfamily 1 group I member 3
Radix Paeoniae Alba (RPA)	MOL000422	kaempferol	Insulin receptor
Radix Paeoniae Alba (RPA)	MOL000422	kaempferol	Type I iodothyronine deiodinase
Radix Paeoniae Alba (RPA)	MOL000422	kaempferol	Serine/threonine-protein phosphatase 2B catalytic subunit alpha isoform
Radix Paeoniae Alba (RPA)	MOL000422	kaempferol	Peroxidase C1A
Radix Paeoniae Alba (RPA)	MOL000422	kaempferol	Glutathione S-transferase Mu 1
Radix Paeoniae Alba (RPA)	MOL000422	kaempferol	Glutathione S-transferase Mu 2
Radix Paeoniae Alba (RPA)	MOL000422	kaempferol	Aldo-keto reductase family 1 member
Radix Paeoniae Alba (RPA)	MOL000422	kaempferol	Antileukoproteinase
Radix Paeoniae Alba (RPA)	MOL000492	(+)-catechin	Prostaglandin G/H synthase 1
Radix Paeoniae Alba (RPA)	MOL000492	(+)-catechin	Estrogen receptor
Radix Paeoniae Alba (RPA)	MOL000492	(+)-catechin	Prostaglandin G/H synthase 2
Radix Paeoniae Alba (RPA)	MOL000492	(+)-catechin	Heat shock protein HSP 90
Radix Paeoniae Alba (RPA)	MOL000492	(+)-catechin	Beta-lactamase
Radix Paeoniae Alba (RPA)	MOL000492	(+)-catechin	mRNA of PKA Catalytic Subunit C-
Radix Paeoniae Alba (RPA)	MOL000492	(+)-catechin	Nuclear receptor coactivator 2
Radix Paeoniae Alba (RPA)	MOL000492	(+)-catechin	Calmodulin
Radix Paeoniae Alba (RPA)	MOL000492	(+)-catechin	Retinoic acid receptor RXR-alpha
Radix Paeoniae Alba (RPA)	MOL000492	(+)-catechin	Catalase
Radix Paeoniae Alba (RPA)	MOL000492	(+)-catechin	Hyaluronan synthase 2
Rhizoma Atractylodis Macrocephalae (RAM)	MOL000022	14-acetyl-12-senecioyl-2E,8Z,10E-atractylentriol	Prostaglandin G/H synthase 2
Rhizoma Atractylodis Macrocephalae (RAM)	MOL000033	(3S,8S,9S,10R,13R,14S,17R)-10,13-dimethyl-17-[(2R,5S)-5-propan-2-yloctan-2-yl]-2,3,4,7,8,9,11,12,14,15,16,17-dodecahydro-1H-cyclopenta[a]phenanthren-3-ol	Progesterone receptor
Rhizoma Atractylodis Macrocephalae (RAM)	MOL000049	3β-acetoxyatractylone	Muscarinic acetylcholine receptor M3
Rhizoma Atractylodis Macrocephalae (RAM)	MOL000049	3β-acetoxyatractylone	Thrombin
Rhizoma Atractylodis Macrocephalae (RAM)	MOL000049	3β-acetoxyatractylone	Muscarinic acetylcholine receptor M1
Rhizoma Atractylodis Macrocephalae (RAM)	MOL000049	3β-acetoxyatractylone	Androgen receptor
Rhizoma Atractylodis Macrocephalae (RAM)	MOL000049	3β-acetoxyatractylone	Sodium channel protein type 5 subunit alpha
Rhizoma Atractylodis Macrocephalae (RAM)	MOL000049	3β-acetoxyatractylone	Prostaglandin G/H synthase 2
Rhizoma Atractylodis Macrocephalae (RAM)	MOL000049	3β-acetoxyatractylone	Nitric-oxide synthase, endothelial
Rhizoma Atractylodis Macrocephalae (RAM)	MOL000049	3β-acetoxyatractylone	Retinoic acid receptor RXR-alpha
Rhizoma Atractylodis Macrocephalae (RAM)	MOL000049	3β-acetoxyatractylone	Acetylcholinesterase

Rhizoma Atractylodis Macrocephalae (RAM)	MOL000049	3β-acetoxyatractylone	Alpha-1A adrenergic receptor
Rhizoma Atractylodis Macrocephalae (RAM)	MOL000049	3β-acetoxyatractylone	Muscarinic acetylcholine receptor M2
Rhizoma Atractylodis Macrocephalae (RAM)	MOL000049	3β-acetoxyatractylone	Beta-2 adrenergic receptor
Rhizoma Atractylodis Macrocephalae (RAM)	MOL000049	3β-acetoxyatractylone	Mu-type opioid receptor
Rhizoma Atractylodis Macrocephalae (RAM)	MOL000049	3β-acetoxyatractylone	Gamma-aminobutyric acid receptor subunit alpha-1
Rhizoma Atractylodis Macrocephalae (RAM)	MOL000049	3β-acetoxyatractylone	Dipeptidyl peptidase IV
Rhizoma Atractylodis Macrocephalae (RAM)	MOL000049	3β-acetoxyatractylone	Neuronal acetylcholine receptor protein, alpha-7 chain
Rhizoma Atractylodis Macrocephalae (RAM)	MOL000072	8β-ethoxy atractylenolide Ⅲ	Prostaglandin G/H synthase 2
Rhizoma Atractylodis Macrocephalae (RAM)	MOL000072	8β-ethoxy atractylenolide Ⅲ	Gamma-aminobutyric acid receptor subunit alpha-1
Rhizoma Atractylodis Macrocephalae (RAM)	MOL000072	8β-ethoxy atractylenolide Ⅲ	Neuronal acetylcholine receptor protein, alpha-7 chain
Rhizoma Atractylodis Macrocephalae (RAM)	MOL000072	8β-ethoxy atractylenolide Ⅲ	Nuclear receptor coactivator 2
Rhizoma Atractylodis Macrocephalae (RAM)	MOL000072	8β-ethoxy atractylenolide Ⅲ	Nuclear receptor coactivator 1
Radix Angelicae Sinensis (RAS)	MOL000358	beta-sitosterol	Progesterone receptor
Radix Angelicae Sinensis (RAS)	MOL000358	beta-sitosterol	Nuclear receptor coactivator 2
Radix Angelicae Sinensis (RAS)	MOL000358	beta-sitosterol	Prostaglandin G/H synthase 1
Radix Angelicae Sinensis (RAS)	MOL000358	beta-sitosterol	Prostaglandin G/H synthase 2
Radix Angelicae Sinensis (RAS)	MOL000358	beta-sitosterol	Heat shock protein HSP 90
Radix Angelicae Sinensis (RAS)	MOL000358	beta-sitosterol	Phosphatidylinositol-4,5-bisphosphate 3-kinase catalytic subunit, gamma
Radix Angelicae Sinensis (RAS)	MOL000358	beta-sitosterol	Potassium voltage-gated channel subfamily H member 2
Radix Angelicae Sinensis (RAS)	MOL000358	beta-sitosterol	mRNA of PKA Catalytic Subunit C-alpha
Radix Angelicae Sinensis (RAS)	MOL000358	beta-sitosterol	Dopamine D1 receptor
Radix Angelicae Sinensis (RAS)	MOL000358	beta-sitosterol	Muscarinic acetylcholine receptor M3
Radix Angelicae Sinensis (RAS)	MOL000358	beta-sitosterol	Muscarinic acetylcholine receptor M1
Radix Angelicae Sinensis (RAS)	MOL000358	beta-sitosterol	Sodium channel protein type 5 subunit alpha
Radix Angelicae Sinensis (RAS)	MOL000358	beta-sitosterol	Gamma-aminobutyric-acid receptor alpha-2 subunit
Radix Angelicae Sinensis (RAS)	MOL000358	beta-sitosterol	Muscarinic acetylcholine receptor M4
Radix Angelicae Sinensis (RAS)	MOL000358	beta-sitosterol	CGMP-inhibited 3',5'-cyclic phosphodiesterase A
Radix Angelicae Sinensis (RAS)	MOL000358	beta-sitosterol	5-hydroxytryptamine 2A receptor
Radix Angelicae Sinensis (RAS)	MOL000358	beta-sitosterol	Gamma-aminobutyric-acid receptor alpha-5 subunit
Radix Angelicae Sinensis (RAS)	MOL000358	beta-sitosterol	Alpha-1A adrenergic receptor
Radix Angelicae Sinensis (RAS)	MOL000358	beta-sitosterol	Gamma-aminobutyric-acid receptor alpha-3 subunit

Radix Angelicae Sinensis (RAS)	MOL000358	beta-sitosterol	Muscarinic acetylcholine receptor M2
Radix Angelicae Sinensis (RAS)	MOL000358	beta-sitosterol	Alpha-1B adrenergic receptor
Radix Angelicae Sinensis (RAS)	MOL000358	beta-sitosterol	Beta-2 adrenergic receptor
Radix Angelicae Sinensis (RAS)	MOL000358	beta-sitosterol	Neuronal acetylcholine receptor subunit alpha-2
Radix Angelicae Sinensis (RAS)	MOL000358	beta-sitosterol	Sodium-dependent serotonin transporter
Radix Angelicae Sinensis (RAS)	MOL000358	beta-sitosterol	Mu-type opioid receptor
Radix Angelicae Sinensis (RAS)	MOL000358	beta-sitosterol	Gamma-aminobutyric acid receptor subunit alpha-1
Radix Angelicae Sinensis (RAS)	MOL000358	beta-sitosterol	Neuronal acetylcholine receptor protein, alpha-7 chain
Radix Angelicae Sinensis (RAS)	MOL000358	beta-sitosterol	Cytochrome P450-cam
Radix Angelicae Sinensis (RAS)	MOL000358	beta-sitosterol	Apoptosis regulator Bcl-2
Radix Angelicae Sinensis (RAS)	MOL000358	beta-sitosterol	Apoptosis regulator BAX
Radix Angelicae Sinensis (RAS)	MOL000358	beta-sitosterol	Caspase-9
Radix Angelicae Sinensis (RAS)	MOL000358	beta-sitosterol	Transcription factor AP-1
Radix Angelicae Sinensis (RAS)	MOL000358	beta-sitosterol	Caspase-3
Radix Angelicae Sinensis (RAS)	MOL000358	beta-sitosterol	Caspase-8
Radix Angelicae Sinensis (RAS)	MOL000358	beta-sitosterol	Protein kinase C alpha type
Radix Angelicae Sinensis (RAS)	MOL000358	beta-sitosterol	Transforming growth factor beta-1
Radix Angelicae Sinensis (RAS)	MOL000358	beta-sitosterol	Serum paraoxonase/arylesterase 1
Radix Angelicae Sinensis (RAS)	MOL000358	beta-sitosterol	Microtubule-associated protein 2
Radix Angelicae Sinensis (RAS)	MOL000449	Stigmasterol	Progesterone receptor
Radix Angelicae Sinensis (RAS)	MOL000449	Stigmasterol	Mineralocorticoid receptor
Radix Angelicae Sinensis (RAS)	MOL000449	Stigmasterol	Nuclear receptor coactivator 2
Radix Angelicae Sinensis (RAS)	MOL000449	Stigmasterol	Alcohol dehydrogenase 1C
Radix Angelicae Sinensis (RAS)	MOL000449	Stigmasterol	Ig gamma-1 chain C region
Radix Angelicae Sinensis (RAS)	MOL000449	Stigmasterol	Retinoic acid receptor RXR-alpha
Radix Angelicae Sinensis (RAS)	MOL000449	Stigmasterol	Nuclear receptor coactivator 1
Radix Angelicae Sinensis (RAS)	MOL000449	Stigmasterol	Prostaglandin G/H synthase 1
Radix Angelicae Sinensis (RAS)	MOL000449	Stigmasterol	Prostaglandin G/H synthase 2
Radix Angelicae Sinensis (RAS)	MOL000449	Stigmasterol	Alpha-2A adrenergic receptor
Radix Angelicae Sinensis (RAS)	MOL000449	Stigmasterol	Sodium-dependent noradrenaline transporter
Radix Angelicae Sinensis (RAS)	MOL000449	Stigmasterol	Sodium-dependent dopamine transporter

Radix Angelicae Sinensis (RAS)	MOL000449	Stigmasterol	Beta-2 adrenergic receptor
Radix Angelicae Sinensis (RAS)	MOL000449	Stigmasterol	Aldose reductase
Radix Angelicae Sinensis (RAS)	MOL000449	Stigmasterol	Urokinase-type plasminogen activator
Radix Angelicae Sinensis (RAS)	MOL000449	Stigmasterol	Leukotriene A-4 hydrolase
Radix Angelicae Sinensis (RAS)	MOL000449	Stigmasterol	Amine oxidase [flavin-containing] B
Radix Angelicae Sinensis (RAS)	MOL000449	Stigmasterol	Amine oxidase [flavin-containing] A
Radix Angelicae Sinensis (RAS)	MOL000449	Stigmasterol	mRNA of PKA Catalytic Subunit C-alpha
Radix Angelicae Sinensis (RAS)	MOL000449	Stigmasterol	Chymotrypsinogen B
Radix Angelicae Sinensis (RAS)	MOL000449	Stigmasterol	Muscarinic acetylcholine receptor M3
Radix Angelicae Sinensis (RAS)	MOL000449	Stigmasterol	Muscarinic acetylcholine receptor M1
Radix Angelicae Sinensis (RAS)	MOL000449	Stigmasterol	Beta-1 adrenergic receptor
Radix Angelicae Sinensis (RAS)	MOL000449	Stigmasterol	Sodium channel protein type 5 subunit alpha
Radix Angelicae Sinensis (RAS)	MOL000449	Stigmasterol	5-hydroxytryptamine 2A receptor
Radix Angelicae Sinensis (RAS)	MOL000449	Stigmasterol	Alpha-1A adrenergic receptor
Radix Angelicae Sinensis (RAS)	MOL000449	Stigmasterol	Gamma-aminobutyric-acid receptor alpha-3 subunit
Radix Angelicae Sinensis (RAS)	MOL000449	Stigmasterol	Muscarinic acetylcholine receptor M2
Radix Angelicae Sinensis (RAS)	MOL000449	Stigmasterol	Alpha-1B adrenergic receptor
Radix Angelicae Sinensis (RAS)	MOL000449	Stigmasterol	Gamma-aminobutyric acid receptor subunit alpha-1
Radix Angelicae Sinensis (RAS)	MOL000449	Stigmasterol	Neuronal acetylcholine receptor protein, alpha-7 chain
Radix Codonopsis (RC)	MOL001006	poriferasta-7,22E-dien-3beta-ol	Progesterone receptor
Radix Codonopsis (RC)	MOL001006	poriferasta-7,22E-dien-3beta-ol	Nuclear receptor coactivator 2
Radix Codonopsis (RC)	MOL001006	poriferasta-7,22E-dien-3beta-ol	Mineralocorticoid receptor
Radix Codonopsis (RC)	MOL002140	Perlolyrine	Thrombin
Radix Codonopsis (RC)	MOL002140	Perlolyrine	Prostaglandin G/H synthase 2
Radix Codonopsis (RC)	MOL002140	Perlolyrine	Retinoic acid receptor RXR-alpha
Radix Codonopsis (RC)	MOL002140	Perlolyrine	mRNA of PKA Catalytic Subunit C-
Radix Codonopsis (RC)	MOL002879	Diop	Sodium channel protein type 5 subunit alpha
Radix Codonopsis (RC)	MOL002879	Diop	Beta-2 adrenergic receptor
Radix Codonopsis (RC)	MOL002879	Diop	Muscarinic acetylcholine receptor M3
Radix Codonopsis (RC)	MOL003036	ZINC03978781	Progesterone receptor
Radix Codonopsis (RC)	MOL003036	ZINC03978781	Nuclear receptor coactivator 2
Radix Codonopsis (RC)	MOL003036	ZINC03978781	Mineralocorticoid receptor
Radix Codonopsis (RC)	MOL000449	Stigmasterol	Progesterone receptor
Radix Codonopsis (RC)	MOL000449	Stigmasterol	Mineralocorticoid receptor
Radix Codonopsis (RC)	MOL000449	Stigmasterol	Nuclear receptor coactivator 2
Radix Codonopsis (RC)	MOL000449	Stigmasterol	Alcohol dehydrogenase 1C
Radix Codonopsis (RC)	MOL000449	Stigmasterol	Ig gamma-1 chain C region
Radix Codonopsis (RC)	MOL000449	Stigmasterol	Retinoic acid receptor RXR-alpha
Radix Codonopsis (RC)	MOL000449	Stigmasterol	Nuclear receptor coactivator 1
Radix Codonopsis (RC)	MOL000449	Stigmasterol	Prostaglandin G/H synthase 1
Radix Codonopsis (RC)	MOL000449	Stigmasterol	Prostaglandin G/H synthase 2
Radix Codonopsis (RC)	MOL000449	Stigmasterol	Alpha-2A adrenergic receptor

Radix Codonopsis (RC)	MOL000449	Stigmasterol	Sodium-dependent noradrenaline transporter
Radix Codonopsis (RC)	MOL000449	Stigmasterol	Sodium-dependent dopamine
Radix Codonopsis (RC)	MOL000449	Stigmasterol	Beta-2 adrenergic receptor
Radix Codonopsis (RC)	MOL000449	Stigmasterol	Aldose reductase
Radix Codonopsis (RC)	MOL000449	Stigmasterol	Urokinase-type plasminogen activator
Radix Codonopsis (RC)	MOL000449	Stigmasterol	Leukotriene A-4 hydrolase
Radix Codonopsis (RC)	MOL000449	Stigmasterol	Amine oxidase [flavin-containing] B
Radix Codonopsis (RC)	MOL000449	Stigmasterol	Amine oxidase [flavin-containing] A
Radix Codonopsis (RC)	MOL000449	Stigmasterol	mRNA of PKA Catalytic Subunit C-
Radix Codonopsis (RC)	MOL000449	Stigmasterol	Chymotrypsinogen B
Radix Codonopsis (RC)	MOL000449	Stigmasterol	Muscarinic acetylcholine receptor M3
Radix Codonopsis (RC)	MOL000449	Stigmasterol	Muscarinic acetylcholine receptor M1
Radix Codonopsis (RC)	MOL000449	Stigmasterol	Beta-1 adrenergic receptor
Radix Codonopsis (RC)	MOL000449	Stigmasterol	Sodium channel protein type 5 subunit alpha
Radix Codonopsis (RC)	MOL000449	Stigmasterol	5-hydroxytryptamine 2A receptor
Radix Codonopsis (RC)	MOL000449	Stigmasterol	Alpha-1A adrenergic receptor
Radix Codonopsis (RC)	MOL000449	Stigmasterol	Gamma-aminobutyric-acid receptor alpha-3 subunit
Radix Codonopsis (RC)	MOL000449	Stigmasterol	Muscarinic acetylcholine receptor M2
Radix Codonopsis (RC)	MOL000449	Stigmasterol	Alpha-1B adrenergic receptor
Radix Codonopsis (RC)	MOL000449	Stigmasterol	Gamma-aminobutyric acid receptor subunit alpha-1
Radix Codonopsis (RC)	MOL000449	Stigmasterol	Neuronal acetylcholine receptor protein, alpha-7 chain
Radix Codonopsis (RC)	MOL003896	7-Methoxy-2-methyl	Nitric oxide synthase, inducible
Radix Codonopsis (RC)	MOL003896	7-Methoxy-2-methyl	Prostaglandin G/H synthase 1
Radix Codonopsis (RC)	MOL003896	7-Methoxy-2-methyl	Dopamine D1 receptor
Radix Codonopsis (RC)	MOL003896	7-Methoxy-2-methyl	Muscarinic acetylcholine receptor M3
Radix Codonopsis (RC)	MOL003896	7-Methoxy-2-methyl	Thrombin
Radix Codonopsis (RC)	MOL003896	7-Methoxy-2-methyl	Muscarinic acetylcholine receptor M1
Radix Codonopsis (RC)	MOL003896	7-Methoxy-2-methyl	Estrogen receptor
Radix Codonopsis (RC)	MOL003896	7-Methoxy-2-methyl	Androgen receptor
Radix Codonopsis (RC)	MOL003896	7-Methoxy-2-methyl	Beta-1 adrenergic receptor
Radix Codonopsis (RC)	MOL003896	7-Methoxy-2-methyl isoflavone	Sodium channel protein type 5 subunit alpha
Radix Codonopsis (RC)	MOL003896	7-Methoxy-2-methyl isoflavone	Peroxisome proliferator activated receptor gamma
Radix Codonopsis (RC)	MOL003896	7-Methoxy-2-methyl	Prostaglandin G/H synthase 2
Radix Codonopsis (RC)	MOL003896	7-Methoxy-2-methyl	Retinoic acid receptor RXR-alpha
Radix Codonopsis (RC)	MOL003896	7-Methoxy-2-methyl	Acetylcholinesterase
Radix Codonopsis (RC)	MOL003896	7-Methoxy-2-methyl isoflavone	CGMP-inhibited 3',5'-cyclic phosphodiesterase A
Radix Codonopsis (RC)	MOL003896	7-Methoxy-2-methyl	Alpha-1B adrenergic receptor
Radix Codonopsis (RC)	MOL003896	7-Methoxy-2-methyl	Sodium-dependent dopamine
Radix Codonopsis (RC)	MOL003896	7-Methoxy-2-methyl	Beta-2 adrenergic receptor
Radix Codonopsis (RC)	MOL003896	7-Methoxy-2-methyl	Alpha-1D adrenergic receptor
Radix Codonopsis (RC)	MOL003896	7-Methoxy-2-methyl	Sodium-dependent serotonin transporter
Radix Codonopsis (RC)	MOL003896	7-Methoxy-2-methyl	Estrogen receptor beta
Radix Codonopsis (RC)	MOL003896	7-Methoxy-2-methyl isoflavone	Gamma-aminobutyric acid receptor subunit alpha-1
Radix Codonopsis (RC)	MOL003896	7-Methoxy-2-methyl	Dipeptidyl peptidase IV
Radix Codonopsis (RC)	MOL003896	7-Methoxy-2-methyl	Mitogen-activated protein kinase 14
Radix Codonopsis (RC)	MOL003896	7-Methoxy-2-methyl	Glycogen synthase kinase-3 beta
Radix Codonopsis (RC)	MOL003896	7-Methoxy-2-methyl	Heat shock protein HSP 90
Radix Codonopsis (RC)	MOL003896	7-Methoxy-2-methyl	Cell division protein kinase 2
Radix Codonopsis (RC)	MOL003896	7-Methoxy-2-methyl	Leukotriene A-4 hydrolase
Radix Codonopsis (RC)	MOL003896	7-Methoxy-2-methyl	Amine oxidase [flavin-containing] B
Radix Codonopsis (RC)	MOL003896	7-Methoxy-2-methyl isoflavone	Neuronal acetylcholine receptor protein, alpha-7 chain
Radix Codonopsis (RC)	MOL003896	7-Methoxy-2-methyl	Serine/threonine-protein kinase Chk1
Radix Codonopsis (RC)	MOL003896	7-Methoxy-2-methyl	mRNA of PKA Catalytic Subunit C-

Radix Codonopsis (RC)	MOL003896	7-Methoxy-2-methyl	Ig gamma-1 chain C region
Radix Codonopsis (RC)	MOL003896	7-Methoxy-2-methyl	Trypsin-1
Radix Codonopsis (RC)	MOL003896	7-Methoxy-2-methyl isoflavone	Proto-oncogene serine/threonine-protein kinase Pim-1
Radix Codonopsis (RC)	MOL003896	7-Methoxy-2-methyl	Cyclin-A2
Radix Codonopsis (RC)	MOL003896	7-Methoxy-2-methyl	Nuclear receptor coactivator 1
Radix Codonopsis (RC)	MOL003896	7-Methoxy-2-methyl isoflavone	cAMP-dependent protein kinase inhibitor alpha
Radix Codonopsis (RC)	MOL003896	7-Methoxy-2-methyl	Calmodulin
Radix Codonopsis (RC)	MOL003896	7-Methoxy-2-methyl	Muscarinic acetylcholine receptor M5
Radix Codonopsis (RC)	MOL003896	7-Methoxy-2-methyl	Nitric-oxide synthase, endothelial
Radix Codonopsis (RC)	MOL003896	7-Methoxy-2-methyl	Mu-type opioid receptor
Radix Codonopsis (RC)	MOL003896	7-Methoxy-2-methyl	Nuclear receptor coactivator 2
Radix Codonopsis (RC)	MOL004355	Spinasterol	Progesterone receptor
Radix Codonopsis (RC)	MOL004355	Spinasterol	Mineralocorticoid receptor
Radix Codonopsis (RC)	MOL004355	Spinasterol	Nuclear receptor coactivator 2
Radix Codonopsis (RC)	MOL005321	Frutinone A	Prostaglandin G/H synthase 1
Radix Codonopsis (RC)	MOL005321	Frutinone A	Thrombin
Radix Codonopsis (RC)	MOL005321	Frutinone A	Androgen receptor
Radix Codonopsis (RC)	MOL005321	Frutinone A	Sodium channel protein type 5 subunit alpha
Radix Codonopsis (RC)	MOL005321	Frutinone A	Peroxisome proliferator activated receptor gamma
Radix Codonopsis (RC)	MOL005321	Frutinone A	Prostaglandin G/H synthase 2
Radix Codonopsis (RC)	MOL005321	Frutinone A	Retinoic acid receptor RXR-alpha
Radix Codonopsis (RC)	MOL005321	Frutinone A	CGMP-inhibited 3',5'-cyclic phosphodiesterase A
Radix Codonopsis (RC)	MOL005321	Frutinone A	Beta-2 adrenergic receptor
Radix Codonopsis (RC)	MOL005321	Frutinone A	Gamma-aminobutyric acid receptor subunit alpha-1
Radix Codonopsis (RC)	MOL005321	Frutinone A	Dipeptidyl peptidase IV
Radix Codonopsis (RC)	MOL005321	Frutinone A	Heat shock protein HSP 90
Radix Codonopsis (RC)	MOL005321	Frutinone A	Phosphatidylinositol-4,5-bisphosphate 3-kinase catalytic subunit, gamma
Radix Codonopsis (RC)	MOL005321	Frutinone A	Neuronal acetylcholine receptor protein, alpha-7 chain
Radix Codonopsis (RC)	MOL005321	Frutinone A	mRNA of PKA Catalytic Subunit C-
Radix Codonopsis (RC)	MOL005321	Frutinone A	Acetylcholinesterase
Radix Codonopsis (RC)	MOL000006	luteolin	Prostaglandin G/H synthase 1
Radix Codonopsis (RC)	MOL000006	luteolin	Androgen receptor
Radix Codonopsis (RC)	MOL000006	luteolin	Prostaglandin G/H synthase 2
Radix Codonopsis (RC)	MOL000006	luteolin	Heat shock protein HSP 90
Radix Codonopsis (RC)	MOL000006	luteolin	Trypsin-1
Radix Codonopsis (RC)	MOL000006	luteolin	Nuclear receptor coactivator 2
Radix Codonopsis (RC)	MOL000006	luteolin	mRNA of PKA Catalytic Subunit C-
Radix Codonopsis (RC)	MOL000006	luteolin	Dipeptidyl peptidase IV
Radix Codonopsis (RC)	MOL000006	luteolin	Phosphatidylinositol-4,5-bisphosphate 3-kinase catalytic subunit, gamma
Radix Codonopsis (RC)	MOL000006	luteolin	Transcription factor p65
Radix Codonopsis (RC)	MOL000006	luteolin	Epidermal growth factor receptor
Radix Codonopsis (RC)	MOL000006	luteolin	RAC-alpha serine/threonine-protein kinase
Radix Codonopsis (RC)	MOL000006	luteolin	Vascular endothelial growth factor A
Radix Codonopsis (RC)	MOL000006	luteolin	G1/S-specific cyclin-D1
Radix Codonopsis (RC)	MOL000006	luteolin	Bcl-2-like protein 1
Radix Codonopsis (RC)	MOL000006	luteolin	Cyclin-dependent kinase inhibitor 1
Radix Codonopsis (RC)	MOL000006	luteolin	Caspase-9
Radix Codonopsis (RC)	MOL000006	luteolin	72 kDa type IV collagenase
Radix Codonopsis (RC)	MOL000006	luteolin	Matrix metalloproteinase-9
Radix Codonopsis (RC)	MOL000006	luteolin	Mitogen-activated protein kinase 1
Radix Codonopsis (RC)	MOL000006	luteolin	Interleukin-10
Radix Codonopsis (RC)	MOL000006	luteolin	Retinoblastoma-associated protein
Radix Codonopsis (RC)	MOL000006	luteolin	Cell division protein kinase 4

Radix Codonopsis (RC)	MOL000006	luteolin	Tumor necrosis factor
Radix Codonopsis (RC)	MOL000006	luteolin	Transcription factor AP-1
Radix Codonopsis (RC)	MOL000006	luteolin	Interleukin-6
Radix Codonopsis (RC)	MOL000006	luteolin	Caspase-3
Radix Codonopsis (RC)	MOL000006	luteolin	Cellular tumor antigen p53
Radix Codonopsis (RC)	MOL000006	luteolin	NF-kappa-B inhibitor alpha
Radix Codonopsis (RC)	MOL000006	luteolin	Xanthine dehydrogenase/oxidase
Radix Codonopsis (RC)	MOL000006	luteolin	DNA topoisomerase 1
Radix Codonopsis (RC)	MOL000006	luteolin	E3 ubiquitin-protein ligase Mdm2
Radix Codonopsis (RC)	MOL000006	luteolin	Amyloid beta A4 protein
Radix Codonopsis (RC)	MOL000006	luteolin	Interstitial collagenase
Radix Codonopsis (RC)	MOL000006	luteolin	Proliferating cell nuclear antigen
Radix Codonopsis (RC)	MOL000006	luteolin	Receptor tyrosine-protein kinase erbB-2
Radix Codonopsis (RC)	MOL000006	luteolin	Peroxisome proliferator-activated receptor gamma
Radix Codonopsis (RC)	MOL000006	luteolin	Heme oxygenase 1
Radix Codonopsis (RC)	MOL000006	luteolin	Caspase-7
Radix Codonopsis (RC)	MOL000006	luteolin	Intercellular adhesion molecule 1
Radix Codonopsis (RC)	MOL000006	luteolin	Induced myeloid leukemia cell differentiation protein Mcl-1
Radix Codonopsis (RC)	MOL000006	luteolin	Baculoviral IAP repeat-containing protein 5
Radix Codonopsis (RC)	MOL000006	luteolin	Interleukin-2
Radix Codonopsis (RC)	MOL000006	luteolin	G2/mitotic-specific cyclin-B1
Radix Codonopsis (RC)	MOL000006	luteolin	Tyrosinase
Radix Codonopsis (RC)	MOL000006	luteolin	Interferon gamma
Radix Codonopsis (RC)	MOL000006	luteolin	Interleukin-4
Radix Codonopsis (RC)	MOL000006	luteolin	DNA topoisomerase 2-alpha
Radix Codonopsis (RC)	MOL000006	luteolin	Glutathione S-transferase P
Radix Codonopsis (RC)	MOL000006	luteolin	Baculoviral IAP repeat-containing protein 4
Radix Codonopsis (RC)	MOL000006	luteolin	Solute carrier family 2, facilitated glucose transporter member 4
Radix Codonopsis (RC)	MOL000006	luteolin	Insulin receptor
Radix Codonopsis (RC)	MOL000006	luteolin	CD40 ligand
Radix Codonopsis (RC)	MOL000006	luteolin	Prostaglandin E synthase
Radix Codonopsis (RC)	MOL000006	luteolin	Kinetochore protein Nuf2
Radix Codonopsis (RC)	MOL000006	luteolin	Adenylate cyclase type 2
Radix Codonopsis (RC)	MOL000006	luteolin	Hepatocyte growth factor receptor
Radix Codonopsis (RC)	MOL006774	stigmast-7-enol	Progesterone receptor
Radix Codonopsis (RC)	MOL006774	stigmast-7-enol	Nuclear receptor coactivator 2
Radix Codonopsis (RC)	MOL007059	3-beta-Hydroxymethyllenetanshiquino	Dopamine D1 receptor
Radix Codonopsis (RC)	MOL007059	3-beta-Hydroxymethyllenetanshiquino	Thrombin
Radix Codonopsis (RC)	MOL007059	3-beta-Hydroxymethyllenetanshiquino	Muscarinic acetylcholine receptor M1
Radix Codonopsis (RC)	MOL007059	3-beta-Hydroxymethyllenetanshiquino	Prostaglandin G/H synthase 2
Radix Codonopsis (RC)	MOL007059	3-beta-Hydroxymethyllenetanshiquino	Carbonic anhydrase II
Radix Codonopsis (RC)	MOL007059	3-beta-Hydroxymethyllenetanshiquino	Retinoic acid receptor RXR-alpha
Radix Codonopsis (RC)	MOL007059	3-beta-Hydroxymethyllenetanshiquino	Delta-type opioid receptor
Radix Codonopsis (RC)	MOL007059	3-beta-Hydroxymethyllenetanshiquino	Acetylcholinesterase
Radix Codonopsis (RC)	MOL007059	3-beta-Hydroxymethyllenetanshiquino	Alpha-1A adrenergic receptor
Radix Codonopsis (RC)	MOL007059	3-beta-Hydroxymethyllenetanshiquino	Beta-2 adrenergic receptor
Radix Codonopsis (RC)	MOL007059	3-beta-Hydroxymethyllenetanshiquino	Mu-type opioid receptor

Radix Codonopsis (RC)	MOL007059	3-beta-Hydroxymethyllenetanshiquino	Dipeptidyl peptidase IV
Radix Codonopsis (RC)	MOL007059	3-beta-Hydroxymethyllenetanshiquino	Heat shock protein HSP 90
Radix Codonopsis (RC)	MOL007059	3-beta-Hydroxymethyllenetanshiquino	Neuronal acetylcholine receptor protein, alpha-7 chain
Radix Codonopsis (RC)	MOL007059	3-beta-Hydroxymethyllenetanshiquino	Ig gamma-1 chain C region
Radix Codonopsis (RC)	MOL007059	3-beta-Hydroxymethyllenetanshiquino	Trypsin-1
Radix Codonopsis (RC)	MOL007059	3-beta-Hydroxymethyllenetanshiquino	Nuclear receptor coactivator 1
Radix Codonopsis (RC)	MOL007514	methyl icosa-11,14-dienoate	Nuclear receptor coactivator 2
Radix Codonopsis (RC)	MOL008393	7-(beta-Xylosyl)cephalomannine_qt	Tubulin beta-1 chain
Radix Codonopsis (RC)	MOL008397	Daturilin	Glucocorticoid receptor
Radix Codonopsis (RC)	MOL008400	glycitein	Prostaglandin G/H synthase 1
Radix Codonopsis (RC)	MOL008400	glycitein	Estrogen receptor
Radix Codonopsis (RC)	MOL008400	glycitein	Androgen receptor
Radix Codonopsis (RC)	MOL008400	glycitein	Peroxisome proliferator activated receptor gamma
Radix Codonopsis (RC)	MOL008400	glycitein	Prostaglandin G/H synthase 2
Radix Codonopsis (RC)	MOL008400	glycitein	Retinoic acid receptor RXR-alpha
Radix Codonopsis (RC)	MOL008400	glycitein	CGMP-inhibited 3',5'-cyclic phosphodiesterase A
Radix Codonopsis (RC)	MOL008400	glycitein	Estrogen receptor beta
Radix Codonopsis (RC)	MOL008400	glycitein	Mitogen-activated protein kinase 14
Radix Codonopsis (RC)	MOL008400	glycitein	Glycogen synthase kinase-3 beta
Radix Codonopsis (RC)	MOL008400	glycitein	Heat shock protein HSP 90
Radix Codonopsis (RC)	MOL008400	glycitein	Cell division protein kinase 2
Radix Codonopsis (RC)	MOL008400	glycitein	Serine/threonine-protein kinase Chk1
Radix Codonopsis (RC)	MOL008400	glycitein	Trypsin-1
Radix Codonopsis (RC)	MOL008400	glycitein	Proto-oncogene serine/threonine-protein kinase Pim-1
Radix Codonopsis (RC)	MOL008400	glycitein	Cyclin-A2
Radix Codonopsis (RC)	MOL008400	glycitein	Calmodulin
Radix Codonopsis (RC)	MOL008400	glycitein	mRNA of PKA Catalytic Subunit C-
Radix Codonopsis (RC)	MOL008400	glycitein	Nuclear receptor coactivator 1
Radix Codonopsis (RC)	MOL008400	glycitein	Nitric oxide synthase, inducible
Radix Codonopsis (RC)	MOL008400	glycitein	Amyloid beta A4 protein
Radix Codonopsis (RC)	MOL008400	glycitein	Collagenase 3
Radix Codonopsis (RC)	MOL008400	glycitein	Neutrophil collagenase
Radix Codonopsis (RC)	MOL008407	(8S,9S,10R,13R,14S,17R)-17-[(E,2R,5S)-5-ethyl-6-methylhept-3-en-2-yl]-10,13-dimethyl-1,2,4,7,8,9,11,12,14,15,16,17-dodecahydrocyclopenta[a]phenanthren-3-one	Progesterone receptor
Radix Codonopsis (RC)	MOL008407	(8S,9S,10R,13R,14S,17R)-17-[(E,2R,5S)-5-ethyl-6-methylhept-3-en-2-yl]-10,13-dimethyl-1,2,4,7,8,9,11,12,14,15,16,17-dodecahydrocyclopenta[a]phenanthren-3-one	Mineralocorticoid receptor
Radix Codonopsis (RC)	MOL008411	11-Hydroxyrankinidine	Estrogen receptor
Radix Codonopsis (RC)	MOL008411	11-Hydroxyrankinidine	Sodium channel protein type 5 subunit alpha
Radix Codonopsis (RC)	MOL008411	11-Hydroxyrankinidine	Mu-type opioid receptor
Radix Codonopsis (RC)	MOL008411	11-Hydroxyrankinidine	Cell division protein kinase 2
Radix Glycyrrhizae (RG)	MOL001484	Inermine	Prostaglandin G/H synthase 1
Radix Glycyrrhizae (RG)	MOL001484	Inermine	Muscarinic acetylcholine receptor M3

Radix Glycyrrhizae (RG)	MOL001484	Inermine	Sodium channel protein type 5 subunit alpha
Radix Glycyrrhizae (RG)	MOL001484	Inermine	Prostaglandin G/H synthase 2
Radix Glycyrrhizae (RG)	MOL001484	Inermine	5-hydroxytryptamine receptor 3A
Radix Glycyrrhizae (RG)	MOL001484	Inermine	Retinoic acid receptor RXR-alpha
Radix Glycyrrhizae (RG)	MOL001484	Inermine	Alpha-1B adrenergic receptor
Radix Glycyrrhizae (RG)	MOL001484	Inermine	Alpha-1D adrenergic receptor
Radix Glycyrrhizae (RG)	MOL001484	Inermine	Phosphatidylinositol-4,5-bisphosphate 3-kinase catalytic subunit, gamma
Radix Glycyrrhizae (RG)	MOL001484	Inermine	mRNA of PKA Catalytic Subunit C-
Radix Glycyrrhizae (RG)	MOL001484	Inermine	Ig gamma-1 chain C region
Radix Glycyrrhizae (RG)	MOL001484	Inermine	Trypsin-1
Radix Glycyrrhizae (RG)	MOL001484	Inermine	Calmodulin
Radix Glycyrrhizae (RG)	MOL001484	Inermine	Muscarinic acetylcholine receptor M1
Radix Glycyrrhizae (RG)	MOL001484	Inermine	Beta-2 adrenergic receptor
Radix Glycyrrhizae (RG)	MOL001484	Inermine	Mu-type opioid receptor
Radix Glycyrrhizae (RG)	MOL001484	Inermine	Heat shock protein HSP 90
Radix Glycyrrhizae (RG)	MOL001792	DFV	Prostaglandin G/H synthase 1
Radix Glycyrrhizae (RG)	MOL001792	DFV	Estrogen receptor
Radix Glycyrrhizae (RG)	MOL001792	DFV	Prostaglandin G/H synthase 2
Radix Glycyrrhizae (RG)	MOL001792	DFV	Retinoic acid receptor RXR-alpha
Radix Glycyrrhizae (RG)	MOL001792	DFV	Beta-2 adrenergic receptor
Radix Glycyrrhizae (RG)	MOL001792	DFV	Heat shock protein HSP 90
Radix Glycyrrhizae (RG)	MOL001792	DFV	Phosphatidylinositol-4,5-bisphosphate 3-kinase catalytic subunit, gamma
Radix Glycyrrhizae (RG)	MOL001792	DFV	mRNA of PKA Catalytic Subunit C-
Radix Glycyrrhizae (RG)	MOL001792	DFV	Beta-lactamase
Radix Glycyrrhizae (RG)	MOL001792	DFV	Amine oxidase [flavin-containing] B
Radix Glycyrrhizae (RG)	MOL001792	DFV	Sodium-dependent serotonin transporter
Radix Glycyrrhizae (RG)	MOL001792	DFV	cAMP-dependent protein kinase inhibitor alpha
Radix Glycyrrhizae (RG)	MOL000211	Mairin	Progesterone receptor
Radix Glycyrrhizae (RG)	MOL002311	Glycyrol	Nitric oxide synthase, inducible
Radix Glycyrrhizae (RG)	MOL002311	Glycyrol	Estrogen receptor
Radix Glycyrrhizae (RG)	MOL002311	Glycyrol	Peroxisome proliferator activated receptor gamma
Radix Glycyrrhizae (RG)	MOL002311	Glycyrol	Prostaglandin G/H synthase 2
Radix Glycyrrhizae (RG)	MOL002311	Glycyrol	Vascular endothelial growth factor receptor 2
Radix Glycyrrhizae (RG)	MOL002311	Glycyrol	Mitogen-activated protein kinase 14
Radix Glycyrrhizae (RG)	MOL002311	Glycyrol	Glycogen synthase kinase-3 beta
Radix Glycyrrhizae (RG)	MOL002311	Glycyrol	Serine/threonine-protein kinase Chk1
Radix Glycyrrhizae (RG)	MOL002311	Glycyrol	Proto-oncogene serine/threonine-protein kinase Pim-1
Radix Glycyrrhizae (RG)	MOL002311	Glycyrol	Cyclin-A2
Radix Glycyrrhizae (RG)	MOL002311	Glycyrol	Thrombin
Radix Glycyrrhizae (RG)	MOL000239	Jaranol	Nitric oxide synthase, inducible
Radix Glycyrrhizae (RG)	MOL000239	Jaranol	Prostaglandin G/H synthase 1
Radix Glycyrrhizae (RG)	MOL000239	Jaranol	Androgen receptor
Radix Glycyrrhizae (RG)	MOL000239	Jaranol	Sodium channel protein type 5 subunit alpha
Radix Glycyrrhizae (RG)	MOL000239	Jaranol	Prostaglandin G/H synthase 2
Radix Glycyrrhizae (RG)	MOL000239	Jaranol	Estrogen receptor beta
Radix Glycyrrhizae (RG)	MOL000239	Jaranol	Dipeptidyl peptidase IV
Radix Glycyrrhizae (RG)	MOL000239	Jaranol	Heat shock protein HSP 90
Radix Glycyrrhizae (RG)	MOL000239	Jaranol	Cell division protein kinase 2
Radix Glycyrrhizae (RG)	MOL000239	Jaranol	Serine/threonine-protein kinase Chk1
Radix Glycyrrhizae (RG)	MOL000239	Jaranol	Trypsin-1
Radix Glycyrrhizae (RG)	MOL000239	Jaranol	Nuclear receptor coactivator 2
Radix Glycyrrhizae (RG)	MOL000239	Jaranol	Calmodulin
Radix Glycyrrhizae (RG)	MOL002565	Medicarpin	Nitric oxide synthase, inducible
Radix Glycyrrhizae (RG)	MOL002565	Medicarpin	Prostaglandin G/H synthase 1
Radix Glycyrrhizae (RG)	MOL002565	Medicarpin	Dopamine D1 receptor

Radix Glycyrrhizae (RG)	MOL002565	Medicarpin	Muscarinic acetylcholine receptor M3
Radix Glycyrrhizae (RG)	MOL002565	Medicarpin	Muscarinic acetylcholine receptor M1
Radix Glycyrrhizae (RG)	MOL002565	Medicarpin	Estrogen receptor
Radix Glycyrrhizae (RG)	MOL002565	Medicarpin	Sodium channel protein type 5 subunit alpha
Radix Glycyrrhizae (RG)	MOL002565	Medicarpin	Muscarinic acetylcholine receptor M5
Radix Glycyrrhizae (RG)	MOL002565	Medicarpin	Prostaglandin G/H synthase 2
Radix Glycyrrhizae (RG)	MOL002565	Medicarpin	Muscarinic acetylcholine receptor M4
Radix Glycyrrhizae (RG)	MOL002565	Medicarpin	Retinoic acid receptor RXR-alpha
Radix Glycyrrhizae (RG)	MOL002565	Medicarpin	5-hydroxytryptamine 2A receptor
Radix Glycyrrhizae (RG)	MOL002565	Medicarpin	Alpha-1A adrenergic receptor
Radix Glycyrrhizae (RG)	MOL002565	Medicarpin	Muscarinic acetylcholine receptor M2
Radix Glycyrrhizae (RG)	MOL002565	Medicarpin	Alpha-1B adrenergic receptor
Radix Glycyrrhizae (RG)	MOL002565	Medicarpin	Sodium-dependent dopamine
Radix Glycyrrhizae (RG)	MOL002565	Medicarpin	Beta-2 adrenergic receptor
Radix Glycyrrhizae (RG)	MOL002565	Medicarpin	Sodium-dependent serotonin transporter
Radix Glycyrrhizae (RG)	MOL002565	Medicarpin	Mu-type opioid receptor
Radix Glycyrrhizae (RG)	MOL002565	Medicarpin	Estrogen receptor beta
Radix Glycyrrhizae (RG)	MOL002565	Medicarpin	Dipeptidyl peptidase IV
Radix Glycyrrhizae (RG)	MOL002565	Medicarpin	Mitogen-activated protein kinase 10
Radix Glycyrrhizae (RG)	MOL002565	Medicarpin	Heat shock protein HSP 90
Radix Glycyrrhizae (RG)	MOL002565	Medicarpin	Cell division protein kinase 2
Radix Glycyrrhizae (RG)	MOL002565	Medicarpin	Phosphatidylinositol-4,5-bisphosphate 3-kinase catalytic subunit, gamma
Radix Glycyrrhizae (RG)	MOL002565	Medicarpin	Neuronal acetylcholine receptor protein, alpha-7 chain
Radix Glycyrrhizae (RG)	MOL002565	Medicarpin	mRNA of PKA Catalytic Subunit C-
Radix Glycyrrhizae (RG)	MOL002565	Medicarpin	Trypsin-1
Radix Glycyrrhizae (RG)	MOL002565	Medicarpin	Proto-oncogene serine/threonine-protein kinase Pim-1
Radix Glycyrrhizae (RG)	MOL002565	Medicarpin	Cyclin-A2
Radix Glycyrrhizae (RG)	MOL002565	Medicarpin	Calmodulin
Radix Glycyrrhizae (RG)	MOL002565	Medicarpin	Delta-type opioid receptor
Radix Glycyrrhizae (RG)	MOL002565	Medicarpin	CGMP-inhibited 3',5'-cyclic phosphodiesterase A
Radix Glycyrrhizae (RG)	MOL002565	Medicarpin	Alpha-1D adrenergic receptor
Radix Glycyrrhizae (RG)	MOL000354	isorhamnetin	Nitric oxide synthase, inducible
Radix Glycyrrhizae (RG)	MOL000354	isorhamnetin	Prostaglandin G/H synthase 1
Radix Glycyrrhizae (RG)	MOL000354	isorhamnetin	Estrogen receptor
Radix Glycyrrhizae (RG)	MOL000354	isorhamnetin	Androgen receptor
Radix Glycyrrhizae (RG)	MOL000354	isorhamnetin	Peroxisome proliferator activated receptor gamma
Radix Glycyrrhizae (RG)	MOL000354	isorhamnetin	Prostaglandin G/H synthase 2
Radix Glycyrrhizae (RG)	MOL000354	isorhamnetin	mRNA of Protein-tyrosine phosphatase, non-receptor type 1
Radix Glycyrrhizae (RG)	MOL000354	isorhamnetin	Estrogen receptor beta
Radix Glycyrrhizae (RG)	MOL000354	isorhamnetin	Dipeptidyl peptidase IV
Radix Glycyrrhizae (RG)	MOL000354	isorhamnetin	Mitogen-activated protein kinase 14
Radix Glycyrrhizae (RG)	MOL000354	isorhamnetin	Glycogen synthase kinase-3 beta
Radix Glycyrrhizae (RG)	MOL000354	isorhamnetin	Heat shock protein HSP 90
Radix Glycyrrhizae (RG)	MOL000354	isorhamnetin	Cell division protein kinase 2
Radix Glycyrrhizae (RG)	MOL000354	isorhamnetin	Phosphatidylinositol-4,5-bisphosphate 3-kinase catalytic subunit, gamma
Radix Glycyrrhizae (RG)	MOL000354	isorhamnetin	mRNA of PKA Catalytic Subunit C-
Radix Glycyrrhizae (RG)	MOL000354	isorhamnetin	Trypsin-1
Radix Glycyrrhizae (RG)	MOL000354	isorhamnetin	Proto-oncogene serine/threonine-protein kinase Pim-1
Radix Glycyrrhizae (RG)	MOL000354	isorhamnetin	Cyclin-A2
Radix Glycyrrhizae (RG)	MOL000354	isorhamnetin	Nuclear receptor coactivator 2
Radix Glycyrrhizae (RG)	MOL000354	isorhamnetin	Calmodulin
Radix Glycyrrhizae (RG)	MOL000354	isorhamnetin	Glycogen phosphorylase, muscle form
Radix Glycyrrhizae (RG)	MOL000354	isorhamnetin	Peroxisome proliferator activated receptor delta

Radix Glycyrrhizae (RG)	MOL000354	isorhamnetin	Serine/threonine-protein kinase Chk1
Radix Glycyrrhizae (RG)	MOL000354	isorhamnetin	Aldose reductase
Radix Glycyrrhizae (RG)	MOL000354	isorhamnetin	Nuclear receptor coactivator 1
Radix Glycyrrhizae (RG)	MOL000354	isorhamnetin	Coagulation factor VII
Radix Glycyrrhizae (RG)	MOL000354	isorhamnetin	Thrombin
Radix Glycyrrhizae (RG)	MOL000354	isorhamnetin	Nitric-oxide synthase, endothelial
Radix Glycyrrhizae (RG)	MOL000354	isorhamnetin	Acetylcholinesterase
Radix Glycyrrhizae (RG)	MOL000354	isorhamnetin	Gamma-aminobutyric acid receptor subunit alpha-1
Radix Glycyrrhizae (RG)	MOL000354	isorhamnetin	Amine oxidase [flavin-containing] B
Radix Glycyrrhizae (RG)	MOL000354	isorhamnetin	Glutamate receptor 2
Radix Glycyrrhizae (RG)	MOL000354	isorhamnetin	Cytochrome P450-cam
Radix Glycyrrhizae (RG)	MOL000354	isorhamnetin	Transcription factor p65
Radix Glycyrrhizae (RG)	MOL000354	isorhamnetin	Xanthine dehydrogenase/oxidase
Radix Glycyrrhizae (RG)	MOL000354	isorhamnetin	Neutrophil cytosol factor 1
Radix Glycyrrhizae (RG)	MOL000354	isorhamnetin	Oxidized low-density lipoprotein receptor 1
Radix Glycyrrhizae (RG)	MOL000359	sitosterol	Progesterone receptor
Radix Glycyrrhizae (RG)	MOL000359	sitosterol	Nuclear receptor coactivator 2
Radix Glycyrrhizae (RG)	MOL000359	sitosterol	Mineralocorticoid receptor
Radix Glycyrrhizae (RG)	MOL003656	Lupiwighteone	Nitric oxide synthase, inducible
Radix Glycyrrhizae (RG)	MOL003656	Lupiwighteone	Thrombin
Radix Glycyrrhizae (RG)	MOL003656	Lupiwighteone	Estrogen receptor
Radix Glycyrrhizae (RG)	MOL003656	Lupiwighteone	Androgen receptor
Radix Glycyrrhizae (RG)	MOL003656	Lupiwighteone	Sodium channel protein type 5 subunit alpha
Radix Glycyrrhizae (RG)	MOL003656	Lupiwighteone	Peroxisome proliferator activated receptor gamma
Radix Glycyrrhizae (RG)	MOL003656	Lupiwighteone	Coagulation factor Xa
Radix Glycyrrhizae (RG)	MOL003656	Lupiwighteone	Prostaglandin G/H synthase 2
Radix Glycyrrhizae (RG)	MOL003656	Lupiwighteone	DNA topoisomerase II
Radix Glycyrrhizae (RG)	MOL003656	Lupiwighteone	Estrogen receptor beta
Radix Glycyrrhizae (RG)	MOL003656	Lupiwighteone	Dipeptidyl peptidase IV
Radix Glycyrrhizae (RG)	MOL003656	Lupiwighteone	Mitogen-activated protein kinase 14
Radix Glycyrrhizae (RG)	MOL003656	Lupiwighteone	Glycogen synthase kinase-3 beta
Radix Glycyrrhizae (RG)	MOL003656	Lupiwighteone	Heat shock protein HSP 90
Radix Glycyrrhizae (RG)	MOL003656	Lupiwighteone	Cell division protein kinase 2
Radix Glycyrrhizae (RG)	MOL003656	Lupiwighteone	Serine/threonine-protein kinase Chk1
Radix Glycyrrhizae (RG)	MOL003656	Lupiwighteone	Trypsin-1
Radix Glycyrrhizae (RG)	MOL003656	Lupiwighteone	Proto-oncogene serine/threonine-protein kinase Pim-1
Radix Glycyrrhizae (RG)	MOL003656	Lupiwighteone	Cyclin-A2
Radix Glycyrrhizae (RG)	MOL003656	Lupiwighteone	Nuclear receptor coactivator 2
Radix Glycyrrhizae (RG)	MOL003656	Lupiwighteone	Calmodulin
Radix Glycyrrhizae (RG)	MOL003896	7-Methoxy-2-methyl	Nitric oxide synthase, inducible
Radix Glycyrrhizae (RG)	MOL003896	7-Methoxy-2-methyl	Prostaglandin G/H synthase 1
Radix Glycyrrhizae (RG)	MOL003896	7-Methoxy-2-methyl	Dopamine D1 receptor
Radix Glycyrrhizae (RG)	MOL003896	7-Methoxy-2-methyl	Muscarinic acetylcholine receptor M3
Radix Glycyrrhizae (RG)	MOL003896	7-Methoxy-2-methyl	Thrombin
Radix Glycyrrhizae (RG)	MOL003896	7-Methoxy-2-methyl	Muscarinic acetylcholine receptor M1
Radix Glycyrrhizae (RG)	MOL003896	7-Methoxy-2-methyl	Estrogen receptor
Radix Glycyrrhizae (RG)	MOL003896	7-Methoxy-2-methyl	Androgen receptor
Radix Glycyrrhizae (RG)	MOL003896	7-Methoxy-2-methyl	Beta-1 adrenergic receptor
Radix Glycyrrhizae (RG)	MOL003896	7-Methoxy-2-methyl isoflavone	Sodium channel protein type 5 subunit alpha
Radix Glycyrrhizae (RG)	MOL003896	7-Methoxy-2-methyl isoflavone	Peroxisome proliferator activated receptor gamma
Radix Glycyrrhizae (RG)	MOL003896	7-Methoxy-2-methyl	Prostaglandin G/H synthase 2
Radix Glycyrrhizae (RG)	MOL003896	7-Methoxy-2-methyl	Retinoic acid receptor RXR-alpha
Radix Glycyrrhizae (RG)	MOL003896	7-Methoxy-2-methyl	Acetylcholinesterase
Radix Glycyrrhizae (RG)	MOL003896	7-Methoxy-2-methyl isoflavone	CGMP-inhibited 3',5'-cyclic phosphodiesterase A
Radix Glycyrrhizae (RG)	MOL003896	7-Methoxy-2-methyl	Alpha-1B adrenergic receptor

Radix Glycyrrhizae (RG)	MOL003896	7-Methoxy-2-methyl	Sodium-dependent dopamine
Radix Glycyrrhizae (RG)	MOL003896	7-Methoxy-2-methyl	Beta-2 adrenergic receptor
Radix Glycyrrhizae (RG)	MOL003896	7-Methoxy-2-methyl	Alpha-1D adrenergic receptor
Radix Glycyrrhizae (RG)	MOL003896	7-Methoxy-2-methyl	Sodium-dependent serotonin transporter
Radix Glycyrrhizae (RG)	MOL003896	7-Methoxy-2-methyl	Estrogen receptor beta
Radix Glycyrrhizae (RG)	MOL003896	7-Methoxy-2-methyl isoflavone	Gamma-aminobutyric acid receptor subunit alpha-1
Radix Glycyrrhizae (RG)	MOL003896	7-Methoxy-2-methyl	Dipeptidyl peptidase IV
Radix Glycyrrhizae (RG)	MOL003896	7-Methoxy-2-methyl	Mitogen-activated protein kinase 14
Radix Glycyrrhizae (RG)	MOL003896	7-Methoxy-2-methyl	Glycogen synthase kinase-3 beta
Radix Glycyrrhizae (RG)	MOL003896	7-Methoxy-2-methyl	Heat shock protein HSP 90
Radix Glycyrrhizae (RG)	MOL003896	7-Methoxy-2-methyl	Cell division protein kinase 2
Radix Glycyrrhizae (RG)	MOL003896	7-Methoxy-2-methyl	Leukotriene A-4 hydrolase
Radix Glycyrrhizae (RG)	MOL003896	7-Methoxy-2-methyl	Amine oxidase [flavin-containing] B
Radix Glycyrrhizae (RG)	MOL003896	7-Methoxy-2-methyl isoflavone	Neuronal acetylcholine receptor protein, alpha-7 chain
Radix Glycyrrhizae (RG)	MOL003896	7-Methoxy-2-methyl	Serine/threonine-protein kinase Chk1
Radix Glycyrrhizae (RG)	MOL003896	7-Methoxy-2-methyl	mRNA of PKA Catalytic Subunit C-
Radix Glycyrrhizae (RG)	MOL003896	7-Methoxy-2-methyl	Ig gamma-1 chain C region
Radix Glycyrrhizae (RG)	MOL003896	7-Methoxy-2-methyl	Trypsin-1
Radix Glycyrrhizae (RG)	MOL003896	7-Methoxy-2-methyl isoflavone	Proto-oncogene serine/threonine-protein kinase Pim-1
Radix Glycyrrhizae (RG)	MOL003896	7-Methoxy-2-methyl	Cyclin-A2
Radix Glycyrrhizae (RG)	MOL003896	7-Methoxy-2-methyl	Nuclear receptor coactivator 1
Radix Glycyrrhizae (RG)	MOL003896	7-Methoxy-2-methyl isoflavone	cAMP-dependent protein kinase inhibitor alpha
Radix Glycyrrhizae (RG)	MOL003896	7-Methoxy-2-methyl	Calmodulin
Radix Glycyrrhizae (RG)	MOL003896	7-Methoxy-2-methyl	Muscarinic acetylcholine receptor M5
Radix Glycyrrhizae (RG)	MOL003896	7-Methoxy-2-methyl	Nitric-oxide synthase, endothelial
Radix Glycyrrhizae (RG)	MOL003896	7-Methoxy-2-methyl	Mu-type opioid receptor
Radix Glycyrrhizae (RG)	MOL003896	7-Methoxy-2-methyl	Nuclear receptor coactivator 2
Radix Glycyrrhizae (RG)	MOL000392	formononetin	Nitric oxide synthase, inducible
Radix Glycyrrhizae (RG)	MOL000392	formononetin	Prostaglandin G/H synthase 1
Radix Glycyrrhizae (RG)	MOL000392	formononetin	Muscarinic acetylcholine receptor M1
Radix Glycyrrhizae (RG)	MOL000392	formononetin	Estrogen receptor
Radix Glycyrrhizae (RG)	MOL000392	formononetin	Androgen receptor
Radix Glycyrrhizae (RG)	MOL000392	formononetin	Peroxisome proliferator activated receptor gamma
Radix Glycyrrhizae (RG)	MOL000392	formononetin	Prostaglandin G/H synthase 2
Radix Glycyrrhizae (RG)	MOL000392	formononetin	Retinoic acid receptor RXR-alpha
Radix Glycyrrhizae (RG)	MOL000392	formononetin	CGMP-inhibited 3',5'-cyclic phosphodiesterase A
Radix Glycyrrhizae (RG)	MOL000392	formononetin	Alpha-1A adrenergic receptor
Radix Glycyrrhizae (RG)	MOL000392	formononetin	Sodium-dependent dopamine
Radix Glycyrrhizae (RG)	MOL000392	formononetin	Beta-2 adrenergic receptor
Radix Glycyrrhizae (RG)	MOL000392	formononetin	Sodium-dependent serotonin transporter
Radix Glycyrrhizae (RG)	MOL000392	formononetin	Estrogen receptor beta
Radix Glycyrrhizae (RG)	MOL000392	formononetin	Dipeptidyl peptidase IV
Radix Glycyrrhizae (RG)	MOL000392	formononetin	Mitogen-activated protein kinase 14
Radix Glycyrrhizae (RG)	MOL000392	formononetin	Glycogen synthase kinase-3 beta
Radix Glycyrrhizae (RG)	MOL000392	formononetin	Heat shock protein HSP 90
Radix Glycyrrhizae (RG)	MOL000392	formononetin	Cell division protein kinase 2
Radix Glycyrrhizae (RG)	MOL000392	formononetin	Amine oxidase [flavin-containing] B
Radix Glycyrrhizae (RG)	MOL000392	formononetin	Serine/threonine-protein kinase Chk1
Radix Glycyrrhizae (RG)	MOL000392	formononetin	mRNA of PKA Catalytic Subunit C-
Radix Glycyrrhizae (RG)	MOL000392	formononetin	Trypsin-1
Radix Glycyrrhizae (RG)	MOL000392	formononetin	Proto-oncogene serine/threonine-protein kinase Pim-1
Radix Glycyrrhizae (RG)	MOL000392	formononetin	Cyclin-A2
Radix Glycyrrhizae (RG)	MOL000392	formononetin	Calmodulin
Radix Glycyrrhizae (RG)	MOL000392	formononetin	cAMP-dependent protein kinase inhibitor alpha
Radix Glycyrrhizae (RG)	MOL000392	formononetin	Thrombin

Radix Glycyrrhizae (RG)	MOL000392	formononetin	Nitric-oxide synthase, endothelial
Radix Glycyrrhizae (RG)	MOL000392	formononetin	Acetylcholinesterase
Radix Glycyrrhizae (RG)	MOL000392	formononetin	Beta-lactamase
Radix Glycyrrhizae (RG)	MOL000392	formononetin	Transcription factor AP-1
Radix Glycyrrhizae (RG)	MOL000392	formononetin	Peroxisome proliferator-activated receptor gamma
Radix Glycyrrhizae (RG)	MOL000392	formononetin	Interleukin-4
Radix Glycyrrhizae (RG)	MOL000392	formononetin	NAD-dependent deacetylase sirtuin-1
Radix Glycyrrhizae (RG)	MOL000392	formononetin	ATP synthase subunit beta,
Radix Glycyrrhizae (RG)	MOL000392	formononetin	NADH-ubiquinone oxidoreductase
Radix Glycyrrhizae (RG)	MOL000392	formononetin	3 beta-hydroxysteroid dehydrogenase/Delta 5-->4-isomerase type 2
Radix Glycyrrhizae (RG)	MOL000392	formononetin	3 beta-hydroxysteroid dehydrogenase/Delta 5-->4-isomerase type 1
Radix Glycyrrhizae (RG)	MOL000417	Calycosin	Nitric oxide synthase, inducible
Radix Glycyrrhizae (RG)	MOL000417	Calycosin	Prostaglandin G/H synthase 1
Radix Glycyrrhizae (RG)	MOL000417	Calycosin	Estrogen receptor
Radix Glycyrrhizae (RG)	MOL000417	Calycosin	Androgen receptor
Radix Glycyrrhizae (RG)	MOL000417	Calycosin	Peroxisome proliferator activated receptor gamma
Radix Glycyrrhizae (RG)	MOL000417	Calycosin	Prostaglandin G/H synthase 2
Radix Glycyrrhizae (RG)	MOL000417	Calycosin	Retinoic acid receptor RXR-alpha
Radix Glycyrrhizae (RG)	MOL000417	Calycosin	CGMP-inhibited 3',5'-cyclic phosphodiesterase A
Radix Glycyrrhizae (RG)	MOL000417	Calycosin	Estrogen receptor beta
Radix Glycyrrhizae (RG)	MOL000417	Calycosin	Dipeptidyl peptidase IV
Radix Glycyrrhizae (RG)	MOL000417	Calycosin	Mitogen-activated protein kinase 14
Radix Glycyrrhizae (RG)	MOL000417	Calycosin	Glycogen synthase kinase-3 beta
Radix Glycyrrhizae (RG)	MOL000417	Calycosin	Heat shock protein HSP 90
Radix Glycyrrhizae (RG)	MOL000417	Calycosin	Cell division protein kinase 2
Radix Glycyrrhizae (RG)	MOL000417	Calycosin	Serine/threonine-protein kinase Chk1
Radix Glycyrrhizae (RG)	MOL000417	Calycosin	mRNA of PKA Catalytic Subunit C-
Radix Glycyrrhizae (RG)	MOL000417	Calycosin	Trypsin-1
Radix Glycyrrhizae (RG)	MOL000417	Calycosin	Proto-oncogene serine/threonine-protein kinase Pim-1
Radix Glycyrrhizae (RG)	MOL000417	Calycosin	Cyclin-A2
Radix Glycyrrhizae (RG)	MOL000417	Calycosin	Nuclear receptor coactivator 2
Radix Glycyrrhizae (RG)	MOL000417	Calycosin	Calmodulin
Radix Glycyrrhizae (RG)	MOL000417	Calycosin	Beta-2 adrenergic receptor
Radix Glycyrrhizae (RG)	MOL000422	kaempferol	Nitric oxide synthase, inducible
Radix Glycyrrhizae (RG)	MOL000422	kaempferol	Prostaglandin G/H synthase 1
Radix Glycyrrhizae (RG)	MOL000422	kaempferol	Androgen receptor
Radix Glycyrrhizae (RG)	MOL000422	kaempferol	Peroxisome proliferator activated receptor gamma
Radix Glycyrrhizae (RG)	MOL000422	kaempferol	Prostaglandin G/H synthase 2
Radix Glycyrrhizae (RG)	MOL000422	kaempferol	Heat shock protein HSP 90
Radix Glycyrrhizae (RG)	MOL000422	kaempferol	Phosphatidylinositol-4,5-bisphosphate 3-kinase catalytic subunit, gamma
Radix Glycyrrhizae (RG)	MOL000422	kaempferol	mRNA of PKA Catalytic Subunit C-
Radix Glycyrrhizae (RG)	MOL000422	kaempferol	Nuclear receptor coactivator 2
Radix Glycyrrhizae (RG)	MOL000422	kaempferol	Dipeptidyl peptidase IV
Radix Glycyrrhizae (RG)	MOL000422	kaempferol	Trypsin-1
Radix Glycyrrhizae (RG)	MOL000422	kaempferol	Progesterone receptor
Radix Glycyrrhizae (RG)	MOL000422	kaempferol	Thrombin
Radix Glycyrrhizae (RG)	MOL000422	kaempferol	Muscarinic acetylcholine receptor M1
Radix Glycyrrhizae (RG)	MOL000422	kaempferol	Nitric-oxide synthase, endothelial
Radix Glycyrrhizae (RG)	MOL000422	kaempferol	Gamma-aminobutyric-acid receptor alpha-2 subunit
Radix Glycyrrhizae (RG)	MOL000422	kaempferol	Acetylcholinesterase
Radix Glycyrrhizae (RG)	MOL000422	kaempferol	Sodium-dependent noradrenaline transporter

Radix Glycyrrhizae (RG)	MOL000422	kaempferol	Muscarinic acetylcholine receptor M2
Radix Glycyrrhizae (RG)	MOL000422	kaempferol	Alpha-1B adrenergic receptor
Radix Glycyrrhizae (RG)	MOL000422	kaempferol	Gamma-aminobutyric acid receptor subunit alpha-1
Radix Glycyrrhizae (RG)	MOL000422	kaempferol	DNA topoisomerase II
Radix Glycyrrhizae (RG)	MOL000422	kaempferol	Coagulation factor VII
Radix Glycyrrhizae (RG)	MOL000422	kaempferol	Calmodulin
Radix Glycyrrhizae (RG)	MOL000422	kaempferol	Transcription factor p65
Radix Glycyrrhizae (RG)	MOL000422	kaempferol	Inhibitor of nuclear factor kappa-B kinase subunit beta
Radix Glycyrrhizae (RG)	MOL000422	kaempferol	RAC-alpha serine/threonine-protein kinase
Radix Glycyrrhizae (RG)	MOL000422	kaempferol	Apoptosis regulator Bcl-2
Radix Glycyrrhizae (RG)	MOL000422	kaempferol	Apoptosis regulator BAX
Radix Glycyrrhizae (RG)	MOL000422	kaempferol	Tumor necrosis factor
Radix Glycyrrhizae (RG)	MOL000422	kaempferol	Transcription factor AP-1
Radix Glycyrrhizae (RG)	MOL000422	kaempferol	Activator of 90 kDa heat shock protein ATPase homolog 1
Radix Glycyrrhizae (RG)	MOL000422	kaempferol	Caspase-3
Radix Glycyrrhizae (RG)	MOL000422	kaempferol	Mitogen-activated protein kinase 8
Radix Glycyrrhizae (RG)	MOL000422	kaempferol	Xanthine dehydrogenase/oxidase
Radix Glycyrrhizae (RG)	MOL000422	kaempferol	Interstitial collagenase
Radix Glycyrrhizae (RG)	MOL000422	kaempferol	Signal transducer and activator of transcription 1-alpha/beta
Radix Glycyrrhizae (RG)	MOL000422	kaempferol	Cell division control protein 2 homolog
Radix Glycyrrhizae (RG)	MOL000422	kaempferol	Peroxisome proliferator-activated receptor gamma
Radix Glycyrrhizae (RG)	MOL000422	kaempferol	Heme oxygenase 1
Radix Glycyrrhizae (RG)	MOL000422	kaempferol	Cytochrome P450 3A4
Radix Glycyrrhizae (RG)	MOL000422	kaempferol	Cytochrome P450 1A2
Radix Glycyrrhizae (RG)	MOL000422	kaempferol	Cytochrome P450 1A1
Radix Glycyrrhizae (RG)	MOL000422	kaempferol	Intercellular adhesion molecule 1
Radix Glycyrrhizae (RG)	MOL000422	kaempferol	E-selectin
Radix Glycyrrhizae (RG)	MOL000422	kaempferol	Vascular cell adhesion protein 1
Radix Glycyrrhizae (RG)	MOL000422	kaempferol	Nuclear receptor subfamily 1 group I member 2
Radix Glycyrrhizae (RG)	MOL000422	kaempferol	Cytochrome P450 1B1
Radix Glycyrrhizae (RG)	MOL000422	kaempferol	Arachidonate 5-lipoxygenase
Radix Glycyrrhizae (RG)	MOL000422	kaempferol	Hyaluronan synthase 2
Radix Glycyrrhizae (RG)	MOL000422	kaempferol	Glutathione S-transferase P
Radix Glycyrrhizae (RG)	MOL000422	kaempferol	Aryl hydrocarbon receptor
Radix Glycyrrhizae (RG)	MOL000422	kaempferol	26S proteasome non-ATPase regulatory subunit 3
Radix Glycyrrhizae (RG)	MOL000422	kaempferol	Solute carrier family 2, facilitated glucose transporter member 4
Radix Glycyrrhizae (RG)	MOL000422	kaempferol	Nuclear receptor subfamily 1 group I member 3
Radix Glycyrrhizae (RG)	MOL000422	kaempferol	Insulin receptor
Radix Glycyrrhizae (RG)	MOL000422	kaempferol	Type I iodothyronine deiodinase
Radix Glycyrrhizae (RG)	MOL000422	kaempferol	Serine/threonine-protein phosphatase 2B catalytic subunit alpha isoform
Radix Glycyrrhizae (RG)	MOL000422	kaempferol	Peroxidase C1A
Radix Glycyrrhizae (RG)	MOL000422	kaempferol	Glutathione S-transferase Mu 1
Radix Glycyrrhizae (RG)	MOL000422	kaempferol	Glutathione S-transferase Mu 2
Radix Glycyrrhizae (RG)	MOL000422	kaempferol	Aldo-keto reductase family 1 member
Radix Glycyrrhizae (RG)	MOL000422	kaempferol	Antileukoproteinase
Radix Glycyrrhizae (RG)	MOL004328	naringenin	Prostaglandin G/H synthase 1
Radix Glycyrrhizae (RG)	MOL004328	naringenin	Estrogen receptor
Radix Glycyrrhizae (RG)	MOL004328	naringenin	Prostaglandin G/H synthase 2
Radix Glycyrrhizae (RG)	MOL004328	naringenin	Heat shock protein HSP 90
Radix Glycyrrhizae (RG)	MOL004328	naringenin	Beta-lactamase
Radix Glycyrrhizae (RG)	MOL004328	naringenin	mRNA of PKA Catalytic Subunit C-

Radix Glycyrrhizae (RG)	MOL004328	naringenin	Phosphatidylinositol-4,5-bisphosphate 3-kinase catalytic subunit, gamma
Radix Glycyrrhizae (RG)	MOL004328	naringenin	Transcription factor p65
Radix Glycyrrhizae (RG)	MOL004328	naringenin	RAC-alpha serine/threonine-protein kinase
Radix Glycyrrhizae (RG)	MOL004328	naringenin	Apoptosis regulator Bcl-2
Radix Glycyrrhizae (RG)	MOL004328	naringenin	Mitogen-activated protein kinase 3
Radix Glycyrrhizae (RG)	MOL004328	naringenin	Mitogen-activated protein kinase 1
Radix Glycyrrhizae (RG)	MOL004328	naringenin	Caspase-3
Radix Glycyrrhizae (RG)	MOL004328	naringenin	Fatty acid synthase
Radix Glycyrrhizae (RG)	MOL004328	naringenin	Low-density lipoprotein receptor
Radix Glycyrrhizae (RG)	MOL004328	naringenin	Bcl2 antagonist of cell death
Radix Glycyrrhizae (RG)	MOL004328	naringenin	Superoxide dismutase [Cu-Zn]
Radix Glycyrrhizae (RG)	MOL004328	naringenin	Catalase
Radix Glycyrrhizae (RG)	MOL004328	naringenin	Peroxisome proliferator-activated receptor gamma
Radix Glycyrrhizae (RG)	MOL004328	naringenin	Microsomal triglyceride transfer protein large subunit
Radix Glycyrrhizae (RG)	MOL004328	naringenin	Apolipoprotein B-100
Radix Glycyrrhizae (RG)	MOL004328	naringenin	Phospholipase B1, membrane-
Radix Glycyrrhizae (RG)	MOL004328	naringenin	3-hydroxy-3-methylglutaryl-coenzyme A reductase
Radix Glycyrrhizae (RG)	MOL004328	naringenin	Cytochrome P450 19A1
Radix Glycyrrhizae (RG)	MOL004328	naringenin	Glutathione S-transferase P
Radix Glycyrrhizae (RG)	MOL004328	naringenin	UDP-glucuronosyltransferase 1-1
Radix Glycyrrhizae (RG)	MOL004328	naringenin	Peroxisome proliferator-activated receptor alpha
Radix Glycyrrhizae (RG)	MOL004328	naringenin	Sterol regulatory element-binding protein 1
Radix Glycyrrhizae (RG)	MOL004328	naringenin	Glutathione reductase, mitochondrial
Radix Glycyrrhizae (RG)	MOL004328	naringenin	Multidrug resistance-associated protein
Radix Glycyrrhizae (RG)	MOL004328	naringenin	Adiponectin
Radix Glycyrrhizae (RG)	MOL004328	naringenin	Sterol O-acyltransferase 2
Radix Glycyrrhizae (RG)	MOL004328	naringenin	Aldo-keto reductase family 1 member
Radix Glycyrrhizae (RG)	MOL004328	naringenin	Aspartate aminotransferase, cytoplasmic
Radix Glycyrrhizae (RG)	MOL004328	naringenin	4-aminobutyrate aminotransferase, mitochondrial
Radix Glycyrrhizae (RG)	MOL004328	naringenin	Liver carboxylesterase 1
Radix Glycyrrhizae (RG)	MOL004328	naringenin	Sterol O-acyltransferase 1
Radix Glycyrrhizae (RG)	MOL004805	(2S)-2-[4-hydroxy-3-(3-methylbut-2-enyl)phenyl]-8,8-dimethyl-2,3-dihydropyrano[2,3-f]chromen-	Nitric oxide synthase, inducible
Radix Glycyrrhizae (RG)	MOL004805	(2S)-2-[4-hydroxy-3-(3-methylbut-2-enyl)phenyl]-8,8-dimethyl-2,3-dihydropyrano[2,3-f]chromen-	Potassium voltage-gated channel subfamily H member 2
Radix Glycyrrhizae (RG)	MOL004805	(2S)-2-[4-hydroxy-3-(3-methylbut-2-enyl)phenyl]-8,8-dimethyl-2,3-dihydropyrano[2,3-f]chromen-	Estrogen receptor
Radix Glycyrrhizae (RG)	MOL004805	(2S)-2-[4-hydroxy-3-(3-methylbut-2-enyl)phenyl]-8,8-dimethyl-2,3-dihydropyrano[2,3-f]chromen-	Androgen receptor
Radix Glycyrrhizae (RG)	MOL004805	(2S)-2-[4-hydroxy-3-(3-methylbut-2-enyl)phenyl]-8,8-dimethyl-2,3-dihydropyrano[2,3-f]chromen-	Peroxisome proliferator activated receptor gamma
Radix Glycyrrhizae (RG)	MOL004805	(2S)-2-[4-hydroxy-3-(3-methylbut-2-enyl)phenyl]-8,8-dimethyl-2,3-dihydropyrano[2,3-f]chromen-	Coagulation factor Xa

Radix Glycyrrhizae (RG)	MOL004805	(2S)-2-[4-hydroxy-3-(3-methylbut-2-enyl)phenyl]-8,8-dimethyl-2,3-dihydropyrano[2,3-f]chromen-	Prostaglandin G/H synthase 2
Radix Glycyrrhizae (RG)	MOL004805	(2S)-2-[4-hydroxy-3-(3-methylbut-2-enyl)phenyl]-8,8-dimethyl-2,3-dihydropyrano[2,3-f]chromen-	Estrogen receptor beta
Radix Glycyrrhizae (RG)	MOL004805	(2S)-2-[4-hydroxy-3-(3-methylbut-2-enyl)phenyl]-8,8-dimethyl-2,3-dihydropyrano[2,3-f]chromen-	Mitogen-activated protein kinase 14
Radix Glycyrrhizae (RG)	MOL004805	(2S)-2-[4-hydroxy-3-(3-methylbut-2-enyl)phenyl]-8,8-dimethyl-2,3-dihydropyrano[2,3-f]chromen-	Glycogen synthase kinase-3 beta
Radix Glycyrrhizae (RG)	MOL004805	(2S)-2-[4-hydroxy-3-(3-methylbut-2-enyl)phenyl]-8,8-dimethyl-2,3-dihydropyrano[2,3-f]chromen-	Proto-oncogene serine/threonine-protein kinase Pim-1
Radix Glycyrrhizae (RG)	MOL004805	(2S)-2-[4-hydroxy-3-(3-methylbut-2-enyl)phenyl]-8,8-dimethyl-2,3-dihydropyrano[2,3-f]chromen-	Calmodulin
Radix Glycyrrhizae (RG)	MOL004806	euchrenone	Nitric oxide synthase, inducible
Radix Glycyrrhizae (RG)	MOL004806	euchrenone	Potassium voltage-gated channel subfamily H member 2
Radix Glycyrrhizae (RG)	MOL004806	euchrenone	Estrogen receptor
Radix Glycyrrhizae (RG)	MOL004806	euchrenone	Sodium channel protein type 5 subunit alpha
Radix Glycyrrhizae (RG)	MOL004806	euchrenone	Coagulation factor Xa
Radix Glycyrrhizae (RG)	MOL004806	euchrenone	Prostaglandin G/H synthase 2
Radix Glycyrrhizae (RG)	MOL004806	euchrenone	Estrogen receptor beta
Radix Glycyrrhizae (RG)	MOL004806	euchrenone	Beta-secretase
Radix Glycyrrhizae (RG)	MOL004806	euchrenone	Proto-oncogene serine/threonine-protein kinase Pim-1
Radix Glycyrrhizae (RG)	MOL004806	euchrenone	Calmodulin
Radix Glycyrrhizae (RG)	MOL004808	glyasperin B	Nitric oxide synthase, inducible
Radix Glycyrrhizae (RG)	MOL004808	glyasperin B	Thrombin
Radix Glycyrrhizae (RG)	MOL004808	glyasperin B	Estrogen receptor
Radix Glycyrrhizae (RG)	MOL004808	glyasperin B	Androgen receptor
Radix Glycyrrhizae (RG)	MOL004808	glyasperin B	Peroxisome proliferator activated receptor gamma
Radix Glycyrrhizae (RG)	MOL004808	glyasperin B	Coagulation factor Xa
Radix Glycyrrhizae (RG)	MOL004808	glyasperin B	Prostaglandin G/H synthase 2
Radix Glycyrrhizae (RG)	MOL004808	glyasperin B	Coagulation factor VII
Radix Glycyrrhizae (RG)	MOL004808	glyasperin B	Vascular endothelial growth factor receptor 2
Radix Glycyrrhizae (RG)	MOL004808	glyasperin B	Acetylcholinesterase
Radix Glycyrrhizae (RG)	MOL004808	glyasperin B	DNA topoisomerase II
Radix Glycyrrhizae (RG)	MOL004808	glyasperin B	Estrogen receptor beta
Radix Glycyrrhizae (RG)	MOL004808	glyasperin B	Dipeptidyl peptidase IV
Radix Glycyrrhizae (RG)	MOL004808	glyasperin B	Glycogen synthase kinase-3 beta
Radix Glycyrrhizae (RG)	MOL004808	glyasperin B	Heat shock protein HSP 90
Radix Glycyrrhizae (RG)	MOL004808	glyasperin B	Cell division protein kinase 2
Radix Glycyrrhizae (RG)	MOL004808	glyasperin B	Trypsin-1
Radix Glycyrrhizae (RG)	MOL004808	glyasperin B	Proto-oncogene serine/threonine-protein kinase Pim-1
Radix Glycyrrhizae (RG)	MOL004808	glyasperin B	Cyclin-A2
Radix Glycyrrhizae (RG)	MOL004808	glyasperin B	Nuclear receptor coactivator 2
Radix Glycyrrhizae (RG)	MOL004808	glyasperin B	Calmodulin
Radix Glycyrrhizae (RG)	MOL004810	glyasperin F	Nitric oxide synthase, inducible
Radix Glycyrrhizae (RG)	MOL004810	glyasperin F	Prostaglandin G/H synthase 1

Radix Glycyrrhizae (RG)	MOL004810	glyasperin F	Estrogen receptor
Radix Glycyrrhizae (RG)	MOL004810	glyasperin F	Androgen receptor
Radix Glycyrrhizae (RG)	MOL004810	glyasperin F	Sodium channel protein type 5 subunit alpha
Radix Glycyrrhizae (RG)	MOL004810	glyasperin F	Peroxisome proliferator activated receptor gamma
Radix Glycyrrhizae (RG)	MOL004810	glyasperin F	Coagulation factor Xa
Radix Glycyrrhizae (RG)	MOL004810	glyasperin F	Prostaglandin G/H synthase 2
Radix Glycyrrhizae (RG)	MOL004810	glyasperin F	DNA topoisomerase II
Radix Glycyrrhizae (RG)	MOL004810	glyasperin F	Estrogen receptor beta
Radix Glycyrrhizae (RG)	MOL004810	glyasperin F	Mitogen-activated protein kinase 14
Radix Glycyrrhizae (RG)	MOL004810	glyasperin F	Glycogen synthase kinase-3 beta
Radix Glycyrrhizae (RG)	MOL004810	glyasperin F	Heat shock protein HSP 90
Radix Glycyrrhizae (RG)	MOL004810	glyasperin F	Cell division protein kinase 2
Radix Glycyrrhizae (RG)	MOL004810	glyasperin F	Trypsin-1
Radix Glycyrrhizae (RG)	MOL004810	glyasperin F	Proto-oncogene serine/threonine-protein kinase Pim-1
Radix Glycyrrhizae (RG)	MOL004810	glyasperin F	Cyclin-A2
Radix Glycyrrhizae (RG)	MOL004810	glyasperin F	Calmodulin
Radix Glycyrrhizae (RG)	MOL004811	Glyasperin C	Nitric oxide synthase, inducible
Radix Glycyrrhizae (RG)	MOL004811	Glyasperin C	Thrombin
Radix Glycyrrhizae (RG)	MOL004811	Glyasperin C	Potassium voltage-gated channel subfamily H member 2
Radix Glycyrrhizae (RG)	MOL004811	Glyasperin C	Estrogen receptor
Radix Glycyrrhizae (RG)	MOL004811	Glyasperin C	Androgen receptor
Radix Glycyrrhizae (RG)	MOL004811	Glyasperin C	Sodium channel protein type 5 subunit alpha
Radix Glycyrrhizae (RG)	MOL004811	Glyasperin C	Peroxisome proliferator activated receptor gamma
Radix Glycyrrhizae (RG)	MOL004811	Glyasperin C	Coagulation factor Xa
Radix Glycyrrhizae (RG)	MOL004811	Glyasperin C	Prostaglandin G/H synthase 2
Radix Glycyrrhizae (RG)	MOL004811	Glyasperin C	Retinoic acid receptor RXR-alpha
Radix Glycyrrhizae (RG)	MOL004811	Glyasperin C	Acetylcholinesterase
Radix Glycyrrhizae (RG)	MOL004811	Glyasperin C	DNA topoisomerase II
Radix Glycyrrhizae (RG)	MOL004811	Glyasperin C	Estrogen receptor beta
Radix Glycyrrhizae (RG)	MOL004811	Glyasperin C	Dipeptidyl peptidase IV
Radix Glycyrrhizae (RG)	MOL004811	Glyasperin C	Mitogen-activated protein kinase 14
Radix Glycyrrhizae (RG)	MOL004811	Glyasperin C	Glycogen synthase kinase-3 beta
Radix Glycyrrhizae (RG)	MOL004811	Glyasperin C	Heat shock protein HSP 90
Radix Glycyrrhizae (RG)	MOL004811	Glyasperin C	Cell division protein kinase 2
Radix Glycyrrhizae (RG)	MOL004811	Glyasperin C	Serine/threonine-protein kinase Chk1
Radix Glycyrrhizae (RG)	MOL004811	Glyasperin C	Trypsin-1
Radix Glycyrrhizae (RG)	MOL004811	Glyasperin C	Proto-oncogene serine/threonine-protein kinase Pim-1
Radix Glycyrrhizae (RG)	MOL004811	Glyasperin C	Cyclin-A2
Radix Glycyrrhizae (RG)	MOL004811	Glyasperin C	Nuclear receptor coactivator 2
Radix Glycyrrhizae (RG)	MOL004811	Glyasperin C	Calmodulin
Radix Glycyrrhizae (RG)	MOL004814	Isotrifoliol	Nitric oxide synthase, inducible
Radix Glycyrrhizae (RG)	MOL004814	Isotrifoliol	Estrogen receptor
Radix Glycyrrhizae (RG)	MOL004814	Isotrifoliol	Androgen receptor
Radix Glycyrrhizae (RG)	MOL004814	Isotrifoliol	Prostaglandin G/H synthase 2
Radix Glycyrrhizae (RG)	MOL004814	Isotrifoliol	Estrogen receptor beta
Radix Glycyrrhizae (RG)	MOL004814	Isotrifoliol	Mitogen-activated protein kinase 14
Radix Glycyrrhizae (RG)	MOL004814	Isotrifoliol	Glycogen synthase kinase-3 beta
Radix Glycyrrhizae (RG)	MOL004814	Isotrifoliol	Heat shock protein HSP 90
Radix Glycyrrhizae (RG)	MOL004814	Isotrifoliol	Cell division protein kinase 2
Radix Glycyrrhizae (RG)	MOL004814	Isotrifoliol	Phosphatidylinositol-4,5-bisphosphate 3-kinase catalytic subunit, gamma
Radix Glycyrrhizae (RG)	MOL004814	Isotrifoliol	Serine/threonine-protein kinase Chk1
Radix Glycyrrhizae (RG)	MOL004814	Isotrifoliol	mRNA of PKA Catalytic Subunit C-
Radix Glycyrrhizae (RG)	MOL004814	Isotrifoliol	Proto-oncogene serine/threonine-protein kinase Pim-1
Radix Glycyrrhizae (RG)	MOL004814	Isotrifoliol	Cyclin-A2

Radix Glycyrrhizae (RG)	MOL004815	(E)-1-(2,4-dihydroxyphenyl)-3-(2,2-dimethylchromen-6-yl)prop-2-en-1-one	Nitric oxide synthase, inducible
Radix Glycyrrhizae (RG)	MOL004815	(E)-1-(2,4-dihydroxyphenyl)-3-(2,2-dimethylchromen-6-yl)prop-2-en-1-one	Prostaglandin G/H synthase 1
Radix Glycyrrhizae (RG)	MOL004815	(E)-1-(2,4-dihydroxyphenyl)-3-(2,2-dimethylchromen-6-yl)prop-2-en-1-one	Estrogen receptor
Radix Glycyrrhizae (RG)	MOL004815	(E)-1-(2,4-dihydroxyphenyl)-3-(2,2-dimethylchromen-6-yl)prop-2-en-1-one	Androgen receptor
Radix Glycyrrhizae (RG)	MOL004815	(E)-1-(2,4-dihydroxyphenyl)-3-(2,2-dimethylchromen-6-yl)prop-2-en-1-one	Sodium channel protein type 5 subunit alpha
Radix Glycyrrhizae (RG)	MOL004815	(E)-1-(2,4-dihydroxyphenyl)-3-(2,2-dimethylchromen-6-yl)prop-2-en-1-one	Peroxisome proliferator activated receptor gamma
Radix Glycyrrhizae (RG)	MOL004815	(E)-1-(2,4-dihydroxyphenyl)-3-(2,2-dimethylchromen-6-yl)prop-2-en-1-one	Coagulation factor Xa
Radix Glycyrrhizae (RG)	MOL004815	(E)-1-(2,4-dihydroxyphenyl)-3-(2,2-dimethylchromen-6-yl)prop-2-en-1-one	Prostaglandin G/H synthase 2
Radix Glycyrrhizae (RG)	MOL004815	(E)-1-(2,4-dihydroxyphenyl)-3-(2,2-dimethylchromen-6-yl)prop-2-en-1-one	Carbonic anhydrase II
Radix Glycyrrhizae (RG)	MOL004815	(E)-1-(2,4-dihydroxyphenyl)-3-(2,2-dimethylchromen-6-yl)prop-2-en-1-one	Retinoic acid receptor RXR-alpha
Radix Glycyrrhizae (RG)	MOL004815	(E)-1-(2,4-dihydroxyphenyl)-3-(2,2-dimethylchromen-6-yl)prop-2-en-1-one	Alpha-1B adrenergic receptor
Radix Glycyrrhizae (RG)	MOL004815	(E)-1-(2,4-dihydroxyphenyl)-3-(2,2-dimethylchromen-6-yl)prop-2-en-1-one	Estrogen receptor beta
Radix Glycyrrhizae (RG)	MOL004815	(E)-1-(2,4-dihydroxyphenyl)-3-(2,2-dimethylchromen-6-yl)prop-2-en-1-one	Mitogen-activated protein kinase 14
Radix Glycyrrhizae (RG)	MOL004815	(E)-1-(2,4-dihydroxyphenyl)-3-(2,2-dimethylchromen-6-yl)prop-2-en-1-one	Glycogen synthase kinase-3 beta
Radix Glycyrrhizae (RG)	MOL004815	(E)-1-(2,4-dihydroxyphenyl)-3-(2,2-dimethylchromen-6-yl)prop-2-en-1-one	Cell division protein kinase 2
Radix Glycyrrhizae (RG)	MOL004815	(E)-1-(2,4-dihydroxyphenyl)-3-(2,2-dimethylchromen-6-yl)prop-2-en-1-one	Serine/threonine-protein kinase Chk1
Radix Glycyrrhizae (RG)	MOL004815	(E)-1-(2,4-dihydroxyphenyl)-3-(2,2-dimethylchromen-6-yl)prop-2-en-1-one	Proto-oncogene serine/threonine-protein kinase Pim-1
Radix Glycyrrhizae (RG)	MOL004815	(E)-1-(2,4-dihydroxyphenyl)-3-(2,2-dimethylchromen-6-yl)prop-2-en-1-one	Cyclin-A2
Radix Glycyrrhizae (RG)	MOL004815	(E)-1-(2,4-dihydroxyphenyl)-3-(2,2-dimethylchromen-6-yl)prop-2-en-1-one	Nuclear receptor coactivator 2
Radix Glycyrrhizae (RG)	MOL004815	(E)-1-(2,4-dihydroxyphenyl)-3-(2,2-dimethylchromen-6-yl)prop-2-en-1-one	Calmodulin
Radix Glycyrrhizae (RG)	MOL004820	kanzonols W	Nitric oxide synthase, inducible
Radix Glycyrrhizae (RG)	MOL004820	kanzonols W	Prostaglandin G/H synthase 1
Radix Glycyrrhizae (RG)	MOL004820	kanzonols W	Estrogen receptor

Radix Glycyrrhizae (RG)	MOL004820	kanzonols W	Androgen receptor
Radix Glycyrrhizae (RG)	MOL004820	kanzonols W	Sodium channel protein type 5 subunit alpha
Radix Glycyrrhizae (RG)	MOL004820	kanzonols W	Peroxisome proliferator activated receptor gamma
Radix Glycyrrhizae (RG)	MOL004820	kanzonols W	Coagulation factor Xa
Radix Glycyrrhizae (RG)	MOL004820	kanzonols W	Prostaglandin G/H synthase 2
Radix Glycyrrhizae (RG)	MOL004820	kanzonols W	Retinoic acid receptor RXR-alpha
Radix Glycyrrhizae (RG)	MOL004820	kanzonols W	DNA topoisomerase II
Radix Glycyrrhizae (RG)	MOL004820	kanzonols W	Estrogen receptor beta
Radix Glycyrrhizae (RG)	MOL004820	kanzonols W	Mitogen-activated protein kinase 14
Radix Glycyrrhizae (RG)	MOL004820	kanzonols W	Glycogen synthase kinase-3 beta
Radix Glycyrrhizae (RG)	MOL004820	kanzonols W	Cell division protein kinase 2
Radix Glycyrrhizae (RG)	MOL004820	kanzonols W	Serine/threonine-protein kinase Chk1
Radix Glycyrrhizae (RG)	MOL004820	kanzonols W	Trypsin-1
Radix Glycyrrhizae (RG)	MOL004820	kanzonols W	Proto-oncogene serine/threonine-protein kinase Pim-1
Radix Glycyrrhizae (RG)	MOL004820	kanzonols W	Cyclin-A2
Radix Glycyrrhizae (RG)	MOL004820	kanzonols W	Nuclear receptor coactivator 2
Radix Glycyrrhizae (RG)	MOL004820	kanzonols W	Nuclear receptor coactivator 1
Radix Glycyrrhizae (RG)	MOL004820	kanzonols W	Calmodulin
Radix Glycyrrhizae (RG)	MOL004824	(2S)-6-(2,4-dihydroxyphenyl)-2-(2-hydroxypropan-2-yl)-4-methoxy-2,3-dihydrofuro[3,2-g]chromen-7-one	Nitric oxide synthase, inducible
Radix Glycyrrhizae (RG)	MOL004824	(2S)-6-(2,4-dihydroxyphenyl)-2-(2-hydroxypropan-2-yl)-4-methoxy-2,3-dihydrofuro[3,2-g]chromen-7-one	Thrombin
Radix Glycyrrhizae (RG)	MOL004824	(2S)-6-(2,4-dihydroxyphenyl)-2-(2-hydroxypropan-2-yl)-4-methoxy-2,3-dihydrofuro[3,2-g]chromen-7-one	Estrogen receptor
Radix Glycyrrhizae (RG)	MOL004824	(2S)-6-(2,4-dihydroxyphenyl)-2-(2-hydroxypropan-2-yl)-4-methoxy-2,3-dihydrofuro[3,2-g]chromen-7-one	Androgen receptor
Radix Glycyrrhizae (RG)	MOL004824	(2S)-6-(2,4-dihydroxyphenyl)-2-(2-hydroxypropan-2-yl)-4-methoxy-2,3-dihydrofuro[3,2-g]chromen-7-one	Peroxisome proliferator activated receptor gamma
Radix Glycyrrhizae (RG)	MOL004824	(2S)-6-(2,4-dihydroxyphenyl)-2-(2-hydroxypropan-2-yl)-4-methoxy-2,3-dihydrofuro[3,2-g]chromen-7-one	Coagulation factor Xa
Radix Glycyrrhizae (RG)	MOL004824	(2S)-6-(2,4-dihydroxyphenyl)-2-(2-hydroxypropan-2-yl)-4-methoxy-2,3-dihydrofuro[3,2-g]chromen-7-one	Prostaglandin G/H synthase 2
Radix Glycyrrhizae (RG)	MOL004824	(2S)-6-(2,4-dihydroxyphenyl)-2-(2-hydroxypropan-2-yl)-4-methoxy-2,3-dihydrofuro[3,2-g]chromen-7-one	Coagulation factor VII
Radix Glycyrrhizae (RG)	MOL004824	(2S)-6-(2,4-dihydroxyphenyl)-2-(2-hydroxypropan-2-yl)-4-methoxy-2,3-dihydrofuro[3,2-g]chromen-7-one	Vascular endothelial growth factor receptor 2
Radix Glycyrrhizae (RG)	MOL004824	(2S)-6-(2,4-dihydroxyphenyl)-2-(2-hydroxypropan-2-yl)-4-methoxy-2,3-dihydrofuro[3,2-g]chromen-7-one	Acetylcholinesterase

Radix Glycyrrhizae (RG)	MOL004824	(2S)-6-(2,4-dihydroxyphenyl)-2-(2-hydroxypropan-2-yl)-4-methoxy-2,3-dihydrofuro[3,2-g]chromen-7-one	DNA topoisomerase II
Radix Glycyrrhizae (RG)	MOL004824	(2S)-6-(2,4-dihydroxyphenyl)-2-(2-hydroxypropan-2-yl)-4-methoxy-2,3-dihydrofuro[3,2-g]chromen-7-one	Estrogen receptor beta
Radix Glycyrrhizae (RG)	MOL004824	(2S)-6-(2,4-dihydroxyphenyl)-2-(2-hydroxypropan-2-yl)-4-methoxy-2,3-dihydrofuro[3,2-g]chromen-7-one	Dipeptidyl peptidase IV
Radix Glycyrrhizae (RG)	MOL004824	(2S)-6-(2,4-dihydroxyphenyl)-2-(2-hydroxypropan-2-yl)-4-methoxy-2,3-dihydrofuro[3,2-g]chromen-7-one	Mitogen-activated protein kinase 14
Radix Glycyrrhizae (RG)	MOL004824	(2S)-6-(2,4-dihydroxyphenyl)-2-(2-hydroxypropan-2-yl)-4-methoxy-2,3-dihydrofuro[3,2-g]chromen-7-one	Glycogen synthase kinase-3 beta
Radix Glycyrrhizae (RG)	MOL004824	(2S)-6-(2,4-dihydroxyphenyl)-2-(2-hydroxypropan-2-yl)-4-methoxy-2,3-dihydrofuro[3,2-g]chromen-7-one	Cell division protein kinase 2
Radix Glycyrrhizae (RG)	MOL004824	(2S)-6-(2,4-dihydroxyphenyl)-2-(2-hydroxypropan-2-yl)-4-methoxy-2,3-dihydrofuro[3,2-g]chromen-7-one	Serine/threonine-protein kinase Chk1
Radix Glycyrrhizae (RG)	MOL004824	(2S)-6-(2,4-dihydroxyphenyl)-2-(2-hydroxypropan-2-yl)-4-methoxy-2,3-dihydrofuro[3,2-g]chromen-7-one	Trypsin-1
Radix Glycyrrhizae (RG)	MOL004824	(2S)-6-(2,4-dihydroxyphenyl)-2-(2-hydroxypropan-2-yl)-4-methoxy-2,3-dihydrofuro[3,2-g]chromen-7-one	Proto-oncogene serine/threonine-protein kinase Pim-1
Radix Glycyrrhizae (RG)	MOL004824	(2S)-6-(2,4-dihydroxyphenyl)-2-(2-hydroxypropan-2-yl)-4-methoxy-2,3-dihydrofuro[3,2-g]chromen-7-one	Cyclin-A2
Radix Glycyrrhizae (RG)	MOL004824	(2S)-6-(2,4-dihydroxyphenyl)-2-(2-hydroxypropan-2-yl)-4-methoxy-2,3-dihydrofuro[3,2-g]chromen-7-one	Calmodulin
Radix Glycyrrhizae (RG)	MOL004827	Semilicoisoflavone B	Nitric oxide synthase, inducible
Radix Glycyrrhizae (RG)	MOL004827	Semilicoisoflavone B	Thrombin
Radix Glycyrrhizae (RG)	MOL004827	Semilicoisoflavone B	Estrogen receptor
Radix Glycyrrhizae (RG)	MOL004827	Semilicoisoflavone B	Androgen receptor
Radix Glycyrrhizae (RG)	MOL004827	Semilicoisoflavone B	Sodium channel protein type 5 subunit alpha
Radix Glycyrrhizae (RG)	MOL004827	Semilicoisoflavone B	Peroxisome proliferator activated receptor gamma
Radix Glycyrrhizae (RG)	MOL004827	Semilicoisoflavone B	Coagulation factor Xa
Radix Glycyrrhizae (RG)	MOL004827	Semilicoisoflavone B	Prostaglandin G/H synthase 2
Radix Glycyrrhizae (RG)	MOL004827	Semilicoisoflavone B	Coagulation factor VII
Radix Glycyrrhizae (RG)	MOL004827	Semilicoisoflavone B	Acetylcholinesterase
Radix Glycyrrhizae (RG)	MOL004827	Semilicoisoflavone B	DNA topoisomerase II
Radix Glycyrrhizae (RG)	MOL004827	Semilicoisoflavone B	Glycogen synthase kinase-3 beta
Radix Glycyrrhizae (RG)	MOL004827	Semilicoisoflavone B	Heat shock protein HSP 90
Radix Glycyrrhizae (RG)	MOL004827	Semilicoisoflavone B	Cell division protein kinase 2
Radix Glycyrrhizae (RG)	MOL004827	Semilicoisoflavone B	Serine/threonine-protein kinase Chk1
Radix Glycyrrhizae (RG)	MOL004827	Semilicoisoflavone B	Trypsin-1
Radix Glycyrrhizae (RG)	MOL004827	Semilicoisoflavone B	Calmodulin

Radix Glycyrrhizae (RG)	MOL004828	Glepidotin A	Nitric oxide synthase, inducible
Radix Glycyrrhizae (RG)	MOL004828	Glepidotin A	Prostaglandin G/H synthase 1
Radix Glycyrrhizae (RG)	MOL004828	Glepidotin A	Thrombin
Radix Glycyrrhizae (RG)	MOL004828	Glepidotin A	Estrogen receptor
Radix Glycyrrhizae (RG)	MOL004828	Glepidotin A	Androgen receptor
Radix Glycyrrhizae (RG)	MOL004828	Glepidotin A	Sodium channel protein type 5 subunit alpha
Radix Glycyrrhizae (RG)	MOL004828	Glepidotin A	Peroxisome proliferator activated receptor gamma
Radix Glycyrrhizae (RG)	MOL004828	Glepidotin A	Coagulation factor Xa
Radix Glycyrrhizae (RG)	MOL004828	Glepidotin A	Prostaglandin G/H synthase 2
Radix Glycyrrhizae (RG)	MOL004828	Glepidotin A	Nitric-oxide synthase, endothelial
Radix Glycyrrhizae (RG)	MOL004828	Glepidotin A	Coagulation factor VII
Radix Glycyrrhizae (RG)	MOL004828	Glepidotin A	Vascular endothelial growth factor receptor 2
Radix Glycyrrhizae (RG)	MOL004828	Glepidotin A	Retinoic acid receptor RXR-alpha
Radix Glycyrrhizae (RG)	MOL004828	Glepidotin A	CGMP-inhibited 3',5'-cyclic phosphodiesterase A
Radix Glycyrrhizae (RG)	MOL004828	Glepidotin A	DNA topoisomerase II
Radix Glycyrrhizae (RG)	MOL004828	Glepidotin A	Dipeptidyl peptidase IV
Radix Glycyrrhizae (RG)	MOL004828	Glepidotin A	Mitogen-activated protein kinase 14
Radix Glycyrrhizae (RG)	MOL004828	Glepidotin A	Glycogen synthase kinase-3 beta
Radix Glycyrrhizae (RG)	MOL004828	Glepidotin A	Heat shock protein HSP 90
Radix Glycyrrhizae (RG)	MOL004828	Glepidotin A	Cell division protein kinase 2
Radix Glycyrrhizae (RG)	MOL004828	Glepidotin A	Serine/threonine-protein kinase Chk1
Radix Glycyrrhizae (RG)	MOL004828	Glepidotin A	Ig gamma-1 chain C region
Radix Glycyrrhizae (RG)	MOL004828	Glepidotin A	Trypsin-1
Radix Glycyrrhizae (RG)	MOL004828	Glepidotin A	Proto-oncogene serine/threonine-protein kinase Pim-1
Radix Glycyrrhizae (RG)	MOL004828	Glepidotin A	Cyclin-A2
Radix Glycyrrhizae (RG)	MOL004828	Glepidotin A	Calmodulin
Radix Glycyrrhizae (RG)	MOL004829	Glepidotin B	Prostaglandin G/H synthase 1
Radix Glycyrrhizae (RG)	MOL004829	Glepidotin B	Estrogen receptor
Radix Glycyrrhizae (RG)	MOL004829	Glepidotin B	Sodium channel protein type 5 subunit alpha
Radix Glycyrrhizae (RG)	MOL004829	Glepidotin B	Coagulation factor Xa
Radix Glycyrrhizae (RG)	MOL004829	Glepidotin B	Prostaglandin G/H synthase 2
Radix Glycyrrhizae (RG)	MOL004829	Glepidotin B	Nitric-oxide synthase, endothelial
Radix Glycyrrhizae (RG)	MOL004829	Glepidotin B	Coagulation factor VII
Radix Glycyrrhizae (RG)	MOL004829	Glepidotin B	Retinoic acid receptor RXR-alpha
Radix Glycyrrhizae (RG)	MOL004829	Glepidotin B	CGMP-inhibited 3',5'-cyclic phosphodiesterase A
Radix Glycyrrhizae (RG)	MOL004829	Glepidotin B	Alpha-1B adrenergic receptor
Radix Glycyrrhizae (RG)	MOL004829	Glepidotin B	DNA topoisomerase II
Radix Glycyrrhizae (RG)	MOL004829	Glepidotin B	Heat shock protein HSP 90
Radix Glycyrrhizae (RG)	MOL004829	Glepidotin B	Ig gamma-1 chain C region
Radix Glycyrrhizae (RG)	MOL004829	Glepidotin B	Nuclear receptor coactivator 1
Radix Glycyrrhizae (RG)	MOL004829	Glepidotin B	Calmodulin
Radix Glycyrrhizae (RG)	MOL004833	Phaseolinisoflavan	Nitric oxide synthase, inducible
Radix Glycyrrhizae (RG)	MOL004833	Phaseolinisoflavan	Muscarinic acetylcholine receptor M1
Radix Glycyrrhizae (RG)	MOL004833	Phaseolinisoflavan	Estrogen receptor
Radix Glycyrrhizae (RG)	MOL004833	Phaseolinisoflavan	Androgen receptor
Radix Glycyrrhizae (RG)	MOL004833	Phaseolinisoflavan	Sodium channel protein type 5 subunit alpha
Radix Glycyrrhizae (RG)	MOL004833	Phaseolinisoflavan	Peroxisome proliferator activated receptor gamma
Radix Glycyrrhizae (RG)	MOL004833	Phaseolinisoflavan	Coagulation factor Xa
Radix Glycyrrhizae (RG)	MOL004833	Phaseolinisoflavan	Prostaglandin G/H synthase 2
Radix Glycyrrhizae (RG)	MOL004833	Phaseolinisoflavan	Retinoic acid receptor RXR-alpha
Radix Glycyrrhizae (RG)	MOL004833	Phaseolinisoflavan	Acetylcholinesterase
Radix Glycyrrhizae (RG)	MOL004833	Phaseolinisoflavan	Alpha-1B adrenergic receptor
Radix Glycyrrhizae (RG)	MOL004833	Phaseolinisoflavan	Beta-2 adrenergic receptor
Radix Glycyrrhizae (RG)	MOL004833	Phaseolinisoflavan	Estrogen receptor beta

Radix Glycyrrhizae (RG)	MOL004833	Phaseolinisoflavan	Mitogen-activated protein kinase 14
Radix Glycyrrhizae (RG)	MOL004833	Phaseolinisoflavan	Glycogen synthase kinase-3 beta
Radix Glycyrrhizae (RG)	MOL004833	Phaseolinisoflavan	Cell division protein kinase 2
Radix Glycyrrhizae (RG)	MOL004833	Phaseolinisoflavan	Serine/threonine-protein kinase Chk1
Radix Glycyrrhizae (RG)	MOL004833	Phaseolinisoflavan	Trypsin-1
Radix Glycyrrhizae (RG)	MOL004833	Phaseolinisoflavan	Proto-oncogene serine/threonine-protein kinase Pim-1
Radix Glycyrrhizae (RG)	MOL004833	Phaseolinisoflavan	Cyclin-A2
Radix Glycyrrhizae (RG)	MOL004833	Phaseolinisoflavan	Nuclear receptor coactivator 1
Radix Glycyrrhizae (RG)	MOL004833	Phaseolinisoflavan	Calmodulin
Radix Glycyrrhizae (RG)	MOL004835	Glypallichalcone	Nitric oxide synthase, inducible
Radix Glycyrrhizae (RG)	MOL004835	Glypallichalcone	Prostaglandin G/H synthase 1
Radix Glycyrrhizae (RG)	MOL004835	Glypallichalcone	Muscarinic acetylcholine receptor M1
Radix Glycyrrhizae (RG)	MOL004835	Glypallichalcone	Estrogen receptor
Radix Glycyrrhizae (RG)	MOL004835	Glypallichalcone	Androgen receptor
Radix Glycyrrhizae (RG)	MOL004835	Glypallichalcone	Sodium channel protein type 5 subunit alpha
Radix Glycyrrhizae (RG)	MOL004835	Glypallichalcone	Peroxisome proliferator activated receptor gamma
Radix Glycyrrhizae (RG)	MOL004835	Glypallichalcone	Prostaglandin G/H synthase 2
Radix Glycyrrhizae (RG)	MOL004835	Glypallichalcone	Carbonic anhydrase II
Radix Glycyrrhizae (RG)	MOL004835	Glypallichalcone	CGMP-inhibited 3',5'-cyclic phosphodiesterase A
Radix Glycyrrhizae (RG)	MOL004835	Glypallichalcone	Alpha-1B adrenergic receptor
Radix Glycyrrhizae (RG)	MOL004835	Glypallichalcone	Sodium-dependent dopamine
Radix Glycyrrhizae (RG)	MOL004835	Glypallichalcone	Beta-2 adrenergic receptor
Radix Glycyrrhizae (RG)	MOL004835	Glypallichalcone	Sodium-dependent serotonin transporter
Radix Glycyrrhizae (RG)	MOL004835	Glypallichalcone	Estrogen receptor beta
Radix Glycyrrhizae (RG)	MOL004835	Glypallichalcone	Mitogen-activated protein kinase 14
Radix Glycyrrhizae (RG)	MOL004835	Glypallichalcone	Glycogen synthase kinase-3 beta
Radix Glycyrrhizae (RG)	MOL004835	Glypallichalcone	Heat shock protein HSP 90
Radix Glycyrrhizae (RG)	MOL004835	Glypallichalcone	Cell division protein kinase 2
Radix Glycyrrhizae (RG)	MOL004835	Glypallichalcone	Leukotriene A-4 hydrolase
Radix Glycyrrhizae (RG)	MOL004835	Glypallichalcone	Amine oxidase [flavin-containing] B
Radix Glycyrrhizae (RG)	MOL004835	Glypallichalcone	Serine/threonine-protein kinase Chk1
Radix Glycyrrhizae (RG)	MOL004835	Glypallichalcone	mRNA of PKA Catalytic Subunit C-
Radix Glycyrrhizae (RG)	MOL004835	Glypallichalcone	Cyclin-A2
Radix Glycyrrhizae (RG)	MOL004835	Glypallichalcone	Nuclear receptor coactivator 1
Radix Glycyrrhizae (RG)	MOL004835	Glypallichalcone	cAMP-dependent protein kinase inhibitor alpha
Radix Glycyrrhizae (RG)	MOL004835	Glypallichalcone	Calmodulin
Radix Glycyrrhizae (RG)	MOL004838	8-(6-hydroxy-2-benzofuranyl)-2,2-dimethyl-5-chromenol	Nitric oxide synthase, inducible
Radix Glycyrrhizae (RG)	MOL004838	8-(6-hydroxy-2-benzofuranyl)-2,2-dimethyl-5-chromenol	Estrogen receptor
Radix Glycyrrhizae (RG)	MOL004838	8-(6-hydroxy-2-benzofuranyl)-2,2-dimethyl-5-chromenol	Prostaglandin G/H synthase 2
Radix Glycyrrhizae (RG)	MOL004838	8-(6-hydroxy-2-benzofuranyl)-2,2-dimethyl-5-chromenol	Retinoic acid receptor RXR-alpha
Radix Glycyrrhizae (RG)	MOL004838	8-(6-hydroxy-2-benzofuranyl)-2,2-dimethyl-5-chromenol	Heat shock protein HSP 90
Radix Glycyrrhizae (RG)	MOL004838	8-(6-hydroxy-2-benzofuranyl)-2,2-dimethyl-5-chromenol	Phosphatidylinositol-4,5-bisphosphate 3-kinase catalytic subunit, gamma
Radix Glycyrrhizae (RG)	MOL004841	Licochalcone B	Nitric oxide synthase, inducible
Radix Glycyrrhizae (RG)	MOL004841	Licochalcone B	Prostaglandin G/H synthase 1
Radix Glycyrrhizae (RG)	MOL004841	Licochalcone B	Estrogen receptor
Radix Glycyrrhizae (RG)	MOL004841	Licochalcone B	Androgen receptor
Radix Glycyrrhizae (RG)	MOL004841	Licochalcone B	Peroxisome proliferator activated receptor gamma
Radix Glycyrrhizae (RG)	MOL004841	Licochalcone B	Prostaglandin G/H synthase 2
Radix Glycyrrhizae (RG)	MOL004841	Licochalcone B	Carbonic anhydrase II
Radix Glycyrrhizae (RG)	MOL004841	Licochalcone B	CGMP-inhibited 3',5'-cyclic phosphodiesterase A

Radix Glycyrrhizae (RG)	MOL004841	Licochalcone B	Beta-2 adrenergic receptor
Radix Glycyrrhizae (RG)	MOL004841	Licochalcone B	Estrogen receptor beta
Radix Glycyrrhizae (RG)	MOL004841	Licochalcone B	Mitogen-activated protein kinase 14
Radix Glycyrrhizae (RG)	MOL004841	Licochalcone B	Glycogen synthase kinase-3 beta
Radix Glycyrrhizae (RG)	MOL004841	Licochalcone B	Heat shock protein HSP 90
Radix Glycyrrhizae (RG)	MOL004841	Licochalcone B	Cell division protein kinase 2
Radix Glycyrrhizae (RG)	MOL004841	Licochalcone B	Serine/threonine-protein kinase Chk1
Radix Glycyrrhizae (RG)	MOL004841	Licochalcone B	mRNA of PKA Catalytic Subunit C-
Radix Glycyrrhizae (RG)	MOL004841	Licochalcone B	Proto-oncogene serine/threonine-protein kinase Pim-1
Radix Glycyrrhizae (RG)	MOL004841	Licochalcone B	Cyclin-A2
Radix Glycyrrhizae (RG)	MOL004841	Licochalcone B	Calmodulin
Radix Glycyrrhizae (RG)	MOL004848	licochalcone G	Nitric oxide synthase, inducible
Radix Glycyrrhizae (RG)	MOL004848	licochalcone G	Estrogen receptor
Radix Glycyrrhizae (RG)	MOL004848	licochalcone G	Androgen receptor
Radix Glycyrrhizae (RG)	MOL004848	licochalcone G	Peroxisome proliferator activated receptor gamma
Radix Glycyrrhizae (RG)	MOL004848	licochalcone G	Coagulation factor Xa
Radix Glycyrrhizae (RG)	MOL004848	licochalcone G	Prostaglandin G/H synthase 2
Radix Glycyrrhizae (RG)	MOL004848	licochalcone G	Vascular endothelial growth factor receptor 2
Radix Glycyrrhizae (RG)	MOL004848	licochalcone G	Estrogen receptor beta
Radix Glycyrrhizae (RG)	MOL004848	licochalcone G	Mitogen-activated protein kinase 14
Radix Glycyrrhizae (RG)	MOL004848	licochalcone G	Glycogen synthase kinase-3 beta
Radix Glycyrrhizae (RG)	MOL004848	licochalcone G	Heat shock protein HSP 90
Radix Glycyrrhizae (RG)	MOL004848	licochalcone G	Cell division protein kinase 2
Radix Glycyrrhizae (RG)	MOL004848	licochalcone G	Ig gamma-1 chain C region
Radix Glycyrrhizae (RG)	MOL004848	licochalcone G	Proto-oncogene serine/threonine-protein kinase Pim-1
Radix Glycyrrhizae (RG)	MOL004848	licochalcone G	Cyclin-A2
Radix Glycyrrhizae (RG)	MOL004848	licochalcone G	Nuclear receptor coactivator 2
Radix Glycyrrhizae (RG)	MOL004848	licochalcone G	Calmodulin
Radix Glycyrrhizae (RG)	MOL004849	3-(2,4-dihydroxyphenyl)-8-(1,1-dimethylprop-2-enyl)-7-hydroxy-5-methoxy-coumarin	Nitric oxide synthase, inducible
Radix Glycyrrhizae (RG)	MOL004849	3-(2,4-dihydroxyphenyl)-8-(1,1-dimethylprop-2-enyl)-7-hydroxy-5-methoxy-coumarin	Thrombin
Radix Glycyrrhizae (RG)	MOL004849	3-(2,4-dihydroxyphenyl)-8-(1,1-dimethylprop-2-enyl)-7-hydroxy-5-methoxy-coumarin	Potassium voltage-gated channel subfamily H member 2
Radix Glycyrrhizae (RG)	MOL004849	3-(2,4-dihydroxyphenyl)-8-(1,1-dimethylprop-2-enyl)-7-hydroxy-5-methoxy-coumarin	Estrogen receptor
Radix Glycyrrhizae (RG)	MOL004849	3-(2,4-dihydroxyphenyl)-8-(1,1-dimethylprop-2-enyl)-7-hydroxy-5-methoxy-coumarin	Androgen receptor
Radix Glycyrrhizae (RG)	MOL004849	3-(2,4-dihydroxyphenyl)-8-(1,1-dimethylprop-2-enyl)-7-hydroxy-5-methoxy-coumarin	Peroxisome proliferator activated receptor gamma
Radix Glycyrrhizae (RG)	MOL004849	3-(2,4-dihydroxyphenyl)-8-(1,1-dimethylprop-2-enyl)-7-hydroxy-5-methoxy-coumarin	Coagulation factor Xa
Radix Glycyrrhizae (RG)	MOL004849	3-(2,4-dihydroxyphenyl)-8-(1,1-dimethylprop-2-enyl)-7-hydroxy-5-methoxy-coumarin	Prostaglandin G/H synthase 2
Radix Glycyrrhizae (RG)	MOL004849	3-(2,4-dihydroxyphenyl)-8-(1,1-dimethylprop-2-enyl)-7-hydroxy-5-methoxy-coumarin	Coagulation factor VII
Radix Glycyrrhizae (RG)	MOL004849	3-(2,4-dihydroxyphenyl)-8-(1,1-dimethylprop-2-enyl)-7-hydroxy-5-methoxy-coumarin	Vascular endothelial growth factor receptor 2

Radix Glycyrrhizae (RG)	MOL004849	3-(2,4-dihydroxyphenyl)-8-(1,1-dimethylprop-2-enyl)-7-hydroxy-5-methoxy-coumarin	DNA topoisomerase II
Radix Glycyrrhizae (RG)	MOL004849	3-(2,4-dihydroxyphenyl)-8-(1,1-dimethylprop-2-enyl)-7-hydroxy-5-methoxy-coumarin	Estrogen receptor beta
Radix Glycyrrhizae (RG)	MOL004849	3-(2,4-dihydroxyphenyl)-8-(1,1-dimethylprop-2-enyl)-7-hydroxy-5-methoxy-coumarin	Dipeptidyl peptidase IV
Radix Glycyrrhizae (RG)	MOL004849	3-(2,4-dihydroxyphenyl)-8-(1,1-dimethylprop-2-enyl)-7-hydroxy-5-methoxy-coumarin	Mitogen-activated protein kinase 14
Radix Glycyrrhizae (RG)	MOL004849	3-(2,4-dihydroxyphenyl)-8-(1,1-dimethylprop-2-enyl)-7-hydroxy-5-methoxy-coumarin	Glycogen synthase kinase-3 beta
Radix Glycyrrhizae (RG)	MOL004849	3-(2,4-dihydroxyphenyl)-8-(1,1-dimethylprop-2-enyl)-7-hydroxy-5-methoxy-coumarin	Heat shock protein HSP 90
Radix Glycyrrhizae (RG)	MOL004849	3-(2,4-dihydroxyphenyl)-8-(1,1-dimethylprop-2-enyl)-7-hydroxy-5-methoxy-coumarin	Cell division protein kinase 2
Radix Glycyrrhizae (RG)	MOL004849	3-(2,4-dihydroxyphenyl)-8-(1,1-dimethylprop-2-enyl)-7-hydroxy-5-methoxy-coumarin	Serine/threonine-protein kinase Chk1
Radix Glycyrrhizae (RG)	MOL004849	3-(2,4-dihydroxyphenyl)-8-(1,1-dimethylprop-2-enyl)-7-hydroxy-5-methoxy-coumarin	Trypsin-1
Radix Glycyrrhizae (RG)	MOL004849	3-(2,4-dihydroxyphenyl)-8-(1,1-dimethylprop-2-enyl)-7-hydroxy-5-methoxy-coumarin	Proto-oncogene serine/threonine-protein kinase Pim-1
Radix Glycyrrhizae (RG)	MOL004849	3-(2,4-dihydroxyphenyl)-8-(1,1-dimethylprop-2-enyl)-7-hydroxy-5-methoxy-coumarin	Nuclear receptor coactivator 2
Radix Glycyrrhizae (RG)	MOL004849	3-(2,4-dihydroxyphenyl)-8-(1,1-dimethylprop-2-enyl)-7-hydroxy-5-methoxy-coumarin	Nuclear receptor coactivator 1
Radix Glycyrrhizae (RG)	MOL004849	3-(2,4-dihydroxyphenyl)-8-(1,1-dimethylprop-2-enyl)-7-hydroxy-5-methoxy-coumarin	Calmodulin
Radix Glycyrrhizae (RG)	MOL004855	Licoricone	Nitric oxide synthase, inducible
Radix Glycyrrhizae (RG)	MOL004855	Licoricone	Thrombin
Radix Glycyrrhizae (RG)	MOL004855	Licoricone	Potassium voltage-gated channel subfamily H member 2
Radix Glycyrrhizae (RG)	MOL004855	Licoricone	Estrogen receptor
Radix Glycyrrhizae (RG)	MOL004855	Licoricone	Androgen receptor
Radix Glycyrrhizae (RG)	MOL004855	Licoricone	Peroxisome proliferator activated receptor gamma
Radix Glycyrrhizae (RG)	MOL004855	Licoricone	Coagulation factor Xa
Radix Glycyrrhizae (RG)	MOL004855	Licoricone	Prostaglandin G/H synthase 2
Radix Glycyrrhizae (RG)	MOL004855	Licoricone	Vascular endothelial growth factor receptor 2
Radix Glycyrrhizae (RG)	MOL004855	Licoricone	DNA topoisomerase II
Radix Glycyrrhizae (RG)	MOL004855	Licoricone	Serine/threonine-protein kinase Chk1
Radix Glycyrrhizae (RG)	MOL004855	Licoricone	Trypsin-1
Radix Glycyrrhizae (RG)	MOL004855	Licoricone	Proto-oncogene serine/threonine-protein kinase Pim-1
Radix Glycyrrhizae (RG)	MOL004855	Licoricone	Nuclear receptor coactivator 2
Radix Glycyrrhizae (RG)	MOL004855	Licoricone	Calmodulin
Radix Glycyrrhizae (RG)	MOL004856	Gancaonin A	Nitric oxide synthase, inducible
Radix Glycyrrhizae (RG)	MOL004856	Gancaonin A	Thrombin
Radix Glycyrrhizae (RG)	MOL004856	Gancaonin A	Estrogen receptor
Radix Glycyrrhizae (RG)	MOL004856	Gancaonin A	Androgen receptor

Radix Glycyrrhizae (RG)	MOL004856	Gancaonin A	Sodium channel protein type 5 subunit alpha
Radix Glycyrrhizae (RG)	MOL004856	Gancaonin A	Peroxisome proliferator activated receptor gamma
Radix Glycyrrhizae (RG)	MOL004856	Gancaonin A	Coagulation factor Xa
Radix Glycyrrhizae (RG)	MOL004856	Gancaonin A	Prostaglandin G/H synthase 2
Radix Glycyrrhizae (RG)	MOL004856	Gancaonin A	Acetylcholinesterase
Radix Glycyrrhizae (RG)	MOL004856	Gancaonin A	DNA topoisomerase II
Radix Glycyrrhizae (RG)	MOL004856	Gancaonin A	Estrogen receptor beta
Radix Glycyrrhizae (RG)	MOL004856	Gancaonin A	Dipeptidyl peptidase IV
Radix Glycyrrhizae (RG)	MOL004856	Gancaonin A	Glycogen synthase kinase-3 beta
Radix Glycyrrhizae (RG)	MOL004856	Gancaonin A	Heat shock protein HSP 90
Radix Glycyrrhizae (RG)	MOL004856	Gancaonin A	Serine/threonine-protein kinase Chk1
Radix Glycyrrhizae (RG)	MOL004856	Gancaonin A	Trypsin-1
Radix Glycyrrhizae (RG)	MOL004856	Gancaonin A	Proto-oncogene serine/threonine-protein kinase Pim-1
Radix Glycyrrhizae (RG)	MOL004856	Gancaonin A	Cyclin-A2
Radix Glycyrrhizae (RG)	MOL004856	Gancaonin A	Nuclear receptor coactivator 2
Radix Glycyrrhizae (RG)	MOL004856	Gancaonin A	Calmodulin
Radix Glycyrrhizae (RG)	MOL004857	Gancaonin B	Nitric oxide synthase, inducible
Radix Glycyrrhizae (RG)	MOL004857	Gancaonin B	Thrombin
Radix Glycyrrhizae (RG)	MOL004857	Gancaonin B	Estrogen receptor
Radix Glycyrrhizae (RG)	MOL004857	Gancaonin B	Androgen receptor
Radix Glycyrrhizae (RG)	MOL004857	Gancaonin B	Peroxisome proliferator activated receptor gamma
Radix Glycyrrhizae (RG)	MOL004857	Gancaonin B	Coagulation factor Xa
Radix Glycyrrhizae (RG)	MOL004857	Gancaonin B	Prostaglandin G/H synthase 2
Radix Glycyrrhizae (RG)	MOL004857	Gancaonin B	Coagulation factor VII
Radix Glycyrrhizae (RG)	MOL004857	Gancaonin B	Vascular endothelial growth factor receptor 2
Radix Glycyrrhizae (RG)	MOL004857	Gancaonin B	Alpha-1B adrenergic receptor
Radix Glycyrrhizae (RG)	MOL004857	Gancaonin B	Beta-2 adrenergic receptor
Radix Glycyrrhizae (RG)	MOL004857	Gancaonin B	DNA topoisomerase II
Radix Glycyrrhizae (RG)	MOL004857	Gancaonin B	Estrogen receptor beta
Radix Glycyrrhizae (RG)	MOL004857	Gancaonin B	Dipeptidyl peptidase IV
Radix Glycyrrhizae (RG)	MOL004857	Gancaonin B	Glycogen synthase kinase-3 beta
Radix Glycyrrhizae (RG)	MOL004857	Gancaonin B	Heat shock protein HSP 90
Radix Glycyrrhizae (RG)	MOL004857	Gancaonin B	Serine/threonine-protein kinase Chk1
Radix Glycyrrhizae (RG)	MOL004857	Gancaonin B	Trypsin-1
Radix Glycyrrhizae (RG)	MOL004857	Gancaonin B	Proto-oncogene serine/threonine-protein kinase Pim-1
Radix Glycyrrhizae (RG)	MOL004857	Gancaonin B	Cyclin-A2
Radix Glycyrrhizae (RG)	MOL004857	Gancaonin B	Nuclear receptor coactivator 2
Radix Glycyrrhizae (RG)	MOL004857	Gancaonin B	Calmodulin
Radix Glycyrrhizae (RG)	MOL004863	3-(3,4-dihydroxyphenyl)-5,7-dihydroxy-8-(3-methylbut-2-enyl)chromone	Nitric oxide synthase, inducible
Radix Glycyrrhizae (RG)	MOL004863	3-(3,4-dihydroxyphenyl)-5,7-dihydroxy-8-(3-methylbut-2-enyl)chromone	Thrombin
Radix Glycyrrhizae (RG)	MOL004863	3-(3,4-dihydroxyphenyl)-5,7-dihydroxy-8-(3-methylbut-2-enyl)chromone	Estrogen receptor
Radix Glycyrrhizae (RG)	MOL004863	3-(3,4-dihydroxyphenyl)-5,7-dihydroxy-8-(3-methylbut-2-enyl)chromone	Androgen receptor
Radix Glycyrrhizae (RG)	MOL004863	3-(3,4-dihydroxyphenyl)-5,7-dihydroxy-8-(3-methylbut-2-enyl)chromone	Peroxisome proliferator activated receptor gamma
Radix Glycyrrhizae (RG)	MOL004863	3-(3,4-dihydroxyphenyl)-5,7-dihydroxy-8-(3-methylbut-2-enyl)chromone	Coagulation factor Xa

Radix Glycyrrhizae (RG)	MOL004863	3-(3,4-dihydroxyphenyl)-5,7-dihydroxy-8-(3-methylbut-2-enyl)chromone	Prostaglandin G/H synthase 2
Radix Glycyrrhizae (RG)	MOL004863	3-(3,4-dihydroxyphenyl)-5,7-dihydroxy-8-(3-methylbut-2-enyl)chromone	mRNA of Protein-tyrosine phosphatase, non-receptor type 1
Radix Glycyrrhizae (RG)	MOL004863	3-(3,4-dihydroxyphenyl)-5,7-dihydroxy-8-(3-methylbut-2-enyl)chromone	Mitogen-activated protein kinase 14
Radix Glycyrrhizae (RG)	MOL004863	3-(3,4-dihydroxyphenyl)-5,7-dihydroxy-8-(3-methylbut-2-enyl)chromone	Glycogen synthase kinase-3 beta
Radix Glycyrrhizae (RG)	MOL004863	3-(3,4-dihydroxyphenyl)-5,7-dihydroxy-8-(3-methylbut-2-enyl)chromone	Heat shock protein HSP 90
Radix Glycyrrhizae (RG)	MOL004863	3-(3,4-dihydroxyphenyl)-5,7-dihydroxy-8-(3-methylbut-2-enyl)chromone	Cell division protein kinase 2
Radix Glycyrrhizae (RG)	MOL004863	3-(3,4-dihydroxyphenyl)-5,7-dihydroxy-8-(3-methylbut-2-enyl)chromone	Serine/threonine-protein kinase Chk1
Radix Glycyrrhizae (RG)	MOL004863	3-(3,4-dihydroxyphenyl)-5,7-dihydroxy-8-(3-methylbut-2-enyl)chromone	Trypsin-1
Radix Glycyrrhizae (RG)	MOL004863	3-(3,4-dihydroxyphenyl)-5,7-dihydroxy-8-(3-methylbut-2-enyl)chromone	Proto-oncogene serine/threonine-protein kinase Pim-1
Radix Glycyrrhizae (RG)	MOL004863	3-(3,4-dihydroxyphenyl)-5,7-dihydroxy-8-(3-methylbut-2-enyl)chromone	Cyclin-A2
Radix Glycyrrhizae (RG)	MOL004863	3-(3,4-dihydroxyphenyl)-5,7-dihydroxy-8-(3-methylbut-2-enyl)chromone	Nuclear receptor coactivator 2
Radix Glycyrrhizae (RG)	MOL004863	3-(3,4-dihydroxyphenyl)-5,7-dihydroxy-8-(3-methylbut-2-enyl)chromone	Calmodulin
Radix Glycyrrhizae (RG)	MOL004864	5,7-dihydroxy-3-(4-methoxyphenyl)-8-(3-methylbut-2-enyl)chromone	Nitric oxide synthase, inducible
Radix Glycyrrhizae (RG)	MOL004864	5,7-dihydroxy-3-(4-methoxyphenyl)-8-(3-methylbut-2-enyl)chromone	Potassium voltage-gated channel subfamily H member 2
Radix Glycyrrhizae (RG)	MOL004864	5,7-dihydroxy-3-(4-methoxyphenyl)-8-(3-methylbut-2-enyl)chromone	Estrogen receptor
Radix Glycyrrhizae (RG)	MOL004864	5,7-dihydroxy-3-(4-methoxyphenyl)-8-(3-methylbut-2-enyl)chromone	Androgen receptor
Radix Glycyrrhizae (RG)	MOL004864	5,7-dihydroxy-3-(4-methoxyphenyl)-8-(3-methylbut-2-enyl)chromone	Peroxisome proliferator activated receptor gamma
Radix Glycyrrhizae (RG)	MOL004864	5,7-dihydroxy-3-(4-methoxyphenyl)-8-(3-methylbut-2-enyl)chromone	Coagulation factor Xa
Radix Glycyrrhizae (RG)	MOL004864	5,7-dihydroxy-3-(4-methoxyphenyl)-8-(3-methylbut-2-enyl)chromone	Prostaglandin G/H synthase 2
Radix Glycyrrhizae (RG)	MOL004864	5,7-dihydroxy-3-(4-methoxyphenyl)-8-(3-methylbut-2-enyl)chromone	DNA topoisomerase II
Radix Glycyrrhizae (RG)	MOL004864	5,7-dihydroxy-3-(4-methoxyphenyl)-8-(3-methylbut-2-enyl)chromone	Estrogen receptor beta

Radix Glycyrrhizae (RG)	MOL004864	5,7-dihydroxy-3-(4-methoxyphenyl)-8-(3-methylbut-2-enyl)chromone	Dipeptidyl peptidase IV
Radix Glycyrrhizae (RG)	MOL004864	5,7-dihydroxy-3-(4-methoxyphenyl)-8-(3-methylbut-2-enyl)chromone	Mitogen-activated protein kinase 14
Radix Glycyrrhizae (RG)	MOL004864	5,7-dihydroxy-3-(4-methoxyphenyl)-8-(3-methylbut-2-enyl)chromone	Glycogen synthase kinase-3 beta
Radix Glycyrrhizae (RG)	MOL004864	5,7-dihydroxy-3-(4-methoxyphenyl)-8-(3-methylbut-2-enyl)chromone	Heat shock protein HSP 90
Radix Glycyrrhizae (RG)	MOL004864	5,7-dihydroxy-3-(4-methoxyphenyl)-8-(3-methylbut-2-enyl)chromone	Cell division protein kinase 2
Radix Glycyrrhizae (RG)	MOL004864	5,7-dihydroxy-3-(4-methoxyphenyl)-8-(3-methylbut-2-enyl)chromone	Serine/threonine-protein kinase Chk1
Radix Glycyrrhizae (RG)	MOL004864	5,7-dihydroxy-3-(4-methoxyphenyl)-8-(3-methylbut-2-enyl)chromone	Trypsin-1
Radix Glycyrrhizae (RG)	MOL004864	5,7-dihydroxy-3-(4-methoxyphenyl)-8-(3-methylbut-2-enyl)chromone	Proto-oncogene serine/threonine-protein kinase Pim-1
Radix Glycyrrhizae (RG)	MOL004864	5,7-dihydroxy-3-(4-methoxyphenyl)-8-(3-methylbut-2-enyl)chromone	Cyclin-A2
Radix Glycyrrhizae (RG)	MOL004864	5,7-dihydroxy-3-(4-methoxyphenyl)-8-(3-methylbut-2-enyl)chromone	Nuclear receptor coactivator 2
Radix Glycyrrhizae (RG)	MOL004864	5,7-dihydroxy-3-(4-methoxyphenyl)-8-(3-methylbut-2-enyl)chromone	Calmodulin
Radix Glycyrrhizae (RG)	MOL004866	2-(3,4-dihydroxyphenyl)-5,7-dihydroxy-6-(3-methylbut-2-enyl)chromone	Thrombin
Radix Glycyrrhizae (RG)	MOL004866	2-(3,4-dihydroxyphenyl)-5,7-dihydroxy-6-(3-methylbut-2-enyl)chromone	Androgen receptor
Radix Glycyrrhizae (RG)	MOL004866	2-(3,4-dihydroxyphenyl)-5,7-dihydroxy-6-(3-methylbut-2-enyl)chromone	Sodium channel protein type 5 subunit alpha
Radix Glycyrrhizae (RG)	MOL004866	2-(3,4-dihydroxyphenyl)-5,7-dihydroxy-6-(3-methylbut-2-enyl)chromone	Peroxisome proliferator activated receptor gamma
Radix Glycyrrhizae (RG)	MOL004866	2-(3,4-dihydroxyphenyl)-5,7-dihydroxy-6-(3-methylbut-2-enyl)chromone	Coagulation factor Xa
Radix Glycyrrhizae (RG)	MOL004866	2-(3,4-dihydroxyphenyl)-5,7-dihydroxy-6-(3-methylbut-2-enyl)chromone	Prostaglandin G/H synthase 2
Radix Glycyrrhizae (RG)	MOL004866	2-(3,4-dihydroxyphenyl)-5,7-dihydroxy-6-(3-methylbut-2-enyl)chromone	Coagulation factor VII
Radix Glycyrrhizae (RG)	MOL004866	2-(3,4-dihydroxyphenyl)-5,7-dihydroxy-6-(3-methylbut-2-enyl)chromone	Beta-2 adrenergic receptor
Radix Glycyrrhizae (RG)	MOL004866	2-(3,4-dihydroxyphenyl)-5,7-dihydroxy-6-(3-methylbut-2-enyl)chromone	Dipeptidyl peptidase IV
Radix Glycyrrhizae (RG)	MOL004866	2-(3,4-dihydroxyphenyl)-5,7-dihydroxy-6-(3-methylbut-2-enyl)chromone	Heat shock protein HSP 90

Radix Glycyrrhizae (RG)	MOL004866	2-(3,4-dihydroxyphenyl)-5,7-dihydroxy-6-(3-methylbut-2-enyl)chromone	Cell division protein kinase 2
Radix Glycyrrhizae (RG)	MOL004866	2-(3,4-dihydroxyphenyl)-5,7-dihydroxy-6-(3-methylbut-2-enyl)chromone	Serine/threonine-protein kinase Chk1
Radix Glycyrrhizae (RG)	MOL004866	2-(3,4-dihydroxyphenyl)-5,7-dihydroxy-6-(3-methylbut-2-enyl)chromone	Trypsin-1
Radix Glycyrrhizae (RG)	MOL004866	2-(3,4-dihydroxyphenyl)-5,7-dihydroxy-6-(3-methylbut-2-enyl)chromone	Proto-oncogene serine/threonine-protein kinase Pim-1
Radix Glycyrrhizae (RG)	MOL004866	2-(3,4-dihydroxyphenyl)-5,7-dihydroxy-6-(3-methylbut-2-enyl)chromone	Cyclin-A2
Radix Glycyrrhizae (RG)	MOL004866	2-(3,4-dihydroxyphenyl)-5,7-dihydroxy-6-(3-methylbut-2-enyl)chromone	Calmodulin
Radix Glycyrrhizae (RG)	MOL004879	Glycyrin	Nitric oxide synthase, inducible
Radix Glycyrrhizae (RG)	MOL004879	Glycyrin	Thrombin
Radix Glycyrrhizae (RG)	MOL004879	Glycyrin	Potassium voltage-gated channel subfamily H member 2
Radix Glycyrrhizae (RG)	MOL004879	Glycyrin	Estrogen receptor
Radix Glycyrrhizae (RG)	MOL004879	Glycyrin	Androgen receptor
Radix Glycyrrhizae (RG)	MOL004879	Glycyrin	Peroxisome proliferator activated receptor gamma
Radix Glycyrrhizae (RG)	MOL004879	Glycyrin	Coagulation factor Xa
Radix Glycyrrhizae (RG)	MOL004879	Glycyrin	Prostaglandin G/H synthase 2
Radix Glycyrrhizae (RG)	MOL004879	Glycyrin	Vascular endothelial growth factor receptor 2
Radix Glycyrrhizae (RG)	MOL004879	Glycyrin	DNA topoisomerase II
Radix Glycyrrhizae (RG)	MOL004879	Glycyrin	Estrogen receptor beta
Radix Glycyrrhizae (RG)	MOL004879	Glycyrin	Dipeptidyl peptidase IV
Radix Glycyrrhizae (RG)	MOL004879	Glycyrin	Serine/threonine-protein kinase Chk1
Radix Glycyrrhizae (RG)	MOL004879	Glycyrin	Trypsin-1
Radix Glycyrrhizae (RG)	MOL004879	Glycyrin	Proto-oncogene serine/threonine-protein kinase Pim-1
Radix Glycyrrhizae (RG)	MOL004879	Glycyrin	Nuclear receptor coactivator 2
Radix Glycyrrhizae (RG)	MOL004879	Glycyrin	Calmodulin
Radix Glycyrrhizae (RG)	MOL004882	Licocoumarone	Estrogen receptor
Radix Glycyrrhizae (RG)	MOL004882	Licocoumarone	Androgen receptor
Radix Glycyrrhizae (RG)	MOL004882	Licocoumarone	Estrogen receptor beta
Radix Glycyrrhizae (RG)	MOL004882	Licocoumarone	Glycogen synthase kinase-3 beta
Radix Glycyrrhizae (RG)	MOL004882	Licocoumarone	Heat shock protein HSP 90
Radix Glycyrrhizae (RG)	MOL004882	Licocoumarone	Cell division protein kinase 2
Radix Glycyrrhizae (RG)	MOL004882	Licocoumarone	Cyclin-A2
Radix Glycyrrhizae (RG)	MOL004883	Licoisoflavone	Nitric oxide synthase, inducible
Radix Glycyrrhizae (RG)	MOL004883	Licoisoflavone	Thrombin
Radix Glycyrrhizae (RG)	MOL004883	Licoisoflavone	Estrogen receptor
Radix Glycyrrhizae (RG)	MOL004883	Licoisoflavone	Androgen receptor
Radix Glycyrrhizae (RG)	MOL004883	Licoisoflavone	Peroxisome proliferator activated receptor gamma
Radix Glycyrrhizae (RG)	MOL004883	Licoisoflavone	Coagulation factor Xa
Radix Glycyrrhizae (RG)	MOL004883	Licoisoflavone	Prostaglandin G/H synthase 2
Radix Glycyrrhizae (RG)	MOL004883	Licoisoflavone	Vascular endothelial growth factor receptor 2
Radix Glycyrrhizae (RG)	MOL004883	Licoisoflavone	DNA topoisomerase II
Radix Glycyrrhizae (RG)	MOL004883	Licoisoflavone	Dipeptidyl peptidase IV
Radix Glycyrrhizae (RG)	MOL004883	Licoisoflavone	Mitogen-activated protein kinase 14
Radix Glycyrrhizae (RG)	MOL004883	Licoisoflavone	Heat shock protein HSP 90
Radix Glycyrrhizae (RG)	MOL004883	Licoisoflavone	Cell division protein kinase 2
Radix Glycyrrhizae (RG)	MOL004883	Licoisoflavone	Serine/threonine-protein kinase Chk1
Radix Glycyrrhizae (RG)	MOL004883	Licoisoflavone	Trypsin-1

Radix Glycyrrhizae (RG)	MOL004883	Licoisoflavone	Proto-oncogene serine/threonine-protein kinase Pim-1
Radix Glycyrrhizae (RG)	MOL004883	Licoisoflavone	Cyclin-A2
Radix Glycyrrhizae (RG)	MOL004883	Licoisoflavone	Nuclear receptor coactivator 2
Radix Glycyrrhizae (RG)	MOL004883	Licoisoflavone	Calmodulin
Radix Glycyrrhizae (RG)	MOL004884	Licoisoflavone B	Nitric oxide synthase, inducible
Radix Glycyrrhizae (RG)	MOL004884	Licoisoflavone B	Thrombin
Radix Glycyrrhizae (RG)	MOL004884	Licoisoflavone B	Estrogen receptor
Radix Glycyrrhizae (RG)	MOL004884	Licoisoflavone B	Androgen receptor
Radix Glycyrrhizae (RG)	MOL004884	Licoisoflavone B	Peroxisome proliferator activated receptor gamma
Radix Glycyrrhizae (RG)	MOL004884	Licoisoflavone B	Coagulation factor Xa
Radix Glycyrrhizae (RG)	MOL004884	Licoisoflavone B	Prostaglandin G/H synthase 2
Radix Glycyrrhizae (RG)	MOL004884	Licoisoflavone B	Acetylcholinesterase
Radix Glycyrrhizae (RG)	MOL004884	Licoisoflavone B	DNA topoisomerase II
Radix Glycyrrhizae (RG)	MOL004884	Licoisoflavone B	Estrogen receptor beta
Radix Glycyrrhizae (RG)	MOL004884	Licoisoflavone B	Glycogen synthase kinase-3 beta
Radix Glycyrrhizae (RG)	MOL004884	Licoisoflavone B	Cell division protein kinase 2
Radix Glycyrrhizae (RG)	MOL004884	Licoisoflavone B	Serine/threonine-protein kinase Chk1
Radix Glycyrrhizae (RG)	MOL004884	Licoisoflavone B	Trypsin-1
Radix Glycyrrhizae (RG)	MOL004884	Licoisoflavone B	Proto-oncogene serine/threonine-protein kinase Pim-1
Radix Glycyrrhizae (RG)	MOL004884	Licoisoflavone B	Cyclin-A2
Radix Glycyrrhizae (RG)	MOL004884	Licoisoflavone B	Calmodulin
Radix Glycyrrhizae (RG)	MOL004885	licoisoflavanone	Nitric oxide synthase, inducible
Radix Glycyrrhizae (RG)	MOL004885	licoisoflavanone	Prostaglandin G/H synthase 1
Radix Glycyrrhizae (RG)	MOL004885	licoisoflavanone	Estrogen receptor
Radix Glycyrrhizae (RG)	MOL004885	licoisoflavanone	Androgen receptor
Radix Glycyrrhizae (RG)	MOL004885	licoisoflavanone	Sodium channel protein type 5 subunit alpha
Radix Glycyrrhizae (RG)	MOL004885	licoisoflavanone	Peroxisome proliferator activated receptor gamma
Radix Glycyrrhizae (RG)	MOL004885	licoisoflavanone	Coagulation factor Xa
Radix Glycyrrhizae (RG)	MOL004885	licoisoflavanone	Prostaglandin G/H synthase 2
Radix Glycyrrhizae (RG)	MOL004885	licoisoflavanone	Coagulation factor VII
Radix Glycyrrhizae (RG)	MOL004885	licoisoflavanone	Acetylcholinesterase
Radix Glycyrrhizae (RG)	MOL004885	licoisoflavanone	DNA topoisomerase II
Radix Glycyrrhizae (RG)	MOL004885	licoisoflavanone	Estrogen receptor beta
Radix Glycyrrhizae (RG)	MOL004885	licoisoflavanone	Glycogen synthase kinase-3 beta
Radix Glycyrrhizae (RG)	MOL004885	licoisoflavanone	Heat shock protein HSP 90
Radix Glycyrrhizae (RG)	MOL004885	licoisoflavanone	Cell division protein kinase 2
Radix Glycyrrhizae (RG)	MOL004885	licoisoflavanone	Trypsin-1
Radix Glycyrrhizae (RG)	MOL004885	licoisoflavanone	Proto-oncogene serine/threonine-protein kinase Pim-1
Radix Glycyrrhizae (RG)	MOL004885	licoisoflavanone	Cyclin-A2
Radix Glycyrrhizae (RG)	MOL004885	licoisoflavanone	Nuclear receptor coactivator 1
Radix Glycyrrhizae (RG)	MOL004885	licoisoflavanone	Calmodulin
Radix Glycyrrhizae (RG)	MOL004891	shinpterocarpin	Nitric oxide synthase, inducible
Radix Glycyrrhizae (RG)	MOL004891	shinpterocarpin	Prostaglandin G/H synthase 1
Radix Glycyrrhizae (RG)	MOL004891	shinpterocarpin	Muscarinic acetylcholine receptor M3
Radix Glycyrrhizae (RG)	MOL004891	shinpterocarpin	Potassium voltage-gated channel subfamily H member 2
Radix Glycyrrhizae (RG)	MOL004891	shinpterocarpin	Muscarinic acetylcholine receptor M1
Radix Glycyrrhizae (RG)	MOL004891	shinpterocarpin	Estrogen receptor
Radix Glycyrrhizae (RG)	MOL004891	shinpterocarpin	Androgen receptor
Radix Glycyrrhizae (RG)	MOL004891	shinpterocarpin	Sodium channel protein type 5 subunit alpha
Radix Glycyrrhizae (RG)	MOL004891	shinpterocarpin	Peroxisome proliferator activated receptor gamma
Radix Glycyrrhizae (RG)	MOL004891	shinpterocarpin	Prostaglandin G/H synthase 2
Radix Glycyrrhizae (RG)	MOL004891	shinpterocarpin	5-hydroxytryptamine receptor 3A
Radix Glycyrrhizae (RG)	MOL004891	shinpterocarpin	Retinoic acid receptor RXR-alpha
Radix Glycyrrhizae (RG)	MOL004891	shinpterocarpin	Delta-type opioid receptor

Radix Glycyrrhizae (RG)	MOL004891	shinpterocarpin	Alpha-1B adrenergic receptor
Radix Glycyrrhizae (RG)	MOL004891	shinpterocarpin	Beta-2 adrenergic receptor
Radix Glycyrrhizae (RG)	MOL004891	shinpterocarpin	Alpha-1D adrenergic receptor
Radix Glycyrrhizae (RG)	MOL004891	shinpterocarpin	Mu-type opioid receptor
Radix Glycyrrhizae (RG)	MOL004891	shinpterocarpin	Estrogen receptor beta
Radix Glycyrrhizae (RG)	MOL004891	shinpterocarpin	Mitogen-activated protein kinase 14
Radix Glycyrrhizae (RG)	MOL004891	shinpterocarpin	Glycogen synthase kinase-3 beta
Radix Glycyrrhizae (RG)	MOL004891	shinpterocarpin	Cell division protein kinase 2
Radix Glycyrrhizae (RG)	MOL004891	shinpterocarpin	Phosphatidylinositol-4,5-bisphosphate 3-kinase catalytic subunit, gamma
Radix Glycyrrhizae (RG)	MOL004891	shinpterocarpin	Neuronal acetylcholine receptor protein, alpha-7 chain
Radix Glycyrrhizae (RG)	MOL004891	shinpterocarpin	mRNA of PKA Catalytic Subunit C-
Radix Glycyrrhizae (RG)	MOL004891	shinpterocarpin	Retinoic acid receptor RXR-beta
Radix Glycyrrhizae (RG)	MOL004891	shinpterocarpin	Trypsin-1
Radix Glycyrrhizae (RG)	MOL004891	shinpterocarpin	Proto-oncogene serine/threonine-protein kinase Pim-1
Radix Glycyrrhizae (RG)	MOL004891	shinpterocarpin	Cyclin-A2
Radix Glycyrrhizae (RG)	MOL004891	shinpterocarpin	Nuclear receptor coactivator 1
Radix Glycyrrhizae (RG)	MOL004891	shinpterocarpin	Calmodulin
Radix Glycyrrhizae (RG)	MOL004898	(E)-3-[3,4-dihydroxy-5-(3-methylbut-2-enyl)phenyl]-1-(2,4-dihydroxyphenyl)prop-2-en-1-one	Estrogen receptor
Radix Glycyrrhizae (RG)	MOL004898	(E)-3-[3,4-dihydroxy-5-(3-methylbut-2-enyl)phenyl]-1-(2,4-dihydroxyphenyl)prop-2-en-1-one	Androgen receptor
Radix Glycyrrhizae (RG)	MOL004898	(E)-3-[3,4-dihydroxy-5-(3-methylbut-2-enyl)phenyl]-1-(2,4-dihydroxyphenyl)prop-2-en-1-one	Peroxisome proliferator activated receptor gamma
Radix Glycyrrhizae (RG)	MOL004898	(E)-3-[3,4-dihydroxy-5-(3-methylbut-2-enyl)phenyl]-1-(2,4-dihydroxyphenyl)prop-2-en-1-one	Prostaglandin G/H synthase 2
Radix Glycyrrhizae (RG)	MOL004898	(E)-3-[3,4-dihydroxy-5-(3-methylbut-2-enyl)phenyl]-1-(2,4-dihydroxyphenyl)prop-2-en-1-one	Mitogen-activated protein kinase 14
Radix Glycyrrhizae (RG)	MOL004898	(E)-3-[3,4-dihydroxy-5-(3-methylbut-2-enyl)phenyl]-1-(2,4-dihydroxyphenyl)prop-2-en-1-one	Glycogen synthase kinase-3 beta
Radix Glycyrrhizae (RG)	MOL004898	(E)-3-[3,4-dihydroxy-5-(3-methylbut-2-enyl)phenyl]-1-(2,4-dihydroxyphenyl)prop-2-en-1-one	Heat shock protein HSP 90
Radix Glycyrrhizae (RG)	MOL004898	(E)-3-[3,4-dihydroxy-5-(3-methylbut-2-enyl)phenyl]-1-(2,4-dihydroxyphenyl)prop-2-en-1-one	Cell division protein kinase 2
Radix Glycyrrhizae (RG)	MOL004898	(E)-3-[3,4-dihydroxy-5-(3-methylbut-2-enyl)phenyl]-1-(2,4-dihydroxyphenyl)prop-2-en-1-one	Proto-oncogene serine/threonine-protein kinase Pim-1
Radix Glycyrrhizae (RG)	MOL004898	(E)-3-[3,4-dihydroxy-5-(3-methylbut-2-enyl)phenyl]-1-(2,4-dihydroxyphenyl)prop-2-en-1-one	Cyclin-A2

Radix Glycyrrhizae (RG)	MOL004898	(E)-3-[3,4-dihydroxy-5-(3-methylbut-2-enyl)phenyl]-1-(2,4-dihydroxyphenyl)prop-2-en-1-one	Nuclear receptor coactivator 2
Radix Glycyrrhizae (RG)	MOL004898	(E)-3-[3,4-dihydroxy-5-(3-methylbut-2-enyl)phenyl]-1-(2,4-dihydroxyphenyl)prop-2-en-1-one	Calmodulin
Radix Glycyrrhizae (RG)	MOL004903	liquiritin	Coagulation factor Xa
Radix Glycyrrhizae (RG)	MOL004903	liquiritin	Coagulation factor VII
Radix Glycyrrhizae (RG)	MOL004903	liquiritin	Calmodulin
Radix Glycyrrhizae (RG)	MOL004903	liquiritin	Prostaglandin G/H synthase 2
Radix Glycyrrhizae (RG)	MOL004903	liquiritin	Vascular endothelial growth factor receptor 2
Radix Glycyrrhizae (RG)	MOL004903	liquiritin	Superoxide dismutase [Cu-Zn]
Radix Glycyrrhizae (RG)	MOL004904	licopyranocoumarin	Nitric oxide synthase, inducible
Radix Glycyrrhizae (RG)	MOL004904	licopyranocoumarin	Thrombin
Radix Glycyrrhizae (RG)	MOL004904	licopyranocoumarin	Estrogen receptor
Radix Glycyrrhizae (RG)	MOL004904	licopyranocoumarin	Androgen receptor
Radix Glycyrrhizae (RG)	MOL004904	licopyranocoumarin	Peroxisome proliferator activated receptor gamma
Radix Glycyrrhizae (RG)	MOL004904	licopyranocoumarin	Coagulation factor Xa
Radix Glycyrrhizae (RG)	MOL004904	licopyranocoumarin	Prostaglandin G/H synthase 2
Radix Glycyrrhizae (RG)	MOL004904	licopyranocoumarin	Coagulation factor VII
Radix Glycyrrhizae (RG)	MOL004904	licopyranocoumarin	Vascular endothelial growth factor receptor 2
Radix Glycyrrhizae (RG)	MOL004904	licopyranocoumarin	Acetylcholinesterase
Radix Glycyrrhizae (RG)	MOL004904	licopyranocoumarin	DNA topoisomerase II
Radix Glycyrrhizae (RG)	MOL004904	licopyranocoumarin	Cell division protein kinase 2
Radix Glycyrrhizae (RG)	MOL004904	licopyranocoumarin	Trypsin-1
Radix Glycyrrhizae (RG)	MOL004904	licopyranocoumarin	Proto-oncogene serine/threonine-protein kinase Pim-1
Radix Glycyrrhizae (RG)	MOL004904	licopyranocoumarin	Cyclin-A2
Radix Glycyrrhizae (RG)	MOL004904	licopyranocoumarin	Calmodulin
Radix Glycyrrhizae (RG)	MOL004907	Glyzaglabrin	Nitric oxide synthase, inducible
Radix Glycyrrhizae (RG)	MOL004907	Glyzaglabrin	Prostaglandin G/H synthase 1
Radix Glycyrrhizae (RG)	MOL004907	Glyzaglabrin	Estrogen receptor
Radix Glycyrrhizae (RG)	MOL004907	Glyzaglabrin	Androgen receptor
Radix Glycyrrhizae (RG)	MOL004907	Glyzaglabrin	Peroxisome proliferator activated receptor gamma
Radix Glycyrrhizae (RG)	MOL004907	Glyzaglabrin	Prostaglandin G/H synthase 2
Radix Glycyrrhizae (RG)	MOL004907	Glyzaglabrin	Estrogen receptor beta
Radix Glycyrrhizae (RG)	MOL004907	Glyzaglabrin	Dipeptidyl peptidase IV
Radix Glycyrrhizae (RG)	MOL004907	Glyzaglabrin	Mitogen-activated protein kinase 14
Radix Glycyrrhizae (RG)	MOL004907	Glyzaglabrin	Glycogen synthase kinase-3 beta
Radix Glycyrrhizae (RG)	MOL004907	Glyzaglabrin	Heat shock protein HSP 90
Radix Glycyrrhizae (RG)	MOL004907	Glyzaglabrin	Cell division protein kinase 2
Radix Glycyrrhizae (RG)	MOL004907	Glyzaglabrin	Phosphatidylinositol-4,5-bisphosphate 3-kinase catalytic subunit, gamma
Radix Glycyrrhizae (RG)	MOL004907	Glyzaglabrin	Serine/threonine-protein kinase Chk1
Radix Glycyrrhizae (RG)	MOL004907	Glyzaglabrin	mRNA of PKA Catalytic Subunit C-
Radix Glycyrrhizae (RG)	MOL004907	Glyzaglabrin	Trypsin-1
Radix Glycyrrhizae (RG)	MOL004907	Glyzaglabrin	Proto-oncogene serine/threonine-protein kinase Pim-1
Radix Glycyrrhizae (RG)	MOL004907	Glyzaglabrin	Cyclin-A2
Radix Glycyrrhizae (RG)	MOL004908	Glabridin	Nitric oxide synthase, inducible
Radix Glycyrrhizae (RG)	MOL004908	Glabridin	Muscarinic acetylcholine receptor M1
Radix Glycyrrhizae (RG)	MOL004908	Glabridin	Estrogen receptor
Radix Glycyrrhizae (RG)	MOL004908	Glabridin	Androgen receptor
Radix Glycyrrhizae (RG)	MOL004908	Glabridin	Sodium channel protein type 5 subunit alpha
Radix Glycyrrhizae (RG)	MOL004908	Glabridin	Peroxisome proliferator activated receptor gamma

Radix Glycyrrhizae (RG)	MOL004908	Glabridin	Prostaglandin G/H synthase 2
Radix Glycyrrhizae (RG)	MOL004908	Glabridin	Retinoic acid receptor RXR-alpha
Radix Glycyrrhizae (RG)	MOL004908	Glabridin	Acetylcholinesterase
Radix Glycyrrhizae (RG)	MOL004908	Glabridin	Alpha-1B adrenergic receptor
Radix Glycyrrhizae (RG)	MOL004908	Glabridin	Beta-2 adrenergic receptor
Radix Glycyrrhizae (RG)	MOL004908	Glabridin	Estrogen receptor beta
Radix Glycyrrhizae (RG)	MOL004908	Glabridin	Mitogen-activated protein kinase 14
Radix Glycyrrhizae (RG)	MOL004908	Glabridin	Glycogen synthase kinase-3 beta
Radix Glycyrrhizae (RG)	MOL004908	Glabridin	Cell division protein kinase 2
Radix Glycyrrhizae (RG)	MOL004908	Glabridin	Serine/threonine-protein kinase Chk1
Radix Glycyrrhizae (RG)	MOL004908	Glabridin	mRNA of PKA Catalytic Subunit C-
Radix Glycyrrhizae (RG)	MOL004908	Glabridin	Retinoic acid receptor RXR-beta
Radix Glycyrrhizae (RG)	MOL004908	Glabridin	Ig gamma-1 chain C region
Radix Glycyrrhizae (RG)	MOL004908	Glabridin	Trypsin-1
Radix Glycyrrhizae (RG)	MOL004908	Glabridin	Proto-oncogene serine/threonine-protein kinase Pim-1
Radix Glycyrrhizae (RG)	MOL004908	Glabridin	Cyclin-A2
Radix Glycyrrhizae (RG)	MOL004908	Glabridin	Nuclear receptor coactivator 2
Radix Glycyrrhizae (RG)	MOL004908	Glabridin	Nuclear receptor coactivator 1
Radix Glycyrrhizae (RG)	MOL004908	Glabridin	Calmodulin
Radix Glycyrrhizae (RG)	MOL004910	Glabranin	Nitric oxide synthase, inducible
Radix Glycyrrhizae (RG)	MOL004910	Glabranin	Prostaglandin G/H synthase 1
Radix Glycyrrhizae (RG)	MOL004910	Glabranin	Estrogen receptor
Radix Glycyrrhizae (RG)	MOL004910	Glabranin	Sodium channel protein type 5 subunit alpha
Radix Glycyrrhizae (RG)	MOL004910	Glabranin	Coagulation factor Xa
Radix Glycyrrhizae (RG)	MOL004910	Glabranin	Prostaglandin G/H synthase 2
Radix Glycyrrhizae (RG)	MOL004910	Glabranin	Nitric-oxide synthase, endothelial
Radix Glycyrrhizae (RG)	MOL004910	Glabranin	CGMP-inhibited 3',5'-cyclic phosphodiesterase A
Radix Glycyrrhizae (RG)	MOL004910	Glabranin	Heat shock protein HSP 90
Radix Glycyrrhizae (RG)	MOL004910	Glabranin	mRNA of PKA Catalytic Subunit C-
Radix Glycyrrhizae (RG)	MOL004910	Glabranin	Calmodulin
Radix Glycyrrhizae (RG)	MOL004911	Glabrene	Nitric oxide synthase, inducible
Radix Glycyrrhizae (RG)	MOL004911	Glabrene	Prostaglandin G/H synthase 1
Radix Glycyrrhizae (RG)	MOL004911	Glabrene	Estrogen receptor
Radix Glycyrrhizae (RG)	MOL004911	Glabrene	Androgen receptor
Radix Glycyrrhizae (RG)	MOL004911	Glabrene	Sodium channel protein type 5 subunit alpha
Radix Glycyrrhizae (RG)	MOL004911	Glabrene	Peroxisome proliferator activated receptor gamma
Radix Glycyrrhizae (RG)	MOL004911	Glabrene	Coagulation factor Xa
Radix Glycyrrhizae (RG)	MOL004911	Glabrene	Prostaglandin G/H synthase 2
Radix Glycyrrhizae (RG)	MOL004911	Glabrene	Retinoic acid receptor RXR-alpha
Radix Glycyrrhizae (RG)	MOL004911	Glabrene	Beta-2 adrenergic receptor
Radix Glycyrrhizae (RG)	MOL004911	Glabrene	Estrogen receptor beta
Radix Glycyrrhizae (RG)	MOL004911	Glabrene	Mitogen-activated protein kinase 14
Radix Glycyrrhizae (RG)	MOL004911	Glabrene	Glycogen synthase kinase-3 beta
Radix Glycyrrhizae (RG)	MOL004911	Glabrene	Heat shock protein HSP 90
Radix Glycyrrhizae (RG)	MOL004911	Glabrene	Cell division protein kinase 2
Radix Glycyrrhizae (RG)	MOL004911	Glabrene	Trypsin-1
Radix Glycyrrhizae (RG)	MOL004911	Glabrene	Proto-oncogene serine/threonine-protein kinase Pim-1
Radix Glycyrrhizae (RG)	MOL004911	Glabrene	Nuclear receptor coactivator 2
Radix Glycyrrhizae (RG)	MOL004911	Glabrene	Calmodulin
Radix Glycyrrhizae (RG)	MOL004912	Glabrone	Nitric oxide synthase, inducible
Radix Glycyrrhizae (RG)	MOL004912	Glabrone	Prostaglandin G/H synthase 1
Radix Glycyrrhizae (RG)	MOL004912	Glabrone	Thrombin
Radix Glycyrrhizae (RG)	MOL004912	Glabrone	Estrogen receptor
Radix Glycyrrhizae (RG)	MOL004912	Glabrone	Androgen receptor
Radix Glycyrrhizae (RG)	MOL004912	Glabrone	Sodium channel protein type 5 subunit alpha

Radix Glycyrrhizae (RG)	MOL004912	Glabrone	Peroxisome proliferator activated receptor gamma
Radix Glycyrrhizae (RG)	MOL004912	Glabrone	Coagulation factor Xa
Radix Glycyrrhizae (RG)	MOL004912	Glabrone	Prostaglandin G/H synthase 2
Radix Glycyrrhizae (RG)	MOL004912	Glabrone	Retinoic acid receptor RXR-alpha
Radix Glycyrrhizae (RG)	MOL004912	Glabrone	Acetylcholinesterase
Radix Glycyrrhizae (RG)	MOL004912	Glabrone	Estrogen receptor beta
Radix Glycyrrhizae (RG)	MOL004912	Glabrone	Dipeptidyl peptidase IV
Radix Glycyrrhizae (RG)	MOL004912	Glabrone	Mitogen-activated protein kinase 14
Radix Glycyrrhizae (RG)	MOL004912	Glabrone	Glycogen synthase kinase-3 beta
Radix Glycyrrhizae (RG)	MOL004912	Glabrone	Cell division protein kinase 2
Radix Glycyrrhizae (RG)	MOL004912	Glabrone	Serine/threonine-protein kinase Chk1
Radix Glycyrrhizae (RG)	MOL004912	Glabrone	Trypsin-1
Radix Glycyrrhizae (RG)	MOL004912	Glabrone	Proto-oncogene serine/threonine-protein kinase Pim-1
Radix Glycyrrhizae (RG)	MOL004912	Glabrone	Cyclin-A2
Radix Glycyrrhizae (RG)	MOL004912	Glabrone	Calmodulin
Radix Glycyrrhizae (RG)	MOL004913	1,3-dihydroxy-9-methoxy-6-benzofurano[3,2-c]chromenone	Estrogen receptor
Radix Glycyrrhizae (RG)	MOL004913	1,3-dihydroxy-9-methoxy-6-benzofurano[3,2-c]chromenone	Peroxisome proliferator activated receptor gamma
Radix Glycyrrhizae (RG)	MOL004913	1,3-dihydroxy-9-methoxy-6-benzofurano[3,2-c]chromenone	Estrogen receptor beta
Radix Glycyrrhizae (RG)	MOL004913	1,3-dihydroxy-9-methoxy-6-benzofurano[3,2-c]chromenone	Mitogen-activated protein kinase 14
Radix Glycyrrhizae (RG)	MOL004913	1,3-dihydroxy-9-methoxy-6-benzofurano[3,2-c]chromenone	Glycogen synthase kinase-3 beta
Radix Glycyrrhizae (RG)	MOL004913	1,3-dihydroxy-9-methoxy-6-benzofurano[3,2-c]chromenone	Heat shock protein HSP 90
Radix Glycyrrhizae (RG)	MOL004913	1,3-dihydroxy-9-methoxy-6-benzofurano[3,2-c]chromenone	Cell division protein kinase 2
Radix Glycyrrhizae (RG)	MOL004913	1,3-dihydroxy-9-methoxy-6-benzofurano[3,2-c]chromenone	Serine/threonine-protein kinase Chk1
Radix Glycyrrhizae (RG)	MOL004913	1,3-dihydroxy-9-methoxy-6-benzofurano[3,2-c]chromenone	mRNA of PKA Catalytic Subunit C-alpha
Radix Glycyrrhizae (RG)	MOL004913	1,3-dihydroxy-9-methoxy-6-benzofurano[3,2-c]chromenone	Cyclin-A2
Radix Glycyrrhizae (RG)	MOL004914	1,3-dihydroxy-8,9-dimethoxy-6-benzofurano[3,2-	Estrogen receptor
Radix Glycyrrhizae (RG)	MOL004914	1,3-dihydroxy-8,9-dimethoxy-6-benzofurano[3,2-	Androgen receptor
Radix Glycyrrhizae (RG)	MOL004914	1,3-dihydroxy-8,9-dimethoxy-6-benzofurano[3,2-	Peroxisome proliferator activated receptor gamma
Radix Glycyrrhizae (RG)	MOL004914	1,3-dihydroxy-8,9-dimethoxy-6-benzofurano[3,2-	Mitogen-activated protein kinase 14
Radix Glycyrrhizae (RG)	MOL004914	1,3-dihydroxy-8,9-dimethoxy-6-benzofurano[3,2-	Glycogen synthase kinase-3 beta
Radix Glycyrrhizae (RG)	MOL004914	1,3-dihydroxy-8,9-dimethoxy-6-benzofurano[3,2-	Heat shock protein HSP 90
Radix Glycyrrhizae (RG)	MOL004914	1,3-dihydroxy-8,9-dimethoxy-6-benzofurano[3,2-	Cell division protein kinase 2
Radix Glycyrrhizae (RG)	MOL004914	1,3-dihydroxy-8,9-dimethoxy-6-benzofurano[3,2-	Serine/threonine-protein kinase Chk1
Radix Glycyrrhizae (RG)	MOL004914	1,3-dihydroxy-8,9-dimethoxy-6-benzofurano[3,2-	mRNA of PKA Catalytic Subunit C-alpha
Radix Glycyrrhizae (RG)	MOL004915	Eurycarpin A	Nitric oxide synthase, inducible
Radix Glycyrrhizae (RG)	MOL004915	Eurycarpin A	Thrombin
Radix Glycyrrhizae (RG)	MOL004915	Eurycarpin A	Estrogen receptor
Radix Glycyrrhizae (RG)	MOL004915	Eurycarpin A	Androgen receptor
Radix Glycyrrhizae (RG)	MOL004915	Eurycarpin A	Sodium channel protein type 5 subunit alpha
Radix Glycyrrhizae (RG)	MOL004915	Eurycarpin A	Peroxisome proliferator activated receptor gamma

Radix Glycyrrhizae (RG)	MOL004915	Eurycarpin A	Coagulation factor Xa
Radix Glycyrrhizae (RG)	MOL004915	Eurycarpin A	Prostaglandin G/H synthase 2
Radix Glycyrrhizae (RG)	MOL004915	Eurycarpin A	Estrogen receptor beta
Radix Glycyrrhizae (RG)	MOL004915	Eurycarpin A	Dipeptidyl peptidase IV
Radix Glycyrrhizae (RG)	MOL004915	Eurycarpin A	Mitogen-activated protein kinase 14
Radix Glycyrrhizae (RG)	MOL004915	Eurycarpin A	Glycogen synthase kinase-3 beta
Radix Glycyrrhizae (RG)	MOL004915	Eurycarpin A	Heat shock protein HSP 90
Radix Glycyrrhizae (RG)	MOL004915	Eurycarpin A	Cell division protein kinase 2
Radix Glycyrrhizae (RG)	MOL004915	Eurycarpin A	Serine/threonine-protein kinase Chk1
Radix Glycyrrhizae (RG)	MOL004915	Eurycarpin A	Trypsin-1
Radix Glycyrrhizae (RG)	MOL004915	Eurycarpin A	Proto-oncogene serine/threonine-protein kinase Pim-1
Radix Glycyrrhizae (RG)	MOL004915	Eurycarpin A	Cyclin-A2
Radix Glycyrrhizae (RG)	MOL004915	Eurycarpin A	Calmodulin
Radix Glycyrrhizae (RG)	MOL004924	(-)-Medicocarpin	Prostaglandin G/H synthase 2
Radix Glycyrrhizae (RG)	MOL004924	(-)-Medicocarpin	Acetylcholinesterase
Radix Glycyrrhizae (RG)	MOL004935	Sigmoidin-B	Estrogen receptor
Radix Glycyrrhizae (RG)	MOL004935	Sigmoidin-B	Coagulation factor Xa
Radix Glycyrrhizae (RG)	MOL004935	Sigmoidin-B	Prostaglandin G/H synthase 2
Radix Glycyrrhizae (RG)	MOL004935	Sigmoidin-B	Vascular endothelial growth factor receptor 2
Radix Glycyrrhizae (RG)	MOL004935	Sigmoidin-B	Heat shock protein HSP 90
Radix Glycyrrhizae (RG)	MOL004935	Sigmoidin-B	Calmodulin
Radix Glycyrrhizae (RG)	MOL004941	(2R)-7-hydroxy-2-(4-hydroxyphenyl)chroman-4-one	Prostaglandin G/H synthase 1
Radix Glycyrrhizae (RG)	MOL004941	(2R)-7-hydroxy-2-(4-hydroxyphenyl)chroman-4-one	Estrogen receptor
Radix Glycyrrhizae (RG)	MOL004941	(2R)-7-hydroxy-2-(4-hydroxyphenyl)chroman-4-one	Prostaglandin G/H synthase 2
Radix Glycyrrhizae (RG)	MOL004941	(2R)-7-hydroxy-2-(4-hydroxyphenyl)chroman-4-one	Retinoic acid receptor RXR-alpha
Radix Glycyrrhizae (RG)	MOL004941	(2R)-7-hydroxy-2-(4-hydroxyphenyl)chroman-4-one	CGMP-inhibited 3',5'-cyclic phosphodiesterase A
Radix Glycyrrhizae (RG)	MOL004941	(2R)-7-hydroxy-2-(4-hydroxyphenyl)chroman-4-one	Beta-2 adrenergic receptor
Radix Glycyrrhizae (RG)	MOL004941	(2R)-7-hydroxy-2-(4-hydroxyphenyl)chroman-4-one	Heat shock protein HSP 90
Radix Glycyrrhizae (RG)	MOL004941	(2R)-7-hydroxy-2-(4-hydroxyphenyl)chroman-4-one	Phosphatidylinositol-4,5-bisphosphate 3-kinase catalytic subunit, gamma
Radix Glycyrrhizae (RG)	MOL004941	(2R)-7-hydroxy-2-(4-hydroxyphenyl)chroman-4-one	Beta-lactamase
Radix Glycyrrhizae (RG)	MOL004941	(2R)-7-hydroxy-2-(4-hydroxyphenyl)chroman-4-one	Amine oxidase [flavin-containing] B
Radix Glycyrrhizae (RG)	MOL004941	(2R)-7-hydroxy-2-(4-hydroxyphenyl)chroman-4-one	mRNA of PKA Catalytic Subunit C-alpha
Radix Glycyrrhizae (RG)	MOL004941	(2R)-7-hydroxy-2-(4-hydroxyphenyl)chroman-4-one	cAMP-dependent protein kinase inhibitor alpha
Radix Glycyrrhizae (RG)	MOL004941	(2R)-7-hydroxy-2-(4-hydroxyphenyl)chroman-4-one	Calmodulin
Radix Glycyrrhizae (RG)	MOL004941	(2R)-7-hydroxy-2-(4-hydroxyphenyl)chroman-4-one	Gamma-aminobutyric acid receptor subunit alpha-1
Radix Glycyrrhizae (RG)	MOL004941	(2R)-7-hydroxy-2-(4-hydroxyphenyl)chroman-4-one	Sodium-dependent serotonin transporter
Radix Glycyrrhizae (RG)	MOL004945	(2S)-7-hydroxy-2-(4-hydroxyphenyl)-8-(3-methylbut-2-enyl)chroman-4-	Nitric oxide synthase, inducible
Radix Glycyrrhizae (RG)	MOL004945	(2S)-7-hydroxy-2-(4-hydroxyphenyl)-8-(3-methylbut-2-enyl)chroman-4-	Prostaglandin G/H synthase 1
Radix Glycyrrhizae (RG)	MOL004945	(2S)-7-hydroxy-2-(4-hydroxyphenyl)-8-(3-methylbut-2-enyl)chroman-4-	Estrogen receptor

Radix Glycyrrhizae (RG)	MOL004945	(2S)-7-hydroxy-2-(4-hydroxyphenyl)-8-(3-methylbut-2-enyl)chroman-4-	Sodium channel protein type 5 subunit alpha
Radix Glycyrrhizae (RG)	MOL004945	(2S)-7-hydroxy-2-(4-hydroxyphenyl)-8-(3-methylbut-2-enyl)chroman-4-	Coagulation factor Xa
Radix Glycyrrhizae (RG)	MOL004945	(2S)-7-hydroxy-2-(4-hydroxyphenyl)-8-(3-methylbut-2-enyl)chroman-4-	Prostaglandin G/H synthase 2
Radix Glycyrrhizae (RG)	MOL004945	(2S)-7-hydroxy-2-(4-hydroxyphenyl)-8-(3-methylbut-2-enyl)chroman-4-	CGMP-inhibited 3',5'-cyclic phosphodiesterase A
Radix Glycyrrhizae (RG)	MOL004945	(2S)-7-hydroxy-2-(4-hydroxyphenyl)-8-(3-methylbut-2-enyl)chroman-4-	Alpha-1B adrenergic receptor
Radix Glycyrrhizae (RG)	MOL004945	(2S)-7-hydroxy-2-(4-hydroxyphenyl)-8-(3-methylbut-2-enyl)chroman-4-	Beta-2 adrenergic receptor
Radix Glycyrrhizae (RG)	MOL004945	(2S)-7-hydroxy-2-(4-hydroxyphenyl)-8-(3-methylbut-2-enyl)chroman-4-	Estrogen receptor beta
Radix Glycyrrhizae (RG)	MOL004945	(2S)-7-hydroxy-2-(4-hydroxyphenyl)-8-(3-methylbut-2-enyl)chroman-4-	Heat shock protein HSP 90
Radix Glycyrrhizae (RG)	MOL004945	(2S)-7-hydroxy-2-(4-hydroxyphenyl)-8-(3-methylbut-2-enyl)chroman-4-	Calmodulin
Radix Glycyrrhizae (RG)	MOL004948	Isoglycyrol	Nitric oxide synthase, inducible
Radix Glycyrrhizae (RG)	MOL004948	Isoglycyrol	Estrogen receptor
Radix Glycyrrhizae (RG)	MOL004948	Isoglycyrol	Androgen receptor
Radix Glycyrrhizae (RG)	MOL004948	Isoglycyrol	Prostaglandin G/H synthase 2
Radix Glycyrrhizae (RG)	MOL004948	Isoglycyrol	Dipeptidyl peptidase IV
Radix Glycyrrhizae (RG)	MOL004948	Isoglycyrol	Glycogen synthase kinase-3 beta
Radix Glycyrrhizae (RG)	MOL004948	Isoglycyrol	Proto-oncogene serine/threonine-protein kinase Pim-1
Radix Glycyrrhizae (RG)	MOL004949	Isolicoflavonol	Nitric oxide synthase, inducible
Radix Glycyrrhizae (RG)	MOL004949	Isolicoflavonol	Thrombin
Radix Glycyrrhizae (RG)	MOL004949	Isolicoflavonol	Estrogen receptor
Radix Glycyrrhizae (RG)	MOL004949	Isolicoflavonol	Androgen receptor
Radix Glycyrrhizae (RG)	MOL004949	Isolicoflavonol	Peroxisome proliferator activated receptor gamma
Radix Glycyrrhizae (RG)	MOL004949	Isolicoflavonol	Coagulation factor Xa
Radix Glycyrrhizae (RG)	MOL004949	Isolicoflavonol	Prostaglandin G/H synthase 2
Radix Glycyrrhizae (RG)	MOL004949	Isolicoflavonol	Glycogen synthase kinase-3 beta
Radix Glycyrrhizae (RG)	MOL004949	Isolicoflavonol	Heat shock protein HSP 90
Radix Glycyrrhizae (RG)	MOL004949	Isolicoflavonol	Cell division protein kinase 2
Radix Glycyrrhizae (RG)	MOL004949	Isolicoflavonol	Trypsin-1
Radix Glycyrrhizae (RG)	MOL004949	Isolicoflavonol	Proto-oncogene serine/threonine-protein kinase Pim-1
Radix Glycyrrhizae (RG)	MOL004949	Isolicoflavonol	Cyclin-A2
Radix Glycyrrhizae (RG)	MOL004949	Isolicoflavonol	Nuclear receptor coactivator 2
Radix Glycyrrhizae (RG)	MOL004949	Isolicoflavonol	Calmodulin
Radix Glycyrrhizae (RG)	MOL004957	HMO	Nitric oxide synthase, inducible
Radix Glycyrrhizae (RG)	MOL004957	HMO	Prostaglandin G/H synthase 1
Radix Glycyrrhizae (RG)	MOL004957	HMO	Muscarinic acetylcholine receptor M1
Radix Glycyrrhizae (RG)	MOL004957	HMO	Estrogen receptor
Radix Glycyrrhizae (RG)	MOL004957	HMO	Androgen receptor
Radix Glycyrrhizae (RG)	MOL004957	HMO	Sodium channel protein type 5 subunit alpha
Radix Glycyrrhizae (RG)	MOL004957	HMO	Peroxisome proliferator activated receptor gamma
Radix Glycyrrhizae (RG)	MOL004957	HMO	Prostaglandin G/H synthase 2
Radix Glycyrrhizae (RG)	MOL004957	HMO	Retinoic acid receptor RXR-alpha

Radix Glycyrrhizae (RG)	MOL004957	HMO	CGMP-inhibited 3',5'-cyclic phosphodiesterase A
Radix Glycyrrhizae (RG)	MOL004957	HMO	Sodium-dependent dopamine
Radix Glycyrrhizae (RG)	MOL004957	HMO	Beta-2 adrenergic receptor
Radix Glycyrrhizae (RG)	MOL004957	HMO	Sodium-dependent serotonin transporter
Radix Glycyrrhizae (RG)	MOL004957	HMO	Estrogen receptor beta
Radix Glycyrrhizae (RG)	MOL004957	HMO	Dipeptidyl peptidase IV
Radix Glycyrrhizae (RG)	MOL004957	HMO	Mitogen-activated protein kinase 14
Radix Glycyrrhizae (RG)	MOL004957	HMO	Glycogen synthase kinase-3 beta
Radix Glycyrrhizae (RG)	MOL004957	HMO	Cell division protein kinase 2
Radix Glycyrrhizae (RG)	MOL004957	HMO	Amine oxidase [flavin-containing] B
Radix Glycyrrhizae (RG)	MOL004957	HMO	Serine/threonine-protein kinase Chk1
Radix Glycyrrhizae (RG)	MOL004957	HMO	mRNA of PKA Catalytic Subunit C-
Radix Glycyrrhizae (RG)	MOL004957	HMO	Ig gamma-1 chain C region
Radix Glycyrrhizae (RG)	MOL004957	HMO	Trypsin-1
Radix Glycyrrhizae (RG)	MOL004957	HMO	Proto-oncogene serine/threonine-protein kinase Pim-1
Radix Glycyrrhizae (RG)	MOL004957	HMO	Cyclin-A2
Radix Glycyrrhizae (RG)	MOL004957	HMO	cAMP-dependent protein kinase inhibitor alpha
Radix Glycyrrhizae (RG)	MOL004957	HMO	Calmodulin
Radix Glycyrrhizae (RG)	MOL004959	1-Methoxyphaseollidin	Nitric oxide synthase, inducible
Radix Glycyrrhizae (RG)	MOL004959	1-Methoxyphaseollidin	Prostaglandin G/H synthase 1
Radix Glycyrrhizae (RG)	MOL004959	1-Methoxyphaseollidin	Thrombin
Radix Glycyrrhizae (RG)	MOL004959	1-Methoxyphaseollidin	Potassium voltage-gated channel subfamily H member 2
Radix Glycyrrhizae (RG)	MOL004959	1-Methoxyphaseollidin	Estrogen receptor
Radix Glycyrrhizae (RG)	MOL004959	1-Methoxyphaseollidin	Androgen receptor
Radix Glycyrrhizae (RG)	MOL004959	1-Methoxyphaseollidin	Sodium channel protein type 5 subunit alpha
Radix Glycyrrhizae (RG)	MOL004959	1-Methoxyphaseollidin	Peroxisome proliferator activated receptor gamma
Radix Glycyrrhizae (RG)	MOL004959	1-Methoxyphaseollidin	Coagulation factor Xa
Radix Glycyrrhizae (RG)	MOL004959	1-Methoxyphaseollidin	Prostaglandin G/H synthase 2
Radix Glycyrrhizae (RG)	MOL004959	1-Methoxyphaseollidin	Nitric-oxide synthase, endothelial
Radix Glycyrrhizae (RG)	MOL004959	1-Methoxyphaseollidin	Vascular endothelial growth factor receptor 2
Radix Glycyrrhizae (RG)	MOL004959	1-Methoxyphaseollidin	Retinoic acid receptor RXR-alpha
Radix Glycyrrhizae (RG)	MOL004959	1-Methoxyphaseollidin	Alpha-1B adrenergic receptor
Radix Glycyrrhizae (RG)	MOL004959	1-Methoxyphaseollidin	Beta-2 adrenergic receptor
Radix Glycyrrhizae (RG)	MOL004959	1-Methoxyphaseollidin	Alpha-1D adrenergic receptor
Radix Glycyrrhizae (RG)	MOL004959	1-Methoxyphaseollidin	DNA topoisomerase II
Radix Glycyrrhizae (RG)	MOL004959	1-Methoxyphaseollidin	Estrogen receptor beta
Radix Glycyrrhizae (RG)	MOL004959	1-Methoxyphaseollidin	Mitogen-activated protein kinase 14
Radix Glycyrrhizae (RG)	MOL004959	1-Methoxyphaseollidin	Glycogen synthase kinase-3 beta
Radix Glycyrrhizae (RG)	MOL004959	1-Methoxyphaseollidin	Heat shock protein HSP 90
Radix Glycyrrhizae (RG)	MOL004959	1-Methoxyphaseollidin	Cell division protein kinase 2
Radix Glycyrrhizae (RG)	MOL004959	1-Methoxyphaseollidin	Phosphatidylinositol-4,5-bisphosphate 3-kinase catalytic subunit, gamma
Radix Glycyrrhizae (RG)	MOL004959	1-Methoxyphaseollidin	Trypsin-1
Radix Glycyrrhizae (RG)	MOL004959	1-Methoxyphaseollidin	Proto-oncogene serine/threonine-protein kinase Pim-1
Radix Glycyrrhizae (RG)	MOL004959	1-Methoxyphaseollidin	Cyclin-A2
Radix Glycyrrhizae (RG)	MOL004959	1-Methoxyphaseollidin	Nuclear receptor coactivator 2
Radix Glycyrrhizae (RG)	MOL004959	1-Methoxyphaseollidin	Nuclear receptor coactivator 1
Radix Glycyrrhizae (RG)	MOL004959	1-Methoxyphaseollidin	Calmodulin
Radix Glycyrrhizae (RG)	MOL004961	Quercetin der.	Nitric oxide synthase, inducible
Radix Glycyrrhizae (RG)	MOL004961	Quercetin der.	Prostaglandin G/H synthase 1
Radix Glycyrrhizae (RG)	MOL004961	Quercetin der.	Estrogen receptor
Radix Glycyrrhizae (RG)	MOL004961	Quercetin der.	Androgen receptor
Radix Glycyrrhizae (RG)	MOL004961	Quercetin der.	Sodium channel protein type 5 subunit alpha

Radix Glycyrrhizae (RG)	MOL004961	Quercetin der.	Peroxisome proliferator activated receptor gamma
Radix Glycyrrhizae (RG)	MOL004961	Quercetin der.	Prostaglandin G/H synthase 2
Radix Glycyrrhizae (RG)	MOL004961	Quercetin der.	mRNA of Protein-tyrosine phosphatase, non-receptor type 1
Radix Glycyrrhizae (RG)	MOL004961	Quercetin der.	Estrogen receptor beta
Radix Glycyrrhizae (RG)	MOL004961	Quercetin der.	Dipeptidyl peptidase IV
Radix Glycyrrhizae (RG)	MOL004961	Quercetin der.	Mitogen-activated protein kinase 14
Radix Glycyrrhizae (RG)	MOL004961	Quercetin der.	Glycogen synthase kinase-3 beta
Radix Glycyrrhizae (RG)	MOL004961	Quercetin der.	Heat shock protein HSP 90
Radix Glycyrrhizae (RG)	MOL004961	Quercetin der.	Cell division protein kinase 2
Radix Glycyrrhizae (RG)	MOL004961	Quercetin der.	Trypsin-1
Radix Glycyrrhizae (RG)	MOL004961	Quercetin der.	Nuclear receptor coactivator 2
Radix Glycyrrhizae (RG)	MOL004961	Quercetin der.	Calmodulin
Radix Glycyrrhizae (RG)	MOL004966	3'-Hydroxy-4'-O-Methylglabridin	Nitric oxide synthase, inducible
Radix Glycyrrhizae (RG)	MOL004966	3'-Hydroxy-4'-O-Methylglabridin	Prostaglandin G/H synthase 1
Radix Glycyrrhizae (RG)	MOL004966	3'-Hydroxy-4'-O-Methylglabridin	Potassium voltage-gated channel subfamily H member 2
Radix Glycyrrhizae (RG)	MOL004966	3'-Hydroxy-4'-O-Methylglabridin	Estrogen receptor
Radix Glycyrrhizae (RG)	MOL004966	3'-Hydroxy-4'-O-Methylglabridin	Androgen receptor
Radix Glycyrrhizae (RG)	MOL004966	3'-Hydroxy-4'-O-Methylglabridin	Sodium channel protein type 5 subunit alpha
Radix Glycyrrhizae (RG)	MOL004966	3'-Hydroxy-4'-O-Methylglabridin	Peroxisome proliferator activated receptor gamma
Radix Glycyrrhizae (RG)	MOL004966	3'-Hydroxy-4'-O-Methylglabridin	Coagulation factor Xa
Radix Glycyrrhizae (RG)	MOL004966	3'-Hydroxy-4'-O-Methylglabridin	Prostaglandin G/H synthase 2
Radix Glycyrrhizae (RG)	MOL004966	3'-Hydroxy-4'-O-Methylglabridin	Coagulation factor VII
Radix Glycyrrhizae (RG)	MOL004966	3'-Hydroxy-4'-O-Methylglabridin	Vascular endothelial growth factor receptor 2
Radix Glycyrrhizae (RG)	MOL004966	3'-Hydroxy-4'-O-Methylglabridin	Alpha-1B adrenergic receptor
Radix Glycyrrhizae (RG)	MOL004966	3'-Hydroxy-4'-O-Methylglabridin	Beta-2 adrenergic receptor
Radix Glycyrrhizae (RG)	MOL004966	3'-Hydroxy-4'-O-Methylglabridin	DNA topoisomerase II
Radix Glycyrrhizae (RG)	MOL004966	3'-Hydroxy-4'-O-Methylglabridin	Estrogen receptor beta
Radix Glycyrrhizae (RG)	MOL004966	3'-Hydroxy-4'-O-Methylglabridin	Mitogen-activated protein kinase 14
Radix Glycyrrhizae (RG)	MOL004966	3'-Hydroxy-4'-O-Methylglabridin	Glycogen synthase kinase-3 beta
Radix Glycyrrhizae (RG)	MOL004966	3'-Hydroxy-4'-O-Methylglabridin	Heat shock protein HSP 90
Radix Glycyrrhizae (RG)	MOL004966	3'-Hydroxy-4'-O-Methylglabridin	Cell division protein kinase 2
Radix Glycyrrhizae (RG)	MOL004966	3'-Hydroxy-4'-O-Methylglabridin	Serine/threonine-protein kinase Chk1
Radix Glycyrrhizae (RG)	MOL004966	3'-Hydroxy-4'-O-Methylglabridin	mRNA of PKA Catalytic Subunit C-alpha
Radix Glycyrrhizae (RG)	MOL004966	3'-Hydroxy-4'-O-Methylglabridin	Trypsin-1
Radix Glycyrrhizae (RG)	MOL004966	3'-Hydroxy-4'-O-Methylglabridin	Proto-oncogene serine/threonine-protein kinase Pim-1
Radix Glycyrrhizae (RG)	MOL004966	3'-Hydroxy-4'-O-Methylglabridin	Cyclin-A2

Radix Glycyrrhizae (RG)	MOL004966	3'-Hydroxy-4'-O-Methylglabridin	Nuclear receptor coactivator 2
Radix Glycyrrhizae (RG)	MOL004966	3'-Hydroxy-4'-O-Methylglabridin	Nuclear receptor coactivator 1
Radix Glycyrrhizae (RG)	MOL004966	3'-Hydroxy-4'-O-Methylglabridin	Calcium-activated potassium channel subunit alpha 1
Radix Glycyrrhizae (RG)	MOL004966	3'-Hydroxy-4'-O-Methylglabridin	Calmodulin
Radix Glycyrrhizae (RG)	MOL000497	licochalcone a	Nitric oxide synthase, inducible
Radix Glycyrrhizae (RG)	MOL000497	licochalcone a	Prostaglandin G/H synthase 1
Radix Glycyrrhizae (RG)	MOL000497	licochalcone a	Muscarinic acetylcholine receptor M1
Radix Glycyrrhizae (RG)	MOL000497	licochalcone a	Estrogen receptor
Radix Glycyrrhizae (RG)	MOL000497	licochalcone a	Androgen receptor
Radix Glycyrrhizae (RG)	MOL000497	licochalcone a	Sodium channel protein type 5 subunit alpha
Radix Glycyrrhizae (RG)	MOL000497	licochalcone a	Peroxisome proliferator activated receptor gamma
Radix Glycyrrhizae (RG)	MOL000497	licochalcone a	Coagulation factor Xa
Radix Glycyrrhizae (RG)	MOL000497	licochalcone a	Prostaglandin G/H synthase 2
Radix Glycyrrhizae (RG)	MOL000497	licochalcone a	Carbonic anhydrase II
Radix Glycyrrhizae (RG)	MOL000497	licochalcone a	Alpha-1B adrenergic receptor
Radix Glycyrrhizae (RG)	MOL000497	licochalcone a	Sodium-dependent dopamine
Radix Glycyrrhizae (RG)	MOL000497	licochalcone a	Estrogen receptor beta
Radix Glycyrrhizae (RG)	MOL000497	licochalcone a	Mitogen-activated protein kinase 14
Radix Glycyrrhizae (RG)	MOL000497	licochalcone a	Glycogen synthase kinase-3 beta
Radix Glycyrrhizae (RG)	MOL000497	licochalcone a	Heat shock protein HSP 90
Radix Glycyrrhizae (RG)	MOL000497	licochalcone a	Cell division protein kinase 2
Radix Glycyrrhizae (RG)	MOL000497	licochalcone a	Serine/threonine-protein kinase Chk1
Radix Glycyrrhizae (RG)	MOL000497	licochalcone a	Proto-oncogene serine/threonine-protein kinase Pim-1
Radix Glycyrrhizae (RG)	MOL000497	licochalcone a	Cyclin-A2
Radix Glycyrrhizae (RG)	MOL000497	licochalcone a	Calmodulin
Radix Glycyrrhizae (RG)	MOL000497	licochalcone a	Beta-2 adrenergic receptor
Radix Glycyrrhizae (RG)	MOL000497	licochalcone a	Nuclear receptor coactivator 2
Radix Glycyrrhizae (RG)	MOL000497	licochalcone a	Transcription factor p65
Radix Glycyrrhizae (RG)	MOL000497	licochalcone a	Signal transducer and activator of transcription 3
Radix Glycyrrhizae (RG)	MOL000497	licochalcone a	G1/S-specific cyclin-D1
Radix Glycyrrhizae (RG)	MOL000497	licochalcone a	Apoptosis regulator Bcl-2
Radix Glycyrrhizae (RG)	MOL000497	licochalcone a	Eukaryotic translation initiation factor 6
Radix Glycyrrhizae (RG)	MOL000497	licochalcone a	Mitogen-activated protein kinase 1
Radix Glycyrrhizae (RG)	MOL000497	licochalcone a	Retinoblastoma-associated protein
Radix Glycyrrhizae (RG)	MOL000497	licochalcone a	Cell division protein kinase 4
Radix Glycyrrhizae (RG)	MOL000497	licochalcone a	Fos-related antigen 2
Radix Glycyrrhizae (RG)	MOL004974	3'-Methoxyglabridin	Nitric oxide synthase, inducible
Radix Glycyrrhizae (RG)	MOL004974	3'-Methoxyglabridin	Prostaglandin G/H synthase 1
Radix Glycyrrhizae (RG)	MOL004974	3'-Methoxyglabridin	Potassium voltage-gated channel subfamily H member 2
Radix Glycyrrhizae (RG)	MOL004974	3'-Methoxyglabridin	Estrogen receptor
Radix Glycyrrhizae (RG)	MOL004974	3'-Methoxyglabridin	Androgen receptor
Radix Glycyrrhizae (RG)	MOL004974	3'-Methoxyglabridin	Sodium channel protein type 5 subunit alpha
Radix Glycyrrhizae (RG)	MOL004974	3'-Methoxyglabridin	Peroxisome proliferator activated receptor gamma
Radix Glycyrrhizae (RG)	MOL004974	3'-Methoxyglabridin	Coagulation factor Xa
Radix Glycyrrhizae (RG)	MOL004974	3'-Methoxyglabridin	Prostaglandin G/H synthase 2
Radix Glycyrrhizae (RG)	MOL004974	3'-Methoxyglabridin	Coagulation factor VII
Radix Glycyrrhizae (RG)	MOL004974	3'-Methoxyglabridin	Retinoic acid receptor RXR-alpha
Radix Glycyrrhizae (RG)	MOL004974	3'-Methoxyglabridin	Acetylcholinesterase
Radix Glycyrrhizae (RG)	MOL004974	3'-Methoxyglabridin	Alpha-1B adrenergic receptor
Radix Glycyrrhizae (RG)	MOL004974	3'-Methoxyglabridin	Beta-2 adrenergic receptor
Radix Glycyrrhizae (RG)	MOL004974	3'-Methoxyglabridin	DNA topoisomerase II
Radix Glycyrrhizae (RG)	MOL004974	3'-Methoxyglabridin	Estrogen receptor beta

Radix Glycyrrhizae (RG)	MOL004974	3'-Methoxyglabridin	Mitogen-activated protein kinase 14
Radix Glycyrrhizae (RG)	MOL004974	3'-Methoxyglabridin	Glycogen synthase kinase-3 beta
Radix Glycyrrhizae (RG)	MOL004974	3'-Methoxyglabridin	Heat shock protein HSP 90
Radix Glycyrrhizae (RG)	MOL004974	3'-Methoxyglabridin	Cell division protein kinase 2
Radix Glycyrrhizae (RG)	MOL004974	3'-Methoxyglabridin	Serine/threonine-protein kinase Chk1
Radix Glycyrrhizae (RG)	MOL004974	3'-Methoxyglabridin	Trypsin-1
Radix Glycyrrhizae (RG)	MOL004974	3'-Methoxyglabridin	Proto-oncogene serine/threonine-protein kinase Pim-1
Radix Glycyrrhizae (RG)	MOL004974	3'-Methoxyglabridin	Cyclin-A2
Radix Glycyrrhizae (RG)	MOL004974	3'-Methoxyglabridin	Nuclear receptor coactivator 2
Radix Glycyrrhizae (RG)	MOL004974	3'-Methoxyglabridin	Nuclear receptor coactivator 1
Radix Glycyrrhizae (RG)	MOL004974	3'-Methoxyglabridin	Calcium-activated potassium channel subunit alpha 1
Radix Glycyrrhizae (RG)	MOL004974	3'-Methoxyglabridin	Calmodulin
Radix Glycyrrhizae (RG)	MOL004978	2-[(3R)-8,8-dimethyl-3,4-dihydro-2H-pyrano[6,5-f]chromen-3-yl]-5-methoxyphenol	Nitric oxide synthase, inducible
Radix Glycyrrhizae (RG)	MOL004978	2-[(3R)-8,8-dimethyl-3,4-dihydro-2H-pyrano[6,5-f]chromen-3-yl]-5-methoxyphenol	Prostaglandin G/H synthase 1
Radix Glycyrrhizae (RG)	MOL004978	2-[(3R)-8,8-dimethyl-3,4-dihydro-2H-pyrano[6,5-f]chromen-3-yl]-5-methoxyphenol	Muscarinic acetylcholine receptor M3
Radix Glycyrrhizae (RG)	MOL004978	2-[(3R)-8,8-dimethyl-3,4-dihydro-2H-pyrano[6,5-f]chromen-3-yl]-5-methoxyphenol	Potassium voltage-gated channel subfamily H member 2
Radix Glycyrrhizae (RG)	MOL004978	2-[(3R)-8,8-dimethyl-3,4-dihydro-2H-pyrano[6,5-f]chromen-3-yl]-5-methoxyphenol	Muscarinic acetylcholine receptor M1
Radix Glycyrrhizae (RG)	MOL004978	2-[(3R)-8,8-dimethyl-3,4-dihydro-2H-pyrano[6,5-f]chromen-3-yl]-5-methoxyphenol	Estrogen receptor
Radix Glycyrrhizae (RG)	MOL004978	2-[(3R)-8,8-dimethyl-3,4-dihydro-2H-pyrano[6,5-f]chromen-3-yl]-5-methoxyphenol	Androgen receptor
Radix Glycyrrhizae (RG)	MOL004978	2-[(3R)-8,8-dimethyl-3,4-dihydro-2H-pyrano[6,5-f]chromen-3-yl]-5-methoxyphenol	Sodium channel protein type 5 subunit alpha
Radix Glycyrrhizae (RG)	MOL004978	2-[(3R)-8,8-dimethyl-3,4-dihydro-2H-pyrano[6,5-f]chromen-3-yl]-5-methoxyphenol	Peroxisome proliferator activated receptor gamma
Radix Glycyrrhizae (RG)	MOL004978	2-[(3R)-8,8-dimethyl-3,4-dihydro-2H-pyrano[6,5-f]chromen-3-yl]-5-methoxyphenol	Coagulation factor Xa
Radix Glycyrrhizae (RG)	MOL004978	2-[(3R)-8,8-dimethyl-3,4-dihydro-2H-pyrano[6,5-f]chromen-3-yl]-5-methoxyphenol	Prostaglandin G/H synthase 2
Radix Glycyrrhizae (RG)	MOL004978	2-[(3R)-8,8-dimethyl-3,4-dihydro-2H-pyrano[6,5-f]chromen-3-yl]-5-methoxyphenol	Nitric-oxide synthase, endothelial

Radix Glycyrrhizae (RG)	MOL004978	2-[(3R)-8,8-dimethyl-3,4-dihydro-2H-pyrano[6,5-f]chromen-3-yl]-5-methoxyphenol	Retinoic acid receptor RXR-alpha
Radix Glycyrrhizae (RG)	MOL004978	2-[(3R)-8,8-dimethyl-3,4-dihydro-2H-pyrano[6,5-f]chromen-3-yl]-5-methoxyphenol	Acetylcholinesterase
Radix Glycyrrhizae (RG)	MOL004978	2-[(3R)-8,8-dimethyl-3,4-dihydro-2H-pyrano[6,5-f]chromen-3-yl]-5-methoxyphenol	Alpha-1B adrenergic receptor
Radix Glycyrrhizae (RG)	MOL004978	2-[(3R)-8,8-dimethyl-3,4-dihydro-2H-pyrano[6,5-f]chromen-3-yl]-5-methoxyphenol	Sodium-dependent dopamine transporter
Radix Glycyrrhizae (RG)	MOL004978	2-[(3R)-8,8-dimethyl-3,4-dihydro-2H-pyrano[6,5-f]chromen-3-yl]-5-methoxyphenol	Beta-2 adrenergic receptor
Radix Glycyrrhizae (RG)	MOL004978	2-[(3R)-8,8-dimethyl-3,4-dihydro-2H-pyrano[6,5-f]chromen-3-yl]-5-methoxyphenol	Estrogen receptor beta
Radix Glycyrrhizae (RG)	MOL004978	2-[(3R)-8,8-dimethyl-3,4-dihydro-2H-pyrano[6,5-f]chromen-3-yl]-5-methoxyphenol	Mitogen-activated protein kinase 14
Radix Glycyrrhizae (RG)	MOL004978	2-[(3R)-8,8-dimethyl-3,4-dihydro-2H-pyrano[6,5-f]chromen-3-yl]-5-methoxyphenol	Glycogen synthase kinase-3 beta
Radix Glycyrrhizae (RG)	MOL004978	2-[(3R)-8,8-dimethyl-3,4-dihydro-2H-pyrano[6,5-f]chromen-3-yl]-5-methoxyphenol	Cell division protein kinase 2
Radix Glycyrrhizae (RG)	MOL004978	2-[(3R)-8,8-dimethyl-3,4-dihydro-2H-pyrano[6,5-f]chromen-3-yl]-5-methoxyphenol	Serine/threonine-protein kinase Chk1
Radix Glycyrrhizae (RG)	MOL004978	2-[(3R)-8,8-dimethyl-3,4-dihydro-2H-pyrano[6,5-f]chromen-3-yl]-5-methoxyphenol	mRNA of PKA Catalytic Subunit C-alpha
Radix Glycyrrhizae (RG)	MOL004978	2-[(3R)-8,8-dimethyl-3,4-dihydro-2H-pyrano[6,5-f]chromen-3-yl]-5-methoxyphenol	Retinoic acid receptor RXR-beta
Radix Glycyrrhizae (RG)	MOL004978	2-[(3R)-8,8-dimethyl-3,4-dihydro-2H-pyrano[6,5-f]chromen-3-yl]-5-methoxyphenol	Trypsin-1
Radix Glycyrrhizae (RG)	MOL004978	2-[(3R)-8,8-dimethyl-3,4-dihydro-2H-pyrano[6,5-f]chromen-3-yl]-5-methoxyphenol	Proto-oncogene serine/threonine-protein kinase Pim-1
Radix Glycyrrhizae (RG)	MOL004978	2-[(3R)-8,8-dimethyl-3,4-dihydro-2H-pyrano[6,5-f]chromen-3-yl]-5-methoxyphenol	Cyclin-A2

Radix Glycyrrhizae (RG)	MOL004978	2-[(3R)-8,8-dimethyl-3,4-dihydro-2H-pyrano[6,5-f]chromen-3-yl]-5-methoxyphenol	Nuclear receptor coactivator 2
Radix Glycyrrhizae (RG)	MOL004978	2-[(3R)-8,8-dimethyl-3,4-dihydro-2H-pyrano[6,5-f]chromen-3-yl]-5-methoxyphenol	Nuclear receptor coactivator 1
Radix Glycyrrhizae (RG)	MOL004978	2-[(3R)-8,8-dimethyl-3,4-dihydro-2H-pyrano[6,5-f]chromen-3-yl]-5-methoxyphenol	Calcium-activated potassium channel subunit alpha 1
Radix Glycyrrhizae (RG)	MOL004978	2-[(3R)-8,8-dimethyl-3,4-dihydro-2H-pyrano[6,5-f]chromen-3-yl]-5-methoxyphenol	Calmodulin
Radix Glycyrrhizae (RG)	MOL004980	Inflacoumarin A	Thrombin
Radix Glycyrrhizae (RG)	MOL004980	Inflacoumarin A	Estrogen receptor
Radix Glycyrrhizae (RG)	MOL004980	Inflacoumarin A	Androgen receptor
Radix Glycyrrhizae (RG)	MOL004980	Inflacoumarin A	Peroxisome proliferator activated receptor gamma
Radix Glycyrrhizae (RG)	MOL004980	Inflacoumarin A	Coagulation factor Xa
Radix Glycyrrhizae (RG)	MOL004980	Inflacoumarin A	Prostaglandin G/H synthase 2
Radix Glycyrrhizae (RG)	MOL004980	Inflacoumarin A	Beta-2 adrenergic receptor
Radix Glycyrrhizae (RG)	MOL004980	Inflacoumarin A	Dipeptidyl peptidase IV
Radix Glycyrrhizae (RG)	MOL004980	Inflacoumarin A	Heat shock protein HSP 90
Radix Glycyrrhizae (RG)	MOL004980	Inflacoumarin A	Trypsin-1
Radix Glycyrrhizae (RG)	MOL004980	Inflacoumarin A	Proto-oncogene serine/threonine-protein kinase Pim-1
Radix Glycyrrhizae (RG)	MOL004980	Inflacoumarin A	Nuclear receptor coactivator 2
Radix Glycyrrhizae (RG)	MOL004980	Inflacoumarin A	Calmodulin
Radix Glycyrrhizae (RG)	MOL004980	Inflacoumarin A	Prostaglandin G/H synthase 1
Radix Glycyrrhizae (RG)	MOL004980	Inflacoumarin A	Sodium channel protein type 5 subunit alpha
Radix Glycyrrhizae (RG)	MOL004985	icos-5-enoic acid	Nuclear receptor coactivator 2
Radix Glycyrrhizae (RG)	MOL004988	Kanzonol F	Estrogen receptor
Radix Glycyrrhizae (RG)	MOL004988	Kanzonol F	Androgen receptor
Radix Glycyrrhizae (RG)	MOL004988	Kanzonol F	Coagulation factor Xa
Radix Glycyrrhizae (RG)	MOL004988	Kanzonol F	Prostaglandin G/H synthase 2
Radix Glycyrrhizae (RG)	MOL004988	Kanzonol F	Estrogen receptor beta
Radix Glycyrrhizae (RG)	MOL004988	Kanzonol F	Proto-oncogene serine/threonine-protein kinase Pim-1
Radix Glycyrrhizae (RG)	MOL004988	Kanzonol F	Nuclear receptor coactivator 2
Radix Glycyrrhizae (RG)	MOL004988	Kanzonol F	Calmodulin
Radix Glycyrrhizae (RG)	MOL004989	6-prenylated eriodictyol	Nitric oxide synthase, inducible
Radix Glycyrrhizae (RG)	MOL004989	6-prenylated eriodictyol	Estrogen receptor
Radix Glycyrrhizae (RG)	MOL004989	6-prenylated eriodictyol	Sodium channel protein type 5 subunit alpha
Radix Glycyrrhizae (RG)	MOL004989	6-prenylated eriodictyol	Coagulation factor Xa
Radix Glycyrrhizae (RG)	MOL004989	6-prenylated eriodictyol	Prostaglandin G/H synthase 2
Radix Glycyrrhizae (RG)	MOL004989	6-prenylated eriodictyol	Coagulation factor VII
Radix Glycyrrhizae (RG)	MOL004989	6-prenylated eriodictyol	Heat shock protein HSP 90
Radix Glycyrrhizae (RG)	MOL004989	6-prenylated eriodictyol	Calmodulin
Radix Glycyrrhizae (RG)	MOL004990	7,2',4'-trihydroxy − 5-methoxy-3 − arylcoumarin	Nitric oxide synthase, inducible
Radix Glycyrrhizae (RG)	MOL004990	7,2',4'-trihydroxy − 5-methoxy-3 − arylcoumarin	Prostaglandin G/H synthase 1
Radix Glycyrrhizae (RG)	MOL004990	7,2',4'-trihydroxy − 5-methoxy-3 − arylcoumarin	Estrogen receptor
Radix Glycyrrhizae (RG)	MOL004990	7,2',4'-trihydroxy − 5-methoxy-3 − arylcoumarin	Androgen receptor
Radix Glycyrrhizae (RG)	MOL004990	7,2',4'-trihydroxy − 5-methoxy-3 − arylcoumarin	Peroxisome proliferator activated receptor gamma

Radix Glycyrrhizae (RG)	MOL004990	7,2',4'-trihydroxy − 5-methoxy-3 − arylcoumarin	Prostaglandin G/H synthase 2
Radix Glycyrrhizae (RG)	MOL004990	7,2',4'-trihydroxy − 5-methoxy-3 − arylcoumarin	Estrogen receptor beta
Radix Glycyrrhizae (RG)	MOL004990	7,2',4'-trihydroxy − 5-methoxy-3 − arylcoumarin	Dipeptidyl peptidase IV
Radix Glycyrrhizae (RG)	MOL004990	7,2',4'-trihydroxy − 5-methoxy-3 − arylcoumarin	Mitogen-activated protein kinase 14
Radix Glycyrrhizae (RG)	MOL004990	7,2',4'-trihydroxy − 5-methoxy-3 − arylcoumarin	Glycogen synthase kinase-3 beta
Radix Glycyrrhizae (RG)	MOL004990	7,2',4'-trihydroxy − 5-methoxy-3 − arylcoumarin	Heat shock protein HSP 90
Radix Glycyrrhizae (RG)	MOL004990	7,2',4'-trihydroxy − 5-methoxy-3 − arylcoumarin	Cell division protein kinase 2
Radix Glycyrrhizae (RG)	MOL004990	7,2',4'-trihydroxy − 5-methoxy-3 − arylcoumarin	Serine/threonine-protein kinase Chk1
Radix Glycyrrhizae (RG)	MOL004990	7,2',4'-trihydroxy − 5-methoxy-3 − arylcoumarin	mRNA of PKA Catalytic Subunit C-alpha
Radix Glycyrrhizae (RG)	MOL004990	7,2',4'-trihydroxy − 5-methoxy-3 − arylcoumarin	Proto-oncogene serine/threonine-protein kinase Pim-1
Radix Glycyrrhizae (RG)	MOL004991	7-Acetoxy-2-methylisoflavone	Nitric oxide synthase, inducible
Radix Glycyrrhizae (RG)	MOL004991	7-Acetoxy-2-methylisoflavone	Prostaglandin G/H synthase 1
Radix Glycyrrhizae (RG)	MOL004991	7-Acetoxy-2-methylisoflavone	Thrombin
Radix Glycyrrhizae (RG)	MOL004991	7-Acetoxy-2-methylisoflavone	Estrogen receptor
Radix Glycyrrhizae (RG)	MOL004991	7-Acetoxy-2-methylisoflavone	Androgen receptor
Radix Glycyrrhizae (RG)	MOL004991	7-Acetoxy-2-methylisoflavone	Sodium channel protein type 5 subunit alpha
Radix Glycyrrhizae (RG)	MOL004991	7-Acetoxy-2-methylisoflavone	Peroxisome proliferator activated receptor gamma
Radix Glycyrrhizae (RG)	MOL004991	7-Acetoxy-2-methylisoflavone	Prostaglandin G/H synthase 2
Radix Glycyrrhizae (RG)	MOL004991	7-Acetoxy-2-methylisoflavone	Nitric-oxide synthase, endothelial
Radix Glycyrrhizae (RG)	MOL004991	7-Acetoxy-2-methylisoflavone	Retinoic acid receptor RXR-alpha
Radix Glycyrrhizae (RG)	MOL004991	7-Acetoxy-2-methylisoflavone	Acetylcholinesterase
Radix Glycyrrhizae (RG)	MOL004991	7-Acetoxy-2-methylisoflavone	CGMP-inhibited 3',5'-cyclic phosphodiesterase A
Radix Glycyrrhizae (RG)	MOL004991	7-Acetoxy-2-methylisoflavone	Alpha-1B adrenergic receptor
Radix Glycyrrhizae (RG)	MOL004991	7-Acetoxy-2-methylisoflavone	Beta-2 adrenergic receptor
Radix Glycyrrhizae (RG)	MOL004991	7-Acetoxy-2-methylisoflavone	Alpha-1D adrenergic receptor
Radix Glycyrrhizae (RG)	MOL004991	7-Acetoxy-2-methylisoflavone	Gamma-aminobutyric acid receptor subunit alpha-1
Radix Glycyrrhizae (RG)	MOL004991	7-Acetoxy-2-methylisoflavone	Dipeptidyl peptidase IV
Radix Glycyrrhizae (RG)	MOL004991	7-Acetoxy-2-methylisoflavone	Mitogen-activated protein kinase 14
Radix Glycyrrhizae (RG)	MOL004991	7-Acetoxy-2-methylisoflavone	Glycogen synthase kinase-3 beta
Radix Glycyrrhizae (RG)	MOL004991	7-Acetoxy-2-methylisoflavone	Heat shock protein HSP 90
Radix Glycyrrhizae (RG)	MOL004991	7-Acetoxy-2-methylisoflavone	Cell division protein kinase 2
Radix Glycyrrhizae (RG)	MOL004991	7-Acetoxy-2-methylisoflavone	Serine/threonine-protein kinase Chk1
Radix Glycyrrhizae (RG)	MOL004991	7-Acetoxy-2-methylisoflavone	Trypsin-1
Radix Glycyrrhizae (RG)	MOL004991	7-Acetoxy-2-methylisoflavone	Nuclear receptor coactivator 2
Radix Glycyrrhizae (RG)	MOL004991	7-Acetoxy-2-methylisoflavone	Calmodulin
Radix Glycyrrhizae (RG)	MOL004993	8-prenylated eriodictyol	Estrogen receptor
Radix Glycyrrhizae (RG)	MOL004993	8-prenylated eriodictyol	Sodium channel protein type 5 subunit alpha
Radix Glycyrrhizae (RG)	MOL004993	8-prenylated eriodictyol	Coagulation factor Xa
Radix Glycyrrhizae (RG)	MOL004993	8-prenylated eriodictyol	Prostaglandin G/H synthase 2
Radix Glycyrrhizae (RG)	MOL004993	8-prenylated eriodictyol	Coagulation factor VII
Radix Glycyrrhizae (RG)	MOL004993	8-prenylated eriodictyol	Heat shock protein HSP 90
Radix Glycyrrhizae (RG)	MOL004993	8-prenylated eriodictyol	Nuclear receptor coactivator 1
Radix Glycyrrhizae (RG)	MOL004993	8-prenylated eriodictyol	Calmodulin
Radix Glycyrrhizae (RG)	MOL004996	gadelaidic acid	Nuclear receptor coactivator 2
Radix Glycyrrhizae (RG)	MOL000500	Vestitol	Nitric oxide synthase, inducible
Radix Glycyrrhizae (RG)	MOL000500	Vestitol	Prostaglandin G/H synthase 1
Radix Glycyrrhizae (RG)	MOL000500	Vestitol	Muscarinic acetylcholine receptor M1
Radix Glycyrrhizae (RG)	MOL000500	Vestitol	Estrogen receptor

Radix Glycyrrhizae (RG)	MOL000500	Vestitol	Androgen receptor
Radix Glycyrrhizae (RG)	MOL000500	Vestitol	Sodium channel protein type 5 subunit alpha
Radix Glycyrrhizae (RG)	MOL000500	Vestitol	Peroxisome proliferator activated receptor gamma
Radix Glycyrrhizae (RG)	MOL000500	Vestitol	Prostaglandin G/H synthase 2
Radix Glycyrrhizae (RG)	MOL000500	Vestitol	Muscarinic acetylcholine receptor M4
Radix Glycyrrhizae (RG)	MOL000500	Vestitol	Retinoic acid receptor RXR-alpha
Radix Glycyrrhizae (RG)	MOL000500	Vestitol	CGMP-inhibited 3',5'-cyclic phosphodiesterase A
Radix Glycyrrhizae (RG)	MOL000500	Vestitol	5-hydroxytryptamine 2A receptor
Radix Glycyrrhizae (RG)	MOL000500	Vestitol	Alpha-1A adrenergic receptor
Radix Glycyrrhizae (RG)	MOL000500	Vestitol	Alpha-1B adrenergic receptor
Radix Glycyrrhizae (RG)	MOL000500	Vestitol	Sodium-dependent dopamine
Radix Glycyrrhizae (RG)	MOL000500	Vestitol	Beta-2 adrenergic receptor
Radix Glycyrrhizae (RG)	MOL000500	Vestitol	Sodium-dependent serotonin transporter
Radix Glycyrrhizae (RG)	MOL000500	Vestitol	Estrogen receptor beta
Radix Glycyrrhizae (RG)	MOL000500	Vestitol	Dipeptidyl peptidase IV
Radix Glycyrrhizae (RG)	MOL000500	Vestitol	Mitogen-activated protein kinase 14
Radix Glycyrrhizae (RG)	MOL000500	Vestitol	Glycogen synthase kinase-3 beta
Radix Glycyrrhizae (RG)	MOL000500	Vestitol	Heat shock protein HSP 90
Radix Glycyrrhizae (RG)	MOL000500	Vestitol	Cell division protein kinase 2
Radix Glycyrrhizae (RG)	MOL000500	Vestitol	Serine/threonine-protein kinase Chk1
Radix Glycyrrhizae (RG)	MOL000500	Vestitol	mRNA of PKA Catalytic Subunit C-
Radix Glycyrrhizae (RG)	MOL000500	Vestitol	Trypsin-1
Radix Glycyrrhizae (RG)	MOL000500	Vestitol	Proto-oncogene serine/threonine-protein kinase Pim-1
Radix Glycyrrhizae (RG)	MOL000500	Vestitol	Cyclin-A2
Radix Glycyrrhizae (RG)	MOL000500	Vestitol	cAMP-dependent protein kinase inhibitor alpha
Radix Glycyrrhizae (RG)	MOL000500	Vestitol	Calmodulin
Radix Glycyrrhizae (RG)	MOL005000	Gancaonin G	Nitric oxide synthase, inducible
Radix Glycyrrhizae (RG)	MOL005000	Gancaonin G	Thrombin
Radix Glycyrrhizae (RG)	MOL005000	Gancaonin G	Estrogen receptor
Radix Glycyrrhizae (RG)	MOL005000	Gancaonin G	Androgen receptor
Radix Glycyrrhizae (RG)	MOL005000	Gancaonin G	Peroxisome proliferator activated receptor gamma
Radix Glycyrrhizae (RG)	MOL005000	Gancaonin G	Coagulation factor Xa
Radix Glycyrrhizae (RG)	MOL005000	Gancaonin G	Prostaglandin G/H synthase 2
Radix Glycyrrhizae (RG)	MOL005000	Gancaonin G	Nitric-oxide synthase, endothelial
Radix Glycyrrhizae (RG)	MOL005000	Gancaonin G	DNA topoisomerase II
Radix Glycyrrhizae (RG)	MOL005000	Gancaonin G	Estrogen receptor beta
Radix Glycyrrhizae (RG)	MOL005000	Gancaonin G	Dipeptidyl peptidase IV
Radix Glycyrrhizae (RG)	MOL005000	Gancaonin G	Mitogen-activated protein kinase 14
Radix Glycyrrhizae (RG)	MOL005000	Gancaonin G	Glycogen synthase kinase-3 beta
Radix Glycyrrhizae (RG)	MOL005000	Gancaonin G	Heat shock protein HSP 90
Radix Glycyrrhizae (RG)	MOL005000	Gancaonin G	Serine/threonine-protein kinase Chk1
Radix Glycyrrhizae (RG)	MOL005000	Gancaonin G	Trypsin-1
Radix Glycyrrhizae (RG)	MOL005000	Gancaonin G	Proto-oncogene serine/threonine-protein kinase Pim-1
Radix Glycyrrhizae (RG)	MOL005000	Gancaonin G	Cyclin-A2
Radix Glycyrrhizae (RG)	MOL005000	Gancaonin G	Nuclear receptor coactivator 2
Radix Glycyrrhizae (RG)	MOL005000	Gancaonin G	Calmodulin
Radix Glycyrrhizae (RG)	MOL005001	Gancaonin H	Estrogen receptor
Radix Glycyrrhizae (RG)	MOL005001	Gancaonin H	Androgen receptor
Radix Glycyrrhizae (RG)	MOL005001	Gancaonin H	Coagulation factor Xa
Radix Glycyrrhizae (RG)	MOL005001	Gancaonin H	Prostaglandin G/H synthase 2
Radix Glycyrrhizae (RG)	MOL005001	Gancaonin H	Vascular endothelial growth factor receptor 2
Radix Glycyrrhizae (RG)	MOL005001	Gancaonin H	DNA topoisomerase II
Radix Glycyrrhizae (RG)	MOL005001	Gancaonin H	Heat shock protein HSP 90
Radix Glycyrrhizae (RG)	MOL005001	Gancaonin H	Trypsin-1

Radix Glycyrrhizae (RG)	MOL005001	Gancaonin H	Proto-oncogene serine/threonine-protein kinase Pim-1
Radix Glycyrrhizae (RG)	MOL005001	Gancaonin H	Cyclin-A2
Radix Glycyrrhizae (RG)	MOL005001	Gancaonin H	Nuclear receptor coactivator 2
Radix Glycyrrhizae (RG)	MOL005001	Gancaonin H	Calmodulin
Radix Glycyrrhizae (RG)	MOL005003	Licoagrocarpin	Nitric oxide synthase, inducible
Radix Glycyrrhizae (RG)	MOL005003	Licoagrocarpin	Prostaglandin G/H synthase 1
Radix Glycyrrhizae (RG)	MOL005003	Licoagrocarpin	Muscarinic acetylcholine receptor M3
Radix Glycyrrhizae (RG)	MOL005003	Licoagrocarpin	Thrombin
Radix Glycyrrhizae (RG)	MOL005003	Licoagrocarpin	Potassium voltage-gated channel subfamily H member 2
Radix Glycyrrhizae (RG)	MOL005003	Licoagrocarpin	Muscarinic acetylcholine receptor M1
Radix Glycyrrhizae (RG)	MOL005003	Licoagrocarpin	Estrogen receptor
Radix Glycyrrhizae (RG)	MOL005003	Licoagrocarpin	Androgen receptor
Radix Glycyrrhizae (RG)	MOL005003	Licoagrocarpin	Sodium channel protein type 5 subunit alpha
Radix Glycyrrhizae (RG)	MOL005003	Licoagrocarpin	Peroxisome proliferator activated receptor gamma
Radix Glycyrrhizae (RG)	MOL005003	Licoagrocarpin	Coagulation factor Xa
Radix Glycyrrhizae (RG)	MOL005003	Licoagrocarpin	Muscarinic acetylcholine receptor M5
Radix Glycyrrhizae (RG)	MOL005003	Licoagrocarpin	Prostaglandin G/H synthase 2
Radix Glycyrrhizae (RG)	MOL005003	Licoagrocarpin	Nitric-oxide synthase, endothelial
Radix Glycyrrhizae (RG)	MOL005003	Licoagrocarpin	Retinoic acid receptor RXR-alpha
Radix Glycyrrhizae (RG)	MOL005003	Licoagrocarpin	Acetylcholinesterase
Radix Glycyrrhizae (RG)	MOL005003	Licoagrocarpin	Alpha-1B adrenergic receptor
Radix Glycyrrhizae (RG)	MOL005003	Licoagrocarpin	Beta-2 adrenergic receptor
Radix Glycyrrhizae (RG)	MOL005003	Licoagrocarpin	Estrogen receptor beta
Radix Glycyrrhizae (RG)	MOL005003	Licoagrocarpin	Mitogen-activated protein kinase 14
Radix Glycyrrhizae (RG)	MOL005003	Licoagrocarpin	Glycogen synthase kinase-3 beta
Radix Glycyrrhizae (RG)	MOL005003	Licoagrocarpin	Heat shock protein HSP 90
Radix Glycyrrhizae (RG)	MOL005003	Licoagrocarpin	Cell division protein kinase 2
Radix Glycyrrhizae (RG)	MOL005003	Licoagrocarpin	Retinoic acid receptor RXR-beta
Radix Glycyrrhizae (RG)	MOL005003	Licoagrocarpin	Trypsin-1
Radix Glycyrrhizae (RG)	MOL005003	Licoagrocarpin	Proto-oncogene serine/threonine-protein kinase Pim-1
Radix Glycyrrhizae (RG)	MOL005003	Licoagrocarpin	Cyclin-A2
Radix Glycyrrhizae (RG)	MOL005003	Licoagrocarpin	Nuclear receptor coactivator 2
Radix Glycyrrhizae (RG)	MOL005003	Licoagrocarpin	Calmodulin
Radix Glycyrrhizae (RG)	MOL005007	Glyasperins M	Nitric oxide synthase, inducible
Radix Glycyrrhizae (RG)	MOL005007	Glyasperins M	Prostaglandin G/H synthase 1
Radix Glycyrrhizae (RG)	MOL005007	Glyasperins M	Potassium voltage-gated channel subfamily H member 2
Radix Glycyrrhizae (RG)	MOL005007	Glyasperins M	Estrogen receptor
Radix Glycyrrhizae (RG)	MOL005007	Glyasperins M	Androgen receptor
Radix Glycyrrhizae (RG)	MOL005007	Glyasperins M	Sodium channel protein type 5 subunit alpha
Radix Glycyrrhizae (RG)	MOL005007	Glyasperins M	Peroxisome proliferator activated receptor gamma
Radix Glycyrrhizae (RG)	MOL005007	Glyasperins M	Coagulation factor Xa
Radix Glycyrrhizae (RG)	MOL005007	Glyasperins M	Prostaglandin G/H synthase 2
Radix Glycyrrhizae (RG)	MOL005007	Glyasperins M	Coagulation factor VII
Radix Glycyrrhizae (RG)	MOL005007	Glyasperins M	Vascular endothelial growth factor receptor 2
Radix Glycyrrhizae (RG)	MOL005007	Glyasperins M	Acetylcholinesterase
Radix Glycyrrhizae (RG)	MOL005007	Glyasperins M	DNA topoisomerase II
Radix Glycyrrhizae (RG)	MOL005007	Glyasperins M	Estrogen receptor beta
Radix Glycyrrhizae (RG)	MOL005007	Glyasperins M	Peroxisome proliferator activated receptor delta
Radix Glycyrrhizae (RG)	MOL005007	Glyasperins M	Glycogen synthase kinase-3 beta
Radix Glycyrrhizae (RG)	MOL005007	Glyasperins M	Heat shock protein HSP 90
Radix Glycyrrhizae (RG)	MOL005007	Glyasperins M	Cell division protein kinase 2
Radix Glycyrrhizae (RG)	MOL005007	Glyasperins M	mRNA of PKA Catalytic Subunit C-
Radix Glycyrrhizae (RG)	MOL005007	Glyasperins M	Trypsin-1

Radix Glycyrrhizae (RG)	MOL005007	Glyasperins M	Proto-oncogene serine/threonine-protein kinase Pim-1
Radix Glycyrrhizae (RG)	MOL005007	Glyasperins M	Cyclin-A2
Radix Glycyrrhizae (RG)	MOL005007	Glyasperins M	Nuclear receptor coactivator 2
Radix Glycyrrhizae (RG)	MOL005007	Glyasperins M	Nuclear receptor coactivator 1
Radix Glycyrrhizae (RG)	MOL005007	Glyasperins M	Calcium-activated potassium channel subunit alpha 1
Radix Glycyrrhizae (RG)	MOL005007	Glyasperins M	Calmodulin
Radix Glycyrrhizae (RG)	MOL005008	Glycyrrhiza flavonol A	Nitric oxide synthase, inducible
Radix Glycyrrhizae (RG)	MOL005008	Glycyrrhiza flavonol A	Estrogen receptor
Radix Glycyrrhizae (RG)	MOL005008	Glycyrrhiza flavonol A	Androgen receptor
Radix Glycyrrhizae (RG)	MOL005008	Glycyrrhiza flavonol A	Coagulation factor Xa
Radix Glycyrrhizae (RG)	MOL005008	Glycyrrhiza flavonol A	Prostaglandin G/H synthase 2
Radix Glycyrrhizae (RG)	MOL005008	Glycyrrhiza flavonol A	Coagulation factor VII
Radix Glycyrrhizae (RG)	MOL005008	Glycyrrhiza flavonol A	Acetylcholinesterase
Radix Glycyrrhizae (RG)	MOL005008	Glycyrrhiza flavonol A	DNA topoisomerase II
Radix Glycyrrhizae (RG)	MOL005008	Glycyrrhiza flavonol A	Estrogen receptor beta
Radix Glycyrrhizae (RG)	MOL005008	Glycyrrhiza flavonol A	Dipeptidyl peptidase IV
Radix Glycyrrhizae (RG)	MOL005008	Glycyrrhiza flavonol A	Glycogen synthase kinase-3 beta
Radix Glycyrrhizae (RG)	MOL005008	Glycyrrhiza flavonol A	Heat shock protein HSP 90
Radix Glycyrrhizae (RG)	MOL005008	Glycyrrhiza flavonol A	Cell division protein kinase 2
Radix Glycyrrhizae (RG)	MOL005008	Glycyrrhiza flavonol A	Trypsin-1
Radix Glycyrrhizae (RG)	MOL005008	Glycyrrhiza flavonol A	Proto-oncogene serine/threonine-protein kinase Pim-1
Radix Glycyrrhizae (RG)	MOL005008	Glycyrrhiza flavonol A	Cyclin-A2
Radix Glycyrrhizae (RG)	MOL005008	Glycyrrhiza flavonol A	Calmodulin
Radix Glycyrrhizae (RG)	MOL005012	Licoagroisoflavone	Nitric oxide synthase, inducible
Radix Glycyrrhizae (RG)	MOL005012	Licoagroisoflavone	Thrombin
Radix Glycyrrhizae (RG)	MOL005012	Licoagroisoflavone	Estrogen receptor
Radix Glycyrrhizae (RG)	MOL005012	Licoagroisoflavone	Androgen receptor
Radix Glycyrrhizae (RG)	MOL005012	Licoagroisoflavone	Sodium channel protein type 5 subunit alpha
Radix Glycyrrhizae (RG)	MOL005012	Licoagroisoflavone	Peroxisome proliferator activated receptor gamma
Radix Glycyrrhizae (RG)	MOL005012	Licoagroisoflavone	Coagulation factor Xa
Radix Glycyrrhizae (RG)	MOL005012	Licoagroisoflavone	Prostaglandin G/H synthase 2
Radix Glycyrrhizae (RG)	MOL005012	Licoagroisoflavone	Estrogen receptor beta
Radix Glycyrrhizae (RG)	MOL005012	Licoagroisoflavone	Dipeptidyl peptidase IV
Radix Glycyrrhizae (RG)	MOL005012	Licoagroisoflavone	Mitogen-activated protein kinase 14
Radix Glycyrrhizae (RG)	MOL005012	Licoagroisoflavone	Glycogen synthase kinase-3 beta
Radix Glycyrrhizae (RG)	MOL005012	Licoagroisoflavone	Cell division protein kinase 2
Radix Glycyrrhizae (RG)	MOL005012	Licoagroisoflavone	Serine/threonine-protein kinase Chk1
Radix Glycyrrhizae (RG)	MOL005012	Licoagroisoflavone	Trypsin-1
Radix Glycyrrhizae (RG)	MOL005012	Licoagroisoflavone	Proto-oncogene serine/threonine-protein kinase Pim-1
Radix Glycyrrhizae (RG)	MOL005012	Licoagroisoflavone	Cyclin-A2
Radix Glycyrrhizae (RG)	MOL005012	Licoagroisoflavone	Calmodulin
Radix Glycyrrhizae (RG)	MOL005016	Odoratin	Nitric oxide synthase, inducible
Radix Glycyrrhizae (RG)	MOL005016	Odoratin	Prostaglandin G/H synthase 1
Radix Glycyrrhizae (RG)	MOL005016	Odoratin	Estrogen receptor
Radix Glycyrrhizae (RG)	MOL005016	Odoratin	Androgen receptor
Radix Glycyrrhizae (RG)	MOL005016	Odoratin	Sodium channel protein type 5 subunit alpha
Radix Glycyrrhizae (RG)	MOL005016	Odoratin	Peroxisome proliferator activated receptor gamma
Radix Glycyrrhizae (RG)	MOL005016	Odoratin	Prostaglandin G/H synthase 2
Radix Glycyrrhizae (RG)	MOL005016	Odoratin	Retinoic acid receptor RXR-alpha
Radix Glycyrrhizae (RG)	MOL005016	Odoratin	Estrogen receptor beta
Radix Glycyrrhizae (RG)	MOL005016	Odoratin	Dipeptidyl peptidase IV
Radix Glycyrrhizae (RG)	MOL005016	Odoratin	Mitogen-activated protein kinase 14
Radix Glycyrrhizae (RG)	MOL005016	Odoratin	Glycogen synthase kinase-3 beta
Radix Glycyrrhizae (RG)	MOL005016	Odoratin	Heat shock protein HSP 90
Radix Glycyrrhizae (RG)	MOL005016	Odoratin	Cell division protein kinase 2

Radix Glycyrrhizae (RG)	MOL005016	Odoratin	Serine/threonine-protein kinase Chk1
Radix Glycyrrhizae (RG)	MOL005016	Odoratin	Trypsin-1
Radix Glycyrrhizae (RG)	MOL005016	Odoratin	Proto-oncogene serine/threonine-protein kinase Pim-1
Radix Glycyrrhizae (RG)	MOL005016	Odoratin	Cyclin-A2
Radix Glycyrrhizae (RG)	MOL005016	Odoratin	Nuclear receptor coactivator 2
Radix Glycyrrhizae (RG)	MOL005016	Odoratin	Calmodulin
Radix Glycyrrhizae (RG)	MOL005017	Phaseol	Thrombin
Radix Glycyrrhizae (RG)	MOL005017	Phaseol	Estrogen receptor
Radix Glycyrrhizae (RG)	MOL005017	Phaseol	Androgen receptor
Radix Glycyrrhizae (RG)	MOL005017	Phaseol	Peroxisome proliferator activated receptor gamma
Radix Glycyrrhizae (RG)	MOL005017	Phaseol	Prostaglandin G/H synthase 2
Radix Glycyrrhizae (RG)	MOL005017	Phaseol	Vascular endothelial growth factor receptor 2
Radix Glycyrrhizae (RG)	MOL005017	Phaseol	Mitogen-activated protein kinase 14
Radix Glycyrrhizae (RG)	MOL005017	Phaseol	Glycogen synthase kinase-3 beta
Radix Glycyrrhizae (RG)	MOL005017	Phaseol	Heat shock protein HSP 90
Radix Glycyrrhizae (RG)	MOL005017	Phaseol	Cell division protein kinase 2
Radix Glycyrrhizae (RG)	MOL005017	Phaseol	Serine/threonine-protein kinase Chk1
Radix Glycyrrhizae (RG)	MOL005017	Phaseol	mRNA of PKA Catalytic Subunit C-
Radix Glycyrrhizae (RG)	MOL005017	Phaseol	Proto-oncogene serine/threonine-protein kinase Pim-1
Radix Glycyrrhizae (RG)	MOL005017	Phaseol	Cyclin-A2
Radix Glycyrrhizae (RG)	MOL005018	Xambioona	Nitric oxide synthase, inducible
Radix Glycyrrhizae (RG)	MOL005018	Xambioona	Estrogen receptor
Radix Glycyrrhizae (RG)	MOL005018	Xambioona	Coagulation factor Xa
Radix Glycyrrhizae (RG)	MOL005018	Xambioona	Prostaglandin G/H synthase 2
Radix Glycyrrhizae (RG)	MOL005018	Xambioona	Estrogen receptor beta
Radix Glycyrrhizae (RG)	MOL005018	Xambioona	Proto-oncogene serine/threonine-protein kinase Pim-1
Radix Glycyrrhizae (RG)	MOL005018	Xambioona	Nuclear receptor coactivator 2
Radix Glycyrrhizae (RG)	MOL005018	Xambioona	Calmodulin
Radix Glycyrrhizae (RG)	MOL005020	dehydroglyasperins C	Nitric oxide synthase, inducible
Radix Glycyrrhizae (RG)	MOL005020	dehydroglyasperins C	Estrogen receptor
Radix Glycyrrhizae (RG)	MOL005020	dehydroglyasperins C	Androgen receptor
Radix Glycyrrhizae (RG)	MOL005020	dehydroglyasperins C	Sodium channel protein type 5 subunit alpha
Radix Glycyrrhizae (RG)	MOL005020	dehydroglyasperins C	Peroxisome proliferator activated receptor gamma
Radix Glycyrrhizae (RG)	MOL005020	dehydroglyasperins C	Coagulation factor Xa
Radix Glycyrrhizae (RG)	MOL005020	dehydroglyasperins C	Prostaglandin G/H synthase 2
Radix Glycyrrhizae (RG)	MOL005020	dehydroglyasperins C	Beta-2 adrenergic receptor
Radix Glycyrrhizae (RG)	MOL005020	dehydroglyasperins C	Estrogen receptor beta
Radix Glycyrrhizae (RG)	MOL005020	dehydroglyasperins C	Mitogen-activated protein kinase 14
Radix Glycyrrhizae (RG)	MOL005020	dehydroglyasperins C	Heat shock protein HSP 90
Radix Glycyrrhizae (RG)	MOL005020	dehydroglyasperins C	Cell division protein kinase 2
Radix Glycyrrhizae (RG)	MOL005020	dehydroglyasperins C	Serine/threonine-protein kinase Chk1
Radix Glycyrrhizae (RG)	MOL005020	dehydroglyasperins C	Trypsin-1
Radix Glycyrrhizae (RG)	MOL005020	dehydroglyasperins C	Proto-oncogene serine/threonine-protein kinase Pim-1
Radix Glycyrrhizae (RG)	MOL005020	dehydroglyasperins C	Cyclin-A2
Radix Glycyrrhizae (RG)	MOL005020	dehydroglyasperins C	Nuclear receptor coactivator 2
Radix Glycyrrhizae (RG)	MOL005020	dehydroglyasperins C	Calmodulin
Radix Glycyrrhizae (RG)	MOL000098	quercetin	Prostaglandin G/H synthase 1
Radix Glycyrrhizae (RG)	MOL000098	quercetin	Androgen receptor
Radix Glycyrrhizae (RG)	MOL000098	quercetin	Peroxisome proliferator activated receptor gamma
Radix Glycyrrhizae (RG)	MOL000098	quercetin	Prostaglandin G/H synthase 2
Radix Glycyrrhizae (RG)	MOL000098	quercetin	Heat shock protein HSP 90
Radix Glycyrrhizae (RG)	MOL000098	quercetin	Phosphatidylinositol-4,5-bisphosphate 3-kinase catalytic subunit, gamma
Radix Glycyrrhizae (RG)	MOL000098	quercetin	Nuclear receptor coactivator 2

Radix Glycyrrhizae (RG)	MOL000098	quercetin	Dipeptidyl peptidase IV
Radix Glycyrrhizae (RG)	MOL000098	quercetin	Aldose reductase
Radix Glycyrrhizae (RG)	MOL000098	quercetin	Trypsin-1
Radix Glycyrrhizae (RG)	MOL000098	quercetin	DNA topoisomerase II
Radix Glycyrrhizae (RG)	MOL000098	quercetin	Thrombin
Radix Glycyrrhizae (RG)	MOL000098	quercetin	Potassium voltage-gated channel subfamily H member 2
Radix Glycyrrhizae (RG)	MOL000098	quercetin	Sodium channel protein type 5 subunit alpha
Radix Glycyrrhizae (RG)	MOL000098	quercetin	Coagulation factor Xa
Radix Glycyrrhizae (RG)	MOL000098	quercetin	Beta-2 adrenergic receptor
Radix Glycyrrhizae (RG)	MOL000098	quercetin	Stromelysin-1
Radix Glycyrrhizae (RG)	MOL000098	quercetin	mRNA of PKA Catalytic Subunit C-
Radix Glycyrrhizae (RG)	MOL000098	quercetin	Coagulation factor VII
Radix Glycyrrhizae (RG)	MOL000098	quercetin	Nitric-oxide synthase, endothelial
Radix Glycyrrhizae (RG)	MOL000098	quercetin	Retinoic acid receptor RXR-alpha
Radix Glycyrrhizae (RG)	MOL000098	quercetin	Acetylcholinesterase
Radix Glycyrrhizae (RG)	MOL000098	quercetin	Gamma-aminobutyric acid receptor subunit alpha-1
Radix Glycyrrhizae (RG)	MOL000098	quercetin	Amine oxidase [flavin-containing] B
Radix Glycyrrhizae (RG)	MOL000098	quercetin	Transcription factor p65
Radix Glycyrrhizae (RG)	MOL000098	quercetin	Epidermal growth factor receptor
Radix Glycyrrhizae (RG)	MOL000098	quercetin	RAC-alpha serine/threonine-protein kinase
Radix Glycyrrhizae (RG)	MOL000098	quercetin	Vascular endothelial growth factor A
Radix Glycyrrhizae (RG)	MOL000098	quercetin	G1/S-specific cyclin-D1
Radix Glycyrrhizae (RG)	MOL000098	quercetin	Apoptosis regulator Bcl-2
Radix Glycyrrhizae (RG)	MOL000098	quercetin	Bcl-2-like protein 1
Radix Glycyrrhizae (RG)	MOL000098	quercetin	Proto-oncogene c-Fos
Radix Glycyrrhizae (RG)	MOL000098	quercetin	Cyclin-dependent kinase inhibitor 1
Radix Glycyrrhizae (RG)	MOL000098	quercetin	Eukaryotic translation initiation factor 6
Radix Glycyrrhizae (RG)	MOL000098	quercetin	Apoptosis regulator BAX
Radix Glycyrrhizae (RG)	MOL000098	quercetin	Caspase-9
Radix Glycyrrhizae (RG)	MOL000098	quercetin	Urokinase-type plasminogen activator
Radix Glycyrrhizae (RG)	MOL000098	quercetin	72 kDa type IV collagenase
Radix Glycyrrhizae (RG)	MOL000098	quercetin	Matrix metalloproteinase-9
Radix Glycyrrhizae (RG)	MOL000098	quercetin	Mitogen-activated protein kinase 1
Radix Glycyrrhizae (RG)	MOL000098	quercetin	Interleukin-10
Radix Glycyrrhizae (RG)	MOL000098	quercetin	Pro-epidermal growth factor
Radix Glycyrrhizae (RG)	MOL000098	quercetin	Retinoblastoma-associated protein
Radix Glycyrrhizae (RG)	MOL000098	quercetin	Tumor necrosis factor
Radix Glycyrrhizae (RG)	MOL000098	quercetin	Transcription factor AP-1
Radix Glycyrrhizae (RG)	MOL000098	quercetin	Interleukin-6
Radix Glycyrrhizae (RG)	MOL000098	quercetin	Cyclin-dependent kinase inhibitor 2A, isoforms 1/2/3
Radix Glycyrrhizae (RG)	MOL000098	quercetin	Activator of 90 kDa heat shock protein ATPase homolog 1
Radix Glycyrrhizae (RG)	MOL000098	quercetin	Caspase-3
Radix Glycyrrhizae (RG)	MOL000098	quercetin	Cellular tumor antigen p53
Radix Glycyrrhizae (RG)	MOL000098	quercetin	ETS domain-containing protein Elk-1
Radix Glycyrrhizae (RG)	MOL000098	quercetin	NF-kappa-B inhibitor alpha
Radix Glycyrrhizae (RG)	MOL000098	quercetin	NADPH--cytochrome P450 reductase
Radix Glycyrrhizae (RG)	MOL000098	quercetin	Ornithine decarboxylase
Radix Glycyrrhizae (RG)	MOL000098	quercetin	Xanthine dehydrogenase/oxidase
Radix Glycyrrhizae (RG)	MOL000098	quercetin	Caspase-8
Radix Glycyrrhizae (RG)	MOL000098	quercetin	DNA topoisomerase 1
Radix Glycyrrhizae (RG)	MOL000098	quercetin	RAF proto-oncogene serine/threonine-protein kinase
Radix Glycyrrhizae (RG)	MOL000098	quercetin	Superoxide dismutase [Cu-Zn]
Radix Glycyrrhizae (RG)	MOL000098	quercetin	Protein kinase C alpha type
Radix Glycyrrhizae (RG)	MOL000098	quercetin	Interstitial collagenase
Radix Glycyrrhizae (RG)	MOL000098	quercetin	Hypoxia-inducible factor 1-alpha

Radix Glycyrrhizae (RG)	MOL000098	quercetin	Signal transducer and activator of transcription 1-alpha/beta
Radix Glycyrrhizae (RG)	MOL000098	quercetin	Protein CBFA2T1
Radix Glycyrrhizae (RG)	MOL000098	quercetin	Probable E3 ubiquitin-protein ligase HERC5
Radix Glycyrrhizae (RG)	MOL000098	quercetin	Cell division control protein 2 homolog
Radix Glycyrrhizae (RG)	MOL000098	quercetin	78 kDa glucose-regulated protein
Radix Glycyrrhizae (RG)	MOL000098	quercetin	Receptor tyrosine-protein kinase erbB-2
Radix Glycyrrhizae (RG)	MOL000098	quercetin	Peroxisome proliferator-activated receptor gamma
Radix Glycyrrhizae (RG)	MOL000098	quercetin	Acetyl-CoA carboxylase 1
Radix Glycyrrhizae (RG)	MOL000098	quercetin	Heme oxygenase 1
Radix Glycyrrhizae (RG)	MOL000098	quercetin	Cytochrome P450 3A4
Radix Glycyrrhizae (RG)	MOL000098	quercetin	Cytochrome P450 1A2
Radix Glycyrrhizae (RG)	MOL000098	quercetin	Caveolin-1
Radix Glycyrrhizae (RG)	MOL000098	quercetin	Myc proto-oncogene protein
Radix Glycyrrhizae (RG)	MOL000098	quercetin	Tissue factor
Radix Glycyrrhizae (RG)	MOL000098	quercetin	Gap junction alpha-1 protein
Radix Glycyrrhizae (RG)	MOL000098	quercetin	Cytochrome P450 1A1
Radix Glycyrrhizae (RG)	MOL000098	quercetin	Intercellular adhesion molecule 1
Radix Glycyrrhizae (RG)	MOL000098	quercetin	Interleukin-1 beta
Radix Glycyrrhizae (RG)	MOL000098	quercetin	C-C motif chemokine 2
Radix Glycyrrhizae (RG)	MOL000098	quercetin	E-selectin
Radix Glycyrrhizae (RG)	MOL000098	quercetin	Vascular cell adhesion protein 1
Radix Glycyrrhizae (RG)	MOL000098	quercetin	Prostaglandin E2 receptor EP3 subtype
Radix Glycyrrhizae (RG)	MOL000098	quercetin	Interleukin-8
Radix Glycyrrhizae (RG)	MOL000098	quercetin	Protein kinase C beta type
Radix Glycyrrhizae (RG)	MOL000098	quercetin	Baculoviral IAP repeat-containing protein 5
Radix Glycyrrhizae (RG)	MOL000098	quercetin	Dual oxidase 2
Radix Glycyrrhizae (RG)	MOL000098	quercetin	Nitric oxide synthase, endothelial
Radix Glycyrrhizae (RG)	MOL000098	quercetin	Heat shock protein beta-1
Radix Glycyrrhizae (RG)	MOL000098	quercetin	Transforming growth factor beta-1
Radix Glycyrrhizae (RG)	MOL000098	quercetin	Estrogen sulfotransferase
Radix Glycyrrhizae (RG)	MOL000098	quercetin	Maltase-glucoamylase, intestinal
Radix Glycyrrhizae (RG)	MOL000098	quercetin	Interleukin-2
Radix Glycyrrhizae (RG)	MOL000098	quercetin	Nuclear receptor subfamily 1 group I member 2
Radix Glycyrrhizae (RG)	MOL000098	quercetin	Cytochrome P450 1B1
Radix Glycyrrhizae (RG)	MOL000098	quercetin	G2/mitotic-specific cyclin-B1
Radix Glycyrrhizae (RG)	MOL000098	quercetin	Tissue-type plasminogen activator
Radix Glycyrrhizae (RG)	MOL000098	quercetin	Thrombomodulin
Radix Glycyrrhizae (RG)	MOL000098	quercetin	Plasminogen activator inhibitor 1
Radix Glycyrrhizae (RG)	MOL000098	quercetin	Collagen alpha-1(I) chain
Radix Glycyrrhizae (RG)	MOL000098	quercetin	Interferon gamma
Radix Glycyrrhizae (RG)	MOL000098	quercetin	Arachidonate 5-lipoxygenase
Radix Glycyrrhizae (RG)	MOL000098	quercetin	Phosphatidylinositol-3,4,5-trisphosphate 3-phosphatase and dual-specificity protein phosphatase PTEN
Radix Glycyrrhizae (RG)	MOL000098	quercetin	Interleukin-1 alpha
Radix Glycyrrhizae (RG)	MOL000098	quercetin	Myeloperoxidase
Radix Glycyrrhizae (RG)	MOL000098	quercetin	DNA topoisomerase 2-alpha
Radix Glycyrrhizae (RG)	MOL000098	quercetin	Neutrophil cytosol factor 1
Radix Glycyrrhizae (RG)	MOL000098	quercetin	ATP-binding cassette sub-family G member 2
Radix Glycyrrhizae (RG)	MOL000098	quercetin	Hyaluronan synthase 2
Radix Glycyrrhizae (RG)	MOL000098	quercetin	Glutathione S-transferase P
Radix Glycyrrhizae (RG)	MOL000098	quercetin	Nuclear factor erythroid 2-related factor
Radix Glycyrrhizae (RG)	MOL000098	quercetin	NAD(P)H dehydrogenase [quinone] 1
Radix Glycyrrhizae (RG)	MOL000098	quercetin	Poly [ADP-ribose] polymerase 1
Radix Glycyrrhizae (RG)	MOL000098	quercetin	Aryl hydrocarbon receptor
Radix Glycyrrhizae (RG)	MOL000098	quercetin	26S proteasome non-ATPase regulatory subunit 3

Radix Glycyrrhizae (RG)	MOL000098	quercetin	Solute carrier family 2, facilitated glucose transporter member 4
Radix Glycyrrhizae (RG)	MOL000098	quercetin	Collagen alpha-1(III) chain
Radix Glycyrrhizae (RG)	MOL000098	quercetin	DNA gyrase subunit B
Radix Glycyrrhizae (RG)	MOL000098	quercetin	C-X-C motif chemokine 11
Radix Glycyrrhizae (RG)	MOL000098	quercetin	C-X-C motif chemokine 2
Radix Glycyrrhizae (RG)	MOL000098	quercetin	DDB1- and CUL4-associated factor 5
Radix Glycyrrhizae (RG)	MOL000098	quercetin	Nuclear receptor subfamily 1 group I member 3
Radix Glycyrrhizae (RG)	MOL000098	quercetin	Serine/threonine-protein kinase Chk2
Radix Glycyrrhizae (RG)	MOL000098	quercetin	Insulin receptor
Radix Glycyrrhizae (RG)	MOL000098	quercetin	Claudin-4
Radix Glycyrrhizae (RG)	MOL000098	quercetin	Peroxisome proliferator-activated receptor alpha
Radix Glycyrrhizae (RG)	MOL000098	quercetin	Peroxisome proliferator-activated receptor delta
Radix Glycyrrhizae (RG)	MOL000098	quercetin	Heat shock factor protein 1
Radix Glycyrrhizae (RG)	MOL000098	quercetin	C-reactive protein
Radix Glycyrrhizae (RG)	MOL000098	quercetin	C-X-C motif chemokine 10
Radix Glycyrrhizae (RG)	MOL000098	quercetin	Inhibitor of nuclear factor kappa-B kinase subunit alpha
Radix Glycyrrhizae (RG)	MOL000098	quercetin	Osteopontin
Radix Glycyrrhizae (RG)	MOL000098	quercetin	Runt-related transcription factor 2
Radix Glycyrrhizae (RG)	MOL000098	quercetin	Ras association domain-containing protein 1
Radix Glycyrrhizae (RG)	MOL000098	quercetin	Transcription factor E2F1
Radix Glycyrrhizae (RG)	MOL000098	quercetin	Transcription factor E2F2
Radix Glycyrrhizae (RG)	MOL000098	quercetin	Prostatic acid phosphatase
Radix Glycyrrhizae (RG)	MOL000098	quercetin	Cathepsin D
Radix Glycyrrhizae (RG)	MOL000098	quercetin	Insulin-like growth factor-binding protein 3
Radix Glycyrrhizae (RG)	MOL000098	quercetin	Insulin-like growth factor II
Radix Glycyrrhizae (RG)	MOL000098	quercetin	CD40 ligand
Radix Glycyrrhizae (RG)	MOL000098	quercetin	Interferon regulatory factor 1
Radix Glycyrrhizae (RG)	MOL000098	quercetin	Receptor tyrosine-protein kinase erbB-3
Radix Glycyrrhizae (RG)	MOL000098	quercetin	Serum paraoxonase/arylesterase 1
Radix Glycyrrhizae (RG)	MOL000098	quercetin	Type I iodothyronine deiodinase
Radix Glycyrrhizae (RG)	MOL000098	quercetin	Procollagen C-endopeptidase enhancer
Radix Glycyrrhizae (RG)	MOL000098	quercetin	Puromycin-sensitive aminopeptidase
Radix Glycyrrhizae (RG)	MOL000098	quercetin	Hexokinase-2
Radix Glycyrrhizae (RG)	MOL000098	quercetin	Homeobox protein Nkx-3.1
Radix Glycyrrhizae (RG)	MOL000098	quercetin	Ras GTPase-activating protein 1
Radix Glycyrrhizae (RG)	MOL000098	quercetin	Peroxidase C1A
Radix Glycyrrhizae (RG)	MOL000098	quercetin	Glutathione S-transferase Mu 1
Radix Glycyrrhizae (RG)	MOL000098	quercetin	Glutathione S-transferase Mu 2
Fructus Chebulae (FC)	MOL001002	ellagic acid	Cell division protein kinase 2
Fructus Chebulae (FC)	MOL001002	ellagic acid	Estrogen receptor
Fructus Chebulae (FC)	MOL001002	ellagic acid	Androgen receptor
Fructus Chebulae (FC)	MOL001002	ellagic acid	Progesterone receptor
Fructus Chebulae (FC)	MOL001002	ellagic acid	Heat shock protein HSP 90
Fructus Chebulae (FC)	MOL001002	ellagic acid	Transcription factor p65
Fructus Chebulae (FC)	MOL001002	ellagic acid	Vascular endothelial growth factor A
Fructus Chebulae (FC)	MOL001002	ellagic acid	Cyclin-dependent kinase inhibitor 1
Fructus Chebulae (FC)	MOL001002	ellagic acid	72 kDa type IV collagenase
Fructus Chebulae (FC)	MOL001002	ellagic acid	Matrix metalloproteinase-9
Fructus Chebulae (FC)	MOL001002	ellagic acid	NF-kappa-B inhibitor alpha
Fructus Chebulae (FC)	MOL001002	ellagic acid	Interleukin-8
Fructus Chebulae (FC)	MOL001002	ellagic acid	Protein kinase C beta type
Fructus Chebulae (FC)	MOL001002	ellagic acid	Glutathione S-transferase P
Fructus Chebulae (FC)	MOL001002	ellagic acid	Insulin-like growth factor II
Fructus Chebulae (FC)	MOL001002	ellagic acid	Glutathione S-transferase Mu 1
Fructus Chebulae (FC)	MOL001002	ellagic acid	Glutathione S-transferase Mu 2
Fructus Chebulae (FC)	MOL001002	ellagic acid	Glutathione S-transferase A1

Fructus Chebulae (FC)	MOL001002	ellagic acid	Glutathione S-transferase A2
Fructus Chebulae (FC)	MOL001002	ellagic acid	Chitin synthase 2
Fructus Chebulae (FC)	MOL006376	7-Dehydrosigmasterol	Progesterone receptor
Fructus Chebulae (FC)	MOL006376	7-Dehydrosigmasterol	Nuclear receptor coactivator 2
Fructus Chebulae (FC)	MOL006826	chebulic acid	Prostaglandin G/H synthase 2
Fructus Chebulae (FC)	MOL009135	ellipticine	Prostaglandin G/H synthase 1
Fructus Chebulae (FC)	MOL009135	ellipticine	Prostaglandin G/H synthase 2
Fructus Chebulae (FC)	MOL009135	ellipticine	Retinoic acid receptor RXR-alpha
Fructus Chebulae (FC)	MOL009135	ellipticine	mRNA of PKA Catalytic Subunit C-
Fructus Chebulae (FC)	MOL009135	ellipticine	Nuclear receptor coactivator 2
Fructus Chebulae (FC)	MOL009135	ellipticine	cAMP-dependent protein kinase inhibitor alpha
Fructus Chebulae (FC)	MOL009135	ellipticine	Apoptosis regulator Bcl-2
Fructus Chebulae (FC)	MOL009135	ellipticine	Bcl-2-like protein 1
Fructus Chebulae (FC)	MOL009135	ellipticine	Cyclin-dependent kinase inhibitor 1
Fructus Chebulae (FC)	MOL009135	ellipticine	Eukaryotic translation initiation factor 6
Fructus Chebulae (FC)	MOL009135	ellipticine	Apoptosis regulator BAX
Fructus Chebulae (FC)	MOL009135	ellipticine	Caspase-9
Fructus Chebulae (FC)	MOL009135	ellipticine	Caspase-3
Fructus Chebulae (FC)	MOL009135	ellipticine	Cellular tumor antigen p53
Fructus Chebulae (FC)	MOL009135	ellipticine	Caspase-8
Fructus Chebulae (FC)	MOL009135	ellipticine	Cell division control protein 2 homolog
Fructus Chebulae (FC)	MOL009135	ellipticine	Cytochrome P450 1A2
Fructus Chebulae (FC)	MOL009135	ellipticine	Cytochrome P450 1A1
Fructus Chebulae (FC)	MOL009135	ellipticine	G2/mitotic-specific cyclin-B1
Fructus Chebulae (FC)	MOL009135	ellipticine	Baculoviral IAP repeat-containing protein 4
Fructus Chebulae (FC)	MOL009135	ellipticine	Ras-specific guanine nucleotide-releasing factor 1
Fructus Chebulae (FC)	MOL009135	ellipticine	Cell division protein kinase 12
Fructus Chebulae (FC)	MOL009136	Peraksine	Muscarinic acetylcholine receptor M3
Fructus Chebulae (FC)	MOL009136	Peraksine	Muscarinic acetylcholine receptor M1
Fructus Chebulae (FC)	MOL009136	Peraksine	Androgen receptor
Fructus Chebulae (FC)	MOL009136	Peraksine	Sodium channel protein type 5 subunit alpha
Fructus Chebulae (FC)	MOL009136	Peraksine	Muscarinic acetylcholine receptor M5
Fructus Chebulae (FC)	MOL009136	Peraksine	5-hydroxytryptamine receptor 3A
Fructus Chebulae (FC)	MOL009136	Peraksine	Muscarinic acetylcholine receptor M4
Fructus Chebulae (FC)	MOL009136	Peraksine	Delta-type opioid receptor
Fructus Chebulae (FC)	MOL009136	Peraksine	Acetylcholinesterase
Fructus Chebulae (FC)	MOL009136	Peraksine	5-hydroxytryptamine 2A receptor
Fructus Chebulae (FC)	MOL009136	Peraksine	Alpha-1A adrenergic receptor
Fructus Chebulae (FC)	MOL009136	Peraksine	Muscarinic acetylcholine receptor M2
Fructus Chebulae (FC)	MOL009136	Peraksine	Alpha-1B adrenergic receptor
Fructus Chebulae (FC)	MOL009136	Peraksine	Beta-2 adrenergic receptor
Fructus Chebulae (FC)	MOL009136	Peraksine	Alpha-1D adrenergic receptor
Fructus Chebulae (FC)	MOL009136	Peraksine	Sodium-dependent serotonin transporter
Fructus Chebulae (FC)	MOL009136	Peraksine	Mu-type opioid receptor
Fructus Chebulae (FC)	MOL009137	(R)-(6-methoxy-4-quinolyl)-[(2R,4R,5S)-5-vinylquinuclidin-2-yl]methanol	Prostaglandin G/H synthase 1
Fructus Chebulae (FC)	MOL009137	(R)-(6-methoxy-4-quinolyl)-[(2R,4R,5S)-5-vinylquinuclidin-2-yl]methanol	Muscarinic acetylcholine receptor M3
Fructus Chebulae (FC)	MOL009137	(R)-(6-methoxy-4-quinolyl)-[(2R,4R,5S)-5-vinylquinuclidin-2-yl]methanol	Potassium voltage-gated channel subfamily H member 2
Fructus Chebulae (FC)	MOL009137	(R)-(6-methoxy-4-quinolyl)-[(2R,4R,5S)-5-vinylquinuclidin-2-yl]methanol	Muscarinic acetylcholine receptor M1
Fructus Chebulae (FC)	MOL009137	(R)-(6-methoxy-4-quinolyl)-[(2R,4R,5S)-5-vinylquinuclidin-2-yl]methanol	Beta-1 adrenergic receptor

Fructus Chebulae (FC)	MOL009137	(R)-(6-methoxy-4-quinolyl)-[(2R,4R,5S)-5-vinylquinuclidin-2-yl]methanol	Sodium channel protein type 5 subunit alpha
Fructus Chebulae (FC)	MOL009137	(R)-(6-methoxy-4-quinolyl)-[(2R,4R,5S)-5-vinylquinuclidin-2-yl]methanol	Muscarinic acetylcholine receptor M5
Fructus Chebulae (FC)	MOL009137	(R)-(6-methoxy-4-quinolyl)-[(2R,4R,5S)-5-vinylquinuclidin-2-yl]methanol	Prostaglandin G/H synthase 2
Fructus Chebulae (FC)	MOL009137	(R)-(6-methoxy-4-quinolyl)-[(2R,4R,5S)-5-vinylquinuclidin-2-yl]methanol	Alpha-2A adrenergic receptor
Fructus Chebulae (FC)	MOL009137	(R)-(6-methoxy-4-quinolyl)-[(2R,4R,5S)-5-vinylquinuclidin-2-yl]methanol	5-hydroxytryptamine receptor 3A
Fructus Chebulae (FC)	MOL009137	(R)-(6-methoxy-4-quinolyl)-[(2R,4R,5S)-5-vinylquinuclidin-2-yl]methanol	Alpha-2C adrenergic receptor
Fructus Chebulae (FC)	MOL009137	(R)-(6-methoxy-4-quinolyl)-[(2R,4R,5S)-5-vinylquinuclidin-2-yl]methanol	D(4) dopamine receptor
Fructus Chebulae (FC)	MOL009137	(R)-(6-methoxy-4-quinolyl)-[(2R,4R,5S)-5-vinylquinuclidin-2-yl]methanol	Muscarinic acetylcholine receptor M4
Fructus Chebulae (FC)	MOL009137	(R)-(6-methoxy-4-quinolyl)-[(2R,4R,5S)-5-vinylquinuclidin-2-yl]methanol	Retinoic acid receptor RXR-alpha
Fructus Chebulae (FC)	MOL009137	(R)-(6-methoxy-4-quinolyl)-[(2R,4R,5S)-5-vinylquinuclidin-2-yl]methanol	Delta-type opioid receptor
Fructus Chebulae (FC)	MOL009137	(R)-(6-methoxy-4-quinolyl)-[(2R,4R,5S)-5-vinylquinuclidin-2-yl]methanol	CGMP-inhibited 3',5'-cyclic phosphodiesterase A
Fructus Chebulae (FC)	MOL009137	(R)-(6-methoxy-4-quinolyl)-[(2R,4R,5S)-5-vinylquinuclidin-2-yl]methanol	5-hydroxytryptamine 2A receptor
Fructus Chebulae (FC)	MOL009137	(R)-(6-methoxy-4-quinolyl)-[(2R,4R,5S)-5-vinylquinuclidin-2-yl]methanol	Alpha-1A adrenergic receptor
Fructus Chebulae (FC)	MOL009137	(R)-(6-methoxy-4-quinolyl)-[(2R,4R,5S)-5-vinylquinuclidin-2-yl]methanol	5-hydroxytryptamine 2C receptor
Fructus Chebulae (FC)	MOL009137	(R)-(6-methoxy-4-quinolyl)-[(2R,4R,5S)-5-vinylquinuclidin-2-yl]methanol	Alpha-2B adrenergic receptor
Fructus Chebulae (FC)	MOL009137	(R)-(6-methoxy-4-quinolyl)-[(2R,4R,5S)-5-vinylquinuclidin-2-yl]methanol	Alpha-1B adrenergic receptor
Fructus Chebulae (FC)	MOL009137	(R)-(6-methoxy-4-quinolyl)-[(2R,4R,5S)-5-vinylquinuclidin-2-yl]methanol	D(3) dopamine receptor
Fructus Chebulae (FC)	MOL009137	(R)-(6-methoxy-4-quinolyl)-[(2R,4R,5S)-5-vinylquinuclidin-2-yl]methanol	Sodium-dependent dopamine transporter
Fructus Chebulae (FC)	MOL009137	(R)-(6-methoxy-4-quinolyl)-[(2R,4R,5S)-5-vinylquinuclidin-2-yl]methanol	5-hydroxytryptamine 7 receptor
Fructus Chebulae (FC)	MOL009137	(R)-(6-methoxy-4-quinolyl)-[(2R,4R,5S)-5-vinylquinuclidin-2-yl]methanol	Beta-2 adrenergic receptor
Fructus Chebulae (FC)	MOL009137	(R)-(6-methoxy-4-quinolyl)-[(2R,4R,5S)-5-vinylquinuclidin-2-yl]methanol	Alpha-1D adrenergic receptor

Fructus Chebulae (FC)	MOL009137	(R)-(6-methoxy-4-quinolyl)-[(2R,4R,5S)-5-vinylquinuclidin-2-yl]methanol	Sodium-dependent serotonin transporter
Fructus Chebulae (FC)	MOL009137	(R)-(6-methoxy-4-quinolyl)-[(2R,4R,5S)-5-vinylquinuclidin-2-yl]methanol	D(2) dopamine receptor
Fructus Chebulae (FC)	MOL009137	(R)-(6-methoxy-4-quinolyl)-[(2R,4R,5S)-5-vinylquinuclidin-2-yl]methanol	Epidermal growth factor receptor
Fructus Chebulae (FC)	MOL009137	(R)-(6-methoxy-4-quinolyl)-[(2R,4R,5S)-5-vinylquinuclidin-2-yl]methanol	Mu-type opioid receptor
Fructus Chebulae (FC)	MOL009137	(R)-(6-methoxy-4-quinolyl)-[(2R,4R,5S)-5-vinylquinuclidin-2-yl]methanol	Heat shock protein HSP 90
Fructus Chebulae (FC)	MOL009137	(R)-(6-methoxy-4-quinolyl)-[(2R,4R,5S)-5-vinylquinuclidin-2-yl]methanol	Neuronal acetylcholine receptor protein, alpha-7 chain
Fructus Chebulae (FC)	MOL009137	(R)-(6-methoxy-4-quinolyl)-[(2R,4R,5S)-5-vinylquinuclidin-2-yl]methanol	mRNA of PKA Catalytic Subunit C-alpha
Fructus Chebulae (FC)	MOL009137	(R)-(6-methoxy-4-quinolyl)-[(2R,4R,5S)-5-vinylquinuclidin-2-yl]methanol	Calmodulin
Fructus Chebulae (FC)	MOL009149	Cheilanthifoline	Prostaglandin G/H synthase 1
Fructus Chebulae (FC)	MOL009149	Cheilanthifoline	Dopamine D1 receptor
Fructus Chebulae (FC)	MOL009149	Cheilanthifoline	Muscarinic acetylcholine receptor M3
Fructus Chebulae (FC)	MOL009149	Cheilanthifoline	Potassium voltage-gated channel subfamily H member 2
Fructus Chebulae (FC)	MOL009149	Cheilanthifoline	Muscarinic acetylcholine receptor M1
Fructus Chebulae (FC)	MOL009149	Cheilanthifoline	Sodium channel protein type 5 subunit alpha
Fructus Chebulae (FC)	MOL009149	Cheilanthifoline	Coagulation factor Xa
Fructus Chebulae (FC)	MOL009149	Cheilanthifoline	Muscarinic acetylcholine receptor M5
Fructus Chebulae (FC)	MOL009149	Cheilanthifoline	Prostaglandin G/H synthase 2
Fructus Chebulae (FC)	MOL009149	Cheilanthifoline	5-hydroxytryptamine receptor 3A
Fructus Chebulae (FC)	MOL009149	Cheilanthifoline	Muscarinic acetylcholine receptor M4
Fructus Chebulae (FC)	MOL009149	Cheilanthifoline	Retinoic acid receptor RXR-alpha
Fructus Chebulae (FC)	MOL009149	Cheilanthifoline	Delta-type opioid receptor
Fructus Chebulae (FC)	MOL009149	Cheilanthifoline	CGMP-inhibited 3',5'-cyclic phosphodiesterase A
Fructus Chebulae (FC)	MOL009149	Cheilanthifoline	5-hydroxytryptamine 2A receptor
Fructus Chebulae (FC)	MOL009149	Cheilanthifoline	5-hydroxytryptamine 2C receptor
Fructus Chebulae (FC)	MOL009149	Cheilanthifoline	Muscarinic acetylcholine receptor M2
Fructus Chebulae (FC)	MOL009149	Cheilanthifoline	Alpha-2B adrenergic receptor
Fructus Chebulae (FC)	MOL009149	Cheilanthifoline	Alpha-1B adrenergic receptor
Fructus Chebulae (FC)	MOL009149	Cheilanthifoline	D(3) dopamine receptor
Fructus Chebulae (FC)	MOL009149	Cheilanthifoline	Sodium-dependent dopamine
Fructus Chebulae (FC)	MOL009149	Cheilanthifoline	Beta-2 adrenergic receptor
Fructus Chebulae (FC)	MOL009149	Cheilanthifoline	Alpha-1D adrenergic receptor
Fructus Chebulae (FC)	MOL009149	Cheilanthifoline	Sodium-dependent serotonin transporter
Fructus Chebulae (FC)	MOL009149	Cheilanthifoline	Mu-type opioid receptor
Fructus Chebulae (FC)	MOL009149	Cheilanthifoline	Heat shock protein HSP 90
Fructus Chebulae (FC)	MOL009149	Cheilanthifoline	mRNA of PKA Catalytic Subunit C-
Fructus Chebulae (FC)	MOL009149	Cheilanthifoline	Calmodulin
Fructus Chebulae (FC)	MOL009149	Cheilanthifoline	Coagulation factor VII
Radix Aucklandiae (RA)	MOL010813	Benzo[a]carbazole	Prostaglandin G/H synthase 1
Radix Aucklandiae (RA)	MOL010813	Benzo[a]carbazole	Prostaglandin G/H synthase 2
Radix Aucklandiae (RA)	MOL010813	Benzo[a]carbazole	Amine oxidase [flavin-containing] B
Radix Aucklandiae (RA)	MOL010813	Benzo[a]carbazole	mRNA of PKA Catalytic Subunit C-
Radix Aucklandiae (RA)	MOL010813	Benzo[a]carbazole	Nuclear receptor coactivator 2
Radix Aucklandiae (RA)	MOL010813	Benzo[a]carbazole	cAMP-dependent protein kinase inhibitor alpha

Radix Aucklandiae (RA)	MOL010828	cynaropicrin	Prostaglandin G/H synthase 2
Radix Aucklandiae (RA)	MOL010828	cynaropicrin	Nuclear receptor coactivator 2
Radix Aucklandiae (RA)	MOL000211	Mairin	Progesterone receptor
Radix Aucklandiae (RA)	MOL000359	sitosterol	Progesterone receptor
Radix Aucklandiae (RA)	MOL000359	sitosterol	Nuclear receptor coactivator 2
Radix Aucklandiae (RA)	MOL000359	sitosterol	Mineralocorticoid receptor
Radix Aucklandiae (RA)	MOL000449	Stigmasterol	Progesterone receptor
Radix Aucklandiae (RA)	MOL000449	Stigmasterol	Mineralocorticoid receptor
Radix Aucklandiae (RA)	MOL000449	Stigmasterol	Nuclear receptor coactivator 2
Radix Aucklandiae (RA)	MOL000449	Stigmasterol	Alcohol dehydrogenase 1C
Radix Aucklandiae (RA)	MOL000449	Stigmasterol	Ig gamma-1 chain C region
Radix Aucklandiae (RA)	MOL000449	Stigmasterol	Retinoic acid receptor RXR-alpha
Radix Aucklandiae (RA)	MOL000449	Stigmasterol	Nuclear receptor coactivator 1
Radix Aucklandiae (RA)	MOL000449	Stigmasterol	Prostaglandin G/H synthase 1
Radix Aucklandiae (RA)	MOL000449	Stigmasterol	Prostaglandin G/H synthase 2
Radix Aucklandiae (RA)	MOL000449	Stigmasterol	Alpha-2A adrenergic receptor
Radix Aucklandiae (RA)	MOL000449	Stigmasterol	Sodium-dependent noradrenaline transporter
Radix Aucklandiae (RA)	MOL000449	Stigmasterol	Sodium-dependent dopamine
Radix Aucklandiae (RA)	MOL000449	Stigmasterol	Beta-2 adrenergic receptor
Radix Aucklandiae (RA)	MOL000449	Stigmasterol	Aldose reductase
Radix Aucklandiae (RA)	MOL000449	Stigmasterol	Urokinase-type plasminogen activator
Radix Aucklandiae (RA)	MOL000449	Stigmasterol	Leukotriene A-4 hydrolase
Radix Aucklandiae (RA)	MOL000449	Stigmasterol	Amine oxidase [flavin-containing] B
Radix Aucklandiae (RA)	MOL000449	Stigmasterol	Amine oxidase [flavin-containing] A
Radix Aucklandiae (RA)	MOL000449	Stigmasterol	mRNA of PKA Catalytic Subunit C-
Radix Aucklandiae (RA)	MOL000449	Stigmasterol	Chymotrypsinogen B
Radix Aucklandiae (RA)	MOL000449	Stigmasterol	Muscarinic acetylcholine receptor M3
Radix Aucklandiae (RA)	MOL000449	Stigmasterol	Muscarinic acetylcholine receptor M1
Radix Aucklandiae (RA)	MOL000449	Stigmasterol	Beta-1 adrenergic receptor
Radix Aucklandiae (RA)	MOL000449	Stigmasterol	Sodium channel protein type 5 subunit alpha
Radix Aucklandiae (RA)	MOL000449	Stigmasterol	5-hydroxytryptamine 2A receptor
Radix Aucklandiae (RA)	MOL000449	Stigmasterol	Alpha-1A adrenergic receptor
Radix Aucklandiae (RA)	MOL000449	Stigmasterol	Gamma-aminobutyric-acid receptor alpha-3 subunit
Radix Aucklandiae (RA)	MOL000449	Stigmasterol	Muscarinic acetylcholine receptor M2
Radix Aucklandiae (RA)	MOL000449	Stigmasterol	Alpha-1B adrenergic receptor
Radix Aucklandiae (RA)	MOL000449	Stigmasterol	Gamma-aminobutyric acid receptor subunit alpha-1
Radix Aucklandiae (RA)	MOL000449	Stigmasterol	Neuronal acetylcholine receptor protein, alpha-7 chain
Semen Myristicae (SM)	MOL000358	beta-sitosterol	Progesterone receptor
Semen Myristicae (SM)	MOL000358	beta-sitosterol	Nuclear receptor coactivator 2
Semen Myristicae (SM)	MOL000358	beta-sitosterol	Prostaglandin G/H synthase 1
Semen Myristicae (SM)	MOL000358	beta-sitosterol	Prostaglandin G/H synthase 2
Semen Myristicae (SM)	MOL000358	beta-sitosterol	Heat shock protein HSP 90
Semen Myristicae (SM)	MOL000358	beta-sitosterol	Phosphatidylinositol-4,5-bisphosphate 3-kinase catalytic subunit, gamma
Semen Myristicae (SM)	MOL000358	beta-sitosterol	Potassium voltage-gated channel subfamily H member 2
Semen Myristicae (SM)	MOL000358	beta-sitosterol	mRNA of PKA Catalytic Subunit C-
Semen Myristicae (SM)	MOL000358	beta-sitosterol	Dopamine D1 receptor
Semen Myristicae (SM)	MOL000358	beta-sitosterol	Muscarinic acetylcholine receptor M3
Semen Myristicae (SM)	MOL000358	beta-sitosterol	Muscarinic acetylcholine receptor M1
Semen Myristicae (SM)	MOL000358	beta-sitosterol	Sodium channel protein type 5 subunit alpha
Semen Myristicae (SM)	MOL000358	beta-sitosterol	Gamma-aminobutyric-acid receptor alpha-2 subunit
Semen Myristicae (SM)	MOL000358	beta-sitosterol	Muscarinic acetylcholine receptor M4
Semen Myristicae (SM)	MOL000358	beta-sitosterol	CGMP-inhibited 3',5'-cyclic phosphodiesterase A
Semen Myristicae (SM)	MOL000358	beta-sitosterol	5-hydroxytryptamine 2A receptor

Semen Myristicae (SM)	MOL000358	beta-sitosterol	Gamma-aminobutyric-acid receptor alpha-5 subunit
Semen Myristicae (SM)	MOL000358	beta-sitosterol	Alpha-1A adrenergic receptor
Semen Myristicae (SM)	MOL000358	beta-sitosterol	Gamma-aminobutyric-acid receptor alpha-3 subunit
Semen Myristicae (SM)	MOL000358	beta-sitosterol	Muscarinic acetylcholine receptor M2
Semen Myristicae (SM)	MOL000358	beta-sitosterol	Alpha-1B adrenergic receptor
Semen Myristicae (SM)	MOL000358	beta-sitosterol	Beta-2 adrenergic receptor
Semen Myristicae (SM)	MOL000358	beta-sitosterol	Neuronal acetylcholine receptor subunit alpha-2
Semen Myristicae (SM)	MOL000358	beta-sitosterol	Sodium-dependent serotonin transporter
Semen Myristicae (SM)	MOL000358	beta-sitosterol	Mu-type opioid receptor
Semen Myristicae (SM)	MOL000358	beta-sitosterol	Gamma-aminobutyric acid receptor subunit alpha-1
Semen Myristicae (SM)	MOL000358	beta-sitosterol	Neuronal acetylcholine receptor protein, alpha-7 chain
Semen Myristicae (SM)	MOL000358	beta-sitosterol	Cytochrome P450-cam
Semen Myristicae (SM)	MOL000358	beta-sitosterol	Apoptosis regulator Bcl-2
Semen Myristicae (SM)	MOL000358	beta-sitosterol	Apoptosis regulator BAX
Semen Myristicae (SM)	MOL000358	beta-sitosterol	Caspase-9
Semen Myristicae (SM)	MOL000358	beta-sitosterol	Transcription factor AP-1
Semen Myristicae (SM)	MOL000358	beta-sitosterol	Caspase-3
Semen Myristicae (SM)	MOL000358	beta-sitosterol	Caspase-8
Semen Myristicae (SM)	MOL000358	beta-sitosterol	Protein kinase C alpha type
Semen Myristicae (SM)	MOL000358	beta-sitosterol	Transforming growth factor beta-1
Semen Myristicae (SM)	MOL000358	beta-sitosterol	Serum paraoxonase/arylesterase 1
Semen Myristicae (SM)	MOL000358	beta-sitosterol	Microtubule-associated protein 2
Semen Myristicae (SM)	MOL009243	Isoguaiacin	Nitric oxide synthase, inducible
Semen Myristicae (SM)	MOL009243	Isoguaiacin	Prostaglandin G/H synthase 1
Semen Myristicae (SM)	MOL009243	Isoguaiacin	Muscarinic acetylcholine receptor M3
Semen Myristicae (SM)	MOL009243	Isoguaiacin	Muscarinic acetylcholine receptor M1
Semen Myristicae (SM)	MOL009243	Isoguaiacin	Estrogen receptor
Semen Myristicae (SM)	MOL009243	Isoguaiacin	Androgen receptor
Semen Myristicae (SM)	MOL009243	Isoguaiacin	Sodium channel protein type 5 subunit alpha
Semen Myristicae (SM)	MOL009243	Isoguaiacin	Peroxisome proliferator activated receptor gamma
Semen Myristicae (SM)	MOL009243	Isoguaiacin	Prostaglandin G/H synthase 2
Semen Myristicae (SM)	MOL009243	Isoguaiacin	Nitric-oxide synthase, endothelial
Semen Myristicae (SM)	MOL009243	Isoguaiacin	Retinoic acid receptor RXR-alpha
Semen Myristicae (SM)	MOL009243	Isoguaiacin	Delta-type opioid receptor
Semen Myristicae (SM)	MOL009243	Isoguaiacin	CGMP-inhibited 3',5'-cyclic phosphodiesterase A
Semen Myristicae (SM)	MOL009243	Isoguaiacin	Alpha-1B adrenergic receptor
Semen Myristicae (SM)	MOL009243	Isoguaiacin	Sodium-dependent dopamine
Semen Myristicae (SM)	MOL009243	Isoguaiacin	Beta-2 adrenergic receptor
Semen Myristicae (SM)	MOL009243	Isoguaiacin	Alpha-1D adrenergic receptor
Semen Myristicae (SM)	MOL009243	Isoguaiacin	Mu-type opioid receptor
Semen Myristicae (SM)	MOL009243	Isoguaiacin	Estrogen receptor beta
Semen Myristicae (SM)	MOL009243	Isoguaiacin	Mitogen-activated protein kinase 14
Semen Myristicae (SM)	MOL009243	Isoguaiacin	Glycogen synthase kinase-3 beta
Semen Myristicae (SM)	MOL009243	Isoguaiacin	Heat shock protein HSP 90
Semen Myristicae (SM)	MOL009243	Isoguaiacin	Serine/threonine-protein kinase Chk1
Semen Myristicae (SM)	MOL009243	Isoguaiacin	Proto-oncogene serine/threonine-protein kinase Pim-1
Semen Myristicae (SM)	MOL009243	Isoguaiacin	Cyclin-A2
Semen Myristicae (SM)	MOL009243	Isoguaiacin	Nuclear receptor coactivator 2
Semen Myristicae (SM)	MOL009243	Isoguaiacin	Calmodulin
Semen Myristicae (SM)	MOL009254	galbacin	Potassium voltage-gated channel subfamily H member 2
Semen Myristicae (SM)	MOL009254	galbacin	Sodium channel protein type 5 subunit alpha
Semen Myristicae (SM)	MOL009254	galbacin	Prostaglandin G/H synthase 2

Semen Myristicae (SM)	MOL009254	galbacin	Retinoic acid receptor RXR-alpha
Semen Myristicae (SM)	MOL009254	galbacin	Beta-2 adrenergic receptor
Semen Myristicae (SM)	MOL009254	galbacin	Heat shock protein HSP 90
Semen Myristicae (SM)	MOL009255	5-[(2S,3S)-7-methoxy-3-methyl-5-[(E)-prop-1-enyl]-2,3-dihydrobenzofuran-2-yl]-1.3-benzodioxole	Prostaglandin G/H synthase 1
Semen Myristicae (SM)	MOL009255	5-[(2S,3S)-7-methoxy-3-methyl-5-[(E)-prop-1-enyl]-2,3-dihydrobenzofuran-2-yl]-1.3-benzodioxole	Muscarinic acetylcholine receptor M3
Semen Myristicae (SM)	MOL009255	5-[(2S,3S)-7-methoxy-3-methyl-5-[(E)-prop-1-enyl]-2,3-dihydrobenzofuran-2-yl]-1.3-benzodioxole	Thrombin
Semen Myristicae (SM)	MOL009255	5-[(2S,3S)-7-methoxy-3-methyl-5-[(E)-prop-1-enyl]-2,3-dihydrobenzofuran-2-yl]-1.3-benzodioxole	Muscarinic acetylcholine receptor M1
Semen Myristicae (SM)	MOL009255	5-[(2S,3S)-7-methoxy-3-methyl-5-[(E)-prop-1-enyl]-2,3-dihydrobenzofuran-2-yl]-1.3-benzodioxole	Beta-1 adrenergic receptor
Semen Myristicae (SM)	MOL009255	5-[(2S,3S)-7-methoxy-3-methyl-5-[(E)-prop-1-enyl]-2,3-dihydrobenzofuran-2-yl]-1.3-benzodioxole	Sodium channel protein type 5 subunit alpha
Semen Myristicae (SM)	MOL009255	5-[(2S,3S)-7-methoxy-3-methyl-5-[(E)-prop-1-enyl]-2,3-dihydrobenzofuran-2-yl]-1.3-benzodioxole	Prostaglandin G/H synthase 2
Semen Myristicae (SM)	MOL009255	5-[(2S,3S)-7-methoxy-3-methyl-5-[(E)-prop-1-enyl]-2,3-dihydrobenzofuran-2-yl]-1.3-benzodioxole	Nitric-oxide synthase, endothelial
Semen Myristicae (SM)	MOL009255	5-[(2S,3S)-7-methoxy-3-methyl-5-[(E)-prop-1-enyl]-2,3-dihydrobenzofuran-2-yl]-1.3-benzodioxole	Alpha-2A adrenergic receptor
Semen Myristicae (SM)	MOL009255	5-[(2S,3S)-7-methoxy-3-methyl-5-[(E)-prop-1-enyl]-2,3-dihydrobenzofuran-2-yl]-1.3-benzodioxole	Alpha-2C adrenergic receptor
Semen Myristicae (SM)	MOL009255	5-[(2S,3S)-7-methoxy-3-methyl-5-[(E)-prop-1-enyl]-2,3-dihydrobenzofuran-2-yl]-1.3-benzodioxole	Sodium-dependent noradrenaline transporter
Semen Myristicae (SM)	MOL009255	5-[(2S,3S)-7-methoxy-3-methyl-5-[(E)-prop-1-enyl]-2,3-dihydrobenzofuran-2-yl]-1.3-benzodioxole	Alpha-1A adrenergic receptor
Semen Myristicae (SM)	MOL009255	5-[(2S,3S)-7-methoxy-3-methyl-5-[(E)-prop-1-enyl]-2,3-dihydrobenzofuran-2-yl]-1.3-benzodioxole	Muscarinic acetylcholine receptor M2
Semen Myristicae (SM)	MOL009255	5-[(2S,3S)-7-methoxy-3-methyl-5-[(E)-prop-1-enyl]-2,3-dihydrobenzofuran-2-yl]-1.3-benzodioxole	Alpha-1B adrenergic receptor
Semen Myristicae (SM)	MOL009255	5-[(2S,3S)-7-methoxy-3-methyl-5-[(E)-prop-1-enyl]-2,3-dihydrobenzofuran-2-yl]-1.3-benzodioxole	Sodium-dependent dopamine transporter

Semen Myristicae (SM)	MOL009255	5-[(2S,3S)-7-methoxy-3-methyl-5-[(E)-prop-1-enyl]-2,3-dihydrobenzofuran-2-yl]-1.3-benzodioxole	Beta-2 adrenergic receptor
Semen Myristicae (SM)	MOL009255	5-[(2S,3S)-7-methoxy-3-methyl-5-[(E)-prop-1-enyl]-2,3-dihydrobenzofuran-2-yl]-1.3-benzodioxole	Gamma-aminobutyric acid receptor subunit alpha-1
Semen Myristicae (SM)	MOL009255	5-[(2S,3S)-7-methoxy-3-methyl-5-[(E)-prop-1-enyl]-2,3-dihydrobenzofuran-2-yl]-1.3-benzodioxole	Beta-lactamase
Semen Myristicae (SM)	MOL009255	5-[(2S,3S)-7-methoxy-3-methyl-5-[(E)-prop-1-enyl]-2,3-dihydrobenzofuran-2-yl]-1.3-benzodioxole	Leukotriene A-4 hydrolase
Semen Myristicae (SM)	MOL009255	5-[(2S,3S)-7-methoxy-3-methyl-5-[(E)-prop-1-enyl]-2,3-dihydrobenzofuran-2-yl]-1.3-benzodioxole	Amine oxidase [flavin-containing] B
Semen Myristicae (SM)	MOL009255	5-[(2S,3S)-7-methoxy-3-methyl-5-[(E)-prop-1-enyl]-2,3-dihydrobenzofuran-2-yl]-1.3-benzodioxole	Trypsin-1
Semen Myristicae (SM)	MOL009255	5-[(2S,3S)-7-methoxy-3-methyl-5-[(E)-prop-1-enyl]-2,3-dihydrobenzofuran-2-yl]-1.3-benzodioxole	Potassium voltage-gated channel subfamily H member 2
Semen Myristicae (SM)	MOL009255	5-[(2S,3S)-7-methoxy-3-methyl-5-[(E)-prop-1-enyl]-2,3-dihydrobenzofuran-2-yl]-1.3-benzodioxole	Estrogen receptor
Semen Myristicae (SM)	MOL009255	5-[(2S,3S)-7-methoxy-3-methyl-5-[(E)-prop-1-enyl]-2,3-dihydrobenzofuran-2-yl]-1.3-benzodioxole	Coagulation factor Xa
Semen Myristicae (SM)	MOL009255	5-[(2S,3S)-7-methoxy-3-methyl-5-[(E)-prop-1-enyl]-2,3-dihydrobenzofuran-2-yl]-1.3-benzodioxole	Retinoic acid receptor RXR-alpha
Semen Myristicae (SM)	MOL009255	5-[(2S,3S)-7-methoxy-3-methyl-5-[(E)-prop-1-enyl]-2,3-dihydrobenzofuran-2-yl]-1.3-benzodioxole	Acetylcholinesterase
Semen Myristicae (SM)	MOL009255	5-[(2S,3S)-7-methoxy-3-methyl-5-[(E)-prop-1-enyl]-2,3-dihydrobenzofuran-2-yl]-1.3-benzodioxole	CGMP-inhibited 3',5'-cyclic phosphodiesterase A
Semen Myristicae (SM)	MOL009255	5-[(2S,3S)-7-methoxy-3-methyl-5-[(E)-prop-1-enyl]-2,3-dihydrobenzofuran-2-yl]-1.3-benzodioxole	Alpha-1D adrenergic receptor
Semen Myristicae (SM)	MOL009255	5-[(2S,3S)-7-methoxy-3-methyl-5-[(E)-prop-1-enyl]-2,3-dihydrobenzofuran-2-yl]-1.3-benzodioxole	Heat shock protein HSP 90
Semen Myristicae (SM)	MOL009255	5-[(2S,3S)-7-methoxy-3-methyl-5-[(E)-prop-1-enyl]-2,3-dihydrobenzofuran-2-yl]-1.3-benzodioxole	mRNA of PKA Catalytic Subunit C-alpha

Semen Myristicae (SM)	MOL009255	5-[(2S,3S)-7-methoxy-3-methyl-5-[(E)-prop-1-enyl]-2,3-dihydrobenzofuran-2-yl]-1,3-benzodioxole	Retinoic acid receptor RXR-beta
Semen Myristicae (SM)	MOL009255	5-[(2S,3S)-7-methoxy-3-methyl-5-[(E)-prop-1-enyl]-2,3-dihydrobenzofuran-2-yl]-1,3-benzodioxole	Calmodulin
Semen Myristicae (SM)	MOL009259	Kudos	Thrombin
Semen Myristicae (SM)	MOL009259	Kudos	Sodium channel protein type 5 subunit alpha
Semen Myristicae (SM)	MOL009259	Kudos	Coagulation factor Xa
Semen Myristicae (SM)	MOL009259	Kudos	Prostaglandin G/H synthase 2
Semen Myristicae (SM)	MOL009259	Kudos	Dipeptidyl peptidase IV
Semen Myristicae (SM)	MOL009259	Kudos	mRNA of PKA Catalytic Subunit C-
Semen Myristicae (SM)	MOL009259	Kudos	Calmodulin
Semen Myristicae (SM)	MOL009263	saucernetindiol	Prostaglandin G/H synthase 1
Semen Myristicae (SM)	MOL009263	saucernetindiol	Potassium voltage-gated channel subfamily H member 2
Semen Myristicae (SM)	MOL009263	saucernetindiol	Muscarinic acetylcholine receptor M1
Semen Myristicae (SM)	MOL009263	saucernetindiol	Estrogen receptor
Semen Myristicae (SM)	MOL009263	saucernetindiol	Sodium channel protein type 5 subunit alpha
Semen Myristicae (SM)	MOL009263	saucernetindiol	Coagulation factor Xa
Semen Myristicae (SM)	MOL009263	saucernetindiol	Prostaglandin G/H synthase 2
Semen Myristicae (SM)	MOL009263	saucernetindiol	Retinoic acid receptor RXR-alpha
Semen Myristicae (SM)	MOL009263	saucernetindiol	Alpha-1B adrenergic receptor
Semen Myristicae (SM)	MOL009263	saucernetindiol	Sodium-dependent dopamine
Semen Myristicae (SM)	MOL009263	saucernetindiol	Beta-2 adrenergic receptor
Semen Myristicae (SM)	MOL009263	saucernetindiol	Heat shock protein HSP 90
Semen Myristicae (SM)	MOL009263	saucernetindiol	Proto-oncogene serine/threonine-protein kinase Pim-1
Semen Myristicae (SM)	MOL009263	saucernetindiol	Nuclear receptor coactivator 2
Semen Myristicae (SM)	MOL009263	saucernetindiol	Calmodulin
Semen Myristicae (SM)	MOL009264	tetrahydrofuroguaiacin B	Muscarinic acetylcholine receptor M1
Semen Myristicae (SM)	MOL009264	tetrahydrofuroguaiacin B	Estrogen receptor
Semen Myristicae (SM)	MOL009264	tetrahydrofuroguaiacin B	Sodium channel protein type 5 subunit alpha
Semen Myristicae (SM)	MOL009264	tetrahydrofuroguaiacin B	Prostaglandin G/H synthase 2
Semen Myristicae (SM)	MOL009264	tetrahydrofuroguaiacin B	Alpha-1B adrenergic receptor
Semen Myristicae (SM)	MOL009264	tetrahydrofuroguaiacin B	Beta-2 adrenergic receptor
Semen Myristicae (SM)	MOL009264	tetrahydrofuroguaiacin B	Heat shock protein HSP 90
Semen Myristicae (SM)	MOL009264	tetrahydrofuroguaiacin B	Proto-oncogene serine/threonine-protein kinase Pim-1
Semen Myristicae (SM)	MOL009264	tetrahydrofuroguaiacin B	Nuclear receptor coactivator 2
Semen Myristicae (SM)	MOL009264	tetrahydrofuroguaiacin B	Calmodulin
Semen Myristicae (SM)	MOL009265	threo-austrobailignan-5	Nitric oxide synthase, inducible
Semen Myristicae (SM)	MOL009265	threo-austrobailignan-5	Prostaglandin G/H synthase 1
Semen Myristicae (SM)	MOL009265	threo-austrobailignan-5	Muscarinic acetylcholine receptor M1
Semen Myristicae (SM)	MOL009265	threo-austrobailignan-5	Estrogen receptor
Semen Myristicae (SM)	MOL009265	threo-austrobailignan-5	Androgen receptor
Semen Myristicae (SM)	MOL009265	threo-austrobailignan-5	Sodium channel protein type 5 subunit alpha
Semen Myristicae (SM)	MOL009265	threo-austrobailignan-5	Peroxisome proliferator activated receptor gamma
Semen Myristicae (SM)	MOL009265	threo-austrobailignan-5	Coagulation factor Xa
Semen Myristicae (SM)	MOL009265	threo-austrobailignan-5	Prostaglandin G/H synthase 2
Semen Myristicae (SM)	MOL009265	threo-austrobailignan-5	Retinoic acid receptor RXR-alpha
Semen Myristicae (SM)	MOL009265	threo-austrobailignan-5	Alpha-1B adrenergic receptor
Semen Myristicae (SM)	MOL009265	threo-austrobailignan-5	Beta-2 adrenergic receptor
Semen Myristicae (SM)	MOL009265	threo-austrobailignan-5	Estrogen receptor beta
Semen Myristicae (SM)	MOL009265	threo-austrobailignan-5	Mitogen-activated protein kinase 14
Semen Myristicae (SM)	MOL009265	threo-austrobailignan-5	Glycogen synthase kinase-3 beta

Semen Myristicae (SM)	MOL009265	threo-austrobailignan-5	Heat shock protein HSP 90
Semen Myristicae (SM)	MOL009265	threo-austrobailignan-5	Serine/threonine-protein kinase Chk1
Semen Myristicae (SM)	MOL009265	threo-austrobailignan-5	Ig gamma-1 chain C region
Semen Myristicae (SM)	MOL009265	threo-austrobailignan-5	Proto-oncogene serine/threonine-protein kinase Pim-1
Semen Myristicae (SM)	MOL009265	threo-austrobailignan-5	Cyclin-A2
Semen Myristicae (SM)	MOL009265	threo-austrobailignan-5	Nuclear receptor coactivator 2
Semen Myristicae (SM)	MOL009265	threo-austrobailignan-5	Calmodulin
Cortex Cinnamomi (CC)	MOL000131	EIC	Prostaglandin G/H synthase 1
Cortex Cinnamomi (CC)	MOL000131	EIC	Prostaglandin G/H synthase 2
Cortex Cinnamomi (CC)	MOL000131	EIC	Retinoic acid receptor RXR-alpha
Cortex Cinnamomi (CC)	MOL000131	EIC	Nuclear receptor coactivator 2
Cortex Cinnamomi (CC)	MOL000131	EIC	Lysozyme
Cortex Cinnamomi (CC)	MOL000131	EIC	Nicotinate-nucleotide--dimethylbenzimidazole phosphoribosyltransferase
Cortex Cinnamomi (CC)	MOL000131	EIC	Sodium-dependent noradrenaline transporter
Cortex Cinnamomi (CC)	MOL000131	EIC	Ig gamma-1 chain C region
Cortex Cinnamomi (CC)	MOL000131	EIC	Gamma-aminobutyric-acid receptor alpha-2 subunit
Cortex Cinnamomi (CC)	MOL000131	EIC	Gamma-aminobutyric acid receptor subunit alpha-1
Cortex Cinnamomi (CC)	MOL000131	EIC	Cytochrome P450-cam
Cortex Cinnamomi (CC)	MOL000131	EIC	Transient receptor potential cation channel subfamily V member 1
Cortex Cinnamomi (CC)	MOL000131	EIC	Muscarinic acetylcholine receptor M1
Cortex Cinnamomi (CC)	MOL000131	EIC	Muscarinic acetylcholine receptor M2
Cortex Cinnamomi (CC)	MOL000131	EIC	Gamma-aminobutyric-acid receptor subunit alpha-6
Cortex Cinnamomi (CC)	MOL000208	()-Aromadendrene	Muscarinic acetylcholine receptor M3
Cortex Cinnamomi (CC)	MOL000208	()-Aromadendrene	Muscarinic acetylcholine receptor M2
Cortex Cinnamomi (CC)	MOL000208	()-Aromadendrene	Muscarinic acetylcholine receptor M1
Cortex Cinnamomi (CC)	MOL000208	()-Aromadendrene	Alcohol dehydrogenase 1C
Cortex Cinnamomi (CC)	MOL000208	()-Aromadendrene	Lysozyme
Cortex Cinnamomi (CC)	MOL000208	()-Aromadendrene	Nicotinate-nucleotide--dimethylbenzimidazole phosphoribosyltransferase
Cortex Cinnamomi (CC)	MOL000266	beta-Cubebene	Muscarinic acetylcholine receptor M1
Cortex Cinnamomi (CC)	MOL000266	beta-Cubebene	Muscarinic acetylcholine receptor M2
Cortex Cinnamomi (CC)	MOL000266	beta-Cubebene	Gamma-aminobutyric acid receptor subunit alpha-1
Cortex Cinnamomi (CC)	MOL000266	beta-Cubebene	Prostaglandin G/H synthase 2
Cortex Cinnamomi (CC)	MOL000266	beta-Cubebene	Neuronal acetylcholine receptor subunit alpha-2
Cortex Cinnamomi (CC)	MOL000266	beta-Cubebene	Sodium-dependent noradrenaline transporter
Cortex Cinnamomi (CC)	MOL002697	junipene	Muscarinic acetylcholine receptor M3
Cortex Cinnamomi (CC)	MOL002697	junipene	Muscarinic acetylcholine receptor M2
Cortex Cinnamomi (CC)	MOL002697	junipene	Gamma-aminobutyric acid receptor subunit alpha-1
Cortex Cinnamomi (CC)	MOL002697	junipene	Gamma-aminobutyric-acid receptor alpha-2 subunit
Cortex Cinnamomi (CC)	MOL002697	junipene	Alpha-1B adrenergic receptor
Cortex Cinnamomi (CC)	MOL002697	junipene	Neuronal acetylcholine receptor subunit alpha-2
Cortex Cinnamomi (CC)	MOL003522	()-Sativene	Muscarinic acetylcholine receptor M3
Cortex Cinnamomi (CC)	MOL003522	()-Sativene	Muscarinic acetylcholine receptor M1
Cortex Cinnamomi (CC)	MOL003522	()-Sativene	Prostaglandin G/H synthase 2
Cortex Cinnamomi (CC)	MOL003522	()-Sativene	Gamma-aminobutyric-acid receptor alpha-2 subunit
Cortex Cinnamomi (CC)	MOL003522	()-Sativene	Gamma-aminobutyric-acid receptor alpha-3 subunit

Cortex Cinnamomi (CC)	MOL003522	()-Sativene	Muscarinic acetylcholine receptor M2
Cortex Cinnamomi (CC)	MOL003522	()-Sativene	Alpha-1B adrenergic receptor
Cortex Cinnamomi (CC)	MOL003522	()-Sativene	Neuronal acetylcholine receptor subunit alpha-2
Cortex Cinnamomi (CC)	MOL003522	()-Sativene	Gamma-aminobutyric acid receptor subunit alpha-1
Cortex Cinnamomi (CC)	MOL003522	()-Sativene	Neuronal acetylcholine receptor protein, alpha-7 chain
Cortex Cinnamomi (CC)	MOL003522	()-Sativene	Nuclear receptor coactivator 2
Cortex Cinnamomi (CC)	MOL003538	()-Ledene	Nuclear receptor coactivator 2
Cortex Cinnamomi (CC)	MOL003538	()-Ledene	Muscarinic acetylcholine receptor M3
Cortex Cinnamomi (CC)	MOL003538	()-Ledene	Muscarinic acetylcholine receptor M1
Cortex Cinnamomi (CC)	MOL002003	(-)-Caryophyllene oxide	Muscarinic acetylcholine receptor M3
Cortex Cinnamomi (CC)	MOL002003	(-)-Caryophyllene oxide	Thrombin
Cortex Cinnamomi (CC)	MOL002003	(-)-Caryophyllene oxide	Muscarinic acetylcholine receptor M1
Cortex Cinnamomi (CC)	MOL002003	(-)-Caryophyllene oxide	Prostaglandin G/H synthase 2
Cortex Cinnamomi (CC)	MOL002003	(-)-Caryophyllene oxide	Acetylcholinesterase
Cortex Cinnamomi (CC)	MOL002003	(-)-Caryophyllene oxide	Muscarinic acetylcholine receptor M2
Cortex Cinnamomi (CC)	MOL002003	(-)-Caryophyllene oxide	Alpha-1B adrenergic receptor
Cortex Cinnamomi (CC)	MOL002003	(-)-Caryophyllene oxide	Gamma-aminobutyric acid receptor subunit alpha-1
Cortex Cinnamomi (CC)	MOL002003	(-)-Caryophyllene oxide	Dipeptidyl peptidase IV
Cortex Cinnamomi (CC)	MOL002003	(-)-Caryophyllene oxide	Gamma-aminobutyric-acid receptor subunit alpha-6
Cortex Cinnamomi (CC)	MOL000057	DIBP	Muscarinic acetylcholine receptor M3
Cortex Cinnamomi (CC)	MOL000057	DIBP	Muscarinic acetylcholine receptor M1
Cortex Cinnamomi (CC)	MOL000057	DIBP	Sodium-dependent noradrenaline transporter
Cortex Cinnamomi (CC)	MOL000057	DIBP	Sodium-dependent dopamine
Cortex Cinnamomi (CC)	MOL000057	DIBP	Beta-2 adrenergic receptor
Cortex Cinnamomi (CC)	MOL000057	DIBP	Sodium-dependent serotonin transporter
Cortex Cinnamomi (CC)	MOL000057	DIBP	Gamma-aminobutyric acid receptor subunit alpha-1
Cortex Cinnamomi (CC)	MOL000057	DIBP	Muscarinic acetylcholine receptor M2
Cortex Cinnamomi (CC)	MOL000057	DIBP	5-hydroxytryptamine 2A receptor
Cortex Cinnamomi (CC)	MOL000057	DIBP	Progesterone receptor
Cortex Cinnamomi (CC)	MOL000057	DIBP	Mineralocorticoid receptor
Cortex Cinnamomi (CC)	MOL000057	DIBP	Glucocorticoid receptor
Cortex Cinnamomi (CC)	MOL000057	DIBP	Nuclear receptor coactivator 2
Cortex Cinnamomi (CC)	MOL000057	DIBP	Retinoic acid receptor RXR-alpha
Cortex Cinnamomi (CC)	MOL000612	(-)-alpha-cedrene	Muscarinic acetylcholine receptor M3
Cortex Cinnamomi (CC)	MOL000612	(-)-alpha-cedrene	Prostaglandin G/H synthase 2
Cortex Cinnamomi (CC)	MOL000612	(-)-alpha-cedrene	Gamma-aminobutyric-acid receptor alpha-2 subunit
Cortex Cinnamomi (CC)	MOL000612	(-)-alpha-cedrene	Retinoic acid receptor RXR-alpha
Cortex Cinnamomi (CC)	MOL000612	(-)-alpha-cedrene	Gamma-aminobutyric acid receptor subunit alpha-1
Cortex Cinnamomi (CC)	MOL000612	(-)-alpha-cedrene	Nuclear receptor coactivator 2
Cortex Cinnamomi (CC)	MOL000612	(-)-alpha-cedrene	Muscarinic acetylcholine receptor M1
Cortex Cinnamomi (CC)	MOL000612	(-)-alpha-cedrene	Alcohol dehydrogenase 1B
Cortex Cinnamomi (CC)	MOL000612	(-)-alpha-cedrene	Alcohol dehydrogenase 1C
Cortex Cinnamomi (CC)	MOL000675	oleic acid	Prostaglandin G/H synthase 1
Cortex Cinnamomi (CC)	MOL000675	oleic acid	Nuclear receptor coactivator 2
Cortex Cinnamomi (CC)	MOL000675	oleic acid	Prostaglandin G/H synthase 2
Cortex Cinnamomi (CC)	MOL000675	oleic acid	Alcohol dehydrogenase 1B
Cortex Cinnamomi (CC)	MOL000675	oleic acid	Alcohol dehydrogenase 1C
Cortex Cinnamomi (CC)	MOL000675	oleic acid	Alcohol dehydrogenase 1A
Cortex Cinnamomi (CC)	MOL000675	oleic acid	Lysozyme
Cortex Cinnamomi (CC)	MOL000675	oleic acid	Nicotinate-nucleotide--dimethylbenzimidazole phosphoribosyltransferase
Cortex Cinnamomi (CC)	MOL000675	oleic acid	Trypsin-3
Cortex Cinnamomi (CC)	MOL000675	oleic acid	Retinoic acid receptor RXR-alpha

Cortex Cinnamomi (CC)	MOL000675	oleic acid	Cytochrome P450-cam
Cortex Cinnamomi (CC)	MOL000675	oleic acid	Urokinase-type plasminogen activator
Cortex Cinnamomi (CC)	MOL000675	oleic acid	Superoxide dismutase [Cu-Zn]
Cortex Cinnamomi (CC)	MOL000675	oleic acid	Catalase
Cortex Cinnamomi (CC)	MOL000675	oleic acid	Telomerase protein component 1
Cortex Cinnamomi (CC)	MOL000675	oleic acid	Endothelin-1
Cortex Cinnamomi (CC)	MOL000675	oleic acid	Receptor tyrosine-protein kinase erbB-2
Cortex Cinnamomi (CC)	MOL000675	oleic acid	Peroxisome proliferator-activated receptor gamma
Cortex Cinnamomi (CC)	MOL000675	oleic acid	Lipoprotein lipase
Cortex Cinnamomi (CC)	MOL000675	oleic acid	Neuromodulin
Cortex Cinnamomi (CC)	MOL000675	oleic acid	Plasminogen activator inhibitor 1
Cortex Cinnamomi (CC)	MOL000675	oleic acid	Brain-derived neurotrophic factor
Cortex Cinnamomi (CC)	MOL000675	oleic acid	3-hydroxy-3-methylglutaryl-coenzyme A reductase
Cortex Cinnamomi (CC)	MOL000675	oleic acid	Myeloperoxidase
Cortex Cinnamomi (CC)	MOL000675	oleic acid	Peroxisome proliferator-activated receptor alpha
Cortex Cinnamomi (CC)	MOL000675	oleic acid	Peroxisome proliferator-activated receptor delta
Cortex Cinnamomi (CC)	MOL000675	oleic acid	C-reactive protein
Cortex Cinnamomi (CC)	MOL000675	oleic acid	Serum paraoxonase/arylesterase 1
Cortex Cinnamomi (CC)	MOL000675	oleic acid	Insulin
Cortex Cinnamomi (CC)	MOL000675	oleic acid	Plasminogen
Cortex Cinnamomi (CC)	MOL000675	oleic acid	Fatty acid-binding protein, liver
Cortex Cinnamomi (CC)	MOL000675	oleic acid	Retinol-binding protein 2
Cortex Cinnamomi (CC)	MOL000675	oleic acid	Glucagon
Cortex Cinnamomi (CC)	MOL000675	oleic acid	Glutamyl aminopeptidase
Cortex Cinnamomi (CC)	MOL000675	oleic acid	Mitochondrial uncoupling protein 2
Cortex Cinnamomi (CC)	MOL000675	oleic acid	Sterol O-acyltransferase 1
Cortex Cinnamomi (CC)	MOL000675	oleic acid	Cholecystokinin
Cortex Cinnamomi (CC)	MOL000675	oleic acid	Cbp/p300-interacting transactivator 1
Cortex Cinnamomi (CC)	MOL000675	oleic acid	BDNF/NT-3 growth factors receptor
Cortex Cinnamomi (CC)	MOL000675	oleic acid	Pancreas/duodenum homeobox protein
Cortex Cinnamomi (CC)	MOL000675	oleic acid	Solute carrier family 2, facilitated glucose transporter member 2
Cortex Cinnamomi (CC)	MOL000675	oleic acid	Peptidyl-glycine alpha-amidating monooxygenase
Cortex Cinnamomi (CC)	MOL000675	oleic acid	Acyl-CoA desaturase
Cortex Cinnamomi (CC)	MOL000675	oleic acid	Mitochondrial uncoupling protein 3
Cortex Cinnamomi (CC)	MOL000675	oleic acid	Cholesteryl ester transfer protein
Cortex Cinnamomi (CC)	MOL000675	oleic acid	Peptide YY
Cortex Cinnamomi (CC)	MOL000675	oleic acid	Aspartyl aminopeptidase
Cortex Cinnamomi (CC)	MOL000675	oleic acid	Cell-death-related nuclease 7
Pericarpium Papaveris (PP)	MOL006980	papaverine	Prostaglandin G/H synthase 1
Pericarpium Papaveris (PP)	MOL006980	papaverine	Muscarinic acetylcholine receptor M3
Pericarpium Papaveris (PP)	MOL006980	papaverine	Potassium voltage-gated channel subfamily H member 2
Pericarpium Papaveris (PP)	MOL006980	papaverine	Sodium channel protein type 5 subunit alpha
Pericarpium Papaveris (PP)	MOL006980	papaverine	Coagulation factor Xa
Pericarpium Papaveris (PP)	MOL006980	papaverine	Muscarinic acetylcholine receptor M5
Pericarpium Papaveris (PP)	MOL006980	papaverine	Prostaglandin G/H synthase 2
Pericarpium Papaveris (PP)	MOL006980	papaverine	Retinoic acid receptor RXR-alpha
Pericarpium Papaveris (PP)	MOL006980	papaverine	Alpha-1B adrenergic receptor
Pericarpium Papaveris (PP)	MOL006980	papaverine	Beta-2 adrenergic receptor
Pericarpium Papaveris (PP)	MOL006980	papaverine	Sodium-dependent serotonin transporter
Pericarpium Papaveris (PP)	MOL006980	papaverine	Heat shock protein HSP 90
Pericarpium Papaveris (PP)	MOL006980	papaverine	mRNA of PKA Catalytic Subunit C-
Pericarpium Papaveris (PP)	MOL006980	papaverine	Retinoic acid receptor RXR-beta
Pericarpium Papaveris (PP)	MOL006980	papaverine	Nuclear receptor coactivator 2
Pericarpium Papaveris (PP)	MOL006980	papaverine	cAMP and cAMP-inhibited cGMP 3',5'-cyclic phosphodiesterase 10A

Pericarpium Papaveris (PP)	MOL006980	papaverine	Calmodulin
Pericarpium Papaveris (PP)	MOL006980	papaverine	Tyrosyl-DNA phosphodiesterase 1
Pericarpium Papaveris (PP)	MOL006980	papaverine	Lipoprotein lipase
Pericarpium Papaveris (PP)	MOL006980	papaverine	Amine oxidase [flavin-containing] A
Pericarpium Papaveris (PP)	MOL006980	papaverine	ADM
Pericarpium Papaveris (PP)	MOL006982	codeine	Dopamine D1 receptor
Pericarpium Papaveris (PP)	MOL006982	codeine	Muscarinic acetylcholine receptor M3
Pericarpium Papaveris (PP)	MOL006982	codeine	Muscarinic acetylcholine receptor M1
Pericarpium Papaveris (PP)	MOL006982	codeine	Androgen receptor
Pericarpium Papaveris (PP)	MOL006982	codeine	D(1B) dopamine receptor
Pericarpium Papaveris (PP)	MOL006982	codeine	Sodium channel protein type 5 subunit alpha
Pericarpium Papaveris (PP)	MOL006982	codeine	Muscarinic acetylcholine receptor M5
Pericarpium Papaveris (PP)	MOL006982	codeine	5-hydroxytryptamine receptor 3A
Pericarpium Papaveris (PP)	MOL006982	codeine	Muscarinic acetylcholine receptor M4
Pericarpium Papaveris (PP)	MOL006982	codeine	Delta-type opioid receptor
Pericarpium Papaveris (PP)	MOL006982	codeine	Acetylcholinesterase
Pericarpium Papaveris (PP)	MOL006982	codeine	Histamine H1 receptor
Pericarpium Papaveris (PP)	MOL006982	codeine	5-hydroxytryptamine 2A receptor
Pericarpium Papaveris (PP)	MOL006982	codeine	Alpha-1A adrenergic receptor
Pericarpium Papaveris (PP)	MOL006982	codeine	Muscarinic acetylcholine receptor M2
Pericarpium Papaveris (PP)	MOL006982	codeine	Alpha-1B adrenergic receptor
Pericarpium Papaveris (PP)	MOL006982	codeine	Kappa-type opioid receptor
Pericarpium Papaveris (PP)	MOL006982	codeine	Beta-2 adrenergic receptor
Pericarpium Papaveris (PP)	MOL006982	codeine	Alpha-1D adrenergic receptor
Pericarpium Papaveris (PP)	MOL006982	codeine	Neuronal acetylcholine receptor subunit alpha-2
Pericarpium Papaveris (PP)	MOL006982	codeine	Sodium-dependent serotonin transporter
Pericarpium Papaveris (PP)	MOL006982	codeine	D(2) dopamine receptor
Pericarpium Papaveris (PP)	MOL006982	codeine	Mu-type opioid receptor
Pericarpium Papaveris (PP)	MOL006982	codeine	Neuronal acetylcholine receptor protein, alpha-7 chain
Pericarpium Papaveris (PP)	MOL000787	Fumarine	Prostaglandin G/H synthase 1
Pericarpium Papaveris (PP)	MOL000787	Fumarine	Muscarinic acetylcholine receptor M3
Pericarpium Papaveris (PP)	MOL000787	Fumarine	Potassium voltage-gated channel subfamily H member 2
Pericarpium Papaveris (PP)	MOL000787	Fumarine	Muscarinic acetylcholine receptor M1
Pericarpium Papaveris (PP)	MOL000787	Fumarine	Sodium channel protein type 5 subunit alpha
Pericarpium Papaveris (PP)	MOL000787	Fumarine	Coagulation factor Xa
Pericarpium Papaveris (PP)	MOL000787	Fumarine	Muscarinic acetylcholine receptor M5
Pericarpium Papaveris (PP)	MOL000787	Fumarine	Prostaglandin G/H synthase 2
Pericarpium Papaveris (PP)	MOL000787	Fumarine	5-hydroxytryptamine receptor 3A
Pericarpium Papaveris (PP)	MOL000787	Fumarine	Coagulation factor VII
Pericarpium Papaveris (PP)	MOL000787	Fumarine	Muscarinic acetylcholine receptor M4
Pericarpium Papaveris (PP)	MOL000787	Fumarine	Delta-type opioid receptor
Pericarpium Papaveris (PP)	MOL000787	Fumarine	5-hydroxytryptamine 2A receptor
Pericarpium Papaveris (PP)	MOL000787	Fumarine	Alpha-1B adrenergic receptor
Pericarpium Papaveris (PP)	MOL000787	Fumarine	Beta-2 adrenergic receptor
Pericarpium Papaveris (PP)	MOL000787	Fumarine	Alpha-1D adrenergic receptor
Pericarpium Papaveris (PP)	MOL000787	Fumarine	Mu-type opioid receptor
Pericarpium Papaveris (PP)	MOL000787	Fumarine	Heat shock protein HSP 90
Pericarpium Papaveris (PP)	MOL000787	Fumarine	mRNA of PKA Catalytic Subunit C-
Pericarpium Papaveris (PP)	MOL000787	Fumarine	Calmodulin
Pericarpium Papaveris (PP)	MOL000787	Fumarine	Sodium-dependent serotonin transporter
Pericarpium Papaveris (PP)	MOL000787	Fumarine	Voltage-dependent L-type calcium channel subunit alpha-1S
Pericarpium Papaveris (PP)	MOL000787	Fumarine	CGMP-inhibited 3',5'-cyclic phosphodiesterase A
Pericarpium Papaveris (PP)	MOL000787	Fumarine	Sodium-dependent dopamine
Pericarpium Papaveris (PP)	MOL000787	Fumarine	Type IV phosphodiesterase
Pericarpium Papaveris (PP)	MOL000787	Fumarine	DNA topoisomerase II
Pericarpium Papaveris (PP)	MOL000787	Fumarine	Dopamine D1 receptor

Pericarpium Papaveris (PP)	MOL000787	Fumarine	Vascular endothelial growth factor receptor 2
Pericarpium Papaveris (PP)	MOL009324	Cryptogenin	Mineralocorticoid receptor
Pericarpium Papaveris (PP)	MOL009327	Noskapin	Thrombin
Pericarpium Papaveris (PP)	MOL009327	Noskapin	Potassium voltage-gated channel subfamily H member 2
Pericarpium Papaveris (PP)	MOL009327	Noskapin	Androgen receptor
Pericarpium Papaveris (PP)	MOL009327	Noskapin	Sodium channel protein type 5 subunit alpha
Pericarpium Papaveris (PP)	MOL009327	Noskapin	Coagulation factor Xa
Pericarpium Papaveris (PP)	MOL009327	Noskapin	Muscarinic acetylcholine receptor M5
Pericarpium Papaveris (PP)	MOL009327	Noskapin	Prostaglandin G/H synthase 2
Pericarpium Papaveris (PP)	MOL009327	Noskapin	Vascular endothelial growth factor receptor 2
Pericarpium Papaveris (PP)	MOL009327	Noskapin	Acetylcholinesterase
Pericarpium Papaveris (PP)	MOL009327	Noskapin	DNA topoisomerase II
Pericarpium Papaveris (PP)	MOL009327	Noskapin	Heat shock protein HSP 90
Pericarpium Papaveris (PP)	MOL009327	Noskapin	Trypsin-1
Pericarpium Papaveris (PP)	MOL009327	Noskapin	Calcium-activated potassium channel subunit alpha 1
Pericarpium Papaveris (PP)	MOL009327	Noskapin	Calmodulin
Pericarpium Papaveris (PP)	MOL009328	5-[[(1S)-6,7-dimethoxy-2-methyl-3,4-dihydro-1H-isoquinolin-1-yl]methyl]-2-methoxyphenol	Prostaglandin G/H synthase 1
Pericarpium Papaveris (PP)	MOL009328	5-[[(1S)-6,7-dimethoxy-2-methyl-3,4-dihydro-1H-isoquinolin-1-yl]methyl]-2-methoxyphenol	Dopamine D1 receptor
Pericarpium Papaveris (PP)	MOL009328	5-[[(1S)-6,7-dimethoxy-2-methyl-3,4-dihydro-1H-isoquinolin-1-yl]methyl]-2-methoxyphenol	Muscarinic acetylcholine receptor M3
Pericarpium Papaveris (PP)	MOL009328	5-[[(1S)-6,7-dimethoxy-2-methyl-3,4-dihydro-1H-isoquinolin-1-yl]methyl]-2-methoxyphenol	Potassium voltage-gated channel subfamily H member 2
Pericarpium Papaveris (PP)	MOL009328	5-[[(1S)-6,7-dimethoxy-2-methyl-3,4-dihydro-1H-isoquinolin-1-yl]methyl]-2-methoxyphenol	Muscarinic acetylcholine receptor M1
Pericarpium Papaveris (PP)	MOL009328	5-[[(1S)-6,7-dimethoxy-2-methyl-3,4-dihydro-1H-isoquinolin-1-yl]methyl]-2-methoxyphenol	Beta-1 adrenergic receptor
Pericarpium Papaveris (PP)	MOL009328	5-[[(1S)-6,7-dimethoxy-2-methyl-3,4-dihydro-1H-isoquinolin-1-yl]methyl]-2-methoxyphenol	Sodium channel protein type 5 subunit alpha
Pericarpium Papaveris (PP)	MOL009328	5-[[(1S)-6,7-dimethoxy-2-methyl-3,4-dihydro-1H-isoquinolin-1-yl]methyl]-2-methoxyphenol	Coagulation factor Xa
Pericarpium Papaveris (PP)	MOL009328	5-[[(1S)-6,7-dimethoxy-2-methyl-3,4-dihydro-1H-isoquinolin-1-yl]methyl]-2-methoxyphenol	Muscarinic acetylcholine receptor M5
Pericarpium Papaveris (PP)	MOL009328	5-[[(1S)-6,7-dimethoxy-2-methyl-3,4-dihydro-1H-isoquinolin-1-yl]methyl]-2-methoxyphenol	Prostaglandin G/H synthase 2

Pericarpium Papaveris (PP)	MOL009328	5-[[(1S)-6,7-dimethoxy-2-methyl-3,4-dihydro-1H-isoquinolin-1-yl]methyl]-2-methoxyphenol	Alpha-2A adrenergic receptor
Pericarpium Papaveris (PP)	MOL009328	5-[[(1S)-6,7-dimethoxy-2-methyl-3,4-dihydro-1H-isoquinolin-1-yl]methyl]-2-methoxyphenol	Alpha-2C adrenergic receptor
Pericarpium Papaveris (PP)	MOL009328	5-[[(1S)-6,7-dimethoxy-2-methyl-3,4-dihydro-1H-isoquinolin-1-yl]methyl]-2-methoxyphenol	D(4) dopamine receptor
Pericarpium Papaveris (PP)	MOL009328	5-[[(1S)-6,7-dimethoxy-2-methyl-3,4-dihydro-1H-isoquinolin-1-yl]methyl]-2-methoxyphenol	Muscarinic acetylcholine receptor M4
Pericarpium Papaveris (PP)	MOL009328	5-[[(1S)-6,7-dimethoxy-2-methyl-3,4-dihydro-1H-isoquinolin-1-yl]methyl]-2-methoxyphenol	Retinoic acid receptor RXR-alpha
Pericarpium Papaveris (PP)	MOL009328	5-[[(1S)-6,7-dimethoxy-2-methyl-3,4-dihydro-1H-isoquinolin-1-yl]methyl]-2-methoxyphenol	Delta-type opioid receptor
Pericarpium Papaveris (PP)	MOL009328	5-[[(1S)-6,7-dimethoxy-2-methyl-3,4-dihydro-1H-isoquinolin-1-yl]methyl]-2-methoxyphenol	CGMP-inhibited 3',5'-cyclic phosphodiesterase A
Pericarpium Papaveris (PP)	MOL009328	5-[[(1S)-6,7-dimethoxy-2-methyl-3,4-dihydro-1H-isoquinolin-1-yl]methyl]-2-methoxyphenol	5-hydroxytryptamine 2A receptor
Pericarpium Papaveris (PP)	MOL009328	5-[[(1S)-6,7-dimethoxy-2-methyl-3,4-dihydro-1H-isoquinolin-1-yl]methyl]-2-methoxyphenol	Sodium-dependent noradrenaline transporter
Pericarpium Papaveris (PP)	MOL009328	5-[[(1S)-6,7-dimethoxy-2-methyl-3,4-dihydro-1H-isoquinolin-1-yl]methyl]-2-methoxyphenol	Alpha-1A adrenergic receptor
Pericarpium Papaveris (PP)	MOL009328	5-[[(1S)-6,7-dimethoxy-2-methyl-3,4-dihydro-1H-isoquinolin-1-yl]methyl]-2-methoxyphenol	5-hydroxytryptamine 2C receptor
Pericarpium Papaveris (PP)	MOL009328	5-[[(1S)-6,7-dimethoxy-2-methyl-3,4-dihydro-1H-isoquinolin-1-yl]methyl]-2-methoxyphenol	Muscarinic acetylcholine receptor M2
Pericarpium Papaveris (PP)	MOL009328	5-[[(1S)-6,7-dimethoxy-2-methyl-3,4-dihydro-1H-isoquinolin-1-yl]methyl]-2-methoxyphenol	Alpha-2B adrenergic receptor
Pericarpium Papaveris (PP)	MOL009328	5-[[(1S)-6,7-dimethoxy-2-methyl-3,4-dihydro-1H-isoquinolin-1-yl]methyl]-2-methoxyphenol	Alpha-1B adrenergic receptor
Pericarpium Papaveris (PP)	MOL009328	5-[[(1S)-6,7-dimethoxy-2-methyl-3,4-dihydro-1H-isoquinolin-1-yl]methyl]-2-methoxyphenol	D(3) dopamine receptor

Pericarpium Papaveris (PP)	MOL009328	5-[[(1S)-6,7-dimethoxy-2-methyl-3,4-dihydro-1H-isoquinolin-1-yl]methyl]-2-methoxyphenol	Sodium-dependent dopamine transporter
Pericarpium Papaveris (PP)	MOL009328	5-[[(1S)-6,7-dimethoxy-2-methyl-3,4-dihydro-1H-isoquinolin-1-yl]methyl]-2-methoxyphenol	Beta-2 adrenergic receptor
Pericarpium Papaveris (PP)	MOL009328	5-[[(1S)-6,7-dimethoxy-2-methyl-3,4-dihydro-1H-isoquinolin-1-yl]methyl]-2-methoxyphenol	Alpha-1D adrenergic receptor
Pericarpium Papaveris (PP)	MOL009328	5-[[(1S)-6,7-dimethoxy-2-methyl-3,4-dihydro-1H-isoquinolin-1-yl]methyl]-2-methoxyphenol	Sodium-dependent serotonin transporter
Pericarpium Papaveris (PP)	MOL009328	5-[[(1S)-6,7-dimethoxy-2-methyl-3,4-dihydro-1H-isoquinolin-1-yl]methyl]-2-methoxyphenol	D(2) dopamine receptor
Pericarpium Papaveris (PP)	MOL009328	5-[[(1S)-6,7-dimethoxy-2-methyl-3,4-dihydro-1H-isoquinolin-1-yl]methyl]-2-methoxyphenol	Mu-type opioid receptor
Pericarpium Papaveris (PP)	MOL009328	5-[[(1S)-6,7-dimethoxy-2-methyl-3,4-dihydro-1H-isoquinolin-1-yl]methyl]-2-methoxyphenol	Pregnane X receptor
Pericarpium Papaveris (PP)	MOL009328	5-[[(1S)-6,7-dimethoxy-2-methyl-3,4-dihydro-1H-isoquinolin-1-yl]methyl]-2-methoxyphenol	Heat shock protein HSP 90
Pericarpium Papaveris (PP)	MOL009328	5-[[(1S)-6,7-dimethoxy-2-methyl-3,4-dihydro-1H-isoquinolin-1-yl]methyl]-2-methoxyphenol	Retinoic acid receptor RXR-beta
Pericarpium Papaveris (PP)	MOL009328	5-[[(1S)-6,7-dimethoxy-2-methyl-3,4-dihydro-1H-isoquinolin-1-yl]methyl]-2-methoxyphenol	Calmodulin
Pericarpium Papaveris (PP)	MOL009329	Narcein	Potassium voltage-gated channel subfamily H member 2
Pericarpium Papaveris (PP)	MOL009329	Narcein	Sodium channel protein type 5 subunit alpha
Pericarpium Papaveris (PP)	MOL009329	Narcein	Coagulation factor Xa
Pericarpium Papaveris (PP)	MOL009329	Narcein	Prostaglandin G/H synthase 2
Pericarpium Papaveris (PP)	MOL009329	Narcein	Coagulation factor VII
Pericarpium Papaveris (PP)	MOL009329	Narcein	Vascular endothelial growth factor receptor 2
Pericarpium Papaveris (PP)	MOL009329	Narcein	DNA topoisomerase II
Pericarpium Papaveris (PP)	MOL009329	Narcein	Heat shock protein HSP 90
Pericarpium Papaveris (PP)	MOL009329	Narcein	Calcium-activated potassium channel subunit alpha 1
Pericarpium Papaveris (PP)	MOL009329	Narcein	Calmodulin
Pericarpium Papaveris (PP)	MOL009330	Noscapine	Prostaglandin G/H synthase 1
Pericarpium Papaveris (PP)	MOL009330	Noscapine	Thrombin
Pericarpium Papaveris (PP)	MOL009330	Noscapine	Potassium voltage-gated channel subfamily H member 2
Pericarpium Papaveris (PP)	MOL009330	Noscapine	Androgen receptor
Pericarpium Papaveris (PP)	MOL009330	Noscapine	Sodium channel protein type 5 subunit alpha
Pericarpium Papaveris (PP)	MOL009330	Noscapine	Coagulation factor Xa
Pericarpium Papaveris (PP)	MOL009330	Noscapine	Muscarinic acetylcholine receptor M5

Pericarpium Papaveris (PP)	MOL009330	Noscapine	Prostaglandin G/H synthase 2
Pericarpium Papaveris (PP)	MOL009330	Noscapine	Vascular endothelial growth factor receptor 2
Pericarpium Papaveris (PP)	MOL009330	Noscapine	Acetylcholinesterase
Pericarpium Papaveris (PP)	MOL009330	Noscapine	DNA topoisomerase II
Pericarpium Papaveris (PP)	MOL009330	Noscapine	Heat shock protein HSP 90
Pericarpium Papaveris (PP)	MOL009330	Noscapine	Trypsin-1
Pericarpium Papaveris (PP)	MOL009330	Noscapine	Calcium-activated potassium channel subunit alpha 1
Pericarpium Papaveris (PP)	MOL009330	Noscapine	Calmodulin
Pericarpium Papaveris (PP)	MOL009331	Palaudine	Prostaglandin G/H synthase 1
Pericarpium Papaveris (PP)	MOL009331	Palaudine	Sodium channel protein type 5 subunit alpha
Pericarpium Papaveris (PP)	MOL009331	Palaudine	Coagulation factor Xa
Pericarpium Papaveris (PP)	MOL009331	Palaudine	Muscarinic acetylcholine receptor M5
Pericarpium Papaveris (PP)	MOL009331	Palaudine	Prostaglandin G/H synthase 2
Pericarpium Papaveris (PP)	MOL009331	Palaudine	Retinoic acid receptor RXR-alpha
Pericarpium Papaveris (PP)	MOL009331	Palaudine	Alpha-1B adrenergic receptor
Pericarpium Papaveris (PP)	MOL009331	Palaudine	Beta-2 adrenergic receptor
Pericarpium Papaveris (PP)	MOL009331	Palaudine	Heat shock protein HSP 90
Pericarpium Papaveris (PP)	MOL009331	Palaudine	Nuclear receptor coactivator 2
Pericarpium Papaveris (PP)	MOL009331	Palaudine	Calmodulin
Pericarpium Papaveris (PP)	MOL009335	Erythroculine	Prostaglandin G/H synthase 1
Pericarpium Papaveris (PP)	MOL009335	Erythroculine	Muscarinic acetylcholine receptor M3
Pericarpium Papaveris (PP)	MOL009335	Erythroculine	Muscarinic acetylcholine receptor M1
Pericarpium Papaveris (PP)	MOL009335	Erythroculine	Sodium channel protein type 5 subunit alpha
Pericarpium Papaveris (PP)	MOL009335	Erythroculine	Muscarinic acetylcholine receptor M5
Pericarpium Papaveris (PP)	MOL009335	Erythroculine	Prostaglandin G/H synthase 2
Pericarpium Papaveris (PP)	MOL009335	Erythroculine	Muscarinic acetylcholine receptor M4
Pericarpium Papaveris (PP)	MOL009335	Erythroculine	Delta-type opioid receptor
Pericarpium Papaveris (PP)	MOL009335	Erythroculine	Alpha-1B adrenergic receptor
Pericarpium Papaveris (PP)	MOL009335	Erythroculine	Beta-2 adrenergic receptor
Pericarpium Papaveris (PP)	MOL009335	Erythroculine	Alpha-1D adrenergic receptor
Pericarpium Papaveris (PP)	MOL009335	Erythroculine	Mu-type opioid receptor
Pericarpium Papaveris (PP)	MOL009335	Erythroculine	Neuronal acetylcholine receptor protein, alpha-7 chain
Pericarpium Papaveris (PP)	MOL009338	Norswertianin	Prostaglandin G/H synthase 1
Pericarpium Papaveris (PP)	MOL009338	Norswertianin	Androgen receptor
Pericarpium Papaveris (PP)	MOL009338	Norswertianin	Prostaglandin G/H synthase 2
Pericarpium Papaveris (PP)	MOL009338	Norswertianin	DNA topoisomerase II
Pericarpium Papaveris (PP)	MOL009338	Norswertianin	Heat shock protein HSP 90
Pericarpium Papaveris (PP)	MOL009338	Norswertianin	Phosphatidylinositol-4,5-bisphosphate 3-kinase catalytic subunit, gamma

Corresponding target gene symbols of active

Herb	MolId	MolName	Symbol
Radix Paeoniae Alba (RPA)	MOL001918	paeoniflorgenone	GABRA1
Radix Paeoniae Alba (RPA)	MOL001919	(3S,5R,8R,9R,10S,14S)-3,17-dihydroxy-4,4,8,10,14-pentamethyl-2,3,5,6,7,9-hexahydro-1H-cyclopenta[a]phenanthrene-15,16-dione	PGR
Radix Paeoniae Alba (RPA)	MOL001919	(3S,5R,8R,9R,10S,14S)-3,17-dihydroxy-4,4,8,10,14-pentamethyl-2,3,5,6,7,9-hexahydro-1H-cyclopenta[a]phenanthrene-15,16-dione	NR3C2
Radix Paeoniae Alba (RPA)	MOL001924	paeoniflorin	TNFAIP6
Radix Paeoniae Alba (RPA)	MOL001924	paeoniflorin	IL6R

Radix Paeoniae Alba (RPA)	MOL001924	paeoniflorin	CD14
Radix Paeoniae Alba (RPA)	MOL001924	paeoniflorin	LBP
Radix Paeoniae Alba (RPA)	MOL000211	Mairin	PGR
Radix Paeoniae Alba (RPA)	MOL000358	beta-sitosterol	PGR
Radix Paeoniae Alba (RPA)	MOL000358	beta-sitosterol	NCOA2
Radix Paeoniae Alba (RPA)	MOL000358	beta-sitosterol	PTGS1
Radix Paeoniae Alba (RPA)	MOL000358	beta-sitosterol	PTGS2
Radix Paeoniae Alba (RPA)	MOL000358	beta-sitosterol	HSP90AB1
Radix Paeoniae Alba (RPA)	MOL000358	beta-sitosterol	KCNH2
Radix Paeoniae Alba (RPA)	MOL000358	beta-sitosterol	DRD1
Radix Paeoniae Alba (RPA)	MOL000358	beta-sitosterol	CHRM3
Radix Paeoniae Alba (RPA)	MOL000358	beta-sitosterol	CHRM1
Radix Paeoniae Alba (RPA)	MOL000358	beta-sitosterol	SCN5A
Radix Paeoniae Alba (RPA)	MOL000358	beta-sitosterol	CHRM4
Radix Paeoniae Alba (RPA)	MOL000358	beta-sitosterol	ADRA1A
Radix Paeoniae Alba (RPA)	MOL000358	beta-sitosterol	CHRM2
Radix Paeoniae Alba (RPA)	MOL000358	beta-sitosterol	ADRA1B
Radix Paeoniae Alba (RPA)	MOL000358	beta-sitosterol	ADRB2
Radix Paeoniae Alba (RPA)	MOL000358	beta-sitosterol	CHRNA2
Radix Paeoniae Alba (RPA)	MOL000358	beta-sitosterol	SLC6A4
Radix Paeoniae Alba (RPA)	MOL000358	beta-sitosterol	OPRM1
Radix Paeoniae Alba (RPA)	MOL000358	beta-sitosterol	GABRA1
Radix Paeoniae Alba (RPA)	MOL000358	beta-sitosterol	BCL2
Radix Paeoniae Alba (RPA)	MOL000358	beta-sitosterol	BAX
Radix Paeoniae Alba (RPA)	MOL000358	beta-sitosterol	CASP9
Radix Paeoniae Alba (RPA)	MOL000358	beta-sitosterol	JUN
Radix Paeoniae Alba (RPA)	MOL000358	beta-sitosterol	CASP3
Radix Paeoniae Alba (RPA)	MOL000358	beta-sitosterol	CASP8
Radix Paeoniae Alba (RPA)	MOL000358	beta-sitosterol	PRKCA
Radix Paeoniae Alba (RPA)	MOL000358	beta-sitosterol	PON1
Radix Paeoniae Alba (RPA)	MOL000358	beta-sitosterol	MAP2
Radix Paeoniae Alba (RPA)	MOL000359	sitosterol	PGR
Radix Paeoniae Alba (RPA)	MOL000359	sitosterol	NCOA2
Radix Paeoniae Alba (RPA)	MOL000359	sitosterol	NR3C2
Radix Paeoniae Alba (RPA)	MOL000422	kaempferol	NOS2
Radix Paeoniae Alba (RPA)	MOL000422	kaempferol	PTGS1
Radix Paeoniae Alba (RPA)	MOL000422	kaempferol	AR
Radix Paeoniae Alba (RPA)	MOL000422	kaempferol	PPARG
Radix Paeoniae Alba (RPA)	MOL000422	kaempferol	PTGS2
Radix Paeoniae Alba (RPA)	MOL000422	kaempferol	HSP90AB1
Radix Paeoniae Alba (RPA)	MOL000422	kaempferol	NCOA2
Radix Paeoniae Alba (RPA)	MOL000422	kaempferol	DPP4
Radix Paeoniae Alba (RPA)	MOL000422	kaempferol	PRSS1
Radix Paeoniae Alba (RPA)	MOL000422	kaempferol	PGR
Radix Paeoniae Alba (RPA)	MOL000422	kaempferol	CHRM1
Radix Paeoniae Alba (RPA)	MOL000422	kaempferol	ACHE
Radix Paeoniae Alba (RPA)	MOL000422	kaempferol	SLC6A2
Radix Paeoniae Alba (RPA)	MOL000422	kaempferol	CHRM2
Radix Paeoniae Alba (RPA)	MOL000422	kaempferol	ADRA1B
Radix Paeoniae Alba (RPA)	MOL000422	kaempferol	GABRA1
Radix Paeoniae Alba (RPA)	MOL000422	kaempferol	F7
Radix Paeoniae Alba (RPA)	MOL000422	kaempferol	CAMKMT
Radix Paeoniae Alba (RPA)	MOL000422	kaempferol	RELA
Radix Paeoniae Alba (RPA)	MOL000422	kaempferol	IKBKB
Radix Paeoniae Alba (RPA)	MOL000422	kaempferol	AKT1
Radix Paeoniae Alba (RPA)	MOL000422	kaempferol	BCL2
Radix Paeoniae Alba (RPA)	MOL000422	kaempferol	BAX
Radix Paeoniae Alba (RPA)	MOL000422	kaempferol	TNFAIP6
Radix Paeoniae Alba (RPA)	MOL000422	kaempferol	JUN
Radix Paeoniae Alba (RPA)	MOL000422	kaempferol	AHSA1
Radix Paeoniae Alba (RPA)	MOL000422	kaempferol	CASP3
Radix Paeoniae Alba (RPA)	MOL000422	kaempferol	MAPK8
Radix Paeoniae Alba (RPA)	MOL000422	kaempferol	MMP1

Radix Paeoniae Alba (RPA)	MOL000422	kaempferol	STAT1
Radix Paeoniae Alba (RPA)	MOL000422	kaempferol	CDK1
Radix Paeoniae Alba (RPA)	MOL000422	kaempferol	PPARG
Radix Paeoniae Alba (RPA)	MOL000422	kaempferol	HMOX1
Radix Paeoniae Alba (RPA)	MOL000422	kaempferol	CYP3A4
Radix Paeoniae Alba (RPA)	MOL000422	kaempferol	CYP1A2
Radix Paeoniae Alba (RPA)	MOL000422	kaempferol	CYP1A1
Radix Paeoniae Alba (RPA)	MOL000422	kaempferol	ICAM1
Radix Paeoniae Alba (RPA)	MOL000422	kaempferol	SELE
Radix Paeoniae Alba (RPA)	MOL000422	kaempferol	VCAM1
Radix Paeoniae Alba (RPA)	MOL000422	kaempferol	NR1I2
Radix Paeoniae Alba (RPA)	MOL000422	kaempferol	CYP1B1
Radix Paeoniae Alba (RPA)	MOL000422	kaempferol	ALOX5
Radix Paeoniae Alba (RPA)	MOL000422	kaempferol	HAS2
Radix Paeoniae Alba (RPA)	MOL000422	kaempferol	GSTP1
Radix Paeoniae Alba (RPA)	MOL000422	kaempferol	AHR
Radix Paeoniae Alba (RPA)	MOL000422	kaempferol	PSMD3
Radix Paeoniae Alba (RPA)	MOL000422	kaempferol	SLC2A4
Radix Paeoniae Alba (RPA)	MOL000422	kaempferol	NR1I3
Radix Paeoniae Alba (RPA)	MOL000422	kaempferol	INSRR
Radix Paeoniae Alba (RPA)	MOL000422	kaempferol	DIO1
Radix Paeoniae Alba (RPA)	MOL000422	kaempferol	PPP3CA
Radix Paeoniae Alba (RPA)	MOL000422	kaempferol	GSTM1
Radix Paeoniae Alba (RPA)	MOL000422	kaempferol	GSTM2
Radix Paeoniae Alba (RPA)	MOL000422	kaempferol	AKR1C3
Radix Paeoniae Alba (RPA)	MOL000422	kaempferol	SLPI
Radix Paeoniae Alba (RPA)	MOL000492	(+)-catechin	PTGS1
Radix Paeoniae Alba (RPA)	MOL000492	(+)-catechin	ESR1
Radix Paeoniae Alba (RPA)	MOL000492	(+)-catechin	PTGS2
Radix Paeoniae Alba (RPA)	MOL000492	(+)-catechin	HSP90AB1
Radix Paeoniae Alba (RPA)	MOL000492	(+)-catechin	DPEP1
Radix Paeoniae Alba (RPA)	MOL000492	(+)-catechin	NCOA2
Radix Paeoniae Alba (RPA)	MOL000492	(+)-catechin	CAMKMT
Radix Paeoniae Alba (RPA)	MOL000492	(+)-catechin	RXRA
Radix Paeoniae Alba (RPA)	MOL000492	(+)-catechin	CAT
Radix Paeoniae Alba (RPA)	MOL000492	(+)-catechin	HAS2
Rhizoma Atractylodis Macrocephalae (RAM)	MOL000022	14-acetyl-12-senecioyl-2E,8Z,10E-atractylentriol	PTGS2
Rhizoma Atractylodis Macrocephalae (RAM)	MOL000033	(3S,8S,9S,10R,13R,14S,17R)-10,13-dimethyl-17-[(2R,5S)-5-propan-2-yloctan-2-yl]-2,3,4,7,8,9,11,12,14,15,16,17-dodecahydro-1H-cyclopenta[a]phenanthren-3-ol	PGR
Rhizoma Atractylodis Macrocephalae (RAM)	MOL000049	3β-acetoxyatractylone	CHRM3
Rhizoma Atractylodis Macrocephalae (RAM)	MOL000049	3β-acetoxyatractylone	CHRM1
Rhizoma Atractylodis Macrocephalae (RAM)	MOL000049	3β-acetoxyatractylone	AR
Rhizoma Atractylodis Macrocephalae (RAM)	MOL000049	3β-acetoxyatractylone	SCN5A
Rhizoma Atractylodis Macrocephalae (RAM)	MOL000049	3β-acetoxyatractylone	PTGS2
Rhizoma Atractylodis Macrocephalae (RAM)	MOL000049	3β-acetoxyatractylone	RXRA
Rhizoma Atractylodis Macrocephalae (RAM)	MOL000049	3β-acetoxyatractylone	ACHE
Rhizoma Atractylodis Macrocephalae (RAM)	MOL000049	3β-acetoxyatractylone	ADRA1A
Rhizoma Atractylodis Macrocephalae (RAM)	MOL000049	3β-acetoxyatractylone	CHRM2

Rhizoma Atractylodis Macrocephalae (RAM)	MOL000049	3β-acetoxyatractylone	ADRB2
Rhizoma Atractylodis Macrocephalae (RAM)	MOL000049	3β-acetoxyatractylone	OPRM1
Rhizoma Atractylodis Macrocephalae (RAM)	MOL000049	3β-acetoxyatractylone	GABRA1
Rhizoma Atractylodis Macrocephalae (RAM)	MOL000049	3β-acetoxyatractylone	DPP4
Rhizoma Atractylodis Macrocephalae (RAM)	MOL000072	8β-ethoxy atractylenolide Ⅲ	PTGS2
Rhizoma Atractylodis Macrocephalae (RAM)	MOL000072	8β-ethoxy atractylenolide Ⅲ	GABRA1
Rhizoma Atractylodis Macrocephalae (RAM)	MOL000072	8β-ethoxy atractylenolide Ⅲ	NCOA2
Rhizoma Atractylodis Macrocephalae (RAM)	MOL000072	8β-ethoxy atractylenolide Ⅲ	NCOA1
Radix Angelicae Sinensis (RAS)	MOL000358	beta-sitosterol	PGR
Radix Angelicae Sinensis (RAS)	MOL000358	beta-sitosterol	NCOA2
Radix Angelicae Sinensis (RAS)	MOL000358	beta-sitosterol	PTGS1
Radix Angelicae Sinensis (RAS)	MOL000358	beta-sitosterol	PTGS2
Radix Angelicae Sinensis (RAS)	MOL000358	beta-sitosterol	HSP90AB1
Radix Angelicae Sinensis (RAS)	MOL000358	beta-sitosterol	KCNH2
Radix Angelicae Sinensis (RAS)	MOL000358	beta-sitosterol	DRD1
Radix Angelicae Sinensis (RAS)	MOL000358	beta-sitosterol	CHRM3
Radix Angelicae Sinensis (RAS)	MOL000358	beta-sitosterol	CHRM1
Radix Angelicae Sinensis (RAS)	MOL000358	beta-sitosterol	SCN5A
Radix Angelicae Sinensis (RAS)	MOL000358	beta-sitosterol	CHRM4
Radix Angelicae Sinensis (RAS)	MOL000358	beta-sitosterol	ADRA1A
Radix Angelicae Sinensis (RAS)	MOL000358	beta-sitosterol	CHRM2
Radix Angelicae Sinensis (RAS)	MOL000358	beta-sitosterol	ADRA1B
Radix Angelicae Sinensis (RAS)	MOL000358	beta-sitosterol	ADRB2
Radix Angelicae Sinensis (RAS)	MOL000358	beta-sitosterol	CHRNA2
Radix Angelicae Sinensis (RAS)	MOL000358	beta-sitosterol	SLC6A4
Radix Angelicae Sinensis (RAS)	MOL000358	beta-sitosterol	OPRM1
Radix Angelicae Sinensis (RAS)	MOL000358	beta-sitosterol	GABRA1
Radix Angelicae Sinensis (RAS)	MOL000358	beta-sitosterol	BCL2
Radix Angelicae Sinensis (RAS)	MOL000358	beta-sitosterol	BAX
Radix Angelicae Sinensis (RAS)	MOL000358	beta-sitosterol	CASP9
Radix Angelicae Sinensis (RAS)	MOL000358	beta-sitosterol	JUN

Radix Angelicae Sinensis (RAS)	MOL000358	beta-sitosterol	CASP3
Radix Angelicae Sinensis (RAS)	MOL000358	beta-sitosterol	CASP8
Radix Angelicae Sinensis (RAS)	MOL000358	beta-sitosterol	PRKCA
Radix Angelicae Sinensis (RAS)	MOL000358	beta-sitosterol	PON1
Radix Angelicae Sinensis (RAS)	MOL000358	beta-sitosterol	MAP2
Radix Angelicae Sinensis (RAS)	MOL000449	Stigmasterol	PGR
Radix Angelicae Sinensis (RAS)	MOL000449	Stigmasterol	NR3C2
Radix Angelicae Sinensis (RAS)	MOL000449	Stigmasterol	NCOA2
Radix Angelicae Sinensis (RAS)	MOL000449	Stigmasterol	ADH1C
Radix Angelicae Sinensis (RAS)	MOL000449	Stigmasterol	IGHG1
Radix Angelicae Sinensis (RAS)	MOL000449	Stigmasterol	RXRA
Radix Angelicae Sinensis (RAS)	MOL000449	Stigmasterol	NCOA1
Radix Angelicae Sinensis (RAS)	MOL000449	Stigmasterol	PTGS1
Radix Angelicae Sinensis (RAS)	MOL000449	Stigmasterol	PTGS2
Radix Angelicae Sinensis (RAS)	MOL000449	Stigmasterol	ADRA2A
Radix Angelicae Sinensis (RAS)	MOL000449	Stigmasterol	SLC6A2
Radix Angelicae Sinensis (RAS)	MOL000449	Stigmasterol	SLC6A3
Radix Angelicae Sinensis (RAS)	MOL000449	Stigmasterol	ADRB2
Radix Angelicae Sinensis (RAS)	MOL000449	Stigmasterol	AKR1B1
Radix Angelicae Sinensis (RAS)	MOL000449	Stigmasterol	PLAU
Radix Angelicae Sinensis (RAS)	MOL000449	Stigmasterol	LTA4H
Radix Angelicae Sinensis (RAS)	MOL000449	Stigmasterol	MAOB
Radix Angelicae Sinensis (RAS)	MOL000449	Stigmasterol	MAOA
Radix Angelicae Sinensis (RAS)	MOL000449	Stigmasterol	CTRB1
Radix Angelicae Sinensis (RAS)	MOL000449	Stigmasterol	CHRM3
Radix Angelicae Sinensis (RAS)	MOL000449	Stigmasterol	CHRM1
Radix Angelicae Sinensis (RAS)	MOL000449	Stigmasterol	ADRB1
Radix Angelicae Sinensis (RAS)	MOL000449	Stigmasterol	SCN5A
Radix Angelicae Sinensis (RAS)	MOL000449	Stigmasterol	ADRA1A
Radix Angelicae Sinensis (RAS)	MOL000449	Stigmasterol	CHRM2
Radix Angelicae Sinensis (RAS)	MOL000449	Stigmasterol	ADRA1B

Radix Angelicae Sinensis (RAS)	MOL000449	Stigmasterol	GABRA1
Radix Codonopsis (RC)	MOL001006	poriferasta-7,22E-dien-3beta-ol	PGR
Radix Codonopsis (RC)	MOL001006	poriferasta-7,22E-dien-3beta-ol	NCOA2
Radix Codonopsis (RC)	MOL001006	poriferasta-7,22E-dien-3beta-ol	NR3C2
Radix Codonopsis (RC)	MOL002140	Perlolyrine	PTGS2
Radix Codonopsis (RC)	MOL002140	Perlolyrine	RXRA
Radix Codonopsis (RC)	MOL002879	Diop	SCN5A
Radix Codonopsis (RC)	MOL002879	Diop	ADRB2
Radix Codonopsis (RC)	MOL002879	Diop	CHRM3
Radix Codonopsis (RC)	MOL003036	ZINC03978781	PGR
Radix Codonopsis (RC)	MOL003036	ZINC03978781	NCOA2
Radix Codonopsis (RC)	MOL003036	ZINC03978781	NR3C2
Radix Codonopsis (RC)	MOL000449	Stigmasterol	PGR
Radix Codonopsis (RC)	MOL000449	Stigmasterol	NR3C2
Radix Codonopsis (RC)	MOL000449	Stigmasterol	NCOA2
Radix Codonopsis (RC)	MOL000449	Stigmasterol	ADH1C
Radix Codonopsis (RC)	MOL000449	Stigmasterol	IGHG1
Radix Codonopsis (RC)	MOL000449	Stigmasterol	RXRA
Radix Codonopsis (RC)	MOL000449	Stigmasterol	NCOA1
Radix Codonopsis (RC)	MOL000449	Stigmasterol	PTGS1
Radix Codonopsis (RC)	MOL000449	Stigmasterol	PTGS2
Radix Codonopsis (RC)	MOL000449	Stigmasterol	ADRA2A
Radix Codonopsis (RC)	MOL000449	Stigmasterol	SLC6A2
Radix Codonopsis (RC)	MOL000449	Stigmasterol	SLC6A3
Radix Codonopsis (RC)	MOL000449	Stigmasterol	ADRB2
Radix Codonopsis (RC)	MOL000449	Stigmasterol	AKR1B1
Radix Codonopsis (RC)	MOL000449	Stigmasterol	PLAU
Radix Codonopsis (RC)	MOL000449	Stigmasterol	LTA4H
Radix Codonopsis (RC)	MOL000449	Stigmasterol	MAOB
Radix Codonopsis (RC)	MOL000449	Stigmasterol	MAOA
Radix Codonopsis (RC)	MOL000449	Stigmasterol	CTRB1
Radix Codonopsis (RC)	MOL000449	Stigmasterol	CHRM3
Radix Codonopsis (RC)	MOL000449	Stigmasterol	CHRM1
Radix Codonopsis (RC)	MOL000449	Stigmasterol	ADRB1
Radix Codonopsis (RC)	MOL000449	Stigmasterol	SCN5A
Radix Codonopsis (RC)	MOL000449	Stigmasterol	ADRA1A
Radix Codonopsis (RC)	MOL000449	Stigmasterol	CHRM2
Radix Codonopsis (RC)	MOL000449	Stigmasterol	ADRA1B
Radix Codonopsis (RC)	MOL000449	Stigmasterol	GABRA1
Radix Codonopsis (RC)	MOL003896	7-Methoxy-2-methyl	NOS2
Radix Codonopsis (RC)	MOL003896	7-Methoxy-2-methyl	PTGS1
Radix Codonopsis (RC)	MOL003896	7-Methoxy-2-methyl	DRD1
Radix Codonopsis (RC)	MOL003896	7-Methoxy-2-methyl	CHRM3
Radix Codonopsis (RC)	MOL003896	7-Methoxy-2-methyl	CHRM1
Radix Codonopsis (RC)	MOL003896	7-Methoxy-2-methyl	ESR1
Radix Codonopsis (RC)	MOL003896	7-Methoxy-2-methyl	AR
Radix Codonopsis (RC)	MOL003896	7-Methoxy-2-methyl	ADRB1
Radix Codonopsis (RC)	MOL003896	7-Methoxy-2-methyl	SCN5A
Radix Codonopsis (RC)	MOL003896	7-Methoxy-2-methyl	PPARG
Radix Codonopsis (RC)	MOL003896	7-Methoxy-2-methyl	PTGS2
Radix Codonopsis (RC)	MOL003896	7-Methoxy-2-methyl	RXRA
Radix Codonopsis (RC)	MOL003896	7-Methoxy-2-methyl	ACHE
Radix Codonopsis (RC)	MOL003896	7-Methoxy-2-methyl	ADRA1B
Radix Codonopsis (RC)	MOL003896	7-Methoxy-2-methyl	SLC6A3
Radix Codonopsis (RC)	MOL003896	7-Methoxy-2-methyl	ADRB2
Radix Codonopsis (RC)	MOL003896	7-Methoxy-2-methyl	ADRA1D
Radix Codonopsis (RC)	MOL003896	7-Methoxy-2-methyl	SLC6A4
Radix Codonopsis (RC)	MOL003896	7-Methoxy-2-methyl	ESR2
Radix Codonopsis (RC)	MOL003896	7-Methoxy-2-methyl	GABRA1
Radix Codonopsis (RC)	MOL003896	7-Methoxy-2-methyl	DPP4
Radix Codonopsis (RC)	MOL003896	7-Methoxy-2-methyl	MAPK14
Radix Codonopsis (RC)	MOL003896	7-Methoxy-2-methyl	GSK3B

Radix Codonopsis (RC)	MOL003896	7-Methoxy-2-methyl	HSP90AB1
Radix Codonopsis (RC)	MOL003896	7-Methoxy-2-methyl	CDK2
Radix Codonopsis (RC)	MOL003896	7-Methoxy-2-methyl	LTA4H
Radix Codonopsis (RC)	MOL003896	7-Methoxy-2-methyl	MAOB
Radix Codonopsis (RC)	MOL003896	7-Methoxy-2-methyl	CHEK1
Radix Codonopsis (RC)	MOL003896	7-Methoxy-2-methyl	IGHG1
Radix Codonopsis (RC)	MOL003896	7-Methoxy-2-methyl	PRSS1
Radix Codonopsis (RC)	MOL003896	7-Methoxy-2-methyl	CCNA2
Radix Codonopsis (RC)	MOL003896	7-Methoxy-2-methyl	NCOA1
Radix Codonopsis (RC)	MOL003896	7-Methoxy-2-methyl	PKIA
Radix Codonopsis (RC)	MOL003896	7-Methoxy-2-methyl	CAMKMT
Radix Codonopsis (RC)	MOL003896	7-Methoxy-2-methyl	CHRM5
Radix Codonopsis (RC)	MOL003896	7-Methoxy-2-methyl	OPRM1
Radix Codonopsis (RC)	MOL003896	7-Methoxy-2-methyl	NCOA2
Radix Codonopsis (RC)	MOL004355	Spinasterol	PGR
Radix Codonopsis (RC)	MOL004355	Spinasterol	NR3C2
Radix Codonopsis (RC)	MOL004355	Spinasterol	NCOA2
Radix Codonopsis (RC)	MOL005321	Frutinone A	PTGS1
Radix Codonopsis (RC)	MOL005321	Frutinone A	AR
Radix Codonopsis (RC)	MOL005321	Frutinone A	SCN5A
Radix Codonopsis (RC)	MOL005321	Frutinone A	PPARG
Radix Codonopsis (RC)	MOL005321	Frutinone A	PTGS2
Radix Codonopsis (RC)	MOL005321	Frutinone A	RXRA
Radix Codonopsis (RC)	MOL005321	Frutinone A	ADRB2
Radix Codonopsis (RC)	MOL005321	Frutinone A	GABRA1
Radix Codonopsis (RC)	MOL005321	Frutinone A	DPP4
Radix Codonopsis (RC)	MOL005321	Frutinone A	HSP90AB1
Radix Codonopsis (RC)	MOL005321	Frutinone A	ACHE
Radix Codonopsis (RC)	MOL000006	luteolin	PTGS1
Radix Codonopsis (RC)	MOL000006	luteolin	AR
Radix Codonopsis (RC)	MOL000006	luteolin	PTGS2
Radix Codonopsis (RC)	MOL000006	luteolin	HSP90AB1
Radix Codonopsis (RC)	MOL000006	luteolin	PRSS1
Radix Codonopsis (RC)	MOL000006	luteolin	NCOA2
Radix Codonopsis (RC)	MOL000006	luteolin	DPP4
Radix Codonopsis (RC)	MOL000006	luteolin	RELA
Radix Codonopsis (RC)	MOL000006	luteolin	EGFR
Radix Codonopsis (RC)	MOL000006	luteolin	AKT1
Radix Codonopsis (RC)	MOL000006	luteolin	VEGFA
Radix Codonopsis (RC)	MOL000006	luteolin	CCND1
Radix Codonopsis (RC)	MOL000006	luteolin	BCL2L1
Radix Codonopsis (RC)	MOL000006	luteolin	CDKN1A
Radix Codonopsis (RC)	MOL000006	luteolin	CASP9
Radix Codonopsis (RC)	MOL000006	luteolin	MMP2
Radix Codonopsis (RC)	MOL000006	luteolin	MMP9
Radix Codonopsis (RC)	MOL000006	luteolin	MAPK1
Radix Codonopsis (RC)	MOL000006	luteolin	IL10RA
Radix Codonopsis (RC)	MOL000006	luteolin	RB1
Radix Codonopsis (RC)	MOL000006	luteolin	CDK4
Radix Codonopsis (RC)	MOL000006	luteolin	TNFAIP6
Radix Codonopsis (RC)	MOL000006	luteolin	JUN
Radix Codonopsis (RC)	MOL000006	luteolin	IL6R
Radix Codonopsis (RC)	MOL000006	luteolin	CASP3
Radix Codonopsis (RC)	MOL000006	luteolin	TP53
Radix Codonopsis (RC)	MOL000006	luteolin	NFKBIA
Radix Codonopsis (RC)	MOL000006	luteolin	TOP1
Radix Codonopsis (RC)	MOL000006	luteolin	MDM2
Radix Codonopsis (RC)	MOL000006	luteolin	APP
Radix Codonopsis (RC)	MOL000006	luteolin	MMP1
Radix Codonopsis (RC)	MOL000006	luteolin	PCNA
Radix Codonopsis (RC)	MOL000006	luteolin	ERBB2
Radix Codonopsis (RC)	MOL000006	luteolin	PPARG
Radix Codonopsis (RC)	MOL000006	luteolin	HMOX1

Radix Codonopsis (RC)	MOL000006	luteolin	CASP7
Radix Codonopsis (RC)	MOL000006	luteolin	ICAM1
Radix Codonopsis (RC)	MOL000006	luteolin	MCL1
Radix Codonopsis (RC)	MOL000006	luteolin	BIRC5
Radix Codonopsis (RC)	MOL000006	luteolin	IL2RA
Radix Codonopsis (RC)	MOL000006	luteolin	CCNB1
Radix Codonopsis (RC)	MOL000006	luteolin	TYR
Radix Codonopsis (RC)	MOL000006	luteolin	IFNG
Radix Codonopsis (RC)	MOL000006	luteolin	IL4
Radix Codonopsis (RC)	MOL000006	luteolin	TOP2A
Radix Codonopsis (RC)	MOL000006	luteolin	GSTP1
Radix Codonopsis (RC)	MOL000006	luteolin	XIAP
Radix Codonopsis (RC)	MOL000006	luteolin	SLC2A4
Radix Codonopsis (RC)	MOL000006	luteolin	INSRR
Radix Codonopsis (RC)	MOL000006	luteolin	CD40LG
Radix Codonopsis (RC)	MOL000006	luteolin	PTGES
Radix Codonopsis (RC)	MOL000006	luteolin	NUF2
Radix Codonopsis (RC)	MOL000006	luteolin	ADCY2
Radix Codonopsis (RC)	MOL000006	luteolin	MET
Radix Codonopsis (RC)	MOL006774	stigmast-7-enol	PGR
Radix Codonopsis (RC)	MOL006774	stigmast-7-enol	NCOA2
Radix Codonopsis (RC)	MOL007059	3-beta-Hydroxymethyllenetanshiquino	DRD1
Radix Codonopsis (RC)	MOL007059	3-beta-Hydroxymethyllenetanshiquino	CHRM1
Radix Codonopsis (RC)	MOL007059	3-beta-Hydroxymethyllenetanshiquino	PTGS2
Radix Codonopsis (RC)	MOL007059	3-beta-Hydroxymethyllenetanshiquino	CA2
Radix Codonopsis (RC)	MOL007059	3-beta-Hydroxymethyllenetanshiquino	RXRA
Radix Codonopsis (RC)	MOL007059	3-beta-Hydroxymethyllenetanshiquino	OPRD1
Radix Codonopsis (RC)	MOL007059	3-beta-Hydroxymethyllenetanshiquino	ACHE
Radix Codonopsis (RC)	MOL007059	3-beta-Hydroxymethyllenetanshiquino	ADRA1A
Radix Codonopsis (RC)	MOL007059	3-beta-Hydroxymethyllenetanshiquino	ADRB2
Radix Codonopsis (RC)	MOL007059	3-beta-Hydroxymethyllenetanshiquino	OPRM1
Radix Codonopsis (RC)	MOL007059	3-beta-Hydroxymethyllenetanshiquino	DPP4
Radix Codonopsis (RC)	MOL007059	3-beta-Hydroxymethyllenetanshiquino	HSP90AB1
Radix Codonopsis (RC)	MOL007059	3-beta-Hydroxymethyllenetanshiquino	IGHG1
Radix Codonopsis (RC)	MOL007059	3-beta-Hydroxymethyllenetanshiquino	PRSS1
Radix Codonopsis (RC)	MOL007059	3-beta-Hydroxymethyllenetanshiquino	NCOA1
Radix Codonopsis (RC)	MOL007514	methyl icosa-11,14-dienoate	NCOA2
Radix Codonopsis (RC)	MOL008393	7-(beta-Xylosyl)cephalomannine_qt	TUBB1
Radix Codonopsis (RC)	MOL008397	Daturilin	NR3C1
Radix Codonopsis (RC)	MOL008400	glycitein	PTGS1
Radix Codonopsis (RC)	MOL008400	glycitein	ESR1
Radix Codonopsis (RC)	MOL008400	glycitein	AR
Radix Codonopsis (RC)	MOL008400	glycitein	PPARG
Radix Codonopsis (RC)	MOL008400	glycitein	PTGS2
Radix Codonopsis (RC)	MOL008400	glycitein	RXRA
Radix Codonopsis (RC)	MOL008400	glycitein	ESR2
Radix Codonopsis (RC)	MOL008400	glycitein	MAPK14

Radix Codonopsis (RC)	MOL008400	glycitein	GSK3B
Radix Codonopsis (RC)	MOL008400	glycitein	HSP90AB1
Radix Codonopsis (RC)	MOL008400	glycitein	CDK2
Radix Codonopsis (RC)	MOL008400	glycitein	CHEK1
Radix Codonopsis (RC)	MOL008400	glycitein	PRSS1
Radix Codonopsis (RC)	MOL008400	glycitein	CCNA2
Radix Codonopsis (RC)	MOL008400	glycitein	CAMKMT
Radix Codonopsis (RC)	MOL008400	glycitein	NCOA1
Radix Codonopsis (RC)	MOL008400	glycitein	NOS2
Radix Codonopsis (RC)	MOL008400	glycitein	APP
Radix Codonopsis (RC)	MOL008400	glycitein	MMP13
Radix Codonopsis (RC)	MOL008400	glycitein	MMP8
Radix Codonopsis (RC)	MOL008407	(8S,9S,10R,13R,14S,17R)-17-[(E,2R,5S)-5-ethyl-6-methylhept-3-en-2-yl]-10,13-dimethyl-1,2,4,7,8,9,11,12,14,15,16,17-dodecahydrocyclopenta[a]phenanthren-3-one	PGR
Radix Codonopsis (RC)	MOL008407	(8S,9S,10R,13R,14S,17R)-17-[(E,2R,5S)-5-ethyl-6-methylhept-3-en-2-yl]-10,13-dimethyl-1,2,4,7,8,9,11,12,14,15,16,17-dodecahydrocyclopenta[a]phenanthren-3-one	NR3C2
Radix Codonopsis (RC)	MOL008411	11-Hydroxyrankinidine	ESR1
Radix Codonopsis (RC)	MOL008411	11-Hydroxyrankinidine	SCN5A
Radix Codonopsis (RC)	MOL008411	11-Hydroxyrankinidine	OPRM1
Radix Codonopsis (RC)	MOL008411	11-Hydroxyrankinidine	CDK2
Radix Glycyrrhizae (RG)	MOL001484	Inermine	PTGS1
Radix Glycyrrhizae (RG)	MOL001484	Inermine	CHRM3
Radix Glycyrrhizae (RG)	MOL001484	Inermine	SCN5A
Radix Glycyrrhizae (RG)	MOL001484	Inermine	PTGS2
Radix Glycyrrhizae (RG)	MOL001484	Inermine	HTR3A
Radix Glycyrrhizae (RG)	MOL001484	Inermine	RXRA
Radix Glycyrrhizae (RG)	MOL001484	Inermine	ADRA1B
Radix Glycyrrhizae (RG)	MOL001484	Inermine	ADRA1D
Radix Glycyrrhizae (RG)	MOL001484	Inermine	IGHG1
Radix Glycyrrhizae (RG)	MOL001484	Inermine	PRSS1
Radix Glycyrrhizae (RG)	MOL001484	Inermine	CAMKMT
Radix Glycyrrhizae (RG)	MOL001484	Inermine	CHRM1
Radix Glycyrrhizae (RG)	MOL001484	Inermine	ADRB2
Radix Glycyrrhizae (RG)	MOL001484	Inermine	OPRM1
Radix Glycyrrhizae (RG)	MOL001484	Inermine	HSP90AB1
Radix Glycyrrhizae (RG)	MOL001792	DFV	PTGS1
Radix Glycyrrhizae (RG)	MOL001792	DFV	ESR1
Radix Glycyrrhizae (RG)	MOL001792	DFV	PTGS2
Radix Glycyrrhizae (RG)	MOL001792	DFV	RXRA
Radix Glycyrrhizae (RG)	MOL001792	DFV	ADRB2
Radix Glycyrrhizae (RG)	MOL001792	DFV	HSP90AB1
Radix Glycyrrhizae (RG)	MOL001792	DFV	DPEP1
Radix Glycyrrhizae (RG)	MOL001792	DFV	MAOB
Radix Glycyrrhizae (RG)	MOL001792	DFV	SLC6A4
Radix Glycyrrhizae (RG)	MOL001792	DFV	PKIA
Radix Glycyrrhizae (RG)	MOL000211	Mairin	PGR
Radix Glycyrrhizae (RG)	MOL002311	Glycyrol	NOS2
Radix Glycyrrhizae (RG)	MOL002311	Glycyrol	ESR1
Radix Glycyrrhizae (RG)	MOL002311	Glycyrol	PPARG
Radix Glycyrrhizae (RG)	MOL002311	Glycyrol	PTGS2
Radix Glycyrrhizae (RG)	MOL002311	Glycyrol	KDR
Radix Glycyrrhizae (RG)	MOL002311	Glycyrol	MAPK14
Radix Glycyrrhizae (RG)	MOL002311	Glycyrol	GSK3B

Radix Glycyrrhizae (RG)	MOL002311	Glycyrol	CHEK1
Radix Glycyrrhizae (RG)	MOL002311	Glycyrol	CCNA2
Radix Glycyrrhizae (RG)	MOL000239	Jaranol	NOS2
Radix Glycyrrhizae (RG)	MOL000239	Jaranol	PTGS1
Radix Glycyrrhizae (RG)	MOL000239	Jaranol	AR
Radix Glycyrrhizae (RG)	MOL000239	Jaranol	SCN5A
Radix Glycyrrhizae (RG)	MOL000239	Jaranol	PTGS2
Radix Glycyrrhizae (RG)	MOL000239	Jaranol	ESR2
Radix Glycyrrhizae (RG)	MOL000239	Jaranol	DPP4
Radix Glycyrrhizae (RG)	MOL000239	Jaranol	HSP90AB1
Radix Glycyrrhizae (RG)	MOL000239	Jaranol	CDK2
Radix Glycyrrhizae (RG)	MOL000239	Jaranol	CHEK1
Radix Glycyrrhizae (RG)	MOL000239	Jaranol	PRSS1
Radix Glycyrrhizae (RG)	MOL000239	Jaranol	NCOA2
Radix Glycyrrhizae (RG)	MOL000239	Jaranol	CAMKMT
Radix Glycyrrhizae (RG)	MOL002565	Medicarpin	NOS2
Radix Glycyrrhizae (RG)	MOL002565	Medicarpin	PTGS1
Radix Glycyrrhizae (RG)	MOL002565	Medicarpin	DRD1
Radix Glycyrrhizae (RG)	MOL002565	Medicarpin	CHRM3
Radix Glycyrrhizae (RG)	MOL002565	Medicarpin	CHRM1
Radix Glycyrrhizae (RG)	MOL002565	Medicarpin	ESR1
Radix Glycyrrhizae (RG)	MOL002565	Medicarpin	SCN5A
Radix Glycyrrhizae (RG)	MOL002565	Medicarpin	CHRM5
Radix Glycyrrhizae (RG)	MOL002565	Medicarpin	PTGS2
Radix Glycyrrhizae (RG)	MOL002565	Medicarpin	CHRM4
Radix Glycyrrhizae (RG)	MOL002565	Medicarpin	RXRA
Radix Glycyrrhizae (RG)	MOL002565	Medicarpin	ADRA1A
Radix Glycyrrhizae (RG)	MOL002565	Medicarpin	CHRM2
Radix Glycyrrhizae (RG)	MOL002565	Medicarpin	ADRA1B
Radix Glycyrrhizae (RG)	MOL002565	Medicarpin	SLC6A3
Radix Glycyrrhizae (RG)	MOL002565	Medicarpin	ADRB2
Radix Glycyrrhizae (RG)	MOL002565	Medicarpin	SLC6A4
Radix Glycyrrhizae (RG)	MOL002565	Medicarpin	OPRM1
Radix Glycyrrhizae (RG)	MOL002565	Medicarpin	ESR2
Radix Glycyrrhizae (RG)	MOL002565	Medicarpin	DPP4
Radix Glycyrrhizae (RG)	MOL002565	Medicarpin	MAPK10
Radix Glycyrrhizae (RG)	MOL002565	Medicarpin	HSP90AB1
Radix Glycyrrhizae (RG)	MOL002565	Medicarpin	CDK2
Radix Glycyrrhizae (RG)	MOL002565	Medicarpin	PRSS1
Radix Glycyrrhizae (RG)	MOL002565	Medicarpin	CCNA2
Radix Glycyrrhizae (RG)	MOL002565	Medicarpin	CAMKMT
Radix Glycyrrhizae (RG)	MOL002565	Medicarpin	OPRD1
Radix Glycyrrhizae (RG)	MOL002565	Medicarpin	ADRA1D
Radix Glycyrrhizae (RG)	MOL000354	isorhamnetin	NOS2
Radix Glycyrrhizae (RG)	MOL000354	isorhamnetin	PTGS1
Radix Glycyrrhizae (RG)	MOL000354	isorhamnetin	ESR1
Radix Glycyrrhizae (RG)	MOL000354	isorhamnetin	AR
Radix Glycyrrhizae (RG)	MOL000354	isorhamnetin	PPARG
Radix Glycyrrhizae (RG)	MOL000354	isorhamnetin	PTGS2
Radix Glycyrrhizae (RG)	MOL000354	isorhamnetin	ESR2
Radix Glycyrrhizae (RG)	MOL000354	isorhamnetin	DPP4
Radix Glycyrrhizae (RG)	MOL000354	isorhamnetin	MAPK14
Radix Glycyrrhizae (RG)	MOL000354	isorhamnetin	GSK3B
Radix Glycyrrhizae (RG)	MOL000354	isorhamnetin	HSP90AB1
Radix Glycyrrhizae (RG)	MOL000354	isorhamnetin	CDK2
Radix Glycyrrhizae (RG)	MOL000354	isorhamnetin	PRSS1
Radix Glycyrrhizae (RG)	MOL000354	isorhamnetin	CCNA2
Radix Glycyrrhizae (RG)	MOL000354	isorhamnetin	NCOA2
Radix Glycyrrhizae (RG)	MOL000354	isorhamnetin	CAMKMT
Radix Glycyrrhizae (RG)	MOL000354	isorhamnetin	PYGM
Radix Glycyrrhizae (RG)	MOL000354	isorhamnetin	PPARD
Radix Glycyrrhizae (RG)	MOL000354	isorhamnetin	CHEK1
Radix Glycyrrhizae (RG)	MOL000354	isorhamnetin	AKR1B1

Radix Glycyrrhizae (RG)	MOL000354	isorhamnetin	NCOA1
Radix Glycyrrhizae (RG)	MOL000354	isorhamnetin	F7
Radix Glycyrrhizae (RG)	MOL000354	isorhamnetin	ACHE
Radix Glycyrrhizae (RG)	MOL000354	isorhamnetin	GABRA1
Radix Glycyrrhizae (RG)	MOL000354	isorhamnetin	MAOB
Radix Glycyrrhizae (RG)	MOL000354	isorhamnetin	GRIA2
Radix Glycyrrhizae (RG)	MOL000354	isorhamnetin	RELA
Radix Glycyrrhizae (RG)	MOL000354	isorhamnetin	NCF1
Radix Glycyrrhizae (RG)	MOL000354	isorhamnetin	OLR1
Radix Glycyrrhizae (RG)	MOL000359	sitosterol	PGR
Radix Glycyrrhizae (RG)	MOL000359	sitosterol	NCOA2
Radix Glycyrrhizae (RG)	MOL000359	sitosterol	NR3C2
Radix Glycyrrhizae (RG)	MOL003656	Lupiwighteone	NOS2
Radix Glycyrrhizae (RG)	MOL003656	Lupiwighteone	ESR1
Radix Glycyrrhizae (RG)	MOL003656	Lupiwighteone	AR
Radix Glycyrrhizae (RG)	MOL003656	Lupiwighteone	SCN5A
Radix Glycyrrhizae (RG)	MOL003656	Lupiwighteone	PPARG
Radix Glycyrrhizae (RG)	MOL003656	Lupiwighteone	PTGS2
Radix Glycyrrhizae (RG)	MOL003656	Lupiwighteone	ESR2
Radix Glycyrrhizae (RG)	MOL003656	Lupiwighteone	DPP4
Radix Glycyrrhizae (RG)	MOL003656	Lupiwighteone	MAPK14
Radix Glycyrrhizae (RG)	MOL003656	Lupiwighteone	GSK3B
Radix Glycyrrhizae (RG)	MOL003656	Lupiwighteone	HSP90AB1
Radix Glycyrrhizae (RG)	MOL003656	Lupiwighteone	CDK2
Radix Glycyrrhizae (RG)	MOL003656	Lupiwighteone	CHEK1
Radix Glycyrrhizae (RG)	MOL003656	Lupiwighteone	PRSS1
Radix Glycyrrhizae (RG)	MOL003656	Lupiwighteone	CCNA2
Radix Glycyrrhizae (RG)	MOL003656	Lupiwighteone	NCOA2
Radix Glycyrrhizae (RG)	MOL003656	Lupiwighteone	CAMKMT
Radix Glycyrrhizae (RG)	MOL003896	7-Methoxy-2-methyl	NOS2
Radix Glycyrrhizae (RG)	MOL003896	7-Methoxy-2-methyl	PTGS1
Radix Glycyrrhizae (RG)	MOL003896	7-Methoxy-2-methyl	DRD1
Radix Glycyrrhizae (RG)	MOL003896	7-Methoxy-2-methyl	CHRM3
Radix Glycyrrhizae (RG)	MOL003896	7-Methoxy-2-methyl	CHRM1
Radix Glycyrrhizae (RG)	MOL003896	7-Methoxy-2-methyl	ESR1
Radix Glycyrrhizae (RG)	MOL003896	7-Methoxy-2-methyl	AR
Radix Glycyrrhizae (RG)	MOL003896	7-Methoxy-2-methyl	ADRB1
Radix Glycyrrhizae (RG)	MOL003896	7-Methoxy-2-methyl	SCN5A
Radix Glycyrrhizae (RG)	MOL003896	7-Methoxy-2-methyl	PPARG
Radix Glycyrrhizae (RG)	MOL003896	7-Methoxy-2-methyl	PTGS2
Radix Glycyrrhizae (RG)	MOL003896	7-Methoxy-2-methyl	RXRA
Radix Glycyrrhizae (RG)	MOL003896	7-Methoxy-2-methyl	ACHE
Radix Glycyrrhizae (RG)	MOL003896	7-Methoxy-2-methyl	ADRA1B
Radix Glycyrrhizae (RG)	MOL003896	7-Methoxy-2-methyl	SLC6A3
Radix Glycyrrhizae (RG)	MOL003896	7-Methoxy-2-methyl	ADRB2
Radix Glycyrrhizae (RG)	MOL003896	7-Methoxy-2-methyl	ADRA1D
Radix Glycyrrhizae (RG)	MOL003896	7-Methoxy-2-methyl	SLC6A4
Radix Glycyrrhizae (RG)	MOL003896	7-Methoxy-2-methyl	ESR2
Radix Glycyrrhizae (RG)	MOL003896	7-Methoxy-2-methyl	GABRA1
Radix Glycyrrhizae (RG)	MOL003896	7-Methoxy-2-methyl	DPP4
Radix Glycyrrhizae (RG)	MOL003896	7-Methoxy-2-methyl	MAPK14
Radix Glycyrrhizae (RG)	MOL003896	7-Methoxy-2-methyl	GSK3B
Radix Glycyrrhizae (RG)	MOL003896	7-Methoxy-2-methyl	HSP90AB1
Radix Glycyrrhizae (RG)	MOL003896	7-Methoxy-2-methyl	CDK2
Radix Glycyrrhizae (RG)	MOL003896	7-Methoxy-2-methyl	LTA4H
Radix Glycyrrhizae (RG)	MOL003896	7-Methoxy-2-methyl	MAOB
Radix Glycyrrhizae (RG)	MOL003896	7-Methoxy-2-methyl	CHEK1
Radix Glycyrrhizae (RG)	MOL003896	7-Methoxy-2-methyl	IGHG1
Radix Glycyrrhizae (RG)	MOL003896	7-Methoxy-2-methyl	PRSS1
Radix Glycyrrhizae (RG)	MOL003896	7-Methoxy-2-methyl	CCNA2
Radix Glycyrrhizae (RG)	MOL003896	7-Methoxy-2-methyl	NCOA1
Radix Glycyrrhizae (RG)	MOL003896	7-Methoxy-2-methyl	PKIA
Radix Glycyrrhizae (RG)	MOL003896	7-Methoxy-2-methyl	CAMKMT

Radix Glycyrrhizae (RG)	MOL003896	7-Methoxy-2-methyl	CHRM5
Radix Glycyrrhizae (RG)	MOL003896	7-Methoxy-2-methyl	OPRM1
Radix Glycyrrhizae (RG)	MOL003896	7-Methoxy-2-methyl	NCOA2
Radix Glycyrrhizae (RG)	MOL000392	formononetin	NOS2
Radix Glycyrrhizae (RG)	MOL000392	formononetin	PTGS1
Radix Glycyrrhizae (RG)	MOL000392	formononetin	CHRM1
Radix Glycyrrhizae (RG)	MOL000392	formononetin	ESR1
Radix Glycyrrhizae (RG)	MOL000392	formononetin	AR
Radix Glycyrrhizae (RG)	MOL000392	formononetin	PPARG
Radix Glycyrrhizae (RG)	MOL000392	formononetin	PTGS2
Radix Glycyrrhizae (RG)	MOL000392	formononetin	RXRA
Radix Glycyrrhizae (RG)	MOL000392	formononetin	ADRA1A
Radix Glycyrrhizae (RG)	MOL000392	formononetin	SLC6A3
Radix Glycyrrhizae (RG)	MOL000392	formononetin	ADRB2
Radix Glycyrrhizae (RG)	MOL000392	formononetin	SLC6A4
Radix Glycyrrhizae (RG)	MOL000392	formononetin	ESR2
Radix Glycyrrhizae (RG)	MOL000392	formononetin	DPP4
Radix Glycyrrhizae (RG)	MOL000392	formononetin	MAPK14
Radix Glycyrrhizae (RG)	MOL000392	formononetin	GSK3B
Radix Glycyrrhizae (RG)	MOL000392	formononetin	HSP90AB1
Radix Glycyrrhizae (RG)	MOL000392	formononetin	CDK2
Radix Glycyrrhizae (RG)	MOL000392	formononetin	MAOB
Radix Glycyrrhizae (RG)	MOL000392	formononetin	CHEK1
Radix Glycyrrhizae (RG)	MOL000392	formononetin	PRSS1
Radix Glycyrrhizae (RG)	MOL000392	formononetin	CCNA2
Radix Glycyrrhizae (RG)	MOL000392	formononetin	CAMKMT
Radix Glycyrrhizae (RG)	MOL000392	formononetin	PKIA
Radix Glycyrrhizae (RG)	MOL000392	formononetin	ACHE
Radix Glycyrrhizae (RG)	MOL000392	formononetin	DPEP1
Radix Glycyrrhizae (RG)	MOL000392	formononetin	JUN
Radix Glycyrrhizae (RG)	MOL000392	formononetin	PPARG
Radix Glycyrrhizae (RG)	MOL000392	formononetin	IL4
Radix Glycyrrhizae (RG)	MOL000392	formononetin	ATP5F1B
Radix Glycyrrhizae (RG)	MOL000392	formononetin	HSD3B2
Radix Glycyrrhizae (RG)	MOL000392	formononetin	HSD3B1
Radix Glycyrrhizae (RG)	MOL000417	Calycosin	NOS2
Radix Glycyrrhizae (RG)	MOL000417	Calycosin	PTGS1
Radix Glycyrrhizae (RG)	MOL000417	Calycosin	ESR1
Radix Glycyrrhizae (RG)	MOL000417	Calycosin	AR
Radix Glycyrrhizae (RG)	MOL000417	Calycosin	PPARG
Radix Glycyrrhizae (RG)	MOL000417	Calycosin	PTGS2
Radix Glycyrrhizae (RG)	MOL000417	Calycosin	RXRA
Radix Glycyrrhizae (RG)	MOL000417	Calycosin	ESR2
Radix Glycyrrhizae (RG)	MOL000417	Calycosin	DPP4
Radix Glycyrrhizae (RG)	MOL000417	Calycosin	MAPK14
Radix Glycyrrhizae (RG)	MOL000417	Calycosin	GSK3B
Radix Glycyrrhizae (RG)	MOL000417	Calycosin	HSP90AB1
Radix Glycyrrhizae (RG)	MOL000417	Calycosin	CDK2
Radix Glycyrrhizae (RG)	MOL000417	Calycosin	CHEK1
Radix Glycyrrhizae (RG)	MOL000417	Calycosin	PRSS1
Radix Glycyrrhizae (RG)	MOL000417	Calycosin	CCNA2
Radix Glycyrrhizae (RG)	MOL000417	Calycosin	NCOA2
Radix Glycyrrhizae (RG)	MOL000417	Calycosin	CAMKMT
Radix Glycyrrhizae (RG)	MOL000417	Calycosin	ADRB2
Radix Glycyrrhizae (RG)	MOL000422	kaempferol	NOS2
Radix Glycyrrhizae (RG)	MOL000422	kaempferol	PTGS1
Radix Glycyrrhizae (RG)	MOL000422	kaempferol	AR
Radix Glycyrrhizae (RG)	MOL000422	kaempferol	PPARG
Radix Glycyrrhizae (RG)	MOL000422	kaempferol	PTGS2
Radix Glycyrrhizae (RG)	MOL000422	kaempferol	HSP90AB1
Radix Glycyrrhizae (RG)	MOL000422	kaempferol	NCOA2
Radix Glycyrrhizae (RG)	MOL000422	kaempferol	DPP4
Radix Glycyrrhizae (RG)	MOL000422	kaempferol	PRSS1

Radix Glycyrrhizae (RG)	MOL000422	kaempferol	PGR
Radix Glycyrrhizae (RG)	MOL000422	kaempferol	CHRM1
Radix Glycyrrhizae (RG)	MOL000422	kaempferol	ACHE
Radix Glycyrrhizae (RG)	MOL000422	kaempferol	SLC6A2
Radix Glycyrrhizae (RG)	MOL000422	kaempferol	CHRM2
Radix Glycyrrhizae (RG)	MOL000422	kaempferol	ADRA1B
Radix Glycyrrhizae (RG)	MOL000422	kaempferol	GABRA1
Radix Glycyrrhizae (RG)	MOL000422	kaempferol	F7
Radix Glycyrrhizae (RG)	MOL000422	kaempferol	CAMKMT
Radix Glycyrrhizae (RG)	MOL000422	kaempferol	RELA
Radix Glycyrrhizae (RG)	MOL000422	kaempferol	IKBKB
Radix Glycyrrhizae (RG)	MOL000422	kaempferol	AKT1
Radix Glycyrrhizae (RG)	MOL000422	kaempferol	BCL2
Radix Glycyrrhizae (RG)	MOL000422	kaempferol	BAX
Radix Glycyrrhizae (RG)	MOL000422	kaempferol	TNFAIP6
Radix Glycyrrhizae (RG)	MOL000422	kaempferol	JUN
Radix Glycyrrhizae (RG)	MOL000422	kaempferol	AHSA1
Radix Glycyrrhizae (RG)	MOL000422	kaempferol	CASP3
Radix Glycyrrhizae (RG)	MOL000422	kaempferol	MAPK8
Radix Glycyrrhizae (RG)	MOL000422	kaempferol	MMP1
Radix Glycyrrhizae (RG)	MOL000422	kaempferol	STAT1
Radix Glycyrrhizae (RG)	MOL000422	kaempferol	CDK1
Radix Glycyrrhizae (RG)	MOL000422	kaempferol	PPARG
Radix Glycyrrhizae (RG)	MOL000422	kaempferol	HMOX1
Radix Glycyrrhizae (RG)	MOL000422	kaempferol	CYP3A4
Radix Glycyrrhizae (RG)	MOL000422	kaempferol	CYP1A2
Radix Glycyrrhizae (RG)	MOL000422	kaempferol	CYP1A1
Radix Glycyrrhizae (RG)	MOL000422	kaempferol	ICAM1
Radix Glycyrrhizae (RG)	MOL000422	kaempferol	SELE
Radix Glycyrrhizae (RG)	MOL000422	kaempferol	VCAM1
Radix Glycyrrhizae (RG)	MOL000422	kaempferol	NR1I2
Radix Glycyrrhizae (RG)	MOL000422	kaempferol	CYP1B1
Radix Glycyrrhizae (RG)	MOL000422	kaempferol	ALOX5
Radix Glycyrrhizae (RG)	MOL000422	kaempferol	HAS2
Radix Glycyrrhizae (RG)	MOL000422	kaempferol	GSTP1
Radix Glycyrrhizae (RG)	MOL000422	kaempferol	AHR
Radix Glycyrrhizae (RG)	MOL000422	kaempferol	PSMD3
Radix Glycyrrhizae (RG)	MOL000422	kaempferol	SLC2A4
Radix Glycyrrhizae (RG)	MOL000422	kaempferol	NR1I3
Radix Glycyrrhizae (RG)	MOL000422	kaempferol	INSRR
Radix Glycyrrhizae (RG)	MOL000422	kaempferol	DIO1
Radix Glycyrrhizae (RG)	MOL000422	kaempferol	PPP3CA
Radix Glycyrrhizae (RG)	MOL000422	kaempferol	GSTM1
Radix Glycyrrhizae (RG)	MOL000422	kaempferol	GSTM2
Radix Glycyrrhizae (RG)	MOL000422	kaempferol	AKR1C3
Radix Glycyrrhizae (RG)	MOL000422	kaempferol	SLPI
Radix Glycyrrhizae (RG)	MOL004328	naringenin	PTGS1
Radix Glycyrrhizae (RG)	MOL004328	naringenin	ESR1
Radix Glycyrrhizae (RG)	MOL004328	naringenin	PTGS2
Radix Glycyrrhizae (RG)	MOL004328	naringenin	HSP90AB1
Radix Glycyrrhizae (RG)	MOL004328	naringenin	DPEP1
Radix Glycyrrhizae (RG)	MOL004328	naringenin	RELA
Radix Glycyrrhizae (RG)	MOL004328	naringenin	AKT1
Radix Glycyrrhizae (RG)	MOL004328	naringenin	BCL2
Radix Glycyrrhizae (RG)	MOL004328	naringenin	MAPK3
Radix Glycyrrhizae (RG)	MOL004328	naringenin	MAPK1
Radix Glycyrrhizae (RG)	MOL004328	naringenin	CASP3
Radix Glycyrrhizae (RG)	MOL004328	naringenin	FASN
Radix Glycyrrhizae (RG)	MOL004328	naringenin	LDLR
Radix Glycyrrhizae (RG)	MOL004328	naringenin	BAD
Radix Glycyrrhizae (RG)	MOL004328	naringenin	SOD1
Radix Glycyrrhizae (RG)	MOL004328	naringenin	CAT
Radix Glycyrrhizae (RG)	MOL004328	naringenin	PPARG

Radix Glycyrrhizae (RG)	MOL004328	naringenin	MTTP
Radix Glycyrrhizae (RG)	MOL004328	naringenin	APOB
Radix Glycyrrhizae (RG)	MOL004328	naringenin	PLB1
Radix Glycyrrhizae (RG)	MOL004328	naringenin	HMGCR
Radix Glycyrrhizae (RG)	MOL004328	naringenin	CYP19A1
Radix Glycyrrhizae (RG)	MOL004328	naringenin	GSTP1
Radix Glycyrrhizae (RG)	MOL004328	naringenin	UGT1A1
Radix Glycyrrhizae (RG)	MOL004328	naringenin	PPARA
Radix Glycyrrhizae (RG)	MOL004328	naringenin	SREBF1
Radix Glycyrrhizae (RG)	MOL004328	naringenin	GSR
Radix Glycyrrhizae (RG)	MOL004328	naringenin	ABCC1
Radix Glycyrrhizae (RG)	MOL004328	naringenin	ADIPOR2
Radix Glycyrrhizae (RG)	MOL004328	naringenin	SOAT2
Radix Glycyrrhizae (RG)	MOL004328	naringenin	AKR1C1
Radix Glycyrrhizae (RG)	MOL004328	naringenin	GOT1
Radix Glycyrrhizae (RG)	MOL004328	naringenin	ABAT
Radix Glycyrrhizae (RG)	MOL004328	naringenin	CES1
Radix Glycyrrhizae (RG)	MOL004328	naringenin	SOAT1
Radix Glycyrrhizae (RG)	MOL004805	(2S)-2-[4-hydroxy-3-(3-methylbut-2-enyl)phenyl]-8,8-dimethyl-2,3-dihydropyrano[2,3-f]chromen-	NOS2
Radix Glycyrrhizae (RG)	MOL004805	(2S)-2-[4-hydroxy-3-(3-methylbut-2-enyl)phenyl]-8,8-dimethyl-2,3-dihydropyrano[2,3-f]chromen-	KCNH2
Radix Glycyrrhizae (RG)	MOL004805	(2S)-2-[4-hydroxy-3-(3-methylbut-2-enyl)phenyl]-8,8-dimethyl-2,3-dihydropyrano[2,3-f]chromen-	ESR1
Radix Glycyrrhizae (RG)	MOL004805	(2S)-2-[4-hydroxy-3-(3-methylbut-2-enyl)phenyl]-8,8-dimethyl-2,3-dihydropyrano[2,3-f]chromen-	AR
Radix Glycyrrhizae (RG)	MOL004805	(2S)-2-[4-hydroxy-3-(3-methylbut-2-enyl)phenyl]-8,8-dimethyl-2,3-dihydropyrano[2,3-f]chromen-	PPARG
Radix Glycyrrhizae (RG)	MOL004805	(2S)-2-[4-hydroxy-3-(3-methylbut-2-enyl)phenyl]-8,8-dimethyl-2,3-dihydropyrano[2,3-f]chromen-	PTGS2
Radix Glycyrrhizae (RG)	MOL004805	(2S)-2-[4-hydroxy-3-(3-methylbut-2-enyl)phenyl]-8,8-dimethyl-2,3-dihydropyrano[2,3-f]chromen-	ESR2
Radix Glycyrrhizae (RG)	MOL004805	(2S)-2-[4-hydroxy-3-(3-methylbut-2-enyl)phenyl]-8,8-dimethyl-2,3-dihydropyrano[2,3-f]chromen-	MAPK14
Radix Glycyrrhizae (RG)	MOL004805	(2S)-2-[4-hydroxy-3-(3-methylbut-2-enyl)phenyl]-8,8-dimethyl-2,3-dihydropyrano[2,3-f]chromen-	GSK3B
Radix Glycyrrhizae (RG)	MOL004805	(2S)-2-[4-hydroxy-3-(3-methylbut-2-enyl)phenyl]-8,8-dimethyl-2,3-dihydropyrano[2,3-f]chromen-	CAMKMT
Radix Glycyrrhizae (RG)	MOL004806	euchrenone	NOS2
Radix Glycyrrhizae (RG)	MOL004806	euchrenone	KCNH2
Radix Glycyrrhizae (RG)	MOL004806	euchrenone	ESR1
Radix Glycyrrhizae (RG)	MOL004806	euchrenone	SCN5A
Radix Glycyrrhizae (RG)	MOL004806	euchrenone	PTGS2

Radix Glycyrrhizae (RG)	MOL004806	euchrenone	ESR2
Radix Glycyrrhizae (RG)	MOL004806	euchrenone	BACE2
Radix Glycyrrhizae (RG)	MOL004806	euchrenone	CAMKMT
Radix Glycyrrhizae (RG)	MOL004808	glyasperin B	NOS2
Radix Glycyrrhizae (RG)	MOL004808	glyasperin B	ESR1
Radix Glycyrrhizae (RG)	MOL004808	glyasperin B	AR
Radix Glycyrrhizae (RG)	MOL004808	glyasperin B	PPARG
Radix Glycyrrhizae (RG)	MOL004808	glyasperin B	PTGS2
Radix Glycyrrhizae (RG)	MOL004808	glyasperin B	F7
Radix Glycyrrhizae (RG)	MOL004808	glyasperin B	KDR
Radix Glycyrrhizae (RG)	MOL004808	glyasperin B	ACHE
Radix Glycyrrhizae (RG)	MOL004808	glyasperin B	ESR2
Radix Glycyrrhizae (RG)	MOL004808	glyasperin B	DPP4
Radix Glycyrrhizae (RG)	MOL004808	glyasperin B	GSK3B
Radix Glycyrrhizae (RG)	MOL004808	glyasperin B	HSP90AB1
Radix Glycyrrhizae (RG)	MOL004808	glyasperin B	CDK2
Radix Glycyrrhizae (RG)	MOL004808	glyasperin B	PRSS1
Radix Glycyrrhizae (RG)	MOL004808	glyasperin B	CCNA2
Radix Glycyrrhizae (RG)	MOL004808	glyasperin B	NCOA2
Radix Glycyrrhizae (RG)	MOL004808	glyasperin B	CAMKMT
Radix Glycyrrhizae (RG)	MOL004810	glyasperin F	NOS2
Radix Glycyrrhizae (RG)	MOL004810	glyasperin F	PTGS1
Radix Glycyrrhizae (RG)	MOL004810	glyasperin F	ESR1
Radix Glycyrrhizae (RG)	MOL004810	glyasperin F	AR
Radix Glycyrrhizae (RG)	MOL004810	glyasperin F	SCN5A
Radix Glycyrrhizae (RG)	MOL004810	glyasperin F	PPARG
Radix Glycyrrhizae (RG)	MOL004810	glyasperin F	PTGS2
Radix Glycyrrhizae (RG)	MOL004810	glyasperin F	ESR2
Radix Glycyrrhizae (RG)	MOL004810	glyasperin F	MAPK14
Radix Glycyrrhizae (RG)	MOL004810	glyasperin F	GSK3B
Radix Glycyrrhizae (RG)	MOL004810	glyasperin F	HSP90AB1
Radix Glycyrrhizae (RG)	MOL004810	glyasperin F	CDK2
Radix Glycyrrhizae (RG)	MOL004810	glyasperin F	PRSS1
Radix Glycyrrhizae (RG)	MOL004810	glyasperin F	CCNA2
Radix Glycyrrhizae (RG)	MOL004810	glyasperin F	CAMKMT
Radix Glycyrrhizae (RG)	MOL004811	Glyasperin C	NOS2
Radix Glycyrrhizae (RG)	MOL004811	Glyasperin C	KCNH2
Radix Glycyrrhizae (RG)	MOL004811	Glyasperin C	ESR1
Radix Glycyrrhizae (RG)	MOL004811	Glyasperin C	AR
Radix Glycyrrhizae (RG)	MOL004811	Glyasperin C	SCN5A
Radix Glycyrrhizae (RG)	MOL004811	Glyasperin C	PPARG
Radix Glycyrrhizae (RG)	MOL004811	Glyasperin C	PTGS2
Radix Glycyrrhizae (RG)	MOL004811	Glyasperin C	RXRA
Radix Glycyrrhizae (RG)	MOL004811	Glyasperin C	ACHE
Radix Glycyrrhizae (RG)	MOL004811	Glyasperin C	ESR2
Radix Glycyrrhizae (RG)	MOL004811	Glyasperin C	DPP4
Radix Glycyrrhizae (RG)	MOL004811	Glyasperin C	MAPK14
Radix Glycyrrhizae (RG)	MOL004811	Glyasperin C	GSK3B
Radix Glycyrrhizae (RG)	MOL004811	Glyasperin C	HSP90AB1
Radix Glycyrrhizae (RG)	MOL004811	Glyasperin C	CDK2
Radix Glycyrrhizae (RG)	MOL004811	Glyasperin C	CHEK1
Radix Glycyrrhizae (RG)	MOL004811	Glyasperin C	PRSS1
Radix Glycyrrhizae (RG)	MOL004811	Glyasperin C	CCNA2
Radix Glycyrrhizae (RG)	MOL004811	Glyasperin C	NCOA2
Radix Glycyrrhizae (RG)	MOL004811	Glyasperin C	CAMKMT
Radix Glycyrrhizae (RG)	MOL004814	Isotrifoliol	NOS2
Radix Glycyrrhizae (RG)	MOL004814	Isotrifoliol	ESR1
Radix Glycyrrhizae (RG)	MOL004814	Isotrifoliol	AR
Radix Glycyrrhizae (RG)	MOL004814	Isotrifoliol	PTGS2
Radix Glycyrrhizae (RG)	MOL004814	Isotrifoliol	ESR2
Radix Glycyrrhizae (RG)	MOL004814	Isotrifoliol	MAPK14
Radix Glycyrrhizae (RG)	MOL004814	Isotrifoliol	GSK3B
Radix Glycyrrhizae (RG)	MOL004814	Isotrifoliol	HSP90AB1

Radix Glycyrrhizae (RG)	MOL004814	Isotrifoliol	CDK2
Radix Glycyrrhizae (RG)	MOL004814	Isotrifoliol	CHEK1
Radix Glycyrrhizae (RG)	MOL004814	Isotrifoliol	CCNA2
Radix Glycyrrhizae (RG)	MOL004815	(E)-1-(2,4-dihydroxyphenyl)-3-(2,2-dimethylchromen-6-yl)prop-2-en-1-one	NOS2
Radix Glycyrrhizae (RG)	MOL004815	(E)-1-(2,4-dihydroxyphenyl)-3-(2,2-dimethylchromen-6-yl)prop-2-en-1-one	PTGS1
Radix Glycyrrhizae (RG)	MOL004815	(E)-1-(2,4-dihydroxyphenyl)-3-(2,2-dimethylchromen-6-yl)prop-2-en-1-one	ESR1
Radix Glycyrrhizae (RG)	MOL004815	(E)-1-(2,4-dihydroxyphenyl)-3-(2,2-dimethylchromen-6-yl)prop-2-en-1-one	AR
Radix Glycyrrhizae (RG)	MOL004815	(E)-1-(2,4-dihydroxyphenyl)-3-(2,2-dimethylchromen-6-yl)prop-2-en-1-one	SCN5A
Radix Glycyrrhizae (RG)	MOL004815	(E)-1-(2,4-dihydroxyphenyl)-3-(2,2-dimethylchromen-6-yl)prop-2-en-1-one	PPARG
Radix Glycyrrhizae (RG)	MOL004815	(E)-1-(2,4-dihydroxyphenyl)-3-(2,2-dimethylchromen-6-yl)prop-2-en-1-one	PTGS2
Radix Glycyrrhizae (RG)	MOL004815	(E)-1-(2,4-dihydroxyphenyl)-3-(2,2-dimethylchromen-6-yl)prop-2-en-1-one	CA2
Radix Glycyrrhizae (RG)	MOL004815	(E)-1-(2,4-dihydroxyphenyl)-3-(2,2-dimethylchromen-6-yl)prop-2-en-1-one	RXRA
Radix Glycyrrhizae (RG)	MOL004815	(E)-1-(2,4-dihydroxyphenyl)-3-(2,2-dimethylchromen-6-yl)prop-2-en-1-one	ADRA1B
Radix Glycyrrhizae (RG)	MOL004815	(E)-1-(2,4-dihydroxyphenyl)-3-(2,2-dimethylchromen-6-yl)prop-2-en-1-one	ESR2
Radix Glycyrrhizae (RG)	MOL004815	(E)-1-(2,4-dihydroxyphenyl)-3-(2,2-dimethylchromen-6-yl)prop-2-en-1-one	MAPK14
Radix Glycyrrhizae (RG)	MOL004815	(E)-1-(2,4-dihydroxyphenyl)-3-(2,2-dimethylchromen-6-yl)prop-2-en-1-one	GSK3B
Radix Glycyrrhizae (RG)	MOL004815	(E)-1-(2,4-dihydroxyphenyl)-3-(2,2-dimethylchromen-6-yl)prop-2-en-1-one	CDK2
Radix Glycyrrhizae (RG)	MOL004815	(E)-1-(2,4-dihydroxyphenyl)-3-(2,2-dimethylchromen-6-yl)prop-2-en-1-one	CHEK1
Radix Glycyrrhizae (RG)	MOL004815	(E)-1-(2,4-dihydroxyphenyl)-3-(2,2-dimethylchromen-6-yl)prop-2-en-1-one	CCNA2
Radix Glycyrrhizae (RG)	MOL004815	(E)-1-(2,4-dihydroxyphenyl)-3-(2,2-dimethylchromen-6-yl)prop-2-en-1-one	NCOA2
Radix Glycyrrhizae (RG)	MOL004815	(E)-1-(2,4-dihydroxyphenyl)-3-(2,2-dimethylchromen-6-yl)prop-2-en-1-one	CAMKMT
Radix Glycyrrhizae (RG)	MOL004820	kanzonols W	NOS2
Radix Glycyrrhizae (RG)	MOL004820	kanzonols W	PTGS1
Radix Glycyrrhizae (RG)	MOL004820	kanzonols W	ESR1
Radix Glycyrrhizae (RG)	MOL004820	kanzonols W	AR
Radix Glycyrrhizae (RG)	MOL004820	kanzonols W	SCN5A
Radix Glycyrrhizae (RG)	MOL004820	kanzonols W	PPARG

Radix Glycyrrhizae (RG)	MOL004820	kanzonols W	PTGS2
Radix Glycyrrhizae (RG)	MOL004820	kanzonols W	RXRA
Radix Glycyrrhizae (RG)	MOL004820	kanzonols W	ESR2
Radix Glycyrrhizae (RG)	MOL004820	kanzonols W	MAPK14
Radix Glycyrrhizae (RG)	MOL004820	kanzonols W	GSK3B
Radix Glycyrrhizae (RG)	MOL004820	kanzonols W	CDK2
Radix Glycyrrhizae (RG)	MOL004820	kanzonols W	CHEK1
Radix Glycyrrhizae (RG)	MOL004820	kanzonols W	PRSS1
Radix Glycyrrhizae (RG)	MOL004820	kanzonols W	CCNA2
Radix Glycyrrhizae (RG)	MOL004820	kanzonols W	NCOA2
Radix Glycyrrhizae (RG)	MOL004820	kanzonols W	NCOA1
Radix Glycyrrhizae (RG)	MOL004820	kanzonols W	CAMKMT
Radix Glycyrrhizae (RG)	MOL004824	(2S)-6-(2,4-dihydroxyphenyl)-2-(2-hydroxypropan-2-yl)-4-methoxy-2,3-dihydrofuro[3,2-g]chromen-7-one	NOS2
Radix Glycyrrhizae (RG)	MOL004824	(2S)-6-(2,4-dihydroxyphenyl)-2-(2-hydroxypropan-2-yl)-4-methoxy-2,3-dihydrofuro[3,2-g]chromen-7-one	ESR1
Radix Glycyrrhizae (RG)	MOL004824	(2S)-6-(2,4-dihydroxyphenyl)-2-(2-hydroxypropan-2-yl)-4-methoxy-2,3-dihydrofuro[3,2-g]chromen-7-one	AR
Radix Glycyrrhizae (RG)	MOL004824	(2S)-6-(2,4-dihydroxyphenyl)-2-(2-hydroxypropan-2-yl)-4-methoxy-2,3-dihydrofuro[3,2-g]chromen-7-one	PPARG
Radix Glycyrrhizae (RG)	MOL004824	(2S)-6-(2,4-dihydroxyphenyl)-2-(2-hydroxypropan-2-yl)-4-methoxy-2,3-dihydrofuro[3,2-g]chromen-7-one	PTGS2
Radix Glycyrrhizae (RG)	MOL004824	(2S)-6-(2,4-dihydroxyphenyl)-2-(2-hydroxypropan-2-yl)-4-methoxy-2,3-dihydrofuro[3,2-g]chromen-7-one	F7
Radix Glycyrrhizae (RG)	MOL004824	(2S)-6-(2,4-dihydroxyphenyl)-2-(2-hydroxypropan-2-yl)-4-methoxy-2,3-dihydrofuro[3,2-g]chromen-7-one	KDR
Radix Glycyrrhizae (RG)	MOL004824	(2S)-6-(2,4-dihydroxyphenyl)-2-(2-hydroxypropan-2-yl)-4-methoxy-2,3-dihydrofuro[3,2-g]chromen-7-one	ACHE
Radix Glycyrrhizae (RG)	MOL004824	(2S)-6-(2,4-dihydroxyphenyl)-2-(2-hydroxypropan-2-yl)-4-methoxy-2,3-dihydrofuro[3,2-g]chromen-7-one	ESR2
Radix Glycyrrhizae (RG)	MOL004824	(2S)-6-(2,4-dihydroxyphenyl)-2-(2-hydroxypropan-2-yl)-4-methoxy-2,3-dihydrofuro[3,2-g]chromen-7-one	DPP4
Radix Glycyrrhizae (RG)	MOL004824	(2S)-6-(2,4-dihydroxyphenyl)-2-(2-hydroxypropan-2-yl)-4-methoxy-2,3-dihydrofuro[3,2-g]chromen-7-one	MAPK14
Radix Glycyrrhizae (RG)	MOL004824	(2S)-6-(2,4-dihydroxyphenyl)-2-(2-hydroxypropan-2-yl)-4-methoxy-2,3-dihydrofuro[3,2-g]chromen-7-one	GSK3B

Radix Glycyrrhizae (RG)	MOL004824	(2S)-6-(2,4-dihydroxyphenyl)-2-(2-hydroxypropan-2-yl)-4-methoxy-2,3-dihydrofuro[3,2-g]chromen-7-one	CDK2
Radix Glycyrrhizae (RG)	MOL004824	(2S)-6-(2,4-dihydroxyphenyl)-2-(2-hydroxypropan-2-yl)-4-methoxy-2,3-dihydrofuro[3,2-g]chromen-7-one	CHEK1
Radix Glycyrrhizae (RG)	MOL004824	(2S)-6-(2,4-dihydroxyphenyl)-2-(2-hydroxypropan-2-yl)-4-methoxy-2,3-dihydrofuro[3,2-g]chromen-7-one	PRSS1
Radix Glycyrrhizae (RG)	MOL004824	(2S)-6-(2,4-dihydroxyphenyl)-2-(2-hydroxypropan-2-yl)-4-methoxy-2,3-dihydrofuro[3,2-g]chromen-7-one	CCNA2
Radix Glycyrrhizae (RG)	MOL004824	(2S)-6-(2,4-dihydroxyphenyl)-2-(2-hydroxypropan-2-yl)-4-methoxy-2,3-dihydrofuro[3,2-g]chromen-7-one	CAMKMT
Radix Glycyrrhizae (RG)	MOL004827	Semilicoisoflavone B	NOS2
Radix Glycyrrhizae (RG)	MOL004827	Semilicoisoflavone B	ESR1
Radix Glycyrrhizae (RG)	MOL004827	Semilicoisoflavone B	AR
Radix Glycyrrhizae (RG)	MOL004827	Semilicoisoflavone B	SCN5A
Radix Glycyrrhizae (RG)	MOL004827	Semilicoisoflavone B	PPARG
Radix Glycyrrhizae (RG)	MOL004827	Semilicoisoflavone B	PTGS2
Radix Glycyrrhizae (RG)	MOL004827	Semilicoisoflavone B	F7
Radix Glycyrrhizae (RG)	MOL004827	Semilicoisoflavone B	ACHE
Radix Glycyrrhizae (RG)	MOL004827	Semilicoisoflavone B	GSK3B
Radix Glycyrrhizae (RG)	MOL004827	Semilicoisoflavone B	HSP90AB1
Radix Glycyrrhizae (RG)	MOL004827	Semilicoisoflavone B	CDK2
Radix Glycyrrhizae (RG)	MOL004827	Semilicoisoflavone B	CHEK1
Radix Glycyrrhizae (RG)	MOL004827	Semilicoisoflavone B	PRSS1
Radix Glycyrrhizae (RG)	MOL004827	Semilicoisoflavone B	CAMKMT
Radix Glycyrrhizae (RG)	MOL004828	Glepidotin A	NOS2
Radix Glycyrrhizae (RG)	MOL004828	Glepidotin A	PTGS1
Radix Glycyrrhizae (RG)	MOL004828	Glepidotin A	ESR1
Radix Glycyrrhizae (RG)	MOL004828	Glepidotin A	AR
Radix Glycyrrhizae (RG)	MOL004828	Glepidotin A	SCN5A
Radix Glycyrrhizae (RG)	MOL004828	Glepidotin A	PPARG
Radix Glycyrrhizae (RG)	MOL004828	Glepidotin A	PTGS2
Radix Glycyrrhizae (RG)	MOL004828	Glepidotin A	F7
Radix Glycyrrhizae (RG)	MOL004828	Glepidotin A	KDR
Radix Glycyrrhizae (RG)	MOL004828	Glepidotin A	RXRA
Radix Glycyrrhizae (RG)	MOL004828	Glepidotin A	DPP4
Radix Glycyrrhizae (RG)	MOL004828	Glepidotin A	MAPK14
Radix Glycyrrhizae (RG)	MOL004828	Glepidotin A	GSK3B
Radix Glycyrrhizae (RG)	MOL004828	Glepidotin A	HSP90AB1
Radix Glycyrrhizae (RG)	MOL004828	Glepidotin A	CDK2
Radix Glycyrrhizae (RG)	MOL004828	Glepidotin A	CHEK1
Radix Glycyrrhizae (RG)	MOL004828	Glepidotin A	IGHG1
Radix Glycyrrhizae (RG)	MOL004828	Glepidotin A	PRSS1
Radix Glycyrrhizae (RG)	MOL004828	Glepidotin A	CCNA2
Radix Glycyrrhizae (RG)	MOL004828	Glepidotin A	CAMKMT
Radix Glycyrrhizae (RG)	MOL004829	Glepidotin B	PTGS1
Radix Glycyrrhizae (RG)	MOL004829	Glepidotin B	ESR1
Radix Glycyrrhizae (RG)	MOL004829	Glepidotin B	SCN5A
Radix Glycyrrhizae (RG)	MOL004829	Glepidotin B	PTGS2
Radix Glycyrrhizae (RG)	MOL004829	Glepidotin B	F7
Radix Glycyrrhizae (RG)	MOL004829	Glepidotin B	RXRA
Radix Glycyrrhizae (RG)	MOL004829	Glepidotin B	ADRA1B
Radix Glycyrrhizae (RG)	MOL004829	Glepidotin B	HSP90AB1
Radix Glycyrrhizae (RG)	MOL004829	Glepidotin B	IGHG1

Radix Glycyrrhizae (RG)	MOL004829	Glepidotin B	NCOA1
Radix Glycyrrhizae (RG)	MOL004829	Glepidotin B	CAMKMT
Radix Glycyrrhizae (RG)	MOL004833	Phaseolinisoflavan	NOS2
Radix Glycyrrhizae (RG)	MOL004833	Phaseolinisoflavan	CHRM1
Radix Glycyrrhizae (RG)	MOL004833	Phaseolinisoflavan	ESR1
Radix Glycyrrhizae (RG)	MOL004833	Phaseolinisoflavan	AR
Radix Glycyrrhizae (RG)	MOL004833	Phaseolinisoflavan	SCN5A
Radix Glycyrrhizae (RG)	MOL004833	Phaseolinisoflavan	PPARG
Radix Glycyrrhizae (RG)	MOL004833	Phaseolinisoflavan	PTGS2
Radix Glycyrrhizae (RG)	MOL004833	Phaseolinisoflavan	RXRA
Radix Glycyrrhizae (RG)	MOL004833	Phaseolinisoflavan	ACHE
Radix Glycyrrhizae (RG)	MOL004833	Phaseolinisoflavan	ADRA1B
Radix Glycyrrhizae (RG)	MOL004833	Phaseolinisoflavan	ADRB2
Radix Glycyrrhizae (RG)	MOL004833	Phaseolinisoflavan	ESR2
Radix Glycyrrhizae (RG)	MOL004833	Phaseolinisoflavan	MAPK14
Radix Glycyrrhizae (RG)	MOL004833	Phaseolinisoflavan	GSK3B
Radix Glycyrrhizae (RG)	MOL004833	Phaseolinisoflavan	CDK2
Radix Glycyrrhizae (RG)	MOL004833	Phaseolinisoflavan	CHEK1
Radix Glycyrrhizae (RG)	MOL004833	Phaseolinisoflavan	PRSS1
Radix Glycyrrhizae (RG)	MOL004833	Phaseolinisoflavan	CCNA2
Radix Glycyrrhizae (RG)	MOL004833	Phaseolinisoflavan	NCOA1
Radix Glycyrrhizae (RG)	MOL004833	Phaseolinisoflavan	CAMKMT
Radix Glycyrrhizae (RG)	MOL004835	Glypallichalcone	NOS2
Radix Glycyrrhizae (RG)	MOL004835	Glypallichalcone	PTGS1
Radix Glycyrrhizae (RG)	MOL004835	Glypallichalcone	CHRM1
Radix Glycyrrhizae (RG)	MOL004835	Glypallichalcone	ESR1
Radix Glycyrrhizae (RG)	MOL004835	Glypallichalcone	AR
Radix Glycyrrhizae (RG)	MOL004835	Glypallichalcone	SCN5A
Radix Glycyrrhizae (RG)	MOL004835	Glypallichalcone	PPARG
Radix Glycyrrhizae (RG)	MOL004835	Glypallichalcone	PTGS2
Radix Glycyrrhizae (RG)	MOL004835	Glypallichalcone	CA2
Radix Glycyrrhizae (RG)	MOL004835	Glypallichalcone	ADRA1B
Radix Glycyrrhizae (RG)	MOL004835	Glypallichalcone	SLC6A3
Radix Glycyrrhizae (RG)	MOL004835	Glypallichalcone	ADRB2
Radix Glycyrrhizae (RG)	MOL004835	Glypallichalcone	SLC6A4
Radix Glycyrrhizae (RG)	MOL004835	Glypallichalcone	ESR2
Radix Glycyrrhizae (RG)	MOL004835	Glypallichalcone	MAPK14
Radix Glycyrrhizae (RG)	MOL004835	Glypallichalcone	GSK3B
Radix Glycyrrhizae (RG)	MOL004835	Glypallichalcone	HSP90AB1
Radix Glycyrrhizae (RG)	MOL004835	Glypallichalcone	CDK2
Radix Glycyrrhizae (RG)	MOL004835	Glypallichalcone	LTA4H
Radix Glycyrrhizae (RG)	MOL004835	Glypallichalcone	MAOB
Radix Glycyrrhizae (RG)	MOL004835	Glypallichalcone	CHEK1
Radix Glycyrrhizae (RG)	MOL004835	Glypallichalcone	CCNA2
Radix Glycyrrhizae (RG)	MOL004835	Glypallichalcone	NCOA1
Radix Glycyrrhizae (RG)	MOL004835	Glypallichalcone	PKIA
Radix Glycyrrhizae (RG)	MOL004835	Glypallichalcone	CAMKMT
Radix Glycyrrhizae (RG)	MOL004838	8-(6-hydroxy-2-benzofuranyl)-2,2-dimethyl-5-chromenol	NOS2
Radix Glycyrrhizae (RG)	MOL004838	8-(6-hydroxy-2-benzofuranyl)-2,2-dimethyl-5-chromenol	ESR1
Radix Glycyrrhizae (RG)	MOL004838	8-(6-hydroxy-2-benzofuranyl)-2,2-dimethyl-5-chromenol	PTGS2
Radix Glycyrrhizae (RG)	MOL004838	8-(6-hydroxy-2-benzofuranyl)-2,2-dimethyl-5-chromenol	RXRA
Radix Glycyrrhizae (RG)	MOL004838	8-(6-hydroxy-2-benzofuranyl)-2,2-dimethyl-5-chromenol	HSP90AB1
Radix Glycyrrhizae (RG)	MOL004841	Licochalcone B	NOS2
Radix Glycyrrhizae (RG)	MOL004841	Licochalcone B	PTGS1
Radix Glycyrrhizae (RG)	MOL004841	Licochalcone B	ESR1
Radix Glycyrrhizae (RG)	MOL004841	Licochalcone B	AR
Radix Glycyrrhizae (RG)	MOL004841	Licochalcone B	PPARG
Radix Glycyrrhizae (RG)	MOL004841	Licochalcone B	PTGS2

Radix Glycyrrhizae (RG)	MOL004841	Licochalcone B	CA2
Radix Glycyrrhizae (RG)	MOL004841	Licochalcone B	ADRB2
Radix Glycyrrhizae (RG)	MOL004841	Licochalcone B	ESR2
Radix Glycyrrhizae (RG)	MOL004841	Licochalcone B	MAPK14
Radix Glycyrrhizae (RG)	MOL004841	Licochalcone B	GSK3B
Radix Glycyrrhizae (RG)	MOL004841	Licochalcone B	HSP90AB1
Radix Glycyrrhizae (RG)	MOL004841	Licochalcone B	CDK2
Radix Glycyrrhizae (RG)	MOL004841	Licochalcone B	CHEK1
Radix Glycyrrhizae (RG)	MOL004841	Licochalcone B	CCNA2
Radix Glycyrrhizae (RG)	MOL004841	Licochalcone B	CAMKMT
Radix Glycyrrhizae (RG)	MOL004848	licochalcone G	NOS2
Radix Glycyrrhizae (RG)	MOL004848	licochalcone G	ESR1
Radix Glycyrrhizae (RG)	MOL004848	licochalcone G	AR
Radix Glycyrrhizae (RG)	MOL004848	licochalcone G	PPARG
Radix Glycyrrhizae (RG)	MOL004848	licochalcone G	PTGS2
Radix Glycyrrhizae (RG)	MOL004848	licochalcone G	KDR
Radix Glycyrrhizae (RG)	MOL004848	licochalcone G	ESR2
Radix Glycyrrhizae (RG)	MOL004848	licochalcone G	MAPK14
Radix Glycyrrhizae (RG)	MOL004848	licochalcone G	GSK3B
Radix Glycyrrhizae (RG)	MOL004848	licochalcone G	HSP90AB1
Radix Glycyrrhizae (RG)	MOL004848	licochalcone G	CDK2
Radix Glycyrrhizae (RG)	MOL004848	licochalcone G	IGHG1
Radix Glycyrrhizae (RG)	MOL004848	licochalcone G	CCNA2
Radix Glycyrrhizae (RG)	MOL004848	licochalcone G	NCOA2
Radix Glycyrrhizae (RG)	MOL004848	licochalcone G	CAMKMT
Radix Glycyrrhizae (RG)	MOL004849	3-(2,4-dihydroxyphenyl)-8-(1,1-dimethylprop-2-enyl)-7-hydroxy-5-methoxy-coumarin	NOS2
Radix Glycyrrhizae (RG)	MOL004849	3-(2,4-dihydroxyphenyl)-8-(1,1-dimethylprop-2-enyl)-7-hydroxy-5-methoxy-coumarin	KCNH2
Radix Glycyrrhizae (RG)	MOL004849	3-(2,4-dihydroxyphenyl)-8-(1,1-dimethylprop-2-enyl)-7-hydroxy-5-methoxy-coumarin	ESR1
Radix Glycyrrhizae (RG)	MOL004849	3-(2,4-dihydroxyphenyl)-8-(1,1-dimethylprop-2-enyl)-7-hydroxy-5-methoxy-coumarin	AR
Radix Glycyrrhizae (RG)	MOL004849	3-(2,4-dihydroxyphenyl)-8-(1,1-dimethylprop-2-enyl)-7-hydroxy-5-methoxy-coumarin	PPARG
Radix Glycyrrhizae (RG)	MOL004849	3-(2,4-dihydroxyphenyl)-8-(1,1-dimethylprop-2-enyl)-7-hydroxy-5-methoxy-coumarin	PTGS2
Radix Glycyrrhizae (RG)	MOL004849	3-(2,4-dihydroxyphenyl)-8-(1,1-dimethylprop-2-enyl)-7-hydroxy-5-methoxy-coumarin	F7
Radix Glycyrrhizae (RG)	MOL004849	3-(2,4-dihydroxyphenyl)-8-(1,1-dimethylprop-2-enyl)-7-hydroxy-5-methoxy-coumarin	KDR
Radix Glycyrrhizae (RG)	MOL004849	3-(2,4-dihydroxyphenyl)-8-(1,1-dimethylprop-2-enyl)-7-hydroxy-5-methoxy-coumarin	ESR2
Radix Glycyrrhizae (RG)	MOL004849	3-(2,4-dihydroxyphenyl)-8-(1,1-dimethylprop-2-enyl)-7-hydroxy-5-methoxy-coumarin	DPP4
Radix Glycyrrhizae (RG)	MOL004849	3-(2,4-dihydroxyphenyl)-8-(1,1-dimethylprop-2-enyl)-7-hydroxy-5-methoxy-coumarin	MAPK14
Radix Glycyrrhizae (RG)	MOL004849	3-(2,4-dihydroxyphenyl)-8-(1,1-dimethylprop-2-enyl)-7-hydroxy-5-methoxy-coumarin	GSK3B

Radix Glycyrrhizae (RG)	MOL004849	3-(2,4-dihydroxyphenyl)-8-(1,1-dimethylprop-2-enyl)-7-hydroxy-5-methoxy-coumarin	HSP90AB1
Radix Glycyrrhizae (RG)	MOL004849	3-(2,4-dihydroxyphenyl)-8-(1,1-dimethylprop-2-enyl)-7-hydroxy-5-methoxy-coumarin	CDK2
Radix Glycyrrhizae (RG)	MOL004849	3-(2,4-dihydroxyphenyl)-8-(1,1-dimethylprop-2-enyl)-7-hydroxy-5-methoxy-coumarin	CHEK1
Radix Glycyrrhizae (RG)	MOL004849	3-(2,4-dihydroxyphenyl)-8-(1,1-dimethylprop-2-enyl)-7-hydroxy-5-methoxy-coumarin	PRSS1
Radix Glycyrrhizae (RG)	MOL004849	3-(2,4-dihydroxyphenyl)-8-(1,1-dimethylprop-2-enyl)-7-hydroxy-5-methoxy-coumarin	NCOA2
Radix Glycyrrhizae (RG)	MOL004849	3-(2,4-dihydroxyphenyl)-8-(1,1-dimethylprop-2-enyl)-7-hydroxy-5-methoxy-coumarin	NCOA1
Radix Glycyrrhizae (RG)	MOL004849	3-(2,4-dihydroxyphenyl)-8-(1,1-dimethylprop-2-enyl)-7-hydroxy-5-methoxy-coumarin	CAMKMT
Radix Glycyrrhizae (RG)	MOL004855	Licoricone	NOS2
Radix Glycyrrhizae (RG)	MOL004855	Licoricone	KCNH2
Radix Glycyrrhizae (RG)	MOL004855	Licoricone	ESR1
Radix Glycyrrhizae (RG)	MOL004855	Licoricone	AR
Radix Glycyrrhizae (RG)	MOL004855	Licoricone	PPARG
Radix Glycyrrhizae (RG)	MOL004855	Licoricone	PTGS2
Radix Glycyrrhizae (RG)	MOL004855	Licoricone	KDR
Radix Glycyrrhizae (RG)	MOL004855	Licoricone	CHEK1
Radix Glycyrrhizae (RG)	MOL004855	Licoricone	PRSS1
Radix Glycyrrhizae (RG)	MOL004855	Licoricone	NCOA2
Radix Glycyrrhizae (RG)	MOL004855	Licoricone	CAMKMT
Radix Glycyrrhizae (RG)	MOL004856	Gancaonin A	NOS2
Radix Glycyrrhizae (RG)	MOL004856	Gancaonin A	ESR1
Radix Glycyrrhizae (RG)	MOL004856	Gancaonin A	AR
Radix Glycyrrhizae (RG)	MOL004856	Gancaonin A	SCN5A
Radix Glycyrrhizae (RG)	MOL004856	Gancaonin A	PPARG
Radix Glycyrrhizae (RG)	MOL004856	Gancaonin A	PTGS2
Radix Glycyrrhizae (RG)	MOL004856	Gancaonin A	ACHE
Radix Glycyrrhizae (RG)	MOL004856	Gancaonin A	ESR2
Radix Glycyrrhizae (RG)	MOL004856	Gancaonin A	DPP4
Radix Glycyrrhizae (RG)	MOL004856	Gancaonin A	GSK3B
Radix Glycyrrhizae (RG)	MOL004856	Gancaonin A	HSP90AB1
Radix Glycyrrhizae (RG)	MOL004856	Gancaonin A	CHEK1
Radix Glycyrrhizae (RG)	MOL004856	Gancaonin A	PRSS1
Radix Glycyrrhizae (RG)	MOL004856	Gancaonin A	CCNA2
Radix Glycyrrhizae (RG)	MOL004856	Gancaonin A	NCOA2
Radix Glycyrrhizae (RG)	MOL004856	Gancaonin A	CAMKMT
Radix Glycyrrhizae (RG)	MOL004857	Gancaonin B	NOS2
Radix Glycyrrhizae (RG)	MOL004857	Gancaonin B	ESR1
Radix Glycyrrhizae (RG)	MOL004857	Gancaonin B	AR
Radix Glycyrrhizae (RG)	MOL004857	Gancaonin B	PPARG
Radix Glycyrrhizae (RG)	MOL004857	Gancaonin B	PTGS2
Radix Glycyrrhizae (RG)	MOL004857	Gancaonin B	F7
Radix Glycyrrhizae (RG)	MOL004857	Gancaonin B	KDR
Radix Glycyrrhizae (RG)	MOL004857	Gancaonin B	ADRA1B
Radix Glycyrrhizae (RG)	MOL004857	Gancaonin B	ADRB2
Radix Glycyrrhizae (RG)	MOL004857	Gancaonin B	ESR2
Radix Glycyrrhizae (RG)	MOL004857	Gancaonin B	DPP4
Radix Glycyrrhizae (RG)	MOL004857	Gancaonin B	GSK3B
Radix Glycyrrhizae (RG)	MOL004857	Gancaonin B	HSP90AB1
Radix Glycyrrhizae (RG)	MOL004857	Gancaonin B	CHEK1
Radix Glycyrrhizae (RG)	MOL004857	Gancaonin B	PRSS1

Radix Glycyrrhizae (RG)	MOL004857	Gancaonin B	CCNA2
Radix Glycyrrhizae (RG)	MOL004857	Gancaonin B	NCOA2
Radix Glycyrrhizae (RG)	MOL004857	Gancaonin B	CAMKMT
Radix Glycyrrhizae (RG)	MOL004863	3-(3,4-dihydroxyphenyl)-5,7-dihydroxy-8-(3-methylbut-2-enyl)chromone	NOS2
Radix Glycyrrhizae (RG)	MOL004863	3-(3,4-dihydroxyphenyl)-5,7-dihydroxy-8-(3-methylbut-2-enyl)chromone	ESR1
Radix Glycyrrhizae (RG)	MOL004863	3-(3,4-dihydroxyphenyl)-5,7-dihydroxy-8-(3-methylbut-2-enyl)chromone	AR
Radix Glycyrrhizae (RG)	MOL004863	3-(3,4-dihydroxyphenyl)-5,7-dihydroxy-8-(3-methylbut-2-enyl)chromone	PPARG
Radix Glycyrrhizae (RG)	MOL004863	3-(3,4-dihydroxyphenyl)-5,7-dihydroxy-8-(3-methylbut-2-enyl)chromone	PTGS2
Radix Glycyrrhizae (RG)	MOL004863	3-(3,4-dihydroxyphenyl)-5,7-dihydroxy-8-(3-methylbut-2-enyl)chromone	MAPK14
Radix Glycyrrhizae (RG)	MOL004863	3-(3,4-dihydroxyphenyl)-5,7-dihydroxy-8-(3-methylbut-2-enyl)chromone	GSK3B
Radix Glycyrrhizae (RG)	MOL004863	3-(3,4-dihydroxyphenyl)-5,7-dihydroxy-8-(3-methylbut-2-enyl)chromone	HSP90AB1
Radix Glycyrrhizae (RG)	MOL004863	3-(3,4-dihydroxyphenyl)-5,7-dihydroxy-8-(3-methylbut-2-enyl)chromone	CDK2
Radix Glycyrrhizae (RG)	MOL004863	3-(3,4-dihydroxyphenyl)-5,7-dihydroxy-8-(3-methylbut-2-enyl)chromone	CHEK1
Radix Glycyrrhizae (RG)	MOL004863	3-(3,4-dihydroxyphenyl)-5,7-dihydroxy-8-(3-methylbut-2-enyl)chromone	PRSS1
Radix Glycyrrhizae (RG)	MOL004863	3-(3,4-dihydroxyphenyl)-5,7-dihydroxy-8-(3-methylbut-2-enyl)chromone	CCNA2
Radix Glycyrrhizae (RG)	MOL004863	3-(3,4-dihydroxyphenyl)-5,7-dihydroxy-8-(3-methylbut-2-enyl)chromone	NCOA2
Radix Glycyrrhizae (RG)	MOL004863	3-(3,4-dihydroxyphenyl)-5,7-dihydroxy-8-(3-methylbut-2-enyl)chromone	CAMKMT
Radix Glycyrrhizae (RG)	MOL004864	5,7-dihydroxy-3-(4-methoxyphenyl)-8-(3-methylbut-2-enyl)chromone	NOS2
Radix Glycyrrhizae (RG)	MOL004864	5,7-dihydroxy-3-(4-methoxyphenyl)-8-(3-methylbut-2-enyl)chromone	KCNH2
Radix Glycyrrhizae (RG)	MOL004864	5,7-dihydroxy-3-(4-methoxyphenyl)-8-(3-methylbut-2-enyl)chromone	ESR1
Radix Glycyrrhizae (RG)	MOL004864	5,7-dihydroxy-3-(4-methoxyphenyl)-8-(3-methylbut-2-enyl)chromone	AR
Radix Glycyrrhizae (RG)	MOL004864	5,7-dihydroxy-3-(4-methoxyphenyl)-8-(3-methylbut-2-enyl)chromone	PPARG
Radix Glycyrrhizae (RG)	MOL004864	5,7-dihydroxy-3-(4-methoxyphenyl)-8-(3-methylbut-2-enyl)chromone	PTGS2

Radix Glycyrrhizae (RG)	MOL004864	5,7-dihydroxy-3-(4-methoxyphenyl)-8-(3-methylbut-2-enyl)chromone	ESR2
Radix Glycyrrhizae (RG)	MOL004864	5,7-dihydroxy-3-(4-methoxyphenyl)-8-(3-methylbut-2-enyl)chromone	DPP4
Radix Glycyrrhizae (RG)	MOL004864	5,7-dihydroxy-3-(4-methoxyphenyl)-8-(3-methylbut-2-enyl)chromone	MAPK14
Radix Glycyrrhizae (RG)	MOL004864	5,7-dihydroxy-3-(4-methoxyphenyl)-8-(3-methylbut-2-enyl)chromone	GSK3B
Radix Glycyrrhizae (RG)	MOL004864	5,7-dihydroxy-3-(4-methoxyphenyl)-8-(3-methylbut-2-enyl)chromone	HSP90AB1
Radix Glycyrrhizae (RG)	MOL004864	5,7-dihydroxy-3-(4-methoxyphenyl)-8-(3-methylbut-2-enyl)chromone	CDK2
Radix Glycyrrhizae (RG)	MOL004864	5,7-dihydroxy-3-(4-methoxyphenyl)-8-(3-methylbut-2-enyl)chromone	CHEK1
Radix Glycyrrhizae (RG)	MOL004864	5,7-dihydroxy-3-(4-methoxyphenyl)-8-(3-methylbut-2-enyl)chromone	PRSS1
Radix Glycyrrhizae (RG)	MOL004864	5,7-dihydroxy-3-(4-methoxyphenyl)-8-(3-methylbut-2-enyl)chromone	CCNA2
Radix Glycyrrhizae (RG)	MOL004864	5,7-dihydroxy-3-(4-methoxyphenyl)-8-(3-methylbut-2-enyl)chromone	NCOA2
Radix Glycyrrhizae (RG)	MOL004864	5,7-dihydroxy-3-(4-methoxyphenyl)-8-(3-methylbut-2-enyl)chromone	CAMKMT
Radix Glycyrrhizae (RG)	MOL004866	2-(3,4-dihydroxyphenyl)-5,7-dihydroxy-6-(3-methylbut-2-enyl)chromone	AR
Radix Glycyrrhizae (RG)	MOL004866	2-(3,4-dihydroxyphenyl)-5,7-dihydroxy-6-(3-methylbut-2-enyl)chromone	SCN5A
Radix Glycyrrhizae (RG)	MOL004866	2-(3,4-dihydroxyphenyl)-5,7-dihydroxy-6-(3-methylbut-2-enyl)chromone	PPARG
Radix Glycyrrhizae (RG)	MOL004866	2-(3,4-dihydroxyphenyl)-5,7-dihydroxy-6-(3-methylbut-2-enyl)chromone	PTGS2
Radix Glycyrrhizae (RG)	MOL004866	2-(3,4-dihydroxyphenyl)-5,7-dihydroxy-6-(3-methylbut-2-enyl)chromone	F7
Radix Glycyrrhizae (RG)	MOL004866	2-(3,4-dihydroxyphenyl)-5,7-dihydroxy-6-(3-methylbut-2-enyl)chromone	ADRB2
Radix Glycyrrhizae (RG)	MOL004866	2-(3,4-dihydroxyphenyl)-5,7-dihydroxy-6-(3-methylbut-2-enyl)chromone	DPP4
Radix Glycyrrhizae (RG)	MOL004866	2-(3,4-dihydroxyphenyl)-5,7-dihydroxy-6-(3-methylbut-2-enyl)chromone	HSP90AB1
Radix Glycyrrhizae (RG)	MOL004866	2-(3,4-dihydroxyphenyl)-5,7-dihydroxy-6-(3-methylbut-2-enyl)chromone	CDK2
Radix Glycyrrhizae (RG)	MOL004866	2-(3,4-dihydroxyphenyl)-5,7-dihydroxy-6-(3-methylbut-2-enyl)chromone	CHEK1

Radix Glycyrrhizae (RG)	MOL004866	2-(3,4-dihydroxyphenyl)-5,7-dihydroxy-6-(3-methylbut-2-enyl)chromone	PRSS1
Radix Glycyrrhizae (RG)	MOL004866	2-(3,4-dihydroxyphenyl)-5,7-dihydroxy-6-(3-methylbut-2-enyl)chromone	CCNA2
Radix Glycyrrhizae (RG)	MOL004866	2-(3,4-dihydroxyphenyl)-5,7-dihydroxy-6-(3-methylbut-2-enyl)chromone	CAMKMT
Radix Glycyrrhizae (RG)	MOL004879	Glycyrin	NOS2
Radix Glycyrrhizae (RG)	MOL004879	Glycyrin	KCNH2
Radix Glycyrrhizae (RG)	MOL004879	Glycyrin	ESR1
Radix Glycyrrhizae (RG)	MOL004879	Glycyrin	AR
Radix Glycyrrhizae (RG)	MOL004879	Glycyrin	PPARG
Radix Glycyrrhizae (RG)	MOL004879	Glycyrin	PTGS2
Radix Glycyrrhizae (RG)	MOL004879	Glycyrin	KDR
Radix Glycyrrhizae (RG)	MOL004879	Glycyrin	ESR2
Radix Glycyrrhizae (RG)	MOL004879	Glycyrin	DPP4
Radix Glycyrrhizae (RG)	MOL004879	Glycyrin	CHEK1
Radix Glycyrrhizae (RG)	MOL004879	Glycyrin	PRSS1
Radix Glycyrrhizae (RG)	MOL004879	Glycyrin	NCOA2
Radix Glycyrrhizae (RG)	MOL004879	Glycyrin	CAMKMT
Radix Glycyrrhizae (RG)	MOL004882	Licocoumarone	ESR1
Radix Glycyrrhizae (RG)	MOL004882	Licocoumarone	AR
Radix Glycyrrhizae (RG)	MOL004882	Licocoumarone	ESR2
Radix Glycyrrhizae (RG)	MOL004882	Licocoumarone	GSK3B
Radix Glycyrrhizae (RG)	MOL004882	Licocoumarone	HSP90AB1
Radix Glycyrrhizae (RG)	MOL004882	Licocoumarone	CDK2
Radix Glycyrrhizae (RG)	MOL004882	Licocoumarone	CCNA2
Radix Glycyrrhizae (RG)	MOL004883	Licoisoflavone	NOS2
Radix Glycyrrhizae (RG)	MOL004883	Licoisoflavone	ESR1
Radix Glycyrrhizae (RG)	MOL004883	Licoisoflavone	AR
Radix Glycyrrhizae (RG)	MOL004883	Licoisoflavone	PPARG
Radix Glycyrrhizae (RG)	MOL004883	Licoisoflavone	PTGS2
Radix Glycyrrhizae (RG)	MOL004883	Licoisoflavone	KDR
Radix Glycyrrhizae (RG)	MOL004883	Licoisoflavone	DPP4
Radix Glycyrrhizae (RG)	MOL004883	Licoisoflavone	MAPK14
Radix Glycyrrhizae (RG)	MOL004883	Licoisoflavone	HSP90AB1
Radix Glycyrrhizae (RG)	MOL004883	Licoisoflavone	CDK2
Radix Glycyrrhizae (RG)	MOL004883	Licoisoflavone	CHEK1
Radix Glycyrrhizae (RG)	MOL004883	Licoisoflavone	PRSS1
Radix Glycyrrhizae (RG)	MOL004883	Licoisoflavone	CCNA2
Radix Glycyrrhizae (RG)	MOL004883	Licoisoflavone	NCOA2
Radix Glycyrrhizae (RG)	MOL004883	Licoisoflavone	CAMKMT
Radix Glycyrrhizae (RG)	MOL004884	Licoisoflavone B	NOS2
Radix Glycyrrhizae (RG)	MOL004884	Licoisoflavone B	ESR1
Radix Glycyrrhizae (RG)	MOL004884	Licoisoflavone B	AR
Radix Glycyrrhizae (RG)	MOL004884	Licoisoflavone B	PPARG
Radix Glycyrrhizae (RG)	MOL004884	Licoisoflavone B	PTGS2
Radix Glycyrrhizae (RG)	MOL004884	Licoisoflavone B	ACHE
Radix Glycyrrhizae (RG)	MOL004884	Licoisoflavone B	ESR2
Radix Glycyrrhizae (RG)	MOL004884	Licoisoflavone B	GSK3B
Radix Glycyrrhizae (RG)	MOL004884	Licoisoflavone B	CDK2
Radix Glycyrrhizae (RG)	MOL004884	Licoisoflavone B	CHEK1
Radix Glycyrrhizae (RG)	MOL004884	Licoisoflavone B	PRSS1
Radix Glycyrrhizae (RG)	MOL004884	Licoisoflavone B	CCNA2
Radix Glycyrrhizae (RG)	MOL004884	Licoisoflavone B	CAMKMT
Radix Glycyrrhizae (RG)	MOL004885	licoisoflavanone	NOS2
Radix Glycyrrhizae (RG)	MOL004885	licoisoflavanone	PTGS1
Radix Glycyrrhizae (RG)	MOL004885	licoisoflavanone	ESR1
Radix Glycyrrhizae (RG)	MOL004885	licoisoflavanone	AR
Radix Glycyrrhizae (RG)	MOL004885	licoisoflavanone	SCN5A
Radix Glycyrrhizae (RG)	MOL004885	licoisoflavanone	PPARG

Radix Glycyrrhizae (RG)	MOL004885	licoisoflavanone	PTGS2
Radix Glycyrrhizae (RG)	MOL004885	licoisoflavanone	F7
Radix Glycyrrhizae (RG)	MOL004885	licoisoflavanone	ACHE
Radix Glycyrrhizae (RG)	MOL004885	licoisoflavanone	ESR2
Radix Glycyrrhizae (RG)	MOL004885	licoisoflavanone	GSK3B
Radix Glycyrrhizae (RG)	MOL004885	licoisoflavanone	HSP90AB1
Radix Glycyrrhizae (RG)	MOL004885	licoisoflavanone	CDK2
Radix Glycyrrhizae (RG)	MOL004885	licoisoflavanone	PRSS1
Radix Glycyrrhizae (RG)	MOL004885	licoisoflavanone	CCNA2
Radix Glycyrrhizae (RG)	MOL004885	licoisoflavanone	NCOA1
Radix Glycyrrhizae (RG)	MOL004885	licoisoflavanone	CAMKMT
Radix Glycyrrhizae (RG)	MOL004891	shinpterocarpin	NOS2
Radix Glycyrrhizae (RG)	MOL004891	shinpterocarpin	PTGS1
Radix Glycyrrhizae (RG)	MOL004891	shinpterocarpin	CHRM3
Radix Glycyrrhizae (RG)	MOL004891	shinpterocarpin	KCNH2
Radix Glycyrrhizae (RG)	MOL004891	shinpterocarpin	CHRM1
Radix Glycyrrhizae (RG)	MOL004891	shinpterocarpin	ESR1
Radix Glycyrrhizae (RG)	MOL004891	shinpterocarpin	AR
Radix Glycyrrhizae (RG)	MOL004891	shinpterocarpin	SCN5A
Radix Glycyrrhizae (RG)	MOL004891	shinpterocarpin	PPARG
Radix Glycyrrhizae (RG)	MOL004891	shinpterocarpin	PTGS2
Radix Glycyrrhizae (RG)	MOL004891	shinpterocarpin	HTR3A
Radix Glycyrrhizae (RG)	MOL004891	shinpterocarpin	RXRA
Radix Glycyrrhizae (RG)	MOL004891	shinpterocarpin	OPRD1
Radix Glycyrrhizae (RG)	MOL004891	shinpterocarpin	ADRA1B
Radix Glycyrrhizae (RG)	MOL004891	shinpterocarpin	ADRB2
Radix Glycyrrhizae (RG)	MOL004891	shinpterocarpin	ADRA1D
Radix Glycyrrhizae (RG)	MOL004891	shinpterocarpin	OPRM1
Radix Glycyrrhizae (RG)	MOL004891	shinpterocarpin	ESR2
Radix Glycyrrhizae (RG)	MOL004891	shinpterocarpin	MAPK14
Radix Glycyrrhizae (RG)	MOL004891	shinpterocarpin	GSK3B
Radix Glycyrrhizae (RG)	MOL004891	shinpterocarpin	CDK2
Radix Glycyrrhizae (RG)	MOL004891	shinpterocarpin	RXRB
Radix Glycyrrhizae (RG)	MOL004891	shinpterocarpin	PRSS1
Radix Glycyrrhizae (RG)	MOL004891	shinpterocarpin	CCNA2
Radix Glycyrrhizae (RG)	MOL004891	shinpterocarpin	NCOA1
Radix Glycyrrhizae (RG)	MOL004891	shinpterocarpin	CAMKMT
Radix Glycyrrhizae (RG)	MOL004898	(E)-3-[3,4-dihydroxy-5-(3-methylbut-2-enyl)phenyl]-1-(2,4-dihydroxyphenyl)prop-2-en-1-one	ESR1
Radix Glycyrrhizae (RG)	MOL004898	(E)-3-[3,4-dihydroxy-5-(3-methylbut-2-enyl)phenyl]-1-(2,4-dihydroxyphenyl)prop-2-en-1-one	AR
Radix Glycyrrhizae (RG)	MOL004898	(E)-3-[3,4-dihydroxy-5-(3-methylbut-2-enyl)phenyl]-1-(2,4-dihydroxyphenyl)prop-2-en-1-one	PPARG
Radix Glycyrrhizae (RG)	MOL004898	(E)-3-[3,4-dihydroxy-5-(3-methylbut-2-enyl)phenyl]-1-(2,4-dihydroxyphenyl)prop-2-en-1-one	PTGS2
Radix Glycyrrhizae (RG)	MOL004898	(E)-3-[3,4-dihydroxy-5-(3-methylbut-2-enyl)phenyl]-1-(2,4-dihydroxyphenyl)prop-2-en-1-one	MAPK14
Radix Glycyrrhizae (RG)	MOL004898	(E)-3-[3,4-dihydroxy-5-(3-methylbut-2-enyl)phenyl]-1-(2,4-dihydroxyphenyl)prop-2-en-1-one	GSK3B

Radix Glycyrrhizae (RG)	MOL004898	(E)-3-[3,4-dihydroxy-5-(3-methylbut-2-enyl)phenyl]-1-(2,4-dihydroxyphenyl)prop-2-en-1-one	HSP90AB1
Radix Glycyrrhizae (RG)	MOL004898	(E)-3-[3,4-dihydroxy-5-(3-methylbut-2-enyl)phenyl]-1-(2,4-dihydroxyphenyl)prop-2-en-1-one	CDK2
Radix Glycyrrhizae (RG)	MOL004898	(E)-3-[3,4-dihydroxy-5-(3-methylbut-2-enyl)phenyl]-1-(2,4-dihydroxyphenyl)prop-2-en-1-one	CCNA2
Radix Glycyrrhizae (RG)	MOL004898	(E)-3-[3,4-dihydroxy-5-(3-methylbut-2-enyl)phenyl]-1-(2,4-dihydroxyphenyl)prop-2-en-1-one	NCOA2
Radix Glycyrrhizae (RG)	MOL004898	(E)-3-[3,4-dihydroxy-5-(3-methylbut-2-enyl)phenyl]-1-(2,4-dihydroxyphenyl)prop-2-en-1-one	CAMKMT
Radix Glycyrrhizae (RG)	MOL004903	liquiritin	F7
Radix Glycyrrhizae (RG)	MOL004903	liquiritin	CAMKMT
Radix Glycyrrhizae (RG)	MOL004903	liquiritin	PTGS2
Radix Glycyrrhizae (RG)	MOL004903	liquiritin	KDR
Radix Glycyrrhizae (RG)	MOL004903	liquiritin	SOD1
Radix Glycyrrhizae (RG)	MOL004904	licopyranocoumarin	NOS2
Radix Glycyrrhizae (RG)	MOL004904	licopyranocoumarin	ESR1
Radix Glycyrrhizae (RG)	MOL004904	licopyranocoumarin	AR
Radix Glycyrrhizae (RG)	MOL004904	licopyranocoumarin	PPARG
Radix Glycyrrhizae (RG)	MOL004904	licopyranocoumarin	PTGS2
Radix Glycyrrhizae (RG)	MOL004904	licopyranocoumarin	F7
Radix Glycyrrhizae (RG)	MOL004904	licopyranocoumarin	KDR
Radix Glycyrrhizae (RG)	MOL004904	licopyranocoumarin	ACHE
Radix Glycyrrhizae (RG)	MOL004904	licopyranocoumarin	CDK2
Radix Glycyrrhizae (RG)	MOL004904	licopyranocoumarin	PRSS1
Radix Glycyrrhizae (RG)	MOL004904	licopyranocoumarin	CCNA2
Radix Glycyrrhizae (RG)	MOL004904	licopyranocoumarin	CAMKMT
Radix Glycyrrhizae (RG)	MOL004907	Glyzaglabrin	NOS2
Radix Glycyrrhizae (RG)	MOL004907	Glyzaglabrin	PTGS1
Radix Glycyrrhizae (RG)	MOL004907	Glyzaglabrin	ESR1
Radix Glycyrrhizae (RG)	MOL004907	Glyzaglabrin	AR
Radix Glycyrrhizae (RG)	MOL004907	Glyzaglabrin	PPARG
Radix Glycyrrhizae (RG)	MOL004907	Glyzaglabrin	PTGS2
Radix Glycyrrhizae (RG)	MOL004907	Glyzaglabrin	ESR2
Radix Glycyrrhizae (RG)	MOL004907	Glyzaglabrin	DPP4
Radix Glycyrrhizae (RG)	MOL004907	Glyzaglabrin	MAPK14
Radix Glycyrrhizae (RG)	MOL004907	Glyzaglabrin	GSK3B
Radix Glycyrrhizae (RG)	MOL004907	Glyzaglabrin	HSP90AB1
Radix Glycyrrhizae (RG)	MOL004907	Glyzaglabrin	CDK2
Radix Glycyrrhizae (RG)	MOL004907	Glyzaglabrin	CHEK1
Radix Glycyrrhizae (RG)	MOL004907	Glyzaglabrin	PRSS1
Radix Glycyrrhizae (RG)	MOL004907	Glyzaglabrin	CCNA2
Radix Glycyrrhizae (RG)	MOL004908	Glabridin	NOS2
Radix Glycyrrhizae (RG)	MOL004908	Glabridin	CHRM1
Radix Glycyrrhizae (RG)	MOL004908	Glabridin	ESR1
Radix Glycyrrhizae (RG)	MOL004908	Glabridin	AR
Radix Glycyrrhizae (RG)	MOL004908	Glabridin	SCN5A
Radix Glycyrrhizae (RG)	MOL004908	Glabridin	PPARG
Radix Glycyrrhizae (RG)	MOL004908	Glabridin	PTGS2
Radix Glycyrrhizae (RG)	MOL004908	Glabridin	RXRA
Radix Glycyrrhizae (RG)	MOL004908	Glabridin	ACHE
Radix Glycyrrhizae (RG)	MOL004908	Glabridin	ADRA1B
Radix Glycyrrhizae (RG)	MOL004908	Glabridin	ADRB2

Radix Glycyrrhizae (RG)	MOL004908	Glabridin	ESR2
Radix Glycyrrhizae (RG)	MOL004908	Glabridin	MAPK14
Radix Glycyrrhizae (RG)	MOL004908	Glabridin	GSK3B
Radix Glycyrrhizae (RG)	MOL004908	Glabridin	CDK2
Radix Glycyrrhizae (RG)	MOL004908	Glabridin	CHEK1
Radix Glycyrrhizae (RG)	MOL004908	Glabridin	RXRB
Radix Glycyrrhizae (RG)	MOL004908	Glabridin	IGHG1
Radix Glycyrrhizae (RG)	MOL004908	Glabridin	PRSS1
Radix Glycyrrhizae (RG)	MOL004908	Glabridin	CCNA2
Radix Glycyrrhizae (RG)	MOL004908	Glabridin	NCOA2
Radix Glycyrrhizae (RG)	MOL004908	Glabridin	NCOA1
Radix Glycyrrhizae (RG)	MOL004908	Glabridin	CAMKMT
Radix Glycyrrhizae (RG)	MOL004910	Glabranin	NOS2
Radix Glycyrrhizae (RG)	MOL004910	Glabranin	PTGS1
Radix Glycyrrhizae (RG)	MOL004910	Glabranin	ESR1
Radix Glycyrrhizae (RG)	MOL004910	Glabranin	SCN5A
Radix Glycyrrhizae (RG)	MOL004910	Glabranin	PTGS2
Radix Glycyrrhizae (RG)	MOL004910	Glabranin	HSP90AB1
Radix Glycyrrhizae (RG)	MOL004910	Glabranin	CAMKMT
Radix Glycyrrhizae (RG)	MOL004911	Glabrene	NOS2
Radix Glycyrrhizae (RG)	MOL004911	Glabrene	PTGS1
Radix Glycyrrhizae (RG)	MOL004911	Glabrene	ESR1
Radix Glycyrrhizae (RG)	MOL004911	Glabrene	AR
Radix Glycyrrhizae (RG)	MOL004911	Glabrene	SCN5A
Radix Glycyrrhizae (RG)	MOL004911	Glabrene	PPARG
Radix Glycyrrhizae (RG)	MOL004911	Glabrene	PTGS2
Radix Glycyrrhizae (RG)	MOL004911	Glabrene	RXRA
Radix Glycyrrhizae (RG)	MOL004911	Glabrene	ADRB2
Radix Glycyrrhizae (RG)	MOL004911	Glabrene	ESR2
Radix Glycyrrhizae (RG)	MOL004911	Glabrene	MAPK14
Radix Glycyrrhizae (RG)	MOL004911	Glabrene	GSK3B
Radix Glycyrrhizae (RG)	MOL004911	Glabrene	HSP90AB1
Radix Glycyrrhizae (RG)	MOL004911	Glabrene	CDK2
Radix Glycyrrhizae (RG)	MOL004911	Glabrene	PRSS1
Radix Glycyrrhizae (RG)	MOL004911	Glabrene	NCOA2
Radix Glycyrrhizae (RG)	MOL004911	Glabrene	CAMKMT
Radix Glycyrrhizae (RG)	MOL004912	Glabrone	NOS2
Radix Glycyrrhizae (RG)	MOL004912	Glabrone	PTGS1
Radix Glycyrrhizae (RG)	MOL004912	Glabrone	ESR1
Radix Glycyrrhizae (RG)	MOL004912	Glabrone	AR
Radix Glycyrrhizae (RG)	MOL004912	Glabrone	SCN5A
Radix Glycyrrhizae (RG)	MOL004912	Glabrone	PPARG
Radix Glycyrrhizae (RG)	MOL004912	Glabrone	PTGS2
Radix Glycyrrhizae (RG)	MOL004912	Glabrone	RXRA
Radix Glycyrrhizae (RG)	MOL004912	Glabrone	ACHE
Radix Glycyrrhizae (RG)	MOL004912	Glabrone	ESR2
Radix Glycyrrhizae (RG)	MOL004912	Glabrone	DPP4
Radix Glycyrrhizae (RG)	MOL004912	Glabrone	MAPK14
Radix Glycyrrhizae (RG)	MOL004912	Glabrone	GSK3B
Radix Glycyrrhizae (RG)	MOL004912	Glabrone	CDK2
Radix Glycyrrhizae (RG)	MOL004912	Glabrone	CHEK1
Radix Glycyrrhizae (RG)	MOL004912	Glabrone	PRSS1
Radix Glycyrrhizae (RG)	MOL004912	Glabrone	CCNA2
Radix Glycyrrhizae (RG)	MOL004912	Glabrone	CAMKMT
Radix Glycyrrhizae (RG)	MOL004913	1,3-dihydroxy-9-methoxy-6-benzofurano[3,2-c]chromenone	ESR1
Radix Glycyrrhizae (RG)	MOL004913	1,3-dihydroxy-9-methoxy-6-benzofurano[3,2-c]chromenone	PPARG
Radix Glycyrrhizae (RG)	MOL004913	1,3-dihydroxy-9-methoxy-6-benzofurano[3,2-c]chromenone	ESR2
Radix Glycyrrhizae (RG)	MOL004913	1,3-dihydroxy-9-methoxy-6-benzofurano[3,2-c]chromenone	MAPK14

Radix Glycyrrhizae (RG)	MOL004913	1,3-dihydroxy-9-methoxy-6-benzofurano[3,2-c]chromenone	GSK3B
Radix Glycyrrhizae (RG)	MOL004913	1,3-dihydroxy-9-methoxy-6-benzofurano[3,2-c]chromenone	HSP90AB1
Radix Glycyrrhizae (RG)	MOL004913	1,3-dihydroxy-9-methoxy-6-benzofurano[3,2-c]chromenone	CDK2
Radix Glycyrrhizae (RG)	MOL004913	1,3-dihydroxy-9-methoxy-6-benzofurano[3,2-c]chromenone	CHEK1
Radix Glycyrrhizae (RG)	MOL004913	1,3-dihydroxy-9-methoxy-6-benzofurano[3,2-c]chromenone	CCNA2
Radix Glycyrrhizae (RG)	MOL004914	1,3-dihydroxy-8,9-dimethoxy-6-benzofurano[3,2-	ESR1
Radix Glycyrrhizae (RG)	MOL004914	1,3-dihydroxy-8,9-dimethoxy-6-benzofurano[3,2-	AR
Radix Glycyrrhizae (RG)	MOL004914	1,3-dihydroxy-8,9-dimethoxy-6-benzofurano[3,2-	PPARG
Radix Glycyrrhizae (RG)	MOL004914	1,3-dihydroxy-8,9-dimethoxy-6-benzofurano[3,2-	MAPK14
Radix Glycyrrhizae (RG)	MOL004914	1,3-dihydroxy-8,9-dimethoxy-6-benzofurano[3,2-	GSK3B
Radix Glycyrrhizae (RG)	MOL004914	1,3-dihydroxy-8,9-dimethoxy-6-benzofurano[3,2-	HSP90AB1
Radix Glycyrrhizae (RG)	MOL004914	1,3-dihydroxy-8,9-dimethoxy-6-benzofurano[3,2-	CDK2
Radix Glycyrrhizae (RG)	MOL004914	1,3-dihydroxy-8,9-dimethoxy-6-benzofurano[3,2-	CHEK1
Radix Glycyrrhizae (RG)	MOL004915	Eurycarpin A	NOS2
Radix Glycyrrhizae (RG)	MOL004915	Eurycarpin A	ESR1
Radix Glycyrrhizae (RG)	MOL004915	Eurycarpin A	AR
Radix Glycyrrhizae (RG)	MOL004915	Eurycarpin A	SCN5A
Radix Glycyrrhizae (RG)	MOL004915	Eurycarpin A	PPARG
Radix Glycyrrhizae (RG)	MOL004915	Eurycarpin A	PTGS2
Radix Glycyrrhizae (RG)	MOL004915	Eurycarpin A	ESR2
Radix Glycyrrhizae (RG)	MOL004915	Eurycarpin A	DPP4
Radix Glycyrrhizae (RG)	MOL004915	Eurycarpin A	MAPK14
Radix Glycyrrhizae (RG)	MOL004915	Eurycarpin A	GSK3B
Radix Glycyrrhizae (RG)	MOL004915	Eurycarpin A	HSP90AB1
Radix Glycyrrhizae (RG)	MOL004915	Eurycarpin A	CDK2
Radix Glycyrrhizae (RG)	MOL004915	Eurycarpin A	CHEK1
Radix Glycyrrhizae (RG)	MOL004915	Eurycarpin A	PRSS1
Radix Glycyrrhizae (RG)	MOL004915	Eurycarpin A	CCNA2
Radix Glycyrrhizae (RG)	MOL004915	Eurycarpin A	CAMKMT
Radix Glycyrrhizae (RG)	MOL004924	(-)-Medicocarpin	PTGS2
Radix Glycyrrhizae (RG)	MOL004924	(-)-Medicocarpin	ACHE
Radix Glycyrrhizae (RG)	MOL004935	Sigmoidin-B	ESR1
Radix Glycyrrhizae (RG)	MOL004935	Sigmoidin-B	PTGS2
Radix Glycyrrhizae (RG)	MOL004935	Sigmoidin-B	KDR
Radix Glycyrrhizae (RG)	MOL004935	Sigmoidin-B	HSP90AB1
Radix Glycyrrhizae (RG)	MOL004935	Sigmoidin-B	CAMKMT
Radix Glycyrrhizae (RG)	MOL004941	(2R)-7-hydroxy-2-(4-hydroxyphenyl)chroman-4-one	PTGS1
Radix Glycyrrhizae (RG)	MOL004941	(2R)-7-hydroxy-2-(4-hydroxyphenyl)chroman-4-one	ESR1
Radix Glycyrrhizae (RG)	MOL004941	(2R)-7-hydroxy-2-(4-hydroxyphenyl)chroman-4-one	PTGS2
Radix Glycyrrhizae (RG)	MOL004941	(2R)-7-hydroxy-2-(4-hydroxyphenyl)chroman-4-one	RXRA
Radix Glycyrrhizae (RG)	MOL004941	(2R)-7-hydroxy-2-(4-hydroxyphenyl)chroman-4-one	ADRB2
Radix Glycyrrhizae (RG)	MOL004941	(2R)-7-hydroxy-2-(4-hydroxyphenyl)chroman-4-one	HSP90AB1
Radix Glycyrrhizae (RG)	MOL004941	(2R)-7-hydroxy-2-(4-hydroxyphenyl)chroman-4-one	DPEP1

Radix Glycyrrhizae (RG)	MOL004941	(2R)-7-hydroxy-2-(4-hydroxyphenyl)chroman-4-one	MAOB
Radix Glycyrrhizae (RG)	MOL004941	(2R)-7-hydroxy-2-(4-hydroxyphenyl)chroman-4-one	PKIA
Radix Glycyrrhizae (RG)	MOL004941	(2R)-7-hydroxy-2-(4-hydroxyphenyl)chroman-4-one	CAMKMT
Radix Glycyrrhizae (RG)	MOL004941	(2R)-7-hydroxy-2-(4-hydroxyphenyl)chroman-4-one	GABRA1
Radix Glycyrrhizae (RG)	MOL004941	(2R)-7-hydroxy-2-(4-hydroxyphenyl)chroman-4-one	SLC6A4
Radix Glycyrrhizae (RG)	MOL004945	(2S)-7-hydroxy-2-(4-hydroxyphenyl)-8-(3-methylbut-2-enyl)chroman-4-	NOS2
Radix Glycyrrhizae (RG)	MOL004945	(2S)-7-hydroxy-2-(4-hydroxyphenyl)-8-(3-methylbut-2-enyl)chroman-4-	PTGS1
Radix Glycyrrhizae (RG)	MOL004945	(2S)-7-hydroxy-2-(4-hydroxyphenyl)-8-(3-methylbut-2-enyl)chroman-4-	ESR1
Radix Glycyrrhizae (RG)	MOL004945	(2S)-7-hydroxy-2-(4-hydroxyphenyl)-8-(3-methylbut-2-enyl)chroman-4-	SCN5A
Radix Glycyrrhizae (RG)	MOL004945	(2S)-7-hydroxy-2-(4-hydroxyphenyl)-8-(3-methylbut-2-enyl)chroman-4-	PTGS2
Radix Glycyrrhizae (RG)	MOL004945	(2S)-7-hydroxy-2-(4-hydroxyphenyl)-8-(3-methylbut-2-enyl)chroman-4-	ADRA1B
Radix Glycyrrhizae (RG)	MOL004945	(2S)-7-hydroxy-2-(4-hydroxyphenyl)-8-(3-methylbut-2-enyl)chroman-4-	ADRB2
Radix Glycyrrhizae (RG)	MOL004945	(2S)-7-hydroxy-2-(4-hydroxyphenyl)-8-(3-methylbut-2-enyl)chroman-4-	ESR2
Radix Glycyrrhizae (RG)	MOL004945	(2S)-7-hydroxy-2-(4-hydroxyphenyl)-8-(3-methylbut-2-enyl)chroman-4-	HSP90AB1
Radix Glycyrrhizae (RG)	MOL004945	(2S)-7-hydroxy-2-(4-hydroxyphenyl)-8-(3-methylbut-2-enyl)chroman-4-	CAMKMT
Radix Glycyrrhizae (RG)	MOL004948	Isoglycyrol	NOS2
Radix Glycyrrhizae (RG)	MOL004948	Isoglycyrol	ESR1
Radix Glycyrrhizae (RG)	MOL004948	Isoglycyrol	AR
Radix Glycyrrhizae (RG)	MOL004948	Isoglycyrol	PTGS2
Radix Glycyrrhizae (RG)	MOL004948	Isoglycyrol	DPP4
Radix Glycyrrhizae (RG)	MOL004948	Isoglycyrol	GSK3B
Radix Glycyrrhizae (RG)	MOL004949	Isolicoflavonol	NOS2
Radix Glycyrrhizae (RG)	MOL004949	Isolicoflavonol	ESR1
Radix Glycyrrhizae (RG)	MOL004949	Isolicoflavonol	AR
Radix Glycyrrhizae (RG)	MOL004949	Isolicoflavonol	PPARG
Radix Glycyrrhizae (RG)	MOL004949	Isolicoflavonol	PTGS2
Radix Glycyrrhizae (RG)	MOL004949	Isolicoflavonol	GSK3B
Radix Glycyrrhizae (RG)	MOL004949	Isolicoflavonol	HSP90AB1
Radix Glycyrrhizae (RG)	MOL004949	Isolicoflavonol	CDK2
Radix Glycyrrhizae (RG)	MOL004949	Isolicoflavonol	PRSS1
Radix Glycyrrhizae (RG)	MOL004949	Isolicoflavonol	CCNA2
Radix Glycyrrhizae (RG)	MOL004949	Isolicoflavonol	NCOA2
Radix Glycyrrhizae (RG)	MOL004949	Isolicoflavonol	CAMKMT
Radix Glycyrrhizae (RG)	MOL004957	HMO	NOS2
Radix Glycyrrhizae (RG)	MOL004957	HMO	PTGS1
Radix Glycyrrhizae (RG)	MOL004957	HMO	CHRM1
Radix Glycyrrhizae (RG)	MOL004957	HMO	ESR1
Radix Glycyrrhizae (RG)	MOL004957	HMO	AR

Radix Glycyrrhizae (RG)	MOL004957	HMO	SCN5A
Radix Glycyrrhizae (RG)	MOL004957	HMO	PPARG
Radix Glycyrrhizae (RG)	MOL004957	HMO	PTGS2
Radix Glycyrrhizae (RG)	MOL004957	HMO	RXRA
Radix Glycyrrhizae (RG)	MOL004957	HMO	SLC6A3
Radix Glycyrrhizae (RG)	MOL004957	HMO	ADRB2
Radix Glycyrrhizae (RG)	MOL004957	HMO	SLC6A4
Radix Glycyrrhizae (RG)	MOL004957	HMO	ESR2
Radix Glycyrrhizae (RG)	MOL004957	HMO	DPP4
Radix Glycyrrhizae (RG)	MOL004957	HMO	MAPK14
Radix Glycyrrhizae (RG)	MOL004957	HMO	GSK3B
Radix Glycyrrhizae (RG)	MOL004957	HMO	CDK2
Radix Glycyrrhizae (RG)	MOL004957	HMO	MAOB
Radix Glycyrrhizae (RG)	MOL004957	HMO	CHEK1
Radix Glycyrrhizae (RG)	MOL004957	HMO	IGHG1
Radix Glycyrrhizae (RG)	MOL004957	HMO	PRSS1
Radix Glycyrrhizae (RG)	MOL004957	HMO	CCNA2
Radix Glycyrrhizae (RG)	MOL004957	HMO	PKIA
Radix Glycyrrhizae (RG)	MOL004957	HMO	CAMKMT
Radix Glycyrrhizae (RG)	MOL004959	1-Methoxyphaseollidin	NOS2
Radix Glycyrrhizae (RG)	MOL004959	1-Methoxyphaseollidin	PTGS1
Radix Glycyrrhizae (RG)	MOL004959	1-Methoxyphaseollidin	KCNH2
Radix Glycyrrhizae (RG)	MOL004959	1-Methoxyphaseollidin	ESR1
Radix Glycyrrhizae (RG)	MOL004959	1-Methoxyphaseollidin	AR
Radix Glycyrrhizae (RG)	MOL004959	1-Methoxyphaseollidin	SCN5A
Radix Glycyrrhizae (RG)	MOL004959	1-Methoxyphaseollidin	PPARG
Radix Glycyrrhizae (RG)	MOL004959	1-Methoxyphaseollidin	PTGS2
Radix Glycyrrhizae (RG)	MOL004959	1-Methoxyphaseollidin	KDR
Radix Glycyrrhizae (RG)	MOL004959	1-Methoxyphaseollidin	RXRA
Radix Glycyrrhizae (RG)	MOL004959	1-Methoxyphaseollidin	ADRA1B
Radix Glycyrrhizae (RG)	MOL004959	1-Methoxyphaseollidin	ADRB2
Radix Glycyrrhizae (RG)	MOL004959	1-Methoxyphaseollidin	ADRA1D
Radix Glycyrrhizae (RG)	MOL004959	1-Methoxyphaseollidin	ESR2
Radix Glycyrrhizae (RG)	MOL004959	1-Methoxyphaseollidin	MAPK14
Radix Glycyrrhizae (RG)	MOL004959	1-Methoxyphaseollidin	GSK3B
Radix Glycyrrhizae (RG)	MOL004959	1-Methoxyphaseollidin	HSP90AB1
Radix Glycyrrhizae (RG)	MOL004959	1-Methoxyphaseollidin	CDK2
Radix Glycyrrhizae (RG)	MOL004959	1-Methoxyphaseollidin	PRSS1
Radix Glycyrrhizae (RG)	MOL004959	1-Methoxyphaseollidin	CCNA2
Radix Glycyrrhizae (RG)	MOL004959	1-Methoxyphaseollidin	NCOA2
Radix Glycyrrhizae (RG)	MOL004959	1-Methoxyphaseollidin	NCOA1
Radix Glycyrrhizae (RG)	MOL004959	1-Methoxyphaseollidin	CAMKMT
Radix Glycyrrhizae (RG)	MOL004961	Quercetin der.	NOS2
Radix Glycyrrhizae (RG)	MOL004961	Quercetin der.	PTGS1
Radix Glycyrrhizae (RG)	MOL004961	Quercetin der.	ESR1
Radix Glycyrrhizae (RG)	MOL004961	Quercetin der.	AR
Radix Glycyrrhizae (RG)	MOL004961	Quercetin der.	SCN5A
Radix Glycyrrhizae (RG)	MOL004961	Quercetin der.	PPARG
Radix Glycyrrhizae (RG)	MOL004961	Quercetin der.	PTGS2
Radix Glycyrrhizae (RG)	MOL004961	Quercetin der.	ESR2
Radix Glycyrrhizae (RG)	MOL004961	Quercetin der.	DPP4
Radix Glycyrrhizae (RG)	MOL004961	Quercetin der.	MAPK14
Radix Glycyrrhizae (RG)	MOL004961	Quercetin der.	GSK3B
Radix Glycyrrhizae (RG)	MOL004961	Quercetin der.	HSP90AB1
Radix Glycyrrhizae (RG)	MOL004961	Quercetin der.	CDK2
Radix Glycyrrhizae (RG)	MOL004961	Quercetin der.	PRSS1
Radix Glycyrrhizae (RG)	MOL004961	Quercetin der.	NCOA2
Radix Glycyrrhizae (RG)	MOL004961	Quercetin der.	CAMKMT
Radix Glycyrrhizae (RG)	MOL004966	3'-Hydroxy-4'-O-Methylglabridin	NOS2
Radix Glycyrrhizae (RG)	MOL004966	3'-Hydroxy-4'-O-Methylglabridin	PTGS1

Radix Glycyrrhizae (RG)	MOL004966	3'-Hydroxy-4'-O-Methylglabridin	KCNH2
Radix Glycyrrhizae (RG)	MOL004966	3'-Hydroxy-4'-O-Methylglabridin	ESR1
Radix Glycyrrhizae (RG)	MOL004966	3'-Hydroxy-4'-O-Methylglabridin	AR
Radix Glycyrrhizae (RG)	MOL004966	3'-Hydroxy-4'-O-Methylglabridin	SCN5A
Radix Glycyrrhizae (RG)	MOL004966	3'-Hydroxy-4'-O-Methylglabridin	PPARG
Radix Glycyrrhizae (RG)	MOL004966	3'-Hydroxy-4'-O-Methylglabridin	PTGS2
Radix Glycyrrhizae (RG)	MOL004966	3'-Hydroxy-4'-O-Methylglabridin	F7
Radix Glycyrrhizae (RG)	MOL004966	3'-Hydroxy-4'-O-Methylglabridin	KDR
Radix Glycyrrhizae (RG)	MOL004966	3'-Hydroxy-4'-O-Methylglabridin	ADRA1B
Radix Glycyrrhizae (RG)	MOL004966	3'-Hydroxy-4'-O-Methylglabridin	ADRB2
Radix Glycyrrhizae (RG)	MOL004966	3'-Hydroxy-4'-O-Methylglabridin	ESR2
Radix Glycyrrhizae (RG)	MOL004966	3'-Hydroxy-4'-O-Methylglabridin	MAPK14
Radix Glycyrrhizae (RG)	MOL004966	3'-Hydroxy-4'-O-Methylglabridin	GSK3B
Radix Glycyrrhizae (RG)	MOL004966	3'-Hydroxy-4'-O-Methylglabridin	HSP90AB1
Radix Glycyrrhizae (RG)	MOL004966	3'-Hydroxy-4'-O-Methylglabridin	CDK2
Radix Glycyrrhizae (RG)	MOL004966	3'-Hydroxy-4'-O-Methylglabridin	CHEK1
Radix Glycyrrhizae (RG)	MOL004966	3'-Hydroxy-4'-O-Methylglabridin	PRSS1
Radix Glycyrrhizae (RG)	MOL004966	3'-Hydroxy-4'-O-Methylglabridin	CCNA2
Radix Glycyrrhizae (RG)	MOL004966	3'-Hydroxy-4'-O-Methylglabridin	NCOA2
Radix Glycyrrhizae (RG)	MOL004966	3'-Hydroxy-4'-O-Methylglabridin	NCOA1
Radix Glycyrrhizae (RG)	MOL004966	3'-Hydroxy-4'-O-Methylglabridin	CAMKMT
Radix Glycyrrhizae (RG)	MOL000497	licochalcone a	NOS2
Radix Glycyrrhizae (RG)	MOL000497	licochalcone a	PTGS1
Radix Glycyrrhizae (RG)	MOL000497	licochalcone a	CHRM1
Radix Glycyrrhizae (RG)	MOL000497	licochalcone a	ESR1
Radix Glycyrrhizae (RG)	MOL000497	licochalcone a	AR
Radix Glycyrrhizae (RG)	MOL000497	licochalcone a	SCN5A
Radix Glycyrrhizae (RG)	MOL000497	licochalcone a	PPARG
Radix Glycyrrhizae (RG)	MOL000497	licochalcone a	PTGS2
Radix Glycyrrhizae (RG)	MOL000497	licochalcone a	CA2
Radix Glycyrrhizae (RG)	MOL000497	licochalcone a	ADRA1B
Radix Glycyrrhizae (RG)	MOL000497	licochalcone a	SLC6A3
Radix Glycyrrhizae (RG)	MOL000497	licochalcone a	ESR2
Radix Glycyrrhizae (RG)	MOL000497	licochalcone a	MAPK14
Radix Glycyrrhizae (RG)	MOL000497	licochalcone a	GSK3B
Radix Glycyrrhizae (RG)	MOL000497	licochalcone a	HSP90AB1
Radix Glycyrrhizae (RG)	MOL000497	licochalcone a	CDK2
Radix Glycyrrhizae (RG)	MOL000497	licochalcone a	CHEK1
Radix Glycyrrhizae (RG)	MOL000497	licochalcone a	CCNA2
Radix Glycyrrhizae (RG)	MOL000497	licochalcone a	CAMKMT
Radix Glycyrrhizae (RG)	MOL000497	licochalcone a	ADRB2
Radix Glycyrrhizae (RG)	MOL000497	licochalcone a	NCOA2

Radix Glycyrrhizae (RG)	MOL000497	licochalcone a	RELA
Radix Glycyrrhizae (RG)	MOL000497	licochalcone a	STAT3
Radix Glycyrrhizae (RG)	MOL000497	licochalcone a	CCND1
Radix Glycyrrhizae (RG)	MOL000497	licochalcone a	BCL2
Radix Glycyrrhizae (RG)	MOL000497	licochalcone a	EIF6
Radix Glycyrrhizae (RG)	MOL000497	licochalcone a	MAPK1
Radix Glycyrrhizae (RG)	MOL000497	licochalcone a	RB1
Radix Glycyrrhizae (RG)	MOL000497	licochalcone a	CDK4
Radix Glycyrrhizae (RG)	MOL000497	licochalcone a	FOSL2
Radix Glycyrrhizae (RG)	MOL004974	3'-Methoxyglabridin	NOS2
Radix Glycyrrhizae (RG)	MOL004974	3'-Methoxyglabridin	PTGS1
Radix Glycyrrhizae (RG)	MOL004974	3'-Methoxyglabridin	KCNH2
Radix Glycyrrhizae (RG)	MOL004974	3'-Methoxyglabridin	ESR1
Radix Glycyrrhizae (RG)	MOL004974	3'-Methoxyglabridin	AR
Radix Glycyrrhizae (RG)	MOL004974	3'-Methoxyglabridin	SCN5A
Radix Glycyrrhizae (RG)	MOL004974	3'-Methoxyglabridin	PPARG
Radix Glycyrrhizae (RG)	MOL004974	3'-Methoxyglabridin	PTGS2
Radix Glycyrrhizae (RG)	MOL004974	3'-Methoxyglabridin	F7
Radix Glycyrrhizae (RG)	MOL004974	3'-Methoxyglabridin	RXRA
Radix Glycyrrhizae (RG)	MOL004974	3'-Methoxyglabridin	ACHE
Radix Glycyrrhizae (RG)	MOL004974	3'-Methoxyglabridin	ADRA1B
Radix Glycyrrhizae (RG)	MOL004974	3'-Methoxyglabridin	ADRB2
Radix Glycyrrhizae (RG)	MOL004974	3'-Methoxyglabridin	ESR2
Radix Glycyrrhizae (RG)	MOL004974	3'-Methoxyglabridin	MAPK14
Radix Glycyrrhizae (RG)	MOL004974	3'-Methoxyglabridin	GSK3B
Radix Glycyrrhizae (RG)	MOL004974	3'-Methoxyglabridin	HSP90AB1
Radix Glycyrrhizae (RG)	MOL004974	3'-Methoxyglabridin	CDK2
Radix Glycyrrhizae (RG)	MOL004974	3'-Methoxyglabridin	CHEK1
Radix Glycyrrhizae (RG)	MOL004974	3'-Methoxyglabridin	PRSS1
Radix Glycyrrhizae (RG)	MOL004974	3'-Methoxyglabridin	CCNA2
Radix Glycyrrhizae (RG)	MOL004974	3'-Methoxyglabridin	NCOA2
Radix Glycyrrhizae (RG)	MOL004974	3'-Methoxyglabridin	NCOA1
Radix Glycyrrhizae (RG)	MOL004974	3'-Methoxyglabridin	CAMKMT
Radix Glycyrrhizae (RG)	MOL004978	2-[(3R)-8,8-dimethyl-3,4-dihydro-2H-pyrano[6,5-f]chromen-3-yl]-5-methoxyphenol	NOS2
Radix Glycyrrhizae (RG)	MOL004978	2-[(3R)-8,8-dimethyl-3,4-dihydro-2H-pyrano[6,5-f]chromen-3-yl]-5-methoxyphenol	PTGS1
Radix Glycyrrhizae (RG)	MOL004978	2-[(3R)-8,8-dimethyl-3,4-dihydro-2H-pyrano[6,5-f]chromen-3-yl]-5-methoxyphenol	CHRM3
Radix Glycyrrhizae (RG)	MOL004978	2-[(3R)-8,8-dimethyl-3,4-dihydro-2H-pyrano[6,5-f]chromen-3-yl]-5-methoxyphenol	KCNH2
Radix Glycyrrhizae (RG)	MOL004978	2-[(3R)-8,8-dimethyl-3,4-dihydro-2H-pyrano[6,5-f]chromen-3-yl]-5-methoxyphenol	CHRM1
Radix Glycyrrhizae (RG)	MOL004978	2-[(3R)-8,8-dimethyl-3,4-dihydro-2H-pyrano[6,5-f]chromen-3-yl]-5-methoxyphenol	ESR1
Radix Glycyrrhizae (RG)	MOL004978	2-[(3R)-8,8-dimethyl-3,4-dihydro-2H-pyrano[6,5-f]chromen-3-yl]-5-methoxyphenol	AR

Radix Glycyrrhizae (RG)	MOL004978	2-[(3R)-8,8-dimethyl-3,4-dihydro-2H-pyrano[6,5-f]chromen-3-yl]-5-methoxyphenol	SCN5A
Radix Glycyrrhizae (RG)	MOL004978	2-[(3R)-8,8-dimethyl-3,4-dihydro-2H-pyrano[6,5-f]chromen-3-yl]-5-methoxyphenol	PPARG
Radix Glycyrrhizae (RG)	MOL004978	2-[(3R)-8,8-dimethyl-3,4-dihydro-2H-pyrano[6,5-f]chromen-3-yl]-5-methoxyphenol	PTGS2
Radix Glycyrrhizae (RG)	MOL004978	2-[(3R)-8,8-dimethyl-3,4-dihydro-2H-pyrano[6,5-f]chromen-3-yl]-5-methoxyphenol	RXRA
Radix Glycyrrhizae (RG)	MOL004978	2-[(3R)-8,8-dimethyl-3,4-dihydro-2H-pyrano[6,5-f]chromen-3-yl]-5-methoxyphenol	ACHE
Radix Glycyrrhizae (RG)	MOL004978	2-[(3R)-8,8-dimethyl-3,4-dihydro-2H-pyrano[6,5-f]chromen-3-yl]-5-methoxyphenol	ADRA1B
Radix Glycyrrhizae (RG)	MOL004978	2-[(3R)-8,8-dimethyl-3,4-dihydro-2H-pyrano[6,5-f]chromen-3-yl]-5-methoxyphenol	SLC6A3
Radix Glycyrrhizae (RG)	MOL004978	2-[(3R)-8,8-dimethyl-3,4-dihydro-2H-pyrano[6,5-f]chromen-3-yl]-5-methoxyphenol	ADRB2
Radix Glycyrrhizae (RG)	MOL004978	2-[(3R)-8,8-dimethyl-3,4-dihydro-2H-pyrano[6,5-f]chromen-3-yl]-5-methoxyphenol	ESR2
Radix Glycyrrhizae (RG)	MOL004978	2-[(3R)-8,8-dimethyl-3,4-dihydro-2H-pyrano[6,5-f]chromen-3-yl]-5-methoxyphenol	MAPK14
Radix Glycyrrhizae (RG)	MOL004978	2-[(3R)-8,8-dimethyl-3,4-dihydro-2H-pyrano[6,5-f]chromen-3-yl]-5-methoxyphenol	GSK3B
Radix Glycyrrhizae (RG)	MOL004978	2-[(3R)-8,8-dimethyl-3,4-dihydro-2H-pyrano[6,5-f]chromen-3-yl]-5-methoxyphenol	CDK2
Radix Glycyrrhizae (RG)	MOL004978	2-[(3R)-8,8-dimethyl-3,4-dihydro-2H-pyrano[6,5-f]chromen-3-yl]-5-methoxyphenol	CHEK1
Radix Glycyrrhizae (RG)	MOL004978	2-[(3R)-8,8-dimethyl-3,4-dihydro-2H-pyrano[6,5-f]chromen-3-yl]-5-methoxyphenol	RXRB
Radix Glycyrrhizae (RG)	MOL004978	2-[(3R)-8,8-dimethyl-3,4-dihydro-2H-pyrano[6,5-f]chromen-3-yl]-5-methoxyphenol	PRSS1

Radix Glycyrrhizae (RG)	MOL004978	2-[(3R)-8,8-dimethyl-3,4-dihydro-2H-pyrano[6,5-f]chromen-3-yl]-5-methoxyphenol	CCNA2
Radix Glycyrrhizae (RG)	MOL004978	2-[(3R)-8,8-dimethyl-3,4-dihydro-2H-pyrano[6,5-f]chromen-3-yl]-5-methoxyphenol	NCOA2
Radix Glycyrrhizae (RG)	MOL004978	2-[(3R)-8,8-dimethyl-3,4-dihydro-2H-pyrano[6,5-f]chromen-3-yl]-5-methoxyphenol	NCOA1
Radix Glycyrrhizae (RG)	MOL004978	2-[(3R)-8,8-dimethyl-3,4-dihydro-2H-pyrano[6,5-f]chromen-3-yl]-5-methoxyphenol	CAMKMT
Radix Glycyrrhizae (RG)	MOL004980	Inflacoumarin A	ESR1
Radix Glycyrrhizae (RG)	MOL004980	Inflacoumarin A	AR
Radix Glycyrrhizae (RG)	MOL004980	Inflacoumarin A	PPARG
Radix Glycyrrhizae (RG)	MOL004980	Inflacoumarin A	PTGS2
Radix Glycyrrhizae (RG)	MOL004980	Inflacoumarin A	ADRB2
Radix Glycyrrhizae (RG)	MOL004980	Inflacoumarin A	DPP4
Radix Glycyrrhizae (RG)	MOL004980	Inflacoumarin A	HSP90AB1
Radix Glycyrrhizae (RG)	MOL004980	Inflacoumarin A	PRSS1
Radix Glycyrrhizae (RG)	MOL004980	Inflacoumarin A	NCOA2
Radix Glycyrrhizae (RG)	MOL004980	Inflacoumarin A	CAMKMT
Radix Glycyrrhizae (RG)	MOL004980	Inflacoumarin A	PTGS1
Radix Glycyrrhizae (RG)	MOL004980	Inflacoumarin A	SCN5A
Radix Glycyrrhizae (RG)	MOL004985	icos-5-enoic acid	NCOA2
Radix Glycyrrhizae (RG)	MOL004988	Kanzonol F	ESR1
Radix Glycyrrhizae (RG)	MOL004988	Kanzonol F	AR
Radix Glycyrrhizae (RG)	MOL004988	Kanzonol F	PTGS2
Radix Glycyrrhizae (RG)	MOL004988	Kanzonol F	ESR2
Radix Glycyrrhizae (RG)	MOL004988	Kanzonol F	NCOA2
Radix Glycyrrhizae (RG)	MOL004988	Kanzonol F	CAMKMT
Radix Glycyrrhizae (RG)	MOL004989	6-prenylated eriodictyol	NOS2
Radix Glycyrrhizae (RG)	MOL004989	6-prenylated eriodictyol	ESR1
Radix Glycyrrhizae (RG)	MOL004989	6-prenylated eriodictyol	SCN5A
Radix Glycyrrhizae (RG)	MOL004989	6-prenylated eriodictyol	PTGS2
Radix Glycyrrhizae (RG)	MOL004989	6-prenylated eriodictyol	F7
Radix Glycyrrhizae (RG)	MOL004989	6-prenylated eriodictyol	HSP90AB1
Radix Glycyrrhizae (RG)	MOL004989	6-prenylated eriodictyol	CAMKMT
Radix Glycyrrhizae (RG)	MOL004990	7,2',4'-trihydroxy − 5-methoxy-3 − arylcoumarin	NOS2
Radix Glycyrrhizae (RG)	MOL004990	7,2',4'-trihydroxy − 5-methoxy-3 − arylcoumarin	PTGS1
Radix Glycyrrhizae (RG)	MOL004990	7,2',4'-trihydroxy − 5-methoxy-3 − arylcoumarin	ESR1
Radix Glycyrrhizae (RG)	MOL004990	7,2',4'-trihydroxy − 5-methoxy-3 − arylcoumarin	AR
Radix Glycyrrhizae (RG)	MOL004990	7,2',4'-trihydroxy − 5-methoxy-3 − arylcoumarin	PPARG
Radix Glycyrrhizae (RG)	MOL004990	7,2',4'-trihydroxy − 5-methoxy-3 − arylcoumarin	PTGS2
Radix Glycyrrhizae (RG)	MOL004990	7,2',4'-trihydroxy − 5-methoxy-3 − arylcoumarin	ESR2
Radix Glycyrrhizae (RG)	MOL004990	7,2',4'-trihydroxy − 5-methoxy-3 − arylcoumarin	DPP4
Radix Glycyrrhizae (RG)	MOL004990	7,2',4'-trihydroxy − 5-methoxy-3 − arylcoumarin	MAPK14
Radix Glycyrrhizae (RG)	MOL004990	7,2',4'-trihydroxy − 5-methoxy-3 − arylcoumarin	GSK3B

Radix Glycyrrhizae (RG)	MOL004990	7,2',4'-trihydroxy − 5-methoxy-3 − arylcoumarin	HSP90AB1
Radix Glycyrrhizae (RG)	MOL004990	7,2',4'-trihydroxy − 5-methoxy-3 − arylcoumarin	CDK2
Radix Glycyrrhizae (RG)	MOL004990	7,2',4'-trihydroxy − 5-methoxy-3 − arylcoumarin	CHEK1
Radix Glycyrrhizae (RG)	MOL004991	7-Acetoxy-2-methylisoflavone	NOS2
Radix Glycyrrhizae (RG)	MOL004991	7-Acetoxy-2-methylisoflavone	PTGS1
Radix Glycyrrhizae (RG)	MOL004991	7-Acetoxy-2-methylisoflavone	ESR1
Radix Glycyrrhizae (RG)	MOL004991	7-Acetoxy-2-methylisoflavone	AR
Radix Glycyrrhizae (RG)	MOL004991	7-Acetoxy-2-methylisoflavone	SCN5A
Radix Glycyrrhizae (RG)	MOL004991	7-Acetoxy-2-methylisoflavone	PPARG
Radix Glycyrrhizae (RG)	MOL004991	7-Acetoxy-2-methylisoflavone	PTGS2
Radix Glycyrrhizae (RG)	MOL004991	7-Acetoxy-2-methylisoflavone	RXRA
Radix Glycyrrhizae (RG)	MOL004991	7-Acetoxy-2-methylisoflavone	ACHE
Radix Glycyrrhizae (RG)	MOL004991	7-Acetoxy-2-methylisoflavone	ADRA1B
Radix Glycyrrhizae (RG)	MOL004991	7-Acetoxy-2-methylisoflavone	ADRB2
Radix Glycyrrhizae (RG)	MOL004991	7-Acetoxy-2-methylisoflavone	ADRA1D
Radix Glycyrrhizae (RG)	MOL004991	7-Acetoxy-2-methylisoflavone	GABRA1
Radix Glycyrrhizae (RG)	MOL004991	7-Acetoxy-2-methylisoflavone	DPP4
Radix Glycyrrhizae (RG)	MOL004991	7-Acetoxy-2-methylisoflavone	MAPK14
Radix Glycyrrhizae (RG)	MOL004991	7-Acetoxy-2-methylisoflavone	GSK3B
Radix Glycyrrhizae (RG)	MOL004991	7-Acetoxy-2-methylisoflavone	HSP90AB1
Radix Glycyrrhizae (RG)	MOL004991	7-Acetoxy-2-methylisoflavone	CDK2
Radix Glycyrrhizae (RG)	MOL004991	7-Acetoxy-2-methylisoflavone	CHEK1
Radix Glycyrrhizae (RG)	MOL004991	7-Acetoxy-2-methylisoflavone	PRSS1
Radix Glycyrrhizae (RG)	MOL004991	7-Acetoxy-2-methylisoflavone	NCOA2
Radix Glycyrrhizae (RG)	MOL004991	7-Acetoxy-2-methylisoflavone	CAMKMT
Radix Glycyrrhizae (RG)	MOL004993	8-prenylated eriodictyol	ESR1
Radix Glycyrrhizae (RG)	MOL004993	8-prenylated eriodictyol	SCN5A
Radix Glycyrrhizae (RG)	MOL004993	8-prenylated eriodictyol	PTGS2
Radix Glycyrrhizae (RG)	MOL004993	8-prenylated eriodictyol	F7
Radix Glycyrrhizae (RG)	MOL004993	8-prenylated eriodictyol	HSP90AB1
Radix Glycyrrhizae (RG)	MOL004993	8-prenylated eriodictyol	NCOA1
Radix Glycyrrhizae (RG)	MOL004993	8-prenylated eriodictyol	CAMKMT
Radix Glycyrrhizae (RG)	MOL004996	gadelaidic acid	NCOA2
Radix Glycyrrhizae (RG)	MOL000500	Vestitol	NOS2
Radix Glycyrrhizae (RG)	MOL000500	Vestitol	PTGS1
Radix Glycyrrhizae (RG)	MOL000500	Vestitol	CHRM1
Radix Glycyrrhizae (RG)	MOL000500	Vestitol	ESR1
Radix Glycyrrhizae (RG)	MOL000500	Vestitol	AR
Radix Glycyrrhizae (RG)	MOL000500	Vestitol	SCN5A
Radix Glycyrrhizae (RG)	MOL000500	Vestitol	PPARG
Radix Glycyrrhizae (RG)	MOL000500	Vestitol	PTGS2
Radix Glycyrrhizae (RG)	MOL000500	Vestitol	CHRM4
Radix Glycyrrhizae (RG)	MOL000500	Vestitol	RXRA
Radix Glycyrrhizae (RG)	MOL000500	Vestitol	ADRA1A
Radix Glycyrrhizae (RG)	MOL000500	Vestitol	ADRA1B
Radix Glycyrrhizae (RG)	MOL000500	Vestitol	SLC6A3
Radix Glycyrrhizae (RG)	MOL000500	Vestitol	ADRB2
Radix Glycyrrhizae (RG)	MOL000500	Vestitol	SLC6A4
Radix Glycyrrhizae (RG)	MOL000500	Vestitol	ESR2
Radix Glycyrrhizae (RG)	MOL000500	Vestitol	DPP4
Radix Glycyrrhizae (RG)	MOL000500	Vestitol	MAPK14
Radix Glycyrrhizae (RG)	MOL000500	Vestitol	GSK3B
Radix Glycyrrhizae (RG)	MOL000500	Vestitol	HSP90AB1
Radix Glycyrrhizae (RG)	MOL000500	Vestitol	CDK2
Radix Glycyrrhizae (RG)	MOL000500	Vestitol	CHEK1
Radix Glycyrrhizae (RG)	MOL000500	Vestitol	PRSS1
Radix Glycyrrhizae (RG)	MOL000500	Vestitol	CCNA2
Radix Glycyrrhizae (RG)	MOL000500	Vestitol	PKIA
Radix Glycyrrhizae (RG)	MOL000500	Vestitol	CAMKMT
Radix Glycyrrhizae (RG)	MOL005000	Gancaonin G	NOS2

Radix Glycyrrhizae (RG)	MOL005000	Gancaonin G	ESR1
Radix Glycyrrhizae (RG)	MOL005000	Gancaonin G	AR
Radix Glycyrrhizae (RG)	MOL005000	Gancaonin G	PPARG
Radix Glycyrrhizae (RG)	MOL005000	Gancaonin G	PTGS2
Radix Glycyrrhizae (RG)	MOL005000	Gancaonin G	ESR2
Radix Glycyrrhizae (RG)	MOL005000	Gancaonin G	DPP4
Radix Glycyrrhizae (RG)	MOL005000	Gancaonin G	MAPK14
Radix Glycyrrhizae (RG)	MOL005000	Gancaonin G	GSK3B
Radix Glycyrrhizae (RG)	MOL005000	Gancaonin G	HSP90AB1
Radix Glycyrrhizae (RG)	MOL005000	Gancaonin G	CHEK1
Radix Glycyrrhizae (RG)	MOL005000	Gancaonin G	PRSS1
Radix Glycyrrhizae (RG)	MOL005000	Gancaonin G	CCNA2
Radix Glycyrrhizae (RG)	MOL005000	Gancaonin G	NCOA2
Radix Glycyrrhizae (RG)	MOL005000	Gancaonin G	CAMKMT
Radix Glycyrrhizae (RG)	MOL005001	Gancaonin H	ESR1
Radix Glycyrrhizae (RG)	MOL005001	Gancaonin H	AR
Radix Glycyrrhizae (RG)	MOL005001	Gancaonin H	PTGS2
Radix Glycyrrhizae (RG)	MOL005001	Gancaonin H	KDR
Radix Glycyrrhizae (RG)	MOL005001	Gancaonin H	HSP90AB1
Radix Glycyrrhizae (RG)	MOL005001	Gancaonin H	PRSS1
Radix Glycyrrhizae (RG)	MOL005001	Gancaonin H	CCNA2
Radix Glycyrrhizae (RG)	MOL005001	Gancaonin H	NCOA2
Radix Glycyrrhizae (RG)	MOL005001	Gancaonin H	CAMKMT
Radix Glycyrrhizae (RG)	MOL005003	Licoagrocarpin	NOS2
Radix Glycyrrhizae (RG)	MOL005003	Licoagrocarpin	PTGS1
Radix Glycyrrhizae (RG)	MOL005003	Licoagrocarpin	CHRM3
Radix Glycyrrhizae (RG)	MOL005003	Licoagrocarpin	KCNH2
Radix Glycyrrhizae (RG)	MOL005003	Licoagrocarpin	CHRM1
Radix Glycyrrhizae (RG)	MOL005003	Licoagrocarpin	ESR1
Radix Glycyrrhizae (RG)	MOL005003	Licoagrocarpin	AR
Radix Glycyrrhizae (RG)	MOL005003	Licoagrocarpin	SCN5A
Radix Glycyrrhizae (RG)	MOL005003	Licoagrocarpin	PPARG
Radix Glycyrrhizae (RG)	MOL005003	Licoagrocarpin	CHRM5
Radix Glycyrrhizae (RG)	MOL005003	Licoagrocarpin	PTGS2
Radix Glycyrrhizae (RG)	MOL005003	Licoagrocarpin	RXRA
Radix Glycyrrhizae (RG)	MOL005003	Licoagrocarpin	ACHE
Radix Glycyrrhizae (RG)	MOL005003	Licoagrocarpin	ADRA1B
Radix Glycyrrhizae (RG)	MOL005003	Licoagrocarpin	ADRB2
Radix Glycyrrhizae (RG)	MOL005003	Licoagrocarpin	ESR2
Radix Glycyrrhizae (RG)	MOL005003	Licoagrocarpin	MAPK14
Radix Glycyrrhizae (RG)	MOL005003	Licoagrocarpin	GSK3B
Radix Glycyrrhizae (RG)	MOL005003	Licoagrocarpin	HSP90AB1
Radix Glycyrrhizae (RG)	MOL005003	Licoagrocarpin	CDK2
Radix Glycyrrhizae (RG)	MOL005003	Licoagrocarpin	RXRB
Radix Glycyrrhizae (RG)	MOL005003	Licoagrocarpin	PRSS1
Radix Glycyrrhizae (RG)	MOL005003	Licoagrocarpin	CCNA2
Radix Glycyrrhizae (RG)	MOL005003	Licoagrocarpin	NCOA2
Radix Glycyrrhizae (RG)	MOL005003	Licoagrocarpin	CAMKMT
Radix Glycyrrhizae (RG)	MOL005007	Glyasperins M	NOS2
Radix Glycyrrhizae (RG)	MOL005007	Glyasperins M	PTGS1
Radix Glycyrrhizae (RG)	MOL005007	Glyasperins M	KCNH2
Radix Glycyrrhizae (RG)	MOL005007	Glyasperins M	ESR1
Radix Glycyrrhizae (RG)	MOL005007	Glyasperins M	AR
Radix Glycyrrhizae (RG)	MOL005007	Glyasperins M	SCN5A
Radix Glycyrrhizae (RG)	MOL005007	Glyasperins M	PPARG
Radix Glycyrrhizae (RG)	MOL005007	Glyasperins M	PTGS2
Radix Glycyrrhizae (RG)	MOL005007	Glyasperins M	F7
Radix Glycyrrhizae (RG)	MOL005007	Glyasperins M	KDR
Radix Glycyrrhizae (RG)	MOL005007	Glyasperins M	ACHE
Radix Glycyrrhizae (RG)	MOL005007	Glyasperins M	ESR2
Radix Glycyrrhizae (RG)	MOL005007	Glyasperins M	PPARD
Radix Glycyrrhizae (RG)	MOL005007	Glyasperins M	GSK3B
Radix Glycyrrhizae (RG)	MOL005007	Glyasperins M	HSP90AB1

Radix Glycyrrhizae (RG)	MOL005007	Glyasperins M	CDK2
Radix Glycyrrhizae (RG)	MOL005007	Glyasperins M	PRSS1
Radix Glycyrrhizae (RG)	MOL005007	Glyasperins M	CCNA2
Radix Glycyrrhizae (RG)	MOL005007	Glyasperins M	NCOA2
Radix Glycyrrhizae (RG)	MOL005007	Glyasperins M	NCOA1
Radix Glycyrrhizae (RG)	MOL005007	Glyasperins M	CAMKMT
Radix Glycyrrhizae (RG)	MOL005008	Glycyrrhiza flavonol A	NOS2
Radix Glycyrrhizae (RG)	MOL005008	Glycyrrhiza flavonol A	ESR1
Radix Glycyrrhizae (RG)	MOL005008	Glycyrrhiza flavonol A	AR
Radix Glycyrrhizae (RG)	MOL005008	Glycyrrhiza flavonol A	PTGS2
Radix Glycyrrhizae (RG)	MOL005008	Glycyrrhiza flavonol A	F7
Radix Glycyrrhizae (RG)	MOL005008	Glycyrrhiza flavonol A	ACHE
Radix Glycyrrhizae (RG)	MOL005008	Glycyrrhiza flavonol A	ESR2
Radix Glycyrrhizae (RG)	MOL005008	Glycyrrhiza flavonol A	DPP4
Radix Glycyrrhizae (RG)	MOL005008	Glycyrrhiza flavonol A	GSK3B
Radix Glycyrrhizae (RG)	MOL005008	Glycyrrhiza flavonol A	HSP90AB1
Radix Glycyrrhizae (RG)	MOL005008	Glycyrrhiza flavonol A	CDK2
Radix Glycyrrhizae (RG)	MOL005008	Glycyrrhiza flavonol A	PRSS1
Radix Glycyrrhizae (RG)	MOL005008	Glycyrrhiza flavonol A	CCNA2
Radix Glycyrrhizae (RG)	MOL005008	Glycyrrhiza flavonol A	CAMKMT
Radix Glycyrrhizae (RG)	MOL005012	Licoagroisoflavone	NOS2
Radix Glycyrrhizae (RG)	MOL005012	Licoagroisoflavone	ESR1
Radix Glycyrrhizae (RG)	MOL005012	Licoagroisoflavone	AR
Radix Glycyrrhizae (RG)	MOL005012	Licoagroisoflavone	SCN5A
Radix Glycyrrhizae (RG)	MOL005012	Licoagroisoflavone	PPARG
Radix Glycyrrhizae (RG)	MOL005012	Licoagroisoflavone	PTGS2
Radix Glycyrrhizae (RG)	MOL005012	Licoagroisoflavone	ESR2
Radix Glycyrrhizae (RG)	MOL005012	Licoagroisoflavone	DPP4
Radix Glycyrrhizae (RG)	MOL005012	Licoagroisoflavone	MAPK14
Radix Glycyrrhizae (RG)	MOL005012	Licoagroisoflavone	GSK3B
Radix Glycyrrhizae (RG)	MOL005012	Licoagroisoflavone	CDK2
Radix Glycyrrhizae (RG)	MOL005012	Licoagroisoflavone	CHEK1
Radix Glycyrrhizae (RG)	MOL005012	Licoagroisoflavone	PRSS1
Radix Glycyrrhizae (RG)	MOL005012	Licoagroisoflavone	CCNA2
Radix Glycyrrhizae (RG)	MOL005012	Licoagroisoflavone	CAMKMT
Radix Glycyrrhizae (RG)	MOL005016	Odoratin	NOS2
Radix Glycyrrhizae (RG)	MOL005016	Odoratin	PTGS1
Radix Glycyrrhizae (RG)	MOL005016	Odoratin	ESR1
Radix Glycyrrhizae (RG)	MOL005016	Odoratin	AR
Radix Glycyrrhizae (RG)	MOL005016	Odoratin	SCN5A
Radix Glycyrrhizae (RG)	MOL005016	Odoratin	PPARG
Radix Glycyrrhizae (RG)	MOL005016	Odoratin	PTGS2
Radix Glycyrrhizae (RG)	MOL005016	Odoratin	RXRA
Radix Glycyrrhizae (RG)	MOL005016	Odoratin	ESR2
Radix Glycyrrhizae (RG)	MOL005016	Odoratin	DPP4
Radix Glycyrrhizae (RG)	MOL005016	Odoratin	MAPK14
Radix Glycyrrhizae (RG)	MOL005016	Odoratin	GSK3B
Radix Glycyrrhizae (RG)	MOL005016	Odoratin	HSP90AB1
Radix Glycyrrhizae (RG)	MOL005016	Odoratin	CDK2
Radix Glycyrrhizae (RG)	MOL005016	Odoratin	CHEK1
Radix Glycyrrhizae (RG)	MOL005016	Odoratin	PRSS1
Radix Glycyrrhizae (RG)	MOL005016	Odoratin	CCNA2
Radix Glycyrrhizae (RG)	MOL005016	Odoratin	NCOA2
Radix Glycyrrhizae (RG)	MOL005016	Odoratin	CAMKMT
Radix Glycyrrhizae (RG)	MOL005017	Phaseol	ESR1
Radix Glycyrrhizae (RG)	MOL005017	Phaseol	AR
Radix Glycyrrhizae (RG)	MOL005017	Phaseol	PPARG
Radix Glycyrrhizae (RG)	MOL005017	Phaseol	PTGS2
Radix Glycyrrhizae (RG)	MOL005017	Phaseol	KDR
Radix Glycyrrhizae (RG)	MOL005017	Phaseol	MAPK14
Radix Glycyrrhizae (RG)	MOL005017	Phaseol	GSK3B
Radix Glycyrrhizae (RG)	MOL005017	Phaseol	HSP90AB1
Radix Glycyrrhizae (RG)	MOL005017	Phaseol	CDK2

Radix Glycyrrhizae (RG)	MOL005017	Phaseol	CHEK1
Radix Glycyrrhizae (RG)	MOL005017	Phaseol	CCNA2
Radix Glycyrrhizae (RG)	MOL005018	Xambioona	NOS2
Radix Glycyrrhizae (RG)	MOL005018	Xambioona	ESR1
Radix Glycyrrhizae (RG)	MOL005018	Xambioona	PTGS2
Radix Glycyrrhizae (RG)	MOL005018	Xambioona	ESR2
Radix Glycyrrhizae (RG)	MOL005018	Xambioona	NCOA2
Radix Glycyrrhizae (RG)	MOL005018	Xambioona	CAMKMT
Radix Glycyrrhizae (RG)	MOL005020	dehydroglyasperins C	NOS2
Radix Glycyrrhizae (RG)	MOL005020	dehydroglyasperins C	ESR1
Radix Glycyrrhizae (RG)	MOL005020	dehydroglyasperins C	AR
Radix Glycyrrhizae (RG)	MOL005020	dehydroglyasperins C	SCN5A
Radix Glycyrrhizae (RG)	MOL005020	dehydroglyasperins C	PPARG
Radix Glycyrrhizae (RG)	MOL005020	dehydroglyasperins C	PTGS2
Radix Glycyrrhizae (RG)	MOL005020	dehydroglyasperins C	ADRB2
Radix Glycyrrhizae (RG)	MOL005020	dehydroglyasperins C	ESR2
Radix Glycyrrhizae (RG)	MOL005020	dehydroglyasperins C	MAPK14
Radix Glycyrrhizae (RG)	MOL005020	dehydroglyasperins C	HSP90AB1
Radix Glycyrrhizae (RG)	MOL005020	dehydroglyasperins C	CDK2
Radix Glycyrrhizae (RG)	MOL005020	dehydroglyasperins C	CHEK1
Radix Glycyrrhizae (RG)	MOL005020	dehydroglyasperins C	PRSS1
Radix Glycyrrhizae (RG)	MOL005020	dehydroglyasperins C	CCNA2
Radix Glycyrrhizae (RG)	MOL005020	dehydroglyasperins C	NCOA2
Radix Glycyrrhizae (RG)	MOL005020	dehydroglyasperins C	CAMKMT
Radix Glycyrrhizae (RG)	MOL000098	quercetin	PTGS1
Radix Glycyrrhizae (RG)	MOL000098	quercetin	AR
Radix Glycyrrhizae (RG)	MOL000098	quercetin	PPARG
Radix Glycyrrhizae (RG)	MOL000098	quercetin	PTGS2
Radix Glycyrrhizae (RG)	MOL000098	quercetin	HSP90AB1
Radix Glycyrrhizae (RG)	MOL000098	quercetin	NCOA2
Radix Glycyrrhizae (RG)	MOL000098	quercetin	DPP4
Radix Glycyrrhizae (RG)	MOL000098	quercetin	AKR1B1
Radix Glycyrrhizae (RG)	MOL000098	quercetin	PRSS1
Radix Glycyrrhizae (RG)	MOL000098	quercetin	KCNH2
Radix Glycyrrhizae (RG)	MOL000098	quercetin	SCN5A
Radix Glycyrrhizae (RG)	MOL000098	quercetin	ADRB2
Radix Glycyrrhizae (RG)	MOL000098	quercetin	MMP3
Radix Glycyrrhizae (RG)	MOL000098	quercetin	F7
Radix Glycyrrhizae (RG)	MOL000098	quercetin	RXRA
Radix Glycyrrhizae (RG)	MOL000098	quercetin	ACHE
Radix Glycyrrhizae (RG)	MOL000098	quercetin	GABRA1
Radix Glycyrrhizae (RG)	MOL000098	quercetin	MAOB
Radix Glycyrrhizae (RG)	MOL000098	quercetin	RELA
Radix Glycyrrhizae (RG)	MOL000098	quercetin	EGFR
Radix Glycyrrhizae (RG)	MOL000098	quercetin	AKT1
Radix Glycyrrhizae (RG)	MOL000098	quercetin	VEGFA
Radix Glycyrrhizae (RG)	MOL000098	quercetin	CCND1
Radix Glycyrrhizae (RG)	MOL000098	quercetin	BCL2
Radix Glycyrrhizae (RG)	MOL000098	quercetin	BCL2L1
Radix Glycyrrhizae (RG)	MOL000098	quercetin	FOS
Radix Glycyrrhizae (RG)	MOL000098	quercetin	CDKN1A
Radix Glycyrrhizae (RG)	MOL000098	quercetin	EIF6
Radix Glycyrrhizae (RG)	MOL000098	quercetin	BAX
Radix Glycyrrhizae (RG)	MOL000098	quercetin	CASP9
Radix Glycyrrhizae (RG)	MOL000098	quercetin	PLAU
Radix Glycyrrhizae (RG)	MOL000098	quercetin	MMP2
Radix Glycyrrhizae (RG)	MOL000098	quercetin	MMP9
Radix Glycyrrhizae (RG)	MOL000098	quercetin	MAPK1
Radix Glycyrrhizae (RG)	MOL000098	quercetin	IL10RA
Radix Glycyrrhizae (RG)	MOL000098	quercetin	EGF
Radix Glycyrrhizae (RG)	MOL000098	quercetin	RB1
Radix Glycyrrhizae (RG)	MOL000098	quercetin	TNFAIP6
Radix Glycyrrhizae (RG)	MOL000098	quercetin	JUN

Radix Glycyrrhizae (RG)	MOL000098	quercetin	IL6R
Radix Glycyrrhizae (RG)	MOL000098	quercetin	AHSA1
Radix Glycyrrhizae (RG)	MOL000098	quercetin	CASP3
Radix Glycyrrhizae (RG)	MOL000098	quercetin	TP53
Radix Glycyrrhizae (RG)	MOL000098	quercetin	ELK1
Radix Glycyrrhizae (RG)	MOL000098	quercetin	NFKBIA
Radix Glycyrrhizae (RG)	MOL000098	quercetin	POR
Radix Glycyrrhizae (RG)	MOL000098	quercetin	ODC1
Radix Glycyrrhizae (RG)	MOL000098	quercetin	CASP8
Radix Glycyrrhizae (RG)	MOL000098	quercetin	TOP1
Radix Glycyrrhizae (RG)	MOL000098	quercetin	RAF1
Radix Glycyrrhizae (RG)	MOL000098	quercetin	SOD1
Radix Glycyrrhizae (RG)	MOL000098	quercetin	PRKCA
Radix Glycyrrhizae (RG)	MOL000098	quercetin	MMP1
Radix Glycyrrhizae (RG)	MOL000098	quercetin	HIF1A
Radix Glycyrrhizae (RG)	MOL000098	quercetin	STAT1
Radix Glycyrrhizae (RG)	MOL000098	quercetin	RUNX1T1
Radix Glycyrrhizae (RG)	MOL000098	quercetin	CDK1
Radix Glycyrrhizae (RG)	MOL000098	quercetin	HSPA5
Radix Glycyrrhizae (RG)	MOL000098	quercetin	ERBB2
Radix Glycyrrhizae (RG)	MOL000098	quercetin	PPARG
Radix Glycyrrhizae (RG)	MOL000098	quercetin	ACACA
Radix Glycyrrhizae (RG)	MOL000098	quercetin	HMOX1
Radix Glycyrrhizae (RG)	MOL000098	quercetin	CYP3A4
Radix Glycyrrhizae (RG)	MOL000098	quercetin	CYP1A2
Radix Glycyrrhizae (RG)	MOL000098	quercetin	CAV1
Radix Glycyrrhizae (RG)	MOL000098	quercetin	MYC
Radix Glycyrrhizae (RG)	MOL000098	quercetin	F3
Radix Glycyrrhizae (RG)	MOL000098	quercetin	GJA1
Radix Glycyrrhizae (RG)	MOL000098	quercetin	CYP1A1
Radix Glycyrrhizae (RG)	MOL000098	quercetin	ICAM1
Radix Glycyrrhizae (RG)	MOL000098	quercetin	IL1B
Radix Glycyrrhizae (RG)	MOL000098	quercetin	CCL2
Radix Glycyrrhizae (RG)	MOL000098	quercetin	SELE
Radix Glycyrrhizae (RG)	MOL000098	quercetin	VCAM1
Radix Glycyrrhizae (RG)	MOL000098	quercetin	PTGER3
Radix Glycyrrhizae (RG)	MOL000098	quercetin	CXCL8
Radix Glycyrrhizae (RG)	MOL000098	quercetin	PRKCB
Radix Glycyrrhizae (RG)	MOL000098	quercetin	BIRC5
Radix Glycyrrhizae (RG)	MOL000098	quercetin	DUOX2
Radix Glycyrrhizae (RG)	MOL000098	quercetin	NOS3
Radix Glycyrrhizae (RG)	MOL000098	quercetin	HSPB1
Radix Glycyrrhizae (RG)	MOL000098	quercetin	SULT1E1
Radix Glycyrrhizae (RG)	MOL000098	quercetin	IL2RA
Radix Glycyrrhizae (RG)	MOL000098	quercetin	NR1I2
Radix Glycyrrhizae (RG)	MOL000098	quercetin	CYP1B1
Radix Glycyrrhizae (RG)	MOL000098	quercetin	CCNB1
Radix Glycyrrhizae (RG)	MOL000098	quercetin	PLAT
Radix Glycyrrhizae (RG)	MOL000098	quercetin	THBD
Radix Glycyrrhizae (RG)	MOL000098	quercetin	SERPINE1
Radix Glycyrrhizae (RG)	MOL000098	quercetin	COL1A1
Radix Glycyrrhizae (RG)	MOL000098	quercetin	IFNG
Radix Glycyrrhizae (RG)	MOL000098	quercetin	ALOX5
Radix Glycyrrhizae (RG)	MOL000098	quercetin	IL1A
Radix Glycyrrhizae (RG)	MOL000098	quercetin	MPO
Radix Glycyrrhizae (RG)	MOL000098	quercetin	TOP2A
Radix Glycyrrhizae (RG)	MOL000098	quercetin	NCF1
Radix Glycyrrhizae (RG)	MOL000098	quercetin	ABCG2
Radix Glycyrrhizae (RG)	MOL000098	quercetin	HAS2
Radix Glycyrrhizae (RG)	MOL000098	quercetin	GSTP1
Radix Glycyrrhizae (RG)	MOL000098	quercetin	NFE2L2
Radix Glycyrrhizae (RG)	MOL000098	quercetin	NQO1
Radix Glycyrrhizae (RG)	MOL000098	quercetin	PARP1

Radix Glycyrrhizae (RG)	MOL000098	quercetin	AHR
Radix Glycyrrhizae (RG)	MOL000098	quercetin	PSMD3
Radix Glycyrrhizae (RG)	MOL000098	quercetin	SLC2A4
Radix Glycyrrhizae (RG)	MOL000098	quercetin	COL3A1
Radix Glycyrrhizae (RG)	MOL000098	quercetin	CXCL11
Radix Glycyrrhizae (RG)	MOL000098	quercetin	CXCL2
Radix Glycyrrhizae (RG)	MOL000098	quercetin	DCAF5
Radix Glycyrrhizae (RG)	MOL000098	quercetin	NR1I3
Radix Glycyrrhizae (RG)	MOL000098	quercetin	CHEK2
Radix Glycyrrhizae (RG)	MOL000098	quercetin	INSRR
Radix Glycyrrhizae (RG)	MOL000098	quercetin	CLDN4
Radix Glycyrrhizae (RG)	MOL000098	quercetin	PPARA
Radix Glycyrrhizae (RG)	MOL000098	quercetin	PPARD
Radix Glycyrrhizae (RG)	MOL000098	quercetin	HSF1
Radix Glycyrrhizae (RG)	MOL000098	quercetin	CXCL10
Radix Glycyrrhizae (RG)	MOL000098	quercetin	CHUK
Radix Glycyrrhizae (RG)	MOL000098	quercetin	SPP1
Radix Glycyrrhizae (RG)	MOL000098	quercetin	RUNX2
Radix Glycyrrhizae (RG)	MOL000098	quercetin	RASSF1
Radix Glycyrrhizae (RG)	MOL000098	quercetin	E2F1
Radix Glycyrrhizae (RG)	MOL000098	quercetin	E2F2
Radix Glycyrrhizae (RG)	MOL000098	quercetin	ACP3
Radix Glycyrrhizae (RG)	MOL000098	quercetin	CTSD
Radix Glycyrrhizae (RG)	MOL000098	quercetin	IGFBP3
Radix Glycyrrhizae (RG)	MOL000098	quercetin	IGF2
Radix Glycyrrhizae (RG)	MOL000098	quercetin	CD40LG
Radix Glycyrrhizae (RG)	MOL000098	quercetin	IRF1
Radix Glycyrrhizae (RG)	MOL000098	quercetin	ERBB3
Radix Glycyrrhizae (RG)	MOL000098	quercetin	PON1
Radix Glycyrrhizae (RG)	MOL000098	quercetin	DIO1
Radix Glycyrrhizae (RG)	MOL000098	quercetin	PCOLCE
Radix Glycyrrhizae (RG)	MOL000098	quercetin	NPEPPS
Radix Glycyrrhizae (RG)	MOL000098	quercetin	HK2
Radix Glycyrrhizae (RG)	MOL000098	quercetin	RASA1
Radix Glycyrrhizae (RG)	MOL000098	quercetin	GSTM1
Radix Glycyrrhizae (RG)	MOL000098	quercetin	GSTM2
Fructus Chebulae (FC)	MOL001002	ellagic acid	CDK2
Fructus Chebulae (FC)	MOL001002	ellagic acid	ESR1
Fructus Chebulae (FC)	MOL001002	ellagic acid	AR
Fructus Chebulae (FC)	MOL001002	ellagic acid	PGR
Fructus Chebulae (FC)	MOL001002	ellagic acid	HSP90AB1
Fructus Chebulae (FC)	MOL001002	ellagic acid	RELA
Fructus Chebulae (FC)	MOL001002	ellagic acid	VEGFA
Fructus Chebulae (FC)	MOL001002	ellagic acid	CDKN1A
Fructus Chebulae (FC)	MOL001002	ellagic acid	MMP2
Fructus Chebulae (FC)	MOL001002	ellagic acid	MMP9
Fructus Chebulae (FC)	MOL001002	ellagic acid	NFKBIA
Fructus Chebulae (FC)	MOL001002	ellagic acid	CXCL8
Fructus Chebulae (FC)	MOL001002	ellagic acid	PRKCB
Fructus Chebulae (FC)	MOL001002	ellagic acid	GSTP1
Fructus Chebulae (FC)	MOL001002	ellagic acid	IGF2
Fructus Chebulae (FC)	MOL001002	ellagic acid	GSTM1
Fructus Chebulae (FC)	MOL001002	ellagic acid	GSTM2
Fructus Chebulae (FC)	MOL001002	ellagic acid	GSTA1
Fructus Chebulae (FC)	MOL001002	ellagic acid	GSTA2
Fructus Chebulae (FC)	MOL006376	7-Dehydrosigmasterol	PGR
Fructus Chebulae (FC)	MOL006376	7-Dehydrosigmasterol	NCOA2
Fructus Chebulae (FC)	MOL006826	chebulic acid	PTGS2
Fructus Chebulae (FC)	MOL009135	ellipticine	PTGS1
Fructus Chebulae (FC)	MOL009135	ellipticine	PTGS2
Fructus Chebulae (FC)	MOL009135	ellipticine	RXRA
Fructus Chebulae (FC)	MOL009135	ellipticine	NCOA2
Fructus Chebulae (FC)	MOL009135	ellipticine	PKIA

Fructus Chebulae (FC)	MOL009135	ellipticine	BCL2
Fructus Chebulae (FC)	MOL009135	ellipticine	BCL2L1
Fructus Chebulae (FC)	MOL009135	ellipticine	CDKN1A
Fructus Chebulae (FC)	MOL009135	ellipticine	EIF6
Fructus Chebulae (FC)	MOL009135	ellipticine	BAX
Fructus Chebulae (FC)	MOL009135	ellipticine	CASP9
Fructus Chebulae (FC)	MOL009135	ellipticine	CASP3
Fructus Chebulae (FC)	MOL009135	ellipticine	TP53
Fructus Chebulae (FC)	MOL009135	ellipticine	CASP8
Fructus Chebulae (FC)	MOL009135	ellipticine	CDK1
Fructus Chebulae (FC)	MOL009135	ellipticine	CYP1A2
Fructus Chebulae (FC)	MOL009135	ellipticine	CYP1A1
Fructus Chebulae (FC)	MOL009135	ellipticine	CCNB1
Fructus Chebulae (FC)	MOL009135	ellipticine	XIAP
Fructus Chebulae (FC)	MOL009135	ellipticine	RASGRF1
Fructus Chebulae (FC)	MOL009135	ellipticine	CDK12
Fructus Chebulae (FC)	MOL009136	Peraksine	CHRM3
Fructus Chebulae (FC)	MOL009136	Peraksine	CHRM1
Fructus Chebulae (FC)	MOL009136	Peraksine	AR
Fructus Chebulae (FC)	MOL009136	Peraksine	SCN5A
Fructus Chebulae (FC)	MOL009136	Peraksine	CHRM5
Fructus Chebulae (FC)	MOL009136	Peraksine	HTR3A
Fructus Chebulae (FC)	MOL009136	Peraksine	CHRM4
Fructus Chebulae (FC)	MOL009136	Peraksine	OPRD1
Fructus Chebulae (FC)	MOL009136	Peraksine	ACHE
Fructus Chebulae (FC)	MOL009136	Peraksine	ADRA1A
Fructus Chebulae (FC)	MOL009136	Peraksine	CHRM2
Fructus Chebulae (FC)	MOL009136	Peraksine	ADRA1B
Fructus Chebulae (FC)	MOL009136	Peraksine	ADRB2
Fructus Chebulae (FC)	MOL009136	Peraksine	ADRA1D
Fructus Chebulae (FC)	MOL009136	Peraksine	SLC6A4
Fructus Chebulae (FC)	MOL009136	Peraksine	OPRM1
Fructus Chebulae (FC)	MOL009137	(R)-(6-methoxy-4-quinolyl)-[(2R,4R,5S)-5-vinylquinuclidin-2-yl]methanol	PTGS1
Fructus Chebulae (FC)	MOL009137	(R)-(6-methoxy-4-quinolyl)-[(2R,4R,5S)-5-vinylquinuclidin-2-yl]methanol	CHRM3
Fructus Chebulae (FC)	MOL009137	(R)-(6-methoxy-4-quinolyl)-[(2R,4R,5S)-5-vinylquinuclidin-2-yl]methanol	KCNH2
Fructus Chebulae (FC)	MOL009137	(R)-(6-methoxy-4-quinolyl)-[(2R,4R,5S)-5-vinylquinuclidin-2-yl]methanol	CHRM1
Fructus Chebulae (FC)	MOL009137	(R)-(6-methoxy-4-quinolyl)-[(2R,4R,5S)-5-vinylquinuclidin-2-yl]methanol	ADRB1
Fructus Chebulae (FC)	MOL009137	(R)-(6-methoxy-4-quinolyl)-[(2R,4R,5S)-5-vinylquinuclidin-2-yl]methanol	SCN5A
Fructus Chebulae (FC)	MOL009137	(R)-(6-methoxy-4-quinolyl)-[(2R,4R,5S)-5-vinylquinuclidin-2-yl]methanol	CHRM5
Fructus Chebulae (FC)	MOL009137	(R)-(6-methoxy-4-quinolyl)-[(2R,4R,5S)-5-vinylquinuclidin-2-yl]methanol	PTGS2
Fructus Chebulae (FC)	MOL009137	(R)-(6-methoxy-4-quinolyl)-[(2R,4R,5S)-5-vinylquinuclidin-2-yl]methanol	ADRA2A
Fructus Chebulae (FC)	MOL009137	(R)-(6-methoxy-4-quinolyl)-[(2R,4R,5S)-5-vinylquinuclidin-2-yl]methanol	HTR3A

Fructus Chebulae (FC)	MOL009137	(R)-(6-methoxy-4-quinolyl)-[(2R,4R,5S)-5-vinylquinuclidin-2-yl]methanol	ADRA2C
Fructus Chebulae (FC)	MOL009137	(R)-(6-methoxy-4-quinolyl)-[(2R,4R,5S)-5-vinylquinuclidin-2-yl]methanol	DRD4
Fructus Chebulae (FC)	MOL009137	(R)-(6-methoxy-4-quinolyl)-[(2R,4R,5S)-5-vinylquinuclidin-2-yl]methanol	CHRM4
Fructus Chebulae (FC)	MOL009137	(R)-(6-methoxy-4-quinolyl)-[(2R,4R,5S)-5-vinylquinuclidin-2-yl]methanol	RXRA
Fructus Chebulae (FC)	MOL009137	(R)-(6-methoxy-4-quinolyl)-[(2R,4R,5S)-5-vinylquinuclidin-2-yl]methanol	OPRD1
Fructus Chebulae (FC)	MOL009137	(R)-(6-methoxy-4-quinolyl)-[(2R,4R,5S)-5-vinylquinuclidin-2-yl]methanol	ADRA1A
Fructus Chebulae (FC)	MOL009137	(R)-(6-methoxy-4-quinolyl)-[(2R,4R,5S)-5-vinylquinuclidin-2-yl]methanol	ADRA2B
Fructus Chebulae (FC)	MOL009137	(R)-(6-methoxy-4-quinolyl)-[(2R,4R,5S)-5-vinylquinuclidin-2-yl]methanol	ADRA1B
Fructus Chebulae (FC)	MOL009137	(R)-(6-methoxy-4-quinolyl)-[(2R,4R,5S)-5-vinylquinuclidin-2-yl]methanol	DRD3
Fructus Chebulae (FC)	MOL009137	(R)-(6-methoxy-4-quinolyl)-[(2R,4R,5S)-5-vinylquinuclidin-2-yl]methanol	SLC6A3
Fructus Chebulae (FC)	MOL009137	(R)-(6-methoxy-4-quinolyl)-[(2R,4R,5S)-5-vinylquinuclidin-2-yl]methanol	ADRB2
Fructus Chebulae (FC)	MOL009137	(R)-(6-methoxy-4-quinolyl)-[(2R,4R,5S)-5-vinylquinuclidin-2-yl]methanol	ADRA1D
Fructus Chebulae (FC)	MOL009137	(R)-(6-methoxy-4-quinolyl)-[(2R,4R,5S)-5-vinylquinuclidin-2-yl]methanol	SLC6A4
Fructus Chebulae (FC)	MOL009137	(R)-(6-methoxy-4-quinolyl)-[(2R,4R,5S)-5-vinylquinuclidin-2-yl]methanol	DRD2
Fructus Chebulae (FC)	MOL009137	(R)-(6-methoxy-4-quinolyl)-[(2R,4R,5S)-5-vinylquinuclidin-2-yl]methanol	EGFR
Fructus Chebulae (FC)	MOL009137	(R)-(6-methoxy-4-quinolyl)-[(2R,4R,5S)-5-vinylquinuclidin-2-yl]methanol	OPRM1
Fructus Chebulae (FC)	MOL009137	(R)-(6-methoxy-4-quinolyl)-[(2R,4R,5S)-5-vinylquinuclidin-2-yl]methanol	HSP90AB1
Fructus Chebulae (FC)	MOL009137	(R)-(6-methoxy-4-quinolyl)-[(2R,4R,5S)-5-vinylquinuclidin-2-yl]methanol	CAMKMT
Fructus Chebulae (FC)	MOL009149	Cheilanthifoline	PTGS1
Fructus Chebulae (FC)	MOL009149	Cheilanthifoline	DRD1
Fructus Chebulae (FC)	MOL009149	Cheilanthifoline	CHRM3
Fructus Chebulae (FC)	MOL009149	Cheilanthifoline	KCNH2
Fructus Chebulae (FC)	MOL009149	Cheilanthifoline	CHRM1
Fructus Chebulae (FC)	MOL009149	Cheilanthifoline	SCN5A
Fructus Chebulae (FC)	MOL009149	Cheilanthifoline	CHRM5
Fructus Chebulae (FC)	MOL009149	Cheilanthifoline	PTGS2
Fructus Chebulae (FC)	MOL009149	Cheilanthifoline	HTR3A

Fructus Chebulae (FC)	MOL009149	Cheilanthifoline	CHRM4
Fructus Chebulae (FC)	MOL009149	Cheilanthifoline	RXRA
Fructus Chebulae (FC)	MOL009149	Cheilanthifoline	OPRD1
Fructus Chebulae (FC)	MOL009149	Cheilanthifoline	CHRM2
Fructus Chebulae (FC)	MOL009149	Cheilanthifoline	ADRA2B
Fructus Chebulae (FC)	MOL009149	Cheilanthifoline	ADRA1B
Fructus Chebulae (FC)	MOL009149	Cheilanthifoline	DRD3
Fructus Chebulae (FC)	MOL009149	Cheilanthifoline	SLC6A3
Fructus Chebulae (FC)	MOL009149	Cheilanthifoline	ADRB2
Fructus Chebulae (FC)	MOL009149	Cheilanthifoline	ADRA1D
Fructus Chebulae (FC)	MOL009149	Cheilanthifoline	SLC6A4
Fructus Chebulae (FC)	MOL009149	Cheilanthifoline	OPRM1
Fructus Chebulae (FC)	MOL009149	Cheilanthifoline	HSP90AB1
Fructus Chebulae (FC)	MOL009149	Cheilanthifoline	CAMKMT
Fructus Chebulae (FC)	MOL009149	Cheilanthifoline	F7
Radix Aucklandiae (RA)	MOL010813	Benzo[a]carbazole	PTGS1
Radix Aucklandiae (RA)	MOL010813	Benzo[a]carbazole	PTGS2
Radix Aucklandiae (RA)	MOL010813	Benzo[a]carbazole	MAOB
Radix Aucklandiae (RA)	MOL010813	Benzo[a]carbazole	NCOA2
Radix Aucklandiae (RA)	MOL010813	Benzo[a]carbazole	PKIA
Radix Aucklandiae (RA)	MOL010828	cynaropicrin	PTGS2
Radix Aucklandiae (RA)	MOL010828	cynaropicrin	NCOA2
Radix Aucklandiae (RA)	MOL000211	Mairin	PGR
Radix Aucklandiae (RA)	MOL000359	sitosterol	PGR
Radix Aucklandiae (RA)	MOL000359	sitosterol	NCOA2
Radix Aucklandiae (RA)	MOL000359	sitosterol	NR3C2
Radix Aucklandiae (RA)	MOL000449	Stigmasterol	PGR
Radix Aucklandiae (RA)	MOL000449	Stigmasterol	NR3C2
Radix Aucklandiae (RA)	MOL000449	Stigmasterol	NCOA2
Radix Aucklandiae (RA)	MOL000449	Stigmasterol	ADH1C
Radix Aucklandiae (RA)	MOL000449	Stigmasterol	IGHG1
Radix Aucklandiae (RA)	MOL000449	Stigmasterol	RXRA
Radix Aucklandiae (RA)	MOL000449	Stigmasterol	NCOA1
Radix Aucklandiae (RA)	MOL000449	Stigmasterol	PTGS1
Radix Aucklandiae (RA)	MOL000449	Stigmasterol	PTGS2
Radix Aucklandiae (RA)	MOL000449	Stigmasterol	ADRA2A
Radix Aucklandiae (RA)	MOL000449	Stigmasterol	SLC6A2
Radix Aucklandiae (RA)	MOL000449	Stigmasterol	SLC6A3
Radix Aucklandiae (RA)	MOL000449	Stigmasterol	ADRB2
Radix Aucklandiae (RA)	MOL000449	Stigmasterol	AKR1B1
Radix Aucklandiae (RA)	MOL000449	Stigmasterol	PLAU
Radix Aucklandiae (RA)	MOL000449	Stigmasterol	LTA4H
Radix Aucklandiae (RA)	MOL000449	Stigmasterol	MAOB
Radix Aucklandiae (RA)	MOL000449	Stigmasterol	MAOA
Radix Aucklandiae (RA)	MOL000449	Stigmasterol	CTRB1
Radix Aucklandiae (RA)	MOL000449	Stigmasterol	CHRM3
Radix Aucklandiae (RA)	MOL000449	Stigmasterol	CHRM1
Radix Aucklandiae (RA)	MOL000449	Stigmasterol	ADRB1
Radix Aucklandiae (RA)	MOL000449	Stigmasterol	SCN5A
Radix Aucklandiae (RA)	MOL000449	Stigmasterol	ADRA1A
Radix Aucklandiae (RA)	MOL000449	Stigmasterol	CHRM2
Radix Aucklandiae (RA)	MOL000449	Stigmasterol	ADRA1B
Radix Aucklandiae (RA)	MOL000449	Stigmasterol	GABRA1
Semen Myristicae (SM)	MOL000358	beta-sitosterol	PGR
Semen Myristicae (SM)	MOL000358	beta-sitosterol	NCOA2
Semen Myristicae (SM)	MOL000358	beta-sitosterol	PTGS1
Semen Myristicae (SM)	MOL000358	beta-sitosterol	PTGS2
Semen Myristicae (SM)	MOL000358	beta-sitosterol	HSP90AB1
Semen Myristicae (SM)	MOL000358	beta-sitosterol	KCNH2
Semen Myristicae (SM)	MOL000358	beta-sitosterol	DRD1
Semen Myristicae (SM)	MOL000358	beta-sitosterol	CHRM3
Semen Myristicae (SM)	MOL000358	beta-sitosterol	CHRM1
Semen Myristicae (SM)	MOL000358	beta-sitosterol	SCN5A

Semen Myristicae (SM)	MOL000358	beta-sitosterol	CHRM4
Semen Myristicae (SM)	MOL000358	beta-sitosterol	ADRA1A
Semen Myristicae (SM)	MOL000358	beta-sitosterol	CHRM2
Semen Myristicae (SM)	MOL000358	beta-sitosterol	ADRA1B
Semen Myristicae (SM)	MOL000358	beta-sitosterol	ADRB2
Semen Myristicae (SM)	MOL000358	beta-sitosterol	CHRNA2
Semen Myristicae (SM)	MOL000358	beta-sitosterol	SLC6A4
Semen Myristicae (SM)	MOL000358	beta-sitosterol	OPRM1
Semen Myristicae (SM)	MOL000358	beta-sitosterol	GABRA1
Semen Myristicae (SM)	MOL000358	beta-sitosterol	BCL2
Semen Myristicae (SM)	MOL000358	beta-sitosterol	BAX
Semen Myristicae (SM)	MOL000358	beta-sitosterol	CASP9
Semen Myristicae (SM)	MOL000358	beta-sitosterol	JUN
Semen Myristicae (SM)	MOL000358	beta-sitosterol	CASP3
Semen Myristicae (SM)	MOL000358	beta-sitosterol	CASP8
Semen Myristicae (SM)	MOL000358	beta-sitosterol	PRKCA
Semen Myristicae (SM)	MOL000358	beta-sitosterol	PON1
Semen Myristicae (SM)	MOL000358	beta-sitosterol	MAP2
Semen Myristicae (SM)	MOL009243	Isoguaiacin	NOS2
Semen Myristicae (SM)	MOL009243	Isoguaiacin	PTGS1
Semen Myristicae (SM)	MOL009243	Isoguaiacin	CHRM3
Semen Myristicae (SM)	MOL009243	Isoguaiacin	CHRM1
Semen Myristicae (SM)	MOL009243	Isoguaiacin	ESR1
Semen Myristicae (SM)	MOL009243	Isoguaiacin	AR
Semen Myristicae (SM)	MOL009243	Isoguaiacin	SCN5A
Semen Myristicae (SM)	MOL009243	Isoguaiacin	PPARG
Semen Myristicae (SM)	MOL009243	Isoguaiacin	PTGS2
Semen Myristicae (SM)	MOL009243	Isoguaiacin	RXRA
Semen Myristicae (SM)	MOL009243	Isoguaiacin	OPRD1
Semen Myristicae (SM)	MOL009243	Isoguaiacin	ADRA1B
Semen Myristicae (SM)	MOL009243	Isoguaiacin	SLC6A3
Semen Myristicae (SM)	MOL009243	Isoguaiacin	ADRB2
Semen Myristicae (SM)	MOL009243	Isoguaiacin	ADRA1D
Semen Myristicae (SM)	MOL009243	Isoguaiacin	OPRM1
Semen Myristicae (SM)	MOL009243	Isoguaiacin	ESR2
Semen Myristicae (SM)	MOL009243	Isoguaiacin	MAPK14
Semen Myristicae (SM)	MOL009243	Isoguaiacin	GSK3B
Semen Myristicae (SM)	MOL009243	Isoguaiacin	HSP90AB1
Semen Myristicae (SM)	MOL009243	Isoguaiacin	CHEK1
Semen Myristicae (SM)	MOL009243	Isoguaiacin	CCNA2
Semen Myristicae (SM)	MOL009243	Isoguaiacin	NCOA2
Semen Myristicae (SM)	MOL009243	Isoguaiacin	CAMKMT
Semen Myristicae (SM)	MOL009254	galbacin	KCNH2
Semen Myristicae (SM)	MOL009254	galbacin	SCN5A
Semen Myristicae (SM)	MOL009254	galbacin	PTGS2
Semen Myristicae (SM)	MOL009254	galbacin	RXRA
Semen Myristicae (SM)	MOL009254	galbacin	ADRB2
Semen Myristicae (SM)	MOL009254	galbacin	HSP90AB1
Semen Myristicae (SM)	MOL009255	5-[(2S,3S)-7-methoxy-3-methyl-5-[(E)-prop-1-enyl]-2,3-dihydrobenzofuran-2-yl]-1.3-benzodioxole	PTGS1
Semen Myristicae (SM)	MOL009255	5-[(2S,3S)-7-methoxy-3-methyl-5-[(E)-prop-1-enyl]-2,3-dihydrobenzofuran-2-yl]-1.3-benzodioxole	CHRM3
Semen Myristicae (SM)	MOL009255	5-[(2S,3S)-7-methoxy-3-methyl-5-[(E)-prop-1-enyl]-2,3-dihydrobenzofuran-2-yl]-1.3-benzodioxole	CHRM1

Semen Myristicae (SM)	MOL009255	5-[(2S,3S)-7-methoxy-3-methyl-5-[(E)-prop-1-enyl]-2,3-dihydrobenzofuran-2-yl]-1.3-benzodioxole	ADRB1
Semen Myristicae (SM)	MOL009255	5-[(2S,3S)-7-methoxy-3-methyl-5-[(E)-prop-1-enyl]-2,3-dihydrobenzofuran-2-yl]-1.3-benzodioxole	SCN5A
Semen Myristicae (SM)	MOL009255	5-[(2S,3S)-7-methoxy-3-methyl-5-[(E)-prop-1-enyl]-2,3-dihydrobenzofuran-2-yl]-1.3-benzodioxole	PTGS2
Semen Myristicae (SM)	MOL009255	5-[(2S,3S)-7-methoxy-3-methyl-5-[(E)-prop-1-enyl]-2,3-dihydrobenzofuran-2-yl]-1.3-benzodioxole	ADRA2A
Semen Myristicae (SM)	MOL009255	5-[(2S,3S)-7-methoxy-3-methyl-5-[(E)-prop-1-enyl]-2,3-dihydrobenzofuran-2-yl]-1.3-benzodioxole	ADRA2C
Semen Myristicae (SM)	MOL009255	5-[(2S,3S)-7-methoxy-3-methyl-5-[(E)-prop-1-enyl]-2,3-dihydrobenzofuran-2-yl]-1.3-benzodioxole	SLC6A2
Semen Myristicae (SM)	MOL009255	5-[(2S,3S)-7-methoxy-3-methyl-5-[(E)-prop-1-enyl]-2,3-dihydrobenzofuran-2-yl]-1.3-benzodioxole	ADRA1A
Semen Myristicae (SM)	MOL009255	5-[(2S,3S)-7-methoxy-3-methyl-5-[(E)-prop-1-enyl]-2,3-dihydrobenzofuran-2-yl]-1.3-benzodioxole	CHRM2
Semen Myristicae (SM)	MOL009255	5-[(2S,3S)-7-methoxy-3-methyl-5-[(E)-prop-1-enyl]-2,3-dihydrobenzofuran-2-yl]-1.3-benzodioxole	ADRA1B
Semen Myristicae (SM)	MOL009255	5-[(2S,3S)-7-methoxy-3-methyl-5-[(E)-prop-1-enyl]-2,3-dihydrobenzofuran-2-yl]-1.3-benzodioxole	SLC6A3
Semen Myristicae (SM)	MOL009255	5-[(2S,3S)-7-methoxy-3-methyl-5-[(E)-prop-1-enyl]-2,3-dihydrobenzofuran-2-yl]-1.3-benzodioxole	ADRB2
Semen Myristicae (SM)	MOL009255	5-[(2S,3S)-7-methoxy-3-methyl-5-[(E)-prop-1-enyl]-2,3-dihydrobenzofuran-2-yl]-1.3-benzodioxole	GABRA1
Semen Myristicae (SM)	MOL009255	5-[(2S,3S)-7-methoxy-3-methyl-5-[(E)-prop-1-enyl]-2,3-dihydrobenzofuran-2-yl]-1.3-benzodioxole	DPEP1
Semen Myristicae (SM)	MOL009255	5-[(2S,3S)-7-methoxy-3-methyl-5-[(E)-prop-1-enyl]-2,3-dihydrobenzofuran-2-yl]-1.3-benzodioxole	LTA4H
Semen Myristicae (SM)	MOL009255	5-[(2S,3S)-7-methoxy-3-methyl-5-[(E)-prop-1-enyl]-2,3-dihydrobenzofuran-2-yl]-1.3-benzodioxole	MAOB

Semen Myristicae (SM)	MOL009255	5-[(2S,3S)-7-methoxy-3-methyl-5-[(E)-prop-1-enyl]-2,3-dihydrobenzofuran-2-yl]-1.3-benzodioxole	PRSS1
Semen Myristicae (SM)	MOL009255	5-[(2S,3S)-7-methoxy-3-methyl-5-[(E)-prop-1-enyl]-2,3-dihydrobenzofuran-2-yl]-1.3-benzodioxole	KCNH2
Semen Myristicae (SM)	MOL009255	5-[(2S,3S)-7-methoxy-3-methyl-5-[(E)-prop-1-enyl]-2,3-dihydrobenzofuran-2-yl]-1.3-benzodioxole	ESR1
Semen Myristicae (SM)	MOL009255	5-[(2S,3S)-7-methoxy-3-methyl-5-[(E)-prop-1-enyl]-2,3-dihydrobenzofuran-2-yl]-1.3-benzodioxole	RXRA
Semen Myristicae (SM)	MOL009255	5-[(2S,3S)-7-methoxy-3-methyl-5-[(E)-prop-1-enyl]-2,3-dihydrobenzofuran-2-yl]-1.3-benzodioxole	ACHE
Semen Myristicae (SM)	MOL009255	5-[(2S,3S)-7-methoxy-3-methyl-5-[(E)-prop-1-enyl]-2,3-dihydrobenzofuran-2-yl]-1.3-benzodioxole	ADRA1D
Semen Myristicae (SM)	MOL009255	5-[(2S,3S)-7-methoxy-3-methyl-5-[(E)-prop-1-enyl]-2,3-dihydrobenzofuran-2-yl]-1.3-benzodioxole	HSP90AB1
Semen Myristicae (SM)	MOL009255	5-[(2S,3S)-7-methoxy-3-methyl-5-[(E)-prop-1-enyl]-2,3-dihydrobenzofuran-2-yl]-1.3-benzodioxole	RXRB
Semen Myristicae (SM)	MOL009255	5-[(2S,3S)-7-methoxy-3-methyl-5-[(E)-prop-1-enyl]-2,3-dihydrobenzofuran-2-yl]-1.3-benzodioxole	CAMKMT
Semen Myristicae (SM)	MOL009259	Kudos	SCN5A
Semen Myristicae (SM)	MOL009259	Kudos	PTGS2
Semen Myristicae (SM)	MOL009259	Kudos	DPP4
Semen Myristicae (SM)	MOL009259	Kudos	CAMKMT
Semen Myristicae (SM)	MOL009263	saucernetindiol	PTGS1
Semen Myristicae (SM)	MOL009263	saucernetindiol	KCNH2
Semen Myristicae (SM)	MOL009263	saucernetindiol	CHRM1
Semen Myristicae (SM)	MOL009263	saucernetindiol	ESR1
Semen Myristicae (SM)	MOL009263	saucernetindiol	SCN5A
Semen Myristicae (SM)	MOL009263	saucernetindiol	PTGS2
Semen Myristicae (SM)	MOL009263	saucernetindiol	RXRA
Semen Myristicae (SM)	MOL009263	saucernetindiol	ADRA1B
Semen Myristicae (SM)	MOL009263	saucernetindiol	SLC6A3
Semen Myristicae (SM)	MOL009263	saucernetindiol	ADRB2
Semen Myristicae (SM)	MOL009263	saucernetindiol	HSP90AB1
Semen Myristicae (SM)	MOL009263	saucernetindiol	NCOA2
Semen Myristicae (SM)	MOL009263	saucernetindiol	CAMKMT
Semen Myristicae (SM)	MOL009264	tetrahydrofuroguaiacin B	CHRM1
Semen Myristicae (SM)	MOL009264	tetrahydrofuroguaiacin B	ESR1
Semen Myristicae (SM)	MOL009264	tetrahydrofuroguaiacin B	SCN5A
Semen Myristicae (SM)	MOL009264	tetrahydrofuroguaiacin B	PTGS2
Semen Myristicae (SM)	MOL009264	tetrahydrofuroguaiacin B	ADRA1B
Semen Myristicae (SM)	MOL009264	tetrahydrofuroguaiacin B	ADRB2
Semen Myristicae (SM)	MOL009264	tetrahydrofuroguaiacin B	HSP90AB1
Semen Myristicae (SM)	MOL009264	tetrahydrofuroguaiacin B	NCOA2
Semen Myristicae (SM)	MOL009264	tetrahydrofuroguaiacin B	CAMKMT
Semen Myristicae (SM)	MOL009265	threo-austrobailignan-5	NOS2

Semen Myristicae (SM)	MOL009265	threo-austrobailignan-5	PTGS1
Semen Myristicae (SM)	MOL009265	threo-austrobailignan-5	CHRM1
Semen Myristicae (SM)	MOL009265	threo-austrobailignan-5	ESR1
Semen Myristicae (SM)	MOL009265	threo-austrobailignan-5	AR
Semen Myristicae (SM)	MOL009265	threo-austrobailignan-5	SCN5A
Semen Myristicae (SM)	MOL009265	threo-austrobailignan-5	PPARG
Semen Myristicae (SM)	MOL009265	threo-austrobailignan-5	PTGS2
Semen Myristicae (SM)	MOL009265	threo-austrobailignan-5	RXRA
Semen Myristicae (SM)	MOL009265	threo-austrobailignan-5	ADRA1B
Semen Myristicae (SM)	MOL009265	threo-austrobailignan-5	ADRB2
Semen Myristicae (SM)	MOL009265	threo-austrobailignan-5	ESR2
Semen Myristicae (SM)	MOL009265	threo-austrobailignan-5	MAPK14
Semen Myristicae (SM)	MOL009265	threo-austrobailignan-5	GSK3B
Semen Myristicae (SM)	MOL009265	threo-austrobailignan-5	HSP90AB1
Semen Myristicae (SM)	MOL009265	threo-austrobailignan-5	CHEK1
Semen Myristicae (SM)	MOL009265	threo-austrobailignan-5	IGHG1
Semen Myristicae (SM)	MOL009265	threo-austrobailignan-5	CCNA2
Semen Myristicae (SM)	MOL009265	threo-austrobailignan-5	NCOA2
Semen Myristicae (SM)	MOL009265	threo-austrobailignan-5	CAMKMT
Cortex Cinnamomi (CC)	MOL000131	EIC	PTGS1
Cortex Cinnamomi (CC)	MOL000131	EIC	PTGS2
Cortex Cinnamomi (CC)	MOL000131	EIC	RXRA
Cortex Cinnamomi (CC)	MOL000131	EIC	NCOA2
Cortex Cinnamomi (CC)	MOL000131	EIC	LYG1
Cortex Cinnamomi (CC)	MOL000131	EIC	SLC6A2
Cortex Cinnamomi (CC)	MOL000131	EIC	IGHG1
Cortex Cinnamomi (CC)	MOL000131	EIC	GABRA1
Cortex Cinnamomi (CC)	MOL000131	EIC	TRPV1
Cortex Cinnamomi (CC)	MOL000131	EIC	CHRM1
Cortex Cinnamomi (CC)	MOL000131	EIC	CHRM2
Cortex Cinnamomi (CC)	MOL000208	()-Aromadendrene	CHRM3
Cortex Cinnamomi (CC)	MOL000208	()-Aromadendrene	CHRM2
Cortex Cinnamomi (CC)	MOL000208	()-Aromadendrene	CHRM1
Cortex Cinnamomi (CC)	MOL000208	()-Aromadendrene	ADH1C
Cortex Cinnamomi (CC)	MOL000208	()-Aromadendrene	LYG1
Cortex Cinnamomi (CC)	MOL000266	beta-Cubebene	CHRM1
Cortex Cinnamomi (CC)	MOL000266	beta-Cubebene	CHRM2
Cortex Cinnamomi (CC)	MOL000266	beta-Cubebene	GABRA1
Cortex Cinnamomi (CC)	MOL000266	beta-Cubebene	PTGS2
Cortex Cinnamomi (CC)	MOL000266	beta-Cubebene	CHRNA2
Cortex Cinnamomi (CC)	MOL000266	beta-Cubebene	SLC6A2
Cortex Cinnamomi (CC)	MOL002697	junipene	CHRM3
Cortex Cinnamomi (CC)	MOL002697	junipene	CHRM2
Cortex Cinnamomi (CC)	MOL002697	junipene	GABRA1
Cortex Cinnamomi (CC)	MOL002697	junipene	ADRA1B
Cortex Cinnamomi (CC)	MOL002697	junipene	CHRNA2
Cortex Cinnamomi (CC)	MOL003522	()-Sativene	CHRM3
Cortex Cinnamomi (CC)	MOL003522	()-Sativene	CHRM1
Cortex Cinnamomi (CC)	MOL003522	()-Sativene	PTGS2
Cortex Cinnamomi (CC)	MOL003522	()-Sativene	CHRM2
Cortex Cinnamomi (CC)	MOL003522	()-Sativene	ADRA1B
Cortex Cinnamomi (CC)	MOL003522	()-Sativene	CHRNA2
Cortex Cinnamomi (CC)	MOL003522	()-Sativene	GABRA1
Cortex Cinnamomi (CC)	MOL003522	()-Sativene	NCOA2
Cortex Cinnamomi (CC)	MOL003538	()-Ledene	NCOA2
Cortex Cinnamomi (CC)	MOL003538	()-Ledene	CHRM3
Cortex Cinnamomi (CC)	MOL003538	()-Ledene	CHRM1
Cortex Cinnamomi (CC)	MOL002003	(-)-Caryophyllene oxide	CHRM3
Cortex Cinnamomi (CC)	MOL002003	(-)-Caryophyllene oxide	CHRM1
Cortex Cinnamomi (CC)	MOL002003	(-)-Caryophyllene oxide	PTGS2
Cortex Cinnamomi (CC)	MOL002003	(-)-Caryophyllene oxide	ACHE
Cortex Cinnamomi (CC)	MOL002003	(-)-Caryophyllene oxide	CHRM2
Cortex Cinnamomi (CC)	MOL002003	(-)-Caryophyllene oxide	ADRA1B

Cortex Cinnamomi (CC)	MOL002003	(-)-Caryophyllene oxide	GABRA1
Cortex Cinnamomi (CC)	MOL002003	(-)-Caryophyllene oxide	DPP4
Cortex Cinnamomi (CC)	MOL000057	DIBP	CHRM3
Cortex Cinnamomi (CC)	MOL000057	DIBP	CHRM1
Cortex Cinnamomi (CC)	MOL000057	DIBP	SLC6A2
Cortex Cinnamomi (CC)	MOL000057	DIBP	SLC6A3
Cortex Cinnamomi (CC)	MOL000057	DIBP	ADRB2
Cortex Cinnamomi (CC)	MOL000057	DIBP	SLC6A4
Cortex Cinnamomi (CC)	MOL000057	DIBP	GABRA1
Cortex Cinnamomi (CC)	MOL000057	DIBP	CHRM2
Cortex Cinnamomi (CC)	MOL000057	DIBP	PGR
Cortex Cinnamomi (CC)	MOL000057	DIBP	NR3C2
Cortex Cinnamomi (CC)	MOL000057	DIBP	NR3C1
Cortex Cinnamomi (CC)	MOL000057	DIBP	NCOA2
Cortex Cinnamomi (CC)	MOL000057	DIBP	RXRA
Cortex Cinnamomi (CC)	MOL000612	(-)-alpha-cedrene	CHRM3
Cortex Cinnamomi (CC)	MOL000612	(-)-alpha-cedrene	PTGS2
Cortex Cinnamomi (CC)	MOL000612	(-)-alpha-cedrene	RXRA
Cortex Cinnamomi (CC)	MOL000612	(-)-alpha-cedrene	GABRA1
Cortex Cinnamomi (CC)	MOL000612	(-)-alpha-cedrene	NCOA2
Cortex Cinnamomi (CC)	MOL000612	(-)-alpha-cedrene	CHRM1
Cortex Cinnamomi (CC)	MOL000612	(-)-alpha-cedrene	ADH1B
Cortex Cinnamomi (CC)	MOL000612	(-)-alpha-cedrene	ADH1C
Cortex Cinnamomi (CC)	MOL000675	oleic acid	PTGS1
Cortex Cinnamomi (CC)	MOL000675	oleic acid	NCOA2
Cortex Cinnamomi (CC)	MOL000675	oleic acid	PTGS2
Cortex Cinnamomi (CC)	MOL000675	oleic acid	ADH1B
Cortex Cinnamomi (CC)	MOL000675	oleic acid	ADH1C
Cortex Cinnamomi (CC)	MOL000675	oleic acid	ADH1A
Cortex Cinnamomi (CC)	MOL000675	oleic acid	LYG1
Cortex Cinnamomi (CC)	MOL000675	oleic acid	PRSS3
Cortex Cinnamomi (CC)	MOL000675	oleic acid	RXRA
Cortex Cinnamomi (CC)	MOL000675	oleic acid	PLAU
Cortex Cinnamomi (CC)	MOL000675	oleic acid	SOD1
Cortex Cinnamomi (CC)	MOL000675	oleic acid	CAT
Cortex Cinnamomi (CC)	MOL000675	oleic acid	TEP1
Cortex Cinnamomi (CC)	MOL000675	oleic acid	EDN1
Cortex Cinnamomi (CC)	MOL000675	oleic acid	ERBB2
Cortex Cinnamomi (CC)	MOL000675	oleic acid	PPARG
Cortex Cinnamomi (CC)	MOL000675	oleic acid	LPL
Cortex Cinnamomi (CC)	MOL000675	oleic acid	GAP43
Cortex Cinnamomi (CC)	MOL000675	oleic acid	SERPINE1
Cortex Cinnamomi (CC)	MOL000675	oleic acid	BDNF
Cortex Cinnamomi (CC)	MOL000675	oleic acid	HMGCR
Cortex Cinnamomi (CC)	MOL000675	oleic acid	MPO
Cortex Cinnamomi (CC)	MOL000675	oleic acid	PPARA
Cortex Cinnamomi (CC)	MOL000675	oleic acid	PPARD
Cortex Cinnamomi (CC)	MOL000675	oleic acid	PON1
Cortex Cinnamomi (CC)	MOL000675	oleic acid	INS
Cortex Cinnamomi (CC)	MOL000675	oleic acid	SERPINB2
Cortex Cinnamomi (CC)	MOL000675	oleic acid	FABP1
Cortex Cinnamomi (CC)	MOL000675	oleic acid	RBP2
Cortex Cinnamomi (CC)	MOL000675	oleic acid	GCG
Cortex Cinnamomi (CC)	MOL000675	oleic acid	ENPEP
Cortex Cinnamomi (CC)	MOL000675	oleic acid	UCP2
Cortex Cinnamomi (CC)	MOL000675	oleic acid	SOAT1
Cortex Cinnamomi (CC)	MOL000675	oleic acid	CCKAR
Cortex Cinnamomi (CC)	MOL000675	oleic acid	CITED1
Cortex Cinnamomi (CC)	MOL000675	oleic acid	NTRK2
Cortex Cinnamomi (CC)	MOL000675	oleic acid	PDX1
Cortex Cinnamomi (CC)	MOL000675	oleic acid	SLC2A2
Cortex Cinnamomi (CC)	MOL000675	oleic acid	PAM
Cortex Cinnamomi (CC)	MOL000675	oleic acid	SCD

Cortex Cinnamomi (CC)	MOL000675	oleic acid	UCP3
Cortex Cinnamomi (CC)	MOL000675	oleic acid	CETP
Cortex Cinnamomi (CC)	MOL000675	oleic acid	PYY
Cortex Cinnamomi (CC)	MOL000675	oleic acid	DNPEP
Pericarpium Papaveris (PP)	MOL006980	papaverine	PTGS1
Pericarpium Papaveris (PP)	MOL006980	papaverine	CHRM3
Pericarpium Papaveris (PP)	MOL006980	papaverine	KCNH2
Pericarpium Papaveris (PP)	MOL006980	papaverine	SCN5A
Pericarpium Papaveris (PP)	MOL006980	papaverine	CHRM5
Pericarpium Papaveris (PP)	MOL006980	papaverine	PTGS2
Pericarpium Papaveris (PP)	MOL006980	papaverine	RXRA
Pericarpium Papaveris (PP)	MOL006980	papaverine	ADRA1B
Pericarpium Papaveris (PP)	MOL006980	papaverine	ADRB2
Pericarpium Papaveris (PP)	MOL006980	papaverine	SLC6A4
Pericarpium Papaveris (PP)	MOL006980	papaverine	HSP90AB1
Pericarpium Papaveris (PP)	MOL006980	papaverine	RXRB
Pericarpium Papaveris (PP)	MOL006980	papaverine	NCOA2
Pericarpium Papaveris (PP)	MOL006980	papaverine	PDE10A
Pericarpium Papaveris (PP)	MOL006980	papaverine	CAMKMT
Pericarpium Papaveris (PP)	MOL006980	papaverine	TDP1
Pericarpium Papaveris (PP)	MOL006980	papaverine	LPL
Pericarpium Papaveris (PP)	MOL006980	papaverine	MAOA
Pericarpium Papaveris (PP)	MOL006980	papaverine	ADM
Pericarpium Papaveris (PP)	MOL006982	codeine	DRD1
Pericarpium Papaveris (PP)	MOL006982	codeine	CHRM3
Pericarpium Papaveris (PP)	MOL006982	codeine	CHRM1
Pericarpium Papaveris (PP)	MOL006982	codeine	AR
Pericarpium Papaveris (PP)	MOL006982	codeine	DRD5
Pericarpium Papaveris (PP)	MOL006982	codeine	SCN5A
Pericarpium Papaveris (PP)	MOL006982	codeine	CHRM5
Pericarpium Papaveris (PP)	MOL006982	codeine	HTR3A
Pericarpium Papaveris (PP)	MOL006982	codeine	CHRM4
Pericarpium Papaveris (PP)	MOL006982	codeine	OPRD1
Pericarpium Papaveris (PP)	MOL006982	codeine	ACHE
Pericarpium Papaveris (PP)	MOL006982	codeine	HRH1
Pericarpium Papaveris (PP)	MOL006982	codeine	ADRA1A
Pericarpium Papaveris (PP)	MOL006982	codeine	CHRM2
Pericarpium Papaveris (PP)	MOL006982	codeine	ADRA1B
Pericarpium Papaveris (PP)	MOL006982	codeine	OPRK1
Pericarpium Papaveris (PP)	MOL006982	codeine	ADRB2
Pericarpium Papaveris (PP)	MOL006982	codeine	ADRA1D
Pericarpium Papaveris (PP)	MOL006982	codeine	CHRNA2
Pericarpium Papaveris (PP)	MOL006982	codeine	SLC6A4
Pericarpium Papaveris (PP)	MOL006982	codeine	DRD2
Pericarpium Papaveris (PP)	MOL006982	codeine	OPRM1
Pericarpium Papaveris (PP)	MOL000787	Fumarine	PTGS1
Pericarpium Papaveris (PP)	MOL000787	Fumarine	CHRM3
Pericarpium Papaveris (PP)	MOL000787	Fumarine	KCNH2
Pericarpium Papaveris (PP)	MOL000787	Fumarine	CHRM1
Pericarpium Papaveris (PP)	MOL000787	Fumarine	SCN5A
Pericarpium Papaveris (PP)	MOL000787	Fumarine	CHRM5
Pericarpium Papaveris (PP)	MOL000787	Fumarine	PTGS2
Pericarpium Papaveris (PP)	MOL000787	Fumarine	HTR3A
Pericarpium Papaveris (PP)	MOL000787	Fumarine	F7
Pericarpium Papaveris (PP)	MOL000787	Fumarine	CHRM4
Pericarpium Papaveris (PP)	MOL000787	Fumarine	OPRD1
Pericarpium Papaveris (PP)	MOL000787	Fumarine	ADRA1B
Pericarpium Papaveris (PP)	MOL000787	Fumarine	ADRB2
Pericarpium Papaveris (PP)	MOL000787	Fumarine	ADRA1D
Pericarpium Papaveris (PP)	MOL000787	Fumarine	OPRM1
Pericarpium Papaveris (PP)	MOL000787	Fumarine	HSP90AB1
Pericarpium Papaveris (PP)	MOL000787	Fumarine	CAMKMT
Pericarpium Papaveris (PP)	MOL000787	Fumarine	SLC6A4

Pericarpium Papaveris (PP)	MOL000787	Fumarine	CACNA1S
Pericarpium Papaveris (PP)	MOL000787	Fumarine	SLC6A3
Pericarpium Papaveris (PP)	MOL000787	Fumarine	DRD1
Pericarpium Papaveris (PP)	MOL000787	Fumarine	KDR
Pericarpium Papaveris (PP)	MOL009324	Cryptogenin	NR3C2
Pericarpium Papaveris (PP)	MOL009327	Noskapin	KCNH2
Pericarpium Papaveris (PP)	MOL009327	Noskapin	AR
Pericarpium Papaveris (PP)	MOL009327	Noskapin	SCN5A
Pericarpium Papaveris (PP)	MOL009327	Noskapin	CHRM5
Pericarpium Papaveris (PP)	MOL009327	Noskapin	PTGS2
Pericarpium Papaveris (PP)	MOL009327	Noskapin	KDR
Pericarpium Papaveris (PP)	MOL009327	Noskapin	ACHE
Pericarpium Papaveris (PP)	MOL009327	Noskapin	HSP90AB1
Pericarpium Papaveris (PP)	MOL009327	Noskapin	PRSS1
Pericarpium Papaveris (PP)	MOL009327	Noskapin	CAMKMT
Pericarpium Papaveris (PP)	MOL009328	5-[[(1S)-6,7-dimethoxy-2-methyl-3,4-dihydro-1H-isoquinolin-1-yl]methyl]-2-methoxyphenol	PTGS1
Pericarpium Papaveris (PP)	MOL009328	5-[[(1S)-6,7-dimethoxy-2-methyl-3,4-dihydro-1H-isoquinolin-1-yl]methyl]-2-methoxyphenol	DRD1
Pericarpium Papaveris (PP)	MOL009328	5-[[(1S)-6,7-dimethoxy-2-methyl-3,4-dihydro-1H-isoquinolin-1-yl]methyl]-2-methoxyphenol	CHRM3
Pericarpium Papaveris (PP)	MOL009328	5-[[(1S)-6,7-dimethoxy-2-methyl-3,4-dihydro-1H-isoquinolin-1-yl]methyl]-2-methoxyphenol	KCNH2
Pericarpium Papaveris (PP)	MOL009328	5-[[(1S)-6,7-dimethoxy-2-methyl-3,4-dihydro-1H-isoquinolin-1-yl]methyl]-2-methoxyphenol	CHRM1
Pericarpium Papaveris (PP)	MOL009328	5-[[(1S)-6,7-dimethoxy-2-methyl-3,4-dihydro-1H-isoquinolin-1-yl]methyl]-2-methoxyphenol	ADRB1
Pericarpium Papaveris (PP)	MOL009328	5-[[(1S)-6,7-dimethoxy-2-methyl-3,4-dihydro-1H-isoquinolin-1-yl]methyl]-2-methoxyphenol	SCN5A
Pericarpium Papaveris (PP)	MOL009328	5-[[(1S)-6,7-dimethoxy-2-methyl-3,4-dihydro-1H-isoquinolin-1-yl]methyl]-2-methoxyphenol	CHRM5
Pericarpium Papaveris (PP)	MOL009328	5-[[(1S)-6,7-dimethoxy-2-methyl-3,4-dihydro-1H-isoquinolin-1-yl]methyl]-2-methoxyphenol	PTGS2
Pericarpium Papaveris (PP)	MOL009328	5-[[(1S)-6,7-dimethoxy-2-methyl-3,4-dihydro-1H-isoquinolin-1-yl]methyl]-2-methoxyphenol	ADRA2A
Pericarpium Papaveris (PP)	MOL009328	5-[[(1S)-6,7-dimethoxy-2-methyl-3,4-dihydro-1H-isoquinolin-1-yl]methyl]-2-methoxyphenol	ADRA2C
Pericarpium Papaveris (PP)	MOL009328	5-[[(1S)-6,7-dimethoxy-2-methyl-3,4-dihydro-1H-isoquinolin-1-yl]methyl]-2-methoxyphenol	DRD4

Pericarpium Papaveris (PP)	MOL009328	5-[[(1S)-6,7-dimethoxy-2-methyl-3,4-dihydro-1H-isoquinolin-1-yl]methyl]-2-methoxyphenol	CHRM4
Pericarpium Papaveris (PP)	MOL009328	5-[[(1S)-6,7-dimethoxy-2-methyl-3,4-dihydro-1H-isoquinolin-1-yl]methyl]-2-methoxyphenol	RXRA
Pericarpium Papaveris (PP)	MOL009328	5-[[(1S)-6,7-dimethoxy-2-methyl-3,4-dihydro-1H-isoquinolin-1-yl]methyl]-2-methoxyphenol	OPRD1
Pericarpium Papaveris (PP)	MOL009328	5-[[(1S)-6,7-dimethoxy-2-methyl-3,4-dihydro-1H-isoquinolin-1-yl]methyl]-2-methoxyphenol	SLC6A2
Pericarpium Papaveris (PP)	MOL009328	5-[[(1S)-6,7-dimethoxy-2-methyl-3,4-dihydro-1H-isoquinolin-1-yl]methyl]-2-methoxyphenol	ADRA1A
Pericarpium Papaveris (PP)	MOL009328	5-[[(1S)-6,7-dimethoxy-2-methyl-3,4-dihydro-1H-isoquinolin-1-yl]methyl]-2-methoxyphenol	CHRM2
Pericarpium Papaveris (PP)	MOL009328	5-[[(1S)-6,7-dimethoxy-2-methyl-3,4-dihydro-1H-isoquinolin-1-yl]methyl]-2-methoxyphenol	ADRA2B
Pericarpium Papaveris (PP)	MOL009328	5-[[(1S)-6,7-dimethoxy-2-methyl-3,4-dihydro-1H-isoquinolin-1-yl]methyl]-2-methoxyphenol	ADRA1B
Pericarpium Papaveris (PP)	MOL009328	5-[[(1S)-6,7-dimethoxy-2-methyl-3,4-dihydro-1H-isoquinolin-1-yl]methyl]-2-methoxyphenol	DRD3
Pericarpium Papaveris (PP)	MOL009328	5-[[(1S)-6,7-dimethoxy-2-methyl-3,4-dihydro-1H-isoquinolin-1-yl]methyl]-2-methoxyphenol	SLC6A3
Pericarpium Papaveris (PP)	MOL009328	5-[[(1S)-6,7-dimethoxy-2-methyl-3,4-dihydro-1H-isoquinolin-1-yl]methyl]-2-methoxyphenol	ADRB2
Pericarpium Papaveris (PP)	MOL009328	5-[[(1S)-6,7-dimethoxy-2-methyl-3,4-dihydro-1H-isoquinolin-1-yl]methyl]-2-methoxyphenol	ADRA1D
Pericarpium Papaveris (PP)	MOL009328	5-[[(1S)-6,7-dimethoxy-2-methyl-3,4-dihydro-1H-isoquinolin-1-yl]methyl]-2-methoxyphenol	SLC6A4
Pericarpium Papaveris (PP)	MOL009328	5-[[(1S)-6,7-dimethoxy-2-methyl-3,4-dihydro-1H-isoquinolin-1-yl]methyl]-2-methoxyphenol	DRD2
Pericarpium Papaveris (PP)	MOL009328	5-[[(1S)-6,7-dimethoxy-2-methyl-3,4-dihydro-1H-isoquinolin-1-yl]methyl]-2-methoxyphenol	OPRM1

Pericarpium Papaveris (PP)	MOL009328	5-[[(1S)-6,7-dimethoxy-2-methyl-3,4-dihydro-1H-isoquinolin-1-yl]methyl]-2-methoxyphenol	NR1I2
Pericarpium Papaveris (PP)	MOL009328	5-[[(1S)-6,7-dimethoxy-2-methyl-3,4-dihydro-1H-isoquinolin-1-yl]methyl]-2-methoxyphenol	HSP90AB1
Pericarpium Papaveris (PP)	MOL009328	5-[[(1S)-6,7-dimethoxy-2-methyl-3,4-dihydro-1H-isoquinolin-1-yl]methyl]-2-methoxyphenol	RXRB
Pericarpium Papaveris (PP)	MOL009328	5-[[(1S)-6,7-dimethoxy-2-methyl-3,4-dihydro-1H-isoquinolin-1-yl]methyl]-2-methoxyphenol	CAMKMT
Pericarpium Papaveris (PP)	MOL009329	Narcein	KCNH2
Pericarpium Papaveris (PP)	MOL009329	Narcein	SCN5A
Pericarpium Papaveris (PP)	MOL009329	Narcein	PTGS2
Pericarpium Papaveris (PP)	MOL009329	Narcein	F7
Pericarpium Papaveris (PP)	MOL009329	Narcein	KDR
Pericarpium Papaveris (PP)	MOL009329	Narcein	HSP90AB1
Pericarpium Papaveris (PP)	MOL009329	Narcein	CAMKMT
Pericarpium Papaveris (PP)	MOL009330	Noscapine	PTGS1
Pericarpium Papaveris (PP)	MOL009330	Noscapine	KCNH2
Pericarpium Papaveris (PP)	MOL009330	Noscapine	AR
Pericarpium Papaveris (PP)	MOL009330	Noscapine	SCN5A
Pericarpium Papaveris (PP)	MOL009330	Noscapine	CHRM5
Pericarpium Papaveris (PP)	MOL009330	Noscapine	PTGS2
Pericarpium Papaveris (PP)	MOL009330	Noscapine	KDR
Pericarpium Papaveris (PP)	MOL009330	Noscapine	ACHE
Pericarpium Papaveris (PP)	MOL009330	Noscapine	HSP90AB1
Pericarpium Papaveris (PP)	MOL009330	Noscapine	PRSS1
Pericarpium Papaveris (PP)	MOL009330	Noscapine	CAMKMT
Pericarpium Papaveris (PP)	MOL009331	Palaudine	PTGS1
Pericarpium Papaveris (PP)	MOL009331	Palaudine	SCN5A
Pericarpium Papaveris (PP)	MOL009331	Palaudine	CHRM5
Pericarpium Papaveris (PP)	MOL009331	Palaudine	PTGS2
Pericarpium Papaveris (PP)	MOL009331	Palaudine	RXRA
Pericarpium Papaveris (PP)	MOL009331	Palaudine	ADRA1B
Pericarpium Papaveris (PP)	MOL009331	Palaudine	ADRB2
Pericarpium Papaveris (PP)	MOL009331	Palaudine	HSP90AB1
Pericarpium Papaveris (PP)	MOL009331	Palaudine	NCOA2
Pericarpium Papaveris (PP)	MOL009331	Palaudine	CAMKMT
Pericarpium Papaveris (PP)	MOL009335	Erythroculine	PTGS1
Pericarpium Papaveris (PP)	MOL009335	Erythroculine	CHRM3
Pericarpium Papaveris (PP)	MOL009335	Erythroculine	CHRM1
Pericarpium Papaveris (PP)	MOL009335	Erythroculine	SCN5A
Pericarpium Papaveris (PP)	MOL009335	Erythroculine	CHRM5
Pericarpium Papaveris (PP)	MOL009335	Erythroculine	PTGS2
Pericarpium Papaveris (PP)	MOL009335	Erythroculine	CHRM4
Pericarpium Papaveris (PP)	MOL009335	Erythroculine	OPRD1
Pericarpium Papaveris (PP)	MOL009335	Erythroculine	ADRA1B
Pericarpium Papaveris (PP)	MOL009335	Erythroculine	ADRB2
Pericarpium Papaveris (PP)	MOL009335	Erythroculine	ADRA1D
Pericarpium Papaveris (PP)	MOL009335	Erythroculine	OPRM1
Pericarpium Papaveris (PP)	MOL009338	Norswertianin	PTGS1
Pericarpium Papaveris (PP)	MOL009338	Norswertianin	AR
Pericarpium Papaveris (PP)	MOL009338	Norswertianin	PTGS2
Pericarpium Papaveris (PP)	MOL009338	Norswertianin	HSP90AB1

277 active target genes of ZRYZD

GABRA1

PGR
NR3C2
TNFAIP6
IL6R
CD14
LBP
NCOA2
PTGS1
PTGS2
HSP90AB1
KCNH2
DRD1
CHRM3
CHRM1
SCN5A
CHRM4
ADRA1A
CHRM2
ADRA1B
ADRB2
CHRNA2
SLC6A4
OPRM1
BCL2
BAX
CASP9
JUN
CASP3
CASP8
PRKCA
PON1
MAP2
NOS2
AR
PPARG
DPP4
PRSS1
ACHE
SLC6A2
F7
CAMKMT
RELA
IKBKB
AKT1
AHSA1
MAPK8
MMP1
STAT1
CDK1
HMOX1
CYP3A4
CYP1A2
CYP1A1
ICAM1
SELE
VCAM1
NR1I2
CYP1B1
ALOX5
HAS2
GSTP1
AHR
PSMD3

SLC2A4
NR1I3
INSRR
DIO1
PPP3CA
GSTM1
GSTM2
AKR1C3
SLPI
ESR1
DPEP1
RXRA
CAT
NCOA1
ADH1C
IGHG1
ADRA2A
SLC6A3
AKR1B1
PLAU
LTA4H
MAOB
MAOA
CTRB1
ADRB1
ADRA1D
ESR2
MAPK14
GSK3B
CDK2
CHEK1
CCNA2
PKIA
CHRM5
EGFR
VEGFA
CCND1
BCL2L1
CDKN1A
MMP2
MMP9
MAPK1
IL10RA
RB1
CDK4
TP53
NFKBIA
TOP1
MDM2
APP
PCNA
ERBB2
CASP7
MCL1
BIRC5
IL2RA
CCNB1
TYR
IFNG
IL4
TOP2A
XIAP
CD40LG

PTGES
NUF2
ADCY2
MET
CA2
OPRD1
TUBB1
NR3C1
MMP13
MMP8
HTR3A
KDR
MAPK10
PYGM
PPARD
GRIA2
NCF1
OLR1
ATP5F1B
HSD3B2
HSD3B1
MAPK3
FASN
LDLR
BAD
SOD1
MTTP
APOB
PLB1
HMGCR
CYP19A1
UGT1A1
PPARA
SREBF1
GSR
ABCC1
ADIPOR2
SOAT2
AKR1C1
GOT1
ABAT
CES1
SOAT1
BACE2
RXRB
STAT3
EIF6
FOSL2
MMP3
FOS
EGF
ELK1
POR
ODC1
RAF1
HIF1A
RUNX1T1
HSPA5
ACACA
CAV1
MYC
F3
GJA1

IL1B
CCL2
PTGER3
CXCL8
PRKCB
DUOX2
NOS3
HSPB1
SULT1E1
PLAT
THBD
SERPINE1
COL1A1
IL1A
MPO
ABCG2
NFE2L2
NQO1
PARP1
COL3A1
CXCL11
CXCL2
DCAF5
CHEK2
CLDN4
HSF1
CXCL10
CHUK
SPP1
RUNX2
RASSF1
E2F1
E2F2
ACP3
CTSD
IGFBP3
IGF2
IRF1
ERBB3
PCOLCE
NPEPPS
HK2
RASA1
GSTA1
GSTA2
RASGRF1
CDK12
ADRA2C
DRD4
ADRA2B
DRD3
DRD2
LYG1
TRPV1
ADH1B
ADH1A
PRSS3
TEP1
EDN1
LPL
GAP43
BDNF
INS

SERPINB2
FABP1
RBP2
GCG
ENPEP
UCP2
CCKAR
CITED1
NTRK2
PDX1
SLC2A2
PAM
SCD
UCP3
CETP
PYY
DNPEP
PDE10A
TDP1
ADM
DRD5
HRH1
OPRK1
CACNA1S
