# Supplementary material for: Analysis of the Efficacy and Pharmacological Mechanisms of Action of Zhenren Yangzang Decoction on Ulcerative Colitis Using Meta-Analysis and Network Pharmacology
Source: Evid Based Complement Alternat Med. 2021 Dec 28;2021:4512755. doi: 10.1155/2021/4512755 (PMC8727130; doi:10.1155/2021/4512755)
Supplement: Supplementary Materials — Figure S1: Risk of bias graph. Figure S2: risk of bias summary. Figure S3: forest plot of comparison of serum cytokines. Figure S4: forest plot of comparison of the total syndrome score of TCM. Table S1: basic information on the active compounds in ZRYZD. Table S2: gene symbols and entrezID of active target genes. Table S3: compounds ranked by the degree in the network. Supplementary File 1: compounds of ZRYZD from TCMSP. Supplementary File 2: corresponding target genes of ZRYZD. Supplementary File 3: UC-related target genes. Supplementary File 4: GO functional enrichment analysis. Supplementary File 5: KEGG pathway enrichment analysis. Supplementary File 6: data of compound-target networks. Supplementary File 7: data of key compound-target networks. Supplementary File 8: data of PPI network. [file 4512755.f1.zip › 4512755.f1/Supplementary File 3 UC-related genes.pdf]

Supplementary File 3 UC-related genes

UC-related target genes from GeneCards

Gene Symbol	Description	Category	GC Id	Relevance score
NOD2	Nucleotide Binding Oligomerization Domain Containing 2	Protein Coding	GC16P050693	61.31760406
IL6	Interleukin 6	Protein Coding	GC07P022725	44.76377106
IL10	Interleukin 10	Protein Coding	GC01M206767	43.48468018
IL23R	Interleukin 23 Receptor	Protein Coding	GC01P067138	38.40746689
TNF	Tumor Necrosis Factor	Protein Coding	GC06P058308	37.86817551
HLA-DRB1	Major Histocompatibility Complex, Class II, DR Beta 1	Protein Coding	GC06M032578	34.47991943
TLR4	Toll Like Receptor 4	Protein Coding	GC09P117704	33.98320007
IL1B	Interleukin 1 Beta	Protein Coding	GC02M112829	32.96234131
ABCB1	ATP Binding Cassette Subfamily B Member 1	Protein Coding	GC07M087504	28.63883591
IL10RA	Interleukin 10 Receptor Subunit Alpha	Protein Coding	GC11P117987	28.12284851
CXCL8	C-X-C Motif Chemokine Ligand 8	Protein Coding	GC04P073740	27.71114731
INAVA	Innate Immunity Activator	Protein Coding	GC01P200892	27.31973267
MPO	Myeloperoxidase	Protein Coding	GC17M058269	26.61129761
IL1RN	Interleukin 1 Receptor Antagonist	Protein Coding	GC02P117302	26.30961227
HLA-B	Major Histocompatibility Complex, Class I, B	Protein Coding	GC06M049073	25.47437668
IL2	Interleukin 2	Protein Coding	GC04M122451	25.03628159
IFNG	Interferon Gamma	Protein Coding	GC12M068154	24.93884087
ATG16L1	Autophagy Related 16 Like 1	Protein Coding	GC02P233220	24.71202087
IRF5	Interferon Regulatory Factor 5	Protein Coding	GC07P128937	24.69466591
PRKCQ	Protein Kinase C Theta	Protein Coding	GC10M006393	23.71396065
CTLA4	Cytotoxic T-Lymphocyte Associated Protein 4	Protein Coding	GC02P203867	23.12203598
CRP	C-Reactive Protein	Protein Coding	GC01M159720	22.71346283
TP53	Tumor Protein P53	Protein Coding	GC17M007661	22.49951935
SYK	Spleen Associated Tyrosine Kinase	Protein Coding	GC09P091451	22.49906349
PLA2G4A	Phospholipase A2 Group IVA	Protein Coding	GC01P186798	22.3304348
TLR2	Toll Like Receptor 2	Protein Coding	GC04P153684	22.13373566
IRGM	Immunity Related GTPase M	Protein Coding	GC05P150846	22.11695862
MLH1	MutL Homolog 1	Protein Coding	GC03P036993	21.76278496
MEFV	MEFV Innate Immuity Regulator, Pyrin	Protein Coding	GC16M003970	21.67788696
IL4	Interleukin 4	Protein Coding	GC05P132673	21.38653564
FAS	Fas Cell Surface Death Receptor	Protein Coding	GC10P088969	21.20170593
CCR6	C-C Motif Chemokine Receptor 6	Protein Coding	GC06P167111	21.05269623
CTNNB1	Catenin Beta 1	Protein Coding	GC03P041236	20.93886948
IL10RB	Interleukin 10 Receptor Subunit Beta	Protein Coding	GC21P033266	20.90147781
MST1	Macrophage Stimulating 1	Protein Coding	GC03M049683	20.89999771
IL17A	Interleukin 17A	Protein Coding	GC06P052186	20.89733505
SLC11A1	Solute Carrier Family 11 Member 1	Protein Coding	GC02P218382	20.32146645
TGFB1	Transforming Growth Factor Beta 1	Protein Coding	GC19M041301	20.10072899
RELA	RELA Proto-Oncogene, NF-KB	Protein Coding	GC11M065653	19.91537476
ELANE	Elastase, Neutrophil Expressed	Protein Coding	GC19P000871	19.26919174
BTNL2	Butyrophilin Like 2	Protein Coding	GC06M032393	19.19261742
ICAM1	Intercellular Adhesion Molecule 1	Protein Coding	GC19P010270	19.13099289
PTGS2	Prostaglandin-Endoperoxide Synthase 2	Protein Coding	GC01M186640	18.74561691
STAT4	Signal Transducer And Activator Of Transcription 4	Protein Coding	GC02M191029	18.49963951
TNFAIP3	TNF Alpha Induced Protein 3	Protein Coding	GC06P137866	18.47131729
GAST	Gastrin	Protein Coding	GC17P041712	18.06595421
AKT1	AKT Serine/Threonine Kinase 1	Protein Coding	GC14M104769	18.04285431
TNFSF15	TNF Superfamily Member 15	Protein Coding	GC09M114784	17.95145035
SMAD4	SMAD Family Member 4	Protein Coding	GC18P051028	17.85460854
PPARG	Peroxisome Proliferator Activated Receptor Gamma	Protein Coding	GC03P012287	17.72105026
SMAD7	SMAD Family Member 7	Protein Coding	GC18M048919	17.67390251
MUC2	Mucin 2, Oligomeric Mucus/Gel-	Protein Coding	GC11P001074	17.60122299

STAT3	Signal Transducer And Activator Of Transcription 3	Protein Coding	GC17M042313	17.49925613
PRTN3	Proteinase 3	Protein Coding	GC19P000840	17.47821236
MSH2	MutS Homolog 2	Protein Coding	GC02P047402	17.31903458
MMP1	Matrix Metallopeptidase 1	Protein Coding	GC11M102810	17.26772499
IL13	Interleukin 13	Protein Coding	GC05P132656	17.10809708
IL1R1	Interleukin 1 Receptor Type 1	Protein Coding	GC02P102136	17.08084488
LTF	Lactotransferrin	Protein Coding	GC03M046435	17.07393265
GPR35	G Protein-Coupled Receptor 35	Protein Coding	GC02P240605	16.98833275
CHEK2	Checkpoint Kinase 2	Protein Coding	GC22M028687	16.98612022
TNFRSF1A	TNF Receptor Superfamily Member 1A	Protein Coding	GC12M006328	16.89917946
TPMT	Thiopurine S-Methyltransferase	Protein Coding	GC06M018128	16.81839752
NLRP3	NLR Family Pyrin Domain Containing	Protein Coding	GC01P247415	16.78907776
IL22	Interleukin 22	Protein Coding	GC12M068248	16.57520866
VEGFA	Vascular Endothelial Growth Factor A	Protein Coding	GC06P043770	16.47510719
MSH6	MutS Homolog 6	Protein Coding	GC02P047695	16.40106392
PSTPIP1	Proline-Serine-Threonine Phosphatase Interacting Protein 1	Protein Coding	GC15P076993	16.32021713
MMP9	Matrix Metallopeptidase 9	Protein Coding	GC20P046008	16.16921043
HLA-DQB1	Major Histocompatibility Complex, Class II, DQ Beta 1	Protein Coding	GC06M049175	15.94390869
FOXP3	Forkhead Box P3	Protein Coding	GC0XM049250	15.93480873
CDH1	Cadherin 1	Protein Coding	GC16P068737	15.77756405
S100A8	S100 Calcium Binding Protein A8	Protein Coding	GC01M153391	15.7771244
MYO9B	Myosin IXB	Protein Coding	GC19P027765	15.60248375
IL21	Interleukin 21	Protein Coding	GC04M122612	15.59376144
CCR3	C-C Motif Chemokine Receptor 3	Protein Coding	GC03P046163	15.54749298
TCF4	Transcription Factor 4	Protein Coding	GC18M055222	15.42352486
REL	REL Proto-Oncogene, NF-KB Subunit	Protein Coding	GC02P060881	15.41527748
ZFP90	ZFP90 Zinc Finger Protein	Protein Coding	GC16P068530	15.39490795
CD4	CD4 Molecule	Protein Coding	GC12P006786	15.37931347
NOS2	Nitric Oxide Synthase 2	Protein Coding	GC17M027756	15.21494675
APC	APC Regulator Of WNT Signaling Pathway	Protein Coding	GC05P112707	15.19603729
REG4	Regenerating Family Member 4	Protein Coding	GC01M119794	15.07591915
CTSG	Cathepsin G	Protein Coding	GC14M024573	15.02377892
CYP2C19	Cytochrome P450 Family 2 Subfamily C Member 19	Protein Coding	GC10P094762	15.0123148
NFKB1	Nuclear Factor Kappa B Subunit 1	Protein Coding	GC04P102501	14.87479782
PTGS1	Prostaglandin-Endoperoxide Synthase 1	Protein Coding	GC09P122370	14.72512531
IL23A	Interleukin 23 Subunit Alpha	Protein Coding	GC12P056509	14.66438389
PTPN22	Protein Tyrosine Phosphatase Non-Receptor Type 22	Protein Coding	GC01M113813	14.56217194
ALB	Albumin	Protein Coding	GC04P073397	14.48280334
JAK2	Janus Kinase 2	Protein Coding	GC09P004985	14.47116852
HRH2	Histamine Receptor H2	Protein Coding	GC05P175659	14.45461273
LACC1	Laccase Domain Containing 1	Protein Coding	GC13P043879	14.36295795
S100A12	S100 Calcium Binding Protein A12	Protein Coding	GC01M153373	14.27696609
SMAD3	SMAD Family Member 3	Protein Coding	GC15P067063	14.05381107
CYBC1	Cytochrome B-245 Chaperone 1	Protein Coding	GC17M082443	13.96999264
IL18	Interleukin 18	Protein Coding	GC11M112143	13.94542408
CLDN18	Claudin 18	Protein Coding	GC03P137998	13.84757805
NCF4	Neutrophil Cytosolic Factor 4	Protein Coding	GC22P036860	13.74964333
BPI	Bactericidal Permeability Increasing Protein	Protein Coding	GC20P038304	13.73793793
SST	Somatostatin	Protein Coding	GC03M187668	13.44919872
ERAP1	Endoplasmic Reticulum Aminopeptidase 1	Protein Coding	GC05M096760	13.41335583
IL12A	Interleukin 12A	Protein Coding	GC03P159988	13.34497452
CARD9	Caspase Recruitment Domain Family Member 9	Protein Coding	GC09M136361	13.29592133
NOD1	Nucleotide Binding Oligomerization Domain Containing 1	Protein Coding	GC07M030424	13.28494263

TNFRSF1B	TNF Receptor Superfamily Member 1B	Protein Coding	GC01P012167	13.26928139
PIK3CA	Phosphatidylinositol-4,5-Bisphosphate 3-Kinase Catalytic Subunit Alpha	Protein Coding	GC03P179148	13.25791645
CIITA	Class II Major Histocompatibility Complex Transactivator	Protein Coding	GC16P010879	13.22174168
CAT	Catalase	Protein Coding	GC11P034460	13.06159687
C4A	Complement C4A (Rodgers Blood Group)	Protein Coding	GC06P058333	12.97477913
CCR1	C-C Motif Chemokine Receptor 1	Protein Coding	GC03M046218	12.9359827
SLC22A4	Solute Carrier Family 22 Member 4	Protein Coding	GC05P132294	12.87298775
ATP4A	ATPase H+/K+ Transporting Subunit Alpha	Protein Coding	GC19M048913	12.75074387
HLA-A	Major Histocompatibility Complex, Class I, A	Protein Coding	GC06P058250	12.72643089
CCL2	C-C Motif Chemokine Ligand 2	Protein Coding	GC17P034255	12.71583557
CARD8	Caspase Recruitment Domain Family Member 8	Protein Coding	GC19M048900	12.63211155
KRAS	KRAS Proto-Oncogene, GTPase	Protein Coding	GC12M025204	12.60593796
SLC22A5	Solute Carrier Family 22 Member 5	Protein Coding	GC05P132369	12.59175301
CLDN2	Claudin 2	Protein Coding	GC0XP106900	12.58665085
F5	Coagulation Factor V	Protein Coding	GC01M169511	12.5197506
DEFB4A	Defensin Beta 4A	Protein Coding	GC08P007895	12.50758743
SOCS1	Suppressor Of Cytokine Signaling 1	Protein Coding	GC16M011255	12.38961029
DUOX2	Dual Oxidase 2	Protein Coding	GC15M045092	12.37053013
CARMIL2	Capping Protein Regulator And Myosin 1 Linker 2	Protein Coding	GC16P067644	12.33283615
MUC12	Mucin 12, Cell Surface Associated	Protein Coding	GC07P100969	12.27649021
CCL11	C-C Motif Chemokine Ligand 11	Protein Coding	GC17P034285	12.27423286
IL2RA	Interleukin 2 Receptor Subunit Alpha	Protein Coding	GC10M006010	12.26793575
NGF	Nerve Growth Factor	Protein Coding	GC01M115285	12.18489742
F2	Coagulation Factor II, Thrombin	Protein Coding	GC11P046720	12.17591095
UCN	Urocortin	Protein Coding	GC02M027308	12.14851379
MET	MET Proto-Oncogene, Receptor Tyrosine Kinase	Protein Coding	GC07P116672	12.13383484
IL12B	Interleukin 12B	Protein Coding	GC05M159314	12.00514221
BRAF	B-Raf Proto-Oncogene, Serine/Threonine Kinase	Protein Coding	GC07M140718	11.91674232
CHGA	Chromogranin A	Protein Coding	GC14P092929	11.9086256
NTRK1	Neurotrophic Receptor Tyrosine Kinase	Protein Coding	GC01P156815	11.87965107
CD40LG	CD40 Ligand	Protein Coding	GC0XP136649	11.82111549
EGF	Epidermal Growth Factor	Protein Coding	GC04P109912	11.78321457
FGFR2	Fibroblast Growth Factor Receptor 2	Protein Coding	GC10M121478	11.66446686
FGF2	Fibroblast Growth Factor 2	Protein Coding	GC04P122826	11.65516281
UBAC2	UBA Domain Containing 2	Protein Coding	GC13P099200	11.59321404
FASLG	Fas Ligand	Protein Coding	GC01P172628	11.49381351
KLRC4	Killer Cell Lectin Like Receptor C4	Protein Coding	GC12M016313	11.48016262
ICOSLG	Inducible T Cell Costimulator Ligand	Protein Coding	GC21M044222	11.47869396
CCND1	Cyclin D1	Protein Coding	GC11P069641	11.46121979
RIPK1	Receptor Interacting Serine/Threonine Kinase 1	Protein Coding	GC06P003073	11.45344162
IL1A	Interleukin 1 Alpha	Protein Coding	GC02M112773	11.44147682
KIF1A	Kinesin Family Member 1A	Protein Coding	GC02M240713	11.43692493
NCF1	Neutrophil Cytosolic Factor 1	Protein Coding	GC07P074773	11.43011093
IL7R	Interleukin 7 Receptor	Protein Coding	GC05P035852	11.23851204
TFF3	Trefoil Factor 3	Protein Coding	GC21M042311	11.22328568
HLA-DQA1	Major Histocompatibility Complex, Class II, DQ Alpha 1	Protein Coding	GC06P058340	11.20988464
SLC37A4	Solute Carrier Family 37 Member 4	Protein Coding	GC11M119024	11.12964725
MIR21	MicroRNA 21	RNA Gene	GC17P059841	11.11921883
CDKN1A	Cyclin Dependent Kinase Inhibitor 1A	Protein Coding	GC06P058459	11.07493877
HMOX1	Heme Oxygenase 1	Protein Coding	GC22P035380	11.01327324
H19	H19 Imprinted Maternally Expressed Transcript	RNA Gene	GC11M001995	10.97323704

IL37	Interleukin 37	Protein Coding	GC02P117299	10.96678162
NKX2-3	NK2 Homeobox 3	Protein Coding	GC10P099532	10.94610023
TIMP1	TIMP Metallopeptidase Inhibitor 1	Protein Coding	GC0XP047583	10.91073608
TLR5	Toll Like Receptor 5	Protein Coding	GC01M223185	10.88015461
IBD3	Inflammatory Bowel Disease 3	Genetic Locus	GC06U990223	10.81249428
IFNG-AS1	IFNG Antisense RNA 1	RNA Gene	GC12P067989	10.74646759
S100A9	S100 Calcium Binding Protein A9	Protein Coding	GC01P153357	10.66565132
BAX	BCL2 Associated X, Apoptosis	Protein Coding	GC19P048954	10.65752506
VDR	Vitamin D Receptor	Protein Coding	GC12M047841	10.6275444
XIAP	X-Linked Inhibitor Of Apoptosis	Protein Coding	GC0XP123859	10.5450592
CXCL5	C-X-C Motif Chemokine Ligand 5	Protein Coding	GC04M073995	10.51688576
PTEN	Phosphatase And Tensin Homolog	Protein Coding	GC10P087863	10.47771454
CD8A	CD8a Molecule	Protein Coding	GC02M086784	10.46233368
CSF2	Colony Stimulating Factor 2	Protein Coding	GC05P132073	10.39040375
ITGB4	Integrin Subunit Beta 4	Protein Coding	GC17P075721	10.379179
POLD1	DNA Polymerase Delta 1, Catalytic Subunit	Protein Coding	GC19P050385	10.37687683
MTHFR	Methylenetetrahydrofolate Reductase	Protein Coding	GC01M011785	10.34389114
LTA	Lymphotoxin Alpha	Protein Coding	GC06P058305	10.31660652
FGFR3	Fibroblast Growth Factor Receptor 3	Protein Coding	GC04P001795	10.29501343
APOH	Apolipoprotein H	Protein Coding	GC17M066212	10.293993
PLCG2	Phospholipase C Gamma 2	Protein Coding	GC16P081773	10.23431969
HP	Haptoglobin	Protein Coding	GC16P072089	10.17968941
TLR9	Toll Like Receptor 9	Protein Coding	GC03M052222	10.16080284
MMP2	Matrix Metallopeptidase 2	Protein Coding	GC16P055390	10.15537453
BACH2	BTB Domain And CNC Homolog 2	Protein Coding	GC06M089926	10.14404583
TFF2	Trefoil Factor 2	Protein Coding	GC21M042346	10.13309765
FCGR2A	Fc Fragment Of IgG Receptor IIa	Protein Coding	GC01P161505	10.13227654
ITGB2	Integrin Subunit Beta 2	Protein Coding	GC21M044885	10.08755493
MIF	Macrophage Migration Inhibitory	Protein Coding	GC22P023894	10.07565117
RTEL1	Regulator Of Telomere Elongation Helicase 1	Protein Coding	GC20P063658	10.06011581
MIR126	MicroRNA 126	RNA Gene	GC09P136670	10.05615807
ERBB2	Erb-B2 Receptor Tyrosine Kinase 2	Protein Coding	GC17P039687	9.972842216
STAT1	Signal Transducer And Activator Of Transcription 1	Protein Coding	GC02M190908	9.931564331
PYY	Peptide YY	Protein Coding	GC17M043952	9.881679535
CDKN1B	Cyclin Dependent Kinase Inhibitor 1B	Protein Coding	GC12P013453	9.848312378
RNASE3	Ribonuclease A Family Member 3	Protein Coding	GC14P020891	9.841001511
PTPN2	Protein Tyrosine Phosphatase Non-Receptor Type 2	Protein Coding	GC18M019720	9.830123901
PDGFB	Platelet Derived Growth Factor Subunit	Protein Coding	GC22M050295	9.792757034
SERPINE1	Serpin Family E Member 1	Protein Coding	GC07P101127	9.685308456
SERPINC1	Serpin Family C Member 1	Protein Coding	GC01M174280	9.661153793
TFF1	Trefoil Factor 1	Protein Coding	GC21M042362	9.599998474
IBD21	Inflammatory Bowel Disease-21	Genetic Locus	GC18U900466	9.592031479
CCL5	C-C Motif Chemokine Ligand 5	Protein Coding	GC17M035871	9.540237427
CCN2	Cellular Communication Network Factor 2	Protein Coding	GC06M131948	9.496431351
SLC6A4	Solute Carrier Family 6 Member 4	Protein Coding	GC17M030194	9.486737251
IL17F	Interleukin 17F	Protein Coding	GC06M052209	9.47974968
CLDN4	Claudin 4	Protein Coding	GC07P073799	9.479379654
CP	Ceruloplasmin	Protein Coding	GC03M149162	9.474424362
PTGER4	Prostaglandin E Receptor 4	Protein Coding	GC05P040679	9.459547997
FERMT1	FERM Domain Containing Kindlin 1	Protein Coding	GC20M006074	9.411668777
LOC106029312	Williams-Beuren Syndrome Medial Block B Recombination Region	Biological Region	GC07P074733	9.394348145
MVK	Mevalonate Kinase	Protein Coding	GC12P109573	9.373778343
HLA-C	Major Histocompatibility Complex, Class I, C	Protein Coding	GC06M049072	9.373630524
ACE	Angiotensin I Converting Enzyme	Protein Coding	GC17P063477	9.371356964
MMP3	Matrix Metallopeptidase 3	Protein Coding	GC11M102835	9.324460983
CD40	CD40 Molecule	Protein Coding	GC20P046118	9.271253586

CXCL10	C-X-C Motif Chemokine Ligand 10	Protein Coding	GC04M076021	9.259418488
ATP12A	ATPase H+/K+ Transporting Non-Gastric Alpha2 Subunit	Protein Coding	GC13P024680	9.22936821
IL5	Interleukin 5	Protein Coding	GC05M132541	9.216200829
ATM	ATM Serine/Threonine Kinase	Protein Coding	GC11P108222	9.207765579
MADCAM1	Mucosal Vascular Addressin Cell Adhesion Molecule 1	Protein Coding	GC19P000499	9.203808784
XDH	Xanthine Dehydrogenase	Protein Coding	GC02M031334	9.194812775
NCF4-AS1	NCF4 Antisense RNA 1	RNA Gene	GC22M036848	9.191848755
SPP1	Secreted Phosphoprotein 1	Protein Coding	GC04P087975	9.153892517
MIR143	MicroRNA 143	RNA Gene	GC05P149410	9.080171585
IBD5	Inflammatory Bowel Disease 5	Genetic Locus	GC05U990104	9.079391479
MICA	MHC Class I Polypeptide-Related Sequence A	Protein Coding	GC06P031399	9.061130524
CFB	Complement Factor B	Protein Coding	GC06P031945	9.051132202
PSC	Cholangitis, Primary Sclerosing	Genetic Locus	GC03U901834	9.005079269
MASP2	MBL Associated Serine Protease 2	Protein Coding	GC01M011026	8.986049652
IL15	Interleukin 15	Protein Coding	GC04P141636	8.982059479
MIR34A	MicroRNA 34a	RNA Gene	GC01M009151	8.97936821
FOXD2-AS1	FOXD2 Adjacent Opposite Strand	RNA Gene	GC01M047432	8.95472908
VCAM1	Vascular Cell Adhesion Molecule 1	Protein Coding	GC01P100719	8.935503006
CD14	CD14 Molecule	Protein Coding	GC05M140631	8.90542984
CYP2C9	Cytochrome P450 Family 2 Subfamily C Member 9	Protein Coding	GC10P094938	8.903923035
DEFA5	Defensin Alpha 5	Protein Coding	GC08M007057	8.898145676
DLG5	Discs Large MAGUK Scaffold Protein	Protein Coding	GC10M077790	8.885620117
ZAP70	Zeta Chain Of T Cell Receptor Associated Protein Kinase 70	Protein Coding	GC02P097696	8.885093689
MAP3K7	Mitogen-Activated Protein Kinase Kinase Kinase 7	Protein Coding	GC06M090513	8.882299423
HGF	Hepatocyte Growth Factor	Protein Coding	GC07M081699	8.880085945
PTPRC	Protein Tyrosine Phosphatase Receptor Type C	Protein Coding	GC01P198607	8.841666222
BCL10	BCL10 Immune Signaling Adaptor	Protein Coding	GC01M085265	8.815534592
PLA2G2A	Phospholipase A2 Group IIA	Protein Coding	GC01M019975	8.798325539
RET	Ret Proto-Oncogene	Protein Coding	GC10P043081	8.789283752
MIR145	MicroRNA 145	RNA Gene	GC05P149430	8.74485302
CYP3A4	Cytochrome P450 Family 3 Subfamily A Member 4	Protein Coding	GC07M099759	8.69412899
IGF2	Insulin Like Growth Factor 2	Protein Coding	GC11M002130	8.688007355
CDKN2A	Cyclin Dependent Kinase Inhibitor 2A	Protein Coding	GC09M021967	8.629448891
CD79A	CD79a Molecule	Protein Coding	GC19P041877	8.624217033
INS	Insulin	Protein Coding	GC11M002159	8.615140915
PALB2	Partner And Localizer Of BRCA2	Protein Coding	GC16M023603	8.605494499
PLG	Plasminogen	Protein Coding	GC06P160702	8.599830627
IBD2	Inflammatory Bowel Disease 2	Genetic Locus	GC12U990041	8.555940628
HLA-DRA	Major Histocompatibility Complex, Class II, DR Alpha	Protein Coding	GC06P032439	8.552776337
NFKBIA	NFKB Inhibitor Alpha	Protein Coding	GC14M035401	8.534114838
LAMB1	Laminin Subunit Beta 1	Protein Coding	GC07M107923	8.466656685
TERT	Telomerase Reverse Transcriptase	Protein Coding	GC05M001253	8.413776398
CCK	Cholecystokinin	Protein Coding	GC03M042274	8.404132843
EPX	Eosinophil Peroxidase	Protein Coding	GC17P058192	8.398321152
ITGA4	Integrin Subunit Alpha 4	Protein Coding	GC02P181456	8.367609024
CALCA	Calcitonin Related Polypeptide Alpha	Protein Coding	GC11M014945	8.34990406
EPCAM	Epithelial Cell Adhesion Molecule	Protein Coding	GC02P047345	8.347817421
LCN2	Lipocalin 2	Protein Coding	GC09P128149	8.34458828
MUC5AC	Mucin 5AC, Oligomeric Mucus/Gel-Forming	Protein Coding	GC11P001151	8.329996109
CCR5	C-C Motif Chemokine Receptor 5	Protein Coding	GC03P046383	8.325737953
HPS1	HPS1 Biogenesis Of Lysosomal Organelles Complex 3 Subunit 1	Protein Coding	GC10M098416	8.298640251
CD80	CD80 Molecule	Protein Coding	GC03M119524	8.247433662

CASP1	Caspase 1	Protein Coding	GC11M105025	8.216702461
EP300	E1A Binding Protein P300	Protein Coding	GC22P041091	8.208216667
CCT5	Chaperonin Containing TCP1 Subunit 5	Protein Coding	GC05P010236	8.2023592
MICB	MHC Class I Polypeptide-Related Sequence B	Protein Coding	GC06P058302	8.186508179
MIR141	MicroRNA 141	RNA Gene	GC12P013193	8.141168594
FCGR3A	Fc Fragment Of IgG Receptor IIIa	Protein Coding	GC01M161541	8.131402969
HIF1A	Hypoxia Inducible Factor 1 Subunit	Protein Coding	GC14P061695	8.09721756
CXCR2	C-X-C Motif Chemokine Receptor 2	Protein Coding	GC02P218125	8.063299179
CYLD	CYLD Lysine 63 Deubiquitinase	Protein Coding	GC16P050742	8.012464523
SCT	Secretin	Protein Coding	GC11M000626	7.997873783
GRP	Gastrin Releasing Peptide	Protein Coding	GC18P059220	7.997140884
CCL20	C-C Motif Chemokine Ligand 20	Protein Coding	GC02P227820	7.934663773
CDKN2B	Cyclin Dependent Kinase Inhibitor 2B	Protein Coding	GC09M022002	7.911164284
NAT2	N-Acetyltransferase 2	Protein Coding	GC08P018391	7.865217209
TACR1	Tachykinin Receptor 1	Protein Coding	GC02M075010	7.857832909
EGFR	Epidermal Growth Factor Receptor	Protein Coding	GC07P055019	7.850435257
CD28	CD28 Molecule	Protein Coding	GC02P203706	7.835213184
IL21-AS1	IL21 Antisense RNA 1	RNA Gene	GC04P122544	7.832902431
TYK2	Tyrosine Kinase 2	Protein Coding	GC19M010350	7.828643322
IL26	Interleukin 26	Protein Coding	GC12M068201	7.754127502
MLN	Motilin	Protein Coding	GC06M033794	7.726494789
IL11	Interleukin 11	Protein Coding	GC19M055364	7.725746155
RIPK2	Receptor Interacting Serine/Threonine Kinase 2	Protein Coding	GC08P089758	7.725734711
SELE	Selectin E	Protein Coding	GC01M169722	7.723042965
KRT7	Keratin 7	Protein Coding	GC12P052232	7.659073353
CCL3	C-C Motif Chemokine Ligand 3	Protein Coding	GC17M036088	7.643129826
HSPA1L	Heat Shock Protein Family A (Hsp70) Member 1 Like	Protein Coding	GC06M031809	7.641442299
ITGAL	Integrin Subunit Alpha L	Protein Coding	GC16P030472	7.601589203
VIP	Vasoactive Intestinal Peptide	Protein Coding	GC06P152750	7.595669746
HPS4	HPS4 Biogenesis Of Lysosomal Organelles Complex 3 Subunit 2	Protein Coding	GC22M026443	7.477524281
WRN	WRN RecQ Like Helicase	Protein Coding	GC08P031033	7.477056026
CYP2D6	Cytochrome P450 Family 2 Subfamily D Member 6	Protein Coding	GC22M042126	7.453791618
FUT2	Fucosyltransferase 2	Protein Coding	GC19P048695	7.395876884
IL33	Interleukin 33	Protein Coding	GC09P006206	7.368725777
RASGRP1	RAS Guanyl Releasing Protein 1	Protein Coding	GC15M038488	7.367242813
CSF3	Colony Stimulating Factor 3	Protein Coding	GC17P040015	7.339954376
ARID1A	AT-Rich Interaction Domain 1A	Protein Coding	GC01P026693	7.326479912
TCN2	Transcobalamin 2	Protein Coding	GC22P030606	7.277854443
IL1RAPL2	Interleukin 1 Receptor Accessory Protein Like 2	Protein Coding	GC0XP104566	7.257511139
IBD8	Inflammatory Bowel Disease 8	Genetic Locus	GC16U990238	7.229406357
ITGAM	Integrin Subunit Alpha M	Protein Coding	GC16P033523	7.184260368
CASP10	Caspase 10	Protein Coding	GC02P201182	7.18032074
FGF10	Fibroblast Growth Factor 10	Protein Coding	GC05M044340	7.174581051
TAC1	Tachykinin Precursor 1	Protein Coding	GC07P097731	7.163695335
RTEL1-TNFRSF6B	RTEL1-TNFRSF6B Readthrough (NMD Candidate)	RNA Gene	GC20P063657	7.145471573
CXCL2	C-X-C Motif Chemokine Ligand 2	Protein Coding	GC04M074097	7.080943108
MT-CO1	Mitochondrially Encoded Cytochrome C Oxidase I	Protein Coding	GCMTP005906	7.071482182
TAP2	Transporter 2, ATP Binding Cassette Subfamily B Member	Protein Coding	GC06M032821	7.053660393
SOD1	Superoxide Dismutase 1	Protein Coding	GC21P031659	7.052682877
TAP1	Transporter 1, ATP Binding Cassette Subfamily B Member	Protein Coding	GC06M049182	7.02402544
POLE	DNA Polymerase Epsilon, Catalytic Subunit	Protein Coding	GC12M132641	7.016212463
CXCL1	C-X-C Motif Chemokine Ligand 1	Protein Coding	GC04P073869	7.009435654

FGF7	Fibroblast Growth Factor 7	Protein Coding	GC15P049423	7.007907391
FCGR3B	Fc Fragment Of IgG Receptor IIIb	Protein Coding	GC01M161623	6.987466812
SOCS3	Suppressor Of Cytokine Signaling 3	Protein Coding	GC17M078356	6.980952263
PDCD1	Programmed Cell Death 1	Protein Coding	GC02M241849	6.974276543
LRBA	LPS Responsive Beige-Like Anchor Protein	Protein Coding	GC04M150264	6.971558094
CD55	CD55 Molecule (Cromer Blood Group)	Protein Coding	GC01P207321	6.970367432
FN1	Fibronectin 1	Protein Coding	GC02M215360	6.961168289
CDH3	Cadherin 3	Protein Coding	GC16P068637	6.934838295
TJP1	Tight Junction Protein 1	Protein Coding	GC15M029699	6.921606064
IBD7	Inflammatory Bowel Disease 7	Genetic Locus	GC01U990338	6.91445446
TLR3	Toll Like Receptor 3	Protein Coding	GC04P186059	6.890994072
CYP1A2	Cytochrome P450 Family 1 Subfamily A Member 2	Protein Coding	GC15P074748	6.861077785
AXIN2	Axin 2	Protein Coding	GC17M065528	6.855237961
LGALS3	Galectin 3	Protein Coding	GC14P055124	6.850400925
HLA-DPB1	Major Histocompatibility Complex, Class II, DP Beta 1	Protein Coding	GC06P058347	6.81320715
BRCA1	BRCA1 DNA Repair Associated	Protein Coding	GC17M043044	6.769587517
PECAM1	Platelet And Endothelial Cell Adhesion Molecule 1	Protein Coding	GC17M064319	6.765677452
TGM2	Transglutaminase 2	Protein Coding	GC20M038127	6.756747723
HNF4A	Hepatocyte Nuclear Factor 4 Alpha	Protein Coding	GC20P044355	6.741879463
IFNGR1	Interferon Gamma Receptor 1	Protein Coding	GC06M137197	6.73415184
GHRL	Ghrelin And Obestatin Prepropeptide	Protein Coding	GC03M010285	6.733631134
ALOX5	Arachidonate 5-Lipoxygenase	Protein Coding	GC10P045374	6.733430862
PIK3R1	Phosphoinositide-3-Kinase Regulatory Subunit 1	Protein Coding	GC05P068215	6.72923708
TAPBP	TAP Binding Protein	Protein Coding	GC06M033299	6.69504118
IGF1	Insulin Like Growth Factor 1	Protein Coding	GC12M102395	6.682122707
AURKA	Aurora Kinase A	Protein Coding	GC20M056370	6.652181625
IKZF1	IKAROS Family Zinc Finger 1	Protein Coding	GC07P050303	6.622612476
PMS2	PMS1 Homolog 2, Mismatch Repair System Component	Protein Coding	GC07M005973	6.613560677
IBD6	Inflammatory Bowel Disease 6	Genetic Locus	GC19U990206	6.594575882
MLH3	MutL Homolog 3	Protein Coding	GC14M075013	6.593871117
BLM	BLM RecQ Like Helicase	Protein Coding	GC15P090717	6.574238777
NR1I2	Nuclear Receptor Subfamily 1 Group I Member 2	Protein Coding	GC03P119780	6.569467545
SRC	SRC Proto-Oncogene, Non-Receptor Tyrosine Kinase	Protein Coding	GC20P037344	6.561339378
GSTM1	Glutathione S-Transferase Mu 1	Protein Coding	GC01P109687	6.553784847
SH2D1A	SH2 Domain Containing 1A	Protein Coding	GC0XP124227	6.553699493
BRINP3	BMP/Retinoic Acid Inducible Neural Specific 3	Protein Coding	GC01M190067	6.548114777
IL27	Interleukin 27	Protein Coding	GC16M028517	6.547070503
CDX2	Caudal Type Homeobox 2	Protein Coding	GC13M027962	6.539418221
RAB7A	RAB7A, Member RAS Oncogene	Protein Coding	GC03P131753	6.531345844
PRKCD	Protein Kinase C Delta	Protein Coding	GC03P053156	6.530422211
CHUK	Component Of Inhibitor Of Nuclear Factor Kappa B Kinase Complex	Protein Coding	GC10M100188	6.50288868
AQP8	Aquaporin 8	Protein Coding	GC16P026880	6.496910095
CRHR2	Corticotropin Releasing Hormone Receptor 2	Protein Coding	GC07M030651	6.495278358
GSTP1	Glutathione S-Transferase Pi 1	Protein Coding	GC11P067583	6.459961891
DCLRE1C	DNA Cross-Link Repair 1C	Protein Coding	GC10M014897	6.457407475
BCL2	BCL2 Apoptosis Regulator	Protein Coding	GC18M063123	6.455981255
TREX1	Three Prime Repair Exonuclease 1	Protein Coding	GC03P048466	6.43630743
IL19	Interleukin 19	Protein Coding	GC01P206770	6.43164444
STAT6	Signal Transducer And Activator Of Transcription 6	Protein Coding	GC12M057095	6.43059206
SLC26A3	Solute Carrier Family 26 Member 3	Protein Coding	GC07M107765	6.425840855
MIR140	MicroRNA 140	RNA Gene	GC16P069934	6.3942132

ODC1	Ornithine Decarboxylase 1	Protein Coding	GC02M010432	6.383761406
WAS	WASP Actin Nucleation Promoting Factor	Protein Coding	GC0XP048676	6.383522034
DEFB1	Defensin Beta 1	Protein Coding	GC08M006870	6.364779472
STAT5B	Signal Transducer And Activator Of Transcription 5B	Protein Coding	GC17M042199	6.364320278
NRAS	NRAS Proto-Oncogene, GTPase	Protein Coding	GC01M114704	6.323966026
MT-CYB	Mitochondrially Encoded Cytochrome	Protein Coding	GCMTP014749	6.320727348
CD86	CD86 Molecule	Protein Coding	GC03P122055	6.314284325
PDGFRL	Platelet Derived Growth Factor Receptor Like	Protein Coding	GC08P017576	6.306474209
DMBT1	Deleted In Malignant Brain Tumors 1	Protein Coding	GC10P122560	6.29813385
NR1H4	Nuclear Receptor Subfamily 1 Group H Member 4	Protein Coding	GC12P100473	6.289614677
HSPA4	Heat Shock Protein Family A (Hsp70) Member 4	Protein Coding	GC05P133051	6.273174286
MYC	MYC Proto-Oncogene, BHLH Transcription Factor	Protein Coding	GC08P127735	6.271646976
MUC6	Mucin 6, Oligomeric Mucus/Gel-	Protein Coding	GC11M001012	6.269100189
F3	Coagulation Factor III, Tissue Factor	Protein Coding	GC01M094530	6.263585567
BRCA2	BRCA2 DNA Repair Associated	Protein Coding	GC13P032315	6.260746002
SELP	Selectin P	Protein Coding	GC01M169558	6.255179405
TOR1A	Torsin Family 1 Member A	Protein Coding	GC09M129812	6.2354002
MIR127	MicroRNA 127	RNA Gene	GC14P106464	6.227228642
GREM1	Gremlin 1, DAN Family BMP	Protein Coding	GC15P033371	6.225271225
SOD2	Superoxide Dismutase 2	Protein Coding	GC06M159669	6.224704742
MKI67	Marker Of Proliferation Ki-67	Protein Coding	GC10M128096	6.222579002
COL7A1	Collagen Type VII Alpha 1 Chain	Protein Coding	GC03M048564	6.221595764
RNF186	Ring Finger Protein 186	Protein Coding	GC01M019814	6.200605392
PRF1	Perforin 1	Protein Coding	GC10M070597	6.193634033
PRSS1	Serine Protease 1	Protein Coding	GC07P146073	6.188678741
ARID1B	AT-Rich Interaction Domain 1B	Protein Coding	GC06P156777	6.188150883
HMGB1	High Mobility Group Box 1	Protein Coding	GC13M030456	6.18522644
MBL2	Mannose Binding Lectin 2	Protein Coding	GC10M052760	6.172304153
IL12RB2	Interleukin 12 Receptor Subunit Beta 2	Protein Coding	GC01P067307	6.152909756
CXCR1	C-X-C Motif Chemokine Receptor 1	Protein Coding	GC02M218162	6.152275562
JAK1	Janus Kinase 1	Protein Coding	GC01M064833	6.148966789
ITGAX	Integrin Subunit Alpha X	Protein Coding	GC16P033540	6.136202812
MYO1B	Myosin IB	Protein Coding	GC02P191246	6.134639263
IBD9	Inflammatory Bowel Disease 9	Genetic Locus	GC03U900016	6.11601162
IBD4	Inflammatory Bowel Disease 4	Genetic Locus	GC14U990396	6.11601162
SELL	Selectin L	Protein Coding	GC01M169690	6.109817505
SKIV2L	Ski2 Like RNA Helicase	Protein Coding	GC06P058332	6.092332363
IL18RAP	Interleukin 18 Receptor Accessory Protein	Protein Coding	GC02P102418	6.09213829
MIR192	MicroRNA 192	RNA Gene	GC11M064891	6.08631134
MIR203A	MicroRNA 203a	RNA Gene	GC14P106542	6.080626488
INS-IGF2	INS-IGF2 Readthrough	Protein Coding	GC11M002227	6.072246552
HPS3	HPS3 Biogenesis Of Lysosomal Organelles Complex 2 Subunit 1	Protein Coding	GC03P149129	6.071705818
PLAU	Plasminogen Activator, Urokinase	Protein Coding	GC10P073909	6.055727005
KRT20	Keratin 20	Protein Coding	GC17M040875	6.050296783
CAV1	Caveolin 1	Protein Coding	GC07P116524	6.042789936
LEPR	Leptin Receptor	Protein Coding	GC01P065421	6.040823936
SOX9	SRY-Box Transcription Factor 9	Protein Coding	GC17P072121	6.034566879
STXBP2	Syntaxin Binding Protein 2	Protein Coding	GC19P007642	6.028534889
AGER	Advanced Glycosylation End-Product Specific Receptor	Protein Coding	GC06M032180	6.023378372
RFX5	Regulatory Factor X5	Protein Coding	GC01M151340	6.023333549
GUSB	Glucuronidase Beta	Protein Coding	GC07M065960	6.00185585
PTPN11	Protein Tyrosine Phosphatase Non-Receptor Type 11	Protein Coding	GC12P112418	5.999073029
INPP5D	Inositol Polyphosphate-5-Phosphatase	Protein Coding	GC02P233059	5.983467102

TMEFF2	Transmembrane Protein With EGF Like And Two Follistatin Like Domains 2	Protein Coding	GC02M191950	5.972218513
CTNNA1	Catenin Alpha 1	Protein Coding	GC05P138613	5.961451054
MUTYH	MutY DNA Glycosylase	Protein Coding	GC01M045329	5.952507496
OTUD3	OTU Deubiquitinase 3	Protein Coding	GC01P019881	5.94311142
SRP54	Signal Recognition Particle 54	Protein Coding	GC14P034981	5.93765974
IBD11	Inflammatory Bowel Disease 11	Genetic Locus	GC07U903153	5.928936958
IBD12	Inflammatory Bowel Disease 12	Genetic Locus	GC03U901153	5.928936958
IBD15	Inflammatory Bowel Disease-15	Genetic Locus	GC10U901075	5.928936958
IBD16	Inflammatory Bowel Disease-16	Genetic Locus	GC09U901202	5.928936958
IBD18	Inflammatory Bowel Disease-18	Genetic Locus	GC05U901053	5.928936958
IBD20	Inflammatory Bowel Disease-20	Genetic Locus	GC10U901067	5.928936958
IBD22	Inflammatory Bowel Disease-22	Genetic Locus	GC17U901173	5.928936958
IBD23	Inflammatory Bowel Disease-23	Genetic Locus	GC01U902305	5.928936958
IBD24	Inflammatory Bowel Disease-24	Genetic Locus	GC20U900442	5.928936958
IBD26	Inflammatory Bowel Disease-26	Genetic Locus	GC12U901067	5.928936958
IBD27	Inflammatory Bowel Disease-27	Genetic Locus	GC13U900664	5.928936958
AQP5	Aquaporin 5	Protein Coding	GC12P049961	5.90793705
TGFA	Transforming Growth Factor Alpha	Protein Coding	GC02M070447	5.907927513
PTPN12	Protein Tyrosine Phosphatase Non-Receptor Type 12	Protein Coding	GC07P077537	5.861460686
TRAF3IP2	TRAF3 Interacting Protein 2	Protein Coding	GC06M111555	5.848082542
STAT5A	Signal Transducer And Activator Of Transcription 5A	Protein Coding	GC17P042287	5.845172405
LEP	Leptin	Protein Coding	GC07P128241	5.825382233
DCC	DCC Netrin 1 Receptor	Protein Coding	GC18P052340	5.82449007
CYP3A5	Cytochrome P450 Family 3 Subfamily A Member 5	Protein Coding	GC07M099648	5.808550835
IDO1	Indoleamine 2,3-Dioxygenase 1	Protein Coding	GC08P039891	5.780506134
LIFR	LIF Receptor Subunit Alpha	Protein Coding	GC05M038475	5.780373573
CYBA	Cytochrome B-245 Alpha Chain	Protein Coding	GC16M088643	5.759849548
BGLAP	Bone Gamma-Carboxyglutamate	Protein Coding	GC01P156242	5.754108429
MIRLET7A1	MicroRNA Let-7a-1	RNA Gene	GC09P094175	5.752267838
ADA	Adenosine Deaminase	Protein Coding	GC20M044620	5.749444962
THBD	Thrombomodulin	Protein Coding	GC20M023026	5.729369164
HLA-DPA1	Major Histocompatibility Complex, Class II, DP Alpha 1	Protein Coding	GC06M033064	5.726010323
CHI3L1	Chitinase 3 Like 1	Protein Coding	GC01M203148	5.719378471
CLEC7A	C-Type Lectin Domain Containing 7A	Protein Coding	GC12M016310	5.718157291
ERCC6	ERCC Excision Repair 6, Chromatin Remodeling Factor	Protein Coding	GC10M049454	5.688949585
MMP7	Matrix Metallopeptidase 7	Protein Coding	GC11M102425	5.688003063
LGR5	Leucine Rich Repeat Containing G Protein-Coupled Receptor 5	Protein Coding	GC12P071439	5.682612419
LMNA	Lamin A/C	Protein Coding	GC01P156082	5.679790974
MIR26A1	MicroRNA 26a-1	RNA Gene	GC03P037969	5.678523064
IRF1	Interferon Regulatory Factor 1	Protein Coding	GC05M132440	5.675714493
TNFRSF6B	TNF Receptor Superfamily Member 6b	Protein Coding	GC20P063696	5.66686058
F2RL1	F2R Like Trypsin Receptor 1	Protein Coding	GC05P076818	5.647530556
IL17RA	Interleukin 17 Receptor A	Protein Coding	GC22P017133	5.64624691
CDC42	Cell Division Cycle 42	Protein Coding	GC01P022134	5.641329288
LOC110806262	Solute Carrier Family 6 Member 4 Gene Promoter	Biological Region	GC17P030235	5.628918648
ORMDL3	ORMDL Sphingolipid Biosynthesis Regulator 3	Protein Coding	GC17M039921	5.623872757
MIR146A	MicroRNA 146a	RNA Gene	GC05P160485	5.617111206
MUC3A	Mucin 3A, Cell Surface Associated	Protein Coding	GC07P100949	5.610493183
SERPINA1	Serpin Family A Member 1	Protein Coding	GC14M094376	5.604316711
AP3B1	Adaptor Related Protein Complex 3 Subunit Beta 1	Protein Coding	GC05M078000	5.595770359
MIR4284	MicroRNA 4284	RNA Gene	GC07P073711	5.579289436
CCR9	C-C Motif Chemokine Receptor 9	Protein Coding	GC03P045903	5.567486763

IRAK1	Interleukin 1 Receptor Associated Kinase 1	Protein Coding	GC0XM154010	5.540263653
ATRIP	ATR Interacting Protein	Protein Coding	GC03P048449	5.526231766
IFNA1	Interferon Alpha 1	Protein Coding	GC09P021532	5.518873215
RBFOX1	RNA Binding Fox-1 Homolog 1	Protein Coding	GC16P006296	5.506595612
IL6R	Interleukin 6 Receptor	Protein Coding	GC01P154405	5.504656792
RUNX3	RUNX Family Transcription Factor 3	Protein Coding	GC01M024899	5.503659248
CXCL12	C-X-C Motif Chemokine Ligand 12	Protein Coding	GC10M044294	5.486069679
SLC9A3	Solute Carrier Family 9 Member A3	Protein Coding	GC05M000472	5.471357822
BUB1	BUB1 Mitotic Checkpoint Serine/Threonine Kinase	Protein Coding	GC02M110637	5.467207432
BUB1B	BUB1 Mitotic Checkpoint Serine/Threonine Kinase B	Protein Coding	GC15P040161	5.467207432
HSPD1	Heat Shock Protein Family D (Hsp60) Member 1	Protein Coding	GC02M197486	5.46625042
JUN	Jun Proto-Oncogene, AP-1 Transcription Factor Subunit	Protein Coding	GC01M058780	5.464234829
NLRP12	NLR Family Pyrin Domain Containing	Protein Coding	GC19M053793	5.459561348
PLA2G2E	Phospholipase A2 Group IIE	Protein Coding	GC01M019920	5.450335503
DNMT1	DNA Methyltransferase 1	Protein Coding	GC19M010133	5.439753056
TET2	Tet Methylcytosine Dioxygenase 2	Protein Coding	GC04P105145	5.427830219
CYBB	Cytochrome B-245 Beta Chain	Protein Coding	GC0XP037780	5.41838932
GALNT12	Polypeptide N-Acetylgalactosaminyltransferase 12	Protein Coding	GC09P098807	5.396001816
TREM1	Triggering Receptor Expressed On Myeloid Cells 1	Protein Coding	GC06M041267	5.393668175
MUC1	Mucin 1, Cell Surface Associated	Protein Coding	GC01M155185	5.383597374
VWF	Von Willebrand Factor	Protein Coding	GC12M005917	5.372971535
MIR451A	MicroRNA 451a	RNA Gene	GC17M028861	5.341984749
MIR215	MicroRNA 215	RNA Gene	GC01M220117	5.341984749
IBD25	Inflammatory Bowel Disease-25	Genetic Locus	GC00U932269	5.341984749
BCL2L1	BCL2 Like 1	Protein Coding	GC20M031664	5.340324402
NFE2L2	Nuclear Factor, Erythroid 2 Like 2	Protein Coding	GC02M177227	5.33805275
IFIH1	Interferon Induced With Helicase C Domain 1	Protein Coding	GC02M162267	5.326368809
POMC	Proopiomelanocortin	Protein Coding	GC02M025160	5.310775757
FBXW7	F-Box And WD Repeat Domain Containing 7	Protein Coding	GC04M152321	5.288543701
MMP13	Matrix Metallopeptidase 13	Protein Coding	GC11M102942	5.287534714
CCNY	Cyclin Y	Protein Coding	GC10P035254	5.283667564
IRF8	Interferon Regulatory Factor 8	Protein Coding	GC16P085898	5.280574322
DMD	Dystrophin	Protein Coding	GC0XM031097	5.280066013
DEFB103B	Defensin Beta 103B	Protein Coding	GC08M007430	5.26854372
HLA-E	Major Histocompatibility Complex, Class I, E	Protein Coding	GC06P058281	5.266252518
ESR1	Estrogen Receptor 1	Protein Coding	GC06P151656	5.263726711
MT-ND4L	Mitochondrially Encoded NADH:Ubiquinone Oxidoreductase Core Subunit 4L	Protein Coding	GCMTP010472	5.260036469
HRAS	HRas Proto-Oncogene, GTPase	Protein Coding	GC11M001303	5.256195068
NTS	Neurotensin	Protein Coding	GC12P085876	5.246242046
NQO1	NAD(P)H Quinone Dehydrogenase 1	Protein Coding	GC16M069706	5.239370823
MIR34C	MicroRNA 34c	RNA Gene	GC11P111591	5.230867386
GJB6	Gap Junction Protein Beta 6	Protein Coding	GC13M020221	5.228308678
ICAM3	Intercellular Adhesion Molecule 3	Protein Coding	GC19M010337	5.226905823
PUS10	Pseudouridine Synthase 10	Protein Coding	GC02M060940	5.223807335
CD27	CD27 Molecule	Protein Coding	GC12P013138	5.22038269
GBA	Glucosylceramidase Beta	Protein Coding	GC01M155234	5.210807323
GSDMB	Gasdermin B	Protein Coding	GC17M039904	5.204782486
CXCR3	C-X-C Motif Chemokine Receptor 3	Protein Coding	GC0XM071615	5.202939987
IFNA2	Interferon Alpha 2	Protein Coding	GC09M021384	5.202699184
MT-CO2	Mitochondrially Encoded Cytochrome C Oxidase II	Protein Coding	GCMTP007587	5.201128006

HLA-DRB9	Major Histocompatibility Complex, Class II, DR Beta 9 (Pseudogene)	Pseudogene	GC06M049161	5.191112518
CLDN8	Claudin 8	Protein Coding	GC21M030214	5.185097218
GPX1	Glutathione Peroxidase 1	Protein Coding	GC03M049537	5.17061615
GSTT1	Glutathione S-Transferase Theta 1	Protein Coding	GC22Mi00270	5.156296253
HLA-G	Major Histocompatibility Complex, Class I, G	Protein Coding	GC06P058239	5.155706882
FCGR2B	Fc Fragment Of IgG Receptor IIb	Protein Coding	GC01P161663	5.153928757
NOTCH1	Notch Receptor 1	Protein Coding	GC09M136832	5.130631447
BRIP1	BRCA1 Interacting Helicase 1	Protein Coding	GC17M061679	5.126017094
TCERG1	Transcription Elongation Regulator 1	Protein Coding	GC05P146447	5.126017094
CYP1A1	Cytochrome P450 Family 1 Subfamily A Member 1	Protein Coding	GC15M074719	5.120201588
HSPA2	Heat Shock Protein Family A (Hsp70) Member 2	Protein Coding	GC14P064535	5.118468761
IL3	Interleukin 3	Protein Coding	GC05P132060	5.111611843
C4B	Complement C4B (Chido Blood	Protein Coding	GC06P032014	5.098910332
ABCG2	ATP Binding Cassette Subfamily G Member 2 (Junior Blood Group)	Protein Coding	GC04M088090	5.09278965
ITGB1	Integrin Subunit Beta 1	Protein Coding	GC10M032892	5.084383011
CCKBR	Cholecystokinin B Receptor	Protein Coding	GC11P006259	5.081203461
NR3C1	Nuclear Receptor Subfamily 3 Group C Member 1	Protein Coding	GC05M143277	5.041526794
IL6ST	Interleukin 6 Cytokine Family Signal Transducer	Protein Coding	GC05M055935	5.035651684
KIT	KIT Proto-Oncogene, Receptor Tyrosine Kinase	Protein Coding	GC04P054657	5.033737183
GNAS	GNAS Complex Locus	Protein Coding	GC20P058839	5.029192448
KIF21B	Kinesin Family Member 21B	Protein Coding	GC01M200938	5.027646065
PON1	Paraoxonase 1	Protein Coding	GC07M095297	5.02449894
MTR	5-Methyltetrahydrofolate-Homocysteine Methyltransferase	Protein Coding	GC01P236795	5.022118568
FOS	Fos Proto-Oncogene, AP-1 Transcription Factor Subunit	Protein Coding	GC14P075278	5.010425568
MYB	MYB Proto-Oncogene, Transcription Factor	Protein Coding	GC06P135180	5.010133743
ECM1	Extracellular Matrix Protein 1	Protein Coding	GC01P150508	5.005101681
CD44	CD44 Molecule (Indian Blood Group)	Protein Coding	GC11P035139	5.000553131
PDGFRB	Platelet Derived Growth Factor Receptor Beta	Protein Coding	GC05M150113	5.000266075
CSF1	Colony Stimulating Factor 1	Protein Coding	GC01P109911	4.99961853
RFXANK	Regulatory Factor X Associated Ankyrin Containing Protein	Protein Coding	GC19P019192	4.992415905
CXCL9	C-X-C Motif Chemokine Ligand 9	Protein Coding	GC04M076001	4.978755951
MIR29A	MicroRNA 29a	RNA Gene	GC07M130876	4.958843708
CD46	CD46 Molecule	Protein Coding	GC01P207752	4.951176643
LRRK2	Leucine Rich Repeat Kinase 2	Protein Coding	GC12P040196	4.950107574
INSL6	Insulin Like 6	Protein Coding	GC09M004991	4.942044258
JAK3	Janus Kinase 3	Protein Coding	GC19M017824	4.94011116
EDN1	Endothelin 1	Protein Coding	GC06P012256	4.928060055
LAMC2	Laminin Subunit Gamma 2	Protein Coding	GC01P183186	4.924345016
SATB2	SATB Homeobox 2	Protein Coding	GC02M199269	4.90970993
AHR	Aryl Hydrocarbon Receptor	Protein Coding	GC07P016916	4.906763554
RFXAP	Regulatory Factor X Associated Protein	Protein Coding	GC13P036819	4.898230076
H2AC18	H2A Clustered Histone 18	Protein Coding	GC01M150202	4.887226582
MIR196B	MicroRNA 196b	RNA Gene	GC07M027366	4.882732391
NLRP6	NLR Family Pyrin Domain Containing	Protein Coding	GC11P000269	4.863068104
IL4R	Interleukin 4 Receptor	Protein Coding	GC16P027325	4.857217789
CX3CR1	C-X3-C Motif Chemokine Receptor 1	Protein Coding	GC03M039279	4.852750778
MAPK14	Mitogen-Activated Protein Kinase 14	Protein Coding	GC06P058451	4.84853363
FFAR2	Free Fatty Acid Receptor 2	Protein Coding	GC19P041415	4.843500137
PTPRJ	Protein Tyrosine Phosphatase Receptor Type J	Protein Coding	GC11P048002	4.840956688

DLC1	DLC1 Rho GTPase Activating Protein	Protein Coding	GC08M013083	4.840956688
FZD3	Frizzled Class Receptor 3	Protein Coding	GC08P028494	4.840956688
RAD54B	RAD54 Homolog B	Protein Coding	GC08M094371	4.840956688
FLCN	Folliculin	Protein Coding	GC17M017206	4.840956688
PMS1	PMS1 Homolog 1, Mismatch Repair System Component	Protein Coding	GC02P189784	4.840956688
MCC	MCC Regulator Of WNT Signaling Pathway	Protein Coding	GC05M113022	4.840956688
SLC9A9	Solute Carrier Family 9 Member A9	Protein Coding	GC03M143265	4.840956688
AMER1	APC Membrane Recruitment Protein 1	Protein Coding	GC0XM064185	4.840956688
PKHD1	PKHD1 Ciliary IPT Domain Containing Fibrocystin/Polyductin	Protein Coding	GC06M051588	4.840956688
MT-ND1	Mitochondrially Encoded NADH:Ubiquinone Oxidoreductase Core Subunit 1	Protein Coding	GCMTP003309	4.840956688
C11orf65	Chromosome 11 Open Reading Frame	Protein Coding	GC11M108308	4.840956688
MIR34B	MicroRNA 34b	RNA Gene	GC11P111546	4.840956688
MIR100	MicroRNA 100	RNA Gene	GC11M122152	4.840956688
MIR342	MicroRNA 342	RNA Gene	GC14P100109	4.840956688
CRCS11	Colorectal Cancer, Susceptibility To,	Genetic Locus	GC20U900444	4.840956688
CRCS2	Colorectal Cancer, Susceptibility To, 2	Genetic Locus	GC08U901124	4.840956688
CRCS5	Colorectal Cancer, Susceptibility To, 5	Genetic Locus	GC10U901096	4.840956688
CRCS6	Colorectal Cancer, Susceptibility To, 6	Genetic Locus	GC08U901098	4.840956688
CRCS7	Colorectal Cancer, Susceptibility To, 7	Genetic Locus	GC11U901214	4.840956688
CRCS8	Colorectal Cancer, Susceptibility To, 8	Genetic Locus	GC14U900916	4.840956688
CRCS9	Colorectal Cancer, Susceptibility To, 9	Genetic Locus	GC16U901133	4.840956688
HDC	Histidine Decarboxylase	Protein Coding	GC15M050241	4.833342552
SIAE	Sialic Acid Acetylesterase	Protein Coding	GC11M124633	4.80668354
IGF1R	Insulin Like Growth Factor 1 Receptor	Protein Coding	GC15P098648	4.802274227
F13A1	Coagulation Factor XIII A Chain	Protein Coding	GC06M006144	4.788828373
BSN	Bassoon Presynaptic Cytomatrix	Protein Coding	GC03P049554	4.773397446
SMAD2	SMAD Family Member 2	Protein Coding	GC18M047809	4.754115105
RHOA	Ras Homolog Family Member A	Protein Coding	GC03M049359	4.750461102
TBX21	T-Box Transcription Factor 21	Protein Coding	GC17P047733	4.743451595
XRCC1	X-Ray Repair Cross Complementing 1	Protein Coding	GC19M043543	4.736963272
TRAF3IP2-AS1	TRAF3IP2 Antisense RNA 1	RNA Gene	GC06P111483	4.732922077
C3	Complement C3	Protein Coding	GC19M006677	4.730078697
DPP4	Dipeptidyl Peptidase 4	Protein Coding	GC02M161992	4.723836899
PDGFRA	Platelet Derived Growth Factor Receptor Alpha	Protein Coding	GC04P054229	4.722854137
NAT1	N-Acetyltransferase 1	Protein Coding	GC08P018179	4.720440388
TIMP2	TIMP Metallopeptidase Inhibitor 2	Protein Coding	GC17M078852	4.716907024
RAC1	Rac Family Small GTPase 1	Protein Coding	GC07P006377	4.698544502
CD209	CD209 Molecule	Protein Coding	GC19M007739	4.688280106
DPYD	Dihydropyrimidine Dehydrogenase	Protein Coding	GC01M097015	4.686062813
MIR155	MicroRNA 155	RNA Gene	GC21P025573	4.684669495
CCL7	C-C Motif Chemokine Ligand 7	Protein Coding	GC17P034270	4.67009449
HAVCR2	Hepatitis A Virus Cellular Receptor 2	Protein Coding	GC05M157063	4.661985397
ETS1	ETS Proto-Oncogene 1, Transcription Factor	Protein Coding	GC11M128458	4.651488304
PARP1	Poly(ADP-Ribose) Polymerase 1	Protein Coding	GC01M226360	4.651191235
PIK3CD	Phosphatidylinositol-4,5-Bisphosphate 3-Kinase Catalytic Subunit Delta	Protein Coding	GC01P009629	4.649760723
KIAA1109	KIAA1109	Protein Coding	GC04P122152	4.620446205
NLRP1	NLR Family Pyrin Domain Containing	Protein Coding	GC17M005499	4.61490345
MAPK3	Mitogen-Activated Protein Kinase 3	Protein Coding	GC16M031685	4.602758408
ADIPOQ	Adiponectin, C1Q And Collagen Domain Containing	Protein Coding	GC03P186842	4.600897312
KLRK1	Killer Cell Lectin Like Receptor K1	Protein Coding	GC12M016312	4.596976757
B2M	Beta-2-Microglobulin	Protein Coding	GC15P044711	4.590127945
CEP43	Centrosomal Protein 43	Protein Coding	GC06P167001	4.585112572
DLD	Dihydrolipoamide Dehydrogenase	Protein Coding	GC07P107890	4.584471703

IL17REL	Interleukin 17 Receptor E Like	Protein Coding	GC22M050169	4.575429916
TTC7A	Tetratricopeptide Repeat Domain 7A	Protein Coding	GC02P046906	4.564817429
SLIT2	Slit Guidance Ligand 2	Protein Coding	GC04P020287	4.5631423
IL2RB	Interleukin 2 Receptor Subunit Beta	Protein Coding	GC22M037125	4.549527645
CXCR4	C-X-C Motif Chemokine Receptor 4	Protein Coding	GC02M136114	4.541909218
CASR	Calcium Sensing Receptor	Protein Coding	GC03P122183	4.528421879
G6PC3	Glucose-6-Phosphatase Catalytic Subunit 3	Protein Coding	GC17P044070	4.524396896
EBI3	Epstein-Barr Virus Induced 3	Protein Coding	GC19P004233	4.5169487
NFATC1	Nuclear Factor Of Activated T Cells 1	Protein Coding	GC18P079395	4.516537666
HLA-DRB5	Major Histocompatibility Complex, Class II, DR Beta 5	Protein Coding	GC06M049163	4.507162094
EPO	Erythropoietin	Protein Coding	GC07P100720	4.504007816
GCG	Glucagon	Protein Coding	GC02M162142	4.503176689
TMSB4X	Thymosin Beta 4 X-Linked	Protein Coding	GC0XP012975	4.49353075
PLAUR	Plasminogen Activator, Urokinase Receptor	Protein Coding	GC19M043646	4.491315365
NOS3	Nitric Oxide Synthase 3	Protein Coding	GC07P150990	4.477231979
CASP3	Caspase 3	Protein Coding	GC04M184627	4.468459129
ANTXR2	ANTXR Cell Adhesion Molecule 2	Protein Coding	GC04M079901	4.466005325
LBR	Lamin B Receptor	Protein Coding	GC01M225401	4.462207794
MYD88	MYD88 Innate Immune Signal Transduction Adaptor	Protein Coding	GC03P038139	4.44951725
ENTPD1	Ectonucleoside Triphosphate Diphosphohydrolase 1	Protein Coding	GC10P095711	4.435255527
ITGAE	Integrin Subunit Alpha E	Protein Coding	GC17M003722	4.434802055
PSMB9	Proteasome 20S Subunit Beta 9	Protein Coding	GC06P058343	4.434258461
PTH	Parathyroid Hormone	Protein Coding	GC11M013492	4.431459427
NORAD	Non-Coding RNA Activated By DNA Damage	RNA Gene	GC20M036116	4.422253609
NOX1	NADPH Oxidase 1	Protein Coding	GC0XM100843	4.421844482
WNT4	Wnt Family Member 4	Protein Coding	GC01M022190	4.407792568
ERCC1	ERCC Excision Repair 1, Endonuclease Non-Catalytic Subunit	Protein Coding	GC19M048721	4.405452728
SLC44A4	Solute Carrier Family 44 Member 4	Protein Coding	GC06M031863	4.404116631
CCL26	C-C Motif Chemokine Ligand 26	Protein Coding	GC07M075769	4.394666195
MIR31	MicroRNA 31	RNA Gene	GC09M021520	4.38805151
MMP8	Matrix Metallopeptidase 8	Protein Coding	GC11M102617	4.367744446
MGMT	O-6-Methylguanine-DNA Methyltransferase	Protein Coding	GC10P129467	4.366889
IL18R1	Interleukin 18 Receptor 1	Protein Coding	GC02P102311	4.360421181
BIRC3	Baculoviral IAP Repeat Containing 3	Protein Coding	GC11P102317	4.357974529
APOE	Apolipoprotein E	Protein Coding	GC19P044906	4.354453564
GZMB	Granzyme B	Protein Coding	GC14M024630	4.344996452
IKBKG	Inhibitor Of Nuclear Factor Kappa B Kinase Regulatory Subunit Gamma	Protein Coding	GC0XP154541	4.34484005
CDKAL1	CDK5 Regulatory Subunit Associated Protein 1 Like 1	Protein Coding	GC06P020534	4.332320213
LINC01475	Long Intergenic Non-Protein Coding RNA 1475	RNA Gene	GC10M099526	4.329584122
TNFSF10	TNF Superfamily Member 10	Protein Coding	GC03M172505	4.327876568
STING1	Stimulator Of Interferon Response CGAMP Interactor 1	Protein Coding	GC05M139476	4.30860281
ZPBP2	Zona Pellucida Binding Protein 2	Protein Coding	GC17P039869	4.300489426
CYP2E1	Cytochrome P450 Family 2 Subfamily E Member 1	Protein Coding	GC10P133520	4.279425144
DOCK8	Dedicator Of Cytokinesis 8	Protein Coding	GC09P000214	4.268322945
FFAR3	Free Fatty Acid Receptor 3	Protein Coding	GC19P041776	4.266131401
GPT	Glutamic--Pyruvic Transaminase	Protein Coding	GC08P144502	4.263992786
CASP8	Caspase 8	Protein Coding	GC02P201233	4.262453556
MIR24-1	MicroRNA 24-1	RNA Gene	GC09P095086	4.260861397
PRKN	Parkin RBR E3 Ubiquitin Protein	Protein Coding	GC06M161348	4.260037899

CYP1B1	Cytochrome P450 Family 1 Subfamily B Member 1	Protein Coding	GC02M038066	4.243651867
MTRR	5-Methyltetrahydrofolate-Homocysteine Methyltransferase	Protein Coding	GC05P007851	4.242434978
HERC2	HECT And RLD Domain Containing E3 Ubiquitin Protein Ligase 2	Protein Coding	GC15M028111	4.238735199
AMACR	Alpha-Methylacyl-CoA Racemase	Protein Coding	GC05M033986	4.229970932
MIR106B	MicroRNA 106b	RNA Gene	GC07M100605	4.226085186
IL1RL1	Interleukin 1 Receptor Like 1	Protein Coding	GC02P102294	4.221509457
RNASEH2C	Ribonuclease H2 Subunit C	Protein Coding	GC11M065714	4.207246304
LYZ	Lysozyme	Protein Coding	GC12P069348	4.203668118
MIR142	MicroRNA 142	RNA Gene	GC17M058331	4.196019173
ITLN1	Intelectin 1	Protein Coding	GC01M160876	4.190085411
COL14A1	Collagen Type XIV Alpha 1 Chain	Protein Coding	GC08P120073	4.184928894
IL9	Interleukin 9	Protein Coding	GC05M135891	4.180634499
BMP6	Bone Morphogenetic Protein 6	Protein Coding	GC06P007726	4.180584908
KNG1	Kininogen 1	Protein Coding	GC03P186717	4.18017292
KDR	Kinase Insert Domain Receptor	Protein Coding	GC04M055078	4.178035736
FMNL2	Formin Like 2	Protein Coding	GC02P152335	4.173502922
KRT8	Keratin 8	Protein Coding	GC12M052897	4.16936636
TPI1	Triosephosphate Isomerase 1	Protein Coding	GC12P013180	4.160318375
ERGIC1	Endoplasmic Reticulum-Golgi Intermediate Compartment 1	Protein Coding	GC05P172834	4.152613163
TNFRSF14	TNF Receptor Superfamily Member 14	Protein Coding	GC01P002555	4.14734745
CLDN5	Claudin 5	Protein Coding	GC22M019523	4.147111893
MIR222	MicroRNA 222	RNA Gene	GC0XM045747	4.145535469
LGALS1	Galectin 1	Protein Coding	GC22P037675	4.124821663
GATA3	GATA Binding Protein 3	Protein Coding	GC10P008045	4.124360561
CFTR	CF Transmembrane Conductance Regulator	Protein Coding	GC07P117287	4.118645668
ENO1	Enolase 1	Protein Coding	GC01M008861	4.098435402
PSMB8	Proteasome 20S Subunit Beta 8	Protein Coding	GC06M032840	4.098086834
MIR642A	MicroRNA 642a	RNA Gene	GC19P045674	4.097336292
HSPA1B	Heat Shock Protein Family A (Hsp70) Member 1B	Protein Coding	GC06P058327	4.095950127
SERPINF2	Serpin Family F Member 2	Protein Coding	GC17P001742	4.092998505
PIGR	Polymeric Immunoglobulin Receptor	Protein Coding	GC01M206928	4.092729568
ARPC2	Actin Related Protein 2/3 Complex Subunit 2	Protein Coding	GC02P218217	4.088812351
CLDN3	Claudin 3	Protein Coding	GC07M073768	4.086014271
MIR214	MicroRNA 214	RNA Gene	GC01M172234	4.080337524
CD36	CD36 Molecule	Protein Coding	GC07P080369	4.080066204
IL1RL2	Interleukin 1 Receptor Like 2	Protein Coding	GC02P102186	4.074865818
CTSD	Cathepsin D	Protein Coding	GC11M001752	4.074107647
CD274	CD274 Molecule	Protein Coding	GC09P005450	4.073084831
BIRC5	Baculoviral IAP Repeat Containing 5	Protein Coding	GC17P078214	4.071007252
TTC37	Tetratricopeptide Repeat Domain 37	Protein Coding	GC05M095463	4.061399937
ADAD1	Adenosine Deaminase Domain Containing 1	Protein Coding	GC04P122378	4.057835579
GPX2	Glutathione Peroxidase 2	Protein Coding	GC14M064939	4.056686401
TNFRSF4	TNF Receptor Superfamily Member 4	Protein Coding	GC01M001211	4.056197166
MST1R	Macrophage Stimulating 1 Receptor	Protein Coding	GC03M050176	4.054501057
KIR3DL1	Killer Cell Immunoglobulin Like Receptor, Three Ig Domains And Long Cytoplasmic Tail 1	Protein Coding	GC19P056885	4.052411079
STX11	Syntaxin 11	Protein Coding	GC06P144150	4.049696922
AREG	Amphiregulin	Protein Coding	GC04P074445	4.045385838
HDAC9	Histone Deacetylase 9	Protein Coding	GC07P018086	4.043595791
NR1H2	Nuclear Receptor Subfamily 1 Group H Member 2	Protein Coding	GC19P050329	4.04178524
DEFA6	Defensin Alpha 6	Protein Coding	GC08M006924	4.038972855
NFKBIL1	NFKB Inhibitor Like 1	Protein Coding	GC06P058304	4.023340225
SCG2	Secretogranin II	Protein Coding	GC02M223596	4.02010107

TFRC	Transferrin Receptor	Protein Coding	GC03M196027	4.018126011
IGSF6	Immunoglobulin Superfamily Member	Protein Coding	GC16M021639	4.014704704
WASL	WASP Like Actin Nucleation Promoting Factor	Protein Coding	GC07M123681	4.009766102
CARD6	Caspase Recruitment Domain Family Member 6	Protein Coding	GC05P040841	4.001578331
CLIC1	Chloride Intracellular Channel 1	Protein Coding	GC06M049127	4.00094223
CLDN1	Claudin 1	Protein Coding	GC03M190305	3.995974064
SLC17A5	Solute Carrier Family 17 Member 5	Protein Coding	GC06M073593	3.989099503
NOTCH4	Notch Receptor 4	Protein Coding	GC06M049148	3.986665726
CCL4	C-C Motif Chemokine Ligand 4	Protein Coding	GC17P036103	3.982458591
CHGB	Chromogranin B	Protein Coding	GC20P005911	3.981480122
MIR598	MicroRNA 598	RNA Gene	GC08M011035	3.979260445
SMURF1	SMAD Specific E3 Ubiquitin Protein Ligase 1	Protein Coding	GC07M099027	3.975193739
PCNA	Proliferating Cell Nuclear Antigen	Protein Coding	GC20M005114	3.967512131
ADAM17	ADAM Metallopeptidase Domain 17	Protein Coding	GC02M009488	3.96628952
GAPDH	Glyceraldehyde-3-Phosphate Dehydrogenase	Protein Coding	GC12P013162	3.964569092
NCAM1	Neural Cell Adhesion Molecule 1	Protein Coding	GC11P112961	3.952435017
HPGD	15-Hydroxyprostaglandin	Protein Coding	GC04M174490	3.951249599
PDE4A	Phosphodiesterase 4A	Protein Coding	GC19P010416	3.940480471
GPBAR1	G Protein-Coupled Bile Acid Receptor	Protein Coding	GC02P218259	3.934834242
DPF2	Double PHD Fingers 2	Protein Coding	GC11P065356	3.933744669
CD163	CD163 Molecule	Protein Coding	GC12M007471	3.929499626
RORC	RAR Related Orphan Receptor C	Protein Coding	GC01M151806	3.923522949
IL1R2	Interleukin 1 Receptor Type 2	Protein Coding	GC02P101991	3.915358543
ARPC1B	Actin Related Protein 2/3 Complex Subunit 1B	Protein Coding	GC07P099374	3.914891005
KRT19	Keratin 19	Protein Coding	GC17M041523	3.912536621
HNMT	Histamine N-Methyltransferase	Protein Coding	GC02P137964	3.912345886
MIR193A	MicroRNA 193a	RNA Gene	GC17P031559	3.904427052
CD59	CD59 Molecule (CD59 Blood Group)	Protein Coding	GC11M033704	3.898500919
DNAH8	Dynein Axonemal Heavy Chain 8	Protein Coding	GC06P058478	3.897442102
TP73	Tumor Protein P73	Protein Coding	GC01P003652	3.895909071
FGF23	Fibroblast Growth Factor 23	Protein Coding	GC12M004368	3.885583639
MMP10	Matrix Metallopeptidase 10	Protein Coding	GC11M102770	3.883780956
CFI	Complement Factor I	Protein Coding	GC04M109732	3.879736423
JAZF1	JAZF Zinc Finger 1	Protein Coding	GC07M027830	3.876430988
BANK1	B Cell Scaffold Protein With Ankyrin Repeats 1	Protein Coding	GC04P101411	3.876430988
ICAM2	Intercellular Adhesion Molecule 2	Protein Coding	GC17M064002	3.873286247
LGALS9	Galectin 9	Protein Coding	GC17P027629	3.865354538
ABCC2	ATP Binding Cassette Subfamily C Member 2	Protein Coding	GC10P099782	3.859766483
LYST	Lysosomal Trafficking Regulator	Protein Coding	GC01M235661	3.853312492
PTX3	Pentraxin 3	Protein Coding	GC03P157436	3.851355076
CD69	CD69 Molecule	Protein Coding	GC12M016305	3.836389065
MCL1	MCL1 Apoptosis Regulator, BCL2 Family Member	Protein Coding	GC01M150561	3.827115059
RHOB	Ras Homolog Family Member B	Protein Coding	GC02P020447	3.825116396
ENG	Endoglin	Protein Coding	GC09M127815	3.821447372
MIR200C	MicroRNA 200c	RNA Gene	GC12P013192	3.81831789
TNFRSF9	TNF Receptor Superfamily Member 9	Protein Coding	GC01M007915	3.816335201
IKBKB	Inhibitor Of Nuclear Factor Kappa B Kinase Subunit Beta	Protein Coding	GC08P042271	3.812565088
CHAT	Choline O-Acetyltransferase	Protein Coding	GC10P049609	3.799603939
CRH	Corticotropin Releasing Hormone	Protein Coding	GC08M066176	3.79227829
LSP1	Lymphocyte Specific Protein 1	Protein Coding	GC11P001852	3.789489746
MIR200B	MicroRNA 200b	RNA Gene	GC01P001167	3.788418531
HBEGF	Heparin Binding EGF Like Growth	Protein Coding	GC05M140332	3.785998583
BVES	Blood Vessel Epicardial Substance	Protein Coding	GC06M105096	3.783994436
MIR223	MicroRNA 223	RNA Gene	GC0XP066018	3.782684565

KRT18	Keratin 18	Protein Coding	GC12P052948	3.782481194
CCL25	C-C Motif Chemokine Ligand 25	Protein Coding	GC19P008052	3.7761693
BLK	BLK Proto-Oncogene, Src Family Tyrosine Kinase	Protein Coding	GC08P011486	3.774923801
CDKN2B-	CDKN2B Antisense RNA 1	RNA Gene	GC09P021994	3.773068905
BTK	Bruton Tyrosine Kinase	Protein Coding	GC0XM101349	3.771018505
FERMT3	FERM Domain Containing Kindlin 3	Protein Coding	GC11P064207	3.769852161
HLA-T	Major Histocompatibility Complex, Class I, T (Pseudogene)	Pseudogene	GC06P058242	3.769605398
MTHFD1	Methylenetetrahydrofolate Dehydrogenase, Cyclohydrolase And Formyltetrahydrofolate Synthetase 1	Protein Coding	GC14P064388	3.768350124
CTSB	Cathepsin B	Protein Coding	GC08M011842	3.765686989
SP140	SP140 Nuclear Body Protein	Protein Coding	GC02P230203	3.763207912
MUC4	Mucin 4, Cell Surface Associated	Protein Coding	GC03M195746	3.7598629
PTGER3	Prostaglandin E Receptor 3	Protein Coding	GC01M070852	3.756435633
MMP14	Matrix Metallopeptidase 14	Protein Coding	GC14P026861	3.753677368
GOT1	Glutamic-Oxaloacetic Transaminase 1	Protein Coding	GC10M099396	3.74865818
IL18BP	Interleukin 18 Binding Protein	Protein Coding	GC11P071998	3.744641781
MIR3936	MicroRNA 3936	RNA Gene	GC05M132365	3.740290642
ERAP2	Endoplasmic Reticulum Aminopeptidase 2	Protein Coding	GC05P096875	3.739394188
CCHCR1	Coiled-Coil Alpha-Helical Rod Protein	Protein Coding	GC06M049064	3.729874134
HPRT1	Hypoxanthine Phosphoribosyltransferase 1	Protein Coding	GC0XP134460	3.72903204
TNIP1	TNFAIP3 Interacting Protein 1	Protein Coding	GC05M151029	3.725317955
BLOC1S6	Biogenesis Of Lysosomal Organelles Complex 1 Subunit 6	Protein Coding	GC15P045588	3.71663785
SLC16A1	Solute Carrier Family 16 Member 1	Protein Coding	GC01M112932	3.711354256
TEK	TEK Receptor Tyrosine Kinase	Protein Coding	GC09P027109	3.707580328
SLC15A1	Solute Carrier Family 15 Member 1	Protein Coding	GC13M098683	3.699439764
ESR2	Estrogen Receptor 2	Protein Coding	GC14M064084	3.689844608
KLF6	Kruppel Like Factor 6	Protein Coding	GC10M003779	3.687590599
FUT3	Fucosyltransferase 3 (Lewis Blood Group)	Protein Coding	GC19M005843	3.668585539
FOSL1	FOS Like 1, AP-1 Transcription Factor Subunit	Protein Coding	GC11M071734	3.661357641
MDM2	MDM2 Proto-Oncogene	Protein Coding	GC12P068808	3.660514832
GSR	Glutathione-Disulfide Reductase	Protein Coding	GC08M030678	3.65223217
ISG15	ISG15 Ubiquitin Like Modifier	Protein Coding	GC01P001001	3.640165806
CFLAR	CASP8 And FADD Like Apoptosis Regulator	Protein Coding	GC02P201117	3.633009195
CD34	CD34 Molecule	Protein Coding	GC01M207880	3.627595425
TNFSF4	TNF Superfamily Member 4	Protein Coding	GC01M173183	3.626401186
MIR150	MicroRNA 150	RNA Gene	GC19M049500	3.61554265
LITAF	Lipopolysaccharide Induced TNF	Protein Coding	GC16M011547	3.594087601
USP12	Ubiquitin Specific Peptidase 12	Protein Coding	GC13M027066	3.582978487
IL20	Interleukin 20	Protein Coding	GC01P206866	3.568258286
TPPP	Tubulin Polymerization Promoting	Protein Coding	GC05M000659	3.56778717
CEP72	Centrosomal Protein 72	Protein Coding	GC05P000612	3.56778717
FCGR2C	Fc Fragment Of IgG Receptor IIc (Gene/Pseudogene)	Protein Coding	GC01P161583	3.56778717
AIRE	Autoimmune Regulator	Protein Coding	GC21P044285	3.558382988
MALAT1	Metastasis Associated Lung Adenocarcinoma Transcript 1	RNA Gene	GC11P066818	3.552625656
PADI4	Peptidyl Arginine Deiminase 4	Protein Coding	GC01P017308	3.551231384
CYCS	Cytochrome C, Somatic	Protein Coding	GC07M025118	3.551021576
CRHR1	Corticotropin Releasing Hormone Receptor 1	Protein Coding	GC17P045784	3.550623894
BBC3	BCL2 Binding Component 3	Protein Coding	GC19M047220	3.54665947
TCF7L2	Transcription Factor 7 Like 2	Protein Coding	GC10P112950	3.541967392
H2AX	H2A.X Variant Histone	Protein Coding	GC11M119097	3.54167366
DES	Desmin	Protein Coding	GC02P219418	3.532038927

PRG2	Proteoglycan 2, Pro Eosinophil Major Basic Protein	Protein Coding	GC11M057386	3.524595737
TNFRSF10A	TNF Receptor Superfamily Member	Protein Coding	GC08M023190	3.521443605
PARK7	Parkinsonism Associated Deglycase	Protein Coding	GC01P008025	3.521297455
F2R	Coagulation Factor II Thrombin	Protein Coding	GC05P076716	3.514681578
MIR195	MicroRNA 195	RNA Gene	GC17M007018	3.513520956
SIRT1	Sirtuin 1	Protein Coding	GC10P067884	3.504477501
MIR122	MicroRNA 122	RNA Gene	GC18P058451	3.502309084
ICOS	Inducible T Cell Costimulator	Protein Coding	GC02P203937	3.494501352
GNA12	G Protein Subunit Alpha 12	Protein Coding	GC07M002728	3.493155479
DAP	Death Associated Protein	Protein Coding	GC05M010679	3.493155479
MIR221	MicroRNA 221	RNA Gene	GC0XM045746	3.492258549
FHIT	Fragile Histidine Triad Diadenosine Triphosphatase	Protein Coding	GC03M059747	3.479558945
TLR1	Toll Like Receptor 1	Protein Coding	GC04M038793	3.474606752
LGALS4	Galectin 4	Protein Coding	GC19M048410	3.464309692
SERPINA4	Serpin Family A Member 4	Protein Coding	GC14P094561	3.457278252
NRIP1	Nuclear Receptor Interacting Protein 1	Protein Coding	GC21M014961	3.448604107
ANGPT1	Angiopoietin 1	Protein Coding	GC08M107246	3.439809561
SLC40A1	Solute Carrier Family 40 Member 1	Protein Coding	GC02M189560	3.437915802
CCL24	C-C Motif Chemokine Ligand 24	Protein Coding	GC07M075818	3.429262638
BIRC2	Baculoviral IAP Repeat Containing 2	Protein Coding	GC11P102347	3.4225173
MIR424	MicroRNA 424	RNA Gene	GC0XM134660	3.418700695
IFNB1	Interferon Beta 1	Protein Coding	GC09M021077	3.416519165
APEX1	Apurinic/Apyrimidinic Endodeoxyribonuclease 1	Protein Coding	GC14P020455	3.413998365
HSPA6	Heat Shock Protein Family A (Hsp70) Member 6	Protein Coding	GC01P161524	3.413559198
ATF6	Activating Transcription Factor 6	Protein Coding	GC01P161766	3.410503387
PPIG	Peptidylprolyl Isomerase G	Protein Coding	GC02P169584	3.408787012
IGHE	Immunoglobulin Heavy Constant	Protein Coding	GC14M110575	3.402381182
APEH	Acylaminoacyl-Peptide Hydrolase	Protein Coding	GC03P049673	3.394553185
SMARCB1	SWI/SNF Related, Matrix Associated, Actin Dependent Regulator Of Chromatin, Subfamily B, Member 1	Protein Coding	GC22P023786	3.394541979
SLAMF1	Signaling Lymphocytic Activation Molecule Family Member 1	Protein Coding	GC01M160608	3.393943071
OR5V1	Olfactory Receptor Family 5 Subfamily V Member 1	Protein Coding	GC06M029353	3.393484592
TNFRSF18	TNF Receptor Superfamily Member 18	Protein Coding	GC01M001203	3.38670826
PTPRS	Protein Tyrosine Phosphatase Receptor Type S	Protein Coding	GC19M005157	3.385066032
CEACAM6	CEA Cell Adhesion Molecule 6	Protein Coding	GC19P041750	3.384496212
EZH2	Enhancer Of Zeste 2 Polycomb Repressive Complex 2 Subunit	Protein Coding	GC07M148807	3.383299112
FLT1	Fms Related Receptor Tyrosine Kinase	Protein Coding	GC13M028300	3.382499218
MIR483	MicroRNA 483	RNA Gene	GC11M002228	3.379379272
NUP210	Nucleoporin 210	Protein Coding	GC03M017371	3.378604889
SULT1A1	Sulfotransferase Family 1A Member 1	Protein Coding	GC16M028606	3.378484249
CFH	Complement Factor H	Protein Coding	GC01P196621	3.368458271
INSL4	Insulin Like 4	Protein Coding	GC09P005231	3.365678787
NLRC4	NLR Family CARD Domain	Protein Coding	GC02M032224	3.364046097
ANXA5	Annexin A5	Protein Coding	GC04M121667	3.361418724
C1S	Complement C1s	Protein Coding	GC12P013204	3.355348349
TLR8	Toll Like Receptor 8	Protein Coding	GC0XP012924	3.347032547
MAGI1	Membrane Associated Guanylate Kinase, WW And PDZ Domain	Protein Coding	GC03M065330	3.343193531
PTGES	Prostaglandin E Synthase	Protein Coding	GC09M129738	3.339421511
GPR12	G Protein-Coupled Receptor 12	Protein Coding	GC13M026755	3.335320711
PARD3	Par-3 Family Cell Polarity Regulator	Protein Coding	GC10M034110	3.319885254
COG6	Component Of Oligomeric Golgi Complex 6	Protein Coding	GC13P039655	3.30322361
TRAF6	TNF Receptor Associated Factor 6	Protein Coding	GC11M036467	3.302589893

MIR27A	MicroRNA 27a	RNA Gene	GC19M014149	3.301205635
ALDH2	Aldehyde Dehydrogenase 2 Family Member	Protein Coding	GC12P111766	3.299277067
MMP12	Matrix Metallopeptidase 12	Protein Coding	GC11M102862	3.297683954
IL12RB1	Interleukin 12 Receptor Subunit Beta 1	Protein Coding	GC19M018058	3.295893669
RB1	RB Transcriptional Corepressor 1	Protein Coding	GC13P048303	3.284836054
AOC1	Amine Oxidase Copper Containing 1	Protein Coding	GC07P150824	3.282246113
GATD3	Glutamine Amidotransferase Class 1 Domain Containing 3	Protein Coding	GC21P044135	3.278872967
SBNO2	Strawberry Notch Homolog 2	Protein Coding	GC19M001107	3.27572298
PTCH1	Patched 1	Protein Coding	GC09M095442	3.271292925
MB	Myoglobin	Protein Coding	GC22M035606	3.262176037
VIM	Vimentin	Protein Coding	GC10P017227	3.261744976
TDGF1	Teratocarcinoma-Derived Growth Factor 1	Protein Coding	GC03P046577	3.257343769
EGR1	Early Growth Response 1	Protein Coding	GC05P138465	3.253792763
GPSM3	G Protein Signaling Modulator 3	Protein Coding	GC06M049147	3.235784054
IFNGR2	Interferon Gamma Receptor 2	Protein Coding	GC21P033402	3.235389233
HSPA1A	Heat Shock Protein Family A (Hsp70) Member 1A	Protein Coding	GC06P058328	3.232810974
CAMP	Cathelicidin Antimicrobial Peptide	Protein Coding	GC03P048359	3.232786179
PBX2	PBX Homeobox 2	Protein Coding	GC06M032184	3.228808403
MTOR	Mechanistic Target Of Rapamycin	Protein Coding	GC01M011106	3.225527525
CCL21	C-C Motif Chemokine Ligand 21	Protein Coding	GC09M034709	3.22497654
PLA2G7	Phospholipase A2 Group VII	Protein Coding	GC06M049418	3.222138882
FLT4	Fms Related Receptor Tyrosine Kinase	Protein Coding	GC05M180607	3.220872164
CEACAM5	CEA Cell Adhesion Molecule 5	Protein Coding	GC19P042039	3.214197874
TAGLN	Transgelin	Protein Coding	GC11P117199	3.210941315
COL17A1	Collagen Type XVII Alpha 1 Chain	Protein Coding	GC10M104031	3.205590725
CD24	CD24 Molecule	Protein Coding	GC06M106969	3.204554558
RECQL4	RecQ Like Helicase 4	Protein Coding	GC08M144512	3.204314232
COL1A1	Collagen Type I Alpha 1 Chain	Protein Coding	GC17M050183	3.199678421
SI	Sucrase-Isomaltase	Protein Coding	GC03M164978	3.194516182
EXO1	Exonuclease 1	Protein Coding	GC01P241847	3.194092512
MUC17	Mucin 17, Cell Surface Associated	Protein Coding	GC07P101020	3.190705299
ADCY10	Adenylate Cyclase 10	Protein Coding	GC01M167809	3.187153578
IL16	Interleukin 16	Protein Coding	GC15P081159	3.183465958
CDK4	Cyclin Dependent Kinase 4	Protein Coding	GC12M057743	3.178319931
UBE2L3	Ubiquitin Conjugating Enzyme E2 L3	Protein Coding	GC22P021549	3.176960468
CLDN7	Claudin 7	Protein Coding	GC17M007259	3.172602654
MIR199A1	MicroRNA 199a-1	RNA Gene	GC19M010817	3.158570051
INPP5E	Inositol Polyphosphate-5-Phosphatase	Protein Coding	GC09M136428	3.156127453
UGT1A6	UDP Glucuronosyltransferase Family 1 Member A6	Protein Coding	GC02P233691	3.156031609
DNMT3B	DNA Methyltransferase 3 Beta	Protein Coding	GC20P032762	3.153710842
ALOX12	Arachidonate 12-Lipoxygenase, 12S	Protein Coding	GC17P006995	3.153254032
SCYL1	SCY1 Like Pseudokinase 1	Protein Coding	GC11P065525	3.148246288
MIR210	MicroRNA 210	RNA Gene	GC11M001307	3.142729759
CD1A	CD1a Molecule	Protein Coding	GC01P158255	3.138727665
S100B	S100 Calcium Binding Protein B	Protein Coding	GC21M048631	3.138420105
TGFBR2	Transforming Growth Factor Beta Receptor 2	Protein Coding	GC03P030623	3.135755301
IL6-AS1	IL6 Antisense RNA 1	RNA Gene	GC07M022728	3.135681391
CCNA2	Cyclin A2	Protein Coding	GC04M121816	3.134578466
MIR200A	MicroRNA 200a	RNA Gene	GC01P002223	3.130750179
IDH1	Isocitrate Dehydrogenase (NADP(+)) 1	Protein Coding	GC02M208236	3.124005795
PPARA	Peroxisome Proliferator Activated Receptor Alpha	Protein Coding	GC22P046150	3.119888783
NCF2	Neutrophil Cytosolic Factor 2	Protein Coding	GC01M183555	3.118212223
POU5F1	POU Class 5 Homeobox 1	Protein Coding	GC06M049067	3.117991924
CTC1	CST Telomere Replication Complex Component 1	Protein Coding	GC17M009358	3.116493702
VTN	Vitronectin	Protein Coding	GC17M031461	3.115269661

PI3	Peptidase Inhibitor 3	Protein Coding	GC20P045174	3.113992214
TLR6	Toll Like Receptor 6	Protein Coding	GC04M038828	3.110791683
TNFRSF11B	TNF Receptor Superfamily Member	Protein Coding	GC08M118923	3.10562706
MAP2K1	Mitogen-Activated Protein Kinase Kinase 1	Protein Coding	GC15P066386	3.103982925
WASHC5	WASH Complex Subunit 5	Protein Coding	GC08M129570	3.095163584
FABP2	Fatty Acid Binding Protein 2	Protein Coding	GC04M119317	3.086678982
CASP9	Caspase 9	Protein Coding	GC01M015491	3.08010006
CDK2	Cyclin Dependent Kinase 2	Protein Coding	GC12P055966	3.076726913
ACKR2	Atypical Chemokine Receptor 2	Protein Coding	GC03P042804	3.074156284
SMARCA4	SWI/SNF Related, Matrix Associated, Actin Dependent Regulator Of Chromatin, Subfamily A, Member 4	Protein Coding	GC19P010932	3.073336124
ANXA1	Annexin A1	Protein Coding	GC09P073151	3.071945667
TF	Transferrin	Protein Coding	GC03P133666	3.067597866
ALDH1A1	Aldehyde Dehydrogenase 1 Family Member A1	Protein Coding	GC09M072900	3.067343712
PPM1L	Protein Phosphatase, Mg2+/Mn2+ Dependent 1L	Protein Coding	GC03P160755	3.066622257
ADGRE3	Adhesion G Protein-Coupled Receptor	Protein Coding	GC19M014619	3.066622257
EPHX1	Epoxide Hydrolase 1	Protein Coding	GC01P225810	3.065740108
ITK	IL2 Inducible T Cell Kinase	Protein Coding	GC05P157158	3.064249754
GUCY2C	Guanylate Cyclase 2C	Protein Coding	GC12M014612	3.063585281
CD248	CD248 Molecule	Protein Coding	GC11M066314	3.059728622
RAB27A	RAB27A, Member RAS Oncogene Family	Protein Coding	GC15M055202	3.055705547
LRP6	LDL Receptor Related Protein 6	Protein Coding	GC12M016340	3.050489426
TNPO3	Transportin 3	Protein Coding	GC07M128954	3.049941063
SAG	S-Antigen Visual Arrestin	Protein Coding	GC02P233333	3.048444748
JRKL	JRK Like	Protein Coding	GC11P096389	3.048444748
LST1	Leukocyte Specific Transcript 1	Protein Coding	GC06P058309	3.046359301
DAPK1	Death Associated Protein Kinase 1	Protein Coding	GC09P087497	3.045425415
CALR	Calreticulin	Protein Coding	GC19P012938	3.043394089
UCN2	Urocortin 2	Protein Coding	GC03M048561	3.038745403
GCKR	Glucokinase Regulator	Protein Coding	GC02P027496	3.038660526
KPNA7	Karyopherin Subunit Alpha 7	Protein Coding	GC07M099173	3.038660526
PROCR	Protein C Receptor	Protein Coding	GC20P035252	3.038300514
CYP27B1	Cytochrome P450 Family 27 Subfamily B Member 1	Protein Coding	GC12M057757	3.035820007
CTTN	Cortactin	Protein Coding	GC11P070398	3.035730839
MIR205	MicroRNA 205	RNA Gene	GC01P209432	3.0331285
OCLN	Occludin	Protein Coding	GC05P069492	3.024831295
BSG	Basigin (Ok Blood Group)	Protein Coding	GC19P000571	3.024102926
DAB2	DAB Adaptor Protein 2	Protein Coding	GC05M039371	3.023985386
IL13RA2	Interleukin 13 Receptor Subunit Alpha	Protein Coding	GC0XM115003	3.021356106
MIR15A	MicroRNA 15a	RNA Gene	GC13M050049	3.020375252
HOXA13	Homeobox A13	Protein Coding	GC07M027370	3.015438318
IDH2	Isocitrate Dehydrogenase (NADP(+)) 2	Protein Coding	GC15M090083	3.013313055
CHEK1	Checkpoint Kinase 1	Protein Coding	GC11P125625	3.012170315
RPS6KB1	Ribosomal Protein S6 Kinase B1	Protein Coding	GC17P059893	3.010018587
XBP1	X-Box Binding Protein 1	Protein Coding	GC22M028794	3.008569241
TXN	Thioredoxin	Protein Coding	GC09M110243	3.00589323
GPD2	Glycerol-3-Phosphate Dehydrogenase 2	Protein Coding	GC02P156435	3.003894567
IFITM3	Interferon Induced Transmembrane Protein 3	Protein Coding	GC11M000319	3.000776768
RPL39P28	Ribosomal Protein L39 Pseudogene 28	Pseudogene	GC12M068001	3.000776768
TLR7	Toll Like Receptor 7	Protein Coding	GC0XP012867	2.997173309
MGAT5	Alpha-1,6-Mannosylglycoprotein 6-Beta-N-Acetylglucosaminyltransferase	Protein Coding	GC02P134119	2.989356995
SHH	Sonic Hedgehog Signaling Molecule	Protein Coding	GC07M155799	2.979353428
MIR144	MicroRNA 144	RNA Gene	GC17M031468	2.975070953
HSF2	Heat Shock Transcription Factor 2	Protein Coding	GC06P122399	2.97225523
LPL	Lipoprotein Lipase	Protein Coding	GC08P019901	2.965034485

MAGI2	Membrane Associated Guanylate Kinase, WW And PDZ Domain	Protein Coding	GC07M078017	2.960053921
SRSF6	Serine And Arginine Rich Splicing Factor 6	Protein Coding	GC20P043457	2.955579519
RPS14P1	Ribosomal Protein S14 Pseudogene 1	Pseudogene	GC01M206695	2.952447414
GMPPB	GDP-Mannose Pyrophosphorylase B	Protein Coding	GC03M049716	2.945627689
SFTPD	Surfactant Protein D	Protein Coding	GC10M079937	2.94159174
ZGPAT	Zinc Finger CCCH-Type And G-Patch Domain Containing	Protein Coding	GC20P063707	2.930682659
VIL1	Villin 1	Protein Coding	GC02P218419	2.920403719
GTF2H4	General Transcription Factor IIH Subunit 4	Protein Coding	GC06P058290	2.9089818
MAPK8	Mitogen-Activated Protein Kinase 8	Protein Coding	GC10P048306	2.908303738
SLC19A1	Solute Carrier Family 19 Member 1	Protein Coding	GC21M045493	2.907470226
UBA7	Ubiquitin Like Modifier Activating Enzyme 7	Protein Coding	GC03M049805	2.905908585
ACTA2	Actin Alpha 2, Smooth Muscle	Protein Coding	GC10M088935	2.900230169
TNFRSF25	TNF Receptor Superfamily Member 25	Protein Coding	GC01M006460	2.899212837
PERP	P53 Apoptosis Effector Related To PMP22	Protein Coding	GC06M138088	2.89563179
NOS1	Nitric Oxide Synthase 1	Protein Coding	GC12M117208	2.891417503
CLU	Clusterin	Protein Coding	GC08M027596	2.888332367
BMP4	Bone Morphogenetic Protein 4	Protein Coding	GC14M053949	2.8874681
CFDP1	Craniofacial Development Protein 1	Protein Coding	GC16M075294	2.884737015
MIR19A	MicroRNA 19a	RNA Gene	GC13P091471	2.882544041
FYN	FYN Proto-Oncogene, Src Family Tyrosine Kinase	Protein Coding	GC06M111660	2.873184443
SLC22A23	Solute Carrier Family 22 Member 23	Protein Coding	GC06M003269	2.87293911
MIR196A1	MicroRNA 196a-1	RNA Gene	GC17M048632	2.869436741
SERPINH1	Serpin Family H Member 1	Protein Coding	GC11P075562	2.868783236
MUC3B	Mucin 3B, Cell Surface Associated	Protein Coding	GC07U903146	2.866207361
TH	Tyrosine Hydroxylase	Protein Coding	GC11M002163	2.863307953
REG1A	Regenerating Family Member 1 Alpha	Protein Coding	GC02P079120	2.861798763
PRRC2A	Proline Rich Coiled-Coil 2A	Protein Coding	GC06P058310	2.861608982
RHOD	Ras Homolog Family Member D	Protein Coding	GC11P067069	2.859848976
IKZF3	IKAROS Family Zinc Finger 3	Protein Coding	GC17M039759	2.859793186
PLCH2	Phospholipase C Eta 2	Protein Coding	GC01P002421	2.859793186
SLC2A4RG	SLC2A4 Regulator	Protein Coding	GC20P063739	2.859793186
MMEL1	Membrane Metalloendopeptidase Like	Protein Coding	GC01M002590	2.859793186
TNFSF8	TNF Superfamily Member 8	Protein Coding	GC09M114893	2.859793186
NXPE1	Neurexophilin And PC-Esterase Domain Family Member 1	Protein Coding	GC11M114521	2.859793186
ENTR1	Endosome Associated Trafficking Regulator 1	Protein Coding	GC09M136403	2.859793186
LINC00598	Long Intergenic Non-Protein Coding RNA 598	RNA Gene	GC13M040088	2.859793186
PITX1-AS1	PITX1 Antisense RNA 1	RNA Gene	GC05P135035	2.859793186
BDNF	Brain Derived Neurotrophic Factor	Protein Coding	GC11M027654	2.85740304
FCAR	Fc Fragment Of IgA Receptor	Protein Coding	GC19P057016	2.852687836
DNAL4	Dynein Axonemal Light Chain 4	Protein Coding	GC22M038778	2.846854687
REG3A	Regenerating Family Member 3 Alpha	Protein Coding	GC02M079157	2.834452629
TIAL1	TIA1 Cytotoxic Granule Associated RNA Binding Protein Like 1	Protein Coding	GC10M119571	2.832091093
SEC16A	SEC16 Homolog A, Endoplasmic Reticulum Export Factor	Protein Coding	GC09M136440	2.819867373
HMGB2	High Mobility Group Box 2	Protein Coding	GC04M173331	2.818183661
ITPA	Inosine Triphosphatase	Protein Coding	GC20P003365	2.817051411
P4HA2	Prolyl 4-Hydroxylase Subunit Alpha 2	Protein Coding	GC05M132191	2.814940453
MIR124-1	MicroRNA 124-1	RNA Gene	GC08M009903	2.813624859
FADD	Fas Associated Via Death Domain	Protein Coding	GC11P070203	2.812305689
MSH5	MutS Homolog 5	Protein Coding	GC06P058324	2.809771061
CD244	CD244 Molecule	Protein Coding	GC01M160830	2.807280779
EPC1	Enhancer Of Polycomb Homolog 1	Protein Coding	GC10M032268	2.805334568

PLA2G4F	Phospholipase A2 Group IVF	Protein Coding	GC15M042139	2.805334568
DMAP1	DNA Methyltransferase 1 Associated Protein 1	Protein Coding	GC01P044214	2.805334568
HVCN1	Hydrogen Voltage Gated Channel 1	Protein Coding	GC12M110627	2.805334568
TMEM201	Transmembrane Protein 201	Protein Coding	GC01P009588	2.805334568
METTL9	Methyltransferase Like 9	Protein Coding	GC16P021615	2.805334568
IGSF6-DREV1	Region Containing Immunoglobulin Superfamily, Member 6 And DREV1	Uncategorized	GC16U900497	2.805334568
MIR20A	MicroRNA 20a	RNA Gene	GC13P091472	2.804739237
MIR25	MicroRNA 25	RNA Gene	GC07M100093	2.804739237
KCP	Kielin Cysteine Rich BMP Regulator	Protein Coding	GC07M128862	2.799721956
MROH3P	Maestro Heat Like Repeat Family Member 3, Pseudogene	Pseudogene	GC01P200920	2.796648741
CYP21A2	Cytochrome P450 Family 21 Subfamily A Member 2	Protein Coding	GC06P058334	2.796322823
U2AF1	U2 Small Nuclear RNA Auxiliary Factor 1	Protein Coding	GC21M043092	2.793002605
PHB	Prohibitin	Protein Coding	GC17M049404	2.788365841
DCLK1	Doublecortin Like Kinase 1	Protein Coding	GC13M035768	2.786621332
NRP1	Neuropilin 1	Protein Coding	GC10M033177	2.779112816
USP4	Ubiquitin Specific Peptidase 4	Protein Coding	GC03M049277	2.775705099
ENO2	Enolase 2	Protein Coding	GC12P006913	2.774648428
PRDM1	PR/SET Domain 1	Protein Coding	GC06P105993	2.774316788
ANGPTL4	Angiopoietin Like 4	Protein Coding	GC19P008363	2.770564079
PROM1	Prominin 1	Protein Coding	GC04M015965	2.769141674
KRT1	Keratin 1	Protein Coding	GC12M052674	2.768013
MIR320A	MicroRNA 320a	RNA Gene	GC08M022249	2.760382652
KLF4	Kruppel Like Factor 4	Protein Coding	GC09M107484	2.760133505
IL25	Interleukin 25	Protein Coding	GC14P026866	2.756122589
AICDA	Activation Induced Cytidine Deaminase	Protein Coding	GC12M008602	2.751930714
GLP2R	Glucagon Like Peptide 2 Receptor	Protein Coding	GC17P009822	2.749878883
GSN	Gelsolin	Protein Coding	GC09P121201	2.747339249
TRPV4	Transient Receptor Potential Cation Channel Subfamily V Member 4	Protein Coding	GC12M109783	2.74594903
TYMS	Thymidylate Synthetase	Protein Coding	GC18P000657	2.745801449
MIRLET7I	MicroRNA Let-7i	RNA Gene	GC12P062606	2.734656811
FASN	Fatty Acid Synthase	Protein Coding	GC17M082078	2.731348038
TGFB3	Transforming Growth Factor Beta 3	Protein Coding	GC14M075958	2.729678392
NDRG1	N-Myc Downstream Regulated 1	Protein Coding	GC08M133237	2.725729704
IP6K1	Inositol Hexakisphosphate Kinase 1	Protein Coding	GC03M050159	2.725578785
PFKFB4	6-Phosphofructo-2-Kinase/Fructose-2,6-Biphosphatase 4	Protein Coding	GC03M048517	2.725578785
IP6K2	Inositol Hexakisphosphate Kinase 2	Protein Coding	GC03M048688	2.725578785
SP1	Sp1 Transcription Factor	Protein Coding	GC12P053380	2.72369051
MIR182	MicroRNA 182	RNA Gene	GC07M129770	2.719152212
RARRES2	Retinoic Acid Receptor Responder 2	Protein Coding	GC07M150333	2.717495203
YAP1	Yes1 Associated Transcriptional Regulator	Protein Coding	GC11P102110	2.713131666
IFNAR1	Interferon Alpha And Beta Receptor Subunit 1	Protein Coding	GC21P033324	2.71170783
GPR65	G Protein-Coupled Receptor 65	Protein Coding	GC14P088005	2.708227873
SLC5A8	Solute Carrier Family 5 Member 8	Protein Coding	GC12M101155	2.706077337
KEAP1	Kelch Like ECH Associated Protein 1	Protein Coding	GC19M010486	2.705426216
MDH2	Malate Dehydrogenase 2	Protein Coding	GC07P076048	2.703724146
CSN1S1	Casein Alpha S1	Protein Coding	GC04P069932	2.701978922
ERBB4	Erb-B2 Receptor Tyrosine Kinase 4	Protein Coding	GC02M211375	2.699473381
SNAI1	Snail Family Transcriptional Repressor	Protein Coding	GC20P049982	2.689509392
ABL1	ABL Proto-Oncogene 1, Non-Receptor Tyrosine Kinase	Protein Coding	GC09P130713	2.689328671
TNFRSF10B	TNF Receptor Superfamily Member	Protein Coding	GC08M023020	2.687960148
HSPA8	Heat Shock Protein Family A (Hsp70) Member 8	Protein Coding	GC11M123057	2.680380821
CXCL3	C-X-C Motif Chemokine Ligand 3	Protein Coding	GC04M074036	2.676275253

MLXIPL	MLX Interacting Protein Like	Protein Coding	GC07M073593	2.676023006
TEP1	Telomerase Associated Protein 1	Protein Coding	GC14M020365	2.670790195
NF1	Neurofibromin 1	Protein Coding	GC17P031094	2.670036793
TSLP	Thymic Stromal Lymphopoietin	Protein Coding	GC05P111070	2.668251038
TRIM39	Tripartite Motif Containing 39	Protein Coding	GC06P058274	2.666193247
FLOT1	Flotillin 1	Protein Coding	GC06M049044	2.666119576
DAXX	Death Domain Associated Protein	Protein Coding	GC06M033318	2.664566755
CCN4	Cellular Communication Network Factor 4	Protein Coding	GC08P133192	2.661743402
DDB2	Damage Specific DNA Binding Protein	Protein Coding	GC11P047237	2.646484852
MIR93	MicroRNA 93	RNA Gene	GC07M100602	2.645860434
WNT2	Wnt Family Member 2	Protein Coding	GC07M117277	2.643161297
LIF	LIF Interleukin 6 Family Cytokine	Protein Coding	GC22M030240	2.641541004
COX5A	Cytochrome C Oxidase Subunit 5A	Protein Coding	GC15M074919	2.637547016
IL1RAP	Interleukin 1 Receptor Accessory	Protein Coding	GC03P190514	2.630175352
MIR15B	MicroRNA 15b	RNA Gene	GC03P160404	2.629002571
FKBP15	FKBP Prolyl Isomerase Family Member 15	Protein Coding	GC09M113161	2.626627922
GDF15	Growth Differentiation Factor 15	Protein Coding	GC19P027797	2.622390509
PTGER2	Prostaglandin E Receptor 2	Protein Coding	GC14P052314	2.619499922
TUG1	Taurine Up-Regulated 1	RNA Gene	GC22P030969	2.617917299
FPR1	Formyl Peptide Receptor 1	Protein Coding	GC19M051745	2.61660552
F2RL3	F2R Like Thrombin Or Trypsin Receptor 3	Protein Coding	GC19P016888	2.615855694
RSPO1	R-Spondin 1	Protein Coding	GC01M037612	2.614655495
G6PC1	Glucose-6-Phosphatase Catalytic Subunit 1	Protein Coding	GC17P045036	2.612273216
PITX1	Paired Like Homeodomain 1	Protein Coding	GC05M135027	2.609574318
PNMT	Phenylethanolamine N-	Protein Coding	GC17P039667	2.609574318
NKX3-1	NK3 Homeobox 1	Protein Coding	GC08M023678	2.609574318
UTS2	Urotensin 2	Protein Coding	GC01M007843	2.609574318
PIM3	Pim-3 Proto-Oncogene, Serine/Threonine Kinase	Protein Coding	GC22P049960	2.609574318
OLIG3	Oligodendrocyte Transcription Factor 3	Protein Coding	GC06M137492	2.609574318
SERINC3	Serine Incorporator 3	Protein Coding	GC20M044496	2.609574318
STMN3	Stathmin 3	Protein Coding	GC20M063639	2.609574318
AAMP	Angio Associated Migratory Cell	Protein Coding	GC02M218264	2.609574318
EXOC3	Exocyst Complex Component 3	Protein Coding	GC05P000443	2.609574318
SNAPC4	Small Nuclear RNA Activating Complex Polypeptide 4	Protein Coding	GC09M136375	2.609574318
ZBTB46	Zinc Finger And BTB Domain Containing 46	Protein Coding	GC20M063743	2.609574318
AMIGO3	Adhesion Molecule With Ig Like Domain 3	Protein Coding	GC03M049718	2.609574318
PRXL2B	Peroxiredoxin Like 2B	Protein Coding	GC01P002587	2.609574318
LOC100996583	Uncharacterized LOC100996583	RNA Gene	GC01P002568	2.609574318
HSD11B2	Hydroxysteroid 11-Beta	Protein Coding	GC16P067433	2.607228756
NFKBIZ	NFKB Inhibitor Zeta	Protein Coding	GC03P101827	2.606742144
TYMP	Thymidine Phosphorylase	Protein Coding	GC22M050525	2.605536461
UBAC1	UBA Domain Containing 1	Protein Coding	GC09M135932	2.601281166
TNFSF13B	TNF Superfamily Member 13b	Protein Coding	GC13P108251	2.59874177
FOXO3	Forkhead Box O3	Protein Coding	GC06P108559	2.592011213
WASHC4	WASH Complex Subunit 4	Protein Coding	GC12P105108	2.586220741
HTR3A	5-Hydroxytryptamine Receptor 3A	Protein Coding	GC11P113974	2.584484816
CREB1	CAMP Responsive Element Binding Protein 1	Protein Coding	GC02P207529	2.582422256
AK2	Adenylate Kinase 2	Protein Coding	GC01M033007	2.581670284
PSORS1C1	Psoriasis Susceptibility 1 Candidate 1	Protein Coding	GC06P031114	2.580816269
TOLLIP	Toll Interacting Protein	Protein Coding	GC11M001274	2.580800056
PKM	Pyruvate Kinase M1/2	Protein Coding	GC15M072199	2.58015871
HSPG2	Heparan Sulfate Proteoglycan 2	Protein Coding	GC01M021822	2.577660322

PTPRO	Protein Tyrosine Phosphatase Receptor Type O	Protein Coding	GC12P015366	2.576501369
MDK	Midkine	Protein Coding	GC11P046380	2.576501369
KAT5	Lysine Acetyltransferase 5	Protein Coding	GC11P065711	2.575743198
RDX	Radixin	Protein Coding	GC11M109864	2.568436861
MME	Membrane Metalloendopeptidase	Protein Coding	GC03P155024	2.567410946
VCL	Vinculin	Protein Coding	GC10P073995	2.565592289
HSP90AA1	Heat Shock Protein 90 Alpha Family Class A Member 1	Protein Coding	GC14M102080	2.565293312
DVL1	Dishevelled Segment Polarity Protein 1	Protein Coding	GC01M001335	2.552558422
MUC16	Mucin 16, Cell Surface Associated	Protein Coding	GC19M008848	2.546663284
IER3	Immediate Early Response 3	Protein Coding	GC06M030743	2.543234348
S1PR1	Sphingosine-1-Phosphate Receptor 1	Protein Coding	GC01P101236	2.542637825
TRPV1	Transient Receptor Potential Cation Channel Subfamily V Member 1	Protein Coding	GC17M003565	2.540592432
MIR106A	MicroRNA 106a	RNA Gene	GC0XM134219	2.540180922
ADRA2A	Adrenoceptor Alpha 2A	Protein Coding	GC10P111077	2.539652348
MUC20	Mucin 20, Cell Surface Associated	Protein Coding	GC03P195720	2.539327145
KRT15	Keratin 15	Protein Coding	GC17M041513	2.538962126
MIR196A2	MicroRNA 196a-2	RNA Gene	GC12P054542	2.532645702
TGFBR1	Transforming Growth Factor Beta Receptor 1	Protein Coding	GC09P099104	2.530440331
ITGA5	Integrin Subunit Alpha 5	Protein Coding	GC12M054469	2.527314425
CFHR1	Complement Factor H Related 1	Protein Coding	GC01P196822	2.526212454
WNT16	Wnt Family Member 16	Protein Coding	GC07P121325	2.52545023
ADORA3	Adenosine A3 Receptor	Protein Coding	GC01M111499	2.523755074
GLI1	GLI Family Zinc Finger 1	Protein Coding	GC12P057460	2.520491838
STK39	Serine/Threonine Kinase 39	Protein Coding	GC02M167954	2.518633842
RCL1	RNA Terminal Phosphate Cyclase Like	Protein Coding	GC09P004782	2.513648033
HLA-DRB3	Major Histocompatibility Complex, Class II, DR Beta 3	Protein Coding	GC06Mn03715	2.512004375
XRCC5	X-Ray Repair Cross Complementing 5	Protein Coding	GC02P216107	2.510718346
SFRP2	Secreted Frizzled Related Protein 2	Protein Coding	GC04M153780	2.510144234
GAL	Galanin And GMAP Prepropeptide	Protein Coding	GC11P068684	2.508176327
HAMP	Hepcidin Antimicrobial Peptide	Protein Coding	GC19P041412	2.507675886
HSP90B1	Heat Shock Protein 90 Beta Family Member 1	Protein Coding	GC12P103930	2.505652428
LINC00243	Long Intergenic Non-Protein Coding RNA 243	RNA Gene	GC06M049042	2.499988556
HDGF	Heparin Binding Growth Factor	Protein Coding	GC01M156800	2.499746799
IL7	Interleukin 7	Protein Coding	GC08M078689	2.495172262
TFAP2A	Transcription Factor AP-2 Alpha	Protein Coding	GC06M010393	2.492806435
CD27-AS1	CD27 Antisense RNA 1	RNA Gene	GC12M006439	2.490917921
NR4A1	Nuclear Receptor Subfamily 4 Group A Member 1	Protein Coding	GC12P052022	2.490694761
FLVCR1	FLVCR Heme Transporter 1	Protein Coding	GC01P212858	2.487361193
UBE4A	Ubiquitination Factor E4A	Protein Coding	GC11P118359	2.486635447
REV3L	REV3 Like, DNA Directed Polymerase Zeta Catalytic Subunit	Protein Coding	GC06M111299	2.48624754
TNFSF12	TNF Superfamily Member 12	Protein Coding	GC17P009335	2.485201836
XRCC6	X-Ray Repair Cross Complementing 6	Protein Coding	GC22P041622	2.482366562
TBXT	T-Box Transcription Factor T	Protein Coding	GC06M166158	2.478410721
RUNX1	RUNX Family Transcription Factor 1	Protein Coding	GC21M034787	2.477157354
P2RX3	Purinergic Receptor P2X 3	Protein Coding	GC11P057356	2.474074602
DUOXA2	Dual Oxidase Maturation Factor 2	Protein Coding	GC15P045114	2.473435402
AGT	Angiotensinogen	Protein Coding	GC01M230702	2.472409725
CREBBP	CREB Binding Protein	Protein Coding	GC16M004002	2.467034101
AFP	Alpha Fetoprotein	Protein Coding	GC04P073431	2.466137171
MAPK1	Mitogen-Activated Protein Kinase 1	Protein Coding	GC22M021754	2.464361668
LRP5	LDL Receptor Related Protein 5	Protein Coding	GC11P068298	2.463799238
UGT1A1	UDP Glucuronosyltransferase Family 1 Member A1	Protein Coding	GC02P233760	2.462043285

DDR1	Discoidin Domain Receptor Tyrosine Kinase 1	Protein Coding	GC06P058289	2.461120844
TRA	T Cell Receptor Alpha Locus	Protein Coding	GC14P021621	2.451304197
MAD2L1	Mitotic Arrest Deficient 2 Like 1	Protein Coding	GC04M120055	2.450685024
TNFRSF10D	TNF Receptor Superfamily Member	Protein Coding	GC08M023135	2.450685024
GUCA2A	Guanylate Cyclase Activator 2A	Protein Coding	GC01M042162	2.450685024
FLII	FLII Actin Remodeling Protein	Protein Coding	GC17M018244	2.448996544
IGFBP3	Insulin Like Growth Factor Binding Protein 3	Protein Coding	GC07M045912	2.4487679
LYRM4	LYR Motif Containing 4	Protein Coding	GC06M005032	2.448234797
MUC19	Mucin 19, Oligomeric	Protein Coding	GC12P040393	2.448234797
IL15RA	Interleukin 15 Receptor Subunit Alpha	Protein Coding	GC10M005943	2.444879055
EPB42	Erythrocyte Membrane Protein Band	Protein Coding	GC15M043293	2.443192005
KDM4C	Lysine Demethylase 4C	Protein Coding	GC09P006720	2.441658735
APAF1	Apoptotic Peptidase Activating Factor	Protein Coding	GC12P098645	2.441452503
MSH3	MutS Homolog 3	Protein Coding	GC05P080654	2.441452503
NUDT15	Nudix Hydrolase 15	Protein Coding	GC13P048037	2.441277742
NT5E	5'-Nucleotidase Ecto	Protein Coding	GC06P085449	2.440476179
ANGPT2	Angiopoietin 2	Protein Coding	GC08M006499	2.438059807
HPSE	Heparanase	Protein Coding	GC04M083292	2.435198307
FGFR1	Fibroblast Growth Factor Receptor 1	Protein Coding	GC08M038400	2.431594372
MFGE8	Milk Fat Globule EGF And Factor V/VIII Domain Containing	Protein Coding	GC15M088898	2.42933321
GGT1	Gamma-Glutamyltransferase 1	Protein Coding	GC22P024583	2.428750992
CA2	Carbonic Anhydrase 2	Protein Coding	GC08P085463	2.42287302
ZMIZ1	Zinc Finger MIZ-Type Containing 1	Protein Coding	GC10P079068	2.422721863
CACNA2D1	Calcium Voltage-Gated Channel Auxiliary Subunit Alpha2delta 1	Protein Coding	GC07M081946	2.422499657
VARS2	Valyl-TRNA Synthetase 2,	Protein Coding	GC06P058292	2.422499657
ACTR3B	Actin Related Protein 3B	Protein Coding	GC07P152759	2.422499657
COL13A1	Collagen Type XIII Alpha 1 Chain	Protein Coding	GC10P069801	2.422499657
NCAPD2	Non-SMC Condensin I Complex Subunit D2	Protein Coding	GC12P006493	2.422499657
CPXM2	Carboxypeptidase X, M14 Family Member 2	Protein Coding	GC10M123710	2.422499657
CAVIN1	Caveolae Associated Protein 1	Protein Coding	GC17M042404	2.422499657
CALHM6	Calcium Homeostasis Modulator Family Member 6	Protein Coding	GC06P116462	2.422499657
MIR26B	MicroRNA 26b	RNA Gene	GC02P218402	2.422499657
HCG9	HLA Complex Group 9	RNA Gene	GC06P058246	2.422499657
HCG26	HLA Complex Group 26	RNA Gene	GC06P058301	2.422499657
MIR588	MicroRNA 588	RNA Gene	GC06P126484	2.422499657
GRB2	Growth Factor Receptor Bound Protein	Protein Coding	GC17M075318	2.419885397
MIR206	MicroRNA 206	RNA Gene	GC06P052144	2.412064075
CEACAM1	CEA Cell Adhesion Molecule 1	Protein Coding	GC19M042507	2.411261797
CCL17	C-C Motif Chemokine Ligand 17	Protein Coding	GC16P057405	2.409724712
CD83	CD83 Molecule	Protein Coding	GC06P014117	2.407797337
MIR30A	MicroRNA 30a	RNA Gene	GC06M071403	2.406183481
SYVN1	Synoviolin 1	Protein Coding	GC11M071656	2.40524435
RRM2B	Ribonucleotide Reductase Regulatory TP53 Inducible Subunit M2B	Protein Coding	GC08M102204	2.403946161
HSD11B1	Hydroxysteroid 11-Beta	Protein Coding	GC01P209686	2.402334929
BDKRB2	Bradykinin Receptor B2	Protein Coding	GC14P096205	2.398932934
TIMP3	TIMP Metallopeptidase Inhibitor 3	Protein Coding	GC22P032800	2.39797616
NFKB2	Nuclear Factor Kappa B Subunit 2	Protein Coding	GC10P102394	2.387108088
MLKL	Mixed Lineage Kinase Domain Like Pseudokinase	Protein Coding	GC16M074672	2.385401726
SULT1A2	Sulfotransferase Family 1A Member 2	Protein Coding	GC16M028591	2.384210587
GLI3	GLI Family Zinc Finger 3	Protein Coding	GC07M041960	2.384065866
NR5A2	Nuclear Receptor Subfamily 5 Group A Member 2	Protein Coding	GC01P199996	2.383906126
C1orf141	Chromosome 1 Open Reading Frame	Protein Coding	GC01M067092	2.382806063
MIR17	MicroRNA 17	RNA Gene	GC13P091350	2.374859571

GUCA2B	Guanylate Cyclase Activator 2B	Protein Coding	GC01P042153	2.373196363
PROS1	Protein S	Protein Coding	GC03M093873	2.372889757
TRAF2	TNF Receptor Associated Factor 2	Protein Coding	GC09P136881	2.369910479
PRPF8	Pre-MRNA Processing Factor 8	Protein Coding	GC17M001650	2.368267775
HLA-DQA2	Major Histocompatibility Complex, Class II, DQ Alpha 2	Protein Coding	GC06P032741	2.366362095
GJA1	Gap Junction Protein Alpha 1	Protein Coding	GC06P121436	2.363511562
ABCC3	ATP Binding Cassette Subfamily C Member 3	Protein Coding	GC17P050634	2.361723185
ALPI	Alkaline Phosphatase, Intestinal	Protein Coding	GC02P232456	2.355756283
PAK1	P21 (RAC1) Activated Kinase 1	Protein Coding	GC11M077321	2.3504529
SPINK1	Serine Peptidase Inhibitor Kazal Type 1	Protein Coding	GC05M147825	2.348980904
NOL3	Nucleolar Protein 3	Protein Coding	GC16P067188	2.340536356
SYP	Synaptophysin	Protein Coding	GC0XM049187	2.338814497
MIR149	MicroRNA 149	RNA Gene	GC02P240456	2.332869291
DNMT3A	DNA Methyltransferase 3 Alpha	Protein Coding	GC02M025228	2.330291271
ITGA2	Integrin Subunit Alpha 2	Protein Coding	GC05P052989	2.325936079
RETN	Resistin	Protein Coding	GC19P007669	2.321208
ALOX15	Arachidonate 15-Lipoxygenase	Protein Coding	GC17M004630	2.318053246
MIR125A	MicroRNA 125a	RNA Gene	GC19P051720	2.317450523
PRDX1	Peroxiredoxin 1	Protein Coding	GC01M045511	2.316484928
F11R	F11 Receptor	Protein Coding	GC01M160995	2.312925339
UBASH3B	Ubiquitin Associated And SH3 Domain Containing B	Protein Coding	GC11P122655	2.311907291
IGLL5	Immunoglobulin Lambda Like Polypeptide 5	Protein Coding	GC22P027386	2.310692787
CYP4F3	Cytochrome P450 Family 4 Subfamily F Member 3	Protein Coding	GC19P015640	2.309760094
G6PD	Glucose-6-Phosphate Dehydrogenase	Protein Coding	GC0XM154531	2.306203842
TMEM221	Transmembrane Protein 221	Protein Coding	GC19M017435	2.30068779
SAA4	Serum Amyloid A4, Constitutive	Protein Coding	GC11M018234	2.295862198
MIR191	MicroRNA 191	RNA Gene	GC03M049511	2.294769287
CDO1	Cysteine Dioxygenase Type 1	Protein Coding	GC05M115804	2.293868542
TXK	TXK Tyrosine Kinase	Protein Coding	GC04M048076	2.289897442
MECP2	Methyl-CpG Binding Protein 2	Protein Coding	GC0XM154021	2.287275791
HSPB1	Heat Shock Protein Family B (Small) Member 1	Protein Coding	GC07P076302	2.28622961
GPR183	G Protein-Coupled Receptor 183	Protein Coding	GC13M099298	2.285270214
NEU1	Neuraminidase 1	Protein Coding	GC06M031857	2.284974098
HSPA5	Heat Shock Protein Family A (Hsp70) Member 5	Protein Coding	GC09M125234	2.284748077
EPHB4	EPH Receptor B4	Protein Coding	GC07M100803	2.283927441
MIR148A	MicroRNA 148a	RNA Gene	GC07M025993	2.277617455
MIR499A	MicroRNA 499a	RNA Gene	GC20P034990	2.277351856
EPHA3	EPH Receptor A3	Protein Coding	GC03P089077	2.269262552
FCGR1A	Fc Fragment Of IgG Receptor Ia	Protein Coding	GC01P149754	2.268510342
DPEP1	Dipeptidase 1	Protein Coding	GC16P089613	2.263282299
SETD2	SET Domain Containing 2, Histone Lysine Methyltransferase	Protein Coding	GC03M047033	2.260502338
SRF	Serum Response Factor	Protein Coding	GC06P043171	2.259732723
LCK	LCK Proto-Oncogene, Src Family Tyrosine Kinase	Protein Coding	GC01P032251	2.25779438
FCN3	Ficolin 3	Protein Coding	GC01M027398	2.255932093
PPP1R14A	Protein Phosphatase 1 Regulatory Inhibitor Subunit 14A	Protein Coding	GC19M038251	2.25570631
KIR2DL3	Killer Cell Immunoglobulin Like Receptor, Two Ig Domains And Long Cytoplasmic Tail 3	Protein Coding	GC19P057267	2.248356819
OSCAR	Osteoclast Associated Ig-Like Receptor	Protein Coding	GC19M054094	2.247098684
TRAF1	TNF Receptor Associated Factor 1	Protein Coding	GC09M120902	2.246613264
TGFB2	Transforming Growth Factor Beta 2	Protein Coding	GC01P218345	2.243509054
LTBR	Lymphotoxin Beta Receptor	Protein Coding	GC12P006375	2.240722179

BMI1	BMI1 Proto-Oncogene, Polycomb Ring Finger	Protein Coding	GC10P022326	2.238879681
JAG1	Jagged Canonical Notch Ligand 1	Protein Coding	GC20M010637	2.238822937
MUC13	Mucin 13, Cell Surface Associated	Protein Coding	GC03M124905	2.23405385
CEACAM3	CEA Cell Adhesion Molecule 3	Protein Coding	GC19P041796	2.232859135
MIR378A	MicroRNA 378a	RNA Gene	GC05P149732	2.231870651
GZMA	Granzyme A	Protein Coding	GC05P055102	2.230305195
CXCL11	C-X-C Motif Chemokine Ligand 11	Protein Coding	GC04M076033	2.224616766
ITGB3	Integrin Subunit Beta 3	Protein Coding	GC17P047254	2.224482298
NGFR	Nerve Growth Factor Receptor	Protein Coding	GC17P049495	2.223907471
DLAT	Dihydrolipoamide S-Acetyltransferase	Protein Coding	GC11P112024	2.220843315
HTR4	5-Hydroxytryptamine Receptor 4	Protein Coding	GC05M148451	2.218595505
KITLG	KIT Ligand	Protein Coding	GC12M088492	2.21674943
GOLGB1	Golgin B1	Protein Coding	GC03M121663	2.212016821
STK11	Serine/Threonine Kinase 11	Protein Coding	GC19P001177	2.206728935
TWIST1	Twist Family BHLH Transcription Factor 1	Protein Coding	GC07M019020	2.206350803
ARMH3	Armadillo Like Helical Domain Containing 3	Protein Coding	GC10M101846	2.201954842
MIR103A2	MicroRNA 103a-2	RNA Gene	GC20P003917	2.201954842
CAV2	Caveolin 2	Protein Coding	GC07P116287	2.200849533
COL4A5	Collagen Type IV Alpha 5 Chain	Protein Coding	GC0XP108439	2.200371981
SERPINA3	Serpin Family A Member 3	Protein Coding	GC14P094612	2.200304747
KIR2DL1	Killer Cell Immunoglobulin Like Receptor, Two Ig Domains And Long Cytoplasmic Tail 1	Protein Coding	GC19P056878	2.197931528
CCN1	Cellular Communication Network Factor 1	Protein Coding	GC01P085581	2.196918488
TNFRSF8	TNF Receptor Superfamily Member 8	Protein Coding	GC01P012063	2.194870949
ATP6V1G2	ATPase H+ Transporting V1 Subunit	Protein Coding	GC06M049113	2.193674803
DST	Dystonin	Protein Coding	GC06M056457	2.193383455
MT-ND4	Mitochondrially Encoded NADH:Ubiquinone Oxidoreductase Core Subunit 4	Protein Coding	GCMTP010762	2.191684246
CCR2	C-C Motif Chemokine Receptor 2	Protein Coding	GC03P046356	2.184646368
RASSF5	Ras Association Domain Family Member 5	Protein Coding	GC01P206507	2.18198967
TNFSF14	TNF Superfamily Member 14	Protein Coding	GC19M006663	2.181389332
IL13RA1	Interleukin 13 Receptor Subunit Alpha	Protein Coding	GC0XP118727	2.180823088
ST14	ST14 Transmembrane Serine Protease Matriptase	Protein Coding	GC11P130159	2.170773983
CNR1	Cannabinoid Receptor 1	Protein Coding	GC06M088139	2.163163662
NTF3	Neurotrophin 3	Protein Coding	GC12P005432	2.161371708
PF4	Platelet Factor 4	Protein Coding	GC04M073980	2.158733845
TXNIP	Thioredoxin Interacting Protein	Protein Coding	GC01M145992	2.158671856
CREM	CAMP Responsive Element Modulator	Protein Coding	GC10P035126	2.158539772
ADORA2A	Adenosine A2a Receptor	Protein Coding	GC22P024417	2.158322811
SNAI2	Snail Family Transcriptional Repressor	Protein Coding	GC08M048917	2.154368877
SPHK1	Sphingosine Kinase 1	Protein Coding	GC17P076376	2.154041767
NPSR1	Neuropeptide S Receptor 1	Protein Coding	GC07P034664	2.153530598
GART	Phosphoribosylglycinamide Formyltransferase, Phosphoribosylglycinamide Synthetase, Phosphoribosylaminoimidazole Synthetase	Protein Coding	GC21M033503	2.152756691
RGS14	Regulator Of G Protein Signaling 14	Protein Coding	GC05P177357	2.152756691
ASCL2	Achaete-Scute Family BHLH Transcription Factor 2	Protein Coding	GC11M002269	2.150955439
NTAN1	N-Terminal Asparagine Amidase	Protein Coding	GC16M015037	2.1471591
CCL22	C-C Motif Chemokine Ligand 22	Protein Coding	GC16P057359	2.146427155
LY9	Lymphocyte Antigen 9	Protein Coding	GC01P160796	2.141642094
AGR2	Anterior Gradient 2, Protein Disulphide Isomerase Family Member	Protein Coding	GC07M016898	2.138998985

NLRP7	NLR Family Pyrin Domain Containing	Protein Coding	GC19M054923	2.13708353
SERPINB1	Serpin Family B Member 1	Protein Coding	GC06M002833	2.135876179
PFKM	Phosphofructokinase, Muscle	Protein Coding	GC12P048105	2.135387659
CPOX	Coproporphyrinogen Oxidase	Protein Coding	GC03M098576	2.13456583
MIR146B	MicroRNA 146b	RNA Gene	GC10P102436	2.134141922
KIR2DL2	Killer Cell Immunoglobulin Like Receptor, Two Ig Domains And Long Cytoplasmic Tail 2	Protein Coding	GC19Mr00108	2.133339405
KIR2DS2	Killer Cell Immunoglobulin Like Receptor, Two Ig Domains And Short Cytoplasmic Tail 2	Protein Coding	GC19MR00122	2.133339405
MAP2K2	Mitogen-Activated Protein Kinase Kinase 2	Protein Coding	GC19M004090	2.130518436
SDC1	Syndecan 1	Protein Coding	GC02M020200	2.128703117
RAD51	RAD51 Recombinase	Protein Coding	GC15P040694	2.128495455
SSR2	Signal Sequence Receptor Subunit 2	Protein Coding	GC01M156009	2.125382423
CHD4	Chromodomain Helicase DNA Binding Protein 4	Protein Coding	GC12M006570	2.124853611
LOX	Lysyl Oxidase	Protein Coding	GC05M122063	2.119800091
CYP7A1	Cytochrome P450 Family 7 Subfamily A Member 1	Protein Coding	GC08M058476	2.119164467
ARG1	Arginase 1	Protein Coding	GC06P131473	2.118257284
HTR2A	5-Hydroxytryptamine Receptor 2A	Protein Coding	GC13M046831	2.115084171
GSDMA	Gasdermin A	Protein Coding	GC17P039962	2.111245394
FLG	Filaggrin	Protein Coding	GC01M152274	2.110759974
IL24	Interleukin 24	Protein Coding	GC01P206897	2.110621452
APOB	Apolipoprotein B	Protein Coding	GC02M020956	2.109253883
MIRLET7E	MicroRNA Let-7e	RNA Gene	GC19P051718	2.1052742
FKBP5	FKBP Prolyl Isomerase 5	Protein Coding	GC06M049256	2.103704453
LMAN1	Lectin, Mannose Binding 1	Protein Coding	GC18M059327	2.103704453
SERPINF1	Serpin Family F Member 1	Protein Coding	GC17P001761	2.103704453
GTF2E2	General Transcription Factor IIE Subunit 2	Protein Coding	GC08M030578	2.103704453
GAS7	Growth Arrest Specific 7	Protein Coding	GC17M009910	2.103704453
PSD	Pleckstrin And Sec7 Domain	Protein Coding	GC10M102403	2.103704453
CADM2	Cell Adhesion Molecule 2	Protein Coding	GC03P085008	2.103704453
S100Z	S100 Calcium Binding Protein Z	Protein Coding	GC05P076850	2.103704453
PRAC2	PRAC2 Small Nuclear Protein	Protein Coding	GC17P048720	2.103704453
BLVRB	Biliverdin Reductase B	Protein Coding	GC19M040447	2.100826263
ITGB7	Integrin Subunit Beta 7	Protein Coding	GC12M053191	2.098919868
TERF2	Telomeric Repeat Binding Factor 2	Protein Coding	GC16M069355	2.098795652
CD1D	CD1d Molecule	Protein Coding	GC01P158178	2.098681927
SHC1	SHC Adaptor Protein 1	Protein Coding	GC01M154962	2.097476482
EZR	Ezrin	Protein Coding	GC06M158765	2.092067719
TACR2	Tachykinin Receptor 2	Protein Coding	GC10M069403	2.089029312
MTMR3	Myotubularin Related Protein 3	Protein Coding	GC22P029885	2.08787632
HORMAD2	HORMA Domain Containing 2	Protein Coding	GC22P030080	2.08787632
SLC3A2	Solute Carrier Family 3 Member 2	Protein Coding	GC11P062856	2.085040092
LTB	Lymphotoxin Beta	Protein Coding	GC06M049117	2.084350109
USP25	Ubiquitin Specific Peptidase 25	Protein Coding	GC21P015730	2.084311485
SLPI	Secretory Leukocyte Peptidase	Protein Coding	GC20M045252	2.082861185
ETS2	ETS Proto-Oncogene 2, Transcription Factor	Protein Coding	GC21P038805	2.079563141
LBP	Lipopolysaccharide Binding Protein	Protein Coding	GC20P038346	2.07638216
SYNE1	Spectrin Repeat Containing Nuclear Envelope Protein 1	Protein Coding	GC06M152121	2.07586813
DHFR	Dihydrofolate Reductase	Protein Coding	GC05M080626	2.074714661
NR0B2	Nuclear Receptor Subfamily 0 Group B Member 2	Protein Coding	GC01M026922	2.071870089
PTK2	Protein Tyrosine Kinase 2	Protein Coding	GC08M140657	2.071744204
HTR1A	5-Hydroxytryptamine Receptor 1A	Protein Coding	GC05M063960	2.069051504
CBL	Cbl Proto-Oncogene	Protein Coding	GC11P119206	2.068938494
TMPO	Thymopoietin	Protein Coding	GC12P098515	2.068938494

MIR30C1	MicroRNA 30c-1	RNA Gene	GC01P040757	2.065024853
DACT1	Dishevelled Binding Antagonist Of Beta Catenin 1	Protein Coding	GC14P058633	2.063565254
RAB7B	RAB7B, Member RAS Oncogene	Protein Coding	GC01M205976	2.063565254
UBE2N	Ubiquitin Conjugating Enzyme E2 N	Protein Coding	GC12M093406	2.05708456
PTGDR2	Prostaglandin D2 Receptor 2	Protein Coding	GC11M060850	2.055088282
GPANK1	G-Patch Domain And Ankyrin Repeats	Protein Coding	GC06M049120	2.054177284
RAG2	Recombination Activating 2	Protein Coding	GC11M036575	2.053284645
MIR9-1	MicroRNA 9-1	RNA Gene	GC01M156420	2.050828457
ARRB1	Arrestin Beta 1	Protein Coding	GC11M075261	2.046729326
SLC2A1	Solute Carrier Family 2 Member 1	Protein Coding	GC01M042925	2.041882753
CTBP1	C-Terminal Binding Protein 1	Protein Coding	GC04M001211	2.041470528
SGF29	SAGA Complex Associated Factor 29	Protein Coding	GC16P033197	2.041065693
ERCC2	ERCC Excision Repair 2, TFIIH Core Complex Helicase Subunit	Protein Coding	GC19M045349	2.040863752
KL	Klotho	Protein Coding	GC13P033016	2.039370775
IFNAR2	Interferon Alpha And Beta Receptor Subunit 2	Protein Coding	GC21P033229	2.03457737
ALPP	Alkaline Phosphatase, Placental	Protein Coding	GC02P232378	2.03377676
VDAC1	Voltage Dependent Anion Channel 1	Protein Coding	GC05M133975	2.031398296
EDNRA	Endothelin Receptor Type A	Protein Coding	GC04P147480	2.031162024
MLNR	Motilin Receptor	Protein Coding	GC13P049220	2.027945518
AMBP	Alpha-1-Microglobulin/Bikunin Precursor	Protein Coding	GC09M114060	2.020079613
SEPSECS	Sep (O-Phosphoserine) TRNA:Sec (Selenocysteine) TRNA Synthase	Protein Coding	GC04M025121	2.016329765
TICAM1	Toll Like Receptor Adaptor Molecule 1	Protein Coding	GC19M004815	2.015293121
RAB11A	RAB11A, Member RAS Oncogene Family	Protein Coding	GC15P078986	2.013477802
SETDB1	SET Domain Bifurcated Histone Lysine Methyltransferase 1	Protein Coding	GC01P150926	2.010756493
SLC39A8	Solute Carrier Family 39 Member 8	Protein Coding	GC04M102252	2.008620262
LALBA	Lactalbumin Alpha	Protein Coding	GC12M048567	2.007785559
GIMAP5	GTPase, IMAP Family Member 5	Protein Coding	GC07P150722	2.005193949
E2F1	E2F Transcription Factor 1	Protein Coding	GC20M033675	2.00453949
MCCD1	Mitochondrial Coiled-Coil Domain 1	Protein Coding	GC06P031528	2.003526926
MCM2	Minichromosome Maintenance Complex Component 2	Protein Coding	GC03P127598	1.996366024
NAGLU	N-Acetyl-Alpha-Glucosaminidase	Protein Coding	GC17P042543	1.993469
MIR22	MicroRNA 22	RNA Gene	GC17M001713	1.992949247
REG1B	Regenerating Family Member 1 Beta	Protein Coding	GC02M079086	1.989919782
EIF2S1	Eukaryotic Translation Initiation Factor 2 Subunit Alpha	Protein Coding	GC14P067359	1.988876581
NEAT1	Nuclear Paraspeckle Assembly Transcript 1	RNA Gene	GC11P066811	1.987593174
MIR139	MicroRNA 139	RNA Gene	GC11M072615	1.987103462
MIR338	MicroRNA 338	RNA Gene	GC17M081126	1.987103462
CGN	Cingulin	Protein Coding	GC01P151483	1.986323595
S100A1	S100 Calcium Binding Protein A1	Protein Coding	GC01P153627	1.985487819
IL32	Interleukin 32	Protein Coding	GC16P006225	1.985274196
FAP	Fibroblast Activation Protein Alpha	Protein Coding	GC02M162170	1.982104301
PRODH	Proline Dehydrogenase 1	Protein Coding	GC22M018912	1.981699228
MMP26	Matrix Metallopeptidase 26	Protein Coding	GC11P004706	1.975459814
ARFRP1	ADP Ribosylation Factor Related Protein 1	Protein Coding	GC20M063698	1.975030303
PLIN2	Perilipin 2	Protein Coding	GC09M019131	1.974544644
PSMB10	Proteasome 20S Subunit Beta 10	Protein Coding	GC16M067937	1.970457554
MIR204	MicroRNA 204	RNA Gene	GC09M070809	1.968764186
AQP4	Aquaporin 4	Protein Coding	GC18M026852	1.964419603
AQP7	Aquaporin 7	Protein Coding	GC09M033384	1.964419603
FZD8	Frizzled Class Receptor 8	Protein Coding	GC10M035638	1.964197636
MUS81	MUS81 Structure-Specific Endonuclease Subunit	Protein Coding	GC11P066858	1.963240147

REN	Renin	Protein Coding	GC01M204154	1.962740302
ST6GALNAC1	ST6 N-Acetylgalactosaminide Alpha-2,6-Sialyltransferase 1	Protein Coding	GC17M076624	1.961085558
MAFK	MAF BZIP Transcription Factor K	Protein Coding	GC07P001531	1.961085558
P2RX7	Purinergic Receptor P2X 7	Protein Coding	GC12P123863	1.95437932
SLC37A1	Solute Carrier Family 37 Member 1	Protein Coding	GC21P042536	1.951966405
PLEK	Pleckstrin	Protein Coding	GC02P068365	1.951432228
SERPINB2	Serpin Family B Member 2	Protein Coding	GC18P063871	1.943588853
TOP1	DNA Topoisomerase I	Protein Coding	GC20P041028	1.940956235
BECN1	Beclin 1	Protein Coding	GC17M042810	1.935521603
CD3D	CD3d Molecule	Protein Coding	GC11M118338	1.933153391
CYP24A1	Cytochrome P450 Family 24 Subfamily A Member 1	Protein Coding	GC20M054153	1.932068348
SLC11A2	Solute Carrier Family 11 Member 2	Protein Coding	GC12M050952	1.931959867
CASP5	Caspase 5	Protein Coding	GC11M104995	1.931750178
MIR99A	MicroRNA 99a	RNA Gene	GC21P016539	1.931157112
MIR18A	MicroRNA 18a	RNA Gene	GC13P091475	1.931157112
NBN	Nibrin	Protein Coding	GC08M089933	1.93057549
LGALS3BP	Galectin 3 Binding Protein	Protein Coding	GC17M078971	1.928995728
SLAMF6	SLAM Family Member 6	Protein Coding	GC01M160454	1.92863071
FLI1	Fli-1 Proto-Oncogene, ETS Transcription Factor	Protein Coding	GC11P128686	1.928262949
LACTB	Lactamase Beta	Protein Coding	GC15P079621	1.926800013
FURIN	Furin, Paired Basic Amino Acid Cleaving Enzyme	Protein Coding	GC15P090868	1.924675822
SIGLEC5	Sialic Acid Binding Ig Like Lectin 5	Protein Coding	GC19M051611	1.917483091
MMP11	Matrix Metallopeptidase 11	Protein Coding	GC22P023768	1.917171717
PRKCB	Protein Kinase C Beta	Protein Coding	GC16P023993	1.915290236
CNTF	Ciliary Neurotrophic Factor	Protein Coding	GC11P058622	1.915290236
LEF1	Lymphoid Enhancer Binding Factor 1	Protein Coding	GC04M108047	1.914534926
SOX2	SRY-Box Transcription Factor 2	Protein Coding	GC03P181711	1.914534926
HDAC1	Histone Deacetylase 1	Protein Coding	GC01P032292	1.913936853
ATG5	Autophagy Related 5	Protein Coding	GC06M106045	1.911211491
KCNQ1OT1	KCNQ1 Opposite Strand/Antisense Transcript 1	RNA Gene	GC11M002661	1.911211491
PVT1	Pvt1 Oncogene	RNA Gene	GC08P127827	1.911211491
HOXA11-AS	HOXA11 Antisense RNA	RNA Gene	GC07P027184	1.911211491
ABCB11	ATP Binding Cassette Subfamily B Member 11	Protein Coding	GC02M168922	1.910826445
TPM1	Tropomyosin 1	Protein Coding	GC15P080570	1.909552336
RNF5	Ring Finger Protein 5	Protein Coding	GC06P058337	1.90920496
WNT3A	Wnt Family Member 3A	Protein Coding	GC01P228554	1.90908432
UGT1A7	UDP Glucuronosyltransferase Family 1 Member A7	Protein Coding	GC02P233681	1.904358268
TKT	Transketolase	Protein Coding	GC03M053224	1.902330995
MIR133A1	MicroRNA 133a-1	RNA Gene	GC18M021826	1.901090503
SULT2A1	Sulfotransferase Family 2A Member 1	Protein Coding	GC19M047870	1.89877367
SLC10A2	Solute Carrier Family 10 Member 2	Protein Coding	GC13M103043	1.89877367
USF1	Upstream Transcription Factor 1	Protein Coding	GC01M161039	1.896929026
PLCL1	Phospholipase C Like 1 (Inactive)	Protein Coding	GC02P197804	1.896929026
RMI2	RecQ Mediated Genome Instability 2	Protein Coding	GC16P011250	1.896929026
ARHGAP45	Rho GTPase Activating Protein 45	Protein Coding	GC19P001065	1.896929026
CISD1	CDGSH Iron Sulfur Domain 1	Protein Coding	GC10P058269	1.895721912
ALDOB	Aldolase, Fructose-Bisphosphate B	Protein Coding	GC09M101420	1.892050743
SULT1A3	Sulfotransferase Family 1A Member 3	Protein Coding	GC16P030199	1.890680194
MIR16-1	MicroRNA 16-1	RNA Gene	GC13M050048	1.890680194
PPBP	Pro-Platelet Basic Protein	Protein Coding	GC04M073986	1.889178157
GPX4	Glutathione Peroxidase 4	Protein Coding	GC19P001103	1.885655284
ITIH4	Inter-Alpha-Trypsin Inhibitor Heavy Chain 4	Protein Coding	GC03M052812	1.885655284
CEP250	Centrosomal Protein 250	Protein Coding	GC20P035455	1.885655284
LIME1	Lck Interacting Transmembrane	Protein Coding	GC20P063736	1.885655284
WNT3	Wnt Family Member 3	Protein Coding	GC17M046762	1.884103179

KLK3	Kallikrein Related Peptidase 3	Protein Coding	GC19P050854	1.881061554
DDX39B	DExD-Box Helicase 39B	Protein Coding	GC06M031530	1.880697608
ACHE	Acetylcholinesterase (Cartwright Blood Group)	Protein Coding	GC07M100889	1.878092885
ENPP7	Ectonucleotide Pyrophosphatase/Phosphodiesterase 7	Protein Coding	GC17P079730	1.877661943
ADCY7	Adenylate Cyclase 7	Protein Coding	GC16P050423	1.876311421
CLN3	CLN3 Lysosomal/Endosomal Transmembrane Protein, Battenin	Protein Coding	GC16M028466	1.876311421
ATXN2L	Ataxin 2 Like	Protein Coding	GC16P033255	1.876311421
MIR23B	MicroRNA 23b	RNA Gene	GC09P095085	1.87031889
MIR455	MicroRNA 455	RNA Gene	GC09P114209	1.87031889
MIR675	MicroRNA 675	RNA Gene	GC11M001997	1.87031889
LAG3	Lymphocyte Activating 3	Protein Coding	GC12P013176	1.868380427
RNF128	Ring Finger Protein 128	Protein Coding	GC0XP106693	1.867845893
C1R	Complement C1r	Protein Coding	GC12M007296	1.867804646
C2	Complement C2	Protein Coding	GC06P031897	1.867804646
PRDX4	Peroxiredoxin 4	Protein Coding	GC0XP023665	1.867601871
ATF3	Activating Transcription Factor 3	Protein Coding	GC01P212565	1.866605401
KHDRBS1	KH RNA Binding Domain Containing, Signal Transduction Associated 1	Protein Coding	GC01P032013	1.866439104
DRD2	Dopamine Receptor D2	Protein Coding	GC11M113409	1.864715695
APOA4	Apolipoprotein A4	Protein Coding	GC11M116820	1.862760067
SLC6A14	Solute Carrier Family 6 Member 14	Protein Coding	GC0XP116436	1.861692667
KHDRBS3	KH RNA Binding Domain Containing, Signal Transduction Associated 3	Protein Coding	GC08P135457	1.861692667
MAPK9	Mitogen-Activated Protein Kinase 9	Protein Coding	GC05M180235	1.861400843
ACP1	Acid Phosphatase 1	Protein Coding	GC02P000254	1.861366749
FCGRT	Fc Fragment Of IgG Receptor And Transporter	Protein Coding	GC19P049506	1.861366749
PIK3CB	Phosphatidylinositol-4,5-Bisphosphate 3-Kinase Catalytic Subunit Beta	Protein Coding	GC03M138652	1.860403061
CCR7	C-C Motif Chemokine Receptor 7	Protein Coding	GC17M040556	1.858063459
DSG2	Desmoglein 2	Protein Coding	GC18P031498	1.857569098
CLK2	CDC Like Kinase 2	Protein Coding	GC01M155262	1.857053757
FDX1	Ferredoxin 1	Protein Coding	GC11P110429	1.85658145
XPO1	Exportin 1	Protein Coding	GC02M061445	1.855515957
RPL24	Ribosomal Protein L24	Protein Coding	GC03M101681	1.85228467
NCR3	Natural Cytotoxicity Triggering Receptor 3	Protein Coding	GC06M031588	1.849273682
IRS2	Insulin Receptor Substrate 2	Protein Coding	GC13M109752	1.847625613
PRKCA	Protein Kinase C Alpha	Protein Coding	GC17P066302	1.84389627
ADCYAP1	Adenylate Cyclase Activating Polypeptide 1	Protein Coding	GC18P000895	1.842970371
MRC1	Mannose Receptor C-Type 1	Protein Coding	GC10P017809	1.841296196
PMM2	Phosphomannomutase 2	Protein Coding	GC16P008788	1.839913845
HLA-DOA	Major Histocompatibility Complex, Class II, DO Alpha	Protein Coding	GC06M033004	1.836220026
MAP3K8	Mitogen-Activated Protein Kinase Kinase Kinase 8	Protein Coding	GC10P030458	1.836209774
RAF1	Raf-1 Proto-Oncogene, Serine/Threonine Kinase	Protein Coding	GC03M012583	1.833784342
ADH1B	Alcohol Dehydrogenase 1B (Class I), Beta Polypeptide	Protein Coding	GC04M099304	1.830739141
EPHX2	Epoxide Hydrolase 2	Protein Coding	GC08P027490	1.829595804
CYP2J2	Cytochrome P450 Family 2 Subfamily J Member 2	Protein Coding	GC01M059893	1.829595804
IL17RD	Interleukin 17 Receptor D	Protein Coding	GC03M057089	1.829595804
CDH2	Cadherin 2	Protein Coding	GC18M027950	1.825088859
MAP2K5	Mitogen-Activated Protein Kinase Kinase 5	Protein Coding	GC15P078990	1.820608616
TCF3	Transcription Factor 3	Protein Coding	GC19M001609	1.820608616

MAPRE1	Microtubule Associated Protein RP/EB Family Member 1	Protein Coding	GC20P032819	1.820608616
NUDT1	Nudix Hydrolase 1	Protein Coding	GC07P002242	1.820608616
SF3A1	Splicing Factor 3a Subunit 1	Protein Coding	GC22M030331	1.820608616
CDK5RAP1	CDK5 Regulatory Subunit Associated Protein 1	Protein Coding	GC20M033358	1.820608616
SDF4	Stromal Cell Derived Factor 4	Protein Coding	GC01M001216	1.820608616
HOTAIRM1	HOXA Transcript Antisense RNA, Myeloid-Specific 1	RNA Gene	GC07P027095	1.820608616
SCARNA5	Small Cajal Body-Specific RNA 5	RNA Gene	GC02P233275	1.820608616
LINC00460	Long Intergenic Non-Protein Coding RNA 460	RNA Gene	GC13P106376	1.820608616
CEBPB	CCAAT Enhancer Binding Protein Beta	Protein Coding	GC20P050190	1.820377588
ANXA7	Annexin A7	Protein Coding	GC10M073375	1.819440246
DDX58	DExD/H-Box Helicase 58	Protein Coding	GC09M032455	1.815336823
SIKE1	Suppressor Of IKBKE 1	Protein Coding	GC01M114769	1.812685013
CTNND1	Catenin Delta 1	Protein Coding	GC11P057904	1.811689377
FCRL3	Fc Receptor Like 3	Protein Coding	GC01M157674	1.810165644
ELF3	E74 Like ETS Transcription Factor 3	Protein Coding	GC01P202007	1.808282137
MSN	Moesin	Protein Coding	GC0XP065588	1.807069421
CTDSPL	CTD Small Phosphatase Like	Protein Coding	GC03P037861	1.80589056
ABCB4	ATP Binding Cassette Subfamily B Member 4	Protein Coding	GC07M087401	1.805534005
MIR193B	MicroRNA 193b	RNA Gene	GC16P014307	1.803025246
MIR28	MicroRNA 28	RNA Gene	GC03P188688	1.803025246
SLCO1B3	Solute Carrier Organic Anion Transporter Family Member 1B3	Protein Coding	GC12P020810	1.802981138
ADAM15	ADAM Metallopeptidase Domain 15	Protein Coding	GC01P155023	1.796360731
STX2	Syntaxin 2	Protein Coding	GC12M130789	1.795701504
CASP14	Caspase 14	Protein Coding	GC19P015049	1.795421839
ACADS	Acyl-CoA Dehydrogenase Short Chain	Protein Coding	GC12P120947	1.79426384
MAPKAPK2	MAPK Activated Protein Kinase 2	Protein Coding	GC01P206684	1.790742636
PPP5C	Protein Phosphatase 5 Catalytic Subunit	Protein Coding	GC19P046346	1.790742636
MSTO1	Misato Mitochondrial Distribution And Morphology Regulator 1	Protein Coding	GC01P155610	1.790742636
ZPBP	Zona Pellucida Binding Protein	Protein Coding	GC07M049850	1.790742636
TRIB1	Tribbles Pseudokinase 1	Protein Coding	GC08P125430	1.790742636
TSPAN33	Tetraspanin 33	Protein Coding	GC07P130275	1.790742636
HSD17B10	Hydroxysteroid 17-Beta Dehydrogenase 10	Protein Coding	GC0XM053431	1.790727258
FGF19	Fibroblast Growth Factor 19	Protein Coding	GC11M071943	1.788080692
RHOH	Ras Homolog Family Member H	Protein Coding	GC04P040192	1.787430525
ATP6V0A1	ATPase H+ Transporting V0 Subunit	Protein Coding	GC17P042458	1.787165761
CAV3	Caveolin 3	Protein Coding	GC03P008733	1.786130667
PTGDS	Prostaglandin D2 Synthase	Protein Coding	GC09P137013	1.785979033
GAS5	Growth Arrest Specific 5	RNA Gene	GC01M173947	1.781687498
ALK	ALK Receptor Tyrosine Kinase	Protein Coding	GC02M029190	1.781192064
RASSF1	Ras Association Domain Family Member 1	Protein Coding	GC03M050329	1.777735233
IGES	Immunoglobulin E Concentration,	Genetic Locus	GC05U990033	1.774606347
HEY2	Hes Related Family BHLH Transcription Factor With YRPW	Protein Coding	GC06P125730	1.773209214
TNC	Tenascin C	Protein Coding	GC09M115019	1.772827148
CDK1	Cyclin Dependent Kinase 1	Protein Coding	GC10P060772	1.770136833
IRF4	Interferon Regulatory Factor 4	Protein Coding	GC06P000391	1.769625545
PSTPIP2	Proline-Serine-Threonine Phosphatase Interacting Protein 2	Protein Coding	GC18M045983	1.769500732
TMPRSS6	Transmembrane Serine Protease 6	Protein Coding	GC22M037066	1.764622927
SLC7A5	Solute Carrier Family 7 Member 5	Protein Coding	GC16M087830	1.760798454
EWSR1	EWS RNA Binding Protein 1	Protein Coding	GC22P029269	1.760145426
THBS1	Thrombospondin 1	Protein Coding	GC15P039581	1.757929087
FLVCR2	FLVCR Heme Transporter 2	Protein Coding	GC14P075578	1.757172346
SLC48A1	Solute Carrier Family 48 Member 1	Protein Coding	GC12P047753	1.757172346

KLKB1	Kallikrein B1	Protein Coding	GC04P186208	1.756485224
MYH11	Myosin Heavy Chain 11	Protein Coding	GC16M015704	1.7536062
AKR1B1	Aldo-Keto Reductase Family 1 Member B	Protein Coding	GC07M134442	1.750640988
ZNF148	Zinc Finger Protein 148	Protein Coding	GC03M125225	1.749709845
IGFBP2	Insulin Like Growth Factor Binding Protein 2	Protein Coding	GC02P216632	1.747246504
DSG3	Desmoglein 3	Protein Coding	GC18P031447	1.741838932
MAF	MAF BZIP Transcription Factor	Protein Coding	GC16M079204	1.741639614
TBXAS1	Thromboxane A Synthase 1	Protein Coding	GC07P139777	1.738806963
CYSLTR1	Cysteinyl Leukotriene Receptor 1	Protein Coding	GC0XM078271	1.731501698
NCAPD3	Non-SMC Condensin II Complex Subunit D3	Protein Coding	GC11M134150	1.731163383
BCL2L12	BCL2 Like 12	Protein Coding	GC19P049782	1.731163383
IL22RA2	Interleukin 22 Receptor Subunit Alpha	Protein Coding	GC06M137143	1.726198673
CYLD-AS1	CYLD Antisense RNA 1	RNA Gene	GC16M050736	1.725029588
LGALS2	Galectin 2	Protein Coding	GC22M037570	1.723839521
GGH	Gamma-Glutamyl Hydrolase	Protein Coding	GC08M063015	1.72284615
CCR4	C-C Motif Chemokine Receptor 4	Protein Coding	GC03P032951	1.719776392
STC1	Stanniocalcin 1	Protein Coding	GC08M023841	1.718780518
CFP	Complement Factor Properdin	Protein Coding	GC0XM047624	1.715967178
FOSL2	FOS Like 2, AP-1 Transcription Factor Subunit	Protein Coding	GC02P028392	1.712285876
TAGAP	T Cell Activation RhoGTPase Activating Protein	Protein Coding	GC06M159034	1.712285876
TNFSF18	TNF Superfamily Member 18	Protein Coding	GC01M173009	1.712285876
AXIN1	Axin 1	Protein Coding	GC16M000287	1.712035537
LOXL2	Lysyl Oxidase Like 2	Protein Coding	GC08M023296	1.711699009
SLC10A1	Solute Carrier Family 10 Member 1	Protein Coding	GC14M069775	1.711699009
CYP8B1	Cytochrome P450 Family 8 Subfamily B Member 1	Protein Coding	GC03M042856	1.711699009
DNAL1	Dynein Axonemal Light Chain 1	Protein Coding	GC14P073644	1.711699009
SP100	SP100 Nuclear Antigen	Protein Coding	GC02P230415	1.711699009
SCTR	Secretin Receptor	Protein Coding	GC02M119439	1.711699009
SLC51A	Solute Carrier Family 51 Subunit Alpha	Protein Coding	GC03P196211	1.711699009
WNT5A	Wnt Family Member 5A	Protein Coding	GC03M055465	1.711440206
LDHA	Lactate Dehydrogenase A	Protein Coding	GC11P018394	1.709502459
MIR124-3	MicroRNA 124-3	RNA Gene	GC20P063180	1.708860397
PLK1	Polo Like Kinase 1	Protein Coding	GC16P023988	1.708205342
FOXF1	Forkhead Box F1	Protein Coding	GC16P086510	1.706498623
CAMK2G	Calcium/Calmodulin Dependent Protein Kinase II Gamma	Protein Coding	GC10M073812	1.705773592
DSG1	Desmoglein 1	Protein Coding	GC18P031318	1.704237342
UBC	Ubiquitin C	Protein Coding	GC12M124911	1.700921297
PMEL	Premelanosome Protein	Protein Coding	GC12M055954	1.700859547
CCNB1	Cyclin B1	Protein Coding	GC05P069167	1.694582343
MIR10B	MicroRNA 10b	RNA Gene	GC02P176150	1.694582343
IL21R	Interleukin 21 Receptor	Protein Coding	GC16P027413	1.694016218
VNN1	Vanin 1	Protein Coding	GC06M132680	1.688174725
IL17C	Interleukin 17C	Protein Coding	GC16P088638	1.68524313
PPL	Periplakin	Protein Coding	GC16M004872	1.683991909
TRPA1	Transient Receptor Potential Cation Channel Subfamily A Member 1	Protein Coding	GC08M072019	1.682691813
POSTN	Periostin	Protein Coding	GC13M037562	1.68105197
MEP1A	Meprin A Subunit Alpha	Protein Coding	GC06P046793	1.677364826
IL36A	Interleukin 36 Alpha	Protein Coding	GC02P113005	1.676723719
DDC	Dopa Decarboxylase	Protein Coding	GC07M050458	1.676619411
RARG	Retinoic Acid Receptor Gamma	Protein Coding	GC12M053210	1.674618244
GABPA	GA Binding Protein Transcription Factor Subunit Alpha	Protein Coding	GC21P025734	1.674618244
IL1F10	Interleukin 1 Family Member 10	Protein Coding	GC02P113067	1.674618244
IL36B	Interleukin 36 Beta	Protein Coding	GC02M113022	1.674618244
RPL35P3	Ribosomal Protein L35 Pseudogene 3	Pseudogene	GC06M105302	1.674618244

MIR27B	MicroRNA 27b	RNA Gene	GC09P095097	1.674525976
RBP3	Retinol Binding Protein 3	Protein Coding	GC10P047348	1.673545599
AR	Androgen Receptor	Protein Coding	GC0XP067544	1.672779322
IKBKE	Inhibitor Of Nuclear Factor Kappa B Kinase Subunit Epsilon	Protein Coding	GC01P206470	1.66690588
ASH1L	ASH1 Like Histone Lysine Methyltransferase	Protein Coding	GC01M155335	1.66690588
SETD1A	SET Domain Containing 1A, Histone Lysine Methyltransferase	Protein Coding	GC16P033497	1.66690588
POLA2	DNA Polymerase Alpha 2, Accessory Subunit	Protein Coding	GC11P065359	1.66690588
USP19	Ubiquitin Specific Peptidase 19	Protein Coding	GC03M049514	1.66690588
NRBP1	Nuclear Receptor Binding Protein 1	Protein Coding	GC02P027427	1.66690588
ATG2A	Autophagy Related 2A	Protein Coding	GC11M071625	1.66690588
ST7	Suppression Of Tumorigenicity 7	Protein Coding	GC07P117043	1.66690588
CD3G	CD3g Molecule	Protein Coding	GC11P118344	1.666325212
ALG3	ALG3 Alpha-1,3- Mannosyltransferase	Protein Coding	GC03M184244	1.666325212
BICD1	BICD Cargo Adaptor 1	Protein Coding	GC12P032107	1.666325212
TNRC6A	Trinucleotide Repeat Containing Adaptor 6A	Protein Coding	GC16P024611	1.666325212
PSMC3IP	PSMC3 Interacting Protein	Protein Coding	GC17M042572	1.666325212
NEK5	NIMA Related Kinase 5	Protein Coding	GC13M052033	1.666325212
VWA5A	Von Willebrand Factor A Domain Containing 5A	Protein Coding	GC11P124115	1.666325212
FEN1	Flap Structure-Specific Endonuclease 1	Protein Coding	GC11P061793	1.66144371
AKR1A1	Aldo-Keto Reductase Family 1 Member A1	Protein Coding	GC01P045550	1.660789371
C1GALT1C1	C1GALT1 Specific Chaperone 1	Protein Coding	GC0XM120625	1.660672903
ANKRD55	Ankyrin Repeat Domain 55	Protein Coding	GC05M056099	1.660053968
INTS11	Integrator Complex Subunit 11	Protein Coding	GC01M003078	1.660053968
ILK	Integrin Linked Kinase	Protein Coding	GC11P006604	1.656470299
GALC	Galactosylceramidase	Protein Coding	GC14M087837	1.655213237
CTSW	Cathepsin W	Protein Coding	GC11P065879	1.655213237
NDUFA13	NADH:Ubiquinone Oxidoreductase Subunit A13	Protein Coding	GC19P019515	1.654727936
EPN3	Epsin 3	Protein Coding	GC17P050532	1.654727936
SERPINB5	Serpin Family B Member 5	Protein Coding	GC18P063476	1.652066946
CXCR5	C-X-C Motif Chemokine Receptor 5	Protein Coding	GC11P118912	1.650226951
ANXA2	Annexin A2	Protein Coding	GC15M060347	1.640787125
LAMP1	Lysosomal Associated Membrane Protein 1	Protein Coding	GC13P113297	1.640444279
SIGIRR	Single Ig And TIR Domain Containing	Protein Coding	GC11M001293	1.640299201
TRB	T Cell Receptor Beta Locus	Protein Coding	GC07P146323	1.640063763
MTMR2	Myotubularin Related Protein 2	Protein Coding	GC11M095822	1.639096379
NSD1	Nuclear Receptor Binding SET Domain Protein 1	Protein Coding	GC05P177134	1.639096379
CLEC16A	C-Type Lectin Domain Containing 16A	Protein Coding	GC16P010944	1.638628721
MMP19	Matrix Metallopeptidase 19	Protein Coding	GC12M055835	1.634971142
ERN1	Endoplasmic Reticulum To Nucleus Signaling 1	Protein Coding	GC17M064039	1.634426713
HHEX	Hematopoietically Expressed	Protein Coding	GC10P092689	1.634426713
KSR1	Kinase Suppressor Of Ras 1	Protein Coding	GC17P027456	1.630419016
FAAH	Fatty Acid Amide Hydrolase	Protein Coding	GC01P046394	1.630164385
MIR181A1	MicroRNA 181a-1	RNA Gene	GC01M198860	1.629449368
DEFA1	Defensin Alpha 1	Protein Coding	GC08M006977	1.629204512
ZEB1	Zinc Finger E-Box Binding Homeobox	Protein Coding	GC10P031318	1.628409982
HTRA1	HtrA Serine Peptidase 1	Protein Coding	GC10P122461	1.624491692
RPS2P34	Ribosomal Protein S2 Pseudogene 34	Pseudogene	GC09P082130	1.624491692
LOC442427	Putative UPF0607 Protein ENSP00000381418	Pseudogene	GC09M082431	1.624491692
RUNX2	RUNX Family Transcription Factor 2	Protein Coding	GC06P058536	1.622858286
RPL6	Ribosomal Protein L6	Protein Coding	GC12M112320	1.622457504
ZNF354B	Zinc Finger Protein 354B	Protein Coding	GC05P178859	1.621096134

MIR423	MicroRNA 423	RNA Gene	GC17P030117	1.620550275
MIR29B1	MicroRNA 29b-1	RNA Gene	GC07M130877	1.619558811
WNT1	Wnt Family Member 1	Protein Coding	GC12P049053	1.61820066
BAK1	BCL2 Antagonist/Killer 1	Protein Coding	GC06M033572	1.61820066
TCF7	Transcription Factor 7	Protein Coding	GC05P134114	1.617602587
TIAM1	TIAM Rac1 Associated GEF 1	Protein Coding	GC21M031118	1.617602587
CDCP1	CUB Domain Containing Protein 1	Protein Coding	GC03M045082	1.617602587
RECK	Reversion Inducing Cysteine Rich Protein With Kazal Motifs	Protein Coding	GC09P036036	1.613534689
NAMPT	Nicotinamide	Protein Coding	GC07M106248	1.610750675
CA1	Carbonic Anhydrase 1	Protein Coding	GC08M085327	1.61042881
CFHR2	Complement Factor H Related 2	Protein Coding	GC01P196943	1.610195398
KIR3DL2	Killer Cell Immunoglobulin Like Receptor, Three Ig Domains And Long Cytoplasmic Tail 2	Protein Coding	GC19P057015	1.609590292
MDC1	Mediator Of DNA Damage Checkpoint	Protein Coding	GC06M049038	1.608512521
EED	Embryonic Ectoderm Development	Protein Coding	GC11P086244	1.608278275
MAGEA1	MAGE Family Member A1	Protein Coding	GC0XP153179	1.607881665
HLA-DMB	Major Histocompatibility Complex, Class II, DM Beta	Protein Coding	GC06M032934	1.607287884
HLA-DMA	Major Histocompatibility Complex, Class II, DM Alpha	Protein Coding	GC06M049187	1.607287884
HLA-DOB	Major Histocompatibility Complex, Class II, DO Beta	Protein Coding	GC06M049180	1.607287884
NTSR1	Neurotensin Receptor 1	Protein Coding	GC20P062708	1.606413245
CIDEB	Cell Death Inducing DFFA Like Effector B	Protein Coding	GC14M024305	1.603530049
VEGFC	Vascular Endothelial Growth Factor C	Protein Coding	GC04M176683	1.602744222
ADCY3	Adenylate Cyclase 3	Protein Coding	GC02M024819	1.600594759
CARD11	Caspase Recruitment Domain Family Member 11	Protein Coding	GC07M002906	1.600594759
PTGIR	Prostaglandin I2 Receptor	Protein Coding	GC19M048824	1.600594759
RPS6KA4	Ribosomal Protein S6 Kinase A4	Protein Coding	GC11P064360	1.600594759
ATF4	Activating Transcription Factor 4	Protein Coding	GC22P039558	1.600594759
HDAC7	Histone Deacetylase 7	Protein Coding	GC12M047782	1.600594759
RIT1	Ras Like Without CAAX 1	Protein Coding	GC01M155897	1.600594759
LNPEP	Leucyl And Cystinyl Aminopeptidase	Protein Coding	GC05P096935	1.600594759
CALM3	Calmodulin 3	Protein Coding	GC19P046601	1.600594759
FADS2	Fatty Acid Desaturase 2	Protein Coding	GC11P061792	1.600594759
PMPCA	Peptidase, Mitochondrial Processing Subunit Alpha	Protein Coding	GC09P136410	1.600594759
SPRY4	Sprouty RTK Signaling Antagonist 4	Protein Coding	GC05M142310	1.600594759
TNNI2	Troponin I2, Fast Skeletal Type	Protein Coding	GC11P001839	1.600594759
CD226	CD226 Molecule	Protein Coding	GC18M069831	1.600594759
MANBA	Mannosidase Beta	Protein Coding	GC04M102631	1.600594759
PNKD	PNKD Metallo-Beta-Lactamase Domain Containing	Protein Coding	GC02P218270	1.600594759
BRD7	Bromodomain Containing 7	Protein Coding	GC16M050313	1.600594759
ITPKA	Inositol-Trisphosphate 3-Kinase A	Protein Coding	GC15P041493	1.600594759
DUSP16	Dual Specificity Phosphatase 16	Protein Coding	GC12M012473	1.600594759
FIBP	FGF1 Intracellular Binding Protein	Protein Coding	GC11M071731	1.600594759
NDUFAF1	NADH:Ubiquinone Oxidoreductase Complex Assembly Factor 1	Protein Coding	GC15M041387	1.600594759
SKAP2	Src Kinase Associated Phosphoprotein	Protein Coding	GC07M026654	1.600594759
SPRED2	Sprouty Related EVH1 Domain Containing 2	Protein Coding	GC02M065307	1.600594759
CD6	CD6 Molecule	Protein Coding	GC11P060971	1.600594759
ACSL6	Acyl-CoA Synthetase Long Chain Family Member 6	Protein Coding	GC05M131949	1.600594759
CTDSP1	CTD Small Phosphatase 1	Protein Coding	GC02P218398	1.600594759
CRTC3	CREB Regulated Transcription Coactivator 3	Protein Coding	GC15P090529	1.600594759
GPR18	G Protein-Coupled Receptor 18	Protein Coding	GC13M099254	1.600594759

PDLIM4	PDZ And LIM Domain 4	Protein Coding	GC05P132257	1.600594759
LPXN	Leupaxin	Protein Coding	GC11M071338	1.600594759
SEMA6D	Semaphorin 6D	Protein Coding	GC15P047184	1.600594759
SLC7A10	Solute Carrier Family 7 Member 10	Protein Coding	GC19M033208	1.600594759
MAML2	Mastermind Like Transcriptional Coactivator 2	Protein Coding	GC11M095976	1.600594759
ZNF365	Zinc Finger Protein 365	Protein Coding	GC10P062374	1.600594759
DOK3	Docking Protein 3	Protein Coding	GC05M177501	1.600594759
CPEB4	Cytoplasmic Polyadenylation Element Binding Protein 4	Protein Coding	GC05P173888	1.600594759
FCRLA	Fc Receptor Like A	Protein Coding	GC01P161708	1.600594759
CHP1	Calcineurin Like EF-Hand Protein 1	Protein Coding	GC15P041230	1.600594759
NDFIP1	Nedd4 Family Interacting Protein 1	Protein Coding	GC05P142108	1.600594759
SLC39A11	Solute Carrier Family 39 Member 11	Protein Coding	GC17M072645	1.600594759
THADA	THADA Armadillo Repeat Containing	Protein Coding	GC02M043193	1.600594759
SFMBT1	Scm Like With Four Mbt Domains 1	Protein Coding	GC03M052913	1.600594759
SNX32	Sorting Nexin 32	Protein Coding	GC11P065833	1.600594759
ZNF300	Zinc Finger Protein 300	Protein Coding	GC05M150894	1.600594759
ARHGAP30	Rho GTPase Activating Protein 30	Protein Coding	GC01M161046	1.600594759
TMBIM1	Transmembrane BAX Inhibitor Motif Containing 1	Protein Coding	GC02M218274	1.600594759
PHTF1	Putative Homeodomain Transcription Factor 1	Protein Coding	GC01M113696	1.600594759
TSPAN14	Tetraspanin 14	Protein Coding	GC10P086023	1.600594759
DENND1B	DENN Domain Containing 1B	Protein Coding	GC01M197473	1.600594759
TM9SF4	Transmembrane 9 Superfamily Member	Protein Coding	GC20P032109	1.600594759
SNX20	Sorting Nexin 20	Protein Coding	GC16M050700	1.600594759
RFTN2	Raftlin Family Member 2	Protein Coding	GC02M197568	1.600594759
CCDC85B	Coiled-Coil Domain Containing 85B	Protein Coding	GC11P065890	1.600594759
CCDC116	Coiled-Coil Domain Containing 116	Protein Coding	GC22P027570	1.600594759
NUSAP1	Nucleolar And Spindle Associated Protein 1	Protein Coding	GC15P041514	1.600594759
TMEM50B	Transmembrane Protein 50B	Protein Coding	GC21M033432	1.600594759
YDJC	YdjC Chitooligosaccharide Deacetylase Homolog	Protein Coding	GC22M021628	1.600594759
TTYH3	Tweety Family Member 3	Protein Coding	GC07P002641	1.600594759
ZNF831	Zinc Finger Protein 831	Protein Coding	GC20P059126	1.600594759
PUSL1	Pseudouridine Synthase Like 1	Protein Coding	GC01P001308	1.600594759
NXPE4	Neurexophilin And PC-Esterase Domain Family Member 4	Protein Coding	GC11M114570	1.600594759
C10orf55	Chromosome 10 Putative Open Reading Frame 55	RNA Gene	GC10M073909	1.600594759
AHSA2P	Activator Of HSP90 ATPase Homolog 2, Pseudogene	Pseudogene	GC02P061179	1.600594759
IRF1-AS1	IRF1 Antisense RNA 1	RNA Gene	GC05P132420	1.600594759
FLJ31356	Uncharacterized Protein FLJ31356	RNA Gene	GC02M028384	1.600594759
SFRP1	Secreted Frizzled Related Protein 1	Protein Coding	GC08M041262	1.599906087
ANTXR1	ANTXR Cell Adhesion Molecule 1	Protein Coding	GC02P068977	1.599848866
TERF2IP	TERF2 Interacting Protein	Protein Coding	GC16P075647	1.596803427
DSP	Desmoplakin	Protein Coding	GC06P007541	1.595282793
HCRTR1	Hypocretin Receptor 1	Protein Coding	GC01P031587	1.594856739
TRAP1	TNF Receptor Associated Protein 1	Protein Coding	GC16M004000	1.594856739
ACTB	Actin Beta	Protein Coding	GC07M005527	1.59297955
TRIM33	Tripartite Motif Containing 33	Protein Coding	GC01M114392	1.59146142
METAP2	Methionyl Aminopeptidase 2	Protein Coding	GC12P095473	1.590016842
TRDMT1	TRNA Aspartic Acid Methyltransferase	Protein Coding	GC10M017138	1.590016842
ANG	Angiogenin	Protein Coding	GC14P022303	1.588349104
CD48	CD48 Molecule	Protein Coding	GC01M160648	1.587264299
ELAVL1	ELAV Like RNA Binding Protein 1	Protein Coding	GC19M007958	1.585820913
TP53BP1	Tumor Protein P53 Binding Protein 1	Protein Coding	GC15M043403	1.58424902
CEACAM7	CEA Cell Adhesion Molecule 7	Protein Coding	GC19M041673	1.580926418
CXCL13	C-X-C Motif Chemokine Ligand 13	Protein Coding	GC04P077511	1.57572639
MIR301A	MicroRNA 301a	RNA Gene	GC17M059151	1.570111752

GZMM	Granzyme M	Protein Coding	GC19P000544	1.569410563
FAF1	Fas Associated Factor 1	Protein Coding	GC01M050439	1.567213297
MIR132	MicroRNA 132	RNA Gene	GC17M002049	1.566705465
NPHP1	Nephrocystin 1	Protein Coding	GC02M110122	1.565032482
PSMD4	Proteasome 26S Subunit Ubiquitin Receptor, Non-ATPase 4	Protein Coding	GC01P151256	1.565032482
ABCB8	ATP Binding Cassette Subfamily B Member 8	Protein Coding	GC07P151028	1.565032482
TNFRSF10C	TNF Receptor Superfamily Member	Protein Coding	GC08P023102	1.565032482
NACA	Nascent Polypeptide Associated Complex Subunit Alpha	Protein Coding	GC12M056712	1.565032482
CSNK1A1L	Casein Kinase 1 Alpha 1 Like	Protein Coding	GC13M037103	1.565032482
FAM215A	Family With Sequence Similarity 215 Member A	RNA Gene	GC17P043917	1.565032482
BORCS8-MEF2B	BORCS8-MEF2B Readthrough	Protein Coding	GC19M019146	1.565032482
OCTN3	Organic Cation Transporter 3	Protein Coding	GC05U900771	1.565032482
ITGA6	Integrin Subunit Alpha 6	Protein Coding	GC02P172427	1.56243825
CEP76	Centrosomal Protein 76	Protein Coding	GC18M019718	1.561206818
TLR10	Toll Like Receptor 10	Protein Coding	GC04M038773	1.560414195
CYTH1	Cytohesin 1	Protein Coding	GC17M078674	1.557875633
TRG	T Cell Receptor Gamma Locus	Protein Coding	GC07M038240	1.557143092
DDIT4	DNA Damage Inducible Transcript 4	Protein Coding	GC10P072273	1.556235552
LRG1	Leucine Rich Alpha-2-Glycoprotein 1	Protein Coding	GC19M004546	1.556235552
DEFB104A	Defensin Beta 104A	Protein Coding	GC08P007836	1.556235552
SEC31B	SEC31 Homolog B, COPII Coat Complex Component	Protein Coding	GC10M100486	1.555950403
REEP1	Receptor Accessory Protein 1	Protein Coding	GC02M086213	1.554166317
MIR185	MicroRNA 185	RNA Gene	GC22P020297	1.553256631
VIPR1	Vasoactive Intestinal Peptide Receptor	Protein Coding	GC03P042490	1.553066015
SOCS2	Suppressor Of Cytokine Signaling 2	Protein Coding	GC12P093569	1.552201748
NRON	Non-Coding Repressor Of NFAT	RNA Gene	GC09M126407	1.551879406
LEPQTL1	Leptin, Serum Levels Of	Genetic Locus	GC02U903086	1.55148077
ZFP36L1	ZFP36 Ring Finger Protein Like 1	Protein Coding	GC14M068787	1.551291347
GBGT1	Globoside Alpha-1,3-N-Acetylgalactosaminyltransferase 1 (FORS Blood Group)	Protein Coding	GC09M133152	1.551291347
RING1	Ring Finger Protein 1	Protein Coding	GC06P033208	1.55115962
GP2	Glycoprotein 2	Protein Coding	GC16M020309	1.545448542
RXRB	Retinoid X Receptor Beta	Protein Coding	GC06M033193	1.544183731
CCRL2	C-C Motif Chemokine Receptor Like 2	Protein Coding	GC03P046407	1.544183731
CD19	CD19 Molecule	Protein Coding	GC16P033267	1.537396789
ABCC1	ATP Binding Cassette Subfamily C Member 1	Protein Coding	GC16P015949	1.537153006
FOXE1	Forkhead Box E1	Protein Coding	GC09P097853	1.535826683
EVPL	Envoplakin	Protein Coding	GC17M076004	1.53316021
CAMK4	Calcium/Calmodulin Dependent Protein Kinase IV	Protein Coding	GC05P111223	1.531560898
RBBP5	RB Binding Protein 5, Histone Lysine Methyltransferase Complex Subunit	Protein Coding	GC01M205055	1.530276418
MYCN	MYCN Proto-Oncogene, BHLH Transcription Factor	Protein Coding	GC02P015949	1.527597785
XRCC3	X-Ray Repair Cross Complementing 3	Protein Coding	GC14M103697	1.527597785
MIR183	MicroRNA 183	RNA Gene	GC07M129801	1.527597785
MIR454	MicroRNA 454	RNA Gene	GC17M059137	1.527597785
ACAT2	Acetyl-CoA Acetyltransferase 2	Protein Coding	GC06P159778	1.526411414
TUBB6	Tubulin Beta 6 Class V	Protein Coding	GC18P012307	1.526411414
DNAJC15	DnaJ Heat Shock Protein Family (Hsp40) Member C15	Protein Coding	GC13P043023	1.526411414
KCNQ1	Potassium Voltage-Gated Channel Subfamily Q Member 1	Protein Coding	GC11P002444	1.52616477
CBS	Cystathionine Beta-Synthase	Protein Coding	GC21M043053	1.52616477
ATF2	Activating Transcription Factor 2	Protein Coding	GC02M175072	1.52616477

PER3	Period Circadian Regulator 3	Protein Coding	GC01P007785	1.523105979
CCL19	C-C Motif Chemokine Ligand 19	Protein Coding	GC09M034692	1.523105979
BDH2	3-Hydroxybutyrate Dehydrogenase 2	Protein Coding	GC04M103077	1.523105979
LRRC3C	Leucine Rich Repeat Containing 3C	Protein Coding	GC17P039948	1.523105979
IRF3	Interferon Regulatory Factor 3	Protein Coding	GC19M049659	1.518408298
PPOX	Protoporphyrinogen Oxidase	Protein Coding	GC01P161205	1.516350746
EFNB2	Ephrin B2	Protein Coding	GC13M106489	1.516350746
PPP2R3C	Protein Phosphatase 2 Regulatory Subunit B"Gamma	Protein Coding	GC14M035085	1.516350746
WDR6	WD Repeat Domain 6	Protein Coding	GC03P049007	1.516350746
CXCL6	C-X-C Motif Chemokine Ligand 6	Protein Coding	GC04P073837	1.515669346
FOXP1	Forkhead Box P1	Protein Coding	GC03M070926	1.513262391
PDCD4	Programmed Cell Death 4	Protein Coding	GC10P110871	1.512670279
ACTC1	Actin Alpha Cardiac Muscle 1	Protein Coding	GC15M034790	1.512100697
FOXO1	Forkhead Box O1	Protein Coding	GC13M040555	1.511733294
SELPLG	Selectin P Ligand	Protein Coding	GC12M108621	1.511198044
CMA1	Chymase 1	Protein Coding	GC14M024506	1.510478497
BTRC	Beta-Transducin Repeat Containing E3 Ubiquitin Protein Ligase	Protein Coding	GC10P101354	1.50857234
ABCA1	ATP Binding Cassette Subfamily A Member 1	Protein Coding	GC09M104781	1.50607121
RAD21	RAD21 Cohesin Complex Component	Protein Coding	GC08M116846	1.502050877
C1GALT1	Core 1 Synthase, Glycoprotein-N-Acetylgalactosamine 3-Beta-Galactosyltransferase 1	Protein Coding	GC07P007156	1.500112534
PDGFA	Platelet Derived Growth Factor Subunit	Protein Coding	GC07M000497	1.496742964
MIR135B	MicroRNA 135b	RNA Gene	GC01M205448	1.496371984
ANPEP	Alanyl Aminopeptidase, Membrane	Protein Coding	GC15M089784	1.495853066
MRE11	MRE11 Homolog, Double Strand Break Repair Nuclease	Protein Coding	GC11M095000	1.489170313
SDHA	Succinate Dehydrogenase Complex Flavoprotein Subunit A	Protein Coding	GC05P000208	1.487543702
SCNN1B	Sodium Channel Epithelial 1 Subunit	Protein Coding	GC16P023278	1.487543702
AKR1C3	Aldo-Keto Reductase Family 1 Member C3	Protein Coding	GC10P005035	1.487543702
HMGA1	High Mobility Group AT-Hook 1	Protein Coding	GC06P058420	1.487543702
SCNN1G	Sodium Channel Epithelial 1 Subunit Gamma	Protein Coding	GC16P023182	1.487543702
CYC1	Cytochrome C1	Protein Coding	GC08P144095	1.487543702
KCNE3	Potassium Voltage-Gated Channel Subfamily E Regulatory Subunit 3	Protein Coding	GC11M074454	1.487543702
AQP9	Aquaporin 9	Protein Coding	GC15P058138	1.487543702
EYA4	EYA Transcriptional Coactivator And Phosphatase 4	Protein Coding	GC06P133240	1.487543702
SLC18A3	Solute Carrier Family 18 Member A3	Protein Coding	GC10P049610	1.487543702
TFPI	Tissue Factor Pathway Inhibitor	Protein Coding	GC02M187464	1.487543702
CPA6	Carboxypeptidase A6	Protein Coding	GC08M067422	1.487543702
CITED2	Cbp/P300 Interacting Transactivator With Glu/Asp Rich Carboxy-Terminal Domain 2	Protein Coding	GC06M139371	1.487543702
TTBK2	Tau Tubulin Kinase 2	Protein Coding	GC15M042738	1.487543702
TUSC3	Tumor Suppressor Candidate 3	Protein Coding	GC08P015417	1.487543702
UTS2R	Urotensin 2 Receptor	Protein Coding	GC17P082374	1.487543702
IFITM1	Interferon Induced Transmembrane Protein 1	Protein Coding	GC11P000313	1.487543702
GPR55	G Protein-Coupled Receptor 55	Protein Coding	GC02M230907	1.487543702
NMB	Neuromedin B	Protein Coding	GC15M084655	1.487543702
MXI1	MAX Interactor 1, Dimerization	Protein Coding	GC10P110208	1.487543702
PDCD1LG2	Programmed Cell Death 1 Ligand 2	Protein Coding	GC09P005510	1.487543702
UQCRQ	Ubiquinol-Cytochrome C Reductase Complex III Subunit VII	Protein Coding	GC05P132866	1.487543702
MARVELD2	MARVEL Domain Containing 2	Protein Coding	GC05P069415	1.487543702
NEK6	NIMA Related Kinase 6	Protein Coding	GC09P124259	1.487543702

ACSM3	Acyl-CoA Synthetase Medium Chain Family Member 3	Protein Coding	GC16P020610	1.487543702
APBA1	Amyloid Beta Precursor Protein Binding Family A Member 1	Protein Coding	GC09M069427	1.487543702
SERPINA12	Serpin Family A Member 12	Protein Coding	GC14M096476	1.487543702
SMOX	Spermine Oxidase	Protein Coding	GC20P004120	1.487543702
CCL16	C-C Motif Chemokine Ligand 16	Protein Coding	GC17M035976	1.487543702
NUCKS1	Nuclear Casein Kinase And Cyclin Dependent Kinase Substrate 1	Protein Coding	GC01M205712	1.487543702
TIMD4	T Cell Immunoglobulin And Mucin Domain Containing 4	Protein Coding	GC05M156919	1.487543702
LRCH1	Leucine Rich Repeats And Calponin Homology Domain Containing 1	Protein Coding	GC13P046553	1.487543702
RC3H1	Ring Finger And CCCH-Type Domains	Protein Coding	GC01M173931	1.487543702
BAHD1	Bromo Adjacent Homology Domain Containing 1	Protein Coding	GC15P040439	1.487543702
ANP32E	Acidic Nuclear Phosphoprotein 32 Family Member E	Protein Coding	GC01M150218	1.487543702
TAC4	Tachykinin Precursor 4	Protein Coding	GC17M049838	1.487543702
RAVER2	Ribonucleoprotein, PTB Binding 2	Protein Coding	GC01P064745	1.487543702
CAVIN3	Caveolae Associated Protein 3	Protein Coding	GC11M006319	1.487543702
IGHG3	Immunoglobulin Heavy Constant Gamma 3 (G3m Marker)	Protein Coding	GC14M110582	1.487543702
EXOC3-AS1	EXOC3 Antisense RNA 1	RNA Gene	GC05M000443	1.487543702
MIR4728	MicroRNA 4728	RNA Gene	GC17P039726	1.487543702
LOC110594336	MS1 Minisatellite Repeat Instability Region	Biological Region	GC01P031428	1.487543702
BDKRB1	Bradykinin Receptor B1	Protein Coding	GC14P096275	1.487211347
CORO1A	Coronin 1A	Protein Coding	GC16P033417	1.485896587
PDE4D	Phosphodiesterase 4D	Protein Coding	GC05M058969	1.483188152
TRIM21	Tripartite Motif Containing 21	Protein Coding	GC11M004384	1.48232007
BCL3	BCL3 Transcription Coactivator	Protein Coding	GC19P044747	1.479991913
RUNX1T1	RUNX1 Partner Transcriptional Co-Repressor 1	Protein Coding	GC08M091954	1.478321075
MIR574	MicroRNA 574	RNA Gene	GC04P038874	1.476874948
MIR151A	MicroRNA 151a	RNA Gene	GC08M140733	1.476874948
YES1	YES Proto-Oncogene 1, Src Family Tyrosine Kinase	Protein Coding	GC18M000721	1.473802567
CALCOCO2	Calcium Binding And Coiled-Coil Domain 2	Protein Coding	GC17P048830	1.473802567
MIRLET7F1	MicroRNA Let-7f-1	RNA Gene	GC09P094193	1.473802567
LMNB2	Lamin B2	Protein Coding	GC19M002556	1.471717119
AZU1	Azurocidin 1	Protein Coding	GC19P000825	1.470178723
SPINK5	Serine Peptidase Inhibitor Kazal Type 5	Protein Coding	GC05P148025	1.467393517
TYR	Tyrosinase	Protein Coding	GC11P089177	1.464984894
CBR3-AS1	CBR3 Antisense RNA 1	RNA Gene	GC21M036131	1.461177826
CNTNAP2	Contactin Associated Protein 2	Protein Coding	GC07P146116	1.458083868
MIR10A	MicroRNA 10a	RNA Gene	GC17M048579	1.457414389
RXRA	Retinoid X Receptor Alpha	Protein Coding	GC09P134317	1.449391365
CD38	CD38 Molecule	Protein Coding	GC04P015779	1.449234009
MGAM	Maltase-Glucoamylase	Protein Coding	GC07P146237	1.448504925
BANF1	BAF Nuclear Assembly Factor 1	Protein Coding	GC11P066002	1.448221922
SLC26A6	Solute Carrier Family 26 Member 6	Protein Coding	GC03M048625	1.447627902
PROC	Protein C, Inactivator Of Coagulation Factors Va And VIIIa	Protein Coding	GC02P127418	1.44647038
COMT	Catechol-O-Methyltransferase	Protein Coding	GC22P019941	1.442210913
LAMB3	Laminin Subunit Beta 3	Protein Coding	GC01M209614	1.439692616
KIF15	Kinesin Family Member 15	Protein Coding	GC03P045693	1.43609035
HMGCR	3-Hydroxy-3-Methylglutaryl-CoA Reductase	Protein Coding	GC05P075336	1.435017347
XRCC4	X-Ray Repair Cross Complementing 4	Protein Coding	GC05P083077	1.434891462
BAP1	BRCA1 Associated Protein 1	Protein Coding	GC03M052401	1.431420565
MIR590	MicroRNA 590	RNA Gene	GC07P074191	1.430395722

APOA1	Apolipoprotein A1	Protein Coding	GC11M116835	1.427671194
OSMR	Oncostatin M Receptor	Protein Coding	GC05P038845	1.426987767
CALB2	Calbindin 2	Protein Coding	GC16P071392	1.424746513
RAG1	Recombination Activating 1	Protein Coding	GC11P036522	1.423183203
OSM	Oncostatin M	Protein Coding	GC22M030262	1.422289252
EHMT2	Euchromatic Histone Lysine Methyltransferase 2	Protein Coding	GC06M031879	1.421438098
CASP7	Caspase 7	Protein Coding	GC10P113679	1.419958353
AGFG1	ArfGAP With FG Repeats 1	Protein Coding	GC02P227473	1.417472124
BLOC1S2	Biogenesis Of Lysosomal Organelles Complex 1 Subunit 2	Protein Coding	GC10M100273	1.417472124
TRIM31	Tripartite Motif Containing 31	Protein Coding	GC06M049019	1.416582942
ADAMTS13	ADAM Metallopeptidase With Thrombospondin Type 1 Motif 13	Protein Coding	GC09P133414	1.416257381
AHSG	Alpha 2-HS Glycoprotein	Protein Coding	GC03P186612	1.413164496
FLNC	Filamin C	Protein Coding	GC07P128830	1.410164356
SUOX	Sulfite Oxidase	Protein Coding	GC12P055997	1.410164356
NRM	Nurim	Protein Coding	GC06M049037	1.410164356
POLR1H	RNA Polymerase I Subunit H	Protein Coding	GC06P060771	1.410164356
MSTO2P	Misato Family Member 2, Pseudogene	Pseudogene	GC01P155745	1.410164356
CX3CL1	C-X3-C Motif Chemokine Ligand 1	Protein Coding	GC16P057372	1.40872097
AP4B1-AS1	AP4B1 Antisense RNA 1	RNA Gene	GC01P113813	1.407090902
CCDC88B	Coiled-Coil Domain Containing 88B	Protein Coding	GC11P064340	1.405967951
DSC3	Desmocollin 3	Protein Coding	GC18M030990	1.402263641
MAGT1	Magnesium Transporter 1	Protein Coding	GC0XM077840	1.399844766
FGFR4	Fibroblast Growth Factor Receptor 4	Protein Coding	GC05P177086	1.395335317
POLB	DNA Polymerase Beta	Protein Coding	GC08P042338	1.395335317
MTHFD1L	Methylenetetrahydrofolate Dehydrogenase (NADP+ Dependent) 1	Protein Coding	GC06P150865	1.395335317
KCNN4	Potassium Calcium-Activated Channel Subfamily N Member 4	Protein Coding	GC19M048587	1.393708706
RIPK3	Receptor Interacting Serine/Threonine Kinase 3	Protein Coding	GC14M024336	1.388664961
PRKD1	Protein Kinase D1	Protein Coding	GC14M029576	1.386357546
THY1	Thy-1 Cell Surface Antigen	Protein Coding	GC11M119417	1.381837845
AIM2	Absent In Melanoma 2	Protein Coding	GC01M159062	1.380523682
ARFGAP1	ADP Ribosylation Factor GTPase Activating Protein 1	Protein Coding	GC20P063272	1.374065399
C4BPB	Complement Component 4 Binding Protein Beta	Protein Coding	GC01P207088	1.374065399
TATDN1	TatD DNase Domain Containing 1	Protein Coding	GC08M124488	1.374065399
MIR429	MicroRNA 429	RNA Gene	GC01P002224	1.372618198
HMGA2	High Mobility Group AT-Hook 2	Protein Coding	GC12P065824	1.366424561
CD160	CD160 Molecule	Protein Coding	GC01P145719	1.363041997
DEFA3	Defensin Alpha 3	Protein Coding	GC08M007015	1.362460375
GSK3B	Glycogen Synthase Kinase 3 Beta	Protein Coding	GC03M119821	1.361569524
PIK3R3	Phosphoinositide-3-Kinase Regulatory Subunit 3	Protein Coding	GC01M046041	1.360974073
PAGR1	PAXIP1 Associated Glutamate Rich Protein 1	Protein Coding	GC16P033389	1.355511785
TPO	Thyroid Peroxidase	Protein Coding	GC02P001374	1.354565263
CIT	Citron Rho-Interacting Serine/Threonine Kinase	Protein Coding	GC12M119650	1.354035974
CAST	Calpastatin	Protein Coding	GC05P096525	1.354035974
POLR1D	RNA Polymerase I And III Subunit D	Protein Coding	GC13P027620	1.354035974
CAPN10	Calpain 10	Protein Coding	GC02P240586	1.354035974
EMG1	EMG1 N1-Specific Pseudouridine Methyltransferase	Protein Coding	GC12P006970	1.354035974
HSD17B8	Hydroxysteroid 17-Beta	Protein Coding	GC06P058365	1.354035974
KLRD1	Killer Cell Lectin Like Receptor D1	Protein Coding	GC12P010226	1.351928592
CASP4	Caspase 4	Protein Coding	GC11M104942	1.350113153
FANCC	FA Complementation Group C	Protein Coding	GC09M095099	1.348971367

MAP2K4	Mitogen-Activated Protein Kinase Kinase 4	Protein Coding	GC17P012020	1.348971367
LYVE1	Lymphatic Vessel Endothelial Hyaluronan Receptor 1	Protein Coding	GC11M010732	1.348971367
VEGFD	Vascular Endothelial Growth Factor D	Protein Coding	GC0XM015345	1.348971367
SLC12A2	Solute Carrier Family 12 Member 2	Protein Coding	GC05P128083	1.348324776
MIR29C	MicroRNA 29c	RNA Gene	GC01M207802	1.345158339
FOLR1	Folate Receptor Alpha	Protein Coding	GC11P072190	1.342255354
SEMA3E	Semaphorin 3E	Protein Coding	GC07M083363	1.339740038
FBXW11	F-Box And WD Repeat Domain Containing 11	Protein Coding	GC05M171861	1.339457393
LXN	Latexin	Protein Coding	GC03M158645	1.327020168
UBE2D1	Ubiquitin Conjugating Enzyme E2 D1	Protein Coding	GC10P058334	1.325134754
GLMN	Glomulin, FKBP Associated Protein	Protein Coding	GC01M092246	1.325134754
TECPR1	Tectonin Beta-Propeller Repeat Containing 1	Protein Coding	GC07M098214	1.325134754
CFL1	Cofilin 1	Protein Coding	GC11M065823	1.325125217
BCL2L11	BCL2 Like 11	Protein Coding	GC02P111119	1.324761868
IKZF4	IKAROS Family Zinc Finger 4	Protein Coding	GC12P056007	1.322747946
CSNK2B	Casein Kinase 2 Beta	Protein Coding	GC06P058312	1.322227478
BRD2	Bromodomain Containing 2	Protein Coding	GC06P058346	1.322227478
B3GALT4	Beta-1,3-Galactosyltransferase 4	Protein Coding	GC06P033277	1.322227478
ATF6B	Activating Transcription Factor 6 Beta	Protein Coding	GC06M032115	1.322227478
VPS52	VPS52 Subunit Of GARP Complex	Protein Coding	GC06M049211	1.322227478
CYP21A1P	Cytochrome P450 Family 21 Subfamily A Member 1, Pseudogene	Pseudogene	GC06P032005	1.322227478
HLA-DRB6	Major Histocompatibility Complex, Class II, DR Beta 6 (Pseudogene)	Pseudogene	GC06M049165	1.322227478
HLA-DQB1-AS1	HLA-DQB1 Antisense RNA 1	RNA Gene	GC06P032659	1.322227478
MIR92A1	MicroRNA 92a-1	RNA Gene	GC13P091474	1.321613312
INSL5	Insulin Like 5	Protein Coding	GC01M066797	1.320270061
HLA-DQB2	Major Histocompatibility Complex, Class II, DQ Beta 2	Protein Coding	GC06M032756	1.319154024
TNFRSF13B	TNF Receptor Superfamily Member	Protein Coding	GC17M016929	1.316838384
STX8	Syntaxin 8	Protein Coding	GC17M009250	1.314285874
DDB1	Damage Specific DNA Binding Protein	Protein Coding	GC11M071444	1.313344717
DNA2	DNA Replication Helicase/Nuclease 2	Protein Coding	GC10M068414	1.313344717
CXCR6	C-X-C Motif Chemokine Receptor 6	Protein Coding	GC03P045982	1.312773705
PRKAA1	Protein Kinase AMP-Activated Catalytic Subunit Alpha 1	Protein Coding	GC05M040759	1.308302045
PPP2R1A	Protein Phosphatase 2 Scaffold Subunit Aalpha	Protein Coding	GC19P052189	1.30660224
CRNDE	Colorectal Neoplasia Differentially Expressed	RNA Gene	GC16M054845	1.30660224
SSRP1	Structure Specific Recognition Protein	Protein Coding	GC11M071318	1.303766251
SCD	Stearoyl-CoA Desaturase	Protein Coding	GC10P100347	1.30046916
HADH	Hydroxyacyl-CoA Dehydrogenase	Protein Coding	GC04P107989	1.30046916
KCNA3	Potassium Voltage-Gated Channel Subfamily A Member 3	Protein Coding	GC01M110654	1.30046916
ATG7	Autophagy Related 7	Protein Coding	GC03P011273	1.30046916
ACAP1	ArfGAP With Coiled-Coil, Ankyrin Repeat And PH Domains 1	Protein Coding	GC17P009319	1.30046916
OVGP1	Oviductal Glycoprotein 1	Protein Coding	GC01M111414	1.30046916
KCNN1	Potassium Calcium-Activated Channel Subfamily N Member 1	Protein Coding	GC19P027784	1.296438098
ZFP36	ZFP36 Ring Finger Protein	Protein Coding	GC19P039406	1.296438098
CHRNA5	Cholinergic Receptor Nicotinic Alpha 5 Subunit	Protein Coding	GC15P078565	1.294730186
CUX1	Cut Like Homeobox 1	Protein Coding	GC07P101815	1.294730186
SUN2	Sad1 And UNC84 Domain Containing	Protein Coding	GC22M049698	1.292290449
MX1	MX Dynamin Like GTPase 1	Protein Coding	GC21P041420	1.291525841
TYRP1	Tyrosinase Related Protein 1	Protein Coding	GC09P012683	1.289606452

MIR23A	MicroRNA 23a	RNA Gene	GC19M014150	1.289211988
RETNLB	Resistin Like Beta	Protein Coding	GC03M108743	1.288336873
MIR181A2	MicroRNA 181a-2	RNA Gene	GC09P124692	1.286728144
PRSS8	Serine Protease 8	Protein Coding	GC16M031822	1.285121441
SPI1	Spi-1 Proto-Oncogene	Protein Coding	GC11M071235	1.285121441
IL9R	Interleukin 9 Receptor	Protein Coding	GC0XP155997	1.285121441
NTN1	Netrin 1	Protein Coding	GC17P009021	1.281812429
DSC1	Desmocollin 1	Protein Coding	GC18M031129	1.281529069
RAB5A	RAB5A, Member RAS Oncogene	Protein Coding	GC03P019963	1.280842662
HLA-DRB4	Major Histocompatibility Complex, Class II, DR Beta 4	Protein Coding	GC06Mo03851	1.280461788
TOP2A	DNA Topoisomerase II Alpha	Protein Coding	GC17M040388	1.272926331
UCHL1	Ubiquitin C-Terminal Hydrolase L1	Protein Coding	GC04P041256	1.272589684
LYN	LYN Proto-Oncogene, Src Family Tyrosine Kinase	Protein Coding	GC08P055879	1.272589684
WNT10B	Wnt Family Member 10B	Protein Coding	GC12M048965	1.272589684
OGG1	8-Oxoguanine DNA Glycosylase	Protein Coding	GC03P009816	1.272589684
HIC1	HIC ZBTB Transcriptional Repressor 1	Protein Coding	GC17P002054	1.272578716
NINJ1	Ninjurin 1	Protein Coding	GC09M093121	1.271099091
RAB5B	RAB5B, Member RAS Oncogene	Protein Coding	GC12P055973	1.271009326
RBM5	RNA Binding Motif Protein 5	Protein Coding	GC03P050148	1.271009326
KLHDC8B	Kelch Domain Containing 8B	Protein Coding	GC03P049171	1.271009326
RBM6	RNA Binding Motif Protein 6	Protein Coding	GC03P049940	1.271009326
NICN1	Nicolin 1	Protein Coding	GC03M049422	1.271009326
IHO1	Interactor Of HORMAD1 1	Protein Coding	GC03P049333	1.271009326
INKA1	Inka Box Actin Regulator 1	Protein Coding	GC03P049803	1.271009326
HCG27	HLA Complex Group 27	RNA Gene	GC06P031197	1.271009326
MIR4456	MicroRNA 4456	RNA Gene	GC05M000536	1.271009326
ENSG00000271581	HLA Complex Group 4 (HCG4) Pseudogene	Pseudogene	GC06P058297	1.271009326
MK280269-056		RNA Gene	GC06M050091	1.271009326
MN298114-196		RNA Gene	GC06M050097	1.271009326
RBP4	Retinol Binding Protein 4	Protein Coding	GC10M093591	1.266486764
PFKFB3	6-Phosphofructo-2-Kinase/Fructose-2,6-Biphosphatase 3	Protein Coding	GC10P006144	1.265302896
SLC9A8	Solute Carrier Family 9 Member A8	Protein Coding	GC20P049813	1.265302896
ZFP91	ZFP91 Zinc Finger Protein, Atypical E3 Ubiquitin Ligase	Protein Coding	GC11P058580	1.265302896
SGK1	Serum/Glucocorticoid Regulated Kinase 1	Protein Coding	GC06M134169	1.264857411
CARD14	Caspase Recruitment Domain Family Member 14	Protein Coding	GC17P080170	1.261325717
KLRC1	Killer Cell Lectin Like Receptor C1	Protein Coding	GC12M016317	1.261325717
CD2	CD2 Molecule	Protein Coding	GC01P116754	1.261047125
TEC	Tec Protein Tyrosine Kinase	Protein Coding	GC04M048137	1.259700537
CYTOR	Cytoskeleton Regulator RNA	RNA Gene	GC02P087644	1.256068468
KRT17	Keratin 17	Protein Coding	GC17M041619	1.252887487
MIR130A	MicroRNA 130a	RNA Gene	GC11P057641	1.247033596
BLZF1	Basic Leucine Zipper Nuclear Factor 1	Protein Coding	GC01P169367	1.246665239
ARRB2	Arrestin Beta 2	Protein Coding	GC17P004711	1.246500492
FOXP2	Forkhead Box P2	Protein Coding	GC07P114086	1.246446371
FSCN1	Fascin Actin-Bundling Protein 1	Protein Coding	GC07P005592	1.246194124
CSE1L	Chromosome Segregation 1 Like	Protein Coding	GC20P049046	1.245823622
SAA1	Serum Amyloid A1	Protein Coding	GC11P018267	1.244864821
PGLYRP1	Peptidoglycan Recognition Protein 1	Protein Coding	GC19M048760	1.243505597
RARB	Retinoic Acid Receptor Beta	Protein Coding	GC03P024830	1.239711523
C5AR1	Complement C5a Receptor 1	Protein Coding	GC19P047290	1.239511728
MAP3K11	Mitogen-Activated Protein Kinase Kinase Kinase 11	Protein Coding	GC11M071696	1.238016844
FCN2	Ficolin 2	Protein Coding	GC09P134864	1.237435818
SKI	SKI Proto-Oncogene	Protein Coding	GC01P002228	1.237280846

EPRS1	Glutamyl-Prolyl-TRNA Synthetase 1	Protein Coding	GC01M219969	1.233324289
CACNA1S	Calcium Voltage-Gated Channel Subunit Alpha1 S	Protein Coding	GC01M201008	1.231290221
XPC	XPC Complex Subunit, DNA Damage Recognition And Repair Factor	Protein Coding	GC03M017380	1.231290221
SLC34A1	Solute Carrier Family 34 Member 1	Protein Coding	GC05P177380	1.231290221
EFEMP2	EGF Containing Fibulin Extracellular Matrix Protein 2	Protein Coding	GC11M071732	1.231290221
POLR1C	RNA Polymerase I And III Subunit C	Protein Coding	GC06P058522	1.231290221
DNMT3L	DNA Methyltransferase 3 Like	Protein Coding	GC21M044246	1.231290221
MYT1L	Myelin Transcription Factor 1 Like	Protein Coding	GC02M001789	1.231290221
SLC39A7	Solute Carrier Family 39 Member 7	Protein Coding	GC06P033200	1.231290221
RPL3	Ribosomal Protein L3	Protein Coding	GC22M049702	1.231290221
HMGN1	High Mobility Group Nucleosome Binding Domain 1	Protein Coding	GC21M039342	1.231290221
AAGAB	Alpha And Gamma Adaptin Binding Protein	Protein Coding	GC15M067200	1.231290221
FNBP1	Formin Binding Protein 1	Protein Coding	GC09M129887	1.231290221
THEM4	Thioesterase Superfamily Member 4	Protein Coding	GC01M151870	1.231290221
RCE1	Ras Converting CAAX Endopeptidase	Protein Coding	GC11P066842	1.231290221
NCKIPSD	NCK Interacting Protein With SH3 Domain	Protein Coding	GC03M048667	1.231290221
INO80	INO80 Complex ATPase Subunit	Protein Coding	GC15M040979	1.231290221
NOP2	NOP2 Nucleolar Protein	Protein Coding	GC12M006556	1.231290221
ZNF341	Zinc Finger Protein 341	Protein Coding	GC20P033731	1.231290221
ENGASE	Endo-Beta-N-Acetylglucosaminidase	Protein Coding	GC17P079071	1.231290221
MPIG6B	Megakaryocyte And Platelet Inhibitory Receptor G6b	Protein Coding	GC06P059621	1.231290221
MTERF4	Mitochondrial Transcription Termination Factor 4	Protein Coding	GC02M241072	1.231290221
ZNRD2	Zinc Ribbon Domain Containing 2	Protein Coding	GC11P066823	1.231290221
PHETA1	PH Domain Containing Endocytic Trafficking Adaptor 1	Protein Coding	GC12M111361	1.231290221
CCDC26	CCDC26 Long Non-Coding RNA	RNA Gene	GC08M129261	1.231290221
DBH	Dopamine Beta-Hydroxylase	Protein Coding	GC09P133636	1.231181383
C19orf33	Chromosome 19 Open Reading Frame	Protein Coding	GC19P038304	1.228988886
MIR324	MicroRNA 324	RNA Gene	GC17M007223	1.228373766
MIR490	MicroRNA 490	RNA Gene	GC07P136903	1.228373766
MLANA	Melan-A	Protein Coding	GC09P005928	1.227476835
CD63	CD63 Molecule	Protein Coding	GC12M055725	1.226338506
PRDX6	Peroxiredoxin 6	Protein Coding	GC01P173477	1.225689173
ADAM10	ADAM Metallopeptidase Domain 10	Protein Coding	GC15M058588	1.225564718
IRF7	Interferon Regulatory Factor 7	Protein Coding	GC11M000612	1.223291874
WAKMAR2	Wound And Keratinocyte Migration Associated LncRNA 2	RNA Gene	GC06M137820	1.222651243
PLA2G3	Phospholipase A2 Group III	Protein Coding	GC22M031364	1.221961379
RHO	Rhodopsin	Protein Coding	GC03P131767	1.220944524
PRKAB1	Protein Kinase AMP-Activated Non-Catalytic Subunit Beta 1	Protein Coding	GC12P119632	1.220016479
SH2B3	SH2B Adaptor Protein 3	Protein Coding	GC12P111405	1.220016479
AMT	Aminomethyltransferase	Protein Coding	GC03M049541	1.220016479
GRB7	Growth Factor Receptor Bound Protein	Protein Coding	GC17P039751	1.220016479
CDK12	Cyclin Dependent Kinase 12	Protein Coding	GC17P039461	1.220016479
CTDP1	CTD Phosphatase Subunit 1	Protein Coding	GC18P079679	1.220016479
CCNT1	Cyclin T1	Protein Coding	GC12M048688	1.220016479
PHB2	Prohibitin 2	Protein Coding	GC12M006965	1.220016479
IL22RA1	Interleukin 22 Receptor Subunit Alpha	Protein Coding	GC01M024119	1.220016479
SLC25A28	Solute Carrier Family 25 Member 28	Protein Coding	GC10M099610	1.220016479
MIR611	MicroRNA 611	RNA Gene	GC11M061792	1.220016479
MIR3936HG	MIR3936 Host Gene	RNA Gene	GC05M132312	1.220016479
CCR5AS	CCR5 Antisense RNA	RNA Gene	GC03M046365	1.220016479
UBAP2L	Ubiquitin Associated Protein 2 Like	Protein Coding	GC01P154219	1.218938708
STN1	STN1 Subunit Of CST Complex	Protein Coding	GC10M103895	1.217959762

IGF2R	Insulin Like Growth Factor 2 Receptor	Protein Coding	GC06P159969	1.215999365
MIR29B2	MicroRNA 29b-2	RNA Gene	GC01M207806	1.215999365
DUSP1	Dual Specificity Phosphatase 1	Protein Coding	GC05M172768	1.215865374
RO60	Ro60, Y RNA Binding Protein	Protein Coding	GC01P193059	1.214896441
BMP2	Bone Morphogenetic Protein 2	Protein Coding	GC20P006696	1.213765621
LIG4	DNA Ligase 4	Protein Coding	GC13M108207	1.213662863
NHEJ1	Non-Homologous End Joining Factor 1	Protein Coding	GC02M219089	1.213662863
RBX1	Ring-Box 1	Protein Coding	GC22P040951	1.210733175
STUB1	STIP1 Homology And U-Box Containing Protein 1	Protein Coding	GC16P006127	1.209675074
TNK2	Tyrosine Kinase Non Receptor 2	Protein Coding	GC03M195863	1.204322577
PSMG1	Proteasome Assembly Chaperone 1	Protein Coding	GC21M039174	1.199519277
HHIP	Hedgehog Interacting Protein	Protein Coding	GC04P144645	1.194045901
LCT	Lactase	Protein Coding	GC02M135787	1.187662363
DDIT3	DNA Damage Inducible Transcript 3	Protein Coding	GC12M057516	1.182323456
ALCAM	Activated Leukocyte Cell Adhesion Molecule	Protein Coding	GC03P105366	1.182323456
TRAF3	TNF Receptor Associated Factor 3	Protein Coding	GC14P106523	1.182084799
CDC25C	Cell Division Cycle 25C	Protein Coding	GC05M138285	1.181986809
GNB3	G Protein Subunit Beta 3	Protein Coding	GC12P006839	1.181986809
IRS1	Insulin Receptor Substrate 1	Protein Coding	GC02M226731	1.181986809
LGR6	Leucine Rich Repeat Containing G Protein-Coupled Receptor 6	Protein Coding	GC01P202193	1.181986809
PTPRU	Protein Tyrosine Phosphatase Receptor Type U	Protein Coding	GC01P029236	1.181986809
UHRF1	Ubiquitin Like With PHD And Ring Finger Domains 1	Protein Coding	GC19P004910	1.181986809
ZBTB14	Zinc Finger And BTB Domain Containing 14	Protein Coding	GC18M005289	1.181986809
H3-2	H3.2 Histone (Putative)	Protein Coding	GC01M143894	1.181986809
SMIM35	Small Integral Membrane Protein 35	Protein Coding	GC11M118003	1.181986809
PHEX	Phosphate Regulating Endopeptidase Homolog X-Linked	Protein Coding	GC0XP022032	1.178843021
ACACA	Acetyl-CoA Carboxylase Alpha	Protein Coding	GC17M037084	1.177131653
BAG1	BAG Cochaperone 1	Protein Coding	GC09M033245	1.177131653
NELL1	Neural EGFL Like 1	Protein Coding	GC11P020647	1.176300049
GHSR	Growth Hormone Secretagogue	Protein Coding	GC03M172443	1.176289439
FZD7	Frizzled Class Receptor 7	Protein Coding	GC02P202034	1.173899651
EPHA2	EPH Receptor A2	Protein Coding	GC01M016124	1.173347831
EPHA1	EPH Receptor A1	Protein Coding	GC07M143390	1.173347831
SLC2A14	Solute Carrier Family 2 Member 14	Protein Coding	GC12M007812	1.172960162
PTPN6	Protein Tyrosine Phosphatase Non-Receptor Type 6	Protein Coding	GC12P013190	1.169544697
IRAK3	Interleukin 1 Receptor Associated Kinase 3	Protein Coding	GC12P066192	1.165616274
RPS3	Ribosomal Protein S3	Protein Coding	GC11P076427	1.164296985
PIK3CG	Phosphatidylinositol-4,5-Bisphosphate 3-Kinase Catalytic Subunit Gamma	Protein Coding	GC07P106865	1.16332984
JUP	Junction Plakoglobin	Protein Coding	GC17M041754	1.16332984
IFI27	Interferon Alpha Inducible Protein 27	Protein Coding	GC14P094104	1.16332984
MIR375	MicroRNA 375	RNA Gene	GC02M219001	1.16332984
MIRLET7B	MicroRNA Let-7b	RNA Gene	GC22P046119	1.161080122
MIR135A1	MicroRNA 135a-1	RNA Gene	GC03M052296	1.161080122
MIR103A1	MicroRNA 103a-1	RNA Gene	GC05M168560	1.161080122
MIR224	MicroRNA 224	RNA Gene	GC0XM151958	1.161080122
MIR542	MicroRNA 542	RNA Gene	GC0XM134638	1.161080122
COPS5	COP9 Signalosome Subunit 5	Protein Coding	GC08M067043	1.16039145
MTSS1	MTSS I-BAR Domain Containing 1	Protein Coding	GC08M124550	1.16039145
PHOX2B	Paired Like Homeobox 2B	Protein Coding	GC04M041746	1.152920246
TST	Thiosulfate Sulfurtransferase	Protein Coding	GC22M037010	1.152920246
EIF3C	Eukaryotic Translation Initiation Factor 3 Subunit C	Protein Coding	GC16P033222	1.152920246

NUPR1	Nuclear Protein 1, Transcriptional Regulator	Protein Coding	GC16M028532	1.152920246
PI4KA	Phosphatidylinositol 4-Kinase Alpha	Protein Coding	GC22M020707	1.151029825
FBXW4	F-Box And WD Repeat Domain Containing 4	Protein Coding	GC10M101610	1.151029825
ITGAV	Integrin Subunit Alpha V	Protein Coding	GC02P186589	1.150215864
WIF1	WNT Inhibitory Factor 1	Protein Coding	GC12M065050	1.150215864
MACF1	Microtubule Actin Crosslinking Factor	Protein Coding	GC01P039082	1.149355412
ABCF2	ATP Binding Cassette Subfamily F Member 2	Protein Coding	GC07M151211	1.149117708
CD47	CD47 Molecule	Protein Coding	GC03M108043	1.144270539
TNFSF11	TNF Superfamily Member 11	Protein Coding	GC13P042562	1.14324975
MAPK10	Mitogen-Activated Protein Kinase 10	Protein Coding	GC04M085990	1.142151952
LPIN2	Lipin 2	Protein Coding	GC18M002916	1.138580084
MIR219A1	MicroRNA 219a-1	RNA Gene	GC06P033207	1.132600188
DDAH2	Dimethylarginine Dimethylaminohydrolase 2	Protein Coding	GC06M031727	1.132079601
KIFC1	Kinesin Family Member C1	Protein Coding	GC06P033391	1.132079601
PPT2	Palmitoyl-Protein Thioesterase 2	Protein Coding	GC06P032153	1.132079601
FKBPL	FKBP Prolyl Isomerase Like	Protein Coding	GC06M049142	1.132079601
WDR46	WD Repeat Domain 46	Protein Coding	GC06M049214	1.132079601
EGFL8	EGF Like Domain Multiple 8	Protein Coding	GC06P058336	1.132079601
NELFE	Negative Elongation Factor Complex Member E	Protein Coding	GC06M031952	1.132079601
SAPCD1	Suppressor APC Domain Containing 1	Protein Coding	GC06P058326	1.132079601
HCG25	HLA Complex Group 25	RNA Gene	GC06P058370	1.132079601
HSALNG0049429		RNA Gene	GC06P058928	1.132079601
HSALNG0049430		RNA Gene	GC06M032641	1.132079601
NONHSAG045982.2		RNA Gene	GC06M032630	1.132079601
LOC107986589	Uncharacterized LOC107986589	RNA Gene	GC06M050071	1.132079601
MON1A	MON1 Homolog A, Secretory Trafficking Associated	Protein Coding	GC03M050187	1.131721377
C7	Complement C7	Protein Coding	GC05P040909	1.131263971
SLC9A1	Solute Carrier Family 9 Member A1	Protein Coding	GC01M027109	1.128103971
GPC5	Glypican 5	Protein Coding	GC13P091398	1.128103971
PTPN1	Protein Tyrosine Phosphatase Non-Receptor Type 1	Protein Coding	GC20P050510	1.125103831
ADRA1B	Adrenoceptor Alpha 1B	Protein Coding	GC05P159867	1.125103831
PIP5K1A	Phosphatidylinositol-4-Phosphate 5-Kinase Type 1 Alpha	Protein Coding	GC01P151198	1.125103831
TNXB	Tenascin XB	Protein Coding	GC06M049135	1.125103831
CRB1	Crumbs Cell Polarity Complex Component 1	Protein Coding	GC01P197170	1.125103831
CDSN	Corneodesmosin	Protein Coding	GC06M031115	1.125103831
FLAD1	Flavin Adenine Dinucleotide	Protein Coding	GC01P154983	1.125103831
THBS3	Thrombospondin 3	Protein Coding	GC01M155195	1.125103831
ATP6V1F	ATPase H+ Transporting V1 Subunit F	Protein Coding	GC07P128862	1.125103831
DPM3	Dolichyl-Phosphate Mannosyltransferase Subunit 3,	Protein Coding	GC01M155112	1.125103831
DHX16	DEAH-Box Helicase 16	Protein Coding	GC06M030653	1.125103831
LSM2	LSM2 Homolog, U6 Small Nuclear RNA And MRNA Degradation	Protein Coding	GC06M049132	1.125103831
ABCF1	ATP Binding Cassette Subfamily F Member 1	Protein Coding	GC06P030571	1.125103831
ATP8B2	ATPase Phospholipid Transporting 8B2	Protein Coding	GC01P154325	1.125103831
RNF114	Ring Finger Protein 114	Protein Coding	GC20P049936	1.125103831
ZFP57	ZFP57 Zinc Finger Protein	Protein Coding	GC06M029672	1.125103831
AIF1	Allograft Inflammatory Factor 1	Protein Coding	GC06P058306	1.125103831

PPP1R10	Protein Phosphatase 1 Regulatory Subunit 10	Protein Coding	GC06M030600	1.125103831
NSMCE2	NSE2 (MMS21) Homolog, SMC5-SMC6 Complex SUMO Ligase	Protein Coding	GC08P125091	1.125103831
UBE2Q1	Ubiquitin Conjugating Enzyme E2 Q1	Protein Coding	GC01M154521	1.125103831
CCDC8	Coiled-Coil Domain Containing 8	Protein Coding	GC19M046410	1.125103831
ZNF687	Zinc Finger Protein 687	Protein Coding	GC01P151281	1.125103831
TCF19	Transcription Factor 19	Protein Coding	GC06P058296	1.125103831
PYGO2	Pygopus Family PHD Finger 2	Protein Coding	GC01M154957	1.125103831
GNL1	G Protein Nucleolar 1 (Putative)	Protein Coding	GC06M030541	1.125103831
LRRC2	Leucine Rich Repeat Containing 2	Protein Coding	GC03M046515	1.125103831
DXO	Decapping Exoribonuclease	Protein Coding	GC06M031969	1.125103831
GON4L	Gon-4 Like	Protein Coding	GC01M155754	1.125103831
ZBTB12	Zinc Finger And BTB Domain Containing 12	Protein Coding	GC06M031899	1.125103831
ATAT1	Alpha Tubulin Acetyltransferase 1	Protein Coding	GC06P030626	1.125103831
KRTCAP2	Keratinocyte Associated Protein 2	Protein Coding	GC01M155141	1.125103831
RPP21	Ribonuclease P/MRP Subunit P21	Protein Coding	GC06P030345	1.125103831
PRR3	Proline Rich 3	Protein Coding	GC06P058282	1.125103831
OSGIN2	Oxidative Stress Induced Growth Inhibitor Family Member 2	Protein Coding	GC08P089901	1.125103831
PSORS1C2	Psoriasis Susceptibility 1 Candidate 2	Protein Coding	GC06M031137	1.125103831
C6orf47	Chromosome 6 Open Reading Frame	Protein Coding	GC06M049119	1.125103831
LY6G5B	Lymphocyte Antigen 6 Family Member G5B	Protein Coding	GC06P058316	1.125103831
FCMR	Fc Fragment Of IgM Receptor	Protein Coding	GC01M206904	1.125103831
HLA-H	Major Histocompatibility Complex, Class I, H (Pseudogene)	Pseudogene	GC06P058241	1.125103831
KHDC4	KH Domain Containing 4, Pre-MRNA Splicing Factor	Protein Coding	GC01M155913	1.125103831
RUSC1-AS1	RUSC1 Antisense RNA 1	RNA Gene	GC01M155317	1.125103831
SPATA48	Spermatogenesis Associated 48	Protein Coding	GC07P050095	1.125103831
HCG22	HLA Complex Group 22 (Gene/Pseudogene)	Protein Coding	GC06P031053	1.125103831
HCG18	HLA Complex Group 18	RNA Gene	GC06M049022	1.125103831
HLA-J	Major Histocompatibility Complex, Class I, J (Pseudogene)	Pseudogene	GC06P058252	1.125103831
HLA-F-AS1	HLA-F Antisense RNA 1	RNA Gene	GC06M048997	1.125103831
GBAP1	Glucosylceramidase Beta Pseudogene 1	Pseudogene	GC01M155213	1.125103831
HCG4B	HLA Complex Group 4B	RNA Gene	GC06M049013	1.125103831
ZNRD1ASP	Zinc Ribbon Domain Containing 1 Antisense, Pseudogene	Pseudogene	GC06M049017	1.125103831
TPI1P2	Triosephosphate Isomerase 1 Pseudogene 2	Pseudogene	GC07P129055	1.125103831
ASH1L-AS1	ASH1L Antisense RNA 1	RNA Gene	GC01P155561	1.125103831
HLA-L	Major Histocompatibility Complex, Class I, L (Pseudogene)	Pseudogene	GC06P058273	1.125103831
LINC01271	Long Intergenic Non-Protein Coding RNA 1271	RNA Gene	GC20M050310	1.125103831
LINC01273	Long Intergenic Non-Protein Coding RNA 1273	RNA Gene	GC20P050173	1.125103831
HLA-K	Major Histocompatibility Complex, Class I, K (Pseudogene)	Pseudogene	GC06P058247	1.125103831
TRIM31-AS1	TRIM31 Antisense RNA 1	RNA Gene	GC06P058256	1.125103831
FLNC-AS1	FLNC Antisense RNA 1	RNA Gene	GC07M128851	1.125103831
MICD	MHC Class I Polypeptide-Related Sequence D (Pseudogene)	Pseudogene	GC06M049016	1.125103831
LOC285626	Uncharacterized LOC285626	RNA Gene	GC05P159326	1.125103831
RIPK2-DT	RIPK2 Divergent Transcript	RNA Gene	GC08M089546	1.125103831
WASHC5-	WASHC5 Antisense RNA 1	RNA Gene	GC08P125141	1.125103831
HLA-W	Major Histocompatibility Complex, Class I, W (Pseudogene)	Pseudogene	GC06P058245	1.125103831

MICE	MHC Class I Polypeptide-Related Sequence E (Pseudogene)	Pseudogene	GC06M049000	1.125103831
LINC02009	Long Intergenic Non-Protein Coding RNA 2009	RNA Gene	GC03M046415	1.125103831
TSBP1-AS1	TSBP1 And BTNL2 Antisense RNA 1	RNA Gene	GC06P060951	1.125103831
RPL23AP1	Ribosomal Protein L23a Pseudogene 1	Pseudogene	GC06M048998	1.125103831
HLA-U	Major Histocompatibility Complex, Class I, U (Pseudogene)	Pseudogene	GC06P058249	1.125103831
PAIP1P1	Poly(A) Binding Protein Interacting Protein 1 Pseudogene 1	Pseudogene	GC06M030186	1.125103831
STK19B	Serine/Threonine Kinase 19B (Pseudogene)	Pseudogene	GC06P032013	1.125103831
RPL3P2	Ribosomal Protein L3 Pseudogene 2	Pseudogene	GC06P031280	1.125103831
ENSG00000270120	Novel Transcript	RNA Gene	GC16P050712	1.125103831
ENSG00000271380	Novel Transcript, Antisense To SHC1 And PYGO2	Uncategorized	GC01P154961	1.125103831
ENSG00000271553	Novel Transcript	RNA Gene	GC07P128667	1.125103831
UQCRC2P1	Ubiquinol-Cytochrome C Reductase Core Protein 2 Pseudogene 1	Pseudogene	GC03M046311	1.125103831
RN7SL636P	RNA, 7SL, Cytoplasmic 636,	Pseudogene	GC20M050454	1.125103831
ENSG00000285163	Novel Transcript	RNA Gene	GC16P085924	1.125103831
ENSG00000269667	Novel Transcript	RNA Gene	GC16P085990	1.125103831
ENSG00000272540	Novel Transcript, Antisense To TUBB	RNA Gene	GC06M049043	1.125103831
ENSG00000235620	Novel Transcript	RNA Gene	GC07M050274	1.125103831
TRL-AAG6-1	TRNA-Leu (Anticodon AAG) 6-1	RNA Gene	GC20M050337	1.125103831
LOC105447645	Uncharacterized LOC105447645	RNA Gene	GC19M049275	1.125103831
ENSG00000271267	ATP Synthase, H+ Transporting, Mitochondrial Fo Complex, Subunit F2 (ATP5J2) Pseudogene	Pseudogene	GC01M155568	1.125103831
ENSG00000285040	Novel Transcript	RNA Gene	GC16M085963	1.125103831
ENSG00000242162	Pseudogene Similar To Part Of Sec61 Alpha 1 Subunit (S. Cerevisiae) (SEC61A1)	Pseudogene	GC07M129066	1.125103831
lnc-IKZF1-6		RNA Gene	GC07P050275	1.125103831
HSALNG0061260		RNA Gene	GC07M128933	1.125103831
HSALNG0061261		RNA Gene	GC07P130328	1.125103831
lnc-IRF5-3		RNA Gene	GC07P130367	1.125103831
ENSG00000230521	HLA Complex Group 4 Pseudogene 7	Pseudogene	GC06M049007	1.125103831
ENSG00000237669	HLA Complex Group 4 Pseudogene 3	Pseudogene	GC06M049473	1.125103831
ENSG00000224163	Pseudogene Similar To Part Of Filamin C, Gamma (Actin Binding Protein 280) (FLNC)	Pseudogene	GC07M128913	1.125103831
RF00005-118		RNA Gene	GC20M050339	1.125103831
ENSG00000227766	HLA Complex Group 4 Pseudogene 5	Pseudogene	GC06M049011	1.125103831
HSALNG0066829-001		RNA Gene	GC08M089764	1.125103831
HSALNG0130758		RNA Gene	GC20M050344	1.125103831
piR-56133-186		RNA Gene	GC05M159366	1.125103831

NONHSAG019426.2		RNA Gene	GC16P050713	1.125103831
HSALNG0049239		RNA Gene	GC06P058888	1.125103831
DAG1	Dystroglycan 1	Protein Coding	GC03P049482	1.122030497
SYNGR1	Synaptogyrin 1	Protein Coding	GC22P039350	1.122030497
CCL8	C-C Motif Chemokine Ligand 8	Protein Coding	GC17P034319	1.122030497
CDH22	Cadherin 22	Protein Coding	GC20M046173	1.122030497
TENM3	Teneurin Transmembrane Protein 3	Protein Coding	GC04P181448	1.122030497
PRRT1	Proline Rich Transmembrane Protein 1	Protein Coding	GC06M049143	1.122030497
CTIF	Cap Binding Complex Dependent Translation Initiation Factor	Protein Coding	GC18P048539	1.122030497
PROX2	Prospero Homeobox 2	Protein Coding	GC14M074852	1.122030497
SNHG32	Small Nucleolar RNA Host Gene 32	RNA Gene	GC06P060927	1.122030497
CSK	C-Terminal Src Kinase	Protein Coding	GC15P074782	1.119930744
HOTAIR	HOX Transcript Antisense RNA	RNA Gene	GC12M053962	1.119930744
MYDGF	Myeloid Derived Growth Factor	Protein Coding	GC19M004641	1.119730473
TNFAIP6	TNF Alpha Induced Protein 6	Protein Coding	GC02P151357	1.118934512
UCP2	Uncoupling Protein 2	Protein Coding	GC11M073974	1.11683023
TNFRSF17	TNF Receptor Superfamily Member 17	Protein Coding	GC16P011965	1.11683023
PA2G4	Proliferation-Associated 2G4	Protein Coding	GC12P056466	1.115899324
KCNMA1	Potassium Calcium-Activated Channel Subfamily M Alpha 1	Protein Coding	GC10M076869	1.114046574
ACVR2A	Activin A Receptor Type 2A	Protein Coding	GC02P147844	1.111594677
PTPRT	Protein Tyrosine Phosphatase Receptor Type T	Protein Coding	GC20M042072	1.111348152
MIR346	MicroRNA 346	RNA Gene	GC10M086264	1.109506369
CD84	CD84 Molecule	Protein Coding	GC01M160541	1.108700037
NME1	NME/NM23 Nucleoside Diphosphate Kinase 1	Protein Coding	GC17P051154	1.108116031
UCA1	Urothelial Cancer Associated 1	RNA Gene	GC19P015828	1.108116031
PTENP1	Phosphatase And Tensin Homolog Pseudogene 1	Pseudogene	GC09M033673	1.108116031
CD300LF	CD300 Molecule Like Family Member	Protein Coding	GC17M074694	1.106109619
RBPJ	Recombination Signal Binding Protein For Immunoglobulin Kappa J Region	Protein Coding	GC04P026165	1.105516195
USP7	Ubiquitin Specific Peptidase 7	Protein Coding	GC16M008892	1.100485325
TMEM258	Transmembrane Protein 258	Protein Coding	GC11M061768	1.09748292
SESN2	Sestrin 2	Protein Coding	GC01P028270	1.097312689
REEP6	Receptor Accessory Protein 6	Protein Coding	GC19P001491	1.096333027
KLF1	Kruppel Like Factor 1	Protein Coding	GC19M012884	1.094646215
ACSL4	Acyl-CoA Synthetase Long Chain Family Member 4	Protein Coding	GC0XM109624	1.08908391
CARD16	Caspase Recruitment Domain Family Member 16	Protein Coding	GC11M105041	1.085940003
LY96	Lymphocyte Antigen 96	Protein Coding	GC08P073991	1.085861444
CCND2	Cyclin D2	Protein Coding	GC12P013105	1.084698439
PRKCZ	Protein Kinase C Zeta	Protein Coding	GC01P002050	1.083296776
KLF5	Kruppel Like Factor 5	Protein Coding	GC13P073054	1.083296776
NR2C2AP	Nuclear Receptor 2C2 Associated	Protein Coding	GC19M019201	1.08005178
H2AC21	H2A Clustered Histone 21	Protein Coding	GC01M150214	1.08005178
TBX20	T-Box Transcription Factor 20	Protein Coding	GC07M035237	1.076634049
CR1	Complement C3b/C4b Receptor 1 (Knops Blood Group)	Protein Coding	GC01P207496	1.076524019
CTSL	Cathepsin L	Protein Coding	GC09P087725	1.076460719
HDAC2	Histone Deacetylase 2	Protein Coding	GC06M113933	1.072726965
SLC29A1	Solute Carrier Family 29 Member 1 (Augustine Blood Group)	Protein Coding	GC06P044219	1.072726965
IGFBP7	Insulin Like Growth Factor Binding Protein 7	Protein Coding	GC04M057030	1.072726965
FOXM1	Forkhead Box M1	Protein Coding	GC12M002857	1.072726965
CDH13	Cadherin 13	Protein Coding	GC16P082626	1.072726965

ST6GAL1	ST6 Beta-Galactoside Alpha-2,6-Sialyltransferase 1	Protein Coding	GC03P186930	1.072726965
CLCA1	Chloride Channel Accessory 1	Protein Coding	GC01P086468	1.072726965
EREG	Epiregulin	Protein Coding	GC04P074366	1.072726965
ARHGEF6	Rac/Cdc42 Guanine Nucleotide Exchange Factor 6	Protein Coding	GC0XM136665	1.071558714
LAMP2	Lysosomal Associated Membrane Protein 2	Protein Coding	GC0XM120426	1.070666075
CSNK1A1	Casein Kinase 1 Alpha 1	Protein Coding	GC05M149492	1.070090294
CDX1	Caudal Type Homeobox 1	Protein Coding	GC05P150166	1.070090294
CDK6	Cyclin Dependent Kinase 6	Protein Coding	GC07M092604	1.069494963
MAP2K3	Mitogen-Activated Protein Kinase Kinase 3	Protein Coding	GC17P030261	1.069494963
BAD	BCL2 Associated Agonist Of Cell	Protein Coding	GC11M071604	1.069494963
UMPS	Uridine Monophosphate Synthetase	Protein Coding	GC03P124730	1.069494963
CD82	CD82 Molecule	Protein Coding	GC11P044586	1.069494963
WNT9A	Wnt Family Member 9A	Protein Coding	GC01M227920	1.069494963
HOTTIP	HOXA Distal Transcript Antisense	RNA Gene	GC07P027198	1.069494963
CLSTN2	Calsyntenin 2	Protein Coding	GC03P139935	1.068832159
APOBEC3G	Apolipoprotein B MRNA Editing Enzyme Catalytic Subunit 3G	Protein Coding	GC22P039078	1.068775177
NFIL3	Nuclear Factor, Interleukin 3 Regulated	Protein Coding	GC09M091409	1.068775177
SCGN	Secretagogin, EF-Hand Calcium Binding Protein	Protein Coding	GC06P025652	1.067488432
BRD4	Bromodomain Containing 4	Protein Coding	GC19M015236	1.067241907
BCL2L15	BCL2 Like 15	Protein Coding	GC01M113876	1.066816688
SLC22A2	Solute Carrier Family 22 Member 2	Protein Coding	GC06M160173	1.064383745
ATP7A	ATPase Copper Transporting Alpha	Protein Coding	GC0XP077948	1.063910961
STX3	Syntaxin 3	Protein Coding	GC11P059713	1.061620593
ITGA1	Integrin Subunit Alpha 1	Protein Coding	GC05P052788	1.061001062
KRT38	Keratin 38	Protein Coding	GC17M041436	1.058272004
ANKRD49	Ankyrin Repeat Domain 49	Protein Coding	GC11P094493	1.056894064
ERBB3	Erb-B2 Receptor Tyrosine Kinase 3	Protein Coding	GC12P056469	1.0526371
DIABLO	Diablo IAP-Binding Mitochondrial Protein	Protein Coding	GC12M122208	1.0526371
CA9	Carbonic Anhydrase 9	Protein Coding	GC09P035673	1.0526371
FANCG	FA Complementation Group G	Protein Coding	GC09M035073	1.0526371
FANCE	FA Complementation Group E	Protein Coding	GC06P058438	1.0526371
SPRY4-IT1	SPRY4 Intronic Transcript 1	RNA Gene	GC05M142318	1.0526371
CCND3	Cyclin D3	Protein Coding	GC06M041934	1.047148228
ABCB5	ATP Binding Cassette Subfamily B Member 5	Protein Coding	GC07P020615	1.047148228
FERMT2	FERM Domain Containing Kindlin 2	Protein Coding	GC14M052857	1.045330882
PAQR7	Progestin And AdipoQ Receptor Family Member 7	Protein Coding	GC01M025861	1.040074348
FBXW2	F-Box And WD Repeat Domain Containing 2	Protein Coding	GC09M120751	1.040074348
KLHL24	Kelch Like Family Member 24	Protein Coding	GC03P183635	1.040074348
CCDC65	Coiled-Coil Domain Containing 65	Protein Coding	GC12P048904	1.040074348
ATP5F1E	ATP Synthase F1 Subunit Epsilon	Protein Coding	GC20M059026	1.040074348
PSMA6	Proteasome 20S Subunit Alpha 6	Protein Coding	GC14P035278	1.028790951
CUL2	Cullin 2	Protein Coding	GC10M035046	1.028790951
DYRK2	Dual Specificity Tyrosine Phosphorylation Regulated Kinase 2	Protein Coding	GC12P067558	1.028790951
USP20	Ubiquitin Specific Peptidase 20	Protein Coding	GC09P129834	1.028790951
TIMP4	TIMP Metallopeptidase Inhibitor 4	Protein Coding	GC03M012153	1.028288364
CUL4B	Cullin 4B	Protein Coding	GC0XM120524	1.028284192
CUL4A	Cullin 4A	Protein Coding	GC13P113208	1.028284192
IL17RB	Interleukin 17 Receptor B	Protein Coding	GC03P053855	1.028284192
NEIL2	Nei Like DNA Glycosylase 2	Protein Coding	GC08P011769	1.028284192
COPS6	COP9 Signalosome Subunit 6	Protein Coding	GC07P100088	1.028284192
NOP58	NOP58 Ribonucleoprotein	Protein Coding	GC02P202265	1.028284192

BCL7C	BAF Chromatin Remodeling Complex Subunit BCL7C	Protein Coding	GC16M031794	1.028284192
WDTC1	WD And Tetratricopeptide Repeats 1	Protein Coding	GC01P027245	1.028284192
EGR2	Early Growth Response 2	Protein Coding	GC10M062811	1.0281955
UBASH3A	Ubiquitin Associated And SH3 Domain Containing A	Protein Coding	GC21P042403	1.0281955
BRMS1	BRMS1 Transcriptional Repressor And Anoikis Regulator	Protein Coding	GC11M071762	1.0281955
TRIM39-RPP21	TRIM39-RPP21 Readthrough	Protein Coding	GC06P058277	1.0281955
BBOX1	Gamma-Butyrobetaine Hydroxylase 1	Protein Coding	GC11P027019	1.020504117
TCN1	Transcobalamin 1	Protein Coding	GC11M071378	1.020504117
FCHO2	FCH And Mu Domain Containing Endocytic Adaptor 2	Protein Coding	GC05P072955	1.020504117
PPARD	Peroxisome Proliferator Activated Receptor Delta	Protein Coding	GC06P058435	1.018355131
PPARGC1A	PPARG Coactivator 1 Alpha	Protein Coding	GC04M023755	1.018355131
GCNT3	Glucosaminyl (N-Acetyl) Transferase 3, Mucin Type	Protein Coding	GC15P059594	1.018355131
NKX2-1	NK2 Homeobox 1	Protein Coding	GC14M036516	1.017513275
BID	BH3 Interacting Domain Death Agonist	Protein Coding	GC22M017734	1.017513275
DKK1	Dickkopf WNT Signaling Pathway Inhibitor 1	Protein Coding	GC10P052314	1.017513275
GADD45A	Growth Arrest And DNA Damage Inducible Alpha	Protein Coding	GC01P067685	1.017513275
FBP1	Fructose-Bisphosphatase 1	Protein Coding	GC09M094603	1.01701045
ADSL	Adenylosuccinate Lyase	Protein Coding	GC22P040346	1.01701045
PHKA2	Phosphorylase Kinase Regulatory Subunit Alpha 2	Protein Coding	GC0XM018892	1.01701045
GAB1	GRB2 Associated Binding Protein 1	Protein Coding	GC04P143336	1.01701045
BCAM	Basal Cell Adhesion Molecule (Lutheran Blood Group)	Protein Coding	GC19P044810	1.01701045
PLXDC2	Plexin Domain Containing 2	Protein Coding	GC10P019769	1.01701045
TASP1	Taspase 1	Protein Coding	GC20M013105	1.01701045
NFATC2	Nuclear Factor Of Activated T Cells 2	Protein Coding	GC20M051386	1.012859344
MARCKS	Myristoylated Alanine Rich Protein Kinase C Substrate	Protein Coding	GC06P113857	1.012859344
IL2RG	Interleukin 2 Receptor Subunit Gamma	Protein Coding	GC0XM071108	1.010568142
BLOC1S1	Biogenesis Of Lysosomal Organelles Complex 1 Subunit 1	Protein Coding	GC12P055775	1.010039091
ABCB9	ATP Binding Cassette Subfamily B Member 9	Protein Coding	GC12M122920	1.009290934
DSC2	Desmocollin 2	Protein Coding	GC18M031058	1.005218863
APLN	Apelin	Protein Coding	GC0XM129645	0.996759832
SOD3	Superoxide Dismutase 3	Protein Coding	GC04P024798	0.991286457
EDN2	Endothelin 2	Protein Coding	GC01M041478	0.97961843
NUMB	NUMB Endocytic Adaptor Protein	Protein Coding	GC14M073275	0.979487479
PRMT1	Protein Arginine Methyltransferase 1	Protein Coding	GC19P049675	0.979487479
EIF4G1	Eukaryotic Translation Initiation Factor 4 Gamma 1	Protein Coding	GC03P184314	0.979487479
MCM7	Minichromosome Maintenance Complex Component 7	Protein Coding	GC07M100092	0.979487479
TUBB2A	Tubulin Beta 2A Class IIa	Protein Coding	GC06M003153	0.979487479
IGFBP1	Insulin Like Growth Factor Binding Protein 1	Protein Coding	GC07P046936	0.979487479
MTA1	Metastasis Associated 1	Protein Coding	GC14P105419	0.979487479
TPBG	Trophoblast Glycoprotein	Protein Coding	GC06P082363	0.979487479
SELENOP	Selenoprotein P	Protein Coding	GC05M042800	0.979487479
PLA2G6	Phospholipase A2 Group VI	Protein Coding	GC22M050884	0.978892088
SOS1	SOS Ras/Rac Guanine Nucleotide Exchange Factor 1	Protein Coding	GC02M038981	0.978892088
ARAF	A-Raf Proto-Oncogene, Serine/Threonine Kinase	Protein Coding	GC0XP047562	0.978892088

WNT10A	Wnt Family Member 10A	Protein Coding	GC02P218880	0.978892088
FABP4	Fatty Acid Binding Protein 4	Protein Coding	GC08M081478	0.978892088
PTPN13	Protein Tyrosine Phosphatase Non-Receptor Type 13	Protein Coding	GC04P086594	0.978892088
ENDOG	Endonuclease G	Protein Coding	GC09P128818	0.978892088
CDKN3	Cyclin Dependent Kinase Inhibitor 3	Protein Coding	GC14P054398	0.978892088
FUT4	Fucosyltransferase 4	Protein Coding	GC11P094544	0.978892088
PTPA	Protein Phosphatase 2 Phosphatase Activator	Protein Coding	GC09P129111	0.978892088
MIR133B	MicroRNA 133b	RNA Gene	GC06P052148	0.978892088
MIR129-2	MicroRNA 129-2	RNA Gene	GC11P043677	0.978892088
MIR137	MicroRNA 137	RNA Gene	GC01M098046	0.978892088
AKT2	AKT Serine/Threonine Kinase 2	Protein Coding	GC19M040230	0.976255417
AKT3	AKT Serine/Threonine Kinase 3	Protein Coding	GC01M243488	0.976255417
EIF4E	Eukaryotic Translation Initiation Factor 4E	Protein Coding	GC04M098879	0.976255417
WNT7A	Wnt Family Member 7A	Protein Coding	GC03M017377	0.976255417
ZEB2	Zinc Finger E-Box Binding Homeobox	Protein Coding	GC02M144384	0.976255417
WNT2B	Wnt Family Member 2B	Protein Coding	GC01P112466	0.976255417
S100A4	S100 Calcium Binding Protein A4	Protein Coding	GC01M153543	0.976255417
TPX2	TPX2 Microtubule Nucleation Factor	Protein Coding	GC20P031739	0.976255417
WNT6	Wnt Family Member 6	Protein Coding	GC02P218859	0.976255417
CDH17	Cadherin 17	Protein Coding	GC08M094127	0.976255417
HAVCR1	Hepatitis A Virus Cellular Receptor 1	Protein Coding	GC05M157028	0.976255417
RBM10	RNA Binding Motif Protein 10	Protein Coding	GC0XP047240	0.976255417
WNT8B	Wnt Family Member 8B	Protein Coding	GC10P100463	0.976255417
PDPN	Podoplanin	Protein Coding	GC01P013583	0.976255417
MTDH	Metadherin	Protein Coding	GC08P097643	0.976255417
H4-16	H4 Histone 16	Protein Coding	GC12M016380	0.976255417
MEG3	Maternally Expressed 3	RNA Gene	GC14P106791	0.976255417
CASC2	Cancer Susceptibility 2	RNA Gene	GC10P118046	0.976255417
XIST	X Inactive Specific Transcript	RNA Gene	GC0XM073820	0.976255417
SOX2-OT	SOX2 Overlapping Transcript	RNA Gene	GC03P180989	0.976255417
TINCR	TINCR Ubiquitin Domain Containing	RNA Gene	GC19M005558	0.976255417
DANCR	Differentiation Antagonizing Non-Protein Coding RNA	RNA Gene	GC04P052712	0.976255417
FER1L4	Fer-1 Like Family Member 4 (Pseudogene)	Pseudogene	GC20M035558	0.976255417
MIR125B1	MicroRNA 125b-1	RNA Gene	GC11M122100	0.976255417
SNHG12	Small Nucleolar RNA Host Gene 12	RNA Gene	GC01M028578	0.976255417
HULC	Hepatocellular Carcinoma Up-Regulated Long Non-Coding RNA	RNA Gene	GC06P008438	0.976255417
MIRLET7G	MicroRNA Let-7g	RNA Gene	GC03M052268	0.976255417
MIR345	MicroRNA 345	RNA Gene	GC14P100307	0.976255417
SNHG1	Small Nucleolar RNA Host Gene 1	RNA Gene	GC11M071539	0.976255417
SNHG5	Small Nucleolar RNA Host Gene 5	RNA Gene	GC06M085650	0.976255417
ZFAS1	ZNFX1 Antisense RNA 1	RNA Gene	GC20P049276	0.976255417
LINC00261	Long Intergenic Non-Protein Coding RNA 261	RNA Gene	GC20M022547	0.976255417
MIR107	MicroRNA 107	RNA Gene	GC10M089600	0.976255417
MIR24-2	MicroRNA 24-2	RNA Gene	GC19M014148	0.976255417
MIR31HG	MIR31 Host Gene	RNA Gene	GC09M021439	0.976255417
BCAR4	Breast Cancer Anti-Estrogen Resistance	RNA Gene	GC16M011819	0.976255417
HNF1A-AS1	HNF1A Antisense RNA 1	RNA Gene	GC12M121584	0.976255417
HOXA-AS2	HOXA Cluster Antisense RNA 2	RNA Gene	GC07P027107	0.976255417
PCAT1	Prostate Cancer Associated Transcript 1	RNA Gene	GC08P126553	0.976255417
MIR296	MicroRNA 296	RNA Gene	GC20M058817	0.976255417
MIR130B	MicroRNA 130b	RNA Gene	GC22P027760	0.976255417
SNHG20	Small Nucleolar RNA Host Gene 20	RNA Gene	GC17P077087	0.976255417
SNHG16	Small Nucleolar RNA Host Gene 16	RNA Gene	GC17P076811	0.976255417
LINC-ROR	Long Intergenic Non-Protein Coding RNA, Regulator Of Reprogramming	RNA Gene	GC18M057054	0.976255417
SNHG6	Small Nucleolar RNA Host Gene 6	RNA Gene	GC08M066921	0.976255417

ZEB1-AS1	ZEB1 Antisense RNA 1	RNA Gene	GC10M031166	0.976255417
AFAP1-AS1	AFAP1 Antisense RNA 1	RNA Gene	GC04P007756	0.976255417
RPL34-DT	RPL34 Divergent Transcript	RNA Gene	GC04M108539	0.976255417
TUSC7	Tumor Suppressor Candidate 7	RNA Gene	GC03P116647	0.976255417
CCAT1	Colon Cancer Associated Transcript 1	RNA Gene	GC08M127207	0.976255417
HEIH	Hepatocellular Carcinoma Up-Regulated EZH2-Associated Long Non-	RNA Gene	GC05M180976	0.976255417
FEZF1-AS1	FEZF1 Antisense RNA 1	RNA Gene	GC07P122303	0.976255417
BLACAT1	Bladder Cancer Associated Transcript 1	RNA Gene	GC01M205386	0.976255417
CCAT2	Colon Cancer Associated Transcript 2	RNA Gene	GC08P127400	0.976255417
GAPLINC	Gastric Adenocarcinoma Associated, Positive CD44 Regulator, Long Intergenic Non-Coding RNA	RNA Gene	GC18P003467	0.976255417
PRNCR1	Prostate Cancer Associated Non-Coding RNA 1	RNA Gene	GC08P127079	0.976255417
TMEM238L	Transmembrane Protein 238 Like	Protein Coding	GC17M010795	0.976255417
BANCR	BRAF-Activated Non-Protein Coding RNA	RNA Gene	GC09M069296	0.976255417
NPTN-IT1	NPTN Intronic Transcript 1	RNA Gene	GC15M073566	0.976255417
NCRUPAR	Non-Protein Coding RNA, Upstream Of F2R/PAR1	RNA Gene	GC05P076711	0.976255417
PANDAR	Promoter Of CDKN1A Antisense DNA Damage Activated RNA	RNA Gene	GC06M036673	0.976255417
GHET1	Gastric Carcinoma Proliferation Enhancing Transcript 1	RNA Gene	GC07P149094	0.976255417
DUXAP9	Double Homeobox A Pseudogene 9	Pseudogene	GC14P021915	0.976255417
TP53COR1	Tumor Protein P53 Pathway Corepressor 1	RNA Gene	GC06U903133	0.976255417
LNCRNA-ATB	Long Noncoding RNA Activated By TGF-Beta	RNA Gene	GC14P026644	0.976255417
TNPO1	Transportin 1	Protein Coding	GC05P072816	0.975748301
H2BC21	H2B Clustered Histone 21	Protein Coding	GC01M150210	0.974106431
ST8SIA4	ST8 Alpha-N-Acetyl-Neuraminide Alpha-2,8-Sialyltransferase 4	Protein Coding	GC05M100806	0.961973071
SLC39A12	Solute Carrier Family 39 Member 12	Protein Coding	GC10P017951	0.961973071
DCLRE1B	DNA Cross-Link Repair 1B	Protein Coding	GC01P113905	0.961973071
MFSD2A	Major Facilitator Superfamily Domain Containing 2A	Protein Coding	GC01P039955	0.961202264
GDNF	Glial Cell Derived Neurotrophic Factor	Protein Coding	GC05M037812	0.959906816
BIN1	Bridging Integrator 1	Protein Coding	GC02M127048	0.957645535
BMP7	Bone Morphogenetic Protein 7	Protein Coding	GC20M057168	0.95523715
MYO5B	Myosin VB	Protein Coding	GC18M049822	0.952375293
FCN1	Ficolin 1	Protein Coding	GC09M135040	0.952375293
RBMS1	RNA Binding Motif Single Stranded Interacting Protein 1	Protein Coding	GC02M160272	0.952375293
OPRM1	Opioid Receptor Mu 1	Protein Coding	GC06P154075	0.95134002
HCK	HCK Proto-Oncogene, Src Family Tyrosine Kinase	Protein Coding	GC20P032052	0.950699329
CSF2RA	Colony Stimulating Factor 2 Receptor Subunit Alpha	Protein Coding	GC0XP001349	0.950699329
RNASET2	Ribonuclease T2	Protein Coding	GC06M166924	0.950699329
PADI2	Peptidyl Arginine Deiminase 2	Protein Coding	GC01M017066	0.950699329
SLAMF7	SLAM Family Member 7	Protein Coding	GC01P160709	0.950699329
CD5	CD5 Molecule	Protein Coding	GC11P061114	0.950699329
POP4	POP4 Homolog, Ribonuclease P/MRP Subunit	Protein Coding	GC19P029604	0.950699329
SELENOS	Selenoprotein S	Protein Coding	GC15M108722	0.947330952
CTSE	Cathepsin E	Protein Coding	GC01M206009	0.943576574
GC	GC Vitamin D Binding Protein	Protein Coding	GC04M071741	0.942668319
MMP28	Matrix Metallopeptidase 28	Protein Coding	GC17M035756	0.941355407
SMPD1	Sphingomyelin Phosphodiesterase 1	Protein Coding	GC11P006390	0.941175818
CAMK2A	Calcium/Calmodulin Dependent Protein Kinase II Alpha	Protein Coding	GC05M150219	0.934955955

RAD50	RAD50 Double Strand Break Repair Protein	Protein Coding	GC05P132556	0.934955955
F12	Coagulation Factor XII	Protein Coding	GC05M177402	0.934955955
FES	FES Proto-Oncogene, Tyrosine Kinase	Protein Coding	GC15P090883	0.934955955
PTGIS	Prostaglandin I2 Synthase	Protein Coding	GC20M049503	0.934955955
PDXK	Pyridoxal Kinase	Protein Coding	GC21P043719	0.934955955
CEBPA	CCAAT Enhancer Binding Protein	Protein Coding	GC19M033299	0.934955955
GLS	Glutaminase	Protein Coding	GC02P190880	0.934955955
GALT	Galactose-1-Phosphate Uridylyltransferase	Protein Coding	GC09P036236	0.934955955
SCNN1A	Sodium Channel Epithelial 1 Subunit Alpha	Protein Coding	GC12M006346	0.934955955
SLC25A20	Solute Carrier Family 25 Member 20	Protein Coding	GC03M048869	0.934955955
CD81	CD81 Molecule	Protein Coding	GC11P002434	0.934955955
CTSF	Cathepsin F	Protein Coding	GC11M071788	0.934955955
IMPDH2	Inosine Monophosphate Dehydrogenase	Protein Coding	GC03M049512	0.934955955
PPP1CB	Protein Phosphatase 1 Catalytic Subunit Beta	Protein Coding	GC02P028752	0.934955955
PRKD2	Protein Kinase D2	Protein Coding	GC19M046674	0.934955955
PRKAR2A	Protein Kinase CAMP-Dependent Type II Regulatory Subunit Alpha	Protein Coding	GC03M048744	0.934955955
DOT1L	DOT1 Like Histone Lysine Methyltransferase	Protein Coding	GC19P002164	0.934955955
ANXA11	Annexin A11	Protein Coding	GC10M080150	0.934955955
GRK6	G Protein-Coupled Receptor Kinase 6	Protein Coding	GC05P177403	0.934955955
CHRNB2	Cholinergic Receptor Nicotinic Beta 2 Subunit	Protein Coding	GC01P154568	0.934955955
LAMB2	Laminin Subunit Beta 2	Protein Coding	GC03M049121	0.934955955
MSX1	Msh Homeobox 1	Protein Coding	GC04P004861	0.934955955
IL11RA	Interleukin 11 Receptor Subunit Alpha	Protein Coding	GC09P034650	0.934955955
NEK9	NIMA Related Kinase 9	Protein Coding	GC14M075079	0.934955955
SEC24C	SEC24 Homolog C, COPII Coat Complex Component	Protein Coding	GC10P073744	0.934955955
RAPGEF3	Rap Guanine Nucleotide Exchange Factor 3	Protein Coding	GC12M047736	0.934955955
VAMP1	Vesicle Associated Membrane Protein	Protein Coding	GC12M006462	0.934955955
HDAC11	Histone Deacetylase 11	Protein Coding	GC03P013478	0.934955955
HINT1	Histidine Triad Nucleotide Binding Protein 1	Protein Coding	GC05M131159	0.934955955
POFUT1	Protein O-Fucosyltransferase 1	Protein Coding	GC20P032207	0.934955955
PPIF	Peptidylprolyl Isomerase F	Protein Coding	GC10P086014	0.934955955
PPP1R1B	Protein Phosphatase 1 Regulatory Inhibitor Subunit 1B	Protein Coding	GC17P039626	0.934955955
MAPKAPK5	MAPK Activated Protein Kinase 5	Protein Coding	GC12P111842	0.934955955
MPDZ	Multiple PDZ Domain Crumbs Cell Polarity Complex Component	Protein Coding	GC09M013095	0.934955955
PTPRK	Protein Tyrosine Phosphatase Receptor Type K	Protein Coding	GC06M127949	0.934955955
TNNT3	Troponin T3, Fast Skeletal Type	Protein Coding	GC11P001920	0.934955955
TUFM	Tu Translation Elongation Factor, Mitochondrial	Protein Coding	GC16M031436	0.934955955
ADAMTS4	ADAM Metallopeptidase With Thrombospondin Type 1 Motif 4	Protein Coding	GC01M161184	0.934955955
ATP6V1G3	ATPase H+ Transporting V1 Subunit	Protein Coding	GC01M198492	0.934955955
ATXN2	Ataxin 2	Protein Coding	GC12M111443	0.934955955
AUH	AU RNA Binding Methylglutaconyl-CoA Hydratase	Protein Coding	GC09M091213	0.934955955
ABI1	Abl Interactor 1	Protein Coding	GC10M026746	0.934955955
CCS	Copper Chaperone For Superoxide Dismutase	Protein Coding	GC11P066593	0.934955955
FCER1G	Fc Fragment Of IgE Receptor Ig	Protein Coding	GC01P161215	0.934955955
HIBCH	3-Hydroxyisobutyryl-CoA Hydrolase	Protein Coding	GC02M190189	0.934955955
KAT6B	Lysine Acetyltransferase 6B	Protein Coding	GC10P074843	0.934955955

PNPLA8	Patatin Like Phospholipase Domain Containing 8	Protein Coding	GC07M108470	0.934955955
PLXNB1	Plexin B1	Protein Coding	GC03M048403	0.934955955
POLR2E	RNA Polymerase II, I And III Subunit	Protein Coding	GC19M001086	0.934955955
LZTR1	Leucine Zipper Like Transcription Regulator 1	Protein Coding	GC22P021221	0.934955955
MEIS1	Meis Homeobox 1	Protein Coding	GC02P066433	0.934955955
PTBP1	Polypyrimidine Tract Binding Protein 1	Protein Coding	GC19P000797	0.934955955
TCAP	Titin-Cap	Protein Coding	GC17P039673	0.934955955
TRPM2	Transient Receptor Potential Cation Channel Subfamily M Member 2	Protein Coding	GC21P044350	0.934955955
SLC26A4	Solute Carrier Family 26 Member 4	Protein Coding	GC07P107660	0.934955955
SLC13A2	Solute Carrier Family 13 Member 2	Protein Coding	GC17P028473	0.934955955
UQCRC1	Ubiquinol-Cytochrome C Reductase Core Protein 1	Protein Coding	GC03M048598	0.934955955
SENP1	SUMO Specific Peptidase 1	Protein Coding	GC12M048042	0.934955955
RBMX	RNA Binding Motif Protein X-Linked	Protein Coding	GC0XM136848	0.934955955
AGPAT1	1-Acylglycerol-3-Phosphate O-Acyltransferase 1	Protein Coding	GC06M032168	0.934955955
EMP2	Epithelial Membrane Protein 2	Protein Coding	GC16M010541	0.934955955
HOXA10	Homeobox A10	Protein Coding	GC07M027369	0.934955955
KLC2	Kinesin Light Chain 2	Protein Coding	GC11P066257	0.934955955
KDELR2	KDEL Endoplasmic Reticulum Protein Retention Receptor 2	Protein Coding	GC07M006447	0.934955955
NEUROD2	Neuronal Differentiation 2	Protein Coding	GC17M039603	0.934955955
PRPF6	Pre-MRNA Processing Factor 6	Protein Coding	GC20P063981	0.934955955
TMED10	Transmembrane P24 Trafficking Protein 10	Protein Coding	GC14M075132	0.934955955
TOM1	Target Of Myb1 Membrane Trafficking Protein	Protein Coding	GC22P035299	0.934955955
SCNN1D	Sodium Channel Epithelial 1 Subunit Delta	Protein Coding	GC01P001280	0.934955955
BRAP	BRCA1 Associated Protein	Protein Coding	GC12M111642	0.934955955
AFF4	AF4/FMR2 Family Member 4	Protein Coding	GC05M132875	0.934955955
BATF	Basic Leucine Zipper ATF-Like Transcription Factor	Protein Coding	GC14P075523	0.934955955
HOXA11	Homeobox A11	Protein Coding	GC07M027365	0.934955955
HIPK1	Homeodomain Interacting Protein Kinase 1	Protein Coding	GC01P113929	0.934955955
HLA-F	Major Histocompatibility Complex, Class I, F	Protein Coding	GC06P058234	0.934955955
GLYAT	Glycine-N-Acyltransferase	Protein Coding	GC11M071339	0.934955955
KIF3A	Kinesin Family Member 3A	Protein Coding	GC05M132689	0.934955955
HIBADH	3-Hydroxyisobutyrate Dehydrogenase	Protein Coding	GC07M027525	0.934955955
LHX3	LIM Homeobox 3	Protein Coding	GC09M136196	0.934955955
NPEPPS	Aminopeptidase Puromycin Sensitive	Protein Coding	GC17P047522	0.934955955
NRCAM	Neuronal Cell Adhesion Molecule	Protein Coding	GC07M108147	0.934955955
PPA2	Inorganic Pyrophosphatase 2	Protein Coding	GC04M105369	0.934955955
LPAR5	Lysophosphatidic Acid Receptor 5	Protein Coding	GC12M006618	0.934955955
LRIG2	Leucine Rich Repeats And Immunoglobulin Like Domains 2	Protein Coding	GC01P113073	0.934955955
PASK	PAS Domain Containing Serine/Threonine Kinase	Protein Coding	GC02M241106	0.934955955
MED1	Mediator Complex Subunit 1	Protein Coding	GC17M039404	0.934955955
SLC2A13	Solute Carrier Family 2 Member 13	Protein Coding	GC12M039755	0.934955955
UBE2J2	Ubiquitin Conjugating Enzyme E2 J2	Protein Coding	GC01M003068	0.934955955
RAB1B	RAB1B, Member RAS Oncogene	Protein Coding	GC11P066894	0.934955955
PEX13	Peroxisomal Biogenesis Factor 13	Protein Coding	GC02P061017	0.934955955
TOP3B	DNA Topoisomerase III Beta	Protein Coding	GC22M021957	0.934955955
VAMP3	Vesicle Associated Membrane Protein	Protein Coding	GC01P007765	0.934955955
CAMTA1	Calmodulin Binding Transcription Activator 1	Protein Coding	GC01P006825	0.934955955
DBN1	Drebrin 1	Protein Coding	GC05M177456	0.934955955

DSE	Dermatan Sulfate Epimerase	Protein Coding	GC06P116255	0.934955955
ACTR1A	Actin Related Protein 1A	Protein Coding	GC10M102479	0.934955955
BAZ1A	Bromodomain Adjacent To Zinc Finger Domain 1A	Protein Coding	GC14M034752	0.934955955
APOBEC3A	Apolipoprotein B MRNA Editing Enzyme Catalytic Subunit 3A	Protein Coding	GC22P038952	0.934955955
DAGLB	Diacylglycerol Lipase Beta	Protein Coding	GC07M006416	0.934955955
DAP3	Death Associated Protein 3	Protein Coding	GC01P155688	0.934955955
HIF3A	Hypoxia Inducible Factor 3 Subunit	Protein Coding	GC19P046297	0.934955955
GNPDA1	Glucosamine-6-Phosphate Deaminase 1	Protein Coding	GC05M141991	0.934955955
GDI2	GDP Dissociation Inhibitor 2	Protein Coding	GC10M005765	0.934955955
LIMD1	LIM Domain Containing 1	Protein Coding	GC03P045555	0.934955955
GRIN3B	Glutamate Ionotropic Receptor NMDA Type Subunit 3B	Protein Coding	GC19P001000	0.934955955
FKRP	Fukutin Related Protein	Protein Coding	GC19P046746	0.934955955
KCNK10	Potassium Two Pore Domain Channel Subfamily K Member 10	Protein Coding	GC14M088180	0.934955955
FAU	FAU Ubiquitin Like And Ribosomal Protein S30 Fusion	Protein Coding	GC11M065120	0.934955955
NDUFAF3	NADH:Ubiquinone Oxidoreductase Complex Assembly Factor 3	Protein Coding	GC03P049020	0.934955955
PPIL2	Peptidylprolyl Isomerase Like 2	Protein Coding	GC22P027916	0.934955955
JDP2	Jun Dimerization Protein 2	Protein Coding	GC14P075427	0.934955955
NDST2	N-Deacetylase And N-Sulfotransferase	Protein Coding	GC10M073801	0.934955955
RBM4	RNA Binding Motif Protein 4	Protein Coding	GC11P066638	0.934955955
STARD3	StAR Related Lipid Transfer Domain Containing 3	Protein Coding	GC17P039637	0.934955955
STK19	Serine/Threonine Kinase 19	Protein Coding	GC06P031971	0.934955955
SON	SON DNA And RNA Binding Protein	Protein Coding	GC21P033542	0.934955955
TAX1BP1	Tax1 Binding Protein 1	Protein Coding	GC07P027739	0.934955955
SLC6A7	Solute Carrier Family 6 Member 7	Protein Coding	GC05P150189	0.934955955
SLC35D1	Solute Carrier Family 35 Member D1	Protein Coding	GC01M066999	0.934955955
SH3PXD2B	SH3 And PX Domains 2B	Protein Coding	GC05M172325	0.934955955
OLIG2	Oligodendrocyte Transcription Factor 2	Protein Coding	GC21P033025	0.934955955
SNRPD2	Small Nuclear Ribonucleoprotein D2 Polypeptide	Protein Coding	GC19M048749	0.934955955
TAS1R3	Taste 1 Receptor Member 3	Protein Coding	GC01P001331	0.934955955
TNS1	Tensin 1	Protein Coding	GC02M217799	0.934955955
SART1	Spliceosome Associated Factor 1, Recruiter Of U4/U6.U5 Tri-SnRNP	Protein Coding	GC11P066871	0.934955955
B4GALT5	Beta-1,4-Galactosyltransferase 5	Protein Coding	GC20M049632	0.934955955
ASIC2	Acid Sensing Ion Channel Subunit 2	Protein Coding	GC17M033013	0.934955955
CDK11B	Cyclin Dependent Kinase 11B	Protein Coding	GC01M003099	0.934955955
DNMBP	Dynamin Binding Protein	Protein Coding	GC10M099875	0.934955955
ATP8B3	ATPase Phospholipid Transporting 8B3	Protein Coding	GC19M002389	0.934955955
B3GALT6	Beta-1,3-Galactosyltransferase 6	Protein Coding	GC01P001232	0.934955955
ARIH2	Ariadne RBR E3 Ubiquitin Protein Ligase 2	Protein Coding	GC03P048918	0.934955955
APOM	Apolipoprotein M	Protein Coding	GC06P058317	0.934955955
CELSR3	Cadherin EGF LAG Seven-Pass G-Type Receptor 3	Protein Coding	GC03M048641	0.934955955
HCN3	Hyperpolarization Activated Cyclic Nucleotide Gated Potassium Channel 3	Protein Coding	GC01P155277	0.934955955
CST6	Cystatin E/M	Protein Coding	GC11P066877	0.934955955
IFT172	Intraflagellar Transport 172	Protein Coding	GC02M027724	0.934955955
NAB1	NGFI-A Binding Protein 1	Protein Coding	GC02P190646	0.934955955
LRRC32	Leucine Rich Repeat Containing 32	Protein Coding	GC11M076657	0.934955955
RALGAPA1	Ral GTPase Activating Protein Catalytic Subunit Alpha 1	Protein Coding	GC14M035538	0.934955955
SF3B2	Splicing Factor 3b Subunit 2	Protein Coding	GC11P066050	0.934955955
TDRKH	Tudor And KH Domain Containing	Protein Coding	GC01M151830	0.934955955
SYMPK	Symplekin	Protein Coding	GC19M045815	0.934955955
SLC16A10	Solute Carrier Family 16 Member 10	Protein Coding	GC06P111087	0.934955955

SIX5	SIX Homeobox 5	Protein Coding	GC19M045764	0.934955955
UQCR10	Ubiquinol-Cytochrome C Reductase, Complex III Subunit X	Protein Coding	GC22P029767	0.934955955
USP21	Ubiquitin Specific Peptidase 21	Protein Coding	GC01P161159	0.934955955
RBM17	RNA Binding Motif Protein 17	Protein Coding	GC10P006088	0.934955955
RIN1	Ras And Rab Interactor 1	Protein Coding	GC11M071761	0.934955955
RPL37	Ribosomal Protein L37	Protein Coding	GC05M040825	0.934955955
YY1AP1	YY1 Associated Protein 1	Protein Coding	GC01M155659	0.934955955
WTAP	WT1 Associated Protein	Protein Coding	GC06P159725	0.934955955
TUFT1	Tuftelin 1	Protein Coding	GC01P151513	0.934955955
ASAP2	ArfGAP With SH3 Domain, Ankyrin Repeat And PH Domain 2	Protein Coding	GC02P009206	0.934955955
CBFA2T2	CBFA2/RUNX1 Partner Transcriptional Co-Repressor 2	Protein Coding	GC20P033490	0.934955955
DAZAP1	DAZ Associated Protein 1	Protein Coding	GC19P001407	0.934955955
ADGRL2	Adhesion G Protein-Coupled Receptor	Protein Coding	GC01P081306	0.934955955
APOL6	Apolipoprotein L6	Protein Coding	GC22P035648	0.934955955
BBS1	Bardet-Biedl Syndrome 1	Protein Coding	GC11P066956	0.934955955
DIDO1	Death Inducer-Obliterator 1	Protein Coding	GC20M062877	0.934955955
HBP1	HMG-Box Transcription Factor 1	Protein Coding	GC07P107168	0.934955955
GPSM1	G Protein Signaling Modulator 1	Protein Coding	GC09P136327	0.934955955
COG5	Component Of Oligomeric Golgi Complex 5	Protein Coding	GC07M107201	0.934955955
GRHL3	Grainyhead Like Transcription Factor 3	Protein Coding	GC01P024319	0.934955955
GJA10	Gap Junction Protein Alpha 10	Protein Coding	GC06P089894	0.934955955
KCTD15	Potassium Channel Tetramerization Domain Containing 15	Protein Coding	GC19P033843	0.934955955
NRBF2	Nuclear Receptor Binding Factor 2	Protein Coding	GC10P063133	0.934955955
PGS1	Phosphatidylglycerophosphate Synthase	Protein Coding	GC17P078378	0.934955955
ITGB1BP1	Integrin Subunit Beta 1 Binding Protein	Protein Coding	GC02M009391	0.934955955
PCOLCE	Procollagen C-Endopeptidase Enhancer	Protein Coding	GC07P100602	0.934955955
PAPOLG	Poly(A) Polymerase Gamma	Protein Coding	GC02P060756	0.934955955
MAN2A2	Mannosidase Alpha Class 2A Member	Protein Coding	GC15P090902	0.934955955
MPPE1	Metallophosphoesterase 1	Protein Coding	GC18M019708	0.934955955
MRPL11	Mitochondrial Ribosomal Protein L11	Protein Coding	GC11M071769	0.934955955
SEPHS2	Selenophosphate Synthetase 2	Protein Coding	GC16M031753	0.934955955
SRRT	Serrate, RNA Effector Molecule	Protein Coding	GC07P100875	0.934955955
TPD52L2	TPD52 Like 2	Protein Coding	GC20P063865	0.934955955
TRAPPC10	Trafficking Protein Particle Complex Subunit 10	Protein Coding	GC21P044012	0.934955955
TIPIN	TIMELESS Interacting Protein	Protein Coding	GC15M068961	0.934955955
THEMIS	Thymocyte Selection Associated	Protein Coding	GC06M127708	0.934955955
USP36	Ubiquitin Specific Peptidase 36	Protein Coding	GC17M078787	0.934955955
USP37	Ubiquitin Specific Peptidase 37	Protein Coding	GC02M218450	0.934955955
RAPGEF6	Rap Guanine Nucleotide Exchange Factor 6	Protein Coding	GC05M131423	0.934955955
OLIG1	Oligodendrocyte Transcription Factor 1	Protein Coding	GC21P033070	0.934955955
PGAP3	Post-GPI Attachment To Proteins Phospholipase 3	Protein Coding	GC17M039676	0.934955955
SNX27	Sorting Nexin 27	Protein Coding	GC01P151611	0.934955955
TOR1B	Torsin Family 1 Member B	Protein Coding	GC09P129803	0.934955955
AURKAIP1	Aurora Kinase A Interacting Protein 1	Protein Coding	GC01M001373	0.934955955
ATP6V0E1	ATPase H+ Transporting V0 Subunit	Protein Coding	GC05P172983	0.934955955
ACYP1	Acylphosphatase 1	Protein Coding	GC14M075053	0.934955955
ADO	2-Aminoethanethiol Dioxygenase	Protein Coding	GC10P062804	0.934955955
CDK11A	Cyclin Dependent Kinase 11A	Protein Coding	GC01M001702	0.934955955
BOD1	Biorientation Of Chromosomes In Cell Division 1	Protein Coding	GC05M173607	0.934955955
CBLL1	Cbl Proto-Oncogene Like 1	Protein Coding	GC07P107743	0.934955955
ECD	Ecdysoneless Cell Cycle Regulator	Protein Coding	GC10M073130	0.934955955
DOK6	Docking Protein 6	Protein Coding	GC18P069401	0.934955955
DPH5	Diphthamide Biosynthesis 5	Protein Coding	GC01M100989	0.934955955
DRAP1	DR1 Associated Protein 1	Protein Coding	GC11P066864	0.934955955

DUSP12	Dual Specificity Phosphatase 12	Protein Coding	GC01P161749	0.934955955
CPSF3	Cleavage And Polyadenylation Specific Factor 3	Protein Coding	GC02P009423	0.934955955
FBXL20	F-Box And Leucine Rich Repeat Protein 20	Protein Coding	GC17M039252	0.934955955
ERRFI1	ERBB Receptor Feedback Inhibitor 1	Protein Coding	GC01M008004	0.934955955
ING4	Inhibitor Of Growth Family Member 4	Protein Coding	GC12M006650	0.934955955
GPN1	GPN-Loop GTPase 1	Protein Coding	GC02P027628	0.934955955
FNIP1	Folliculin Interacting Protein 1	Protein Coding	GC05M131641	0.934955955
CH25H	Cholesterol 25-Hydroxylase	Protein Coding	GC10M089205	0.934955955
TM9SF2	Transmembrane 9 Superfamily Member	Protein Coding	GC13P099446	0.934955955
FAM177A1	Family With Sequence Similarity 177 Member A1	Protein Coding	GC14P035044	0.934955955
MRPL23	Mitochondrial Ribosomal Protein L23	Protein Coding	GC11P001948	0.934955955
PLB1	Phospholipase B1	Protein Coding	GC02P028460	0.934955955
PMF1	Polyamine Modulated Factor 1	Protein Coding	GC01P156212	0.934955955
MED24	Mediator Complex Subunit 24	Protein Coding	GC17M040019	0.934955955
OLFML3	Olfactomedin Like 3	Protein Coding	GC01P113979	0.934955955
PKIG	CAMP-Dependent Protein Kinase Inhibitor Gamma	Protein Coding	GC20P044531	0.934955955
TRIM8	Tripartite Motif Containing 8	Protein Coding	GC10P102643	0.934955955
TRIM26	Tripartite Motif Containing 26	Protein Coding	GC06M030184	0.934955955
SLC12A9	Solute Carrier Family 12 Member 9	Protein Coding	GC07P100826	0.934955955
UCKL1	Uridine-Cytidine Kinase 1 Like 1	Protein Coding	GC20M063939	0.934955955
RBM7	RNA Binding Motif Protein 7	Protein Coding	GC11P114401	0.934955955
REXO2	RNA Exonuclease 2	Protein Coding	GC11P114439	0.934955955
RLN2	Relaxin 2	Protein Coding	GC09M005306	0.934955955
RSPH4A	Radial Spoke Head Component 4A	Protein Coding	GC06P116616	0.934955955
ZBTB40	Zinc Finger And BTB Domain Containing 40	Protein Coding	GC01P022428	0.934955955
ZNF3	Zinc Finger Protein 3	Protein Coding	GC07M100063	0.934955955
BAG6	BAG Cochaperone 6	Protein Coding	GC06M031639	0.934955955
ATAD3B	ATPase Family AAA Domain Containing 3B	Protein Coding	GC01P002252	0.934955955
CASC3	CASC3 Exon Junction Complex	Protein Coding	GC17P040140	0.934955955
DUSP8	Dual Specificity Phosphatase 8	Protein Coding	GC11M001531	0.934955955
EAPP	E2F Associated Phosphoprotein	Protein Coding	GC14M034516	0.934955955
DDX27	DEAD-Box Helicase 27	Protein Coding	GC20P049219	0.934955955
ACAP3	ArfGAP With Coiled-Coil, Ankyrin Repeat And PH Domains 3	Protein Coding	GC01M001292	0.934955955
ANKRD30A	Ankyrin Repeat Domain 30A	Protein Coding	GC10P037134	0.934955955
CCNL2	Cyclin L2	Protein Coding	GC01M001385	0.934955955
CENPO	Centromere Protein O	Protein Coding	GC02P024793	0.934955955
CEP192	Centrosomal Protein 192	Protein Coding	GC18P015003	0.934955955
BCAP29	B Cell Receptor Associated Protein 29	Protein Coding	GC07P107579	0.934955955
CEP89	Centrosomal Protein 89	Protein Coding	GC19M032878	0.934955955
DNAH17	Dynein Axonemal Heavy Chain 17	Protein Coding	GC17M078423	0.934955955
DNAJC9	DnaJ Heat Shock Protein Family (Hsp40) Member C9	Protein Coding	GC10M073183	0.934955955
EMC1	ER Membrane Protein Complex	Protein Coding	GC01M019215	0.934955955
FITM2	Fat Storage Inducing Transmembrane Protein 2	Protein Coding	GC20M044302	0.934955955
IFNLR1	Interferon Lambda Receptor 1	Protein Coding	GC01M024230	0.934955955
DNAJC27	DnaJ Heat Shock Protein Family (Hsp40) Member C27	Protein Coding	GC02M024943	0.934955955
GPR25	G Protein-Coupled Receptor 25	Protein Coding	GC01P200872	0.934955955
RSPH3	Radial Spoke Head 3	Protein Coding	GC06M158948	0.934955955
NAALADL1	N-Acetylated Alpha-Linked Acidic Dipeptidase Like 1	Protein Coding	GC11M065044	0.934955955
MRPL20	Mitochondrial Ribosomal Protein L20	Protein Coding	GC01M001401	0.934955955
MRPS27	Mitochondrial Ribosomal Protein S27	Protein Coding	GC05M072219	0.934955955
LPCAT3	Lysophosphatidylcholine Acyltransferase 3	Protein Coding	GC12M006976	0.934955955

NPAS4	Neuronal PAS Domain Protein 4	Protein Coding	GC11P066924	0.934955955
PSMG2	Proteasome Assembly Chaperone 2	Protein Coding	GC18P014993	0.934955955
LY6G6F	Lymphocyte Antigen 6 Family Member G6F	Protein Coding	GC06P031706	0.934955955
MYOZ1	Myozenin 1	Protein Coding	GC10M073631	0.934955955
LYRM7	LYR Motif Containing 7	Protein Coding	GC05P131170	0.934955955
LRCH4	Leucine Rich Repeats And Calponin Homology Domain Containing 4	Protein Coding	GC07M100574	0.934955955
P4HTM	Prolyl 4-Hydroxylase, Transmembrane	Protein Coding	GC03P049317	0.934955955
PWP2	PWP2 Small Subunit Processome Component	Protein Coding	GC21P044107	0.934955955
SHISA5	Shisa Family Member 5	Protein Coding	GC03M048468	0.934955955
SERBP1	SERPINE1 MRNA Binding Protein 1	Protein Coding	GC01M067407	0.934955955
STAC2	SH3 And Cysteine Rich Domain 2	Protein Coding	GC17M039250	0.934955955
SNX13	Sorting Nexin 13	Protein Coding	GC07M017798	0.934955955
TAPBPL	TAP Binding Protein Like	Protein Coding	GC12P006451	0.934955955
TSPAN32	Tetraspanin 32	Protein Coding	GC11P002302	0.934955955
THEM5	Thioesterase Superfamily Member 5	Protein Coding	GC01M151850	0.934955955
SLC9A4	Solute Carrier Family 9 Member A4	Protein Coding	GC02P102456	0.934955955
YTHDF1	YTH N6-Methyladenosine RNA Binding Protein 1	Protein Coding	GC20M063195	0.934955955
VARS1	Valyl-TRNA Synthetase 1	Protein Coding	GC06M049131	0.934955955
QSOX2	Quiescin Sulfhydryl Oxidase 2	Protein Coding	GC09M136206	0.934955955
RAB24	RAB24, Member RAS Oncogene	Protein Coding	GC05M177301	0.934955955
RBM14	RNA Binding Motif Protein 14	Protein Coding	GC11P066972	0.934955955
PRELID1	PRELI Domain Containing 1	Protein Coding	GC05P177305	0.934955955
RPL26L1	Ribosomal Protein L26 Like 1	Protein Coding	GC05P172958	0.934955955
SENP7	SUMO Specific Peptidase 7	Protein Coding	GC03M101324	0.934955955
RNF126	Ring Finger Protein 126	Protein Coding	GC19M000647	0.934955955
RTF1	RTF1 Homolog, Paf1/RNA Polymerase II Complex Component	Protein Coding	GC15P041535	0.934955955
TNRC6C	Trinucleotide Repeat Containing Adaptor 6C	Protein Coding	GC17P077959	0.934955955
WIPF2	WAS/WASL Interacting Protein Family Member 2	Protein Coding	GC17P040219	0.934955955
ZNF384	Zinc Finger Protein 384	Protein Coding	GC12M006715	0.934955955
ASCC2	Activating Signal Cointegrator 1 Complex Subunit 2	Protein Coding	GC22M029788	0.934955955
BNIP1	BCL2 Interacting Protein 1	Protein Coding	GC05P173144	0.934955955
DYDC1	DPY30 Domain Containing 1	Protein Coding	GC10M080336	0.934955955
COMMD7	COMM Domain Containing 7	Protein Coding	GC20M032702	0.934955955
CELF3	CUGBP Elav-Like Family Member 3	Protein Coding	GC01M151674	0.934955955
DALRD3	DALR Anticodon Binding Domain Containing 3	Protein Coding	GC03M049015	0.934955955
FYB1	FYN Binding Protein 1	Protein Coding	GC05M039105	0.934955955
IRF2BP1	Interferon Regulatory Factor 2 Binding Protein 1	Protein Coding	GC19M048758	0.934955955
GPR107	G Protein-Coupled Receptor 107	Protein Coding	GC09P130053	0.934955955
GRAMD1B	GRAM Domain Containing 1B	Protein Coding	GC11P123358	0.934955955
PPP1R11	Protein Phosphatase 1 Regulatory Inhibitor Subunit 11	Protein Coding	GC06P058255	0.934955955
MED16	Mediator Complex Subunit 16	Protein Coding	GC19M001123	0.934955955
MACROH2A	MacroH2A.1 Histone	Protein Coding	GC05M135334	0.934955955
RNPEPL1	Arginyl Aminopeptidase Like 1	Protein Coding	GC02P240565	0.934955955
TRIM10	Tripartite Motif Containing 10	Protein Coding	GC06M030151	0.934955955
TRIM40	Tripartite Motif Containing 40	Protein Coding	GC06P058271	0.934955955
RNF123	Ring Finger Protein 123	Protein Coding	GC03P049689	0.934955955
RNF145	Ring Finger Protein 145	Protein Coding	GC05M159157	0.934955955
UBLCP1	Ubiquitin Like Domain Containing CTD Phosphatase 1	Protein Coding	GC05P159263	0.934955955
UBTD2	Ubiquitin Domain Containing 2	Protein Coding	GC05M172209	0.934955955
QRICH1	Glutamine Rich 1	Protein Coding	GC03M049513	0.934955955
RBM4B	RNA Binding Motif Protein 4B	Protein Coding	GC11M066664	0.934955955

SPSB2	SplA/Ryanodine Receptor Domain And SOCS Box Containing 2	Protein Coding	GC12M006851	0.934955955
ABHD16A	Abhydrolase Domain Containing 16A, Phospholipase	Protein Coding	GC06M049122	0.934955955
AMZ1	Archaelysin Family Metallopeptidase 1	Protein Coding	GC07P002679	0.934955955
ASB6	Ankyrin Repeat And SOCS Box Containing 6	Protein Coding	GC09M129639	0.934955955
ASB8	Ankyrin Repeat And SOCS Box Containing 8	Protein Coding	GC12M048147	0.934955955
CDC42SE2	CDC42 Small Effector 2	Protein Coding	GC05P131245	0.934955955
ACRBP	Acrosin Binding Protein	Protein Coding	GC12M006638	0.934955955
BBS12	Bardet-Biedl Syndrome 12	Protein Coding	GC04P122702	0.934955955
EXD1	Exonuclease 3'-5' Domain Containing 1	Protein Coding	GC15M041182	0.934955955
ENDOU	Endonuclease, Poly(U) Specific	Protein Coding	GC12M047709	0.934955955
FBXO24	F-Box Protein 24	Protein Coding	GC07P100583	0.934955955
FUT11	Fucosyltransferase 11	Protein Coding	GC10P073772	0.934955955
GAL3ST2	Galactose-3-O-Sulfotransferase 2	Protein Coding	GC02P241777	0.934955955
FNDC3A	Fibronectin Type III Domain Containing 3A	Protein Coding	GC13P048975	0.934955955
CMC1	C-X9-C Motif Containing 1	Protein Coding	GC03P028249	0.934955955
GIGYF1	GRB10 Interacting GYF Protein 1	Protein Coding	GC07M100679	0.934955955
ZWILCH	Zwilch Kinetochore Protein	Protein Coding	GC15P066504	0.934955955
FCAMR	Fc Fragment Of IgA And IgM Receptor	Protein Coding	GC01M206957	0.934955955
MXRA8	Matrix Remodeling Associated 8	Protein Coding	GC01M001352	0.934955955
MYRF	Myelin Regulatory Factor	Protein Coding	GC11P061753	0.934955955
MSL1	MSL Complex Subunit 1	Protein Coding	GC17P040122	0.934955955
NGRN	Neugrin, Neurite Outgrowth Associated	Protein Coding	GC15P090265	0.934955955
NKD1	NKD Inhibitor Of WNT Signaling Pathway 1	Protein Coding	GC16P050548	0.934955955
PRM2	Protamine 2	Protein Coding	GC16M011324	0.934955955
PILRB	Paired Immunoglobin Like Type 2 Receptor Beta	Protein Coding	GC07P100353	0.934955955
PNRC2	Proline Rich Nuclear Receptor Coactivator 2	Protein Coding	GC01P024086	0.934955955
PPP1R18	Protein Phosphatase 1 Regulatory Subunit 18	Protein Coding	GC06M049035	0.934955955
ITLN2	Intelectin 2	Protein Coding	GC01M160945	0.934955955
OLFML2B	Olfactomedin Like 2B	Protein Coding	GC01M161984	0.934955955
MRPL9	Mitochondrial Ribosomal Protein L9	Protein Coding	GC01M151759	0.934955955
LSM14A	LSM14A MRNA Processing Body Assembly Factor	Protein Coding	GC19P034172	0.934955955
LY6G6C	Lymphocyte Antigen 6 Family Member G6C	Protein Coding	GC06M049125	0.934955955
PLEKHG6	Pleckstrin Homology And RhoGEF Domain Containing G6	Protein Coding	GC12P006310	0.934955955
SBK1	SH3 Domain Binding Kinase 1	Protein Coding	GC16P028260	0.934955955
SDF2L1	Stromal Cell Derived Factor 2 Like 1	Protein Coding	GC22P027270	0.934955955
OIP5	Opa Interacting Protein 5	Protein Coding	GC15M041309	0.934955955
TCHHL1	Trichohyalin Like 1	Protein Coding	GC01M152085	0.934955955
TBC1D8	TBC1 Domain Family Member 8	Protein Coding	GC02M101007	0.934955955
TRIM15	Tripartite Motif Containing 15	Protein Coding	GC06P058272	0.934955955
TMEM63B	Transmembrane Protein 63B	Protein Coding	GC06P058527	0.934955955
SGIP1	SH3GL Interacting Endocytic Adaptor	Protein Coding	GC01P066533	0.934955955
QPCTL	Glutaminyl-Peptide Cyclotransferase	Protein Coding	GC19P045692	0.934955955
OR2H2	Olfactory Receptor Family 2 Subfamily H Member 2	Protein Coding	GC06P058231	0.934955955
RAVER1	Ribonucleoprotein, PTB Binding 1	Protein Coding	GC19M010316	0.934955955
TNP2	Transition Protein 2	Protein Coding	GC16M011267	0.934955955
WDR43	WD Repeat Domain 43	Protein Coding	GC02P028894	0.934955955
YIF1A	Yip1 Interacting Factor Homolog A, Membrane Trafficking Protein	Protein Coding	GC11M071756	0.934955955
ZNF366	Zinc Finger Protein 366	Protein Coding	GC05M072442	0.934955955
ZC3H12C	Zinc Finger CCCH-Type Containing	Protein Coding	GC11P110092	0.934955955

ZFPL1	Zinc Finger Protein Like 1	Protein Coding	GC11P065084	0.934955955
ZNF507	Zinc Finger Protein 507	Protein Coding	GC19P032345	0.934955955
ZNF142	Zinc Finger Protein 142	Protein Coding	GC02M218637	0.934955955
C11orf68	Chromosome 11 Open Reading Frame	Protein Coding	GC11M065916	0.934955955
CARS1	Cysteinyl-TRNA Synthetase 1	Protein Coding	GC11M003000	0.934955955
CYTL1	Cytokine Like 1	Protein Coding	GC04M005016	0.934955955
DYDC2	DPY30 Domain Containing 2	Protein Coding	GC10P080344	0.934955955
EVX1	Even-Skipped Homeobox 1	Protein Coding	GC07P027419	0.934955955
FAM53B	Family With Sequence Similarity 53 Member B	Protein Coding	GC10M124619	0.934955955
GMEB2	Glucocorticoid Modulatory Element Binding Protein 2	Protein Coding	GC20M063587	0.934955955
IFFO1	Intermediate Filament Family Orphan 1	Protein Coding	GC12M006538	0.934955955
DNAJC28	DnaJ Heat Shock Protein Family (Hsp40) Member C28	Protein Coding	GC21M033485	0.934955955
GID8	GID Complex Subunit 8 Homolog	Protein Coding	GC20P062938	0.934955955
EIF1AD	Eukaryotic Translation Initiation Factor 1A Domain Containing	Protein Coding	GC11M065996	0.934955955
KANSL2	KAT8 Regulatory NSL Complex Subunit 2	Protein Coding	GC12M048653	0.934955955
FAM118A	Family With Sequence Similarity 118 Member A	Protein Coding	GC22P045308	0.934955955
MXD3	MAX Dimerization Protein 3	Protein Coding	GC05M177533	0.934955955
OAZ3	Ornithine Decarboxylase Antizyme 3	Protein Coding	GC01P151762	0.934955955
LIX1	Limb And CNS Expressed 1	Protein Coding	GC05M097091	0.934955955
MFSD9	Major Facilitator Superfamily Domain Containing 9	Protein Coding	GC02M102700	0.934955955
MIEN1	Migration And Invasion Enhancer 1	Protein Coding	GC17M039728	0.934955955
MIER1	MIER1 Transcriptional Regulator	Protein Coding	GC01P066924	0.934955955
NACC2	NACC Family Member 2	Protein Coding	GC09M136006	0.934955955
NUDT13	Nudix Hydrolase 13	Protein Coding	GC10P073110	0.934955955
RPP25L	Ribonuclease P/MRP Subunit P25 Like	Protein Coding	GC09M034610	0.934955955
SPATA2	Spermatogenesis Associated 2	Protein Coding	GC20M049903	0.934955955
TSSC4	Tumor Suppressing Subtransferable Candidate 4	Protein Coding	GC11P002437	0.934955955
TCTA	T Cell Leukemia Translocation Altered	Protein Coding	GC03P049412	0.934955955
TRIM4	Tripartite Motif Containing 4	Protein Coding	GC07M099876	0.934955955
TMCO4	Transmembrane And Coiled-Coil Domains 4	Protein Coding	GC01M019682	0.934955955
THAP7	THAP Domain Containing 7	Protein Coding	GC22M020999	0.934955955
UQCC1	Ubiquinol-Cytochrome C Reductase Complex Assembly Factor 1	Protein Coding	GC20M035302	0.934955955
RIC8B	RIC8 Guanine Nucleotide Exchange Factor B	Protein Coding	GC12P106774	0.934955955
RSBN1	Round Spermatid Basic Protein 1	Protein Coding	GC01M113761	0.934955955
RPAP3	RNA Polymerase II Associated Protein	Protein Coding	GC12M047661	0.934955955
SKOR1	SKI Family Transcriptional Corepressor 1	Protein Coding	GC15P067819	0.934955955
TMEM106C	Transmembrane Protein 106C	Protein Coding	GC12P047963	0.934955955
TTPAL	Alpha Tocopherol Transfer Protein	Protein Coding	GC20P044476	0.934955955
ZC3H4	Zinc Finger CCCH-Type Containing 4	Protein Coding	GC19M047064	0.934955955
ZCCHC10	Zinc Finger CCHC-Type Containing 10	Protein Coding	GC05M132996	0.934955955
C6orf15	Chromosome 6 Open Reading Frame	Protein Coding	GC06M031111	0.934955955
C9orf78	Chromosome 9 Open Reading Frame	Protein Coding	GC09M129827	0.934955955
EMSY	EMSY Transcriptional Repressor, BRCA2 Interacting	Protein Coding	GC11P076482	0.934955955
FRMD8	FERM Domain Containing 8	Protein Coding	GC11P065386	0.934955955
IQCH	IQ Motif Containing H	Protein Coding	GC15P067254	0.934955955
LCA5L	Lebercilin LCA5 Like	Protein Coding	GC21M039405	0.934955955
EHBP1L1	EH Domain Binding Protein 1 Like 1	Protein Coding	GC11P066825	0.934955955
ZPLD1	Zona Pellucida Like Domain	Protein Coding	GC03P102099	0.934955955
OR10AD1	Olfactory Receptor Family 10 Subfamily AD Member 1	Protein Coding	GC12M048201	0.934955955

LY6G6D	Lymphocyte Antigen 6 Family Member G6D	Protein Coding	GC06P031715	0.934955955
MIER2	MIER Family Member 2	Protein Coding	GC19M000305	0.934955955
WHRN	Whirlin	Protein Coding	GC09M114403	0.934955955
BPIFB3	BPI Fold Containing Family B Member	Protein Coding	GC20P033059	0.934955955
C5orf24	Chromosome 5 Open Reading Frame	Protein Coding	GC05P134845	0.934955955
BPIFA2	BPI Fold Containing Family A Member	Protein Coding	GC20P033161	0.934955955
BOLA2	BolA Family Member 2	Protein Coding	GC16M031590	0.934955955
BPIFB4	BPI Fold Containing Family B Member	Protein Coding	GC20P033079	0.934955955
CCDC61	Coiled-Coil Domain Containing 61	Protein Coding	GC19P045995	0.934955955
CCDC51	Coiled-Coil Domain Containing 51	Protein Coding	GC03M048432	0.934955955
CCDC82	Coiled-Coil Domain Containing 82	Protein Coding	GC11M096352	0.934955955
CNEP1R1	CTD Nuclear Envelope Phosphatase 1 Regulatory Subunit 1	Protein Coding	GC16P050024	0.934955955
AP5B1	Adaptor Related Protein Complex 5 Subunit Beta 1	Protein Coding	GC11M065773	0.934955955
CREBRF	CREB3 Regulatory Factor	Protein Coding	GC05P173056	0.934955955
GPATCH2L	G-Patch Domain Containing 2 Like	Protein Coding	GC14P076151	0.934955955
GPATCH1	G-Patch Domain Containing 1	Protein Coding	GC19P033080	0.934955955
CFAP70	Cilia And Flagella Associated Protein	Protein Coding	GC10M073253	0.934955955
FAXDC2	Fatty Acid Hydroxylase Domain Containing 2	Protein Coding	GC05M154817	0.934955955
MUSTN1	Musculoskeletal, Embryonic Nuclear Protein 1	Protein Coding	GC03M052834	0.934955955
LURAP1L	Leucine Rich Adaptor Protein 1 Like	Protein Coding	GC09P012775	0.934955955
LYZL2	Lysozyme Like 2	Protein Coding	GC10M031337	0.934955955
PTRHD1	Peptidyl-TRNA Hydrolase Domain Containing 1	Protein Coding	GC02M024790	0.934955955
SUPT7L	SPT7 Like, STAGA Complex Subunit Gamma	Protein Coding	GC02M027750	0.934955955
TMEM259	Transmembrane Protein 259	Protein Coding	GC19M001016	0.934955955
SLC45A1	Solute Carrier Family 45 Member 1	Protein Coding	GC01P008317	0.934955955
SH2D4B	SH2 Domain Containing 4B	Protein Coding	GC10P086024	0.934955955
SNN	Stannin	Protein Coding	GC16P011669	0.934955955
ZNF512	Zinc Finger Protein 512	Protein Coding	GC02P027582	0.934955955
BPIFA3	BPI Fold Containing Family A Member	Protein Coding	GC20P033217	0.934955955
CCDC71	Coiled-Coil Domain Containing 71	Protein Coding	GC03M049525	0.934955955
CATIP	Ciliogenesis Associated TTC17 Interacting Protein	Protein Coding	GC02P218356	0.934955955
DEXI	Dexi Homolog	Protein Coding	GC16M010928	0.934955955
GRID2IP	Grid2 Interacting Protein	Protein Coding	GC07M006496	0.934955955
CNOT9	CCR4-NOT Transcription Complex Subunit 9	Protein Coding	GC02P218569	0.934955955
HEATR3	HEAT Repeat Containing 3	Protein Coding	GC16P050065	0.934955955
HECTD4	HECT Domain E3 Ubiquitin Protein Ligase 4	Protein Coding	GC12M112160	0.934955955
LINGO4	Leucine Rich Repeat And Ig Domain Containing 4	Protein Coding	GC01M151812	0.934955955
PPP1R35	Protein Phosphatase 1 Regulatory Subunit 35	Protein Coding	GC07M100660	0.934955955
PCP4L1	Purkinje Cell Protein 4 Like 1	Protein Coding	GC01P161258	0.934955955
SEPTIN1	Septin 1	Protein Coding	GC16M030378	0.934955955
VWA7	Von Willebrand Factor A Domain Containing 7	Protein Coding	GC06M049129	0.934955955
PFN3	Profilin 3	Protein Coding	GC05M177400	0.934955955
SEPTIN8	Septin 8	Protein Coding	GC05M132751	0.934955955
ZMAT5	Zinc Finger Matrin-Type 5	Protein Coding	GC22M029730	0.934955955
C3orf62	Chromosome 3 Open Reading Frame	Protein Coding	GC03M049268	0.934955955
C1orf189	Chromosome 1 Open Reading Frame	Protein Coding	GC01M154199	0.934955955
DUSP28	Dual Specificity Phosphatase 28	Protein Coding	GC02P240560	0.934955955
AQP12A	Aquaporin 12A	Protein Coding	GC02P240691	0.934955955
AQP12B	Aquaporin 12B	Protein Coding	GC02M240676	0.934955955

CNOT11	CCR4-NOT Transcription Complex Subunit 11	Protein Coding	GC02P101252	0.934955955
ZNF774	Zinc Finger Protein 774	Protein Coding	GC15P090352	0.934955955
SAP25	Sin3A Associated Protein 25	Protein Coding	GC07M100670	0.934955955
OR5B12	Olfactory Receptor Family 5 Subfamily B Member 12	Protein Coding	GC11M071335	0.934955955
TDRD10	Tudor Domain Containing 10	Protein Coding	GC01P154502	0.934955955
TSGA10IP	Testis Specific 10 Interacting Protein	Protein Coding	GC11P066867	0.934955955
TMEM89	Transmembrane Protein 89	Protein Coding	GC03M048620	0.934955955
RASL11A	RAS Like Family 11 Member A	Protein Coding	GC13P027270	0.934955955
PLEKHN1	Pleckstrin Homology Domain Containing N1	Protein Coding	GC01P002213	0.934955955
ANKRD33B	Ankyrin Repeat Domain 33B	Protein Coding	GC05P010564	0.934955955
ABRAXAS2	Abraxas 2, BRISC Complex Subunit	Protein Coding	GC10P124802	0.934955955
CCDC184	Coiled-Coil Domain Containing 184	Protein Coding	GC12P048183	0.934955955
CPTP	Ceramide-1-Phosphate Transfer Protein	Protein Coding	GC01P002232	0.934955955
DNLZ	DNL-Type Zinc Finger	Protein Coding	GC09M136359	0.934955955
PRM3	Protamine 3	Protein Coding	GC16M011273	0.934955955
NSG2	Neuronal Vesicle Trafficking Associated 2	Protein Coding	GC05P174047	0.934955955
C11orf87	Chromosome 11 Open Reading Frame	Protein Coding	GC11P109421	0.934955955
C17orf67	Chromosome 17 Open Reading Frame	Protein Coding	GC17M056791	0.934955955
EIPR1	EARP Complex And GARP Complex Interacting Protein 1	Protein Coding	GC02M003188	0.934955955
TMA7	Translation Machinery Associated 7 Homolog	Protein Coding	GC03P048440	0.934955955
PRXL2A	Peroxiredoxin Like 2A	Protein Coding	GC10P086642	0.934955955
MFSD4B	Major Facilitator Superfamily Domain Containing 4B	Protein Coding	GC06P111260	0.934955955
OR5B21	Olfactory Receptor Family 5 Subfamily B Member 21	Protein Coding	GC11M071337	0.934955955
C11orf21	Chromosome 11 Open Reading Frame	Protein Coding	GC11M002295	0.934955955
BICRA	BRD4 Interacting Chromatin Remodeling Complex Associated	Protein Coding	GC19P047625	0.934955955
CDHR4	Cadherin Related Family Member 4	Protein Coding	GC03M049790	0.934955955
CBARP	CACN Subunit Beta Associated Regulatory Protein	Protein Coding	GC19M001228	0.934955955
DUSP29	Dual Specificity Phosphatase 29	Protein Coding	GC10M075028	0.934955955
DYNLT5	Dynein Light Chain Tctex-Type Family Member 5	Protein Coding	GC01P066753	0.934955955
CFAP126	Cilia And Flagella Associated Protein 126	Protein Coding	GC01M161364	0.934955955
FAM205A	Family With Sequence Similarity 205 Member A	Protein Coding	GC09M034723	0.934955955
KRTAP5-5	Keratin Associated Protein 5-5	Protein Coding	GC11P001629	0.934955955
KRTAP5-6	Keratin Associated Protein 5-6	Protein Coding	GC11P001718	0.934955955
LCE3A	Late Cornified Envelope 3A	Protein Coding	GC01M152595	0.934955955
EEF1AKMT2	EEF1A Lysine Methyltransferase 2	Protein Coding	GC10M124768	0.934955955
ZUP1	Zinc Finger Containing Ubiquitin Peptidase 1	Protein Coding	GC06M116636	0.934955955
MUCL3	Mucin Like 3	Protein Coding	GC06P059622	0.934955955
LY6G5C	Lymphocyte Antigen 6 Family Member G5C	Protein Coding	GC06M031676	0.934955955
TMEM116	Transmembrane Protein 116	Protein Coding	GC12M111894	0.934955955
TTLL8	Tubulin Tyrosine Ligase Like 8	Protein Coding	GC22M050015	0.934955955
RIMBP3C	RIMS Binding Protein 3C	Protein Coding	GC22M021577	0.934955955
C9orf163	Chromosome 9 Putative Open Reading Frame 163	Protein Coding	GC09P136483	0.934955955
C5orf47	Chromosome 5 Open Reading Frame	Protein Coding	GC05P173973	0.934955955
DNAI4	Dynein Axonemal Intermediate Chain 4	Protein Coding	GC01M066813	0.934955955
LCE3B	Late Cornified Envelope 3B	Protein Coding	GC01P152613	0.934955955
MFSD13A	Major Facilitator Superfamily Domain Containing 13A	Protein Coding	GC10P102462	0.934955955

PCNX3	Pecanex 3	Protein Coding	GC11P066826	0.934955955
OR5B2	Olfactory Receptor Family 5 Subfamily B Member 2	Protein Coding	GC11M071334	0.934955955
LYRM9	LYR Motif Containing 9	Protein Coding	GC17M027878	0.934955955
TRAPPC3L	Trafficking Protein Particle Complex Subunit 3L	Protein Coding	GC06M116494	0.934955955
INAFM1	InaF Motif Containing 1	Protein Coding	GC19P047282	0.934955955
IGF2-AS	IGF2 Antisense RNA	RNA Gene	GC11P002140	0.934955955
LY6G6E	Lymphocyte Antigen 6 Family Member G6E	Pseudogene	GC06M049124	0.934955955
STIMATE	STIM Activating Enhancer	Protein Coding	GC03M052885	0.934955955
TSBP1	Testis Expressed Basic Protein 1	Protein Coding	GC06M032288	0.934955955
C1QTNF12	C1q And TNF Related 12	Protein Coding	GC01M003064	0.934955955
DELEC1	Deleted In Esophageal Cancer 1	RNA Gene	GC09P116631	0.934955955
ARIH2OS	ARIH2 Opposite Strand LncRNA	RNA Gene	GC03M048955	0.934955955
C20orf203	Chromosome 20 Open Reading Frame 203	Protein Coding	GC20M032631	0.934955955
LINC02694	Long Intergenic Non-Protein Coding RNA 2694	RNA Gene	GC15P038625	0.934955955
PI4KAP2	Phosphatidylinositol 4-Kinase Alpha Pseudogene 2	Pseudogene	GC22M021472	0.934955955
TMEM250	Transmembrane Protein 250	Protein Coding	GC09M136138	0.934955955
TNXA	Tenascin XA (Pseudogene)	Pseudogene	GC06M049139	0.934955955
CALHM4	Calcium Homeostasis Modulator Family Member 4	Protein Coding	GC06P116531	0.934955955
MEIKIN	Meiotic Kinetochore Factor	Protein Coding	GC05M131806	0.934955955
C3orf84	Chromosome 3 Open Reading Frame	Protein Coding	GC03M049528	0.934955955
GABARAPL3	GABA Type A Receptor Associated Protein Like 3 Pseudogene	Pseudogene	GC15M090346	0.934955955
SCARNA12	Small Cajal Body-Specific RNA 12	RNA Gene	GC12M007014	0.934955955
ATP6V1G2-DDX39B	ATP6V1G2-DDX39B Readthrough (NMD Candidate)	RNA Gene	GC06M049115	0.934955955
HCG4	HLA Complex Group 4	RNA Gene	GC06M049006	0.934955955
SCARNA10	Small Cajal Body-Specific RNA 10	RNA Gene	GC12P006510	0.934955955
STIMATE-MUSTN1	STIMATE-MUSTN1 Readthrough	Protein Coding	GC03M052880	0.934955955
ACTA2-AS1	ACTA2 Antisense RNA 1	RNA Gene	GC10P088932	0.934955955
FLG-AS1	FLG Antisense RNA 1	RNA Gene	GC01P152168	0.934955955
GVQW3	GVQW Motif Containing 3	Protein Coding	GC11P076456	0.934955955
MIR1260B	MicroRNA 1260b	RNA Gene	GC11P096341	0.934955955
MSH5-SAPCD1	MSH5-SAPCD1 Readthrough (NMD Candidate)	RNA Gene	GC06P058325	0.934955955
SCARNA11	Small Cajal Body-Specific RNA 11	RNA Gene	GC12M006582	0.934955955
TEX41	Testis Expressed 41	RNA Gene	GC02P144667	0.934955955
SNORA21	Small Nucleolar RNA, H/ACA Box 21	RNA Gene	GC17M038852	0.934955955
ZFP91-CNTF	ZFP91-CNTF Readthrough (NMD Candidate)	RNA Gene	GC11P058579	0.934955955
C1RL-AS1	C1RL Antisense RNA 1	RNA Gene	GC12P013207	0.934955955
ADAM1A	ADAM Metallopeptidase Domain 1A (Pseudogene)	Pseudogene	GC12P111899	0.934955955
JMJD1C-AS1	JMJD1C Antisense RNA 1	RNA Gene	GC10P063465	0.934955955
LINC01620	Long Intergenic Non-Protein Coding RNA 1620	RNA Gene	GC20M044453	0.934955955
JAZF1-AS1	JAZF1 Antisense RNA 1	RNA Gene	GC07P028180	0.934955955
PRR33	Proline Rich 33	Protein Coding	GC11M001887	0.934955955
MIR1301	MicroRNA 1301	RNA Gene	GC02M025328	0.934955955
MIR3909	MicroRNA 3909	RNA Gene	GC22P035335	0.934955955
PPT2-EGFL8	PPT2-EGFL8 Readthrough (NMD Candidate)	RNA Gene	GC06P058335	0.934955955
SNORD16	Small Nucleolar RNA, C/D Box 16	RNA Gene	GC15M069026	0.934955955
CATIP-AS1	CATIP Antisense RNA 1	RNA Gene	GC02M218366	0.934955955
HCG17	HLA Complex Group 17	RNA Gene	GC06M049021	0.934955955
ZSWIM8-	ZSWIM8 Antisense RNA 1	RNA Gene	GC10M073799	0.934955955

FAM99B	Family With Sequence Similarity 99 Member B	RNA Gene	GC11M001684	0.934955955
MIR554	MicroRNA 554	RNA Gene	GC01P151545	0.934955955
MIR647	MicroRNA 647	RNA Gene	GC20M063942	0.934955955
LINC00993	Long Intergenic Non-Protein Coding RNA 993	RNA Gene	GC10P037249	0.934955955
TEX48	Testis Expressed 48	Protein Coding	GC09M114669	0.934955955
SLC26A4-	SLC26A4 Antisense RNA 1	RNA Gene	GC07M107653	0.934955955
C20orf181	Chromosome 20 Open Reading Frame 181	Uncategorized	GC20M063844	0.934955955
ARHGEF38-IT1	ARHGEF38 Intronic Transcript 1	RNA Gene	GC04P105561	0.934955955
FAS-AS1	FAS Antisense RNA 1	RNA Gene	GC10M088991	0.934955955
LINC01185	Long Intergenic Non-Protein Coding RNA 1185	RNA Gene	GC02M060825	0.934955955
LINC00484	Long Intergenic Non-Protein Coding RNA 484	RNA Gene	GC09P091457	0.934955955
LOC100268168	Uncharacterized LOC100268168	RNA Gene	GC05M172954	0.934955955
LINC00548	Long Intergenic Non-Protein Coding RNA 548	RNA Gene	GC13M040087	0.934955955
MIR1208	MicroRNA 1208	RNA Gene	GC08P128150	0.934955955
LURAP1L-AS1	LURAP1L Antisense RNA 1	RNA Gene	GC09M012701	0.934955955
SATB1-AS1	SATB1 Antisense RNA 1	RNA Gene	GC03P018445	0.934955955
SEMA3B-AS1	SEMA3B Antisense RNA 1 (Head To Head)	RNA Gene	GC03M050266	0.934955955
SUGT1P3	SUGT1 Pseudogene 3	Pseudogene	GC13M040882	0.934955955
TET2-AS1	TET2 Antisense RNA 1	RNA Gene	GC04M105171	0.934955955
THAP7-AS1	THAP7 Antisense RNA 1	RNA Gene	GC22P021236	0.934955955
SNORD124	Small Nucleolar RNA, C/D Box 124	RNA Gene	GC17M040027	0.934955955
CRTC3-AS1	CRTC3 Antisense RNA 1	RNA Gene	GC15M090622	0.934955955
IFITM4P	Interferon Induced Transmembrane Protein 4 Pseudogene	Pseudogene	GC06M049001	0.934955955
LINC01250	Long Intergenic Non-Protein Coding RNA 1250	RNA Gene	GC02M002899	0.934955955
LINC01430	Long Intergenic Non-Protein Coding RNA 1430	RNA Gene	GC20M044448	0.934955955
LINC01220	Long Intergenic Non-Protein Coding RNA 1220	RNA Gene	GC14P075294	0.934955955
HCG21	HLA Complex Group 21	RNA Gene	GC06M030945	0.934955955
HORMAD2-AS1	HORMAD2 And MTMR3 Antisense RNA 1	RNA Gene	GC22M030008	0.934955955
FLJ31104	Uncharacterized LOC441072	RNA Gene	GC05P055994	0.934955955
FAM205BP	Family With Sequence Similarity 205 Member B, Pseudogene	Pseudogene	GC09M034837	0.934955955
MIR4425	MicroRNA 4425	RNA Gene	GC01P025023	0.934955955
LINC00824	Long Intergenic Non-Protein Coding RNA 824	RNA Gene	GC08M128405	0.934955955
NRAD1	Non-Coding RNA In The Aldehyde Dehydrogenase 1A Pathway	RNA Gene	GC13P043910	0.934955955
LNC-LBCS	LncRNA Bladder And Prostate Cancer Suppressor, HnRNPK Interacting	RNA Gene	GC06M019322	0.934955955
MMP24OS	MMP24 Opposite Strand	Protein Coding	GC20M035202	0.934955955
MIR623	MicroRNA 623	RNA Gene	GC13P099356	0.934955955
OVOL1-AS1	OVOL1 Antisense RNA 1	RNA Gene	GC11M071715	0.934955955
TNFRSF14-AS1	TNFRSF14 Antisense RNA 1	RNA Gene	GC01M003143	0.934955955
RBFADN	RBFA Downstream Neighbor	RNA Gene	GC18P080048	0.934955955
ZBTB46-AS1	ZBTB46 Antisense RNA 1	RNA Gene	GC20P063808	0.934955955
LOC100288123	Uncharacterized LOC100288123	RNA Gene	GC19P001822	0.934955955
LOC285593	Uncharacterized LOC285593	RNA Gene	GC05P173579	0.934955955

DDR1-DT	DDR1 Divergent Transcript	RNA Gene	GC06M049050	0.934955955
ETS1-AS1	ETS1 Antisense RNA 1	RNA Gene	GC11P128527	0.934955955
FAM238C	Family With Sequence Similarity 238 Member C	RNA Gene	GC10M026932	0.934955955
IGLV5-52	Immunoglobulin Lambda Variable 5-52	Protein Coding	GC22P022318	0.934955955
LINC00581	Long Intergenic Non-Protein Coding RNA 581	RNA Gene	GC06M021486	0.934955955
DKFZP434A062	Uncharacterized LOC26102	RNA Gene	GC09M136322	0.934955955
LINC02202	Long Intergenic Non-Protein Coding RNA 2202	RNA Gene	GC05P159101	0.934955955
LINC02800	Long Intergenic Non-Protein Coding RNA 2800	RNA Gene	GC01P024202	0.934955955
PDCL3P4	PDCL3 Pseudogene 4	Pseudogene	GC03P101712	0.934955955
MIR4686	MicroRNA 4686	RNA Gene	GC11P002173	0.934955955
LINC02098	Long Intergenic Non-Protein Coding RNA 2098	RNA Gene	GC11P128210	0.934955955
LINC00892	Long Intergenic Non-Protein Coding RNA 892	RNA Gene	GC0XP136639	0.934955955
LINC01512	Long Intergenic Non-Protein Coding RNA 1512	RNA Gene	GC06P058525	0.934955955
SMG1P5	SMG1 Pseudogene 5	Pseudogene	GC16M030267	0.934955955
UBE2Q1-AS1	UBE2Q1 Antisense RNA 1	RNA Gene	GC01P154553	0.934955955
LOC101929574	Uncharacterized LOC101929574	RNA Gene	GC10M080519	0.934955955
LOC101927533	Uncharacterized LOC101927533	RNA Gene	GC02P065436	0.934955955
ANKRD33B-AS1	ANKRD33B Antisense RNA 1	RNA Gene	GC05M010628	0.934955955
BSN-DT	BSN Divergent Transcript	RNA Gene	GC03M049552	0.934955955
DM1-AS	DM1 Locus Antisense RNA	RNA Gene	GC19P045768	0.934955955
IL6R-AS1	IL6R Antisense RNA 1	RNA Gene	GC01M154446	0.934955955
IRS3P	Insulin Receptor Substrate 3,	Pseudogene	GC07P100570	0.934955955
MIR6090	MicroRNA 6090	RNA Gene	GC11P128522	0.934955955
LINC02570	Long Intergenic Non-Protein Coding RNA 2570	RNA Gene	GC06M049049	0.934955955
LINC01882	Long Intergenic Non-Protein Coding RNA 1882	RNA Gene	GC18M019834	0.934955955
LINC01989	Long Intergenic Non-Protein Coding RNA 1989	RNA Gene	GC17M034157	0.934955955
SLC12A9-	SLC12A9 Antisense RNA 1	RNA Gene	GC07M100829	0.934955955
ENSG00000244255	Novel Complement Component 2 (C2) And Complement Factor B (CFB)	Protein Coding	GC06P031927	0.934955955
CBLL1-AS1	CBLL1 Antisense RNA 1	RNA Gene	GC07M107736	0.934955955
CDC37P1	Cell Division Cycle 37 Pseudogene 1	Pseudogene	GC16M031407	0.934955955
CCND3P1	Cyclin D3 Pseudogene 1	Pseudogene	GC10M030403	0.934955955
ETF1P1	Eukaryotic Translation Termination Factor 1 Pseudogene 1	Pseudogene	GC06P058253	0.934955955
LINC01147	Long Intergenic Non-Protein Coding RNA 1147	RNA Gene	GC14M088019	0.934955955
MIR6771	MicroRNA 6771	RNA Gene	GC16P050292	0.934955955
LINC02889	Long Intergenic Non-Protein Coding RNA 2889	RNA Gene	GC07M017367	0.934955955
LINC01845	Long Intergenic Non-Protein Coding RNA 1845	RNA Gene	GC05M159448	0.934955955
LINC02213	Long Intergenic Non-Protein Coding RNA 2213	RNA Gene	GC05M010504	0.934955955
PCNPP1	PEST Containing Nuclear Protein Pseudogene 1	Pseudogene	GC12M111666	0.934955955
LINC02888	Long Intergenic Non-Protein Coding RNA 2888	RNA Gene	GC07P017378	0.934955955
LINC02421	Long Intergenic Non-Protein Coding RNA 2421	RNA Gene	GC12M067710	0.934955955

LINC02708	Long Intergenic Non-Protein Coding RNA 2708	RNA Gene	GC11P001688	0.934955955
MICB-DT	MICB Divergent Transcript	RNA Gene	GC06M049092	0.934955955
LINC00604	Long Intergenic Non-Protein Coding RNA 604	RNA Gene	GC05P040261	0.934955955
MHENCR	Melanoma Highly Expressed Competing Endogenous LncRNA For MiR-425 And MiR-489	RNA Gene	GC20P063628	0.934955955
LINC02555	Long Intergenic Non-Protein Coding RNA 2555	RNA Gene	GC12M040141	0.934955955
RPL7P32	Ribosomal Protein L7 Pseudogene 32	Pseudogene	GC07P108510	0.934955955
TDRKH-AS1	TDRKH Antisense RNA 1	RNA Gene	GC01P151853	0.934955955
RFX5-AS1	RFX5 Antisense RNA 1	RNA Gene	GC01P151350	0.934955955
SNORA58B	Small Nucleolar RNA, H/ACA Box	RNA Gene	GC01P154261	0.934955955
STAG3L5P	Stromal Antigen 3-Like 5 Pseudogene	Pseudogene	GC07P100337	0.934955955
ZBTB11-AS1	ZBTB11 Antisense RNA 1	RNA Gene	GC03P101676	0.934955955
ENSG00000253508	Novel Transcript	RNA Gene	GC07M027367	0.934955955
ENSG00000229694	Long Intergenic Non-Protein Coding RNA 484	Uncategorized	GC09P091455	0.934955955
TNRC6C-		RNA Gene	GC17M078107	0.934955955
ABHD17AP4	ABHD17A Pseudogene 4	Pseudogene	GC22M020667	0.934955955
EBAG9P1	EBAG9 Pseudogene 1	Pseudogene	GC10M099697	0.934955955
EIF2S2P3	Eukaryotic Translation Initiation Factor 2 Subunit 2 Beta Pseudogene 3	Pseudogene	GC10M092668	0.934955955
IL1R1-AS1	IL1R1 Antisense RNA 1	RNA Gene	GC02M102174	0.934955955
HNRNPCP4	Heterogeneous Nuclear Ribonucleoprotein C Pseudogene 4	Pseudogene	GC16P011242	0.934955955
RNU6-919P	RNA, U6 Small Nuclear 919,	Pseudogene	GC20P049795	0.934955955
LINC02723	Long Intergenic Non-Protein Coding RNA 2723	RNA Gene	GC11M071608	0.934955955
LINC02354	Long Intergenic Non-Protein Coding RNA 2354	RNA Gene	GC12P047828	0.934955955
LINC02863	Long Intergenic Non-Protein Coding RNA 2863	RNA Gene	GC05M132422	0.934955955
PHBP9	Prohibitin Pseudogene 9	Pseudogene	GC10P100248	0.934955955
RPS5P3	RPS5 Pseudogene 3	Pseudogene	GC21M033481	0.934955955
SUMO2P1	SUMO2 Pseudogene 1	Pseudogene	GC06M048989	0.934955955
TAGAP-AS1	TAGAP Antisense RNA 1	RNA Gene	GC06P159843	0.934955955
TRL-CAG1-6	TRNA-Leu (Anticodon CAG) 1-6	RNA Gene	GC01M161878	0.934955955
RNU7-57P	RNA, U7 Small Nuclear 57 Pseudogene	Pseudogene	GC01P154339	0.934955955
RNY4P10	RNY4 Pseudogene 10	Pseudogene	GC06P033199	0.934955955
RPS10P6	Ribosomal Protein S10 Pseudogene 6	Pseudogene	GC01P151557	0.934955955
RNA5SP192	RNA, 5S Ribosomal Pseudogene 192	Pseudogene	GC05P132848	0.934955955
RPS19P3	Ribosomal Protein S19 Pseudogene 3	Pseudogene	GC14M034568	0.934955955
SCYGR8	Small Cysteine And Glycine Repeat Containing 8	Protein Coding	GC02P227746	0.934955955
LOC105371083	Uncharacterized LOC105371083	RNA Gene	GC16M011501	0.934955955
LOC101928372	Uncharacterized LOC101928372	RNA Gene	GC01P160932	0.934955955
ENSG00000270124	Novel Transcript	RNA Gene	GC16M085616	0.934955955
ENSG00000270210	Novel Transcript	RNA Gene	GC02P028425	0.934955955
ENSG00000270640	Novel Transcript, Sense Intronic To FOSL2	RNA Gene	GC02P028396	0.934955955
ENSG00000260257	Novel Transcript	RNA Gene	GC20P032854	0.934955955
ENSG00000261338	Novel Transcript, Overlapping To ARPC2 And GPBAR1	RNA Gene	GC02P218255	0.934955955
ENSG00000267199	Novel Transcript, Antisense SPIRE1	RNA Gene	GC18P014992	0.934955955

ENSG00000199550	Y RNA	RNA Gene	GC11P002372	0.934955955
ENSG00000238290	Novel Transcript	RNA Gene	GC01P008026	0.934955955
ENSG00000247121	Novel Transcript, Antisense To LNPEP And ERAP2	RNA Gene	GC05M096814	0.934955955
ENSG00000248373	Novel Transcript	RNA Gene	GC04P104653	0.934955955
ENSG00000249624	Novel Protein	Protein Coding	GC21P033246	0.934955955
ENSG00000254295	Novel Transcript	RNA Gene	GC05P172954	0.934955955
ENSG00000255038	Novel Transcript, Antisense To SF3B2	RNA Gene	GC11M066067	0.934955955
LOC112267968	Uncharacterized LOC112267968	Protein Coding	GC06M159053	0.934955955
ENSG00000224228	Novel Transcript	RNA Gene	GC01P172681	0.934955955
ENSG00000232937	Novel Transcript	RNA Gene	GC01P151765	0.934955955
ENSG00000234132	Novel Transcript, Antisense CACNA1S	RNA Gene	GC01P201032	0.934955955
ENSG00000234261	Novel Transcript	RNA Gene	GC06M014406	0.934955955
ENSG00000234630	Novel Transcript	RNA Gene	GC22P027295	0.934955955
ENSG00000235888	Novel Transcript	RNA Gene	GC21M038988	0.934955955
ENSG00000250264	Novel Protein, TAP2-HLA-DOB Readthrough	Protein Coding	GC06M049181	0.934955955
ENSG00000254461	Novel Transcript	RNA Gene	GC11M071752	0.934955955
ENSG00000256433	Novel Transcript	RNA Gene	GC12P006393	0.934955955
ENSG00000205414	Novel Transcript, Antisense To NKD1	RNA Gene	GC16M050606	0.934955955
ENSG00000225172	Novel Transcript	RNA Gene	GC01P198983	0.934955955
ENSG00000225421	Novel Transcript	RNA Gene	GC02M198494	0.934955955
ENSG00000227938	Novel Transcript	RNA Gene	GC02M028448	0.934955955
ENSG00000229299	Novel Transcript	RNA Gene	GC20P063744	0.934955955
ENSG00000229990	Novel Transcript, Antisense To CAMK2G	RNA Gene	GC10P073841	0.934955955
ENSG00000230534	Novel Transcript	RNA Gene	GC10M035098	0.934955955
ENSG00000230533	Novel Transcript	RNA Gene	GC06P137657	0.934955955
ENSG00000234255	Novel Transcript	RNA Gene	GC02M065442	0.934955955
ENSG00000236471	Novel Transcript	RNA Gene	GC21P015067	0.934955955
ENSG00000245156	Novel Transcript	RNA Gene	GC11M066269	0.934955955
ENSG00000253736	Novel Transcript, Antisense To DUSP1	RNA Gene	GC05P172762	0.934955955
ENSG00000228778	Novel Transcript	RNA Gene	GC10P099527	0.934955955
ENSG00000228863	Novel Transcript, Antisense To CD48	RNA Gene	GC01P160671	0.934955955

ENSG00000231355	Novel Transcript	RNA Gene	GC21M033482	0.934955955
ENSG00000254855	Novel Transcript	RNA Gene	GC11M066264	0.934955955
ENSG00000255320	Novel Transcript	RNA Gene	GC11M071750	0.934955955
ENSG00000256967	Novel Transcript	RNA Gene	GC12M007129	0.934955955
ENSG00000258559	Novel Transcript, Sense Overlapping ABCD4	RNA Gene	GC14M074290	0.934955955
ENSG00000258740	Novel Transcript	RNA Gene	GC14M075238	0.934955955
ENSG00000263020	Novel Protein	Protein Coding	GC06P058321	0.934955955
ENSG00000266469	Novel Transcript, Antisense To MED1 & FBXL20	RNA Gene	GC17P039401	0.934955955
ENSG00000261367	Novel Transcript, Antisense To GDPD3	RNA Gene	GC16P033409	0.934955955
ENSG00000262151	Novel Transcript, Antisense To CIITA	RNA Gene	GC16M011139	0.934955955
ENSG00000267480	Novel Transcript, Antisense IMPA2	RNA Gene	GC18M019763	0.934955955
CICP4	Capicua Transcriptional Repressor Pseudogene 4	Pseudogene	GC20M064290	0.934955955
CBX3P9	Chromobox 3 Pseudogene 9	Pseudogene	GC06M116453	0.934955955
CCR12P	C-C Motif Chemokine Receptor 12, Pseudogene	Pseudogene	GC13M099407	0.934955955
GLULP4	Glutamate-Ammonia Ligase	Pseudogene	GC09P034917	0.934955955
KRT18P39	Keratin 18 Pseudogene 39	Pseudogene	GC02M203765	0.934955955
RNU1-134P	RNA, U1 Small Nuclear 134,	Pseudogene	GC20P063908	0.934955955
RNU6-344P	RNA, U6 Small Nuclear 344,	Pseudogene	GC06P106304	0.934955955
RNU6-543P	RNA, U6 Small Nuclear 543,	Pseudogene	GC10P063110	0.934955955
RNU6-925P	RNA, U6 Small Nuclear 925,	Pseudogene	GC08M089900	0.934955955
RNU6-474P	RNA, U6 Small Nuclear 474,	Pseudogene	GC02P203782	0.934955955
RNU6-638P	RNA, U6 Small Nuclear 638,	Pseudogene	GC17M078696	0.934955955
RNU6-70P	RNA, U6 Small Nuclear 70,	Pseudogene	GC13M027343	0.934955955
RNU6-850P	RNA, U6 Small Nuclear 850,	Pseudogene	GC06M031756	0.934955955
TRG-TCC2-6	TRNA-Gly (Anticodon TCC) 2-6	RNA Gene	GC01P161531	0.934955955
RN7SL391P	RNA, 7SL, Cytoplasmic 391,	Pseudogene	GC12P006344	0.934955955
XPC-AS1	XPC Antisense RNA 1	RNA Gene	GC03P014150	0.934955955
RPSAP64	Ribosomal Protein SA Pseudogene 64	Pseudogene	GC21P038895	0.934955955
RNA5SP184	RNA, 5S Ribosomal Pseudogene 184	Pseudogene	GC05M056146	0.934955955
SNRPGP7	Small Nuclear Ribonucleoprotein Polypeptide G Pseudogene 7	Pseudogene	GC02M028460	0.934955955
SPTLC1P1	Serine Palmitoyltransferase Long Chain Base Subunit 1 Pseudogene 1	Pseudogene	GC10P031360	0.934955955
ENSG00000269514	Novel Transcript, Antisense To OR10AD1	RNA Gene	GC12P048198	0.934955955
ENSG00000269621	Novel Transcript, Antisense To MRPL9	RNA Gene	GC01P151740	0.934955955
LOC101927745	Uncharacterized LOC101927745	RNA Gene	GC21M015404	0.934955955
ENSG00000271855	Novel Transcript	RNA Gene	GC02P009555	0.934955955
ENSG00000271936	Novel Transcript, Antisense To ADCY3	RNA Gene	GC02P024825	0.934955955
ENSG00000272305	Novel Transcript	Protein Coding	GC03M053003	0.934955955
ENSG00000272109	Novel Transcript, Antisense To ERAP1	RNA Gene	GC05P096804	0.934955955
ENSG00000272791	Novel Transcript	RNA Gene	GC10P073630	0.934955955

ENSG00000260233	ZNRD2 Antisense RNA 1 (Head To Head)	Uncategorized	GC11M071692	0.934955955
ENSG00000260577	Novel Transcript, Antisense To CDH3	RNA Gene	GC16M068645	0.934955955
ENSG00000260651	Novel Transcript, Antisense NFKB1	RNA Gene	GC04M102500	0.934955955
ENSG00000263033	Novel Transcript	RNA Gene	GC16P011196	0.934955955
ENSG00000202533	Y RNA	RNA Gene	GC05P132468	0.934955955
ENSG00000212228		RNA Gene	GC16M011489	0.934955955
ENSG00000237422	Novel Transcript	RNA Gene	GC09M091007	0.934955955
ENSG00000238280	Novel Transcript	RNA Gene	GC10M062682	0.934955955
ENSG00000224645	Novel Transcript	RNA Gene	GC01P151340	0.934955955
ENSG00000234117	Novel Transcript, Antisense To BET3L	RNA Gene	GC06P116492	0.934955955
ENSG00000252840		RNA Gene	GC01M151527	0.934955955
ENSG00000184441	Novel Transcript, Antisense To C21orf2	RNA Gene	GC21P044331	0.934955955
ENSG00000205537	Novel Transcript, Antisense To VDR	RNA Gene	GC12P047883	0.934955955
ENSG00000224478	Novel Transcript	RNA Gene	GC06P159740	0.934955955
ENSG00000230684	Novel Transcript	RNA Gene	GC09P129933	0.934955955
ENSG00000236308	Novel Transcript, Antisense To CHUK	RNA Gene	GC10P100190	0.934955955
ENSG00000253111	Novel Transcript	RNA Gene	GC08P125466	0.934955955
ENSG00000255135	Novel Transcript	RNA Gene	GC11M076441	0.934955955
ENSG00000255966	Novel Transcript	RNA Gene	GC12M006532	0.934955955
ENSG00000259202	Novel Transcript, Antisense To SMAD3	RNA Gene	GC15M067142	0.934955955
ENSG00000226812	Novel Transcript, Antisense To R3HDML	RNA Gene	GC20M044347	0.934955955
ENSG00000248753	Novel Transcript	RNA Gene	GC05P135120	0.934955955
ENSG00000261573	Novel Transcript	RNA Gene	GC01M198657	0.934955955
ENSG00000267317	Novel Transcript, Antisense To APC2	RNA Gene	GC19M002336	0.934955955
ENSG00000268810	Novel Transcript, Antisense To PPP5C	RNA Gene	GC19M046382	0.934955955
ENSG00000268746	Novel Transcript	RNA Gene	GC19P047607	0.934955955
BTF3P2	BTF3 Pseudogene 2	Pseudogene	GC14M028672	0.934955955
AIMP1P2	Aminoacyl TRNA Synthetase Complex Interacting Multifunctional Protein 1 Pseudogene 2	Pseudogene	GC01M172856	0.934955955
CCT5P2	Chaperonin Containing TCP1 Subunit 5 Pseudogene 2	Pseudogene	GC13P078908	0.934955955
FGFR1OP2P1	FGFR1 Oncogene Partner 2	Pseudogene	GC13P026905	0.934955955
LOC105369302	Uncharacterized LOC105369302	RNA Gene	GC21M015371	0.934955955
LINC01958	Long Intergenic Non-Protein Coding RNA 1958	RNA Gene	GC02P156655	0.934955955

LINC02300	Long Intergenic Non-Protein Coding RNA 2300	RNA Gene	GC14M028558	0.934955955
LINC02635	Long Intergenic Non-Protein Coding RNA 2635	RNA Gene	GC10P034974	0.934955955
NPM1P17	Nucleophosmin 1 Pseudogene 17	Pseudogene	GC03P137723	0.934955955
PEBP1P3	Phosphatidylethanolamine Binding Protein 1 Pseudogene 3	Pseudogene	GC01M198678	0.934955955
NIPA2P5	NIPA2 Pseudogene 5	Pseudogene	GC13P079090	0.934955955
RPL35P9	Ribosomal Protein L35 Pseudogene 9	Pseudogene	GC13P106425	0.934955955
RPLP0P7	Ribosomal Protein Lateral Stalk Subunit P0 Pseudogene 7	Pseudogene	GC02M156777	0.934955955
RPS23P10	Ribosomal Protein S23 Pseudogene 10	Pseudogene	GC01M161536	0.934955955
RNU6-299P	RNA, U6 Small Nuclear 299,	Pseudogene	GC05P056125	0.934955955
RNU6-320P	RNA, U6 Small Nuclear 320,	Pseudogene	GC0XP136583	0.934955955
RNU6-351P	RNA, U6 Small Nuclear 351,	Pseudogene	GC04P104974	0.934955955
RNU6-704P	RNA, U6 Small Nuclear 704,	Pseudogene	GC01M200904	0.934955955
RN7SKP211	RN7SK Pseudogene 211	Pseudogene	GC06P105904	0.934955955
RPL21P33	Ribosomal Protein L21 Pseudogene 33	Pseudogene	GC02P060852	0.934955955
RPL23AP12	Ribosomal Protein L23a Pseudogene 12	Pseudogene	GC21P039127	0.934955955
RN7SL51P	RNA, 7SL, Cytoplasmic 51,	Pseudogene	GC02M062262	0.934955955
RPL5P26	Ribosomal Protein L5 Pseudogene 26	Pseudogene	GC10P069778	0.934955955
SNRPGP8	Small Nuclear Ribonucleoprotein Polypeptide G Pseudogene 8	Pseudogene	GC02M227778	0.934955955
YBX1P5	Y-Box Binding Protein 1 Pseudogene 5	Pseudogene	GC05P072417	0.934955955
LOC100507103	Uncharacterized LOC100507103	RNA Gene	GC09M090974	0.934955955
LOC107984360	Uncharacterized LOC107984360	RNA Gene	GC11P076658	0.934955955
ENSG00000273055	Novel Transcript, Antisense To LAMB1	RNA Gene	GC07P107942	0.934955955
ENSG00000286116	FAS Antisense RNA 1	RNA Gene	GC10M088990	0.934955955
ENSG00000272072	Novel Transcript, Antisense To HBP1	RNA Gene	GC07M107192	0.934955955
ENSG00000284633	Novel Transcript, Antisense To PDGFB	RNA Gene	GC22P039242	0.934955955
ENSG00000284829	Novel Transcript	RNA Gene	GC06M049141	0.934955955
ENSG00000260773	Novel Transcript, Antisense To DIS3L	RNA Gene	GC15M066314	0.934955955
ENSG00000200677	Small Nucleolar RNA SNORD18	RNA Gene	GC15M090755	0.934955955
ENSG00000234789	Novel Transcript	RNA Gene	GC09P129640	0.934955955
ENSG00000242798	Novel Transcript	RNA Gene	GC07M100613	0.934955955
ENSG00000247853	Novel Transcript, Antisense To CHD4	RNA Gene	GC12P013158	0.934955955
ENSG00000248734	Novel Transcript	RNA Gene	GC05P096785	0.934955955
ENSG00000258860	Novel Transcript	RNA Gene	GC14M035362	0.934955955
ENSG00000199332	Y RNA	RNA Gene	GC06M049089	0.934955955
ENSG00000199473	Small Nucleolar RNA SNORA63	RNA Gene	GC07M017379	0.934955955
ENSG00000219159	Novel Transcript	Uncategorized	GC02P240687	0.934955955
ENSG00000219410	Novel Transcript	RNA Gene	GC12P013167	0.934955955
ENSG00000226032	Novel Transcript	RNA Gene	GC06M159042	0.934955955

ENSG00000230537	Novel Transcript	RNA Gene	GC09M091105	0.934955955
ENSG00000235434	Novel Transcript	RNA Gene	GC01P019814	0.934955955
ENSG00000253445	Novel Transcript	RNA Gene	GC05M172689	0.934955955
ENSG00000222701	Y RNA	RNA Gene	GC16P030471	0.934955955
ENSG00000237773	Novel Transcript	RNA Gene	GC07M016971	0.934955955
ENSG00000238326		RNA Gene	GC05M056000	0.934955955
ENSG00000240996	Novel Transcript	RNA Gene	GC10M089694	0.934955955
ENSG00000243696	Novel MUSTN1-ITIH4 Readthrough	Protein Coding	GC03M052813	0.934955955
ENSG00000224000	Novel Transcript	RNA Gene	GC01P172876	0.934955955
ENSG00000254810	Novel Transcript	RNA Gene	GC11M076654	0.934955955
ENSG00000259605	Novel Transcript, Antisense To SIX5	RNA Gene	GC19P045764	0.934955955
ENSG00000263756	Novel Transcript, Antisense To BRD2	RNA Gene	GC06M032972	0.934955955
ENSG00000266202	Novel Protein	Protein Coding	GC17M027798	0.934955955
ENSG00000267303	Novel Transcript	Protein Coding	GC19M010315	0.934955955
BTF3L4P3	Basic Transcription Factor 3 Like 4 Pseudogene 3	Pseudogene	GC06M137543	0.934955955
HNRNPA1P41	Heterogeneous Nuclear Ribonucleoprotein A1 Pseudogene 41	Pseudogene	GC09M004946	0.934955955
IDI1P2	IDI1 Pseudogene 2	Pseudogene	GC08M048297	0.934955955
CHCHD2P3	Coiled-Coil-Helix-Coiled-Coil-Helix Domain Containing 2 Pseudogene 3	Pseudogene	GC19P034126	0.934955955
HMGN2P18	High Mobility Group Nucleosomal Binding Domain 2 Pseudogene 18	Pseudogene	GC01P155148	0.934955955
KRT18P56	Keratin 18 Pseudogene 56	Pseudogene	GC05P040067	0.934955955
POLR2LP1	RNA Polymerase II Subunit L Pseudogene 1	Pseudogene	GC06P058295	0.934955955
NXPE2P1	Neurexophilin And PC-Esterase Domain Family Member 2 Pseudogene	Pseudogene	GC11M114512	0.934955955
PIGCP2	Phosphatidylinositol Glycan Anchor Biosynthesis Class C Pseudogene 2	Pseudogene	GC07P107808	0.934955955
RNU6-144P	RNA, U6 Small Nuclear 144,	Pseudogene	GC08P137105	0.934955955
RNU6-222P	RNA, U6 Small Nuclear 222,	Pseudogene	GC19M054868	0.934955955
RN7SKP113	RN7SK Pseudogene 113	Pseudogene	GC04M004962	0.934955955
RN7SKP226	RN7SK Pseudogene 226	Pseudogene	GC08P128220	0.934955955
RNU7-15P	RNA, U7 Small Nuclear 15 Pseudogene	Pseudogene	GC05M132842	0.934955955
RPS29P21	Ribosomal Protein S29 Pseudogene 21	Pseudogene	GC17P059912	0.934955955
LOC100129776	Eukaryotic Translation Initiation Factor 2 Subunit Gamma Pseudogene	Pseudogene	GC01P008189	0.934955955
ENSG00000268069	Novel Transcript, Antisense To HDAC7	RNA Gene	GC12P047784	0.934955955
ENSG00000276496		RNA Gene	GC07P027186	0.934955955
ENSG00000278020		RNA Gene	GC07P027188	0.934955955
ENSG00000283265	Novel Transcript	RNA Gene	GC06P137693	0.934955955
ENSG00000283286	Novel Transcript	RNA Gene	GC05P040415	0.934955955

ENSG00000274737	Novel Transcript, Sense Intronic To HDAC7	RNA Gene	GC12M047817	0.934955955
ENSG00000283321	Novel Protein	Protein Coding	GC07P017299	0.934955955
ENSG00000283573	Novel Transcript	RNA Gene	GC06P058524	0.934955955
ENSG00000288583	Novel Transcript	RNA Gene	GC09P036357	0.934955955
ENSG00000272630	Novel Transcript	RNA Gene	GC10M073098	0.934955955
ENSG00000273961		RNA Gene	GC07P027409	0.934955955
ENSG00000277966		RNA Gene	GC07P027408	0.934955955
ENSG00000278334		RNA Gene	GC07P027185	0.934955955
ENSG00000278592		RNA Gene	GC07P027410	0.934955955
ENSG00000283782	Novel Protein	Protein Coding	GC05P132417	0.934955955
ENSG00000284779	Novel Protein	Protein Coding	GC11M002226	0.934955955
ENSG00000285413	Novel Transcript	RNA Gene	GC21M044167	0.934955955
ENSG00000285446	Novel Protein	Protein Coding	GC06P116399	0.934955955
ENSG00000287771	Novel Transcript	RNA Gene	GC02P102037	0.934955955
ENSG00000262020	Novel Transcript, Antisense To CLEC16A	RNA Gene	GC16M011066	0.934955955
lnc-FOSL2-2		RNA Gene	GC02P028390	0.934955955
lnc-TMED10-5		RNA Gene	GC14M075276	0.934955955
ENSG00000236710	Proteasome (Prosome, Macropain) Subunit, Alpha Type, 1 (PSMA1) Pseudogene	Pseudogene	GC11M003085	0.934955955
ENSG00000252461		RNA Gene	GC16M029345	0.934955955
lnc-GCA-5		RNA Gene	GC02P162247	0.934955955
lnc-IL10-5		RNA Gene	GC01M206769	0.934955955
lnc-SCNN1A-1		RNA Gene	GC12M006333	0.934955955
lnc-TH-1		RNA Gene	GC11M002206	0.934955955
lnc-TNFSF18-1		RNA Gene	GC01M172774	0.934955955
hsa-miR-5096-095		RNA Gene	GC07M028151	0.934955955
lnc-ASIC2-2		RNA Gene	GC17M034219	0.934955955
lnc-HOXA11-1		RNA Gene	GC07M027371	0.934955955
lnc-HOXA13-1		RNA Gene	GC07M027373	0.934955955
lnc-LAMB4-2		RNA Gene	GC07M107948	0.934955955
lnc-MAP3K7-3		RNA Gene	GC06M090088	0.934955955
lnc-PTGIR-2		RNA Gene	GC19M048827	0.934955955
ENSG00000225544	Pseudogene Similar To Part Of Metallophosphoesterase 1 (MPPE1)	Pseudogene	GC22M021885	0.934955955
ENSG00000225931	Novel Transcript	Uncategorized	GC01P002566	0.934955955
ENSG00000237371	Novel Transcript	RNA Gene	GC20P064056	0.934955955

lnc-TMEM50B-3		RNA Gene	GC21M033384	0.934955955
ENSG00000224431	Cytoplasmic Linker Associated Protein 2 (CLASP2) Pseudogene	Pseudogene	GC05P132199	0.934955955
ENSG00000228037	Novel Transcript	Uncategorized	GC01P002581	0.934955955
ENSG00000266728	Novel Protein	Protein Coding	GC17P027623	0.934955955
lnc-DDR1-4		RNA Gene	GC06P059317	0.934955955
ZYXP1	Zyxin Pseudogene 1	Pseudogene	GC08M137425	0.934955955
LOC107984859	Uncharacterized LOC107984859	RNA Gene	GC16P011213	0.934955955
LOC100131264	Prefoldin Subunit 4 Pseudogene	Pseudogene	GC07P002835	0.934955955
LOC100533842	ATP Binding Cassette Subfamily G Member 2 (Junior Blood Group) Pseudogene	Pseudogene	GC02P227819	0.934955955
HSALNG0046732		RNA Gene	GC05P173890	0.934955955
HSALNG0020025		RNA Gene	GC02P162245	0.934955955
HSALNG0027634		RNA Gene	GC03M101843	0.934955955
HSALNG0049103		RNA Gene	GC06M049609	0.934955955
ENSG00000285551	Novel Transcript	RNA Gene	GC10P062520	0.934955955
ENSG00000286974	Novel Transcript, Antisense To TNXB	RNA Gene	GC06P058715	0.934955955
ENSG00000288064	Novel Transcript, Antisense To STAT4	RNA Gene	GC02P191045	0.934955955
HSALNG0000116		RNA Gene	GC01P002351	0.934955955
HSALNG0001400		RNA Gene	GC01P019765	0.934955955
HSALNG0001696		RNA Gene	GC01P024193	0.934955955
HSALNG0007924		RNA Gene	GC01M161280	0.934955955
HSALNG0007949		RNA Gene	GC01M161909	0.934955955
ENSG00000271547	Chromosome 20 Open Reading Frame 27 (C20orf27) Pseudogene	Pseudogene	GC12M048801	0.934955955
ENSG00000286186	Novel Transcript, Antisense To NFATC1	RNA Gene	GC18M079460	0.934955955
ENSG00000287967	Novel Transcript, Antisense To UQCR10	RNA Gene	GC22M029767	0.934955955
ENSG00000288630	Novel Transcript	RNA Gene	GC16P033719	0.934955955
HSALNG0000115		RNA Gene	GC01M003187	0.934955955
ENSG00000272779	BMS1, Ribosome Biogenesis Factor (BSM1) Pseudogene	Pseudogene	GC22P022303	0.934955955
HSALNG0000276		RNA Gene	GC01M003144	0.934955955
HSALNG0007192-001		RNA Gene	GC01P151850	0.934955955
HSALNG0007192-002		RNA Gene	GC01P151851	0.934955955
ENSG00000273112	Novel Transcript, Readthrough Between FCGR2A And FCGR2C Polymormphic Pseudogene	RNA Gene	GC01P161514	0.934955955

ENSG00000275693		RNA Gene	GC10P088992	0.934955955
ENSG00000285616	Novel Transcript	RNA Gene	GC05M040474	0.934955955
ENSG00000285837	Novel Protein	Protein Coding	GC10P062375	0.934955955
ENSG00000286629	Novel Transcript, Antisense To ERN1	RNA Gene	GC17P064067	0.934955955
AB372574		RNA Gene	GC01P151827	0.934955955
LOC105375746	Uncharacterized LOC105375746	RNA Gene	GC08P125522	0.934955955
HSALNG0084963		RNA Gene	GC11P066859	0.934955955
HSALNG0122988		RNA Gene	GC19P001186	0.934955955
lnc-ANKRD33B-		RNA Gene	GC05P010698	0.934955955
lnc-ARFRP1-		RNA Gene	GC20M063695	0.934955955
lnc-C1QTNF1-9		RNA Gene	GC17P078758	0.934955955
lnc-CLEC16A-4		RNA Gene	GC16P011212	0.934955955
lnc-HDAC11-4		RNA Gene	GC03P013452	0.934955955
lnc-HLA-DQA1-9		RNA Gene	GC06P059361	0.934955955
lnc-IL19-2		RNA Gene	GC01P206766	0.934955955
lnc-IL31RA-5		RNA Gene	GC05P056122	0.934955955
lnc-IL6ST-2		RNA Gene	GC05M056111	0.934955955
lnc-IRF1-1		RNA Gene	GC05M132473	0.934955955
lnc-IRF1-7		RNA Gene	GC05M132407	0.934955955
lnc-ITGB1BP1-3		RNA Gene	GC02M009254	0.934955955
lnc-MIEN1-1		RNA Gene	GC17M039760	0.934955955
lnc-MMEL1-1		RNA Gene	GC01M003146	0.934955955
lnc-MTPAP-7		RNA Gene	GC10M030433	0.934955955
lnc-NBN-9		RNA Gene	GC08M089835	0.934955955
lnc-NFKBIZ-		RNA Gene	GC03P101863	0.934955955
lnc-NFKBIZ-		RNA Gene	GC03P101864	0.934955955
lnc-PWP2-2		RNA Gene	GC21P044202	0.934955955
lnc-RAB5B-2		RNA Gene	GC12P055998	0.934955955
lnc-RIPK2-3		RNA Gene	GC08P089837	0.934955955
lnc-SEH1L-5		RNA Gene	GC18P014994	0.934955955
lnc-SNX13-5		RNA Gene	GC07M017341	0.934955955
lnc-STMN3-5		RNA Gene	GC20M063699	0.934955955
lnc-TMED10-4		RNA Gene	GC14M075273	0.934955955
lnc-TNFRSF14-3		RNA Gene	GC01P002569	0.934955955
lnc-TNNI2-2		RNA Gene	GC11P001859	0.934955955
lnc-TRRAP-5		RNA Gene	GC07P099144	0.934955955
lnc-WASHC5-9		RNA Gene	GC08M129340	0.934955955
lnc-ZCCHC24-7		RNA Gene	GC10M079298	0.934955955
MN298114-220		RNA Gene	GC07P002668	0.934955955
lnc-DUSP1-7		RNA Gene	GC05M172887	0.934955955
lnc-ETS1-8		RNA Gene	GC11M128301	0.934955955
lnc-IGF2-4		RNA Gene	GC11M002229	0.934955955
lnc-LIPG-7		RNA Gene	GC18P048862	0.934955955
lnc-MST1-1		RNA Gene	GC03M049685	0.934955955
lnc-NDFIP1-1		RNA Gene	GC05P144229	0.934955955

lnc-OLIG3-1	RNA Gene	GC06M137671	0.934955955
lnc-PSORS1C2-1	RNA Gene	GC06M049063	0.934955955
lnc-RTN4IP1-6	RNA Gene	GC06M106085	0.934955955
lnc-SCGB2B2-	RNA Gene	GC19M034168	0.934955955
lnc-SCNN1A-2	RNA Gene	GC12M006330	0.934955955
lnc-TMEM258-1	RNA Gene	GC11M071452	0.934955955
lnc-TNFRSF14-1	RNA Gene	GC01P002553	0.934955955
lnc-TNFRSF1A-1	RNA Gene	GC12M006348	0.934955955
lnc-TNFSF18-3	RNA Gene	GC01M172885	0.934955955
lnc-TRAPPC3L-2	RNA Gene	GC06M116454	0.934955955
lnc-ZC3H12C-5	RNA Gene	GC11P110022	0.934955955
MK280269-012	RNA Gene	GC11M076581	0.934955955
HSALNG0082198	RNA Gene	GC11P002149	0.934955955
HSALNG0082195	RNA Gene	GC11P002131	0.934955955
HSALNG0084964	RNA Gene	GC11M072352	0.934955955
HSALNG0084965	RNA Gene	GC11M072353	0.934955955
HSALNG0086605	RNA Gene	GC11P096272	0.934955955
HSALNG0088101	RNA Gene	GC11P128480	0.934955955
HSALNG0088102	RNA Gene	GC11M128489	0.934955955
HSALNG0092134	RNA Gene	GC12M067850	0.934955955
HSALNG0102509	RNA Gene	GC14P075279	0.934955955
HSALNG0102510	RNA Gene	GC14M075280	0.934955955
HSALNG0132765	RNA Gene	GC21M033400	0.934955955
lnc-AHR-4	RNA Gene	GC07P017341	0.934955955
lnc-C9orf78-2	RNA Gene	GC09M129895	0.934955955
lnc-CEP76-3	RNA Gene	GC18M019843	0.934955955
lnc-CREB5-4	RNA Gene	GC07P028146	0.934955955
lnc-FAM109A-1	RNA Gene	GC12M111488	0.934955955
lnc-GRB7-1	RNA Gene	GC17P039760	0.934955955
lnc-HLA-A-2	RNA Gene	GC06P059352	0.934955955
lnc-HLA-DRB1-7	RNA Gene	GC06M032609	0.934955955
lnc-HOXA13-3	RNA Gene	GC07M027374	0.934955955
lnc-MMEL1-2	RNA Gene	GC01M002581	0.934955955
lnc-MRPL23-	RNA Gene	GC11P002138	0.934955955
lnc-PDGFB-3	RNA Gene	GC22M050182	0.934955955
lnc-PTGER4-	RNA Gene	GC05P040391	0.934955955
lnc-PTGIR-1	RNA Gene	GC19M048828	0.934955955
lnc-TM9SF2-	RNA Gene	GC13P099261	0.934955955

lnc-USP25-6		RNA Gene	GC21P015434	0.934955955
HSALNG0088845		RNA Gene	GC12P013558	0.934955955
HSALNG0088846		RNA Gene	GC12P013559	0.934955955
HSALNG0094037		RNA Gene	GC12P111432	0.934955955
HSALNG0103019		RNA Gene	GC14M088005	0.934955955
HSALNG0115857-002		RNA Gene	GC17M034278	0.934955955
HSALNG0120135		RNA Gene	GC18M019792	0.934955955
HSALNG0120134		RNA Gene	GC18P015132	0.934955955
HSALNG0122987		RNA Gene	GC19P001184	0.934955955
L13712-019		RNA Gene	GC20P032744	0.934955955
lnc-ARHGAP20-11		RNA Gene	GC11M110068	0.934955955
HSALNG0017399		RNA Gene	GC02P102064	0.934955955
HSALNG0022244		RNA Gene	GC02M218275	0.934955955
HSALNG0023077		RNA Gene	GC02P233219	0.934955955
HSALNG0046921		RNA Gene	GC05P177362	0.934955955
HSALNG0077026		RNA Gene	GC10M031387	0.934955955
HSALNG0077027		RNA Gene	GC10P030437	0.934955955
HSALNG0013864-002		RNA Gene	GC02M028386	0.934955955
HSALNG0013864-001		RNA Gene	GC02M028385	0.934955955
HSALNG0052454		RNA Gene	GC06P106087	0.934955955
ENSG00000213386	Family With Sequence Similarity 58, Member A (FAM58A) Pseudogene	Pseudogene	GC05M172766	0.934955955
ENSG00000224988	60S Ribosomal Protein L34 (RPL34) Pseudogene	Pseudogene	GC09M129883	0.934955955
ENSG00000253683	Coiled-Coil Domain Containing 72 (CCDC72) Pseudogene	Pseudogene	GC05M172684	0.934955955
ENSG00000232499	Heterogeneous Nuclear Ribonucleoprotein A3 (HnRNPA3) Pseudogene	Pseudogene	GC01P113450	0.934955955
ENSG00000240399	Ribosomal Protein L37 (RPL37) Pseudogene	Pseudogene	GC12M048055	0.934955955
piR-43104-029		RNA Gene	GC19P001183	0.934955955
piR-52079-043		RNA Gene	GC12P047811	0.934955955
RF00017-		RNA Gene	GC16P068598	0.934955955
RF00017-		RNA Gene	GC19M001122	0.934955955
HSALNG0087041		RNA Gene	GC11M110123	0.934955955
HSALNG0088100		RNA Gene	GC11M128462	0.934955955
HSALNG0088844		RNA Gene	GC12P013557	0.934955955

HSALNG0135137	RNA Gene	GC22P035300	0.934955955
lnc-ANKRD33B-	RNA Gene	GC05P010693	0.934955955
lnc-ATP6V1G3-5	RNA Gene	GC01M198672	0.934955955
lnc-ATP6V1G3-6	RNA Gene	GC01M198680	0.934955955
lnc-CALM3-3	RNA Gene	GC19P046626	0.934955955
piR-39701-054	RNA Gene	GC06M090275	0.934955955
piR-44878-042	RNA Gene	GC07M100830	0.934955955
piR-48950-118	RNA Gene	GC03P046392	0.934955955
piR-55650-032	RNA Gene	GC10M006056	0.934955955
RF00017-	RNA Gene	GC02M102159	0.934955955
RF00017-306	RNA Gene	GC01M151828	0.934955955
RF00017-	RNA Gene	GC20P032805	0.934955955
lnc-DSE-1	RNA Gene	GC06P116446	0.934955955
lnc-FCGR3A-2	RNA Gene	GC01M161894	0.934955955
lnc-FCGR3A-4	RNA Gene	GC01M161899	0.934955955
lnc-FNBP1-2	RNA Gene	GC09M129884	0.934955955
lnc-IKZF1-5	RNA Gene	GC07P050289	0.934955955
lnc-LAMB4-3	RNA Gene	GC07M107935	0.934955955
lnc-NEK7-4	RNA Gene	GC01P198678	0.934955955
lnc-OR8D4-1	RNA Gene	GC11P123455	0.934955955
lnc-PLEKHG6-4	RNA Gene	GC12P013130	0.934955955
lnc-PSMA6-6	RNA Gene	GC14P035366	0.934955955
lnc-RTTN-6	RNA Gene	GC18M069930	0.934955955
lnc-SBNO2-2	RNA Gene	GC19M001176	0.934955955
lnc-SCGB2B2-4	RNA Gene	GC19M033978	0.934955955
lnc-SLC34A1-3	RNA Gene	GC05P177360	0.934955955
lnc-TMEM268-4	RNA Gene	GC09P114783	0.934955955
lnc-USP20-5	RNA Gene	GC09P129883	0.934955955
lnc-USP36-4	RNA Gene	GC17M078675	0.934955955
MK280466	RNA Gene	GC05M096918	0.934955955
piR-48759-287	RNA Gene	GC07P099216	0.934955955
piR-51137-090	RNA Gene	GC04M105200	0.934955955
piR-53431-298	RNA Gene	GC18P015291	0.934955955
RF00017-	RNA Gene	GC14M075279	0.934955955
piR-37824	RNA Gene	GC02M102141	0.934955955
piR-57133-098	RNA Gene	GC12P014071	0.934955955
piR-59241	RNA Gene	GC15P080468	0.934955955
RF00017-	RNA Gene	GC12M111250	0.934955955
RF00017-	RNA Gene	GC19M033265	0.934955955
MN309183	RNA Gene	GC10M073914	0.934955955
OA985550	RNA Gene	GC01M161915	0.934955955
NONHSAG003874.2	RNA Gene	GC01P198639	0.934955955
NONHSAG041785.2	RNA Gene	GC05P144271	0.934955955

NONHSAG043568.2		RNA Gene	GC06P059645	0.934955955
MN309431		RNA Gene	GC12P013757	0.934955955
MN830915		RNA Gene	GC17P034219	0.934955955
NONHSAG001750.2		RNA Gene	GC01P067182	0.934955955
NONHSAG026080.2		RNA Gene	GC19P046347	0.934955955
NONHSAG045707.2		RNA Gene	GC06M050115	0.934955955
piR-33458		RNA Gene	GC15P079885	0.934955955
ENSG00000227758	HLA Complex Group 9 Pseudogene 5	Pseudogene	GC06P058235	0.934955955
ENSG00000254755	Nucleolin (NCL) Pseudogene	Pseudogene	GC11P076591	0.934955955
ENSG00000262488	Rho GTPase Activating Protein 21 (ARHGAP21) Pseudogene	Pseudogene	GC16M011140	0.934955955
HSALNG0078328		RNA Gene	GC10M062510	0.934955955
HSALNG0078890		RNA Gene	GC10M073917	0.934955955
HSALNG0079213		RNA Gene	GC10M080599	0.934955955
lnc-CTNND2-10		RNA Gene	GC05M010704	0.934955955
lnc-CTIF-9		RNA Gene	GC18P048873	0.934955955
MN309174-057		RNA Gene	GC10P086229	0.934955955
RF00017-		RNA Gene	GC05P055959	0.934955955
RF00017-		RNA Gene	GC05P144323	0.934955955
RF00017-		RNA Gene	GC05M173966	0.934955955
RF00017-		RNA Gene	GC06M050868	0.934955955
RF00017-		RNA Gene	GC06M050854	0.934955955
RF00017-		RNA Gene	GC06M105986	0.934955955
RF00017-		RNA Gene	GC07M107580	0.934955955
RF00483		RNA Gene	GC11M002234	0.934955955
RF00017-		RNA Gene	GC06M050863	0.934955955
RF00017-		RNA Gene	GC07M099128	0.934955955
RF00017-		RNA Gene	GC07M100711	0.934955955
CHORDC1P5	CHORDC1 Pseudogene 5	Pseudogene	GC01P070536	0.934955955
LOC102723798	Uncharacterized LOC102723798	RNA Gene	GC19P001163	0.934955955
LOC107984500	Uncharacterized LOC107984500	RNA Gene	GC12P013701	0.934955955
LOC107984526	Uncharacterized LOC107984526	RNA Gene	GC12M067849	0.934955955
LOC107984669	Uncharacterized LOC107984669	RNA Gene	GC14M088007	0.934955955
LOC105371080	Uncharacterized LOC105371080	RNA Gene	GC16M011138	0.934955955
LOC105373724	Uncharacterized LOC105373724	RNA Gene	GC02P162246	0.934955955
HSALNG0027636		RNA Gene	GC03M101857	0.934955955
HSALNG0027637		RNA Gene	GC03P101865	0.934955955
HSALNG0044839		RNA Gene	GC05P132450	0.934955955
HSALNG0060048		RNA Gene	GC07P100827	0.934955955
HSALNG0066832		RNA Gene	GC08M089827	0.934955955

HSALNG0073924		RNA Gene	GC09P114784	0.934955955
HSALNG0075913		RNA Gene	GC10P006045	0.934955955
HSALNG0021408		RNA Gene	GC02M198495	0.934955955
HSALNG0023605		RNA Gene	GC02M240616	0.934955955
HSALNG0023609		RNA Gene	GC02M240647	0.934955955
HSALNG0043662		RNA Gene	GC05P096846	0.934955955
HSALNG0049423		RNA Gene	GC06P058926	0.934955955
HSALNG0049424		RNA Gene	GC06M049684	0.934955955
HSALNG0053003		RNA Gene	GC06M116438	0.934955955
HSALNG0068535		RNA Gene	GC08M128542	0.934955955
ENSG00000285560	Novel Transcript, Antisense To CRTC3	RNA Gene	GC15M090631	0.934955955
HSALNG0000283		RNA Gene	GC01M003148	0.934955955
HSALNG0000284		RNA Gene	GC01P002588	0.934955955
HSALNG0008492		RNA Gene	GC01P172848	0.934955955
HQ292134		RNA Gene	GC01P161533	0.934955955
HSALNG0000278		RNA Gene	GC01M003145	0.934955955
5EW4_A-041		RNA Gene	GC05M177546	0.934955955
HG983680		RNA Gene	GC01P161532	0.934955955
HSALNG0007401		RNA Gene	GC01M154451	0.934955955
ENSG00000285552	Novel Transcript	RNA Gene	GC05P040463	0.934955955
HSALNG0001405		RNA Gene	GC01P019861	0.934955955
HSALNG0080050		RNA Gene	GC10M099531	0.934955955
HSALNG0080051		RNA Gene	GC10P099533	0.934955955
HSALNG0100581		RNA Gene	GC14P035354	0.934955955
HSALNG0100582		RNA Gene	GC14P035367	0.934955955
HSALNG0108142		RNA Gene	GC15M090591	0.934955955
HSALNG0115423		RNA Gene	GC17M027845	0.934955955
HSALNG0130310		RNA Gene	GC20M044419	0.934955955
HSALNG0130695		RNA Gene	GC20M049812	0.934955955
HSALNG0136252		RNA Gene	GC22P050016	0.934955955
lnc-ACAP3-2-001		RNA Gene	GC01M003079	0.934955955
lnc-CCDC8-6		RNA Gene	GC19M046344	0.934955955
lnc-CDH3-5		RNA Gene	GC16P068636	0.934955955
lnc-DNLZ-1		RNA Gene	GC09M136363	0.934955955
lnc-FOXD1-3		RNA Gene	GC05M073243	0.934955955

lnc-GOT1-1		RNA Gene	GC10M099517	0.934955955
lnc-HLA-DRB1-3		RNA Gene	GC06M032659	0.934955955
lnc-HNF4A-1		RNA Gene	GC20P044435	0.934955955
lnc-IQCH-5		RNA Gene	GC15P079643	0.934955955
lnc-PRKCD-2		RNA Gene	GC03P053048	0.934955955
lnc-SLC37A1-2		RNA Gene	GC21P042439	0.934955955
lnc-TTC33-6		RNA Gene	GC05M040484	0.934955955
lnc-IL12B-2		RNA Gene	GC05M159381	0.934955955
MK280144-295		RNA Gene	GC18P015166	0.934955955
MK280194		RNA Gene	GC16M028497	0.934955955
MN298214		RNA Gene	GC05P132451	0.934955955
HSALNG0085864		RNA Gene	GC11M076569	0.934955955
HSALNG0085865		RNA Gene	GC11P076573	0.934955955
HSALNG0094038		RNA Gene	GC12P111446	0.934955955
HSALNG0109573		RNA Gene	GC16M011055	0.934955955
HSALNG0109575		RNA Gene	GC16M011064	0.934955955
HSALNG0110697		RNA Gene	GC16P033781	0.934955955
HSALNG0132000		RNA Gene	GC21P015422	0.934955955
lnc-CAB39L-		RNA Gene	GC13M049044	0.934955955
lnc-CEP76-2		RNA Gene	GC18M019842	0.934955955
LOC112268088	Uncharacterized LOC112268088	RNA Gene	GC12P013715	0.934955955
lnc-REL-2		RNA Gene	GC02P060853	0.934955955
lnc-WNT4-6		RNA Gene	GC01M022366	0.934955955
HSALNG0096529-003		RNA Gene	GC13M040415	0.934955955
HSALNG0106744		RNA Gene	GC15P079457	0.934955955
HSALNG0133110		RNA Gene	GC21M039094	0.934955955
HSALNG0133104		RNA Gene	GC21M039033	0.934955955
HSALNG0133311		RNA Gene	GC21M042422	0.934955955
HSALNG0020027		RNA Gene	GC02M162268	0.934955955
HSALNG0021690		RNA Gene	GC02M203744	0.934955955
HSALNG0036088		RNA Gene	GC04M102566	0.934955955
HSALNG0044846		RNA Gene	GC05P132467	0.934955955
HSALNG0044847		RNA Gene	GC05M132472	0.934955955
HSALNG0048249		RNA Gene	GC06P014719	0.934955955
HSALNG0049105		RNA Gene	GC06P058853	0.934955955
HSALNG0068336		RNA Gene	GC08M129288	0.934955955
HSALNG0074774		RNA Gene	GC09M129891	0.934955955

HSALNG0074773		RNA Gene	GC09P129885	0.934955955
HSALNG0012886		RNA Gene	GC02M009255	0.934955955
HSALNG0017397		RNA Gene	GC02P102041	0.934955955
HSALNG0017398-002		RNA Gene	GC02M102057	0.934955955
HSALNG0041154		RNA Gene	GC05P035873	0.934955955
HSALNG0052448		RNA Gene	GC06M106046	0.934955955
HSALNG0055751		RNA Gene	GC07P002758	0.934955955
HSALNG0068536-001		RNA Gene	GC08P128544	0.934955955
HSALNG0068536-002		RNA Gene	GC08P128548	0.934955955
HSALNG0069758		RNA Gene	GC09M004978	0.934955955
HSALNG0074775		RNA Gene	GC09P129893	0.934955955
ENSG00000227836	Ribosomal Protein S20 (RPS20) Pseudogene	Pseudogene	GC05P096943	0.934955955
ENSG00000220412	Protein Tyrosine Phosphatase, Non-Receptor Type 11 (PTPN11)	Pseudogene	GC06M137706	0.934955955
ENSG00000254926	Glycine-N-Acyltransferase (GLYAT) Pseudogene	Pseudogene	GC11M071340	0.934955955
piR-37170-040		RNA Gene	GC02M218145	0.934955955
piR-43099-059		RNA Gene	GC10M079279	0.934955955
piR-45035-151		RNA Gene	GC05M096819	0.934955955
piR-47234		RNA Gene	GC15P080278	0.934955955
piR-48852		RNA Gene	GC03P049531	0.934955955
piR-61240-151		RNA Gene	GC02M191093	0.934955955
RF00001-253		RNA Gene	GC05M056147	0.934955955
RF00017-		RNA Gene	GC22P027931	0.934955955
RF00017-		RNA Gene	GC22P027935	0.934955955
RF00017-		RNA Gene	GC03P049431	0.934955955
HSALNG0098906		RNA Gene	GC13M099358	0.934955955
HSALNG0098908		RNA Gene	GC13M099394	0.934955955
HSALNG0110496		RNA Gene	GC16M028520	0.934955955
HSALNG0110740		RNA Gene	GC16P033457	0.934955955
HSALNG0116274		RNA Gene	GC17P039754	0.934955955
HSALNG0130316		RNA Gene	GC20P044447	0.934955955
HSALNG0131998-001		RNA Gene	GC21M015367	0.934955955
piR-41306-095		RNA Gene	GC16P011228	0.934955955
piR-43325-002		RNA Gene	GC01M019812	0.934955955
piR-44610-008		RNA Gene	GC05P040403	0.934955955
piR-48007		RNA Gene	GC12M111490	0.934955955

piR-48259	RNA Gene	GC18P079423	0.934955955
piR-48325-111	RNA Gene	GC21P039100	0.934955955
piR-51327	RNA Gene	GC12M111437	0.934955955
piR-52079-091	RNA Gene	GC18M069859	0.934955955
piR-52916-031	RNA Gene	GC06M159081	0.934955955
piR-55655-375	RNA Gene	GC22M050167	0.934955955
piR-57133-396	RNA Gene	GC22M050829	0.934955955
RF00017-	RNA Gene	GC02M024872	0.934955955
lnc-DYRK2-15	RNA Gene	GC12P068103	0.934955955
lnc-ICAM3-1	RNA Gene	GC19M010351	0.934955955
lnc-ITGB1BP1-2	RNA Gene	GC02M009272	0.934955955
lnc-PRDM1-1	RNA Gene	GC06P106077	0.934955955
lnc-SFMBT1-	RNA Gene	GC03M052954	0.934955955
piR-36455	RNA Gene	GC12M111444	0.934955955
piR-38259	RNA Gene	GC12M111486	0.934955955
piR-39099-151	RNA Gene	GC03P013448	0.934955955
piR-43105-342	RNA Gene	GC02M025261	0.934955955
piR-49732-033	RNA Gene	GC10M006055	0.934955955
piR-50346	RNA Gene	GC12M111491	0.934955955
piR-50437-360	RNA Gene	GC02M024901	0.934955955
piR-51449	RNA Gene	GC12P111448	0.934955955
piR-56480-015	RNA Gene	GC12M111439	0.934955955
RF00017-	RNA Gene	GC13M040467	0.934955955
piR-38319	RNA Gene	GC01P198684	0.934955955
piR-50444-308	RNA Gene	GC03P046390	0.934955955
piR-50893	RNA Gene	GC01P002229	0.934955955
piR-55281-150	RNA Gene	GC13M099289	0.934955955
RF00017-	RNA Gene	GC04P101880	0.934955955
NONHSAG017238.2	RNA Gene	GC15P079763	0.934955955
piR-31937-039	RNA Gene	GC11P128318	0.934955955
piR-31937-163	RNA Gene	GC04P105169	0.934955955
piR-31937-161	RNA Gene	GC04M101809	0.934955955
MN309174-586	RNA Gene	GC07M002758	0.934955955
piR-30396	RNA Gene	GC15P079788	0.934955955
piR-33432-055	RNA Gene	GC10M062607	0.934955955
MN309188	RNA Gene	GC10M079292	0.934955955
NONHSAG031883.2-001	RNA Gene	GC20P046119	0.934955955
NONHSAG031883.2-002	RNA Gene	GC20P046128	0.934955955
NONHSAG045774.2	RNA Gene	GC06P059692	0.934955955
piR-31152	RNA Gene	GC07M002819	0.934955955

HSALNG0078329		RNA Gene	GC10P062545	0.934955955
HSALNG0079128		RNA Gene	GC10P086115	0.934955955
HSALNG0009608		RNA Gene	GC01P200902	0.934955955
HSALNG0079551		RNA Gene	GC10P088990	0.934955955
RF00017-		RNA Gene	GC05P056156	0.934955955
RF00017-		RNA Gene	GC05M172914	0.934955955
RF00994-753		RNA Gene	GC04P102592	0.934955955
RF00017-		RNA Gene	GC06M090260	0.934955955
RF00017-		RNA Gene	GC09M129899	0.934955955
LOC102724748	Uncharacterized LOC102724748	RNA Gene	GC05P096784	0.934955955
LOC105369818	Uncharacterized LOC105369818	RNA Gene	GC12P068198	0.934955955
LOC107984384	Uncharacterized LOC107984384	RNA Gene	GC11M109875	0.934955955
LOC105373831	Uncharacterized LOC105373831	RNA Gene	GC02M198493	0.934955955
LOC101927897	Uncharacterized LOC101927897	RNA Gene	GC18M079457	0.934955955
LOC105372877	Uncharacterized LOC105372877	RNA Gene	GC01M206748	0.934955955
LOC105377139	Uncharacterized LOC105377139	RNA Gene	GC21M044172	0.934955955
LOC107985484	Uncharacterized LOC107985484	RNA Gene	GC21M039030	0.934955955
LOC107985926	Uncharacterized LOC107985926	RNA Gene	GC02P102032	0.934955955
HSALNG0015266		RNA Gene	GC02P060949	0.934955955
HSALNG0015265		RNA Gene	GC02P060942	0.934955955
HSALNG0015371		RNA Gene	GC02M062331	0.934955955
HSALNG0042579		RNA Gene	GC05P072413	0.934955955
HSALNG0060455		RNA Gene	GC07M107857	0.934955955
HSALNG0015546		RNA Gene	GC02M065457	0.934955955
HSALNG0026207		RNA Gene	GC03M052969	0.934955955
HSALNG0046199		RNA Gene	GC05M159447	0.934955955
HSALNG0053005		RNA Gene	GC06P116447	0.934955955
HSALNG0068897-001		RNA Gene	GC08P137116	0.934955955
HSALNG0068898		RNA Gene	GC08M137122	0.934955955
ENSG00000279625	TEC	Uncategorized	GC01M022364	0.934955955
HSALNG0007877-002		RNA Gene	GC01M160870	0.934955955
HSALNG0007877-001		RNA Gene	GC01M160869	0.934955955
HSALNG0001583		RNA Gene	GC01P022383	0.934955955
HSALNG0006204		RNA Gene	GC01M113940	0.934955955

HSALNG0007483		RNA Gene	GC01M155226	0.934955955
ENSG00000259970	Novel Transcript	RNA Gene	GC03M049684	0.934955955
HSALNG0080046		RNA Gene	GC10M099523	0.934955955
HSALNG0115400		RNA Gene	GC17P030338	0.934955955
HSALNG0115422		RNA Gene	GC17M027788	0.934955955
HSALNG0123928		RNA Gene	GC19M010352	0.934955955
HSALNG0127574		RNA Gene	GC19M054867	0.934955955
HSALNG0130311		RNA Gene	GC20M044426	0.934955955
HSALNG0130312		RNA Gene	GC20M044430	0.934955955
HSALNG0130314		RNA Gene	GC20P044443	0.934955955
HSALNG0133108		RNA Gene	GC21M039093	0.934955955
lnc-ZMAT5-2		RNA Gene	GC22M029746	0.934955955
lnc-TNFSF15-5		RNA Gene	GC09M114817	0.934955955
HSALNG0091478		RNA Gene	GC12P056006	0.934955955
HSALNG0134847		RNA Gene	GC22P029768	0.934955955
lnc-BOLL-6		RNA Gene	GC02M198007	0.934955955
HSALNG0084356		RNA Gene	GC11P058636	0.934955955
HSALNG0084357		RNA Gene	GC11P058653	0.934955955
HSALNG0088853-002		RNA Gene	GC12P013567	0.934955955
HSALNG0092143		RNA Gene	GC12P068113	0.934955955
HSALNG0095902		RNA Gene	GC13P026963	0.934955955
HSALNG0111330		RNA Gene	GC16M050302	0.934955955
HSALNG0021401		RNA Gene	GC02M198008	0.934955955
HSALNG0023723		RNA Gene	GC02M241794	0.934955955
HSALNG0032571-002		RNA Gene	GC04P004971	0.934955955
HSALNG0041355		RNA Gene	GC05P040225	0.934955955
HSALNG0049125		RNA Gene	GC06M049612	0.934955955
HSALNG0049272		RNA Gene	GC06M049652	0.934955955
HSALNG0049431		RNA Gene	GC06M049686	0.934955955
HSALNG0050236		RNA Gene	GC06P059172	0.934955955
HSALNG0060454		RNA Gene	GC07P107847	0.934955955
HSALNG0015256		RNA Gene	GC02P060859	0.934955955

HSALNG0032579	RNA Gene	GC04M004984	0.934955955
HSALNG0041369	RNA Gene	GC05P040464	0.934955955
HSALNG0041374	RNA Gene	GC05P040512	0.934955955
HSALNG0042617-002	RNA Gene	GC05M073244	0.934955955
HSALNG0049351	RNA Gene	GC06P058910	0.934955955
HSALNG0050238	RNA Gene	GC06M049872	0.934955955
HSALNG0052450	RNA Gene	GC06M106068	0.934955955
HSALNG0055975	RNA Gene	GC07P006504	0.934955955
HSALNG0077262	RNA Gene	GC10M034994	0.934955955
HSALNG0077263	RNA Gene	GC10P035012	0.934955955
piR-40398	RNA Gene	GC18M020032	0.934955955
piR-41306-110	RNA Gene	GC17M027548	0.934955955
piR-43939-002	RNA Gene	GC01P007955	0.934955955
piR-44610-011	RNA Gene	GC06M032994	0.934955955
piR-51267-027	RNA Gene	GC07M017417	0.934955955
piR-60146-087	RNA Gene	GC02P197990	0.934955955
RF00017-	RNA Gene	GC16P034025	0.934955955
RF00017-405	RNA Gene	GC01M206773	0.934955955
HSALNG0084553	RNA Gene	GC11P061781	0.934955955
HSALNG0092137	RNA Gene	GC12P068196	0.934955955
HSALNG0113256	RNA Gene	GC16P085964	0.934955955
HSALNG0129763	RNA Gene	GC20M035208	0.934955955
hsa-miR-5095-436	RNA Gene	GC06P137645	0.934955955
piR-38580-144	RNA Gene	GC19P034160	0.934955955
piR-39098-183	RNA Gene	GC04M122670	0.934955955
piR-48325-110	RNA Gene	GC21M039092	0.934955955
piR-55186-001	RNA Gene	GC01P008021	0.934955955
piR-58297-114	RNA Gene	GC12M055999	0.934955955
piR-58538-006	RNA Gene	GC16P085977	0.934955955
piR-58538-001	RNA Gene	GC16P085972	0.934955955
piR-58538-007	RNA Gene	GC16P085978	0.934955955
piR-58538-008	RNA Gene	GC16P085979	0.934955955
piR-58538-013	RNA Gene	GC16P085985	0.934955955

piR-58538-010		RNA Gene	GC16P085981	0.934955955
piR-58538-015		RNA Gene	GC16P085987	0.934955955
piR-61101-576		RNA Gene	GC06P060745	0.934955955
piR-61945-308		RNA Gene	GC20P044436	0.934955955
RF00017-263		RNA Gene	GC01M113952	0.934955955
RF00017-		RNA Gene	GC02M060983	0.934955955
RF00017-		RNA Gene	GC02P203755	0.934955955
lnc-ICAM3-2		RNA Gene	GC19M010354	0.934955955
piR-39767-002		RNA Gene	GC01M003147	0.934955955
piR-46002-152		RNA Gene	GC13M049019	0.934955955
piR-46391-002		RNA Gene	GC01P154462	0.934955955
piR-48749-023		RNA Gene	GC02M198029	0.934955955
piR-48759-204		RNA Gene	GC03P053025	0.934955955
piR-48820-008		RNA Gene	GC01M155215	0.934955955
piR-50308-096		RNA Gene	GC11M076585	0.934955955
piR-56341-188		RNA Gene	GC05P135093	0.934955955
piR-56451-093		RNA Gene	GC07M107849	0.934955955
piR-55655-210		RNA Gene	GC16P033415	0.934955955
piR-56341-125		RNA Gene	GC02M198679	0.934955955
piR-58538-012		RNA Gene	GC16P085984	0.934955955
piR-58538-009		RNA Gene	GC16P085980	0.934955955
piR-58538-014		RNA Gene	GC16P085986	0.934955955
piR-58538-011		RNA Gene	GC16P085983	0.934955955
piR-58538-016		RNA Gene	GC16P085988	0.934955955
NONHSAG046314.2		RNA Gene	GC06M050130	0.934955955
piR-32461-022		RNA Gene	GC10M062677	0.934955955
piR-33804-078		RNA Gene	GC07P017398	0.934955955
NONHSAG008489.2		RNA Gene	GC11P061783	0.934955955
piR-32285-085		RNA Gene	GC07P107810	0.934955955
NONHSAG045795.2		RNA Gene	GC06P059699	0.934955955
LOC646347	Spermine Synthase Pseudogene	Pseudogene	GC01P160919	0.934955955
HSALNG0078331		RNA Gene	GC10P062683	0.934955955
HSALNG0009609		RNA Gene	GC01P200908	0.934955955
lnc-CYTL1-3		RNA Gene	GC04M004973	0.934955955
MN298667		RNA Gene	GC09P136374	0.934955955

RF00017-		RNA Gene	GC05P072398	0.934955955
RF00017-		RNA Gene	GC05P144325	0.934955955
RF00994-809		RNA Gene	GC05P010687	0.934955955
RF00994-819		RNA Gene	GC05M040176	0.934955955
LOC105371887	Uncharacterized LOC105371887	RNA Gene	GC17P072640	0.934955955
LOC105375444	Uncharacterized LOC105375444	RNA Gene	GC07M107856	0.934955955
HSALNG0013825		RNA Gene	GC02P027507	0.934955955
piR-43107-300		RNA Gene	GC08M137128	0.934955955
RF00017-212		RNA Gene	GC01M067259	0.934955955
piR-32810-138		RNA Gene	GC09M114834	0.934955955
HSALNG0079716		RNA Gene	GC10P092671	0.934955955
HSALNG0079717		RNA Gene	GC10M092671	0.934955955
DVL2	Dishevelled Segment Polarity Protein 2	Protein Coding	GC17M007225	0.932829022
MCM4	Minichromosome Maintenance Complex Component 4	Protein Coding	GC08P047965	0.932659864
CIRBP	Cold Inducible RNA Binding Protein	Protein Coding	GC19P001259	0.932502508
SLC23A1	Solute Carrier Family 23 Member 1	Protein Coding	GC05M139377	0.929340422
MIR30E	MicroRNA 30e	RNA Gene	GC01P040754	0.92816925
COL6A3	Collagen Type VI Alpha 3 Chain	Protein Coding	GC02M237324	0.923105359
P4HB	Prolyl 4-Hydroxylase Subunit Beta	Protein Coding	GC17M081843	0.922585249
ASPH	Aspartate Beta-Hydroxylase	Protein Coding	GC08M061500	0.922585249
ASGR1	Asialoglycoprotein Receptor 1	Protein Coding	GC17M007173	0.922518134
SELENBP1	Selenium Binding Protein 1	Protein Coding	GC01M151364	0.919024408
MAZ	MYC Associated Zinc Finger Protein	Protein Coding	GC16P029806	0.919024408
CHMP5	Charged Multivesicular Body Protein 5	Protein Coding	GC09P033257	0.919024408
KRT9	Keratin 9	Protein Coding	GC17M041565	0.918429017
PNP	Purine Nucleoside Phosphorylase	Protein Coding	GC14P020468	0.917328656
CNGA2	Cyclic Nucleotide Gated Channel Subunit Alpha 2	Protein Coding	GC0XP151734	0.917328656
SYNE2	Spectrin Repeat Containing Nuclear Envelope Protein 2	Protein Coding	GC14P063761	0.917328656
LIN54	Lin-54 DREAM MuvB Core Complex Component	Protein Coding	GC04M082909	0.917328656
HMGN4	High Mobility Group Nucleosomal Binding Domain 4	Protein Coding	GC06P026538	0.917328656
ALOX15B	Arachidonate 15-Lipoxygenase Type B	Protein Coding	GC17P008039	0.912078202
NCR1	Natural Cytotoxicity Triggering Receptor 1	Protein Coding	GC19P054906	0.911244392
CNR2	Cannabinoid Receptor 2	Protein Coding	GC01M023870	0.909843326
HRH1	Histamine Receptor H1	Protein Coding	GC03P011163	0.906831741
IFIT2	Interferon Induced Protein With Tetratricopeptide Repeats 2	Protein Coding	GC10P089284	0.906054854
MIR449A	MicroRNA 449a	RNA Gene	GC05M055171	0.902678251
CD74	CD74 Molecule	Protein Coding	GC05M150378	0.899163127
RBMS2	RNA Binding Motif Single Stranded Interacting Protein 2	Protein Coding	GC12P056663	0.898166776
GIP	Gastric Inhibitory Polypeptide	Protein Coding	GC17M048958	0.89745152
NR3C2	Nuclear Receptor Subfamily 3 Group C Member 2	Protein Coding	GC04M148078	0.894555032
SIK2	Salt Inducible Kinase 2	Protein Coding	GC11P111640	0.892157972
POU2F1	POU Class 2 Homeobox 1	Protein Coding	GC01P167190	0.891228914
LILRB4	Leukocyte Immunoglobulin Like Receptor B4	Protein Coding	GC19P054643	0.891228914
CCL13	C-C Motif Chemokine Ligand 13	Protein Coding	GC17P034356	0.891228914
IL34	Interleukin 34	Protein Coding	GC16P070728	0.886736929
INSR	Insulin Receptor	Protein Coding	GC19M007112	0.885652542

CSNK2A1	Casein Kinase 2 Alpha 1	Protein Coding	GC20M000472	0.885652542
EPHB2	EPH Receptor B2	Protein Coding	GC01P022710	0.885652542
FZD4	Frizzled Class Receptor 4	Protein Coding	GC11M086945	0.885652542
PIK3R2	Phosphoinositide-3-Kinase Regulatory Subunit 2	Protein Coding	GC19P018153	0.885652542
CACNA1G	Calcium Voltage-Gated Channel Subunit Alpha1 G	Protein Coding	GC17P050561	0.885652542
CYP19A1	Cytochrome P450 Family 19 Subfamily A Member 1	Protein Coding	GC15M051208	0.885652542
CASP2	Caspase 2	Protein Coding	GC07P146115	0.885652542
DICER1	Dicer 1, Ribonuclease III	Protein Coding	GC14M095086	0.885652542
PRKACB	Protein Kinase CAMP-Activated Catalytic Subunit Beta	Protein Coding	GC01P084078	0.885652542
RHEB	Ras Homolog, MTORC1 Binding	Protein Coding	GC07M151466	0.885652542
PLK4	Polo Like Kinase 4	Protein Coding	GC04P127880	0.885652542
ADORA1	Adenosine A1 Receptor	Protein Coding	GC01P203090	0.885652542
FZD6	Frizzled Class Receptor 6	Protein Coding	GC08P103298	0.885652542
HK2	Hexokinase 2	Protein Coding	GC02P074833	0.885652542
HTR7	5-Hydroxytryptamine Receptor 7	Protein Coding	GC10M090740	0.885652542
FOLH1	Folate Hydrolase 1	Protein Coding	GC11M071265	0.885652542
KDM1A	Lysine Demethylase 1A	Protein Coding	GC01P023019	0.885652542
PIK3R4	Phosphoinositide-3-Kinase Regulatory Subunit 4	Protein Coding	GC03M130678	0.885652542
PPP2R1B	Protein Phosphatase 2 Scaffold Subunit Abeta	Protein Coding	GC11M111695	0.885652542
KDM6A	Lysine Demethylase 6A	Protein Coding	GC0XP044873	0.885652542
LRP1	LDL Receptor Related Protein 1	Protein Coding	GC12P057128	0.885652542
RRM2	Ribonucleotide Reductase Regulatory Subunit M2	Protein Coding	GC02P010123	0.885652542
DCN	Decorin	Protein Coding	GC12M091140	0.885652542
HNRNPA1	Heterogeneous Nuclear Ribonucleoprotein A1	Protein Coding	GC12P054280	0.885652542
SOX5	SRY-Box Transcription Factor 5	Protein Coding	GC12M023529	0.885652542
AKAP9	A-Kinase Anchoring Protein 9	Protein Coding	GC07P091940	0.885652542
E2F4	E2F Transcription Factor 4	Protein Coding	GC16P067192	0.885652542
EPHB6	EPH Receptor B6	Protein Coding	GC07P146108	0.885652542
FZD1	Frizzled Class Receptor 1	Protein Coding	GC07P091264	0.885652542
HNRNPK	Heterogeneous Nuclear Ribonucleoprotein K	Protein Coding	GC09M086732	0.885652542
PSMB7	Proteasome 20S Subunit Beta 7	Protein Coding	GC09M124353	0.885652542
PIK3R5	Phosphoinositide-3-Kinase Regulatory Subunit 5	Protein Coding	GC17M008878	0.885652542
MELK	Maternal Embryonic Leucine Zipper Kinase	Protein Coding	GC09P036572	0.885652542
PXN	Paxillin	Protein Coding	GC12M120210	0.885652542
RALA	RAS Like Proto-Oncogene A	Protein Coding	GC07P039622	0.885652542
RALB	RAS Like Proto-Oncogene B	Protein Coding	GC02P120240	0.885652542
SHMT1	Serine Hydroxymethyltransferase 1	Protein Coding	GC17M022154	0.885652542
ROS1	ROS Proto-Oncogene 1, Receptor Tyrosine Kinase	Protein Coding	GC06M117287	0.885652542
SEMA4A	Semaphorin 4A	Protein Coding	GC01P156147	0.885652542
TFDP1	Transcription Factor Dp-1	Protein Coding	GC13P113584	0.885652542
SAT1	Spermidine/Spermine N1-Acetyltransferase 1	Protein Coding	GC0XP023784	0.885652542
WNT11	Wnt Family Member 11	Protein Coding	GC11M076186	0.885652542
CES2	Carboxylesterase 2	Protein Coding	GC16P066934	0.885652542
BCAR1	BCAR1 Scaffold Protein, Cas Family Member	Protein Coding	GC16M075228	0.885652542
FZD10	Frizzled Class Receptor 10	Protein Coding	GC12P130162	0.885652542
PDGFD	Platelet Derived Growth Factor D	Protein Coding	GC11M103907	0.885652542
NUAK1	NUAK Family Kinase 1	Protein Coding	GC12M106063	0.885652542
STK24	Serine/Threonine Kinase 24	Protein Coding	GC13M098445	0.885652542
TPH1	Tryptophan Hydroxylase 1	Protein Coding	GC11M018040	0.885652542

WNT5B	Wnt Family Member 5B	Protein Coding	GC12P001529	0.885652542
TNIK	TRAF2 And NCK Interacting Kinase	Protein Coding	GC03M171061	0.885652542
WNT7B	Wnt Family Member 7B	Protein Coding	GC22M045920	0.885652542
ACSL5	Acyl-CoA Synthetase Long Chain Family Member 5	Protein Coding	GC10P112374	0.885652542
FPGS	Folylpolyglutamate Synthase	Protein Coding	GC09P127794	0.885652542
GRPR	Gastrin Releasing Peptide Receptor	Protein Coding	GC0XP016141	0.885652542
POLE2	DNA Polymerase Epsilon 2, Accessory Subunit	Protein Coding	GC14M049643	0.885652542
MBD4	Methyl-CpG Binding Domain 4, DNA Glycosylase	Protein Coding	GC03M129430	0.885652542
QKI	QKI, KH Domain Containing RNA Binding	Protein Coding	GC06P163414	0.885652542
PTGES2	Prostaglandin E Synthase 2	Protein Coding	GC09M128120	0.885652542
ST3GAL1	ST3 Beta-Galactoside Alpha-2,3-Sialyltransferase 1	Protein Coding	GC08M133454	0.885652542
STMN1	Stathmin 1	Protein Coding	GC01M025884	0.885652542
TRIM28	Tripartite Motif Containing 28	Protein Coding	GC19P058544	0.885652542
SLC16A7	Solute Carrier Family 16 Member 7	Protein Coding	GC12P059596	0.885652542
PTTG1	PTTG1 Regulator Of Sister Chromatid Separation, Securin	Protein Coding	GC05P160422	0.885652542
BNIP3	BCL2 Interacting Protein 3	Protein Coding	GC10M131966	0.885652542
LASP1	LIM And SH3 Protein 1	Protein Coding	GC17P038869	0.885652542
S100A11	S100 Calcium Binding Protein A11	Protein Coding	GC01M152032	0.885652542
XRCC2	X-Ray Repair Cross Complementing 2	Protein Coding	GC07M152644	0.885652542
RALGDS	Ral Guanine Nucleotide Dissociation Stimulator	Protein Coding	GC09M133097	0.885652542
ST3GAL4	ST3 Beta-Galactoside Alpha-2,3-Sialyltransferase 4	Protein Coding	GC11P126355	0.885652542
AKAP12	A-Kinase Anchoring Protein 12	Protein Coding	GC06P151239	0.885652542
FUT6	Fucosyltransferase 6	Protein Coding	GC19M005830	0.885652542
GTF2B	General Transcription Factor IIB	Protein Coding	GC01M088853	0.885652542
CHKA	Choline Kinase Alpha	Protein Coding	GC11M068052	0.885652542
LIMS1	LIM Zinc Finger Domain Containing 1	Protein Coding	GC02P108534	0.885652542
IRS4	Insulin Receptor Substrate 4	Protein Coding	GC0XM108720	0.885652542
PHLPP2	PH Domain And Leucine Rich Repeat Protein Phosphatase 2	Protein Coding	GC16M071637	0.885652542
KMT2C	Lysine Methyltransferase 2C	Protein Coding	GC07M152134	0.885652542
PTP4A1	Protein Tyrosine Phosphatase 4A1	Protein Coding	GC06P063521	0.885652542
PTP4A3	Protein Tyrosine Phosphatase 4A3	Protein Coding	GC08P141391	0.885652542
TCF7L1	Transcription Factor 7 Like 1	Protein Coding	GC02P085133	0.885652542
RASSF2	Ras Association Domain Family Member 2	Protein Coding	GC20M004780	0.885652542
WNT8A	Wnt Family Member 8A	Protein Coding	GC05P138097	0.885652542
WNT9B	Wnt Family Member 9B	Protein Coding	GC17P046833	0.885652542
AGO2	Argonaute RISC Catalytic Component	Protein Coding	GC08M140522	0.885652542
CRYGC	Crystallin Gamma C	Protein Coding	GC02M208128	0.885652542
FAT4	FAT Atypical Cadherin 4	Protein Coding	GC04P125315	0.885652542
NFYB	Nuclear Transcription Factor Y Subunit Beta	Protein Coding	GC12M104117	0.885652542
PMAIP1	Phorbol-12-Myristate-13-Acetate-Induced Protein 1	Protein Coding	GC18P059899	0.885652542
PHLPP1	PH Domain And Leucine Rich Repeat Protein Phosphatase 1	Protein Coding	GC18P062715	0.885652542
LLGL1	LLGL Scribble Cell Polarity Complex Component 1	Protein Coding	GC17P018225	0.885652542
PZP	PZP Alpha-2-Macroglobulin Like	Protein Coding	GC12M009148	0.885652542
ST6GALNAC2	ST6 N-Acetylgalactosaminide Alpha-2,6-Sialyltransferase 2	Protein Coding	GC17M076565	0.885652542
USP28	Ubiquitin Specific Peptidase 28	Protein Coding	GC11M113797	0.885652542
CD276	CD276 Molecule	Protein Coding	GC15P073683	0.885652542
EIF5A2	Eukaryotic Translation Initiation Factor 5A2	Protein Coding	GC03M170888	0.885652542

HLTF	Helicase Like Transcription Factor	Protein Coding	GC03M149030	0.885652542
ST3GAL2	ST3 Beta-Galactoside Alpha-2,3-Sialyltransferase 2	Protein Coding	GC16M070375	0.885652542
GPA33	Glycoprotein A33	Protein Coding	GC01M167052	0.885652542
NANOG	Nanog Homeobox	Protein Coding	GC12P007787	0.885652542
PLA2G4B	Phospholipase A2 Group IVB	Protein Coding	GC15P041837	0.885652542
YBX1	Y-Box Binding Protein 1	Protein Coding	GC01P042682	0.885652542
RNF43	Ring Finger Protein 43	Protein Coding	GC17M058352	0.885652542
TNS4	Tensin 4	Protein Coding	GC17M040475	0.885652542
ZFP36L2	ZFP36 Ring Finger Protein Like 2	Protein Coding	GC02M043184	0.885652542
ZNF217	Zinc Finger Protein 217	Protein Coding	GC20M053567	0.885652542
HAPLN3	Hyaluronan And Proteoglycan Link Protein 3	Protein Coding	GC15M088877	0.885652542
GRK2	G Protein-Coupled Receptor Kinase 2	Protein Coding	GC11P067266	0.885652542
HOXB8	Homeobox B8	Protein Coding	GC17M048611	0.885652542
HOXB6	Homeobox B6	Protein Coding	GC17M048646	0.885652542
CLDND1	Claudin Domain Containing 1	Protein Coding	GC03M098497	0.885652542
EPDR1	Ependymin Related 1	Protein Coding	GC07P037696	0.885652542
HTR3E	5-Hydroxytryptamine Receptor 3E	Protein Coding	GC03P184098	0.885652542
GXYLT2	Glucoside Xylosyltransferase 2	Protein Coding	GC03P072888	0.885652542
CSMD3	CUB And Sushi Multiple Domains 3	Protein Coding	GC08M112223	0.885652542
MACC1	MET Transcriptional Regulator	Protein Coding	GC07M020140	0.885652542
VWA2	Von Willebrand Factor A Domain Containing 2	Protein Coding	GC10P114239	0.885652542
SEPTIN9	Septin 9	Protein Coding	GC17P077282	0.885652542
DDX53	DEAD-Box Helicase 53	Protein Coding	GC0XP022999	0.885652542
PSG2	Pregnancy Specific Beta-1-Glycoprotein 2	Protein Coding	GC19M043064	0.885652542
MUC21	Mucin 21, Cell Surface Associated	Protein Coding	GC06P058293	0.885652542
WRAP73	WD Repeat Containing, Antisense To TP73	Protein Coding	GC01M003630	0.885652542
GSDME	Gasdermin E	Protein Coding	GC07M024699	0.885652542
KRTDAP	Keratinocyte Differentiation Associated Protein	Protein Coding	GC19M049287	0.885652542
P3H3	Prolyl 3-Hydroxylase 3	Protein Coding	GC12P013171	0.885652542
OTOP2	Otopetrin 2	Protein Coding	GC17P074924	0.885652542
MRGPRE	MAS Related GPR Family Member E	Protein Coding	GC11M003226	0.885652542
MRGPRG	MAS Related GPR Family Member G	Protein Coding	GC11M003218	0.885652542
KRTAP9-2	Keratin Associated Protein 9-2	Protein Coding	GC17P041256	0.885652542
WDCP	WD Repeat And Coiled Coil	Protein Coding	GC02M024032	0.885652542
COLCA2	Colorectal Cancer Associated 2	Protein Coding	GC11P111298	0.885652542
TP53TG1	TP53 Target 1	RNA Gene	GC07M087325	0.885652542
COLCA1	Colorectal Cancer Associated 1	RNA Gene	GC11M111290	0.885652542
LINC00472	Long Intergenic Non-Protein Coding RNA 472	RNA Gene	GC06M071344	0.885652542
FTX	FTX Transcript, XIST Regulator	RNA Gene	GC0XM073946	0.885652542
MIR96	MicroRNA 96	RNA Gene	GC07M129774	0.885652542
MIR128-2	MicroRNA 128-2	RNA Gene	GC03P035750	0.885652542
MIR32	MicroRNA 32	RNA Gene	GC09M109046	0.885652542
MIR339	MicroRNA 339	RNA Gene	GC07M001022	0.885652542
HOXB-AS3	HOXB Cluster Antisense RNA 3	RNA Gene	GC17P048549	0.885652542
MIR328	MicroRNA 328	RNA Gene	GC16M067203	0.885652542
MIR340	MicroRNA 340	RNA Gene	GC05M180015	0.885652542
SCARNA6	Small Cajal Body-Specific RNA 6	RNA Gene	GC02P233288	0.885652542
BCYRN1	Brain Cytoplasmic RNA 1	RNA Gene	GC02P047331	0.885652542
DLEU7-AS1	DLEU7 Antisense RNA 1	RNA Gene	GC13P050105	0.885652542
FBXL19-AS1	FBXL19 Antisense RNA 1	RNA Gene	GC16M031799	0.885652542
MIR497	MicroRNA 497	RNA Gene	GC17M007022	0.885652542
MIR7-3	MicroRNA 7-3	RNA Gene	GC19P004770	0.885652542
MRGPRG-AS1	MRGPRG Antisense RNA 1	RNA Gene	GC11P003218	0.885652542
SOX21-AS1	SOX21 Antisense Divergent Transcript	RNA Gene	GC13P094712	0.885652542
SNHG3	Small Nucleolar RNA Host Gene 3	RNA Gene	GC01P028506	0.885652542

SNHG17	Small Nucleolar RNA Host Gene 17	RNA Gene	GC20M038521	0.885652542
CAHM	Colon Adenocarcinoma	RNA Gene	GC06M163413	0.885652542
LEF1-AS1	LEF1 Antisense RNA 1	RNA Gene	GC04P108167	0.885652542
LINC-PINT	Long Intergenic Non-Protein Coding RNA, P53 Induced Transcript	RNA Gene	GC07M130934	0.885652542
SNORA54	Small Nucleolar RNA, H/ACA Box 54	RNA Gene	GC11M002968	0.885652542
IQANK1	IQ Motif And Ankyrin Repeat Containing 1	Protein Coding	GC08P143825	0.885652542
MIR372	MicroRNA 372	RNA Gene	GC19P056829	0.885652542
MNX1-AS1	MNX1 Antisense RNA 1 (Head To	RNA Gene	GC07P157010	0.885652542
MACROD2-AS1	MACROD2 Antisense RNA 1	RNA Gene	GC20M014866	0.885652542
MIR95	MicroRNA 95	RNA Gene	GC04M008007	0.885652542
MIR422A	MicroRNA 422a	RNA Gene	GC15M063870	0.885652542
VIM-AS1	VIM Antisense RNA 1	RNA Gene	GC10M017172	0.885652542
RNY1	RNA, Ro60-Associated Y1	RNA Gene	GC07M148987	0.885652542
CASC11	Cancer Susceptibility 11	RNA Gene	GC08M129249	0.885652542
CASC19	Cancer Susceptibility 19	RNA Gene	GC08M129239	0.885652542
CASC8	Cancer Susceptibility 8	RNA Gene	GC08M129240	0.885652542
FOXP4-AS1	FOXP4 Antisense RNA 1	RNA Gene	GC06M041494	0.885652542
HIF1A-AS1	HIF1A Antisense RNA 1	RNA Gene	GC14M061681	0.885652542
LINC01133	Long Intergenic Non-Protein Coding RNA 1133	RNA Gene	GC01P159959	0.885652542
LINC00659	Long Intergenic Non-Protein Coding RNA 659	RNA Gene	GC20M062774	0.885652542
POU5F1P4	POU Class 5 Homeobox 1 Pseudogene	Pseudogene	GC01P155444	0.885652542
NNT-AS1	NNT Antisense RNA 1	RNA Gene	GC05M043775	0.885652542
MIR625	MicroRNA 625	RNA Gene	GC14P065471	0.885652542
RNY3	RNA, Ro60-Associated Y3	RNA Gene	GC07P149006	0.885652542
C10orf143	Chromosome 10 Open Reading Frame 143	Protein Coding	GC10M130020	0.885652542
DPP10-AS1	DPP10 Antisense RNA 1	RNA Gene	GC02M115130	0.885652542
DLGAP4-	DLGAP4 Antisense RNA 1	RNA Gene	GC20M036509	0.885652542
GABPB1-	GABPB1 Antisense RNA 1	RNA Gene	GC15P050354	0.885652542
LINC00858	Long Intergenic Non-Protein Coding RNA 858	RNA Gene	GC10P086049	0.885652542
LINC01567	Long Intergenic Non-Protein Coding RNA 1567	RNA Gene	GC16M024661	0.885652542
SBDSP1	SBDS Pseudogene 1	Pseudogene	GC07P072860	0.885652542
SLC25A25-AS1	SLC25A25 Antisense RNA 1	RNA Gene	GC09M128108	0.885652542
BACE1-AS	BACE1 Antisense RNA	RNA Gene	GC11P117323	0.885652542
HIPK1-AS1	HIPK1 Antisense RNA 1	RNA Gene	GC01M113923	0.885652542
LINC01507	Long Intergenic Non-Protein Coding RNA 1507	RNA Gene	GC09P079824	0.885652542
GSEC	G-Quadruplex Forming Sequence Containing LncRNA	RNA Gene	GC11M126340	0.885652542
MAMDC2-AS1	MAMDC2 Antisense RNA 1	RNA Gene	GC09M070036	0.885652542
CLMAT3	Colorectal Liver Metastasis Associated Transcript 3	RNA Gene	GC05P151677	0.885652542
LINC01618	Long Intergenic Non-Protein Coding RNA 1618	RNA Gene	GC04P052743	0.885652542
LINC01630	Long Intergenic Non-Protein Coding RNA 1630	RNA Gene	GC18P051348	0.885652542
GPRC5D-	GPRC5D And HEBP1 Antisense RNA	RNA Gene	GC12P013471	0.885652542
EHHADH-AS1	EHHADH Antisense RNA 1	RNA Gene	GC03P185162	0.885652542
ZNF582-DT	ZNF582 Divergent Transcript	RNA Gene	GC19P057111	0.885652542
MROCKI	MARCKS Cis Regulating LncRNA Promoter Of Cytokines And	RNA Gene	GC06M113869	0.885652542
PURPL	P53 Upregulated Regulator Of P53	RNA Gene	GC05P027217	0.885652542
TUSC8	Tumor Suppressor Candidate 8	RNA Gene	GC13M044400	0.885652542

DUXAP10	Double Homeobox A Pseudogene 10	Pseudogene	GC14M019275	0.885652542
AOC4P	Amine Oxidase Copper Containing 4, Pseudogene	Pseudogene	GC17P042865	0.885652542
LINC01527	Long Intergenic Non-Protein Coding RNA 1527	RNA Gene	GC01M152930	0.885652542
LINC00538	Long Intergenic Non-Protein Coding RNA 538	RNA Gene	GC01P213924	0.885652542
PINCR	P53-Induced Noncoding RNA	RNA Gene	GC0XP043177	0.885652542
RBM5-AS1	RBM5 Antisense RNA 1	RNA Gene	GC03M050099	0.885652542
WSPAR	WNT Signaling Pathway Activating Non-Coding RNA	RNA Gene	GC05P133914	0.885652542
LAMC1-AS1	LAMC1 Antisense RNA 1	RNA Gene	GC01M183138	0.885652542
LINC02446	Long Intergenic Non-Protein Coding RNA 2446	RNA Gene	GC12P013340	0.885652542
LINC02223	Long Intergenic Non-Protein Coding RNA 2223	RNA Gene	GC05P017986	0.885652542
BTG3-AS1	BTG3 Antisense RNA 1	RNA Gene	GC21P017612	0.885652542
LINC02086	Long Intergenic Non-Protein Coding RNA 2086	RNA Gene	GC17P048646	0.885652542
LINC01617	Long Intergenic Non-Protein Coding RNA 1617	RNA Gene	GC08P073879	0.885652542
DACOR1	DNMT1-Associated Colon Cancer Repressed LncRNA 1	RNA Gene	GC15U902209	0.885652542
SEC63P1	SEC63 Homolog, Protein Translocation Regulator Pseudogene 1	Pseudogene	GC01P097545	0.885652542
FGF7P5	Fibroblast Growth Factor 7 Pseudogene	Pseudogene	GC09P043942	0.885652542
IBD19	Inflammatory Bowel Disease 19	Genetic Locus	GC00U934034	0.885652542
TLE1	TLE Family Member 1, Transcriptional Corepressor	Protein Coding	GC09M081583	0.884484291
IL17D	Interleukin 17D	Protein Coding	GC13P020702	0.884484291
GAS6	Growth Arrest Specific 6	Protein Coding	GC13M113820	0.878458023
SECTM1	Secreted And Transmembrane 1	Protein Coding	GC17M082321	0.87321049
PTER	Phosphotriesterase Related	Protein Coding	GC10P016436	0.87321049
RPN2	Ribophorin II	Protein Coding	GC20P037178	0.872685313
DCT	Dopachrome Tautomerase	Protein Coding	GC13M094436	0.872089982
PLAA	Phospholipase A2 Activating Protein	Protein Coding	GC09M026903	0.865291655
VPS35	VPS35 Retromer Complex Component	Protein Coding	GC16M046782	0.864057183
CAPZB	Capping Actin Protein Of Muscle Z-Line Subunit Beta	Protein Coding	GC01M019339	0.864057183
PDZK1	PDZ Domain Containing 1	Protein Coding	GC01M145670	0.864057183
MIR425	MicroRNA 425	RNA Gene	GC03M049502	0.861837864
PI4KB	Phosphatidylinositol 4-Kinase Beta	Protein Coding	GC01M151291	0.849226594
PEBP1	Phosphatidylethanolamine Binding Protein 1	Protein Coding	GC12P118135	0.844650745
RGN	Regucalcin	Protein Coding	GC0XP047212	0.841895461
MIR186	MicroRNA 186	RNA Gene	GC01M071067	0.834929705
C10orf67	Chromosome 10 Open Reading Frame	Protein Coding	GC10M023201	0.831769705
FMR1	FMRP Translational Regulator 1	Protein Coding	GC0XP147928	0.830639958
CLCN5	Chloride Voltage-Gated Channel 5	Protein Coding	GC0XP049922	0.830639958
NECTIN1	Nectin Cell Adhesion Molecule 1	Protein Coding	GC11M119624	0.828683138
ATP1A1	ATPase Na+/K+ Transporting Subunit Alpha 1	Protein Coding	GC01P116372	0.825189471
COPA	COPI Coat Complex Subunit Alpha	Protein Coding	GC01M160288	0.825189471
KIF21A	Kinesin Family Member 21A	Protein Coding	GC12M039293	0.825189471
FBLIM1	Filamin Binding LIM Protein 1	Protein Coding	GC01P015756	0.824599147
ARNT	Aryl Hydrocarbon Receptor Nuclear Translocator	Protein Coding	GC01M150809	0.81922847
C5	Complement C5	Protein Coding	GC09M120952	0.818004847
ETV6	ETS Variant Transcription Factor 6	Protein Coding	GC12P011649	0.807090342
ADM	Adrenomedullin	Protein Coding	GC11P010304	0.80576396
ENPP2	Ectonucleotide Pyrophosphatase/Phosphodiesterase 2	Protein Coding	GC08M119556	0.798341453
RAC2	Rac Family Small GTPase 2	Protein Coding	GC22M037227	0.797975361

MIR650	MicroRNA 650	RNA Gene	GC22P022822	0.797375917
CST3	Cystatin C	Protein Coding	GC20M023627	0.793305635
SKP2	S-Phase Kinase Associated Protein 2	Protein Coding	GC05P036151	0.782879353
SPRY2	Sprouty RTK Signaling Antagonist 2	Protein Coding	GC13M080335	0.779250979
SFTPA2	Surfactant Protein A2	Protein Coding	GC10M079651	0.778850436
VPS33B	VPS33B Late Endosome And Lysosome Associated	Protein Coding	GC15M090998	0.778850436
CD207	CD207 Molecule	Protein Coding	GC02M070830	0.775224507
IL17RE	Interleukin 17 Receptor E	Protein Coding	GC03P011100	0.771366715
ABCC6	ATP Binding Cassette Subfamily C Member 6	Protein Coding	GC16M016148	0.768469274
ACO1	Aconitase 1	Protein Coding	GC09P032374	0.768469274
MECOM	MDS1 And EVI1 Complex Locus	Protein Coding	GC03M169083	0.760270357
CD151	CD151 Molecule (Raph Blood Group)	Protein Coding	GC11P001093	0.760270357
VASP	Vasodilator Stimulated Phosphoprotein	Protein Coding	GC19P045507	0.749902844
PYCARD	PYD And CARD Domain Containing	Protein Coding	GC16M031201	0.749426842
AOC3	Amine Oxidase Copper Containing 3	Protein Coding	GC17P042851	0.747268021
MIR582	MicroRNA 582	RNA Gene	GC05M059703	0.74432683
RPS11	Ribosomal Protein S11	Protein Coding	GC19P049496	0.736805022
TARDBP	TAR DNA Binding Protein	Protein Coding	GC01P011013	0.735443592
CYP51A1	Cytochrome P450 Family 51 Subfamily A Member 1	Protein Coding	GC07M092112	0.735443592
MTM1	Myotubularin 1	Protein Coding	GC0XP150562	0.735443592
FTCD	Formimidoyltransferase	Protein Coding	GC21M048626	0.735443592
PPP2R2C	Protein Phosphatase 2 Regulatory Subunit Bgamma	Protein Coding	GC04M006322	0.735443592
RBBP4	RB Binding Protein 4, Chromatin Remodeling Factor	Protein Coding	GC01P032651	0.735443592
PPM1K	Protein Phosphatase, Mg2+/Mn2+ Dependent 1K	Protein Coding	GC04M088258	0.735443592
PUS1	Pseudouridine Synthase 1	Protein Coding	GC12P131929	0.735443592
ANKH	ANKH Inorganic Pyrophosphate Transport Regulator	Protein Coding	GC05M014706	0.735443592
SLC30A5	Solute Carrier Family 30 Member 5	Protein Coding	GC05P069093	0.735443592
ASGR2	Asialoglycoprotein Receptor 2	Protein Coding	GC17M007101	0.735443592
NPL	N-Acetylneuraminate Pyruvate Lyase	Protein Coding	GC01P182758	0.735443592
GOLM1	Golgi Membrane Protein 1	Protein Coding	GC09M086026	0.735443592
HOGA1	4-Hydroxy-2-Oxoglutarate Aldolase 1	Protein Coding	GC10P097585	0.735443592
ZNF354A	Zinc Finger Protein 354A	Protein Coding	GC05M178711	0.735443592
ISG20L2	Interferon Stimulated Exonuclease Gene 20 Like 2	Protein Coding	GC01M156723	0.735443592
MEPE	Matrix Extracellular	Protein Coding	GC04P087821	0.735443592
USP50	Ubiquitin Specific Peptidase 50	Protein Coding	GC15M050518	0.735443592
KAAG1	Kidney Associated Antigen 1	Protein Coding	GC06P024356	0.735443592
FRG2C	FSHD Region Gene 2 Family Member	Protein Coding	GC03P075646	0.735443592
MT-TF	Mitochondrially Encoded TRNA-Phe (UUU/C)	RNA Gene	GCMTP000580	0.735443592
MIR4741	MicroRNA 4741	RNA Gene	GC18P022933	0.735443592
TFPI2	Tissue Factor Pathway Inhibitor 2	Protein Coding	GC07M093885	0.731354535
CXCL16	C-X-C Motif Chemokine Ligand 16	Protein Coding	GC17M004733	0.730968595
BCHE	Butyrylcholinesterase	Protein Coding	GC03M165772	0.726804614
FADS1	Fatty Acid Desaturase 1	Protein Coding	GC11M061799	0.725098014
PON2	Paraoxonase 2	Protein Coding	GC07M095404	0.725098014
TAB1	TGF-Beta Activated Kinase 1 (MAP3K7) Binding Protein 1	Protein Coding	GC22P039413	0.725098014
PNPLA3	Patatin Like Phospholipase Domain Containing 3	Protein Coding	GC22P043923	0.725098014
MGAT3	Beta-1,4-Mannosyl-Glycoprotein 4-Beta-N-Acetylglucosaminyltransferase	Protein Coding	GC22P039447	0.725098014
IPMK	Inositol Polyphosphate Multikinase	Protein Coding	GC10M058191	0.725098014
PRICKLE2	Prickle Planar Cell Polarity Protein 2	Protein Coding	GC03M064079	0.725098014
ABCC4	ATP Binding Cassette Subfamily C Member 4	Protein Coding	GC13M095019	0.723860025

ASAH1	N-Acylsphingosine Amidohydrolase 1	Protein Coding	GC08M018055	0.720606029
TRIM58	Tripartite Motif Containing 58	Protein Coding	GC01P247857	0.720606029
SATB1	SATB Homeobox 1	Protein Coding	GC03M018364	0.720257282
ALDH9A1	Aldehyde Dehydrogenase 9 Family Member A1	Protein Coding	GC01M165671	0.711493254
SIRT6	Sirtuin 6	Protein Coding	GC19M004174	0.703799784
AXL	AXL Receptor Tyrosine Kinase	Protein Coding	GC19P041219	0.70034045
AUTS2	Activator Of Transcription And Developmental Regulator AUTS2	Protein Coding	GC07P069598	0.70034045
SIRT2	Sirtuin 2	Protein Coding	GC19M038878	0.697409809
SLC6A8	Solute Carrier Family 6 Member 8	Protein Coding	GC0XP153688	0.695463002
SOAT1	Sterol O-Acyltransferase 1	Protein Coding	GC01P179262	0.695463002
ALPK1	Alpha Kinase 1	Protein Coding	GC04P112285	0.695273817
PDLIM1	PDZ And LIM Domain 1	Protein Coding	GC10M095237	0.683643222
GALK1	Galactokinase 1	Protein Coding	GC17M075751	0.675229728
MARS1	Methionyl-TRNA Synthetase 1	Protein Coding	GC12P057476	0.675229728
ZNF281	Zinc Finger Protein 281	Protein Coding	GC01M200404	0.670016885
NPY	Neuropeptide Y	Protein Coding	GC07P024290	0.667351127
MAPT	Microtubule Associated Protein Tau	Protein Coding	GC17P045894	0.665638804
CACNA1C	Calcium Voltage-Gated Channel Subunit Alpha1 C	Protein Coding	GC12P001970	0.665638804
PGM1	Phosphoglucomutase 1	Protein Coding	GC01P063593	0.665638804
VRK1	VRK Serine/Threonine Kinase 1	Protein Coding	GC14P096797	0.665638804
ATP2B2	ATPase Plasma Membrane Ca2+ Transporting 2	Protein Coding	GC03M010324	0.665638804
EIF2AK2	Eukaryotic Translation Initiation Factor 2 Alpha Kinase 2	Protein Coding	GC02M037099	0.665638804
RPS6KA2	Ribosomal Protein S6 Kinase A2	Protein Coding	GC06M166409	0.665638804
CTSZ	Cathepsin Z	Protein Coding	GC20M058995	0.665638804
SPHK2	Sphingosine Kinase 2	Protein Coding	GC19P048619	0.665638804
LAMA5	Laminin Subunit Alpha 5	Protein Coding	GC20M062307	0.665638804
PLTP	Phospholipid Transfer Protein	Protein Coding	GC20M045898	0.665638804
TNFRSF12A	TNF Receptor Superfamily Member	Protein Coding	GC16P003018	0.665638804
IL31RA	Interleukin 31 Receptor A	Protein Coding	GC05P055840	0.665638804
RASGRF1	Ras Protein Specific Guanine Nucleotide Releasing Factor 1	Protein Coding	GC15M078959	0.665638804
CDC37	Cell Division Cycle 37, HSP90 Cochaperone	Protein Coding	GC19M010391	0.665638804
MPG	N-Methylpurine DNA Glycosylase	Protein Coding	GC16P006094	0.665638804
NLRP2	NLR Family Pyrin Domain Containing	Protein Coding	GC19P054953	0.665638804
SPRED1	Sprouty Related EVH1 Domain Containing 1	Protein Coding	GC15P038252	0.665638804
CREB5	CAMP Responsive Element Binding Protein 5	Protein Coding	GC07P028305	0.665638804
EPS8L2	EPS8 Like 2	Protein Coding	GC11P000694	0.665638804
NOX3	NADPH Oxidase 3	Protein Coding	GC06M155395	0.665638804
SULF2	Sulfatase 2	Protein Coding	GC20M047656	0.665638804
UBQLN4	Ubiquilin 4	Protein Coding	GC01M156033	0.665638804
FLRT1	Fibronectin Leucine Rich Transmembrane Protein 1	Protein Coding	GC11P064036	0.665638804
DBP	D-Box Binding PAR BZIP Transcription Factor	Protein Coding	GC19M048630	0.665638804
RAB13	RAB13, Member RAS Oncogene	Protein Coding	GC01M153981	0.665638804
STXBP4	Syntaxin Binding Protein 4	Protein Coding	GC17P054968	0.665638804
CEBPG	CCAAT Enhancer Binding Protein Gamma	Protein Coding	GC19P033373	0.665638804
CSMD2	CUB And Sushi Multiple Domains 2	Protein Coding	GC01M033513	0.665638804
PF4V1	Platelet Factor 4 Variant 1	Protein Coding	GC04P073853	0.665638804
RSPO3	R-Spondin 3	Protein Coding	GC06P127118	0.665638804
SNX7	Sorting Nexin 7	Protein Coding	GC01P098590	0.665638804
SLC10A4	Solute Carrier Family 10 Member 4	Protein Coding	GC04P048485	0.665638804
SYT4	Synaptotagmin 4	Protein Coding	GC18M043267	0.665638804
TUBD1	Tubulin Delta 1	Protein Coding	GC17M059859	0.665638804

ADAM30	ADAM Metallopeptidase Domain 30	Protein Coding	GC01M119893	0.665638804
MPPED2	Metallophosphoesterase Domain Containing 2	Protein Coding	GC11M030406	0.665638804
TMTC2	Transmembrane O-Mannosyltransferase Targeting Cadherins 2	Protein Coding	GC12P082687	0.665638804
GALNTL6	Polypeptide N-Acetylgalactosaminyltransferase Like 6	Protein Coding	GC04P171813	0.665638804
SLAIN2	SLAIN Motif Family Member 2	Protein Coding	GC04P048343	0.665638804
IZUMO1	Izumo Sperm-Egg Fusion 1	Protein Coding	GC19M048740	0.665638804
ZNF532	Zinc Finger Protein 532	Protein Coding	GC18P058862	0.665638804
PHACTR2	Phosphatase And Actin Regulator 2	Protein Coding	GC06P143536	0.665638804
RABEP2	Rabaptin, RAB GTPase Binding Effector Protein 2	Protein Coding	GC16M031447	0.665638804
OR2AT4	Olfactory Receptor Family 2 Subfamily AT Member 4	Protein Coding	GC11M075088	0.665638804
TRPT1	TRNA Phosphotransferase 1	Protein Coding	GC11M064223	0.665638804
MORC4	MORC Family CW-Type Zinc Finger 4	Protein Coding	GC0XM106813	0.665638804
ZNF432	Zinc Finger Protein 432	Protein Coding	GC19M052031	0.665638804
BABAM2	BRISC And BRCA1 A Complex Member 2	Protein Coding	GC02P027889	0.665638804
PRRC1	Proline Rich Coiled-Coil 1	Protein Coding	GC05P127517	0.665638804
RIMBP3	RIMS Binding Protein 3	Protein Coding	GC22M018605	0.665638804
LRRC61	Leucine Rich Repeat Containing 61	Protein Coding	GC07P150309	0.665638804
TM6SF2	Transmembrane 6 Superfamily Member	Protein Coding	GC19M019264	0.665638804
C1orf53	Chromosome 1 Open Reading Frame	Protein Coding	GC01P197871	0.665638804
C2orf74	Chromosome 2 Open Reading Frame	Protein Coding	GC02P061146	0.665638804
BORCS5	BLOC-1 Related Complex Subunit 5	Protein Coding	GC12P013393	0.665638804
SANBR	SANT And BTB Domain Regulator Of CSR	Protein Coding	GC02P061068	0.665638804
CIBAR2	CBY1 Interacting BAR Domain Containing 2	Protein Coding	GC16M085099	0.665638804
PPAN-P2RY11	PPAN-P2RY11 Readthrough	Protein Coding	GC19P010108	0.665638804
ZNF300P1	Zinc Finger Protein 300 Pseudogene 1	Pseudogene	GC05M150930	0.665638804
MIR6727	MicroRNA 6727	RNA Gene	GC01M003077	0.665638804
OR2AT1P	Olfactory Receptor Family 2 Subfamily AT Member 1 Pseudogene	Pseudogene	GC11M075131	0.665638804
OR7E116P	Olfactory Receptor Family 7 Subfamily E Member 116 Pseudogene	Pseudogene	GC09P090232	0.665638804
RPL35AP7	Ribosomal Protein L35a Pseudogene 7	Pseudogene	GC01M164925	0.665638804
RPL21P108	Ribosomal Protein L21 Pseudogene	Pseudogene	GC13M074377	0.665638804
RNU7-67P	RNA, U7 Small Nuclear 67 Pseudogene	Pseudogene	GC08P101307	0.665638804
RPL18P7	Ribosomal Protein L18 Pseudogene 7	Pseudogene	GC08P113377	0.665638804
IGF2BP1	Insulin Like Growth Factor 2 MRNA Binding Protein 1	Protein Coding	GC17P048997	0.656841934
LAMP3	Lysosomal Associated Membrane Protein 3	Protein Coding	GC03M183122	0.656406105
GLP1R	Glucagon Like Peptide 1 Receptor	Protein Coding	GC06P039048	0.645231605
EDNRB	Endothelin Receptor Type B	Protein Coding	GC13M077895	0.64477551
PRDM16	PR/SET Domain 16	Protein Coding	GC01P003068	0.637524724
GTF2H1	General Transcription Factor IIH Subunit 1	Protein Coding	GC11P018323	0.637524724
KEL	Kell Metallo-Endopeptidase (Kell Blood Group)	Protein Coding	GC07M142970	0.637524724
LMO2	LIM Domain Only 2	Protein Coding	GC11M033858	0.637524724
DUOX1	Dual Oxidase 1	Protein Coding	GC15P045129	0.637524724
XK	X-Linked Kx Blood Group	Protein Coding	GC0XP037685	0.637524724
PPP1R12C	Protein Phosphatase 1 Regulatory Subunit 12C	Protein Coding	GC19M055110	0.637524724
SSTR2	Somatostatin Receptor 2	Protein Coding	GC17P073165	0.627017736
AMPD2	Adenosine Monophosphate Deaminase	Protein Coding	GC01P109616	0.627017736
AMPD3	Adenosine Monophosphate Deaminase	Protein Coding	GC11P010309	0.627017736
TAOK1	TAO Kinase 1	Protein Coding	GC17P030465	0.627017736

IGSF3	Immunoglobulin Superfamily Member	Protein Coding	GC01M116574	0.627017736
CASP6	Caspase 6	Protein Coding	GC04M109688	0.626771092
CYP2A6	Cytochrome P450 Family 2 Subfamily A Member 6	Protein Coding	GC19M040843	0.626771092
CDH11	Cadherin 11	Protein Coding	GC16M064943	0.626771092
GRN	Granulin Precursor	Protein Coding	GC17P044345	0.626771092
TRD	T Cell Receptor Delta Locus	Protein Coding	GC14P026787	0.626771092
IQGAP2	IQ Motif Containing GTPase Activating Protein 2	Protein Coding	GC05P076403	0.626681149
MIR495	MicroRNA 495	RNA Gene	GC14P106827	0.626681149
TTK	TTK Protein Kinase	Protein Coding	GC06P080003	0.626250923
CENPE	Centromere Protein E	Protein Coding	GC04M103105	0.626250923
MINPP1	Multiple Inositol-Polyphosphate Phosphatase 1	Protein Coding	GC10P087504	0.626250923
CDC20	Cell Division Cycle 20	Protein Coding	GC01P043358	0.626250923
CETN2	Centrin 2	Protein Coding	GC0XM152827	0.626250923
NDC80	NDC80 Kinetochore Complex	Protein Coding	GC18P002571	0.626250923
MSLN	Mesothelin	Protein Coding	GC16P006129	0.626250923
CETN3	Centrin 3	Protein Coding	GC05M090392	0.626250923
CEP135	Centrosomal Protein 135	Protein Coding	GC04P055948	0.626250923
ZW10	Zw10 Kinetochore Protein	Protein Coding	GC11M113733	0.626250923
PPFIBP2	PPFIA Binding Protein 2	Protein Coding	GC11P007491	0.626250923
SASS6	SAS-6 Centriolar Assembly Protein	Protein Coding	GC01M100083	0.626250923
KIAA1217	KIAA1217	Protein Coding	GC10P023695	0.626250923
PHF7	PHD Finger Protein 7	Protein Coding	GC03P052411	0.626250923
TCFL5	Transcription Factor Like 5	Protein Coding	GC20M062841	0.626250923
MUC15	Mucin 15, Cell Surface Associated	Protein Coding	GC11M026537	0.626250923
MIS12	MIS12 Kinetochore Complex	Protein Coding	GC17P005486	0.626250923
SGO1	Shugoshin 1	Protein Coding	GC03M020159	0.626250923
MIR412	MicroRNA 412	RNA Gene	GC14P106821	0.626250923
MIR1185-1	MicroRNA 1185-1	RNA Gene	GC14P106794	0.626250923
MIR4729	MicroRNA 4729	RNA Gene	GC17P059366	0.626250923
MIR6086	MicroRNA 6086	RNA Gene	GC0XP013590	0.626250923
MAP3K14	Mitogen-Activated Protein Kinase Kinase Kinase 14	Protein Coding	GC17M045263	0.626183867
IQGAP1	IQ Motif Containing GTPase Activating Protein 1	Protein Coding	GC15P090388	0.615407348
B3GAT1	Beta-1,3-Glucuronyltransferase 1	Protein Coding	GC11M134378	0.590210915
TRIM5	Tripartite Motif Containing 5	Protein Coding	GC11M005796	0.590210915
OMP	Olfactory Marker Protein	Protein Coding	GC11P077102	0.590020239
CRYAB	Crystallin Alpha B	Protein Coding	GC11M111908	0.588745415
CD101	CD101 Molecule	Protein Coding	GC01P117001	0.588745415
CPT1A	Carnitine Palmitoyltransferase 1A	Protein Coding	GC11M068754	0.588150024
ACE2	Angiotensin Converting Enzyme 2	Protein Coding	GC0XM015494	0.588150024
PGK1	Phosphoglycerate Kinase 1	Protein Coding	GC0XP077949	0.588150024
ADAM9	ADAM Metallopeptidase Domain 9	Protein Coding	GC08P038996	0.588150024
AKR1C2	Aldo-Keto Reductase Family 1 Member C2	Protein Coding	GC10M004987	0.588150024
ARHGDIA	Rho GDP Dissociation Inhibitor Alpha	Protein Coding	GC17M081867	0.588150024
ITGB6	Integrin Subunit Beta 6	Protein Coding	GC02M160099	0.588150024
GRM8	Glutamate Metabotropic Receptor 8	Protein Coding	GC07M126438	0.588150024
CYP26B1	Cytochrome P450 Family 26 Subfamily B Member 1	Protein Coding	GC02M072129	0.588150024
PRLR	Prolactin Receptor	Protein Coding	GC05M035048	0.588150024
NR1H3	Nuclear Receptor Subfamily 1 Group H Member 3	Protein Coding	GC11P047248	0.588150024
MTNR1B	Melatonin Receptor 1B	Protein Coding	GC11P092969	0.588150024
OTC	Ornithine Transcarbamylase	Protein Coding	GC0XP038353	0.588150024
CES1	Carboxylesterase 1	Protein Coding	GC16M055836	0.588150024
FPR2	Formyl Peptide Receptor 2	Protein Coding	GC19P051752	0.588150024
GALNT2	Polypeptide N-Acetylgalactosaminyltransferase 2	Protein Coding	GC01P230057	0.588150024

ACADVL	Acyl-CoA Dehydrogenase Very Long Chain	Protein Coding	GC17P007219	0.588150024
CBR1	Carbonyl Reductase 1	Protein Coding	GC21P036069	0.588150024
MGLL	Monoglyceride Lipase	Protein Coding	GC03M127689	0.588150024
ANXA4	Annexin A4	Protein Coding	GC02P069644	0.588150024
HNF4G	Hepatocyte Nuclear Factor 4 Gamma	Protein Coding	GC08P075407	0.588150024
MTNR1A	Melatonin Receptor 1A	Protein Coding	GC04M186533	0.588150024
AKR1C1	Aldo-Keto Reductase Family 1 Member C1	Protein Coding	GC10P004963	0.588150024
GSTA1	Glutathione S-Transferase Alpha 1	Protein Coding	GC06M052791	0.588150024
HES1	Hes Family BHLH Transcription Factor	Protein Coding	GC03P194136	0.588150024
PANX1	Pannexin 1	Protein Coding	GC11P094128	0.588150024
TPP1	Tripeptidyl Peptidase 1	Protein Coding	GC11M006620	0.588150024
ADAM19	ADAM Metallopeptidase Domain 19	Protein Coding	GC05M157395	0.588150024
GSTA2	Glutathione S-Transferase Alpha 2	Protein Coding	GC06M052750	0.588150024
JUNB	JunB Proto-Oncogene, AP-1 Transcription Factor Subunit	Protein Coding	GC19P012791	0.588150024
CLDN11	Claudin 11	Protein Coding	GC03P170418	0.588150024
FLOT2	Flotillin 2	Protein Coding	GC17M031467	0.588150024
SULT1B1	Sulfotransferase Family 1B Member 1	Protein Coding	GC04M069721	0.588150024
TAGLN2	Transgelin 2	Protein Coding	GC01M159918	0.588150024
PPP2R5E	Protein Phosphatase 2 Regulatory Subunit B'Epsilon	Protein Coding	GC14M063371	0.588150024
ALDOC	Aldolase, Fructose-Bisphosphate C	Protein Coding	GC17M031463	0.588150024
FGL2	Fibrinogen Like 2	Protein Coding	GC07M077193	0.588150024
GSTK1	Glutathione S-Transferase Kappa 1	Protein Coding	GC07P146111	0.588150024
FN3K	Fructosamine 3 Kinase	Protein Coding	GC17P082735	0.588150024
HSPB2	Heat Shock Protein Family B (Small) Member 2	Protein Coding	GC11P111913	0.588150024
FIS1	Fission, Mitochondrial 1	Protein Coding	GC07M101239	0.588150024
HNRNPH3	Heterogeneous Nuclear Ribonucleoprotein H3	Protein Coding	GC10P068331	0.588150024
PANX2	Pannexin 2	Protein Coding	GC22P050170	0.588150024
PSG1	Pregnancy Specific Beta-1-Glycoprotein 1	Protein Coding	GC19M042866	0.588150024
RNF20	Ring Finger Protein 20	Protein Coding	GC09P101533	0.588150024
USO1	USO1 Vesicle Transport Factor	Protein Coding	GC04P075724	0.588150024
PPP3R2	Protein Phosphatase 3 Regulatory Subunit B, Beta	Protein Coding	GC09M101591	0.588150024
CLEC2D	C-Type Lectin Domain Family 2 Member D	Protein Coding	GC12P013303	0.588150024
CLEC4A	C-Type Lectin Domain Family 4 Member A	Protein Coding	GC12P013229	0.588150024
EXOSC1	Exosome Component 1	Protein Coding	GC10M097435	0.588150024
IBSP	Integrin Binding Sialoprotein	Protein Coding	GC04P087799	0.588150024
NOXO1	NADPH Oxidase Organizer 1	Protein Coding	GC16M003802	0.588150024
LSAMP	Limbic System Associated Membrane Protein	Protein Coding	GC03M115802	0.588150024
BEST2	Bestrophin 2	Protein Coding	GC19P012751	0.588150024
CNN3	Calponin 3	Protein Coding	GC01M094896	0.588150024
MAGI3	Membrane Associated Guanylate Kinase, WW And PDZ Domain	Protein Coding	GC01P113390	0.588150024
PIH1D1	PIH1 Domain Containing 1	Protein Coding	GC19M050461	0.588150024
DNPH1	2'-Deoxynucleoside 5'-Phosphate N-Hydrolase 1	Protein Coding	GC06M049364	0.588150024
ACTL8	Actin Like 8	Protein Coding	GC01P017756	0.588150024
DNAH12	Dynein Axonemal Heavy Chain 12	Protein Coding	GC03M057293	0.588150024
ZNF649	Zinc Finger Protein 649	Protein Coding	GC19M051889	0.588150024
ZG16	Zymogen Granule Protein 16	Protein Coding	GC16P029760	0.588150024
BEST4	Bestrophin 4	Protein Coding	GC01M044782	0.588150024
ENHO	Energy Homeostasis Associated	Protein Coding	GC09M034516	0.588150024
PROSER1	Proline And Serine Rich 1	Protein Coding	GC13M039009	0.588150024
H2BC3	H2B Clustered Histone 3	Protein Coding	GC06M026044	0.588150024

MIR595	MicroRNA 595	RNA Gene	GC07M158532	0.588150024
MIR1246	MicroRNA 1246	RNA Gene	GC02M176600	0.588150024
LOC400867	Uncharacterized LOC400867	RNA Gene	GC21M038877	0.588150024
IL6STP1	Interleukin 6 Signal Transducer Pseudogene 1	Pseudogene	GC17P015782	0.588150024
NM	Neutrophil Migration	Genetic Locus	GC07U990060	0.588150024
YWHAB	Tyrosine 3-Monooxygenase/Tryptophan 5-Monooxygenase Activation Protein	Protein Coding	GC20P044885	0.587813437
USP8	Ubiquitin Specific Peptidase 8	Protein Coding	GC15P050424	0.587813437
RNF31	Ring Finger Protein 31	Protein Coding	GC14P024146	0.587813437
SHARPIN	SHANK Associated RH Domain Interactor	Protein Coding	GC08M144098	0.587813437
MYLK	Myosin Light Chain Kinase	Protein Coding	GC03M123610	0.582571983
GPR15	G Protein-Coupled Receptor 15	Protein Coding	GC03P098531	0.576539636
GKN1	Gastrokine 1	Protein Coding	GC02P068974	0.575944304
PAH	Phenylalanine Hydroxylase	Protein Coding	GC12M102836	0.575755715
AQP3	Aquaporin 3 (Gill Blood Group)	Protein Coding	GC09M033431	0.573709428
MAP3K1	Mitogen-Activated Protein Kinase Kinase Kinase 1	Protein Coding	GC05P056815	0.567206383
PON3	Paraoxonase 3	Protein Coding	GC07M095359	0.567206383
ICA1	Islet Cell Autoantigen 1	Protein Coding	GC07M008119	0.567206383
SUV39H1	Suppressor Of Variegation 3-9 Homolog 1	Protein Coding	GC0XP048701	0.563602388
VCAN	Versican	Protein Coding	GC05P083471	0.560897589
CRTC1	CREB Regulated Transcription Coactivator 1	Protein Coding	GC19P027836	0.552892327
TTF2	Transcription Termination Factor 2	Protein Coding	GC01P117060	0.546809793
P2RY6	Pyrimidinergic Receptor P2Y6	Protein Coding	GC11P073264	0.544074416
AQP10	Aquaporin 10	Protein Coding	GC01P154321	0.544074416
EPAS1	Endothelial PAS Domain Protein 1	Protein Coding	GC02P046293	0.540391088
STK38	Serine/Threonine Kinase 38	Protein Coding	GC06M036493	0.535277545
SOCS5	Suppressor Of Cytokine Signaling 5	Protein Coding	GC02P046698	0.533778191
MIR802	MicroRNA 802	RNA Gene	GC21P035720	0.533778191
TRAF4	TNF Receptor Associated Factor 4	Protein Coding	GC17P030417	0.532936156
SREBF2	Sterol Regulatory Element Binding Transcription Factor 2	Protein Coding	GC22P041833	0.532936156
MYOD1	Myogenic Differentiation 1	Protein Coding	GC11P017741	0.532348931
MYOG	Myogenin	Protein Coding	GC01M203083	0.532348931
CD99	CD99 Molecule (Xg Blood Group)	Protein Coding	GC0XP002691	0.522029877
CD247	CD247 Molecule	Protein Coding	GC01M167399	0.511002719
ABCB7	ATP Binding Cassette Subfamily B Member 7	Protein Coding	GC0XM075053	0.510756075
RPS6KA3	Ribosomal Protein S6 Kinase A3	Protein Coding	GC0XM020149	0.4939785
SLC15A2	Solute Carrier Family 15 Member 2	Protein Coding	GC03P121894	0.4939785
GRAP2	GRB2 Related Adaptor Protein 2	Protein Coding	GC22P039901	0.491842538
NTRK3	Neurotrophic Receptor Tyrosine Kinase	Protein Coding	GC15M087859	0.483408749
RYR2	Ryanodine Receptor 2	Protein Coding	GC01P237042	0.483408749
CHRNA7	Cholinergic Receptor Nicotinic Alpha 7 Subunit	Protein Coding	GC15P031923	0.483408749
LTC4S	Leukotriene C4 Synthase	Protein Coding	GC05P179793	0.483408749
SLCO4A1	Solute Carrier Organic Anion Transporter Family Member 4A1	Protein Coding	GC20P063041	0.483408749
CCNE1	Cyclin E1	Protein Coding	GC19P029811	0.472135007
C13orf42	Chromosome 13 Open Reading Frame	Protein Coding	GC13M051119	0.472135007
UBR5	Ubiquitin Protein Ligase E3 Component N-Recognin 5	Protein Coding	GC08M102252	0.458861142
PPM1D	Protein Phosphatase, Mg2+/Mn2+ Dependent 1D	Protein Coding	GC17P060600	0.453410625
TSPO	Translocator Protein	Protein Coding	GC22P043151	0.452974826
TRAF5	TNF Receptor Associated Factor 5	Protein Coding	GC01P211326	0.438337713
LPO	Lactoperoxidase	Protein Coding	GC17P058218	0.429126859
PROK2	Prokineticin 2	Protein Coding	GC03M071771	0.423586428

RNF183	Ring Finger Protein 183	Protein Coding	GC09M113297	0.423586428
MIR19B1	MicroRNA 19b-1	RNA Gene	GC13P091469	0.423586428
CDH5	Cadherin 5	Protein Coding	GC16P066366	0.417763144
NAA15	N-Alpha-Acetyltransferase 15, NatA Auxiliary Subunit	Protein Coding	GC04P139301	0.417763144
TRIM22	Tripartite Motif Containing 22	Protein Coding	GC11P005689	0.417763144
KLRB1	Killer Cell Lectin Like Receptor B1	Protein Coding	GC12M016303	0.401075512
OLFM4	Olfactomedin 4	Protein Coding	GC13P053028	0.401075512
ZNF133	Zinc Finger Protein 133	Protein Coding	GC20P018288	0.401075512
OTUD1	OTU Deubiquitinase 1	Protein Coding	GC10P023439	0.401075512
FUT7	Fucosyltransferase 7	Protein Coding	GC09M137030	0.390916228
PNOC	Prepronociceptin	Protein Coding	GC08P028316	0.390505791
LIMK2	LIM Domain Kinase 2	Protein Coding	GC22P031212	0.390169203
COL5A1	Collagen Type V Alpha 1 Chain	Protein Coding	GC09P134641	0.390169203
KMT2A	Lysine Methyltransferase 2A	Protein Coding	GC11P118436	0.390169203
PARP2	Poly(ADP-Ribose) Polymerase 2	Protein Coding	GC14P020343	0.390169203
ACTR2	Actin Related Protein 2	Protein Coding	GC02P065227	0.390169203
CHD3	Chromodomain Helicase DNA Binding Protein 3	Protein Coding	GC17P009353	0.390169203
MBD3	Methyl-CpG Binding Domain Protein 3	Protein Coding	GC19M002366	0.390169203
SYNPO	Synaptopodin	Protein Coding	GC05P150601	0.390169203
KAT2B	Lysine Acetyltransferase 2B	Protein Coding	GC03P020043	0.384718716
ROCK2	Rho Associated Coiled-Coil Containing Protein Kinase 2	Protein Coding	GC02M011240	0.384718716
LTB4R	Leukotriene B4 Receptor	Protein Coding	GC14P024311	0.384718716
MIP	Major Intrinsic Protein Of Lens Fiber	Protein Coding	GC12M056449	0.384718716
PCSK1	Proprotein Convertase Subtilisin/Kexin Type 1	Protein Coding	GC05M096391	0.378895432
TNFSF13	TNF Superfamily Member 13	Protein Coding	GC17P007558	0.378895432
PSME3	Proteasome Activator Subunit 3	Protein Coding	GC17P042824	0.378895432
CD177	CD177 Molecule	Protein Coding	GC19P043353	0.378895432
EIF5B	Eukaryotic Translation Initiation Factor 5B	Protein Coding	GC02P099320	0.378895432
SLC1A1	Solute Carrier Family 1 Member 1	Protein Coding	GC09P004490	0.324022532
SLC16A9	Solute Carrier Family 16 Member 9	Protein Coding	GC10M059650	0.324022532
CNTN3	Contactin 3	Protein Coding	GC03M074232	0.324022532
AVIL	Advillin	Protein Coding	GC12M057793	0.324022532
NTF4	Neurotrophin 4	Protein Coding	GC19M049605	0.31918177
EPHB3	EPH Receptor B3	Protein Coding	GC03P184561	0.297266245
CCL28	C-C Motif Chemokine Ligand 28	Protein Coding	GC05M043356	0.29088378
P2RY2	Purinergic Receptor P2Y2	Protein Coding	GC11P073202	0.264563292
TPM3	Tropomyosin 3	Protein Coding	GC01M154127	0.264563292
FUT8	Fucosyltransferase 8	Protein Coding	GC14P065411	0.264563292
MNAT1	MNAT1 Component Of CDK Activating Kinase	Protein Coding	GC14P060734	0.264563292
MPST	Mercaptopyruvate Sulfurtransferase	Protein Coding	GC22P037019	0.264563292
CCDC22	Coiled-Coil Domain Containing 22	Protein Coding	GC0XP049468	0.264563292
BRWD1	Bromodomain And WD Repeat Domain Containing 1	Protein Coding	GC21M039184	0.264563292
TIGAR	TP53 Induced Glycolysis Regulatory Phosphatase	Protein Coding	GC12P013106	0.264563292
FCGBP	Fc Fragment Of IgG Binding Protein	Protein Coding	GC19M039863	0.264563292
WASHC2C	WASH Complex Subunit 2C	Protein Coding	GC10P045729	0.264563292
HDAC4	Histone Deacetylase 4	Protein Coding	GC02M239048	0.255766451
EGLN3	Egl-9 Family Hypoxia Inducible Factor	Protein Coding	GC14M033924	0.255766451
GUCY2D	Guanylate Cyclase 2D, Retinal	Protein Coding	GC17P008002	0.255766451
MYL9	Myosin Light Chain 9	Protein Coding	GC20P036541	0.255766451
MDM4	MDM4 Regulator Of P53	Protein Coding	GC01P204516	0.255766451
GFRA1	GDNF Family Receptor Alpha 1	Protein Coding	GC10M116056	0.255766451
MIR22HG	MIR22 Host Gene	RNA Gene	GC17M001791	0.255766451
EIF2B4	Eukaryotic Translation Initiation Factor 2B Subunit Delta	Protein Coding	GC02M027364	0.225942209
CLSPN	Claspin	Protein Coding	GC01M035720	0.225942209

ATF1	Activating Transcription Factor 1	Protein Coding	GC12P050763	0.22569558
MCM3	Minichromosome Maintenance Complex Component 3	Protein Coding	GC06M052264	0.22569558
FSTL1	Follistatin Like 1	Protein Coding	GC03M120392	0.22569558
UBD	Ubiquitin D	Protein Coding	GC06M048986	0.22569558
PRKG1	Protein Kinase CGMP-Dependent 1	Protein Coding	GC10P050991	0.187074497
BMPR2	Bone Morphogenetic Protein Receptor Type 2	Protein Coding	GC02P202376	0.187074497
ACAT1	Acetyl-CoA Acetyltransferase 1	Protein Coding	GC11P108121	0.187074497
DYRK1A	Dual Specificity Tyrosine Phosphorylation Regulated Kinase 1A	Protein Coding	GC21P037365	0.187074497
CYP11B1	Cytochrome P450 Family 11 Subfamily B Member 1	Protein Coding	GC08M142872	0.187074497
PLD1	Phospholipase D1	Protein Coding	GC03M171600	0.187074497
GRM3	Glutamate Metabotropic Receptor 3	Protein Coding	GC07P086643	0.187074497
CACNA1E	Calcium Voltage-Gated Channel Subunit Alpha1 E	Protein Coding	GC01P181317	0.187074497
ITGB5	Integrin Subunit Beta 5	Protein Coding	GC03M124761	0.187074497
WEE1	WEE1 G2 Checkpoint Kinase	Protein Coding	GC11P009573	0.187074497
UBE2D3	Ubiquitin Conjugating Enzyme E2 D3	Protein Coding	GC04M102794	0.187074497
B4GALT1	Beta-1,4-Galactosyltransferase 1	Protein Coding	GC09M033100	0.187074497
DOCK2	Dedicator Of Cytokinesis 2	Protein Coding	GC05P169637	0.187074497
SLC29A2	Solute Carrier Family 29 Member 2	Protein Coding	GC11M071767	0.187074497
PTGFR	Prostaglandin F Receptor	Protein Coding	GC01P078303	0.187074497
TNFRSF11A	TNF Receptor Superfamily Member	Protein Coding	GC18P062325	0.187074497
CACNA1I	Calcium Voltage-Gated Channel Subunit Alpha1 I	Protein Coding	GC22P039570	0.187074497
CNTN1	Contactin 1	Protein Coding	GC12P040692	0.187074497
CTRC	Chymotrypsin C	Protein Coding	GC01P015438	0.187074497
ENPEP	Glutamyl Aminopeptidase	Protein Coding	GC04P110365	0.187074497
INHBA	Inhibin Subunit Beta A	Protein Coding	GC07M041668	0.187074497
CHD2	Chromodomain Helicase DNA Binding Protein 2	Protein Coding	GC15P092900	0.187074497
KCNN3	Potassium Calcium-Activated Channel Subfamily N Member 3	Protein Coding	GC01M154697	0.187074497
RPSA	Ribosomal Protein SA	Protein Coding	GC03P039406	0.187074497
PSMA7	Proteasome 20S Subunit Alpha 7	Protein Coding	GC20M062136	0.187074497
SLCO2B1	Solute Carrier Organic Anion Transporter Family Member 2B1	Protein Coding	GC11P076426	0.187074497
UBB	Ubiquitin B	Protein Coding	GC17P016380	0.187074497
VAPA	VAMP Associated Protein A	Protein Coding	GC18P009904	0.187074497
VAV2	Vav Guanine Nucleotide Exchange Factor 2	Protein Coding	GC09M133761	0.187074497
AKR1B10	Aldo-Keto Reductase Family 1 Member B10	Protein Coding	GC07P134527	0.187074497
DPP6	Dipeptidyl Peptidase Like 6	Protein Coding	GC07P153748	0.187074497
CACNB1	Calcium Voltage-Gated Channel Auxiliary Subunit Beta 1	Protein Coding	GC17M039173	0.187074497
DLG1	Discs Large MAGUK Scaffold Protein	Protein Coding	GC03M197042	0.187074497
CYFIP2	Cytoplasmic FMR1 Interacting Protein	Protein Coding	GC05P157267	0.187074497
FABP1	Fatty Acid Binding Protein 1	Protein Coding	GC02M088122	0.187074497
ROBO2	Roundabout Guidance Receptor 2	Protein Coding	GC03P075955	0.187074497
SMYD2	SET And MYND Domain Containing 2	Protein Coding	GC01P214281	0.187074497
SLC8A3	Solute Carrier Family 8 Member A3	Protein Coding	GC14M070044	0.187074497
TSFM	Ts Translation Elongation Factor, Mitochondrial	Protein Coding	GC12P057778	0.187074497
SLC28A2	Solute Carrier Family 28 Member 2	Protein Coding	GC15P045252	0.187074497
SLIT1	Slit Guidance Ligand 1	Protein Coding	GC10M096998	0.187074497
PEX5	Peroxisomal Biogenesis Factor 5	Protein Coding	GC12P013209	0.187074497
ITGA11	Integrin Subunit Alpha 11	Protein Coding	GC15M068296	0.187074497
NRG3	Neuregulin 3	Protein Coding	GC10P086025	0.187074497
TIRAP	TIR Domain Containing Adaptor	Protein Coding	GC11P126284	0.187074497
SEPHS1	Selenophosphate Synthetase 1	Protein Coding	GC10M013317	0.187074497

ALDH1L2	Aldehyde Dehydrogenase 1 Family Member L2	Protein Coding	GC12M105019	0.187074497
CAPZA1	Capping Actin Protein Of Muscle Z-Line Subunit Alpha 1	Protein Coding	GC01P112619	0.187074497
CADPS	Calcium Dependent Secretion Activator	Protein Coding	GC03M062398	0.187074497
CALU	Calumenin	Protein Coding	GC07P128739	0.187074497
CPD	Carboxypeptidase D	Protein Coding	GC17P030378	0.187074497
ELMO1	Engulfment And Cell Motility 1	Protein Coding	GC07M036860	0.187074497
IGF2BP3	Insulin Like Growth Factor 2 MRNA Binding Protein 3	Protein Coding	GC07M023316	0.187074497
GPM6A	Glycoprotein M6A	Protein Coding	GC04M175632	0.187074497
CNN1	Calponin 1	Protein Coding	GC19P011539	0.187074497
FABP5	Fatty Acid Binding Protein 5	Protein Coding	GC08P081282	0.187074497
PDSS2	Decaprenyl Diphosphate Synthase Subunit 2	Protein Coding	GC06M107152	0.187074497
NSMAF	Neutral Sphingomyelinase Activation Associated Factor	Protein Coding	GC08M058569	0.187074497
MEGF10	Multiple EGF Like Domains 10	Protein Coding	GC05P127230	0.187074497
SORBS1	Sorbin And SH3 Domain Containing 1	Protein Coding	GC10M095311	0.187074497
TRAIP	TRAF Interacting Protein	Protein Coding	GC03M050155	0.187074497
SMARCD3	SWI/SNF Related, Matrix Associated, Actin Dependent Regulator Of Chromatin, Subfamily D, Member 3	Protein Coding	GC07M151238	0.187074497
SBF1	SET Binding Factor 1	Protein Coding	GC22M050445	0.187074497
CDK18	Cyclin Dependent Kinase 18	Protein Coding	GC01P205504	0.187074497
ARID5B	AT-Rich Interaction Domain 5B	Protein Coding	GC10P061901	0.187074497
EPB41L2	Erythrocyte Membrane Protein Band 4.1 Like 2	Protein Coding	GC06M130820	0.187074497
HNRNPM	Heterogeneous Nuclear Ribonucleoprotein M	Protein Coding	GC19P008444	0.187074497
PARVB	Parvin Beta	Protein Coding	GC22P043999	0.187074497
LRRC4C	Leucine Rich Repeat Containing 4C	Protein Coding	GC11M040113	0.187074497
PIK3AP1	Phosphoinositide-3-Kinase Adaptor Protein 1	Protein Coding	GC10M096593	0.187074497
STAB2	Stabilin 2	Protein Coding	GC12P103587	0.187074497
TNFRSF19	TNF Receptor Superfamily Member 19	Protein Coding	GC13P023570	0.187074497
PTPRR	Protein Tyrosine Phosphatase Receptor Type R	Protein Coding	GC12M070638	0.187074497
ZNF423	Zinc Finger Protein 423	Protein Coding	GC16M049487	0.187074497
ARHGAP24	Rho GTPase Activating Protein 24	Protein Coding	GC04P085475	0.187074497
CD200R1	CD200 Receptor 1	Protein Coding	GC03M112921	0.187074497
LRP1B	LDL Receptor Related Protein 1B	Protein Coding	GC02M140231	0.187074497
NCR2	Natural Cytotoxicity Triggering Receptor 2	Protein Coding	GC06P058494	0.187074497
SMYD1	SET And MYND Domain Containing 1	Protein Coding	GC02P088068	0.187074497
VPS26A	VPS26, Retromer Complex Component	Protein Coding	GC10P069123	0.187074497
TAF3	TATA-Box Binding Protein Associated Factor 3	Protein Coding	GC10P007860	0.187074497
CSMD1	CUB And Sushi Multiple Domains 1	Protein Coding	GC08M002953	0.187074497
DNER	Delta/Notch Like EGF Repeat	Protein Coding	GC02M229357	0.187074497
ELL2	Elongation Factor For RNA Polymerase II 2	Protein Coding	GC05M095885	0.187074497
GPR158	G Protein-Coupled Receptor 158	Protein Coding	GC10P025174	0.187074497
COL19A1	Collagen Type XIX Alpha 1 Chain	Protein Coding	GC06P069866	0.187074497
KCNIP4	Potassium Voltage-Gated Channel Interacting Protein 4	Protein Coding	GC04M020728	0.187074497
POU6F2	POU Class 6 Homeobox 2	Protein Coding	GC07P038977	0.187074497
LY75	Lymphocyte Antigen 75	Protein Coding	GC02M159803	0.187074497
PLCH1	Phospholipase C Eta 1	Protein Coding	GC03M155381	0.187074497
LRRTM4	Leucine Rich Repeat Transmembrane Neuronal 4	Protein Coding	GC02M076747	0.187074497
SOX13	SRY-Box Transcription Factor 13	Protein Coding	GC01P204074	0.187074497
USH2A	Usherin	Protein Coding	GC01M215622	0.187074497

SNRPC	Small Nuclear Ribonucleoprotein Polypeptide C	Protein Coding	GC06P058428	0.187074497
ADAMDEC1	ADAM Like Decysin 1	Protein Coding	GC08P024384	0.187074497
ABI3	ABI Family Member 3	Protein Coding	GC17P049210	0.187074497
ANO2	Anoctamin 2	Protein Coding	GC12M005532	0.187074497
CCDC40	Coiled-Coil Domain Containing 40	Protein Coding	GC17P080037	0.187074497
CCNG2	Cyclin G2	Protein Coding	GC04P077158	0.187074497
CRISP3	Cysteine Rich Secretory Protein 3	Protein Coding	GC06M049727	0.187074497
HCST	Hematopoietic Cell Signal Transducer	Protein Coding	GC19P041441	0.187074497
JAKMIP1	Janus Kinase And Microtubule Interacting Protein 1	Protein Coding	GC04M006026	0.187074497
MRPL28	Mitochondrial Ribosomal Protein L28	Protein Coding	GC16M000357	0.187074497
MPRIP	Myosin Phosphatase Rho Interacting Protein	Protein Coding	GC17P017042	0.187074497
MEF2B	Myocyte Enhancer Factor 2B	Protein Coding	GC19M019150	0.187074497
RALGAPA2	Ral GTPase Activating Protein Catalytic Subunit Alpha 2	Protein Coding	GC20M020374	0.187074497
RBM26	RNA Binding Motif Protein 26	Protein Coding	GC13M079311	0.187074497
RAB3C	RAB3C, Member RAS Oncogene	Protein Coding	GC05P058582	0.187074497
TMEM132D	Transmembrane Protein 132D	Protein Coding	GC12M129071	0.187074497
WARS1	Tryptophanyl-TRNA Synthetase 1	Protein Coding	GC14M100334	0.187074497
DERL3	Derlin 3	Protein Coding	GC22M023834	0.187074497
EYS	Eyes Shut Homolog	Protein Coding	GC06M063719	0.187074497
HOXD3	Homeobox D3	Protein Coding	GC02P176136	0.187074497
HUNK	Hormonally Up-Regulated Neu-Associated Kinase	Protein Coding	GC21P031873	0.187074497
GDE1	Glycerophosphodiester Phosphodiesterase 1	Protein Coding	GC16M019513	0.187074497
LINGO2	Leucine Rich Repeat And Ig Domain Containing 2	Protein Coding	GC09M027940	0.187074497
MDGA2	MAM Domain Containing Glycosylphosphatidylinositol Anchor 2	Protein Coding	GC14M046839	0.187074497
SYT9	Synaptotagmin 9	Protein Coding	GC11P007238	0.187074497
SCUBE1	Signal Peptide, CUB Domain And EGF Like Domain Containing 1	Protein Coding	GC22M043197	0.187074497
UBE2U	Ubiquitin Conjugating Enzyme E2 U	Protein Coding	GC01P064237	0.187074497
SYTL1	Synaptotagmin Like 1	Protein Coding	GC01P027399	0.187074497
CPNE8	Copine 8	Protein Coding	GC12M038646	0.187074497
IL31	Interleukin 31	Protein Coding	GC12M122173	0.187074497
PLD5	Phospholipase D Family Member 5	Protein Coding	GC01M242082	0.187074497
TRIM6	Tripartite Motif Containing 6	Protein Coding	GC11P005596	0.187074497
TBC1D14	TBC1 Domain Family Member 14	Protein Coding	GC04P006910	0.187074497
RTCB	RNA 2',3'-Cyclic Phosphate And 5'-OH Ligase	Protein Coding	GC22M032387	0.187074497
BEND7	BEN Domain Containing 7	Protein Coding	GC10M013392	0.187074497
CCDC93	Coiled-Coil Domain Containing 93	Protein Coding	GC02M117915	0.187074497
CNTNAP5	Contactin Associated Protein Family Member 5	Protein Coding	GC02P124025	0.187074497
ARMC3	Armadillo Repeat Containing 3	Protein Coding	GC10P022928	0.187074497
CEP131	Centrosomal Protein 131	Protein Coding	GC17M081189	0.187074497
DNAH10	Dynein Axonemal Heavy Chain 10	Protein Coding	GC12P123930	0.187074497
DNASE2B	Deoxyribonuclease 2 Beta	Protein Coding	GC01P084398	0.187074497
FRY	FRY Microtubule Binding Protein	Protein Coding	GC13P031865	0.187074497
MON2	MON2 Homolog, Regulator Of Endosome-To-Golgi Trafficking	Protein Coding	GC12P062466	0.187074497
SHISA6	Shisa Family Member 6	Protein Coding	GC17P011241	0.187074497
SLC38A10	Solute Carrier Family 38 Member 10	Protein Coding	GC17M081244	0.187074497
SGCZ	Sarcoglycan Zeta	Protein Coding	GC08M014089	0.187074497
SAMD14	Sterile Alpha Motif Domain Containing 14	Protein Coding	GC17M050110	0.187074497
VPS13C	Vacuolar Protein Sorting 13 Homolog	Protein Coding	GC15M061852	0.187074497
CEACAM4	CEA Cell Adhesion Molecule 4	Protein Coding	GC19M048493	0.187074497
JPH4	Junctophilin 4	Protein Coding	GC14M023568	0.187074497

EFCAB6	EF-Hand Calcium Binding Domain 6	Protein Coding	GC22M049732	0.187074497
UMODL1	Uromodulin Like 1	Protein Coding	GC21P042062	0.187074497
SNTG1	Syntrophin Gamma 1	Protein Coding	GC08P049909	0.187074497
ZNF804B	Zinc Finger Protein 804B	Protein Coding	GC07P088759	0.187074497
KMT5B	Lysine Methyltransferase 5B	Protein Coding	GC11M071879	0.187074497
TMEM182	Transmembrane Protein 182	Protein Coding	GC02P102744	0.187074497
ZNF385D	Zinc Finger Protein 385D	Protein Coding	GC03M021412	0.187074497
CEP170B	Centrosomal Protein 170B	Protein Coding	GC14P104865	0.187074497
RELL1	RELT Like 1	Protein Coding	GC04M037592	0.187074497
TMEM255B	Transmembrane Protein 255B	Protein Coding	GC13P113759	0.187074497
TTLL2	Tubulin Tyrosine Ligase Like 2	Protein Coding	GC06P167325	0.187074497
EMC9	ER Membrane Protein Complex	Protein Coding	GC14M024138	0.187074497
TENT2	Terminal Nucleotidyltransferase 2	Protein Coding	GC05P079613	0.187074497
DEFB103A	Defensin Beta 103A	Protein Coding	GC08P007881	0.187074497
TOPAZ1	Testis And Ovary Specific TOPAZ 1	Protein Coding	GC03P044283	0.187074497
REC114	REC114 Meiotic Recombination	Protein Coding	GC15P073443	0.187074497
TRIM6-TRIM34	TRIM6-TRIM34 Readthrough	Protein Coding	GC11P005597	0.187074497
CFAP92	Cilia And Flagella Associated Protein 92 (Putative)	Protein Coding	GC03M129463	0.187074497
NPSR1-AS1	NPSR1 Antisense RNA 1	RNA Gene	GC07M034387	0.187074497
CLRN1-AS1	CLRN1 Antisense RNA 1	RNA Gene	GC03P150852	0.187074497
LINC00469	Long Intergenic Non-Protein Coding RNA 469	RNA Gene	GC17M073750	0.187074497
MIR19B2	MicroRNA 19b-2	RNA Gene	GC0XM134207	0.187074497
LINC00649	Long Intergenic Non-Protein Coding RNA 649	RNA Gene	GC21P033915	0.187074497
SNORD48	Small Nucleolar RNA, C/D Box 48	RNA Gene	GC06P058330	0.187074497
ANKRD34C-AS1	ANKRD34C Antisense RNA 1	RNA Gene	GC15M079141	0.187074497
PRICKLE2-AS3	PRICKLE2 Antisense RNA 3	RNA Gene	GC03P064187	0.187074497
LACTB2-AS1	LACTB2 Antisense RNA 1	RNA Gene	GC08P070608	0.187074497
ZNF815P	Zinc Finger Protein 815, Pseudogene	Pseudogene	GC07P005823	0.187074497
LINC00331	Long Intergenic Non-Protein Coding RNA 331	RNA Gene	GC13M078787	0.187074497
LOC100506258	Uncharacterized LOC100506258	RNA Gene	GC11M007383	0.187074497
LINC00836	Long Intergenic Non-Protein Coding RNA 836	RNA Gene	GC10P025651	0.187074497
FILNC1	FOXO Induced Long Non-Coding	RNA Gene	GC06P139677	0.187074497
LINC02367	Long Intergenic Non-Protein Coding RNA 2367	RNA Gene	GC12P013660	0.187074497
LINC01342	Long Intergenic Non-Protein Coding RNA 1342	RNA Gene	GC01P002226	0.187074497
LOC101929710	Uncharacterized LOC101929710	RNA Gene	GC05P095962	0.187074497
IATPR	ITGB1 Adjacent Tumor Promoting LncRNA	RNA Gene	GC10M033073	0.187074497
NIPAL4-DT	NIPAL4 Divergent Transcript	RNA Gene	GC05M157364	0.187074497
ISCA2P1	Iron-Sulfur Cluster Assembly 2 Pseudogene 1	Pseudogene	GC22M026644	0.187074497
LRRTM4-	LRRTM4 Antisense RNA 1	RNA Gene	GC02P076986	0.187074497
PPIAP33	Peptidylprolyl Isomerase A Pseudogene 33	Pseudogene	GC09P007499	0.187074497
LOC101927484	Uncharacterized LOC101927484	RNA Gene	GC12M055023	0.187074497
IDDM15	Insulin Dependent Diabetes Mellitus 15	Genetic Locus	GC06U990067	0.187074497
