# Supplementary material for: Analysis of the Efficacy and Pharmacological Mechanisms of Action of Zhenren Yangzang Decoction on Ulcerative Colitis Using Meta-Analysis and Network Pharmacology
Source: Evid Based Complement Alternat Med. 2021 Dec 28;2021:4512755. doi: 10.1155/2021/4512755 (PMC8727130; doi:10.1155/2021/4512755)
Supplement: Supplementary Materials — Figure S1: Risk of bias graph. Figure S2: risk of bias summary. Figure S3: forest plot of comparison of serum cytokines. Figure S4: forest plot of comparison of the total syndrome score of TCM. Table S1: basic information on the active compounds in ZRYZD. Table S2: gene symbols and entrezID of active target genes. Table S3: compounds ranked by the degree in the network. Supplementary File 1: compounds of ZRYZD from TCMSP. Supplementary File 2: corresponding target genes of ZRYZD. Supplementary File 3: UC-related target genes. Supplementary File 4: GO functional enrichment analysis. Supplementary File 5: KEGG pathway enrichment analysis. Supplementary File 6: data of compound-target networks. Supplementary File 7: data of key compound-target networks. Supplementary File 8: data of PPI network. [file 4512755.f1.zip › 4512755.f1/Supplementary File 4 GO functional enrichment analysis.pdf]

Supplementary File 4 GO functional enrichment analysis

ONTOLOGY	ID	Description	GeneRatio	BgRatio	pvalue	p.adjust	qvalue	geneID	Count
BP	GO:0062197	cellular response to chemical stress	47/187	360/18866	2.91E-39	1.25E-35	5.66E-36	PTGS2/BCL2/JUN/CASP3/PPARG/RELA/AKT1/MAPK8/CDK1/HMOX1/CYP1B1/ALOX5/GSTP1/AKR1C3/DPEP1/CAT/AKR1B1/CDK2/CCNA2/EGFR/MMP2/MMP9/MAPK1/TP53/MDM2/PCNA/MCL1/MET/MAPK10/NCF1/MAPK3/BAD/SOD1/GSR/MMP3/FOS/HIF1A/CAV1/NOS3/HSPB1/MP	47
BP	GO:0032496	response to lipopolysaccharide	44/187	334/18866	7.08E-37	1.52E-33	6.89E-34	CD14/LBP/PTGS2/OPRM1/CASP9/CASP3/CASP8/PRKCA/NOS2/RELA/AKT1/MAPK8/CYP1A2/CYP1A1/ICAM1/SELE/GSTP1/SLPI/MAPK14/MAPK1/IL10RA/CDK4/NFKBIA/PTGES/PPARD/MAPK3/APOB/UGT1A1/FOS/GJA1/IL1B/CCL2/CXCL8/NOS3/THBD/SERPINE1/IL1A/MPO/CXCL11/C	44
BP	GO:0002237	response to molecule of bacterial origin	44/187	356/18866	1.20E-35	1.62E-32	7.37E-33	CD14/LBP/PTGS2/OPRM1/CASP9/CASP3/CASP8/PRKCA/NOS2/RELA/AKT1/MAPK8/CYP1A2/CYP1A1/ICAM1/SELE/GSTP1/SLPI/MAPK14/MAPK1/IL10RA/CDK4/NFKBIA/PTGES/PPARD/MAPK3/APOB/UGT1A1/FOS/GJA1/IL1B/CCL2/CXCL8/NOS3/THBD/SERPINE1/IL1A/MPO/CXCL11/C	44

BP	GO:0006979	response to oxidative stress	48/187	458/18866	1.51E-35	1.62E-32	7.37E-33	PTGS1/PTGS2/BCL2/JUN/CASP3/RELA/AKT1/MAPK8/STAT1/CDK1/HMOX1/CYP1B1/ALOX5/GSTP1/AKR1C3/DPEP1/CAT/CDK2/CCNA2/EGFR/MMP2/MMP9/MAPK1/TP53/MDM2/PCNA/MCL1/MET/NCF1/MAPK3/BAD/SOD1/GSR/MMP3/FOS/HIF1A/DUOX2/NOS3/HSPB1/COL1A1/MPO/NFE2L2/NQO1/PARP1/CH	48
BP	GO:0000302	response to reactive oxygen species	37/187	235/18866	6.81E-34	5.07E-31	2.30E-31	BCL2/JUN/CASP3/RELA/AKT1/MAPK8/STAT1/CDK1/HMOX1/CYP1B1/GSTP1/AKR1C3/DPEP1/CAT/CDK2/CCNA2/EGFR/MMP2/MMP9/MAPK1/MDM2/PCNA/MET/NCF1/MAPK3/BAD/SOD1/MMP3/FOS/NOS3/COL1A1/MPO/NFE2L2/NQO1/CHUK/EDN1/FABP1	37
BP	GO:0010038	response to metal ion	43/187	366/18866	7.09E-34	5.07E-31	2.30E-31	CD14/PTGS2/BCL2/CASP9/JUN/CASP3/CASP8/AKT1/MAPK8/CDK1/HMOX1/CYP1A2/CYP1A1/ICAM1/AKR1C3/DPEP1/CAT/EGFR/CCND1/MMP9/MAPK1/CDK4/MDM2/PCNA/CCNB1/PTGES/CA2/NCF1/MAPK3/BAD/SOD1/GOT1/FOS/HIF1A/HSPA5/CAV1/IL1A/NFE	43

BP	GO:0034599	cellular response to oxidative stress	40/187	310/18866	4.05E-33	2.49E-30	1.13E-30	BCL2/JUN/RELA/AKT1/MAPK8/CDK1/HMOX1/CYP1B1/ALOX5/GSTP1/AKR1C3/DPEP1/CAT/CDK2/CCNA2/EGFR/MMP2/MMP9/MAPK1/TP53/MDM2/PCNA/MCL1/MET/NCF1/MAPK3/SOD1/GSR/MMP3/FOS/HIF1A/NOS3/HSPB1/MPO/NFE2L2/NQO1/PARP1/CHUK/I	40
BP	GO:0042493	response to drug	41/187	397/18866	5.18E-30	2.78E-27	1.26E-27	PTGS2/SLC6A4/BCL2/CASP3/NOS2/PPARG/RELA/STAT1/CDK1/CYP3A4/CYP1A2/CYP1A1/ICAM1/NR1I2/DPEP1/CAT/EGFR/CCND1/CDKN1A/CDK4/TP53/TOP1/MDM2/CCNB1/BAD/SOD1/UGT1A1/ABCC1/STAT3/FOS/HSPA5/MYC/IL1B/COL1A1/NFE2L2/CHEK2/CH	41
BP	GO:0009636	response to toxic substance	33/187	250/18866	1.15E-27	5.48E-25	2.49E-25	PTGS1/PTGS2/SLC6A4/BCL2/BAX/PON1/CDK1/CYP1A1/CYP1B1/GSTP1/AHR/GSTM1/CAT/CDKN1A/MAPK1/CDK4/MDM2/CCNB1/MAPK3/SOD1/GSR/CES1/FOS/DUOX2/NOS3/MPO/ABCG2/NFE2L2/NQO1/CHUK/GSTA1/DRD2/FABP1	33

BP	GO:0072593	reactive oxygen species metabolic process	34/187	288/18866	7.64E-27	3.28E-24	1.49E-24	PTGS2/BCL2/NOS2/AKT1/CYP1A2/CYP1A1/ICAM1/CYP1B1/ALOX5/GSTP1/AKR1C3/CAT/MAPK14/EGFR/CDKN1A/TP53/IFNG/MMP8/NCF1/SOD1/STAT3/MMP3/HIF1A/CAV1/IL1B/DUOX2/NOS3/MPO/NFE2L2/NQO1/HK3/TRPV1/EDN1/INS	34
BP	GO:0070482	response to oxygen levels	38/187	396/18866	1.26E-26	4.91E-24	2.23E-24	PTGS2/SLC6A4/BCL2/CASP3/NOS2/PPARG/DPP4/AKT1/HMOX1/CYP1A1/ICAM1/CAT/PLAU/CCNA2/VEGFA/CDKN1A/MMP2/CDK4/TP53/MDM2/CCNB1/PPARD/BAD/PPARA/RAF1/HIF1A/CAV1/MYC/COL1A1/NFE2L2/E2F1/NPEPPS/HK2/[illegible]	38
BP	GO:0034614	cellular response to reactive oxygen species	28/187	170/18866	2.61E-26	9.34E-24	4.24E-24	JUN/RELA/AKT1/MAPK8/CDK1/CYP1B1/AKR1C3/DPEP1/CDK2/CCNA2/EGFR/MMP2/MMP9/MAPK1/MDM2/PCNA/MET/NCF1/MAPK3/SOD1/MMP3/FOS/NOS3/MPO/NFE2L2/NQO1/CHUK/FAB	28
BP	GO:0031667	response to nutrient levels	40/187	473/18866	6.35E-26	2.10E-23	9.52E-24	PTGS2/SLC6A4/OPRM1/BCL2/JUN/PON1/PPARG/RELA/AKT1/MAPK8/STAT1/HMOX1/CYP1A1/ICAM1/GSTP1/AKR1C3/CAT/EGFR/CCND1/CDKN1A/MAPK1/TP53/MDM2/TYR/PPARD/MAPK3/SOD1/HMGCR/UGT1A1/PPARA/HSPA5/COL1A1/MPO/NFE2L2/NQO1/CX	40

BP	GO:0048545	response to steroid hormone	34/187	346/18866	3.49E-24	1.07E-21	4.86E-22	NR3C2/PTGS2/BCL2/CASP9/CASP3/AR/RELA/ICAM1/GSTP1/AKR1C3/ESR1/RXRA/ESR2/EGFR/CCND1/CDKN1A/MDM2/PCNA/CA2/NR3C1/PPARD/BAD/UGT1A1/PPARA/GOT1/RXRB/FOS/CAV1/COL1A1/PARP1/CLDN4/SPP1/EDN1/ADM	34
BP	GO:0001666	response to hypoxia	34/187	359/18866	1.17E-23	3.36E-21	1.52E-21	PTGS2/SLC6A4/BCL2/CASP3/NOS2/DPP4/AKT1/HMOX1/CYP1A1/ICAM1/CAT/PLAU/CCNA2/VEGFA/MMP2/TP53/MDM2/CCNB1/PPARD/BAD/PPARA/RAF1/HIF1A/CAV1/MYC/NFE2L2/E2F1/NPEPPS/HK2/DRD2/EDN1/FABP1/UCP2/ADM	34
BP	GO:0036293	response to decreased oxygen levels	34/187	371/18866	3.44E-23	9.23E-21	4.19E-21	PTGS2/SLC6A4/BCL2/CASP3/NOS2/DPP4/AKT1/HMOX1/CYP1A1/ICAM1/CAT/PLAU/CCNA2/VEGFA/MMP2/TP53/MDM2/CCNB1/PPARD/BAD/PPARA/RAF1/HIF1A/CAV1/MYC/NFE2L2/E2F1/NPEPPS/HK2/DRD2/EDN1/FABP1/UCP2/ADM	34
BP	GO:0071241	cellular response to inorganic substance	27/187	221/18866	7.85E-22	1.98E-19	8.99E-20	PTGS2/JUN/AKT1/MAPK8/HMOX1/CYP1A2/CYP1A1/AKR1C3/DPEP1/CDK2/CCNA2/EGFR/MMP9/MAPK1/CCNB1/NCF1/MAPK3/BAD/SOD1/MMP3/FOS/HSPA5/NFE2L2/NQO1/PARP1/CHUK/F	27

BP	GO:0046686	response to cadmium ion	18/187	64/18866	8.98E-22	2.14E-19	9.72E-20	JUN/AKT1/MAPK8/CDK1/HMOX1/CYP1A2/AKR1C3/CAT/EGFR/MMP9/MAPK1/PCNA/NCF1/MAPK3/SOD1/GOT1/FOS/	18
BP	GO:2001233	regulation of apoptotic signaling pathway	34/187	413/18866	1.11E-21	2.50E-19	1.14E-19	PTGS2/BCL2/BAX/CASP8/AR/RELA/AKT1/MAPK8/HMOX1/ICAM1/GSTP1/GSK3B/BCL2L1/MMP9/RB1/TP53/MDM2/MCL1/IL4/BAD/SOD1/RAF1/HIF1A/CAV1/IL1B/NOS3/HSPB1/SERPINE1/IL1A/NFE2L2/PARP1/E2F1/	34
BP	GO:0062012	regulation of small molecule metabolic process	35/187	456/18866	2.61E-21	5.61E-19	2.55E-19	PTGS2/NOS2/PPARG/AKT1/AKR1C3/GSK3B/TP53/IFNG/IL4/PPARD/FASN/BAD/SOD1/APOB/HMGCR/PPARA/CES1/STAT3/EGF/ODC1/HIF1A/ACACA/CAV1/IL1B/NOS3/NQO1/PARP1/IGFBP3/IGF2/INS/FABP1/GCG/SCD/ADM/HPH1	35
BP	GO:0009612	response to mechanical stimulus	26/187	209/18866	2.95E-21	6.04E-19	2.74E-19	PTGS2/JUN/CASP8/PPARG/RELA/AKT1/MAPK8/STAT1/MAPK14/CHEK1/EGFR/NFKBIA/CCNB1/MAPK3/BAD/GOT1/FOS/RAF1/GJA1/IL1B/COL1A1/MPO/CXCL10/IRF1/DRD2/EDN1	26
BP	GO:2001234	negative regulation of apoptotic signaling pathway	27/187	233/18866	3.24E-21	6.32E-19	2.87E-19	PTGS2/BCL2/BAX/CASP8/AR/RELA/AKT1/HMOX1/ICAM1/GSTP1/GSK3B/BCL2L1/MMP9/RB1/MDM2/MCL1/IL4/RAF1/HIF1A/IL1B/NOS3/HSPB1/SERPINE1/IL1A/NFE2L2/BDNF/INS	27

BP	GO:0097305	response to alcohol	27/187	234/18866	3.63E-21	6.77E-19	3.07E-19	CD14/OPRM1/CASP8/PPARG/AKT1/CDK1/ICAM1/GSTP1/AHR/AKR1C3/CAT/CCND1/BCL2L1/CDKN1A/CDK4/HTR3A/BAD/SOD1/HMGCR/UGT1A1/CES1/STAT3/FOS/ACACA/NQO1/PARP1/DP	27
BP	GO:0033002	muscle cell proliferation	27/187	244/18866	1.10E-20	1.98E-18	8.97E-19	IL6R/PTGS2/JUN/PPARG/AKT1/STAT1/CDK1/HMOX1/GSTP1/RXRA/AKR1B1/MAPK14/EGFR/CDKN1A/MMP2/MMP9/MAPK1/MDM2/CCNB1/IFNG/PPARD/HMGCR/STAT3/GJA1/NOS3/IGFBP	27
BP	GO:0071216	cellular response to biotic stimulus	27/187	246/18866	1.37E-20	2.35E-18	1.07E-18	CD14/LBP/PRKCA/NOS2/RELA/AKT1/MAPK8/ICAM1/GSTP1/MAPK14/GSK3B/MAPK1/CDK4/TP53/NFKBIA/PPARD/MAPK3/HSPA5/IL1B/CCL2/CXCL8/NOS3/SERPINE1/IL1A/CXCL11/CXCL	27
BP	GO:0071496	cellular response to external stimulus	30/187	326/18866	1.43E-20	2.35E-18	1.07E-18	PTGS2/BCL2/JUN/CASP8/PPARG/AKT1/MAPK8/HMOX1/ICAM1/VCAM1/GSTP1/AKR1C3/CHEK1/EGFR/CDKN1A/MAPK1/TP53/MDM2/MAPK3/BAD/PPARA/GOT1/FOS/HSPA5/GJA1/IL1B/COL1A1/NFE2L2/IRF1/LPL	30
BP	GO:2000377	regulation of reactive oxygen species metabolic process	25/187	200/18866	1.60E-20	2.54E-18	1.15E-18	PTGS2/BCL2/AKT1/ICAM1/CYP1B1/ALOX5/GSTP1/AKR1C3/MAPK14/EGFR/CDKN1A/TP53/IFNG/MMP8/SOD1/STAT3/MMP3/HIF1A/CAV1/IL1B/NFE2L2/HK2/TRPV1/	25

BP	GO:0032355	response to estradiol	22/187	141/18866	2.56E-20	3.93E-18	1.78E-18	PTGS2/SLC6A4/CASP9/CASP3/CASP8/CYP1A2/GSTP1/ESR1/CAT/ESR2/CCNA2/EGFR/CCND1/PCNA/BAD/APOB/CYP19A1/UGT1A1/STAT3/GJA1/COL1A1/NQO1	22
BP	GO:1903409	reactive oxygen species biosynthetic process	21/187	128/18866	6.51E-20	9.64E-18	4.38E-18	PTGS2/NOS2/AKT1/CYP1A2/CYP1A1/ICAM1/CYP1B1/ALOX5/IFNG/MMP8/SOD1/STAT3/CAV1/IL1B/DUOX2/NOS3/MPO/NQO1/TRPV1/EDN1/IN	21
BP	GO:0009314	response to radiation	33/187	447/18866	1.35E-19	1.93E-17	8.78E-18	PTGS2/OPRM1/BCL2/BAX/CASP9/CASP3/RELA/AKT1/MAPK8/ICAM1/CAT/MAPK14/CHEK1/EGFR/CCND1/BCL2L1/CDKN1A/TP53/MDM2/PCNA/TYR/MAPK10/HMGCR/FOS/HIF1A/HSPA5/MYC/THBD/PARP1/CHEK2/CXCL10/DRD3/HRH1	33
BP	GO:0071214	cellular response to abiotic stimulus	29/187	331/18866	2.56E-19	3.37E-17	1.53E-17	PTGS2/BAX/CASP9/CASP3/CASP8/AKT1/MAPK8/AKR1B1/MAPK14/CHEK1/EGFR/BCL2L1/CDKN1A/TP53/MDM2/PCNA/MAPK10/MAPK3/BAD/GOT1/HSPA5/MYC/GJA1/IL1B/COL1A1/PARP1/CHEK2/IRF1/TRPV1	29
BP	GO:0104004	cellular response to environmental stimulus	29/187	331/18866	2.56E-19	3.37E-17	1.53E-17	PTGS2/BAX/CASP9/CASP3/CASP8/AKT1/MAPK8/AKR1B1/MAPK14/CHEK1/EGFR/BCL2L1/CDKN1A/TP53/MDM2/PCNA/MAPK10/MAPK3/BAD/GOT1/HSPA5/MYC/GJA1/IL1B/COL1A1/PARP1/CHEK2/IRF1/TRPV1	29

BP	GO:1901654	response to ketone	24/187	200/18866	2.59E-19	3.37E-17	1.53E-17	CASP9/AR/PPARG/RELA/AKT1/ICAM1/AHR/AKR1C3/AKR1B1/EGFR/CCND1/BCL2L1/CDKN1A/CDK4/PCNA/NR3C1/BAD/FOS/ACACA/CAV1/PARP1/CLDN4/SPP1/ED	24
BP	GO:0050727	regulation of inflammatory response	32/187	425/18866	2.85E-19	3.60E-17	1.63E-17	TNFAIP6/LBP/PTGS2/NOS2/PPARG/RELA/SELE/ALOX5/GSTP1/ESR1/MAPK14/EGFR/MMP9/RB1/NFKBIA/IL2RA/IFNG/IL4/XIAP/MMP8/PPARD/SOD1/CYP19A1/PPARA/ABCC1/STAT3/MMP3/IL1B/PTGER3/SERPINE1/	32
BP	GO:0071276	cellular response to cadmium ion	14/187	38/18866	4.24E-19	5.20E-17	2.36E-17	JUN/AKT1/MAPK8/HMOX1/CYP1A2/AKR1C3/EGFR/MMP9/MAPK1/NCF1/MAPK3/SOD1/FOS/C	14
BP	GO:0097191	extrinsic apoptotic signaling pathway	25/187	230/18866	5.06E-19	6.04E-17	2.74E-17	IL6R/BCL2/BAX/CASP9/CASP3/CASP8/AR/RELA/AKT1/HMOX1/ICAM1/GSTP1/GSK3B/BCL2L1/MCL1/IFNG/IL4/BAD/RAF1/CAV1/IL1B/NOS3/SERPINE1/IL1A/ERBB3	25
BP	GO:0071222	cellular response to lipopolysaccharide	24/187	208/18866	6.57E-19	7.62E-17	3.46E-17	CD14/LBP/PRKCA/NOS2/RELA/AKT1/MAPK8/ICAM1/GSTP1/MAPK14/MAPK1/CDK4/NFKBIA/PPARD/MAPK3/IL1B/CCL2/CXCL8/NOS3/SERPINE1/IL1A/CXCL11/CXCL2/CXCL10	24

BP	GO:0071248	cellular response to metal ion	23/187	193/18866	1.77E-18	2.00E-16	9.06E-17	PTGS2/JUN/AKT1/MAPK8/HMOX1/CYP1A2/CYP1A1/AKR1C3/DPEP1/EGFR/MMP9/MAPK1/CCNB1/NCF1/MAPK3/SOD1/FOS/HSPA5/NFE2L2/NQO1/PARP1/CHUK/EDN	23
BP	GO:0007584	response to nutrient	22/187	171/18866	1.90E-18	2.09E-16	9.50E-17	PTGS2/SLC6A4/PPARG/RELA/STAT1/HMOX1/CYP1A1/GSTP1/AKR1C3/CAT/EGFR/CCND1/MDM2/TYR/PPARD/HMGCR/UGT1A1/COL1A1/NQO1/CXCL10/SPP1/LPL	22
BP	GO:0071219	cellular response to molecule of bacterial origin	24/187	222/18866	3.05E-18	3.23E-16	1.46E-16	CD14/LBP/PRKCA/NOS2/RELA/AKT1/MAPK8/ICAM1/GSTP1/MAPK14/MAPK1/CDK4/NFKBIA/PPARD/MAPK3/IL1B/CCL2/CXCL8/NOS3/SERPINE1/IL1A/CXCL11/CXCL2/CXCL10	24
BP	GO:0008202	steroid metabolic process	28/187	332/18866	3.08E-18	3.23E-16	1.46E-16	PON1/CYP3A4/CYP1A2/CYP1A1/NR1I2/CYP1B1/AKR1C3/ESR1/RXRA/CAT/AKR1B1/IFNG/IL4/PPARD/FASN/SOD1/APOB/HMGCR/CYP19A1/UGT1A1/AKR1C1/CES1/SOAT1/ACACA/IL1B/SPP1/SCD/ADM	28
BP	GO:0006631	fatty acid metabolic process	30/187	396/18866	3.53E-18	3.60E-16	1.64E-16	PTGS1/PTGS2/PON1/PPARG/AKT1/CYP3A4/CYP1A2/CYP1A1/CYP1B1/ALOX5/GSTP1/GSTM1/AKR1C3/MAPK14/PTGES/PPARD/MAPK3/FASN/PPARA/AKR1C1/CES1/ACACA/CAV1/IL1B/GSTA1/EDN1/LPL/INS/FABP	30

BP	GO:0044706	multi-multicellular organism process	24/187	226/18866	4.64E-18	4.63E-16	2.10E-16	PTGS2/SLC6A4/BCL2/AR/AKT1/CYP1A1/ESR1/RXRA/AKR1B1/VEGFA/MMP2/MMP9/MAPK1/PPARD/MAPK3/SOD1/FOS/GJA1/IL1B/THBD/CLDN4/SPP1/EDN1/ADM	24
BP	GO:0043434	response to peptide hormone	31/187	447/18866	1.15E-17	1.12E-15	5.10E-16	PTGS2/PPARG/RELA/AKT1/STAT1/ICAM1/GSTP1/CAT/GSK3B/CCNA2/CDK4/MDM2/CA2/PPARA/GOT1/STAT3/CAV1/GJA1/IL1B/PRKCB/COL1A1/NFE2L2/PARP1/CHUK/IGF2/TRPV1/EDN1/LPL/INS/GCG/ADM	31
BP	GO:0007568	aging	27/187	319/18866	1.18E-17	1.13E-15	5.12E-16	PTGS2/BCL2/CASP9/JUN/RELA/AKT1/CDK1/CYP1A1/ICAM1/CAT/MAPK14/CHEK1/CDKN1A/MAPK1/TP53/MAPK3/SOD1/HMGCR/STAT3/FOS/SERPINE1/MPO/NFE2L2/NQO1/CHEK2/EDN1/	27
BP	GO:0071466	cellular response to xenobiotic stimulus	19/187	123/18866	1.32E-17	1.24E-15	5.61E-16	PTGS1/CYP3A4/CYP1A2/CYP1A1/NR1I2/CYP1B1/GSTP1/AHR/GSTM1/DPEP1/RB1/PCNA/UGT1A1/AKR1C1/CES1/NQO1/E2F1/GSTA1/GSTA2	19
BP	GO:0009410	response to xenobiotic stimulus	19/187	130/18866	3.88E-17	3.54E-15	1.61E-15	PTGS1/CYP3A4/CYP1A2/CYP1A1/NR1I2/CYP1B1/GSTP1/AHR/GSTM1/DPEP1/RB1/PCNA/UGT1A1/AKR1C1/CES1/NQO1/E2F1/GSTA1/GSTA2	19

BP	GO:0048660	regulation of smooth muscle cell proliferation	21/187	173/18866	3.98E-17	3.56E-15	1.62E-15	IL6R/PTGS2/JUN/PPARG/AKT1/STAT1/HMOX1/GSTP1/AKR1B1/EGFR/CDKN1A/MMP2/MMP9/MDM2/IFNG/PPARD/HMGCR/GJA1/NOS3/IGF	21
BP	GO:0048659	smooth muscle cell proliferation	21/187	175/18866	5.06E-17	4.34E-15	1.97E-15	IL6R/PTGS2/JUN/PPARG/AKT1/STAT1/HMOX1/GSTP1/AKR1B1/EGFR/CDKN1A/MMP2/MMP9/MDM2/IFNG/PPARD/HMGCR/GJA1/NOS3/IGF	21
BP	GO:1901568	fatty acid derivative metabolic process	21/187	175/18866	5.06E-17	4.34E-15	1.97E-15	PTGS1/PTGS2/PON1/CYP1A2/CYP1A1/CYP1B1/ALOX5/GSTP1/GSTM1/AKR1C3/DPEP1/PTGES/MAPK3/FASN/ABCC1/AKR1C1/ACACA/IL1B/GSTA1/EDN1/SCD	21
BP	GO:0062013	positive regulation of small molecule metabolic process	20/187	154/18866	6.03E-17	5.07E-15	2.30E-15	PTGS2/NOS2/PPARG/AKT1/IFNG/IL4/PPARD/PPARA/CES1/STAT3/EGF/HIF1A/IL1B/NOS3/IGF2/INS/FABP1/GCG/ADM/	20
BP	GO:0009411	response to UV	19/187	140/18866	1.62E-16	1.33E-14	6.05E-15	PTGS2/BCL2/BAX/CASP9/CASP3/RELA/AKT1/MAPK8/CAT/CHEK1/EGFR/CCND1/CDKN1A/TP53/MDM2/PCNA/TYR/MYC/PARP1	19
BP	GO:0031960	response to corticosteroid	20/187	164/18866	2.12E-16	1.71E-14	7.78E-15	PTGS2/BCL2/CASP9/CASP3/ICAM1/GSTP1/AKR1C3/EGFR/CCND1/CDKN1A/PCNA/NR3C1/BAD/UGT1A1/GOT1/FOS/COL1A1/PARP1/EDN1/AD	20
BP	GO:0006690	icosanoid metabolic process	18/187	122/18866	2.28E-16	1.81E-14	8.21E-15	PTGS1/PTGS2/PON1/CYP1A2/CYP1A1/CYP1B1/ALOX5/GSTP1/GSTM1/AKR1C3/DPEP1/PTGES/MAPK3/ABCC1/AKR1C1/IL1B/GSTA1/EDN1	18

BP	GO:0070997	neuron death	27/187	360/18866	2.51E-16	1.96E-14	8.89E-15	BCL2/BAX/CASP9/JUN/CASP3/CASP8/AKT1/HMOX1/GSK3B/BCL2L1/RB1/TP53/MCL1/IFNG/XIAP/BAD/SOD1/PPARA/STAT3/FOS/HIF1A/HSPA5/CCL2/NQO1/PARP1/ERBB3/BDNF	27
BP	GO:1901653	cellular response to peptide	28/187	398/18866	3.49E-16	2.67E-14	1.21E-14	PPARG/RELA/AKT1/STAT1/ICAM1/VCAM1/GSTP1/AKR1B1/GSK3B/CCNA2/CDK4/TP53/MDM2/CA2/ABCC1/GOT1/STAT3/CAV1/GJA1/IL1B/PRKCB/NFE2L2/PARP1/IGF2/EDN1/LPL/INS/GCG	28
BP	GO:0018209	peptidyl-serine modification	26/187	333/18866	3.55E-16	2.68E-14	1.21E-14	PTGS2/BCL2/BAX/PRKCA/IKBKB/AKT1/MAPK8/CDK1/MAPK14/GSK3B/CDK2/EGFR/VEGFA/MAPK1/TOP1/CCNB1/IFNG/MAPK3/RAF1/CAV1/PRKCB/PARP1/CHEK2/CHUK/BDNF/GCG	26
BP	GO:2001237	negative regulation of extrinsic apoptotic signaling pathway	17/187	107/18866	4.40E-16	3.21E-14	1.46E-14	BCL2/CASP8/AR/RELA/AKT1/HMOX1/ICAM1/GSTP1/GSK3B/BCL2L1/MCL1/IL4/RAF1/IL1B/NOS3/SERPINE1/IL1A	17
BP	GO:0048511	rhythmic process	25/187	305/18866	4.41E-16	3.21E-14	1.46E-14	SLC6A4/OPRM1/JUN/CASP3/NOS2/PPARG/MAPK8/CDK1/AHR/ESR1/GSK3B/EGFR/CDK4/TP53/TOP1/PCNA/TOP2A/MAPK10/PPARA/HSPA5/NOS3/SERPINE1/CLDN4/DRD2/BDNF	25

BP	GO:0007565	female pregnancy	21/187	196/18866	5.26E-16	3.76E-14	1.71E-14	PTGS2/BCL2/AR/AKT1/ESR1/RXRA/AKR1B1/VEGFA/MMP2/MMP9/MAPK1/PPARD/MAPK3/SOD1/FOS/GJA1/IL1B/THBD/CLDN4/SPP1/ADM	21
BP	GO:0018105	peptidyl-serine phosphorylation	25/187	310/18866	6.46E-16	4.49E-14	2.04E-14	PTGS2/BCL2/BAX/PRKCA/IKBKB/AKT1/MAPK8/CDK1/MAPK14/GSK3B/CDK2/EGFR/VEGFA/MAPK1/TOP1/CCNB1/IFNG/MAPK3/RAF1/CAV1/PRKCB/CHEK2/CHUK/BDNF/GCG	25
BP	GO:0048608	reproductive structure development	29/187	443/18866	6.59E-16	4.49E-14	2.04E-14	PTGS2/BCL2/BAX/CASP3/CASP8/AR/PPARG/AKT1/ICAM1/AKR1C3/ESR1/RXRA/MAPK14/EGFR/VEGFA/CCND1/BCL2L1/MAPK1/PPARD/MAPK3/SOD1/CYP19A1/HIF1A/HSPA5/GJA1/NOS3/SPP1/IGF2/ADM	29
BP	GO:0048732	gland development	29/187	443/18866	6.59E-16	4.49E-14	2.04E-14	BCL2/BAX/JUN/AR/RELA/AKT1/HMOX1/CYP1A1/ESR1/RXRA/EGFR/VEGFA/CCND1/MAPK1/PCNA/TYR/MET/MAPK3/FASN/SOD1/CYP19A1/UGT1A1/EGF/RAF1/HIF1A/CAV1/IGF2/HK2/DRD	29
BP	GO:0060326	cell chemotaxis	25/187	311/18866	6.96E-16	4.67E-14	2.12E-14	IL6R/LBP/DPP4/ALOX5/GSTP1/DPEP1/MAPK14/VEGFA/MAPK1/IL4/MET/KDR/MAPK3/CYP19A1/ABCC1/IL1B/CCL2/CXCL8/HSPB1/SERPINE1/CXCL11/CXCL2/CXCL10/EDN1/HRH1	25

BP	GO:0002526	acute inflammatory response	17/187	111/18866	8.35E-16	5.43E-14	2.46E-14	IL6R/LBP/PTGS2/OPRM1/PPARG/ICAM1/GSTP1/IL4/PTGES/UGT1A1/STAT3/F3/IL1B/PTGER3/IL1A/TRPV1/INS	17
BP	GO:0061458	reproductive system development	29/187	447/18866	8.35E-16	5.43E-14	2.46E-14	PTGS2/BCL2/BAX/CASP3/CASP8/AR/PPARG/AKT1/ICAM1/AKR1C3/ESR1/RXRA/MAPK14/EGFR/VEGFA/CCND1/BCL2L1/MAPK1/PPARD/MAPK3/SOD1/CYP19A1/HIF1A/HSPA5/GJA1/NOS3/SPP1/IGF2/ADM	29
BP	GO:0009416	response to light stimulus	25/187	319/18866	1.26E-15	8.08E-14	3.67E-14	PTGS2/BCL2/BAX/CASP9/CASP3/RELA/AKT1/MAPK8/CAT/CHEK1/EGFR/CCND1/CDKN1A/TP53/MDM2/PCNA/TYR/MAPK10/HMGCR/FOS/HIF1A/MYC/PARP1/DRD2/	25
BP	GO:0051090	regulation of DNA-binding transcription factor activity	29/187	455/18866	1.33E-15	8.39E-14	3.81E-14	JUN/AR/PPARG/RELA/IKBKB/AKT1/MAPK8/HMOX1/ICAM1/CYP1B1/ESR1/CAT/ESR2/MAPK14/VEGFA/MAPK1/RB1/NFKBIA/CD40LG/MAPK10/MAPK3/STAT3/FOS/CAV1/IL1B/PRKCB/CH	29
BP	GO:0034612	response to tumor necrosis factor	25/187	320/18866	1.36E-15	8.43E-14	3.83E-14	CD14/PTGS2/CASP3/CASP8/RELA/IKBKB/AKT1/STAT1/ICAM1/SELE/GSTP1/MAPK14/MAPK1/TP53/NFKBIA/CD40LG/MAPK3/APOB/CCL2/CXCL8/COL1A1/NFE2L2/CHUK/TRPV1/EDN1	25

BP	GO:0097193	intrinsic apoptotic signaling pathway	24/187	290/18866	1.43E-15	8.79E-14	3.99E-14	PTGS2/BCL2/BAX/CASP9/CASP3/AKT1/HMOX1/CYP1B1/BCL2L1/CDKN1A/MMP9/TP53/MDM2/MCL1/BAD/SOD1/HIF1A/CAV1/HSPB1/NFE2L2/PARP1/CHEK2/E2F1/IN	24
BP	GO:0051222	positive regulation of protein transport	26/187	354/18866	1.55E-15	9.39E-14	4.26E-14	CD14/PTGS2/BCL2/CASP8/ACHE/MAPK8/CDK1/MAPK14/GSK3B/EGFR/MAPK1/TP53/MDM2/ERBB2/IFNG/PPARD/BAD/HIF1A/GJA1/IL1B/IL1A/E2F1/NPEPPS/DRD2/INS	26
BP	GO:0003018	vascular process in circulatory system	20/187	190/18866	3.82E-15	2.28E-13	1.03E-13	PTGS2/ADRA1B/SLC6A4/AKT1/HMOX1/ICAM1/ADRA2A/EGFR/VEGFA/PPARD/SOD1/HMGCR/CAV1/GJA1/NOS3/ABCG2/EDN1/INS/ADM/HR	20
BP	GO:0035296	regulation of tube diameter	18/187	143/18866	4.06E-15	2.35E-13	1.07E-13	PTGS2/ADRA1B/SLC6A4/AKT1/HMOX1/ICAM1/ADRA2A/EGFR/PPARD/SOD1/HMGCR/CAV1/GJA1/NOS3/EDN1/INS/ADM/HRH1	18
BP	GO:0097746	regulation of blood vessel diameter	18/187	143/18866	4.06E-15	2.35E-13	1.07E-13	PTGS2/ADRA1B/SLC6A4/AKT1/HMOX1/ICAM1/ADRA2A/EGFR/PPARD/SOD1/HMGCR/CAV1/GJA1/NOS3/EDN1/INS/ADM/HRH1	18
BP	GO:1904951	positive regulation of establishment of protein localization	26/187	370/18866	4.47E-15	2.56E-13	1.16E-13	CD14/PTGS2/BCL2/CASP8/ACHE/MAPK8/CDK1/MAPK14/GSK3B/EGFR/MAPK1/TP53/MDM2/ERBB2/IFNG/PPARD/BAD/HIF1A/GJA1/IL1B/IL1A/E2F1/NPEPPS/DRD2/INS	26

BP	GO:0035150	regulation of tube size	18/187	144/18866	4.60E-15	2.60E-13	1.18E-13	PTGS2/ADRA1B/SLC6A4/AKT1/HMOX1/ICAM1/ADRA2A/EGFR/PPARD/SOD1/HMGCR/CAV1/GJA1/NOS3/EDN1/INS/ADM/HRH1	18
BP	GO:0042542	response to hydrogen peroxide	18/187	146/18866	5.89E-15	3.28E-13	1.49E-13	BCL2/JUN/CASP3/RELA/STAT1/CDK1/HMOX1/CYP1B1/CAT/MDM2/PCNA/MET/BAD/SOD1/COL1A1/NFE2L2/NQO1/FA	18
BP	GO:2000379	positive regulation of reactive oxygen species metabolic process	16/187	106/18866	7.69E-15	4.23E-13	1.92E-13	PTGS2/AKT1/ICAM1/GSTP1/AKR1C3/MAPK14/EGFR/CDKN1A/TP53/IFNG/MMP8/SOD1/IL1B/NFE2L2/TRPV1/EDN1	16
BP	GO:0050673	epithelial cell proliferation	28/187	453/18866	9.33E-15	5.07E-13	2.30E-13	BAX/JUN/PRKCA/AR/PPARG/AKT1/STAT1/HMOX1/ALOX5/ESR1/EGFR/VEGFA/CCND1/MAPK1/RB1/ERBB2/KDR/PPARD/BAD/STAT3/HIF1A/CAV1/MYC/F3/GJA1/CCL2/IGFBP3/IGF2	28
BP	GO:0006066	alcohol metabolic process	26/187	385/18866	1.15E-14	6.16E-13	2.80E-13	PON1/CYP3A4/CYP1A2/CYP1A1/CYP1B1/AKR1C3/RXRA/CAT/AKR1B1/IFNG/IL4/PPARD/FASN/SOD1/APOB/PLB1/HMGCR/AKR1C1/GOT1/CES1/SOAT1/ACACA/IL1B/ADH1B/SCD/HRH1	26
BP	GO:0070542	response to fatty acid	15/187	90/18866	1.19E-14	6.30E-13	2.86E-13	PTGS2/PON1/PPARG/AKT1/AKR1C3/CAT/CDK4/CCNB1/BAD/APOB/ACACA/E2F1/EDN1/LPL/	15

BP	GO:1901214	regulation of neuron death	24/187	321/18866	1.39E-14	7.29E-13	3.31E-13	BCL2/BAX/CASP9/JUN/CASP3/CASP8/AKT1/HMOX1/GSK3B/BCL2L1/TP53/MCL1/IFNG/BAD/SOD1/PPARA/STAT3/FOS/HIF1A/CCL2/NQO1/PARP1/ERBB3/BDNF	24
BP	GO:0071453	cellular response to oxygen levels	21/187	235/18866	2.06E-14	1.07E-12	4.84E-13	PTGS2/BCL2/PPARG/AKT1/HMOX1/ICAM1/CCNA2/VEGFA/TP53/MDM2/CCNB1/PPARD/BAD/HIF1A/CAV1/MYC/NFE2L2/E2F1/NPEPPS/EDN1	21
BP	GO:0045787	positive regulation of cell cycle	26/187	396/18866	2.23E-14	1.14E-12	5.18E-13	SLC6A4/BAX/PRKCA/AKT1/CDK1/CYP1A1/CDK2/CHEK1/EGFR/CCND1/CDKN1A/RB1/CDK4/TP53/MDM2/PCNA/CCNB1/EGF/IL1B/IL1A/CHEK2/E2F1/IGF2/DRD2/EDN	26
BP	GO:0090068	positive regulation of cell cycle process	23/187	302/18866	3.46E-14	1.75E-12	7.94E-13	BAX/AKT1/CDK1/CYP1A1/CDK2/EGFR/CCND1/CDKN1A/RB1/CDK4/TP53/MDM2/PCNA/CCNB1/EGF/IL1B/IL1A/CHEK2/E2F1/IGF2/DRD2/EDN1/	23
BP	GO:2001236	regulation of extrinsic apoptotic signaling pathway	18/187	162/18866	3.73E-14	1.86E-12	8.46E-13	BCL2/CASP8/AR/RELA/AKT1/HMOX1/ICAM1/GSTP1/GSK3B/BCL2L1/MCL1/IL4/RAF1/CAV1/IL1B/NOS3/SERPINE1/IL1A	18
BP	GO:0006805	xenobiotic metabolic process	16/187	118/18866	4.38E-14	2.14E-12	9.70E-13	PTGS1/CYP3A4/CYP1A2/CYP1A1/NR1I2/CYP1B1/GSTP1/AHR/GSTM1/DPEP1/UGT1A1/AKR1C1/CES1/NQO1/GSTA1/G	16
BP	GO:0033559	unsaturated fatty acid metabolic process	16/187	118/18866	4.38E-14	2.14E-12	9.70E-13	PTGS1/PTGS2/CYP1A2/CYP1A1/CYP1B1/ALOX5/GSTP1/GSTM1/AKR1C3/PTGES/MAPK3/AKR1C1/IL1B/GSTA1/EDN1/	16

BP	GO:0006367	transcription initiation from RNA polymerase II promoter	19/187	189/18866	4.49E-14	2.16E-12	9.82E-13	NR3C2/BAX/AR/PPARG/CDK1/NR1I2/ESR1/RXRA/ESR2/CCND1/CDKN1A/CDK4/TP53/CCNB1/NR3C1/PPARD/PPARA/RXRB/RUNX2	19
BP	GO:0001819	positive regulation of cytokine production	27/187	447/18866	5.11E-14	2.44E-12	1.11E-12	IL6R/CD14/LBP/PTGS2/CASP8/RELA/STAT1/CYP1B1/ADRA2A/MAPK14/IFNG/IL4/CD40LG/MMP8/SOD1/STAT3/HIF1A/F3/IL1B/HSPB1/SERPINE1/IL1A/CHUK/IRF1/DRD2/LPL/INS	27
BP	GO:0006352	DNA-templated transcription, initiation	21/187	249/18866	6.51E-14	3.07E-12	1.39E-12	NR3C2/BAX/JUN/AR/PPARG/CDK1/NR1I2/ESR1/RXRA/ESR2/CCND1/CDKN1A/CDK4/TP53/CCNB1/NR3C1/PPARD/MAPK3/PPARA/RXRB/RUNX	21
BP	GO:0045471	response to ethanol	16/187	122/18866	7.48E-14	3.49E-12	1.58E-12	CD14/OPRM1/CASP8/CDK1/ICAM1/GSTP1/CAT/CCND1/HTR3A/BAD/SOD1/HMGCR/UGT1A1/STAT3/NQO1/DRD2	16
BP	GO:0048145	regulation of fibroblast proliferation	14/187	83/18866	7.88E-14	3.64E-12	1.65E-12	BAX/JUN/PPARG/GSTP1/ESR1/CCNA2/EGFR/CDKN1A/CDK4/TP53/CCNB1/FOSL2/MYC/E2F1	14
BP	GO:0048144	fibroblast proliferation	14/187	84/18866	9.37E-14	4.28E-12	1.94E-12	BAX/JUN/PPARG/GSTP1/ESR1/CCNA2/EGFR/CDKN1A/CDK4/TP53/CCNB1/FOSL2/MYC/E2F1	14
BP	GO:0051384	response to glucocorticoid	17/187	147/18866	1.01E-13	4.56E-12	2.07E-12	PTGS2/BCL2/CASP9/CASP3/ICAM1/GSTP1/EGFR/CCND1/CDKN1A/PCNA/NR3C1/BAD/UGT1A1/GOT1/FOS/EDN1/ADM	17
BP	GO:0097756	negative regulation of blood vessel diameter	14/187	86/18866	1.32E-13	5.89E-12	2.67E-12	PTGS2/ADRA1B/SLC6A4/AKT1/ICAM1/ADRA2A/EGFR/HMGCR/CAV1/GJA1/EDN1/INS/ADM/H	14

BP	GO:0052547	regulation of peptidase activity	27/187	466/18866	1.39E-13	6.14E-12	2.79E-12	PTGS2/BAX/CASP9/CASP8/PPARG/AKT1/SLPI/DPEP1/MAPK14/VEGFA/MMP9/MDM2/BIRC5/XIAP/MAPK3/BAD/STAT3/RAF1/CAV1/MYC/F3/SERPINE1/CLDN4/CTSD/PCOLCE/SERPINB2/FA	27
BP	GO:0050678	regulation of epithelial cell proliferation	25/187	395/18866	1.68E-13	7.36E-12	3.34E-12	BAX/JUN/PRKCA/AR/PPARG/AKT1/STAT1/HMOX1/ALOX5/EGFR/VEGFA/CCND1/RB1/ERBB2/KDR/PPARD/BAD/STAT3/HIF1A/CAV1/MYC/F3/GJA1/CCL2/IGF2	25
BP	GO:0030595	leukocyte chemotaxis	20/187	232/18866	1.76E-13	7.62E-12	3.46E-12	IL6R/LBP/DPP4/ALOX5/DPEP1/MAPK14/VEGFA/MAPK1/IL4/MAPK3/CYP19A1/IL1B/CCL2/CXCL8/SERPINE1/CXCL11/CXCL2/CXCL10/EDN1/H	20
BP	GO:0009266	response to temperature stimulus	20/187	233/18866	1.91E-13	8.18E-12	3.71E-12	CD14/PTGS2/CASP8/PPARG/AKT1/HMOX1/GSK3B/CDKN1A/MAPK1/NFKBIA/MAPK3/SOD1/FOS/NOS3/IL1A/CXCL10/TRPV1/LPL/UCP2/AD	20
BP	GO:1904018	positive regulation of vasculature development	20/187	235/18866	2.24E-13	9.52E-12	4.32E-12	IL6R/PTGS2/PRKCA/HMOX1/CYP1B1/VEGFA/KDR/STAT3/HIF1A/F3/IL1B/CXCL8/PRKCB/NOS3/HSPB1/SERPINE1/IL1A/NFE2L2/HK2/ADM	20
BP	GO:0045766	positive regulation of angiogenesis	19/187	208/18866	2.57E-13	1.07E-11	4.86E-12	PTGS2/PRKCA/HMOX1/CYP1B1/VEGFA/KDR/STAT3/HIF1A/F3/IL1B/CXCL8/PRKCB/NOS3/HSPB1/SERPINE1/IL1A/NFE2L2/HK2/ADM	19

BP	GO:0071456	cellular response to hypoxia	19/187	208/18866	2.57E-13	1.07E-11	4.86E-12	PTGS2/BCL2/AKT1/HMOX1/ICAM1/CCNA2/VEGFA/TP53/MDM2/CCNB1/PPARD/BAD/HIF1A/MYC/NFE2L2/E2F1/NPEPPS/EDN1/FABP1	19
BP	GO:0051098	regulation of binding	24/187	367/18866	2.63E-13	1.09E-11	4.93E-12	BAX/JUN/PON1/PPARG/AKT1/MAPK8/HMOX1/SLPI/GSK3B/CDKN1A/MMP9/RB1/NFKBIA/IFNG/MET/MMP8/MAPK3/PPARA/EGF/CAV1/PARP1/E2F1/BDNF/GCG	24
BP	GO:0033273	response to vitamin	14/187	91/18866	2.97E-13	1.21E-11	5.51E-12	PTGS2/PPARG/RELA/CYP1A1/GSTP1/CAT/EGFR/CCND1/MDM2/TYR/PPARD/COL1A1/CXCL10/SPP1	14
BP	GO:2000045	regulation of G1/S transition of mitotic cell cycle	18/187	185/18866	3.79E-13	1.54E-11	6.97E-12	BCL2/BAX/AKT1/CDK1/CYP1A1/CDK2/EGFR/CCND1/CDKN1A/RB1/CDK4/TP53/MDM2/PCNA/CCNB1/CCL2/CHEK2/E2	18
BP	GO:0038034	signal transduction in absence of ligand	13/187	75/18866	4.34E-13	1.72E-11	7.82E-12	BCL2/BAX/CASP9/CASP3/AKT1/GSK3B/BCL2L1/MCL1/IL4/BAD/IL1B/IL1A/ERBB3	13
BP	GO:0097192	extrinsic apoptotic signaling pathway in absence of ligand	13/187	75/18866	4.34E-13	1.72E-11	7.82E-12	BCL2/BAX/CASP9/CASP3/AKT1/GSK3B/BCL2L1/MCL1/IL4/BAD/IL1B/IL1A/ERBB3	13
BP	GO:1990748	cellular detoxification	15/187	114/18866	4.40E-13	1.73E-11	7.87E-12	PTGS1/PTGS2/GSTP1/GSTM1/CAT/SOD1/GSR/DUOX2/NOS3/MPO/ABCG2/NFE2L2/NQO1/GSTA1/FABP1	15
BP	GO:0007623	circadian rhythm	19/187	218/18866	6.00E-13	2.32E-11	1.05E-11	SLC6A4/JUN/NOS2/PPARG/MAPK8/CDK1/AHR/GSK3B/EGFR/CDK4/TP53/TOP1/TOP2A/MAPK10/PPARA/SERPINE1/CLDN4/DRD2/BDNF	19

BP	GO:0036294	cellular response to decreased oxygen levels	19/187	218/18866	6.00E-13	2.32E-11	1.05E-11	PTGS2/BCL2/AKT1/HMOX1/ICAM1/CCNA2/VEGFA/TP53/MDM2/CCNB1/PPARD/BAD/HIF1A/MYC/NFE2L2/E2F1/NPEPPS/EDN1/FABP1	19
BP	GO:2001242	regulation of intrinsic apoptotic signaling pathway	17/187	166/18866	7.59E-13	2.91E-11	1.32E-11	PTGS2/BCL2/BAX/AKT1/BCL2L1/MMP9/TP53/MDM2/MCL1/BAD/SOD1/HIF1A/CAV1/HSPB1/NFE2L2/PARP1/INS	17
BP	GO:0097529	myeloid leukocyte migration	19/187	222/18866	8.31E-13	3.16E-11	1.43E-11	IL6R/LBP/DPP4/DPEP1/MAPK14/VEGFA/MAPK1/IL4/MAPK3/CYP19A1/IL1B/CCL2/CXCL8/SERPINE1/CXCL11/CXCL2/CXCL10/EDN1/HRH1	19
BP	GO:0006809	nitric oxide biosynthetic process	13/187	79/18866	8.74E-13	3.26E-11	1.48E-11	PTGS2/NOS2/AKT1/ICAM1/CYP1B1/IFNG/MMP8/CAV1/IL1B/NOS3/NQO1/TRPV1/EDN1	13
BP	GO:0071260	cellular response to mechanical stimulus	13/187	79/18866	8.74E-13	3.26E-11	1.48E-11	PTGS2/CASP8/AKT1/MAPK8/CHEK1/EGFR/MAPK3/BAD/GOT1/GJA1/IL1B/COL1A1/IRF1	13
BP	GO:0031668	cellular response to extracellular stimulus	20/187	253/18866	8.93E-13	3.31E-11	1.50E-11	BCL2/JUN/PPARG/MAPK8/HMOX1/ICAM1/VCAM1/GSTP1/AKR1C3/CDKN1A/MAPK1/TP53/MDM2/MAPK3/PPARA/FOS/HSPA5/COL1A1/NFE2L	20
BP	GO:0016125	sterol metabolic process	17/187	169/18866	1.02E-12	3.74E-11	1.70E-11	PON1/CYP3A4/CYP1A2/CYP1B1/RXRA/CAT/IL4/PPARD/FASN/SOD1/APOB/HMGCR/CYP19A1/CES1/SOAT1/ACACA/SC	17
BP	GO:0010212	response to ionizing radiation	16/187	144/18866	1.03E-12	3.77E-11	1.71E-11	BCL2/BAX/CASP3/ICAM1/MAPK14/CCND1/BCL2L1/CDKN1A/TP53/MDM2/HSPA5/MYC/THBD/PARP1/CHEK2/CXCL	16

BP	GO:0120254	olefinic compound metabolic process	15/187	121/18866	1.07E-12	3.87E-11	1.76E-11	PTGS1/PTGS2/CYP1A2/CYP1A1/CYP1B1/ALOX5/GSTP1/GSTM1/AKR1C3/PTGES/MAPK3/CYP19A1/AKR1C1/GSTA1/A	15
BP	GO:0019216	regulation of lipid metabolic process	25/187	431/18866	1.17E-12	4.20E-11	1.91E-11	PTGS2/PPARG/AKT1/CYP1A1/AKR1C3/RXRA/ADRA2A/RB1/CDK4/IFNG/PPARD/FASN/SOD1/APOB/HMGCR/UGT1A1/PPARA/CES1/ACACA/CAV1/IL1B/INS/FABP1/S	25
BP	GO:0097237	cellular response to toxic substance	15/187	122/18866	1.21E-12	4.31E-11	1.96E-11	PTGS1/PTGS2/GSTP1/GSTM1/CAT/SOD1/GSR/DUOX2/NOS3/MPO/ABCG2/NFE2L2/NQO1/GSTA1/FABP1	15
BP	GO:0050920	regulation of chemotaxis	19/187	229/18866	1.45E-12	5.09E-11	2.31E-11	IL6R/LBP/DPP4/GSTP1/MAPK14/VEGFA/MAPK1/IL4/MET/KDR/MAPK3/CYP19A1/F3/CCL2/CXCL8/HSPB1/SERPINE1/CXCL10/EDN1	19
BP	GO:0048661	positive regulation of smooth muscle cell proliferation	14/187	103/18866	1.72E-12	6.02E-11	2.73E-11	IL6R/PTGS2/JUN/AKT1/STAT1/HMOX1/AKR1B1/EGFR/MMP2/MMP9/MDM2/HMGCR/GJA1/ED	14
BP	GO:0014074	response to purine-containing compound	16/187	149/18866	1.76E-12	6.11E-11	2.77E-11	PTGS2/SLC6A4/PPARG/RELA/STAT1/AHR/TYR/SOD1/FOS/HSPA5/IL1B/DUOX2/THBD/COL1A1/TRPV1/CACNA1S	16
BP	GO:0071375	cellular response to peptide hormone stimulus	22/187	330/18866	1.89E-12	6.50E-11	2.95E-11	PPARG/RELA/AKT1/STAT1/GSTP1/GSK3B/CCNA2/CDK4/MDM2/CA2/GOT1/STAT3/CAV1/IL1B/PRKCB/NFE2L2/PARP1/IGF2/EDN1/LPL/INS/G	22
BP	GO:2000134	negative regulation of G1/S transition of mitotic cell cycle	15/187	126/18866	1.96E-12	6.60E-11	3.00E-11	BCL2/BAX/CDK1/CDK2/CCND1/CDKN1A/RB1/CDK4/TP53/MDM2/PCNA/CCNB1/CCL2/CHEK2/	15

BP	GO:0045765	regulation of angiogenesis	24/187	403/18866	1.97E-12	6.60E-11	3.00E-11	PTGS2/PRKCA/PPARG/STAT1/HMOX1/CYP1B1/ALOX5/VEGFA/ERBB2/KDR/STAT3/HIF1A/F3/IL1B/CXCL8/PRKCB/NOS3/HSPB1/SERPINE1/IL1A/NFE2L2/CXCL10/HK	24
BP	GO:0043279	response to alkaloid	14/187	104/18866	1.98E-12	6.60E-11	3.00E-11	OPRM1/CASP3/PPARG/RELA/ICAM1/CCNA2/BCL2L1/MDM2/CASP7/HTR3A/HSPA5/DRD2/TRPV1/CACNA1S	14
BP	GO:0046209	nitric oxide metabolic process	13/187	84/18866	1.98E-12	6.60E-11	3.00E-11	PTGS2/NOS2/AKT1/ICAM1/CYP1B1/IFNG/MMP8/CAV1/IL1B/NOS3/NQO1/TRPV1/EDN1	13
BP	GO:0071356	cellular response to tumor necrosis factor	21/187	297/18866	2.02E-12	6.68E-11	3.03E-11	CASP8/RELA/IKBKB/AKT1/STAT1/ICAM1/GSTP1/MAPK14/MAPK1/TP53/NFKBIA/CD40LG/MAPK3/APOB/CCL2/CXCL8/COL1A1/NFE2L2/CHUK/TRPV1/EDN1	21
BP	GO:0061041	regulation of wound healing	16/187	151/18866	2.17E-12	7.11E-11	3.23E-11	PRKCA/ALOX5/ADRA2A/PLAU/HMGCR/CAV1/F3/GJA1/DUOX2/NOS3/THBD/SERPINE1/NFE2L2/CLDN4/EDN1/SERPI	16
BP	GO:0071621	granulocyte chemotaxis	15/187	127/18866	2.20E-12	7.17E-11	3.25E-11	LBP/DPP4/DPEP1/MAPK14/MAPK1/IL4/MAPK3/IL1B/CCL2/CXCL8/CXCL11/CXCL2/CXCL10/EDN1/HRH1	15
BP	GO:1901342	regulation of vasculature development	25/187	444/18866	2.26E-12	7.29E-11	3.31E-11	IL6R/PTGS2/PRKCA/PPARG/STAT1/HMOX1/CYP1B1/ALOX5/VEGFA/ERBB2/KDR/STAT3/HIF1A/F3/IL1B/CXCL8/PRKCB/NOS3/HSPB1/SERPINE1/IL1A/NFE2L2/CXCL10/HK2/ADM	25

BP	GO:1902806	regulation of cell cycle G1/S phase transition	18/187	206/18866	2.40E-12	7.69E-11	3.49E-11	BCL2/BAX/AKT1/CDK1/CYP1A1/CDK2/EGFR/CCND1/CDKN1A/RB1/CDK4/TP53/MDM2/PCNA/CCNB1/CCL2/CHEK2/E2	18
BP	GO:2001057	reactive nitrogen species metabolic process	13/187	87/18866	3.16E-12	1.01E-10	4.56E-11	PTGS2/NOS2/AKT1/ICAM1/CYP1B1/IFNG/MMP8/CAV1/IL1B/NOS3/NQO1/TRPV1/EDN1	13
BP	GO:0001890	placenta development	16/187	156/18866	3.60E-12	1.14E-10	5.16E-11	PTGS2/CASP8/PPARG/AKT1/RXRA/MAPK14/EGFR/MAPK1/PPARD/MAPK3/SOD1/HIF1A/GJA1/SPP1/IGF2/ADM	16
BP	GO:1903034	regulation of response to wounding	17/187	183/18866	3.73E-12	1.17E-10	5.31E-11	PRKCA/ALOX5/ADRA2A/PLAU/HMGCR/CAV1/F3/GJA1/DUOX2/NOS3/THBD/SERPINE1/NFE2L2/CLDN4/SPP1/EDN1/SERPINB2	17
BP	GO:0001101	response to acid chemical	15/187	132/18866	3.90E-12	1.20E-10	5.44E-11	CASP3/RELA/ICAM1/GSTP1/AKR1B1/EGFR/VEGFA/BCL2L1/MMP2/PCNA/MAPK10/BAD/COL1A1/CHUK/EDN1	15
BP	GO:1902807	negative regulation of cell cycle G1/S phase transition	15/187	132/18866	3.90E-12	1.20E-10	5.44E-11	BCL2/BAX/CDK1/CDK2/CCND1/CDKN1A/RB1/CDK4/TP53/MDM2/PCNA/CCNB1/CCL2/CHEK2/	15
BP	GO:0002685	regulation of leukocyte migration	18/187	212/18866	3.91E-12	1.20E-10	5.44E-11	IL6R/LBP/DPP4/AKT1/HMOX1/ICAM1/SELE/MAPK14/VEGFA/MAPK1/IL4/MAPK3/CYP19A1/CCL2/CXCL8/SERPINE1/CXCL10/EDN1	18
BP	GO:0051402	neuron apoptotic process	19/187	245/18866	4.80E-12	1.46E-10	6.63E-11	BCL2/BAX/CASP9/JUN/CASP3/HMOX1/BCL2L1/RB1/TP53/MCL1/XIAP/SOD1/HIF1A/HSPA5/CCL2/NQO1/PARP1/ERBB3	19

BP	GO:0046683	response to organophosphorus	15/187	134/18866	4.86E-12	1.47E-10	6.67E-11	PTGS2/SLC6A4/RELA/STAT1/AHR/TYR/SOD1/AKR1C1/FOS/HSPA5/IL1B/DUOX2/THBD/COL1A1/TRPV1	15
BP	GO:0043281	regulation of cysteine-type endopeptidase activity involved in apoptotic process	18/187	215/18866	4.96E-12	1.49E-10	6.76E-11	PTGS2/BAX/CASP9/CASP8/PPARG/AKT1/DPEP1/VEGFA/MMP9/MDM2/BIRC5/XIAP/BAD/RAF1/MYC/F3/CTSD/FABP1	18
BP	GO:0008217	regulation of blood pressure	17/187	187/18866	5.30E-12	1.58E-10	7.17E-11	PTGS1/PTGS2/ADRA1B/NOS2/AR/PPARG/HMOX1/SOD1/PPARA/CES1/GJA1/NOS3/DRD2/TRPV1/EDN1/ENPEP/ADM	17
BP	GO:0046879	hormone secretion	21/187	314/18866	5.87E-12	1.74E-10	7.88E-11	PRKCA/NOS2/DPP4/ALOX5/ADRA2A/EGFR/IFNG/PPARD/BAD/HMGCR/CYP19A1/RAF1/HIF1A/GJA1/IL1B/SPP1/DRD2/EDN1/INS/GCG/ADM	21
BP	GO:0010332	response to gamma radiation	11/187	56/18866	6.82E-12	2.01E-10	9.10E-11	BCL2/BAX/BCL2L1/CDKN1A/TP53/MDM2/HSPA5/MYC/PARP1/CHEK2/CXCL10	11
BP	GO:0016101	diterpenoid metabolic process	14/187	114/18866	7.13E-12	2.08E-10	9.45E-11	CYP3A4/CYP1A2/CYP1A1/CYP1B1/AKR1C3/AKR1B1/EGFR/PPARD/APOB/PLB1/UGT1A1/AKR1C1/ADH1B/LPL	14
BP	GO:0048638	regulation of developmental growth	22/187	353/18866	7.19E-12	2.09E-10	9.47E-11	SLC6A4/BCL2/AR/AKT1/CDK1/MAPK14/GSK3B/VEGFA/CDKN1A/MAPK1/CDK4/CCNB1/PPARD/SOD1/PPARA/STAT3/GJA1/SPP1/IGF2/DRD2/EDN1/BDNF	22
BP	GO:0098754	detoxification	15/187	138/18866	7.48E-12	2.16E-10	9.79E-11	PTGS1/PTGS2/GSTP1/GSTM1/CAT/SOD1/GSR/DUOX2/NOS3/MPO/ABCG2/NFE2L2/NQO1/GSTA1/FABP1	15

BP	GO:0042445	hormone metabolic process	18/187	221/18866	7.89E-12	2.26E-10	1.03E-10	CYP3A4/CYP1A2/CYP1A1/CYP1B1/AKR1C3/ESR1/AKR1B1/PLB1/CYP19A1/UGT1A1/AKR1C1/CES1/HIF1A/DUOX2/SPP1/ADH1B/ENPEP/ADM	18
BP	GO:0006869	lipid transport	23/187	393/18866	8.39E-12	2.38E-10	1.08E-10	LBP/PON1/NOS2/PPARG/AKT1/RXRA/NFKBIA/PPARD/APOB/CYP19A1/PPARA/ABCC1/AKR1C1/CES1/SOAT1/EGF/ACACA/CAV1/IL1B/SPP1/DRD2/EDN1/FABP1	23
BP	GO:0032102	negative regulation of response to external stimulus	24/187	433/18866	8.98E-12	2.54E-10	1.15E-10	TNFAIP6/PPARG/DPP4/ALOX5/GSTP1/PLAU/RB1/IL2RA/IL4/PPARD/SOD1/HMGCR/CYP19A1/PPARA/GJA1/CCL2/NOS3/THBD/SERPINE1/SPP1/DRD2/EDN1/INS/SERPI[illegible]	24
BP	GO:0031100	animal organ regeneration	12/187	75/18866	9.56E-12	2.68E-10	1.22E-10	PPARG/CDK1/HMOX1/GSTP1/CCNA2/EGFR/CCND1/CDKN1A/CDK4/PCNA/UGT1A1/ADM	12
BP	GO:0009914	hormone transport	21/187	323/18866	1.00E-11	2.80E-10	1.27E-10	PRKCA/NOS2/DPP4/ALOX5/ADRA2A/EGFR/IFNG/PPARD/BAD/HMGCR/CYP19A1/RAF1/HIF1A/GJA1/IL1B/SPP1/DRD2/EDN1/INS/GCG/ADM	21
BP	GO:0008631	intrinsic apoptotic signaling pathway in response to oxidative stress	10/187	43/18866	1.04E-11	2.88E-10	1.30E-10	BCL2/AKT1/CYP1B1/MCL1/SOD1/HIF1A/HSPB1/NFE2L2/PARP1/INS	10
BP	GO:0045931	positive regulation of mitotic cell cycle	16/187	168/18866	1.13E-11	3.10E-10	1.41E-10	PRKCA/AKT1/CDK1/CYP1A1/EGFR/CCND1/RB1/CDK4/MDM2/CCNB1/EGF/IL1B/IL1A/IGF2/EDN1/INS	16

BP	GO:0022407	regulation of cell-cell adhesion	24/187	439/18866	1.20E-11	3.28E-10	1.49E-10	CASP3/PRKCA/DPP4/RELA/AKT1/ICAM1/SELE/VCAM1/ALOX5/MAPK14/VEGFA/ERBB2/IL2RA/IFNG/IL4/CD40LG/BAD/PPARA/CAV1/IL1B/CCL2/IL1A/IGF2/IRF1	24
BP	GO:0031663	lipopolysaccharide-mediated signaling pathway	11/187	59/18866	1.25E-11	3.39E-10	1.54E-10	CD14/LBP/PRKCA/AKT1/MAPK14/MAPK1/NFKBIA/MAPK3/IL1B/CCL2/NOS3	11
BP	GO:0010876	lipid localization	24/187	440/18866	1.26E-11	3.39E-10	1.54E-10	LBP/PON1/NOS2/PPARG/AKT1/RXRA/NFKBIA/PPARD/APOB/CYP19A1/PPARA/ABCC1/AKR1C1/CES1/SOAT1/EGF/ACACA/CAV1/IL1B/SPP1/DRD2/EDN1/LPL/FABP1	24
BP	GO:1901216	positive regulation of neuron death	13/187	97/18866	1.32E-11	3.52E-10	1.60E-10	BAX/CASP9/JUN/CASP3/CASP8/GSK3B/TP53/MCL1/IFNG/BAD/FOS/NQO1/PARP1	13
BP	GO:1901655	cellular response to ketone	13/187	97/18866	1.32E-11	3.52E-10	1.60E-10	CASP9/AR/PPARG/AKT1/ICAM1/AHR/AKR1C3/AKR1B1/EGFR/CDK4/NR3C1/ACACA/SPP1	13
BP	GO:0006720	isoprenoid metabolic process	15/187	144/18866	1.39E-11	3.67E-10	1.66E-10	CYP3A4/CYP1A2/CYP1A1/CYP1B1/AKR1C3/AKR1B1/EGFR/PPARD/APOB/PLB1/HMGCR/UGT1A1/AKR1C1/ADH1B/	15
BP	GO:0050921	positive regulation of chemotaxis	15/187	144/18866	1.39E-11	3.67E-10	1.66E-10	IL6R/LBP/MAPK14/VEGFA/MAPK1/IL4/MET/KDR/MAPK3/F3/CXCL8/HSPB1/SERPINE1/CXCL10/EDN1	15

BP	GO:0042110	T cell activation	25/187	483/18866	1.41E-11	3.69E-10	1.68E-10	BCL2/BAX/CASP3/CASP8/DPP4/AKT1/ICAM1/VCAM1/TP53/ERBB2/IL2RA/IFNG/IL4/CD40LG/BAD/SOD1/STAT3/CAV1/IL1B/CCL2/IL1A/RUNX2/IGF2/IRF1/INS	25
BP	GO:1903037	regulation of leukocyte cell-cell adhesion	21/187	329/18866	1.42E-11	3.69E-10	1.68E-10	CASP3/DPP4/RELA/AKT1/ICAM1/SELE/VCAM1/ALOX5/ERBB2/IL2RA/IFNG/IL4/CD40LG/BAD/PPARA/CAV1/IL1B/CCL2/IL1A/IGF2/IRF1	21
BP	GO:0019229	regulation of vasoconstriction	11/187	60/18866	1.52E-11	3.92E-10	1.78E-10	PTGS2/ADRA1B/AKT1/ICAM1/ADRA2A/EGFR/CAV1/GJA1/EDN1/ADM/HRH1	11
BP	GO:0042310	vasoconstriction	12/187	78/18866	1.55E-11	3.98E-10	1.81E-10	PTGS2/ADRA1B/SLC6A4/AKT1/ICAM1/ADRA2A/EGFR/CAV1/GJA1/EDN1/ADM/HRH1	12
BP	GO:0031099	regeneration	17/187	201/18866	1.69E-11	4.32E-10	1.96E-10	BCL2/JUN/PPARG/CDK1/HMOX1/GSTP1/CCNA2/EGFR/CCND1/CDKN1A/CDK4/PCNA/CCNB1/PPARD/UGT1A1/SPP1/A	17
BP	GO:0006633	fatty acid biosynthetic process	16/187	173/18866	1.76E-11	4.48E-10	2.03E-10	PTGS1/PTGS2/CYP3A4/CYP1A2/CYP1A1/ALOX5/GSTP1/GSTM1/AKR1C3/PTGES/FASN/ACACA/IL1B/EDN1/LPL/SCD	16
BP	GO:0045862	positive regulation of proteolysis	22/187	370/18866	1.81E-11	4.56E-10	2.07E-10	BAX/CASP9/CASP8/PPARG/AKT1/ADRA2A/MAPK14/GSK3B/MDM2/IFNG/MAPK3/BAD/STAT3/EGF/CAV1/MYC/F3/IL1B/NFE2L2/CLDN4/CTSD/PCOLCE	22

BP	GO:0030522	intracellular receptor signaling pathway	19/187	265/18866	1.90E-11	4.76E-10	2.16E-10	NR3C2/CASP8/AR/PPARG/RELA/NR1I2/AHR/AKR1C3/ESR1/RXRA/ESR2/NFKBIA/XIAP/NR3C1/PPARD/PPARA/RXRB/STAT3/PARP1	19
BP	GO:0009743	response to carbohydrate	18/187	233/18866	1.92E-11	4.78E-10	2.17E-10	PTGS2/CASP3/ICAM1/GSTP1/CAT/ADRA2A/PPARD/BAD/APOB/HMGCR/GOT1/RAF1/HIF1A/GJA1/IL1B/PRKCB/LPL/G	18
BP	GO:0007050	cell cycle arrest	18/187	234/18866	2.06E-11	5.10E-10	2.32E-10	BAX/CDK1/CDK2/CCND1/CDKN1A/RB1/CDK4/TP53/MDM2/PCNA/CCNB1/IFNG/MYC/CXCL8/CHEK2/RASSF1/E2F1/IRF	18
BP	GO:0046883	regulation of hormone secretion	19/187	267/18866	2.16E-11	5.33E-10	2.42E-10	PRKCA/NOS2/DPP4/ALOX5/ADRA2A/EGFR/IFNG/PPARD/BAD/HMGCR/CYP19A1/HIF1A/GJA1/IL1B/SPP1/DRD2/EDN1/INS/GCG	19
BP	GO:0002688	regulation of leukocyte chemotaxis	14/187	124/18866	2.28E-11	5.55E-10	2.52E-10	IL6R/LBP/DPP4/MAPK14/VEGFA/MAPK1/IL4/MAPK3/CYP19A1/CCL2/CXCL8/SERPINE1/CXCL10/EDN1	14
BP	GO:0006721	terpenoid metabolic process	14/187	124/18866	2.28E-11	5.55E-10	2.52E-10	CYP3A4/CYP1A2/CYP1A1/CYP1B1/AKR1C3/AKR1B1/EGFR/PPARD/APOB/PLB1/UGT1A1/AKR1C1/ADH1B/LPL	14
BP	GO:1903829	positive regulation of cellular protein localization	21/187	338/18866	2.36E-11	5.72E-10	2.60E-10	PTGS2/BCL2/CASP8/AKT1/MAPK8/CDK1/MAPK14/GSK3B/EGFR/MAPK1/TP53/MDM2/ERBB2/IFNG/BAD/EGF/IL1B/PARP1/E2F1/NPEPPS/INS	21

BP	GO:0071383	cellular response to steroid hormone stimulus	17/187	206/18866	2.50E-11	6.04E-10	2.74E-10	NR3C2/CASP9/AR/ICAM1/GSTP1/AKR1C3/ESR1/RXRA/ESR2/EGFR/NR3C1/PPARD/UGT1A1/PPARA/RXRB/PARP1/EDN1	17
BP	GO:0097530	granulocyte migration	15/187	150/18866	2.52E-11	6.04E-10	2.74E-10	LBP/DPP4/DPEP1/MAPK14/MAPK1/IL4/MAPK3/IL1B/CCL2/CXCL8/CXCL11/CXCL2/CXCL10/EDN1/HRH1	15
BP	GO:0019932	second-messenger-mediated signaling	24/187	456/18866	2.65E-11	6.30E-10	2.86E-10	ADRA1B/OPRM1/PRKCA/NOS2/SELE/VCAM1/AHR/ADRA2A/GSK3B/EGFR/VEGFA/KDR/PTGER3/CXCL8/NOS3/CXCL11/CXCL10/ERBB3/DRD2/EDN1/INS/GCG/ADM/H	24
BP	GO:0031571	mitotic G1 DNA damage checkpoint	11/187	63/18866	2.66E-11	6.30E-10	2.86E-10	BAX/CDK1/CDK2/CCND1/CDKN1A/TP53/MDM2/PCNA/CCNB1/CHEK2/E2F1	11
BP	GO:0006953	acute-phase response	10/187	47/18866	2.71E-11	6.39E-10	2.90E-10	IL6R/LBP/PTGS2/UGT1A1/STAT3/IL1B/PTGER3/IL1A/TRPV1/INS	10
BP	GO:0098869	cellular oxidant detoxification	13/187	103/18866	2.88E-11	6.76E-10	3.07E-10	PTGS1/PTGS2/GSTP1/CAT/SOD1/GSR/DUOX2/NOS3/MPO/NFE2L2/NQO1/GSTA1/FABP1	13
BP	GO:2000116	regulation of cysteine-type endopeptidase activity	18/187	239/18866	2.93E-11	6.83E-10	3.10E-10	PTGS2/BAX/CASP9/CASP8/PPARG/AKT1/DPEP1/VEGFA/MMP9/MDM2/BIRC5/XIAP/BAD/RAF1/MYC/F3/CTSD/FABP1	18
BP	GO:0044783	G1 DNA damage checkpoint	11/187	64/18866	3.18E-11	7.30E-10	3.31E-10	BAX/CDK1/CDK2/CCND1/CDKN1A/TP53/MDM2/PCNA/CCNB1/CHEK2/E2F1	11
BP	GO:0044819	mitotic G1/S transition checkpoint	11/187	64/18866	3.18E-11	7.30E-10	3.31E-10	BAX/CDK1/CDK2/CCND1/CDKN1A/TP53/MDM2/PCNA/CCNB1/CHEK2/E2F1	11

BP	GO:0060135	maternal process involved in female pregnancy	11/187	64/18866	3.18E-11	7.30E-10	3.31E-10	PTGS2/AR/AKT1/ESR1/RXRA/AKR1B1/MAPK1/PPARD/MAPK3/GJA1/S	11
BP	GO:0008203	cholesterol metabolic process	15/187	153/18866	3.35E-11	7.65E-10	3.47E-10	PON1/CYP3A4/CYP1A2/RXRA/CAT/IL4/PPARD/FASN/SOD1/APOB/HMGCR/CES1/SOAT1/ACACA/SCD	15
BP	GO:0044843	cell cycle G1/S phase transition	20/187	310/18866	3.69E-11	8.39E-10	3.81E-10	BCL2/BAX/AKT1/CDK1/CYP1A1/CDK2/CCNA2/EGFR/CCND1/CDKN1A/RB1/CDK4/TP53/MDM2/PCNA/CCNB1/MYC/CCL2/CHEK2/E2F1	20
BP	GO:0090316	positive regulation of intracellular protein transport	16/187	182/18866	3.81E-11	8.61E-10	3.91E-10	PTGS2/BCL2/CASP8/MAPK8/CDK1/MAPK14/GSK3B/MAPK1/TP53/MDM2/ERBB2/IFNG/BAD/IL1B/E2F1/NPEPPS	16
BP	GO:0006692	prostanoid metabolic process	10/187	49/18866	4.23E-11	9.45E-10	4.29E-10	PTGS1/PTGS2/GSTP1/GSTM1/AKR1C3/PTGES/AKR1C1/IL1B/GSTA1/E	10
BP	GO:0006693	prostaglandin metabolic process	10/187	49/18866	4.23E-11	9.45E-10	4.29E-10	PTGS1/PTGS2/GSTP1/GSTM1/AKR1C3/PTGES/AKR1C1/IL1B/GSTA1/E	10
BP	GO:0071312	cellular response to alkaloid	9/187	35/18866	4.30E-11	9.58E-10	4.35E-10	OPRM1/CASP3/ICAM1/CCNA2/BCL2L1/MDM2/CASP7/TRPV1/CACNA1	9
BP	GO:0001936	regulation of endothelial cell proliferation	16/187	184/18866	4.50E-11	9.95E-10	4.52E-10	JUN/PRKCA/PPARG/AKT1/STAT1/HMOX1/ALOX5/VEGFA/KDR/STAT3/HIF1A/CAV1/F3/GJA1/CCL2/IGF2	16
BP	GO:0050708	regulation of protein secretion	21/187	352/18866	5.04E-11	1.11E-09	5.03E-10	CD14/OPRM1/PRKCA/NOS2/DPP4/ACHE/ALOX5/ADRA2A/EGFR/IFNG/CD40LG/PPARD/BAD/HMGCR/HIF1A/GJA1/IL1B/IL1A/DRD2/INS/GCG	21

BP	GO:0001523	retinoid metabolic process	13/187	108/18866	5.31E-11	1.16E-09	5.28E-10	CYP3A4/CYP1A2/CYP1A1/CYP1B1/AKR1C3/AKR1B1/PPARD/APOB/PLB1/UGT1A1/AKR1C1/ADH1B/LPL	13
BP	GO:0048146	positive regulation of fibroblast proliferation	10/187	51/18866	6.46E-11	1.40E-09	6.36E-10	JUN/ESR1/CCNA2/EGFR/CDKN1A/CDK4/CCNB1/FOSL2/MYC/E2F1	10
BP	GO:1902893	regulation of pri-miRNA transcription by RNA polymerase II	10/187	51/18866	6.46E-11	1.40E-09	6.36E-10	JUN/PPARG/RELA/TP53/NR3C1/PPARD/PPARA/STAT3/FOS/HIF1A	10
BP	GO:0034754	cellular hormone metabolic process	14/187	134/18866	6.56E-11	1.42E-09	6.42E-10	CYP3A4/CYP1A2/CYP1A1/CYP1B1/AKR1C3/ESR1/AKR1B1/PLB1/CYP19A1/UGT1A1/AKR1C1/SPP1/ADH1B/ADM	14
BP	GO:0000082	G1/S transition of mitotic cell cycle	19/187	287/18866	7.53E-11	1.62E-09	7.34E-10	BCL2/BAX/AKT1/CDK1/CYP1A1/CDK2/EGFR/CCND1/CDKN1A/RB1/CDK4/TP53/MDM2/PCNA/CCNB1/MYC/CCL2/CHEK2/E2F1	19
BP	GO:1902652	secondary alcohol metabolic process	15/187	162/18866	7.61E-11	1.62E-09	7.35E-10	PON1/CYP3A4/CYP1A2/RXRA/CAT/IL4/PPARD/FASN/SOD1/APOB/HMGCR/CES1/SOAT1/ACACA/SCD	15
BP	GO:0031669	cellular response to nutrient levels	17/187	221/18866	7.62E-11	1.62E-09	7.35E-10	BCL2/JUN/PPARG/MAPK8/HMOX1/ICAM1/AKR1C3/CDKN1A/MAPK1/TP53/MDM2/MAPK3/PPARA/HSPA5/COL1A1/NFE2L2/LPL	17
BP	GO:0035265	organ growth	16/187	191/18866	7.89E-11	1.67E-09	7.58E-10	SLC6A4/BCL2/AR/AKT1/CDK1/ESR1/RXRA/MAPK14/MAPK1/CCNB1/MMP13/SOD1/CYP19A1/PPARA/GJA1/EDN1	16

BP	GO:0007159	leukocyte cell-cell adhesion	21/187	364/18866	9.38E-11	1.97E-09	8.96E-10	CASP3/DPP4/RELA/AKT1/ICAM1/SELE/VCAM1/ALOX5/ERBB2/IL2RA/IFNG/IL4/CD40LG/BAD/PPARA/CAV1/IL1B/CCL2/IL1A/IGF2/IRF1	21
BP	GO:0001676	long-chain fatty acid metabolic process	13/187	113/18866	9.48E-11	1.99E-09	9.01E-10	PTGS1/PTGS2/CYP3A4/CYP1A2/CYP1A1/CYP1B1/ALOX5/GSTP1/GSTM1/AKR1C3/PTGES/MAPK3/GSTA1	13
BP	GO:0061614	pri-miRNA transcription by RNA polymerase II	10/187	53/18866	9.69E-11	2.01E-09	9.14E-10	JUN/PPARG/RELA/TP53/NR3C1/PPARD/PPARA/STAT3/FOS/HIF1A	10
BP	GO:0030072	peptide hormone secretion	18/187	257/18866	9.71E-11	2.01E-09	9.14E-10	PRKCA/NOS2/DPP4/ALOX5/ADRA2A/EGFR/IFNG/PPARD/BAD/HMGCR/RAF1/HIF1A/GJA1/IL1B/DRD2/EDN1/INS/GCG	18
BP	GO:0042180	cellular ketone metabolic process	18/187	260/18866	1.17E-10	2.42E-09	1.10E-09	PTGS2/PPARG/AKT1/AKR1C3/AKR1B1/PPARD/HMGCR/CYP19A1/PPARA/AKR1C1/CES1/ODC1/CAV1/IL1B/NQO1/INS/FABP1/ADM	18
BP	GO:0072401	signal transduction involved in DNA integrity checkpoint	11/187	73/18866	1.41E-10	2.88E-09	1.31E-09	BAX/CDK1/CDK2/CHEK1/CDKN1A/TP53/MDM2/PCNA/CCNB1/CHEK2/E2F1	11
BP	GO:0072422	signal transduction involved in DNA damage checkpoint	11/187	73/18866	1.41E-10	2.88E-09	1.31E-09	BAX/CDK1/CDK2/CHEK1/CDKN1A/TP53/MDM2/PCNA/CCNB1/CHEK2/E2F1	11
BP	GO:0001935	endothelial cell proliferation	16/187	199/18866	1.46E-10	2.97E-09	1.35E-09	JUN/PRKCA/PPARG/AKT1/STAT1/HMOX1/ALOX5/VEGFA/KDR/STAT3/HIF1A/CAV1/F3/GJA1/CCL2/IGF2	16
BP	GO:1904019	epithelial cell apoptotic process	13/187	117/18866	1.48E-10	2.99E-09	1.36E-09	HMOX1/ICAM1/AKR1C3/BCL2L1/RB1/IL4/CD40LG/KDR/BAD/CCL2/SERPINE1/NFE2L2/E2F1	13

BP	GO:1902175	regulation of oxidative stress-induced intrinsic apoptotic signaling pathway	8/187	27/18866	1.52E-10	3.05E-09	1.39E-09	AKT1/MCL1/SOD1/HIF1A/HSPB1/NFE2L2/PARP1/INS	8
BP	GO:0016572	histone phosphorylation	9/187	40/18866	1.60E-10	3.20E-09	1.45E-09	PRKCA/CDK1/CDK2/CHEK1/CCNA2/CCNB1/MAPK3/IL1B/PRKCB	9
BP	GO:0031098	stress-activated protein kinase signaling cascade	19/187	300/18866	1.61E-10	3.21E-09	1.46E-09	IKBKB/AKT1/MAPK8/GSTP1/AKR1B1/MAPK14/EGFR/VEGFA/MAPK1/CD40LG/MMP8/MAPK10/NCF1/MAPK3/HMGCR/MYC/IL1B/CHUK/EDN1	19
BP	GO:0006801	superoxide metabolic process	11/187	74/18866	1.64E-10	3.24E-09	1.47E-09	NOS2/GSTP1/EGFR/NCF1/SOD1/DUOX2/NOS3/MPO/NFE2L2/NQO1/ED	11
BP	GO:0072395	signal transduction involved in cell cycle checkpoint	11/187	74/18866	1.64E-10	3.24E-09	1.47E-09	BAX/CDK1/CDK2/CHEK1/CDKN1A/TP53/MDM2/PCNA/CCNB1/CHEK2/E2F1	11
BP	GO:1905952	regulation of lipid localization	15/187	171/18866	1.64E-10	3.24E-09	1.47E-09	PON1/PPARG/AKT1/NFKBIA/PPARD/APOB/CYP19A1/PPARA/CES1/EGF/CAV1/IL1B/SPP1/EDN	15
BP	GO:0006977	DNA damage response, signal transduction by p53 class mediator resulting in cell cycle arrest	10/187	56/18866	1.72E-10	3.37E-09	1.53E-09	BAX/CDK1/CDK2/CDKN1A/TP53/MDM2/PCNA/CCNB1/CHEK2/E2F1	10
BP	GO:1904645	response to amyloid-beta	10/187	56/18866	1.72E-10	3.37E-09	1.53E-09	ICAM1/VCAM1/GSK3B/MMP2/MMP9/MMP13/ABCC1/MMP3/GJA1/PAR	10
BP	GO:0010822	positive regulation of mitochondrion organization	13/187	119/18866	1.83E-10	3.56E-09	1.62E-09	BCL2/BAX/CASP8/MAPK8/GSK3B/MMP9/TP53/KDR/BAD/HIF1A/E2F1/NPEPPS/HK2	13
BP	GO:1903039	positive regulation of leukocyte cell-cell adhesion	17/187	235/18866	2.00E-10	3.86E-09	1.75E-09	DPP4/RELA/AKT1/ICAM1/SELE/VCAM1/ALOX5/IL2RA/IFNG/IL4/CD40LG/BAD/CAV1/IL1B/CCL2/IL1A/IGF2	17
BP	GO:0071398	cellular response to fatty acid	10/187	57/18866	2.07E-10	3.96E-09	1.80E-09	PPARG/AKT1/AKR1C3/CDK4/CCNB1/APOB/ACACA/E2F1/EDN1/LPL	10

BP	GO:0072431	signal transduction involved in mitotic G1 DNA damage checkpoint	10/187	57/18866	2.07E-10	3.96E-09	1.80E-09	BAX/CDK1/CDK2/CDKN1A/TP53/MDM2/PCNA/CCNB1/CHEK2/E2F1	10
BP	GO:1902400	intracellular signal transduction involved in G1 DNA damage checkpoint	10/187	57/18866	2.07E-10	3.96E-09	1.80E-09	BAX/CDK1/CDK2/CDKN1A/TP53/MDM2/PCNA/CCNB1/CHEK2/E2F1	10
BP	GO:0001659	temperature homeostasis	15/187	174/18866	2.10E-10	3.99E-09	1.81E-09	PTGS2/ACHE/VEGFA/RB1/IL4/STAT3/CAV1/GJA1/IL1B/PTGER3/IL1A/DRD2/TRPV1/UCP2/SC	15
BP	GO:1903708	positive regulation of hemopoiesis	16/187	204/18866	2.11E-10	3.99E-09	1.81E-09	JUN/CASP8/PRKCA/STAT1/MAPK14/RB1/IL2RA/IFNG/IL4/CA2/BAD/STAT3/FOS/HIF1A/IL1B/I	16
BP	GO:0036473	cell death in response to oxidative stress	12/187	97/18866	2.16E-10	4.06E-09	1.84E-09	BCL2/AKT1/CYP1B1/MCL1/MET/SOD1/MMP3/HIF1A/HSPB1/NFE2L2/PARP1/INS	12
BP	GO:0002791	regulation of peptide secretion	21/187	381/18866	2.17E-10	4.06E-09	1.84E-09	CD14/OPRM1/PRKCA/NOS2/DPP4/ACHE/ALOX5/ADRA2A/EGFR/IFNG/CD40LG/PPARD/BAD/HMGCR/HIF1A/GJA1/IL1B/IL1A/DRD2/INS/GCG	21
BP	GO:0051091	positive regulation of DNA-binding transcription factor activity	18/187	270/18866	2.18E-10	4.06E-09	1.84E-09	AR/PPARG/RELA/IKBKB/AKT1/ICAM1/ESR1/CAT/ESR2/VEGFA/CD40LG/STAT3/CAV1/IL1B/PRKCB/CHUK/EDN1/INS	18
BP	GO:0035994	response to muscle stretch	7/187	18/18866	2.44E-10	4.53E-09	2.06E-09	JUN/RELA/MAPK14/NFKBIA/FOS/RAF1/EDN1	7
BP	GO:0010634	positive regulation of epithelial cell migration	15/187	176/18866	2.47E-10	4.56E-09	2.07E-09	PTGS2/JUN/PRKCA/AKT1/HMOX1/VEGFA/MMP9/IFNG/MET/KDR/HIF1A/NOS3/HSPB1/NFE2L2/EDN1	15
BP	GO:0071902	positive regulation of protein serine/threonine kinase activity	20/187	345/18866	2.48E-10	4.56E-09	2.07E-09	AKT1/CDK1/ADRA2A/MAPK14/EGFR/VEGFA/CCND1/MAPK1/ERBB2/CCNB1/IFNG/CD40LG/MAPK10/MAPK3/SOD1/EGF/RAF1/IL1B/IGF2/E	20

BP	GO:0007566	embryo implantation	10/187	58/18866	2.48E-10	4.56E-09	2.07E-09	PTGS2/RXRA/VEGFA/MMP2/MMP9/PPARD/SOD1/GJA1/IL1B/SPP1	10
BP	GO:0046902	regulation of mitochondrial membrane permeability	11/187	77/18866	2.56E-10	4.67E-09	2.12E-09	BCL2/BAX/CASP8/MAPK8/GSK3B/BCL2L1/TP53/BAD/STAT3/E2F1/HK	11
BP	GO:0045785	positive regulation of cell adhesion	22/187	428/18866	2.97E-10	5.31E-09	2.41E-09	PRKCA/DPP4/RELA/AKT1/ICAM1/SELE/VCAM1/ALOX5/GSK3B/VEGFA/ERBB2/IL2RA/IFNG/IL4/CD40LG/KDR/BAD/CAV1/IL1B/CCL2/IL1A/IG	22
BP	GO:0006636	unsaturated fatty acid biosynthetic process	10/187	59/18866	2.97E-10	5.31E-09	2.41E-09	PTGS1/PTGS2/ALOX5/GSTP1/GSTM1/AKR1C3/PTGES/IL1B/EDN1/SCD	10
BP	GO:0072413	signal transduction involved in mitotic cell cycle checkpoint	10/187	59/18866	2.97E-10	5.31E-09	2.41E-09	BAX/CDK1/CDK2/CDKN1A/TP53/MDM2/PCNA/CCNB1/CHEK2/E2F1	10
BP	GO:1902402	signal transduction involved in mitotic DNA damage checkpoint	10/187	59/18866	2.97E-10	5.31E-09	2.41E-09	BAX/CDK1/CDK2/CDKN1A/TP53/MDM2/PCNA/CCNB1/CHEK2/E2F1	10
BP	GO:1902403	signal transduction involved in mitotic DNA integrity checkpoint	10/187	59/18866	2.97E-10	5.31E-09	2.41E-09	BAX/CDK1/CDK2/CDKN1A/TP53/MDM2/PCNA/CCNB1/CHEK2/E2F1	10
BP	GO:0030099	myeloid cell differentiation	22/187	431/18866	3.38E-10	6.03E-09	2.74E-09	CASP9/JUN/CASP3/CASP8/PRKCA/PPARG/STAT1/MAPK14/VEGFA/MMP9/RB1/NFKBIA/IFNG/IL4/CA2/FASN/STAT3/FOS/HIF1A/MYC/PRKCB/	22
BP	GO:0008637	apoptotic mitochondrial changes	13/187	125/18866	3.41E-10	6.05E-09	2.75E-09	BCL2/BAX/JUN/CASP8/AKT1/MAPK8/GSK3B/BCL2L1/MMP9/TP53/BAD/E2F1/HK2	13
BP	GO:0050679	positive regulation of epithelial cell proliferation	16/187	211/18866	3.48E-10	6.15E-09	2.79E-09	JUN/PRKCA/AR/AKT1/HMOX1/EGFR/VEGFA/CCND1/ERBB2/KDR/BAD/STAT3/HIF1A/MYC/F3/IGF2	16

BP	GO:0052548	regulation of endopeptidase activity	22/187	434/18866	3.86E-10	6.79E-09	3.08E-09	PTGS2/BAX/CASP9/CASP8/PPARG/AKT1/SLPI/DPEP1/VEGFA/MMP9/MDM2/BIRC5/XIAP/BAD/STAT3/RAF1/MYC/F3/SERPINE1/CTSD/SERPIN	22
BP	GO:0090276	regulation of peptide hormone secretion	16/187	213/18866	4.00E-10	7.01E-09	3.18E-09	PRKCA/NOS2/DPP4/ALOX5/ADRA2A/EGFR/IFNG/PPARD/BAD/HMGCR/HIF1A/GJA1/IL1B/DRD2/INS/GCG	16
BP	GO:0072330	monocarboxylic acid biosynthetic process	17/187	246/18866	4.06E-10	7.09E-09	3.22E-09	PTGS1/PTGS2/CYP3A4/CYP1A2/CYP1A1/ALOX5/GSTP1/GSTM1/AKR1C3/PTGES/FASN/CES1/ACACA/IL1B/EDN1/LPL	17
BP	GO:0043523	regulation of neuron apoptotic process	16/187	214/18866	4.29E-10	7.45E-09	3.38E-09	BCL2/BAX/CASP9/JUN/CASP3/HMOX1/BCL2L1/TP53/MCL1/SOD1/HIF1A/CCL2/NQO1/PARP1/ERBB3/BDNF	16
BP	GO:0032868	response to insulin	18/187	283/18866	4.66E-10	8.07E-09	3.66E-09	PPARG/RELA/AKT1/STAT1/ICAM1/GSTP1/CAT/GSK3B/CDK4/PPARA/GOT1/IL1B/PRKCB/PARP1/IGF2/LPL/INS/ADM	18
BP	GO:0035094	response to nicotine	9/187	45/18866	4.96E-10	8.55E-09	3.88E-09	BCL2/CASP3/RELA/HMOX1/MAPK1/BAD/PPARA/DRD2/EDN1	9
BP	GO:0051403	stress-activated MAPK cascade	18/187	286/18866	5.52E-10	9.49E-09	4.31E-09	IKBKB/AKT1/MAPK8/GSTP1/MAPK14/EGFR/VEGFA/MAPK1/CD40LG/MMP8/MAPK10/NCF1/MAPK3/HMGCR/MYC/IL1B/CHUK/EDN1	18
BP	GO:0010878	cholesterol storage	7/187	20/18866	5.84E-10	9.99E-09	4.53E-09	PPARG/PPARD/APOB/PPARA/CES1/SOAT1/LPL	7

BP	GO:0051235	maintenance of location	19/187	324/18866	5.90E-10	1.00E-08	4.56E-09	BAX/PPARG/AKT1/NFKBIA/PPARD/APOB/PPARA/CES1/SOAT1/HSPA5/CAV1/GJA1/IL1B/CXCL11/CXCL10/HK2/DRD2/TRPV1/LPL	19
BP	GO:0000075	cell cycle checkpoint	16/187	219/18866	6.03E-10	1.02E-08	4.64E-09	BAX/CDK1/MAPK14/CDK2/CHEK1/CCND1/BCL2L1/CDKN1A/RB1/TP53/MDM2/PCNA/CCNB1/TOP2A/CHEK2/E2F1	16
BP	GO:0071156	regulation of cell cycle arrest	12/187	106/18866	6.17E-10	1.04E-08	4.73E-09	BAX/CDK1/CDK2/CCND1/CDKN1A/CDK4/TP53/MDM2/PCNA/CCNB1/CHEK2/E2F1	12
BP	GO:0010631	epithelial cell migration	20/187	365/18866	6.66E-10	1.12E-08	5.09E-09	PTGS2/JUN/PRKCA/PPARG/DPP4/AKT1/HMOX1/CYP1B1/VEGFA/MMP9/IFNG/IL4/MET/KDR/PPARD/HIF1A/NOS3/HSPB1/NFE2L2/EDN1	20
BP	GO:1903426	regulation of reactive oxygen species biosynthetic process	12/187	107/18866	6.89E-10	1.16E-08	5.24E-09	PTGS2/AKT1/ICAM1/ALOX5/IFNG/MMP8/STAT3/CAV1/IL1B/TRPV1/EDN1/INS	12
BP	GO:0032388	positive regulation of intracellular transport	16/187	222/18866	7.36E-10	1.23E-08	5.58E-09	PTGS2/BCL2/CASP8/MAPK8/CDK1/MAPK14/GSK3B/MAPK1/TP53/MDM2/ERBB2/IFNG/BAD/IL1B/E2F1/NPEPPS	16
BP	GO:0042770	signal transduction in response to DNA damage	13/187	133/18866	7.42E-10	1.24E-08	5.61E-09	BAX/CASP9/CDK1/MAPK14/CDK2/CHEK1/CDKN1A/TP53/MDM2/PCNA/CCNB1/CHEK2/E2F1	13
BP	GO:0031570	DNA integrity checkpoint	14/187	161/18866	7.67E-10	1.26E-08	5.74E-09	BAX/CDK1/MAPK14/CDK2/CHEK1/CCND1/CDKN1A/TP53/MDM2/PCNA/CCNB1/TOP2A/CHEK2/E2F1	14

BP	GO:0090132	epithelium migration	20/187	368/18866	7.68E-10	1.26E-08	5.74E-09	PTGS2/JUN/PRKCA/PPARG/DPP4/AKT1/HMOX1/CYP1B1/VEGFA/MMP9/IFNG/IL4/MET/KDR/PPARD/HIF1A/NOS3/HSPB1/NFE2L2/EDN1	20
BP	GO:0044774	mitotic DNA integrity checkpoint	12/187	108/18866	7.69E-10	1.26E-08	5.74E-09	BAX/CDK1/CDK2/CCND1/CDKN1A/TP53/MDM2/PCNA/CCNB1/TOP2A/CHEK2/E2F1	12
BP	GO:0010821	regulation of mitochondrion organization	15/187	191/18866	7.78E-10	1.27E-08	5.79E-09	BCL2/BAX/CASP8/AKT1/MAPK8/GSK3B/BCL2L1/MMP9/TP53/KDR/BAD/HIF1A/E2F1/NPEPPS/	15
BP	GO:0010039	response to iron ion	8/187	33/18866	9.01E-10	1.47E-08	6.67E-09	BCL2/HMOX1/CYP1A1/CCND1/MDM2/CCNB1/HIF1A/DRD2	8
BP	GO:1903706	regulation of hemopoiesis	23/187	498/18866	9.17E-10	1.49E-08	6.77E-09	JUN/CASP8/PRKCA/STAT1/MAPK14/RB1/NFKBIA/ERBB2/IL2RA/IFNG/IL4/CA2/BAD/SOD1/STAT3/FOS/HIF1A/MYC/IL1B/PRKCB/IL1A/NFE2L	23
BP	GO:0090130	tissue migration	20/187	374/18866	1.02E-09	1.65E-08	7.47E-09	PTGS2/JUN/PRKCA/PPARG/DPP4/AKT1/HMOX1/CYP1B1/VEGFA/MMP9/IFNG/IL4/MET/KDR/PPARD/HIF1A/NOS3/HSPB1/NFE2L2/EDN1	20
BP	GO:0090559	regulation of membrane permeability	11/187	88/18866	1.11E-09	1.80E-08	8.16E-09	BCL2/BAX/CASP8/MAPK8/GSK3B/BCL2L1/TP53/BAD/STAT3/E2F1/HK	11
BP	GO:0007093	mitotic cell cycle checkpoint	14/187	166/18866	1.15E-09	1.85E-08	8.38E-09	BAX/CDK1/CDK2/CCND1/BCL2L1/CDKN1A/RB1/TP53/MDM2/PCNA/CCNB1/TOP2A/CHEK2/E	14
BP	GO:0045907	positive regulation of vasoconstriction	8/187	34/18866	1.17E-09	1.87E-08	8.49E-09	PTGS2/ADRA1B/AKT1/ICAM1/EGFR/CAV1/GJA1/HRH1	8

BP	GO:0006694	steroid biosynthetic process	15/187	197/18866	1.20E-09	1.91E-08	8.66E-09	CYP3A4/CYP1A1/AKR1C3/AKR1B1/IFNG/FASN/SOD1/APOB/HMGCR/CYP19A1/CES1/ACACA/IL1B/SCD/ADM	15
BP	GO:0009306	protein secretion	22/187	462/18866	1.24E-09	1.98E-08	8.97E-09	CD14/OPRM1/PRKCA/NOS2/DPP4/ACHE/ALOX5/ADRA2A/EGFR/IFNG/CD40LG/PPARD/BAD/HMGCR/RAF1/HIF1A/GJA1/IL1B/IL1A/DRD2/INS/	22
BP	GO:0008625	extrinsic apoptotic signaling pathway via death domain receptors	11/187	89/18866	1.26E-09	1.99E-08	9.05E-09	BCL2/BAX/CASP8/HMOX1/ICAM1/GSK3B/BCL2L1/BAD/RAF1/NOS3/SERPINE1	11
BP	GO:0055093	response to hyperoxia	7/187	22/18866	1.26E-09	1.99E-08	9.05E-09	PPARG/CYP1A1/CAT/CDKN1A/CDK4/CAV1/COL1A1	7
BP	GO:0035592	establishment of protein localization to extracellular region	22/187	463/18866	1.29E-09	2.03E-08	9.23E-09	CD14/OPRM1/PRKCA/NOS2/DPP4/ACHE/ALOX5/ADRA2A/EGFR/IFNG/CD40LG/PPARD/BAD/HMGCR/RAF1/HIF1A/GJA1/IL1B/IL1A/DRD2/INS/	22
BP	GO:0032963	collagen metabolic process	12/187	113/18866	1.30E-09	2.04E-08	9.28E-09	PPARG/MMP1/MMP2/MMP9/MMP13/MMP8/PPARD/GOT1/MMP3/HIF1A/COL1A1/CTSD	12
BP	GO:0051047	positive regulation of secretion	19/187	340/18866	1.32E-09	2.06E-08	9.34E-09	CD14/SLC6A4/ACHE/EGFR/IFNG/IL4/PPARD/BAD/CYP19A1/HIF1A/GJA1/IL1B/IL1A/SPP1/DRD2/TRPV1/EDN1/INS/GCG	19
BP	GO:0045930	negative regulation of mitotic cell cycle	19/187	341/18866	1.38E-09	2.15E-08	9.77E-09	BCL2/BAX/CDK1/CDK2/CHEK1/EGFR/CCND1/BCL2L1/CDKN1A/RB1/CDK4/TP53/MDM2/PCNA/CCNB1/TOP2A/CCL2/CHEK2/E2F1	19

BP	GO:0043200	response to amino acid	12/187	114/18866	1.45E-09	2.24E-08	1.01E-08	CASP3/RELA/ICAM1/GSTP1/EGFR/BCL2L1/MMP2/PCNA/BAD/COL1A1/CHUK/EDN1	12
BP	GO:0051100	negative regulation of binding	14/187	169/18866	1.45E-09	2.24E-08	1.01E-08	BAX/JUN/AKT1/MAPK8/HMOX1/SLPI/GSK3B/CDKN1A/NFKBIA/MET/MAPK3/PPARA/CAV1/E	14
BP	GO:0043405	regulation of MAP kinase activity	19/187	342/18866	1.45E-09	2.24E-08	1.01E-08	CDK1/GSTP1/ADRA2A/MAPK14/EGFR/VEGFA/MAPK1/ERBB2/CD40LG/MAPK10/MAPK3/SOD1/HMGCR/EGF/RAF1/CAV1/IL1B/CDK12/EDN1	19
BP	GO:1901030	positive regulation of mitochondrial outer membrane permeabilization involved in apoptotic signaling pathway	8/187	35/18866	1.50E-09	2.30E-08	1.04E-08	BCL2/BAX/CASP8/MAPK8/GSK3B/TP53/BAD/E2F1	8
BP	GO:0007596	blood coagulation	19/187	343/18866	1.52E-09	2.33E-08	1.06E-08	PRKCA/ADRA2A/PLAU/MAPK1/CD40LG/MAPK3/RAF1/CAV1/F3/PRKCB/NOS3/HSPB1/THBD/SERPINE1/COL1A1/NFE2L2/IRF1/EDN1/SERPINB	19
BP	GO:0060249	anatomical structure homeostasis	22/187	469/18866	1.64E-09	2.50E-08	1.13E-08	PTGS2/BCL2/BAX/PRKCA/AKR1B1/EGFR/VEGFA/MAPK1/RB1/PCNA/CA2/MAPK3/SOD1/HIF1A/ACACA/MYC/GJA1/NOS3/HSPB1/PARP1/SPP1	22
BP	GO:0002687	positive regulation of leukocyte migration	13/187	142/18866	1.67E-09	2.53E-08	1.15E-08	IL6R/LBP/ICAM1/SELE/MAPK14/VEGFA/MAPK1/IL4/MAPK3/CXCL8/SERPINE1/CXCL10/EDN1	13
BP	GO:0030879	mammary gland development	13/187	142/18866	1.67E-09	2.53E-08	1.15E-08	BAX/AR/AKT1/ESR1/VEGFA/CCND1/MAPK1/FASN/CYP19A1/EGF/HIF1A/CAV1/HK2	13

BP	GO:0071692	protein localization to extracellular region	22/187	470/18866	1.71E-09	2.57E-08	1.17E-08	CD14/OPRM1/PRKCA/NOS2/DPP4/ACHE/ALOX5/ADRA2A/EGFR/IFNG/CD40LG/PPARD/BAD/HMGCR/RAF1/HIF1A/GJA1/IL1B/IL1A/DRD2/INS/	22
BP	GO:1900407	regulation of cellular response to oxidative stress	11/187	92/18866	1.81E-09	2.71E-08	1.23E-08	AKT1/ALOX5/MCL1/MET/SOD1/MMP3/HIF1A/HSPB1/NFE2L2/PARP1/IN	11
BP	GO:0010506	regulation of autophagy	19/187	347/18866	1.85E-09	2.76E-08	1.25E-08	BCL2/CASP3/AKT1/MAPK8/HMOX1/GSK3B/IL10RA/TP53/MCL1/IFNG/IL4/MET/KDR/MAPK3/BAD/STAT3/HIF1A/HSPB	19
BP	GO:0001893	maternal placenta development	8/187	36/18866	1.91E-09	2.85E-08	1.29E-08	PTGS2/AKT1/RXRA/MAPK1/PPARD/MAPK3/GJA1/SPP1	8
BP	GO:0007599	hemostasis	19/187	348/18866	1.94E-09	2.88E-08	1.31E-08	PRKCA/ADRA2A/PLAU/MAPK1/CD40LG/MAPK3/RAF1/CAV1/F3/PRKCB/NOS3/HSPB1/THBD/SERPINE1/COL1A1/NFE2L2/IRF1/EDN1/SERPINB	19
BP	GO:0035690	cellular response to drug	10/187	71/18866	1.97E-09	2.91E-08	1.32E-08	NOS2/DPEP1/EGFR/TP53/HSPA5/MYC/IL1B/NFE2L2/CHEK2/EDN1	10
BP	GO:0050817	coagulation	19/187	349/18866	2.03E-09	3.00E-08	1.36E-08	PRKCA/ADRA2A/PLAU/MAPK1/CD40LG/MAPK3/RAF1/CAV1/F3/PRKCB/NOS3/HSPB1/THBD/SERPINE1/COL1A1/NFE2L2/IRF1/EDN1/SERPINB	19
BP	GO:0031331	positive regulation of cellular catabolic process	20/187	390/18866	2.09E-09	3.08E-08	1.40E-08	BAX/AKT1/HMOX1/ADRA2A/GSK3B/MDM2/IFNG/IL4/KDR/MAPK3/BAD/PPARA/EGF/HIF1A/CAV1/IL1B/NFE2L2/HK2/INS/FABP1	20
BP	GO:0015718	monocarboxylic acid transport	14/187	174/18866	2.13E-09	3.12E-08	1.41E-08	NOS2/PPARG/AKT1/RXRA/PPARD/PPARA/AKR1C1/CES1/ACACA/IL1B/ABCG2/DRD2/EDN1/FA	14

BP	GO:0070663	regulation of leukocyte proliferation	16/187	240/18866	2.29E-09	3.34E-08	1.52E-08	BCL2/CASP3/VCAM1/GSTP1/AHR/CDKN1A/MAPK1/ERBB2/IL2RA/IL4/CD40LG/MAPK3/IL1B/IL1A/IGF2/IRF1	16
BP	GO:0070661	leukocyte proliferation	18/187	313/18866	2.33E-09	3.38E-08	1.53E-08	BCL2/BAX/CASP3/VCAM1/GSTP1/AHR/CDKN1A/MAPK1/TP53/ERBB2/IL2RA/IL4/CD40LG/MAPK3/IL1B/IL1A/IGF2/IRF	18
BP	GO:1903532	positive regulation of secretion by cell	18/187	313/18866	2.33E-09	3.38E-08	1.53E-08	CD14/SLC6A4/ACHE/EGFR/IFNG/IL4/PPARD/BAD/CYP19A1/HIF1A/GJA1/IL1B/IL1A/SPP1/DRD2/EDN1/INS/GCG	18
BP	GO:0046890	regulation of lipid biosynthetic process	15/187	207/18866	2.37E-09	3.42E-08	1.55E-08	PTGS2/AKT1/AKR1C3/CDK4/IFNG/FASN/SOD1/APOB/HMGCR/CES1/ACACA/IL1B/INS/SCD/A	15
BP	GO:0022409	positive regulation of cell-cell adhesion	17/187	279/18866	2.78E-09	4.01E-08	1.82E-08	DPP4/RELA/AKT1/ICAM1/SELE/VCAM1/ALOX5/IL2RA/IFNG/IL4/CD40LG/BAD/CAV1/IL1B/CCL2/IL1A/IGF2	17
BP	GO:0002690	positive regulation of leukocyte chemotaxis	11/187	96/18866	2.87E-09	4.11E-08	1.87E-08	IL6R/LBP/MAPK14/VEGFA/MAPK1/IL4/MAPK3/CXCL8/SERPINE1/CXCL10/EDN1	11
BP	GO:0002573	myeloid leukocyte differentiation	15/187	210/18866	2.89E-09	4.13E-08	1.87E-08	JUN/CASP8/PRKCA/PPARG/MAPK14/VEGFA/MMP9/RB1/IFNG/IL4/CA2/FASN/FOS/MYC/PARP1	15
BP	GO:0051881	regulation of mitochondrial membrane potential	10/187	74/18866	2.98E-09	4.25E-08	1.93E-08	BCL2/BAX/AKT1/BCL2L1/KDR/BAD/SOD1/GOT1/PARP1/TRPV1	10
BP	GO:0030073	insulin secretion	15/187	213/18866	3.51E-09	4.98E-08	2.26E-08	PRKCA/NOS2/DPP4/ALOX5/ADRA2A/IFNG/PPARD/BAD/HMGCR/RAF1/HIF1A/GJA1/IL1B/DRD2/GCG	15

BP	GO:0050796	regulation of insulin secretion	14/187	181/18866	3.55E-09	5.02E-08	2.28E-08	PRKCA/NOS2/DPP4/ALOX5/ADRA2A/IFNG/PPARD/BAD/HMGCR/HIF1A/GJA1/IL1B/DRD2/GC	14
BP	GO:0000077	DNA damage checkpoint	13/187	151/18866	3.57E-09	5.02E-08	2.28E-08	BAX/CDK1/MAPK14/CDK2/CHEK1/CCND1/CDKN1A/TP53/MDM2/PCNA/CCNB1/CHEK2/E2F1	13
BP	GO:0050810	regulation of steroid biosynthetic process	11/187	98/18866	3.58E-09	5.02E-08	2.28E-08	AKR1C3/IFNG/FASN/SOD1/APOB/HMGCR/CES1/ACACA/IL1B/SCD/AD	11
BP	GO:2001243	negative regulation of intrinsic apoptotic signaling pathway	11/187	98/18866	3.58E-09	5.02E-08	2.28E-08	PTGS2/BCL2/AKT1/BCL2L1/MMP9/MDM2/MCL1/HIF1A/HSPB1/NFE2L2	11
BP	GO:0051101	regulation of DNA binding	12/187	124/18866	3.82E-09	5.34E-08	2.42E-08	JUN/PPARG/MAPK8/HMOX1/MMP9/RB1/NFKBIA/IFNG/MMP8/EGF/PARP1/E2F1	12
BP	GO:0009895	negative regulation of catabolic process	18/187	323/18866	3.83E-09	5.34E-08	2.42E-08	BCL2/NOS2/RELA/AKT1/HMOX1/ADRA2A/MAPK14/EGFR/IL10RA/TP53/MCL1/MET/HMGCR/PPARA/STAT3/IL1B/E2F1	18
BP	GO:1903524	positive regulation of blood circulation	10/187	76/18866	3.90E-09	5.41E-08	2.46E-08	PTGS2/ADRA1B/AKT1/ICAM1/EGFR/CAV1/GJA1/EDN1/ADM/HRH1	10
BP	GO:0034349	glial cell apoptotic process	6/187	15/18866	4.07E-09	5.63E-08	2.56E-08	CASP9/CASP3/PRKCA/RB1/TP53/CCL2	6
BP	GO:0150076	neuroinflammatory response	10/187	77/18866	4.44E-09	6.10E-08	2.77E-08	PTGS2/JUN/EGFR/MMP9/IFNG/IL4/MMP8/MMP3/IL1B/TRPV1	10
BP	GO:1903201	regulation of oxidative stress-induced cell death	10/187	77/18866	4.44E-09	6.10E-08	2.77E-08	AKT1/MCL1/MET/SOD1/MMP3/HIF1A/HSPB1/NFE2L2/PARP1/INS	10
BP	GO:0008585	female gonad development	11/187	100/18866	4.45E-09	6.10E-08	2.77E-08	BCL2/BAX/CASP3/ICAM1/ESR1/VEGFA/BCL2L1/SOD1/CYP19A1/HSPA5/NOS3	11
BP	GO:0019748	secondary metabolic process	9/187	57/18866	4.55E-09	6.20E-08	2.81E-08	BCL2/CYP1A2/CYP1A1/CYP1B1/AKR1C3/AKR1B1/TYR/AKR1C1/NFE2L	9

BP	GO:0042743	hydrogen peroxide metabolic process	9/187	57/18866	4.55E-09	6.20E-08	2.81E-08	CYP1A2/CYP1A1/CAT/EGFR/SOD1/STAT3/MMP3/DUOX2/MPO	9
BP	GO:0046394	carboxylic acid biosynthetic process	19/187	367/18866	4.64E-09	6.30E-08	2.86E-08	PTGS1/PTGS2/CYP3A4/CYP1A2/CYP1A1/ALOX5/GSTP1/GSTM1/AKR1C3/PTGES/FASN/GOT1/CES1/EGF/ACACA/IL1B/EDN1/LPL/SCD	19
BP	GO:1902895	positive regulation of pri-miRNA transcription by RNA polymerase II	8/187	40/18866	4.70E-09	6.37E-08	2.89E-08	JUN/PPARG/RELA/TP53/NR3C1/STAT3/FOS/HIF1A	8
BP	GO:0016053	organic acid biosynthetic process	19/187	368/18866	4.85E-09	6.55E-08	2.97E-08	PTGS1/PTGS2/CYP3A4/CYP1A2/CYP1A1/ALOX5/GSTP1/GSTM1/AKR1C3/PTGES/FASN/GOT1/CES1/EGF/ACACA/IL1B/EDN1/LPL/SCD	19
BP	GO:0044773	mitotic DNA damage checkpoint	11/187	101/18866	4.95E-09	6.62E-08	3.00E-08	BAX/CDK1/CDK2/CCND1/CDKN1A/TP53/MDM2/PCNA/CCNB1/CHEK2/E2F1	11
BP	GO:0045639	positive regulation of myeloid cell differentiation	11/187	101/18866	4.95E-09	6.62E-08	3.00E-08	JUN/CASP8/PRKCA/STAT1/MAPK14/RB1/IFNG/CA2/STAT3/FOS/HIF1A	11
BP	GO:1902882	regulation of response to oxidative stress	11/187	101/18866	4.95E-09	6.62E-08	3.00E-08	AKT1/ALOX5/MCL1/MET/SOD1/MMP3/HIF1A/HSPB1/NFE2L2/PARP1/IN	11
BP	GO:0009896	positive regulation of catabolic process	21/187	454/18866	5.07E-09	6.76E-08	3.07E-08	BAX/AKT1/HMOX1/ADRA2A/GSK3B/MDM2/IFNG/IL4/KDR/MAPK3/BAD/PPARA/EGF/HIF1A/CAV1/GJA1/IL1B/NFE2L2/HK2/INS/FABP1	21
BP	GO:0019369	arachidonic acid metabolic process	9/187	58/18866	5.34E-09	7.07E-08	3.21E-08	PTGS1/PTGS2/CYP1A2/CYP1A1/CYP1B1/ALOX5/AKR1C3/PTGES/MAP	9

BP	GO:0001503	ossification	20/187	412/18866	5.34E-09	7.07E-08	3.21E-08	IL6R/PTGS2/BCL2/ACHE/AKT1/ALOX5/CAT/MAPK14/EGFR/MMP2/MAPK1/MMP13/MAPK3/FASN/HIF1A/COL1A1/SPP1/RUNX2/IGFBP3/IGF2	20
BP	GO:0001655	urogenital system development	18/187	330/18866	5.36E-09	7.08E-08	3.21E-08	IL6R/BCL2/BAX/CASP9/AR/STAT1/ESR1/RXRA/CAT/AKR1B1/VEGFA/MMP9/CA2/CYP19A1/ODC1/MYC/BDNF/ENPEP	18
BP	GO:0032147	activation of protein kinase activity	18/187	331/18866	5.62E-09	7.40E-08	3.36E-08	IL6R/AKT1/CDK1/ADRA2A/MAPK14/EGFR/VEGFA/MAPK1/IL4/CD40LG/MAPK10/MAPK3/SOD1/EGF/RAF1/IL1B/DRD2	18
BP	GO:0043536	positive regulation of blood vessel endothelial cell migration	10/187	79/18866	5.73E-09	7.53E-08	3.42E-08	PTGS2/PRKCA/AKT1/HMOX1/VEGFA/KDR/HIF1A/NOS3/HSPB1/NFE2L	10
BP	GO:1902042	negative regulation of extrinsic apoptotic signaling pathway via death domain receptors	8/187	41/18866	5.79E-09	7.55E-08	3.43E-08	CASP8/HMOX1/ICAM1/GSK3B/BCL2L1/RAF1/NOS3/SERPINE1	8
BP	GO:0002064	epithelial cell development	15/187	221/18866	5.80E-09	7.55E-08	3.43E-08	AR/IKBKB/ICAM1/ESR1/AKR1B1/GSK3B/VEGFA/CDKN1A/MET/FASN/BAD/SOD1/FOSL2/HIF1A/IL1B	15
BP	GO:0071478	cellular response to radiation	14/187	188/18866	5.80E-09	7.55E-08	3.43E-08	PTGS2/BAX/CASP9/MAPK14/CHEK1/BCL2L1/CDKN1A/TP53/MDM2/PCNA/HSPA5/MYC/PARP1/CHEK2	14
BP	GO:0008630	intrinsic apoptotic signaling pathway in response to DNA damage	11/187	103/18866	6.10E-09	7.92E-08	3.59E-08	BCL2/BAX/CASP9/HMOX1/BCL2L1/CDKN1A/TP53/MCL1/BAD/CHEK2/E	11
BP	GO:0010565	regulation of cellular ketone metabolic process	14/187	189/18866	6.21E-09	8.03E-08	3.65E-08	PTGS2/PPARG/AKT1/AKR1C3/PPARD/PPARA/CES1/ODC1/CAV1/IL1B/NQO1/INS/FABP1/ADM	14

BP	GO:0018108	peptidyl-tyrosine phosphorylation	19/187	374/18866	6.31E-09	8.14E-08	3.69E-08	IL6R/ICAM1/ADRA2A/EGFR/VEGFA/TP53/ERBB2/IFNG/IL4/MET/KDR/NCF1/MAPK3/STAT3/EGF/CAV1/IGF2/ERBB3/	19
BP	GO:1901617	organic hydroxy compound biosynthetic process	16/187	258/18866	6.47E-09	8.31E-08	3.77E-08	CYP3A4/AKR1C3/AKR1B1/TYR/IFNG/FASN/SOD1/APOB/HMGCR/CYP19A1/GOT1/CES1/ACACA/IL1B/SCD/HRH1	16
BP	GO:0019218	regulation of steroid metabolic process	12/187	131/18866	7.17E-09	9.18E-08	4.17E-08	AKR1C3/IFNG/FASN/SOD1/APOB/HMGCR/UGT1A1/CES1/ACACA/IL1B/SCD/ADM	12
BP	GO:0018212	peptidyl-tyrosine modification	19/187	377/18866	7.19E-09	9.18E-08	4.17E-08	IL6R/ICAM1/ADRA2A/EGFR/VEGFA/TP53/ERBB2/IFNG/IL4/MET/KDR/NCF1/MAPK3/STAT3/EGF/CAV1/IGF2/ERBB3/	19
BP	GO:0019915	lipid storage	10/187	81/18866	7.35E-09	9.30E-08	4.22E-08	PPARG/NFKBIA/PPARD/APOB/PPARA/CES1/SOAT1/CAV1/IL1B/LPL	10
BP	GO:0034644	cellular response to UV	10/187	81/18866	7.35E-09	9.30E-08	4.22E-08	PTGS2/BAX/CASP9/CHEK1/CDKN1A/TP53/MDM2/PCNA/MYC/PARP1	10
BP	GO:0071158	positive regulation of cell cycle arrest	10/187	81/18866	7.35E-09	9.30E-08	4.22E-08	BAX/CDK1/CDK2/CDKN1A/TP53/MDM2/PCNA/CCNB1/CHEK2/E2F1	10
BP	GO:0046545	development of primary female sexual characteristics	11/187	105/18866	7.49E-09	9.46E-08	4.29E-08	BCL2/BAX/CASP3/ICAM1/ESR1/VEGFA/BCL2L1/SOD1/CYP19A1/HSPA5/NOS3	11
BP	GO:0010595	positive regulation of endothelial cell migration	12/187	132/18866	7.82E-09	9.85E-08	4.47E-08	PTGS2/PRKCA/AKT1/HMOX1/VEGFA/MET/KDR/HIF1A/NOS3/HSPB1/NFE2L2/EDN1	12
BP	GO:0030193	regulation of blood coagulation	10/187	82/18866	8.30E-09	1.04E-07	4.73E-08	PRKCA/PLAU/CAV1/F3/NOS3/THBD/SERPINE1/NFE2L2/EDN1/SERPINB	10

BP	GO:0043467	regulation of generation of precursor metabolites and energy	13/187	162/18866	8.40E-09	1.05E-07	4.77E-08	NOS2/AKT1/CDK1/GSK3B/TP53/CCNB1/IFNG/IL4/PPARA/STAT3/HIF1A/IGF2/INS	13
BP	GO:0010632	regulation of epithelial cell migration	17/187	301/18866	8.68E-09	1.08E-07	4.92E-08	PTGS2/JUN/PRKCA/PPARG/AKT1/HMOX1/VEGFA/MMP9/IFNG/IL4/MET/KDR/HIF1A/NOS3/HSPB1/NFE2L2/EDN1	17
BP	GO:0033157	regulation of intracellular protein transport	16/187	264/18866	8.97E-09	1.12E-07	5.07E-08	PTGS2/BCL2/CASP8/MAPK8/CDK1/MAPK14/GSK3B/MAPK1/TP53/MDM2/ERBB2/IFNG/BAD/IL1B/E2F1/NPEPPS	16
BP	GO:1900046	regulation of hemostasis	10/187	83/18866	9.35E-09	1.16E-07	5.27E-08	PRKCA/PLAU/CAV1/F3/NOS3/THBD/SERPINE1/NFE2L2/EDN1/SERPINB	10
BP	GO:0046165	alcohol biosynthetic process	13/187	164/18866	9.74E-09	1.21E-07	5.47E-08	CYP3A4/AKR1B1/IFNG/FASN/SOD1/APOB/HMGCR/GOT1/CES1/ACACA/IL1B/SCD/HRH1	13
BP	GO:0016042	lipid catabolic process	18/187	343/18866	9.78E-09	1.21E-07	5.48E-08	AKT1/CYP3A4/CYP1A2/CYP1B1/AKR1C3/ADRA2A/CDK4/PPARD/APOB/PLB1/CYP19A1/PPARA/CES1/IL1B/SPP1/LPL/INS/FABP1	18
BP	GO:0045913	positive regulation of carbohydrate metabolic process	10/187	84/18866	1.05E-08	1.29E-07	5.88E-08	AKT1/IFNG/BAD/PPARA/EGF/HIF1A/IGF2/INS/GCG/HRH1	10
BP	GO:0036296	response to increased oxygen levels	7/187	29/18866	1.09E-08	1.34E-07	6.07E-08	PPARG/CYP1A1/CAT/CDKN1A/CDK4/CAV1/COL1A1	7
BP	GO:0009408	response to heat	13/187	166/18866	1.13E-08	1.38E-07	6.26E-08	CD14/PTGS2/AKT1/HMOX1/GSK3B/CDKN1A/MAPK1/MAPK3/SOD1/NOS3/IL1A/CXCL10/TRP	13
BP	GO:0043154	negative regulation of cysteine-type endopeptidase activity involved in apoptotic process	10/187	85/18866	1.18E-08	1.44E-07	6.55E-08	PTGS2/AKT1/DPEP1/VEGFA/MMP9/MDM2/BIRC5/XIAP/RAF1/FABP1	10

BP	GO:1901988	negative regulation of cell cycle phase transition	16/187	270/18866	1.23E-08	1.50E-07	6.81E-08	BCL2/BAX/CDK1/CDK2/CHEK1/CCND1/CDKN1A/RB1/CDK4/TP53/MDM2/PCNA/CCNB1/CCL2/CHEK2/E2F1	16
BP	GO:0045429	positive regulation of nitric oxide biosynthetic process	8/187	45/18866	1.26E-08	1.53E-07	6.93E-08	PTGS2/AKT1/ICAM1/IFNG/MMP8/IL1B/TRPV1/EDN1	8
BP	GO:1901028	regulation of mitochondrial outer membrane permeabilization involved in apoptotic signaling pathway	8/187	45/18866	1.26E-08	1.53E-07	6.93E-08	BCL2/BAX/CASP8/MAPK8/GSK3B/TP53/BAD/E2F1	8
BP	GO:0009615	response to virus	18/187	349/18866	1.28E-08	1.54E-07	7.00E-08	BCL2/RELA/IKBKB/STAT1/CYP1A1/MAPK14/BCL2L1/IL2RA/IFNG/IL4/APOB/ODC1/IL1B/DUOX2/HSPB1/CXCL10/CH	18
BP	GO:0030336	negative regulation of cell migration	18/187	350/18866	1.34E-08	1.60E-07	7.28E-08	BCL2/PPARG/DPP4/AKT1/HMOX1/CYP1B1/GSTP1/DPEP1/IL4/PPARD/CYP19A1/STAT3/GJA1/CCL2/SERPINE1/NFE2L2/IGFBP3/DRD2	18
BP	GO:0046942	carboxylic acid transport	18/187	350/18866	1.34E-08	1.60E-07	7.28E-08	NOS2/PPARG/AKT1/RXRA/PPARD/PPARA/ABCC1/AKR1C1/CES1/ACACA/GJA1/IL1B/ABCG2/DRD2/TRPV1/EDN1/BDNF/FABP1	18
BP	GO:0010952	positive regulation of peptidase activity	14/187	201/18866	1.37E-08	1.63E-07	7.42E-08	BAX/CASP9/CASP8/PPARG/MAPK14/MAPK3/BAD/STAT3/CAV1/MYC/F3/CLDN4/CTSD/PCOL	14
BP	GO:0001667	ameboidal-type cell migration	21/187	481/18866	1.39E-08	1.66E-07	7.52E-08	PTGS2/JUN/PRKCA/PPARG/DPP4/AKT1/HMOX1/CYP1B1/VEGFA/MMP9/IFNG/IL4/MET/KDR/PPARD/HIF1A/GJA1/NOS3/HSPB1/NFE2L2/EDN1	21
BP	GO:0001844	protein insertion into mitochondrial membrane involved in apoptotic signaling pathway	7/187	30/18866	1.41E-08	1.68E-07	7.61E-08	BCL2/BAX/CASP8/MAPK8/TP53/BAD/E2F1	7

BP	GO:0010885	regulation of cholesterol storage	6/187	18/18866	1.47E-08	1.75E-07	7.92E-08	PPARG/PPARD/APOB/PPARA/CES1/LPL	6
BP	GO:1904705	regulation of vascular associated smooth muscle cell proliferation	10/187	87/18866	1.49E-08	1.75E-07	7.96E-08	JUN/PPARG/HMOX1/GSTP1/CDKN1A/MMP2/MMP9/MDM2/GJA1/EDN1	10
BP	GO:1990874	vascular associated smooth muscle cell proliferation	10/187	87/18866	1.49E-08	1.75E-07	7.96E-08	JUN/PPARG/HMOX1/GSTP1/CDKN1A/MMP2/MMP9/MDM2/GJA1/EDN1	10
BP	GO:1904407	positive regulation of nitric oxide metabolic process	8/187	46/18866	1.52E-08	1.78E-07	8.10E-08	PTGS2/AKT1/ICAM1/IFNG/MMP8/IL1B/TRPV1/EDN1	8
BP	GO:0015849	organic acid transport	18/187	353/18866	1.53E-08	1.79E-07	8.12E-08	NOS2/PPARG/AKT1/RXRA/PPARD/PPARA/ABCC1/AKR1C1/CES1/ACACA/GJA1/IL1B/ABCG2/DRD2/TRPV1/EDN1/BDNF/FABP1	18
BP	GO:0050818	regulation of coagulation	10/187	88/18866	1.66E-08	1.95E-07	8.83E-08	PRKCA/PLAU/CAV1/F3/NOS3/THBD/SERPINE1/NFE2L2/EDN1/SERPINB	10
BP	GO:0032768	regulation of monooxygenase activity	9/187	66/18866	1.73E-08	2.02E-07	9.15E-08	AKT1/EGFR/IFNG/HIF1A/CAV1/IL1B/NOS3/IL1A/INS	9
BP	GO:0046824	positive regulation of nucleocytoplasmic transport	9/187	66/18866	1.73E-08	2.02E-07	9.15E-08	PTGS2/CDK1/MAPK14/GSK3B/MAPK1/TP53/MDM2/IFNG/IL1B	9
BP	GO:0042572	retinol metabolic process	8/187	47/18866	1.81E-08	2.10E-07	9.52E-08	CYP3A4/CYP1A2/CYP1A1/CYP1B1/AKR1C3/AKR1B1/PLB1/ADH1B	8
BP	GO:0046677	response to antibiotic	8/187	47/18866	1.81E-08	2.10E-07	9.52E-08	CASP9/CASP3/CASP8/CYP1A1/TP53/MDM2/SOD1/HSPA5	8
BP	GO:0042594	response to starvation	14/187	206/18866	1.87E-08	2.16E-07	9.79E-08	BCL2/JUN/PPARG/MAPK8/AKR1C3/CDKN1A/MAPK1/TP53/MAPK3/UGT1A1/PPARA/HSPA5/NFE2L2/ADM	14
BP	GO:0051099	positive regulation of binding	13/187	174/18866	1.99E-08	2.28E-07	1.04E-07	PON1/PPARG/GSK3B/MMP9/RB1/IFNG/MET/MMP8/EGF/CAV1/PARP1/BDNF/GCG	13

BP	GO:0034284	response to monosaccharide	14/187	207/18866	1.99E-08	2.28E-07	1.04E-07	PTGS2/CASP3/ICAM1/GSTP1/CAT/ADRA2A/PPARD/BAD/HMGCR/RAF1/HIF1A/GJA1/LPL/GCG	14
BP	GO:0060749	mammary gland alveolus development	6/187	19/18866	2.13E-08	2.44E-07	1.11E-07	AR/ESR1/VEGFA/CCND1/EGF/HIF1A	6
BP	GO:0061377	mammary gland lobule development	6/187	19/18866	2.13E-08	2.44E-07	1.11E-07	AR/ESR1/VEGFA/CCND1/EGF/HIF1A	6
BP	GO:0009409	response to cold	8/187	48/18866	2.16E-08	2.46E-07	1.11E-07	CASP8/PPARG/NFKBIA/FOS/CXCL10/LPL/UCP2/ADM	8
BP	GO:0001660	fever generation	5/187	10/18866	2.20E-08	2.49E-07	1.13E-07	PTGS2/IL1B/PTGER3/IL1A/TRPV1	5
BP	GO:0045428	regulation of nitric oxide biosynthetic process	9/187	68/18866	2.27E-08	2.57E-07	1.17E-07	PTGS2/AKT1/ICAM1/IFNG/MMP8/CAV1/IL1B/TRPV1/EDN1	9
BP	GO:0018107	peptidyl-threonine phosphorylation	11/187	117/18866	2.35E-08	2.66E-07	1.21E-07	BCL2/PRKCA/AKT1/MAPK8/CDK1/GSK3B/CHEK1/MAPK1/EGF/PRKCB/GCG	11
BP	GO:0006109	regulation of carbohydrate metabolic process	14/187	210/18866	2.38E-08	2.69E-07	1.22E-07	AKT1/GSK3B/TP53/IFNG/BAD/PPARA/STAT3/EGF/HIF1A/IGFBP3/IGF2/INS/GCG/HRH1	14
BP	GO:0070555	response to interleukin-1	14/187	211/18866	2.53E-08	2.84E-07	1.29E-07	PRKCA/RELA/IKBKB/ICAM1/SELE/NFKBIA/MAPK3/HIF1A/IL1B/CCL2/CXCL8/IL1A/CHUK/ED	14
BP	GO:2000146	negative regulation of cell motility	18/187	365/18866	2.55E-08	2.86E-07	1.30E-07	BCL2/PPARG/DPP4/AKT1/HMOX1/CYP1B1/GSTP1/DPEP1/IL4/PPARD/CYP19A1/STAT3/GJA1/CCL2/SERPINE1/NFE2L2/IGFBP3/DRD2	18
BP	GO:0048771	tissue remodeling	13/187	178/18866	2.61E-08	2.91E-07	1.32E-07	BAX/PRKCA/EGFR/MMP2/TP53/MDM2/CA2/HIF1A/CAV1/GJA1/NOS3/IL1A/SPP1	13
BP	GO:1901215	negative regulation of neuron death	14/187	212/18866	2.69E-08	3.00E-07	1.36E-07	BCL2/BAX/JUN/AKT1/HMOX1/GSK3B/BCL2L1/SOD1/PPARA/STAT3/HIF1A/CCL2/ERBB3/BDNF	14

BP	GO:0070371	ERK1 and ERK2 cascade	17/187	325/18866	2.69E-08	3.00E-07	1.36E-07	OPRM1/JUN/PRKCA/ICAM1/GSTP1/EGFR/MAPK1/ERBB2/KDR/MAPK3/HMGCR/EGF/MYC/IL1B/CCL2/DRD2/GCG	17
BP	GO:0046660	female sex differentiation	11/187	119/18866	2.81E-08	3.12E-07	1.42E-07	BCL2/BAX/CASP3/ICAM1/ESR1/VEGFA/BCL2L1/SOD1/CYP19A1/HSPA5/NOS3	11
BP	GO:1903035	negative regulation of response to wounding	10/187	93/18866	2.85E-08	3.15E-07	1.43E-07	ALOX5/PLAU/HMGCR/GJA1/NOS3/THBD/SERPINE1/SPP1/EDN1/SERPI	10
BP	GO:2000117	negative regulation of cysteine-type endopeptidase activity	10/187	93/18866	2.85E-08	3.15E-07	1.43E-07	PTGS2/AKT1/DPEP1/VEGFA/MMP9/MDM2/BIRC5/XIAP/RAF1/FABP1	10
BP	GO:0051767	nitric-oxide synthase biosynthetic process	6/187	20/18866	3.02E-08	3.32E-07	1.51E-07	STAT1/GSTP1/IFNG/KDR/CCL2/EDN1	6
BP	GO:0051769	regulation of nitric-oxide synthase biosynthetic process	6/187	20/18866	3.02E-08	3.32E-07	1.51E-07	STAT1/GSTP1/IFNG/KDR/CCL2/EDN1	6
BP	GO:1904035	regulation of epithelial cell apoptotic process	10/187	94/18866	3.16E-08	3.46E-07	1.57E-07	HMOX1/ICAM1/AKR1C3/IL4/CD40LG/KDR/BAD/CCL2/SERPINE1/NFE	10
BP	GO:1905954	positive regulation of lipid localization	10/187	94/18866	3.16E-08	3.46E-07	1.57E-07	PON1/NFKBIA/APOB/CYP19A1/CES1/CAV1/IL1B/SPP1/EDN1/LPL	10
BP	GO:1901991	negative regulation of mitotic cell cycle phase transition	15/187	251/18866	3.21E-08	3.49E-07	1.59E-07	BCL2/BAX/CDK1/CDK2/CCND1/CDKN1A/RB1/CDK4/TP53/MDM2/PCNA/CCNB1/CCL2/CHEK2/	15
BP	GO:1902105	regulation of leukocyte differentiation	16/187	290/18866	3.36E-08	3.65E-07	1.66E-07	JUN/CASP8/PRKCA/RB1/ERBB2/IL2RA/IFNG/IL4/CA2/BAD/SOD1/FOS/MYC/IL1B/IL1A/IRF1	16
BP	GO:0003073	regulation of systemic arterial blood pressure	10/187	95/18866	3.51E-08	3.79E-07	1.72E-07	ADRA1B/AR/CES1/GJA1/NOS3/DRD2/TRPV1/EDN1/ENPEP/ADM	10
BP	GO:0097194	execution phase of apoptosis	10/187	95/18866	3.51E-08	3.79E-07	1.72E-07	BAX/CASP9/CASP3/CASP8/AKT1/BCL2L1/TP53/CASP7/TOP2A/GCG	10
BP	GO:0042759	long-chain fatty acid biosynthetic process	7/187	34/18866	3.60E-08	3.89E-07	1.76E-07	PTGS2/CYP3A4/CYP1A2/CYP1A1/ALOX5/GSTP1/GSTM1	7

BP	GO:2001235	positive regulation of apoptotic signaling pathway	13/187	183/18866	3.62E-08	3.90E-07	1.77E-07	BCL2/BAX/CASP8/MAPK8/GSK3B/BCL2L1/MMP9/TP53/MCL1/BAD/SOD1/CAV1/E2F1	13
BP	GO:0050863	regulation of T cell activation	17/187	332/18866	3.68E-08	3.95E-07	1.79E-07	CASP3/DPP4/AKT1/VCAM1/ERBB2/IL2RA/IFNG/IL4/CD40LG/BAD/SOD1/CAV1/IL1B/CCL2/IL1A/IGF2/IRF1	17
BP	GO:0001558	regulation of cell growth	19/187	420/18866	4.06E-08	4.34E-07	1.97E-07	BCL2/PPARG/AKT1/ESR2/GSK3B/EGFR/VEGFA/CDKN1A/RB1/TP53/ERBB2/PPARD/PPARA/GJA1/SPP1/IGFBP3/EDN1/BDNF/INS	19
BP	GO:0050999	regulation of nitric-oxide synthase activity	8/187	52/18866	4.16E-08	4.43E-07	2.01E-07	AKT1/EGFR/HIF1A/CAV1/IL1B/NOS3/IL1A/INS	8
BP	GO:0071622	regulation of granulocyte chemotaxis	8/187	52/18866	4.16E-08	4.43E-07	2.01E-07	LBP/DPP4/MAPK14/MAPK1/IL4/MAPK3/CXCL8/EDN1	8
BP	GO:0051146	striated muscle cell differentiation	16/187	295/18866	4.26E-08	4.53E-07	2.06E-07	BCL2/CASP3/AKT1/CDK1/RXRA/MAPK14/VEGFA/RB1/CCNB1/IL4/PPARA/CXCL10/CHUK/IGF2/EDN1/BDNF	16
BP	GO:0014065	phosphatidylinositol 3-kinase signaling	12/187	154/18866	4.44E-08	4.71E-07	2.14E-07	AKT1/CAT/EGFR/MAPK1/ERBB2/KDR/PPARD/NCF1/MAPK3/ERBB3/EDN1/INS	12
BP	GO:0008406	gonad development	14/187	223/18866	5.07E-08	5.36E-07	2.43E-07	BCL2/BAX/CASP3/AR/ICAM1/AKR1C3/ESR1/VEGFA/CCND1/BCL2L1/SOD1/CYP19A1/HSPA5/N[illegible]	14
BP	GO:0018210	peptidyl-threonine modification	11/187	126/18866	5.10E-08	5.38E-07	2.44E-07	BCL2/PRKCA/AKT1/MAPK8/CDK1/GSK3B/CHEK1/MAPK1/EGF/PRKCB/GCG	11
BP	GO:0001894	tissue homeostasis	15/187	261/18866	5.37E-08	5.65E-07	2.57E-07	PTGS2/BCL2/BAX/PRKCA/AKR1B1/EGFR/VEGFA/RB1/CA2/SOD1/ACACA/GJA1/NOS3/HSPB1/[illegible]	15

BP	GO:0043627	response to estrogen	9/187	75/18866	5.45E-08	5.72E-07	2.59E-07	AR/PPARG/HMOX1/ESR1/CCND1/MAPK1/MDM2/CA2/CAV1	9
BP	GO:0006749	glutathione metabolic process	8/187	54/18866	5.65E-08	5.92E-07	2.69E-07	GSTP1/GSTM1/DPEP1/SOD1/GSR/NFE2L2/GSTA1/GSTA2	8
BP	GO:0031349	positive regulation of defense response	18/187	385/18866	5.73E-08	5.99E-07	2.72E-07	LBP/PTGS2/RELA/IKBKB/EGFR/NFKBIA/IFNG/MMP8/MAPK3/ABCC1/STAT3/RAF1/GJA1/IL1B/PTGER3/SERPINE1/CH	18
BP	GO:0050729	positive regulation of inflammatory response	12/187	158/18866	5.90E-08	6.15E-07	2.79E-07	LBP/PTGS2/EGFR/NFKBIA/IFNG/MMP8/ABCC1/STAT3/IL1B/PTGER3/SERPINE1/LPL	12
BP	GO:0045637	regulation of myeloid cell differentiation	15/187	263/18866	5.94E-08	6.16E-07	2.80E-07	JUN/CASP8/PRKCA/STAT1/MAPK14/RB1/NFKBIA/IFNG/IL4/CA2/STAT3/FOS/HIF1A/MYC/PR	15
BP	GO:0050730	regulation of peptidyl-tyrosine phosphorylation	15/187	263/18866	5.94E-08	6.16E-07	2.80E-07	IL6R/ICAM1/ADRA2A/EGFR/VEGFA/TP53/IFNG/IL4/NCF1/STAT3/EGF/CAV1/IGF2/ERBB3/BDNF	15
BP	GO:0032869	cellular response to insulin stimulus	14/187	226/18866	5.99E-08	6.20E-07	2.81E-07	PPARG/RELA/AKT1/STAT1/GSTP1/GSK3B/CDK4/GOT1/IL1B/PRKCB/PARP1/IGF2/LPL/INS	14
BP	GO:1903522	regulation of blood circulation	16/187	303/18866	6.18E-08	6.37E-07	2.89E-07	PTGS2/ADRA1B/AKT1/ICAM1/ADRA2A/EGFR/MDM2/CAV1/GJA1/NOS3/DRD2/TRPV1/EDN1/ADM/HRH1/CACNA1S	16
BP	GO:0043406	positive regulation of MAP kinase activity	15/187	264/18866	6.24E-08	6.43E-07	2.92E-07	CDK1/ADRA2A/MAPK14/EGFR/VEGFA/MAPK1/ERBB2/CD40LG/MAPK10/MAPK3/SOD1/EGF/RAF1/IL1B/EDN1	15
BP	GO:0051092	positive regulation of NF-kappaB transcription factor activity	12/187	159/18866	6.33E-08	6.47E-07	2.94E-07	AR/RELA/IKBKB/ICAM1/CAT/CD40LG/STAT3/CAV1/IL1B/PRKCB/CHUK/INS	12

BP	GO:1902107	positive regulation of leukocyte differentiation	12/187	159/18866	6.33E-08	6.47E-07	2.94E-07	JUN/CASP8/PRKCA/RB1/IL2RA/IFNG/IL4/CA2/BAD/FOS/IL1B/IL1A	12
BP	GO:0015908	fatty acid transport	10/187	101/18866	6.33E-08	6.47E-07	2.94E-07	NOS2/PPARG/AKT1/PPARD/PPARA/ACACA/IL1B/DRD2/EDN1/FABP1	10
BP	GO:0097345	mitochondrial outer membrane permeabilization	8/187	55/18866	6.56E-08	6.69E-07	3.04E-07	BCL2/BAX/CASP8/MAPK8/GSK3B/TP53/BAD/E	8
BP	GO:0042692	muscle cell differentiation	18/187	390/18866	6.97E-08	7.09E-07	3.22E-07	BCL2/CASP3/AKT1/CDK1/RXRA/MAPK14/VEGFA/RB1/MDM2/CCNB1/IL4/PPARA/CXCL10/CHUK/IGF2/EDN1/BDNF/A	18
BP	GO:0045137	development of primary sexual characteristics	14/187	229/18866	7.06E-08	7.17E-07	3.25E-07	BCL2/BAX/CASP3/AR/ICAM1/AKR1C3/ESR1/VEGFA/CCND1/BCL2L1/SOD1/CYP19A1/HSPA5/N	14
BP	GO:0042063	gliogenesis	16/187	307/18866	7.40E-08	7.49E-07	3.40E-07	PPARG/RELA/AKT1/CDK1/GSTP1/EGFR/MAPK1/ERBB2/IFNG/MAPK3/SOD1/STAT3/IL1B/CCL2/E2F1/ERBB3	16
BP	GO:0010001	glial cell differentiation	14/187	230/18866	7.46E-08	7.53E-07	3.42E-07	PPARG/RELA/AKT1/CDK1/GSTP1/EGFR/MAPK1/ERBB2/IFNG/MAPK3/SOD1/STAT3/IL1B/ERB	14
BP	GO:0051051	negative regulation of transport	20/187	483/18866	7.49E-08	7.54E-07	3.42E-07	PTGS2/OPRM1/BCL2/AKT1/HMOX1/ICAM1/ADRA2A/MMP9/HMGCR/EGF/CAV1/GJA1/IL1B/PTGER3/PRKCB/NOS3/ERBB3/DRD2/EDN1/INS	20
BP	GO:0015850	organic hydroxy compound transport	15/187	268/18866	7.61E-08	7.61E-07	3.46E-07	SLC6A4/PON1/PPARG/RXRA/ADRA2A/NFKBIA/APOB/CYP19A1/AKR1C1/CES1/SOAT1/EGF/CAV1/SPP1/DRD2	15
BP	GO:0046887	positive regulation of hormone secretion	11/187	131/18866	7.64E-08	7.61E-07	3.46E-07	EGFR/PPARD/BAD/CYP19A1/HIF1A/GJA1/SPP1/DRD2/EDN1/INS/GCG	11

BP	GO:0030593	neutrophil chemotaxis	10/187	103/18866	7.64E-08	7.61E-07	3.46E-07	LBP/DPP4/DPEP1/IL1B/CCL2/CXCL8/CXCL11/CXCL2/CXCL10/EDN1	10
BP	GO:1901570	fatty acid derivative biosynthetic process	10/187	103/18866	7.64E-08	7.61E-07	3.46E-07	PTGS1/PTGS2/ALOX5/AKR1C3/PTGES/FASN/ACACA/IL1B/EDN1/SCD	10
BP	GO:0050731	positive regulation of peptidyl-tyrosine phosphorylation	13/187	195/18866	7.67E-08	7.61E-07	3.46E-07	IL6R/ICAM1/ADRA2A/VEGFA/TP53/IFNG/IL4/NCF1/STAT3/EGF/IGF2/ERBB3/BDNF	13
BP	GO:1905475	regulation of protein localization to membrane	13/187	195/18866	7.67E-08	7.61E-07	3.46E-07	BCL2/CASP8/AR/AKT1/MAPK8/EGFR/BCL2L1/TP53/ERBB2/IFNG/BAD/E2F1/INS	13
BP	GO:1901522	positive regulation of transcription from RNA polymerase II promoter involved in cellular response to chemical stimulus	6/187	23/18866	7.68E-08	7.61E-07	3.46E-07	RELA/VEGFA/TP53/HIF1A/NFE2L2/RUNX2	6
BP	GO:0061045	negative regulation of wound healing	9/187	78/18866	7.71E-08	7.63E-07	3.46E-07	ALOX5/PLAU/HMGCR/GJA1/NOS3/THBD/SERPINE1/EDN1/SERPINB2	9
BP	GO:0010742	macrophage derived foam cell differentiation	7/187	38/18866	8.18E-08	8.05E-07	3.65E-07	PPARG/STAT1/NFKBIA/APOB/PPARA/SOAT1/LPL	7
BP	GO:0090077	foam cell differentiation	7/187	38/18866	8.18E-08	8.05E-07	3.65E-07	PPARG/STAT1/NFKBIA/APOB/PPARA/SOAT1/LPL	7
BP	GO:0040013	negative regulation of locomotion	18/187	397/18866	9.10E-08	8.94E-07	4.06E-07	BCL2/PPARG/DPP4/AKT1/HMOX1/CYP1B1/GSTP1/DPEP1/IL4/PPARD/CYP19A1/STAT3/GJA1/CCL2/SERPINE1/NFE2L2/IGFBP3/DRD2	18
BP	GO:0043112	receptor metabolic process	13/187	198/18866	9.17E-08	8.99E-07	4.08E-07	PPARG/ACHE/SELE/VEGFA/IFNG/PPARA/EGF/HIF1A/CAV1/CXCL8/DRD2/EDN1/ADM	13
BP	GO:0042391	regulation of membrane potential	19/187	443/18866	9.37E-08	9.17E-07	4.16E-07	OPRM1/BCL2/BAX/JUN/AKT1/GSK3B/BCL2L1/HTR3A/KDR/BAD/SOD1/GOT1/CAV1/GJA1/PARP1/DRD2/TRPV1/EDN1/B	19

BP	GO:0032091	negative regulation of protein binding	10/187	106/18866	1.01E-07	9.82E-07	4.46E-07	BAX/AKT1/MAPK8/SLPI/GSK3B/CDKN1A/MET/MAPK3/PPARA/CAV1	10
BP	GO:0045927	positive regulation of growth	15/187	274/18866	1.02E-07	9.89E-07	4.49E-07	BCL2/AKT1/CDK1/MAPK14/EGFR/VEGFA/MAPK1/ERBB2/CCNB1/PPARD/IGF2/DRD2/EDN1/BDNF/INS	15
BP	GO:0051271	negative regulation of cellular component movement	18/187	400/18866	1.02E-07	9.89E-07	4.49E-07	BCL2/PPARG/DPP4/AKT1/HMOX1/CYP1B1/GSTP1/DPEP1/IL4/PPARD/CYP19A1/STAT3/GJA1/CCL2/SERPINE1/NFE2L2/IGFBP3/DRD2	18
BP	GO:0032368	regulation of lipid transport	11/187	135/18866	1.04E-07	1.01E-06	4.58E-07	PON1/PPARG/AKT1/NFKBIA/CYP19A1/CES1/EGF/CAV1/IL1B/SPP1/ED	11
BP	GO:0032386	regulation of intracellular transport	17/187	358/18866	1.09E-07	1.06E-06	4.79E-07	PTGS2/BCL2/CASP8/MAPK8/CDK1/MAPK14/GSK3B/MAPK1/TP53/MDM2/ERBB2/IFNG/MAPK3/BAD/IL1B/E2F1/NPEPPS	17
BP	GO:0010623	programmed cell death involved in cell development	5/187	13/18866	1.09E-07	1.06E-06	4.79E-07	BCL2/BAX/IL1B/IL1A/BDNF	5
BP	GO:0030330	DNA damage response, signal transduction by p53 class mediator	10/187	107/18866	1.10E-07	1.06E-06	4.81E-07	BAX/CDK1/CDK2/CDKN1A/TP53/MDM2/PCNA/CCNB1/CHEK2/E2F1	10
BP	GO:0034504	protein localization to nucleus	15/187	277/18866	1.17E-07	1.12E-06	5.10E-07	PTGS2/AKT1/CDK1/MAPK14/GSK3B/CDKN1A/MAPK1/TP53/NFKBIA/MDM2/IFNG/STAT3/COL1A1/PARP1/INS	15
BP	GO:1902930	regulation of alcohol biosynthetic process	9/187	82/18866	1.20E-07	1.15E-06	5.21E-07	IFNG/FASN/SOD1/APOB/HMGCR/ACACA/IL1B/SCD/HRH1	9
BP	GO:0043491	protein kinase B signaling	15/187	278/18866	1.23E-07	1.17E-06	5.32E-07	AKT1/AKR1C3/ESR1/EGFR/ERBB2/MET/KDR/EGF/F3/IL1B/CCL2/IGF2/ERBB3/DRD2/INS	15
BP	GO:0033138	positive regulation of peptidyl-serine phosphorylation	10/187	109/18866	1.31E-07	1.25E-06	5.68E-07	PTGS2/BCL2/AKT1/EGFR/VEGFA/IFNG/RAF1/CAV1/BDNF/GCG	10

BP	GO:0001836	release of cytochrome c from mitochondria	8/187	60/18866	1.32E-07	1.25E-06	5.68E-07	BCL2/BAX/JUN/AKT1/BCL2L1/MMP9/TP53/BA	8
BP	GO:0097755	positive regulation of blood vessel diameter	8/187	60/18866	1.32E-07	1.25E-06	5.68E-07	HMOX1/EGFR/PPARD/SOD1/GJA1/NOS3/INS/A	8
BP	GO:1903428	positive regulation of reactive oxygen species biosynthetic process	8/187	60/18866	1.32E-07	1.25E-06	5.68E-07	PTGS2/AKT1/ICAM1/IFNG/MMP8/IL1B/TRPV1/EDN1	8
BP	GO:0046697	decidualization	6/187	25/18866	1.33E-07	1.25E-06	5.69E-07	PTGS2/MAPK1/PPARD/MAPK3/GJA1/SPP1	6
BP	GO:0050714	positive regulation of protein secretion	12/187	172/18866	1.50E-07	1.42E-06	6.43E-07	CD14/ACHE/EGFR/IFNG/PPARD/BAD/HIF1A/GJA1/IL1A/DRD2/INS/GCG	12
BP	GO:1902041	regulation of extrinsic apoptotic signaling pathway via death domain receptors	8/187	61/18866	1.51E-07	1.42E-06	6.43E-07	CASP8/HMOX1/ICAM1/GSK3B/BCL2L1/RAF1/NOS3/SERPINE1	8
BP	GO:1902110	positive regulation of mitochondrial membrane permeability involved in apoptotic process	8/187	61/18866	1.51E-07	1.42E-06	6.43E-07	BCL2/BAX/CASP8/MAPK8/GSK3B/TP53/BAD/E2F1	8
BP	GO:0046651	lymphocyte proliferation	15/187	283/18866	1.55E-07	1.45E-06	6.58E-07	BCL2/BAX/CASP3/VCAM1/AHR/CDKN1A/TP53/ERBB2/IL2RA/IL4/CD40LG/IL1B/IL1A/IGF2/IRF	15
BP	GO:0090399	replicative senescence	5/187	14/18866	1.69E-07	1.58E-06	7.17E-07	CHEK1/CDKN1A/TP53/SERPINE1/CHEK2	5
BP	GO:0035902	response to immobilization stress	6/187	26/18866	1.71E-07	1.58E-06	7.19E-07	PPARG/CYP1A2/CYP1A1/MDM2/GOT1/FOS	6
BP	GO:1900739	regulation of protein insertion into mitochondrial membrane involved in apoptotic signaling pathway	6/187	26/18866	1.71E-07	1.58E-06	7.19E-07	BCL2/CASP8/MAPK8/TP53/BAD/E2F1	6
BP	GO:1900740	positive regulation of protein insertion into mitochondrial membrane involved in apoptotic	6/187	26/18866	1.71E-07	1.58E-06	7.19E-07	BCL2/CASP8/MAPK8/TP53/BAD/E2F1	6
BP	GO:0071384	cellular response to corticosteroid stimulus	8/187	62/18866	1.72E-07	1.58E-06	7.19E-07	CASP9/ICAM1/GSTP1/AKR1C3/EGFR/NR3C1/UGT1A1/EDN1	8
BP	GO:2000351	regulation of endothelial cell apoptotic process	8/187	62/18866	1.72E-07	1.58E-06	7.19E-07	ICAM1/AKR1C3/IL4/CD40LG/KDR/CCL2/SERPINE1/NFE2L2	8
BP	GO:2001244	positive regulation of intrinsic apoptotic signaling pathway	8/187	62/18866	1.72E-07	1.58E-06	7.19E-07	BCL2/BAX/BCL2L1/TP53/MCL1/BAD/SOD1/CA	8

BP	GO:0007006	mitochondrial membrane organization	11/187	142/18866	1.75E-07	1.61E-06	7.30E-07	BCL2/BAX/CASP8/MAPK8/GSK3B/BCL2L1/TP53/BAD/STAT3/E2F1/HK	11
BP	GO:0007249	I-kappaB kinase/NF-kappaB signaling	15/187	286/18866	1.77E-07	1.62E-06	7.35E-07	CD14/CASP8/RELA/IKBKB/AKT1/STAT1/HMOX1/GSTP1/ESR1/NFKBIA/GJA1/IL1B/PRKCB/HSPB1/CHUK	15
BP	GO:0032943	mononuclear cell proliferation	15/187	286/18866	1.77E-07	1.62E-06	7.35E-07	BCL2/BAX/CASP3/VCAM1/AHR/CDKN1A/TP53/ERBB2/IL2RA/IL4/CD40LG/IL1B/IL1A/IGF2/IRF	15
BP	GO:0043542	endothelial cell migration	15/187	286/18866	1.77E-07	1.62E-06	7.35E-07	PTGS2/PRKCA/PPARG/DPP4/AKT1/HMOX1/CYP1B1/VEGFA/MET/KDR/HIF1A/NOS3/HSPB1/NFE2L2/EDN1	15
BP	GO:0140014	mitotic nuclear division	15/187	286/18866	1.77E-07	1.62E-06	7.35E-07	PRKCA/CDK1/CHEK1/RB1/BIRC5/CCNB1/XIAP/EGF/IL1B/PRKCB/IL1A/CHEK2/IGF2/EDN1/INS	15
BP	GO:0001938	positive regulation of endothelial cell proliferation	10/187	113/18866	1.85E-07	1.68E-06	7.64E-07	JUN/PRKCA/AKT1/HMOX1/VEGFA/KDR/STAT3/HIF1A/F3/IGF2	10
BP	GO:0043393	regulation of protein binding	13/187	211/18866	1.92E-07	1.75E-06	7.94E-07	BAX/AKT1/MAPK8/SLPI/GSK3B/CDKN1A/MMP9/MET/MAPK3/PPARA/CAV1/BDNF/GCG	13
BP	GO:1902686	mitochondrial outer membrane permeabilization involved in programmed cell death	8/187	63/18866	1.95E-07	1.77E-06	8.03E-07	BCL2/BAX/CASP8/MAPK8/GSK3B/TP53/BAD/E2F1	8
BP	GO:0046688	response to copper ion	7/187	43/18866	2.00E-07	1.81E-06	8.23E-07	CDK1/CYP1A2/CYP1A1/ICAM1/SOD1/IL1A/NFE2L2	7
BP	GO:0033135	regulation of peptidyl-serine phosphorylation	11/187	145/18866	2.16E-07	1.95E-06	8.86E-07	PTGS2/BCL2/BAX/AKT1/EGFR/VEGFA/IFNG/RAF1/CAV1/BDNF/GCG	11
BP	GO:0006006	glucose metabolic process	13/187	214/18866	2.26E-07	2.04E-06	9.25E-07	AKT1/MAPK14/GSK3B/TP53/PPARD/BAD/PPARA/GOT1/IGFBP3/IGF2/HK2/INS/GCG	13

BP	GO:0050870	positive regulation of T cell activation	13/187	214/18866	2.26E-07	2.04E-06	9.25E-07	DPP4/AKT1/VCAM1/IL2RA/IFNG/IL4/CD40LG/BAD/CAV1/IL1B/CCL2/IL1A/IGF2	13
BP	GO:0046620	regulation of organ growth	10/187	116/18866	2.37E-07	2.12E-06	9.64E-07	SLC6A4/AKT1/CDK1/MAPK14/MAPK1/CCNB1/SOD1/PPARA/GJA1/ED	10
BP	GO:0051924	regulation of calcium ion transport	14/187	253/18866	2.41E-07	2.16E-06	9.81E-07	PTGS2/BCL2/BAX/ICAM1/ADRA2A/EGF/CAV1/GJA1/CCL2/NOS3/CXCL11/CXCL10/DRD2/GCG	14
BP	GO:0051592	response to calcium ion	11/187	147/18866	2.48E-07	2.22E-06	1.01E-06	JUN/AKR1C3/DPEP1/EGFR/CCND1/PTGES/BAD/FOS/HSPA5/CAV1/EDN	11
BP	GO:0035794	positive regulation of mitochondrial membrane permeability	8/187	65/18866	2.50E-07	2.23E-06	1.01E-06	BCL2/BAX/CASP8/MAPK8/GSK3B/TP53/BAD/E	8
BP	GO:0051205	protein insertion into membrane	8/187	65/18866	2.50E-07	2.23E-06	1.01E-06	BCL2/BAX/CASP8/MAPK8/EGFR/TP53/BAD/E2	8
BP	GO:0019318	hexose metabolic process	14/187	254/18866	2.53E-07	2.25E-06	1.02E-06	AKT1/AKR1B1/MAPK14/GSK3B/TP53/PPARD/BAD/PPARA/GOT1/IGFBP3/IGF2/HK2/INS/GCG	14
BP	GO:0051341	regulation of oxidoreductase activity	10/187	117/18866	2.56E-07	2.27E-06	1.03E-06	AKT1/EGFR/IFNG/HIF1A/CAV1/IL1B/NOS3/IL1A/EDN1/INS	10
BP	GO:0006816	calcium ion transport	18/187	426/18866	2.59E-07	2.29E-06	1.04E-06	PTGS2/OPRM1/BCL2/BAX/ICAM1/ADRA2A/EGF/CAV1/GJA1/CCL2/PRKCB/NOS3/CXCL11/CXCL10/DRD2/TRPV1/GCG/CACNA1S	18
BP	GO:0010675	regulation of cellular carbohydrate metabolic process	11/187	148/18866	2.66E-07	2.35E-06	1.07E-06	AKT1/GSK3B/TP53/BAD/PPARA/STAT3/IGFBP3/IGF2/INS/GCG/HRH1	11
BP	GO:0032800	receptor biosynthetic process	6/187	28/18866	2.75E-07	2.42E-06	1.10E-06	PPARG/ACHE/IFNG/PPARA/HIF1A/EDN1	6
BP	GO:0005996	monosaccharide metabolic process	15/187	296/18866	2.76E-07	2.43E-06	1.10E-06	AKT1/AKR1B1/MAPK14/GSK3B/TP53/PPARD/BAD/UGT1A1/PPARA/GOT1/IGFBP3/IGF2/HK2/INS/GCG	15

BP	GO:0051204	protein insertion into mitochondrial membrane	7/187	45/18866	2.77E-07	2.43E-06	1.10E-06	BCL2/BAX/CASP8/MAPK8/TP53/BAD/E2F1	7
BP	GO:0007569	cell aging	10/187	118/18866	2.78E-07	2.43E-06	1.10E-06	BCL2/CDK1/ICAM1/MAPK14/CHEK1/CDKN1A/TP53/SOD1/SERPINE1/CHEK2	10
BP	GO:0048872	homeostasis of number of cells	14/187	256/18866	2.78E-07	2.43E-06	1.10E-06	BCL2/BAX/CASP3/AKT1/STAT1/HMOX1/MAPK14/VEGFA/RB1/IL2RA/SOD1/STAT3/HIF1A/NOS	14
BP	GO:1902108	regulation of mitochondrial membrane permeability involved in apoptotic process	8/187	66/18866	2.82E-07	2.46E-06	1.11E-06	BCL2/BAX/CASP8/MAPK8/GSK3B/TP53/BAD/E2F1	8
BP	GO:2000378	negative regulation of reactive oxygen species metabolic process	8/187	66/18866	2.82E-07	2.46E-06	1.11E-06	BCL2/TP53/STAT3/MMP3/HIF1A/CAV1/HK2/INS	8
BP	GO:0071347	cellular response to interleukin-1	12/187	183/18866	2.95E-07	2.56E-06	1.16E-06	RELA/IKBKB/ICAM1/NFKBIA/MAPK3/HIF1A/IL1B/CCL2/CXCL8/IL1A/CHUK/EDN1	12
BP	GO:0050670	regulation of lymphocyte proliferation	13/187	219/18866	2.95E-07	2.56E-06	1.16E-06	BCL2/CASP3/VCAM1/AHR/CDKN1A/ERBB2/IL2RA/IL4/CD40LG/IL1B/IL1A/IGF2/IRF1	13
BP	GO:0070665	positive regulation of leukocyte proliferation	11/187	150/18866	3.04E-07	2.63E-06	1.20E-06	BCL2/VCAM1/CDKN1A/MAPK1/IL2RA/IL4/CD40LG/MAPK3/IL1B/IL1A/IGF2	11
BP	GO:1905710	positive regulation of membrane permeability	8/187	67/18866	3.17E-07	2.74E-06	1.24E-06	BCL2/BAX/CASP8/MAPK8/GSK3B/TP53/BAD/E	8
BP	GO:0032944	regulation of mononuclear cell proliferation	13/187	221/18866	3.28E-07	2.83E-06	1.28E-06	BCL2/CASP3/VCAM1/AHR/CDKN1A/ERBB2/IL2RA/IL4/CD40LG/IL1B/IL1A/IGF2/IRF1	13
BP	GO:0072577	endothelial cell apoptotic process	8/187	68/18866	3.57E-07	3.07E-06	1.39E-06	ICAM1/AKR1C3/IL4/CD40LG/KDR/CCL2/SERPINE1/NFE2L2	8
BP	GO:0045834	positive regulation of lipid metabolic process	11/187	153/18866	3.72E-07	3.19E-06	1.45E-06	PTGS2/PPARG/AKT1/IFNG/PPARD/PPARA/CES1/IL1B/INS/FABP1/ADM	11

BP	GO:0048639	positive regulation of developmental growth	12/187	187/18866	3.72E-07	3.19E-06	1.45E-06	BCL2/AKT1/CDK1/MAPK14/VEGFA/MAPK1/CCNB1/PPARD/IGF2/DRD2/EDN1/BDNF	12
BP	GO:0030225	macrophage differentiation	7/187	47/18866	3.78E-07	3.22E-06	1.46E-06	CASP8/PRKCA/VEGFA/MMP9/RB1/IFNG/PARP	7
BP	GO:0030574	collagen catabolic process	7/187	47/18866	3.78E-07	3.22E-06	1.46E-06	MMP1/MMP2/MMP9/MMP13/MMP8/MMP3/CTS	7
BP	GO:0070849	response to epidermal growth factor	7/187	47/18866	3.78E-07	3.22E-06	1.46E-06	AKT1/GSTP1/EGFR/MAPK1/ERBB2/MAPK3/COL1A1	7
BP	GO:1990266	neutrophil migration	10/187	122/18866	3.80E-07	3.23E-06	1.46E-06	LBP/DPP4/DPEP1/IL1B/CCL2/CXCL8/CXCL11/CXCL2/CXCL10/EDN1	10
BP	GO:1901987	regulation of cell cycle phase transition	19/187	486/18866	3.90E-07	3.31E-06	1.50E-06	BCL2/BAX/AKT1/CDK1/CYP1A1/CDK2/CHEK1/EGFR/CCND1/CDKN1A/RB1/CDK4/TP53/MDM2/PCNA/CCNB1/CCL2/CHEK2/E2F1	19
BP	GO:0034308	primary alcohol metabolic process	9/187	94/18866	3.92E-07	3.31E-06	1.50E-06	CYP3A4/CYP1A2/CYP1A1/CYP1B1/AKR1C3/AKR1B1/PLB1/AKR1C1/A	9
BP	GO:0051591	response to cAMP	9/187	94/18866	3.92E-07	3.31E-06	1.50E-06	RELA/STAT1/AHR/TYR/FOS/HSPA5/DUOX2/THBD/COL1A1	9
BP	GO:1901992	positive regulation of mitotic cell cycle phase transition	9/187	94/18866	3.92E-07	3.31E-06	1.50E-06	AKT1/CDK1/CYP1A1/EGFR/CCND1/RB1/CDK4/MDM2/CCNB1	9
BP	GO:0048662	negative regulation of smooth muscle cell proliferation	8/187	69/18866	4.00E-07	3.37E-06	1.53E-06	PPARG/HMOX1/GSTP1/CDKN1A/IFNG/PPARD/NOS3/IGFBP3	8
BP	GO:0001516	prostaglandin biosynthetic process	6/187	30/18866	4.26E-07	3.56E-06	1.62E-06	PTGS1/PTGS2/AKR1C3/PTGES/IL1B/EDN1	6
BP	GO:0043457	regulation of cellular respiration	6/187	30/18866	4.26E-07	3.56E-06	1.62E-06	NOS2/CDK1/CCNB1/IFNG/IL4/HIF1A	6
BP	GO:0046457	prostanoid biosynthetic process	6/187	30/18866	4.26E-07	3.56E-06	1.62E-06	PTGS1/PTGS2/AKR1C3/PTGES/IL1B/EDN1	6
BP	GO:0071480	cellular response to gamma radiation	6/187	30/18866	4.26E-07	3.56E-06	1.62E-06	BCL2L1/CDKN1A/TP53/MDM2/HSPA5/CHEK2	6

BP	GO:0030198	extracellular matrix organization	17/187	395/18866	4.39E-07	3.66E-06	1.66E-06	DPP4/PRSS1/MMP1/ICAM1/VCAM1/CYP1B1/MMP2/MMP9/RB1/MMP13/MMP8/KDR/MMP3/CAV1/SERPINE1/COL1A1/	17
BP	GO:0051048	negative regulation of secretion	12/187	190/18866	4.41E-07	3.67E-06	1.66E-06	OPRM1/HMOX1/ADRA2A/HMGCR/EGF/GJA1/IL1B/PTGER3/ERBB3/DRD2/EDN1/INS	12
BP	GO:0016049	cell growth	19/187	490/18866	4.42E-07	3.67E-06	1.66E-06	BCL2/PPARG/AKT1/ESR2/GSK3B/EGFR/VEGFA/CDKN1A/RB1/TP53/ERBB2/PPARD/PPARA/GJA1/SPP1/IGFBP3/EDN1/BDNF/INS	19
BP	GO:0032370	positive regulation of lipid transport	8/187	70/18866	4.48E-07	3.71E-06	1.69E-06	PON1/NFKBIA/CYP19A1/CES1/CAV1/IL1B/SPP1/EDN1	8
BP	GO:0043062	extracellular structure organization	17/187	396/18866	4.54E-07	3.76E-06	1.71E-06	DPP4/PRSS1/MMP1/ICAM1/VCAM1/CYP1B1/MMP2/MMP9/RB1/MMP13/MMP8/KDR/MMP3/CAV1/SERPINE1/COL1A1/	17
BP	GO:0072331	signal transduction by p53 class mediator	14/187	267/18866	4.63E-07	3.83E-06	1.74E-06	BCL2/BAX/AKT1/CDK1/MAPK14/CDK2/CHEK1/CDKN1A/TP53/MDM2/PCNA/CCNB1/CHEK2/E2	14
BP	GO:0042176	regulation of protein catabolic process	17/187	397/18866	4.71E-07	3.88E-06	1.76E-06	NOS2/RELA/AKT1/ADRA2A/GSK3B/EGFR/MDM2/IFNG/HMGCR/EGF/ODC1/CAV1/GJA1/IL1B/NFE2L2/CHEK2/INS	17
BP	GO:1903578	regulation of ATP metabolic process	10/187	125/18866	4.76E-07	3.92E-06	1.78E-06	CDK1/TP53/CCNB1/IFNG/IL4/PPARA/STAT3/HIF1A/PARP1/INS	10
BP	GO:0048015	phosphatidylinositol-mediated signaling	12/187	192/18866	4.94E-07	4.05E-06	1.84E-06	AKT1/CAT/EGFR/MAPK1/ERBB2/KDR/PPARD/NCF1/MAPK3/ERBB3/EDN1/INS	12
BP	GO:0002673	regulation of acute inflammatory response	7/187	49/18866	5.08E-07	4.15E-06	1.88E-06	PTGS2/PPARG/GSTP1/IL4/IL1B/PTGER3/INS	7

BP	GO:0090151	establishment of protein localization to mitochondrial membrane	7/187	49/18866	5.08E-07	4.15E-06	1.88E-06	BCL2/BAX/CASP8/MAPK8/TP53/BAD/E2F1	7
BP	GO:0031649	heat generation	5/187	17/18866	5.09E-07	4.15E-06	1.88E-06	PTGS2/IL1B/PTGER3/IL1A/TRPV1	5
BP	GO:0050665	hydrogen peroxide biosynthetic process	5/187	17/18866	5.09E-07	4.15E-06	1.88E-06	CYP1A2/CYP1A1/SOD1/STAT3/DUOX2	5
BP	GO:0030168	platelet activation	11/187	158/18866	5.13E-07	4.17E-06	1.89E-06	PRKCA/ADRA2A/MAPK1/CD40LG/MAPK3/RAF1/PRKCB/NOS3/HSPB1/THBD/COL1A1	11
BP	GO:0002793	positive regulation of peptide secretion	12/187	193/18866	5.22E-07	4.24E-06	1.92E-06	CD14/ACHE/EGFR/IFNG/PPARD/BAD/HIF1A/GJA1/IL1A/DRD2/INS/GCG	12
BP	GO:1901990	regulation of mitotic cell cycle phase transition	18/187	448/18866	5.40E-07	4.38E-06	1.99E-06	BCL2/BAX/AKT1/CDK1/CYP1A1/CDK2/EGFR/CCND1/CDKN1A/RB1/CDK4/TP53/MDM2/PCNA/CCNB1/CCL2/CHEK2/E2	18
BP	GO:1905477	positive regulation of protein localization to membrane	10/187	127/18866	5.52E-07	4.46E-06	2.03E-06	BCL2/CASP8/AKT1/MAPK8/EGFR/TP53/ERBB2/IFNG/BAD/E2F1	10
BP	GO:0006839	mitochondrial transport	14/187	271/18866	5.54E-07	4.47E-06	2.03E-06	BCL2/BAX/CASP8/MAPK8/GSK3B/BCL2L1/TP53/BAD/STAT3/ACACA/E2F1/NPEPPS/HK2/UCP2	14
BP	GO:0042098	T cell proliferation	12/187	195/18866	5.83E-07	4.69E-06	2.13E-06	BAX/CASP3/VCAM1/TP53/ERBB2/IL2RA/IL4/CD40LG/IL1B/IL1A/IGF2/	12
BP	GO:0045981	positive regulation of nucleotide metabolic process	7/187	50/18866	5.85E-07	4.69E-06	2.13E-06	NOS2/IFNG/IL4/STAT3/HIF1A/NOS3/INS	7
BP	GO:1900544	positive regulation of purine nucleotide metabolic process	7/187	50/18866	5.85E-07	4.69E-06	2.13E-06	NOS2/IFNG/IL4/STAT3/HIF1A/NOS3/INS	7
BP	GO:1903580	positive regulation of ATP metabolic process	7/187	50/18866	5.85E-07	4.69E-06	2.13E-06	CDK1/CCNB1/IFNG/IL4/STAT3/HIF1A/INS	7
BP	GO:0010948	negative regulation of cell cycle process	16/187	359/18866	6.13E-07	4.90E-06	2.22E-06	BCL2/BAX/CDK1/CDK2/CHEK1/CCND1/CDKN1A/RB1/CDK4/TP53/MDM2/PCNA/CCNB1/CCL2/CHEK2/E2F1	16

BP	GO:0048017	inositol lipid-mediated signaling	12/187	196/18866	6.16E-07	4.90E-06	2.23E-06	AKT1/CAT/EGFR/MAPK1/ERBB2/KDR/PPARD/NCF1/MAPK3/ERBB3/EDN1/INS	12
BP	GO:0050728	negative regulation of inflammatory response	12/187	196/18866	6.16E-07	4.90E-06	2.23E-06	TNFAIP6/PPARG/ALOX5/GSTP1/RB1/IL2RA/IL4/PPARD/SOD1/CYP19A1/PPARA/INS	12
BP	GO:0071482	cellular response to light stimulus	10/187	129/18866	6.38E-07	5.07E-06	2.30E-06	PTGS2/BAX/CASP9/CHEK1/CDKN1A/TP53/MDM2/PCNA/MYC/PARP1	10
BP	GO:0008210	estrogen metabolic process	6/187	32/18866	6.40E-07	5.07E-06	2.30E-06	CYP3A4/CYP1A2/CYP1A1/CYP1B1/CYP19A1/UGT1A1	6
BP	GO:0034694	response to prostaglandin	6/187	32/18866	6.40E-07	5.07E-06	2.30E-06	PPARG/AKT1/AKR1C3/APOB/ACACA/EDN1	6
BP	GO:0002696	positive regulation of leukocyte activation	17/187	406/18866	6.42E-07	5.08E-06	2.31E-06	LBP/BCL2/DPP4/AKT1/VCAM1/CDKN1A/IL2RA/IFNG/IL4/CD40LG/MMP8/BAD/CAV1/IL1B/CCL2/IL1A/IGF2	17
BP	GO:0070301	cellular response to hydrogen peroxide	9/187	100/18866	6.67E-07	5.26E-06	2.39E-06	RELA/CDK1/CYP1B1/MDM2/PCNA/MET/NFE2L2/NQO1/FABP1	9
BP	GO:0009267	cellular response to starvation	11/187	163/18866	7.00E-07	5.51E-06	2.50E-06	BCL2/JUN/MAPK8/AKR1C3/CDKN1A/MAPK1/TP53/MAPK3/PPARA/HSPA5/NFE2L2	11
BP	GO:0055088	lipid homeostasis	11/187	163/18866	7.00E-07	5.51E-06	2.50E-06	PPARG/NR1I2/APOB/AKR1C1/GOT1/CES1/SOAT1/ACACA/CAV1/LPL/I	11
BP	GO:0002831	regulation of response to biotic stimulus	17/187	409/18866	7.11E-07	5.58E-06	2.53E-06	LBP/PRKCA/PPARG/RELA/IKBKB/STAT1/IL2RA/IFNG/IL4/XIAP/MAPK3/RAF1/IL1B/CHUK/IRF1/DRD2/INS	17
BP	GO:0019217	regulation of fatty acid metabolic process	9/187	101/18866	7.26E-07	5.68E-06	2.58E-06	PTGS2/PPARG/AKT1/PPARD/PPARA/CAV1/IL1B/INS/FABP1	9

BP	GO:0032872	regulation of stress-activated MAPK cascade	13/187	237/18866	7.26E-07	5.68E-06	2.58E-06	AKT1/GSTP1/EGFR/VEGFA/MAPK1/CD40LG/MMP8/NCF1/MAPK3/HMGCR/MYC/IL1B/EDN1	13
BP	GO:0007259	receptor signaling pathway via JAK-STAT	11/187	164/18866	7.44E-07	5.80E-06	2.63E-06	IL6R/STAT1/CYP1B1/AKR1B1/IL10RA/IFNG/IL4/STAT3/EGF/CAV1/CC	11
BP	GO:0043535	regulation of blood vessel endothelial cell migration	11/187	164/18866	7.44E-07	5.80E-06	2.63E-06	PTGS2/PRKCA/PPARG/AKT1/HMOX1/VEGFA/KDR/HIF1A/NOS3/HSPB1/NFE2L2	11
BP	GO:0015980	energy derivation by oxidation of organic compounds	14/187	278/18866	7.52E-07	5.85E-06	2.65E-06	NOS2/AKT1/CDK1/CYP1A2/CAT/GSK3B/TP53/CCNB1/IFNG/IL4/HIF1A/MYC/IGF2/INS	14
BP	GO:0010594	regulation of endothelial cell migration	13/187	238/18866	7.62E-07	5.91E-06	2.68E-06	PTGS2/PRKCA/PPARG/AKT1/HMOX1/VEGFA/MET/KDR/HIF1A/NOS3/HSPB1/NFE2L2/EDN1	13
BP	GO:0010883	regulation of lipid storage	7/187	52/18866	7.71E-07	5.98E-06	2.71E-06	PPARG/NFKBIA/PPARD/APOB/PPARA/CES1/LP	7
BP	GO:0120255	olefinic compound biosynthetic process	6/187	33/18866	7.76E-07	6.00E-06	2.72E-06	ALOX5/GSTP1/GSTM1/AKR1C3/CYP19A1/ADM	6
BP	GO:0001763	morphogenesis of a branching structure	12/187	201/18866	8.05E-07	6.20E-06	2.82E-06	BCL2/AR/ESR1/RXRA/VEGFA/MET/KDR/EGF/MYC/DRD2/EDN1/ADM	12
BP	GO:0002221	pattern recognition receptor signaling pathway	12/187	201/18866	8.05E-07	6.20E-06	2.82E-06	CD14/LBP/CASP8/RELA/IKBKB/ESR1/NFKBIA/XIAP/APOB/CAV1/CHUK/IRF1	12
BP	GO:0007548	sex differentiation	14/187	280/18866	8.19E-07	6.30E-06	2.86E-06	BCL2/BAX/CASP3/AR/ICAM1/AKR1C3/ESR1/VEGFA/CCND1/BCL2L1/SOD1/CYP19A1/HSPA5/N	14
BP	GO:0070302	regulation of stress-activated protein kinase signaling cascade	13/187	240/18866	8.37E-07	6.43E-06	2.92E-06	AKT1/GSTP1/EGFR/VEGFA/MAPK1/CD40LG/MMP8/NCF1/MAPK3/HMGCR/MYC/IL1B/EDN1	13
BP	GO:0009746	response to hexose	12/187	202/18866	8.48E-07	6.50E-06	2.95E-06	PTGS2/CASP3/ICAM1/ADRA2A/PPARD/BAD/HMGCR/RAF1/HIF1A/GJA1/LPL/GCG	12

BP	GO:0030098	lymphocyte differentiation	16/187	368/18866	8.50E-07	6.50E-06	2.95E-06	BCL2/BAX/VCAM1/TP53/ERBB2/IL2RA/IFNG/IL4/CD40LG/BAD/SOD1/STAT3/IL1B/IL1A/RUNX	16
BP	GO:0001822	kidney development	14/187	283/18866	9.30E-07	7.11E-06	3.22E-06	IL6R/BCL2/BAX/CASP9/STAT1/CAT/AKR1B1/VEGFA/MMP9/CA2/ODC1/MYC/BDNF/ENPEP	14
BP	GO:0043276	anoikis	6/187	34/18866	9.34E-07	7.13E-06	3.23E-06	BCL2/AKT1/MCL1/CAV1/CHEK2/E2F1	6
BP	GO:1902176	negative regulation of oxidative stress-induced intrinsic apoptotic signaling pathway	5/187	19/18866	9.42E-07	7.17E-06	3.25E-06	AKT1/HIF1A/HSPB1/NFE2L2/INS	5
BP	GO:0043270	positive regulation of ion transport	14/187	284/18866	9.70E-07	7.37E-06	3.35E-06	SLC6A4/BAX/AKT1/ADRA2A/IFNG/HTR3A/CAV1/IL1B/CCL2/CXCL11/CXCL10/DRD2/EDN1/G	14
BP	GO:0003012	muscle system process	18/187	467/18866	9.83E-07	7.46E-06	3.38E-06	PTGS2/ADRA1B/PRKCA/HMOX1/ADRA2A/SOD1/PPARA/CAV1/GJA1/IL1B/PTGER3/NOS3/PARP1/CHUK/DRD2/TRPV1/EDN1/CACNA1S	18
BP	GO:0030857	negative regulation of epithelial cell differentiation	7/187	54/18866	1.00E-06	7.59E-06	3.44E-06	STAT1/GSK3B/VEGFA/CCND1/MMP9/IFNG/CA	7
BP	GO:0032757	positive regulation of interleukin-8 production	7/187	54/18866	1.00E-06	7.59E-06	3.44E-06	CD14/LBP/RELA/STAT3/F3/IL1B/SERPINE1	7
BP	GO:0042593	glucose homeostasis	13/187	245/18866	1.06E-06	7.96E-06	3.61E-06	PPARG/AKT1/ICAM1/ALOX5/ADRA2A/BAD/HMGCR/STAT3/RAF1/HIF1A/HK2/INS/GCG	13
BP	GO:0050867	positive regulation of cell activation	17/187	421/18866	1.06E-06	7.98E-06	3.62E-06	LBP/BCL2/DPP4/AKT1/VCAM1/CDKN1A/IL2RA/IFNG/IL4/CD40LG/MMP8/BAD/CAV1/IL1B/CCL2/IL1A/IGF2	17
BP	GO:0033500	carbohydrate homeostasis	13/187	246/18866	1.10E-06	8.31E-06	3.77E-06	PPARG/AKT1/ICAM1/ALOX5/ADRA2A/BAD/HMGCR/STAT3/RAF1/HIF1A/HK2/INS/GCG	13

BP	GO:0070838	divalent metal ion transport	18/187	471/18866	1.11E-06	8.34E-06	3.78E-06	PTGS2/OPRM1/BCL2/BAX/ICAM1/ADRA2A/EGF/CAV1/GJA1/CCL2/PRKCB/NOS3/CXCL11/CXCL10/DRD2/TRPV1/GCG/CACNA1S	18
BP	GO:0030856	regulation of epithelial cell differentiation	11/187	171/18866	1.13E-06	8.44E-06	3.83E-06	IKBKB/STAT1/GSK3B/VEGFA/CCND1/MMP9/IFNG/BAD/CAV1/IL1B/SERPINE1	11
BP	GO:0010827	regulation of glucose transmembrane transport	8/187	79/18866	1.15E-06	8.58E-06	3.90E-06	AKT1/MAPK14/IL1B/PRKCB/NFE2L2/HK2/EDN1/INS	8
BP	GO:1900182	positive regulation of protein localization to nucleus	8/187	80/18866	1.26E-06	9.44E-06	4.28E-06	PTGS2/AKT1/CDK1/MAPK14/MAPK1/IFNG/PARP1/INS	8
BP	GO:0043401	steroid hormone mediated signaling pathway	10/187	139/18866	1.27E-06	9.46E-06	4.29E-06	NR3C2/AR/ESR1/RXRA/ESR2/NR3C1/PPARD/PPARA/RXRB/PARP1	10
BP	GO:0032409	regulation of transporter activity	14/187	291/18866	1.29E-06	9.63E-06	4.37E-06	OPRM1/BCL2/PON1/PPARG/ADRA2A/MMP9/IFNG/HTR3A/CAV1/GJA1/CCL2/RASGRF1/DRD2/I	14
BP	GO:0034405	response to fluid shear stress	6/187	36/18866	1.33E-06	9.84E-06	4.46E-06	PTGS2/AKT1/CA2/GJA1/NOS3/NFE2L2	6
BP	GO:0042554	superoxide anion generation	6/187	36/18866	1.33E-06	9.84E-06	4.46E-06	GSTP1/EGFR/NCF1/SOD1/DUOX2/EDN1	6
BP	GO:1901099	negative regulation of signal transduction in absence of ligand	6/187	36/18866	1.33E-06	9.84E-06	4.46E-06	BCL2/AKT1/BCL2L1/MCL1/IL1B/IL1A	6
BP	GO:2001240	negative regulation of extrinsic apoptotic signaling pathway in absence of ligand	6/187	36/18866	1.33E-06	9.84E-06	4.46E-06	BCL2/AKT1/BCL2L1/MCL1/IL1B/IL1A	6
BP	GO:0097696	receptor signaling pathway via STAT	11/187	174/18866	1.34E-06	9.86E-06	4.48E-06	IL6R/STAT1/CYP1B1/AKR1B1/IL10RA/IFNG/IL4/STAT3/EGF/CAV1/CC	11
BP	GO:0072001	renal system development	14/187	292/18866	1.35E-06	9.93E-06	4.51E-06	IL6R/BCL2/BAX/CASP9/STAT1/CAT/AKR1B1/VEGFA/MMP9/CA2/ODC1/MYC/BDNF/ENPEP	14
BP	GO:0072655	establishment of protein localization to mitochondrion	10/187	140/18866	1.36E-06	9.96E-06	4.52E-06	BCL2/BAX/CASP8/AKT1/MAPK8/TP53/BAD/E2F1/NPEPPS/HK2	10

BP	GO:0072511	divalent inorganic cation transport	18/187	478/18866	1.37E-06	1.01E-05	4.56E-06	PTGS2/OPRM1/BCL2/BAX/ICAM1/ADRA2A/EGF/CAV1/GJA1/CCL2/PRKCB/NOS3/CXCL11/CXCL10/DRD2/TRPV1/GCG/CACNA1S	18
BP	GO:0001701	in utero embryonic development	16/187	383/18866	1.43E-06	1.05E-05	4.77E-06	CASP8/AR/AKT1/RXRA/EGFR/VEGFA/BCL2L1/MAPK1/TP53/CCNB1/APOB/HIF1A/NOS3/IGF2/EDN1/ADM	16
BP	GO:0043122	regulation of I-kappaB kinase/NF-kappaB signaling	13/187	252/18866	1.45E-06	1.06E-05	4.80E-06	CASP8/RELA/IKBKB/AKT1/STAT1/HMOX1/GSTP1/ESR1/GJA1/IL1B/PRKCB/HSPB1/CHUK	13
BP	GO:0034976	response to endoplasmic reticulum stress	14/187	294/18866	1.46E-06	1.06E-05	4.82E-06	BCL2/BAX/JUN/ALOX5/GSK3B/CCND1/BCL2L1/TP53/HSPA5/CAV1/CCL2/CXCL8/NFE2L2/RASG	14
BP	GO:0044262	cellular carbohydrate metabolic process	14/187	294/18866	1.46E-06	1.06E-05	4.82E-06	AKT1/AKR1B1/GSK3B/TP53/BAD/PPARA/GOT1/STAT3/IGFBP3/IGF2/HK2/INS/GCG/HRH1	14
BP	GO:0001541	ovarian follicle development	7/187	57/18866	1.46E-06	1.06E-05	4.82E-06	BCL2/BAX/ICAM1/ESR1/VEGFA/BCL2L1/SOD1	7
BP	GO:0045840	positive regulation of mitotic nuclear division	7/187	57/18866	1.46E-06	1.06E-05	4.82E-06	RB1/EGF/IL1B/IL1A/IGF2/EDN1/INS	7
BP	GO:1901989	positive regulation of cell cycle phase transition	9/187	110/18866	1.50E-06	1.08E-05	4.91E-06	AKT1/CDK1/CYP1A1/EGFR/CCND1/RB1/CDK4/MDM2/CCNB1	9
BP	GO:1904659	glucose transmembrane transport	9/187	110/18866	1.50E-06	1.08E-05	4.91E-06	AKT1/MAPK14/PPARD/IL1B/PRKCB/NFE2L2/HK2/EDN1/INS	9
BP	GO:0030217	T cell differentiation	13/187	253/18866	1.51E-06	1.09E-05	4.96E-06	BCL2/TP53/ERBB2/IL2RA/IFNG/IL4/BAD/SOD1/STAT3/IL1B/IL1A/RUNX2/IRF1	13
BP	GO:0022617	extracellular matrix disassembly	8/187	82/18866	1.53E-06	1.10E-05	5.01E-06	DPP4/PRSS1/MMP1/MMP2/MMP9/MMP13/MMP8/MMP3	8

BP	GO:0042326	negative regulation of phosphorylation	18/187	484/18866	1.64E-06	1.18E-05	5.35E-06	BAX/JUN/CASP3/AKT1/GSTP1/CDKN1A/RB1/CCNB1/IFNG/HMGCR/PPARA/STAT3/CAV1/MYC/IL1B/HSPB1/IGFBP3/D	18
BP	GO:0045598	regulation of fat cell differentiation	10/187	143/18866	1.64E-06	1.18E-05	5.36E-06	PTGS2/PPARG/AKT1/ALOX5/MAPK14/PPARD/RUNX1T1/E2F1/LPL/INS	10
BP	GO:0002763	positive regulation of myeloid leukocyte differentiation	7/187	58/18866	1.65E-06	1.18E-05	5.36E-06	JUN/CASP8/PRKCA/RB1/IFNG/CA2/FOS	7
BP	GO:1903749	positive regulation of establishment of protein localization to	7/187	58/18866	1.65E-06	1.18E-05	5.36E-06	BCL2/CASP8/MAPK8/TP53/BAD/E2F1/NPEPPS	7
BP	GO:0014855	striated muscle cell proliferation	8/187	83/18866	1.68E-06	1.20E-05	5.45E-06	CDK1/RXRA/MAPK14/MAPK1/CCNB1/PPARD/STAT3/GJA1	8
BP	GO:0006275	regulation of DNA replication	9/187	112/18866	1.74E-06	1.24E-05	5.63E-06	JUN/CDK1/CDK2/CCNA2/EGFR/TP53/PCNA/EGF/CHEK2	9
BP	GO:0046822	regulation of nucleocytoplasmic transport	9/187	112/18866	1.74E-06	1.24E-05	5.63E-06	PTGS2/CDK1/MAPK14/GSK3B/MAPK1/TP53/MDM2/IFNG/IL1B	9
BP	GO:0070585	protein localization to mitochondrion	10/187	144/18866	1.75E-06	1.25E-05	5.66E-06	BCL2/BAX/CASP8/AKT1/MAPK8/TP53/BAD/E2F1/NPEPPS/HK2	10
BP	GO:0043525	positive regulation of neuron apoptotic process	7/187	59/18866	1.85E-06	1.31E-05	5.95E-06	BAX/CASP9/JUN/CASP3/TP53/MCL1/NQO1	7
BP	GO:0045454	cell redox homeostasis	7/187	59/18866	1.85E-06	1.31E-05	5.95E-06	NOS2/NCF1/GSR/NOS3/MPO/NFE2L2/NQO1	7
BP	GO:0046456	icosanoid biosynthetic process	7/187	59/18866	1.85E-06	1.31E-05	5.95E-06	PTGS1/PTGS2/ALOX5/AKR1C3/PTGES/IL1B/ED	7
BP	GO:0071385	cellular response to glucocorticoid stimulus	7/187	59/18866	1.85E-06	1.31E-05	5.95E-06	CASP9/ICAM1/GSTP1/EGFR/NR3C1/UGT1A1/E	7
BP	GO:0045923	positive regulation of fatty acid metabolic process	6/187	38/18866	1.86E-06	1.31E-05	5.95E-06	PTGS2/PPARG/PPARD/PPARA/IL1B/FABP1	6
BP	GO:0038127	ERBB signaling pathway	10/187	145/18866	1.87E-06	1.31E-05	5.97E-06	PRKCA/AKT1/ADRA2A/EGFR/MMP9/MAPK1/ERBB2/NCF1/EGF/ERBB3	10
BP	GO:0010887	negative regulation of cholesterol storage	4/187	10/18866	1.87E-06	1.31E-05	5.97E-06	PPARG/PPARD/PPARA/CES1	4
BP	GO:0070391	response to lipoteichoic acid	4/187	10/18866	1.87E-06	1.31E-05	5.97E-06	CD14/LBP/RELA/MAPK14	4

BP	GO:0071223	cellular response to lipoteichoic acid	4/187	10/18866	1.87E-06	1.31E-05	5.97E-06	CD14/LBP/RELA/MAPK14	4
BP	GO:0010951	negative regulation of endopeptidase activity	13/187	258/18866	1.88E-06	1.32E-05	5.97E-06	PTGS2/AKT1/SLPI/DPEP1/VEGFA/MMP9/MDM2/BIRC5/XIAP/RAF1/SERPINE1/SERPINB2/FAB	13
BP	GO:0050890	cognition	14/187	302/18866	2.00E-06	1.40E-05	6.34E-06	PTGS2/SLC6A4/JUN/CASP3/EGFR/MAPK1/HMGCR/FOS/HIF1A/RASGRF1/DRD2/BDNF/INS/HRH	14
BP	GO:0006970	response to osmotic stress	8/187	85/18866	2.01E-06	1.40E-05	6.34E-06	PTGS2/BAX/CASP3/AKR1B1/EGFR/TP53/MAPK10/BAD	8
BP	GO:0010507	negative regulation of autophagy	8/187	85/18866	2.01E-06	1.40E-05	6.34E-06	BCL2/AKT1/HMOX1/IL10RA/TP53/MCL1/MET/STAT3	8
BP	GO:0045844	positive regulation of striated muscle tissue development	8/187	85/18866	2.01E-06	1.40E-05	6.34E-06	BCL2/CDK1/MAPK14/MAPK1/CCNB1/HMGCR/ERBB3/EDN1	8
BP	GO:0048636	positive regulation of muscle organ development	8/187	85/18866	2.01E-06	1.40E-05	6.34E-06	BCL2/CDK1/MAPK14/MAPK1/CCNB1/HMGCR/ERBB3/EDN1	8
BP	GO:0008645	hexose transmembrane transport	9/187	114/18866	2.02E-06	1.40E-05	6.35E-06	AKT1/MAPK14/PPARD/IL1B/PRKCB/NFE2L2/HK2/EDN1/INS	9
BP	GO:0007611	learning or memory	13/187	260/18866	2.05E-06	1.42E-05	6.43E-06	PTGS2/SLC6A4/JUN/CASP3/EGFR/MAPK1/HMGCR/FOS/HIF1A/RASGRF1/DRD2/BDNF/HRH1	13
BP	GO:0043388	positive regulation of DNA binding	7/187	60/18866	2.08E-06	1.43E-05	6.49E-06	PPARG/MMP9/RB1/IFNG/MMP8/EGF/PARP1	7
BP	GO:0050994	regulation of lipid catabolic process	7/187	60/18866	2.08E-06	1.43E-05	6.49E-06	AKT1/ADRA2A/CDK4/PPARA/IL1B/INS/FABP1	7
BP	GO:0061900	glial cell activation	7/187	60/18866	2.08E-06	1.43E-05	6.49E-06	JUN/EGFR/IFNG/IL4/MMP8/IL1B/TRPV1	7
BP	GO:0010888	negative regulation of lipid storage	5/187	22/18866	2.08E-06	1.43E-05	6.49E-06	PPARG/NFKBIA/PPARD/PPARA/CES1	5
BP	GO:0071379	cellular response to prostaglandin stimulus	5/187	22/18866	2.08E-06	1.43E-05	6.49E-06	PPARG/AKT1/AKR1C3/APOB/ACACA	5

BP	GO:0007189	adenylate cyclase-activating G protein-coupled receptor signaling pathway	10/187	147/18866	2.11E-06	1.45E-05	6.57E-06	ADRA1B/OPRM1/PRKCA/ADRA2A/PTGER3/CXCL11/CXCL10/DRD2/GCG/ADM	10
BP	GO:0055123	digestive system development	10/187	147/18866	2.11E-06	1.45E-05	6.57E-06	BCL2/CYP1A1/EGFR/CDKN1A/RB1/CCNB1/HIF1A/CXCL8/IGF2/PYY	10
BP	GO:0043620	regulation of DNA-templated transcription in response to stress	9/187	115/18866	2.17E-06	1.48E-05	6.73E-06	JUN/RELA/HMOX1/CHEK1/VEGFA/TP53/HIF1A/HSPA5/NFE2L2	9
BP	GO:0014910	regulation of smooth muscle cell migration	8/187	86/18866	2.20E-06	1.50E-05	6.80E-06	BCL2/GSTP1/PLAU/MDM2/PPARD/SERPINE1/NFE2L2/IGFBP3	8
BP	GO:1901863	positive regulation of muscle tissue development	8/187	86/18866	2.20E-06	1.50E-05	6.80E-06	BCL2/CDK1/MAPK14/MAPK1/CCNB1/HMGCR/ERBB3/EDN1	8
BP	GO:0010959	regulation of metal ion transport	16/187	396/18866	2.21E-06	1.50E-05	6.82E-06	PTGS2/BCL2/BAX/AKT1/ICAM1/ADRA2A/IFNG/EGF/CAV1/GJA1/CCL2/NOS3/CXCL11/CXCL10/DRD2/GCG	16
BP	GO:0007187	G protein-coupled receptor signaling pathway, coupled to cyclic nucleotide second messenger	13/187	263/18866	2.32E-06	1.58E-05	7.17E-06	ADRA1B/OPRM1/PRKCA/ADRA2A/CCL2/PTGER3/CXCL11/CXCL10/DRD2/EDN1/GCG/ADM/HR	13
BP	GO:0090303	positive regulation of wound healing	7/187	61/18866	2.33E-06	1.58E-05	7.17E-06	ADRA2A/F3/DUOX2/THBD/SERPINE1/NFE2L2/CLDN4	7
BP	GO:0015749	monosaccharide transmembrane transport	9/187	116/18866	2.33E-06	1.58E-05	7.17E-06	AKT1/MAPK14/PPARD/IL1B/PRKCB/NFE2L2/HK2/EDN1/INS	9
BP	GO:0070372	regulation of ERK1 and ERK2 cascade	14/187	306/18866	2.34E-06	1.58E-05	7.17E-06	OPRM1/JUN/PRKCA/ICAM1/GSTP1/EGFR/ERBB2/KDR/MAPK3/HMGCR/IL1B/CCL2/DRD2/GC	14
BP	GO:0043524	negative regulation of neuron apoptotic process	10/187	149/18866	2.39E-06	1.61E-05	7.32E-06	BCL2/BAX/JUN/HMOX1/BCL2L1/SOD1/HIF1A/CCL2/ERBB3/BDNF	10
BP	GO:0006606	protein import into nucleus	10/187	150/18866	2.54E-06	1.71E-05	7.75E-06	PTGS2/AKT1/CDK1/MAPK14/CDKN1A/MAPK1/TP53/NFKBIA/IFNG/ST	10

BP	GO:0045740	positive regulation of DNA replication	6/187	40/18866	2.54E-06	1.71E-05	7.75E-06	JUN/CDK1/CDK2/EGFR/PCNA/EGF	6
BP	GO:0010676	positive regulation of cellular carbohydrate metabolic process	7/187	62/18866	2.61E-06	1.75E-05	7.95E-06	AKT1/BAD/PPARA/IGF2/INS/GCG/HRH1	7
BP	GO:0032755	positive regulation of interleukin-6 production	8/187	88/18866	2.62E-06	1.76E-05	7.98E-06	IL6R/LBP/IFNG/MMP8/STAT3/IL1B/IL1A/LPL	8
BP	GO:0031281	positive regulation of cyclase activity	5/187	23/18866	2.64E-06	1.77E-05	8.02E-06	NOS2/MAPK8/MAPK14/MAPK3/NOS3	5
BP	GO:0015918	sterol transport	9/187	118/18866	2.69E-06	1.80E-05	8.15E-06	PON1/PPARG/NFKBIA/APOB/AKR1C1/CES1/SOAT1/EGF/CAV1	9
BP	GO:0034219	carbohydrate transmembrane transport	9/187	118/18866	2.69E-06	1.80E-05	8.15E-06	AKT1/MAPK14/PPARD/IL1B/PRKCB/NFE2L2/HK2/EDN1/INS	9
BP	GO:0061138	morphogenesis of a branching epithelium	11/187	187/18866	2.70E-06	1.80E-05	8.18E-06	BCL2/AR/ESR1/RXRA/VEGFA/MET/KDR/EGF/MYC/EDN1/ADM	11
BP	GO:2001020	regulation of response to DNA damage stimulus	12/187	226/18866	2.75E-06	1.83E-05	8.32E-06	BCL2/CASP9/CHEK1/EGFR/BCL2L1/TP53/MDM2/PCNA/MCL1/MYC/PARP1/CHEK2	12
BP	GO:0002224	toll-like receptor signaling pathway	10/187	152/18866	2.86E-06	1.90E-05	8.62E-06	CD14/LBP/CASP8/IKBKB/ESR1/NFKBIA/APOB/CAV1/CHUK/IRF1	10
BP	GO:0051251	positive regulation of lymphocyte activation	15/187	357/18866	2.88E-06	1.91E-05	8.68E-06	BCL2/DPP4/AKT1/VCAM1/CDKN1A/IL2RA/IFNG/IL4/CD40LG/BAD/CAV1/IL1B/CCL2/IL1A/IGF	15
BP	GO:0019371	cyclooxygenase pathway	4/187	11/18866	2.92E-06	1.93E-05	8.77E-06	PTGS1/PTGS2/AKR1C3/PTGES	4
BP	GO:0033197	response to vitamin E	4/187	11/18866	2.92E-06	1.93E-05	8.77E-06	PPARG/CAT/CCND1/COL1A1	4
BP	GO:0050804	modulation of chemical synaptic transmission	17/187	454/18866	2.94E-06	1.94E-05	8.83E-06	PTGS2/SLC6A4/ACHE/GSK3B/EGFR/MAPK1/CA2/STAT3/IL1B/CCL2/PRKCB/RASGRF1/DRD2/EDN1/BDNF/INS/HRH1	17
BP	GO:0071548	response to dexamethasone	6/187	41/18866	2.95E-06	1.94E-05	8.83E-06	CASP9/ICAM1/EGFR/PCNA/NR3C1/EDN1	6

BP	GO:0031348	negative regulation of defense response	13/187	269/18866	2.98E-06	1.96E-05	8.90E-06	TNFAIP6/PPARG/ALOX5/GSTP1/RB1/IL2RA/IL4/PPARD/SOD1/CYP19A1/PPARA/DRD2/INS	13
BP	GO:0043534	blood vessel endothelial cell migration	11/187	189/18866	3.00E-06	1.97E-05	8.94E-06	PTGS2/PRKCA/PPARG/AKT1/HMOX1/VEGFA/KDR/HIF1A/NOS3/HSPB1/NFE2L2	11
BP	GO:0099177	regulation of trans-synaptic signaling	17/187	455/18866	3.03E-06	1.99E-05	9.02E-06	PTGS2/SLC6A4/ACHE/GSK3B/EGFR/MAPK1/CA2/STAT3/IL1B/CCL2/PRKCB/RASGRF1/DRD2/EDN1/BDNF/INS/HRH1	17
BP	GO:0016202	regulation of striated muscle tissue development	10/187	153/18866	3.03E-06	1.99E-05	9.02E-06	BCL2/CDK1/MAPK14/MAPK1/CCNB1/HMGCR/PPARA/GJA1/ERBB3/ED	10
BP	GO:0002262	myeloid cell homeostasis	10/187	154/18866	3.22E-06	2.10E-05	9.55E-06	BAX/CASP3/STAT1/HMOX1/MAPK14/VEGFA/RB1/SOD1/STAT3/HIF1A	10
BP	GO:0033344	cholesterol efflux	7/187	64/18866	3.24E-06	2.12E-05	9.61E-06	PON1/NFKBIA/APOB/CES1/SOAT1/EGF/CAV1	7
BP	GO:0010869	regulation of receptor biosynthetic process	5/187	24/18866	3.31E-06	2.15E-05	9.75E-06	PPARG/IFNG/PPARA/HIF1A/EDN1	5
BP	GO:0036003	positive regulation of transcription from RNA polymerase II promoter in response to stress	5/187	24/18866	3.31E-06	2.15E-05	9.75E-06	VEGFA/TP53/HIF1A/HSPA5/NFE2L2	5
BP	GO:0002065	columnar/cuboidal epithelial cell differentiation	9/187	121/18866	3.31E-06	2.15E-05	9.75E-06	RXRA/GSK3B/CDKN1A/FASN/BAD/SOD1/HIF1A/SERPINE1/PYY	9
BP	GO:0010906	regulation of glucose metabolic process	9/187	121/18866	3.31E-06	2.15E-05	9.75E-06	AKT1/GSK3B/TP53/BAD/PPARA/IGFBP3/IGF2/INS/GCG	9
BP	GO:1900542	regulation of purine nucleotide metabolic process	9/187	121/18866	3.31E-06	2.15E-05	9.75E-06	NOS2/IFNG/IL4/PPARA/STAT3/HIF1A/NOS3/PARP1/INS	9
BP	GO:0010466	negative regulation of peptidase activity	13/187	272/18866	3.37E-06	2.18E-05	9.89E-06	PTGS2/AKT1/SLPI/DPEP1/VEGFA/MMP9/MDM2/BIRC5/XIAP/RAF1/SERPINE1/SERPINB2/FAB	13
BP	GO:0097306	cellular response to alcohol	8/187	91/18866	3.38E-06	2.18E-05	9.91E-06	PPARG/AKT1/AHR/AKR1C3/CDK4/UGT1A1/CES1/ACACA	8

BP	GO:0042752	regulation of circadian rhythm	9/187	122/18866	3.55E-06	2.29E-05	1.04E-05	PPARG/MAPK8/CDK1/GSK3B/TP53/TOP2A/MAPK10/PPARA/DRD2	9
BP	GO:0090181	regulation of cholesterol metabolic process	7/187	65/18866	3.60E-06	2.32E-05	1.05E-05	FASN/SOD1/APOB/HMGCR/CES1/ACACA/SCD	7
BP	GO:1905953	negative regulation of lipid localization	7/187	65/18866	3.60E-06	2.32E-05	1.05E-05	PPARG/AKT1/NFKBIA/PPARD/PPARA/CES1/E	7
BP	GO:1901861	regulation of muscle tissue development	10/187	156/18866	3.61E-06	2.32E-05	1.05E-05	BCL2/CDK1/MAPK14/MAPK1/CCNB1/HMGCR/PPARA/GJA1/ERBB3/ED	10
BP	GO:0051052	regulation of DNA metabolic process	15/187	365/18866	3.78E-06	2.42E-05	1.10E-05	BAX/PPARG/AKT1/CDK2/CHEK1/EGFR/CDKN1A/MAPK1/TP53/PCNA/IL4/MAPK3/MYC/GJA1/PARP1	15
BP	GO:0002761	regulation of myeloid leukocyte differentiation	9/187	123/18866	3.80E-06	2.43E-05	1.10E-05	JUN/CASP8/PRKCA/RB1/IFNG/IL4/CA2/FOS/MY	9
BP	GO:0006140	regulation of nucleotide metabolic process	9/187	123/18866	3.80E-06	2.43E-05	1.10E-05	NOS2/IFNG/IL4/PPARA/STAT3/HIF1A/NOS3/PARP1/INS	9
BP	GO:0048634	regulation of muscle organ development	10/187	157/18866	3.82E-06	2.44E-05	1.11E-05	BCL2/CDK1/MAPK14/MAPK1/CCNB1/HMGCR/PPARA/GJA1/ERBB3/ED	10
BP	GO:0150077	regulation of neuroinflammatory response	6/187	43/18866	3.93E-06	2.51E-05	1.14E-05	PTGS2/MMP9/IL4/MMP8/MMP3/IL1B	6
BP	GO:0014909	smooth muscle cell migration	8/187	93/18866	3.98E-06	2.54E-05	1.15E-05	BCL2/GSTP1/PLAU/MDM2/PPARD/SERPINE1/NFE2L2/IGFBP3	8
BP	GO:0045861	negative regulation of proteolysis	15/187	369/18866	4.31E-06	2.74E-05	1.24E-05	PTGS2/AKT1/SLPI/DPEP1/VEGFA/MMP9/TP53/MDM2/BIRC5/XIAP/RAF1/SERPINE1/INS/SERPINB2/FABP1	15
BP	GO:0003014	renal system process	9/187	125/18866	4.34E-06	2.75E-05	1.25E-05	BCL2/AKR1C3/AKR1B1/GJA1/CLDN4/DRD2/TRPV1/EDN1/ADM	9
BP	GO:0034605	cellular response to heat	9/187	125/18866	4.34E-06	2.75E-05	1.25E-05	PTGS2/HMOX1/GSK3B/CDKN1A/MAPK1/MAPK3/IL1A/CXCL10/TRPV	9
BP	GO:0007077	mitotic nuclear envelope disassembly	4/187	12/18866	4.35E-06	2.75E-05	1.25E-05	PRKCA/CDK1/CCNB1/PRKCB	4

BP	GO:0009749	response to glucose	11/187	197/18866	4.47E-06	2.83E-05	1.28E-05	CASP3/ICAM1/ADRA2A/PPARD/BAD/HMGCR/RAF1/HIF1A/GJA1/LPL/G	11
BP	GO:0046777	protein autophosphorylation	12/187	237/18866	4.49E-06	2.84E-05	1.29E-05	JUN/AKT1/GSK3B/EGFR/VEGFA/ERBB2/KDR/MAPK3/CAV1/CHEK2/CDK12/INS	12
BP	GO:0071479	cellular response to ionizing radiation	7/187	68/18866	4.89E-06	3.08E-05	1.40E-05	MAPK14/BCL2L1/CDKN1A/TP53/MDM2/HSPA5/CHEK2	7
BP	GO:0019933	cAMP-mediated signaling	11/187	199/18866	4.92E-06	3.10E-05	1.41E-05	ADRA1B/OPRM1/PRKCA/AHR/ADRA2A/PTGER3/CXCL11/CXCL10/DRD2/GCG/ADM	11
BP	GO:0014066	regulation of phosphatidylinositol 3-kinase signaling	9/187	127/18866	4.94E-06	3.10E-05	1.41E-05	CAT/EGFR/MAPK1/KDR/PPARD/NCF1/MAPK3/ERBB3/INS	9
BP	GO:1900180	regulation of protein localization to nucleus	9/187	127/18866	4.94E-06	3.10E-05	1.41E-05	PTGS2/AKT1/CDK1/MAPK14/GSK3B/MAPK1/IFNG/PARP1/INS	9
BP	GO:0019430	removal of superoxide radicals	5/187	26/18866	5.04E-06	3.16E-05	1.43E-05	SOD1/NOS3/MPO/NFE2L2/NQO1	5
BP	GO:0051346	negative regulation of hydrolase activity	17/187	473/18866	5.08E-06	3.18E-05	1.44E-05	PTGS2/IKBKB/AKT1/SLPI/DPEP1/GSK3B/VEGFA/MMP9/TP53/MDM2/BIRC5/XIAP/RAF1/NOS3/SERPINE1/SERPINB2/F	17
BP	GO:0009755	hormone-mediated signaling pathway	11/187	200/18866	5.17E-06	3.23E-05	1.46E-05	NR3C2/AR/PPARG/ESR1/RXRA/ESR2/NR3C1/PPARD/PPARA/RXRB/PAR	11
BP	GO:1904646	cellular response to amyloid-beta	6/187	45/18866	5.17E-06	3.23E-05	1.46E-05	ICAM1/VCAM1/GSK3B/ABCC1/GJA1/PARP1	6
BP	GO:0042698	ovulation cycle	7/187	69/18866	5.40E-06	3.36E-05	1.53E-05	OPRM1/CASP3/ESR1/EGFR/PCNA/HSPA5/NOS3	7
BP	GO:0071674	mononuclear cell migration	8/187	97/18866	5.46E-06	3.40E-05	1.54E-05	IL6R/MAPK14/MAPK1/IL4/MAPK3/CCL2/SERPINE1/CXCL10	8
BP	GO:0048285	organelle fission	17/187	476/18866	5.52E-06	3.43E-05	1.56E-05	PRKCA/CDK1/CHEK1/RB1/BIRC5/CCNB1/TOP2A/XIAP/KDR/EGF/IL1B/PRKCB/IL1A/CHEK2/IGF2/EDN1/INS	17

BP	GO:0017144	drug metabolic process	6/187	46/18866	5.90E-06	3.65E-05	1.66E-05	CYP3A4/CYP1A2/CYP1A1/NR1I2/DPEP1/UGT1	6
BP	GO:0048538	thymus development	6/187	46/18866	5.90E-06	3.65E-05	1.66E-05	BCL2/MAPK1/TYR/MAPK3/SOD1/RAF1	6
BP	GO:0048010	vascular endothelial growth factor receptor signaling pathway	8/187	98/18866	5.90E-06	3.65E-05	1.66E-05	MAPK14/VEGFA/KDR/NCF1/HIF1A/IL1B/PRKCB/HSPB1	8
BP	GO:0000280	nuclear division	16/187	428/18866	5.95E-06	3.68E-05	1.67E-05	PRKCA/CDK1/CHEK1/RB1/BIRC5/CCNB1/TOP2A/XIAP/EGF/IL1B/PRKCB/IL1A/CHEK2/IGF2/EDN1/INS	16
BP	GO:0002674	negative regulation of acute inflammatory response	4/187	13/18866	6.23E-06	3.84E-05	1.74E-05	PPARG/GSTP1/IL4/INS	4
BP	GO:0043255	regulation of carbohydrate biosynthetic process	8/187	99/18866	6.36E-06	3.91E-05	1.78E-05	AKT1/GSK3B/PPARA/EGF/IGF2/INS/GCG/HRH1	8
BP	GO:0090277	positive regulation of peptide hormone secretion	8/187	99/18866	6.36E-06	3.91E-05	1.78E-05	EGFR/PPARD/BAD/HIF1A/GJA1/DRD2/INS/GCG	8
BP	GO:0010508	positive regulation of autophagy	9/187	131/18866	6.37E-06	3.91E-05	1.78E-05	HMOX1/GSK3B/IFNG/IL4/KDR/MAPK3/BAD/HIF1A/HK2	9
BP	GO:0051785	positive regulation of nuclear division	7/187	71/18866	6.54E-06	4.01E-05	1.82E-05	RB1/EGF/IL1B/IL1A/IGF2/EDN1/INS	7
BP	GO:0061180	mammary gland epithelium development	7/187	71/18866	6.54E-06	4.01E-05	1.82E-05	BAX/AR/AKT1/ESR1/CCND1/MAPK1/HIF1A	7
BP	GO:0042129	regulation of T cell proliferation	10/187	167/18866	6.63E-06	4.05E-05	1.84E-05	CASP3/VCAM1/ERBB2/IL2RA/IL4/CD40LG/IL1B/IL1A/IGF2/IRF1	10
BP	GO:0060485	mesenchyme development	13/187	290/18866	6.75E-06	4.12E-05	1.87E-05	BCL2/STAT1/GSK3B/MAPK1/MDM2/MAPK3/HIF1A/MYC/IL1B/NOS3/COL1A1/ERBB3/EDN1	13
BP	GO:0045807	positive regulation of endocytosis	8/187	100/18866	6.86E-06	4.18E-05	1.90E-05	CD14/PPARG/SELE/VEGFA/IL4/EGF/SERPINE1/DRD2	8
BP	GO:0045600	positive regulation of fat cell differentiation	7/187	72/18866	7.19E-06	4.38E-05	1.99E-05	PTGS2/PPARG/AKT1/MAPK14/PPARD/LPL/INS	7
BP	GO:0032637	interleukin-8 production	8/187	101/18866	7.39E-06	4.48E-05	2.03E-05	CD14/LBP/NOS2/RELA/STAT3/F3/IL1B/SERPIN	8
BP	GO:0010575	positive regulation of vascular endothelial growth factor production	5/187	28/18866	7.41E-06	4.48E-05	2.03E-05	PTGS2/CYP1B1/HIF1A/IL1B/IL1A	5

BP	GO:0071450	cellular response to oxygen radical	5/187	28/18866	7.41E-06	4.48E-05	2.03E-05	SOD1/NOS3/MPO/NFE2L2/NQO1	5
BP	GO:0071451	cellular response to superoxide	5/187	28/18866	7.41E-06	4.48E-05	2.03E-05	SOD1/NOS3/MPO/NFE2L2/NQO1	5
BP	GO:1990776	response to angiotensin	5/187	28/18866	7.41E-06	4.48E-05	2.03E-05	PTGS2/RELA/CA2/CAV1/NFE2L2	5
BP	GO:0042220	response to cocaine	6/187	48/18866	7.60E-06	4.58E-05	2.08E-05	OPRM1/CCNA2/MDM2/HTR3A/HSPA5/DRD2	6
BP	GO:1904036	negative regulation of epithelial cell apoptotic process	6/187	48/18866	7.60E-06	4.58E-05	2.08E-05	HMOX1/ICAM1/IL4/KDR/SERPINE1/NFE2L2	6
BP	GO:2001239	regulation of extrinsic apoptotic signaling pathway in absence of	6/187	48/18866	7.60E-06	4.58E-05	2.08E-05	BCL2/AKT1/BCL2L1/MCL1/IL1B/IL1A	6
BP	GO:1903747	regulation of establishment of protein localization to	7/187	73/18866	7.88E-06	4.74E-05	2.15E-05	BCL2/CASP8/MAPK8/TP53/BAD/E2F1/NPEPPS	7
BP	GO:0000079	regulation of cyclin-dependent protein serine/threonine kinase activity	8/187	102/18866	7.95E-06	4.76E-05	2.16E-05	CASP3/AKT1/CCNA2/EGFR/CCND1/CDKN1A/CDK4/CCNB1	8
BP	GO:0055024	regulation of cardiac muscle tissue development	8/187	102/18866	7.95E-06	4.76E-05	2.16E-05	CDK1/MAPK14/MAPK1/CCNB1/PPARA/GJA1/ERBB3/EDN1	8
BP	GO:0014706	striated muscle tissue development	15/187	389/18866	8.13E-06	4.86E-05	2.21E-05	BCL2/CDK1/RXRA/MAPK14/VEGFA/MAPK1/RB1/CCNB1/HMGCR/PPARA/FOS/CAV1/GJA1/ERBB3/EDN1	15
BP	GO:0003158	endothelium development	9/187	135/18866	8.14E-06	4.86E-05	2.21E-05	IKBKB/ICAM1/VEGFA/MET/KDR/FASN/GJA1/IL1B/CXCL10	9
BP	GO:0048565	digestive tract development	9/187	135/18866	8.14E-06	4.86E-05	2.21E-05	BCL2/CYP1A1/EGFR/CDKN1A/RB1/CCNB1/HIF1A/CXCL8/PYY	9
BP	GO:0051170	import into nucleus	10/187	171/18866	8.17E-06	4.87E-05	2.21E-05	PTGS2/AKT1/CDK1/MAPK14/CDKN1A/MAPK1/TP53/NFKBIA/IFNG/ST	10
BP	GO:0034764	positive regulation of transmembrane transport	11/187	210/18866	8.22E-06	4.90E-05	2.22E-05	BAX/AKT1/MAPK14/IFNG/CA2/HTR3A/CCL2/NFE2L2/CXCL11/CXCL10	11
BP	GO:0051348	negative regulation of transferase activity	13/187	296/18866	8.42E-06	5.01E-05	2.27E-05	CASP3/PPARG/AKT1/GSTP1/GSK3B/CDKN1A/RB1/TP53/IFNG/HMGCR/CAV1/IL1B/HSPB1	13

BP	GO:0031279	regulation of cyclase activity	6/187	49/18866	8.59E-06	5.09E-05	2.31E-05	NOS2/MAPK8/MAPK14/MAPK3/NOS3/DRD2	6
BP	GO:0090199	regulation of release of cytochrome c from mitochondria	6/187	49/18866	8.59E-06	5.09E-05	2.31E-05	BAX/AKT1/BCL2L1/MMP9/TP53/BAD	6
BP	GO:1903036	positive regulation of response to wounding	7/187	74/18866	8.63E-06	5.11E-05	2.32E-05	ADRA2A/F3/DUOX2/THBD/SERPINE1/NFE2L2/CLDN4	7
BP	GO:0050671	positive regulation of lymphocyte proliferation	9/187	136/18866	8.65E-06	5.11E-05	2.32E-05	BCL2/VCAM1/CDKN1A/IL2RA/IL4/CD40LG/IL1B/IL1A/IGF2	9
BP	GO:0051896	regulation of protein kinase B signaling	12/187	253/18866	8.73E-06	5.16E-05	2.34E-05	AKT1/AKR1C3/ESR1/EGFR/ERBB2/MET/EGF/F3/IGF2/ERBB3/DRD2/INS	12
BP	GO:0007263	nitric oxide mediated signal transduction	5/187	29/18866	8.88E-06	5.23E-05	2.37E-05	NOS2/EGFR/VEGFA/NOS3/INS	5
BP	GO:0008209	androgen metabolic process	5/187	29/18866	8.88E-06	5.23E-05	2.37E-05	CYP3A4/ESR1/CYP19A1/SPP1/ADM	5
BP	GO:0051701	interaction with host	11/187	212/18866	8.99E-06	5.29E-05	2.40E-05	CASP8/DPP4/CDK1/ICAM1/RXRA/EGFR/BCL2L1/MET/BAD/CAV1/CXC	11
BP	GO:0045926	negative regulation of growth	12/187	254/18866	9.09E-06	5.34E-05	2.42E-05	SLC6A4/BCL2/PPARG/ESR2/CDKN1A/RB1/TP53/PPARD/PPARA/HIF1A/GJA1/SPP1	12
BP	GO:0032946	positive regulation of mononuclear cell proliferation	9/187	137/18866	9.17E-06	5.38E-05	2.44E-05	BCL2/VCAM1/CDKN1A/IL2RA/IL4/CD40LG/IL1B/IL1A/IGF2	9
BP	GO:0007200	phospholipase C-activating G protein-coupled receptor signaling pathway	8/187	104/18866	9.18E-06	5.38E-05	2.44E-05	ADRA1B/OPRM1/ESR1/ADRA2A/PTGER3/DRD2/EDN1/HRH1	8
BP	GO:0001933	negative regulation of protein phosphorylation	16/187	444/18866	9.43E-06	5.50E-05	2.50E-05	BAX/JUN/CASP3/AKT1/GSTP1/CDKN1A/RB1/CCNB1/IFNG/HMGCR/CAV1/MYC/IL1B/HSPB1/IGFBP3/DRD2	16
BP	GO:0006695	cholesterol biosynthetic process	7/187	75/18866	9.44E-06	5.50E-05	2.50E-05	FASN/SOD1/APOB/HMGCR/CES1/ACACA/SCD	7
BP	GO:1900076	regulation of cellular response to insulin stimulus	7/187	75/18866	9.44E-06	5.50E-05	2.50E-05	RELA/CDK4/IL1B/PRKCB/IGF2/LPL/INS	7
BP	GO:1902653	secondary alcohol biosynthetic process	7/187	75/18866	9.44E-06	5.50E-05	2.50E-05	FASN/SOD1/APOB/HMGCR/CES1/ACACA/SCD	7

BP	GO:0010469	regulation of signaling receptor activity	10/187	174/18866	9.52E-06	5.54E-05	2.51E-05	OPRM1/ADRA2A/PLAU/ESR2/IFNG/NCF1/EGF/CCL2/SERPINE1/RASGRF	10
BP	GO:0030195	negative regulation of blood coagulation	6/187	50/18866	9.68E-06	5.60E-05	2.54E-05	PLAU/NOS3/THBD/SERPINE1/EDN1/SERPINB2	6
BP	GO:0045540	regulation of cholesterol biosynthetic process	6/187	50/18866	9.68E-06	5.60E-05	2.54E-05	FASN/SOD1/APOB/HMGCR/ACACA/SCD	6
BP	GO:0106118	regulation of sterol biosynthetic process	6/187	50/18866	9.68E-06	5.60E-05	2.54E-05	FASN/SOD1/APOB/HMGCR/ACACA/SCD	6
BP	GO:1904707	positive regulation of vascular associated smooth muscle cell proliferation	6/187	50/18866	9.68E-06	5.60E-05	2.54E-05	JUN/MMP2/MMP9/MDM2/GJA1/EDN1	6
BP	GO:0007631	feeding behavior	8/187	105/18866	9.85E-06	5.68E-05	2.58E-05	OPRM1/STAT3/FOS/DRD2/BDNF/INS/GCG/PYY	8
BP	GO:0030301	cholesterol transport	8/187	105/18866	9.85E-06	5.68E-05	2.58E-05	PON1/NFKBIA/APOB/AKR1C1/CES1/SOAT1/EGF/CAV1	8
BP	GO:0062014	negative regulation of small molecule metabolic process	8/187	105/18866	9.85E-06	5.68E-05	2.58E-05	AKT1/AKR1C3/TP53/SOD1/PPARA/STAT3/PARP1/INS	8
BP	GO:0070374	positive regulation of ERK1 and ERK2 cascade	11/187	215/18866	1.03E-05	5.91E-05	2.68E-05	OPRM1/JUN/PRKCA/ICAM1/EGFR/KDR/MAPK3/HMGCR/CCL2/DRD2/	11
BP	GO:0014812	muscle cell migration	8/187	106/18866	1.06E-05	6.05E-05	2.75E-05	BCL2/GSTP1/PLAU/MDM2/PPARD/SERPINE1/NFE2L2/IGFBP3	8
BP	GO:1904029	regulation of cyclin-dependent protein kinase activity	8/187	106/18866	1.06E-05	6.05E-05	2.75E-05	CASP3/AKT1/CCNA2/EGFR/CCND1/CDKN1A/CDK4/CCNB1	8
BP	GO:0000303	response to superoxide	5/187	30/18866	1.06E-05	6.05E-05	2.75E-05	SOD1/NOS3/MPO/NFE2L2/NQO1	5
BP	GO:0048147	negative regulation of fibroblast proliferation	5/187	30/18866	1.06E-05	6.05E-05	2.75E-05	BAX/PPARG/GSTP1/TP53/MYC	5
BP	GO:0006984	ER-nucleus signaling pathway	6/187	51/18866	1.09E-05	6.20E-05	2.82E-05	GSK3B/TP53/HSPA5/CCL2/CXCL8/NFE2L2	6
BP	GO:0071675	regulation of mononuclear cell migration	6/187	51/18866	1.09E-05	6.20E-05	2.82E-05	MAPK14/MAPK1/IL4/MAPK3/SERPINE1/CXCL1	6
BP	GO:1900047	negative regulation of hemostasis	6/187	51/18866	1.09E-05	6.20E-05	2.82E-05	PLAU/NOS3/THBD/SERPINE1/EDN1/SERPINB2	6
BP	GO:0007422	peripheral nervous system development	7/187	77/18866	1.13E-05	6.41E-05	2.91E-05	RELA/AKT1/CDK1/ERBB2/SOD1/ERBB3/BDNF	7

BP	GO:0055017	cardiac muscle tissue growth	8/187	107/18866	1.13E-05	6.44E-05	2.92E-05	CDK1/RXRA/MAPK14/MAPK1/CCNB1/PPARA/GJA1/EDN1	8
BP	GO:0001889	liver development	9/187	141/18866	1.16E-05	6.58E-05	2.98E-05	JUN/RELA/HMOX1/CYP1A1/EGFR/CCND1/PCNA/MET/UGT1A1	9
BP	GO:0051897	positive regulation of protein kinase B signaling	10/187	178/18866	1.16E-05	6.59E-05	2.99E-05	AKR1C3/ESR1/EGFR/ERBB2/MET/EGF/F3/IGF2/ERBB3/INS	10
BP	GO:0009404	toxin metabolic process	4/187	15/18866	1.17E-05	6.62E-05	3.00E-05	CYP1A2/CYP1A1/CYP1B1/NFE2L2	4
BP	GO:0090594	inflammatory response to wounding	4/187	15/18866	1.17E-05	6.62E-05	3.00E-05	HMOX1/ALOX5/HIF1A/IL1A	4
BP	GO:2001028	positive regulation of endothelial cell chemotaxis	4/187	15/18866	1.17E-05	6.62E-05	3.00E-05	VEGFA/MET/KDR/HSPB1	4
BP	GO:0048260	positive regulation of receptor-mediated endocytosis	6/187	52/18866	1.22E-05	6.88E-05	3.12E-05	SELE/VEGFA/IL4/EGF/SERPINE1/DRD2	6
BP	GO:0072332	intrinsic apoptotic signaling pathway by p53 class mediator	7/187	78/18866	1.23E-05	6.90E-05	3.13E-05	BCL2/BAX/CDKN1A/TP53/MDM2/CHEK2/E2F1	7
BP	GO:0046425	regulation of receptor signaling pathway via JAK-STAT	9/187	142/18866	1.23E-05	6.90E-05	3.13E-05	IL6R/CYP1B1/AKR1B1/IL10RA/IFNG/IL4/STAT3/EGF/CAV1	9
BP	GO:0000305	response to oxygen radical	5/187	31/18866	1.25E-05	7.01E-05	3.18E-05	SOD1/NOS3/MPO/NFE2L2/NQO1	5
BP	GO:0032770	positive regulation of monooxygenase activity	5/187	31/18866	1.25E-05	7.01E-05	3.18E-05	AKT1/IFNG/HIF1A/IL1B/INS	5
BP	GO:0006939	smooth muscle contraction	8/187	109/18866	1.30E-05	7.26E-05	3.29E-05	PTGS2/ADRA2A/SOD1/CAV1/PTGER3/DRD2/TRPV1/EDN1	8
BP	GO:0043618	regulation of transcription from RNA polymerase II promoter in response to stress	8/187	109/18866	1.30E-05	7.26E-05	3.29E-05	JUN/HMOX1/CHEK1/VEGFA/TP53/HIF1A/HSPA5/NFE2L2	8
BP	GO:2000278	regulation of DNA biosynthetic process	8/187	109/18866	1.30E-05	7.26E-05	3.29E-05	PPARG/CDKN1A/MAPK1/TP53/PCNA/MAPK3/MYC/GJA1	8
BP	GO:0033044	regulation of chromosome organization	14/187	356/18866	1.32E-05	7.36E-05	3.34E-05	MAPK8/CHEK1/VEGFA/MAPK1/RB1/TP53/CCNB1/TOP2A/MAPK3/MYC/IL1B/PARP1/IGF2/GCG	14

BP	GO:0051480	regulation of cytosolic calcium ion concentration	14/187	357/18866	1.36E-05	7.59E-05	3.44E-05	ADRA1B/BCL2/BAX/ESR1/GOT1/CAV1/GJA1/PTGER3/CXCL11/CXCL10/DRD2/TRPV1/EDN1/A	14
BP	GO:0061008	hepaticobiliary system development	9/187	144/18866	1.37E-05	7.64E-05	3.47E-05	JUN/RELA/HMOX1/CYP1A1/EGFR/CCND1/PCNA/MET/UGT1A1	9
BP	GO:0007517	muscle organ development	15/187	407/18866	1.39E-05	7.73E-05	3.51E-05	BCL2/CDK1/RXRA/MAPK14/MAPK1/RB1/CCNB1/HMGCR/PPARA/FOS/CAV1/GJA1/CXCL10/ERBB3/EDN1	15
BP	GO:0071346	cellular response to interferon-gamma	10/187	182/18866	1.41E-05	7.83E-05	3.56E-05	NOS2/PPARG/STAT1/ICAM1/VCAM1/TP53/IFNG/CCL2/IRF1/EDN1	10
BP	GO:0016485	protein processing	11/187	223/18866	1.45E-05	8.01E-05	3.64E-05	CASP3/CASP8/PLAU/MDM2/XIAP/BAD/CES1/F3/SERPINE1/PARP1/ENP	11
BP	GO:0048678	response to axon injury	7/187	80/18866	1.45E-05	8.01E-05	3.64E-05	BCL2/BAX/JUN/CDK1/SOD1/SPP1/DRD2	7
BP	GO:0055021	regulation of cardiac muscle tissue growth	7/187	80/18866	1.45E-05	8.01E-05	3.64E-05	CDK1/MAPK14/MAPK1/CCNB1/PPARA/GJA1/EDN1	7
BP	GO:1901224	positive regulation of NIK/NF-kappaB signaling	7/187	80/18866	1.45E-05	8.01E-05	3.64E-05	NR3C2/CD14/RELA/EGFR/MMP8/IL1B/EDN1	7
BP	GO:0010165	response to X-ray	5/187	32/18866	1.47E-05	8.08E-05	3.67E-05	CASP3/CCND1/CDKN1A/TP53/THBD	5
BP	GO:0010743	regulation of macrophage derived foam cell differentiation	5/187	32/18866	1.47E-05	8.08E-05	3.67E-05	PPARG/NFKBIA/APOB/PPARA/LPL	5
BP	GO:0038128	ERBB2 signaling pathway	5/187	32/18866	1.47E-05	8.08E-05	3.67E-05	PRKCA/EGFR/ERBB2/EGF/ERBB3	5
BP	GO:0051385	response to mineralocorticoid	5/187	32/18866	1.47E-05	8.08E-05	3.67E-05	CCND1/CDKN1A/FOS/PARP1/EDN1	5
BP	GO:0060537	muscle tissue development	15/187	409/18866	1.47E-05	8.08E-05	3.67E-05	BCL2/CDK1/RXRA/MAPK14/VEGFA/MAPK1/RB1/CCNB1/HMGCR/PPARA/FOS/CAV1/GJA1/ERBB3/EDN1	15
BP	GO:0001959	regulation of cytokine-mediated signaling pathway	10/187	183/18866	1.48E-05	8.11E-05	3.68E-05	CASP8/PPARG/IKBKB/STAT1/GSTP1/IFNG/HIF1A/CAV1/CHUK/EDN1	10

BP	GO:0019935	cyclic-nucleotide-mediated signaling	11/187	224/18866	1.51E-05	8.26E-05	3.75E-05	ADRA1B/OPRM1/PRKCA/AHR/ADRA2A/PTGER3/CXCL11/CXCL10/DRD2/GCG/ADM	11
BP	GO:0030397	membrane disassembly	4/187	16/18866	1.55E-05	8.43E-05	3.83E-05	PRKCA/CDK1/CCNB1/PRKCB	4
BP	GO:0051081	nuclear envelope disassembly	4/187	16/18866	1.55E-05	8.43E-05	3.83E-05	PRKCA/CDK1/CCNB1/PRKCB	4
BP	GO:0051770	positive regulation of nitric-oxide synthase biosynthetic process	4/187	16/18866	1.55E-05	8.43E-05	3.83E-05	STAT1/IFNG/KDR/CCL2	4
BP	GO:0071732	cellular response to nitric oxide	4/187	16/18866	1.55E-05	8.43E-05	3.83E-05	DPEP1/CDK2/CCNA2/MMP3	4
BP	GO:1905050	positive regulation of metallopeptidase activity	4/187	16/18866	1.55E-05	8.43E-05	3.83E-05	MAPK14/MAPK3/STAT3/CLDN4	4
BP	GO:0038061	NIK/NF-kappaB signaling	10/187	184/18866	1.55E-05	8.43E-05	3.83E-05	NR3C2/CD14/RELA/AKT1/EGFR/NFKBIA/MMP8/IL1B/CHUK/EDN1	10
BP	GO:0016126	sterol biosynthetic process	7/187	81/18866	1.57E-05	8.54E-05	3.88E-05	FASN/SOD1/APOB/HMGCR/CES1/ACACA/SCD	7
BP	GO:0002683	negative regulation of immune system process	16/187	463/18866	1.58E-05	8.58E-05	3.89E-05	CASP3/PPARG/DPP4/AKT1/HMOX1/NFKBIA/ERBB2/IL2RA/IL4/CYP19A1/MYC/CCL2/NFE2L2/IRF1/DRD2/INS	16
BP	GO:0002931	response to ischemia	6/187	55/18866	1.69E-05	9.15E-05	4.15E-05	BCL2/CASP9/TP53/CAV1/GJA1/HK2	6
BP	GO:0050819	negative regulation of coagulation	6/187	55/18866	1.69E-05	9.15E-05	4.15E-05	PLAU/NOS3/THBD/SERPINE1/EDN1/SERPINB2	6
BP	GO:0051353	positive regulation of oxidoreductase activity	6/187	55/18866	1.69E-05	9.15E-05	4.15E-05	AKT1/IFNG/HIF1A/IL1B/EDN1/INS	6
BP	GO:0071901	negative regulation of protein serine/threonine kinase activity	9/187	148/18866	1.71E-05	9.22E-05	4.18E-05	CASP3/AKT1/GSTP1/CDKN1A/RB1/HMGCR/CAV1/IL1B/HSPB1	9
BP	GO:0042573	retinoic acid metabolic process	5/187	33/18866	1.72E-05	9.25E-05	4.20E-05	CYP3A4/CYP1A1/AKR1C3/UGT1A1/ADH1B	5
BP	GO:0031056	regulation of histone modification	9/187	149/18866	1.80E-05	9.70E-05	4.40E-05	MAPK8/CHEK1/VEGFA/TP53/CCNB1/MAPK3/IL1B/IGF2/GCG	9
BP	GO:0045580	regulation of T cell differentiation	9/187	150/18866	1.90E-05	0.000102232	4.64E-05	ERBB2/IL2RA/IFNG/IL4/BAD/SOD1/IL1B/IL1A/I	9
BP	GO:0031623	receptor internalization	8/187	115/18866	1.92E-05	0.000102971	4.67E-05	ACHE/SELE/VEGFA/EGF/CAV1/CXCL8/DRD2/A	8

BP	GO:0060419	heart growth	8/187	115/18866	1.92E-05	0.000102971	4.67E-05	CDK1/RXRA/MAPK14/MAPK1/CCNB1/PPARA/GJA1/EDN1	8
BP	GO:0050715	positive regulation of cytokine secretion	5/187	34/18866	2.00E-05	0.000106699	4.84E-05	CD14/IFNG/IL1A/DRD2/INS	5
BP	GO:1902692	regulation of neuroblast proliferation	5/187	34/18866	2.00E-05	0.000106699	4.84E-05	VEGFA/TP53/HIF1A/DRD2/BDNF	5
BP	GO:2000352	negative regulation of endothelial cell apoptotic process	5/187	34/18866	2.00E-05	0.000106699	4.84E-05	ICAM1/IL4/KDR/SERPINE1/NFE2L2	5
BP	GO:0072521	purine-containing compound metabolic process	16/187	472/18866	2.00E-05	0.000106726	4.84E-05	NOS2/IFNG/IL4/FASN/BAD/HMGCR/PPARA/STAT3/HIF1A/ACACA/NOS3/ABCG2/PARP1/HK2/INS/SCD	16
BP	GO:0008643	carbohydrate transport	9/187	151/18866	2.01E-05	0.000106726	4.84E-05	AKT1/MAPK14/PPARD/IL1B/PRKCB/NFE2L2/HK2/EDN1/INS	9
BP	GO:1904892	regulation of receptor signaling pathway via STAT	9/187	151/18866	2.01E-05	0.000106726	4.84E-05	IL6R/CYP1B1/AKR1B1/IL10RA/IFNG/IL4/STAT3/EGF/CAV1	9
BP	GO:0030730	sequestering of triglyceride	4/187	17/18866	2.01E-05	0.00010685	4.85E-05	PPARG/PPARA/IL1B/LP	4
BP	GO:0030308	negative regulation of cell growth	10/187	190/18866	2.05E-05	0.000108672	4.93E-05	BCL2/PPARG/ESR2/CDKN1A/RB1/TP53/PPARD/PPARA/GJA1/SPP1	10
BP	GO:0007204	positive regulation of cytosolic calcium ion concentration	13/187	322/18866	2.06E-05	0.000108949	4.94E-05	ADRA1B/BCL2/BAX/ESR1/CAV1/GJA1/PTGER3/CXCL11/CXCL10/DRD2/TRPV1/EDN1/ADM	13
BP	GO:0022898	regulation of transmembrane transporter activity	12/187	276/18866	2.08E-05	0.000110269	5.00E-05	OPRM1/BCL2/ADRA2A/MMP9/IFNG/HTR3A/CAV1/GJA1/CCL2/RASGRF1/DRD2/INS	12
BP	GO:0007188	adenylate cyclase-modulating G protein-coupled receptor signaling pathway	11/187	233/18866	2.18E-05	0.00011504	5.22E-05	ADRA1B/OPRM1/PRKCA/ADRA2A/PTGER3/CXCL11/CXCL10/DRD2/EDN1/GCG/ADM	11
BP	GO:1900408	negative regulation of cellular response to oxidative stress	6/187	58/18866	2.31E-05	0.000121773	5.53E-05	AKT1/MET/HIF1A/HSPB1/NFE2L2/INS	6
BP	GO:1903202	negative regulation of oxidative stress-induced cell death	6/187	58/18866	2.31E-05	0.000121773	5.53E-05	AKT1/MET/HIF1A/HSPB1/NFE2L2/INS	6
BP	GO:1904031	positive regulation of cyclin-dependent protein kinase activity	5/187	35/18866	2.31E-05	0.00012182	5.53E-05	AKT1/EGFR/CCND1/CDKN1A/CCNB1	5

BP	GO:0045444	fat cell differentiation	11/187	235/18866	2.35E-05	0.000123885	5.62E-05	PTGS2/PPARG/AKT1/ALOX5/MAPK14/CCND1/PPARD/RUNX1T1/E2F1/LPL/INS	11
BP	GO:1901222	regulation of NIK/NF-kappaB signaling	8/187	119/18866	2.46E-05	0.000129426	5.87E-05	NR3C2/CD14/RELA/EGFR/NFKBIA/MMP8/IL1B/EDN1	8
BP	GO:0048754	branching morphogenesis of an epithelial tube	9/187	155/18866	2.47E-05	0.000129452	5.88E-05	BCL2/AR/ESR1/VEGFA/MET/KDR/EGF/MYC/ED	9
BP	GO:1990845	adaptive thermogenesis	9/187	155/18866	2.47E-05	0.000129452	5.88E-05	ACHE/VEGFA/RB1/IL4/CAV1/GJA1/TRPV1/UCP2/SCD	9
BP	GO:0060420	regulation of heart growth	7/187	87/18866	2.52E-05	0.000131696	5.98E-05	CDK1/MAPK14/MAPK1/CCNB1/PPARA/GJA1/EDN1	7
BP	GO:0007219	Notch signaling pathway	10/187	195/18866	2.56E-05	0.000133895	6.08E-05	AKT1/STAT1/EGFR/NFKBIA/IL2RA/GOT1/STAT3/EGF/MYC/NOS3	10
BP	GO:0000187	activation of MAPK activity	9/187	156/18866	2.60E-05	0.000135722	6.16E-05	CDK1/MAPK14/MAPK1/CD40LG/MAPK10/MAPK3/SOD1/EGF/IL1B	9
BP	GO:0045742	positive regulation of epidermal growth factor receptor signaling pathway	5/187	36/18866	2.66E-05	0.000138974	6.31E-05	AKT1/ADRA2A/MMP9/NCF1/EGF	5
BP	GO:0060759	regulation of response to cytokine stimulus	10/187	196/18866	2.68E-05	0.000139388	6.33E-05	CASP8/PPARG/IKBKB/STAT1/GSTP1/IFNG/HIF1A/CAV1/CHUK/EDN1	10
BP	GO:0070098	chemokine-mediated signaling pathway	7/187	88/18866	2.71E-05	0.000141031	6.40E-05	HIF1A/CCL2/CXCL8/CXCL11/CXCL2/CXCL10/EDN1	7
BP	GO:0043271	negative regulation of ion transport	9/187	157/18866	2.73E-05	0.000142069	6.45E-05	PTGS2/BCL2/AKT1/ICAM1/ADRA2A/MMP9/CAV1/NOS3/DRD2	9
BP	GO:0060562	epithelial tube morphogenesis	13/187	331/18866	2.74E-05	0.000142292	6.46E-05	BCL2/CASP3/AR/ESR1/VEGFA/MET/KDR/EGF/HIF1A/MYC/CXCL10/EDN1/ADM	13
BP	GO:1902883	negative regulation of response to oxidative stress	6/187	60/18866	2.81E-05	0.000145664	6.61E-05	AKT1/MET/HIF1A/HSPB1/NFE2L2/INS	6
BP	GO:0035051	cardiocyte differentiation	9/187	158/18866	2.87E-05	0.00014884	6.76E-05	CDK1/RXRA/EGFR/VEGFA/MAPK1/CCNB1/MAPK3/PPARA/EDN1	9

BP	GO:0001892	embryonic placenta development	7/187	89/18866	2.92E-05	0.000150888	6.85E-05	CASP8/AKT1/EGFR/MAPK1/HIF1A/IGF2/ADM	7
BP	GO:0001654	eye development	14/187	384/18866	3.05E-05	0.000157563	7.15E-05	BCL2/BAX/JUN/ACHE/CYP1A1/CYP1B1/RXRA/EGFR/VEGFA/CDK4/STAT3/HIF1A/DRD2/BDNF	14
BP	GO:2000273	positive regulation of signaling receptor activity	5/187	37/18866	3.06E-05	0.00015768	7.16E-05	ADRA2A/IFNG/NCF1/EGF/CCL2	5
BP	GO:0033013	tetrapyrrole metabolic process	6/187	61/18866	3.09E-05	0.000159083	7.22E-05	PRSS1/HMOX1/CYP1A2/CYP1A1/UGT1A1/ABC	6
BP	GO:0046888	negative regulation of hormone secretion	6/187	61/18866	3.09E-05	0.000159083	7.22E-05	ADRA2A/HMGCR/GJA1/IL1B/DRD2/EDN1	6
BP	GO:0046427	positive regulation of receptor signaling pathway via JAK-STAT	7/187	90/18866	3.14E-05	0.000161093	7.31E-05	IL6R/CYP1B1/AKR1B1/IL10RA/IFNG/IL4/STAT3	7
BP	GO:1903351	cellular response to dopamine	7/187	90/18866	3.14E-05	0.000161093	7.31E-05	OPRM1/GSK3B/MAPK1/HTR3A/MAPK3/DRD2/HRH1	7
BP	GO:0017038	protein import	10/187	200/18866	3.18E-05	0.000162897	7.39E-05	PTGS2/AKT1/CDK1/MAPK14/CDKN1A/MAPK1/TP53/NFKBIA/IFNG/ST	10
BP	GO:0051054	positive regulation of DNA metabolic process	10/187	200/18866	3.18E-05	0.000162897	7.39E-05	BAX/AKT1/CDK2/EGFR/MAPK1/PCNA/IL4/MAPK3/MYC/PARP1	10
BP	GO:0033189	response to vitamin A	4/187	19/18866	3.22E-05	0.000164413	7.46E-05	PPARG/CYP1A1/CAT/PPARD	4
BP	GO:0045780	positive regulation of bone	4/187	19/18866	3.22E-05	0.000164413	7.46E-05	PRKCA/EGFR/CA2/SPP1	4
BP	GO:0046852	positive regulation of bone	4/187	19/18866	3.22E-05	0.000164413	7.46E-05	PRKCA/EGFR/CA2/SPP1	4
BP	GO:1902170	cellular response to reactive nitrogen species	4/187	19/18866	3.22E-05	0.000164413	7.46E-05	DPEP1/CDK2/CCNA2/MMP3	4
BP	GO:0051899	membrane depolarization	7/187	91/18866	3.37E-05	0.000171026	7.76E-05	BCL2/JUN/KDR/GOT1/CAV1/PARP1/EDN1	7
BP	GO:0060333	interferon-gamma-mediated signaling pathway	7/187	91/18866	3.37E-05	0.000171026	7.76E-05	PPARG/STAT1/ICAM1/VCAM1/TP53/IFNG/IRF	7
BP	GO:1903350	response to dopamine	7/187	91/18866	3.37E-05	0.000171026	7.76E-05	OPRM1/GSK3B/MAPK1/HTR3A/MAPK3/DRD2/HRH1	7
BP	GO:2000177	regulation of neural precursor cell proliferation	7/187	91/18866	3.37E-05	0.000171026	7.76E-05	SLC6A4/VEGFA/TP53/EGF/HIF1A/DRD2/BDNF	7
BP	GO:0002260	lymphocyte homeostasis	6/187	62/18866	3.39E-05	0.000171806	7.80E-05	BCL2/BAX/CASP3/AKT1/IL2RA/HIF1A	6
BP	GO:0055025	positive regulation of cardiac muscle tissue development	6/187	62/18866	3.39E-05	0.000171806	7.80E-05	CDK1/MAPK14/MAPK1/CCNB1/ERBB3/EDN1	6

BP	GO:0150063	visual system development	14/187	388/18866	3.42E-05	0.000172698	7.84E-05	BCL2/BAX/JUN/ACHE/CYP1A1/CYP1B1/RXRA/EGFR/VEGFA/CDK4/STAT3/HIF1A/DRD2/BDNF	14
BP	GO:0034341	response to interferon-gamma	10/187	202/18866	3.46E-05	0.000174661	7.93E-05	NOS2/PPARG/STAT1/ICAM1/VCAM1/TP53/IFNG/CCL2/IRF1/EDN1	10
BP	GO:0014912	negative regulation of smooth muscle cell migration	5/187	38/18866	3.49E-05	0.000174661	7.93E-05	GSTP1/PPARD/SERPINE1/NFE2L2/IGFBP3	5
BP	GO:0030224	monocyte differentiation	5/187	38/18866	3.49E-05	0.000174661	7.93E-05	JUN/PPARG/VEGFA/FASN/MYC	5
BP	GO:0032094	response to food	5/187	38/18866	3.49E-05	0.000174661	7.93E-05	OPRM1/AKT1/CYP1A1/PPARA/MPO	5
BP	GO:0032885	regulation of polysaccharide biosynthetic process	5/187	38/18866	3.49E-05	0.000174661	7.93E-05	AKT1/GSK3B/EGF/IGF2/INS	5
BP	GO:0043029	T cell homeostasis	5/187	38/18866	3.49E-05	0.000174661	7.93E-05	BCL2/BAX/CASP3/AKT1/IL2RA	5
BP	GO:0071392	cellular response to estradiol stimulus	5/187	38/18866	3.49E-05	0.000174661	7.93E-05	ESR1/ESR2/CCNA2/EGFR/UGT1A1	5
BP	GO:1901186	positive regulation of ERBB signaling pathway	5/187	38/18866	3.49E-05	0.000174661	7.93E-05	AKT1/ADRA2A/MMP9/NCF1/EGF	5
BP	GO:1903131	mononuclear cell differentiation	5/187	38/18866	3.49E-05	0.000174661	7.93E-05	JUN/PPARG/VEGFA/FASN/MYC	5
BP	GO:0035270	endocrine system development	8/187	125/18866	3.51E-05	0.000175535	7.97E-05	IL6R/AKT1/GSK3B/MAPK1/MAPK3/BAD/RAF1/DRD2	8
BP	GO:0006163	purine nucleotide metabolic process	15/187	442/18866	3.62E-05	0.000180567	8.20E-05	NOS2/IFNG/IL4/FASN/BAD/HMGCR/PPARA/STAT3/HIF1A/ACACA/NOS3/PARP1/HK2/INS/SCD	15
BP	GO:0007088	regulation of mitotic nuclear division	9/187	163/18866	3.67E-05	0.000183148	8.31E-05	CHEK1/RB1/CCNB1/EGF/IL1B/IL1A/IGF2/EDN1/INS	9
BP	GO:0046622	positive regulation of organ growth	6/187	63/18866	3.72E-05	0.000185281	8.41E-05	AKT1/CDK1/MAPK14/MAPK1/CCNB1/EDN1	6
BP	GO:0002532	production of molecular mediator involved in inflammatory response	7/187	93/18866	3.88E-05	0.00019255	8.74E-05	LBP/NOS2/ALOX5/MAPK14/STAT3/SERPINE1/I	7
BP	GO:0032677	regulation of interleukin-8 production	7/187	93/18866	3.88E-05	0.00019255	8.74E-05	CD14/LBP/RELA/STAT3/F3/IL1B/SERPINE1	7
BP	GO:1904894	positive regulation of receptor signaling pathway via STAT	7/187	93/18866	3.88E-05	0.00019255	8.74E-05	IL6R/CYP1B1/AKR1B1/IL10RA/IFNG/IL4/STAT3	7

BP	GO:0014037	Schwann cell differentiation	5/187	39/18866	3.97E-05	0.00019691	8.94E-05	RELA/AKT1/CDK1/SOD1/ERBB3	5
BP	GO:0002544	chronic inflammatory response	4/187	20/18866	4.00E-05	0.000197365	8.96E-05	IL4/PTGES/CYP19A1/GJA1	4
BP	GO:0010875	positive regulation of cholesterol efflux	4/187	20/18866	4.00E-05	0.000197365	8.96E-05	PON1/NFKBIA/CES1/CAV1	4
BP	GO:0060965	negative regulation of gene silencing by miRNA	4/187	20/18866	4.00E-05	0.000197365	8.96E-05	PPARG/ESR1/TP53/STAT3	4
BP	GO:0071731	response to nitric oxide	4/187	20/18866	4.00E-05	0.000197365	8.96E-05	DPEP1/CDK2/CCNA2/MMP3	4
BP	GO:0048880	sensory system development	14/187	394/18866	4.04E-05	0.000198903	9.03E-05	BCL2/BAX/JUN/ACHE/CYP1A1/CYP1B1/RXRA/EGFR/VEGFA/CDK4/STAT3/HIF1A/DRD2/BDNF	14
BP	GO:0001885	endothelial cell development	6/187	64/18866	4.07E-05	0.000199995	9.08E-05	IKBKB/ICAM1/VEGFA/MET/FASN/IL1B	6
BP	GO:0002066	columnar/cuboidal epithelial cell development	6/187	64/18866	4.07E-05	0.000199995	9.08E-05	GSK3B/CDKN1A/FASN/BAD/SOD1/HIF1A	6
BP	GO:0051926	negative regulation of calcium ion transport	6/187	64/18866	4.07E-05	0.000199995	9.08E-05	PTGS2/BCL2/ICAM1/ADRA2A/NOS3/DRD2	6
BP	GO:0010660	regulation of muscle cell apoptotic process	7/187	94/18866	4.16E-05	0.000203962	9.26E-05	PPARG/HMOX1/TP53/IFNG/HMGCR/NFE2L2/ED	7
BP	GO:1903531	negative regulation of secretion by cell	9/187	166/18866	4.24E-05	0.000207659	9.42E-05	OPRM1/HMOX1/ADRA2A/HMGCR/GJA1/IL1B/DRD2/EDN1/INS	9
BP	GO:0007265	Ras protein signal transduction	13/187	346/18866	4.33E-05	0.000212071	9.63E-05	JUN/ADRA2A/MAPK14/CDK2/CCNA2/CDKN1A/RB1/TP53/MET/RAF1/CHUK/RASSF1/RASGRF1	13
BP	GO:0034101	erythrocyte homeostasis	8/187	129/18866	4.40E-05	0.0002152	9.77E-05	CASP3/STAT1/HMOX1/MAPK14/VEGFA/RB1/STAT3/HIF1A	8
BP	GO:0034250	positive regulation of cellular amide metabolic process	9/187	167/18866	4.44E-05	0.000216799	9.84E-05	CASP3/RELA/RXRA/MAPK1/CDK4/ERBB2/IFNG/MAPK3/NFE2L2	9
BP	GO:0010573	vascular endothelial growth factor production	6/187	65/18866	4.45E-05	0.000216799	9.84E-05	IL6R/PTGS2/CYP1B1/HIF1A/IL1B/IL1A	6
BP	GO:0035773	insulin secretion involved in cellular response to glucose stimulus	6/187	65/18866	4.45E-05	0.000216799	9.84E-05	ADRA2A/BAD/HMGCR/RAF1/HIF1A/GCG	6
BP	GO:0042307	positive regulation of protein import into nucleus	5/187	40/18866	4.50E-05	0.000218944	9.94E-05	PTGS2/CDK1/MAPK14/MAPK1/IFNG	5

BP	GO:0140353	lipid export from cell	5/187	40/18866	4.50E-05	0.000218944	9.94E-05	NOS2/CYP19A1/IL1B/SPP1/EDN1	5
BP	GO:0048568	embryonic organ development	15/187	451/18866	4.55E-05	0.000221019	0.000100312	CASP8/AKT1/EGFR/VEGFA/MAPK1/TP53/KDR/MAPK3/SOD1/HIF1A/CXCL8/RUNX2/IGF2/EDN1/ADM	15
BP	GO:2000027	regulation of animal organ morphogenesis	11/187	254/18866	4.79E-05	0.000232527	0.000105535	BCL2/BAX/AR/STAT1/ESR1/RXRA/VEGFA/MYC/RUNX2/EDN1/BDNF	11
BP	GO:0046626	regulation of insulin receptor signaling pathway	6/187	66/18866	4.85E-05	0.00023507	0.000106689	RELA/CDK4/IL1B/PRKCB/IGF2/INS	6
BP	GO:0007162	negative regulation of cell adhesion	12/187	301/18866	4.86E-05	0.00023507	0.000106689	CASP3/AKT1/CYP1B1/VEGFA/ERBB2/IL2RA/IL4/PPARA/SERPINE1/COL1A1/IRF1/ERBB3	12
BP	GO:0038095	Fc-epsilon receptor signaling pathway	9/187	169/18866	4.87E-05	0.000235587	0.000106923	JUN/RELA/IKBKB/MAPK8/MAPK1/MAPK10/MAPK3/FOS/CHUK	9
BP	GO:0036499	PERK-mediated unfolded protein response	4/187	21/18866	4.90E-05	0.000235954	0.00010709	HSPA5/CCL2/CXCL8/NFE2L2	4
BP	GO:0046827	positive regulation of protein export from nucleus	4/187	21/18866	4.90E-05	0.000235954	0.00010709	GSK3B/TP53/MDM2/IL1B	4
BP	GO:0061042	vascular wound healing	4/187	21/18866	4.90E-05	0.000235954	0.00010709	ALOX5/VEGFA/KDR/SERPINE1	4
BP	GO:0140467	integrated stress response signaling	4/187	21/18866	4.90E-05	0.000235954	0.00010709	HSPA5/CCL2/CXCL8/NFE2L2	4
BP	GO:0006766	vitamin metabolic process	8/187	131/18866	4.91E-05	0.000236123	0.000107167	PRSS1/CYP3A4/CYP1A1/AKR1C3/IFNG/PPARD/ABCC1/IL1B	8
BP	GO:0033574	response to testosterone	5/187	41/18866	5.09E-05	0.000242735	0.000110168	AR/CDK4/BAD/SPP1/EDN1	5
BP	GO:0038083	peptidyl-tyrosine autophosphorylation	5/187	41/18866	5.09E-05	0.000242735	0.000110168	EGFR/VEGFA/KDR/MAPK3/CAV1	5
BP	GO:0042100	B cell proliferation	7/187	97/18866	5.09E-05	0.000242735	0.000110168	BCL2/BAX/CASP3/AHR/CDKN1A/IL4/CD40LG	7
BP	GO:0120162	positive regulation of cold-induced thermogenesis	7/187	97/18866	5.09E-05	0.000242735	0.000110168	ACHE/VEGFA/IL4/CAV1/GJA1/UCP2/SCD	7
BP	GO:1901184	regulation of ERBB signaling pathway	7/187	97/18866	5.09E-05	0.000242735	0.000110168	AKT1/ADRA2A/EGFR/MMP9/ERBB2/NCF1/EGF	7

BP	GO:1990868	response to chemokine	7/187	97/18866	5.09E-05	0.000242735	0.000110168	HIF1A/CCL2/CXCL8/CXCL11/CXCL2/CXCL10/EDN1	7
BP	GO:1990869	cellular response to chemokine	7/187	97/18866	5.09E-05	0.000242735	0.000110168	HIF1A/CCL2/CXCL8/CXCL11/CXCL2/CXCL10/EDN1	7
BP	GO:0032635	interleukin-6 production	9/187	170/18866	5.10E-05	0.000243101	0.000110334	IL6R/LBP/NOS2/IFNG/MMP8/STAT3/IL1B/IL1A/LPL	9
BP	GO:0006874	cellular calcium ion homeostasis	15/187	456/18866	5.16E-05	0.000245429	0.00011139	ADRA1B/BCL2/BAX/ESR1/GOT1/CAV1/GJA1/PTGER3/PRKCB/CXCL11/CXCL10/DRD2/TRPV1/EDN1/ADM	15
BP	GO:0042476	odontogenesis	8/187	132/18866	5.18E-05	0.000246494	0.000111874	BAX/CA2/PPARA/SERPINE1/COL1A1/RUNX2/EDN1/ADM	8
BP	GO:0050918	positive chemotaxis	6/187	67/18866	5.29E-05	0.000251123	0.000113975	VEGFA/MET/KDR/F3/CXCL8/CXCL10	6
BP	GO:0031334	positive regulation of protein-containing complex assembly	11/187	257/18866	5.33E-05	0.000252846	0.000114757	BAX/MMP1/ICAM1/ESR1/GSK3B/VEGFA/TP53/IFNG/MET/MMP3/PARP1	11
BP	GO:0007254	JNK cascade	10/187	213/18866	5.42E-05	0.00025653	0.000116429	AKT1/MAPK8/GSTP1/EGFR/CD40LG/MMP8/MAPK10/NCF1/IL1B/EDN	10
BP	GO:0016051	carbohydrate biosynthetic process	10/187	213/18866	5.42E-05	0.00025653	0.000116429	AKT1/AKR1B1/GSK3B/PPARA/GOT1/EGF/IGF2/INS/GCG/HRH1	10
BP	GO:0010657	muscle cell apoptotic process	7/187	98/18866	5.43E-05	0.000256676	0.000116495	PPARG/HMOX1/TP53/IFNG/HMGCR/NFE2L2/ED	7
BP	GO:0045833	negative regulation of lipid metabolic process	7/187	98/18866	5.43E-05	0.000256676	0.000116495	AKT1/AKR1C3/ADRA2A/SOD1/UGT1A1/IL1B/I	7
BP	GO:0006913	nucleocytoplasmic transport	13/187	354/18866	5.47E-05	0.000258172	0.000117174	PTGS2/AKT1/CDK1/MAPK14/GSK3B/CDKN1A/MAPK1/TP53/NFKBIA/MDM2/IFNG/STAT3/IL1	13
BP	GO:0010907	positive regulation of glucose metabolic process	5/187	42/18866	5.73E-05	0.000268805	0.000122	AKT1/PPARA/IGF2/INS/GCG	5
BP	GO:0048246	macrophage chemotaxis	5/187	42/18866	5.73E-05	0.000268805	0.000122	MAPK14/MAPK1/MAPK3/CYP19A1/CCL2	5
BP	GO:0071470	cellular response to osmotic stress	5/187	42/18866	5.73E-05	0.000268805	0.000122	PTGS2/CASP3/AKR1B1/MAPK10/BAD	5

BP	GO:1900371	regulation of purine nucleotide biosynthetic process	5/187	42/18866	5.73E-05	0.000268805	0.000122	NOS2/IL4/STAT3/NOS3/PARP1	5
BP	GO:1904591	positive regulation of protein import	5/187	42/18866	5.73E-05	0.000268805	0.000122	PTGS2/CDK1/MAPK14/MAPK1/IFNG	5
BP	GO:0032642	regulation of chemokine production	6/187	68/18866	5.75E-05	0.000269305	0.000122227	IL6R/LBP/GSTP1/HIF1A/IL1B/LPL	6
BP	GO:0040014	regulation of multicellular organism growth	6/187	68/18866	5.75E-05	0.000269305	0.000122227	BCL2/CDK4/SOD1/STAT3/IGF2/DRD2	6
BP	GO:0043280	positive regulation of cysteine-type endopeptidase activity involved in apoptotic process	8/187	134/18866	5.77E-05	0.000269793	0.000122448	BAX/CASP9/CASP8/PPARG/BAD/MYC/F3/CTSD	8
BP	GO:0033209	tumor necrosis factor-mediated signaling pathway	9/187	173/18866	5.84E-05	0.000273019	0.000123913	CASP8/RELA/IKBKB/STAT1/GSTP1/TP53/NFKBIA/CD40LG/CHUK	9
BP	GO:0045088	regulation of innate immune response	12/187	307/18866	5.87E-05	0.000274012	0.000124363	LBP/PPARG/RELA/IKBKB/STAT1/IFNG/XIAP/RAF1/CHUK/IRF1/DRD2/I	12
BP	GO:0035809	regulation of urine volume	4/187	22/18866	5.95E-05	0.000275654	0.000125108	AKR1B1/DRD2/EDN1/ADM	4
BP	GO:0042359	vitamin D metabolic process	4/187	22/18866	5.95E-05	0.000275654	0.000125108	CYP3A4/CYP1A1/IFNG/IL1B	4
BP	GO:0051900	regulation of mitochondrial depolarization	4/187	22/18866	5.95E-05	0.000275654	0.000125108	BCL2/KDR/GOT1/PARP1	4
BP	GO:0071157	negative regulation of cell cycle arrest	4/187	22/18866	5.95E-05	0.000275654	0.000125108	CCND1/CDK4/MDM2/CHEK2	4
BP	GO:1901685	glutathione derivative metabolic process	4/187	22/18866	5.95E-05	0.000275654	0.000125108	GSTP1/GSTM1/GSTA1/GSTA2	4
BP	GO:1901687	glutathione derivative biosynthetic process	4/187	22/18866	5.95E-05	0.000275654	0.000125108	GSTP1/GSTM1/GSTA1/GSTA2	4
BP	GO:0051169	nuclear transport	13/187	357/18866	5.96E-05	0.00027615	0.000125334	PTGS2/AKT1/CDK1/MAPK14/GSK3B/CDKN1A/MAPK1/TP53/NFKBIA/MDM2/IFNG/STAT3/IL1	13
BP	GO:0051651	maintenance of location in cell	10/187	216/18866	6.09E-05	0.000281946	0.000127964	BAX/AKT1/HSPA5/CAV1/GJA1/CXCL11/CXCL10/HK2/DRD2/TRPV1	10
BP	GO:0060038	cardiac muscle cell proliferation	6/187	69/18866	6.25E-05	0.000288769	0.000131061	CDK1/RXRA/MAPK14/MAPK1/CCNB1/GJA1	6
BP	GO:0061448	connective tissue development	11/187	262/18866	6.34E-05	0.000292644	0.000132819	IL6R/RELA/MAPK14/CDK4/MMP13/PPARD/MAPK3/HIF1A/COL1A1/RUNX2/EDN1	11

BP	GO:0050680	negative regulation of epithelial cell proliferation	9/187	175/18866	6.39E-05	0.000294581	0.000133699	AR/PPARG/STAT1/ALOX5/RB1/PPARD/CAV1/GJA1/CCL2	9
BP	GO:0030808	regulation of nucleotide biosynthetic process	5/187	43/18866	6.43E-05	0.000295925	0.000134308	NOS2/IL4/STAT3/NOS3/PARP1	5
BP	GO:0071364	cellular response to epidermal growth factor stimulus	5/187	43/18866	6.43E-05	0.000295925	0.000134308	AKT1/GSTP1/EGFR/ERBB2/COL1A1	5
BP	GO:0042102	positive regulation of T cell proliferation	7/187	101/18866	6.59E-05	0.000302599	0.000137337	VCAM1/IL2RA/IL4/CD40LG/IL1B/IL1A/IGF2	7
BP	GO:0042632	cholesterol homeostasis	7/187	101/18866	6.59E-05	0.000302599	0.000137337	NR1I2/APOB/AKR1C1/CES1/SOAT1/CAV1/LPL	7
BP	GO:0046034	ATP metabolic process	12/187	311/18866	6.65E-05	0.000304852	0.00013836	CDK1/TP53/CCNB1/IFNG/IL4/BAD/PPARA/STAT3/HIF1A/PARP1/HK2/I	12
BP	GO:0016241	regulation of macroautophagy	9/187	176/18866	6.68E-05	0.00030587	0.000138822	CASP3/AKT1/MAPK8/HMOX1/TP53/IL4/KDR/MAPK3/HIF1A	9
BP	GO:0006879	cellular iron ion homeostasis	6/187	70/18866	6.78E-05	0.000310259	0.000140814	HMOX1/IFNG/SOD1/HIF1A/MYC/ABCG2	6
BP	GO:0055074	calcium ion homeostasis	15/187	468/18866	6.91E-05	0.000315796	0.000143327	ADRA1B/BCL2/BAX/ESR1/GOT1/CAV1/GJA1/PTGER3/PRKCB/CXCL11/CXCL10/DRD2/TRPV1/EDN1/ADM	15
BP	GO:0055092	sterol homeostasis	7/187	102/18866	7.02E-05	0.000320296	0.00014537	NR1I2/APOB/AKR1C1/CES1/SOAT1/CAV1/LPL	7
BP	GO:0031330	negative regulation of cellular catabolic process	11/187	265/18866	7.02E-05	0.000320296	0.00014537	BCL2/AKT1/HMOX1/MAPK14/IL10RA/TP53/MCL1/MET/STAT3/E2F1/I	11
BP	GO:0060149	negative regulation of posttranscriptional gene silencing	4/187	23/18866	7.14E-05	0.000324452	0.000147256	PPARG/ESR1/TP53/STAT3	4
BP	GO:0060967	negative regulation of gene silencing by RNA	4/187	23/18866	7.14E-05	0.000324452	0.000147256	PPARG/ESR1/TP53/STAT3	4
BP	GO:1903798	regulation of production of miRNAs involved in gene silencing by	4/187	23/18866	7.14E-05	0.000324452	0.000147256	ESR1/EGFR/TP53/STAT3	4
BP	GO:1904996	positive regulation of leukocyte adhesion to vascular endothelial cell	4/187	23/18866	7.14E-05	0.000324452	0.000147256	RELA/ICAM1/SELE/ALOX5	4
BP	GO:0031670	cellular response to nutrient	5/187	44/18866	7.20E-05	0.000326302	0.000148096	PPARG/HMOX1/MDM2/COL1A1/LPL	5
BP	GO:0032881	regulation of polysaccharide metabolic process	5/187	44/18866	7.20E-05	0.000326302	0.000148096	AKT1/GSK3B/EGF/IGF2/INS	5

BP	GO:0014823	response to activity	6/187	71/18866	7.34E-05	0.000332239	0.00015079	CDK1/CAT/PPARD/HIF1A/EDN1/GCG	6
BP	GO:0032371	regulation of sterol transport	6/187	71/18866	7.34E-05	0.000332239	0.00015079	PON1/PPARG/NFKBIA/CES1/EGF/CAV1	6
BP	GO:0070498	interleukin-1-mediated signaling pathway	7/187	103/18866	7.47E-05	0.000337428	0.000153145	RELA/IKBKB/NFKBIA/MAPK3/IL1B/IL1A/CHU	7
BP	GO:0048469	cell maturation	9/187	179/18866	7.60E-05	0.000343301	0.000155811	BCL2/PPARG/AKR1B1/VEGFA/CDKN1A/RB1/CCNB1/HIF1A/RUNX2	9
BP	GO:0002027	regulation of heart rate	7/187	104/18866	7.94E-05	0.000358031	0.000162496	ADRA1B/MDM2/CAV1/DRD2/TRPV1/EDN1/AD	7
BP	GO:0006775	fat-soluble vitamin metabolic process	5/187	45/18866	8.03E-05	0.00036111	0.000163893	CYP3A4/CYP1A1/IFNG/PPARD/IL1B	5
BP	GO:0032722	positive regulation of chemokine production	5/187	45/18866	8.03E-05	0.00036111	0.000163893	IL6R/LBP/HIF1A/IL1B/LPL	5
BP	GO:0034105	positive regulation of tissue remodeling	5/187	45/18866	8.03E-05	0.00036111	0.000163893	BAX/PRKCA/EGFR/CA2/SPP1	5
BP	GO:0008584	male gonad development	8/187	141/18866	8.26E-05	0.000371092	0.000168424	BCL2/BAX/AR/ICAM1/AKR1C3/ESR1/CCND1/BCL2L1	8
BP	GO:0010950	positive regulation of endopeptidase activity	9/187	181/18866	8.28E-05	0.000371168	0.000168458	BAX/CASP9/CASP8/PPARG/BAD/STAT3/MYC/F3/CTSD	9
BP	GO:0045619	regulation of lymphocyte differentiation	9/187	181/18866	8.28E-05	0.000371168	0.000168458	ERBB2/IL2RA/IFNG/IL4/BAD/SOD1/IL1B/IL1A/I	9
BP	GO:0044321	response to leptin	4/187	24/18866	8.50E-05	0.00037951	0.000172244	CCNA2/CCND1/STAT3/EDN1	4
BP	GO:0050995	negative regulation of lipid catabolic process	4/187	24/18866	8.50E-05	0.00037951	0.000172244	AKT1/ADRA2A/IL1B/INS	4
BP	GO:0051882	mitochondrial depolarization	4/187	24/18866	8.50E-05	0.00037951	0.000172244	BCL2/KDR/GOT1/PARP	4
BP	GO:2000209	regulation of anoikis	4/187	24/18866	8.50E-05	0.00037951	0.000172244	BCL2/MCL1/CAV1/CHEK2	4
BP	GO:0045732	positive regulation of protein catabolic process	10/187	225/18866	8.56E-05	0.000381813	0.00017329	AKT1/ADRA2A/GSK3B/MDM2/IFNG/EGF/CAV1/GJA1/IL1B/NFE2L2	10
BP	GO:0046546	development of primary male sexual characteristics	8/187	142/18866	8.68E-05	0.000386724	0.000175519	BCL2/BAX/AR/ICAM1/AKR1C3/ESR1/CCND1/BCL2L1	8
BP	GO:0010874	regulation of cholesterol efflux	5/187	46/18866	8.94E-05	0.000396057	0.000179755	PON1/NFKBIA/CES1/EGF/CAV1	5
BP	GO:0032570	response to progesterone	5/187	46/18866	8.94E-05	0.000396057	0.000179755	RELA/BAD/FOS/CAV1/CLDN4	5

BP	GO:0061028	establishment of endothelial barrier	5/187	46/18866	8.94E-05	0.000396057	0.000179755	IKBKB/ICAM1/VEGFA/FASN/IL1B	5
BP	GO:0019233	sensory perception of pain	7/187	106/18866	8.96E-05	0.000396057	0.000179755	PTGS2/OPRM1/MAPK1/MAPK3/CCL2/TRPV1/E	7
BP	GO:0032611	interleukin-1 beta production	7/187	106/18866	8.96E-05	0.000396057	0.000179755	CASP8/GSTP1/IFNG/STAT3/IL1B/HSPB1/LPL	7
BP	GO:0071868	cellular response to monoamine stimulus	7/187	106/18866	8.96E-05	0.000396057	0.000179755	OPRM1/GSK3B/MAPK1/HTR3A/MAPK3/DRD2/HRH1	7
BP	GO:0071870	cellular response to catecholamine stimulus	7/187	106/18866	8.96E-05	0.000396057	0.000179755	OPRM1/GSK3B/MAPK1/HTR3A/MAPK3/DRD2/HRH1	7
BP	GO:0008286	insulin receptor signaling pathway	8/187	143/18866	9.12E-05	0.000402874	0.000182849	RELA/AKT1/GSK3B/CDK4/IL1B/PRKCB/IGF2/I	8
BP	GO:0006260	DNA replication	11/187	273/18866	9.15E-05	0.000403783	0.000183261	JUN/CDK1/CDK2/CHEK1/CCNA2/EGFR/TP53/TOP1/PCNA/EGF/CHEK2	11
BP	GO:0106106	cold-induced thermogenesis	8/187	144/18866	9.58E-05	0.000421706	0.000191396	ACHE/VEGFA/RB1/IL4/CAV1/GJA1/UCP2/SCD	8
BP	GO:0120161	regulation of cold-induced thermogenesis	8/187	144/18866	9.58E-05	0.000421706	0.000191396	ACHE/VEGFA/RB1/IL4/CAV1/GJA1/UCP2/SCD	8
BP	GO:0045776	negative regulation of blood pressure	5/187	47/18866	9.93E-05	0.000436575	0.000198144	NOS2/PPARA/NOS3/DRD2/TRPV1	5
BP	GO:0032602	chemokine production	6/187	75/18866	9.99E-05	0.000438109	0.00019884	IL6R/LBP/GSTP1/HIF1A/IL1B/LPL	6
BP	GO:0035924	cellular response to vascular endothelial growth factor stimulus	6/187	75/18866	9.99E-05	0.000438109	0.00019884	RELA/AKT1/MAPK14/VEGFA/KDR/HSPB1	6
BP	GO:0050805	negative regulation of synaptic transmission	6/187	75/18866	9.99E-05	0.000438109	0.00019884	PTGS2/SLC6A4/ACHE/IL1B/DRD2/BDNF	6
BP	GO:0002092	positive regulation of receptor internalization	4/187	25/18866	0.00010046	0.000438109	0.00019884	SELE/VEGFA/EGF/DRD2	4
BP	GO:0050927	positive regulation of positive chemotaxis	4/187	25/18866	0.00010046	0.000438109	0.00019884	VEGFA/KDR/F3/CXCL8	4
BP	GO:0070920	regulation of production of small RNA involved in gene silencing by	4/187	25/18866	0.00010046	0.000438109	0.00019884	ESR1/EGFR/TP53/STAT3	4
BP	GO:1904385	cellular response to angiotensin	4/187	25/18866	0.00010046	0.000438109	0.00019884	RELA/CA2/CAV1/NFE2	4
BP	GO:2001026	regulation of endothelial cell chemotaxis	4/187	25/18866	0.00010046	0.000438109	0.00019884	VEGFA/MET/KDR/HSPB1	4
BP	GO:0071322	cellular response to carbohydrate stimulus	8/187	145/18866	0.000100521	0.000438109	0.00019884	ICAM1/ADRA2A/BAD/HMGCR/RAF1/HIF1A/PRKCB/GCG	8

BP	GO:0032526	response to retinoic acid	7/187	108/18866	0.000100807	0.000438463	0.000199001	SLC6A4/PPARG/RXRA/PTGES/RXRB/GJA1/COL1A1	7
BP	GO:0071887	leukocyte apoptotic process	7/187	108/18866	0.000100807	0.000438463	0.000199001	BAX/CASP9/CASP3/AKT1/TP53/IL2RA/HIF1A	7
BP	GO:0051147	regulation of muscle cell differentiation	9/187	186/18866	0.000102028	0.000443325	0.000201207	BCL2/MAPK14/MDM2/IL4/PPARA/CXCL10/IGF2/EDN1/BDNF	9
BP	GO:0001649	osteoblast differentiation	10/187	231/18866	0.000106456	0.000461634	0.000209517	IL6R/ACHE/AKT1/CAT/FASN/COL1A1/SPP1/RUNX2/IGFBP3/IGF2	10
BP	GO:0048738	cardiac muscle tissue development	10/187	231/18866	0.000106456	0.000461634	0.000209517	CDK1/RXRA/MAPK14/VEGFA/MAPK1/CCNB1/PPARA/GJA1/ERBB3/ED	10
BP	GO:0033077	T cell differentiation in thymus	6/187	76/18866	0.000107615	0.000465716	0.00021137	BCL2/TP53/ERBB2/SOD1/IL1B/IL1A	6
BP	GO:0072091	regulation of stem cell proliferation	6/187	76/18866	0.000107615	0.000465716	0.00021137	VEGFA/TP53/HIF1A/GJA1/DRD2/BDNF	6
BP	GO:0010749	regulation of nitric oxide mediated signal transduction	3/187	10/18866	0.000109256	0.000470919	0.000213731	EGFR/VEGFA/INS	3
BP	GO:0030647	aminoglycoside antibiotic metabolic process	3/187	10/18866	0.000109256	0.000470919	0.000213731	AKR1C3/AKR1B1/AKR1C1	3
BP	GO:0032025	response to cobalt ion	3/187	10/18866	0.000109256	0.000470919	0.000213731	CASP9/CASP3/CASP8	3
BP	GO:0033083	regulation of immature T cell proliferation	3/187	10/18866	0.000109256	0.000470919	0.000213731	ERBB2/IL1B/IL1A	3
BP	GO:0051972	regulation of telomerase activity	5/187	48/18866	0.000109911	0.000471984	0.000214215	PPARG/MAPK1/TP53/MAPK3/MYC	5
BP	GO:0055023	positive regulation of cardiac muscle tissue growth	5/187	48/18866	0.000109911	0.000471984	0.000214215	CDK1/MAPK14/MAPK1/CCNB1/EDN1	5
BP	GO:0060324	face development	5/187	48/18866	0.000109911	0.000471984	0.000214215	MMP2/MAPK1/MAPK3/RAF1/COL1A1	5
BP	GO:0006898	receptor-mediated endocytosis	12/187	328/18866	0.000110053	0.000471984	0.000214215	CD14/ACHE/SELE/VEGFA/IL4/APOB/EGF/CAV1/CXCL8/SERPINE1/DRD2/ADM	12
BP	GO:0042113	B cell activation	12/187	328/18866	0.000110053	0.000471984	0.000214215	BCL2/BAX/CASP3/CASP8/VCAM1/AHR/CDKN1A/TP53/IL4/CD40LG/BAD/PRKCB	12
BP	GO:0043123	positive regulation of I-kappaB kinase/NF-kappaB signaling	9/187	188/18866	0.000110688	0.000473763	0.000215022	CASP8/RELA/IKBKB/AKT1/HMOX1/GJA1/IL1B/PRKCB/CHUK	9

BP	GO:0051783	regulation of nuclear division	9/187	188/18866	0.000110688	0.000473763	0.000215022	CHEK1/RB1/CCNB1/EGF/IL1B/IL1A/IGF2/EDN1/INS	9
BP	GO:0071867	response to monoamine	7/187	110/18866	0.000113151	0.000483342	0.00021937	OPRM1/GSK3B/MAPK1/HTR3A/MAPK3/DRD2/HRH1	7
BP	GO:0071869	response to catecholamine	7/187	110/18866	0.000113151	0.000483342	0.00021937	OPRM1/GSK3B/MAPK1/HTR3A/MAPK3/DRD2/HRH1	7
BP	GO:0030810	positive regulation of nucleotide biosynthetic process	4/187	26/18866	0.00011781	0.000501246	0.000227495	NOS2/IL4/STAT3/NOS3	4
BP	GO:0042730	fibrinolysis	4/187	26/18866	0.00011781	0.000501246	0.000227495	PLAU/THBD/SERPINE1/SERPINB2	4
BP	GO:0050926	regulation of positive chemotaxis	4/187	26/18866	0.00011781	0.000501246	0.000227495	VEGFA/KDR/F3/CXCL8	4
BP	GO:1900373	positive regulation of purine nucleotide biosynthetic process	4/187	26/18866	0.00011781	0.000501246	0.000227495	NOS2/IL4/STAT3/NOS3	4
BP	GO:0034440	lipid oxidation	7/187	111/18866	0.000119769	0.000509078	0.00023105	PPARG/AKT1/ALOX5/MAPK14/PPARD/PPARA/FABP1	7
BP	GO:0006575	cellular modified amino acid metabolic process	9/187	190/18866	0.000119956	0.000509369	0.000231182	GSTP1/GSTM1/DPEP1/SOD1/GSR/DUOX2/NFE2L2/GSTA1/GSTA2	9
BP	GO:0072503	cellular divalent inorganic cation homeostasis	15/187	492/18866	0.000120274	0.000510214	0.000231566	ADRA1B/BCL2/BAX/ESR1/GOT1/CAV1/GJA1/PTGER3/PRKCB/CXCL11/CXCL10/DRD2/TRPV1/EDN1/ADM	15
BP	GO:0002686	negative regulation of leukocyte migration	5/187	49/18866	0.000121425	0.000512565	0.000232633	DPP4/AKT1/HMOX1/CYP19A1/CCL2	5
BP	GO:0007595	lactation	5/187	49/18866	0.000121425	0.000512565	0.000232633	VEGFA/CCND1/HIF1A/CAV1/HK2	5
BP	GO:0070231	T cell apoptotic process	5/187	49/18866	0.000121425	0.000512565	0.000232633	BAX/AKT1/TP53/IL2RA/HIF1A	5
BP	GO:1900087	positive regulation of G1/S transition of mitotic cell cycle	5/187	49/18866	0.000121425	0.000512565	0.000232633	AKT1/CYP1A1/EGFR/CCND1/MDM2	5
BP	GO:1990090	cellular response to nerve growth factor stimulus	5/187	49/18866	0.000121425	0.000512565	0.000232633	AKT1/HSPA5/E2F1/TRPV1/BDNF	5
BP	GO:0071695	anatomical structure maturation	10/187	235/18866	0.000122586	0.000516958	0.000234627	BCL2/PPARG/AKR1B1/VEGFA/CDKN1A/MMP2/RB1/CCNB1/HIF1A/RU	10
BP	GO:0035264	multicellular organism growth	8/187	150/18866	0.000127269	0.000536176	0.000243349	BCL2/AR/CDK4/TP53/SOD1/STAT3/IGF2/DRD2	8

BP	GO:2001056	positive regulation of cysteine-type endopeptidase activity	8/187	151/18866	0.000133266	0.000559476	0.000253924	BAX/CASP9/CASP8/PPARG/BAD/MYC/F3/CTSD	8
BP	GO:0031016	pancreas development	6/187	79/18866	0.00013351	0.000559476	0.000253924	IL6R/AKT1/GSK3B/MET/BAD/IGF2	6
BP	GO:0001774	microglial cell activation	5/187	50/18866	0.000133841	0.000559476	0.000253924	JUN/IFNG/IL4/MMP8/TRPV1	5
BP	GO:0002269	leukocyte activation involved in inflammatory response	5/187	50/18866	0.000133841	0.000559476	0.000253924	JUN/IFNG/IL4/MMP8/TRPV1	5
BP	GO:0003044	regulation of systemic arterial blood pressure mediated by a chemical signal	5/187	50/18866	0.000133841	0.000559476	0.000253924	ADRA1B/CES1/NOS3/EDN1/ENPEP	5
BP	GO:0071715	icosanoid transport	5/187	50/18866	0.000133841	0.000559476	0.000253924	NOS2/ABCC1/IL1B/DRD2/EDN1	5
BP	GO:0097300	programmed necrotic cell death	5/187	50/18866	0.000133841	0.000559476	0.000253924	CD14/BAX/CASP8/TP53/CAV1	5
BP	GO:1901571	fatty acid derivative transport	5/187	50/18866	0.000133841	0.000559476	0.000253924	NOS2/ABCC1/IL1B/DRD2/EDN1	5
BP	GO:0006706	steroid catabolic process	4/187	27/18866	0.000137232	0.000571976	0.000259597	CYP3A4/CYP1A2/CYP19A1/SPP1	4
BP	GO:0010971	positive regulation of G2/M transition of mitotic cell cycle	4/187	27/18866	0.000137232	0.000571976	0.000259597	CDK1/CCND1/CDK4/CCNB1	4
BP	GO:0033081	regulation of T cell differentiation in thymus	4/187	27/18866	0.000137232	0.000571976	0.000259597	ERBB2/SOD1/IL1B/IL1A	4
BP	GO:0060047	heart contraction	11/187	287/18866	0.000142192	0.000592078	0.00026872	ADRA1B/MDM2/SOD1/CAV1/GJA1/NOS3/DRD2/TRPV1/EDN1/ADM/CACNA1S	11
BP	GO:0001678	cellular glucose homeostasis	8/187	153/18866	0.000145962	0.000606785	0.000275396	ICAM1/ADRA2A/BAD/HMGCR/RAF1/HIF1A/HK2/GCG	8
BP	GO:1902275	regulation of chromatin organization	9/187	195/18866	0.000146007	0.000606785	0.000275396	MAPK8/CHEK1/VEGFA/TP53/CCNB1/MAPK3/IL1B/IGF2/GCG	9
BP	GO:0045912	negative regulation of carbohydrate metabolic process	5/187	51/18866	0.000147207	0.000610588	0.000277121	GSK3B/TP53/PPARA/STAT3/INS	5
BP	GO:0071354	cellular response to interleukin-6	5/187	51/18866	0.000147207	0.000610588	0.000277121	IL6R/RELA/STAT1/ICAM1/STAT3	5
BP	GO:0006857	oligopeptide transport	3/187	11/18866	0.000149131	0.000615005	0.000279126	CA2/ABCC1/GJA1	3
BP	GO:0031652	positive regulation of heat	3/187	11/18866	0.000149131	0.000615005	0.000279126	PTGS2/IL1B/PTGER3	3
BP	GO:0033079	immature T cell proliferation	3/187	11/18866	0.000149131	0.000615005	0.000279126	ERBB2/IL1B/IL1A	3
BP	GO:0033483	gas homeostasis	3/187	11/18866	0.000149131	0.000615005	0.000279126	GSTP1/HIF1A/CAV1	3

BP	GO:0043619	regulation of transcription from RNA polymerase II promoter in response to oxidative stress	3/187	11/18866	0.000149131	0.000615005	0.000279126	HMOX1/HIF1A/NFE2L2	3
BP	GO:0051712	positive regulation of killing of cells of other organism	3/187	11/18866	0.000149131	0.000615005	0.000279126	NOS2/IFNG/BAD	3
BP	GO:0071897	DNA biosynthetic process	9/187	196/18866	0.000151747	0.000625191	0.000283749	PPARG/CDKN1A/MAPK1/TP53/PCNA/MAPK3/MYC/GJA1/TEP1	9
BP	GO:0001937	negative regulation of endothelial cell proliferation	6/187	81/18866	0.000153354	0.000631206	0.000286479	PPARG/STAT1/ALOX5/CAV1/GJA1/CCL2	6
BP	GO:0030194	positive regulation of blood coagulation	4/187	28/18866	0.000158869	0.000650788	0.000295366	F3/THBD/SERPINE1/NFE2L2	4
BP	GO:0033598	mammary gland epithelial cell proliferation	4/187	28/18866	0.000158869	0.000650788	0.000295366	BAX/ESR1/CCND1/MAPK1	4
BP	GO:0090200	positive regulation of release of cytochrome c from mitochondria	4/187	28/18866	0.000158869	0.000650788	0.000295366	BAX/MMP9/TP53/BAD	4
BP	GO:1900048	positive regulation of hemostasis	4/187	28/18866	0.000158869	0.000650788	0.000295366	F3/THBD/SERPINE1/NFE2L2	4
BP	GO:1903579	negative regulation of ATP metabolic process	4/187	28/18866	0.000158869	0.000650788	0.000295366	TP53/PPARA/STAT3/PARP1	4
BP	GO:0043254	regulation of protein-containing complex assembly	14/187	449/18866	0.000161235	0.000659331	0.000299244	BAX/MMP1/ICAM1/ESR1/GSK3B/VEGFA/RB1/TP53/IFNG/MET/MMP3/RAF1/HSPA5/PARP1	14
BP	GO:0000186	activation of MAPKK activity	5/187	52/18866	0.000161569	0.000659331	0.000299244	EGFR/MAPK1/MAPK3/EGF/RAF1	5
BP	GO:0060421	positive regulation of heart growth	5/187	52/18866	0.000161569	0.000659331	0.000299244	CDK1/MAPK14/MAPK1/CCNB1/EDN1	5
BP	GO:1990089	response to nerve growth factor	5/187	52/18866	0.000161569	0.000659331	0.000299244	AKT1/HSPA5/E2F1/TRPV1/BDNF	5
BP	GO:0090150	establishment of protein localization to membrane	12/187	342/18866	0.000162561	0.000662749	0.000300795	BCL2/BAX/CASP8/MAPK8/EGFR/TP53/ERBB2/MAPK10/NCF1/BAD/HSPA5/E2F1	12
BP	GO:0045446	endothelial cell differentiation	7/187	117/18866	0.000166396	0.000677742	0.0003076	IKBKB/ICAM1/VEGFA/MET/KDR/FASN/IL1B	7
BP	GO:0010721	negative regulation of cell development	12/187	343/18866	0.000167018	0.00067963	0.000308457	SLC6A4/BCL2/GSK3B/VEGFA/TP53/MDM2/PPARA/STAT3/IL1B/SPP1/TRPV1/BDNF	12

BP	GO:0051604	protein maturation	11/187	293/18866	0.000170305	0.000692346	0.000314228	CASP3/CASP8/PLAU/MDM2/XIAP/BAD/CES1/F3/SERPINE1/PARP1/ENP	11
BP	GO:0048708	astrocyte differentiation	6/187	83/18866	0.000175462	0.000711964	0.000323132	EGFR/MAPK1/IFNG/MAPK3/STAT3/IL1B	6
BP	GO:0071277	cellular response to calcium ion	6/187	83/18866	0.000175462	0.000711964	0.000323132	JUN/AKR1C3/DPEP1/FOS/HSPA5/EDN1	6
BP	GO:0032731	positive regulation of interleukin-1 beta production	5/187	53/18866	0.000176978	0.000716085	0.000325002	CASP8/IFNG/STAT3/HSPB1/LPL	5
BP	GO:0045744	negative regulation of G protein-coupled receptor signaling pathway	5/187	53/18866	0.000176978	0.000716085	0.000325002	OPRM1/MET/CXCL8/DRD2/ADM	5
BP	GO:0061756	leukocyte adhesion to vascular endothelial cell	5/187	53/18866	0.000176978	0.000716085	0.000325002	RELA/ICAM1/SELE/VCAM1/ALOX5	5
BP	GO:0006469	negative regulation of protein kinase activity	10/187	246/18866	0.000177965	0.000719402	0.000326508	CASP3/AKT1/GSTP1/CDKN1A/RB1/IFNG/HMGCR/CAV1/IL1B/HSPB1	10
BP	GO:0001782	B cell homeostasis	4/187	29/18866	0.000182868	0.000735067	0.000333617	BCL2/BAX/CASP3/HIF1	4
BP	GO:0019048	modulation by virus of host process	4/187	29/18866	0.000182868	0.000735067	0.000333617	CASP8/RXRA/BCL2L1/BAD	4
BP	GO:0032373	positive regulation of sterol transport	4/187	29/18866	0.000182868	0.000735067	0.000333617	PON1/NFKBIA/CES1/CAV1	4
BP	GO:0032376	positive regulation of cholesterol transport	4/187	29/18866	0.000182868	0.000735067	0.000333617	PON1/NFKBIA/CES1/CAV1	4
BP	GO:0050820	positive regulation of coagulation	4/187	29/18866	0.000182868	0.000735067	0.000333617	F3/THBD/SERPINE1/NFE2L2	4
BP	GO:0071624	positive regulation of granulocyte chemotaxis	4/187	29/18866	0.000182868	0.000735067	0.000333617	LBP/IL4/CXCL8/EDN1	4
BP	GO:0051153	regulation of striated muscle cell differentiation	7/187	119/18866	0.000184854	0.000742354	0.000336925	BCL2/MAPK14/IL4/PPARA/CXCL10/EDN1/BDN	7
BP	GO:0050886	endocrine process	6/187	84/18866	0.000187421	0.000751962	0.000341285	CES1/GJA1/IL1B/NOS3/EDN1/ENPEP	6
BP	GO:0009150	purine ribonucleotide metabolic process	13/187	401/18866	0.000189766	0.000760657	0.000345232	IFNG/IL4/FASN/BAD/HMGCR/PPARA/STAT3/HIF1A/ACACA/PARP1/HK2/INS/SCD	13
BP	GO:0032675	regulation of interleukin-6 production	8/187	159/18866	0.00019017	0.000761566	0.000345644	IL6R/LBP/IFNG/MMP8/STAT3/IL1B/IL1A/LPL	8
BP	GO:0003015	heart process	11/187	297/18866	0.00019156	0.000766418	0.000347846	ADRA1B/MDM2/SOD1/CAV1/GJA1/NOS3/DRD2/TRPV1/EDN1/ADM/CACNA1S	11
BP	GO:0006983	ER overload response	3/187	12/18866	0.000197392	0.00078609	0.000356775	GSK3B/TP53/HSPA5	3

BP	GO:0042178	xenobiotic catabolic process	3/187	12/18866	0.000197392	0.00078609	0.000356775	CYP1A1/GSTM1/UGT1A	3
BP	GO:0042368	vitamin D biosynthetic process	3/187	12/18866	0.000197392	0.00078609	0.000356775	CYP3A4/IFNG/IL1B	3
BP	GO:0060736	prostate gland growth	3/187	12/18866	0.000197392	0.00078609	0.000356775	AR/ESR1/CYP19A1	3
BP	GO:0072584	caveolin-mediated endocytosis	3/187	12/18866	0.000197392	0.00078609	0.000356775	MAPK1/MAPK3/CAV1	3
BP	GO:0030218	erythrocyte differentiation	7/187	121/18866	0.000204932	0.000814603	0.000369716	CASP3/STAT1/MAPK14/VEGFA/RB1/STAT3/HIF	7
BP	GO:0032612	interleukin-1 production	7/187	121/18866	0.000204932	0.000814603	0.000369716	CASP8/GSTP1/IFNG/STAT3/IL1B/HSPB1/LPL	7
BP	GO:0005979	regulation of glycogen biosynthetic process	4/187	30/18866	0.000209375	0.000827669	0.000375646	AKT1/GSK3B/IGF2/INS	4
BP	GO:0010758	regulation of macrophage chemotaxis	4/187	30/18866	0.000209375	0.000827669	0.000375646	MAPK14/MAPK1/MAPK3/CYP19A1	4
BP	GO:0010962	regulation of glucan biosynthetic process	4/187	30/18866	0.000209375	0.000827669	0.000375646	AKT1/GSK3B/IGF2/INS	4
BP	GO:0035666	TRIF-dependent toll-like receptor signaling pathway	4/187	30/18866	0.000209375	0.000827669	0.000375646	CD14/CASP8/IKBKB/CHUK	4
BP	GO:0060055	angiogenesis involved in wound healing	4/187	30/18866	0.000209375	0.000827669	0.000375646	ALOX5/VEGFA/KDR/SERPINE1	4
BP	GO:1902751	positive regulation of cell cycle G2/M phase transition	4/187	30/18866	0.000209375	0.000827669	0.000375646	CDK1/CCND1/CDK4/CCNB1	4
BP	GO:0070741	response to interleukin-6	5/187	55/18866	0.000211137	0.000833864	0.000378458	IL6R/RELA/STAT1/ICAM1/STAT3	5
BP	GO:0001776	leukocyte homeostasis	6/187	86/18866	0.000213267	0.000839188	0.000380874	BCL2/BAX/CASP3/AKT1/IL2RA/HIF1A	6
BP	GO:0006919	activation of cysteine-type endopeptidase activity involved in apoptotic process	6/187	86/18866	0.000213267	0.000839188	0.000380874	BAX/CASP9/CASP8/PPARG/BAD/F3	6
BP	GO:0034637	cellular carbohydrate biosynthetic process	6/187	86/18866	0.000213267	0.000839188	0.000380874	AKT1/AKR1B1/GSK3B/GOT1/IGF2/INS	6
BP	GO:0045445	myoblast differentiation	6/187	86/18866	0.000213267	0.000839188	0.000380874	MAPK14/RB1/PPARD/HMGCR/CXCL10/IGFBP3	6
BP	GO:0071560	cellular response to transforming growth factor beta stimulus	10/187	252/18866	0.00021617	0.000849833	0.000385705	JUN/TP53/NR3C1/GOT1/FOS/HSPA5/CAV1/COL1A1/PARP1/EDN1	10
BP	GO:0031214	biomineral tissue development	8/187	163/18866	0.000225344	0.000884281	0.00040134	PTGS2/ALOX5/MMP13/PPARA/HIF1A/NOS3/COL1A1/SPP1	8
BP	GO:0110148	biomineralization	8/187	163/18866	0.000225344	0.000884281	0.00040134	PTGS2/ALOX5/MMP13/PPARA/HIF1A/NOS3/COL1A1/SPP1	8
BP	GO:0051781	positive regulation of cell division	6/187	87/18866	0.000227201	0.000889939	0.000403908	CAT/VEGFA/IL1B/IL1A/IGF2/DRD2	6

BP	GO:0055072	iron ion homeostasis	6/187	87/18866	0.000227201	0.000889939	0.000403908	HMOX1/IFNG/SOD1/HIF1A/MYC/ABCG2	6
BP	GO:0090183	regulation of kidney development	5/187	56/18866	0.000229991	0.000900045	0.000408494	IL6R/STAT1/VEGFA/MMP9/MYC	5
BP	GO:0046661	male sex differentiation	8/187	164/18866	0.000234924	0.000918515	0.000416877	BCL2/BAX/AR/ICAM1/AKR1C3/ESR1/CCND1/BCL2L1	8
BP	GO:0022612	gland morphogenesis	7/187	124/18866	0.000238316	0.000926753	0.000420616	BCL2/BAX/AR/ESR1/RXRA/EGFR/CAV1	7
BP	GO:0046031	ADP metabolic process	7/187	124/18866	0.000238316	0.000926753	0.000420616	IFNG/BAD/PPARA/STAT3/HIF1A/HK2/INS	7
BP	GO:0014072	response to isoquinoline alkaloid	4/187	31/18866	0.000238542	0.000926753	0.000420616	OPRM1/RELA/MDM2/DRD2	4
BP	GO:0043278	response to morphine	4/187	31/18866	0.000238542	0.000926753	0.000420616	OPRM1/RELA/MDM2/DRD2	4
BP	GO:0045737	positive regulation of cyclin-dependent protein serine/threonine	4/187	31/18866	0.000238542	0.000926753	0.000420616	AKT1/EGFR/CCND1/CCNB1	4
BP	GO:0045940	positive regulation of steroid metabolic process	4/187	31/18866	0.000238542	0.000926753	0.000420616	IFNG/CES1/IL1B/ADM	4
BP	GO:0071549	cellular response to dexamethasone stimulus	4/187	31/18866	0.000238542	0.000926753	0.000420616	CASP9/ICAM1/EGFR/NR3C1	4
BP	GO:0014068	positive regulation of phosphatidylinositol 3-kinase	6/187	88/18866	0.000241842	0.000937876	0.000425664	CAT/KDR/PPARD/NCF1/ERBB3/INS	6
BP	GO:0046889	positive regulation of lipid biosynthetic process	6/187	88/18866	0.000241842	0.000937876	0.000425664	PTGS2/AKT1/IFNG/IL1B/INS/ADM	6
BP	GO:0008016	regulation of heart contraction	10/187	256/18866	0.00024529	0.00094953	0.000430954	ADRA1B/MDM2/CAV1/GJA1/NOS3/DRD2/TRPV1/EDN1/ADM/CACNA1S	10
BP	GO:0090257	regulation of muscle system process	10/187	256/18866	0.00024529	0.00094953	0.000430954	PTGS2/ADRA1B/PRKCA/ADRA2A/SOD1/PPARA/CAV1/NOS3/PARP1/ED	10
BP	GO:0016570	histone modification	14/187	468/18866	0.000246687	0.000954077	0.000433018	PRKCA/MAPK8/CDK1/CDK2/CHEK1/CCNA2/VEGFA/TP53/CCNB1/MAPK3/IL1B/PRKCB/IGF2/G	14
BP	GO:0061178	regulation of insulin secretion involved in cellular response to glucose stimulus	5/187	57/18866	0.000250098	0.000965532	0.000438216	ADRA2A/BAD/HMGCR/HIF1A/GCG	5
BP	GO:1905517	macrophage migration	5/187	57/18866	0.000250098	0.000965532	0.000438216	MAPK14/MAPK1/MAPK3/CYP19A1/CCL2	5
BP	GO:0043500	muscle adaptation	7/187	125/18866	0.00025037	0.000965713	0.000438299	PRKCA/HMOX1/PPARA/IL1B/NOS3/PARP1/EDN	7

BP	GO:0030100	regulation of endocytosis	9/187	210/18866	0.000254045	0.000974688	0.000442372	CD14/PPARG/SELE/VEGFA/IL4/EGF/CAV1/SERPINE1/DRD2	9
BP	GO:0002679	respiratory burst involved in defense response	3/187	13/18866	0.00025474	0.000974688	0.000442372	LBP/MPO/INS	3
BP	GO:0010745	negative regulation of macrophage derived foam cell differentiation	3/187	13/18866	0.00025474	0.000974688	0.000442372	PPARG/NFKBIA/PPARA	3
BP	GO:0010870	positive regulation of receptor biosynthetic process	3/187	13/18866	0.00025474	0.000974688	0.000442372	IFNG/HIF1A/EDN1	3
BP	GO:0016264	gap junction assembly	3/187	13/18866	0.00025474	0.000974688	0.000442372	CAV1/GJA1/IL1B	3
BP	GO:0030656	regulation of vitamin metabolic process	3/187	13/18866	0.00025474	0.000974688	0.000442372	AKR1C3/IFNG/IL1B	3
BP	GO:0031650	regulation of heat generation	3/187	13/18866	0.00025474	0.000974688	0.000442372	PTGS2/IL1B/PTGER3	3
BP	GO:0033127	regulation of histone	3/187	13/18866	0.00025474	0.000974688	0.000442372	CCNB1/MAPK3/IL1B	3
BP	GO:0045741	positive regulation of epidermal growth factor-activated receptor activity	3/187	13/18866	0.00025474	0.000974688	0.000442372	ADRA2A/NCF1/EGF	3
BP	GO:0051607	defense response to virus	10/187	258/18866	0.000261041	0.000997018	0.000452507	BCL2/RELA/STAT1/BCL2L1/IL2RA/IFNG/IL4/IL1B/CXCL10/IRF1	10
BP	GO:0071559	response to transforming growth factor beta	10/187	258/18866	0.000261041	0.000997018	0.000452507	JUN/TP53/NR3C1/GOT1/FOS/HSPA5/CAV1/COL1A1/PARP1/EDN1	10
BP	GO:0035767	endothelial cell chemotaxis	4/187	32/18866	0.00027052	0.001030473	0.000467691	VEGFA/MET/KDR/HSP	4
BP	GO:0046320	regulation of fatty acid oxidation	4/187	32/18866	0.00027052	0.001030473	0.000467691	PPARG/AKT1/PPARA/FABP1	4
BP	GO:0097421	liver regeneration	4/187	32/18866	0.00027052	0.001030473	0.000467691	HMOX1/EGFR/CCND1/PCNA	4
BP	GO:0009259	ribonucleotide metabolic process	13/187	416/18866	0.00027087	0.001030892	0.000467881	IFNG/IL4/FASN/BAD/HMGCR/PPARA/STAT3/HIF1A/ACACA/PARP1/HK2/INS/SCD	13
BP	GO:0031294	lymphocyte costimulation	5/187	58/18866	0.000271514	0.001032427	0.000468577	DPP4/AKT1/IL4/CD40LG/CAV1	5
BP	GO:0042058	regulation of epidermal growth factor receptor signaling pathway	6/187	90/18866	0.000273348	0.001037561	0.000470908	AKT1/ADRA2A/EGFR/MMP9/NCF1/EGF	6
BP	GO:0043470	regulation of carbohydrate catabolic process	6/187	90/18866	0.000273348	0.001037561	0.000470908	TP53/IFNG/PPARA/STAT3/HIF1A/INS	6
BP	GO:0006936	muscle contraction	12/187	362/18866	0.000273898	0.001038732	0.000471439	PTGS2/ADRA1B/ADRA2A/SOD1/CAV1/GJA1/PTGER3/CHUK/DRD2/TRPV1/EDN1/CACNA1S	12

BP	GO:0034763	negative regulation of transmembrane transport	7/187	128/18866	0.000289506	0.001096955	0.000497864	AKT1/ADRA2A/MMP9/CAV1/IL1B/PRKCB/DR	7
BP	GO:0034103	regulation of tissue remodeling	6/187	91/18866	0.000290264	0.001097888	0.000498287	BAX/PRKCA/EGFR/TP53/CA2/SPP1	6
BP	GO:0042475	odontogenesis of dentin-containing tooth	6/187	91/18866	0.000290264	0.001097888	0.000498287	BAX/CA2/PPARA/SERPINE1/RUNX2/ADM	6
BP	GO:0042306	regulation of protein import into nucleus	5/187	59/18866	0.000294294	0.001110196	0.000503874	PTGS2/CDK1/MAPK14/MAPK1/IFNG	5
BP	GO:0050707	regulation of cytokine secretion	5/187	59/18866	0.000294294	0.001110196	0.000503874	CD14/IFNG/IL1A/DRD2/INS	5
BP	GO:0060043	regulation of cardiac muscle cell proliferation	5/187	59/18866	0.000294294	0.001110196	0.000503874	CDK1/MAPK14/MAPK1/CCNB1/GJA1	5
BP	GO:0048384	retinoic acid receptor signaling pathway	4/187	33/18866	0.000305464	0.001149303	0.000521623	PPARG/AKR1C3/RXRA/RXRB	4
BP	GO:0090022	regulation of neutrophil chemotaxis	4/187	33/18866	0.000305464	0.001149303	0.000521623	LBP/DPP4/CXCL8/EDN1	4
BP	GO:1905048	regulation of metallopeptidase activity	4/187	33/18866	0.000305464	0.001149303	0.000521623	MAPK14/MAPK3/STAT3/CLDN4	4
BP	GO:0031058	positive regulation of histone modification	6/187	92/18866	0.000307991	0.001155773	0.000524559	VEGFA/TP53/CCNB1/MAPK3/IL1B/GCG	6
BP	GO:0045582	positive regulation of T cell differentiation	6/187	92/18866	0.000307991	0.001155773	0.000524559	IL2RA/IFNG/IL4/BAD/IL1B/IL1A	6
BP	GO:1905897	regulation of response to endoplasmic reticulum stress	6/187	92/18866	0.000307991	0.001155773	0.000524559	BAX/ALOX5/BCL2L1/HSPA5/CAV1/NFE2L2	6
BP	GO:0030307	positive regulation of cell growth	8/187	171/18866	0.000311758	0.001167867	0.000530048	BCL2/AKT1/EGFR/VEGFA/ERBB2/EDN1/BDNF/INS	8
BP	GO:0051302	regulation of cell division	8/187	171/18866	0.000311758	0.001167867	0.000530048	CAT/VEGFA/BCL2L1/MYC/IL1B/IL1A/IGF2/DR	8
BP	GO:0048863	stem cell differentiation	10/187	264/18866	0.000313469	0.001173253	0.000532493	ESR1/MAPK1/TP53/MAPK3/STAT3/HIF1A/NFE2L2/RUNX2/CDK12/ED	10
BP	GO:0032732	positive regulation of interleukin-1 production	5/187	60/18866	0.000318494	0.001188951	0.000539617	CASP8/IFNG/STAT3/HSPB1/LPL	5
BP	GO:0061098	positive regulation of protein tyrosine kinase activity	5/187	60/18866	0.000318494	0.001188951	0.000539617	ADRA2A/NCF1/EGF/ERBB3/BDNF	5
BP	GO:1902808	positive regulation of cell cycle G1/S phase transition	5/187	60/18866	0.000318494	0.001188951	0.000539617	AKT1/CYP1A1/EGFR/CCND1/MDM2	5
BP	GO:0010889	regulation of sequestering of triglyceride	3/187	14/18866	0.000321854	0.001194224	0.000542011	PPARG/PPARA/LPL	3
BP	GO:0032310	prostaglandin secretion	3/187	14/18866	0.000321854	0.001194224	0.000542011	NOS2/IL1B/EDN1	3
BP	GO:0046321	positive regulation of fatty acid oxidation	3/187	14/18866	0.000321854	0.001194224	0.000542011	PPARG/PPARA/FABP1	3

BP	GO:0051709	regulation of killing of cells of other organism	3/187	14/18866	0.000321854	0.001194224	0.000542011	NOS2/IFNG/BAD	3
BP	GO:0071236	cellular response to antibiotic	3/187	14/18866	0.000321854	0.001194224	0.000542011	TP53/MDM2/HSPA5	3
BP	GO:1902894	negative regulation of pri-miRNA transcription by RNA polymerase II	3/187	14/18866	0.000321854	0.001194224	0.000542011	RELA/PPARD/PPARA	3
BP	GO:1903799	negative regulation of production of miRNAs involved in gene silencing by miRNA	3/187	14/18866	0.000321854	0.001194224	0.000542011	ESR1/TP53/STAT3	3
BP	GO:0050806	positive regulation of synaptic transmission	8/187	172/18866	0.00032424	0.001202039	0.000545557	PTGS2/GSK3B/EGFR/MAPK1/CA2/CCL2/DRD2/	8
BP	GO:0016569	covalent chromatin modification	14/187	481/18866	0.000325482	0.001205605	0.000547176	PRKCA/MAPK8/CDK1/CDK2/CHEK1/CCNA2/VEGFA/TP53/CCNB1/MAPK3/IL1B/PRKCB/IGF2/G	14
BP	GO:0045185	maintenance of protein location	6/187	93/18866	0.000326556	0.001207498	0.000548035	AKT1/NFKBIA/HSPA5/CAV1/GJA1/HK2	6
BP	GO:0061097	regulation of protein tyrosine kinase activity	6/187	93/18866	0.000326556	0.001207498	0.000548035	ADRA2A/NCF1/EGF/CAV1/ERBB3/BDNF	6
BP	GO:0009165	nucleotide biosynthetic process	10/187	266/18866	0.00033279	0.001229489	0.000558016	PTGS2/NOS2/MAPK1/IL4/FASN/STAT3/ACACA/NOS3/PARP1/SCD	10
BP	GO:0002756	MyD88-independent toll-like receptor signaling pathway	4/187	34/18866	0.000343528	0.001264806	0.000574045	CD14/CASP8/IKBKB/CHUK	4
BP	GO:0033198	response to ATP	4/187	34/18866	0.000343528	0.001264806	0.000574045	PTGS2/SOD1/IL1B/TRP	4
BP	GO:0045648	positive regulation of erythrocyte differentiation	4/187	34/18866	0.000343528	0.001264806	0.000574045	STAT1/MAPK14/STAT3/HIF1A	4
BP	GO:0055094	response to lipoprotein particle	4/187	34/18866	0.000343528	0.001264806	0.000574045	PPARG/AKT1/CES1/LPL	4
BP	GO:0010574	regulation of vascular endothelial growth factor production	5/187	61/18866	0.000344171	0.001265006	0.000574136	PTGS2/CYP1B1/HIF1A/IL1B/IL1A	5
BP	GO:0046324	regulation of glucose import	5/187	61/18866	0.000344171	0.001265006	0.000574136	AKT1/MAPK14/NFE2L2/HK2/INS	5
BP	GO:0019693	ribose phosphate metabolic process	13/187	427/18866	0.000347751	0.001277071	0.000579611	IFNG/IL4/FASN/BAD/HMGCR/PPARA/STAT3/HIF1A/ACACA/PARP1/HK2/INS/SCD	13
BP	GO:0033673	negative regulation of kinase activity	10/187	268/18866	0.000353095	0.001295588	0.000588015	CASP3/AKT1/GSTP1/CDKN1A/RB1/IFNG/HMGCR/CAV1/IL1B/HSPB1	10
BP	GO:0090596	sensory organ morphogenesis	10/187	269/18866	0.000363629	0.001331963	0.000604525	BCL2/BAX/VEGFA/MAPK1/MAPK3/SOD1/STAT3/HIF1A/EDN1/BDNF	10

BP	GO:1901293	nucleoside phosphate biosynthetic process	10/187	269/18866	0.000363629	0.001331963	0.000604525	PTGS2/NOS2/MAPK1/IL4/FASN/STAT3/ACACA/NOS3/PARP1/SCD	10
BP	GO:0007589	body fluid secretion	6/187	95/18866	0.000366311	0.001339501	0.000607946	VEGFA/CCND1/HIF1A/CAV1/HK2/EDN1	6
BP	GO:0050709	negative regulation of protein secretion	6/187	95/18866	0.000366311	0.001339501	0.000607946	OPRM1/ADRA2A/HMGCR/IL1B/DRD2/INS	6
BP	GO:0007405	neuroblast proliferation	5/187	62/18866	0.000371385	0.001354592	0.000614795	VEGFA/TP53/HIF1A/DRD2/BDNF	5
BP	GO:0030888	regulation of B cell proliferation	5/187	62/18866	0.000371385	0.001354592	0.000614795	BCL2/CASP3/AHR/CDKN1A/IL4	5
BP	GO:1904589	regulation of protein import	5/187	62/18866	0.000371385	0.001354592	0.000614795	PTGS2/CDK1/MAPK14/MAPK1/IFNG	5
BP	GO:0071333	cellular response to glucose stimulus	7/187	134/18866	0.00038251	0.001393986	0.000632674	ICAM1/ADRA2A/BAD/HMGCR/RAF1/HIF1A/GC	7
BP	GO:0034765	regulation of ion transmembrane transport	14/187	489/18866	0.000383998	0.001398221	0.000634597	OPRM1/BAX/AKT1/ADRA2A/MMP9/IFNG/HTR3A/CAV1/CCL2/CXCL11/CXCL10/RASGRF1/DRD2/CACNA1S	14
BP	GO:1900077	negative regulation of cellular response to insulin stimulus	4/187	35/18866	0.00038487	0.001400208	0.000635498	RELA/IL1B/PRKCB/LPL	4
BP	GO:0030258	lipid modification	10/187	271/18866	0.000385486	0.001401262	0.000635977	PPARG/AKT1/CYP3A4/CYP1A1/ALOX5/MAPK14/PPARD/PPARA/SOAT1/FABP1	10
BP	GO:0035635	entry of bacterium into host cell	3/187	15/18866	0.000399388	0.001440819	0.00065393	MET/CAV1/CXCL8	3
BP	GO:0042362	fat-soluble vitamin biosynthetic process	3/187	15/18866	0.000399388	0.001440819	0.00065393	CYP3A4/IFNG/IL1B	3
BP	GO:0042574	retinal metabolic process	3/187	15/18866	0.000399388	0.001440819	0.00065393	CYP1B1/AKR1C3/AKR1C1	3
BP	GO:0045651	positive regulation of macrophage differentiation	3/187	15/18866	0.000399388	0.001440819	0.00065393	CASP8/PRKCA/RB1	3
BP	GO:0048308	organelle inheritance	3/187	15/18866	0.000399388	0.001440819	0.00065393	CDK1/MAPK1/MAPK3	3
BP	GO:0048313	Golgi inheritance	3/187	15/18866	0.000399388	0.001440819	0.00065393	CDK1/MAPK1/MAPK3	3
BP	GO:0051044	positive regulation of membrane protein ectodomain proteolysis	3/187	15/18866	0.000399388	0.001440819	0.00065393	ADRA2A/IFNG/IL1B	3
BP	GO:1901550	regulation of endothelial cell development	3/187	15/18866	0.000399388	0.001440819	0.00065393	IKBKB/VEGFA/IL1B	3
BP	GO:1903140	regulation of establishment of endothelial barrier	3/187	15/18866	0.000399388	0.001440819	0.00065393	IKBKB/VEGFA/IL1B	3

BP	GO:0010830	regulation of myotube differentiation	5/187	63/18866	0.000400194	0.001442511	0.000654698	BCL2/MAPK14/IL4/CXCL10/BDNF	5
BP	GO:0044839	cell cycle G2/M phase transition	10/187	273/18866	0.000408426	0.001470952	0.000667606	CDK1/CDK2/CHEK1/CCNA2/CCND1/CDKN1A/CDK4/TP53/CCNB1/CHE	10
BP	GO:0032651	regulation of interleukin-1 beta production	6/187	97/18866	0.000409756	0.001474503	0.000669218	CASP8/GSTP1/IFNG/STAT3/HSPB1/LPL	6
BP	GO:0009135	purine nucleoside diphosphate metabolic process	7/187	136/18866	0.000418337	0.001501606	0.000681519	IFNG/BAD/PPARA/STAT3/HIF1A/HK2/INS	7
BP	GO:0009179	purine ribonucleoside diphosphate metabolic process	7/187	136/18866	0.000418337	0.001501606	0.000681519	IFNG/BAD/PPARA/STAT3/HIF1A/HK2/INS	7
BP	GO:0071331	cellular response to hexose stimulus	7/187	136/18866	0.000418337	0.001501606	0.000681519	ICAM1/ADRA2A/BAD/HMGCR/RAF1/HIF1A/GC	7
BP	GO:0033280	response to vitamin D	4/187	36/18866	0.000429648	0.001535786	0.000697032	PTGS2/TYR/CXCL10/SPP1	4
BP	GO:0070873	regulation of glycogen metabolic process	4/187	36/18866	0.000429648	0.001535786	0.000697032	AKT1/GSK3B/IGF2/INS	4
BP	GO:0071402	cellular response to lipoprotein particle stimulus	4/187	36/18866	0.000429648	0.001535786	0.000697032	PPARG/AKT1/CES1/LPL	4
BP	GO:1901661	quinone metabolic process	4/187	36/18866	0.000429648	0.001535786	0.000697032	AKR1C3/AKR1B1/HMGCR/AKR1C1	4
BP	GO:2000310	regulation of NMDA receptor activity	4/187	36/18866	0.000429648	0.001535786	0.000697032	OPRM1/IFNG/CCL2/RASGRF1	4
BP	GO:0032507	maintenance of protein location in cell	5/187	64/18866	0.000430658	0.001536836	0.000697508	AKT1/HSPA5/CAV1/GJA1/HK2	5
BP	GO:0070265	necrotic cell death	5/187	64/18866	0.000430658	0.001536836	0.000697508	CD14/BAX/CASP8/TP53/CAV1	5
BP	GO:0030183	B cell differentiation	7/187	137/18866	0.000437229	0.001556406	0.00070639	BCL2/BAX/VCAM1/TP53/IL4/CD40LG/BAD	7
BP	GO:0071326	cellular response to monosaccharide stimulus	7/187	137/18866	0.000437229	0.001556406	0.00070639	ICAM1/ADRA2A/BAD/HMGCR/RAF1/HIF1A/GC	7
BP	GO:1903038	negative regulation of leukocyte cell-cell adhesion	7/187	137/18866	0.000437229	0.001556406	0.00070639	CASP3/AKT1/ERBB2/IL2RA/IL4/PPARA/IRF1	7
BP	GO:1901796	regulation of signal transduction by p53 class mediator	8/187	180/18866	0.000439522	0.001563272	0.000709507	BCL2/AKT1/MAPK14/CDK2/CHEK1/TP53/MDM2/CHEK2	8
BP	GO:0055076	transition metal ion homeostasis	7/187	138/18866	0.000456797	0.001623188	0.0007367	HMOX1/IFNG/XIAP/SOD1/HIF1A/MYC/ABCG2	7
BP	GO:0062207	regulation of pattern recognition receptor signaling pathway	6/187	99/18866	0.000457124	0.001623188	0.0007367	CD14/LBP/ESR1/XIAP/CAV1/IRF1	6
BP	GO:0006940	regulation of smooth muscle contraction	5/187	65/18866	0.000462838	0.001640764	0.000744677	PTGS2/ADRA2A/SOD1/CAV1/EDN1	5

BP	GO:0007588	excretion	5/187	65/18866	0.000462838	0.001640764	0.000744677	HMOX1/ABCG2/DRD2/TRPV1/EDN1	5
BP	GO:2001252	positive regulation of chromosome organization	8/187	182/18866	0.000473011	0.001675441	0.000760416	VEGFA/MAPK1/RB1/TP53/CCNB1/MAPK3/IL1B/GCG	8
BP	GO:0009185	ribonucleoside diphosphate metabolic process	7/187	139/18866	0.000477058	0.001683471	0.00076406	IFNG/BAD/PPARA/STAT3/HIF1A/HK2/INS	7
BP	GO:0045730	respiratory burst	4/187	37/18866	0.000478023	0.001683471	0.00076406	LBP/NCF1/MPO/INS	4
BP	GO:0060969	negative regulation of gene silencing	4/187	37/18866	0.000478023	0.001683471	0.00076406	PPARG/ESR1/TP53/STAT3	4
BP	GO:0070423	nucleotide-binding oligomerization domain containing signaling	4/187	37/18866	0.000478023	0.001683471	0.00076406	CASP8/RELA/NFKBIA/XIAP	4
BP	GO:0090050	positive regulation of cell migration involved in sprouting angiogenesis	4/187	37/18866	0.000478023	0.001683471	0.00076406	PTGS2/HMOX1/VEGFA/KDR	4
BP	GO:0090322	regulation of superoxide metabolic process	4/187	37/18866	0.000478023	0.001683471	0.00076406	GSTP1/EGFR/SOD1/NFE2L2	4
BP	GO:1904994	regulation of leukocyte adhesion to vascular endothelial cell	4/187	37/18866	0.000478023	0.001683471	0.00076406	RELA/ICAM1/SELE/ALOX5	4
BP	GO:0048762	mesenchymal cell differentiation	9/187	229/18866	0.000478842	0.001684974	0.000764742	BCL2/STAT1/GSK3B/MAPK1/MAPK3/HIF1A/IL1B/COL1A1/EDN1	9
BP	GO:0002070	epithelial cell maturation	3/187	16/18866	0.000487978	0.001707328	0.000774888	AKR1B1/CDKN1A/HIF1	3
BP	GO:0002523	leukocyte migration involved in inflammatory response	3/187	16/18866	0.000487978	0.001707328	0.000774888	LBP/SELE/ALOX5	3
BP	GO:0019372	lipoxygenase pathway	3/187	16/18866	0.000487978	0.001707328	0.000774888	PTGS2/PON1/ALOX5	3
BP	GO:0032225	regulation of synaptic transmission, dopaminergic	3/187	16/18866	0.000487978	0.001707328	0.000774888	PTGS2/SLC6A4/DRD2	3
BP	GO:0045725	positive regulation of glycogen biosynthetic process	3/187	16/18866	0.000487978	0.001707328	0.000774888	AKT1/IGF2/INS	3
BP	GO:0071380	cellular response to prostaglandin E stimulus	3/187	16/18866	0.000487978	0.001707328	0.000774888	PPARG/AKT1/ACACA	3
BP	GO:0071391	cellular response to estrogen	3/187	16/18866	0.000487978	0.001707328	0.000774888	AR/ESR1/MDM2	3
BP	GO:0043010	camera-type eye development	11/187	332/18866	0.000493827	0.001726383	0.000783536	BAX/JUN/ACHE/CYP1A1/CYP1B1/RXRA/EGFR/VEGFA/CDK4/HIF1A/D	11
BP	GO:0046328	regulation of JNK cascade	8/187	184/18866	0.000508541	0.001773893	0.000805099	AKT1/GSTP1/EGFR/CD40LG/MMP8/NCF1/IL1B/EDN1	8
BP	GO:0002792	negative regulation of peptide secretion	6/187	101/18866	0.000508656	0.001773893	0.000805099	OPRM1/ADRA2A/HMGCR/IL1B/DRD2/INS	6
BP	GO:0042116	macrophage activation	6/187	101/18866	0.000508656	0.001773893	0.000805099	LBP/JUN/IFNG/IL4/MMP8/TRPV1	6

BP	GO:0010661	positive regulation of muscle cell apoptotic process	4/187	38/18866	0.000530154	0.001842874	0.000836407	PPARG/TP53/IFNG/HMGCR	4
BP	GO:0035872	nucleotide-binding domain, leucine rich repeat containing receptor signaling pathway	4/187	38/18866	0.000530154	0.001842874	0.000836407	CASP8/RELA/NFKBIA/XIAP	4
BP	GO:0044003	modulation by symbiont of host process	4/187	38/18866	0.000530154	0.001842874	0.000836407	CASP8/RXRA/BCL2L1/BAD	4
BP	GO:0060045	positive regulation of cardiac muscle cell proliferation	4/187	38/18866	0.000530154	0.001842874	0.000836407	CDK1/MAPK14/MAPK1/CCNB1	4
BP	GO:1903076	regulation of protein localization to plasma membrane	6/187	102/18866	0.000536062	0.001860398	0.00084436	AR/AKT1/EGFR/BCL2L1/IFNG/INS	6
BP	GO:2000060	positive regulation of ubiquitin-dependent protein catabolic process	6/187	102/18866	0.000536062	0.001860398	0.00084436	AKT1/GSK3B/MDM2/EGF/CAV1/NFE2L2	6
BP	GO:0045333	cellular respiration	8/187	187/18866	0.000565866	0.001962247	0.000890585	NOS2/CDK1/CYP1A2/CAT/CCNB1/IFNG/IL4/HIF1A	8
BP	GO:0016239	positive regulation of macroautophagy	5/187	68/18866	0.000570301	0.001972846	0.000895396	HMOX1/IL4/KDR/MAPK3/HIF1A	5
BP	GO:0042446	hormone biosynthetic process	5/187	68/18866	0.000570301	0.001972846	0.000895396	AKR1B1/CYP19A1/HIF1A/DUOX2/ADM	5
BP	GO:0050766	positive regulation of phagocytosis	5/187	68/18866	0.000570301	0.001972846	0.000895396	PPARG/IFNG/SOD1/IL1B/CCL2	5
BP	GO:0001662	behavioral fear response	4/187	39/18866	0.000586203	0.002001027	0.000908186	BCL2/DPP4/GJA1/BDNF	4
BP	GO:0006778	porphyrin-containing compound metabolic process	4/187	39/18866	0.000586203	0.002001027	0.000908186	HMOX1/CYP1A2/CYP1A1/UGT1A1	4
BP	GO:0046326	positive regulation of glucose import	4/187	39/18866	0.000586203	0.002001027	0.000908186	AKT1/MAPK14/NFE2L2/INS	4
BP	GO:1904706	negative regulation of vascular associated smooth muscle cell proliferation	4/187	39/18866	0.000586203	0.002001027	0.000908186	PPARG/HMOX1/GSTP1/CDKN1A	4
BP	GO:2000279	negative regulation of DNA biosynthetic process	4/187	39/18866	0.000586203	0.002001027	0.000908186	PPARG/CDKN1A/TP53/GJA1	4
BP	GO:0006978	DNA damage response, signal transduction by p53 class mediator resulting in transcription of p21 class mediator	3/187	17/18866	0.000588236	0.002001027	0.000908186	CDKN1A/TP53/CHEK2	3
BP	GO:0009299	mRNA transcription	3/187	17/18866	0.000588236	0.002001027	0.000908186	TP53/PPARD/STAT3	3
BP	GO:0010224	response to UV-B	3/187	17/18866	0.000588236	0.002001027	0.000908186	BCL2/RELA/CDKN1A	3
BP	GO:0015732	prostaglandin transport	3/187	17/18866	0.000588236	0.002001027	0.000908186	NOS2/IL1B/EDN1	3
BP	GO:0030540	female genitalia development	3/187	17/18866	0.000588236	0.002001027	0.000908186	BAX/ESR1/CYP19A1	3

BP	GO:0030949	positive regulation of vascular endothelial growth factor receptor signaling pathway	3/187	17/18866	0.000588236	0.002001027	0.000908186	HIF1A/IL1B/PRKCB	3
BP	GO:0032966	negative regulation of collagen biosynthetic process	3/187	17/18866	0.000588236	0.002001027	0.000908186	PPARG/PPARD/GOT1	3
BP	GO:0042448	progesterone metabolic process	3/187	17/18866	0.000588236	0.002001027	0.000908186	AKR1C3/AKR1C1/ADM	3
BP	GO:0042953	lipoprotein transport	3/187	17/18866	0.000588236	0.002001027	0.000908186	PPARG/APOB/PRKCB	3
BP	GO:0055012	ventricular cardiac muscle cell differentiation	3/187	17/18866	0.000588236	0.002001027	0.000908186	CDK1/RXRA/CCNB1	3
BP	GO:0070242	thymocyte apoptotic process	3/187	17/18866	0.000588236	0.002001027	0.000908186	BAX/TP53/HIF1A	3
BP	GO:0070875	positive regulation of glycogen metabolic process	3/187	17/18866	0.000588236	0.002001027	0.000908186	AKT1/IGF2/INS	3
BP	GO:0090336	positive regulation of brown fat cell differentiation	3/187	17/18866	0.000588236	0.002001027	0.000908186	PTGS2/MAPK14/INS	3
BP	GO:1903209	positive regulation of oxidative stress-induced cell death	3/187	17/18866	0.000588236	0.002001027	0.000908186	MCL1/SOD1/MMP3	3
BP	GO:2000811	negative regulation of anoikis	3/187	17/18866	0.000588236	0.002001027	0.000908186	BCL2/MCL1/CAV1	3
BP	GO:2001267	regulation of cysteine-type endopeptidase activity involved in apoptotic signaling pathway	3/187	17/18866	0.000588236	0.002001027	0.000908186	BAX/CASP8/MMP9	3
BP	GO:0044282	small molecule catabolic process	13/187	452/18866	0.000594129	0.002019474	0.000916558	PON1/NOS2/AKT1/CYP1A1/AKR1C3/TP53/PPARD/BAD/PPARA/GOT1/NOS3/HK2/FABP1	13
BP	GO:0021700	developmental maturation	10/187	287/18866	0.000603003	0.002048016	0.000929512	BCL2/PPARG/AKR1B1/VEGFA/CDKN1A/MMP2/RB1/CCNB1/HIF1A/RU	10
BP	GO:0007612	learning	7/187	145/18866	0.000614178	0.00208432	0.000945989	PTGS2/JUN/HMGCR/FOS/HIF1A/DRD2/HRH1	7
BP	GO:0045621	positive regulation of lymphocyte differentiation	6/187	105/18866	0.000625213	0.002118422	0.000961467	IL2RA/IFNG/IL4/BAD/IL1B/IL1A	6
BP	GO:0048259	regulation of receptor-mediated endocytosis	6/187	105/18866	0.000625213	0.002118422	0.000961467	SELE/VEGFA/IL4/EGF/SERPINE1/DRD2	6
BP	GO:0001990	regulation of systemic arterial blood pressure by hormone	4/187	40/18866	0.000646333	0.00218309	0.000990817	CES1/NOS3/EDN1/ENPEP	4
BP	GO:0002209	behavioral defense response	4/187	40/18866	0.000646333	0.00218309	0.000990817	BCL2/DPP4/GJA1/BDNF	4
BP	GO:0008207	C21-steroid hormone metabolic process	4/187	40/18866	0.000646333	0.00218309	0.000990817	AKR1C3/AKR1B1/AKR1C1/ADM	4
BP	GO:0045746	negative regulation of Notch signaling pathway	4/187	40/18866	0.000646333	0.00218309	0.000990817	AKT1/EGFR/NFKBIA/EGF	4

BP	GO:0022408	negative regulation of cell-cell adhesion	8/187	191/18866	0.000650322	0.002193111	0.000995366	CASP3/AKT1/VEGFA/ERBB2/IL2RA/IL4/PPARA/IRF1	8
BP	GO:0048167	regulation of synaptic plasticity	8/187	191/18866	0.000650322	0.002193111	0.000995366	PTGS2/GSK3B/MAPK1/RASGRF1/DRD2/BDNF/INS/HRH1	8
BP	GO:0032374	regulation of cholesterol transport	5/187	70/18866	0.00065168	0.002195968	0.000996662	PON1/NFKBIA/CES1/EGF/CAV1	5
BP	GO:0019395	fatty acid oxidation	6/187	106/18866	0.000657351	0.002211606	0.00100376	PPARG/AKT1/MAPK14/PPARD/PPARA/FABP1	6
BP	GO:1905269	positive regulation of chromatin organization	6/187	106/18866	0.000657351	0.002211606	0.00100376	VEGFA/TP53/CCNB1/MAPK3/IL1B/GCG	6
BP	GO:0044409	entry into host	7/187	147/18866	0.000666256	0.002239811	0.001016561	DPP4/CDK1/ICAM1/EGFR/MET/CAV1/CXCL8	7
BP	GO:0044272	sulfur compound biosynthetic process	8/187	192/18866	0.00067295	0.002260544	0.00102597	GSTP1/GSTM1/FASN/ACACA/NFE2L2/GSTA1/GSTA2/SCD	8
BP	GO:0038093	Fc receptor signaling pathway	9/187	241/18866	0.000690873	0.002318933	0.001052471	JUN/RELA/IKBKB/MAPK8/MAPK1/MAPK10/MAPK3/FOS/CHUK	9
BP	GO:0008277	regulation of G protein-coupled receptor signaling pathway	7/187	148/18866	0.000693577	0.002326192	0.001055766	OPRM1/PRKCA/MET/CXCL8/DRD2/EDN1/ADM	7
BP	GO:0033627	cell adhesion mediated by integrin	5/187	71/18866	0.000695486	0.002328956	0.00105702	DPP4/ICAM1/CYP1B1/PLAU/SERPINE1	5
BP	GO:0042982	amyloid precursor protein metabolic process	5/187	71/18866	0.000695486	0.002328956	0.00105702	CASP3/ACHE/RELA/IFNG/SOAT1	5
BP	GO:0010713	negative regulation of collagen metabolic process	3/187	18/18866	0.000700752	0.002337472	0.001060885	PPARG/PPARD/GOT1	3
BP	GO:0042772	DNA damage response, signal transduction resulting in	3/187	18/18866	0.000700752	0.002337472	0.001060885	CDKN1A/TP53/CHEK2	3
BP	GO:0044872	lipoprotein localization	3/187	18/18866	0.000700752	0.002337472	0.001060885	PPARG/APOB/PRKCB	3
BP	GO:0071318	cellular response to ATP	3/187	18/18866	0.000700752	0.002337472	0.001060885	PTGS2/SOD1/TRPV1	3
BP	GO:1900221	regulation of amyloid-beta clearance	3/187	18/18866	0.000700752	0.002337472	0.001060885	IFNG/IL4/HMGCR	3
BP	GO:0042596	fear response	4/187	41/18866	0.000710707	0.002365165	0.001073454	BCL2/DPP4/GJA1/BDNF	4
BP	GO:0046825	regulation of protein export from nucleus	4/187	41/18866	0.000710707	0.002365165	0.001073454	GSK3B/TP53/MDM2/IL1B	4
BP	GO:1900117	regulation of execution phase of apoptosis	4/187	41/18866	0.000710707	0.002365165	0.001073454	BAX/BCL2L1/TP53/GCG	4
BP	GO:0008593	regulation of Notch signaling pathway	6/187	108/18866	0.000725458	0.002412386	0.001094885	AKT1/EGFR/NFKBIA/STAT3/EGF/NOS3	6
BP	GO:0050663	cytokine secretion	5/187	72/18866	0.000741457	0.002463682	0.001118167	CD14/IFNG/IL1A/DRD2/INS	5

BP	GO:1904062	regulation of cation transmembrane transport	11/187	349/18866	0.00074617	0.002477424	0.001124404	OPRM1/BAX/ADRA2A/MMP9/IFNG/CAV1/CCL2/CXCL11/CXCL10/RASGRF1/DRD2	11
BP	GO:0072595	maintenance of protein localization in organelle	4/187	42/18866	0.000779487	0.00258205	0.001171889	AKT1/HSPA5/GJA1/HK2	4
BP	GO:1902622	regulation of neutrophil migration	4/187	42/18866	0.000779487	0.00258205	0.001171889	LBP/DPP4/CXCL8/EDN1	4
BP	GO:1904037	positive regulation of epithelial cell apoptotic process	4/187	42/18866	0.000779487	0.00258205	0.001171889	AKR1C3/CD40LG/BAD/CCL2	4
BP	GO:0070227	lymphocyte apoptotic process	5/187	73/18866	0.000789662	0.002613738	0.001186271	BAX/AKT1/TP53/IL2RA/HIF1A	5
BP	GO:0006164	purine nucleotide biosynthetic process	8/187	197/18866	0.00079582	0.002630064	0.001193681	NOS2/IL4/FASN/STAT3/ACACA/NOS3/PARP1/S	8
BP	GO:0051216	cartilage development	8/187	197/18866	0.00079582	0.002630064	0.001193681	RELA/MAPK14/MMP13/MAPK3/HIF1A/COL1A1/RUNX2/EDN1	8
BP	GO:0010759	positive regulation of macrophage chemotaxis	3/187	19/18866	0.000826096	0.002719656	0.001234343	MAPK14/MAPK1/MAPK3	3
BP	GO:0019054	modulation by virus of host cellular process	3/187	19/18866	0.000826096	0.002719656	0.001234343	CASP8/BCL2L1/BAD	3
BP	GO:0032026	response to magnesium ion	3/187	19/18866	0.000826096	0.002719656	0.001234343	CD14/CCND1/MDM2	3
BP	GO:0032930	positive regulation of superoxide anion generation	3/187	19/18866	0.000826096	0.002719656	0.001234343	GSTP1/EGFR/SOD1	3
BP	GO:0048245	eosinophil chemotaxis	3/187	19/18866	0.000826096	0.002719656	0.001234343	IL4/CCL2/HRH1	3
BP	GO:0032652	regulation of interleukin-1 production	6/187	111/18866	0.000837706	0.002755763	0.001250731	CASP8/GSTP1/IFNG/STAT3/HSPB1/LPL	6
BP	GO:0032024	positive regulation of insulin secretion	5/187	74/18866	0.000840167	0.00275752	0.001251528	PPARD/BAD/HIF1A/GJA1/GCG	5
BP	GO:0032481	positive regulation of type I interferon production	5/187	74/18866	0.000840167	0.00275752	0.001251528	CD14/RELA/STAT1/CHUK/IRF1	5
BP	GO:0034121	regulation of toll-like receptor signaling pathway	5/187	74/18866	0.000840167	0.00275752	0.001251528	CD14/LBP/ESR1/CAV1/IRF1	5
BP	GO:0045124	regulation of bone resorption	4/187	43/18866	0.000852838	0.002792703	0.001267496	PRKCA/EGFR/CA2/SPP1	4
BP	GO:0046717	acid secretion	4/187	43/18866	0.000852838	0.002792703	0.001267496	CES1/PTGER3/DRD2/TRPV1	4
BP	GO:2000648	positive regulation of stem cell proliferation	4/187	43/18866	0.000852838	0.002792703	0.001267496	VEGFA/HIF1A/GJA1/DRD2	4
BP	GO:0061351	neural precursor cell proliferation	7/187	154/18866	0.000876705	0.002868376	0.001301841	SLC6A4/VEGFA/TP53/EGF/HIF1A/DRD2/BDNF	7
BP	GO:0016052	carbohydrate catabolic process	8/187	200/18866	0.000877801	0.002868376	0.001301841	TP53/IFNG/BAD/PPARA/STAT3/HIF1A/HK2/INS	8

BP	GO:0099565	chemical synaptic transmission, postsynaptic	6/187	112/18866	0.000877952	0.002868376	0.001301841	OPRM1/AKT1/GSK3B/DRD2/TRPV1/BDNF	6
BP	GO:0015909	long-chain fatty acid transport	5/187	75/18866	0.000893041	0.00291324	0.001322203	PPARG/AKT1/ACACA/DRD2/FABP1	5
BP	GO:0046323	glucose import	5/187	75/18866	0.000893041	0.00291324	0.001322203	AKT1/MAPK14/NFE2L2/HK2/INS	5
BP	GO:0009132	nucleoside diphosphate metabolic process	7/187	155/18866	0.00091062	0.002968331	0.001347207	IFNG/BAD/PPARA/STAT3/HIF1A/HK2/INS	7
BP	GO:0035821	modulation of process of other organism	6/187	113/18866	0.000919671	0.002995561	0.001359565	JUN/CASP8/SLPI/RXRA/BCL2L1/BAD	6
BP	GO:1905521	regulation of macrophage migration	4/187	44/18866	0.000930924	0.003029914	0.001375157	MAPK14/MAPK1/MAPK3/CYP19A1	4
BP	GO:0007178	transmembrane receptor protein serine/threonine kinase signaling pathway	11/187	359/18866	0.000939248	0.003054689	0.001386401	JUN/MAPK14/TP53/XIAP/MAPK3/GOT1/FOS/HSPA5/CAV1/PARP1/RUN	11
BP	GO:0008306	associative learning	5/187	76/18866	0.000948352	0.003077306	0.001396666	HMGCR/FOS/HIF1A/DRD2/HRH1	5
BP	GO:0033555	multicellular organismal response to stress	5/187	76/18866	0.000948352	0.003077306	0.001396666	BCL2/DPP4/GJA1/TRPV1/BDNF	5
BP	GO:0071229	cellular response to acid chemical	5/187	76/18866	0.000948352	0.003077306	0.001396666	EGFR/VEGFA/BCL2L1/MMP2/COL1A1	5
BP	GO:0002689	negative regulation of leukocyte chemotaxis	3/187	20/18866	0.00096482	0.003104926	0.001409202	DPP4/CYP19A1/CCL2	3
BP	GO:0007252	I-kappaB phosphorylation	3/187	20/18866	0.00096482	0.003104926	0.001409202	IKBKB/AKT1/CHUK	3
BP	GO:0019373	epoxygenase P450 pathway	3/187	20/18866	0.00096482	0.003104926	0.001409202	CYP1A2/CYP1A1/CYP1B1	3
BP	GO:0031998	regulation of fatty acid beta-	3/187	20/18866	0.00096482	0.003104926	0.001409202	AKT1/PPARA/FABP1	3
BP	GO:0032495	response to muramyl dipeptide	3/187	20/18866	0.00096482	0.003104926	0.001409202	RELA/MAPK14/NFKBIA	3
BP	GO:0051412	response to corticosterone	3/187	20/18866	0.00096482	0.003104926	0.001409202	CCND1/CDKN1A/FOS	3
BP	GO:0060353	regulation of cell adhesion molecule production	3/187	20/18866	0.00096482	0.003104926	0.001409202	CAV1/IL1B/CXCL8	3
BP	GO:1900409	positive regulation of cellular response to oxidative stress	3/187	20/18866	0.00096482	0.003104926	0.001409202	MCL1/SOD1/MMP3	3
BP	GO:1902004	positive regulation of amyloid-beta formation	3/187	20/18866	0.00096482	0.003104926	0.001409202	CASP3/RELA/IFNG	3
BP	GO:1902644	tertiary alcohol metabolic process	3/187	20/18866	0.00096482	0.003104926	0.001409202	AKR1C3/AKR1B1/AKR1C1	3
BP	GO:2001169	regulation of ATP biosynthetic	3/187	20/18866	0.00096482	0.003104926	0.001409202	IL4/STAT3/PARP1	3
BP	GO:0006096	glycolytic process	6/187	115/18866	0.001007678	0.00324042	0.001470697	IFNG/PPARA/STAT3/HIF1A/HK2/INS	6
BP	GO:0010677	negative regulation of cellular carbohydrate metabolic process	4/187	45/18866	0.001013909	0.003241035	0.001470976	GSK3B/PPARA/STAT3/INS	4

BP	GO:0010828	positive regulation of glucose transmembrane transport	4/187	45/18866	0.001013909	0.003241035	0.001470976	AKT1/MAPK14/NFE2L2/INS	4
BP	GO:0031018	endocrine pancreas development	4/187	45/18866	0.001013909	0.003241035	0.001470976	IL6R/AKT1/GSK3B/BAD	4
BP	GO:0034198	cellular response to amino acid starvation	4/187	45/18866	0.001013909	0.003241035	0.001470976	MAPK8/CDKN1A/MAPK1/MAPK3	4
BP	GO:0042771	intrinsic apoptotic signaling pathway in response to DNA damage by p53 class mediator	4/187	45/18866	0.001013909	0.003241035	0.001470976	BCL2/CDKN1A/TP53/CHEK2	4
BP	GO:0060443	mammary gland morphogenesis	4/187	45/18866	0.001013909	0.003241035	0.001470976	BAX/AR/ESR1/CAV1	4
BP	GO:0070266	necroptotic process	4/187	45/18866	0.001013909	0.003241035	0.001470976	CD14/CASP8/TP53/CAV	4
BP	GO:0090311	regulation of protein deacetylation	4/187	45/18866	0.001013909	0.003241035	0.001470976	MAPK8/VEGFA/TP53/IFNG	4
BP	GO:0060402	calcium ion transport into cytosol	7/187	158/18866	0.001018611	0.003253644	0.001476699	BCL2/BAX/CAV1/CXCL11/CXCL10/DRD2/TRPV	7
BP	GO:0006757	ATP generation from ADP	6/187	116/18866	0.001054038	0.003359306	0.001524655	IFNG/PPARA/STAT3/HIF1A/HK2/INS	6
BP	GO:0030518	intracellular steroid hormone receptor signaling pathway	6/187	116/18866	0.001054038	0.003359306	0.001524655	NR3C2/AR/ESR1/ESR2/NR3C1/PARP1	6
BP	GO:0046916	cellular transition metal ion homeostasis	6/187	116/18866	0.001054038	0.003359306	0.001524655	HMOX1/IFNG/SOD1/HIF1A/MYC/ABCG2	6
BP	GO:0000271	polysaccharide biosynthetic process	5/187	78/18866	0.001066568	0.003396718	0.001541634	AKT1/GSK3B/EGF/IGF2/INS	5
BP	GO:0050866	negative regulation of cell activation	8/187	207/18866	0.001095609	0.00348662	0.001582437	CASP3/HMOX1/ERBB2/IL2RA/IL4/NOS3/THBD/IRF1	8
BP	GO:0005978	glycogen biosynthetic process	4/187	46/18866	0.001101956	0.003493869	0.001585727	AKT1/GSK3B/IGF2/INS	4
BP	GO:0009250	glucan biosynthetic process	4/187	46/18866	0.001101956	0.003493869	0.001585727	AKT1/GSK3B/IGF2/INS	4
BP	GO:0010863	positive regulation of phospholipase C activity	4/187	46/18866	0.001101956	0.003493869	0.001585727	SELE/ESR1/EGFR/BDNF	4
BP	GO:0032309	icosanoid secretion	4/187	46/18866	0.001101956	0.003493869	0.001585727	NOS2/IL1B/DRD2/EDN1	4
BP	GO:0043330	response to exogenous dsRNA	4/187	46/18866	0.001101956	0.003493869	0.001585727	MAPK1/NFKBIA/MAPK3/CAV1	4
BP	GO:0009110	vitamin biosynthetic process	3/187	21/18866	0.001117452	0.003511875	0.0015939	CYP3A4/IFNG/IL1B	3
BP	GO:0010893	positive regulation of steroid biosynthetic process	3/187	21/18866	0.001117452	0.003511875	0.0015939	IFNG/IL1B/ADM	3
BP	GO:0014821	phasic smooth muscle contraction	3/187	21/18866	0.001117452	0.003511875	0.0015939	PTGER3/DRD2/EDN1	3
BP	GO:0016137	glycoside metabolic process	3/187	21/18866	0.001117452	0.003511875	0.0015939	AKR1C3/AKR1B1/AKR1C1	3
BP	GO:0030728	ovulation	3/187	21/18866	0.001117452	0.003511875	0.0015939	TNFAIP6/PTGS2/NOS3	3
BP	GO:0030878	thyroid gland development	3/187	21/18866	0.001117452	0.003511875	0.0015939	MAPK1/MAPK3/RAF1	3
BP	GO:0043651	linoleic acid metabolic process	3/187	21/18866	0.001117452	0.003511875	0.0015939	ALOX5/GSTP1/GSTA1	3
BP	GO:0071498	cellular response to fluid shear stress	3/187	21/18866	0.001117452	0.003511875	0.0015939	PTGS2/CA2/NFE2L2	3

BP	GO:0072111	cell proliferation involved in kidney development	3/187	21/18866	0.001117452	0.003511875	0.0015939	IL6R/STAT1/MYC	3
BP	GO:0090312	positive regulation of protein deacetylation	3/187	21/18866	0.001117452	0.003511875	0.0015939	VEGFA/TP53/IFNG	3
BP	GO:1900543	negative regulation of purine nucleotide metabolic process	3/187	21/18866	0.001117452	0.003511875	0.0015939	PPARA/STAT3/PARP1	3
BP	GO:2000737	negative regulation of stem cell differentiation	3/187	21/18866	0.001117452	0.003511875	0.0015939	STAT3/NFE2L2/CDK12	3
BP	GO:0072522	purine-containing compound biosynthetic process	8/187	208/18866	0.001129968	0.003548611	0.001610573	NOS2/IL4/FASN/STAT3/ACACA/NOS3/PARP1/S	8
BP	GO:0050769	positive regulation of neurogenesis	13/187	485/18866	0.001132729	0.003554682	0.001613328	OPRM1/BCL2/PPARG/RELA/VEGFA/IFNG/HIF1A/HSPA5/IL1B/NFE2L2/E2F1/DRD2/BDNF	13
BP	GO:0043312	neutrophil degranulation	13/187	487/18866	0.001175476	0.003686136	0.00167299	TNFAIP6/CD14/ALOX5/GSTP1/SLPI/CAT/PLAU/MAPK14/MMP9/MAPK1/MMP8/MPO/CTSD	13
BP	GO:0008542	visual learning	4/187	47/18866	0.001195231	0.00373218	0.001693887	HMGCR/HIF1A/DRD2/HRH1	4
BP	GO:0014075	response to amine	4/187	47/18866	0.001195231	0.00373218	0.001693887	CDK1/ICAM1/SOD1/DRD2	4
BP	GO:0022602	ovulation cycle process	4/187	47/18866	0.001195231	0.00373218	0.001693887	CASP3/ESR1/HSPA5/NOS3	4
BP	GO:0035722	interleukin-12-mediated signaling pathway	4/187	47/18866	0.001195231	0.00373218	0.001693887	IFNG/SOD1/GSTA2/SERPINB2	4
BP	GO:0048806	genitalia development	4/187	47/18866	0.001195231	0.00373218	0.001693887	BAX/AR/ESR1/CYP19A1	4
BP	GO:0006110	regulation of glycolytic process	5/187	80/18866	0.001195376	0.00373218	0.001693887	IFNG/PPARA/STAT3/HIF1A/INS	5
BP	GO:0006790	sulfur compound metabolic process	11/187	371/18866	0.001223821	0.003818215	0.001732935	GSTP1/GSTM1/DPEP1/FASN/SOD1/GSR/ACACA/NFE2L2/GSTA1/GSTA2/SCD	11
BP	GO:0002283	neutrophil activation involved in immune response	13/187	490/18866	0.001242121	0.003872494	0.00175757	TNFAIP6/CD14/ALOX5/GSTP1/SLPI/CAT/PLAU/MAPK14/MMP9/MAPK1/MMP8/MPO/CTSD	13
BP	GO:0030278	regulation of ossification	8/187	212/18866	0.001276109	0.003975571	0.001804353	IL6R/BCL2/ALOX5/MAPK14/MAPK1/MAPK3/HIF1A/RUNX2	8
BP	GO:0032928	regulation of superoxide anion generation	3/187	22/18866	0.001284504	0.003984374	0.001808348	GSTP1/EGFR/SOD1	3

BP	GO:0045980	negative regulation of nucleotide metabolic process	3/187	22/18866	0.001284504	0.003984374	0.001808348	PPARA/STAT3/PARP1	3
BP	GO:0051000	positive regulation of nitric-oxide synthase activity	3/187	22/18866	0.001284504	0.003984374	0.001808348	AKT1/HIF1A/INS	3
BP	GO:0060352	cell adhesion molecule production	3/187	22/18866	0.001284504	0.003984374	0.001808348	CAV1/IL1B/CXCL8	3
BP	GO:1901739	regulation of myoblast fusion	3/187	22/18866	0.001284504	0.003984374	0.001808348	MAPK14/IL4/CXCL10	3
BP	GO:1902884	positive regulation of response to oxidative stress	3/187	22/18866	0.001284504	0.003984374	0.001808348	MCL1/SOD1/MMP3	3
BP	GO:0031952	regulation of protein autophosphorylation	4/187	48/18866	0.001293896	0.004001943	0.001816322	JUN/VEGFA/CAV1/INS	4
BP	GO:0033628	regulation of cell adhesion mediated by integrin	4/187	48/18866	0.001293896	0.004001943	0.001816322	DPP4/CYP1B1/PLAU/SERPINE1	4
BP	GO:1900274	regulation of phospholipase C	4/187	48/18866	0.001293896	0.004001943	0.001816322	SELE/ESR1/EGFR/BDNF	4
BP	GO:1990928	response to amino acid starvation	4/187	48/18866	0.001293896	0.004001943	0.001816322	MAPK8/CDKN1A/MAPK1/MAPK3	4
BP	GO:0007613	memory	6/187	121/18866	0.001310902	0.004051623	0.00183887	PTGS2/SLC6A4/RASGRF1/DRD2/BDNF/HRH1	6
BP	GO:0032412	regulation of ion transmembrane transporter activity	9/187	265/18866	0.001345132	0.004154426	0.001885528	OPRM1/ADRA2A/MMP9/IFNG/HTR3A/CAV1/CCL2/RASGRF1/DRD2	9
BP	GO:0034329	cell junction assembly	12/187	434/18866	0.00134623	0.004154829	0.001885711	BCL2/PRKCA/ACHE/IKBKB/VEGFA/KDR/CAV1/GJA1/IL1B/CLDN4/DRD2/BDNF	12
BP	GO:0021782	glial cell development	6/187	122/18866	0.001367555	0.004214584	0.001912831	AKT1/GSTP1/EGFR/IFNG/SOD1/IL1B	6
BP	GO:0051928	positive regulation of calcium ion transport	6/187	122/18866	0.001367555	0.004214584	0.001912831	BAX/CAV1/CCL2/CXCL11/CXCL10/GCG	6
BP	GO:0002067	glandular epithelial cell	4/187	49/18866	0.001398116	0.004287223	0.001945799	RXRA/GSK3B/FASN/BA	4
BP	GO:0003254	regulation of membrane	4/187	49/18866	0.001398116	0.004287223	0.001945799	BCL2/KDR/GOT1/PARP	4
BP	GO:0030850	prostate gland development	4/187	49/18866	0.001398116	0.004287223	0.001945799	AR/ESR1/RXRA/CYP19A1	4
BP	GO:0035196	production of miRNAs involved in gene silencing by miRNA	4/187	49/18866	0.001398116	0.004287223	0.001945799	ESR1/EGFR/TP53/STAT3	4
BP	GO:0042149	cellular response to glucose starvation	4/187	49/18866	0.001398116	0.004287223	0.001945799	BCL2/TP53/HSPA5/NFE2L2	4
BP	GO:0060711	labyrinthine layer development	4/187	49/18866	0.001398116	0.004287223	0.001945799	CASP8/AKT1/MAPK1/ADM	4
BP	GO:0071349	cellular response to interleukin-12	4/187	49/18866	0.001398116	0.004287223	0.001945799	IFNG/SOD1/GSTA2/SERPINB2	4
BP	GO:0055013	cardiac muscle cell development	5/187	83/18866	0.001409698	0.004319653	0.001960518	CDK1/VEGFA/CCNB1/PPARA/EDN1	5

BP	GO:0007173	epidermal growth factor receptor signaling pathway	6/187	123/18866	0.001426059	0.004363558	0.001980444	AKT1/ADRA2A/EGFR/MMP9/NCF1/EGF	6
BP	GO:1904375	regulation of protein localization to cell periphery	6/187	123/18866	0.001426059	0.004363558	0.001980444	AR/AKT1/EGFR/BCL2L1/IFNG/INS	6
BP	GO:0002052	positive regulation of neuroblast proliferation	3/187	23/18866	0.001466466	0.004461757	0.002025013	VEGFA/HIF1A/DRD2	3
BP	GO:0010288	response to lead ion	3/187	23/18866	0.001466466	0.004461757	0.002025013	PTGS2/CAT/CDK4	3
BP	GO:0035162	embryonic hemopoiesis	3/187	23/18866	0.001466466	0.004461757	0.002025013	VEGFA/KDR/HIF1A	3
BP	GO:0045649	regulation of macrophage differentiation	3/187	23/18866	0.001466466	0.004461757	0.002025013	CASP8/PRKCA/RB1	3
BP	GO:0051043	regulation of membrane protein ectodomain proteolysis	3/187	23/18866	0.001466466	0.004461757	0.002025013	ADRA2A/IFNG/IL1B	3
BP	GO:0071404	cellular response to low-density lipoprotein particle stimulus	3/187	23/18866	0.001466466	0.004461757	0.002025013	PPARG/CES1/LPL	3
BP	GO:0072677	eosinophil migration	3/187	23/18866	0.001466466	0.004461757	0.002025013	IL4/CCL2/HRH1	3
BP	GO:2000178	negative regulation of neural precursor cell proliferation	3/187	23/18866	0.001466466	0.004461757	0.002025013	SLC6A4/TP53/BDNF	3
BP	GO:1902749	regulation of cell cycle G2/M phase transition	8/187	217/18866	0.001479551	0.004498379	0.002041634	CDK1/CDK2/CHEK1/CCND1/CDKN1A/CDK4/TP53/CCNB1	8
BP	GO:0014031	mesenchymal cell development	5/187	84/18866	0.001487057	0.004518002	0.00205054	BCL2/MAPK1/MAPK3/HIF1A/EDN1	5
BP	GO:0052126	movement in host environment	7/187	169/18866	0.001503395	0.004562418	0.002070699	DPP4/CDK1/ICAM1/EGFR/MET/CAV1/CXCL8	7
BP	GO:0045646	regulation of erythrocyte differentiation	4/187	50/18866	0.001508053	0.004562418	0.002070699	STAT1/MAPK14/STAT3/HIF1A	4
BP	GO:0046850	regulation of bone remodeling	4/187	50/18866	0.001508053	0.004562418	0.002070699	PRKCA/EGFR/CA2/SPP1	4
BP	GO:0070671	response to interleukin-12	4/187	50/18866	0.001508053	0.004562418	0.002070699	IFNG/SOD1/GSTA2/SERPINB2	4
BP	GO:0101023	vascular endothelial cell proliferation	4/187	50/18866	0.001508053	0.004562418	0.002070699	PPARG/STAT3/CCL2/IGF2	4
BP	GO:1905562	regulation of vascular endothelial cell proliferation	4/187	50/18866	0.001508053	0.004562418	0.002070699	PPARG/STAT3/CCL2/IGF2	4
BP	GO:0001952	regulation of cell-matrix adhesion	6/187	125/18866	0.001548776	0.004679026	0.002123623	BCL2/PLAU/GSK3B/VEGFA/KDR/SERPINE1	6
BP	GO:0030968	endoplasmic reticulum unfolded protein response	6/187	125/18866	0.001548776	0.004679026	0.002123623	BAX/CCND1/HSPA5/CCL2/CXCL8/NFE2L2	6
BP	GO:0032874	positive regulation of stress-activated MAPK cascade	7/187	170/18866	0.001555089	0.004694793	0.002130779	VEGFA/CD40LG/MMP8/NCF1/HMGCR/IL1B/ED	7
BP	GO:0032092	positive regulation of protein binding	5/187	85/18866	0.001567497	0.004722292	0.00214326	GSK3B/MMP9/CAV1/BDNF/GCG	5

BP	GO:0070509	calcium ion import	5/187	85/18866	0.001567497	0.004722292	0.00214326	EGF/CCL2/TRPV1/GCG/CACNA1S	5
BP	GO:0110110	positive regulation of animal organ morphogenesis	5/187	85/18866	0.001567497	0.004722292	0.00214326	BAX/AR/VEGFA/MYC/EDN1	5
BP	GO:0055007	cardiac muscle cell differentiation	6/187	126/18866	0.00161307	0.004856176	0.002204024	CDK1/RXRA/VEGFA/CCNB1/PPARA/EDN1	6
BP	GO:0071827	plasma lipoprotein particle organization	4/187	51/18866	0.00162387	0.004885264	0.002217226	APOB/SOAT1/MPO/LPL	4
BP	GO:0006112	energy reserve metabolic process	5/187	86/18866	0.001651092	0.004956739	0.002249666	AKT1/GSK3B/MYC/IGF2/INS	5
BP	GO:0009791	post-embryonic development	5/187	86/18866	0.001651092	0.004956739	0.002249666	BCL2/BAX/CYP1A2/VEGFA/APOB	5
BP	GO:0042509	regulation of tyrosine phosphorylation of STAT protein	5/187	86/18866	0.001651092	0.004956739	0.002249666	IL6R/IFNG/IL4/STAT3/CAV1	5
BP	GO:0070304	positive regulation of stress-activated protein kinase signaling	7/187	172/18866	0.001662627	0.004970595	0.002255955	VEGFA/CD40LG/MMP8/NCF1/HMGCR/IL1B/ED	7
BP	GO:0034695	response to prostaglandin E	3/187	24/18866	0.001663812	0.004970595	0.002255955	PPARG/AKT1/ACACA	3
BP	GO:0042738	exogenous drug catabolic process	3/187	24/18866	0.001663812	0.004970595	0.002255955	CYP3A4/CYP1A2/NR1I2	3
BP	GO:0045821	positive regulation of glycolytic process	3/187	24/18866	0.001663812	0.004970595	0.002255955	IFNG/HIF1A/INS	3
BP	GO:0090335	regulation of brown fat cell differentiation	3/187	24/18866	0.001663812	0.004970595	0.002255955	PTGS2/MAPK14/INS	3
BP	GO:1902993	positive regulation of amyloid precursor protein catabolic process	3/187	24/18866	0.001663812	0.004970595	0.002255955	CASP3/RELA/IFNG	3
BP	GO:2000637	positive regulation of gene silencing by miRNA	3/187	24/18866	0.001663812	0.004970595	0.002255955	EGFR/TP53/STAT3	3
BP	GO:1903052	positive regulation of proteolysis involved in cellular protein catabolic process	6/187	127/18866	0.001679373	0.005013594	0.00227547	AKT1/GSK3B/MDM2/EGF/CAV1/NFE2L2	6
BP	GO:0019722	calcium-mediated signaling	8/187	222/18866	0.001707947	0.005095356	0.002312578	SELE/VCAM1/GSK3B/EGFR/KDR/CXCL8/ERBB3/EDN1	8
BP	GO:0032436	positive regulation of proteasomal ubiquitin-dependent protein catabolic process	5/187	87/18866	0.001737914	0.005181158	0.00235152	AKT1/GSK3B/MDM2/CAV1/NFE2L2	5
BP	GO:0006998	nuclear envelope organization	4/187	52/18866	0.001745728	0.005197233	0.002358816	PRKCA/CDK1/CCNB1/PRKCB	4
BP	GO:0038066	p38MAPK cascade	4/187	52/18866	0.001745728	0.005197233	0.002358816	MAPK14/VEGFA/NCF1/IL1B	4
BP	GO:0072089	stem cell proliferation	6/187	128/18866	0.001747727	0.005199579	0.002359881	VEGFA/TP53/HIF1A/GJA1/DRD2/BDNF	6

BP	GO:0007632	visual behavior	4/187	53/18866	0.001873789	0.005530505	0.002510075	HMGCR/HIF1A/DRD2/HRH1	4
BP	GO:0032964	collagen biosynthetic process	4/187	53/18866	0.001873789	0.005530505	0.002510075	PPARG/PPARD/GOT1/COL1A1	4
BP	GO:0043124	negative regulation of I-kappaB kinase/NF-kappaB signaling	4/187	53/18866	0.001873789	0.005530505	0.002510075	CASP8/STAT1/GSTP1/ESR1	4
BP	GO:0043331	response to dsRNA	4/187	53/18866	0.001873789	0.005530505	0.002510075	MAPK1/NFKBIA/MAPK3/CAV1	4
BP	GO:0043392	negative regulation of DNA binding	4/187	53/18866	0.001873789	0.005530505	0.002510075	JUN/HMOX1/NFKBIA/E2F1	4
BP	GO:0045661	regulation of myoblast differentiation	4/187	53/18866	0.001873789	0.005530505	0.002510075	MAPK14/PPARD/CXCL10/IGFBP3	4
BP	GO:0002053	positive regulation of mesenchymal cell proliferation	3/187	25/18866	0.001876997	0.005530505	0.002510075	STAT1/VEGFA/MYC	3
BP	GO:0060148	positive regulation of posttranscriptional gene silencing	3/187	25/18866	0.001876997	0.005530505	0.002510075	EGFR/TP53/STAT3	3
BP	GO:0060330	regulation of response to interferon-gamma	3/187	25/18866	0.001876997	0.005530505	0.002510075	PPARG/STAT1/IFNG	3
BP	GO:0060334	regulation of interferon-gamma-mediated signaling pathway	3/187	25/18866	0.001876997	0.005530505	0.002510075	PPARG/STAT1/IFNG	3
BP	GO:0060571	morphogenesis of an epithelial fold	3/187	25/18866	0.001876997	0.005530505	0.002510075	AR/EGFR/HIF1A	3
BP	GO:0060575	intestinal epithelial cell	3/187	25/18866	0.001876997	0.005530505	0.002510075	CDKN1A/HIF1A/PYY	3
BP	GO:0071677	positive regulation of mononuclear cell migration	3/187	25/18866	0.001876997	0.005530505	0.002510075	IL4/SERPINE1/CXCL10	3
BP	GO:2000679	positive regulation of transcription regulatory region DNA binding	3/187	25/18866	0.001876997	0.005530505	0.002510075	RB1/IFNG/PARP1	3
BP	GO:0007260	tyrosine phosphorylation of STAT protein	5/187	89/18866	0.001921541	0.005653992	0.002566121	IL6R/IFNG/IL4/STAT3/CAV1	5
BP	GO:0055006	cardiac cell development	5/187	89/18866	0.001921541	0.005653992	0.002566121	CDK1/VEGFA/CCNB1/PPARA/EDN1	5
BP	GO:0010524	positive regulation of calcium ion transport into cytosol	4/187	54/18866	0.002008214	0.005892865	0.002674536	BAX/CAV1/CXCL11/CXCL10	4
BP	GO:0031050	dsRNA processing	4/187	54/18866	0.002008214	0.005892865	0.002674536	ESR1/EGFR/TP53/STAT	4
BP	GO:0070918	production of small RNA involved in gene silencing by RNA	4/187	54/18866	0.002008214	0.005892865	0.002674536	ESR1/EGFR/TP53/STAT3	4
BP	GO:2000179	positive regulation of neural precursor cell proliferation	4/187	54/18866	0.002008214	0.005892865	0.002674536	VEGFA/EGF/HIF1A/DRD2	4
BP	GO:0060401	cytosolic calcium ion transport	7/187	178/18866	0.002020266	0.005924182	0.002688749	BCL2/BAX/CAV1/CXCL11/CXCL10/DRD2/TRPV	7
BP	GO:0002068	glandular epithelial cell development	3/187	26/18866	0.002106456	0.006146795	0.002789785	GSK3B/FASN/BAD	3
BP	GO:0042737	drug catabolic process	3/187	26/18866	0.002106456	0.006146795	0.002789785	CYP3A4/CYP1A2/NR1I2	3

BP	GO:0044068	modulation by symbiont of host cellular process	3/187	26/18866	0.002106456	0.006146795	0.002789785	CASP8/BCL2L1/BAD	3
BP	GO:0060740	prostate gland epithelium morphogenesis	3/187	26/18866	0.002106456	0.006146795	0.002789785	AR/ESR1/RXRA	3
BP	GO:0070633	transepithelial transport	3/187	26/18866	0.002106456	0.006146795	0.002789785	ABCC1/ABCG2/EDN1	3
BP	GO:0090023	positive regulation of neutrophil chemotaxis	3/187	26/18866	0.002106456	0.006146795	0.002789785	LBP/CXCL8/EDN1	3
BP	GO:1905523	positive regulation of macrophage migration	3/187	26/18866	0.002106456	0.006146795	0.002789785	MAPK14/MAPK1/MAPK3	3
BP	GO:0016579	protein deubiquitination	9/187	283/18866	0.002107636	0.006146795	0.002789785	AR/CDK1/ESR1/CCNA2/TP53/NFKBIA/MDM2/HIF1A/MYC	9
BP	GO:0046849	bone remodeling	5/187	91/18866	0.002118972	0.006175659	0.002802885	PRKCA/EGFR/CA2/GJA1/SPP1	5
BP	GO:0006165	nucleoside diphosphate phosphorylation	6/187	133/18866	0.002121696	0.006179403	0.002804584	IFNG/PPARA/STAT3/HIF1A/HK2/INS	6
BP	GO:0071825	protein-lipid complex subunit organization	4/187	55/18866	0.002149161	0.006255151	0.002838963	APOB/SOAT1/MPO/LPL	4
BP	GO:0043903	regulation of symbiotic process	8/187	231/18866	0.002188695	0.0063659	0.002889227	BCL2/JUN/STAT1/SLPI/TOP2A/BAD/CAV1/CXC	8
BP	GO:0019058	viral life cycle	10/187	341/18866	0.002195797	0.006382232	0.00289664	BCL2/DPP4/CDK1/ICAM1/SLPI/EGFR/TOP2A/CAV1/CCL2/CXCL8	10
BP	GO:0008361	regulation of cell size	7/187	181/18866	0.002220063	0.006444037	0.002924691	AKT1/GSK3B/VEGFA/CDK4/SPP1/EDN1/BDNF	7
BP	GO:1905330	regulation of morphogenesis of an epithelium	7/187	181/18866	0.002220063	0.006444037	0.002924691	AR/STAT1/ESR1/RXRA/VEGFA/GJA1/CXCL10	7
BP	GO:0031341	regulation of cell killing	5/187	92/18866	0.002223051	0.00644835	0.002926648	NOS2/ICAM1/IFNG/IL4/BAD	5
BP	GO:0046939	nucleotide phosphorylation	6/187	135/18866	0.002287086	0.006629615	0.003008917	IFNG/PPARA/STAT3/HIF1A/HK2/INS	6
BP	GO:0031295	T cell costimulation	4/187	56/18866	0.002296789	0.006648764	0.003017608	DPP4/AKT1/CD40LG/CAV1	4
BP	GO:0060688	regulation of morphogenesis of a branching structure	4/187	56/18866	0.002296789	0.006648764	0.003017608	AR/ESR1/RXRA/VEGFA	4
BP	GO:0001963	synaptic transmission, dopaminergic	3/187	27/18866	0.002352609	0.006769269	0.0030723	PTGS2/SLC6A4/DRD2	3
BP	GO:0002026	regulation of the force of heart contraction	3/187	27/18866	0.002352609	0.006769269	0.0030723	CAV1/NOS3/ADM	3
BP	GO:0009065	glutamine family amino acid catabolic process	3/187	27/18866	0.002352609	0.006769269	0.0030723	NOS2/GOT1/NOS3	3
BP	GO:0009651	response to salt stress	3/187	27/18866	0.002352609	0.006769269	0.0030723	BAX/AKR1B1/TP53	3

BP	GO:0045672	positive regulation of osteoclast differentiation	3/187	27/18866	0.002352609	0.006769269	0.0030723	IFNG/CA2/FOS	3
BP	GO:0048143	astrocyte activation	3/187	27/18866	0.002352609	0.006769269	0.0030723	EGFR/IFNG/IL1B	3
BP	GO:0050996	positive regulation of lipid catabolic process	3/187	27/18866	0.002352609	0.006769269	0.0030723	PPARA/IL1B/FABP1	3
BP	GO:1903203	regulation of oxidative stress-induced neuron death	3/187	27/18866	0.002352609	0.006769269	0.0030723	MCL1/HIF1A/PARP1	3
BP	GO:2000353	positive regulation of endothelial cell apoptotic process	3/187	27/18866	0.002352609	0.006769269	0.0030723	AKR1C3/CD40LG/CCL2	3
BP	GO:0006986	response to unfolded protein	7/187	183/18866	0.002361513	0.006781255	0.003077741	BAX/CCND1/HSPA5/CCL2/CXCL8/HSPB1/NFE2	7
BP	GO:0043433	negative regulation of DNA-binding transcription factor activity	7/187	183/18866	0.002361513	0.006781255	0.003077741	HMOX1/CYP1B1/ESR1/CAT/RB1/NFKBIA/CHU	7
BP	GO:0055001	muscle cell development	7/187	183/18866	0.002361513	0.006781255	0.003077741	BCL2/CDK1/VEGFA/CCNB1/PPARA/EDN1/ADM	7
BP	GO:0022900	electron transport chain	7/187	184/18866	0.002434795	0.006987015	0.003171127	CDK1/CYP1A2/AKR1B1/CCNB1/NCF1/CYP19A1/GSR	7
BP	GO:0019319	hexose biosynthetic process	5/187	94/18866	0.002442313	0.007003908	0.003178794	AKR1B1/PPARA/GOT1/INS/GCG	5
BP	GO:0002090	regulation of receptor internalization	4/187	57/18866	0.002451257	0.007015496	0.003184053	SELE/VEGFA/EGF/DRD	4
BP	GO:0030520	intracellular estrogen receptor signaling pathway	4/187	57/18866	0.002451257	0.007015496	0.003184053	AR/ESR1/ESR2/PARP1	4
BP	GO:0048016	inositol phosphate-mediated signaling	4/187	57/18866	0.002451257	0.007015496	0.003184053	GSK3B/ERBB3/EDN1/HRH1	4
BP	GO:0044242	cellular lipid catabolic process	8/187	236/18866	0.002498382	0.007145605	0.003243104	AKT1/CYP1B1/AKR1C3/PPARD/APOB/PPARA/LPL/FABP1	8
BP	GO:2001257	regulation of cation channel activity	7/187	185/18866	0.002509816	0.00717353	0.003255778	OPRM1/MMP9/IFNG/CAV1/CCL2/RASGRF1/DR	7
BP	GO:0010656	negative regulation of muscle cell apoptotic process	4/187	58/18866	0.002612719	0.007427167	0.003370894	HMOX1/HMGCR/NFE2L2/EDN1	4
BP	GO:0031638	zymogen activation	4/187	58/18866	0.002612719	0.007427167	0.003370894	CASP8/PLAU/BAD/SERPINE1	4
BP	GO:0002360	T cell lineage commitment	3/187	28/18866	0.002615857	0.007427167	0.003370894	BCL2/TP53/STAT3	3
BP	GO:0008299	isoprenoid biosynthetic process	3/187	28/18866	0.002615857	0.007427167	0.003370894	CYP1A1/AKR1C3/HMGCR	3
BP	GO:0010460	positive regulation of heart rate	3/187	28/18866	0.002615857	0.007427167	0.003370894	ADRA1B/EDN1/ADM	3
BP	GO:0060512	prostate gland morphogenesis	3/187	28/18866	0.002615857	0.007427167	0.003370894	AR/ESR1/RXRA	3
BP	GO:0071280	cellular response to copper ion	3/187	28/18866	0.002615857	0.007427167	0.003370894	CYP1A2/CYP1A1/NFE2	3
BP	GO:0071880	adenylate cyclase-activating adrenergic receptor signaling	3/187	28/18866	0.002615857	0.007427167	0.003370894	ADRA1B/ADRA2A/DRD2	3

BP	GO:1902932	positive regulation of alcohol biosynthetic process	3/187	28/18866	0.002615857	0.007427167	0.003370894	IFNG/IL1B/HRH1	3
BP	GO:2000191	regulation of fatty acid transport	3/187	28/18866	0.002615857	0.007427167	0.003370894	AKT1/IL1B/EDN1	3
BP	GO:0060968	regulation of gene silencing	6/187	140/18866	0.002743032	0.007783103	0.003532439	PPARG/ESR1/CDK2/EGFR/TP53/STAT3	6
BP	GO:0031102	neuron projection regeneration	4/187	59/18866	0.002781332	0.007886563	0.003579396	BCL2/JUN/SPP1/ADM	4
BP	GO:0050764	regulation of phagocytosis	5/187	97/18866	0.002800096	0.007934528	0.003601165	PPARG/IFNG/SOD1/IL1B/CCL2	5
BP	GO:0007586	digestion	6/187	141/18866	0.002841845	0.008047521	0.003652448	PRSS1/ADRA2A/AKR1C1/PTGER3/TRPV1/FABP	6
BP	GO:0001958	endochondral ossification	3/187	29/18866	0.002896587	0.008180953	0.003713007	MMP13/COL1A1/RUNX	3
BP	GO:0031063	regulation of histone deacetylation	3/187	29/18866	0.002896587	0.008180953	0.003713007	MAPK8/VEGFA/TP53	3
BP	GO:0036075	replacement ossification	3/187	29/18866	0.002896587	0.008180953	0.003713007	MMP13/COL1A1/RUNX	3
BP	GO:0048873	homeostasis of number of cells within a tissue	3/187	29/18866	0.002896587	0.008180953	0.003713007	BCL2/BAX/NOS3	3
BP	GO:0001657	ureteric bud development	5/187	98/18866	0.002927362	0.008257008	0.003747526	BCL2/CAT/VEGFA/MYC/BDNF	5
BP	GO:0030316	osteoclast differentiation	5/187	98/18866	0.002927362	0.008257008	0.003747526	MAPK14/IFNG/IL4/CA2/FOS	5
BP	GO:0007292	female gamete generation	6/187	142/18866	0.002943301	0.008291071	0.003762986	TNFAIP6/PTGS2/BCL2/CCNB1/TOP2A/NOS3	6
BP	GO:0072073	kidney epithelium development	6/187	142/18866	0.002943301	0.008291071	0.003762986	BCL2/STAT1/CAT/VEGFA/MYC/BDNF	6
BP	GO:0032653	regulation of interleukin-10 production	4/187	60/18866	0.002957249	0.008324898	0.003778338	IL4/CD40LG/MMP8/STAT3	4
BP	GO:0072006	nephron development	6/187	143/18866	0.003047444	0.008573182	0.003891024	IL6R/BCL2/STAT1/VEGFA/MYC/ENPEP	6
BP	GO:0051817	modulation of process of other organism involved in symbiotic interaction	5/187	99/18866	0.003058758	0.008588128	0.003897808	JUN/CASP8/RXRA/BCL2L1/BAD	5
BP	GO:0072163	mesonephric epithelium development	5/187	99/18866	0.003058758	0.008588128	0.003897808	BCL2/CAT/VEGFA/MYC/BDNF	5
BP	GO:0072164	mesonephric tubule development	5/187	99/18866	0.003058758	0.008588128	0.003897808	BCL2/CAT/VEGFA/MYC/BDNF	5
BP	GO:0070646	protein modification by small protein removal	9/187	300/18866	0.003112255	0.008732621	0.003963388	AR/CDK1/ESR1/CCNA2/TP53/NFKBIA/MDM2/HIF1A/MYC	9
BP	GO:0033619	membrane protein proteolysis	4/187	61/18866	0.003140623	0.008806462	0.003996901	RELA/ADRA2A/IFNG/IL1B	4
BP	GO:0060078	regulation of postsynaptic membrane potential	6/187	144/18866	0.003154316	0.008839085	0.004011707	OPRM1/AKT1/GSK3B/DRD2/TRPV1/BDNF	6

BP	GO:0046364	monosaccharide biosynthetic process	5/187	100/18866	0.003194358	0.008924429	0.004050442	AKR1B1/PPARA/GOT1/INS/GCG	5
BP	GO:0007176	regulation of epidermal growth factor-activated receptor activity	3/187	30/18866	0.003195166	0.008924429	0.004050442	ADRA2A/NCF1/EGF	3
BP	GO:0010800	positive regulation of peptidyl-threonine phosphorylation	3/187	30/18866	0.003195166	0.008924429	0.004050442	MAPK1/EGF/GCG	3
BP	GO:0036475	neuron death in response to oxidative stress	3/187	30/18866	0.003195166	0.008924429	0.004050442	MCL1/HIF1A/PARP1	3
BP	GO:0060142	regulation of syncytium formation by plasma membrane fusion	3/187	30/18866	0.003195166	0.008924429	0.004050442	MAPK14/IL4/CXCL10	3
BP	GO:0010518	positive regulation of phospholipase activity	4/187	62/18866	0.003331604	0.0092887	0.004215769	SELE/ESR1/EGFR/BDNF	4
BP	GO:0010803	regulation of tumor necrosis factor-mediated signaling pathway	4/187	62/18866	0.003331604	0.0092887	0.004215769	CASP8/IKBKB/GSTP1/CHUK	4
BP	GO:0010522	regulation of calcium ion transport into cytosol	5/187	101/18866	0.003334239	0.0092887	0.004215769	BCL2/BAX/CAV1/CXCL11/CXCL10	5
BP	GO:0060191	regulation of lipase activity	5/187	101/18866	0.003334239	0.0092887	0.004215769	SELE/ESR1/EGFR/LPL/BDNF	5
BP	GO:0034620	cellular response to unfolded protein	6/187	146/18866	0.003376426	0.009387952	0.004260816	BAX/CCND1/HSPA5/CCL2/CXCL8/NFE2L2	6
BP	GO:0044344	cellular response to fibroblast growth factor stimulus	6/187	146/18866	0.003376426	0.009387952	0.004260816	MAPK1/MAPK3/CCL2/CXCL8/COL1A1/RUNX2	6
BP	GO:0046631	alpha-beta T cell activation	6/187	146/18866	0.003376426	0.009387952	0.004260816	BCL2/IFNG/IL4/STAT3/IRF1/INS	6
BP	GO:0022600	digestive system process	5/187	102/18866	0.003478474	0.009659179	0.004383915	ADRA2A/AKR1C1/PTGER3/TRPV1/FABP1	5
BP	GO:0043502	regulation of muscle adaptation	5/187	102/18866	0.003478474	0.009659179	0.004383915	PRKCA/PPARA/NOS3/PARP1/EDN1	5
BP	GO:0007043	cell-cell junction assembly	6/187	147/18866	0.003491753	0.009689783	0.004397805	PRKCA/IKBKB/CAV1/GJA1/IL1B/CLDN4	6
BP	GO:0002675	positive regulation of acute inflammatory response	3/187	31/18866	0.003511947	0.009701924	0.004403315	PTGS2/IL1B/PTGER3	3
BP	GO:0034368	protein-lipid complex remodeling	3/187	31/18866	0.003511947	0.009701924	0.004403315	APOB/MPO/LPL	3
BP	GO:0034369	plasma lipoprotein particle	3/187	31/18866	0.003511947	0.009701924	0.004403315	APOB/MPO/LPL	3
BP	GO:0043171	peptide catabolic process	3/187	31/18866	0.003511947	0.009701924	0.004403315	DPEP1/NPEPPS/ENPEP	3
BP	GO:1900745	positive regulation of p38MAPK cascade	3/187	31/18866	0.003511947	0.009701924	0.004403315	VEGFA/NCF1/IL1B	3
BP	GO:1902253	regulation of intrinsic apoptotic signaling pathway by p53 class mediator	3/187	31/18866	0.003511947	0.009701924	0.004403315	BCL2/TP53/MDM2	3
BP	GO:1902624	positive regulation of neutrophil migration	3/187	31/18866	0.003511947	0.009701924	0.004403315	LBP/CXCL8/EDN1	3

BP	GO:0006081	cellular aldehyde metabolic process	4/187	63/18866	0.003530344	0.009733955	0.004417853	RELA/CYP1B1/AKR1C3/AKR1C1	4
BP	GO:0032613	interleukin-10 production	4/187	63/18866	0.003530344	0.009733955	0.004417853	IL4/CD40LG/MMP8/STAT3	4
BP	GO:0070059	intrinsic apoptotic signaling pathway in response to endoplasmic reticulum stress	4/187	63/18866	0.003530344	0.009733955	0.004417853	BCL2/BAX/BCL2L1/TP53	4
BP	GO:0001823	mesonephros development	5/187	103/18866	0.003627141	0.009968834	0.004524455	BCL2/CAT/VEGFA/MYC/BDNF	5
BP	GO:0002042	cell migration involved in sprouting angiogenesis	5/187	103/18866	0.003627141	0.009968834	0.004524455	PTGS2/AKT1/HMOX1/VEGFA/KDR	5
BP	GO:0006885	regulation of pH	5/187	103/18866	0.003627141	0.009968834	0.004524455	BCL2/MAPK1/CA2/MAPK3/EDN1	5
BP	GO:0034766	negative regulation of ion transmembrane transport	5/187	103/18866	0.003627141	0.009968834	0.004524455	AKT1/ADRA2A/MMP9/CAV1/DRD2	5
BP	GO:0060079	excitatory postsynaptic potential	5/187	103/18866	0.003627141	0.009968834	0.004524455	OPRM1/AKT1/GSK3B/DRD2/TRPV1	5
BP	GO:1903364	positive regulation of cellular protein catabolic process	6/187	149/18866	0.003731169	0.010244507	0.004649572	AKT1/GSK3B/MDM2/EGF/CAV1/NFE2L2	6
BP	GO:0045453	bone resorption	4/187	64/18866	0.00373699	0.010244507	0.004649572	PRKCA/EGFR/CA2/SPP1	4
BP	GO:0046173	polyol biosynthetic process	4/187	64/18866	0.00373699	0.010244507	0.004649572	CYP3A4/AKR1B1/GOT1/HRH1	4
BP	GO:1900449	regulation of glutamate receptor signaling pathway	4/187	64/18866	0.00373699	0.010244507	0.004649572	OPRM1/IFNG/CCL2/RASGRF1	4
BP	GO:0032350	regulation of hormone metabolic process	3/187	32/18866	0.003847267	0.010486551	0.004759426	AKR1C3/HIF1A/ADM	3
BP	GO:0034367	protein-containing complex remodeling	3/187	32/18866	0.003847267	0.010486551	0.004759426	APOB/MPO/LPL	3
BP	GO:0042744	hydrogen peroxide catabolic process	3/187	32/18866	0.003847267	0.010486551	0.004759426	CAT/DUOX2/MPO	3
BP	GO:0045589	regulation of regulatory T cell differentiation	3/187	32/18866	0.003847267	0.010486551	0.004759426	IL2RA/IFNG/IRF1	3
BP	GO:0046949	fatty-acyl-CoA biosynthetic process	3/187	32/18866	0.003847267	0.010486551	0.004759426	FASN/ACACA/SCD	3
BP	GO:0051968	positive regulation of synaptic transmission, glutamatergic	3/187	32/18866	0.003847267	0.010486551	0.004759426	PTGS2/EGFR/CCL2	3
BP	GO:0071295	cellular response to vitamin	3/187	32/18866	0.003847267	0.010486551	0.004759426	PPARG/MDM2/COL1A1	3
BP	GO:0071353	cellular response to interleukin-4	3/187	32/18866	0.003847267	0.010486551	0.004759426	CDK4/FASN/HSPA5	3
BP	GO:0071875	adrenergic receptor signaling	3/187	32/18866	0.003847267	0.010486551	0.004759426	ADRA1B/ADRA2A/DRD	3
BP	GO:0016236	macroautophagy	9/187	310/18866	0.003859826	0.010507441	0.004768907	CASP3/AKT1/MAPK8/HMOX1/TP53/IL4/KDR/MAPK3/HIF1A	9

BP	GO:0090287	regulation of cellular response to growth factor stimulus	9/187	310/18866	0.003859826	0.010507441	0.004768907	TP53/XIAP/GOT1/HIF1A/HSPA5/CAV1/IL1B/PRKCB/RUNX2	9
BP	GO:1901888	regulation of cell junction assembly	7/187	200/18866	0.003862866	0.010509052	0.004769638	IKBKB/VEGFA/KDR/CAV1/GJA1/IL1B/BDNF	7
BP	GO:0000086	G2/M transition of mitotic cell cycle	8/187	254/18866	0.00390663	0.010621382	0.004820621	CDK1/CDK2/CCNA2/CCND1/CDKN1A/CDK4/CCNB1/CHEK2	8
BP	GO:0044070	regulation of anion transport	5/187	105/18866	0.00393807	0.010693317	0.004853269	AKT1/CA2/CES1/IL1B/EDN1	5
BP	GO:0097006	regulation of plasma lipoprotein particle levels	5/187	105/18866	0.00393807	0.010693317	0.004853269	HMOX1/APOB/SOAT1/MPO/LPL	5
BP	GO:0051224	negative regulation of protein transport	6/187	151/18866	0.003982566	0.010807305	0.004905004	OPRM1/ADRA2A/HMGCR/IL1B/DRD2/INS	6
BP	GO:0007179	transforming growth factor beta receptor signaling pathway	7/187	202/18866	0.004078428	0.011060448	0.005019895	JUN/TP53/GOT1/FOS/HSPA5/CAV1/PARP1	7
BP	GO:0046632	alpha-beta T cell differentiation	5/187	106/18866	0.004100482	0.011113237	0.005043854	BCL2/IFNG/IL4/STAT3/IRF1	5
BP	GO:0006090	pyruvate metabolic process	6/187	152/18866	0.004112869	0.011139777	0.005055899	IFNG/PPARA/STAT3/HIF1A/HK2/INS	6
BP	GO:0002676	regulation of chronic inflammatory response	2/187	10/18866	0.004173527	0.011159086	0.005064663	IL4/CYP19A1	2
BP	GO:0021936	regulation of cerebellar granule cell precursor proliferation	2/187	10/18866	0.004173527	0.011159086	0.005064663	SLC6A4/EGF	2
BP	GO:0032000	positive regulation of fatty acid beta-oxidation	2/187	10/18866	0.004173527	0.011159086	0.005064663	PPARA/FABP1	2
BP	GO:0032070	regulation of deoxyribonuclease activity	2/187	10/18866	0.004173527	0.011159086	0.005064663	AKT1/PCNA	2
BP	GO:0032308	positive regulation of prostaglandin secretion	2/187	10/18866	0.004173527	0.011159086	0.005064663	IL1B/EDN1	2
BP	GO:0034350	regulation of glial cell apoptotic process	2/187	10/18866	0.004173527	0.011159086	0.005064663	PRKCA/CCL2	2
BP	GO:0045348	positive regulation of MHC class II biosynthetic process	2/187	10/18866	0.004173527	0.011159086	0.005064663	IFNG/IL4	2
BP	GO:0045793	positive regulation of cell size	2/187	10/18866	0.004173527	0.011159086	0.005064663	CDK4/EDN1	2
BP	GO:0051901	positive regulation of mitochondrial depolarization	2/187	10/18866	0.004173527	0.011159086	0.005064663	KDR/PARP1	2
BP	GO:0051918	negative regulation of fibrinolysis	2/187	10/18866	0.004173527	0.011159086	0.005064663	THBD/SERPINE1	2
BP	GO:0060068	vagina development	2/187	10/18866	0.004173527	0.011159086	0.005064663	BAX/ESR1	2
BP	GO:0060346	bone trabecula formation	2/187	10/18866	0.004173527	0.011159086	0.005064663	MMP2/COL1A1	2
BP	GO:0070099	regulation of chemokine-mediated signaling pathway	2/187	10/18866	0.004173527	0.011159086	0.005064663	HIF1A/EDN1	2

BP	GO:0071104	response to interleukin-9	2/187	10/18866	0.004173527	0.011159086	0.005064663	STAT1/STAT3	2
BP	GO:0071394	cellular response to testosterone stimulus	2/187	10/18866	0.004173527	0.011159086	0.005064663	AR/SPP1	2
BP	GO:0072203	cell proliferation involved in metanephros development	2/187	10/18866	0.004173527	0.011159086	0.005064663	STAT1/MYC	2
BP	GO:0120252	hydrocarbon metabolic process	2/187	10/18866	0.004173527	0.011159086	0.005064663	CYP1A1/AKR1B1	2
BP	GO:1900222	negative regulation of amyloid-beta clearance	2/187	10/18866	0.004173527	0.011159086	0.005064663	IFNG/HMGCR	2
BP	GO:1903862	positive regulation of oxidative phosphorylation	2/187	10/18866	0.004173527	0.011159086	0.005064663	CDK1/CCNB1	2
BP	GO:0032922	circadian regulation of gene expression	4/187	66/18866	0.004174585	0.011159086	0.005064663	AHR/TOP1/PPARA/DRD2	4
BP	GO:0050922	negative regulation of chemotaxis	4/187	66/18866	0.004174585	0.011159086	0.005064663	DPP4/GSTP1/CYP19A1/CCL2	4
BP	GO:0001975	response to amphetamine	3/187	33/18866	0.004201448	0.011175226	0.005071988	ICAM1/SOD1/DRD2	3
BP	GO:0010464	regulation of mesenchymal cell proliferation	3/187	33/18866	0.004201448	0.011175226	0.005071988	STAT1/VEGFA/MYC	3
BP	GO:0010955	negative regulation of protein processing	3/187	33/18866	0.004201448	0.011175226	0.005071988	MDM2/XIAP/SERPINE1	3
BP	GO:0040018	positive regulation of multicellular organism growth	3/187	33/18866	0.004201448	0.011175226	0.005071988	BCL2/IGF2/DRD2	3
BP	GO:0046627	negative regulation of insulin receptor signaling pathway	3/187	33/18866	0.004201448	0.011175226	0.005071988	RELA/IL1B/PRKCB	3
BP	GO:0048011	neurotrophin TRK receptor signaling pathway	3/187	33/18866	0.004201448	0.011175226	0.005071988	CASP3/RAF1/BDNF	3
BP	GO:1902003	regulation of amyloid-beta formation	3/187	33/18866	0.004201448	0.011175226	0.005071988	CASP3/RELA/IFNG	3
BP	GO:1903318	negative regulation of protein maturation	3/187	33/18866	0.004201448	0.011175226	0.005071988	MDM2/XIAP/SERPINE1	3
BP	GO:0071774	response to fibroblast growth factor	6/187	153/18866	0.004246301	0.011287534	0.005122961	MAPK1/MAPK3/CCL2/CXCL8/COL1A1/RUNX2	6
BP	GO:0002548	monocyte chemotaxis	4/187	67/18866	0.004405822	0.011661032	0.005292476	IL6R/CCL2/SERPINE1/CXCL10	4
BP	GO:0032890	regulation of organic acid transport	4/187	67/18866	0.004405822	0.011661032	0.005292476	AKT1/CES1/IL1B/EDN1	4
BP	GO:0045123	cellular extravasation	4/187	67/18866	0.004405822	0.011661032	0.005292476	ICAM1/SELE/VCAM1/CCL2	4
BP	GO:0045670	regulation of osteoclast	4/187	67/18866	0.004405822	0.011661032	0.005292476	IFNG/IL4/CA2/FOS	4
BP	GO:0072678	T cell migration	4/187	67/18866	0.004405822	0.011661032	0.005292476	ICAM1/CCL2/CXCL11/CXCL10	4
BP	GO:1900015	regulation of cytokine production involved in inflammatory response	4/187	67/18866	0.004405822	0.011661032	0.005292476	NOS2/ALOX5/MAPK14/STAT3	4
BP	GO:1903672	positive regulation of sprouting angiogenesis	4/187	67/18866	0.004405822	0.011661032	0.005292476	PTGS2/HMOX1/VEGFA/KDR	4

BP	GO:0035966	response to topologically incorrect protein	7/187	205/18866	0.004418727	0.011687982	0.005304708	BAX/CCND1/HSPA5/CCL2/CXCL8/HSPB1/NFE2	7
BP	GO:0006959	humoral immune response	10/187	377/18866	0.004478447	0.011838655	0.005373092	BCL2/ALOX5/SLPI/IFNG/IL1B/CCL2/CXCL8/CXCL11/CXCL2/CXCL10	10
BP	GO:1904950	negative regulation of establishment of protein localization	6/187	155/18866	0.004522729	0.011948355	0.005422881	OPRM1/ADRA2A/HMGCR/IL1B/DRD2/INS	6
BP	GO:0007202	activation of phospholipase C	3/187	34/18866	0.004574795	0.012026695	0.005458436	SELE/EGFR/BDNF	3
BP	GO:0016242	negative regulation of	3/187	34/18866	0.004574795	0.012026695	0.005458436	AKT1/HMOX1/TP53	3
BP	GO:0032148	activation of protein kinase B	3/187	34/18866	0.004574795	0.012026695	0.005458436	AKT1/ADRA2A/INS	3
BP	GO:0035633	maintenance of blood-brain barrier	3/187	34/18866	0.004574795	0.012026695	0.005458436	PTGS2/VEGFA/GJA1	3
BP	GO:0035774	positive regulation of insulin secretion involved in cellular response to glucose stimulus	3/187	34/18866	0.004574795	0.012026695	0.005458436	BAD/HIF1A/GCG	3
BP	GO:0042908	xenobiotic transport	3/187	34/18866	0.004574795	0.012026695	0.005458436	NR1I2/ABCC1/ABCG2	3
BP	GO:0046685	response to arsenic-containing substance	3/187	34/18866	0.004574795	0.012026695	0.005458436	HMOX1/CYP1A1/CDKN1A	3
BP	GO:0070102	interleukin-6-mediated signaling pathway	3/187	34/18866	0.004574795	0.012026695	0.005458436	IL6R/STAT1/STAT3	3
BP	GO:0002286	T cell activation involved in immune response	5/187	109/18866	0.004616406	0.012121242	0.005501347	ICAM1/TP53/IFNG/IL4/STAT3	5
BP	GO:0018958	phenol-containing compound metabolic process	5/187	109/18866	0.004616406	0.012121242	0.005501347	BCL2/AKR1B1/TYR/DUOX2/DRD2	5
BP	GO:0002753	cytoplasmic pattern recognition receptor signaling pathway	4/187	68/18866	0.004645542	0.012186306	0.005530877	CASP8/RELA/NFKBIA/XIAP	4
BP	GO:0051961	negative regulation of nervous system development	9/187	319/18866	0.004646863	0.012186306	0.005530877	SLC6A4/GSK3B/TP53/MDM2/STAT3/IL1B/SPP1/TRPV1/BDNF	9
BP	GO:0048592	eye morphogenesis	6/187	156/18866	0.004665816	0.01222854	0.005550045	BCL2/BAX/VEGFA/STAT3/HIF1A/BDNF	6
BP	GO:0006641	triglyceride metabolic process	5/187	110/18866	0.004798192	0.012560145	0.005700548	CAT/APOB/CAV1/LPL/FABP1	5
BP	GO:1901800	positive regulation of proteasomal protein catabolic process	5/187	110/18866	0.004798192	0.012560145	0.005700548	AKT1/GSK3B/MDM2/CAV1/NFE2L2	5
BP	GO:0071230	cellular response to amino acid stimulus	4/187	69/18866	0.004893885	0.012795035	0.005807155	EGFR/BCL2L1/MMP2/COL1A1	4
BP	GO:2000573	positive regulation of DNA biosynthetic process	4/187	69/18866	0.004893885	0.012795035	0.005807155	MAPK1/PCNA/MAPK3/MYC	4
BP	GO:0006909	phagocytosis	10/187	382/18866	0.004906196	0.012819414	0.00581822	CD14/LBP/PPARG/MAPK1/IFNG/MET/MAPK3/SOD1/IL1B/CCL2	10

BP	GO:0007519	skeletal muscle tissue development	6/187	158/18866	0.004961958	0.012877964	0.005844793	BCL2/MAPK14/RB1/HMGCR/FOS/CAV1	6
BP	GO:1903707	negative regulation of hemopoiesis	6/187	158/18866	0.004961958	0.012877964	0.005844793	NFKBIA/ERBB2/IL4/MYC/NFE2L2/IRF1	6
BP	GO:0000083	regulation of transcription involved in G1/S transition of mitotic cell	3/187	35/18866	0.0049676	0.012877964	0.005844793	RB1/PCNA/E2F1	3
BP	GO:0006691	leukotriene metabolic process	3/187	35/18866	0.0049676	0.012877964	0.005844793	ALOX5/DPEP1/ABCC1	3
BP	GO:0030262	apoptotic nuclear changes	3/187	35/18866	0.0049676	0.012877964	0.005844793	BAX/CASP3/TOP2A	3
BP	GO:0030947	regulation of vascular endothelial growth factor receptor signaling pathway	3/187	35/18866	0.0049676	0.012877964	0.005844793	HIF1A/IL1B/PRKCB	3
BP	GO:0032228	regulation of synaptic transmission, GABAergic	3/187	35/18866	0.0049676	0.012877964	0.005844793	CA2/DRD2/BDNF	3
BP	GO:0032735	positive regulation of interleukin-12 production	3/187	35/18866	0.0049676	0.012877964	0.005844793	MAPK14/IFNG/CD40LG	3
BP	GO:0034390	smooth muscle cell apoptotic	3/187	35/18866	0.0049676	0.012877964	0.005844793	PPARG/IFNG/EDN1	3
BP	GO:0034391	regulation of smooth muscle cell apoptotic process	3/187	35/18866	0.0049676	0.012877964	0.005844793	PPARG/IFNG/EDN1	3
BP	GO:0045066	regulatory T cell differentiation	3/187	35/18866	0.0049676	0.012877964	0.005844793	IL2RA/IFNG/IRF1	3
BP	GO:0070670	response to interleukin-4	3/187	35/18866	0.0049676	0.012877964	0.005844793	CDK4/FASN/HSPA5	3
BP	GO:0098801	regulation of renal system process	3/187	35/18866	0.0049676	0.012877964	0.005844793	GJA1/DRD2/EDN1	3
BP	GO:0051149	positive regulation of muscle cell differentiation	5/187	111/18866	0.004985007	0.012915291	0.005861734	BCL2/MAPK14/MDM2/IL4/EDN1	5
BP	GO:0045216	cell-cell junction organization	7/187	210/18866	0.005033077	0.012927051	0.005867072	PRKCA/IKBKB/VEGFA/CAV1/GJA1/IL1B/CLDN	7
BP	GO:0006527	arginine catabolic process	2/187	11/18866	0.005067838	0.012927051	0.005867072	NOS2/NOS3	2
BP	GO:0010918	positive regulation of mitochondrial membrane potential	2/187	11/18866	0.005067838	0.012927051	0.005867072	AKT1/BAD	2
BP	GO:0017085	response to insecticide	2/187	11/18866	0.005067838	0.012927051	0.005867072	CYP1A1/CCNB1	2
BP	GO:0031284	positive regulation of guanylate cyclase activity	2/187	11/18866	0.005067838	0.012927051	0.005867072	NOS2/NOS3	2
BP	GO:0032306	regulation of prostaglandin secretion	2/187	11/18866	0.005067838	0.012927051	0.005867072	IL1B/EDN1	2
BP	GO:0033148	positive regulation of intracellular estrogen receptor signaling pathway	2/187	11/18866	0.005067838	0.012927051	0.005867072	AR/PARP1	2
BP	GO:0033327	Leydig cell differentiation	2/187	11/18866	0.005067838	0.012927051	0.005867072	AR/CCND1	2
BP	GO:0034776	response to histamine	2/187	11/18866	0.005067838	0.012927051	0.005867072	DRD2/HRH1	2
BP	GO:0042447	hormone catabolic process	2/187	11/18866	0.005067838	0.012927051	0.005867072	CYP19A1/SPP1	2
BP	GO:0043471	regulation of cellular carbohydrate catabolic process	2/187	11/18866	0.005067838	0.012927051	0.005867072	TP53/INS	2
BP	GO:0045899	positive regulation of RNA polymerase II transcription preinitiation complex assembly	2/187	11/18866	0.005067838	0.012927051	0.005867072	ESR1/TP53	2

BP	GO:0051974	negative regulation of telomerase activity	2/187	11/18866	0.005067838	0.012927051	0.005867072	PPARG/TP53	2
BP	GO:0060439	trachea morphogenesis	2/187	11/18866	0.005067838	0.012927051	0.005867072	MAPK1/MAPK3	2
BP	GO:0060525	prostate glandular acinus	2/187	11/18866	0.005067838	0.012927051	0.005867072	ESR1/RXRA	2
BP	GO:0061307	cardiac neural crest cell differentiation involved in heart	2/187	11/18866	0.005067838	0.012927051	0.005867072	MAPK1/MAPK3	2
BP	GO:0061308	cardiac neural crest cell development involved in heart development	2/187	11/18866	0.005067838	0.012927051	0.005867072	MAPK1/MAPK3	2
BP	GO:0070106	interleukin-27-mediated signaling pathway	2/187	11/18866	0.005067838	0.012927051	0.005867072	STAT1/STAT3	2
BP	GO:0070673	response to interleukin-18	2/187	11/18866	0.005067838	0.012927051	0.005867072	AKT1/ALOX5	2
BP	GO:0070757	interleukin-35-mediated signaling pathway	2/187	11/18866	0.005067838	0.012927051	0.005867072	STAT1/STAT3	2
BP	GO:0071281	cellular response to iron ion	2/187	11/18866	0.005067838	0.012927051	0.005867072	HMOX1/CCNB1	2
BP	GO:0090557	establishment of endothelial intestinal barrier	2/187	11/18866	0.005067838	0.012927051	0.005867072	ICAM1/FASN	2
BP	GO:0106049	regulation of cellular response to osmotic stress	2/187	11/18866	0.005067838	0.012927051	0.005867072	PTGS2/BAD	2
BP	GO:1903800	positive regulation of production of miRNAs involved in gene silencing by miRNA	2/187	11/18866	0.005067838	0.012927051	0.005867072	EGFR/TP53	2
BP	GO:1990440	positive regulation of transcription from RNA polymerase II promoter in response to endoplasmic reticulum stress	2/187	11/18866	0.005067838	0.012927051	0.005867072	TP53/HSPA5	2
BP	GO:2001269	positive regulation of cysteine-type endopeptidase activity involved in apoptotic signaling pathway	2/187	11/18866	0.005067838	0.012927051	0.005867072	BAX/CASP8	2
BP	GO:0050777	negative regulation of immune response	6/187	159/18866	0.005115103	0.013024399	0.005911254	PPARG/HMOX1/IL2RA/IL4/DRD2/INS	6
BP	GO:0055067	monovalent inorganic cation homeostasis	6/187	159/18866	0.005115103	0.013024399	0.005911254	BCL2/MAPK1/CA2/MAPK3/DRD2/EDN1	6
BP	GO:2000058	regulation of ubiquitin-dependent protein catabolic process	6/187	159/18866	0.005115103	0.013024399	0.005911254	AKT1/GSK3B/MDM2/EGF/CAV1/NFE2L2	6
BP	GO:0002534	cytokine production involved in inflammatory response	4/187	70/18866	0.005150988	0.013100232	0.005945672	NOS2/ALOX5/MAPK14/STAT3	4
BP	GO:0050795	regulation of behavior	4/187	70/18866	0.005150988	0.013100232	0.005945672	STAT3/GJA1/DRD2/INS	4
BP	GO:0005976	polysaccharide metabolic process	5/187	112/18866	0.005176926	0.013158403	0.005972073	AKT1/GSK3B/EGF/IGF2/INS	5
BP	GO:0001569	branching involved in blood vessel morphogenesis	3/187	36/18866	0.005380141	0.013618483	0.006180885	VEGFA/KDR/EDN1	3
BP	GO:0006007	glucose catabolic process	3/187	36/18866	0.005380141	0.013618483	0.006180885	TP53/BAD/HK2	3

BP	GO:0010543	regulation of platelet activation	3/187	36/18866	0.005380141	0.013618483	0.006180885	PRKCA/NOS3/THBD	3
BP	GO:0031572	G2 DNA damage checkpoint	3/187	36/18866	0.005380141	0.013618483	0.006180885	CDK1/CHEK1/CDKN1A	3
BP	GO:0046676	negative regulation of insulin	3/187	36/18866	0.005380141	0.013618483	0.006180885	ADRA2A/HMGCR/DRD2	3
BP	GO:0051973	positive regulation of telomerase activity	3/187	36/18866	0.005380141	0.013618483	0.006180885	MAPK1/MAPK3/MYC	3
BP	GO:1905332	positive regulation of morphogenesis of an epithelium	3/187	36/18866	0.005380141	0.013618483	0.006180885	AR/VEGFA/GJA1	3
BP	GO:0033692	cellular polysaccharide biosynthetic process	4/187	71/18866	0.005416988	0.013695601	0.006215886	AKT1/GSK3B/IGF2/INS	4
BP	GO:0042531	positive regulation of tyrosine phosphorylation of STAT protein	4/187	71/18866	0.005416988	0.013695601	0.006215886	IL6R/IFNG/IL4/STAT3	4
BP	GO:0000723	telomere maintenance	6/187	161/18866	0.005431765	0.013716803	0.006225509	MAPK1/PCNA/MAPK3/MYC/PARP1/TEP1	6
BP	GO:0034767	positive regulation of ion transmembrane transport	6/187	161/18866	0.005431765	0.013716803	0.006225509	BAX/IFNG/HTR3A/CCL2/CXCL11/CXCL10	6
BP	GO:1903305	regulation of regulated secretory pathway	6/187	162/18866	0.005595371	0.014121651	0.006409253	HMOX1/ADRA2A/GSK3B/IL4/PRKCB/DRD2	6
BP	GO:0042440	pigment metabolic process	4/187	72/18866	0.005692019	0.014357132	0.006516128	BCL2/HMOX1/TYR/UGT1A1	4
BP	GO:0048640	negative regulation of developmental growth	5/187	115/18866	0.005784034	0.014580655	0.006617576	SLC6A4/CDKN1A/PPARA/GJA1/SPP1	5
BP	GO:0006921	cellular component disassembly involved in execution phase of apoptosis	3/187	37/18866	0.005812681	0.014609977	0.006630884	BAX/CASP3/TOP2A	3
BP	GO:0030212	hyaluronan metabolic process	3/187	37/18866	0.005812681	0.014609977	0.006630884	AKT1/EGF/IL1B	3
BP	GO:0071542	dopaminergic neuron differentiation	3/187	37/18866	0.005812681	0.014609977	0.006630884	GSK3B/VEGFA/HIF1A	3
BP	GO:0097242	amyloid-beta clearance	3/187	37/18866	0.005812681	0.014609977	0.006630884	IFNG/IL4/HMGCR	3
BP	GO:0098926	postsynaptic signal transduction	3/187	37/18866	0.005812681	0.014609977	0.006630884	OPRM1/RELA/STAT3	3
BP	GO:0005977	glycogen metabolic process	4/187	73/18866	0.005976214	0.014977167	0.006797537	AKT1/GSK3B/IGF2/INS	4
BP	GO:0010517	regulation of phospholipase activity	4/187	73/18866	0.005976214	0.014977167	0.006797537	SELE/ESR1/EGFR/BDNF	4
BP	GO:0010611	regulation of cardiac muscle hypertrophy	4/187	73/18866	0.005976214	0.014977167	0.006797537	PRKCA/PPARA/PARP1/EDN1	4
BP	GO:0051155	positive regulation of striated muscle cell differentiation	4/187	73/18866	0.005976214	0.014977167	0.006797537	BCL2/MAPK14/IL4/EDN1	4
BP	GO:0051966	regulation of synaptic transmission, glutamatergic	4/187	73/18866	0.005976214	0.014977167	0.006797537	PTGS2/EGFR/CCL2/DRD2	4
BP	GO:0060964	regulation of gene silencing by miRNA	5/187	116/18866	0.005997096	0.014984397	0.006800819	PPARG/ESR1/EGFR/TP53/STAT3	5
BP	GO:0010810	regulation of cell-substrate adhesion	7/187	217/18866	0.00599851	0.014984397	0.006800819	BCL2/PLAU/GSK3B/VEGFA/KDR/SERPINE1/COL1A1	7

BP	GO:0002002	regulation of angiotensin levels in blood	2/187	12/18866	0.006041927	0.014984397	0.006800819	CES1/ENPEP	2
BP	GO:0002003	angiotensin maturation	2/187	12/18866	0.006041927	0.014984397	0.006800819	CES1/ENPEP	2
BP	GO:0010232	vascular transport	2/187	12/18866	0.006041927	0.014984397	0.006800819	GJA1/ABCG2	2
BP	GO:0031392	regulation of prostaglandin biosynthetic process	2/187	12/18866	0.006041927	0.014984397	0.006800819	PTGS2/IL1B	2
BP	GO:0033629	negative regulation of cell adhesion mediated by integrin	2/187	12/18866	0.006041927	0.014984397	0.006800819	CYP1B1/SERPINE1	2
BP	GO:0055015	ventricular cardiac muscle cell development	2/187	12/18866	0.006041927	0.014984397	0.006800819	CDK1/CCNB1	2
BP	GO:0060442	branching involved in prostate gland morphogenesis	2/187	12/18866	0.006041927	0.014984397	0.006800819	ESR1/RXRA	2
BP	GO:0060453	regulation of gastric acid secretion	2/187	12/18866	0.006041927	0.014984397	0.006800819	PTGER3/TRPV1	2
BP	GO:0060742	epithelial cell differentiation involved in prostate gland	2/187	12/18866	0.006041927	0.014984397	0.006800819	AR/RXRA	2
BP	GO:0061517	macrophage proliferation	2/187	12/18866	0.006041927	0.014984397	0.006800819	MAPK1/MAPK3	2
BP	GO:0070243	regulation of thymocyte apoptotic process	2/187	12/18866	0.006041927	0.014984397	0.006800819	TP53/HIF1A	2
BP	GO:0097284	hepatocyte apoptotic process	2/187	12/18866	0.006041927	0.014984397	0.006800819	BCL2L1/RB1	2
BP	GO:0150065	regulation of deacetylase activity	2/187	12/18866	0.006041927	0.014984397	0.006800819	MAPK8/VEGFA	2
BP	GO:1901503	ether biosynthetic process	2/187	12/18866	0.006041927	0.014984397	0.006800819	ALOX5/FASN	2
BP	GO:1904181	positive regulation of membrane depolarization	2/187	12/18866	0.006041927	0.014984397	0.006800819	KDR/PARP1	2
BP	GO:2001214	positive regulation of vasculogenesis	2/187	12/18866	0.006041927	0.014984397	0.006800819	KDR/ADM	2
BP	GO:0014902	myotube differentiation	5/187	117/18866	0.006215627	0.015397395	0.006988262	BCL2/MAPK14/IL4/CXCL10/BDNF	5
BP	GO:0072676	lymphocyte migration	5/187	117/18866	0.006215627	0.015397395	0.006988262	AKT1/ICAM1/CCL2/CXCL11/CXCL10	5
BP	GO:0019433	triglyceride catabolic process	3/187	38/18866	0.006265469	0.015459984	0.007016669	APOB/LPL/FABP1	3
BP	GO:0032733	positive regulation of interleukin-10 production	3/187	38/18866	0.006265469	0.015459984	0.007016669	IL4/CD40LG/STAT3	3
BP	GO:0048009	insulin-like growth factor receptor signaling pathway	3/187	38/18866	0.006265469	0.015459984	0.007016669	AR/AKT1/IGFBP3	3
BP	GO:1905898	positive regulation of response to endoplasmic reticulum stress	3/187	38/18866	0.006265469	0.015459984	0.007016669	BAX/CAV1/NFE2L2	3
BP	GO:2000144	positive regulation of DNA-templated transcription, initiation	3/187	38/18866	0.006265469	0.015459984	0.007016669	JUN/ESR1/TP53	3
BP	GO:0006073	cellular glucan metabolic process	4/187	74/18866	0.006269702	0.015459984	0.007016669	AKT1/GSK3B/IGF2/INS	4
BP	GO:0006635	fatty acid beta-oxidation	4/187	74/18866	0.006269702	0.015459984	0.007016669	AKT1/PPARD/PPARA/FABP1	4
BP	GO:0044042	glucan metabolic process	4/187	74/18866	0.006269702	0.015459984	0.007016669	AKT1/GSK3B/IGF2/INS	4

BP	GO:0035967	cellular response to topologically incorrect protein	6/187	166/18866	0.006286011	0.015491301	0.007030882	BAX/CCND1/HSPA5/CCL2/CXCL8/NFE2L2	6
BP	GO:0021987	cerebral cortex development	5/187	118/18866	0.006439696	0.01584276	0.007190395	BAX/GSK3B/EGFR/BAD/HIF1A	5
BP	GO:0050868	negative regulation of T cell activation	5/187	118/18866	0.006439696	0.01584276	0.007190395	CASP3/ERBB2/IL2RA/IL4/IRF1	5
BP	GO:0051209	release of sequestered calcium ion into cytosol	5/187	118/18866	0.006439696	0.01584276	0.007190395	BAX/CXCL11/CXCL10/DRD2/TRPV1	5
BP	GO:0061844	antimicrobial humoral immune response mediated by antimicrobial peptide	4/187	75/18866	0.006572613	0.016142006	0.007326211	CXCL8/CXCL11/CXCL2/CXCL10	4
BP	GO:0072088	nephron epithelium morphogenesis	4/187	75/18866	0.006572613	0.016142006	0.007326211	BCL2/STAT1/VEGFA/MYC	4
BP	GO:1901983	regulation of protein acetylation	4/187	75/18866	0.006572613	0.016142006	0.007326211	GSK3B/CHEK1/MAPK3/IL1B	4
BP	GO:0060538	skeletal muscle organ development	6/187	168/18866	0.006653685	0.016331281	0.007412115	BCL2/MAPK14/RB1/HMGCR/FOS/CAV1	6
BP	GO:0045165	cell fate commitment	8/187	278/18866	0.006657289	0.016331281	0.007412115	BCL2/CASP3/AR/PPARG/TP53/MCL1/STAT3/RUNX2	8
BP	GO:0051283	negative regulation of sequestering of calcium ion	5/187	119/18866	0.006669376	0.016351589	0.007421332	BAX/CXCL11/CXCL10/DRD2/TRPV1	5
BP	GO:0010831	positive regulation of myotube differentiation	3/187	39/18866	0.006738741	0.016483998	0.007481428	BCL2/MAPK14/IL4	3
BP	GO:0033146	regulation of intracellular estrogen receptor signaling pathway	3/187	39/18866	0.006738741	0.016483998	0.007481428	AR/ESR1/PARP1	3
BP	GO:0034205	amyloid-beta formation	3/187	39/18866	0.006738741	0.016483998	0.007481428	CASP3/RELA/IFNG	3
BP	GO:0038179	neurotrophin signaling pathway	3/187	39/18866	0.006738741	0.016483998	0.007481428	CASP3/RAF1/BDNF	3
BP	GO:0017157	regulation of exocytosis	7/187	222/18866	0.00676845	0.016547242	0.007510131	HMOX1/ADRA2A/GSK3B/IFNG/IL4/PRKCB/DR	7
BP	GO:0055002	striated muscle cell development	6/187	169/18866	0.006843266	0.016720628	0.007588824	BCL2/CDK1/VEGFA/CCNB1/PPARA/EDN1	6
BP	GO:0014743	regulation of muscle hypertrophy	4/187	76/18866	0.006885072	0.016803646	0.007626503	PRKCA/PPARA/PARP1/EDN1	4
BP	GO:0060193	positive regulation of lipase activity	4/187	76/18866	0.006885072	0.016803646	0.007626503	SELE/ESR1/EGFR/BDNF	4
BP	GO:0060147	regulation of posttranscriptional gene silencing	5/187	120/18866	0.006904738	0.016832504	0.0076396	PPARG/ESR1/EGFR/TP53/STAT3	5
BP	GO:0060966	regulation of gene silencing by RNA	5/187	120/18866	0.006904738	0.016832504	0.0076396	PPARG/ESR1/EGFR/TP53/STAT3	5
BP	GO:1990778	protein localization to cell periphery	9/187	340/18866	0.006969464	0.016980652	0.007706839	AR/IKBKB/AKT1/EGFR/BCL2L1/IFNG/MAPK10/CAV1/INS	9

BP	GO:2000241	regulation of reproductive process	6/187	170/18866	0.007036736	0.01713375	0.007776324	AR/ESR1/RXRA/VEGFA/PLB1/GJA1	6
BP	GO:0014842	regulation of skeletal muscle satellite cell proliferation	2/187	13/18866	0.007094142	0.01713375	0.007776324	PPARD/STAT3	2
BP	GO:0031953	negative regulation of protein autophosphorylation	2/187	13/18866	0.007094142	0.01713375	0.007776324	JUN/CAV1	2
BP	GO:0032352	positive regulation of hormone metabolic process	2/187	13/18866	0.007094142	0.01713375	0.007776324	HIF1A/ADM	2
BP	GO:0033089	positive regulation of T cell differentiation in thymus	2/187	13/18866	0.007094142	0.01713375	0.007776324	IL1B/IL1A	2
BP	GO:0033145	positive regulation of intracellular steroid hormone receptor signaling pathway	2/187	13/18866	0.007094142	0.01713375	0.007776324	AR/PARP1	2
BP	GO:0034370	triglyceride-rich lipoprotein particle remodeling	2/187	13/18866	0.007094142	0.01713375	0.007776324	APOB/LPL	2
BP	GO:0034379	very-low-density lipoprotein particle assembly	2/187	13/18866	0.007094142	0.01713375	0.007776324	APOB/SOAT1	2
BP	GO:0043568	positive regulation of insulin-like growth factor receptor signaling pathway	2/187	13/18866	0.007094142	0.01713375	0.007776324	AR/IGFBP3	2
BP	GO:0051481	negative regulation of cytosolic calcium ion concentration	2/187	13/18866	0.007094142	0.01713375	0.007776324	GOT1/DRD2	2
BP	GO:0061029	eyelid development in camera-type	2/187	13/18866	0.007094142	0.01713375	0.007776324	JUN/EGFR	2
BP	GO:0061043	regulation of vascular wound healing	2/187	13/18866	0.007094142	0.01713375	0.007776324	ALOX5/SERPINE1	2
BP	GO:0061430	bone trabecula morphogenesis	2/187	13/18866	0.007094142	0.01713375	0.007776324	MMP2/COL1A1	2
BP	GO:0072683	T cell extravasation	2/187	13/18866	0.007094142	0.01713375	0.007776324	ICAM1/CCL2	2
BP	GO:1902947	regulation of tau-protein kinase activity	2/187	13/18866	0.007094142	0.01713375	0.007776324	RB1/IFNG	2
BP	GO:0043583	ear development	7/187	224/18866	0.007096158	0.01713375	0.007776324	BCL2/CCNA2/MAPK1/MAPK3/SOD1/EDN1/BDN	7
BP	GO:0051282	regulation of sequestering of calcium ion	5/187	121/18866	0.007145851	0.017244036	0.007826378	BAX/CXCL11/CXCL10/DRD2/TRPV1	5
BP	GO:0014015	positive regulation of gliogenesis	4/187	77/18866	0.007207203	0.017317384	0.007859668	PPARG/RELA/IL1B/E2F	4
BP	GO:0032413	negative regulation of ion transmembrane transporter activity	4/187	77/18866	0.007207203	0.017317384	0.007859668	ADRA2A/MMP9/CAV1/DRD2	4
BP	GO:0061418	regulation of transcription from RNA polymerase II promoter in response to hypoxia	4/187	77/18866	0.007207203	0.017317384	0.007859668	VEGFA/TP53/HIF1A/NFE2L2	4
BP	GO:0072028	nephron morphogenesis	4/187	77/18866	0.007207203	0.017317384	0.007859668	BCL2/STAT1/VEGFA/MYC	4

BP	GO:0016054	organic acid catabolic process	8/187	282/18866	0.007230797	0.017317384	0.007859668	PON1/NOS2/AKT1/PPARD/PPARA/GOT1/NOS3/FABP1	8
BP	GO:0046395	carboxylic acid catabolic process	8/187	282/18866	0.007230797	0.017317384	0.007859668	PON1/NOS2/AKT1/PPARD/PPARA/GOT1/NOS3/FABP1	8
BP	GO:0010613	positive regulation of cardiac muscle hypertrophy	3/187	40/18866	0.00723272	0.017317384	0.007859668	PRKCA/PARP1/EDN1	3
BP	GO:0034142	toll-like receptor 4 signaling	3/187	40/18866	0.00723272	0.017317384	0.007859668	CD14/LBP/NFKBIA	3
BP	GO:0050691	regulation of defense response to virus by host	3/187	40/18866	0.00723272	0.017317384	0.007859668	STAT1/IL4/IL1B	3
BP	GO:0050892	intestinal absorption	3/187	40/18866	0.00723272	0.017317384	0.007859668	ADRA2A/AKR1C1/FABP	3
BP	GO:0051281	positive regulation of release of sequestered calcium ion into cytosol	3/187	40/18866	0.00723272	0.017317384	0.007859668	BAX/CXCL11/CXCL10	3
BP	GO:0090184	positive regulation of kidney development	3/187	40/18866	0.00723272	0.017317384	0.007859668	IL6R/VEGFA/MYC	3
BP	GO:1902991	regulation of amyloid precursor protein catabolic process	3/187	40/18866	0.00723272	0.017317384	0.007859668	CASP3/RELA/IFNG	3
BP	GO:1903427	negative regulation of reactive oxygen species biosynthetic process	3/187	40/18866	0.00723272	0.017317384	0.007859668	STAT3/CAV1/INS	3
BP	GO:0002697	regulation of immune effector process	11/187	470/18866	0.007359893	0.017612052	0.007993406	LBP/NOS2/STAT1/HMOX1/ICAM1/IL2RA/IFNG/IL4/CD40LG/IL1B/INS	11
BP	GO:0050688	regulation of defense response to	4/187	78/18866	0.00753913	0.01803091	0.008183509	STAT1/IL2RA/IL4/IL1B	4
BP	GO:0006937	regulation of muscle contraction	6/187	173/18866	0.00764092	0.018233725	0.008275559	PTGS2/ADRA1B/ADRA2A/SOD1/CAV1/EDN1	6
BP	GO:0009152	purine ribonucleotide biosynthetic process	6/187	173/18866	0.00764092	0.018233725	0.008275559	IL4/FASN/STAT3/ACACA/PARP1/SCD	6
BP	GO:0021543	pallium development	6/187	173/18866	0.00764092	0.018233725	0.008275559	BAX/CASP3/GSK3B/EGFR/BAD/HIF1A	6
BP	GO:0050954	sensory perception of mechanical stimulus	6/187	173/18866	0.00764092	0.018233725	0.008275559	CASP3/ICAM1/BIRC5/SOD1/COL1A1/TRPV1	6
BP	GO:0002251	organ or tissue specific immune response	3/187	41/18866	0.007747616	0.018426879	0.008363224	IL6R/NOS2/IL4	3
BP	GO:0007212	dopamine receptor signaling	3/187	41/18866	0.007747616	0.018426879	0.008363224	OPRM1/GSK3B/DRD2	3
BP	GO:0014742	positive regulation of muscle hypertrophy	3/187	41/18866	0.007747616	0.018426879	0.008363224	PRKCA/PARP1/EDN1	3
BP	GO:0030890	positive regulation of B cell proliferation	3/187	41/18866	0.007747616	0.018426879	0.008363224	BCL2/CDKN1A/IL4	3
BP	GO:0035337	fatty-acyl-CoA metabolic process	3/187	41/18866	0.007747616	0.018426879	0.008363224	FASN/ACACA/SCD	3
BP	GO:2000008	regulation of protein localization to cell surface	3/187	41/18866	0.007747616	0.018426879	0.008363224	AKT1/EGF/BDNF	3

BP	GO:0032200	telomere organization	6/187	174/18866	0.007850386	0.018660969	0.008469468	MAPK1/PCNA/MAPK3/MYC/PARP1/TEP1	6
BP	GO:0043407	negative regulation of MAP kinase activity	4/187	79/18866	0.007880971	0.018712946	0.008493058	GSTP1/HMGCR/CAV1/IL1B	4
BP	GO:0099601	regulation of neurotransmitter receptor activity	4/187	79/18866	0.007880971	0.018712946	0.008493058	OPRM1/IFNG/CCL2/RASGRF1	4
BP	GO:0051208	sequestering of calcium ion	5/187	124/18866	0.007904403	0.018758209	0.008513601	BAX/CXCL11/CXCL10/DRD2/TRPV1	5
BP	GO:0007160	cell-matrix adhesion	7/187	230/18866	0.008150074	0.019237453	0.008731111	BCL2/VCAM1/PLAU/GSK3B/VEGFA/KDR/SERPINE1	7
BP	GO:0032479	regulation of type I interferon production	5/187	125/18866	0.008169221	0.019237453	0.008731111	CD14/RELA/STAT1/CHUK/IRF1	5
BP	GO:0010649	regulation of cell communication by electrical coupling	2/187	14/18866	0.008222857	0.019237453	0.008731111	CAV1/GJA1	2
BP	GO:0010917	negative regulation of mitochondrial membrane potential	2/187	14/18866	0.008222857	0.019237453	0.008731111	BAX/TRPV1	2
BP	GO:0014841	skeletal muscle satellite cell proliferation	2/187	14/18866	0.008222857	0.019237453	0.008731111	PPARD/STAT3	2
BP	GO:0014854	response to inactivity	2/187	14/18866	0.008222857	0.019237453	0.008731111	CAT/DRD2	2
BP	GO:0014857	regulation of skeletal muscle cell proliferation	2/187	14/18866	0.008222857	0.019237453	0.008731111	PPARD/STAT3	2
BP	GO:0030213	hyaluronan biosynthetic process	2/187	14/18866	0.008222857	0.019237453	0.008731111	EGF/IL1B	2
BP	GO:0031282	regulation of guanylate cyclase	2/187	14/18866	0.008222857	0.019237453	0.008731111	NOS2/NOS3	2
BP	GO:0032042	mitochondrial DNA metabolic	2/187	14/18866	0.008222857	0.019237453	0.008731111	TP53/PARP1	2
BP	GO:0033604	negative regulation of catecholamine secretion	2/187	14/18866	0.008222857	0.019237453	0.008731111	ADRA2A/DRD2	2
BP	GO:0035437	maintenance of protein localization in endoplasmic reticulum	2/187	14/18866	0.008222857	0.019237453	0.008731111	HSPA5/GJA1	2
BP	GO:0035810	positive regulation of urine volume	2/187	14/18866	0.008222857	0.019237453	0.008731111	DRD2/EDN1	2
BP	GO:0035815	positive regulation of renal sodium excretion	2/187	14/18866	0.008222857	0.019237453	0.008731111	DRD2/EDN1	2
BP	GO:0036295	cellular response to increased oxygen levels	2/187	14/18866	0.008222857	0.019237453	0.008731111	PPARG/CAV1	2
BP	GO:0038166	angiotensin-activated signaling pathway	2/187	14/18866	0.008222857	0.019237453	0.008731111	CA2/CAV1	2
BP	GO:0045472	response to ether	2/187	14/18866	0.008222857	0.019237453	0.008731111	CDK4/MDM2	2
BP	GO:0045898	regulation of RNA polymerase II transcription preinitiation complex assembly	2/187	14/18866	0.008222857	0.019237453	0.008731111	ESR1/TP53	2
BP	GO:0047484	regulation of response to osmotic stress	2/187	14/18866	0.008222857	0.019237453	0.008731111	PTGS2/BAD	2

BP	GO:0050930	induction of positive chemotaxis	2/187	14/18866	0.008222857	0.019237453	0.008731111	VEGFA/CXCL8	2
BP	GO:0051917	regulation of fibrinolysis	2/187	14/18866	0.008222857	0.019237453	0.008731111	THBD/SERPINE1	2
BP	GO:0070431	nucleotide-binding oligomerization domain containing 2 signaling	2/187	14/18866	0.008222857	0.019237453	0.008731111	RELA/NFKBIA	2
BP	GO:0106070	regulation of adenylate cyclase-activating G protein-coupled receptor signaling pathway	2/187	14/18866	0.008222857	0.019237453	0.008731111	OPRM1/PRKCA	2
BP	GO:1901722	regulation of cell proliferation involved in kidney development	2/187	14/18866	0.008222857	0.019237453	0.008731111	IL6R/MYC	2
BP	GO:1903894	regulation of IRE1-mediated unfolded protein response	2/187	14/18866	0.008222857	0.019237453	0.008731111	BAX/HSPA5	2
BP	GO:2001279	regulation of unsaturated fatty acid biosynthetic process	2/187	14/18866	0.008222857	0.019237453	0.008731111	PTGS2/IL1B	2
BP	GO:0014032	neural crest cell development	4/187	80/18866	0.008232844	0.019239847	0.008732197	MAPK1/MAPK3/HIF1A/EDN1	4
BP	GO:0032204	regulation of telomere maintenance	4/187	80/18866	0.008232844	0.019239847	0.008732197	MAPK1/MAPK3/MYC/PARP1	4
BP	GO:0006509	membrane protein ectodomain proteolysis	3/187	42/18866	0.008283626	0.019285037	0.008752707	ADRA2A/IFNG/IL1B	3
BP	GO:0009268	response to pH	3/187	42/18866	0.008283626	0.019285037	0.008752707	CA2/GJA1/TRPV1	3
BP	GO:0034383	low-density lipoprotein particle clearance	3/187	42/18866	0.008283626	0.019285037	0.008752707	HMOX1/APOB/SOAT1	3
BP	GO:0046006	regulation of activated T cell proliferation	3/187	42/18866	0.008283626	0.019285037	0.008752707	CASP3/IL2RA/IGF2	3
BP	GO:0046621	negative regulation of organ growth	3/187	42/18866	0.008283626	0.019285037	0.008752707	SLC6A4/PPARA/GJA1	3
BP	GO:0090278	negative regulation of peptide hormone secretion	3/187	42/18866	0.008283626	0.019285037	0.008752707	ADRA2A/HMGCR/DRD2	3
BP	GO:0090279	regulation of calcium ion import	3/187	42/18866	0.008283626	0.019285037	0.008752707	EGF/CCL2/GCG	3
BP	GO:0030324	lung development	6/187	177/18866	0.008503613	0.019786456	0.008980281	CYP1A2/EGFR/VEGFA/MAPK1/MAPK3/NOS3	6
BP	GO:0050829	defense response to Gram-negative bacterium	4/187	81/18866	0.008594866	0.019987951	0.009071732	IL6R/LBP/NOS2/SERPINE1	4
BP	GO:0019079	viral genome replication	5/187	127/18866	0.008717216	0.020239594	0.009185943	BCL2/SLPI/TOP2A/CCL2/CXCL8	5
BP	GO:0032606	type I interferon production	5/187	127/18866	0.008717216	0.020239594	0.009185943	CD14/RELA/STAT1/CHUK/IRF1	5
BP	GO:0051053	negative regulation of DNA metabolic process	5/187	127/18866	0.008717216	0.020239594	0.009185943	PPARG/CDKN1A/TP53/GJA1/PARP1	5
BP	GO:0045823	positive regulation of heart	3/187	43/18866	0.008840935	0.020493594	0.009301223	ADRA1B/EDN1/ADM	3
BP	GO:0051154	negative regulation of striated muscle cell differentiation	3/187	43/18866	0.008840935	0.020493594	0.009301223	PPARA/CXCL10/BDNF	3

BP	GO:1903146	regulation of autophagy of mitochondrion	3/187	43/18866	0.008840935	0.020493594	0.009301223	TP53/HIF1A/HK2	3
BP	GO:0014013	regulation of gliogenesis	5/187	128/18866	0.009000529	0.020852278	0.009464015	PPARG/RELA/CDK1/IL1B/E2F1	5
BP	GO:0002576	platelet degranulation	5/187	129/18866	0.00929014	0.021491145	0.009753972	VEGFA/SOD1/EGF/SERPINE1/IGF2	5
BP	GO:0001570	vasculogenesis	4/187	83/18866	0.009349804	0.021491145	0.009753972	VEGFA/KDR/CAV1/AD	4
BP	GO:2001021	negative regulation of response to DNA damage stimulus	4/187	83/18866	0.009349804	0.021491145	0.009753972	BCL2/BCL2L1/MDM2/CHEK2	4
BP	GO:0050768	negative regulation of neurogenesis	8/187	295/18866	0.009354583	0.021491145	0.009753972	SLC6A4/GSK3B/TP53/MDM2/STAT3/IL1B/SPP1/BDNF	8
BP	GO:0010463	mesenchymal cell proliferation	3/187	44/18866	0.009419717	0.021491145	0.009753972	STAT1/VEGFA/MYC	3
BP	GO:0032965	regulation of collagen biosynthetic process	3/187	44/18866	0.009419717	0.021491145	0.009753972	PPARG/PPARD/GOT1	3
BP	GO:0042181	ketone biosynthetic process	3/187	44/18866	0.009419717	0.021491145	0.009753972	AKR1C3/CYP19A1/ADM	3
BP	GO:0002281	macrophage activation involved in immune response	2/187	15/18866	0.009426468	0.021491145	0.009753972	LBP/IFNG	2
BP	GO:0006089	lactate metabolic process	2/187	15/18866	0.009426468	0.021491145	0.009753972	TP53/HIF1A	2
BP	GO:0009415	response to water	2/187	15/18866	0.009426468	0.021491145	0.009753972	AKR1B1/MAPK10	2
BP	GO:0014856	skeletal muscle cell proliferation	2/187	15/18866	0.009426468	0.021491145	0.009753972	PPARD/STAT3	2
BP	GO:0016114	terpenoid biosynthetic process	2/187	15/18866	0.009426468	0.021491145	0.009753972	CYP1A1/AKR1C3	2
BP	GO:0020027	hemoglobin metabolic process	2/187	15/18866	0.009426468	0.021491145	0.009753972	CAT/HIF1A	2
BP	GO:0021924	cell proliferation in external granule layer	2/187	15/18866	0.009426468	0.021491145	0.009753972	SLC6A4/EGF	2
BP	GO:0021930	cerebellar granule cell precursor proliferation	2/187	15/18866	0.009426468	0.021491145	0.009753972	SLC6A4/EGF	2
BP	GO:0042159	lipoprotein catabolic process	2/187	15/18866	0.009426468	0.021491145	0.009753972	APOB/CTSD	2
BP	GO:0042976	activation of Janus kinase activity	2/187	15/18866	0.009426468	0.021491145	0.009753972	IL6R/IL4	2
BP	GO:0043374	CD8-positive, alpha-beta T cell differentiation	2/187	15/18866	0.009426468	0.021491145	0.009753972	BCL2/IRF1	2
BP	GO:0044849	estrous cycle	2/187	15/18866	0.009426468	0.021491145	0.009753972	OPRM1/PCNA	2
BP	GO:0045346	regulation of MHC class II biosynthetic process	2/187	15/18866	0.009426468	0.021491145	0.009753972	IFNG/IL4	2
BP	GO:0045820	negative regulation of glycolytic process	2/187	15/18866	0.009426468	0.021491145	0.009753972	PPARA/STAT3	2
BP	GO:0045837	negative regulation of membrane potential	2/187	15/18866	0.009426468	0.021491145	0.009753972	BAX/TRPV1	2
BP	GO:0048070	regulation of developmental pigmentation	2/187	15/18866	0.009426468	0.021491145	0.009753972	BCL2/BAX	2
BP	GO:0048569	post-embryonic animal organ development	2/187	15/18866	0.009426468	0.021491145	0.009753972	BAX/VEGFA	2

BP	GO:0071474	cellular hyperosmotic response	2/187	15/18866	0.009426468	0.021491145	0.009753972	AKR1B1/MAPK10	2
BP	GO:0072075	metanephric mesenchyme	2/187	15/18866	0.009426468	0.021491145	0.009753972	STAT1/MYC	2
BP	GO:0097202	activation of cysteine-type endopeptidase activity	2/187	15/18866	0.009426468	0.021491145	0.009753972	CASP8/BAD	2
BP	GO:1900119	positive regulation of execution phase of apoptosis	2/187	15/18866	0.009426468	0.021491145	0.009753972	BAX/TP53	2
BP	GO:2000402	negative regulation of lymphocyte migration	2/187	15/18866	0.009426468	0.021491145	0.009753972	AKT1/CCL2	2
BP	GO:2001171	positive regulation of ATP biosynthetic process	2/187	15/18866	0.009426468	0.021491145	0.009753972	IL4/STAT3	2
BP	GO:0030323	respiratory tube development	6/187	181/18866	0.009434135	0.021497208	0.009756723	CYP1A2/EGFR/VEGFA/MAPK1/MAPK3/NOS3	6
BP	GO:0048864	stem cell development	4/187	84/18866	0.009742942	0.022189097	0.010070744	MAPK1/MAPK3/HIF1A/EDN1	4
BP	GO:0031589	cell-substrate adhesion	9/187	359/18866	0.009758263	0.022212207	0.010081232	BCL2/VCAM1/PLAU/GSK3B/VEGFA/KDR/PPARD/SERPINE1/COL1A1	9
BP	GO:0003206	cardiac chamber morphogenesis	5/187	131/18866	0.009888521	0.022484862	0.01020498	RXRA/TP53/MDM2/HIF1A/NOS3	5
BP	GO:0032434	regulation of proteasomal ubiquitin-dependent protein catabolic process	5/187	131/18866	0.009888521	0.022484862	0.01020498	AKT1/GSK3B/MDM2/CAV1/NFE2L2	5
BP	GO:0001974	blood vessel remodeling	3/187	45/18866	0.010020132	0.022711946	0.010308044	BAX/MDM2/NOS3	3
BP	GO:0003197	endocardial cushion development	3/187	45/18866	0.010020132	0.022711946	0.010308044	MDM2/NOS3/ERBB3	3
BP	GO:0007520	myoblast fusion	3/187	45/18866	0.010020132	0.022711946	0.010308044	MAPK14/IL4/CXCL10	3
BP	GO:0014002	astrocyte development	3/187	45/18866	0.010020132	0.022711946	0.010308044	EGFR/IFNG/IL1B	3
BP	GO:0014047	glutamate secretion	3/187	45/18866	0.010020132	0.022711946	0.010308044	GJA1/TRPV1/BDNF	3
BP	GO:0043114	regulation of vascular permeability	3/187	45/18866	0.010020132	0.022711946	0.010308044	VEGFA/ADM/HRH1	3
BP	GO:0002695	negative regulation of leukocyte activation	6/187	184/18866	0.010178195	0.023058042	0.010465123	CASP3/HMOX1/ERBB2/IL2RA/IL4/IRF1	6
BP	GO:0042177	negative regulation of protein catabolic process	5/187	133/18866	0.010512878	0.023803685	0.010803541	NOS2/RELA/EGFR/HMGCR/INS	5
BP	GO:0007193	adenylate cyclase-inhibiting G protein-coupled receptor signaling	4/187	86/18866	0.010561086	0.02388764	0.010841645	OPRM1/ADRA2A/DRD2/EDN1	4
BP	GO:0070664	negative regulation of leukocyte proliferation	4/187	86/18866	0.010561086	0.02388764	0.010841645	CASP3/GSTP1/ERBB2/IL2RA	4
BP	GO:0035987	endodermal cell differentiation	3/187	46/18866	0.01064233	0.023919664	0.010856179	MMP2/MMP9/MMP8	3
BP	GO:0045581	negative regulation of T cell differentiation	3/187	46/18866	0.01064233	0.023919664	0.010856179	ERBB2/IL4/IRF1	3
BP	GO:0048066	developmental pigmentation	3/187	46/18866	0.01064233	0.023919664	0.010856179	BCL2/BAX/TYR	3
BP	GO:0050798	activated T cell proliferation	3/187	46/18866	0.01064233	0.023919664	0.010856179	CASP3/IL2RA/IGF2	3
BP	GO:1900744	regulation of p38MAPK cascade	3/187	46/18866	0.01064233	0.023919664	0.010856179	VEGFA/NCF1/IL1B	3

BP	GO:2000142	regulation of DNA-templated transcription, initiation	3/187	46/18866	0.01064233	0.023919664	0.010856179	JUN/ESR1/TP53	3
BP	GO:0009260	ribonucleotide biosynthetic process	6/187	186/18866	0.010696856	0.023919664	0.010856179	IL4/FASN/STAT3/ACACA/PARP1/SCD	6
BP	GO:0007567	parturition	2/187	16/18866	0.010703395	0.023919664	0.010856179	CYP1A1/EDN1	2
BP	GO:0009812	flavonoid metabolic process	2/187	16/18866	0.010703395	0.023919664	0.010856179	CYP1A1/UGT1A1	2
BP	GO:0010225	response to UV-C	2/187	16/18866	0.010703395	0.023919664	0.010856179	TP53/MDM2	2
BP	GO:0021534	cell proliferation in hindbrain	2/187	16/18866	0.010703395	0.023919664	0.010856179	SLC6A4/EGF	2
BP	GO:0034374	low-density lipoprotein particle remodeling	2/187	16/18866	0.010703395	0.023919664	0.010856179	APOB/MPO	2
BP	GO:0043116	negative regulation of vascular permeability	2/187	16/18866	0.010703395	0.023919664	0.010856179	VEGFA/ADM	2
BP	GO:0045342	MHC class II biosynthetic process	2/187	16/18866	0.010703395	0.023919664	0.010856179	IFNG/IL4	2
BP	GO:0045838	positive regulation of membrane potential	2/187	16/18866	0.010703395	0.023919664	0.010856179	AKT1/BAD	2
BP	GO:0048521	negative regulation of behavior	2/187	16/18866	0.010703395	0.023919664	0.010856179	DRD2/INS	2
BP	GO:0060576	intestinal epithelial cell development	2/187	16/18866	0.010703395	0.023919664	0.010856179	CDKN1A/HIF1A	2
BP	GO:0061450	trophoblast cell migration	2/187	16/18866	0.010703395	0.023919664	0.010856179	VEGFA/GJA1	2
BP	GO:1900451	positive regulation of glutamate receptor signaling pathway	2/187	16/18866	0.010703395	0.023919664	0.010856179	IFNG/CCL2	2
BP	GO:1901163	regulation of trophoblast cell	2/187	16/18866	0.010703395	0.023919664	0.010856179	VEGFA/GJA1	2
BP	GO:1903358	regulation of Golgi organization	2/187	16/18866	0.010703395	0.023919664	0.010856179	MAPK1/MAPK3	2
BP	GO:1904294	positive regulation of ERAD	2/187	16/18866	0.010703395	0.023919664	0.010856179	CAV1/NFE2L2	2
BP	GO:2001212	regulation of vasculogenesis	2/187	16/18866	0.010703395	0.023919664	0.010856179	KDR/ADM	2
BP	GO:0046718	viral entry into host cell	5/187	134/18866	0.010834958	0.02420108	0.010983903	DPP4/CDK1/ICAM1/EGFR/CAV1	5
BP	GO:0002285	lymphocyte activation involved in immune response	6/187	187/18866	0.010963099	0.024461842	0.011102252	ICAM1/TP53/IFNG/IL4/CD40LG/STAT3	6
BP	GO:0043409	negative regulation of MAPK cascade	6/187	187/18866	0.010963099	0.024461842	0.011102252	AKT1/GSTP1/HMGCR/CAV1/MYC/IL1B	6
BP	GO:0060291	long-term synaptic potentiation	4/187	87/18866	0.010986299	0.024488152	0.011114193	GSK3B/MAPK1/DRD2/INS	4
BP	GO:0090049	regulation of cell migration involved in sprouting angiogenesis	4/187	87/18866	0.010986299	0.024488152	0.011114193	PTGS2/HMOX1/VEGFA/KDR	4
BP	GO:0007157	heterophilic cell-cell adhesion via plasma membrane cell adhesion molecules	3/187	47/18866	0.01128645	0.025131085	0.011405994	ICAM1/SELE/VCAM1	3
BP	GO:0030261	chromosome condensation	3/187	47/18866	0.01128645	0.025131085	0.011405994	CDK1/CCNB1/TOP2A	3
BP	GO:0032410	negative regulation of transporter activity	4/187	88/18866	0.011422406	0.025420627	0.011537406	ADRA2A/MMP9/CAV1/DRD2	4
BP	GO:0045727	positive regulation of translation	5/187	136/18866	0.011499233	0.025578347	0.011608989	RXRA/MAPK1/CDK4/ERBB2/MAPK3	5

BP	GO:0019730	antimicrobial humoral response	5/187	137/18866	0.011841552	0.026326144	0.011948384	SLPI/CXCL8/CXCL11/CXCL2/CXCL10	5
BP	GO:0014033	neural crest cell differentiation	4/187	89/18866	0.011869506	0.026374632	0.011970391	MAPK1/MAPK3/HIF1A/EDN1	4
BP	GO:0010799	regulation of peptidyl-threonine phosphorylation	3/187	48/18866	0.011952621	0.02647709	0.012016892	MAPK1/EGF/GCG	3
BP	GO:0045601	regulation of endothelial cell differentiation	3/187	48/18866	0.011952621	0.02647709	0.012016892	IKBKB/VEGFA/IL1B	3
BP	GO:0046461	neutral lipid catabolic process	3/187	48/18866	0.011952621	0.02647709	0.012016892	APOB/LPL/FABP1	3
BP	GO:0046464	acylglycerol catabolic process	3/187	48/18866	0.011952621	0.02647709	0.012016892	APOB/LPL/FABP1	3
BP	GO:0051932	synaptic transmission, GABAergic	3/187	48/18866	0.011952621	0.02647709	0.012016892	CA2/DRD2/BDNF	3
BP	GO:1900271	regulation of long-term synaptic potentiation	3/187	48/18866	0.011952621	0.02647709	0.012016892	GSK3B/DRD2/INS	3
BP	GO:0002031	G protein-coupled receptor internalization	2/187	17/18866	0.012052083	0.026492366	0.012023826	DRD2/ADM	2
BP	GO:0010566	regulation of ketone biosynthetic process	2/187	17/18866	0.012052083	0.026492366	0.012023826	AKR1C3/ADM	2
BP	GO:0031000	response to caffeine	2/187	17/18866	0.012052083	0.026492366	0.012023826	PPARG/CACNA1S	2
BP	GO:0031065	positive regulation of histone deacetylation	2/187	17/18866	0.012052083	0.026492366	0.012023826	VEGFA/TP53	2
BP	GO:0032305	positive regulation of icosanoid secretion	2/187	17/18866	0.012052083	0.026492366	0.012023826	IL1B/EDN1	2
BP	GO:0033599	regulation of mammary gland epithelial cell proliferation	2/187	17/18866	0.012052083	0.026492366	0.012023826	BAX/CCND1	2
BP	GO:0045722	positive regulation of	2/187	17/18866	0.012052083	0.026492366	0.012023826	PPARA/GCG	2
BP	GO:0045986	negative regulation of smooth muscle contraction	2/187	17/18866	0.012052083	0.026492366	0.012023826	PTGS2/SOD1	2
BP	GO:0055089	fatty acid homeostasis	2/187	17/18866	0.012052083	0.026492366	0.012023826	GOT1/INS	2
BP	GO:0060263	regulation of respiratory burst	2/187	17/18866	0.012052083	0.026492366	0.012023826	LBP/INS	2
BP	GO:0060644	mammary gland epithelial cell differentiation	2/187	17/18866	0.012052083	0.026492366	0.012023826	AKT1/HIF1A	2
BP	GO:0070293	renal absorption	2/187	17/18866	0.012052083	0.026492366	0.012023826	AKR1C3/CLDN4	2
BP	GO:0071850	mitotic cell cycle arrest	2/187	17/18866	0.012052083	0.026492366	0.012023826	CDKN1A/TP53	2
BP	GO:0090185	negative regulation of kidney development	2/187	17/18866	0.012052083	0.026492366	0.012023826	STAT1/MMP9	2
BP	GO:1904355	positive regulation of telomere capping	2/187	17/18866	0.012052083	0.026492366	0.012023826	MAPK1/MAPK3	2
BP	GO:0006638	neutral lipid metabolic process	5/187	138/18866	0.01219074	0.026756057	0.012143505	CAT/APOB/CAV1/LPL/FABP1	5
BP	GO:0006639	acylglycerol metabolic process	5/187	138/18866	0.01219074	0.026756057	0.012143505	CAT/APOB/CAV1/LPL/FABP1	5

BP	GO:0046330	positive regulation of JNK cascade	5/187	138/18866	0.01219074	0.026756057	0.012143505	CD40LG/MMP8/NCF1/IL1B/EDN1	5
BP	GO:0006111	regulation of gluconeogenesis	3/187	49/18866	0.012640958	0.027687567	0.01256628	PPARA/INS/GCG	3
BP	GO:0010712	regulation of collagen metabolic process	3/187	49/18866	0.012640958	0.027687567	0.01256628	PPARG/PPARD/GOT1	3
BP	GO:0072348	sulfur compound transport	3/187	49/18866	0.012640958	0.027687567	0.01256628	ABCC1/GJA1/ABCG2	3
BP	GO:0120178	steroid hormone biosynthetic process	3/187	49/18866	0.012640958	0.027687567	0.01256628	AKR1B1/CYP19A1/ADM	3
BP	GO:0002833	positive regulation of response to biotic stimulus	7/187	251/18866	0.01275714	0.027927794	0.012675309	LBP/PRKCA/RELA/IKBKB/MAPK3/RAF1/CHU	7
BP	GO:0070588	calcium ion transmembrane transport	8/187	312/18866	0.01279501	0.027958155	0.012689089	OPRM1/BAX/ADRA2A/CXCL11/CXCL10/DRD2/TRPV1/CACNA1S	8
BP	GO:0001656	metanephros development	4/187	91/18866	0.012797059	0.027958155	0.012689089	BCL2/STAT1/AKR1B1/MYC	4
BP	GO:0006094	gluconeogenesis	4/187	91/18866	0.012797059	0.027958155	0.012689089	PPARA/GOT1/INS/GCG	4
BP	GO:1904063	negative regulation of cation transmembrane transport	4/187	91/18866	0.012797059	0.027958155	0.012689089	ADRA2A/MMP9/CAV1/DRD2	4
BP	GO:0007266	Rho protein signal transduction	5/187	140/18866	0.012909961	0.028161821	0.012781525	ADRA2A/MET/RAF1/CHUK/RASGRF1	5
BP	GO:0009308	amine metabolic process	5/187	140/18866	0.012909961	0.028161821	0.012781525	CYP1A1/VCAM1/ODC1/NQO1/INS	5
BP	GO:0097553	calcium ion transmembrane import into cytosol	5/187	140/18866	0.012909961	0.028161821	0.012781525	BAX/CXCL11/CXCL10/DRD2/TRPV1	5
BP	GO:0046390	ribose phosphate biosynthetic process	6/187	194/18866	0.012959675	0.02825591	0.012824228	IL4/FASN/STAT3/ACACA/PARP1/SCD	6
BP	GO:0050864	regulation of B cell activation	6/187	195/18866	0.013264369	0.028905552	0.013119074	BCL2/CASP3/AHR/CDKN1A/IL4/BAD	6
BP	GO:0042987	amyloid precursor protein catabolic process	3/187	50/18866	0.013351569	0.029051335	0.01318524	CASP3/RELA/IFNG	3
BP	GO:0048546	digestive tract morphogenesis	3/187	50/18866	0.013351569	0.029051335	0.01318524	BCL2/EGFR/HIF1A	3
BP	GO:0050873	brown fat cell differentiation	3/187	50/18866	0.013351569	0.029051335	0.01318524	PTGS2/MAPK14/INS	3
BP	GO:0010832	negative regulation of myotube differentiation	2/187	18/18866	0.013470999	0.029148689	0.013229425	CXCL10/BDNF	2
BP	GO:0042136	neurotransmitter biosynthetic	2/187	18/18866	0.013470999	0.029148689	0.013229425	SLC6A4/ACHE	2
BP	GO:0043217	myelin maintenance	2/187	18/18866	0.013470999	0.029148689	0.013229425	AKT1/SOD1	2
BP	GO:0046885	regulation of hormone biosynthetic process	2/187	18/18866	0.013470999	0.029148689	0.013229425	HIF1A/ADM	2
BP	GO:0061298	retina vasculature development in camera-type eye	2/187	18/18866	0.013470999	0.029148689	0.013229425	CYP1B1/HIF1A	2
BP	GO:0070230	positive regulation of lymphocyte apoptotic process	2/187	18/18866	0.013470999	0.029148689	0.013229425	BAX/TP53	2

BP	GO:0071605	monocyte chemotactic protein-1 production	2/187	18/18866	0.013470999	0.029148689	0.013229425	GSTP1/IL1B	2
BP	GO:0071637	regulation of monocyte chemotactic protein-1 production	2/187	18/18866	0.013470999	0.029148689	0.013229425	GSTP1/IL1B	2
BP	GO:0150078	positive regulation of neuroinflammatory response	2/187	18/18866	0.013470999	0.029148689	0.013229425	MMP8/IL1B	2
BP	GO:1902074	response to salt	2/187	18/18866	0.013470999	0.029148689	0.013229425	HSPA5/EDN1	2
BP	GO:2000641	regulation of early endosome to late endosome transport	2/187	18/18866	0.013470999	0.029148689	0.013229425	MAPK1/MAPK3	2
BP	GO:0002040	sprouting angiogenesis	6/187	196/18866	0.01357405	0.029342092	0.013317202	PTGS2/AKT1/HMOX1/ALOX5/VEGFA/KDR	6
BP	GO:0009566	fertilization	6/187	196/18866	0.01357405	0.029342092	0.013317202	BAX/AR/CDK1/BCL2L1/APOB/PLB1	6
BP	GO:0032088	negative regulation of NF-kappaB transcription factor activity	4/187	93/18866	0.013769691	0.029735051	0.013495551	CYP1B1/CAT/NFKBIA/CHUK	4
BP	GO:0072080	nephron tubule development	4/187	93/18866	0.013769691	0.029735051	0.013495551	BCL2/STAT1/VEGFA/MYC	4
BP	GO:0032873	negative regulation of stress-activated MAPK cascade	3/187	51/18866	0.01408455	0.030338672	0.01376951	AKT1/GSTP1/MYC	3
BP	GO:0038084	vascular endothelial growth factor signaling pathway	3/187	51/18866	0.01408455	0.030338672	0.01376951	VEGFA/KDR/HSPB1	3
BP	GO:0050435	amyloid-beta metabolic process	3/187	51/18866	0.01408455	0.030338672	0.01376951	CASP3/RELA/IFNG	3
BP	GO:0070303	negative regulation of stress-activated protein kinase signaling	3/187	51/18866	0.01408455	0.030338672	0.01376951	AKT1/GSTP1/MYC	3
BP	GO:1903573	negative regulation of response to endoplasmic reticulum stress	3/187	51/18866	0.01408455	0.030338672	0.01376951	ALOX5/BCL2L1/HSPA5	3
BP	GO:0030641	regulation of cellular pH	4/187	94/18866	0.01427313	0.030714058	0.013939883	BCL2/MAPK1/CA2/MAPK3	4
BP	GO:0060993	kidney morphogenesis	4/187	94/18866	0.01427313	0.030714058	0.013939883	BCL2/STAT1/VEGFA/MYC	4
BP	GO:1902850	microtubule cytoskeleton organization involved in mitosis	5/187	144/18866	0.014433422	0.031043427	0.01408937	BIRC5/CCNB1/XIAP/GJA1/CHEK2	5
BP	GO:0003007	heart morphogenesis	7/187	258/18866	0.014642991	0.031478398	0.014286786	JUN/RXRA/VEGFA/TP53/MDM2/HIF1A/NOS3	7
BP	GO:0061326	renal tubule development	4/187	95/18866	0.014788096	0.031681016	0.014378746	BCL2/STAT1/VEGFA/MYC	4
BP	GO:0003179	heart valve morphogenesis	3/187	52/18866	0.014839988	0.031681016	0.014378746	RB1/MDM2/NOS3	3
BP	GO:0031103	axon regeneration	3/187	52/18866	0.014839988	0.031681016	0.014378746	BCL2/JUN/SPP1	3
BP	GO:0051339	regulation of lyase activity	3/187	52/18866	0.014839988	0.031681016	0.014378746	NOS2/NOS3/DRD2	3
BP	GO:0072132	mesenchyme morphogenesis	3/187	52/18866	0.014839988	0.031681016	0.014378746	MDM2/MYC/NOS3	3
BP	GO:0090329	regulation of DNA-dependent DNA replication	3/187	52/18866	0.014839988	0.031681016	0.014378746	CDK2/PCNA/CHEK2	3

BP	GO:1903557	positive regulation of tumor necrosis factor superfamily cytokine	3/187	52/18866	0.014839988	0.031681016	0.014378746	CD14/LBP/IFNG	3
BP	GO:2000677	regulation of transcription regulatory region DNA binding	3/187	52/18866	0.014839988	0.031681016	0.014378746	RB1/IFNG/PARP1	3
BP	GO:2001258	negative regulation of cation channel activity	3/187	52/18866	0.014839988	0.031681016	0.014378746	MMP9/CAV1/DRD2	3
BP	GO:0010389	regulation of G2/M transition of mitotic cell cycle	6/187	200/18866	0.014863419	0.031681016	0.014378746	CDK1/CDK2/CCND1/CDKN1A/CDK4/CCNB1	6
BP	GO:0021537	telencephalon development	7/187	259/18866	0.0149278	0.031681016	0.014378746	BAX/CASP3/GSK3B/EGFR/BAD/HIF1A/DRD2	7
BP	GO:0001696	gastric acid secretion	2/187	19/18866	0.014958635	0.031681016	0.014378746	PTGER3/TRPV1	2
BP	GO:0001991	regulation of systemic arterial blood pressure by circulatory renin-angiotensin	2/187	19/18866	0.014958635	0.031681016	0.014378746	CES1/ENPEP	2
BP	GO:0002076	osteoblast development	2/187	19/18866	0.014958635	0.031681016	0.014378746	ACHE/RUNX2	2
BP	GO:0010544	negative regulation of platelet activation	2/187	19/18866	0.014958635	0.031681016	0.014378746	NOS3/THBD	2
BP	GO:0010663	positive regulation of striated muscle cell apoptotic process	2/187	19/18866	0.014958635	0.031681016	0.014378746	TP53/HMGCR	2
BP	GO:0010666	positive regulation of cardiac muscle cell apoptotic process	2/187	19/18866	0.014958635	0.031681016	0.014378746	TP53/HMGCR	2
BP	GO:0010744	positive regulation of macrophage derived foam cell differentiation	2/187	19/18866	0.014958635	0.031681016	0.014378746	APOB/LPL	2
BP	GO:0032095	regulation of response to food	2/187	19/18866	0.014958635	0.031681016	0.014378746	OPRM1/PPARA	2
BP	GO:0034393	positive regulation of smooth muscle cell apoptotic process	2/187	19/18866	0.014958635	0.031681016	0.014378746	PPARG/IFNG	2
BP	GO:0044320	cellular response to leptin stimulus	2/187	19/18866	0.014958635	0.031681016	0.014378746	CCNA2/STAT3	2
BP	GO:0045091	regulation of single stranded viral RNA replication via double stranded DNA intermediate	2/187	19/18866	0.014958635	0.031681016	0.014378746	TOP2A/CXCL8	2
BP	GO:0051546	keratinocyte migration	2/187	19/18866	0.014958635	0.031681016	0.014378746	MMP9/PPARD	2
BP	GO:0060252	positive regulation of glial cell proliferation	2/187	19/18866	0.014958635	0.031681016	0.014378746	IL1B/E2F1	2
BP	GO:0060438	trachea development	2/187	19/18866	0.014958635	0.031681016	0.014378746	MAPK1/MAPK3	2
BP	GO:0070989	oxidative demethylation	2/187	19/18866	0.014958635	0.031681016	0.014378746	CYP3A4/CYP1A2	2
BP	GO:0072074	kidney mesenchyme development	2/187	19/18866	0.014958635	0.031681016	0.014378746	STAT1/MYC	2
BP	GO:0090026	positive regulation of monocyte chemotaxis	2/187	19/18866	0.014958635	0.031681016	0.014378746	SERPINE1/CXCL10	2
BP	GO:2000647	negative regulation of stem cell proliferation	2/187	19/18866	0.014958635	0.031681016	0.014378746	TP53/BDNF	2

BP	GO:2000696	regulation of epithelial cell differentiation involved in kidney development	2/187	19/18866	0.014958635	0.031681016	0.014378746	STAT1/MMP9	2
BP	GO:0035249	synaptic transmission, glutamatergic	4/187	96/18866	0.01531467	0.032419073	0.014713721	PTGS2/EGFR/CCL2/DR	4
BP	GO:1901605	alpha-amino acid metabolic process	6/187	202/18866	0.015539029	0.032877797	0.014921918	NOS2/DPEP1/TYR/GOT1/ODC1/NOS3	6
BP	GO:0035384	thioester biosynthetic process	3/187	53/18866	0.01561796	0.033012261	0.014982945	FASN/ACACA/SCD	3
BP	GO:0071616	acyl-CoA biosynthetic process	3/187	53/18866	0.01561796	0.033012261	0.014982945	FASN/ACACA/SCD	3
BP	GO:1904064	positive regulation of cation transmembrane transport	5/187	147/18866	0.015652189	0.03306833	0.015008393	BAX/IFNG/CCL2/CXCL11/CXCL10	5
BP	GO:0006476	protein deacetylation	4/187	97/18866	0.015852932	0.033475964	0.015193402	MAPK8/VEGFA/TP53/IFNG	4
BP	GO:1903670	regulation of sprouting angiogenesis	5/187	148/18866	0.016073245	0.033924504	0.015396976	PTGS2/HMOX1/ALOX5/VEGFA/KDR	5
BP	GO:0060541	respiratory system development	6/187	204/18866	0.016235658	0.034250458	0.015544913	CYP1A2/EGFR/VEGFA/MAPK1/MAPK3/NOS3	6
BP	GO:0002832	negative regulation of response to biotic stimulus	4/187	98/18866	0.016402956	0.034514347	0.015664682	PPARG/IL2RA/DRD2/INS	4
BP	GO:0006754	ATP biosynthetic process	3/187	54/18866	0.016418533	0.034514347	0.015664682	IL4/STAT3/PARP1	3
BP	GO:0006968	cellular defense response	3/187	54/18866	0.016418533	0.034514347	0.015664682	LBP/RELA/NCF1	3
BP	GO:1901185	negative regulation of ERBB signaling pathway	3/187	54/18866	0.016418533	0.034514347	0.015664682	EGFR/ERBB2/EGF	3
BP	GO:0003323	type B pancreatic cell development	2/187	20/18866	0.016513503	0.034514347	0.015664682	GSK3B/BAD	2
BP	GO:0006525	arginine metabolic process	2/187	20/18866	0.016513503	0.034514347	0.015664682	NOS2/NOS3	2
BP	GO:0010042	response to manganese ion	2/187	20/18866	0.016513503	0.034514347	0.015664682	PTGS2/HSPA5	2
BP	GO:0015669	gas transport	2/187	20/18866	0.016513503	0.034514347	0.015664682	CA2/MYC	2
BP	GO:0032303	regulation of icosanoid secretion	2/187	20/18866	0.016513503	0.034514347	0.015664682	IL1B/EDN1	2
BP	GO:0032332	positive regulation of chondrocyte differentiation	2/187	20/18866	0.016513503	0.034514347	0.015664682	RELA/RUNX2	2
BP	GO:0032727	positive regulation of interferon-alpha production	2/187	20/18866	0.016513503	0.034514347	0.015664682	STAT1/CHUK	2
BP	GO:0034138	toll-like receptor 3 signaling	2/187	20/18866	0.016513503	0.034514347	0.015664682	CASP8/CAV1	2
BP	GO:0039692	single stranded viral RNA replication via double stranded DNA	2/187	20/18866	0.016513503	0.034514347	0.015664682	TOP2A/CXCL8	2
BP	GO:0048670	regulation of collateral sprouting	2/187	20/18866	0.016513503	0.034514347	0.015664682	SPP1/BDNF	2
BP	GO:0060546	negative regulation of necroptotic process	2/187	20/18866	0.016513503	0.034514347	0.015664682	CASP8/CAV1	2
BP	GO:0062099	negative regulation of programmed necrotic cell death	2/187	20/18866	0.016513503	0.034514347	0.015664682	CASP8/CAV1	2
BP	GO:0097709	connective tissue replacement	2/187	20/18866	0.016513503	0.034514347	0.015664682	HIF1A/IL1A	2
BP	GO:1901741	positive regulation of myoblast	2/187	20/18866	0.016513503	0.034514347	0.015664682	MAPK14/IL4	2

BP	GO:2000193	positive regulation of fatty acid transport	2/187	20/18866	0.016513503	0.034514347	0.015664682	IL1B/EDN1	2
BP	GO:0090263	positive regulation of canonical Wnt signaling pathway	5/187	150/18866	0.016937874	0.035384083	0.016059421	EGFR/XIAP/EGF/CAV1/COL1A1	5
BP	GO:0043473	pigmentation	4/187	99/18866	0.016964817	0.035423132	0.016077143	BCL2/BAX/TYR/DRD2	4
BP	GO:0001706	endoderm formation	3/187	55/18866	0.017241766	0.035879255	0.016284159	MMP2/MMP9/MMP8	3
BP	GO:0001954	positive regulation of cell-matrix adhesion	3/187	55/18866	0.017241766	0.035879255	0.016284159	GSK3B/VEGFA/KDR	3
BP	GO:0030199	collagen fibril organization	3/187	55/18866	0.017241766	0.035879255	0.016284159	CYP1B1/RB1/COL1A1	3
BP	GO:0032655	regulation of interleukin-12	3/187	55/18866	0.017241766	0.035879255	0.016284159	MAPK14/IFNG/CD40LG	3
BP	GO:0035065	regulation of histone acetylation	3/187	55/18866	0.017241766	0.035879255	0.016284159	CHEK1/MAPK3/IL1B	3
BP	GO:0045620	negative regulation of lymphocyte differentiation	3/187	55/18866	0.017241766	0.035879255	0.016284159	ERBB2/IL4/IRF1	3
BP	GO:0060350	endochondral bone morphogenesis	3/187	55/18866	0.017241766	0.035879255	0.016284159	MMP13/COL1A1/RUNX	3
BP	GO:0045069	regulation of viral genome replication	4/187	100/18866	0.017538586	0.036479239	0.016556468	BCL2/SLPI/TOP2A/CXCL8	4
BP	GO:0007605	sensory perception of sound	5/187	152/18866	0.017832864	0.037055414	0.016817971	CASP3/ICAM1/BIRC5/SOD1/COL1A1	5
BP	GO:1903169	regulation of calcium ion transmembrane transport	5/187	152/18866	0.017832864	0.037055414	0.016817971	BAX/ADRA2A/CXCL11/CXCL10/DRD2	5
BP	GO:0010665	regulation of cardiac muscle cell apoptotic process	3/187	56/18866	0.018087708	0.037302282	0.016930015	TP53/HMGCR/NFE2L2	3
BP	GO:0019320	hexose catabolic process	3/187	56/18866	0.018087708	0.037302282	0.016930015	TP53/BAD/HK2	3
BP	GO:0070228	regulation of lymphocyte apoptotic process	3/187	56/18866	0.018087708	0.037302282	0.016930015	BAX/TP53/HIF1A	3
BP	GO:0007215	glutamate receptor signaling pathway	4/187	101/18866	0.018124332	0.037302282	0.016930015	OPRM1/IFNG/CCL2/RASGRF1	4
BP	GO:0044264	cellular polysaccharide metabolic process	4/187	101/18866	0.018124332	0.037302282	0.016930015	AKT1/GSK3B/IGF2/INS	4
BP	GO:0002029	desensitization of G protein-coupled receptor signaling pathway	2/187	21/18866	0.01813414	0.037302282	0.016930015	DRD2/ADM	2
BP	GO:0002363	alpha-beta T cell lineage	2/187	21/18866	0.01813414	0.037302282	0.016930015	BCL2/STAT3	2
BP	GO:0002693	positive regulation of cellular extravasation	2/187	21/18866	0.01813414	0.037302282	0.016930015	ICAM1/SELE	2
BP	GO:0010310	regulation of hydrogen peroxide metabolic process	2/187	21/18866	0.01813414	0.037302282	0.016930015	STAT3/MMP3	2
BP	GO:0010829	negative regulation of glucose transmembrane transport	2/187	21/18866	0.01813414	0.037302282	0.016930015	IL1B/PRKCB	2
BP	GO:0022401	negative adaptation of signaling pathway	2/187	21/18866	0.01813414	0.037302282	0.016930015	DRD2/ADM	2
BP	GO:0030220	platelet formation	2/187	21/18866	0.01813414	0.037302282	0.016930015	CASP9/CASP3	2

BP	GO:0035357	peroxisome proliferator activated receptor signaling pathway	2/187	21/18866	0.01813414	0.037302282	0.016930015	PPARG/RXRA	2
BP	GO:0035813	regulation of renal sodium excretion	2/187	21/18866	0.01813414	0.037302282	0.016930015	DRD2/EDN1	2
BP	GO:0046716	muscle cell cellular homeostasis	2/187	21/18866	0.01813414	0.037302282	0.016930015	SOD1/HIF1A	2
BP	GO:0060065	uterus development	2/187	21/18866	0.01813414	0.037302282	0.016930015	ESR1/CYP19A1	2
BP	GO:0060716	labyrinthine layer blood vessel development	2/187	21/18866	0.01813414	0.037302282	0.016930015	AKT1/MAPK1	2
BP	GO:0090201	negative regulation of release of cytochrome c from mitochondria	2/187	21/18866	0.01813414	0.037302282	0.016930015	AKT1/BCL2L1	2
BP	GO:0090280	positive regulation of calcium ion import	2/187	21/18866	0.01813414	0.037302282	0.016930015	CCL2/GCG	2
BP	GO:1903599	positive regulation of autophagy of mitochondrion	2/187	21/18866	0.01813414	0.037302282	0.016930015	HIF1A/HK2	2
BP	GO:1904886	beta-catenin destruction complex disassembly	2/187	21/18866	0.01813414	0.037302282	0.016930015	GSK3B/CAV1	2
BP	GO:0010256	endomembrane system organization	10/187	468/18866	0.018562727	0.038165607	0.017321843	PRKCA/AR/AKT1/CDK1/MAPK1/CCNB1/MAPK3/SOD1/CAV1/PRKCB	10
BP	GO:0051250	negative regulation of lymphocyte activation	5/187	154/18866	0.018758613	0.038549893	0.017496255	CASP3/ERBB2/IL2RA/IL4/IRF1	5
BP	GO:0032615	interleukin-12 production	3/187	57/18866	0.0189564	0.038937716	0.017672272	MAPK14/IFNG/CD40LG	3
BP	GO:1901890	positive regulation of cell junction assembly	4/187	103/18866	0.019332021	0.039690275	0.018013829	VEGFA/KDR/CAV1/BDNF	4
BP	GO:0030902	hindbrain development	5/187	156/18866	0.019715505	0.040458251	0.018362383	SLC6A4/BCL2/TP53/EGF/HSPA5	5
BP	GO:0009235	cobalamin metabolic process	2/187	22/18866	0.019819104	0.040477361	0.018371056	PRSS1/ABCC1	2
BP	GO:0023058	adaptation of signaling pathway	2/187	22/18866	0.019819104	0.040477361	0.018371056	DRD2/ADM	2
BP	GO:0032069	regulation of nuclease activity	2/187	22/18866	0.019819104	0.040477361	0.018371056	AKT1/PCNA	2
BP	GO:0035584	calcium-mediated signaling using intracellular calcium source	2/187	22/18866	0.019819104	0.040477361	0.018371056	VCAM1/KDR	2
BP	GO:0036344	platelet morphogenesis	2/187	22/18866	0.019819104	0.040477361	0.018371056	CASP9/CASP3	2
BP	GO:0043369	CD4-positive or CD8-positive, alpha-beta T cell lineage	2/187	22/18866	0.019819104	0.040477361	0.018371056	BCL2/STAT3	2
BP	GO:0044342	type B pancreatic cell proliferation	2/187	22/18866	0.019819104	0.040477361	0.018371056	BAD/IGFBP3	2
BP	GO:0045655	regulation of monocyte	2/187	22/18866	0.019819104	0.040477361	0.018371056	JUN/MYC	2
BP	GO:0045663	positive regulation of myoblast differentiation	2/187	22/18866	0.019819104	0.040477361	0.018371056	MAPK14/IGFBP3	2
BP	GO:0071459	protein localization to chromosome, centromeric region	2/187	22/18866	0.019819104	0.040477361	0.018371056	CDK1/RB1	2
BP	GO:0000768	syncytium formation by plasma membrane fusion	3/187	58/18866	0.019847874	0.040478348	0.018371503	MAPK14/IL4/CXCL10	3

BP	GO:0010662	regulation of striated muscle cell apoptotic process	3/187	58/18866	0.019847874	0.040478348	0.018371503	TP53/HMGCR/NFE2L2	3
BP	GO:0140253	cell-cell fusion	3/187	58/18866	0.019847874	0.040478348	0.018371503	MAPK14/IL4/CXCL10	3
BP	GO:0003300	cardiac muscle hypertrophy	4/187	104/18866	0.019954089	0.040675643	0.018461048	PRKCA/PPARA/PARP1/EDN1	4
BP	GO:0006520	cellular amino acid metabolic process	8/187	340/18866	0.020362689	0.041488858	0.018830134	NOS2/DPEP1/TYR/GOT1/ODC1/NOS3/NQO1/IN	8
BP	GO:1902106	negative regulation of leukocyte differentiation	4/187	105/18866	0.020588387	0.041908935	0.01902079	ERBB2/IL4/MYC/IRF1	4
BP	GO:2001022	positive regulation of response to DNA damage stimulus	4/187	105/18866	0.020588387	0.041908935	0.01902079	EGFR/PCNA/MYC/PARP1	4
BP	GO:0001658	branching involved in ureteric bud morphogenesis	3/187	59/18866	0.020762153	0.042182641	0.019145014	BCL2/VEGFA/MYC	3
BP	GO:0010659	cardiac muscle cell apoptotic	3/187	59/18866	0.020762153	0.042182641	0.019145014	TP53/HMGCR/NFE2L2	3
BP	GO:0043030	regulation of macrophage activation	3/187	59/18866	0.020762153	0.042182641	0.019145014	LBP/IL4/MMP8	3
BP	GO:0098930	axonal transport	3/187	59/18866	0.020762153	0.042182641	0.019145014	SOD1/HIF1A/HSPB1	3
BP	GO:0050792	regulation of viral process	6/187	217/18866	0.021295951	0.043246698	0.019627947	BCL2/JUN/STAT1/SLPI/TOP2A/CXCL8	6
BP	GO:0000002	mitochondrial genome maintenance	2/187	23/18866	0.021566974	0.043406948	0.019700678	TP53/PARP1	2
BP	GO:0006582	melanin metabolic process	2/187	23/18866	0.021566974	0.043406948	0.019700678	BCL2/TYR	2
BP	GO:0031639	plasminogen activation	2/187	23/18866	0.021566974	0.043406948	0.019700678	PLAU/SERPINE1	2
BP	GO:0032098	regulation of appetite	2/187	23/18866	0.021566974	0.043406948	0.019700678	OPRM1/PPARA	2
BP	GO:0035458	cellular response to interferon-beta	2/187	23/18866	0.021566974	0.043406948	0.019700678	STAT1/IRF1	2
BP	GO:0035743	CD4-positive, alpha-beta T cell cytokine production	2/187	23/18866	0.021566974	0.043406948	0.019700678	IL4/IL1B	2
BP	GO:0035812	renal sodium excretion	2/187	23/18866	0.021566974	0.043406948	0.019700678	DRD2/EDN1	2
BP	GO:0043586	tongue development	2/187	23/18866	0.021566974	0.043406948	0.019700678	EGFR/BDNF	2
BP	GO:0045723	positive regulation of fatty acid biosynthetic process	2/187	23/18866	0.021566974	0.043406948	0.019700678	PTGS2/IL1B	2
BP	GO:0060547	negative regulation of necrotic cell death	2/187	23/18866	0.021566974	0.043406948	0.019700678	CASP8/CAV1	2
BP	GO:0070584	mitochondrion morphogenesis	2/187	23/18866	0.021566974	0.043406948	0.019700678	BAX/BCL2L1	2
BP	GO:0072215	regulation of metanephros development	2/187	23/18866	0.021566974	0.043406948	0.019700678	STAT1/MYC	2
BP	GO:1900273	positive regulation of long-term synaptic potentiation	2/187	23/18866	0.021566974	0.043406948	0.019700678	DRD2/INS	2
BP	GO:1901032	negative regulation of response to reactive oxygen species	2/187	23/18866	0.021566974	0.043406948	0.019700678	MET/NFE2L2	2
BP	GO:1902001	fatty acid transmembrane transport	2/187	23/18866	0.021566974	0.043406948	0.019700678	AKT1/ACACA	2
BP	GO:1902254	negative regulation of intrinsic apoptotic signaling pathway by p53 class mediator	2/187	23/18866	0.021566974	0.043406948	0.019700678	BCL2/MDM2	2

BP	GO:1903206	negative regulation of hydrogen peroxide-induced cell death	2/187	23/18866	0.021566974	0.043406948	0.019700678	MET/NFE2L2	2
BP	GO:1903589	positive regulation of blood vessel endothelial cell proliferation involved in sprouting angiogenesis	2/187	23/18866	0.021566974	0.043406948	0.019700678	HMOX1/VEGFA	2
BP	GO:2000810	regulation of bicellular tight junction assembly	2/187	23/18866	0.021566974	0.043406948	0.019700678	IKBKB/GJA1	2
BP	GO:0006949	syncytium formation	3/187	60/18866	0.021699253	0.043632268	0.019802942	MAPK14/IL4/CXCL10	3
BP	GO:0010823	negative regulation of mitochondrion organization	3/187	60/18866	0.021699253	0.043632268	0.019802942	AKT1/BCL2L1/TP53	3
BP	GO:0014897	striated muscle hypertrophy	4/187	107/18866	0.021893897	0.043961881	0.01995254	PRKCA/PPARA/PARP1/EDN1	4
BP	GO:0035601	protein deacylation	4/187	107/18866	0.021893897	0.043961881	0.01995254	MAPK8/VEGFA/TP53/IFNG	4
BP	GO:0043266	regulation of potassium ion transport	4/187	107/18866	0.021893897	0.043961881	0.01995254	ADRA2A/CAV1/NOS3/DRD2	4
BP	GO:0072659	protein localization to plasma membrane	7/187	281/18866	0.022236129	0.04460932	0.020246387	AR/IKBKB/AKT1/EGFR/BCL2L1/IFNG/INS	7
BP	GO:0007409	axonogenesis	10/187	482/18866	0.022237118	0.04460932	0.020246387	BCL2/PRKCA/GSK3B/VEGFA/MAPK1/ERBB2/MAPK3/SPP1/DRD2/BDNF	10
BP	GO:0003170	heart valve development	3/187	61/18866	0.022659182	0.045328923	0.020572986	RB1/MDM2/NOS3	3
BP	GO:0010658	striated muscle cell apoptotic	3/187	61/18866	0.022659182	0.045328923	0.020572986	TP53/HMGCR/NFE2L2	3
BP	GO:0031343	positive regulation of cell killing	3/187	61/18866	0.022659182	0.045328923	0.020572986	NOS2/IFNG/BAD	3
BP	GO:1903078	positive regulation of protein localization to plasma membrane	3/187	61/18866	0.022659182	0.045328923	0.020572986	AKT1/EGFR/IFNG	3
BP	GO:1904356	regulation of telomere maintenance via telomere lengthening	3/187	61/18866	0.022659182	0.045328923	0.020572986	MAPK1/MAPK3/PARP1	3
BP	GO:2000756	regulation of peptidyl-lysine acetylation	3/187	61/18866	0.022659182	0.045328923	0.020572986	CHEK1/MAPK3/IL1B	3
BP	GO:0014896	muscle hypertrophy	4/187	109/18866	0.023248982	0.046417521	0.021067057	PRKCA/PPARA/PARP1/EDN1	4
BP	GO:0021761	limbic system development	4/187	109/18866	0.023248982	0.046417521	0.021067057	BAX/CASP3/GSK3B/DRD2	4
BP	GO:0002438	acute inflammatory response to antigenic stimulus	2/187	24/18866	0.023376352	0.046417521	0.021067057	OPRM1/ICAM1	2
BP	GO:0007095	mitotic G2 DNA damage checkpoint	2/187	24/18866	0.023376352	0.046417521	0.021067057	CDK1/CDKN1A	2
BP	GO:0032469	endoplasmic reticulum calcium ion homeostasis	2/187	24/18866	0.023376352	0.046417521	0.021067057	BCL2/BAX	2
BP	GO:0034123	positive regulation of toll-like receptor signaling pathway	2/187	24/18866	0.023376352	0.046417521	0.021067057	LBP/CAV1	2
BP	GO:0034505	tooth mineralization	2/187	24/18866	0.023376352	0.046417521	0.021067057	PPARA/COL1A1	2

BP	GO:0044062	regulation of excretion	2/187	24/18866	0.023376352	0.046417521	0.021067057	DRD2/EDN1	2
BP	GO:0044346	fibroblast apoptotic process	2/187	24/18866	0.023376352	0.046417521	0.021067057	TP53/MYC	2
BP	GO:0044550	secondary metabolite biosynthetic process	2/187	24/18866	0.023376352	0.046417521	0.021067057	CYP1A2/TYR	2
BP	GO:0046628	positive regulation of insulin receptor signaling pathway	2/187	24/18866	0.023376352	0.046417521	0.021067057	IGF2/INS	2
BP	GO:0051349	positive regulation of lyase activity	2/187	24/18866	0.023376352	0.046417521	0.021067057	NOS2/NOS3	2
BP	GO:0060444	branching involved in mammary gland duct morphogenesis	2/187	24/18866	0.023376352	0.046417521	0.021067057	AR/ESR1	2
BP	GO:0086064	cell communication by electrical coupling involved in cardiac conduction	2/187	24/18866	0.023376352	0.046417521	0.021067057	CAV1/GJA1	2
BP	GO:1900118	negative regulation of execution phase of apoptosis	2/187	24/18866	0.023376352	0.046417521	0.021067057	BCL2L1/GCG	2
BP	GO:1903306	negative regulation of regulated secretory pathway	2/187	24/18866	0.023376352	0.046417521	0.021067057	HMOX1/ADRA2A	2
BP	GO:0002294	CD4-positive, alpha-beta T cell differentiation involved in immune response	3/187	62/18866	0.02364194	0.046879838	0.021276884	IFNG/IL4/STAT3	3
BP	GO:0045824	negative regulation of innate immune response	3/187	62/18866	0.02364194	0.046879838	0.021276884	PPARG/DRD2/INS	3
BP	GO:0140115	export across plasma membrane	3/187	62/18866	0.02364194	0.046879838	0.021276884	ABCC1/GJA1/ABCG2	3
BP	GO:0072009	nephron epithelium development	4/187	110/18866	0.023945238	0.047458975	0.021539731	BCL2/STAT1/VEGFA/MYC	4
BP	GO:0002699	positive regulation of immune effector process	6/187	223/18866	0.023956114	0.047458975	0.021539731	LBP/NOS2/HMOX1/IFNG/IL4/IL1B	6
BP	GO:0002287	alpha-beta T cell activation involved in immune response	3/187	63/18866	0.02464752	0.048661957	0.022085717	IFNG/IL4/STAT3	3
BP	GO:0002293	alpha-beta T cell differentiation involved in immune response	3/187	63/18866	0.02464752	0.048661957	0.022085717	IFNG/IL4/STAT3	3
BP	GO:0006521	regulation of cellular amino acid metabolic process	3/187	63/18866	0.02464752	0.048661957	0.022085717	ODC1/NQO1/INS	3
BP	GO:0042303	molting cycle	4/187	111/18866	0.024654031	0.048661957	0.022085717	PTGS2/BCL2/RELA/EGF	4
BP	GO:0042633	hair cycle	4/187	111/18866	0.024654031	0.048661957	0.022085717	PTGS2/BCL2/RELA/EGF	4
BP	GO:0061387	regulation of extent of cell growth	4/187	111/18866	0.024654031	0.048661957	0.022085717	GSK3B/VEGFA/SPP1/BDNF	4
BP	GO:0098732	macromolecule deacylation	4/187	111/18866	0.024654031	0.048661957	0.022085717	MAPK8/VEGFA/TP53/IFNG	4
BP	GO:1903008	organelle disassembly	4/187	111/18866	0.024654031	0.048661957	0.022085717	CDK1/TP53/HIF1A/HK2	4
BP	GO:1903050	regulation of proteolysis involved in cellular protein catabolic process	6/187	225/18866	0.024890172	0.049105472	0.02228701	AKT1/GSK3B/MDM2/EGF/CAV1/NFE2L2	6
BP	GO:0003309	type B pancreatic cell differentiation	2/187	25/18866	0.025245862	0.049488805	0.02246099	GSK3B/BAD	2

BP	GO:0019400	alditol metabolic process	2/187	25/18866	0.025245862	0.049488805	0.02246099	AKR1B1/GOT1	2
BP	GO:0032104	regulation of response to extracellular stimulus	2/187	25/18866	0.025245862	0.049488805	0.02246099	OPRM1/PPARA	2
BP	GO:0032107	regulation of response to nutrient levels	2/187	25/18866	0.025245862	0.049488805	0.02246099	OPRM1/PPARA	2
BP	GO:0032647	regulation of interferon-alpha production	2/187	25/18866	0.025245862	0.049488805	0.02246099	STAT1/CHUK	2
BP	GO:0042537	benzene-containing compound metabolic process	2/187	25/18866	0.025245862	0.049488805	0.02246099	GSTM1/UGT1A1	2
BP	GO:0045662	negative regulation of myoblast differentiation	2/187	25/18866	0.025245862	0.049488805	0.02246099	PPARD/CXCL10	2
BP	GO:0050857	positive regulation of antigen receptor-mediated signaling pathway	2/187	25/18866	0.025245862	0.049488805	0.02246099	RELA/PRKCB	2
BP	GO:0051894	positive regulation of focal adhesion assembly	2/187	25/18866	0.025245862	0.049488805	0.02246099	VEGFA/KDR	2
BP	GO:0060259	regulation of feeding behavior	2/187	25/18866	0.025245862	0.049488805	0.02246099	STAT3/INS	2
BP	GO:0060669	embryonic placenta morphogenesis	2/187	25/18866	0.025245862	0.049488805	0.02246099	IGF2/ADM	2
BP	GO:0061050	regulation of cell growth involved in cardiac muscle cell development	2/187	25/18866	0.025245862	0.049488805	0.02246099	PPARA/EDN1	2
BP	GO:0061436	establishment of skin barrier	2/187	25/18866	0.025245862	0.049488805	0.02246099	MET/CLDN4	2
BP	GO:1905564	positive regulation of vascular endothelial cell proliferation	2/187	25/18866	0.025245862	0.049488805	0.02246099	STAT3/IGF2	2
BP	GO:0002437	inflammatory response to antigenic stimulus	3/187	64/18866	0.025675907	0.050240049	0.02280195	OPRM1/ICAM1/IL2RA	3
BP	GO:0032835	glomerulus development	3/187	64/18866	0.025675907	0.050240049	0.02280195	IL6R/BCL2/ENPEP	3
BP	GO:0042130	negative regulation of T cell proliferation	3/187	64/18866	0.025675907	0.050240049	0.02280195	CASP3/ERBB2/IL2RA	3
BP	GO:2000401	regulation of lymphocyte migration	3/187	64/18866	0.025675907	0.050240049	0.02280195	AKT1/CCL2/CXCL10	3
BP	GO:0043161	proteasome-mediated ubiquitin-dependent protein catabolic process	9/187	424/18866	0.025830073	0.05051868	0.022928409	AKT1/CDK1/GSK3B/CDK2/MDM2/CCNB1/HSPA5/CAV1/NFE2L2	9
BP	GO:0002223	stimulatory C-type lectin receptor signaling pathway	4/187	113/18866	0.026109401	0.050995296	0.023144726	RELA/IKBKB/RAF1/CHUK	4
BP	GO:0007009	plasma membrane organization	4/187	113/18866	0.026109401	0.050995296	0.023144726	AR/AKT1/SOD1/CAV1	4
BP	GO:1903555	regulation of tumor necrosis factor superfamily cytokine production	4/187	113/18866	0.026109401	0.050995296	0.023144726	CD14/LBP/GSTP1/IFNG	4
BP	GO:0043588	skin development	9/187	425/18866	0.026171433	0.051093207	0.023189164	BCL2/CASP3/RELA/AKR1C3/EGFR/MET/FOSL2/COL1A1/CLDN4	9
BP	GO:0009206	purine ribonucleoside triphosphate biosynthetic process	3/187	65/18866	0.026727079	0.052035986	0.023617053	IL4/STAT3/PARP1	3
BP	GO:0031640	killing of cells of other organism	3/187	65/18866	0.026727079	0.052035986	0.023617053	NOS2/IFNG/BAD	3

BP	GO:0048247	lymphocyte chemotaxis	3/187	65/18866	0.026727079	0.052035986	0.023617053	CCL2/CXCL11/CXCL10	3
BP	GO:0048645	animal organ formation	3/187	65/18866	0.026727079	0.052035986	0.023617053	AR/MAPK1/MAPK3	3
BP	GO:0060675	ureteric bud morphogenesis	3/187	65/18866	0.026727079	0.052035986	0.023617053	BCL2/VEGFA/MYC	3
BP	GO:0090307	mitotic spindle assembly	3/187	65/18866	0.026727079	0.052035986	0.023617053	BIRC5/XIAP/CHEK2	3
BP	GO:0009062	fatty acid catabolic process	4/187	114/18866	0.026856057	0.052216057	0.023698781	AKT1/PPARD/PPARA/FABP1	4
BP	GO:0030004	cellular monovalent inorganic cation homeostasis	4/187	114/18866	0.026856057	0.052216057	0.023698781	BCL2/MAPK1/CA2/MAPK3	4
BP	GO:0032411	positive regulation of transporter activity	4/187	114/18866	0.026856057	0.052216057	0.023698781	PON1/IFNG/HTR3A/CCL2	4
BP	GO:0002227	innate immune response in mucosa	2/187	26/18866	0.027174145	0.052454409	0.023806959	NOS2/IL4	2
BP	GO:0003081	regulation of systemic arterial blood pressure by renin-angiotensin	2/187	26/18866	0.027174145	0.052454409	0.023806959	CES1/ENPEP	2
BP	GO:0006309	apoptotic DNA fragmentation	2/187	26/18866	0.027174145	0.052454409	0.023806959	BAX/CASP3	2
BP	GO:0006700	C21-steroid hormone biosynthetic process	2/187	26/18866	0.027174145	0.052454409	0.023806959	AKR1B1/ADM	2
BP	GO:0010884	positive regulation of lipid storage	2/187	26/18866	0.027174145	0.052454409	0.023806959	APOB/LPL	2
BP	GO:0018904	ether metabolic process	2/187	26/18866	0.027174145	0.052454409	0.023806959	ALOX5/FASN	2
BP	GO:0032743	positive regulation of interleukin-2 production	2/187	26/18866	0.027174145	0.052454409	0.023806959	IL1B/IL1A	2
BP	GO:0042104	positive regulation of activated T cell proliferation	2/187	26/18866	0.027174145	0.052454409	0.023806959	IL2RA/IGF2	2
BP	GO:0043567	regulation of insulin-like growth factor receptor signaling pathway	2/187	26/18866	0.027174145	0.052454409	0.023806959	AR/IGFBP3	2
BP	GO:0048643	positive regulation of skeletal muscle tissue development	2/187	26/18866	0.027174145	0.052454409	0.023806959	BCL2/HMGCR	2
BP	GO:0051123	RNA polymerase II preinitiation complex assembly	2/187	26/18866	0.027174145	0.052454409	0.023806959	ESR1/TP53	2
BP	GO:0051156	glucose 6-phosphate metabolic process	2/187	26/18866	0.027174145	0.052454409	0.023806959	TP53/HK2	2
BP	GO:0060343	trabecula formation	2/187	26/18866	0.027174145	0.052454409	0.023806959	MMP2/COL1A1	2
BP	GO:0090025	regulation of monocyte chemotaxis	2/187	26/18866	0.027174145	0.052454409	0.023806959	SERPINE1/CXCL10	2
BP	GO:1903649	regulation of cytoplasmic transport	2/187	26/18866	0.027174145	0.052454409	0.023806959	MAPK1/MAPK3	2
BP	GO:1904353	regulation of telomere capping	2/187	26/18866	0.027174145	0.052454409	0.023806959	MAPK1/MAPK3	2
BP	GO:0098657	import into cell	6/187	230/18866	0.027331059	0.05273359	0.023933668	SLC6A4/AKT1/IL10RA/IFNG/DRD2/TRPV1	6
BP	GO:0009145	purine nucleoside triphosphate biosynthetic process	3/187	66/18866	0.027801007	0.053520055	0.024290613	IL4/STAT3/PARP1	3
BP	GO:0034394	protein localization to cell surface	3/187	66/18866	0.027801007	0.053520055	0.024290613	AKT1/EGF/BDNF	3
BP	GO:0046365	monosaccharide catabolic process	3/187	66/18866	0.027801007	0.053520055	0.024290613	TP53/BAD/HK2	3
BP	GO:0072171	mesonephric tubule morphogenesis	3/187	66/18866	0.027801007	0.053520055	0.024290613	BCL2/VEGFA/MYC	3
BP	GO:1905207	regulation of cardiocyte	3/187	66/18866	0.027801007	0.053520055	0.024290613	EGFR/PPARA/EDN1	3

BP	GO:0060828	regulation of canonical Wnt signaling pathway	7/187	295/18866	0.028003382	0.053885486	0.024456468	MAPK14/GSK3B/EGFR/XIAP/EGF/CAV1/COL1A	7
BP	GO:0002220	innate immune response activating cell surface receptor signaling	4/187	116/18866	0.028387492	0.054600136	0.024780819	RELA/IKBKB/RAF1/CHUK	4
BP	GO:0031647	regulation of protein stability	7/187	296/18866	0.028450705	0.054697213	0.024824878	BCL2/CASP3/CDKN1A/MAPK1/TP53/MDM2/CHEK2	7
BP	GO:0001906	cell killing	5/187	172/18866	0.028533148	0.054806623	0.024874535	NOS2/ICAM1/IFNG/IL4/BAD	5
BP	GO:0002244	hematopoietic progenitor cell differentiation	5/187	172/18866	0.028533148	0.054806623	0.024874535	BCL2/TP53/TOP2A/KDR/NFE2L2	5
BP	GO:0033866	nucleoside bisphosphate biosynthetic process	3/187	67/18866	0.028897656	0.055382874	0.025136072	FASN/ACACA/SCD	3
BP	GO:0034030	ribonucleoside bisphosphate biosynthetic process	3/187	67/18866	0.028897656	0.055382874	0.025136072	FASN/ACACA/SCD	3
BP	GO:0034033	purine nucleoside bisphosphate biosynthetic process	3/187	67/18866	0.028897656	0.055382874	0.025136072	FASN/ACACA/SCD	3
BP	GO:0070613	regulation of protein processing	3/187	67/18866	0.028897656	0.055382874	0.025136072	MDM2/XIAP/SERPINE1	3
BP	GO:0071300	cellular response to retinoic acid	3/187	67/18866	0.028897656	0.055382874	0.025136072	SLC6A4/PPARG/COL1A	3
BP	GO:0010996	response to auditory stimulus	2/187	27/18866	0.029159868	0.055439063	0.025161574	CXCL10/DRD2	2
BP	GO:0031954	positive regulation of protein autophosphorylation	2/187	27/18866	0.029159868	0.055439063	0.025161574	VEGFA/INS	2
BP	GO:0032607	interferon-alpha production	2/187	27/18866	0.029159868	0.055439063	0.025161574	STAT1/CHUK	2
BP	GO:0034698	response to gonadotropin	2/187	27/18866	0.029159868	0.055439063	0.025161574	ICAM1/CCNA2	2
BP	GO:0036037	CD8-positive, alpha-beta T cell activation	2/187	27/18866	0.029159868	0.055439063	0.025161574	BCL2/IRF1	2
BP	GO:0042481	regulation of odontogenesis	2/187	27/18866	0.029159868	0.055439063	0.025161574	RUNX2/EDN1	2
BP	GO:0048668	collateral sprouting	2/187	27/18866	0.029159868	0.055439063	0.025161574	SPP1/BDNF	2
BP	GO:0051953	negative regulation of amine	2/187	27/18866	0.029159868	0.055439063	0.025161574	ADRA2A/DRD2	2
BP	GO:0060544	regulation of necroptotic process	2/187	27/18866	0.029159868	0.055439063	0.025161574	CASP8/CAV1	2
BP	GO:0062098	regulation of programmed necrotic cell death	2/187	27/18866	0.029159868	0.055439063	0.025161574	CASP8/CAV1	2
BP	GO:0072337	modified amino acid transport	2/187	27/18866	0.029159868	0.055439063	0.025161574	ABCC1/GJA1	2
BP	GO:0072539	T-helper 17 cell differentiation	2/187	27/18866	0.029159868	0.055439063	0.025161574	IL4/STAT3	2
BP	GO:1900078	positive regulation of cellular response to insulin stimulus	2/187	27/18866	0.029159868	0.055439063	0.025161574	IGF2/INS	2
BP	GO:1900120	regulation of receptor binding	2/187	27/18866	0.029159868	0.055439063	0.025161574	MMP9/BDNF	2
BP	GO:1901623	regulation of lymphocyte chemotaxis	2/187	27/18866	0.029159868	0.055439063	0.025161574	CCL2/CXCL10	2
BP	GO:1905208	negative regulation of cardiocyte differentiation	2/187	27/18866	0.029159868	0.055439063	0.025161574	EGFR/PPARA	2
BP	GO:0002758	innate immune response-activating signal transduction	4/187	117/18866	0.029172337	0.055439063	0.025161574	RELA/IKBKB/RAF1/CHUK	4

BP	GO:0030282	bone mineralization	4/187	117/18866	0.029172337	0.055439063	0.025161574	PTGS2/ALOX5/MMP13/HIF1A	4
BP	GO:0043666	regulation of phosphoprotein phosphatase activity	4/187	117/18866	0.029172337	0.055439063	0.025161574	IKBKB/GSK3B/IGFBP3/DRD2	4
BP	GO:0003205	cardiac chamber development	5/187	174/18866	0.029785252	0.0565788	0.025678855	RXRA/TP53/MDM2/HIF1A/NOS3	5
BP	GO:0007052	mitotic spindle organization	4/187	118/18866	0.029969973	0.056904509	0.025826682	BIRC5/CCNB1/XIAP/CHEK2	4
BP	GO:0034381	plasma lipoprotein particle clearance	3/187	68/18866	0.030016985	0.056918248	0.025832917	HMOX1/APOB/SOAT1	3
BP	GO:0051148	negative regulation of muscle cell differentiation	3/187	68/18866	0.030016985	0.056918248	0.025832917	PPARA/CXCL10/BDNF	3
BP	GO:1904377	positive regulation of protein localization to cell periphery	3/187	68/18866	0.030016985	0.056918248	0.025832917	AKT1/EGFR/IFNG	3
BP	GO:0010921	regulation of phosphatase activity	5/187	175/18866	0.030424114	0.057664778	0.026171737	IKBKB/GSK3B/IFNG/IGFBP3/DRD2	5
BP	GO:0071706	tumor necrosis factor superfamily cytokine production	4/187	119/18866	0.030780429	0.058314378	0.026466565	CD14/LBP/GSTP1/IFNG	4
BP	GO:0048588	developmental cell growth	6/187	237/18866	0.031007345	0.058718365	0.026649918	GSK3B/VEGFA/PPARA/SPP1/EDN1/BDNF	6
BP	GO:1903317	regulation of protein maturation	3/187	69/18866	0.031158944	0.05872379	0.02665238	MDM2/XIAP/SERPINE1	3
BP	GO:0003180	aortic valve morphogenesis	2/187	28/18866	0.031201715	0.05872379	0.02665238	RB1/NOS3	2
BP	GO:0006972	hyperosmotic response	2/187	28/18866	0.031201715	0.05872379	0.02665238	AKR1B1/MAPK10	2
BP	GO:0010818	T cell chemotaxis	2/187	28/18866	0.031201715	0.05872379	0.02665238	CXCL11/CXCL10	2
BP	GO:0022011	myelination in peripheral nervous system	2/187	28/18866	0.031201715	0.05872379	0.02665238	AKT1/SOD1	2
BP	GO:0032292	peripheral nervous system axon ensheathment	2/187	28/18866	0.031201715	0.05872379	0.02665238	AKT1/SOD1	2
BP	GO:0033561	regulation of water loss via skin	2/187	28/18866	0.031201715	0.05872379	0.02665238	MET/CLDN4	2
BP	GO:0035883	enteroendocrine cell differentiation	2/187	28/18866	0.031201715	0.05872379	0.02665238	GSK3B/BAD	2
BP	GO:0045745	positive regulation of G protein-coupled receptor signaling pathway	2/187	28/18866	0.031201715	0.05872379	0.02665238	PRKCA/DRD2	2
BP	GO:0045932	negative regulation of muscle contraction	2/187	28/18866	0.031201715	0.05872379	0.02665238	PTGS2/SOD1	2
BP	GO:0060143	positive regulation of syncytium formation by plasma membrane	2/187	28/18866	0.031201715	0.05872379	0.02665238	MAPK14/IL4	2
BP	GO:0071168	protein localization to chromatin	2/187	28/18866	0.031201715	0.05872379	0.02665238	ESR1/RB1	2
BP	GO:1900101	regulation of endoplasmic reticulum unfolded protein response	2/187	28/18866	0.031201715	0.05872379	0.02665238	BAX/HSPA5	2
BP	GO:1905563	negative regulation of vascular endothelial cell proliferation	2/187	28/18866	0.031201715	0.05872379	0.02665238	PPARG/CCL2	2
BP	GO:0008088	axo-dendritic transport	3/187	70/18866	0.032323479	0.060808368	0.027598487	SOD1/HIF1A/HSPB1	3

BP	GO:0002230	positive regulation of defense response to virus by host	2/187	29/18866	0.033298392	0.06245085	0.028343944	STAT1/IL4	2
BP	GO:0015721	bile acid and bile salt transport	2/187	29/18866	0.033298392	0.06245085	0.028343944	RXRA/AKR1C1	2
BP	GO:0043032	positive regulation of macrophage activation	2/187	29/18866	0.033298392	0.06245085	0.028343944	LBP/MMP8	2
BP	GO:0050901	leukocyte tethering or rolling	2/187	29/18866	0.033298392	0.06245085	0.028343944	SELE/VCAM1	2
BP	GO:0060351	cartilage development involved in endochondral bone morphogenesis	2/187	29/18866	0.033298392	0.06245085	0.028343944	MMP13/COL1A1	2
BP	GO:0150117	positive regulation of cell-substrate junction organization	2/187	29/18866	0.033298392	0.06245085	0.028343944	VEGFA/KDR	2
BP	GO:2000108	positive regulation of leukocyte apoptotic process	2/187	29/18866	0.033298392	0.06245085	0.028343944	BAX/TP53	2
BP	GO:0002292	T cell differentiation involved in immune response	3/187	71/18866	0.03351053	0.062711728	0.028462346	IFNG/IL4/STAT3	3
BP	GO:0009201	ribonucleoside triphosphate biosynthetic process	3/187	71/18866	0.03351053	0.062711728	0.028462346	IL4/STAT3/PARP1	3
BP	GO:0015800	acidic amino acid transport	3/187	71/18866	0.03351053	0.062711728	0.028462346	GJA1/TRPV1/BDNF	3
BP	GO:0045739	positive regulation of DNA repair	3/187	71/18866	0.03351053	0.062711728	0.028462346	EGFR/PCNA/PARP1	3
BP	GO:0050891	multicellular organismal water homeostasis	3/187	71/18866	0.03351053	0.062711728	0.028462346	AKR1B1/MET/CLDN4	3
BP	GO:0030111	regulation of Wnt signaling pathway	8/187	375/18866	0.033737018	0.063108068	0.028642229	ESR1/MAPK14/GSK3B/EGFR/XIAP/EGF/CAV1/COL1A1	8
BP	GO:0042471	ear morphogenesis	4/187	123/18866	0.034150911	0.063854469	0.02898099	MAPK1/MAPK3/SOD1/EDN1	4
BP	GO:0070988	demethylation	3/187	72/18866	0.03472003	0.064862093	0.029438311	CYP3A4/CYP1A2/CYP1A1	3
BP	GO:1904427	positive regulation of calcium ion transmembrane transport	3/187	72/18866	0.03472003	0.064862093	0.029438311	BAX/CXCL11/CXCL10	3
BP	GO:0002698	negative regulation of immune effector process	4/187	124/18866	0.035025794	0.065404844	0.029684643	HMOX1/IL2RA/IL4/INS	4
BP	GO:0007271	synaptic transmission, cholinergic	2/187	30/18866	0.035448622	0.065907724	0.029912881	ACHE/NQO1	2
BP	GO:0033137	negative regulation of peptidyl-serine phosphorylation	2/187	30/18866	0.035448622	0.065907724	0.029912881	BAX/CAV1	2
BP	GO:0035066	positive regulation of histone acetylation	2/187	30/18866	0.035448622	0.065907724	0.029912881	MAPK3/IL1B	2
BP	GO:0042133	neurotransmitter metabolic process	2/187	30/18866	0.035448622	0.065907724	0.029912881	SLC6A4/ACHE	2
BP	GO:0044030	regulation of DNA methylation	2/187	30/18866	0.035448622	0.065907724	0.029912881	MYC/PARP1	2
BP	GO:0045939	negative regulation of steroid metabolic process	2/187	30/18866	0.035448622	0.065907724	0.029912881	SOD1/UGT1A1	2
BP	GO:0070168	negative regulation of biomineral tissue development	2/187	30/18866	0.035448622	0.065907724	0.029912881	HIF1A/NOS3	2

BP	GO:0110150	negative regulation of biomineralization	2/187	30/18866	0.035448622	0.065907724	0.029912881	HIF1A/NOS3	2
BP	GO:1900027	regulation of ruffle assembly	2/187	30/18866	0.035448622	0.065907724	0.029912881	ICAM1/CAV1	2
BP	GO:1903205	regulation of hydrogen peroxide-induced cell death	2/187	30/18866	0.035448622	0.065907724	0.029912881	MET/NFE2L2	2
BP	GO:0030177	positive regulation of Wnt signaling pathway	5/187	183/18866	0.035846011	0.066617716	0.030235118	EGFR/XIAP/EGF/CAV1/COL1A1	5
BP	GO:0072078	nephron tubule morphogenesis	3/187	73/18866	0.035951908	0.066785609	0.030311318	BCL2/VEGFA/MYC	3
BP	GO:0006305	DNA alkylation	3/187	74/18866	0.037206086	0.069025811	0.031328056	FOS/MYC/PARP1	3
BP	GO:0006306	DNA methylation	3/187	74/18866	0.037206086	0.069025811	0.031328056	FOS/MYC/PARP1	3
BP	GO:0046503	glycerolipid catabolic process	3/187	74/18866	0.037206086	0.069025811	0.031328056	APOB/LPL/FABP1	3
BP	GO:0032535	regulation of cellular component size	8/187	383/18866	0.037476359	0.069497197	0.031541999	AKT1/ICAM1/GSK3B/VEGFA/CDK4/SPP1/EDN1/BDNF	8
BP	GO:0010644	cell communication by electrical coupling	2/187	31/18866	0.037651151	0.069550943	0.031566392	CAV1/GJA1	2
BP	GO:0014044	Schwann cell development	2/187	31/18866	0.037651151	0.069550943	0.031566392	AKT1/SOD1	2
BP	GO:0034377	plasma lipoprotein particle assembly	2/187	31/18866	0.037651151	0.069550943	0.031566392	APOB/SOAT1	2
BP	GO:0045987	positive regulation of smooth muscle contraction	2/187	31/18866	0.037651151	0.069550943	0.031566392	PTGS2/EDN1	2
BP	GO:0060261	positive regulation of transcription initiation from RNA polymerase II promoter	2/187	31/18866	0.037651151	0.069550943	0.031566392	ESR1/TP53	2
BP	GO:0060325	face morphogenesis	2/187	31/18866	0.037651151	0.069550943	0.031566392	MMP2/COL1A1	2
BP	GO:0072538	T-helper 17 type immune response	2/187	31/18866	0.037651151	0.069550943	0.031566392	IL4/STAT3	2
BP	GO:1902230	negative regulation of intrinsic apoptotic signaling pathway in response to DNA damage	2/187	31/18866	0.037651151	0.069550943	0.031566392	BCL2/BCL2L1	2
BP	GO:1904292	regulation of ERAD pathway	2/187	31/18866	0.037651151	0.069550943	0.031566392	CAV1/NFE2L2	2
BP	GO:0001704	formation of primary germ layer	4/187	127/18866	0.037728114	0.069633187	0.031603719	MMP2/MMP9/MMP8/GJA1	4
BP	GO:0017015	regulation of transforming growth factor beta receptor signaling	4/187	127/18866	0.037728114	0.069633187	0.031603719	TP53/GOT1/HSPA5/CAV1	4
BP	GO:0043488	regulation of mRNA stability	5/187	186/18866	0.038023343	0.070147921	0.031837336	PRKCA/AKT1/MAPK14/HSPB1/E2F1	5
BP	GO:0002479	antigen processing and presentation of exogenous peptide antigen via MHC class I, TAP-dependent	3/187	75/18866	0.038482481	0.070842749	0.032152691	IKBKB/NCF1/CHUK	3
BP	GO:0009064	glutamine family amino acid metabolic process	3/187	75/18866	0.038482481	0.070842749	0.032152691	NOS2/GOT1/NOS3	3
BP	GO:0033143	regulation of intracellular steroid hormone receptor signaling pathway	3/187	75/18866	0.038482481	0.070842749	0.032152691	AR/ESR1/PARP1	3

BP	GO:0035019	somatic stem cell population maintenance	3/187	75/18866	0.038482481	0.070842749	0.032152691	STAT3/RAF1/MYC	3
BP	GO:0045685	regulation of glial cell differentiation	3/187	75/18866	0.038482481	0.070842749	0.032152691	PPARG/RELA/CDK1	3
BP	GO:1903844	regulation of cellular response to transforming growth factor beta stimulus	4/187	129/18866	0.03959448	0.072858595	0.033067603	TP53/GOT1/HSPA5/CAV1	4
BP	GO:0006278	RNA-dependent DNA biosynthetic process	3/187	76/18866	0.039781004	0.073053759	0.03315618	MAPK1/MAPK3/TEP1	3
BP	GO:0002082	regulation of oxidative phosphorylation	2/187	32/18866	0.039904744	0.073053759	0.03315618	CDK1/CCNB1	2
BP	GO:0003176	aortic valve development	2/187	32/18866	0.039904744	0.073053759	0.03315618	RB1/NOS3	2
BP	GO:0035456	response to interferon-beta	2/187	32/18866	0.039904744	0.073053759	0.03315618	STAT1/IRF1	2
BP	GO:0042168	heme metabolic process	2/187	32/18866	0.039904744	0.073053759	0.03315618	HMOX1/UGT1A1	2
BP	GO:0043304	regulation of mast cell degranulation	2/187	32/18866	0.039904744	0.073053759	0.03315618	HMOX1/IL4	2
BP	GO:0044818	mitotic G2/M transition checkpoint	2/187	32/18866	0.039904744	0.073053759	0.03315618	CDK1/CDKN1A	2
BP	GO:0060603	mammary gland duct morphogenesis	2/187	32/18866	0.039904744	0.073053759	0.03315618	AR/ESR1	2
BP	GO:0061036	positive regulation of cartilage development	2/187	32/18866	0.039904744	0.073053759	0.03315618	RELA/RUNX2	2
BP	GO:0098810	neurotransmitter reuptake	2/187	32/18866	0.039904744	0.073053759	0.03315618	SLC6A4/DRD2	2
BP	GO:1901889	negative regulation of cell junction assembly	2/187	32/18866	0.039904744	0.073053759	0.03315618	IKBKB/IL1B	2
BP	GO:1901976	regulation of cell cycle checkpoint	2/187	32/18866	0.039904744	0.073053759	0.03315618	CCNB1/CHEK2	2
BP	GO:0007369	gastrulation	5/187	189/18866	0.040280142	0.07370957	0.033453827	MMP2/MMP9/TP53/MMP8/GJA1	5
BP	GO:0060411	cardiac septum morphogenesis	3/187	77/18866	0.041101564	0.075116651	0.034092445	TP53/MDM2/NOS3	3
BP	GO:0061333	renal tubule morphogenesis	3/187	77/18866	0.041101564	0.075116651	0.034092445	BCL2/VEGFA/MYC	3
BP	GO:0070830	bicellular tight junction assembly	3/187	77/18866	0.041101564	0.075116651	0.034092445	IKBKB/GJA1/CLDN4	3
BP	GO:0090092	regulation of transmembrane receptor protein serine/threonine kinase signaling pathway	6/187	254/18866	0.041239557	0.075336773	0.034192349	TP53/XIAP/GOT1/HSPA5/CAV1/PARP1	6
BP	GO:0030900	forebrain development	8/187	391/18866	0.041485652	0.075754107	0.03438176	BAX/CASP3/GSK3B/EGFR/BAD/HIF1A/E2F1/DRD2	8
BP	GO:0003231	cardiac ventricle development	4/187	131/18866	0.041512751	0.075771361	0.034389591	RXRA/MDM2/HIF1A/NOS3	4
BP	GO:0002691	regulation of cellular extravasation	2/187	33/18866	0.042208182	0.076747025	0.034832406	ICAM1/SELE	2
BP	GO:0033006	regulation of mast cell activation involved in immune response	2/187	33/18866	0.042208182	0.076747025	0.034832406	HMOX1/IL4	2
BP	GO:0042755	eating behavior	2/187	33/18866	0.042208182	0.076747025	0.034832406	OPRM1/STAT3	2
BP	GO:0045736	negative regulation of cyclin-dependent protein serine/threonine kinase activity	2/187	33/18866	0.042208182	0.076747025	0.034832406	CASP3/CDKN1A	2

BP	GO:0045879	negative regulation of smoothened signaling pathway	2/187	33/18866	0.042208182	0.076747025	0.034832406	RB1/RUNX2	2
BP	GO:0045920	negative regulation of exocytosis	2/187	33/18866	0.042208182	0.076747025	0.034832406	HMOX1/ADRA2A	2
BP	GO:0051984	positive regulation of chromosome segregation	2/187	33/18866	0.042208182	0.076747025	0.034832406	RB1/CCNB1	2
BP	GO:1901797	negative regulation of signal transduction by p53 class mediator	2/187	33/18866	0.042208182	0.076747025	0.034832406	BCL2/MDM2	2
BP	GO:2000758	positive regulation of peptidyl-lysine acetylation	2/187	33/18866	0.042208182	0.076747025	0.034832406	MAPK3/IL1B	2
BP	GO:0030433	ubiquitin-dependent ERAD pathway	3/187	78/18866	0.042444061	0.077045393	0.034967823	HSPA5/CAV1/NFE2L2	3
BP	GO:0043367	CD4-positive, alpha-beta T cell differentiation	3/187	78/18866	0.042444061	0.077045393	0.034967823	IFNG/IL4/STAT3	3
BP	GO:0043900	regulation of multi-organism process	3/187	78/18866	0.042444061	0.077045393	0.034967823	VEGFA/PLB1/GJA1	3
BP	GO:0060395	SMAD protein signal transduction	3/187	78/18866	0.042444061	0.077045393	0.034967823	JUN/FOS/PARP1	3
BP	GO:0006282	regulation of DNA repair	4/187	132/18866	0.042491356	0.077066071	0.034977208	CHEK1/EGFR/PCNA/PARP1	4
BP	GO:0019751	polyol metabolic process	4/187	132/18866	0.042491356	0.077066071	0.034977208	CYP3A4/AKR1B1/GOT1/HRH1	4
BP	GO:0016525	negative regulation of angiogenesis	5/187	192/18866	0.042616852	0.077261041	0.035065697	PPARG/STAT1/ALOX5/SERPINE1/CXCL10	5
BP	GO:0061136	regulation of proteasomal protein catabolic process	5/187	193/18866	0.043413578	0.078672221	0.035706176	AKT1/GSK3B/MDM2/CAV1/NFE2L2	5
BP	GO:0006997	nucleus organization	4/187	133/18866	0.043482936	0.07876466	0.03574813	PRKCA/CDK1/CCNB1/PRKCB	4
BP	GO:0050851	antigen receptor-mediated signaling pathway	7/187	325/18866	0.043598107	0.078939972	0.035827697	BCL2/BAX/RELA/IKBKB/MAPK1/PRKCB/CHU	7
BP	GO:0000422	autophagy of mitochondrion	3/187	79/18866	0.043808394	0.078954422	0.035834256	TP53/HIF1A/HK2	3
BP	GO:0007492	endoderm development	3/187	79/18866	0.043808394	0.078954422	0.035834256	MMP2/MMP9/MMP8	3
BP	GO:0009205	purine ribonucleoside triphosphate metabolic process	3/187	79/18866	0.043808394	0.078954422	0.035834256	IL4/STAT3/PARP1	3
BP	GO:0016575	histone deacetylation	3/187	79/18866	0.043808394	0.078954422	0.035834256	MAPK8/VEGFA/TP53	3
BP	GO:0030104	water homeostasis	3/187	79/18866	0.043808394	0.078954422	0.035834256	AKR1B1/MET/CLDN4	3
BP	GO:0042246	tissue regeneration	3/187	79/18866	0.043808394	0.078954422	0.035834256	CDKN1A/CCNB1/PPAR	3
BP	GO:0050672	negative regulation of lymphocyte proliferation	3/187	79/18866	0.043808394	0.078954422	0.035834256	CASP3/ERBB2/IL2RA	3
BP	GO:0061726	mitochondrion disassembly	3/187	79/18866	0.043808394	0.078954422	0.035834256	TP53/HIF1A/HK2	3
BP	GO:0090398	cellular senescence	3/187	79/18866	0.043808394	0.078954422	0.035834256	MAPK14/CDKN1A/TP53	3
BP	GO:0120192	tight junction assembly	3/187	79/18866	0.043808394	0.078954422	0.035834256	IKBKB/GJA1/CLDN4	3
BP	GO:1900034	regulation of cellular response to	3/187	79/18866	0.043808394	0.078954422	0.035834256	GSK3B/MAPK1/MAPK3	3
BP	GO:2000181	negative regulation of blood vessel morphogenesis	5/187	194/18866	0.044219237	0.079661429	0.036155137	PPARG/STAT1/ALOX5/SERPINE1/CXCL10	5
BP	GO:0045995	regulation of embryonic	4/187	134/18866	0.044487489	0.079773659	0.036206074	AR/CDK1/GJA1/NFE2L2	4

BP	GO:0000737	DNA catabolic process, endonucleolytic	2/187	34/18866	0.044560269	0.079773659	0.036206074	BAX/CASP3	2
BP	GO:0007616	long-term memory	2/187	34/18866	0.044560269	0.079773659	0.036206074	RASGRF1/DRD2	2
BP	GO:0008156	negative regulation of DNA	2/187	34/18866	0.044560269	0.079773659	0.036206074	TP53/CHEK2	2
BP	GO:0008608	attachment of spindle microtubules to kinetochore	2/187	34/18866	0.044560269	0.079773659	0.036206074	RB1/CCNB1	2
BP	GO:0030431	sleep	2/187	34/18866	0.044560269	0.079773659	0.036206074	FOS/DRD2	2
BP	GO:0032212	positive regulation of telomere maintenance via telomerase	2/187	34/18866	0.044560269	0.079773659	0.036206074	MAPK1/MAPK3	2
BP	GO:0036474	cell death in response to hydrogen peroxide	2/187	34/18866	0.044560269	0.079773659	0.036206074	MET/NFE2L2	2
BP	GO:0042311	vasodilation	2/187	34/18866	0.044560269	0.079773659	0.036206074	SOD1/NOS3	2
BP	GO:0043516	regulation of DNA damage response, signal transduction by p53 class mediator	2/187	34/18866	0.044560269	0.079773659	0.036206074	TP53/MDM2	2
BP	GO:0051482	positive regulation of cytosolic calcium ion concentration involved in phospholipase C-activating G protein-coupled signaling pathway	2/187	34/18866	0.044560269	0.079773659	0.036206074	DRD2/EDN1	2
BP	GO:0055022	negative regulation of cardiac muscle tissue growth	2/187	34/18866	0.044560269	0.079773659	0.036206074	PPARA/GJA1	2
BP	GO:0061117	negative regulation of heart growth	2/187	34/18866	0.044560269	0.079773659	0.036206074	PPARA/GJA1	2
BP	GO:0070232	regulation of T cell apoptotic	2/187	34/18866	0.044560269	0.079773659	0.036206074	TP53/HIF1A	2
BP	GO:1904030	negative regulation of cyclin-dependent protein kinase activity	2/187	34/18866	0.044560269	0.079773659	0.036206074	CASP3/CDKN1A	2
BP	GO:0043487	regulation of RNA stability	5/187	195/18866	0.045033839	0.080587858	0.036575606	PRKCA/AKT1/MAPK14/HSPB1/E2F1	5
BP	GO:0003151	outflow tract morphogenesis	3/187	80/18866	0.045194454	0.080740654	0.036644955	JUN/VEGFA/HIF1A	3
BP	GO:0032945	negative regulation of mononuclear cell proliferation	3/187	80/18866	0.045194454	0.080740654	0.036644955	CASP3/ERBB2/IL2RA	3
BP	GO:0042590	antigen processing and presentation of exogenous peptide antigen via MHC class I	3/187	80/18866	0.045194454	0.080740654	0.036644955	IKBKB/NCF1/CHUK	3
BP	GO:2000300	regulation of synaptic vesicle exocytosis	3/187	80/18866	0.045194454	0.080740654	0.036644955	GSK3B/PRKCB/DRD2	3
BP	GO:1903362	regulation of cellular protein catabolic process	6/187	260/18866	0.045305207	0.080904848	0.036719476	AKT1/GSK3B/MDM2/EGF/CAV1/NFE2L2	6
BP	GO:0050853	B cell receptor signaling pathway	4/187	135/18866	0.045505009	0.08122786	0.036866078	BCL2/BAX/MAPK1/PRKCB	4
BP	GO:0001960	negative regulation of cytokine-mediated signaling pathway	3/187	81/18866	0.046602131	0.083048131	0.037692226	PPARG/GSTP1/CAV1	3

BP	GO:0010833	telomere maintenance via telomere lengthening	3/187	81/18866	0.046602131	0.083048131	0.037692226	MAPK1/MAPK3/PARP1	3
BP	GO:0033238	regulation of cellular amine metabolic process	3/187	81/18866	0.046602131	0.083048131	0.037692226	ODC1/NQO1/INS	3
BP	GO:0051279	regulation of release of sequestered calcium ion into cytosol	3/187	81/18866	0.046602131	0.083048131	0.037692226	BAX/CXCL11/CXCL10	3
BP	GO:0003203	endocardial cushion morphogenesis	2/187	35/18866	0.046959824	0.083236386	0.037777667	MDM2/NOS3	2
BP	GO:0006471	protein ADP-ribosylation	2/187	35/18866	0.046959824	0.083236386	0.037777667	IFNG/PARP1	2
BP	GO:0010259	multicellular organism aging	2/187	35/18866	0.046959824	0.083236386	0.037777667	TP53/EDN1	2
BP	GO:0016486	peptide hormone processing	2/187	35/18866	0.046959824	0.083236386	0.037777667	CES1/ENPEP	2
BP	GO:0039694	viral RNA genome replication	2/187	35/18866	0.046959824	0.083236386	0.037777667	TOP2A/CXCL8	2
BP	GO:0045922	negative regulation of fatty acid metabolic process	2/187	35/18866	0.046959824	0.083236386	0.037777667	AKT1/INS	2
BP	GO:0060251	regulation of glial cell proliferation	2/187	35/18866	0.046959824	0.083236386	0.037777667	IL1B/E2F1	2
BP	GO:0060674	placenta blood vessel development	2/187	35/18866	0.046959824	0.083236386	0.037777667	AKT1/MAPK1	2
BP	GO:0065005	protein-lipid complex assembly	2/187	35/18866	0.046959824	0.083236386	0.037777667	APOB/SOAT1	2
BP	GO:0070884	regulation of calcineurin-NFAT signaling cascade	2/187	35/18866	0.046959824	0.083236386	0.037777667	GSK3B/ERBB3	2
BP	GO:0110111	negative regulation of animal organ morphogenesis	2/187	35/18866	0.046959824	0.083236386	0.037777667	BCL2/STAT1	2
BP	GO:0140448	signaling receptor ligand precursor processing	2/187	35/18866	0.046959824	0.083236386	0.037777667	CES1/ENPEP	2
BP	GO:1901020	negative regulation of calcium ion transmembrane transporter activity	2/187	35/18866	0.046959824	0.083236386	0.037777667	ADRA2A/DRD2	2
BP	GO:0009142	nucleoside triphosphate biosynthetic process	3/187	82/18866	0.048031307	0.085065347	0.03860776	IL4/STAT3/PARP1	3
BP	GO:0120193	tight junction organization	3/187	82/18866	0.048031307	0.085065347	0.03860776	IKBKB/GJA1/CLDN4	3
BP	GO:0008544	epidermis development	9/187	477/18866	0.048617261	0.085957696	0.039012762	BCL2/CASP3/RELA/AKR1C3/EGFR/PPARD/SOD1/PPARA/FOSL2	9
BP	GO:0033865	nucleoside bisphosphate metabolic process	4/187	138/18866	0.048635277	0.085957696	0.039012762	FASN/HMGCR/ACACA/SCD	4
BP	GO:0033875	ribonucleoside bisphosphate metabolic process	4/187	138/18866	0.048635277	0.085957696	0.039012762	FASN/HMGCR/ACACA/SCD	4
BP	GO:0034032	purine nucleoside bisphosphate metabolic process	4/187	138/18866	0.048635277	0.085957696	0.039012762	FASN/HMGCR/ACACA/SCD	4
BP	GO:0072329	monocarboxylic acid catabolic process	4/187	138/18866	0.048635277	0.085957696	0.039012762	AKT1/PPARD/PPARA/FABP1	4
BP	GO:0002755	MyD88-dependent toll-like receptor signaling pathway	2/187	36/18866	0.049405688	0.087068398	0.039516865	CD14/IRF1	2
BP	GO:0003382	epithelial cell morphogenesis	2/187	36/18866	0.049405688	0.087068398	0.039516865	AR/MET	2
BP	GO:0033762	response to glucagon	2/187	36/18866	0.049405688	0.087068398	0.039516865	CCNA2/GCG	2

BP	GO:0043368	positive T cell selection	2/187	36/18866	0.049405688	0.087068398	0.039516865	BCL2/STAT3	2
BP	GO:0060323	head morphogenesis	2/187	36/18866	0.049405688	0.087068398	0.039516865	MMP2/COL1A1	2
BP	GO:0071634	regulation of transforming growth factor beta production	2/187	36/18866	0.049405688	0.087068398	0.039516865	PTGS2/HIF1A	2
BP	GO:0106056	regulation of calcineurin-mediated signaling	2/187	36/18866	0.049405688	0.087068398	0.039516865	GSK3B/ERBB3	2
BP	GO:0031145	anaphase-promoting complex-dependent catabolic process	3/187	83/18866	0.049481863	0.087095383	0.039529112	CDK1/CDK2/CCNB1	3
BP	GO:0048041	focal adhesion assembly	3/187	83/18866	0.049481863	0.087095383	0.039529112	BCL2/VEGFA/KDR	3
BP	GO:2000243	positive regulation of reproductive process	3/187	83/18866	0.049481863	0.087095383	0.039529112	AR/VEGFA/PLB1	3
BP	GO:0007272	ensheathment of neurons	4/187	139/18866	0.049704564	0.08737989	0.039658238	AKT1/ERBB2/PPARD/SOD1	4
BP	GO:0008366	axon ensheathment	4/187	139/18866	0.049704564	0.08737989	0.039658238	AKT1/ERBB2/PPARD/SOD1	4
BP	GO:0035304	regulation of protein dephosphorylation	4/187	139/18866	0.049704564	0.08737989	0.039658238	IKBKB/GSK3B/IGFBP3/DRD2	4
CC	GO:0045121	membrane raft	21/187	329/19559	7.31E-12	7.92E-10	5.92E-10	CD14/PTGS2/ADRA1B/SLC6A4/OPRM1/CASP3/CASP8/DPP4/IKBKB/HMOX1/ICAM1/SELE/EGFR/MAPK1/KDR/MAPK3/CAV1/GJA1/NOS3/ABCG	21
CC	GO:0000307	cyclin-dependent protein kinase holoenzyme complex	10/187	43/19559	7.31E-12	7.92E-10	5.92E-10	CDK1/CDK2/CCNA2/CCND1/CDKN1A/RB1/CDK4/PCNA/CCNB1/CDK12	10
CC	GO:0098857	membrane microdomain	21/187	330/19559	7.74E-12	7.92E-10	5.92E-10	CD14/PTGS2/ADRA1B/SLC6A4/OPRM1/CASP3/CASP8/DPP4/IKBKB/HMOX1/ICAM1/SELE/EGFR/MAPK1/KDR/MAPK3/CAV1/GJA1/NOS3/ABCG	21
CC	GO:0098589	membrane region	21/187	343/19559	1.61E-11	1.23E-09	9.21E-10	CD14/PTGS2/ADRA1B/SLC6A4/OPRM1/CASP3/CASP8/DPP4/IKBKB/HMOX1/ICAM1/SELE/EGFR/MAPK1/KDR/MAPK3/CAV1/GJA1/NOS3/ABCG	21
CC	GO:1902554	serine/threonine protein kinase complex	12/187	89/19559	5.12E-11	3.14E-09	2.35E-09	IKBKB/CDK1/CDK2/CCNA2/CCND1/CDKN1A/RB1/CDK4/PCNA/CCNB1/CHUK/CDK12	12

CC	GO:1902911	protein kinase complex	12/187	104/19559	3.29E-10	1.68E-08	1.26E-08	IKBKB/CDK1/CDK2/CCNA2/CCND1/CDKN1A/RB1/CDK4/PCNA/CCNB1/CHUK/CDK12	12
CC	GO:0031983	vesicle lumen	17/187	328/19559	1.84E-08	8.05E-07	6.02E-07	ALOX5/GSTP1/SLPI/CAT/MAPK14/EGFR/VEGFA/MAPK1/MMP8/APOB/EGF/SERPINE1/MPO/CTSD/IGF2/INS/GCG	17
CC	GO:0005667	transcription regulator complex	17/187	413/19559	4.97E-07	1.75E-05	1.31E-05	JUN/PPARG/RELA/AHR/RXRA/CDK2/CCND1/RB1/CDK4/TP53/RXRB/STAT3/FOS/HIF1A/PARP1/RUNX2/E2F1	17
CC	GO:0034774	secretory granule lumen	15/187	322/19559	5.13E-07	1.75E-05	1.31E-05	ALOX5/GSTP1/SLPI/CAT/MAPK14/VEGFA/MAPK1/MMP8/EGF/SERPINE1/MPO/CTSD/IGF2/INS	15
CC	GO:0060205	cytoplasmic vesicle lumen	15/187	326/19559	6.00E-07	1.84E-05	1.38E-05	ALOX5/GSTP1/SLPI/CAT/MAPK14/VEGFA/MAPK1/MMP8/EGF/SERPINE1/MPO/CTSD/IGF2/INS	15
CC	GO:0061695	transferase complex, transferring phosphorus-containing groups	13/187	253/19559	1.02E-06	2.84E-05	2.12E-05	IKBKB/CDK1/CDK2/CCNA2/CCND1/CDKN1A/RB1/CDK4/TP53/PCNA/CCNB1/CHUK/CDK12	13
CC	GO:0005901	caveola	8/187	82/19559	1.17E-06	3.00E-05	2.24E-05	PTGS2/ADRA1B/HMOX1/SELE/MAPK1/MAPK3/CAV1/NOS3	8
CC	GO:0090575	RNA polymerase II transcription regulator complex	10/187	161/19559	3.49E-06	8.25E-05	6.17E-05	JUN/PPARG/RXRA/RB1/TP53/RXRB/STAT3/FOS/HIF1A/E2F1	10
CC	GO:0045178	basal part of cell	7/187	69/19559	4.27E-06	8.99E-05	6.72E-05	EGFR/ERBB2/MET/ABCC1/CLDN4/ERBB3/EDN	7
CC	GO:0045177	apical part of cell	16/187	433/19559	4.39E-06	8.99E-05	6.72E-05	IL6R/DPP4/VCAM1/DPEP1/EGFR/ERBB2/CA2/PLB1/ABCC1/GJA1/DUOX2/ABCG2/CLDN4/ERBB3/FABP1/ENPEP	16
CC	GO:0009925	basal plasma membrane	6/187	51/19559	8.88E-06	0.000170368	0.000127345	EGFR/ERBB2/MET/ABCC1/CLDN4/ERBB3	6

CC	GO:0044853	plasma membrane raft	8/187	113/19559	1.31E-05	0.000236029	0.000176425	PTGS2/ADRA1B/HMOX1/SELE/MAPK1/MAPK3/CAV1/NOS3	8
CC	GO:0101002	ficolin-1-rich granule	8/187	124/19559	2.57E-05	0.000415549	0.000310611	TNFAIP6/ALOX5/GSTP1/CAT/MAPK14/MMP9/MAPK1/CTSD	8
CC	GO:1904813	ficolin-1-rich granule lumen	8/187	124/19559	2.57E-05	0.000415549	0.000310611	TNFAIP6/ALOX5/GSTP1/CAT/MAPK14/MMP9/MAPK1/CTSD	8
CC	GO:0016324	apical plasma membrane	13/187	361/19559	4.65E-05	0.000713055	0.000532988	IL6R/DPP4/DPEP1/EGFR/ERBB2/PLB1/ABCC1/GJA1/DUOX2/ABCG2/CLDN4/ERBB3/ENPEP	13
CC	GO:0031968	organelle outer membrane	10/187	218/19559	4.89E-05	0.00071453	0.000534091	PTGS2/BCL2/BAX/CASP8/BCL2L1/MCL1/BAD/RAF1/GJA1/HK2	10
CC	GO:0019867	outer membrane	10/187	220/19559	5.28E-05	0.000736528	0.000550533	PTGS2/BCL2/BAX/CASP8/BCL2L1/MCL1/BAD/RAF1/GJA1/HK2	10
CC	GO:0005741	mitochondrial outer membrane	9/187	192/19559	9.92E-05	0.001323688	0.000989419	BCL2/BAX/CASP8/BCL2L1/MCL1/BAD/RAF1/GJA1/HK2	9
CC	GO:0005925	focal adhesion	13/187	415/19559	0.000187198	0.002316437	0.00173147	SLC6A4/OPRM1/DPP4/ICAM1/CAT/PLAU/EGFR/MAPK1/MAPK3/HSPA5/CAV1/GJA1/HSPB1	13
CC	GO:0005635	nuclear envelope	14/187	473/19559	0.000190813	0.002316437	0.00173147	PTGS2/ADRA1B/BCL2/BAX/ALOX5/EGFR/CCND1/BCL2L1/CDK4/TP53/PTGES/MAPK3/PTGER3/PARP1	14
CC	GO:0009897	external side of plasma membrane	13/187	417/19559	0.00019618	0.002316437	0.00173147	IL6R/CD14/ICAM1/VCAM1/IL2RA/CD40LG/GSR/F3/THBD/ABCG2/CXCL10/TRPV1/ENPEP	13
CC	GO:0030055	cell-substrate junction	13/187	423/19559	0.000225383	0.002562686	0.001915534	SLC6A4/OPRM1/DPP4/ICAM1/CAT/PLAU/EGFR/MAPK1/MAPK3/HSPA5/CAV1/GJA1/HSPB1	13
CC	GO:0016323	basolateral plasma membrane	9/187	246/19559	0.000620221	0.006683316	0.004995587	IL6R/ADRA2A/EGFR/ERBB2/MET/CA2/ABCC1/CLDN4/ERBB3	9

CC	GO:0031143	pseudopodium	3/187	18/19559	0.000631323	0.006683316	0.004995587	MAPK1/MAPK3/RAF1	3
CC	GO:0005788	endoplasmic reticulum lumen	10/187	308/19559	0.000788493	0.008068914	0.006031282	PTGS2/APOB/CES1/HSPA5/COL1A1/SPP1/IGFBP3/EDN1/INS/GCG	10
CC	GO:0005819	spindle	11/187	367/19559	0.000840298	0.00827838	0.006187851	AKT1/CDK1/MAPK14/MAPK1/RB1/BIRC5/CCNB1/XIAP/NR3C1/HSPB1/RASSF1	11
CC	GO:0062023	collagen-containing extracellular matrix	12/187	427/19559	0.000862893	0.00827838	0.006187851	PRSS1/ACHE/ICAM1/SLPI/MMP2/MMP9/MMP8/F3/SERPINE1/COL1A1/CTSD/PCOLCE	12
CC	GO:0000781	chromosome, telomeric region	7/187	164/19559	0.001028777	0.009452613	0.007065556	CDK1/CDK2/CHEK1/PCNA/PARP1/CHEK2/TEP1	7
CC	GO:0043209	myelin sheath	4/187	47/19559	0.001046869	0.009452613	0.007065556	BCL2/AKR1B1/ERBB2/CA2	4
CC	GO:0120111	neuron projection cytoplasm	5/187	87/19559	0.001485076	0.013026239	0.00973674	OPRM1/MAPK1/SOD1/HIF1A/HSPB1	5
CC	GO:1904724	tertiary granule lumen	4/187	55/19559	0.001886451	0.016087239	0.012024749	TNFAIP6/MMP9/MMP8/CTSD	4
CC	GO:0031965	nuclear membrane	9/187	301/19559	0.002508599	0.020814593	0.015558311	PTGS2/ADRA1B/BCL2/ALOX5/EGFR/CCND1/BCL2L1/CDK4/TP53	9
CC	GO:0030139	endocytic vesicle	9/187	313/19559	0.003251781	0.026270964	0.019636791	DPP4/EGFR/MDM2/NCF1/APOB/CAV1/NOS3/MPO/DRD2	9
CC	GO:0016328	lateral plasma membrane	4/187	65/19559	0.003477943	0.027377657	0.020464013	ABCC1/CLDN4/ERBB3/DRD2	4
CC	GO:0031093	platelet alpha granule lumen	4/187	67/19559	0.003879702	0.02843568	0.021254854	VEGFA/EGF/SERPINE1/IGF2	4
CC	GO:0005641	nuclear envelope lumen	2/187	10/19559	0.003890223	0.02843568	0.021254854	ALOX5/PTGES	2
CC	GO:0034663	endoplasmic reticulum chaperone complex	2/187	10/19559	0.003890223	0.02843568	0.021254854	UGT1A1/HSPA5	2
CC	GO:0032839	dendrite cytoplasm	3/187	34/19559	0.004138435	0.029546499	0.022085159	OPRM1/MAPK1/SOD1	3
CC	GO:0035631	CD40 receptor complex	2/187	11/19559	0.004724915	0.032498169	0.024291448	IKBKB/CHUK	2
CC	GO:0034358	plasma lipoprotein particle	3/187	36/19559	0.004869433	0.032498169	0.024291448	PON1/APOB/LPL	3
CC	GO:1990777	lipoprotein particle	3/187	36/19559	0.004869433	0.032498169	0.024291448	PON1/APOB/LPL	3
CC	GO:0099056	integral component of presynaptic membrane	4/187	74/19559	0.005531293	0.03530115	0.026386596	SLC6A4/OPRM1/HTR3A/DRD2	4
CC	GO:0019908	nuclear cyclin-dependent protein kinase holoenzyme complex	2/187	12/19559	0.005634386	0.03530115	0.026386596	RB1/CDK12	2
CC	GO:0043020	NADPH oxidase complex	2/187	12/19559	0.005634386	0.03530115	0.026386596	NCF1/DUOX2	2

CC	GO:0032994	protein-lipid complex	3/187	39/19559	0.006103696	0.037476694	0.028012753	PON1/APOB/LPL	3
CC	GO:0042627	chylomicron	2/187	13/19559	0.006617146	0.039417527	0.029463469	APOB/LPL	2
CC	GO:0098687	chromosomal region	9/187	350/19559	0.006676584	0.039417527	0.029463469	CDK1/CDK2/CHEK1/PCNA/BIRC5/CCNB1/PARP1/CHEK2/TEP1	9
CC	GO:0098889	intrinsic component of presynaptic membrane	4/187	83/19559	0.008268284	0.047893648	0.035799126	SLC6A4/OPRM1/HTR3A/DRD2	4
CC	GO:0034399	nuclear periphery	5/187	131/19559	0.008552474	0.048622397	0.036343846	ALOX5/TP53/PCNA/RUNX1T1/TEP1	5
CC	GO:0005777	peroxisome	5/187	137/19559	0.010258263	0.056237261	0.042035738	NOS2/CAT/SOD1/HMGCR/FABP1	5
CC	GO:0042579	microbody	5/187	137/19559	0.010258263	0.056237261	0.042035738	NOS2/CAT/SOD1/HMGCR/FABP1	5
CC	GO:0005769	early endosome	9/187	377/19559	0.010566529	0.056910954	0.042539304	VCAM1/EGFR/MAPK1/ERBB2/KDR/MAPK3/APOB/CAV1/GJA1	9
CC	GO:0031970	organelle envelope lumen	4/187	90/19559	0.010921782	0.057810123	0.043211407	ALOX5/CAT/PTGES/SOD1	4
CC	GO:0099568	cytoplasmic region	7/187	254/19559	0.011300929	0.058026021	0.043372785	OPRM1/AKR1B1/MAPK1/SOD1/HIF1A/HSPB1/FABP1	7
CC	GO:0031091	platelet alpha granule	4/187	91/19559	0.01134059	0.058026021	0.043372785	VEGFA/EGF/SERPINE1/IGF2	4
CC	GO:0005902	microvillus	4/187	93/19559	0.012208912	0.061444853	0.045928263	VCAM1/DPEP1/AKR1B1/CA2	4
CC	GO:0005782	peroxisomal matrix	3/187	52/19559	0.013485391	0.065714524	0.049119719	NOS2/CAT/FABP1	3
CC	GO:0031907	microbody lumen	3/187	52/19559	0.013485391	0.065714524	0.049119719	NOS2/CAT/FABP1	3
CC	GO:0016327	apicolateral plasma membrane	2/187	19/19559	0.013971964	0.066791001	0.049924355	THBD/CLDN4	2
CC	GO:0032993	protein-DNA complex	6/187	205/19559	0.014141417	0.066791001	0.049924355	ESR1/TOP1/PCNA/FOS/NFE2L2/PARP1	6
MF	GO:0004879	nuclear receptor activity	13/187	52/18352	3.76E-15	9.91E-13	6.08E-13	NR3C2/AR/PPARG/NR1I2/AHR/ESR1/RXRA/ESR2/NR3C1/PPARD/PPARA/RXRB/STAT3	13
MF	GO:0098531	ligand-activated transcription factor activity	13/187	52/18352	3.76E-15	9.91E-13	6.08E-13	NR3C2/AR/PPARG/NR1I2/AHR/ESR1/RXRA/ESR2/NR3C1/PPARD/PPARA/RXRB/STAT3	13

MF	GO:0140297	DNA-binding transcription factor binding	24/187	347/18352	1.39E-13	2.45E-11	1.50E-11	BCL2/JUN/PPARG/RELA/STAT1/NR1I2/ESR1/RXRA/MAPK14/GSK3B/RB1/NFKBIA/PCNA/PPARD/PPARA/STAT3/FOS/HIF1A/MYC/PRKCB/HSPB1/NFE2L2/PARP1/RU	24
MF	GO:0061629	RNA polymerase II-specific DNA-binding transcription factor binding	21/187	267/18352	4.33E-13	5.70E-11	3.50E-11	JUN/PPARG/RELA/STAT1/NR1I2/ESR1/RXRA/MAPK14/GSK3B/RB1/NFKBIA/PCNA/PPARD/PPARA/STAT3/FOS/HIF1A/PRKCB/HSPB1/NFE2L2	21
MF	GO:0003707	steroid hormone receptor activity	8/187	26/18352	1.34E-10	1.31E-08	8.01E-09	NR3C2/ESR1/RXRA/ESR2/NR3C1/PPARD/PPARA/RXRB	8
MF	GO:0019902	phosphatase binding	16/187	194/18352	1.49E-10	1.31E-08	8.01E-09	BCL2/PPARG/AKT1/STAT1/MAPK14/EGFR/MAPK1/TP53/ERBB2/MET/MAPK3/BAD/SOD1/HMGCR/PPARA/STAT3	16
MF	GO:0005496	steroid binding	12/187	106/18352	8.40E-10	6.33E-08	3.88E-08	NR3C2/AR/CYP3A4/AKR1C3/ESR1/ESR2/NR3C1/UGT1A1/AKR1C1/SOAT1/CAV1/FABP1	12
MF	GO:0016209	antioxidant activity	11/187	86/18352	1.15E-09	6.92E-08	4.24E-08	PTGS1/PTGS2/GSTP1/CAT/SOD1/GSR/DUOX2/MPO/NQO1/GSTA1/FAB	11
MF	GO:0016705	oxidoreductase activity, acting on paired donors, with incorporation or reduction of molecular oxygen	14/187	162/18352	1.18E-09	6.92E-08	4.24E-08	PTGS1/PTGS2/NOS2/HMOX1/CYP3A4/CYP1A2/CYP1A1/CYP1B1/AKR1C3/TYR/CYP19A1/AKR1C1/NOS3/SCD	14
MF	GO:0020037	heme binding	13/187	138/18352	1.63E-09	8.61E-08	5.28E-08	PTGS1/PTGS2/NOS2/HMOX1/CYP3A4/CYP1A2/CYP1A1/CYP1B1/CAT/CYP19A1/DUOX2/NOS3	13
MF	GO:0005126	cytokine receptor binding	17/187	271/18352	2.69E-09	1.29E-07	7.91E-08	IL6R/CASP3/CASP8/STAT1/VEGFA/IFNG/IL4/CD40LG/STAT3/IL1B/CCL2/CXCL8/IL1A/CXCL11/CXCL2/CXCL10/BDNF	17

MF	GO:0046906	tetrapyrrole binding	13/187	148/18352	3.87E-09	1.70E-07	1.04E-07	PTGS1/PTGS2/NOS2/HMOX1/CYP3A4/CYP1A2/CYP1A1/CYP1B1/CAT/CYP19A1/DUOX2/NOS3	13
MF	GO:0019903	protein phosphatase binding	13/187	149/18352	4.20E-09	1.70E-07	1.04E-07	BCL2/PPARG/AKT1/STAT1/MAPK14/EGFR/TP53/ERBB2/MET/BAD/SOD1/HMGCR/STAT3	13
MF	GO:0035173	histone kinase activity	6/187	17/18352	1.16E-08	4.38E-07	2.69E-07	PRKCA/CDK1/CDK2/CHEK1/CCNB1/PRKCB	6
MF	GO:0004674	protein serine/threonine kinase activity	20/187	435/18352	2.09E-08	7.36E-07	4.51E-07	PRKCA/IKBKB/AKT1/MAPK8/CDK1/MAPK14/GSK3B/CDK2/CHEK1/EGFR/MAPK1/CDK4/TOP1/MAPK10/MAPK3/RAF1/PRKCB/CHEK2/CHUK/C	20
MF	GO:0044389	ubiquitin-like protein ligase binding	17/187	316/18352	2.65E-08	8.53E-07	5.23E-07	BCL2/JUN/CASP8/RELA/STAT1/GSK3B/EGFR/CDKN1A/RB1/TP53/NFKBIA/MDM2/CCNB1/HIF1A/HSPA5/CHEK2/ERB	17
MF	GO:0048018	receptor ligand activity	21/187	487/18352	2.75E-08	8.53E-07	5.23E-07	DPP4/VEGFA/IFNG/IL4/CD40LG/EGF/IL1B/CCL2/CXCL8/IL1A/CXCL11/CXCL2/CXCL10/SPP1/IGF2/EDN1/BDNF/INS/GCG/PYY/ADM	21
MF	GO:0030546	signaling receptor activator activity	21/187	492/18352	3.28E-08	9.59E-07	5.88E-07	DPP4/VEGFA/IFNG/IL4/CD40LG/EGF/IL1B/CCL2/CXCL8/IL1A/CXCL11/CXCL2/CXCL10/SPP1/IGF2/EDN1/BDNF/INS/GCG/PYY/ADM	21
MF	GO:0031406	carboxylic acid binding	14/187	212/18352	3.77E-08	1.05E-06	6.41E-07	TNFAIP6/NOS2/PPARG/SELE/GSTP1/AKR1C3/RXRA/PPARD/UGT1A1/AKR1C1/GOT1/NOS3/GSTA1/FABP1	14
MF	GO:0033293	monocarboxylic acid binding	9/187	72/18352	4.78E-08	1.26E-06	7.73E-07	PPARG/GSTP1/AKR1C3/RXRA/PPARD/UGT1A1/AKR1C1/GSTA1/FABP1	9

MF	GO:0070491	repressing transcription factor binding	9/187	74/18352	6.10E-08	1.53E-06	9.39E-07	BCL2/PPARG/RELA/STAT1/PPARD/PPARA/STAT3/MYC/RUNX2	9
MF	GO:0043177	organic acid binding	14/187	224/18352	7.51E-08	1.80E-06	1.10E-06	TNFAIP6/NOS2/PPARG/SELE/GSTP1/AKR1C3/RXRA/PPARD/UGT1A1/AKR1C1/GOT1/NOS3/GSTA1/FABP1	14
MF	GO:0004497	monooxygenase activity	10/187	101/18352	8.17E-08	1.87E-06	1.15E-06	NOS2/CYP3A4/CYP1A2/CYP1A1/CYP1B1/AKR1C3/TYR/CYP19A1/AKR1C1/NOS3	10
MF	GO:0008144	drug binding	10/187	104/18352	1.08E-07	2.37E-06	1.46E-06	SLC6A4/PPARG/NR1I2/GSTP1/TOP2A/PPARD/FASN/PPARA/DRD2/FAB	10
MF	GO:0001223	transcription coactivator binding	6/187	29/18352	4.04E-07	8.25E-06	5.06E-06	AR/RELA/AHR/ESR1/PPARD/PPARA	6
MF	GO:0031625	ubiquitin protein ligase binding	15/187	297/18352	4.07E-07	8.25E-06	5.06E-06	BCL2/JUN/CASP8/RELA/GSK3B/EGFR/CDKN1A/RB1/TP53/NFKBIA/MDM2/HIF1A/HSPA5/CHEK2/ERBB3	15
MF	GO:0001221	transcription cofactor binding	7/187	51/18352	8.08E-07	1.58E-05	9.67E-06	AR/RELA/AHR/ESR1/PPARD/PPARA/NFE2L2	7
MF	GO:0005125	cytokine activity	13/187	235/18352	8.96E-07	1.68E-05	1.03E-05	VEGFA/IFNG/IL4/CD40LG/IL1B/CCL2/CXCL8/IL1A/CXCL11/CXCL2/CXCL10/SPP1/EDN1	13
MF	GO:0004601	peroxidase activity	7/187	52/18352	9.26E-07	1.68E-05	1.03E-05	PTGS1/PTGS2/GSTP1/CAT/DUOX2/MPO/GSTA1	7
MF	GO:0046982	protein heterodimerization activity	15/187	321/18352	1.08E-06	1.91E-05	1.17E-05	ADRA1B/BCL2/BAX/IKBKB/AHR/ADRA2A/BCL2L1/TP53/ERBB2/MCL1/TOP2A/HIF1A/CAV1/CHUK/ERBB3	15
MF	GO:0004707	MAP kinase activity	5/187	20/18352	1.43E-06	2.42E-05	1.49E-05	MAPK8/MAPK14/MAPK1/MAPK10/MAPK3	5
MF	GO:0016684	oxidoreductase activity, acting on peroxide as acceptor	7/187	56/18352	1.55E-06	2.55E-05	1.57E-05	PTGS1/PTGS2/GSTP1/CAT/DUOX2/MPO/GSTA1	7

MF	GO:0033218	amide binding	16/187	381/18352	1.91E-06	3.05E-05	1.87E-05	CD14/LBP/OPRM1/PPARG/ACHE/RELA/GSTP1/GSTM1/RXRA/CAT/NFKBIA/PTGES/FASN/SOAT1/NPEPPS/ENPEP	16
MF	GO:0035257	nuclear hormone receptor binding	10/187	144/18352	2.24E-06	3.43E-05	2.10E-05	PPARG/STAT1/NR1I2/ESR1/RXRA/PCNA/STAT3/HIF1A/PRKCB/PARP1	10
MF	GO:0019207	kinase regulator activity	12/187	216/18352	2.28E-06	3.43E-05	2.10E-05	CASP3/GSTP1/CCNA2/CCND1/CDKN1A/CDK4/CCNB1/EGF/HSPB1/CXCL10/IGF2/ERBB3	12
MF	GO:0019887	protein kinase regulator activity	11/187	185/18352	3.16E-06	4.52E-05	2.77E-05	CASP3/CCNA2/CCND1/CDKN1A/CDK4/CCNB1/EGF/HSPB1/CXCL10/IGF2/ERBB3	11
MF	GO:0051400	BH domain binding	4/187	11/18352	3.26E-06	4.52E-05	2.77E-05	BCL2/BAX/BCL2L1/MC	4
MF	GO:0070513	death domain binding	4/187	11/18352	3.26E-06	4.52E-05	2.77E-05	BCL2/BAX/BCL2L1/MC	4
MF	GO:0042277	peptide binding	14/187	308/18352	3.46E-06	4.67E-05	2.86E-05	CD14/LBP/OPRM1/PPARG/ACHE/RELA/GSTP1/GSTM1/RXRA/CAT/NFKBIA/PTGES/NPEPPS/E	14
MF	GO:0070330	aromatase activity	5/187	25/18352	4.69E-06	6.18E-05	3.79E-05	CYP3A4/CYP1A2/CYP1A1/CYP1B1/CYP19A1	5
MF	GO:0008353	RNA polymerase II CTD heptapeptide repeat kinase activity	4/187	12/18352	4.85E-06	6.23E-05	3.82E-05	CDK1/MAPK1/CDK4/CDK12	4
MF	GO:0016709	oxidoreductase activity, acting on paired donors, with incorporation or reduction of molecular oxygen, NAD(P)H as one donor, and incorporation of one atom of oxygen	6/187	45/18352	6.05E-06	7.59E-05	4.65E-05	NOS2/CYP3A4/CYP1A1/AKR1C3/AKR1C1/NOS3	6
MF	GO:0001046	core promoter sequence-specific DNA binding	6/187	46/18352	6.90E-06	8.45E-05	5.18E-05	RELA/STAT1/TP53/NR3C1/FOS/MYC	6
MF	GO:0042379	chemokine receptor binding	7/187	70/18352	7.11E-06	8.51E-05	5.22E-05	STAT1/STAT3/CCL2/CXCL8/CXCL11/CXCL2/CXCL10	7
MF	GO:0016922	nuclear receptor binding	8/187	101/18352	9.01E-06	0.000105532	6.47E-05	PPARG/NR1I2/ESR1/RXRA/PCNA/STAT3/PRKCB/PARP1	8
MF	GO:0019838	growth factor binding	9/187	136/18352	1.08E-05	0.000123211	7.56E-05	IL6R/EGFR/IL10RA/ERBB2/IL2RA/KDR/COL1A1/IGFBP3/ERBB3	9

MF	GO:0016538	cyclin-dependent protein serine/threonine kinase regulator activity	6/187	50/18352	1.13E-05	0.000125283	7.68E-05	CASP3/CCNA2/CCND1/CDKN1A/CDK4/CCNB1	6
MF	GO:0002020	protease binding	9/187	137/18352	1.14E-05	0.000125283	7.68E-05	BCL2/CASP3/DPP4/GSK3B/TP53/F3/SERPINE1/COL1A1/INS	9
MF	GO:0030332	cyclin binding	5/187	30/18352	1.21E-05	0.000129762	7.96E-05	CDK1/CDK2/CDKN1A/CDK4/CDK12	5
MF	GO:0097153	cysteine-type endopeptidase activity involved in apoptotic process	4/187	15/18352	1.31E-05	0.000137566	8.44E-05	CASP9/CASP3/CASP8/CASP7	4
MF	GO:0051427	hormone receptor binding	10/187	177/18352	1.40E-05	0.000144751	8.88E-05	PPARG/STAT1/NR1I2/ESR1/RXRA/PCNA/STAT3/HIF1A/PRKCB/PARP1	10
MF	GO:0016651	oxidoreductase activity, acting on NAD(P)H	8/187	109/18352	1.58E-05	0.000160196	9.82E-05	NOS2/AKR1C3/NCF1/GSR/AKR1C1/DUOX2/NOS3/NQO1	8
MF	GO:0051721	protein phosphatase 2A binding	5/187	32/18352	1.68E-05	0.000165675	0.000101593	BCL2/AKT1/STAT1/TP53/HMGCR	5
MF	GO:0005178	integrin binding	9/187	144/18352	1.70E-05	0.000165675	0.000101593	PRKCA/ICAM1/VCAM1/EGFR/CD40LG/KDR/IL1B/SPP1/IGF2	9
MF	GO:0033613	activating transcription factor binding	7/187	80/18352	1.73E-05	0.000165675	0.000101593	JUN/PPARG/RELA/RB1/FOS/MYC/NFE2L2	7
MF	GO:0050661	NADP binding	6/187	54/18352	1.78E-05	0.000167244	0.000102554	NOS2/CAT/FASN/HMGCR/GSR/NOS3	6
MF	GO:0016712	oxidoreductase activity, acting on paired donors, with incorporation or reduction of molecular oxygen, reduced flavin or flavoprotein as one donor, and incorporation of one atom of oxygen	5/187	35/18352	2.64E-05	0.000243837	0.000149521	CYP3A4/CYP1A2/CYP1A1/CYP1B1/CYP19A1	5
MF	GO:0017171	serine hydrolase activity	10/187	191/18352	2.70E-05	0.000245749	0.000150694	DPP4/PRSS1/ACHE/MMP1/PLAU/MMP2/MMP9/MMP8/MMP3/F3	10
MF	GO:0004708	MAP kinase kinase activity	4/187	18/18352	2.86E-05	0.00025091	0.000153859	MAPK14/MAPK1/MAPK10/MAPK3	4
MF	GO:0045236	CXCR chemokine receptor binding	4/187	18/18352	2.86E-05	0.00025091	0.000153859	CXCL8/CXCL11/CXCL2/CXCL10	4
MF	GO:0097110	scaffold protein binding	6/187	60/18352	3.27E-05	0.000282932	0.000173494	CASP8/IKBKB/MDM2/MAPK3/NOS3/CHUK	6
MF	GO:0001091	RNA polymerase II general transcription initiation factor binding	4/187	19/18352	3.59E-05	0.000305123	0.000187102	AR/AHR/ESR1/TP53	4

MF	GO:0008395	steroid hydroxylase activity	5/187	38/18352	3.98E-05	0.000332772	0.000204057	CYP3A4/CYP1A2/CYP1A1/CYP1B1/CYP19A1	5
MF	GO:0005504	fatty acid binding	5/187	39/18352	4.53E-05	0.000372659	0.000228516	PPARG/GSTP1/PPARD/GSTA1/FABP1	5
MF	GO:0004175	endopeptidase activity	15/187	440/18352	4.69E-05	0.000380173	0.000233123	CASP9/CASP3/CASP8/DPP4/PRSS1/MMP1/PLAU/MMP2/MMP9/CASP7/MMP13/MMP8/MMP3/F3/	15
MF	GO:0031072	heat shock protein binding	8/187	127/18352	4.77E-05	0.000381134	0.000233712	BAX/CDK1/CYP1A1/AHR/NR3C1/KDR/HIF1A/HSPA5	8
MF	GO:0001664	G protein-coupled receptor binding	12/187	293/18352	4.87E-05	0.000383157	0.000234953	STAT1/ADRA2A/STAT3/CCL2/CXCL8/CXCL11/CXCL2/CXCL10/EDN1/GCG/PYY/ADM	12
MF	GO:0004252	serine-type endopeptidase activity	9/187	169/18352	6.02E-05	0.000466171	0.000285857	DPP4/PRSS1/MMP1/PLAU/MMP2/MMP9/MMP8/MMP3/F3	9
MF	GO:0051879	Hsp90 protein binding	5/187	42/18352	6.52E-05	0.000498224	0.000305512	CYP1A1/AHR/NR3C1/KDR/HIF1A	5
MF	GO:1901681	sulfur compound binding	11/187	262/18352	8.09E-05	0.000608918	0.00037339	GSTP1/GSTM1/VEGFA/PTGES/APOB/SOAT1/MPO/CXCL11/CXCL10/PCOLCE/LPL	11
MF	GO:0004712	protein serine/threonine/tyrosine kinase activity	5/187	45/18352	9.14E-05	0.000678435	0.000416018	AKT1/MAPK14/MAPK1/MAPK10/MAPK3	5
MF	GO:0004364	glutathione transferase activity	4/187	25/18352	0.000111685	0.000817475	0.000501278	GSTP1/GSTM1/GSTA1/GSTA2	4
MF	GO:0032052	bile acid binding	3/187	10/18352	0.000118525	0.00084409	0.000517599	AKR1C3/AKR1C1/FABP	3
MF	GO:0097199	cysteine-type endopeptidase activity involved in apoptotic signaling pathway	3/187	10/18352	0.000118525	0.00084409	0.000517599	CASP9/CASP3/CASP8	3
MF	GO:0032813	tumor necrosis factor receptor superfamily binding	5/187	48/18352	0.000124976	0.000878162	0.000538491	CASP3/CASP8/STAT1/CD40LG/BDNF	5
MF	GO:0008236	serine-type peptidase activity	9/187	187/18352	0.000130638	0.000905869	0.000555481	DPP4/PRSS1/MMP1/PLAU/MMP2/MMP9/MMP8/MMP3/F3	9
MF	GO:0008009	chemokine activity	5/187	49/18352	0.000138037	0.000944746	0.000579321	CCL2/CXCL8/CXCL11/CXCL2/CXCL10	5
MF	GO:0008237	metallopeptidase activity	9/187	189/18352	0.000141584	0.000956603	0.000586591	MMP1/DPEP1/MMP2/MMP9/MMP13/MMP8/MMP3/NPEPPS/ENPEP	9

MF	GO:0035035	histone acetyltransferase binding	4/187	28/18352	0.000176506	0.001157492	0.000709777	STAT1/TP53/PCNA/HIF1A	4
MF	GO:0042826	histone deacetylase binding	7/187	115/18352	0.000176834	0.001157492	0.000709777	RELA/MAPK8/CCND1/TP53/TOP2A/HIF1A/PAR	7
MF	GO:0035258	steroid hormone receptor binding	6/187	81/18352	0.000177907	0.001157492	0.000709777	PPARG/ESR1/PCNA/STAT3/PRKCB/PARP1	6
MF	GO:0004693	cyclin-dependent protein serine/threonine kinase activity	4/187	29/18352	0.000203125	0.001289724	0.000790862	CDK1/CDK2/CDK4/CDK12	4
MF	GO:0097472	cyclin-dependent protein kinase activity	4/187	29/18352	0.000203125	0.001289724	0.000790862	CDK1/CDK2/CDK4/CDK12	4
MF	GO:0004032	alditol:NADP+ 1-oxidoreductase activity	3/187	12/18352	0.000214051	0.001327116	0.000813791	AKR1C3/AKR1B1/AKR1C1	3
MF	GO:0043295	glutathione binding	3/187	12/18352	0.000214051	0.001327116	0.000813791	GSTP1/GSTM1/PTGES	3
MF	GO:0051059	NF-kappaB binding	4/187	31/18352	0.000264853	0.001622994	0.000995225	RELA/GSK3B/NFKBIA/PPARD	4
MF	GO:0016653	oxidoreductase activity, acting on NAD(P)H, heme protein as acceptor	3/187	13/18352	0.000276182	0.001653955	0.00101421	NOS2/NOS3/NQO1	3
MF	GO:1900750	oligopeptide binding	3/187	13/18352	0.000276182	0.001653955	0.00101421	GSTP1/GSTM1/PTGES	3
MF	GO:0015562	efflux transmembrane transporter activity	3/187	14/18352	0.000348873	0.002065801	0.001266755	ABCC1/GJA1/ABCG2	3
MF	GO:0004714	transmembrane receptor protein tyrosine kinase activity	5/187	61/18352	0.000390212	0.002284909	0.001401113	EGFR/ERBB2/MET/KDR/ERBB3	5
MF	GO:0001103	RNA polymerase II repressing transcription factor binding	4/187	35/18352	0.000426952	0.002472568	0.001516186	PPARG/PPARD/PPARA/STAT3	4
MF	GO:0019825	oxygen binding	4/187	36/18352	0.000476524	0.002700303	0.001655833	CYP3A4/CYP1A1/CYP1B1/CYP19A1	4
MF	GO:0097718	disordered domain specific binding	4/187	36/18352	0.000476524	0.002700303	0.001655833	RB1/TP53/MDM2/GJA1	4
MF	GO:0002039	p53 binding	5/187	66/18352	0.000562632	0.003154328	0.001934243	GSK3B/TP53/MDM2/HIF1A/TEP1	5
MF	GO:0061134	peptidase regulator activity	9/187	229/18352	0.000582803	0.003233023	0.001982499	SLPI/DPEP1/BIRC5/XIAP/BAD/CAV1/SERPINE1/PCOLCE/SERPINB2	9
MF	GO:0004222	metalloendopeptidase activity	6/187	108/18352	0.000836483	0.004591944	0.002815793	MMP1/MMP2/MMP9/MMP13/MMP8/MMP3	6
MF	GO:0030331	estrogen receptor binding	4/187	42/18352	0.000863419	0.004690948	0.002876503	PPARG/ESR1/PCNA/PARP1	4
MF	GO:0005506	iron ion binding	7/187	151/18352	0.00091633	0.004927612	0.003021625	CYP3A4/CYP1A2/CYP1A1/CYP1B1/ALOX5/CYP19A1/SCD	7
MF	GO:0043028	cysteine-type endopeptidase regulator activity involved in	4/187	43/18352	0.000944466	0.005027614	0.003082947	DPEP1/BIRC5/XIAP/BAD	4

MF	GO:0140296	general transcription initiation factor binding	4/187	44/18352	0.001030721	0.005431901	0.003330857	AR/AHR/ESR1/TP53	4
MF	GO:0005123	death receptor binding	3/187	20/18352	0.001044538	0.005450215	0.003342087	CASP3/CASP8/BDNF	3
MF	GO:0008106	alcohol dehydrogenase (NADP+) activity	3/187	21/18352	0.001209536	0.00624927	0.00383207	AKR1C3/AKR1B1/AKR1C1	3
MF	GO:0001102	RNA polymerase II activating transcription factor binding	4/187	47/18352	0.001322515	0.006766653	0.004149331	JUN/RB1/FOS/NFE2L2	4
MF	GO:0019199	transmembrane receptor protein kinase activity	5/187	80/18352	0.00134961	0.00683889	0.004193627	EGFR/ERBB2/MET/KDR/ERBB3	5
MF	GO:0016616	oxidoreductase activity, acting on the CH-OH group of donors, NAD or NADP as acceptor	6/187	120/18352	0.001444393	0.007249478	0.0044454	AKR1C3/AKR1B1/FASN/HMGCR/AKR1C1/ADH1B	6
MF	GO:0030295	protein kinase activator activity	5/187	82/18352	0.001506978	0.007492242	0.004594264	CDKN1A/CCNB1/EGF/IGF2/ERBB3	5
MF	GO:0005179	hormone activity	6/187	122/18352	0.001571901	0.007741979	0.004747403	IGF2/EDN1/INS/GCG/PYY/ADM	6
MF	GO:0070412	R-SMAD binding	3/187	23/18352	0.001586667	0.007742347	0.004747629	JUN/FOS/PARP1	3
MF	GO:0070888	E-box binding	4/187	50/18352	0.001667583	0.008062535	0.004943969	PPARG/AHR/HIF1A/MY	4
MF	GO:0008201	heparin binding	7/187	169/18352	0.001756911	0.0084172	0.005161451	VEGFA/APOB/MPO/CXCL11/CXCL10/PCOLCE/LPL	7
MF	GO:0042887	amide transmembrane transporter activity	4/187	51/18352	0.001795269	0.008468816	0.005193102	MCL1/ABCC1/GJA1/ABCG2	4
MF	GO:0000979	RNA polymerase II core promoter sequence-specific DNA binding	3/187	24/18352	0.001799824	0.008468816	0.005193102	RELA/STAT1/FOS	3
MF	GO:0043027	cysteine-type endopeptidase inhibitor activity involved in	3/187	25/18352	0.002030025	0.009467462	0.005805475	DPEP1/BIRC5/XIAP	3
MF	GO:0019209	kinase activator activity	5/187	89/18352	0.002165192	0.009941473	0.006096139	CDKN1A/CCNB1/EGF/IGF2/ERBB3	5
MF	GO:0016614	oxidoreductase activity, acting on CH-OH group of donors	6/187	130/18352	0.002169392	0.009941473	0.006096139	AKR1C3/AKR1B1/FASN/HMGCR/AKR1C1/ADH	6
MF	GO:0072341	modified amino acid binding	5/187	90/18352	0.00227394	0.010259524	0.006291169	GSTP1/GSTM1/DPEP1/PTGES/FASN	5
MF	GO:0016628	oxidoreductase activity, acting on the CH-CH group of donors, NAD or NADP as acceptor	3/187	26/18352	0.002277731	0.010259524	0.006291169	AKR1C3/FASN/AKR1C1	3
MF	GO:0005080	protein kinase C binding	4/187	55/18352	0.002373986	0.010602463	0.006501461	AKT1/TOP2A/PRKCB/HSPB1	4
MF	GO:0016765	transferase activity, transferring alkyl or aryl (other than methyl)	4/187	56/18352	0.00253652	0.011169699	0.006849291	GSTP1/GSTM1/GSTA1/GSTA2	4
MF	GO:0004033	aldo-keto reductase (NADP) activity	3/187	27/18352	0.002543385	0.011169699	0.006849291	AKR1C3/AKR1B1/AKR1C1	3

MF	GO:0005539	glycosaminoglycan binding	8/187	232/18352	0.002660901	0.011589212	0.007106537	TNFAIP6/VEGFA/APOB/MPO/CXCL11/CXCL10/PCOLCE/LPL	8
MF	GO:0004806	triglyceride lipase activity	3/187	29/18352	0.003130213	0.013521492	0.008291417	PLB1/CES1/LPL	3
MF	GO:0070851	growth factor receptor binding	6/187	141/18352	0.003252673	0.013868388	0.008504135	IL6R/VEGFA/IL4/EGF/IL1B/IL1A	6
MF	GO:0005507	copper ion binding	4/187	60/18352	0.00326315	0.013868388	0.008504135	TP53/TYR/SOD1/IL1A	4
MF	GO:0052689	carboxylic ester hydrolase activity	6/187	143/18352	0.003486445	0.014698853	0.009013378	PON1/ACHE/CA2/PLB1/CES1/LPL	6
MF	GO:0005164	tumor necrosis factor receptor	3/187	31/18352	0.003793678	0.015619283	0.009577788	CASP8/STAT1/CD40LG	3
MF	GO:0030291	protein serine/threonine kinase inhibitor activity	3/187	31/18352	0.003793678	0.015619283	0.009577788	CASP3/CDKN1A/HSPB1	3
MF	GO:1904680	peptide transmembrane transporter activity	3/187	31/18352	0.003793678	0.015619283	0.009577788	MCL1/ABCC1/GJA1	3
MF	GO:0051087	chaperone binding	5/187	104/18352	0.004245755	0.017345062	0.010636041	BAX/TP53/BIRC5/SOD1/HSPA5	5
MF	GO:0001094	TFIID-class transcription factor complex binding	2/187	10/18352	0.004404127	0.01745094	0.010700966	AHR/TP53	2
MF	GO:0004955	prostaglandin receptor activity	2/187	10/18352	0.004404127	0.01745094	0.010700966	PPARG/PTGER3	2
MF	GO:0016725	oxidoreductase activity, acting on CH or CH2 groups	2/187	10/18352	0.004404127	0.01745094	0.010700966	CYP3A4/CYP1A2	2
MF	GO:0071723	lipopeptide binding	2/187	10/18352	0.004404127	0.01745094	0.010700966	CD14/LBP	2
MF	GO:0004954	prostanoid receptor activity	2/187	11/18352	0.005346878	0.020872626	0.012799153	PPARG/PTGER3	2
MF	GO:1990459	transferrin receptor binding	2/187	11/18352	0.005346878	0.020872626	0.012799153	IKBKB/CHUK	2
MF	GO:0009055	electron transfer activity	5/187	111/18352	0.005590288	0.021662366	0.013283424	CYP1A2/AKR1B1/NCF1/CYP19A1/GSR	5
MF	GO:0005518	collagen binding	4/187	70/18352	0.005671847	0.021817982	0.013378848	ACHE/MMP9/MMP13/PCOLCE	4
MF	GO:0004857	enzyme inhibitor activity	10/187	383/18352	0.006028239	0.023020884	0.014116471	CASP3/SLPI/DPEP1/CDKN1A/BIRC5/XIAP/UGT1A1/HSPB1/SERPINE1/SERPINB2	10
MF	GO:0001098	basal transcription machinery	4/187	72/18352	0.006264957	0.023325031	0.014302975	AR/AHR/ESR1/TP53	4
MF	GO:0001099	basal RNA polymerase II transcription machinery binding	4/187	72/18352	0.006264957	0.023325031	0.014302975	AR/AHR/ESR1/TP53	4
MF	GO:0004861	cyclin-dependent protein serine/threonine kinase inhibitor activity	2/187	12/18352	0.006373443	0.023325031	0.014302975	CASP3/CDKN1A	2
MF	GO:0043176	amine binding	2/187	12/18352	0.006373443	0.023325031	0.014302975	SLC6A4/HTR3A	2
MF	GO:0051378	serotonin binding	2/187	12/18352	0.006373443	0.023325031	0.014302975	SLC6A4/HTR3A	2
MF	GO:0052650	NADP-retinol dehydrogenase	2/187	12/18352	0.006373443	0.023325031	0.014302975	AKR1C3/AKR1B1	2
MF	GO:0001618	virus receptor activity	4/187	74/18352	0.006897897	0.024898573	0.015267876	DPP4/CDK1/ICAM1/EGF	4

MF	GO:0140272	exogenous protein binding	4/187	74/18352	0.006897897	0.024898573	0.015267876	DPP4/CDK1/ICAM1/EGF	4
MF	GO:0016175	superoxide-generating NAD(P)H oxidase activity	2/187	13/18352	0.007482036	0.026823353	0.016448156	NCF1/DUOX2	2
MF	GO:0016504	peptidase activator activity	3/187	40/18352	0.007798875	0.027770319	0.017028838	BAD/CAV1/PCOLCE	3
MF	GO:0050998	nitric-oxide synthase binding	2/187	14/18352	0.008670896	0.030463748	0.018680457	SLC6A4/CAV1	2
MF	GO:1901338	catecholamine binding	2/187	14/18352	0.008670896	0.030463748	0.018680457	ADRA2A/DRD2	2
MF	GO:0030544	Hsp70 protein binding	3/187	42/18352	0.008928489	0.030956011	0.018982314	BAX/CDK1/CYP1A1	3
MF	GO:1901682	sulfur compound transmembrane transporter activity	3/187	42/18352	0.008928489	0.030956011	0.018982314	ABCC1/GJA1/ABCG2	3
MF	GO:0004953	icosanoid receptor activity	2/187	15/18352	0.009938291	0.034009606	0.020854787	PPARG/PTGER3	2
MF	GO:0036041	long-chain fatty acid binding	2/187	15/18352	0.009938291	0.034009606	0.020854787	PPARG/PPARD	2
MF	GO:0008013	beta-catenin binding	4/187	85/18352	0.011137745	0.036477822	0.022368304	AR/ESR1/GSK3B/GJA1	4
MF	GO:0051219	phosphoprotein binding	4/187	85/18352	0.011137745	0.036477822	0.022368304	MAPK1/RB1/MAPK3/TRPV1	4
MF	GO:0004866	endopeptidase inhibitor activity	6/187	183/18352	0.011256069	0.036477822	0.022368304	SLPI/DPEP1/BIRC5/XIAP/SERPINE1/SERPINB2	6
MF	GO:0004697	protein kinase C activity	2/187	16/18352	0.011282514	0.036477822	0.022368304	PRKCA/PRKCB	2
MF	GO:0004698	calcium-dependent protein kinase C activity	2/187	16/18352	0.011282514	0.036477822	0.022368304	PRKCA/PRKCB	2
MF	GO:0005149	interleukin-1 receptor binding	2/187	16/18352	0.011282514	0.036477822	0.022368304	IL1B/IL1A	2
MF	GO:0005159	insulin-like growth factor receptor binding	2/187	16/18352	0.011282514	0.036477822	0.022368304	IGF2/INS	2
MF	GO:0010181	FMN binding	2/187	16/18352	0.011282514	0.036477822	0.022368304	NOS2/NOS3	2
MF	GO:0070402	NADPH binding	2/187	16/18352	0.011282514	0.036477822	0.022368304	FASN/HMGCR	2
MF	GO:0004177	aminopeptidase activity	3/187	46/18352	0.011461716	0.036831246	0.022585025	DPP4/NPEPPS/ENPEP	3
MF	GO:0048020	CCR chemokine receptor binding	3/187	47/18352	0.012153025	0.038816026	0.023802098	STAT1/STAT3/CCL2	3
MF	GO:0004713	protein tyrosine kinase activity	5/187	135/18352	0.012454874	0.039540473	0.02424633	EGFR/ERBB2/MET/KDR/ERBB3	5
MF	GO:0051117	ATPase binding	4/187	88/18352	0.012530295	0.039541709	0.024247088	AR/ESR1/EGFR/CAV1	4
MF	GO:0030414	peptidase inhibitor activity	6/187	189/18352	0.013035265	0.040890384	0.025074099	SLPI/DPEP1/BIRC5/XIAP/SERPINE1/SERPINB2	6
MF	GO:0061135	endopeptidase regulator activity	6/187	192/18352	0.013995016	0.043641263	0.026760946	SLPI/DPEP1/BIRC5/XIAP/SERPINE1/SERPINB2	6
MF	GO:0008227	G protein-coupled amine receptor activity	3/187	51/18352	0.015154002	0.046977406	0.028806678	ADRA1B/ADRA2A/HRH1	3
MF	GO:0030296	protein tyrosine kinase activator activity	2/187	19/18352	0.015759483	0.048286322	0.02960931	EGF/ERBB3	2
MF	GO:0050664	oxidoreductase activity, acting on NAD(P)H, oxygen as acceptor	2/187	19/18352	0.015759483	0.048286322	0.02960931	NCF1/DUOX2	2
MF	GO:0008238	exopeptidase activity	4/187	95/18352	0.016198996	0.049346073	0.030259152	DPP4/DPEP1/NPEPPS/ENPEP	4

MF	GO:0005516	calmodulin binding	6/187	200/18352	0.016793555	0.050863239	0.031189482	NOS2/AKT1/EGFR/NOS3/TRPV1/CACNA1S	6
MF	GO:0004896	cytokine receptor activity	4/187	97/18352	0.017358279	0.051499378	0.031579564	IL6R/IL10RA/IL2RA/F3	4
MF	GO:0001972	retinoic acid binding	2/187	20/18352	0.017394477	0.051499378	0.031579564	RXRA/UGT1A1	2
MF	GO:0004745	retinol dehydrogenase activity	2/187	20/18352	0.017394477	0.051499378	0.031579564	AKR1C3/ADH1B	2
MF	GO:0072349	modified amino acid transmembrane transporter activity	2/187	20/18352	0.017394477	0.051499378	0.031579564	ABCC1/GJA1	2
MF	GO:0004602	glutathione peroxidase activity	2/187	21/18352	0.019098155	0.056227528	0.03447888	GSTP1/GSTA1	2
MF	GO:0001085	RNA polymerase II transcription factor binding	3/187	56/18352	0.019442034	0.056921954	0.034904704	AR/GSK3B/TP53	3
MF	GO:0043621	protein self-association	3/187	57/18352	0.020371784	0.058988626	0.036171993	PPARG/ACHE/TP53	3
MF	GO:0050840	extracellular matrix binding	3/187	57/18352	0.020371784	0.058988626	0.036171993	ACHE/VEGFA/SPP1	3
MF	GO:0004869	cysteine-type endopeptidase inhibitor activity	3/187	59/18352	0.02230365	0.063880562	0.039171742	DPEP1/BIRC5/XIAP	3
MF	GO:0016627	oxidoreductase activity, acting on the CH-CH group of donors	3/187	59/18352	0.02230365	0.063880562	0.039171742	AKR1C3/FASN/AKR1C1	3
MF	GO:0005158	insulin receptor binding	2/187	23/18352	0.022705372	0.064331888	0.039448496	IGF2/INS	2
MF	GO:0009931	calcium-dependent protein serine/threonine kinase activity	2/187	23/18352	0.022705372	0.064331888	0.039448496	PRKCA/PRKCB	2
MF	GO:0047485	protein N-terminus binding	4/187	107/18352	0.023923838	0.067421725	0.041343193	RELA/TP53/MDM2/PARP1	4
MF	GO:0016655	oxidoreductase activity, acting on NAD(P)H, quinone or similar compound as acceptor	3/187	61/18352	0.024332041	0.067891607	0.041631326	AKR1C3/AKR1C1/NQO1	3
MF	GO:0004709	MAP kinase kinase kinase activity	2/187	24/18352	0.024605876	0.067891607	0.041631326	EGFR/RAF1	2
MF	GO:0010857	calcium-dependent protein kinase activity	2/187	24/18352	0.024605876	0.067891607	0.041631326	PRKCA/PRKCB	2
MF	GO:0070006	metalloaminopeptidase activity	2/187	24/18352	0.024605876	0.067891607	0.041631326	NPEPPS/ENPEP	2
MF	GO:0008083	growth factor activity	5/187	162/18352	0.025266297	0.069350722	0.042526059	VEGFA/IL4/EGF/IGF2/BDNF	5
MF	GO:0004190	aspartic-type endopeptidase activity	2/187	25/18352	0.026568994	0.072174535	0.04425763	CASP3/CTSD	2
MF	GO:0016702	oxidoreductase activity, acting on single donors with incorporation of molecular oxygen, incorporation of two atoms of oxygen	2/187	25/18352	0.026568994	0.072174535	0.04425763	PTGS2/ALOX5	2
MF	GO:0008235	metalloexopeptidase activity	3/187	64/18352	0.027555415	0.074470277	0.045665385	DPEP1/NPEPPS/ENPEP	3
MF	GO:0016701	oxidoreductase activity, acting on single donors with incorporation of molecular oxygen	2/187	26/18352	0.028593266	0.075946195	0.046570422	PTGS2/ALOX5	2
MF	GO:0017025	TBP-class protein binding	2/187	26/18352	0.028593266	0.075946195	0.046570422	AHR/ESR1	2
MF	GO:0070001	aspartic-type peptidase activity	2/187	26/18352	0.028593266	0.075946195	0.046570422	CASP3/CTSD	2
MF	GO:0004860	protein kinase inhibitor activity	3/187	65/18352	0.028677975	0.075946195	0.046570422	CASP3/CDKN1A/HSPB1	3

MF	GO:0004197	cysteine-type endopeptidase activity	4/187	114/18352	0.02930434	0.077216936	0.047349644	CASP9/CASP3/CASP8/CASP7	4
MF	GO:0001968	fibronectin binding	2/187	27/18352	0.030677256	0.079639971	0.048835456	VEGFA/IGFBP3	2
MF	GO:0042910	xenobiotic transmembrane transporter activity	2/187	27/18352	0.030677256	0.079639971	0.048835456	ABCC1/ABCG2	2
MF	GO:0044183	protein folding chaperone	2/187	27/18352	0.030677256	0.079639971	0.048835456	HSPA5/HSPB1	2
