# Supplementary material for: Analysis of the Efficacy and Pharmacological Mechanisms of Action of Zhenren Yangzang Decoction on Ulcerative Colitis Using Meta-Analysis and Network Pharmacology
Source: Evid Based Complement Alternat Med. 2021 Dec 28;2021:4512755. doi: 10.1155/2021/4512755 (PMC8727130; doi:10.1155/2021/4512755)
Supplement: Supplementary Materials — Figure S1: Risk of bias graph. Figure S2: risk of bias summary. Figure S3: forest plot of comparison of serum cytokines. Figure S4: forest plot of comparison of the total syndrome score of TCM. Table S1: basic information on the active compounds in ZRYZD. Table S2: gene symbols and entrezID of active target genes. Table S3: compounds ranked by the degree in the network. Supplementary File 1: compounds of ZRYZD from TCMSP. Supplementary File 2: corresponding target genes of ZRYZD. Supplementary File 3: UC-related target genes. Supplementary File 4: GO functional enrichment analysis. Supplementary File 5: KEGG pathway enrichment analysis. Supplementary File 6: data of compound-target networks. Supplementary File 7: data of key compound-target networks. Supplementary File 8: data of PPI network. [file 4512755.f1.zip › 4512755.f1/Supplementary File 5 KEGG pathway enrichment analysis (1).pdf]

Supplementary File 5 KEGG pathway enrichment analysis

ID	Description	GeneRatio	BgRatio	pvalue	p.adjust	qvalue	geneID	Count
hsa05417	Lipid and atherosclerosis	46/175	215/8085	6.01E-34	1.65E-31	6.14E-32	CD14/LBP/BCL2/BAX/CASP9/JUN/CASP3/CASP8/PRKCA/PPARG/RELA/IKBKB/AKT1/MAPK8/MMP1/CYP1A1/ICAM1/SELE/VCAM1/RXRA/MAPK14/GSK3B/BCL2L1/MMP9/MAPK1/TP53/NFKBIA/CASP7/CD40LG/MAPK10/NCF1/MAPK3/BAD/APOB/RXRB/STAT3/MMP3/FOS/HSPA5/IL1B/CCL2/CXCL8/NOS3/N	46
hsa04933	AGE-RAGE signaling pathway in diabetic complications	32/175	100/8085	1.22E-29	1.67E-27	6.23E-28	BCL2/BAX/JUN/CASP3/PRKCA/RELA/AKT1/MAPK8/STAT1/ICAM1/SELE/VCAM1/MAPK14/VEGFA/CCND1/MMP2/MAPK1/CDK4/MAPK10/MAPK3/STAT3/F3/IL1B/CCL2/CXCL8/PRKCB/NOS3/THBD/SERPI	32

hsa05418	Fluid shear stress and atherosclerosis	34/175	139/8085	4.30E-27	3.93E-25	1.46E-25	BCL2/JUN/RELA/IKBKB/AKT1/MAPK8/HMOX1/ICAM1/SELE/VCAM1/GSTP1/GSTM1/MAPK14/VEGFA/MMP2/MMP9/TP53/IFNG/KDR/MAPK10/NCF1/FOS/CAV1/IL1B/CCL2/NOS3/THBD/IL1A/NFE2L2/NQO	34
hsa05161	Hepatitis B	34/175	162/8085	1.03E-24	7.09E-23	2.64E-23	BCL2/BAX/CASP9/JUN/CASP3/CASP8/PRKCA/RELA/IKBKB/AKT1/MAPK8/STAT1/MAPK14/CDK2/CCNA2/CDKN1A/MMP9/MAPK1/RB1/TP53/NFKBIA/PCNA/BIRC5/MAPK10/MAPK3/BAD/STAT3/FOS/RAF1/MYC/CXCL8/P	34
hsa05215	Prostate cancer	28/175	97/8085	1.41E-24	7.74E-23	2.89E-23	BCL2/CASP9/[illegible]/RELA/IKBKB/AKT1/GSTP1/PLAU/GSK3B/CDK2/EGFR/CCND1/CDKN1A/MMP9/MAPK1/RB1/TP53/NFKBIA/MDM2/ERBB2/MAPK3/BAD/MMP3/EGF/RAF1/CHUK/E2F1/INS	28

hsa05207	Chemical carcinogenesis - receptor activation	37/175	212/8085	7.18E-24	3.28E-22	1.22E-22	BCL2/JUN/PRKCA/AR/RELA/AKT1/CYP3A4/CYP1A2/CYP1A1/CYP1B1/AHR/GSTM1/ESR1/RXRA/ESR2/EGFR/VEGFA/CCND1/MAPK1/RB1/BIRC5/XIAP/MAPK3/BAD/UGT1A1/PPARA/RXRB/STAT3/FOS/EGF/RAF1/MYC/PRKCB/E2F1/GST	37
hsa05212	Pancreatic cancer	25/175	76/8085	1.29E-23	5.06E-22	1.89E-22	BAX/CASP9/RELA/IKBKB/AKT1/MAPK8/STAT1/EGFR/VEGFA/CCND1/BCL2L1/CDKN1A/MAPK1/RB1/CDK4/TP53/ERBB2/MAPK10/MAPK3/BAD/STAT3/EGF/RAF1/CHUK/	25
hsa05219	Bladder cancer	20/175	41/8085	3.08E-23	1.06E-21	3.93E-22	MMP1/EGFR/VEGFA/CCND1/CDKN1A/MMP2/MMP9/MAPK1/RB1/CDK4/TP53/MDM2/ERBB2/MAPK3/EGF/RAF1/MYC/CXCL8/RASSF1/E	20

hsa04657	IL-17 signaling pathway	26/175	94/8085	2.47E-22	7.51E-21	2.80E-21	PTGS2/JUN/CASP3/CASP8/RELA/IKBKB/MAPK8/MMP1/MAPK14/GSK3B/MMP9/MAPK1/NFKBIA/IFNG/IL4/MMP13/MAPK10/MAPK3/MMP3/FOS/IL1B/CCL2/CXCL8/CXCL2/CXCL10/CHUK	26
hsa05160	Hepatitis C	31/175	157/8085	1.02E-21	2.81E-20	1.05E-20	BAX/CASP9/CASP3/CASP8/RELA/IKBKB/AKT1/STAT1/RXRA/GSK3B/CDK2/EGFR/CCND1/CDKN1A/MAPK1/RB1/CDK4/TP53/NFKBIA/IFNG/MAPK3/BAD/PPARA/STAT3/EGF/RAF1/MYC/C	31
hsa05223	Non-small cell lung cancer	23/175	72/8085	1.87E-21	4.57E-20	1.70E-20	BAX/CASP9/PRKCA/AKT1/RXRA/EGFR/CCND1/CDKN1A/MAPK1/RB1/CDK4/TP53/ERBB2/MET/MAPK3/BAD/RXRB/STAT3/EGF/RAF1/PRKCB/RASSF1/	23

hsa04668	TNF signaling pathway	27/175	112/8085	2.00E-21	4.57E-20	1.70E-20	PTGS2/JUN/CASP3/CASP8/RELA/IKBKB/AKT1/MAPK8/ICAM1/SELE/VCAM1/MAPK14/MMP9/MAPK1/NFKBIA/CASP7/MAPK10/MAPK3/MMP3/FOS/IL1B/CCL2/CXCL2/CXCL10/CHUK/IRF1	27
hsa05167	Kaposi sarcoma-associated herpesvirus infection	33/175	194/8085	6.06E-21	1.28E-19	4.76E-20	PTGS2/BAX/CASP9/JUN/CASP3/CASP8/RELA/IKBKB/AKT1/MAPK8/STAT1/ICAM1/MAPK14/GSK3B/VEGFA/CCND1/CDKN1A/MAPK1/RB1/CDK4/TP53/NFKBIA/MAPK10/MAPK3/STAT3/FOS/RAF1/HIF1A	33
hsa01522	Endocrine resistance	25/175	98/8085	1.50E-20	2.94E-19	1.10E-19	BCL2/BAX/JUN/AKT1/MAPK8/ESR1/ESR2/MAPK14/EGFR/CCND1/CDKN1A/MMP2/MMP9/MAPK1/RB1/CDK4/TP53/MDM2/ERBB2/MAPK10/MAPK3/BAD/FOS/RAF1/E2F1	25

hsa05208	Chemical carcinogenesis - reactive oxygen species	34/175	223/8085	5.49E-20	1.00E-18	3.74E-19	JUN/RELA/IKBKB/AKT1/MAPK8/HMOX1/CYP1A2/CYP1A1/CYP1B1/AHR/GSTM1/AKR1C3/CAT/MAPK14/EGFR/VEGFA/MAPK1/NFKBIA/MET/MAPK10/NCF1/MAPK3/BAD/SOD1/AKR1C1/FOS/EGF/RAF1/HIF1A/NFE2L2/NQ	34
hsa05163	Human cytomegalovirus infection	34/175	225/8085	7.36E-20	1.26E-18	4.70E-19	IL6R/PTGS2/BAX/CASP9/CASP3/CASP8/PRKCA/RELA/IKBKB/AKT1/MAPK14/GSK3B/EGFR/VEGFA/CCND1/CDKN1A/MAPK1/IL10RA/RB1/CDK4/TP53/NFKBIA/MDM2/MAPK3/STAT3/RAF1/MYC/IL1B/CCL2/PTGER3/CXCL8/	34
hsa05222	Small cell lung cancer	23/175	92/8085	9.36E-19	1.51E-17	5.62E-18	PTGS2/BCL2/BAX/CASP9/CASP3/NOS2/RELA/IKBKB/AKT1/RXRA/CDK2/CCND1/BCL2L1/CDKN1A/RB1/CDK4/TP53/NFKBIA/XIAP/RXRB/MYC/CHUK/	23

hsa01524	Platinum drug resistance	21/175	73/8085	1.35E-18	2.05E-17	7.65E-18	BCL2/BAX/CASP9/CASP3/CASP8/AKT1/GSTP1/GSTM1/BCL2L1/CDKN1A/MAPK1/TP53/MDM2/ERBB2/BIRC5/TOP2A/XIAP/MAPK3/BAD/GSTA1/GSTA2	21
hsa04210	Apoptosis	26/175	136/8085	6.06E-18	8.73E-17	3.25E-17	BCL2/BAX/CASP9/JUN/CASP3/CASP8/RELA/IKBKB/AKT1/MAPK8/BCL2L1/MAPK1/TP53/NFKBIA/CASP7/MCL1/BIRC5/XIAP/MAPK10/MAPK3/BAD/FOS/RAF1/PARP1/C	26
hsa05145	Toxoplasmosis	24/175	112/8085	7.65E-18	1.05E-16	3.91E-17	BCL2/CASP9/CASP3/CASP8/NOS2/RELA/IKBKB/AKT1/MAPK8/STAT1/ALOX5/MAPK14/BCL2L1/MAPK1/IL10RA/NFKBIA/IFNG/XIAP/CD40LG/MAPK10/MAPK3/BAD/ST	24
hsa05162	Measles	26/175	139/8085	1.07E-17	1.40E-16	5.22E-17	BCL2/BAX/CASP9/JUN/CASP3/CASP8/RELA/IKBKB/AKT1/MAPK8/STAT1/GSK3B/CDK2/CCND1/BCL2L1/CDK4/TP53/NFKBIA/IL2RA/MAPK10/BAD/STAT3/FOS/IL1B/IL	26

hsa04066	HIF-1 signaling pathway	23/175	109/8085	5.62E-17	6.70E-16	2.50E-16	IL6R/BCL2/PRKCA/NOS2/RELA/AKT1/HMOX1/EGFR/VEGFA/CDKN1A/MAPK1/ERBB2/IFNG/MAPK3/STAT3/EGF/HIF1A/PRKCB/NOS3/SERPINE1/HK2/ED	23
hsa05210	Colorectal cancer	21/175	86/8085	5.62E-17	6.70E-16	2.50E-16	BCL2/BAX/CASP9/JUN/CASP3/AKT1/MAPK8/GSK3B/EGFR/CCND1/CDKN1A/MAPK1/TP53/BIRC5/MAPK10/MAPK3/BAD/FOS/EGF/RAF1	21
hsa04151	PI3K-Akt signaling pathway	38/175	354/8085	6.45E-17	7.36E-16	2.74E-16	IL6R/BCL2/CASP9/PRKCA/RELA/IKBKB/AKT1/RXRA/GSK3B/CDK2/EGFR/VEGFA/CCND1/BCL2L1/CDKN1A/MAPK1/CDK4/TP53/MDM2/ERBB2/MCL1/IL2RA/IL4/MET/KDR/MAPK3/BAD/EGF/RAF1/MYC/NOS3/COL1A1/CHUK/SPP1/IGF2/	38

hsa05225	Hepatocellular carcinoma	27/175	168/8085	1.38E-16	1.51E-15	5.64E-16	BAX/PRKCA/AKT1/HMOX1/GSTP1/GSTM1/GSK3B/EGFR/CCND1/BCL2L1/CDKN1A/MAPK1/RB1/CDK4/TP53/MET/MAPK3/BAD/RAF1/MYC/PRKCB/NFE2L2/NQO1/E2F1/IGF2/GSTA1/GSTA2	27
hsa01521	EGFR tyrosine kinase inhibitor resistance	20/175	79/8085	1.50E-16	1.58E-15	5.89E-16	IL6R/BCL2/BAX/PRKCA/AKT1/GSK3B/EGFR/VEGFA/BCL2L1/MAPK1/ERBB2/MET/KDR/MAPK3/BAD/STAT3/EGF/RAF1/PRKCB/ERBB3	20
hsa04659	Th17 cell differentiation	22/175	108/8085	6.20E-16	6.29E-15	2.35E-15	IL6R/JUN/RELA/IKBKB/MAPK8/STAT1/AHR/RXRA/MAPK14/MAPK1/NFKBIA/IL2RA/IFNG/IL4/MAPK10/MAPK3/RXRB/STAT3/FOS/HIF1A/IL1B/CHUK	22
hsa05220	Chronic myeloid leukemia	19/175	76/8085	1.16E-15	1.13E-14	4.22E-15	BAX/RELA/IKBKB/AKT1/CCND1/BCL2L1/CDKN1A/MAPK1/RB1/CDK4/TP53/NFKBIA/MDM2/MAPK3/BAD/RAF1/MYC/CHUK/E2F1	19

hsa05169	Epstein-Barr virus infection	28/175	202/8085	1.90E-15	1.79E-14	6.67E-15	BCL2/BAX/CASP9/JUN/CASP3/CASP8/RELA/IKBKB/AKT1/MAPK8/STAT1/ICAM1/MAPK14/CDK2/CCNA2/CCND1/CDKN1A/RB1/CDK4/TP53/NFKBIA/MDM2/MAPK10/STAT3/MYC/CXCL10	28
hsa04010	MAPK signaling pathway	33/175	294/8085	2.62E-15	2.39E-14	8.90E-15	CD14/JUN/CASP3/PRKCA/RELA/IKBKB/AKT1/MAPK8/MAPK14/EGFR/VEGFA/MAPK1/TP53/ERBB2/MET/KDR/MAPK10/MAPK3/FOS/EGF/RAF1/MYC/IL1B/PRKCB/HSPB1/IL1A/CHUK/IGF2/ERBB3/RASGRF	33
hsa05205	Proteoglycans in cancer	28/175	205/8085	2.80E-15	2.47E-14	9.21E-15	CASP3/PRKCA/AKT1/ESR1/PLAU/MAPK14/EGFR/VEGFA/CCND1/CDKN1A/MMP2/MMP9/MAPK1/TP53/MDM2/ERBB2/MET/KDR/MAPK3/STAT3/RAF1/HIF1A/CAV1/MYC/PRKCB/COL1A1/I	28

hsa04620	Toll-like receptor signaling pathway	21/175	104/8085	3.58E-15	3.07E-14	1.14E-14	CD14/LBP/JUN/CASP8/RELA/IKBKB/AKT1/MAPK8/STAT1/MAPK14/MAPK1/NFKBIA/MAPK10/MAPK3/FOS/IL1B/CXCL8/CXCL11/CXCL10/CHUK/SPP1	21
hsa04115	p53 signaling pathway	18/175	73/8085	8.84E-15	7.34E-14	2.74E-14	BCL2/BAX/CASP9/CASP3/CASP8/CDK1/CDK2/CHEK1/CCND1/BCL2L1/CDKN1A/CDK4/TP53/MDM2/CCNB1/SERPINE1/CHEK2/IGFBP3	18
hsa04218	Cellular senescence	24/175	156/8085	2.14E-14	1.73E-13	6.44E-14	RELA/AKT1/CDK1/MAPK14/CDK2/CHEK1/CCNA2/CCND1/CDKN1A/MAPK1/RB1/CDK4/TP53/MDM2/CCNB1/MAPK3/RAF1/MYC/CXCL8/SERPINE1/IL1A/CHEK2/E2F1/IGF	24
hsa04926	Relaxin signaling pathway	22/175	129/8085	3.12E-14	2.44E-13	9.11E-14	JUN/PRKCA/NOS2/RELA/AKT1/MAPK8/MMP1/MAPK14/EGFR/VEGFA/MMP2/MMP9/MAPK1/NFKBIA/MMP13/MAPK10/MAPK3/FOS/RAF1/NOS3/COL1A1/	22

hsa04917	Prolactin signaling pathway	17/175	70/8085	6.70E-14	5.10E-13	1.90E-13	RELA/AKT1/MAPK8/STAT1/ESR1/ESR2/MAPK14/GSK3B/CCND1/MAPK1/MAPK10/MAPK3/STAT3/FOS/RAF1/IRF1/INS	17
hsa05166	Human T-cell leukemia virus 1 infection	27/175	219/8085	1.20E-13	8.91E-13	3.32E-13	BAX/JUN/RELA/IKBKB/AKT1/MAPK8/ICAM1/CDK2/CHEK1/CCNA2/CCND1/BCL2L1/CDKN1A/MAPK1/RB1/CDK4/TP53/NFKBIA/IL2RA/XIAP/MAPK10/MAPK3/FOS/MYC/CHEK2/CHUK/E2F	27
hsa05164	Influenza A	24/175	172/8085	2.00E-13	1.44E-12	5.37E-13	BAX/CASP9/CASP3/CASP8/PRKCA/PRSS1/RELA/IKBKB/AKT1/STAT1/ICAM1/MAPK1/CDK4/NFKBIA/IFNG/MAPK3/RAF1/IL1B/CCL2/CXCL8/PRKCB/IL1A/CXCL10/CHUK	24
hsa05142	Chagas disease	19/175	102/8085	3.73E-13	2.62E-12	9.78E-13	JUN/CASP8/NOS2/RELA/IKBKB/AKT1/MAPK8/MAPK14/MAPK1/NFKBIA/IFNG/MAPK10/MAPK3/FOS/IL1B/CCL2/CXCL8/SERPINE1/CHU	19

hsa05221	Acute myeloid leukemia	16/175	67/8085	5.02E-13	3.44E-12	1.28E-12	CD14/RELA/IKBKB/AKT1/CCNA2/CCND1/MAPK1/PPARD/MAPK3/BAD/STAT3/RAF1/RUNX1T1/MYC/MPO/CHUK	16
hsa04064	NF-kappa B signaling pathway	19/175	104/8085	5.40E-13	3.61E-12	1.34E-12	CD14/LBP/PTGS2/BCL2/RELA/IKBKB/ICAM1/VCAM1/PLAU/BCL2L1/NFKBIA/XIAP/CD40LG/IL1B/CXCL8/PRKCB/PARP1/CXCL2/CHUK	19
hsa05213	Endometrial cancer	15/175	58/8085	7.78E-13	5.07E-12	1.89E-12	BAX/CASP9/AKT1/GSK3B/EGFR/CCND1/CDKN1A/MAPK1/TP53/ERBB2/MAPK3/BAD/EGF/RAF1/MY	15
hsa05218	Melanoma	16/175	72/8085	1.67E-12	1.07E-11	3.98E-12	BAX/AKT1/EGFR/CCND1/CDKN1A/MAPK1/RB1/CDK4/TP53/MDM2/MET/MAPK3/BAD/EGF/RAF1/E2F	16
hsa04012	ErbB signaling pathway	17/175	85/8085	2.05E-12	1.28E-11	4.76E-12	JUN/PRKCA/AKT1/MAPK8/GSK3B/EGFR/CDKN1A/MAPK1/ERBB2/MAPK10/MAPK3/BAD/EGF/RAF1/MYC/PRKCB/ER	17

hsa05170	Human immunodeficiency virus 1 infection	25/175	212/8085	2.93E-12	1.79E-11	6.65E-12	BCL2/BAX/CASP9/JUN/CASP3/CASP8/PRKCA/RELA/IKBKB/AKT1/MAPK8/CDK1/MAPK14/CHEK1/BCL2L1/MAPK1/NFKBIA/CCNB1/MAPK10/MAPK3/BAD/FOS/RAF1/PP	25
hsa05214	Glioma	16/175	75/8085	3.29E-12	1.96E-11	7.30E-12	BAX/PRKCA/AKT1/EGFR/CCND1/CDKN1A/MAPK1/RB1/CDK4/TP53/MDM2/MAPK3/EGF/RAF1/PRKCB/E2F1	16
hsa05133	Pertussis	16/175	76/8085	4.09E-12	2.38E-11	8.88E-12	CD14/JUN/CASP3/NOS2/RELA/MAPK8/MAPK14/MAPK1/CASP7/MAPK10/MAPK3/FOS/IL1B/CXCL8/IL1A/IRF1	16
hsa05152	Tuberculosis	23/175	180/8085	4.36E-12	2.49E-11	9.28E-12	CD14/LBP/BCL2/BAX/CASP9/CASP3/CASP8/NOS2/RELA/AKT1/MAPK8/STAT1/MAPK14/MAPK1/IL10RA/IFNG/MAPK10/MAPK3/BAD/RAF1/IL1B/IL1A/CT	23
hsa05235	PD-L1 expression and PD-1 checkpoint pathway in cancer	17/175	89/8085	4.51E-12	2.52E-11	9.40E-12	JUN/RELA/IKBKB/AKT1/STAT1/MAPK14/EGFR/MAPK1/NFKBIA/IFNG/MAPK3/STAT3/FOS/EGF/RAF1/HIF1A/CHUK	17

hsa05140	Leishmaniasis	16/175	77/8085	5.06E-12	2.77E-11	1.03E-11	PTGS2/JUN/NOS2/RELA/STAT1/MAPK14/MAPK1/NFKBIA/IFNG/IL4/NCF1/MAPK3/FOS/IL1B/PRKCB/IL	16
hsa04625	C-type lectin receptor signaling pathway	18/175	104/8085	5.98E-12	3.15E-11	1.17E-11	PTGS2/JUN/CASP8/RELA/IKBKB/AKT1/MAPK8/STAT1/MAPK14/MAPK1/NFKBIA/MDM2/MAPK10/MAPK3/RAF1/IL1B/CHUK/IRF1	18
hsa04660	T cell receptor signaling pathway	18/175	104/8085	5.98E-12	3.15E-11	1.17E-11	JUN/RELA/IKBKB/AKT1/MAPK8/MAPK14/GSK3B/MAPK1/CDK4/NFKBIA/IFNG/IL4/CD40LG/MAPK10/MAPK3/FOS/RAF	18
hsa04510	Focal adhesion	24/175	201/8085	6.35E-12	3.28E-11	1.22E-11	BCL2/JUN/PRKCA/AKT1/MAPK8/GSK3B/EGFR/VEGFA/CCND1/MAPK1/ERBB2/XIAP/MET/KDR/MAPK10/MAPK3/BAD/EGF/RAF1/CAV1/PRKCB/COL1A1/SPP1/RASGRF1	24
hsa04932	Non-alcoholic fatty liver disease	21/175	155/8085	1.28E-11	6.50E-11	2.42E-11	IL6R/BAX/JUN/CASP3/CASP8/PPARG/RELA/IKBKB/AKT1/MAPK8/RXRA/MAPK14/GSK3B/CASP7/MAPK10/PPARA/FOS/IL1B/CXCL8/IL1	21

hsa04370	VEGF signaling pathway	14/175	59/8085	1.68E-11	8.35E-11	3.11E-11	PTGS2/CASP9/PRKCA/AKT1/MAPK14/VEGFA/MAPK1/KDR/MAPK3/BAD/RAF1/PRKCB/NOS3/HSPB1	14
hsa04380	Osteoclast differentiation	19/175	128/8085	2.52E-11	1.23E-10	4.59E-11	JUN/PPARG/RELA/IKBKB/AKT1/MAPK8/STAT1/MAPK14/MAPK1/NFKBIA/IFNG/MAPK10/NCF1/MAPK3/FOSL2/FOS/IL1B/IL1A/CHUK	19
hsa04215	Apoptosis - multiple species	11/175	32/8085	3.10E-11	1.49E-10	5.56E-11	BCL2/BAX/CASP9/CASP3/CASP8/MAPK8/BCL2L1/CASP7/BIRC5/XIAP/MAPK10	11
hsa05224	Breast cancer	20/175	147/8085	3.77E-11	1.78E-10	6.62E-11	BAX/JUN/AKT1/ESR1/ESR2/GSK3B/EGFR/CCND1/CDKN1A/MAPK1/RB1/CDK4/TP53/ERBB2/MAPK3/FOS/EGF/RAF1/MYC/E2F1	20
hsa04068	FoxO signaling pathway	19/175	131/8085	3.83E-11	1.78E-10	6.62E-11	IKBKB/AKT1/MAPK8/CAT/MAPK14/CDK2/EGFR/CCND1/CDKN1A/MAPK1/MDM2/CCNB1/MAPK10/MAPK3/STAT3/EGF/RAF1/CHUK/IN	19

hsa05226	Gastric cancer	20/175	149/8085	4.85E-11	2.21E-10	8.25E-11	BCL2/BAX/AKT1/RXRA/GSK3B/CDK2/EGFR/CCND1/CDKN1A/MAPK1/RB1/TP53/ERBB2/MET/MAPK3/RXRB/EGF/RAF1/MYC/E2F1	20
hsa05165	Human papillomavirus infection	29/175	331/8085	8.07E-11	3.62E-10	1.35E-10	PTGS2/BAX/CASP3/CASP8/RELA/IKBKB/AKT1/STAT1/GSK3B/CDK2/CCNA2/EGFR/VEGFA/CCND1/CDKN1A/MAPK1/RB1/CDK4/TP53/MDM2/MAPK3/BAD/EGF/RAF1/COL1A1/CHUK/SPP1/	29
hsa04919	Thyroid hormone signaling pathway	18/175	121/8085	8.37E-11	3.70E-10	1.38E-10	CASP9/PRKCA/AKT1/STAT1/ESR1/RXRA/GSK3B/CCND1/MAPK1/TP53/MDM2/MAPK3/BAD/RXRB/RAF1/HIF1A/MYC/P	18
hsa05206	MicroRNAs in cancer	28/175	310/8085	8.72E-11	3.79E-10	1.41E-10	PTGS2/BCL2/CASP3/PRKCA/IKBKB/HMOX1/CYP1B1/PLAU/EGFR/VEGFA/CCND1/CDKN1A/MMP9/MAPK1/TP53/MDM2/ERBB2/MCL1/MET/MAPK3/ABCC1/STAT3/RAF1/MYC/PRKCB/RA	28

hsa04722	Neurotrophin signaling pathway	17/175	119/8085	5.53E-10	2.37E-09	8.82E-10	BCL2/BAX/JUN/RELA/IKBKB/AKT1/MAPK8/MAPK14/GSK3B/MAPK1/TP53/NFKBIA/MAPK10/MAPK3/BAD/RAF1/BDN	17
hsa05202	Transcriptional misregulation in cancer	21/175	192/8085	7.65E-10	3.23E-09	1.20E-09	CD14/BAX/PPARG/RELA/RXRA/PLAU/CCNA2/BCL2L1/CDKN1A/MMP9/TP53/MDM2/MET/RXRB/MMP3/RUNX1T1/MYC/CXCL8/MPO/RUNX2/IGFBP3	21
hsa04014	Ras signaling pathway	23/175	232/8085	8.03E-10	3.28E-09	1.22E-09	PRKCA/RELA/IKBKB/AKT1/MAPK8/EGFR/VEGFA/BCL2L1/MAPK1/MET/KDR/MAPK10/MAPK3/BAD/EGF/RAF1/PRKCB/CHUK/RASSF1/IGF2/RASGRF1/B	23
hsa05171	Coronavirus disease - COVID-19	23/175	232/8085	8.03E-10	3.28E-09	1.22E-09	IL6R/JUN/PRKCA/RELA/IKBKB/MAPK8/MMP1/STAT1/MAPK14/EGFR/MAPK1/NFKBIA/MAPK10/MAPK3/STAT3/MMP3/FOS/IL1B/CCL2/CXCL8/PRKCB/CXCL10/CHUK	23
hsa04658	Th1 and Th2 cell differentiation	15/175	92/8085	9.04E-10	3.64E-09	1.36E-09	JUN/RELA/IKBKB/MAPK8/STAT1/MAPK14/MAPK1/NFKBIA/IL2RA/IFNG/IL4/MAPK10/MAPK3/FOS/C	15

hsa05120	Epithelial cell signaling in Helicobacter pylori infection	13/175	70/8085	2.38E-09	9.44E-09	3.52E-09	JUN/CASP3/RELA/IKBKB/MAPK8/MAPK14/EGFR/NFKBIA/MET/MAPK10/CXCL8/CXCL2/CHUK	13
hsa05216	Thyroid cancer	10/175	37/8085	3.67E-09	1.44E-08	5.35E-09	BAX/PPARG/RXRA/CCND1/CDKN1A/MAPK1/TP53/MAPK3/RXRB/	10
hsa04071	Sphingolipid signaling pathway	16/175	119/8085	4.53E-09	1.75E-08	6.51E-09	BCL2/BAX/PRKCA/RELA/AKT1/MAPK8/MAPK14/MAPK1/TP53/MAPK10/MAPK3/ABCC1/RAF1/PRKCB/NOS3/CTSD	16
hsa04110	Cell cycle	16/175	124/8085	8.32E-09	3.17E-08	1.18E-08	CDK1/GSK3B/CDK2/CHEK1/CCNA2/CCND1/CDKN1A/RB1/CDK4/TP53/MDM2/PCNA/CCNB1/MYC/CHEK2/E2F1	16
hsa05203	Viral carcinogenesis	20/175	204/8085	1.35E-08	5.06E-08	1.89E-08	BAX/JUN/CASP3/CASP8/RELA/CDK1/CDK2/CHEK1/CCNA2/CCND1/CDKN1A/MAPK1/RB1/CDK4/TP53/NFKBIA/MDM2/MAPK3/BAD/ST	20
hsa05131	Shigellosis	22/175	247/8085	1.43E-08	5.30E-08	1.98E-08	CD14/BCL2/BAX/JUN/RELA/IKBKB/AKT1/MAPK8/MAPK14/GSK3B/EGFR/BCL2L1/MAPK1/TP53/NFKBIA/MDM2/MAPK10/MAPK3/IL1B/CXCL8/CHUK/H	22

hsa04621	NOD-like receptor signaling pathway	19/175	186/8085	1.63E-08	5.94E-08	2.21E-08	BCL2/JUN/CASP8/RELA/IKBKB/MAPK8/STAT1/MAPK14/BCL2L1/MAPK1/NFKBIA/XIAP/MAPK10/MAPK3/IL1B/CCL2/CXCL8/CXCL2/CH	19
hsa05132	Salmonella infection	22/175	249/8085	1.66E-08	5.99E-08	2.23E-08	CD14/BCL2/BAX/JUN/CASP3/CASP8/RELA/IKBKB/AKT1/MAPK8/MAPK14/MAPK1/NFKBIA/CASP7/MAPK10/MAPK3/FOS/RAF1/MYC/IL1B/CXCL8/CHUK	22
hsa04062	Chemokine signaling pathway	19/175	192/8085	2.74E-08	9.74E-08	3.63E-08	RELA/IKBKB/AKT1/STAT1/GSK3B/MAPK1/NFKBIA/NCF1/MAPK3/BAD/STAT3/RAF1/CCL2/CXCL8/PRKCB/CXCL11/CXCL2/CXCL10/C	19
hsa04935	Growth hormone synthesis, secretion and action	15/175	119/8085	3.43E-08	1.21E-07	4.50E-08	PRKCA/AKT1/MAPK8/STAT1/MAPK14/GSK3B/MAPK1/MAPK10/MAPK3/STAT3/FOS/RAF1/PRKCB/IGFBP3/CACNA1S	15
hsa05135	Yersinia infection	16/175	137/8085	3.55E-08	1.23E-07	4.59E-08	JUN/RELA/IKBKB/AKT1/MAPK8/MAPK14/GSK3B/MAPK1/NFKBIA/MAPK10/MAPK3/FOS/IL1B/CCL2/CXCL8/CHUK	16

hsa05130	Pathogenic Escherichia coli infection	19/175	197/8085	4.16E-08	1.43E-07	5.31E-08	BAX/CASP9/JUN/CASP3/CASP8/RELA/IKBKB/MAPK8/MAPK14/MAPK1/NFKBIA/CASP7/MAPK10/MAPK3/FOS/IL1B/CXCL8/CLDN4/CHU	19
hsa04630	JAK-STAT signaling pathway	17/175	162/8085	6.50E-08	2.20E-07	8.19E-08	IL6R/BCL2/AKT1/STAT1/EGFR/CCND1/BCL2L1/CDKN1A/IL10RA/MCL1/IL2RA/IFNG/IL4/STAT3/EGF/RAF1/MYC	17
hsa05231	Choline metabolism in cancer	13/175	98/8085	1.59E-07	5.31E-07	1.98E-07	JUN/PRKCA/AKT1/MAPK8/EGFR/MAPK1/MAPK10/MAPK3/FOS/EGF/RAF1/HIF1A/PRKCB	13
hsa04662	B cell receptor signaling pathway	12/175	82/8085	1.61E-07	5.31E-07	1.98E-07	JUN/RELA/IKBKB/AKT1/GSK3B/MAPK1/NFKBIA/MAPK3/FOS/RAF1/PRKCB/CHUK	12
hsa04920	Adipocytokine signaling pathway	11/175	69/8085	2.19E-07	7.14E-07	2.66E-07	RELA/IKBKB/AKT1/MAPK8/RXRA/NFKBIA/MAPK10/PPARA/RXRB/STAT3/CHUK	11
hsa04915	Estrogen signaling pathway	15/175	138/8085	2.53E-07	8.13E-07	3.03E-07	OPRM1/BCL2/JUN/AKT1/ESR1/ESR2/EGFR/MMP2/MMP9/MAPK1/MAPK3/FOS/RAF1/NOS3/CTSD	15
hsa05230	Central carbon metabolism in cancer	11/175	70/8085	2.55E-07	8.13E-07	3.03E-07	AKT1/EGFR/MAPK1/TP53/ERBB2/MET/MAPK3/RAF1/HIF1A/MYC/H	11

hsa05134	Legionellosis	10/175	57/8085	3.13E-07	9.85E-07	3.67E-07	CD14/CASP9/CASP3/CASP8/RELA/NFKBIA/CASP7/IL1B/CXCL8/CXC	10
hsa05415	Diabetic cardiomyopathy	18/175	203/8085	3.47E-07	1.08E-06	4.02E-07	PRKCA/RELA/AKT1/MAPK8/MAPK14/GSK3B/MMP2/MMP9/MAPK10/NCF1/PPARA/GSR/PRKCB/NOS3/COL1A1/PARP1/CTSD/INS	18
hsa04928	Parathyroid hormone synthesis, secretion and action	13/175	106/8085	4.05E-07	1.25E-06	4.64E-07	BCL2/PRKCA/RXRA/EGFR/CDKN1A/MAPK1/MMP13/MAPK3/RXRB/FOS/RAF1/PRKCB/RUNX2	13
hsa04912	GnRH signaling pathway	12/175	93/8085	6.58E-07	1.98E-06	7.39E-07	JUN/PRKCA/MAPK8/MAPK14/EGFR/MMP2/MAPK1/MAPK10/MAPK3/RAF1/PRKCB/CACNA1S	12
hsa05323	Rheumatoid arthritis	12/175	93/8085	6.58E-07	1.98E-06	7.39E-07	JUN/MMP1/ICAM1/VEGFA/IFNG/MMP3/FOS/IL1B/CCL2/CXCL8/IL1A/CXCL2	12
hsa00980	Metabolism of xenobiotics by cytochrome P450	11/175	78/8085	7.89E-07	2.35E-06	8.76E-07	CYP3A4/CYP1A2/CYP1A1/CYP1B1/GSTP1/GSTM1/UGT1A1/AKR1C1/GSTA1/GSTA2/ADH1B	11
hsa05144	Malaria	9/175	50/8085	9.92E-07	2.92E-06	1.09E-06	ICAM1/SELE/VCAM1/IFNG/CD40LG/MET/IL1B/CCL2/CXCL8	9

hsa04024	cAMP signaling pathway	18/175	219/8085	1.07E-06	3.11E-06	1.16E-06	JUN/RELA/AKT1/MAPK8/MAPK1/NFKBIA/MAPK10/MAPK3/BAD/PPARA/FOS/RAF1/PTGER3/DRD2/EDN1/BDNF/GCG/CACNA1S	18
hsa04914	Progesterone-mediated oocyte maturation	12/175	100/8085	1.46E-06	4.20E-06	1.56E-06	AKT1/MAPK8/CDK1/MAPK14/CDK2/CCNA2/MAPK1/CCNB1/MAPK10/MAPK3/RAF	12
hsa04664	Fc epsilon RI signaling pathway	10/175	68/8085	1.71E-06	4.89E-06	1.82E-06	PRKCA/AKT1/MAPK8/ALOX5/MAPK14/MAPK1/IL4/MAPK10/MAPK3/RAF1	10
hsa05146	Amoebiasis	12/175	102/8085	1.80E-06	5.09E-06	1.90E-06	CD14/CASP3/PRKCA/NOS2/RELA/IFNG/IL1B/CXCL8/PRKCB/HSPB1/COL1A1/CXCL	12
hsa05204	Chemical carcinogenesis - DNA adducts	10/175	69/8085	1.97E-06	5.45E-06	2.03E-06	PTGS2/CYP3A4/CYP1A2/CYP1A1/CYP1B1/GSTP1/GSTM1/UGT1A1/GSTA1/GSTA2	10
hsa05211	Renal cell carcinoma	10/175	69/8085	1.97E-06	5.45E-06	2.03E-06	JUN/AKT1/VEGFA/CDKN1A/MAPK1/MET/MAPK3/BAD/RAF1/HIF1	10
hsa04622	RIG-I-like receptor signaling pathway	10/175	70/8085	2.25E-06	6.18E-06	2.30E-06	CASP8/RELA/IKBKB/MAPK8/MAPK14/NFKBIA/MAPK10/CXCL8/CXCL10/CHUK	10
hsa04931	Insulin resistance	12/175	108/8085	3.32E-06	9.02E-06	3.36E-06	RELA/IKBKB/AKT1/MAPK8/GSK3B/NFKBIA/MAPK10/PPARA/STAT3/PRKCB/NOS3/IN	12

hsa05022	Pathways of neurodegeneration - multiple diseases	27/175	476/8085	3.38E-06	9.09E-06	3.39E-06	PTGS2/BCL2/BA<br>X/CASP9/CASP3/<br>CASP8/PRKCA/N<br>OS2/RELA/MAPK<br>8/CAT/MAPK14/<br>GSK3B/BCL2L1/<br>MAPK1/CASP7/M<br>APK10/MAPK3/B<br>AD/SOD1/RAF1/<br>HSPA5/IL1B/PRK<br>GR/IL1A/BDNF/C	27
hsa04726	Serotonergic synapse	12/175	115/8085	6.45E-06	1.71E-05	6.39E-06	PTGS1/PTGS2/SL<br>C6A4/CASP3/PR<br>KCA/ALOX5/MA<br>PK1/HTR3A/MAP<br>K3/RAF1/PRKCB/<br>CACNA1S	12
hsa04910	Insulin signaling pathway	13/175	137/8085	7.59E-06	2.00E-05	7.45E-06	IKBKB/AKT1/MA<br>PK8/GSK3B/MAP<br>K1/MAPK10/MAP<br>K3/FASN/BAD/R<br>AF1/ACACA/HK2<br>/INS	13
hsa04929	GnRH secretion	9/175	64/8085	8.40E-06	2.19E-05	8.17E-06	PRKCA/AKT1/ES<br>R2/MAPK1/MAP<br>K3/RAF1/PRKCB/<br>SPP1/CACNA1S	9
hsa05143	African trypanosomiasis	7/175	37/8085	1.17E-05	3.04E-05	1.13E-05	PRKCA/ICAM1/S<br>ELE/VCAM1/IFN<br>G/IL1B/PRKCB	7
hsa05010	Alzheimer disease	22/175	369/8085	1.38E-05	3.54E-05	1.32E-05	PTGS2/CASP9/C<br>ASP3/CASP8/NO<br>S2/RELA/IKBKB/<br>AKT1/MAPK8/GS<br>K3B/MAPK1/CAS<br>P7/MAPK10/MAP<br>K3/BAD/RAF1/IL<br>1B/IL1A/CHUK/L<br>PL/INS/CACNA1S	22

hsa04540	Gap junction	10/175	88/8085	1.83E-05	4.65E-05	1.73E-05	PRKCA/CDK1/EGFR/MAPK1/MAPK3/EGF/RAF1/GJA1/PRKCB/DRD2	10
hsa04921	Oxytocin signaling pathway	13/175	154/8085	2.69E-05	6.77E-05	2.52E-05	PTGS2/JUN/PRKCA/EGFR/CCND1/CDKN1A/MAPK1/MAPK3/FOS/RAF1/PRKCB/NOS3/CACNA1S	13
hsa03320	PPAR signaling pathway	9/175	76/8085	3.48E-05	8.66E-05	3.23E-05	PPARG/MMP1/RXRA/PPARD/PPARA/RXRB/LPL/FABP1/SCD	9
hsa01523	Antifolate resistance	6/175	31/8085	4.43E-05	0.000109406	4.08E-05	RELA/IKBKB/ABCC1/IL1B/ABCG2/CHUK	6
hsa00140	Steroid hormone biosynthesis	8/175	61/8085	4.54E-05	0.000111181	4.14E-05	CYP3A4/CYP1A2/CYP1A1/CYP1B1/AKR1C3/CYP19A1/UGT1A1/AKR	8
hsa04140	Autophagy - animal	12/175	141/8085	5.11E-05	0.000123916	4.62E-05	BCL2/AKT1/MAPK8/BCL2L1/MAPK1/MAPK10/MAPK3/BAD/RAF1/HIF1A/CTSD/INS	12
hsa04930	Type II diabetes mellitus	7/175	46/8085	5.18E-05	0.000124562	4.64E-05	IKBKB/MAPK8/MAPK1/MAPK10/MAPK3/HK2/INS	7
hsa05321	Inflammatory bowel disease	8/175	65/8085	7.24E-05	0.000172422	6.43E-05	JUN/RELA/STAT1/IFNG/IL4/STAT3/IL1B/IL1A	8
hsa05020	Prion disease	17/175	273/8085	8.21E-05	0.000194017	7.23E-05	BAX/CASP9/CASP3/MAPK8/MAPK14/GSK3B/MAPK1/MAPK10/NCF1/MAPK3/BAD/SOD1/HSPA5/CAV1/IL1B/IL1A/CAC	17

hsa04913	Ovarian steroidogenesis	7/175	51/8085	0.000102344	0.000239677	8.93E-05	PTGS2/CYP1A1/CYP1B1/ALOX5/AKR1C3/CYP19A1/INS	7
hsa04960	Aldosterone-regulated sodium reabsorption	6/175	37/8085	0.000125657	0.000291781	0.000108731	NR3C2/PRKCA/MAPK1/MAPK3/PRKCB/INS	6
hsa04934	Cushing syndrome	12/175	155/8085	0.000127982	0.000294682	0.000109812	AHR/GSK3B/CDK2/EGFR/CCND1/CDKN1A/MAPK1/RB1/CDK4/MAPK3/E2F1/CACN	12
hsa00982	Drug metabolism - cytochrome P450	8/175	72/8085	0.000150886	0.000341675	0.000127324	CYP3A4/CYP1A2/GSTP1/GSTM1/UGT1A1/GSTA1/GSTA2/ADH1B	8
hsa04137	Mitophagy - animal	8/175	72/8085	0.000150886	0.000341675	0.000127324	JUN/RELA/MAPK8/BCL2L1/TP53/MAPK10/HIF1A/	8
hsa04217	Necroptosis	12/175	159/8085	0.000163028	0.000366146	0.000136443	BCL2/BAX/CASP8/MAPK8/STAT1/IFNG/XIAP/MAPK10/STAT3/IL1B/IL1A/PARP1	12
hsa04015	Rap1 signaling pathway	14/175	210/8085	0.000175445	0.000390828	0.000145641	PRKCA/AKT1/MAPK14/EGFR/VEGFA/MAPK1/MET/KDR/MAPK3/EGF/RAF1/PRKCB/DRD2/INS	14
hsa04020	Calcium signaling pathway	15/175	240/8085	0.000209743	0.000463465	0.000172709	ADRA1B/PRKCA/NOS2/EGFR/VEGFA/ERBB2/MET/KDR/EGF/PTGER3/PRKCB/NOS3/ERBB3/HRH1/CACNA1S	15
hsa05416	Viral myocarditis	7/175	60/8085	0.000289881	0.000635419	0.000236787	CASP9/CASP3/CASP8/ICAM1/CCND1/CD40LG/CA	7

hsa00983	Drug metabolism - other enzymes	8/175	80/8085	0.000315516	0.000686122	0.000255681	CYP3A4/GSTP1/GSTM1/UGT1A1/CES1/MPO/GSTA1/GSTA2	8
hsa04725	Cholinergic synapse	9/175	113/8085	0.000735166	0.001586107	0.000591058	BCL2/PRKCA/ACHE/AKT1/MAPK1/MAPK3/FOS/PRKCB/CACNA1S	9
hsa04670	Leukocyte transendothelial migration	9/175	114/8085	0.000783591	0.001677374	0.000625068	PRKCA/ICAM1/VCAM1/MAPK14/MMP2/MMP9/NCF1/PRKCB/CLDN	9
hsa04152	AMPK signaling pathway	9/175	120/8085	0.00113173	0.00240383	0.00089578	PPARG/AKT1/CCNA2/CCND1/FASN/HMGCR/ACACA/INS/SCD	9
hsa04750	Inflammatory mediator regulation of TRP channels	8/175	98/8085	0.00123193	0.002596529	0.000967589	PRKCA/MAPK8/MAPK14/MAPK10/IL1B/PRKCB/TRPV1/HRH1	8
hsa00480	Glutathione metabolism	6/175	57/8085	0.001372051	0.002869787	0.001069417	GSTP1/GSTM1/GSR/ODC1/GSTA1/GSTA2	6
hsa04061	Viral protein interaction with cytokine and cytokine receptor	8/175	100/8085	0.001404497	0.002915396	0.001086413	IL6R/IL10RA/IL2RA/CCL2/CXCL8/CXCL11/CXCL2/CXCL10	8
hsa04916	Melanogenesis	8/175	101/8085	0.001497705	0.003085498	0.001149801	PRKCA/GSK3B/MAPK1/TYR/MAPK3/RAF1/PRKCB/EDN1	8
hsa04150	mTOR signaling pathway	10/175	155/8085	0.001915936	0.00391766	0.001459904	PRKCA/IKBKB/AKT1/GSK3B/MAPK1/MAPK3/RAF1/PRKCB/CHUK/I	10
hsa00590	Arachidonic acid metabolism	6/175	61/8085	0.001955179	0.003968289	0.001478771	PTGS1/PTGS2/ALOX5/AKR1C3/PTGES/PLB1	6
hsa04728	Dopaminergic synapse	9/175	132/8085	0.002201628	0.004435633	0.001652925	PRKCA/AKT1/MAPK8/MAPK14/GSK3B/MAPK10/FOS/PRKCB/DRD2	9

hsa04623	Cytosolic DNA-sensing pathway	6/175	63/8085	0.00230897	0.004603196	0.001715367	RELA/IKBKB/NFKBIA/IL1B/CXCL10/CHUK	6
hsa04270	Vascular smooth muscle contraction	9/175	133/8085	0.002318398	0.004603196	0.001715367	ADRA1B/PRKCA/MAPK1/MAPK3/RAF1/PRKCB/EDN1/ADM/CACNA	9
hsa04371	Apelin signaling pathway	9/175	138/8085	0.002978333	0.005870959	0.002187795	NOS2/AKT1/CCND1/MAPK1/MAPK3/RAF1/NOS3/SERPINE1/SPP1	9
hsa04211	Longevity regulating pathway	7/175	89/8085	0.003060828	0.005990477	0.002232333	BAX/PPARG/RELA/AKT1/CAT/TP53/INS	7
hsa04310	Wnt signaling pathway	10/175	166/8085	0.003165691	0.006151768	0.002292438	JUN/PRKCA/MAPK8/GSK3B/CCND1/TP53/MAPK10/PPARD/MYC/P	10
hsa04022	cGMP-PKG signaling pathway	10/175	167/8085	0.003305659	0.006378526	0.002376938	ADRA1B/AKT1/ADRA2A/MAPK1/MAPK3/BAD/RAF1/NOS3/INS/CACNA1S	10
hsa04072	Phospholipase D signaling pathway	9/175	148/8085	0.00473899	0.009017245	0.003360249	PRKCA/AKT1/EGFR/MAPK1/MAPK3/EGF/RAF1/CXCL8/INS	9
hsa04723	Retrograde endocannabinoid signaling	9/175	148/8085	0.00473899	0.009017245	0.003360249	PTGS2/PRKCA/MAPK8/MAPK14/MAPK1/MAPK10/MAPK3/PRKCB/CACNA1S	9
hsa04666	Fc gamma R-mediated phagocytosis	7/175	97/8085	0.004941606	0.009337932	0.003479752	PRKCA/AKT1/MAPK1/NCF1/MAPK3/RAF1/PRKCB	7
hsa04114	Oocyte meiosis	8/175	129/8085	0.00678473	0.012732987	0.004744909	AR/CDK1/MAPK14/CDK2/MAPK1/CCNB1/MAPK3/I	8
hsa04650	Natural killer cell mediated cytotoxicity	8/175	131/8085	0.00742551	0.013840746	0.005157712	CASP3/PRKCA/ICAM1/MAPK1/IFNG/MAPK3/RAF1/PRKCB	8

hsa04923	Regulation of lipolysis in adipocytes	5/175	57/8085	0.007572985	0.014020256	0.005224606	PTGS1/PTGS2/AKT1/PTGER3/INS	5
hsa04613	Neutrophil extracellular trap formation	10/175	190/8085	0.008129122	0.014948856	0.005570646	PRKCA/RELA/AKT1/MAPK14/MAPK1/NCF1/MAPK3/RAF1/PRKCB/MPO	10
hsa04730	Long-term depression	5/175	60/8085	0.009379686	0.01713356	0.006384769	PRKCA/MAPK1/MAPK3/RAF1/PRKCB	5
hsa00220	Arginine biosynthesis	3/175	22/8085	0.011342784	0.02058227	0.00766992	NOS2/GOT1/NOS	3
hsa04060	Cytokine-cytokine receptor interaction	13/175	295/8085	0.011549953	0.02082031	0.007758625	IL6R/IL10RA/IL2RA/IFNG/IL4/CD40LG/IL1B/CCL2/CXCL8/IL1A/CXCL11/CXCL2/CXCL1	13
hsa04550	Signaling pathways regulating pluripotency of stem cells	8/175	143/8085	0.012273469	0.021979938	0.008190757	AKT1/MAPK14/GSK3B/MAPK1/MAPK3/STAT3/RAF1/MYC	8
hsa04976	Bile secretion	6/175	89/8085	0.012471484	0.022120091	0.008242984	CYP3A4/RXRA/CA2/HMGCR/UGT1A1/ABCG2	6
hsa00380	Tryptophan metabolism	4/175	42/8085	0.01251319	0.022120091	0.008242984	CYP1A2/CYP1A1/CYP1B1/CAT	4
hsa04940	Type I diabetes mellitus	4/175	43/8085	0.013569001	0.023832732	0.008881195	IFNG/IL1B/IL1A/INS	4
hsa04977	Vitamin digestion and absorption	3/175	24/8085	0.014444912	0.025209591	0.009394277	APOB/PLB1/ABCC1	3
hsa04720	Long-term potentiation	5/175	67/8085	0.014704233	0.025499746	0.009502403	PRKCA/MAPK1/MAPK3/RAF1/PRKCB	5
hsa00830	Retinol metabolism	5/175	68/8085	0.015601106	0.026884925	0.010018585	CYP3A4/CYP1A2/CYP1A1/UGT1A1/ADH1B	5
hsa04261	Adrenergic signaling in cardiomyocytes	8/175	150/8085	0.016003482	0.027405964	0.010212749	ADRA1B/BCL2/PRKCA/AKT1/MAPK14/MAPK1/MAPK3/CACNA1S	8
hsa04611	Platelet activation	7/175	124/8085	0.017862509	0.03039955	0.011328299	PTGS1/AKT1/MAPK14/MAPK1/MAPK3/NOS3/COL1	7

hsa04520	Adherens junction	5/175	71/8085	0.018509001	0.031305348	0.011665842	EGFR/MAPK1/ERBB2/MET/MAPK	5
hsa04640	Hematopoietic cell lineage	6/175	99/8085	0.02017866	0.033919957	0.012640168	IL6R/CD14/IL2RA/IL4/IL1B/IL1A	6
hsa05030	Cocaine addiction	4/175	49/8085	0.021088766	0.03523367	0.01312972	JUN/RELA/DRD2/BDNF	4
hsa04918	Thyroid hormone synthesis	5/175	75/8085	0.022912534	0.038048693	0.014178729	PRKCA/GSR/HSPA5/PRKCB/DUOX2	5
hsa00330	Arginine and proline metabolism	4/175	51/8085	0.024064181	0.039555161	0.01474011	NOS2/GOT1/ODC1/NOS3	4
hsa00591	Linoleic acid metabolism	3/175	29/8085	0.024108438	0.039555161	0.01474011	CYP3A4/CYP1A2/PLB1	3
hsa04530	Tight junction	8/175	169/8085	0.030166448	0.04920004	0.018334244	JUN/MAPK8/CCND1/CDK4/PCNA/ERBB2/MAPK10/CLDN4	8
hsa04080	Neuroactive ligand-receptor interaction	13/175	341/8085	0.033512531	0.054333925	0.020247371	ADRA1B/OPRM1/PRSS1/ADRA2A/NR3C1/PTGER3/DRD2/TRPV1/EDN1/GCG/PYY/ADM/HRH1	13
hsa04610	Complement and coagulation cascades	5/175	85/8085	0.036722461	0.059187967	0.022056215	PLAU/F3/THBD/SERPINE1/SERPINB2	5
hsa04911	Insulin secretion	5/175	86/8085	0.038332665	0.061421931	0.022888695	PRKCA/PRKCB/INS/GCG/CACNA	5
hsa05168	Herpes simplex virus 1 infection	17/175	498/8085	0.041076247	0.065435416	0.024384308	BCL2/BAX/CASP9/CASP3/CASP8/RELA/IKBKB/AKT1/STAT1/BCL2L1/TP53/NFKBIA/IFNG/BAD/IL1B/CCL2/CHUK	17
hsa00350	Tyrosine metabolism	3/175	36/8085	0.042235487	0.066893199	0.024927546	TYR/GOT1/ADH1B	3
hsa05012	Parkinson disease	10/175	249/8085	0.043698388	0.068812405	0.025642732	BAX/CASP9/CASP3/MAPK8/BCL2L1/TP53/MAPK10/HSPA5/NFE2L2/DRD2	10

hsa04213	Longevity regulating pathway - multiple	4/175	62/8085	0.044825468	0.070183876	0.026153807	AKT1/CAT/SOD1/INS	4
hsa05217	Basal cell carcinoma	4/175	63/8085	0.047087295	0.073306356	0.02731739	BAX/GSK3B/CDKN1A/TP53	4
hsa05330	Allograft rejection	3/175	38/8085	0.048376863	0.074888477	0.027906962	IFNG/IL4/CD40L	3
