# Supplementary material for: Analysis of the Efficacy and Pharmacological Mechanisms of Action of Zhenren Yangzang Decoction on Ulcerative Colitis Using Meta-Analysis and Network Pharmacology
Source: Evid Based Complement Alternat Med. 2021 Dec 28;2021:4512755. doi: 10.1155/2021/4512755 (PMC8727130; doi:10.1155/2021/4512755)
Supplement: Supplementary Materials — Figure S1: Risk of bias graph. Figure S2: risk of bias summary. Figure S3: forest plot of comparison of serum cytokines. Figure S4: forest plot of comparison of the total syndrome score of TCM. Table S1: basic information on the active compounds in ZRYZD. Table S2: gene symbols and entrezID of active target genes. Table S3: compounds ranked by the degree in the network. Supplementary File 1: compounds of ZRYZD from TCMSP. Supplementary File 2: corresponding target genes of ZRYZD. Supplementary File 3: UC-related target genes. Supplementary File 4: GO functional enrichment analysis. Supplementary File 5: KEGG pathway enrichment analysis. Supplementary File 6: data of compound-target networks. Supplementary File 7: data of key compound-target networks. Supplementary File 8: data of PPI network. [file 4512755.f1.zip › 4512755.f1/Supplementary File 6 Data of compound-target network.pdf]

**Supplementary File 6 Data of compound–target network**

**network**

| Node1 | Node2 | Net | MolName |
|---|---|---|---|
| MOL001919 | NR3C2 | target | (3S,5R,8R,9R,10S,14S)-3,17-dihydroxy-4,4,8,10,14-pentamethyl-2,3,5,6,7,9-hexahydro-1H-cyclopenta[a]phenanthrene-15,16- |
| MOL001924 | TNFAIP6 | target | paeoniflorin |
| MOL001924 | IL6R | target | paeoniflorin |
| MOL001924 | CD14 | target | paeoniflorin |
| MOL001924 | LBP | target | paeoniflorin |
| MOL000358 | PTGS1 | target | beta-sitosterol |
| MOL000358 | PTGS2 | target | beta-sitosterol |
| MOL000358 | ADRA1B | target | beta-sitosterol |
| MOL000358 | SLC6A4 | target | beta-sitosterol |
| MOL000358 | OPRM1 | target | beta-sitosterol |
| MOL000358 | BCL2 | target | beta-sitosterol |
| MOL000358 | BAX | target | beta-sitosterol |
| MOL000358 | CASP9 | target | beta-sitosterol |
| MOL000358 | JUN | target | beta-sitosterol |
| MOL000358 | CASP3 | target | beta-sitosterol |
| MOL000358 | CASP8 | target | beta-sitosterol |
| MOL000358 | PRKCA | target | beta-sitosterol |
| MOL000358 | PON1 | target | beta-sitosterol |
| MOL000359 | NR3C2 | target | sitosterol |
| MOL000422 | NOS2 | target | kaempferol |
| MOL000422 | PTGS1 | target | kaempferol |
| MOL000422 | AR | target | kaempferol |
| MOL000422 | PPARG | target | kaempferol |
| MOL000422 | PTGS2 | target | kaempferol |
| MOL000422 | DPP4 | target | kaempferol |
| MOL000422 | PRSS1 | target | kaempferol |
| MOL000422 | ACHE | target | kaempferol |
| MOL000422 | ADRA1B | target | kaempferol |
| MOL000422 | RELA | target | kaempferol |
| MOL000422 | IKBKB | target | kaempferol |
| MOL000422 | AKT1 | target | kaempferol |
| MOL000422 | BCL2 | target | kaempferol |
| MOL000422 | BAX | target | kaempferol |
| MOL000422 | TNFAIP6 | target | kaempferol |
| MOL000422 | JUN | target | kaempferol |
| MOL000422 | CASP3 | target | kaempferol |
| MOL000422 | MAPK8 | target | kaempferol |
| MOL000422 | MMP1 | target | kaempferol |
| MOL000422 | STAT1 | target | kaempferol |
| MOL000422 | CDK1 | target | kaempferol |
| MOL000422 | HMOX1 | target | kaempferol |
| MOL000422 | CYP3A4 | target | kaempferol |
| MOL000422 | CYP1A2 | target | kaempferol |
| MOL000422 | CYP1A1 | target | kaempferol |
| MOL000422 | ICAM1 | target | kaempferol |
| MOL000422 | SELE | target | kaempferol |
| MOL000422 | VCAM1 | target | kaempferol |
| MOL000422 | NR1I2 | target | kaempferol |
| MOL000422 | CYP1B1 | target | kaempferol |
| MOL000422 | ALOX5 | target | kaempferol |
| MOL000422 | GSTP1 | target | kaempferol |
| MOL000422 | AHR | target | kaempferol |
| MOL000422 | GSTM1 | target | kaempferol |

| | | | |
|---|---|---|---|
| MOL000422 | AKR1C3 | target | kaempferol |
| MOL000422 | SLPI | target | kaempferol |
| MOL000492 | PTGS1 | target | (+)-catechin |
| MOL000492 | ESR1 | target | (+)-catechin |
| MOL000492 | PTGS2 | target | (+)-catechin |
| MOL000492 | DPEP1 | target | (+)-catechin |
| MOL000492 | RXRA | target | (+)-catechin |
| MOL000492 | CAT | target | (+)-catechin |
| MOL000022 | PTGS2 | target | 14-acetyl-12-senecioyl-2E,8Z,10E-atractylentriol |
| MOL000049 | AR | target | 3β-acetoxyatractylone |
| MOL000049 | PTGS2 | target | 3β-acetoxyatractylone |
| MOL000049 | RXRA | target | 3β-acetoxyatractylone |
| MOL000049 | ACHE | target | 3β-acetoxyatractylone |
| MOL000049 | OPRM1 | target | 3β-acetoxyatractylone |
| MOL000049 | DPP4 | target | 3β-acetoxyatractylone |
| MOL000072 | PTGS2 | target | 8β-ethoxy atractylenolide III |
| MOL000449 | NR3C2 | target | Stigmasterol |
| MOL000449 | RXRA | target | Stigmasterol |
| MOL000449 | PTGS1 | target | Stigmasterol |
| MOL000449 | PTGS2 | target | Stigmasterol |
| MOL000449 | ADRA2A | target | Stigmasterol |
| MOL000449 | AKR1B1 | target | Stigmasterol |
| MOL000449 | PLAU | target | Stigmasterol |
| MOL000449 | ADRA1B | target | Stigmasterol |
| MOL001006 | NR3C2 | target | poriferasta-7,22E-dien-3beta-ol |
| MOL002140 | PTGS2 | target | Perlolyrine |
| MOL002140 | RXRA | target | Perlolyrine |
| MOL003036 | NR3C2 | target | ZINC03978781 |
| MOL003896 | NOS2 | target | 7-Methoxy-2-methyl isoflavone |
| MOL003896 | PTGS1 | target | 7-Methoxy-2-methyl isoflavone |
| MOL003896 | ESR1 | target | 7-Methoxy-2-methyl isoflavone |
| MOL003896 | AR | target | 7-Methoxy-2-methyl isoflavone |
| MOL003896 | PPARG | target | 7-Methoxy-2-methyl isoflavone |
| MOL003896 | PTGS2 | target | 7-Methoxy-2-methyl isoflavone |
| MOL003896 | RXRA | target | 7-Methoxy-2-methyl isoflavone |
| MOL003896 | ACHE | target | 7-Methoxy-2-methyl isoflavone |
| MOL003896 | ADRA1B | target | 7-Methoxy-2-methyl isoflavone |
| MOL003896 | SLC6A4 | target | 7-Methoxy-2-methyl isoflavone |
| MOL003896 | ESR2 | target | 7-Methoxy-2-methyl isoflavone |
| MOL003896 | DPP4 | target | 7-Methoxy-2-methyl isoflavone |
| MOL003896 | MAPK14 | target | 7-Methoxy-2-methyl isoflavone |
| MOL003896 | GSK3B | target | 7-Methoxy-2-methyl isoflavone |
| MOL003896 | CDK2 | target | 7-Methoxy-2-methyl isoflavone |
| MOL003896 | CHEK1 | target | 7-Methoxy-2-methyl isoflavone |
| MOL003896 | PRSS1 | target | 7-Methoxy-2-methyl isoflavone |
| MOL003896 | CCNA2 | target | 7-Methoxy-2-methyl isoflavone |
| MOL003896 | OPRM1 | target | 7-Methoxy-2-methyl isoflavone |
| MOL004355 | NR3C2 | target | Spinasterol |
| MOL005321 | PTGS1 | target | Frutinone A |
| MOL005321 | AR | target | Frutinone A |
| MOL005321 | PPARG | target | Frutinone A |
| MOL005321 | PTGS2 | target | Frutinone A |
| MOL005321 | RXRA | target | Frutinone A |
| MOL005321 | DPP4 | target | Frutinone A |
| MOL005321 | ACHE | target | Frutinone A |
| MOL000006 | PTGS1 | target | luteolin |
| MOL000006 | AR | target | luteolin |
| MOL000006 | PTGS2 | target | luteolin |

| | | | |
|---|---|---|---|
| MOL000006 | PRSS1 | target | luteolin |
| MOL000006 | DPP4 | target | luteolin |
| MOL000006 | RELA | target | luteolin |
| MOL000006 | EGFR | target | luteolin |
| MOL000006 | AKT1 | target | luteolin |
| MOL000006 | VEGFA | target | luteolin |
| MOL000006 | CCND1 | target | luteolin |
| MOL000006 | BCL2L1 | target | luteolin |
| MOL000006 | CDKN1A | target | luteolin |
| MOL000006 | CASP9 | target | luteolin |
| MOL000006 | MMP2 | target | luteolin |
| MOL000006 | MMP9 | target | luteolin |
| MOL000006 | MAPK1 | target | luteolin |
| MOL000006 | IL10RA | target | luteolin |
| MOL000006 | RB1 | target | luteolin |
| MOL000006 | CDK4 | target | luteolin |
| MOL000006 | TNFAIP6 | target | luteolin |
| MOL000006 | JUN | target | luteolin |
| MOL000006 | IL6R | target | luteolin |
| MOL000006 | CASP3 | target | luteolin |
| MOL000006 | TP53 | target | luteolin |
| MOL000006 | NFKBIA | target | luteolin |
| MOL000006 | TOP1 | target | luteolin |
| MOL000006 | MDM2 | target | luteolin |
| MOL000006 | MMP1 | target | luteolin |
| MOL000006 | PCNA | target | luteolin |
| MOL000006 | ERBB2 | target | luteolin |
| MOL000006 | PPARG | target | luteolin |
| MOL000006 | HMOX1 | target | luteolin |
| MOL000006 | CASP7 | target | luteolin |
| MOL000006 | ICAM1 | target | luteolin |
| MOL000006 | MCL1 | target | luteolin |
| MOL000006 | BIRC5 | target | luteolin |
| MOL000006 | IL2RA | target | luteolin |
| MOL000006 | CCNB1 | target | luteolin |
| MOL000006 | TYR | target | luteolin |
| MOL000006 | IFNG | target | luteolin |
| MOL000006 | IL4 | target | luteolin |
| MOL000006 | TOP2A | target | luteolin |
| MOL000006 | GSTP1 | target | luteolin |
| MOL000006 | XIAP | target | luteolin |
| MOL000006 | CD40LG | target | luteolin |
| MOL000006 | PTGES | target | luteolin |
| MOL000006 | MET | target | luteolin |
| MOL007059 | PTGS2 | target | 3-beta-Hydroxymethyllenetanshiquinone |
| MOL007059 | CA2 | target | 3-beta-Hydroxymethyllenetanshiquinone |
| MOL007059 | RXRA | target | 3-beta-Hydroxymethyllenetanshiquinone |
| MOL007059 | ACHE | target | 3-beta-Hydroxymethyllenetanshiquinone |
| MOL007059 | OPRM1 | target | 3-beta-Hydroxymethyllenetanshiquinone |
| MOL007059 | DPP4 | target | 3-beta-Hydroxymethyllenetanshiquinone |
| MOL007059 | PRSS1 | target | 3-beta-Hydroxymethyllenetanshiquinone |
| MOL008397 | NR3C1 | target | Daturilin |
| MOL008400 | PTGS1 | target | glycitein |
| MOL008400 | ESR1 | target | glycitein |
| MOL008400 | AR | target | glycitein |
| MOL008400 | PPARG | target | glycitein |
| MOL008400 | PTGS2 | target | glycitein |
| MOL008400 | RXRA | target | glycitein |

| | | | |
|---|---|---|---|
| MOL008400 | ESR2 | target | glycitein |
| MOL008400 | MAPK14 | target | glycitein |
| MOL008400 | GSK3B | target | glycitein |
| MOL008400 | CDK2 | target | glycitein |
| MOL008400 | CHEK1 | target | glycitein |
| MOL008400 | PRSS1 | target | glycitein |
| MOL008400 | CCNA2 | target | glycitein |
| MOL008400 | NOS2 | target | glycitein |
| MOL008400 | MMP13 | target | glycitein |
| MOL008400 | MMP8 | target | glycitein |
| MOL008407 | NR3C2 | target | (8S,9S,10R,13R,14S,17R)-17-[(E,2R,5S)-5-ethyl-6-methylhept-3-en-2-yl]-10,13-dimethyl-1,2,4,7,8,9,11,12,14,15,16,17-dodecahydrocyclopenta[a]phenanthren-3-one |
| MOL008411 | ESR1 | target | 11-Hydroxyrankinidine |
| MOL008411 | OPRM1 | target | 11-Hydroxyrankinidine |
| MOL008411 | CDK2 | target | 11-Hydroxyrankinidine |
| MOL001484 | PTGS1 | target | Inermine |
| MOL001484 | PTGS2 | target | Inermine |
| MOL001484 | HTR3A | target | Inermine |
| MOL001484 | RXRA | target | Inermine |
| MOL001484 | ADRA1B | target | Inermine |
| MOL001484 | PRSS1 | target | Inermine |
| MOL001484 | OPRM1 | target | Inermine |
| MOL001792 | PTGS1 | target | DFV |
| MOL001792 | ESR1 | target | DFV |
| MOL001792 | PTGS2 | target | DFV |
| MOL001792 | RXRA | target | DFV |
| MOL001792 | DPEP1 | target | DFV |
| MOL001792 | SLC6A4 | target | DFV |
| MOL002311 | NOS2 | target | Glycyrol |
| MOL002311 | ESR1 | target | Glycyrol |
| MOL002311 | PPARG | target | Glycyrol |
| MOL002311 | PTGS2 | target | Glycyrol |
| MOL002311 | KDR | target | Glycyrol |
| MOL002311 | MAPK14 | target | Glycyrol |
| MOL002311 | GSK3B | target | Glycyrol |
| MOL002311 | CHEK1 | target | Glycyrol |
| MOL002311 | CCNA2 | target | Glycyrol |
| MOL000239 | NOS2 | target | Jaranol |
| MOL000239 | PTGS1 | target | Jaranol |
| MOL000239 | AR | target | Jaranol |
| MOL000239 | PTGS2 | target | Jaranol |
| MOL000239 | ESR2 | target | Jaranol |
| MOL000239 | DPP4 | target | Jaranol |
| MOL000239 | CDK2 | target | Jaranol |
| MOL000239 | CHEK1 | target | Jaranol |
| MOL000239 | PRSS1 | target | Jaranol |
| MOL002565 | NOS2 | target | Medicarpin |
| MOL002565 | PTGS1 | target | Medicarpin |
| MOL002565 | ESR1 | target | Medicarpin |
| MOL002565 | PTGS2 | target | Medicarpin |
| MOL002565 | RXRA | target | Medicarpin |
| MOL002565 | ADRA1B | target | Medicarpin |
| MOL002565 | SLC6A4 | target | Medicarpin |
| MOL002565 | OPRM1 | target | Medicarpin |
| MOL002565 | ESR2 | target | Medicarpin |
| MOL002565 | DPP4 | target | Medicarpin |
| MOL002565 | MAPK10 | target | Medicarpin |

| | | | |
|---|---|---|---|
| MOL002565 | CDK2 | target | Medicarpin |
| MOL002565 | PRSS1 | target | Medicarpin |
| MOL002565 | CCNA2 | target | Medicarpin |
| MOL000354 | NOS2 | target | isorhamnetin |
| MOL000354 | PTGS1 | target | isorhamnetin |
| MOL000354 | ESR1 | target | isorhamnetin |
| MOL000354 | AR | target | isorhamnetin |
| MOL000354 | PPARG | target | isorhamnetin |
| MOL000354 | PTGS2 | target | isorhamnetin |
| MOL000354 | ESR2 | target | isorhamnetin |
| MOL000354 | DPP4 | target | isorhamnetin |
| MOL000354 | MAPK14 | target | isorhamnetin |
| MOL000354 | GSK3B | target | isorhamnetin |
| MOL000354 | CDK2 | target | isorhamnetin |
| MOL000354 | PRSS1 | target | isorhamnetin |
| MOL000354 | CCNA2 | target | isorhamnetin |
| MOL000354 | PPARD | target | isorhamnetin |
| MOL000354 | CHEK1 | target | isorhamnetin |
| MOL000354 | AKR1B1 | target | isorhamnetin |
| MOL000354 | ACHE | target | isorhamnetin |
| MOL000354 | RELA | target | isorhamnetin |
| MOL000354 | NCF1 | target | isorhamnetin |
| MOL003656 | NOS2 | target | Lupiwighteone |
| MOL003656 | ESR1 | target | Lupiwighteone |
| MOL003656 | AR | target | Lupiwighteone |
| MOL003656 | PPARG | target | Lupiwighteone |
| MOL003656 | PTGS2 | target | Lupiwighteone |
| MOL003656 | ESR2 | target | Lupiwighteone |
| MOL003656 | DPP4 | target | Lupiwighteone |
| MOL003656 | MAPK14 | target | Lupiwighteone |
| MOL003656 | GSK3B | target | Lupiwighteone |
| MOL003656 | CDK2 | target | Lupiwighteone |
| MOL003656 | CHEK1 | target | Lupiwighteone |
| MOL003656 | PRSS1 | target | Lupiwighteone |
| MOL003656 | CCNA2 | target | Lupiwighteone |
| MOL000392 | NOS2 | target | formononetin |
| MOL000392 | PTGS1 | target | formononetin |
| MOL000392 | ESR1 | target | formononetin |
| MOL000392 | AR | target | formononetin |
| MOL000392 | PPARG | target | formononetin |
| MOL000392 | PTGS2 | target | formononetin |
| MOL000392 | RXRA | target | formononetin |
| MOL000392 | SLC6A4 | target | formononetin |
| MOL000392 | ESR2 | target | formononetin |
| MOL000392 | DPP4 | target | formononetin |
| MOL000392 | MAPK14 | target | formononetin |
| MOL000392 | GSK3B | target | formononetin |
| MOL000392 | CDK2 | target | formononetin |
| MOL000392 | CHEK1 | target | formononetin |
| MOL000392 | PRSS1 | target | formononetin |
| MOL000392 | CCNA2 | target | formononetin |
| MOL000392 | ACHE | target | formononetin |
| MOL000392 | DPEP1 | target | formononetin |
| MOL000392 | JUN | target | formononetin |
| MOL000392 | IL4 | target | formononetin |
| MOL000417 | NOS2 | target | Calycosin |
| MOL000417 | PTGS1 | target | Calycosin |
| MOL000417 | ESR1 | target | Calycosin |

| | | | |
|---|---|---|---|
| MOL000417 | AR | target | Calycosin |
| MOL000417 | PPARG | target | Calycosin |
| MOL000417 | PTGS2 | target | Calycosin |
| MOL000417 | RXRA | target | Calycosin |
| MOL000417 | ESR2 | target | Calycosin |
| MOL000417 | DPP4 | target | Calycosin |
| MOL000417 | MAPK14 | target | Calycosin |
| MOL000417 | GSK3B | target | Calycosin |
| MOL000417 | CDK2 | target | Calycosin |
| MOL000417 | CHEK1 | target | Calycosin |
| MOL000417 | PRSS1 | target | Calycosin |
| MOL000417 | CCNA2 | target | Calycosin |
| MOL004328 | PTGS1 | target | naringenin |
| MOL004328 | ESR1 | target | naringenin |
| MOL004328 | PTGS2 | target | naringenin |
| MOL004328 | DPEP1 | target | naringenin |
| MOL004328 | RELA | target | naringenin |
| MOL004328 | AKT1 | target | naringenin |
| MOL004328 | BCL2 | target | naringenin |
| MOL004328 | MAPK3 | target | naringenin |
| MOL004328 | MAPK1 | target | naringenin |
| MOL004328 | CASP3 | target | naringenin |
| MOL004328 | FASN | target | naringenin |
| MOL004328 | BAD | target | naringenin |
| MOL004328 | SOD1 | target | naringenin |
| MOL004328 | CAT | target | naringenin |
| MOL004328 | PPARG | target | naringenin |
| MOL004328 | APOB | target | naringenin |
| MOL004328 | PLB1 | target | naringenin |
| MOL004328 | HMGCR | target | naringenin |
| MOL004328 | CYP19A1 | target | naringenin |
| MOL004328 | GSTP1 | target | naringenin |
| MOL004328 | UGT1A1 | target | naringenin |
| MOL004328 | PPARA | target | naringenin |
| MOL004328 | GSR | target | naringenin |
| MOL004328 | ABCC1 | target | naringenin |
| MOL004328 | AKR1C1 | target | naringenin |
| MOL004328 | GOT1 | target | naringenin |
| MOL004328 | CES1 | target | naringenin |
| MOL004328 | SOAT1 | target | naringenin |
| MOL004805 | NOS2 | target | (2S)-2-[4-hydroxy-3-(3-methylbut-2-enyl)phenyl]-8,8-dimethyl-2,3-dihydropyrano[2,3-f]chromen-4-one |
| MOL004805 | ESR1 | target | (2S)-2-[4-hydroxy-3-(3-methylbut-2-enyl)phenyl]-8,8-dimethyl-2,3-dihydropyrano[2,3-f]chromen-4-one |
| MOL004805 | AR | target | (2S)-2-[4-hydroxy-3-(3-methylbut-2-enyl)phenyl]-8,8-dimethyl-2,3-dihydropyrano[2,3-f]chromen-4-one |
| MOL004805 | PPARG | target | (2S)-2-[4-hydroxy-3-(3-methylbut-2-enyl)phenyl]-8,8-dimethyl-2,3-dihydropyrano[2,3-f]chromen-4-one |
| MOL004805 | PTGS2 | target | (2S)-2-[4-hydroxy-3-(3-methylbut-2-enyl)phenyl]-8,8-dimethyl-2,3-dihydropyrano[2,3-f]chromen-4-one |
| MOL004805 | ESR2 | target | (2S)-2-[4-hydroxy-3-(3-methylbut-2-enyl)phenyl]-8,8-dimethyl-2,3-dihydropyrano[2,3-f]chromen-4-one |
| MOL004805 | MAPK14 | target | (2S)-2-[4-hydroxy-3-(3-methylbut-2-enyl)phenyl]-8,8-dimethyl-2,3-dihydropyrano[2,3-f]chromen-4-one |
| MOL004805 | GSK3B | target | (2S)-2-[4-hydroxy-3-(3-methylbut-2-enyl)phenyl]-8,8-dimethyl-2,3-dihydropyrano[2,3-f]chromen-4-one |
| MOL004806 | NOS2 | target | euchrenone |
| MOL004806 | ESR1 | target | euchrenone |

| | | | |
|---|---|---|---|
| MOL004806 | PTGS2 | target | euchrenone |
| MOL004806 | ESR2 | target | euchrenone |
| MOL004808 | NOS2 | target | glyasperin B |
| MOL004808 | ESR1 | target | glyasperin B |
| MOL004808 | AR | target | glyasperin B |
| MOL004808 | PPARG | target | glyasperin B |
| MOL004808 | PTGS2 | target | glyasperin B |
| MOL004808 | KDR | target | glyasperin B |
| MOL004808 | ACHE | target | glyasperin B |
| MOL004808 | ESR2 | target | glyasperin B |
| MOL004808 | DPP4 | target | glyasperin B |
| MOL004808 | GSK3B | target | glyasperin B |
| MOL004808 | CDK2 | target | glyasperin B |
| MOL004808 | PRSS1 | target | glyasperin B |
| MOL004808 | CCNA2 | target | glyasperin B |
| MOL004810 | NOS2 | target | glyasperin F |
| MOL004810 | PTGS1 | target | glyasperin F |
| MOL004810 | ESR1 | target | glyasperin F |
| MOL004810 | AR | target | glyasperin F |
| MOL004810 | PPARG | target | glyasperin F |
| MOL004810 | PTGS2 | target | glyasperin F |
| MOL004810 | ESR2 | target | glyasperin F |
| MOL004810 | MAPK14 | target | glyasperin F |
| MOL004810 | GSK3B | target | glyasperin F |
| MOL004810 | CDK2 | target | glyasperin F |
| MOL004810 | PRSS1 | target | glyasperin F |
| MOL004810 | CCNA2 | target | glyasperin F |
| MOL004811 | NOS2 | target | Glyasperin C |
| MOL004811 | ESR1 | target | Glyasperin C |
| MOL004811 | AR | target | Glyasperin C |
| MOL004811 | PPARG | target | Glyasperin C |
| MOL004811 | PTGS2 | target | Glyasperin C |
| MOL004811 | RXRA | target | Glyasperin C |
| MOL004811 | ACHE | target | Glyasperin C |
| MOL004811 | ESR2 | target | Glyasperin C |
| MOL004811 | DPP4 | target | Glyasperin C |
| MOL004811 | MAPK14 | target | Glyasperin C |
| MOL004811 | GSK3B | target | Glyasperin C |
| MOL004811 | CDK2 | target | Glyasperin C |
| MOL004811 | CHEK1 | target | Glyasperin C |
| MOL004811 | PRSS1 | target | Glyasperin C |
| MOL004811 | CCNA2 | target | Glyasperin C |
| MOL004814 | NOS2 | target | Isotrifoliol |
| MOL004814 | ESR1 | target | Isotrifoliol |
| MOL004814 | AR | target | Isotrifoliol |
| MOL004814 | PTGS2 | target | Isotrifoliol |
| MOL004814 | ESR2 | target | Isotrifoliol |
| MOL004814 | MAPK14 | target | Isotrifoliol |
| MOL004814 | GSK3B | target | Isotrifoliol |
| MOL004814 | CDK2 | target | Isotrifoliol |
| MOL004814 | CHEK1 | target | Isotrifoliol |
| MOL004814 | CCNA2 | target | Isotrifoliol |
| MOL004815 | NOS2 | target | (E)-1-(2,4-dihydroxyphenyl)-3-(2,2-dimethylchromen-6-yl)prop-2-en-1-one |
| MOL004815 | PTGS1 | target | (E)-1-(2,4-dihydroxyphenyl)-3-(2,2-dimethylchromen-6-yl)prop-2-en-1-one |
| MOL004815 | ESR1 | target | (E)-1-(2,4-dihydroxyphenyl)-3-(2,2-dimethylchromen-6-yl)prop-2-en-1-one |

| | | | |
|---|---|---|---|
| MOL004815 | AR | target | (E)-1-(2,4-dihydroxyphenyl)-3-(2,2-dimethylchromen-6-yl)prop-2-en-1-one |
| MOL004815 | PPARG | target | (E)-1-(2,4-dihydroxyphenyl)-3-(2,2-dimethylchromen-6-yl)prop-2-en-1-one |
| MOL004815 | PTGS2 | target | (E)-1-(2,4-dihydroxyphenyl)-3-(2,2-dimethylchromen-6-yl)prop-2-en-1-one |
| MOL004815 | CA2 | target | (E)-1-(2,4-dihydroxyphenyl)-3-(2,2-dimethylchromen-6-yl)prop-2-en-1-one |
| MOL004815 | RXRA | target | (E)-1-(2,4-dihydroxyphenyl)-3-(2,2-dimethylchromen-6-yl)prop-2-en-1-one |
| MOL004815 | ADRA1B | target | (E)-1-(2,4-dihydroxyphenyl)-3-(2,2-dimethylchromen-6-yl)prop-2-en-1-one |
| MOL004815 | ESR2 | target | (E)-1-(2,4-dihydroxyphenyl)-3-(2,2-dimethylchromen-6-yl)prop-2-en-1-one |
| MOL004815 | MAPK14 | target | (E)-1-(2,4-dihydroxyphenyl)-3-(2,2-dimethylchromen-6-yl)prop-2-en-1-one |
| MOL004815 | GSK3B | target | (E)-1-(2,4-dihydroxyphenyl)-3-(2,2-dimethylchromen-6-yl)prop-2-en-1-one |
| MOL004815 | CDK2 | target | (E)-1-(2,4-dihydroxyphenyl)-3-(2,2-dimethylchromen-6-yl)prop-2-en-1-one |
| MOL004815 | CHEK1 | target | (E)-1-(2,4-dihydroxyphenyl)-3-(2,2-dimethylchromen-6-yl)prop-2-en-1-one |
| MOL004815 | CCNA2 | target | (E)-1-(2,4-dihydroxyphenyl)-3-(2,2-dimethylchromen-6-yl)prop-2-en-1-one |
| MOL004820 | NOS2 | target | kanzonols W |
| MOL004820 | PTGS1 | target | kanzonols W |
| MOL004820 | ESR1 | target | kanzonols W |
| MOL004820 | AR | target | kanzonols W |
| MOL004820 | PPARG | target | kanzonols W |
| MOL004820 | PTGS2 | target | kanzonols W |
| MOL004820 | RXRA | target | kanzonols W |
| MOL004820 | ESR2 | target | kanzonols W |
| MOL004820 | MAPK14 | target | kanzonols W |
| MOL004820 | GSK3B | target | kanzonols W |
| MOL004820 | CDK2 | target | kanzonols W |
| MOL004820 | CHEK1 | target | kanzonols W |
| MOL004820 | PRSS1 | target | kanzonols W |
| MOL004820 | CCNA2 | target | kanzonols W |
| MOL004824 | NOS2 | target | (2S)-6-(2,4-dihydroxyphenyl)-2-(2-hydroxypropan-2-yl)-4-methoxy-2,3-dihydrofuro[3,2-g]chromen-7-one |
| MOL004824 | ESR1 | target | (2S)-6-(2,4-dihydroxyphenyl)-2-(2-hydroxypropan-2-yl)-4-methoxy-2,3-dihydrofuro[3,2-g]chromen-7-one |
| MOL004824 | AR | target | (2S)-6-(2,4-dihydroxyphenyl)-2-(2-hydroxypropan-2-yl)-4-methoxy-2,3-dihydrofuro[3,2-g]chromen-7-one |
| MOL004824 | PPARG | target | (2S)-6-(2,4-dihydroxyphenyl)-2-(2-hydroxypropan-2-yl)-4-methoxy-2,3-dihydrofuro[3,2-g]chromen-7-one |
| MOL004824 | PTGS2 | target | (2S)-6-(2,4-dihydroxyphenyl)-2-(2-hydroxypropan-2-yl)-4-methoxy-2,3-dihydrofuro[3,2-g]chromen-7-one |
| MOL004824 | KDR | target | (2S)-6-(2,4-dihydroxyphenyl)-2-(2-hydroxypropan-2-yl)-4-methoxy-2,3-dihydrofuro[3,2-g]chromen-7-one |
| MOL004824 | ACHE | target | (2S)-6-(2,4-dihydroxyphenyl)-2-(2-hydroxypropan-2-yl)-4-methoxy-2,3-dihydrofuro[3,2-g]chromen-7-one |
| MOL004824 | ESR2 | target | (2S)-6-(2,4-dihydroxyphenyl)-2-(2-hydroxypropan-2-yl)-4-methoxy-2,3-dihydrofuro[3,2-g]chromen-7-one |
| MOL004824 | DPP4 | target | (2S)-6-(2,4-dihydroxyphenyl)-2-(2-hydroxypropan-2-yl)-4-methoxy-2,3-dihydrofuro[3,2-g]chromen-7-one |
| MOL004824 | MAPK14 | target | (2S)-6-(2,4-dihydroxyphenyl)-2-(2-hydroxypropan-2-yl)-4-methoxy-2,3-dihydrofuro[3,2-g]chromen-7-one |

| | | | |
|---|---|---|---|
| MOL004824 | GSK3B | target | (2S)-6-(2,4-dihydroxyphenyl)-2-(2-hydroxypropan-2-yl)-4-methoxy-2,3-dihydrofuro[3,2-g]chromen-7-one |
| MOL004824 | CDK2 | target | (2S)-6-(2,4-dihydroxyphenyl)-2-(2-hydroxypropan-2-yl)-4-methoxy-2,3-dihydrofuro[3,2-g]chromen-7-one |
| MOL004824 | CHEK1 | target | (2S)-6-(2,4-dihydroxyphenyl)-2-(2-hydroxypropan-2-yl)-4-methoxy-2,3-dihydrofuro[3,2-g]chromen-7-one |
| MOL004824 | PRSS1 | target | (2S)-6-(2,4-dihydroxyphenyl)-2-(2-hydroxypropan-2-yl)-4-methoxy-2,3-dihydrofuro[3,2-g]chromen-7-one |
| MOL004824 | CCNA2 | target | (2S)-6-(2,4-dihydroxyphenyl)-2-(2-hydroxypropan-2-yl)-4-methoxy-2,3-dihydrofuro[3,2-g]chromen-7-one |
| MOL004827 | NOS2 | target | Semilicoisoflavone B |
| MOL004827 | ESR1 | target | Semilicoisoflavone B |
| MOL004827 | AR | target | Semilicoisoflavone B |
| MOL004827 | PPARG | target | Semilicoisoflavone B |
| MOL004827 | PTGS2 | target | Semilicoisoflavone B |
| MOL004827 | ACHE | target | Semilicoisoflavone B |
| MOL004827 | GSK3B | target | Semilicoisoflavone B |
| MOL004827 | CDK2 | target | Semilicoisoflavone B |
| MOL004827 | CHEK1 | target | Semilicoisoflavone B |
| MOL004827 | PRSS1 | target | Semilicoisoflavone B |
| MOL004828 | NOS2 | target | Glepidotin A |
| MOL004828 | PTGS1 | target | Glepidotin A |
| MOL004828 | ESR1 | target | Glepidotin A |
| MOL004828 | AR | target | Glepidotin A |
| MOL004828 | PPARG | target | Glepidotin A |
| MOL004828 | PTGS2 | target | Glepidotin A |
| MOL004828 | KDR | target | Glepidotin A |
| MOL004828 | RXRA | target | Glepidotin A |
| MOL004828 | DPP4 | target | Glepidotin A |
| MOL004828 | MAPK14 | target | Glepidotin A |
| MOL004828 | GSK3B | target | Glepidotin A |
| MOL004828 | CDK2 | target | Glepidotin A |
| MOL004828 | CHEK1 | target | Glepidotin A |
| MOL004828 | PRSS1 | target | Glepidotin A |
| MOL004828 | CCNA2 | target | Glepidotin A |
| MOL004829 | PTGS1 | target | Glepidotin B |
| MOL004829 | ESR1 | target | Glepidotin B |
| MOL004829 | PTGS2 | target | Glepidotin B |
| MOL004829 | RXRA | target | Glepidotin B |
| MOL004829 | ADRA1B | target | Glepidotin B |
| MOL004833 | NOS2 | target | Phaseolinisoflavan |
| MOL004833 | ESR1 | target | Phaseolinisoflavan |
| MOL004833 | AR | target | Phaseolinisoflavan |
| MOL004833 | PPARG | target | Phaseolinisoflavan |
| MOL004833 | PTGS2 | target | Phaseolinisoflavan |
| MOL004833 | RXRA | target | Phaseolinisoflavan |
| MOL004833 | ACHE | target | Phaseolinisoflavan |
| MOL004833 | ADRA1B | target | Phaseolinisoflavan |
| MOL004833 | ESR2 | target | Phaseolinisoflavan |
| MOL004833 | MAPK14 | target | Phaseolinisoflavan |
| MOL004833 | GSK3B | target | Phaseolinisoflavan |
| MOL004833 | CDK2 | target | Phaseolinisoflavan |
| MOL004833 | CHEK1 | target | Phaseolinisoflavan |
| MOL004833 | PRSS1 | target | Phaseolinisoflavan |
| MOL004833 | CCNA2 | target | Phaseolinisoflavan |
| MOL004835 | NOS2 | target | Glypallichalcone |
| MOL004835 | PTGS1 | target | Glypallichalcone |
| MOL004835 | ESR1 | target | Glypallichalcone |

| | | | |
|---|---|---|---|
| MOL004835 | AR | target | Glypallichalcone |
| MOL004835 | PPARG | target | Glypallichalcone |
| MOL004835 | PTGS2 | target | Glypallichalcone |
| MOL004835 | CA2 | target | Glypallichalcone |
| MOL004835 | ADRA1B | target | Glypallichalcone |
| MOL004835 | SLC6A4 | target | Glypallichalcone |
| MOL004835 | ESR2 | target | Glypallichalcone |
| MOL004835 | MAPK14 | target | Glypallichalcone |
| MOL004835 | GSK3B | target | Glypallichalcone |
| MOL004835 | CDK2 | target | Glypallichalcone |
| MOL004835 | CHEK1 | target | Glypallichalcone |
| MOL004835 | CCNA2 | target | Glypallichalcone |
| MOL004838 | NOS2 | target | 8-(6-hydroxy-2-benzofuranyl)-2,2-dimethyl-5-chromenol |
| MOL004838 | ESR1 | target | 8-(6-hydroxy-2-benzofuranyl)-2,2-dimethyl-5-chromenol |
| MOL004838 | PTGS2 | target | 8-(6-hydroxy-2-benzofuranyl)-2,2-dimethyl-5-chromenol |
| MOL004838 | RXRA | target | 8-(6-hydroxy-2-benzofuranyl)-2,2-dimethyl-5-chromenol |
| MOL004841 | NOS2 | target | Licochalcone B |
| MOL004841 | PTGS1 | target | Licochalcone B |
| MOL004841 | ESR1 | target | Licochalcone B |
| MOL004841 | AR | target | Licochalcone B |
| MOL004841 | PPARG | target | Licochalcone B |
| MOL004841 | PTGS2 | target | Licochalcone B |
| MOL004841 | CA2 | target | Licochalcone B |
| MOL004841 | ESR2 | target | Licochalcone B |
| MOL004841 | MAPK14 | target | Licochalcone B |
| MOL004841 | GSK3B | target | Licochalcone B |
| MOL004841 | CDK2 | target | Licochalcone B |
| MOL004841 | CHEK1 | target | Licochalcone B |
| MOL004841 | CCNA2 | target | Licochalcone B |
| MOL004848 | NOS2 | target | licochalcone G |
| MOL004848 | ESR1 | target | licochalcone G |
| MOL004848 | AR | target | licochalcone G |
| MOL004848 | PPARG | target | licochalcone G |
| MOL004848 | PTGS2 | target | licochalcone G |
| MOL004848 | KDR | target | licochalcone G |
| MOL004848 | ESR2 | target | licochalcone G |
| MOL004848 | MAPK14 | target | licochalcone G |
| MOL004848 | GSK3B | target | licochalcone G |
| MOL004848 | CDK2 | target | licochalcone G |
| MOL004848 | CCNA2 | target | licochalcone G |
| MOL004849 | NOS2 | target | 3-(2,4-dihydroxyphenyl)-8-(1,1-dimethylprop-2-enyl)-7-hydroxy-5-methoxy-coumarin |
| MOL004849 | ESR1 | target | 3-(2,4-dihydroxyphenyl)-8-(1,1-dimethylprop-2-enyl)-7-hydroxy-5-methoxy-coumarin |
| MOL004849 | AR | target | 3-(2,4-dihydroxyphenyl)-8-(1,1-dimethylprop-2-enyl)-7-hydroxy-5-methoxy-coumarin |
| MOL004849 | PPARG | target | 3-(2,4-dihydroxyphenyl)-8-(1,1-dimethylprop-2-enyl)-7-hydroxy-5-methoxy-coumarin |
| MOL004849 | PTGS2 | target | 3-(2,4-dihydroxyphenyl)-8-(1,1-dimethylprop-2-enyl)-7-hydroxy-5-methoxy-coumarin |
| MOL004849 | KDR | target | 3-(2,4-dihydroxyphenyl)-8-(1,1-dimethylprop-2-enyl)-7-hydroxy-5-methoxy-coumarin |
| MOL004849 | ESR2 | target | 3-(2,4-dihydroxyphenyl)-8-(1,1-dimethylprop-2-enyl)-7-hydroxy-5-methoxy-coumarin |
| MOL004849 | DPP4 | target | 3-(2,4-dihydroxyphenyl)-8-(1,1-dimethylprop-2-enyl)-7-hydroxy-5-methoxy-coumarin |
| MOL004849 | MAPK14 | target | 3-(2,4-dihydroxyphenyl)-8-(1,1-dimethylprop-2-enyl)-7-hydroxy-5-methoxy-coumarin |

| | | | |
|---|---|---|---|
| MOL004849 | GSK3B | target | 3-(2,4-dihydroxyphenyl)-8-(1,1-dimethylprop-2-enyl)-7-hydroxy-5-methoxy-coumarin |
| MOL004849 | CDK2 | target | 3-(2,4-dihydroxyphenyl)-8-(1,1-dimethylprop-2-enyl)-7-hydroxy-5-methoxy-coumarin |
| MOL004849 | CHEK1 | target | 3-(2,4-dihydroxyphenyl)-8-(1,1-dimethylprop-2-enyl)-7-hydroxy-5-methoxy-coumarin |
| MOL004849 | PRSS1 | target | 3-(2,4-dihydroxyphenyl)-8-(1,1-dimethylprop-2-enyl)-7-hydroxy-5-methoxy-coumarin |
| MOL004855 | NOS2 | target | Licoricone |
| MOL004855 | ESR1 | target | Licoricone |
| MOL004855 | AR | target | Licoricone |
| MOL004855 | PPARG | target | Licoricone |
| MOL004855 | PTGS2 | target | Licoricone |
| MOL004855 | KDR | target | Licoricone |
| MOL004855 | CHEK1 | target | Licoricone |
| MOL004855 | PRSS1 | target | Licoricone |
| MOL004856 | NOS2 | target | Gancaonin A |
| MOL004856 | ESR1 | target | Gancaonin A |
| MOL004856 | AR | target | Gancaonin A |
| MOL004856 | PPARG | target | Gancaonin A |
| MOL004856 | PTGS2 | target | Gancaonin A |
| MOL004856 | ACHE | target | Gancaonin A |
| MOL004856 | ESR2 | target | Gancaonin A |
| MOL004856 | DPP4 | target | Gancaonin A |
| MOL004856 | GSK3B | target | Gancaonin A |
| MOL004856 | CHEK1 | target | Gancaonin A |
| MOL004856 | PRSS1 | target | Gancaonin A |
| MOL004856 | CCNA2 | target | Gancaonin A |
| MOL004857 | NOS2 | target | Gancaonin B |
| MOL004857 | ESR1 | target | Gancaonin B |
| MOL004857 | AR | target | Gancaonin B |
| MOL004857 | PPARG | target | Gancaonin B |
| MOL004857 | PTGS2 | target | Gancaonin B |
| MOL004857 | KDR | target | Gancaonin B |
| MOL004857 | ADRA1B | target | Gancaonin B |
| MOL004857 | ESR2 | target | Gancaonin B |
| MOL004857 | DPP4 | target | Gancaonin B |
| MOL004857 | GSK3B | target | Gancaonin B |
| MOL004857 | CHEK1 | target | Gancaonin B |
| MOL004857 | PRSS1 | target | Gancaonin B |
| MOL004857 | CCNA2 | target | Gancaonin B |
| MOL004863 | NOS2 | target | 3-(3,4-dihydroxyphenyl)-5,7-dihydroxy-8-(3-methylbut-2-enyl)chromone |
| MOL004863 | ESR1 | target | 3-(3,4-dihydroxyphenyl)-5,7-dihydroxy-8-(3-methylbut-2-enyl)chromone |
| MOL004863 | AR | target | 3-(3,4-dihydroxyphenyl)-5,7-dihydroxy-8-(3-methylbut-2-enyl)chromone |
| MOL004863 | PPARG | target | 3-(3,4-dihydroxyphenyl)-5,7-dihydroxy-8-(3-methylbut-2-enyl)chromone |
| MOL004863 | PTGS2 | target | 3-(3,4-dihydroxyphenyl)-5,7-dihydroxy-8-(3-methylbut-2-enyl)chromone |
| MOL004863 | MAPK14 | target | 3-(3,4-dihydroxyphenyl)-5,7-dihydroxy-8-(3-methylbut-2-enyl)chromone |
| MOL004863 | GSK3B | target | 3-(3,4-dihydroxyphenyl)-5,7-dihydroxy-8-(3-methylbut-2-enyl)chromone |
| MOL004863 | CDK2 | target | 3-(3,4-dihydroxyphenyl)-5,7-dihydroxy-8-(3-methylbut-2-enyl)chromone |

| | | | |
|---|---|---|---|
| MOL004863 | CHEK1 | target | 3-(3,4-dihydroxyphenyl)-5,7-dihydroxy-8-(3-methylbut-2-enyl)chromone |
| MOL004863 | PRSS1 | target | 3-(3,4-dihydroxyphenyl)-5,7-dihydroxy-8-(3-methylbut-2-enyl)chromone |
| MOL004863 | CCNA2 | target | 3-(3,4-dihydroxyphenyl)-5,7-dihydroxy-8-(3-methylbut-2-enyl)chromone |
| MOL004864 | NOS2 | target | 5,7-dihydroxy-3-(4-methoxyphenyl)-8-(3-methylbut-2-enyl)chromone |
| MOL004864 | ESR1 | target | 5,7-dihydroxy-3-(4-methoxyphenyl)-8-(3-methylbut-2-enyl)chromone |
| MOL004864 | AR | target | 5,7-dihydroxy-3-(4-methoxyphenyl)-8-(3-methylbut-2-enyl)chromone |
| MOL004864 | PPARG | target | 5,7-dihydroxy-3-(4-methoxyphenyl)-8-(3-methylbut-2-enyl)chromone |
| MOL004864 | PTGS2 | target | 5,7-dihydroxy-3-(4-methoxyphenyl)-8-(3-methylbut-2-enyl)chromone |
| MOL004864 | ESR2 | target | 5,7-dihydroxy-3-(4-methoxyphenyl)-8-(3-methylbut-2-enyl)chromone |
| MOL004864 | DPP4 | target | 5,7-dihydroxy-3-(4-methoxyphenyl)-8-(3-methylbut-2-enyl)chromone |
| MOL004864 | MAPK14 | target | 5,7-dihydroxy-3-(4-methoxyphenyl)-8-(3-methylbut-2-enyl)chromone |
| MOL004864 | GSK3B | target | 5,7-dihydroxy-3-(4-methoxyphenyl)-8-(3-methylbut-2-enyl)chromone |
| MOL004864 | CDK2 | target | 5,7-dihydroxy-3-(4-methoxyphenyl)-8-(3-methylbut-2-enyl)chromone |
| MOL004864 | CHEK1 | target | 5,7-dihydroxy-3-(4-methoxyphenyl)-8-(3-methylbut-2-enyl)chromone |
| MOL004864 | PRSS1 | target | 5,7-dihydroxy-3-(4-methoxyphenyl)-8-(3-methylbut-2-enyl)chromone |
| MOL004864 | CCNA2 | target | 5,7-dihydroxy-3-(4-methoxyphenyl)-8-(3-methylbut-2-enyl)chromone |
| MOL004866 | AR | target | 2-(3,4-dihydroxyphenyl)-5,7-dihydroxy-6-(3-methylbut-2-enyl)chromone |
| MOL004866 | PPARG | target | 2-(3,4-dihydroxyphenyl)-5,7-dihydroxy-6-(3-methylbut-2-enyl)chromone |
| MOL004866 | PTGS2 | target | 2-(3,4-dihydroxyphenyl)-5,7-dihydroxy-6-(3-methylbut-2-enyl)chromone |
| MOL004866 | DPP4 | target | 2-(3,4-dihydroxyphenyl)-5,7-dihydroxy-6-(3-methylbut-2-enyl)chromone |
| MOL004866 | CDK2 | target | 2-(3,4-dihydroxyphenyl)-5,7-dihydroxy-6-(3-methylbut-2-enyl)chromone |
| MOL004866 | CHEK1 | target | 2-(3,4-dihydroxyphenyl)-5,7-dihydroxy-6-(3-methylbut-2-enyl)chromone |
| MOL004866 | PRSS1 | target | 2-(3,4-dihydroxyphenyl)-5,7-dihydroxy-6-(3-methylbut-2-enyl)chromone |
| MOL004866 | CCNA2 | target | 2-(3,4-dihydroxyphenyl)-5,7-dihydroxy-6-(3-methylbut-2-enyl)chromone |
| MOL004879 | NOS2 | target | Glycyrin |
| MOL004879 | ESR1 | target | Glycyrin |
| MOL004879 | AR | target | Glycyrin |
| MOL004879 | PPARG | target | Glycyrin |
| MOL004879 | PTGS2 | target | Glycyrin |
| MOL004879 | KDR | target | Glycyrin |
| MOL004879 | ESR2 | target | Glycyrin |
| MOL004879 | DPP4 | target | Glycyrin |
| MOL004879 | CHEK1 | target | Glycyrin |
| MOL004879 | PRSS1 | target | Glycyrin |

| | | | |
|---|---|---|---|
| MOL004882 | ESR1 | target | Licocoumarone |
| MOL004882 | AR | target | Licocoumarone |
| MOL004882 | ESR2 | target | Licocoumarone |
| MOL004882 | GSK3B | target | Licocoumarone |
| MOL004882 | CDK2 | target | Licocoumarone |
| MOL004882 | CCNA2 | target | Licocoumarone |
| MOL004883 | NOS2 | target | Licoisoflavone |
| MOL004883 | ESR1 | target | Licoisoflavone |
| MOL004883 | AR | target | Licoisoflavone |
| MOL004883 | PPARG | target | Licoisoflavone |
| MOL004883 | PTGS2 | target | Licoisoflavone |
| MOL004883 | KDR | target | Licoisoflavone |
| MOL004883 | DPP4 | target | Licoisoflavone |
| MOL004883 | MAPK14 | target | Licoisoflavone |
| MOL004883 | CDK2 | target | Licoisoflavone |
| MOL004883 | CHEK1 | target | Licoisoflavone |
| MOL004883 | PRSS1 | target | Licoisoflavone |
| MOL004883 | CCNA2 | target | Licoisoflavone |
| MOL004884 | NOS2 | target | Licoisoflavone B |
| MOL004884 | ESR1 | target | Licoisoflavone B |
| MOL004884 | AR | target | Licoisoflavone B |
| MOL004884 | PPARG | target | Licoisoflavone B |
| MOL004884 | PTGS2 | target | Licoisoflavone B |
| MOL004884 | ACHE | target | Licoisoflavone B |
| MOL004884 | ESR2 | target | Licoisoflavone B |
| MOL004884 | GSK3B | target | Licoisoflavone B |
| MOL004884 | CDK2 | target | Licoisoflavone B |
| MOL004884 | CHEK1 | target | Licoisoflavone B |
| MOL004884 | PRSS1 | target | Licoisoflavone B |
| MOL004884 | CCNA2 | target | Licoisoflavone B |
| MOL004885 | NOS2 | target | licoisoflavanone |
| MOL004885 | PTGS1 | target | licoisoflavanone |
| MOL004885 | ESR1 | target | licoisoflavanone |
| MOL004885 | AR | target | licoisoflavanone |
| MOL004885 | PPARG | target | licoisoflavanone |
| MOL004885 | PTGS2 | target | licoisoflavanone |
| MOL004885 | ACHE | target | licoisoflavanone |
| MOL004885 | ESR2 | target | licoisoflavanone |
| MOL004885 | GSK3B | target | licoisoflavanone |
| MOL004885 | CDK2 | target | licoisoflavanone |
| MOL004885 | PRSS1 | target | licoisoflavanone |
| MOL004885 | CCNA2 | target | licoisoflavanone |
| MOL004891 | NOS2 | target | shinpterocarpin |
| MOL004891 | PTGS1 | target | shinpterocarpin |
| MOL004891 | ESR1 | target | shinpterocarpin |
| MOL004891 | AR | target | shinpterocarpin |
| MOL004891 | PPARG | target | shinpterocarpin |
| MOL004891 | PTGS2 | target | shinpterocarpin |
| MOL004891 | HTR3A | target | shinpterocarpin |
| MOL004891 | RXRA | target | shinpterocarpin |
| MOL004891 | ADRA1B | target | shinpterocarpin |
| MOL004891 | OPRM1 | target | shinpterocarpin |
| MOL004891 | ESR2 | target | shinpterocarpin |
| MOL004891 | MAPK14 | target | shinpterocarpin |
| MOL004891 | GSK3B | target | shinpterocarpin |
| MOL004891 | CDK2 | target | shinpterocarpin |
| MOL004891 | RXRB | target | shinpterocarpin |
| MOL004891 | PRSS1 | target | shinpterocarpin |

| | | | |
|---|---|---|---|
| MOL004891 | CCNA2 | target | shinpterocarpin |
| MOL004898 | ESR1 | target | (E)-3-[3,4-dihydroxy-5-(3-methylbut-2-enyl)phenyl]-1-(2,4-dihydroxyphenyl)prop-2-en-1-one |
| MOL004898 | AR | target | (E)-3-[3,4-dihydroxy-5-(3-methylbut-2-enyl)phenyl]-1-(2,4-dihydroxyphenyl)prop-2-en-1-one |
| MOL004898 | PPARG | target | (E)-3-[3,4-dihydroxy-5-(3-methylbut-2-enyl)phenyl]-1-(2,4-dihydroxyphenyl)prop-2-en-1-one |
| MOL004898 | PTGS2 | target | (E)-3-[3,4-dihydroxy-5-(3-methylbut-2-enyl)phenyl]-1-(2,4-dihydroxyphenyl)prop-2-en-1-one |
| MOL004898 | MAPK14 | target | (E)-3-[3,4-dihydroxy-5-(3-methylbut-2-enyl)phenyl]-1-(2,4-dihydroxyphenyl)prop-2-en-1-one |
| MOL004898 | GSK3B | target | (E)-3-[3,4-dihydroxy-5-(3-methylbut-2-enyl)phenyl]-1-(2,4-dihydroxyphenyl)prop-2-en-1-one |
| MOL004898 | CDK2 | target | (E)-3-[3,4-dihydroxy-5-(3-methylbut-2-enyl)phenyl]-1-(2,4-dihydroxyphenyl)prop-2-en-1-one |
| MOL004898 | CCNA2 | target | (E)-3-[3,4-dihydroxy-5-(3-methylbut-2-enyl)phenyl]-1-(2,4-dihydroxyphenyl)prop-2-en-1-one |
| MOL004903 | PTGS2 | target | liquiritin |
| MOL004903 | KDR | target | liquiritin |
| MOL004903 | SOD1 | target | liquiritin |
| MOL004904 | NOS2 | target | licopyranocoumarin |
| MOL004904 | ESR1 | target | licopyranocoumarin |
| MOL004904 | AR | target | licopyranocoumarin |
| MOL004904 | PPARG | target | licopyranocoumarin |
| MOL004904 | PTGS2 | target | licopyranocoumarin |
| MOL004904 | KDR | target | licopyranocoumarin |
| MOL004904 | ACHE | target | licopyranocoumarin |
| MOL004904 | CDK2 | target | licopyranocoumarin |
| MOL004904 | PRSS1 | target | licopyranocoumarin |
| MOL004904 | CCNA2 | target | licopyranocoumarin |
| MOL004907 | NOS2 | target | Glyzaglabrin |
| MOL004907 | PTGS1 | target | Glyzaglabrin |
| MOL004907 | ESR1 | target | Glyzaglabrin |
| MOL004907 | AR | target | Glyzaglabrin |
| MOL004907 | PPARG | target | Glyzaglabrin |
| MOL004907 | PTGS2 | target | Glyzaglabrin |
| MOL004907 | ESR2 | target | Glyzaglabrin |
| MOL004907 | DPP4 | target | Glyzaglabrin |
| MOL004907 | MAPK14 | target | Glyzaglabrin |
| MOL004907 | GSK3B | target | Glyzaglabrin |
| MOL004907 | CDK2 | target | Glyzaglabrin |
| MOL004907 | CHEK1 | target | Glyzaglabrin |
| MOL004907 | PRSS1 | target | Glyzaglabrin |
| MOL004907 | CCNA2 | target | Glyzaglabrin |
| MOL004908 | NOS2 | target | Glabridin |
| MOL004908 | ESR1 | target | Glabridin |
| MOL004908 | AR | target | Glabridin |
| MOL004908 | PPARG | target | Glabridin |
| MOL004908 | PTGS2 | target | Glabridin |
| MOL004908 | RXRA | target | Glabridin |
| MOL004908 | ACHE | target | Glabridin |
| MOL004908 | ADRA1B | target | Glabridin |
| MOL004908 | ESR2 | target | Glabridin |
| MOL004908 | MAPK14 | target | Glabridin |
| MOL004908 | GSK3B | target | Glabridin |
| MOL004908 | CDK2 | target | Glabridin |
| MOL004908 | CHEK1 | target | Glabridin |
| MOL004908 | RXRB | target | Glabridin |

| | | | |
|---|---|---|---|
| MOL004908 | PRSS1 | target | Glabridin |
| MOL004908 | CCNA2 | target | Glabridin |
| MOL004910 | NOS2 | target | Glabranin |
| MOL004910 | PTGS1 | target | Glabranin |
| MOL004910 | ESR1 | target | Glabranin |
| MOL004910 | PTGS2 | target | Glabranin |
| MOL004911 | NOS2 | target | Glabrene |
| MOL004911 | PTGS1 | target | Glabrene |
| MOL004911 | ESR1 | target | Glabrene |
| MOL004911 | AR | target | Glabrene |
| MOL004911 | PPARG | target | Glabrene |
| MOL004911 | PTGS2 | target | Glabrene |
| MOL004911 | RXRA | target | Glabrene |
| MOL004911 | ESR2 | target | Glabrene |
| MOL004911 | MAPK14 | target | Glabrene |
| MOL004911 | GSK3B | target | Glabrene |
| MOL004911 | CDK2 | target | Glabrene |
| MOL004911 | PRSS1 | target | Glabrene |
| MOL004912 | NOS2 | target | Glabrone |
| MOL004912 | PTGS1 | target | Glabrone |
| MOL004912 | ESR1 | target | Glabrone |
| MOL004912 | AR | target | Glabrone |
| MOL004912 | PPARG | target | Glabrone |
| MOL004912 | PTGS2 | target | Glabrone |
| MOL004912 | RXRA | target | Glabrone |
| MOL004912 | ACHE | target | Glabrone |
| MOL004912 | ESR2 | target | Glabrone |
| MOL004912 | DPP4 | target | Glabrone |
| MOL004912 | MAPK14 | target | Glabrone |
| MOL004912 | GSK3B | target | Glabrone |
| MOL004912 | CDK2 | target | Glabrone |
| MOL004912 | CHEK1 | target | Glabrone |
| MOL004912 | PRSS1 | target | Glabrone |
| MOL004912 | CCNA2 | target | Glabrone |
| MOL004913 | ESR1 | target | 1,3-dihydroxy-9-methoxy-6-benzofurano[3,2-c]chromenone |
| MOL004913 | PPARG | target | 1,3-dihydroxy-9-methoxy-6-benzofurano[3,2-c]chromenone |
| MOL004913 | ESR2 | target | 1,3-dihydroxy-9-methoxy-6-benzofurano[3,2-c]chromenone |
| MOL004913 | MAPK14 | target | 1,3-dihydroxy-9-methoxy-6-benzofurano[3,2-c]chromenone |
| MOL004913 | GSK3B | target | 1,3-dihydroxy-9-methoxy-6-benzofurano[3,2-c]chromenone |
| MOL004913 | CDK2 | target | 1,3-dihydroxy-9-methoxy-6-benzofurano[3,2-c]chromenone |
| MOL004913 | CHEK1 | target | 1,3-dihydroxy-9-methoxy-6-benzofurano[3,2-c]chromenone |
| MOL004913 | CCNA2 | target | 1,3-dihydroxy-9-methoxy-6-benzofurano[3,2-c]chromenone |
| MOL004914 | ESR1 | target | 1,3-dihydroxy-8,9-dimethoxy-6-benzofurano[3,2-c]chromenone |
| MOL004914 | AR | target | 1,3-dihydroxy-8,9-dimethoxy-6-benzofurano[3,2-c]chromenone |
| MOL004914 | PPARG | target | 1,3-dihydroxy-8,9-dimethoxy-6-benzofurano[3,2-c]chromenone |
| MOL004914 | MAPK14 | target | 1,3-dihydroxy-8,9-dimethoxy-6-benzofurano[3,2-c]chromenone |
| MOL004914 | GSK3B | target | 1,3-dihydroxy-8,9-dimethoxy-6-benzofurano[3,2-c]chromenone |
| MOL004914 | CDK2 | target | 1,3-dihydroxy-8,9-dimethoxy-6-benzofurano[3,2-c]chromenone |
| MOL004914 | CHEK1 | target | 1,3-dihydroxy-8,9-dimethoxy-6-benzofurano[3,2-c]chromenone |
| MOL004915 | NOS2 | target | Eurycarpin A |
| MOL004915 | ESR1 | target | Eurycarpin A |
| MOL004915 | AR | target | Eurycarpin A |
| MOL004915 | PPARG | target | Eurycarpin A |
| MOL004915 | PTGS2 | target | Eurycarpin A |
| MOL004915 | ESR2 | target | Eurycarpin A |
| MOL004915 | DPP4 | target | Eurycarpin A |
| MOL004915 | MAPK14 | target | Eurycarpin A |
| MOL004915 | GSK3B | target | Eurycarpin A |

| | | | |
|---|---|---|---|
| MOL004915 | CDK2 | target | Eurycarpin A |
| MOL004915 | CHEK1 | target | Eurycarpin A |
| MOL004915 | PRSS1 | target | Eurycarpin A |
| MOL004915 | CCNA2 | target | Eurycarpin A |
| MOL004924 | PTGS2 | target | (-)-Medicocarpin |
| MOL004924 | ACHE | target | (-)-Medicocarpin |
| MOL004935 | ESR1 | target | Sigmoidin-B |
| MOL004935 | PTGS2 | target | Sigmoidin-B |
| MOL004935 | KDR | target | Sigmoidin-B |
| MOL004941 | PTGS1 | target | (2R)-7-hydroxy-2-(4-hydroxyphenyl)chroman-4-one |
| MOL004941 | ESR1 | target | (2R)-7-hydroxy-2-(4-hydroxyphenyl)chroman-4-one |
| MOL004941 | PTGS2 | target | (2R)-7-hydroxy-2-(4-hydroxyphenyl)chroman-4-one |
| MOL004941 | RXRA | target | (2R)-7-hydroxy-2-(4-hydroxyphenyl)chroman-4-one |
| MOL004941 | DPEP1 | target | (2R)-7-hydroxy-2-(4-hydroxyphenyl)chroman-4-one |
| MOL004941 | SLC6A4 | target | (2R)-7-hydroxy-2-(4-hydroxyphenyl)chroman-4-one |
| MOL004945 | NOS2 | target | (2S)-7-hydroxy-2-(4-hydroxyphenyl)-8-(3-methylbut-2-enyl)chroman-4-one |
| MOL004945 | PTGS1 | target | (2S)-7-hydroxy-2-(4-hydroxyphenyl)-8-(3-methylbut-2-enyl)chroman-4-one |
| MOL004945 | ESR1 | target | (2S)-7-hydroxy-2-(4-hydroxyphenyl)-8-(3-methylbut-2-enyl)chroman-4-one |
| MOL004945 | PTGS2 | target | (2S)-7-hydroxy-2-(4-hydroxyphenyl)-8-(3-methylbut-2-enyl)chroman-4-one |
| MOL004945 | ADRA1B | target | (2S)-7-hydroxy-2-(4-hydroxyphenyl)-8-(3-methylbut-2-enyl)chroman-4-one |
| MOL004945 | ESR2 | target | (2S)-7-hydroxy-2-(4-hydroxyphenyl)-8-(3-methylbut-2-enyl)chroman-4-one |
| MOL004948 | NOS2 | target | Isoglycyrol |
| MOL004948 | ESR1 | target | Isoglycyrol |
| MOL004948 | AR | target | Isoglycyrol |
| MOL004948 | PTGS2 | target | Isoglycyrol |
| MOL004948 | DPP4 | target | Isoglycyrol |
| MOL004948 | GSK3B | target | Isoglycyrol |
| MOL004949 | NOS2 | target | Isolicoflavonol |
| MOL004949 | ESR1 | target | Isolicoflavonol |
| MOL004949 | AR | target | Isolicoflavonol |
| MOL004949 | PPARG | target | Isolicoflavonol |
| MOL004949 | PTGS2 | target | Isolicoflavonol |
| MOL004949 | GSK3B | target | Isolicoflavonol |
| MOL004949 | CDK2 | target | Isolicoflavonol |
| MOL004949 | PRSS1 | target | Isolicoflavonol |
| MOL004949 | CCNA2 | target | Isolicoflavonol |
| MOL004957 | NOS2 | target | HMO |
| MOL004957 | PTGS1 | target | HMO |
| MOL004957 | ESR1 | target | HMO |
| MOL004957 | AR | target | HMO |
| MOL004957 | PPARG | target | HMO |
| MOL004957 | PTGS2 | target | HMO |
| MOL004957 | RXRA | target | HMO |
| MOL004957 | SLC6A4 | target | HMO |
| MOL004957 | ESR2 | target | HMO |
| MOL004957 | DPP4 | target | HMO |
| MOL004957 | MAPK14 | target | HMO |
| MOL004957 | GSK3B | target | HMO |
| MOL004957 | CDK2 | target | HMO |
| MOL004957 | CHEK1 | target | HMO |
| MOL004957 | PRSS1 | target | HMO |
| MOL004957 | CCNA2 | target | HMO |

| | | | |
|---|---|---|---|
| MOL004959 | NOS2 | target | 1-Methoxyphaseollidin |
| MOL004959 | PTGS1 | target | 1-Methoxyphaseollidin |
| MOL004959 | ESR1 | target | 1-Methoxyphaseollidin |
| MOL004959 | AR | target | 1-Methoxyphaseollidin |
| MOL004959 | PPARG | target | 1-Methoxyphaseollidin |
| MOL004959 | PTGS2 | target | 1-Methoxyphaseollidin |
| MOL004959 | KDR | target | 1-Methoxyphaseollidin |
| MOL004959 | RXRA | target | 1-Methoxyphaseollidin |
| MOL004959 | ADRA1B | target | 1-Methoxyphaseollidin |
| MOL004959 | ESR2 | target | 1-Methoxyphaseollidin |
| MOL004959 | MAPK14 | target | 1-Methoxyphaseollidin |
| MOL004959 | GSK3B | target | 1-Methoxyphaseollidin |
| MOL004959 | CDK2 | target | 1-Methoxyphaseollidin |
| MOL004959 | PRSS1 | target | 1-Methoxyphaseollidin |
| MOL004959 | CCNA2 | target | 1-Methoxyphaseollidin |
| MOL004961 | NOS2 | target | Quercetin der. |
| MOL004961 | PTGS1 | target | Quercetin der. |
| MOL004961 | ESR1 | target | Quercetin der. |
| MOL004961 | AR | target | Quercetin der. |
| MOL004961 | PPARG | target | Quercetin der. |
| MOL004961 | PTGS2 | target | Quercetin der. |
| MOL004961 | ESR2 | target | Quercetin der. |
| MOL004961 | DPP4 | target | Quercetin der. |
| MOL004961 | MAPK14 | target | Quercetin der. |
| MOL004961 | GSK3B | target | Quercetin der. |
| MOL004961 | CDK2 | target | Quercetin der. |
| MOL004961 | PRSS1 | target | Quercetin der. |
| MOL004966 | NOS2 | target | 3'-Hydroxy-4'-O-Methylglabridin |
| MOL004966 | PTGS1 | target | 3'-Hydroxy-4'-O-Methylglabridin |
| MOL004966 | ESR1 | target | 3'-Hydroxy-4'-O-Methylglabridin |
| MOL004966 | AR | target | 3'-Hydroxy-4'-O-Methylglabridin |
| MOL004966 | PPARG | target | 3'-Hydroxy-4'-O-Methylglabridin |
| MOL004966 | PTGS2 | target | 3'-Hydroxy-4'-O-Methylglabridin |
| MOL004966 | KDR | target | 3'-Hydroxy-4'-O-Methylglabridin |
| MOL004966 | ADRA1B | target | 3'-Hydroxy-4'-O-Methylglabridin |
| MOL004966 | ESR2 | target | 3'-Hydroxy-4'-O-Methylglabridin |
| MOL004966 | MAPK14 | target | 3'-Hydroxy-4'-O-Methylglabridin |
| MOL004966 | GSK3B | target | 3'-Hydroxy-4'-O-Methylglabridin |
| MOL004966 | CDK2 | target | 3'-Hydroxy-4'-O-Methylglabridin |
| MOL004966 | CHEK1 | target | 3'-Hydroxy-4'-O-Methylglabridin |
| MOL004966 | PRSS1 | target | 3'-Hydroxy-4'-O-Methylglabridin |
| MOL004966 | CCNA2 | target | 3'-Hydroxy-4'-O-Methylglabridin |
| MOL000497 | NOS2 | target | licochalcone a |
| MOL000497 | PTGS1 | target | licochalcone a |
| MOL000497 | ESR1 | target | licochalcone a |
| MOL000497 | AR | target | licochalcone a |
| MOL000497 | PPARG | target | licochalcone a |
| MOL000497 | PTGS2 | target | licochalcone a |
| MOL000497 | CA2 | target | licochalcone a |
| MOL000497 | ADRA1B | target | licochalcone a |
| MOL000497 | ESR2 | target | licochalcone a |
| MOL000497 | MAPK14 | target | licochalcone a |
| MOL000497 | GSK3B | target | licochalcone a |
| MOL000497 | CDK2 | target | licochalcone a |
| MOL000497 | CHEK1 | target | licochalcone a |
| MOL000497 | CCNA2 | target | licochalcone a |
| MOL000497 | RELA | target | licochalcone a |
| MOL000497 | STAT3 | target | licochalcone a |

| | | | |
|---|---|---|---|
| MOL000497 | CCND1 | target | licochalcone a |
| MOL000497 | BCL2 | target | licochalcone a |
| MOL000497 | MAPK1 | target | licochalcone a |
| MOL000497 | RB1 | target | licochalcone a |
| MOL000497 | CDK4 | target | licochalcone a |
| MOL000497 | FOSL2 | target | licochalcone a |
| MOL004974 | NOS2 | target | 3'-Methoxyglabridin |
| MOL004974 | PTGS1 | target | 3'-Methoxyglabridin |
| MOL004974 | ESR1 | target | 3'-Methoxyglabridin |
| MOL004974 | AR | target | 3'-Methoxyglabridin |
| MOL004974 | PPARG | target | 3'-Methoxyglabridin |
| MOL004974 | PTGS2 | target | 3'-Methoxyglabridin |
| MOL004974 | RXRA | target | 3'-Methoxyglabridin |
| MOL004974 | ACHE | target | 3'-Methoxyglabridin |
| MOL004974 | ADRA1B | target | 3'-Methoxyglabridin |
| MOL004974 | ESR2 | target | 3'-Methoxyglabridin |
| MOL004974 | MAPK14 | target | 3'-Methoxyglabridin |
| MOL004974 | GSK3B | target | 3'-Methoxyglabridin |
| MOL004974 | CDK2 | target | 3'-Methoxyglabridin |
| MOL004974 | CHEK1 | target | 3'-Methoxyglabridin |
| MOL004974 | PRSS1 | target | 3'-Methoxyglabridin |
| MOL004974 | CCNA2 | target | 3'-Methoxyglabridin |
| MOL004978 | NOS2 | target | 2-[(3R)-8,8-dimethyl-3,4-dihydro-2H-pyrano[6,5-f]chromen-3-yl]-5-methoxyphenol |
| MOL004978 | PTGS1 | target | 2-[(3R)-8,8-dimethyl-3,4-dihydro-2H-pyrano[6,5-f]chromen-3-yl]-5-methoxyphenol |
| MOL004978 | ESR1 | target | 2-[(3R)-8,8-dimethyl-3,4-dihydro-2H-pyrano[6,5-f]chromen-3-yl]-5-methoxyphenol |
| MOL004978 | AR | target | 2-[(3R)-8,8-dimethyl-3,4-dihydro-2H-pyrano[6,5-f]chromen-3-yl]-5-methoxyphenol |
| MOL004978 | PPARG | target | 2-[(3R)-8,8-dimethyl-3,4-dihydro-2H-pyrano[6,5-f]chromen-3-yl]-5-methoxyphenol |
| MOL004978 | PTGS2 | target | 2-[(3R)-8,8-dimethyl-3,4-dihydro-2H-pyrano[6,5-f]chromen-3-yl]-5-methoxyphenol |
| MOL004978 | RXRA | target | 2-[(3R)-8,8-dimethyl-3,4-dihydro-2H-pyrano[6,5-f]chromen-3-yl]-5-methoxyphenol |
| MOL004978 | ACHE | target | 2-[(3R)-8,8-dimethyl-3,4-dihydro-2H-pyrano[6,5-f]chromen-3-yl]-5-methoxyphenol |
| MOL004978 | ADRA1B | target | 2-[(3R)-8,8-dimethyl-3,4-dihydro-2H-pyrano[6,5-f]chromen-3-yl]-5-methoxyphenol |
| MOL004978 | ESR2 | target | 2-[(3R)-8,8-dimethyl-3,4-dihydro-2H-pyrano[6,5-f]chromen-3-yl]-5-methoxyphenol |
| MOL004978 | MAPK14 | target | 2-[(3R)-8,8-dimethyl-3,4-dihydro-2H-pyrano[6,5-f]chromen-3-yl]-5-methoxyphenol |
| MOL004978 | GSK3B | target | 2-[(3R)-8,8-dimethyl-3,4-dihydro-2H-pyrano[6,5-f]chromen-3-yl]-5-methoxyphenol |
| MOL004978 | CDK2 | target | 2-[(3R)-8,8-dimethyl-3,4-dihydro-2H-pyrano[6,5-f]chromen-3-yl]-5-methoxyphenol |
| MOL004978 | CHEK1 | target | 2-[(3R)-8,8-dimethyl-3,4-dihydro-2H-pyrano[6,5-f]chromen-3-yl]-5-methoxyphenol |
| MOL004978 | RXRB | target | 2-[(3R)-8,8-dimethyl-3,4-dihydro-2H-pyrano[6,5-f]chromen-3-yl]-5-methoxyphenol |
| MOL004978 | PRSS1 | target | 2-[(3R)-8,8-dimethyl-3,4-dihydro-2H-pyrano[6,5-f]chromen-3-yl]-5-methoxyphenol |
| MOL004978 | CCNA2 | target | 2-[(3R)-8,8-dimethyl-3,4-dihydro-2H-pyrano[6,5-f]chromen-3-yl]-5-methoxyphenol |
| MOL004980 | ESR1 | target | Inflacoumarin A |
| MOL004980 | AR | target | Inflacoumarin A |

| | | | |
|---|---|---|---|
| MOL004980 | PPARG | target | Inflacoumarin A |
| MOL004980 | PTGS2 | target | Inflacoumarin A |
| MOL004980 | DPP4 | target | Inflacoumarin A |
| MOL004980 | PRSS1 | target | Inflacoumarin A |
| MOL004980 | PTGS1 | target | Inflacoumarin A |
| MOL004988 | ESR1 | target | Kanzonol F |
| MOL004988 | AR | target | Kanzonol F |
| MOL004988 | PTGS2 | target | Kanzonol F |
| MOL004988 | ESR2 | target | Kanzonol F |
| MOL004989 | NOS2 | target | 6-prenylated eriodictyol |
| MOL004989 | ESR1 | target | 6-prenylated eriodictyol |
| MOL004989 | PTGS2 | target | 6-prenylated eriodictyol |
| MOL004990 | NOS2 | target | 7,2',4'-trihydroxy－5-methoxy-3－arylcoumarin |
| MOL004990 | PTGS1 | target | 7,2',4'-trihydroxy－5-methoxy-3－arylcoumarin |
| MOL004990 | ESR1 | target | 7,2',4'-trihydroxy－5-methoxy-3－arylcoumarin |
| MOL004990 | AR | target | 7,2',4'-trihydroxy－5-methoxy-3－arylcoumarin |
| MOL004990 | PPARG | target | 7,2',4'-trihydroxy－5-methoxy-3－arylcoumarin |
| MOL004990 | PTGS2 | target | 7,2',4'-trihydroxy－5-methoxy-3－arylcoumarin |
| MOL004990 | ESR2 | target | 7,2',4'-trihydroxy－5-methoxy-3－arylcoumarin |
| MOL004990 | DPP4 | target | 7,2',4'-trihydroxy－5-methoxy-3－arylcoumarin |
| MOL004990 | MAPK14 | target | 7,2',4'-trihydroxy－5-methoxy-3－arylcoumarin |
| MOL004990 | GSK3B | target | 7,2',4'-trihydroxy－5-methoxy-3－arylcoumarin |
| MOL004990 | CDK2 | target | 7,2',4'-trihydroxy－5-methoxy-3－arylcoumarin |
| MOL004990 | CHEK1 | target | 7,2',4'-trihydroxy－5-methoxy-3－arylcoumarin |
| MOL004991 | NOS2 | target | 7-Acetoxy-2-methylisoflavone |
| MOL004991 | PTGS1 | target | 7-Acetoxy-2-methylisoflavone |
| MOL004991 | ESR1 | target | 7-Acetoxy-2-methylisoflavone |
| MOL004991 | AR | target | 7-Acetoxy-2-methylisoflavone |
| MOL004991 | PPARG | target | 7-Acetoxy-2-methylisoflavone |
| MOL004991 | PTGS2 | target | 7-Acetoxy-2-methylisoflavone |
| MOL004991 | RXRA | target | 7-Acetoxy-2-methylisoflavone |
| MOL004991 | ACHE | target | 7-Acetoxy-2-methylisoflavone |
| MOL004991 | ADRA1B | target | 7-Acetoxy-2-methylisoflavone |
| MOL004991 | DPP4 | target | 7-Acetoxy-2-methylisoflavone |
| MOL004991 | MAPK14 | target | 7-Acetoxy-2-methylisoflavone |
| MOL004991 | GSK3B | target | 7-Acetoxy-2-methylisoflavone |
| MOL004991 | CDK2 | target | 7-Acetoxy-2-methylisoflavone |
| MOL004991 | CHEK1 | target | 7-Acetoxy-2-methylisoflavone |
| MOL004991 | PRSS1 | target | 7-Acetoxy-2-methylisoflavone |
| MOL004993 | ESR1 | target | 8-prenylated eriodictyol |
| MOL004993 | PTGS2 | target | 8-prenylated eriodictyol |
| MOL000500 | NOS2 | target | Vestitol |
| MOL000500 | PTGS1 | target | Vestitol |
| MOL000500 | ESR1 | target | Vestitol |
| MOL000500 | AR | target | Vestitol |
| MOL000500 | PPARG | target | Vestitol |
| MOL000500 | PTGS2 | target | Vestitol |
| MOL000500 | RXRA | target | Vestitol |
| MOL000500 | ADRA1B | target | Vestitol |
| MOL000500 | SLC6A4 | target | Vestitol |
| MOL000500 | ESR2 | target | Vestitol |
| MOL000500 | DPP4 | target | Vestitol |
| MOL000500 | MAPK14 | target | Vestitol |
| MOL000500 | GSK3B | target | Vestitol |
| MOL000500 | CDK2 | target | Vestitol |
| MOL000500 | CHEK1 | target | Vestitol |
| MOL000500 | PRSS1 | target | Vestitol |
| MOL000500 | CCNA2 | target | Vestitol |

| | | | |
|---|---|---|---|
| MOL005000 | NOS2 | target | Gancaonin G |
| MOL005000 | ESR1 | target | Gancaonin G |
| MOL005000 | AR | target | Gancaonin G |
| MOL005000 | PPARG | target | Gancaonin G |
| MOL005000 | PTGS2 | target | Gancaonin G |
| MOL005000 | ESR2 | target | Gancaonin G |
| MOL005000 | DPP4 | target | Gancaonin G |
| MOL005000 | MAPK14 | target | Gancaonin G |
| MOL005000 | GSK3B | target | Gancaonin G |
| MOL005000 | CHEK1 | target | Gancaonin G |
| MOL005000 | PRSS1 | target | Gancaonin G |
| MOL005000 | CCNA2 | target | Gancaonin G |
| MOL005001 | ESR1 | target | Gancaonin H |
| MOL005001 | AR | target | Gancaonin H |
| MOL005001 | PTGS2 | target | Gancaonin H |
| MOL005001 | KDR | target | Gancaonin H |
| MOL005001 | PRSS1 | target | Gancaonin H |
| MOL005001 | CCNA2 | target | Gancaonin H |
| MOL005003 | NOS2 | target | Licoagrocarpin |
| MOL005003 | PTGS1 | target | Licoagrocarpin |
| MOL005003 | ESR1 | target | Licoagrocarpin |
| MOL005003 | AR | target | Licoagrocarpin |
| MOL005003 | PPARG | target | Licoagrocarpin |
| MOL005003 | PTGS2 | target | Licoagrocarpin |
| MOL005003 | RXRA | target | Licoagrocarpin |
| MOL005003 | ACHE | target | Licoagrocarpin |
| MOL005003 | ADRA1B | target | Licoagrocarpin |
| MOL005003 | ESR2 | target | Licoagrocarpin |
| MOL005003 | MAPK14 | target | Licoagrocarpin |
| MOL005003 | GSK3B | target | Licoagrocarpin |
| MOL005003 | CDK2 | target | Licoagrocarpin |
| MOL005003 | RXRB | target | Licoagrocarpin |
| MOL005003 | PRSS1 | target | Licoagrocarpin |
| MOL005003 | CCNA2 | target | Licoagrocarpin |
| MOL005007 | NOS2 | target | Glyasperins M |
| MOL005007 | PTGS1 | target | Glyasperins M |
| MOL005007 | ESR1 | target | Glyasperins M |
| MOL005007 | AR | target | Glyasperins M |
| MOL005007 | PPARG | target | Glyasperins M |
| MOL005007 | PTGS2 | target | Glyasperins M |
| MOL005007 | KDR | target | Glyasperins M |
| MOL005007 | ACHE | target | Glyasperins M |
| MOL005007 | ESR2 | target | Glyasperins M |
| MOL005007 | PPARD | target | Glyasperins M |
| MOL005007 | GSK3B | target | Glyasperins M |
| MOL005007 | CDK2 | target | Glyasperins M |
| MOL005007 | PRSS1 | target | Glyasperins M |
| MOL005007 | CCNA2 | target | Glyasperins M |
| MOL005008 | NOS2 | target | Glycyrrhiza flavonol A |
| MOL005008 | ESR1 | target | Glycyrrhiza flavonol A |
| MOL005008 | AR | target | Glycyrrhiza flavonol A |
| MOL005008 | PTGS2 | target | Glycyrrhiza flavonol A |
| MOL005008 | ACHE | target | Glycyrrhiza flavonol A |
| MOL005008 | ESR2 | target | Glycyrrhiza flavonol A |
| MOL005008 | DPP4 | target | Glycyrrhiza flavonol A |
| MOL005008 | GSK3B | target | Glycyrrhiza flavonol A |
| MOL005008 | CDK2 | target | Glycyrrhiza flavonol A |
| MOL005008 | PRSS1 | target | Glycyrrhiza flavonol A |

| | | | |
|---|---|---|---|
| MOL005008 | CCNA2 | target | Glycyrrhiza flavonol A |
| MOL005012 | NOS2 | target | Licoagroisoflavone |
| MOL005012 | ESR1 | target | Licoagroisoflavone |
| MOL005012 | AR | target | Licoagroisoflavone |
| MOL005012 | PPARG | target | Licoagroisoflavone |
| MOL005012 | PTGS2 | target | Licoagroisoflavone |
| MOL005012 | ESR2 | target | Licoagroisoflavone |
| MOL005012 | DPP4 | target | Licoagroisoflavone |
| MOL005012 | MAPK14 | target | Licoagroisoflavone |
| MOL005012 | GSK3B | target | Licoagroisoflavone |
| MOL005012 | CDK2 | target | Licoagroisoflavone |
| MOL005012 | CHEK1 | target | Licoagroisoflavone |
| MOL005012 | PRSS1 | target | Licoagroisoflavone |
| MOL005012 | CCNA2 | target | Licoagroisoflavone |
| MOL005016 | NOS2 | target | Odoratin |
| MOL005016 | PTGS1 | target | Odoratin |
| MOL005016 | ESR1 | target | Odoratin |
| MOL005016 | AR | target | Odoratin |
| MOL005016 | PPARG | target | Odoratin |
| MOL005016 | PTGS2 | target | Odoratin |
| MOL005016 | RXRA | target | Odoratin |
| MOL005016 | ESR2 | target | Odoratin |
| MOL005016 | DPP4 | target | Odoratin |
| MOL005016 | MAPK14 | target | Odoratin |
| MOL005016 | GSK3B | target | Odoratin |
| MOL005016 | CDK2 | target | Odoratin |
| MOL005016 | CHEK1 | target | Odoratin |
| MOL005016 | PRSS1 | target | Odoratin |
| MOL005016 | CCNA2 | target | Odoratin |
| MOL005017 | ESR1 | target | Phaseol |
| MOL005017 | AR | target | Phaseol |
| MOL005017 | PPARG | target | Phaseol |
| MOL005017 | PTGS2 | target | Phaseol |
| MOL005017 | KDR | target | Phaseol |
| MOL005017 | MAPK14 | target | Phaseol |
| MOL005017 | GSK3B | target | Phaseol |
| MOL005017 | CDK2 | target | Phaseol |
| MOL005017 | CHEK1 | target | Phaseol |
| MOL005017 | CCNA2 | target | Phaseol |
| MOL005018 | NOS2 | target | Xambioona |
| MOL005018 | ESR1 | target | Xambioona |
| MOL005018 | PTGS2 | target | Xambioona |
| MOL005018 | ESR2 | target | Xambioona |
| MOL005020 | NOS2 | target | dehydroglyasperins C |
| MOL005020 | ESR1 | target | dehydroglyasperins C |
| MOL005020 | AR | target | dehydroglyasperins C |
| MOL005020 | PPARG | target | dehydroglyasperins C |
| MOL005020 | PTGS2 | target | dehydroglyasperins C |
| MOL005020 | ESR2 | target | dehydroglyasperins C |
| MOL005020 | MAPK14 | target | dehydroglyasperins C |
| MOL005020 | CDK2 | target | dehydroglyasperins C |
| MOL005020 | CHEK1 | target | dehydroglyasperins C |
| MOL005020 | PRSS1 | target | dehydroglyasperins C |
| MOL005020 | CCNA2 | target | dehydroglyasperins C |
| MOL000098 | PTGS1 | target | quercetin |
| MOL000098 | AR | target | quercetin |
| MOL000098 | PPARG | target | quercetin |
| MOL000098 | PTGS2 | target | quercetin |

| | | | |
|---|---|---|---|
| MOL000098 | DPP4 | target | quercetin |
| MOL000098 | AKR1B1 | target | quercetin |
| MOL000098 | PRSS1 | target | quercetin |
| MOL000098 | MMP3 | target | quercetin |
| MOL000098 | RXRA | target | quercetin |
| MOL000098 | ACHE | target | quercetin |
| MOL000098 | RELA | target | quercetin |
| MOL000098 | EGFR | target | quercetin |
| MOL000098 | AKT1 | target | quercetin |
| MOL000098 | VEGFA | target | quercetin |
| MOL000098 | CCND1 | target | quercetin |
| MOL000098 | BCL2 | target | quercetin |
| MOL000098 | BCL2L1 | target | quercetin |
| MOL000098 | FOS | target | quercetin |
| MOL000098 | CDKN1A | target | quercetin |
| MOL000098 | BAX | target | quercetin |
| MOL000098 | CASP9 | target | quercetin |
| MOL000098 | PLAU | target | quercetin |
| MOL000098 | MMP2 | target | quercetin |
| MOL000098 | MMP9 | target | quercetin |
| MOL000098 | MAPK1 | target | quercetin |
| MOL000098 | IL10RA | target | quercetin |
| MOL000098 | EGF | target | quercetin |
| MOL000098 | RB1 | target | quercetin |
| MOL000098 | TNFAIP6 | target | quercetin |
| MOL000098 | JUN | target | quercetin |
| MOL000098 | IL6R | target | quercetin |
| MOL000098 | CASP3 | target | quercetin |
| MOL000098 | TP53 | target | quercetin |
| MOL000098 | NFKBIA | target | quercetin |
| MOL000098 | ODC1 | target | quercetin |
| MOL000098 | CASP8 | target | quercetin |
| MOL000098 | TOP1 | target | quercetin |
| MOL000098 | RAF1 | target | quercetin |
| MOL000098 | SOD1 | target | quercetin |
| MOL000098 | PRKCA | target | quercetin |
| MOL000098 | MMP1 | target | quercetin |
| MOL000098 | HIF1A | target | quercetin |
| MOL000098 | STAT1 | target | quercetin |
| MOL000098 | RUNX1T1 | target | quercetin |
| MOL000098 | CDK1 | target | quercetin |
| MOL000098 | HSPA5 | target | quercetin |
| MOL000098 | ERBB2 | target | quercetin |
| MOL000098 | ACACA | target | quercetin |
| MOL000098 | HMOX1 | target | quercetin |
| MOL000098 | CYP3A4 | target | quercetin |
| MOL000098 | CYP1A2 | target | quercetin |
| MOL000098 | CAV1 | target | quercetin |
| MOL000098 | MYC | target | quercetin |
| MOL000098 | F3 | target | quercetin |
| MOL000098 | GJA1 | target | quercetin |
| MOL000098 | CYP1A1 | target | quercetin |
| MOL000098 | ICAM1 | target | quercetin |
| MOL000098 | IL1B | target | quercetin |
| MOL000098 | CCL2 | target | quercetin |
| MOL000098 | SELE | target | quercetin |
| MOL000098 | VCAM1 | target | quercetin |
| MOL000098 | PTGER3 | target | quercetin |

| | | | |
|---|---|---|---|
| MOL000098 | CXCL8 | target | quercetin |
| MOL000098 | PRKCB | target | quercetin |
| MOL000098 | BIRC5 | target | quercetin |
| MOL000098 | DUOX2 | target | quercetin |
| MOL000098 | NOS3 | target | quercetin |
| MOL000098 | HSPB1 | target | quercetin |
| MOL000098 | IL2RA | target | quercetin |
| MOL000098 | NR1I2 | target | quercetin |
| MOL000098 | CYP1B1 | target | quercetin |
| MOL000098 | CCNB1 | target | quercetin |
| MOL000098 | THBD | target | quercetin |
| MOL000098 | SERPINE1 | target | quercetin |
| MOL000098 | COL1A1 | target | quercetin |
| MOL000098 | IFNG | target | quercetin |
| MOL000098 | ALOX5 | target | quercetin |
| MOL000098 | IL1A | target | quercetin |
| MOL000098 | MPO | target | quercetin |
| MOL000098 | TOP2A | target | quercetin |
| MOL000098 | NCF1 | target | quercetin |
| MOL000098 | ABCG2 | target | quercetin |
| MOL000098 | GSTP1 | target | quercetin |
| MOL000098 | NFE2L2 | target | quercetin |
| MOL000098 | NQO1 | target | quercetin |
| MOL000098 | PARP1 | target | quercetin |
| MOL000098 | AHR | target | quercetin |
| MOL000098 | CXCL11 | target | quercetin |
| MOL000098 | CXCL2 | target | quercetin |
| MOL000098 | CHEK2 | target | quercetin |
| MOL000098 | CLDN4 | target | quercetin |
| MOL000098 | PPARA | target | quercetin |
| MOL000098 | PPARD | target | quercetin |
| MOL000098 | CXCL10 | target | quercetin |
| MOL000098 | CHUK | target | quercetin |
| MOL000098 | SPP1 | target | quercetin |
| MOL000098 | RUNX2 | target | quercetin |
| MOL000098 | RASSF1 | target | quercetin |
| MOL000098 | E2F1 | target | quercetin |
| MOL000098 | CTSD | target | quercetin |
| MOL000098 | IGFBP3 | target | quercetin |
| MOL000098 | IGF2 | target | quercetin |
| MOL000098 | CD40LG | target | quercetin |
| MOL000098 | IRF1 | target | quercetin |
| MOL000098 | ERBB3 | target | quercetin |
| MOL000098 | PON1 | target | quercetin |
| MOL000098 | PCOLCE | target | quercetin |
| MOL000098 | NPEPPS | target | quercetin |
| MOL000098 | HK2 | target | quercetin |
| MOL000098 | GSTM1 | target | quercetin |
| MOL001002 | CDK2 | target | ellagic acid |
| MOL001002 | ESR1 | target | ellagic acid |
| MOL001002 | AR | target | ellagic acid |
| MOL001002 | RELA | target | ellagic acid |
| MOL001002 | VEGFA | target | ellagic acid |
| MOL001002 | CDKN1A | target | ellagic acid |
| MOL001002 | MMP2 | target | ellagic acid |
| MOL001002 | MMP9 | target | ellagic acid |
| MOL001002 | NFKBIA | target | ellagic acid |
| MOL001002 | CXCL8 | target | ellagic acid |

| | | | |
|---|---|---|---|
| MOL001002 | PRKCB | target | ellagic acid |
| MOL001002 | GSTP1 | target | ellagic acid |
| MOL001002 | IGF2 | target | ellagic acid |
| MOL001002 | GSTM1 | target | ellagic acid |
| MOL001002 | GSTA1 | target | ellagic acid |
| MOL001002 | GSTA2 | target | ellagic acid |
| MOL006826 | PTGS2 | target | chebulic acid |
| MOL009135 | PTGS1 | target | ellipticine |
| MOL009135 | PTGS2 | target | ellipticine |
| MOL009135 | RXRA | target | ellipticine |
| MOL009135 | BCL2 | target | ellipticine |
| MOL009135 | BCL2L1 | target | ellipticine |
| MOL009135 | CDKN1A | target | ellipticine |
| MOL009135 | BAX | target | ellipticine |
| MOL009135 | CASP9 | target | ellipticine |
| MOL009135 | CASP3 | target | ellipticine |
| MOL009135 | TP53 | target | ellipticine |
| MOL009135 | CASP8 | target | ellipticine |
| MOL009135 | CDK1 | target | ellipticine |
| MOL009135 | CYP1A2 | target | ellipticine |
| MOL009135 | CYP1A1 | target | ellipticine |
| MOL009135 | CCNB1 | target | ellipticine |
| MOL009135 | XIAP | target | ellipticine |
| MOL009135 | RASGRF1 | target | ellipticine |
| MOL009135 | CDK12 | target | ellipticine |
| MOL009136 | AR | target | Peraksine |
| MOL009136 | HTR3A | target | Peraksine |
| MOL009136 | ACHE | target | Peraksine |
| MOL009136 | ADRA1B | target | Peraksine |
| MOL009136 | SLC6A4 | target | Peraksine |
| MOL009136 | OPRM1 | target | Peraksine |
| MOL009137 | PTGS1 | target | (R)-(6-methoxy-4-quinolyl)-[(2R,4R,5S)-5-vinylquinuclidin-2-yl]methanol |
| MOL009137 | PTGS2 | target | (R)-(6-methoxy-4-quinolyl)-[(2R,4R,5S)-5-vinylquinuclidin-2-yl]methanol |
| MOL009137 | ADRA2A | target | (R)-(6-methoxy-4-quinolyl)-[(2R,4R,5S)-5-vinylquinuclidin-2-yl]methanol |
| MOL009137 | HTR3A | target | (R)-(6-methoxy-4-quinolyl)-[(2R,4R,5S)-5-vinylquinuclidin-2-yl]methanol |
| MOL009137 | RXRA | target | (R)-(6-methoxy-4-quinolyl)-[(2R,4R,5S)-5-vinylquinuclidin-2-yl]methanol |
| MOL009137 | ADRA1B | target | (R)-(6-methoxy-4-quinolyl)-[(2R,4R,5S)-5-vinylquinuclidin-2-yl]methanol |
| MOL009137 | SLC6A4 | target | (R)-(6-methoxy-4-quinolyl)-[(2R,4R,5S)-5-vinylquinuclidin-2-yl]methanol |
| MOL009137 | DRD2 | target | (R)-(6-methoxy-4-quinolyl)-[(2R,4R,5S)-5-vinylquinuclidin-2-yl]methanol |
| MOL009137 | EGFR | target | (R)-(6-methoxy-4-quinolyl)-[(2R,4R,5S)-5-vinylquinuclidin-2-yl]methanol |
| MOL009137 | OPRM1 | target | (R)-(6-methoxy-4-quinolyl)-[(2R,4R,5S)-5-vinylquinuclidin-2-yl]methanol |
| MOL009149 | PTGS1 | target | Cheilanthifoline |
| MOL009149 | PTGS2 | target | Cheilanthifoline |
| MOL009149 | HTR3A | target | Cheilanthifoline |
| MOL009149 | RXRA | target | Cheilanthifoline |
| MOL009149 | ADRA1B | target | Cheilanthifoline |
| MOL009149 | SLC6A4 | target | Cheilanthifoline |
| MOL009149 | OPRM1 | target | Cheilanthifoline |

| | | | |
|---|---|---|---|
| MOL010813 | PTGS1 | target | Benzo[a]carbazole |
| MOL010813 | PTGS2 | target | Benzo[a]carbazole |
| MOL010828 | PTGS2 | target | cynaropicrin |
| MOL009243 | NOS2 | target | Isoguaiacin |
| MOL009243 | PTGS1 | target | Isoguaiacin |
| MOL009243 | ESR1 | target | Isoguaiacin |
| MOL009243 | AR | target | Isoguaiacin |
| MOL009243 | PPARG | target | Isoguaiacin |
| MOL009243 | PTGS2 | target | Isoguaiacin |
| MOL009243 | RXRA | target | Isoguaiacin |
| MOL009243 | ADRA1B | target | Isoguaiacin |
| MOL009243 | OPRM1 | target | Isoguaiacin |
| MOL009243 | ESR2 | target | Isoguaiacin |
| MOL009243 | MAPK14 | target | Isoguaiacin |
| MOL009243 | GSK3B | target | Isoguaiacin |
| MOL009243 | CHEK1 | target | Isoguaiacin |
| MOL009243 | CCNA2 | target | Isoguaiacin |
| MOL009254 | PTGS2 | target | galbacin |
| MOL009254 | RXRA | target | galbacin |
| MOL009255 | PTGS1 | target | 5-[(2S,3S)-7-methoxy-3-methyl-5-[(E)-prop-1-enyl]-2,3-dihydrobenzofuran-2-yl]-1,3-benzodioxole |
| MOL009255 | PTGS2 | target | 5-[(2S,3S)-7-methoxy-3-methyl-5-[(E)-prop-1-enyl]-2,3-dihydrobenzofuran-2-yl]-1,3-benzodioxole |
| MOL009255 | ADRA2A | target | 5-[(2S,3S)-7-methoxy-3-methyl-5-[(E)-prop-1-enyl]-2,3-dihydrobenzofuran-2-yl]-1,3-benzodioxole |
| MOL009255 | ADRA1B | target | 5-[(2S,3S)-7-methoxy-3-methyl-5-[(E)-prop-1-enyl]-2,3-dihydrobenzofuran-2-yl]-1,3-benzodioxole |
| MOL009255 | DPEP1 | target | 5-[(2S,3S)-7-methoxy-3-methyl-5-[(E)-prop-1-enyl]-2,3-dihydrobenzofuran-2-yl]-1,3-benzodioxole |
| MOL009255 | PRSS1 | target | 5-[(2S,3S)-7-methoxy-3-methyl-5-[(E)-prop-1-enyl]-2,3-dihydrobenzofuran-2-yl]-1,3-benzodioxole |
| MOL009255 | ESR1 | target | 5-[(2S,3S)-7-methoxy-3-methyl-5-[(E)-prop-1-enyl]-2,3-dihydrobenzofuran-2-yl]-1,3-benzodioxole |
| MOL009255 | RXRA | target | 5-[(2S,3S)-7-methoxy-3-methyl-5-[(E)-prop-1-enyl]-2,3-dihydrobenzofuran-2-yl]-1,3-benzodioxole |
| MOL009255 | ACHE | target | 5-[(2S,3S)-7-methoxy-3-methyl-5-[(E)-prop-1-enyl]-2,3-dihydrobenzofuran-2-yl]-1,3-benzodioxole |
| MOL009255 | RXRB | target | 5-[(2S,3S)-7-methoxy-3-methyl-5-[(E)-prop-1-enyl]-2,3-dihydrobenzofuran-2-yl]-1,3-benzodioxole |
| MOL009259 | PTGS2 | target | Kudos |
| MOL009259 | DPP4 | target | Kudos |
| MOL009263 | PTGS1 | target | saucernetindiol |
| MOL009263 | ESR1 | target | saucernetindiol |
| MOL009263 | PTGS2 | target | saucernetindiol |
| MOL009263 | RXRA | target | saucernetindiol |
| MOL009263 | ADRA1B | target | saucernetindiol |
| MOL009264 | ESR1 | target | tetrahydrofuroguaiacin B |
| MOL009264 | PTGS2 | target | tetrahydrofuroguaiacin B |
| MOL009264 | ADRA1B | target | tetrahydrofuroguaiacin B |
| MOL009265 | NOS2 | target | threo-austrobailignan-5 |
| MOL009265 | PTGS1 | target | threo-austrobailignan-5 |
| MOL009265 | ESR1 | target | threo-austrobailignan-5 |
| MOL009265 | AR | target | threo-austrobailignan-5 |
| MOL009265 | PPARG | target | threo-austrobailignan-5 |
| MOL009265 | PTGS2 | target | threo-austrobailignan-5 |
| MOL009265 | RXRA | target | threo-austrobailignan-5 |
| MOL009265 | ADRA1B | target | threo-austrobailignan-5 |
| MOL009265 | ESR2 | target | threo-austrobailignan-5 |

| | | | |
|---|---|---|---|
| MOL009265 | MAPK14 | target | threo-austrobailignan-5 |
| MOL009265 | GSK3B | target | threo-austrobailignan-5 |
| MOL009265 | CHEK1 | target | threo-austrobailignan-5 |
| MOL009265 | CCNA2 | target | threo-austrobailignan-5 |
| MOL000131 | PTGS1 | target | EIC |
| MOL000131 | PTGS2 | target | EIC |
| MOL000131 | RXRA | target | EIC |
| MOL000131 | TRPV1 | target | EIC |
| MOL000266 | PTGS2 | target | beta-Cubebene |
| MOL002697 | ADRA1B | target | junipene |
| MOL003522 | PTGS2 | target | ()-Sativene |
| MOL003522 | ADRA1B | target | ()-Sativene |
| MOL002003 | PTGS2 | target | (-)-Caryophyllene oxide |
| MOL002003 | ACHE | target | (-)-Caryophyllene oxide |
| MOL002003 | ADRA1B | target | (-)-Caryophyllene oxide |
| MOL002003 | DPP4 | target | (-)-Caryophyllene oxide |
| MOL000057 | SLC6A4 | target | DIBP |
| MOL000057 | NR3C2 | target | DIBP |
| MOL000057 | NR3C1 | target | DIBP |
| MOL000057 | RXRA | target | DIBP |
| MOL000612 | PTGS2 | target | (-)-alpha-cedrene |
| MOL000612 | RXRA | target | (-)-alpha-cedrene |
| MOL000612 | ADH1B | target | (-)-alpha-cedrene |
| MOL000675 | PTGS1 | target | oleic acid |
| MOL000675 | PTGS2 | target | oleic acid |
| MOL000675 | ADH1B | target | oleic acid |
| MOL000675 | RXRA | target | oleic acid |
| MOL000675 | PLAU | target | oleic acid |
| MOL000675 | SOD1 | target | oleic acid |
| MOL000675 | CAT | target | oleic acid |
| MOL000675 | TEP1 | target | oleic acid |
| MOL000675 | EDN1 | target | oleic acid |
| MOL000675 | ERBB2 | target | oleic acid |
| MOL000675 | PPARG | target | oleic acid |
| MOL000675 | LPL | target | oleic acid |
| MOL000675 | SERPINE1 | target | oleic acid |
| MOL000675 | BDNF | target | oleic acid |
| MOL000675 | HMGCR | target | oleic acid |
| MOL000675 | MPO | target | oleic acid |
| MOL000675 | PPARA | target | oleic acid |
| MOL000675 | PPARD | target | oleic acid |
| MOL000675 | PON1 | target | oleic acid |
| MOL000675 | INS | target | oleic acid |
| MOL000675 | SERPINB2 | target | oleic acid |
| MOL000675 | FABP1 | target | oleic acid |
| MOL000675 | GCG | target | oleic acid |
| MOL000675 | ENPEP | target | oleic acid |
| MOL000675 | UCP2 | target | oleic acid |
| MOL000675 | SOAT1 | target | oleic acid |
| MOL000675 | SCD | target | oleic acid |
| MOL000675 | PYY | target | oleic acid |
| MOL006980 | PTGS1 | target | papaverine |
| MOL006980 | PTGS2 | target | papaverine |
| MOL006980 | RXRA | target | papaverine |
| MOL006980 | ADRA1B | target | papaverine |
| MOL006980 | SLC6A4 | target | papaverine |
| MOL006980 | RXRB | target | papaverine |
| MOL006980 | LPL | target | papaverine |

| | | | |
|---|---|---|---|
| MOL006980 | ADM | target | papaverine |
| MOL006982 | AR | target | codeine |
| MOL006982 | HTR3A | target | codeine |
| MOL006982 | ACHE | target | codeine |
| MOL006982 | HRH1 | target | codeine |
| MOL006982 | ADRA1B | target | codeine |
| MOL006982 | SLC6A4 | target | codeine |
| MOL006982 | DRD2 | target | codeine |
| MOL006982 | OPRM1 | target | codeine |
| MOL000787 | PTGS1 | target | Fumarine |
| MOL000787 | PTGS2 | target | Fumarine |
| MOL000787 | HTR3A | target | Fumarine |
| MOL000787 | ADRA1B | target | Fumarine |
| MOL000787 | OPRM1 | target | Fumarine |
| MOL000787 | SLC6A4 | target | Fumarine |
| MOL000787 | CACNA1S | target | Fumarine |
| MOL000787 | KDR | target | Fumarine |
| MOL009324 | NR3C2 | target | Cryptogenin |
| MOL009327 | AR | target | Noskapin |
| MOL009327 | PTGS2 | target | Noskapin |
| MOL009327 | KDR | target | Noskapin |
| MOL009327 | ACHE | target | Noskapin |
| MOL009327 | PRSS1 | target | Noskapin |
| MOL009328 | PTGS1 | target | 5-[[(1S)-6,7-dimethoxy-2-methyl-3,4-dihydro-1H-isoquinolin-1-yl]methyl]-2-methoxyphenol |
| MOL009328 | PTGS2 | target | 5-[[(1S)-6,7-dimethoxy-2-methyl-3,4-dihydro-1H-isoquinolin-1-yl]methyl]-2-methoxyphenol |
| MOL009328 | ADRA2A | target | 5-[[(1S)-6,7-dimethoxy-2-methyl-3,4-dihydro-1H-isoquinolin-1-yl]methyl]-2-methoxyphenol |
| MOL009328 | RXRA | target | 5-[[(1S)-6,7-dimethoxy-2-methyl-3,4-dihydro-1H-isoquinolin-1-yl]methyl]-2-methoxyphenol |
| MOL009328 | ADRA1B | target | 5-[[(1S)-6,7-dimethoxy-2-methyl-3,4-dihydro-1H-isoquinolin-1-yl]methyl]-2-methoxyphenol |
| MOL009328 | SLC6A4 | target | 5-[[(1S)-6,7-dimethoxy-2-methyl-3,4-dihydro-1H-isoquinolin-1-yl]methyl]-2-methoxyphenol |
| MOL009328 | DRD2 | target | 5-[[(1S)-6,7-dimethoxy-2-methyl-3,4-dihydro-1H-isoquinolin-1-yl]methyl]-2-methoxyphenol |
| MOL009328 | OPRM1 | target | 5-[[(1S)-6,7-dimethoxy-2-methyl-3,4-dihydro-1H-isoquinolin-1-yl]methyl]-2-methoxyphenol |
| MOL009328 | NR1I2 | target | 5-[[(1S)-6,7-dimethoxy-2-methyl-3,4-dihydro-1H-isoquinolin-1-yl]methyl]-2-methoxyphenol |
| MOL009328 | RXRB | target | 5-[[(1S)-6,7-dimethoxy-2-methyl-3,4-dihydro-1H-isoquinolin-1-yl]methyl]-2-methoxyphenol |
| MOL009329 | PTGS2 | target | Narcein |
| MOL009329 | KDR | target | Narcein |
| MOL009330 | PTGS1 | target | Noscapine |
| MOL009330 | AR | target | Noscapine |
| MOL009330 | PTGS2 | target | Noscapine |
| MOL009330 | KDR | target | Noscapine |
| MOL009330 | ACHE | target | Noscapine |
| MOL009330 | PRSS1 | target | Noscapine |
| MOL009331 | PTGS1 | target | Palaudine |
| MOL009331 | PTGS2 | target | Palaudine |
| MOL009331 | RXRA | target | Palaudine |
| MOL009331 | ADRA1B | target | Palaudine |
| MOL009335 | PTGS1 | target | Erythroculine |
| MOL009335 | PTGS2 | target | Erythroculine |
| MOL009335 | ADRA1B | target | Erythroculine |

| | | | |
|---|---|---|---|
| MOL009335 | OPRM1 | target | Erythroculine |
| MOL009338 | PTGS1 | target | Norswertianin |
| MOL009338 | AR | target | Norswertianin |
| MOL009338 | PTGS2 | target | Norswertianin |

**molLists**
MOL004863
MOL000131
MOL004841
MOL004945
MOL004924
MOL004912
MOL001484
MOL009328
MOL004827
MOL004948
MOL004864
MOL004805
MOL005008
MOL009137
MOL004966
MOL009136
MOL004808
MOL004891
MOL005018
MOL008397
MOL004911
MOL003896
MOL001006
MOL004815
MOL006982
MOL000358
MOL009265
MOL004885
MOL006826
MOL004974
MOL005020
MOL004935
MOL000675
MOL004820
MOL004884
MOL009338
MOL006980
MOL009264
MOL004978
MOL004959
MOL003656
MOL000354
MOL009335
MOL001924
MOL004857
MOL004883
MOL009263
MOL004856
MOL002697
MOL000449
MOL000072
MOL004814

MOL000359
MOL004910
MOL004961
MOL004949
MOL009329
MOL000417
MOL000022
MOL004907
MOL000057
MOL001002
MOL009324
MOL000612
MOL010813
MOL005003
MOL004980
MOL004993
MOL004838
MOL009243
MOL008400
MOL003522
MOL004835
MOL002140
MOL009149
MOL004988
MOL004879
MOL000392
MOL000266
MOL004811
MOL000098
MOL004915
MOL002565
MOL004855
MOL000787
MOL004882
MOL004355
MOL004898
MOL004829
MOL005001
MOL001792
MOL003036
MOL004866
MOL009259
MOL004991
MOL004908
MOL001919
MOL002003
MOL004941
MOL009330
MOL008407
MOL004824
MOL000492
MOL005012
MOL004848
MOL009254
MOL004903
MOL002311
MOL009327
MOL004828

MOL009255
MOL004904
MOL004849
MOL000422
MOL004810
MOL009135
MOL000239
MOL004806
MOL005007
MOL004989
MOL000497
MOL004957
MOL000006
MOL005016
MOL004990
MOL005017
MOL004914
MOL005321
MOL007059
MOL004833
MOL005000
MOL000500
MOL000049
MOL010828
MOL008411
MOL004328
MOL009331
MOL004913

**geneLists**
NR3C2
TNFAIP6
IL6R
CD14
LBP
PTGS1
PTGS2
ADRA1B
SLC6A4
OPRM1
BCL2
BAX
CASP9
JUN
CASP3
CASP8
PRKCA
PON1
NOS2
AR
PPARG
DPP4
PRSS1
ACHE
RELA
IKBKB
AKT1
MAPK8

MMP1
STAT1
CDK1
HMOX1
CYP3A4
CYP1A2
CYP1A1
ICAM1
SELE
VCAM1
NR1I2
CYP1B1
ALOX5
GSTP1
AHR
GSTM1
AKR1C3
SLPI
ESR1
DPEP1
RXRA
CAT
ADRA2A
AKR1B1
PLAU
ESR2
MAPK14
GSK3B
CDK2
CHEK1
CCNA2
EGFR
VEGFA
CCND1
BCL2L1
CDKN1A
MMP2
MMP9
MAPK1
IL10RA
RB1
CDK4
TP53
NFKBIA
TOP1
MDM2
PCNA
ERBB2
CASP7
MCL1
BIRC5
IL2RA
CCNB1
TYR
IFNG
IL4
TOP2A
XIAP

CD40LG
PTGES
MET
CA2
NR3C1
MMP13
MMP8
HTR3A
KDR
MAPK10
PPARD
NCF1
MAPK3
FASN
BAD
SOD1
APOB
PLB1
HMGCR
CYP19A1
UGT1A1
PPARA
GSR
ABCC1
AKR1C1
GOT1
CES1
SOAT1
RXRB
STAT3
FOSL2
MMP3
FOS
EGF
ODC1
RAF1
HIF1A
RUNX1T1
HSPA5
ACACA
CAV1
MYC
F3
GJA1
IL1B
CCL2
PTGER3
CXCL8
PRKCB
DUOX2
NOS3
HSPB1
THBD
SERPINE1
COL1A1
IL1A
MPO
ABCG2

NFE2L2
NQO1
PARP1
CXCL11
CXCL2
CHEK2
CLDN4
CXCL10
CHUK
SPP1
RUNX2
RASSF1
E2F1
CTSD
IGFBP3
IGF2
IRF1
ERBB3
PCOLCE
NPEPPS
HK2
GSTA1
GSTA2
RASGRF1
CDK12
DRD2
TRPV1
ADH1B
TEP1
EDN1
LPL
BDNF
INS
SERPINB2
FABP1
GCG
ENPEP
UCP2
SCD
PYY
ADM
HRH1
CACNA1S
