# Supplementary material for: Analysis of the Efficacy and Pharmacological Mechanisms of Action of Zhenren Yangzang Decoction on Ulcerative Colitis Using Meta-Analysis and Network Pharmacology
Source: Evid Based Complement Alternat Med. 2021 Dec 28;2021:4512755. doi: 10.1155/2021/4512755 (PMC8727130; doi:10.1155/2021/4512755)
Supplement: Supplementary Materials — Figure S1: Risk of bias graph. Figure S2: risk of bias summary. Figure S3: forest plot of comparison of serum cytokines. Figure S4: forest plot of comparison of the total syndrome score of TCM. Table S1: basic information on the active compounds in ZRYZD. Table S2: gene symbols and entrezID of active target genes. Table S3: compounds ranked by the degree in the network. Supplementary File 1: compounds of ZRYZD from TCMSP. Supplementary File 2: corresponding target genes of ZRYZD. Supplementary File 3: UC-related target genes. Supplementary File 4: GO functional enrichment analysis. Supplementary File 5: KEGG pathway enrichment analysis. Supplementary File 6: data of compound-target networks. Supplementary File 7: data of key compound-target networks. Supplementary File 8: data of PPI network. [file 4512755.f1.zip › 4512755.f1/Supplementary File 7 Data of key compound-target network.pdf]

Supplementary File 7 Data of key compound–target network

Node1	Node2	Net
ellipticine	PTGS1	target
ellipticine	PTGS2	target
ellipticine	RXRA	target
ellipticine	BCL2	target
ellipticine	BCL2L1	target
ellipticine	CDKN1A	target
ellipticine	BAX	target
ellipticine	CASP9	target
ellipticine	CASP3	target
ellipticine	TP53	target
ellipticine	CASP8	target
ellipticine	CDK1	target
ellipticine	CYP1A2	target
ellipticine	CYP1A1	target
ellipticine	CCNB1	target
ellipticine	XIAP	target
ellipticine	RASGRF1	target
ellipticine	CDK12	target
ellagic acid	CDK2	target
ellagic acid	ESR1	target
ellagic acid	AR	target
ellagic acid	RELA	target
ellagic acid	VEGFA	target
ellagic acid	CDKN1A	target
ellagic acid	MMP2	target
ellagic acid	MMP9	target
ellagic acid	NFKBIA	target
ellagic acid	CXCL8	target
ellagic acid	PRKCB	target
ellagic acid	GSTP1	target
ellagic acid	IGF2	target
ellagic acid	GSTM1	target
ellagic acid	GSTA1	target
ellagic acid	GSTA2	target
isoguaiacin	NOS2	target
isoguaiacin	PTGS1	target
isoguaiacin	ESR1	target
isoguaiacin	AR	target
isoguaiacin	PPARG	target
isoguaiacin	PTGS2	target
isoguaiacin	RXRA	target
isoguaiacin	ADRA1B	target
isoguaiacin	OPRM1	target
isoguaiacin	ESR2	target
isoguaiacin	MAPK14	target
isoguaiacin	GSK3B	target
isoguaiacin	CHEK1	target
isoguaiacin	CCNA2	target
beta-sitosterol	PTGS1	target
beta-sitosterol	PTGS2	target
beta-sitosterol	ADRA1B	target
beta-sitosterol	SLC6A4	target
beta-sitosterol	OPRM1	target
beta-sitosterol	BCL2	target
beta-sitosterol	BAX	target
beta-sitosterol	CASP9	target
beta-sitosterol	JUN	target
beta-sitosterol	CASP3	target
beta-sitosterol	CASP8	target
beta-sitosterol	PRKCA	target

beta-sitosterol	PON1	target
(S)-Laudanine	PTGS1	target
(S)-Laudanine	PTGS2	target
(S)-Laudanine	ADRA2A	target
(S)-Laudanine	RXRA	target
(S)-Laudanine	ADRA1B	target
(S)-Laudanine	SLC6A4	target
(S)-Laudanine	DRD2	target
(S)-Laudanine	OPRM1	target
(S)-Laudanine	NR1I2	target
(S)-Laudanine	RXRB	target
protopine	PTGS1	target
protopine	PTGS2	target
protopine	HTR3A	target
protopine	ADRA1B	target
protopine	OPRM1	target
protopine	SLC6A4	target
protopine	CACNA1S	target
protopine	KDR	target
codeine	AR	target
codeine	HTR3A	target
codeine	ACHE	target
codeine	HRH1	target
codeine	ADRA1B	target
codeine	SLC6A4	target
codeine	DRD2	target
codeine	OPRM1	target
papaverine	PTGS1	target
papaverine	PTGS2	target
papaverine	RXRA	target
papaverine	ADRA1B	target
papaverine	SLC6A4	target
papaverine	RXRB	target
papaverine	LPL	target
papaverine	ADM	target
cheilanthifoline	PTGS1	target
cheilanthifoline	PTGS2	target
cheilanthifoline	HTR3A	target
cheilanthifoline	RXRA	target
cheilanthifoline	ADRA1B	target
cheilanthifoline	SLC6A4	target
cheilanthifoline	OPRM1	target
noscapine	PTGS1	target
noscapine	AR	target
noscapine	PTGS2	target
noscapine	KDR	target
noscapine	ACHE	target
noscapine	PRSS1	target
peraksine	AR	target
peraksine	HTR3A	target
peraksine	ACHE	target
peraksine	ADRA1B	target
peraksine	SLC6A4	target
peraksine	OPRM1	target
myricanone	PTGS1	target
myricanone	PTGS2	target
myricanone	RXRA	target
myricanone	ADRA1B	target
norswertianin	PTGS1	target
norswertianin	AR	target
norswertianin	PTGS2	target
tetrahydrofuroguaiacin B	ESR1	target

tetrahydrofuroguaiacin B	PTGS2	target
tetrahydrofuroguaiacin B	ADRA1B	target
narceine	PTGS2	target
narceine	KDR	target
permethrin	PTGS2	target
permethrin	DPP4	target
galbacin	PTGS2	target
galbacin	RXRA	target
cryptogenin	NR3C2	target
chebulic acid	PTGS2	target
