# Supplementary material for: Analysis of the Efficacy and Pharmacological Mechanisms of Action of Zhenren Yangzang Decoction on Ulcerative Colitis Using Meta-Analysis and Network Pharmacology
Source: Evid Based Complement Alternat Med. 2021 Dec 28;2021:4512755. doi: 10.1155/2021/4512755 (PMC8727130; doi:10.1155/2021/4512755)
Supplement: Supplementary Materials — Figure S1: Risk of bias graph. Figure S2: risk of bias summary. Figure S3: forest plot of comparison of serum cytokines. Figure S4: forest plot of comparison of the total syndrome score of TCM. Table S1: basic information on the active compounds in ZRYZD. Table S2: gene symbols and entrezID of active target genes. Table S3: compounds ranked by the degree in the network. Supplementary File 1: compounds of ZRYZD from TCMSP. Supplementary File 2: corresponding target genes of ZRYZD. Supplementary File 3: UC-related target genes. Supplementary File 4: GO functional enrichment analysis. Supplementary File 5: KEGG pathway enrichment analysis. Supplementary File 6: data of compound-target networks. Supplementary File 7: data of key compound-target networks. Supplementary File 8: data of PPI network. [file 4512755.f1.zip › 4512755.f1/Supplementary File 8 Data of PPI network (1).pdf]

Supplementary File 8 Data of PPI network

node1	node2
ACHE	PON1
ACHE	SLC6A4
ACHE	GSK3B
ACHE	DRD2
ACHE	PTGS2
ACHE	CASP3
ADM	CXCL8
ADM	VEGFA
ADRA1B	SLC6A4
ADRA2A	SLC6A4
ADRA2A	HTR3A
ADRA2A	PRKCB
ADRA2A	PRKCA
AR	MMP2
AR	MAPK14
AR	CCNB1
AR	CDK2
AR	TP53
AR	CCNA2
AR	BCL2L1
AR	PRKCB
AR	CXCL8
AR	CASP3
AR	GSK3B
AR	NR3C2
AR	CASP8
AR	PTGS2
AR	JUN
AR	MMP9
AR	IGF2
AR	CYP1A1
AR	GSTP1
AR	ESR1
AR	CDKN1A
AR	VEGFA
AR	RELA
AR	CDK1
BAX	CCNB1
BAX	CDK2
BAX	TP53
BAX	MMP9
BAX	PTGS2
BAX	VEGFA
BAX	GSK3B
BAX	CDKN1A
BAX	JUN
BAX	XIAP
BAX	CASP8
BAX	CASP9
BAX	CASP3
BAX	BCL2L1
BAX	BCL2
BCL2	CDK2
BCL2	TP53
BCL2	BAX
BCL2	BCL2L1
BCL2	CASP3
BCL2	CASP9
BCL2	CASP8
BCL2	XIAP

BCL2	RXRB
BCL2	CDK1
BCL2	RXRA
BCL2	ESR1
BCL2L1	NFKBIA
BCL2L1	MMP2
BCL2L1	MAPK14
BCL2L1	CCNB1
BCL2L1	KDR
BCL2L1	CDK2
BCL2L1	TP53
BCL2L1	CCNA2
BCL2L1	PPARG
BCL2L1	BAX
BCL2L1	AR
BCL2L1	CXCL8
BCL2L1	ESR1
BCL2L1	PTGS2
BCL2L1	MMP9
BCL2L1	CDKN1A
BCL2L1	VEGFA
BCL2L1	JUN
BCL2L1	CHEK1
BCL2L1	RELA
BCL2L1	GSK3B
BCL2L1	CDK1
BCL2L1	XIAP
BCL2L1	CASP3
BCL2L1	CASP9
BCL2L1	CASP8
BCL2L1	BCL2
CACNA1S	PRKCB
CACNA1S	RASGRF1
CACNA1S	PRKCA
CASP3	NFKBIA
CASP3	MMP2
CASP3	MAPK14
CASP3	CCNB1
CASP3	KDR
CASP3	CDK2
CASP3	TP53
CASP3	CCNA2
CASP3	PPARG
CASP3	BAX
CASP3	BCL2L1
CASP3	ACHE
CASP3	PRKCB
CASP3	CXCL8
CASP3	IGF2
CASP3	DPP4
CASP3	NOS2
CASP3	ESR2
CASP3	RELA
CASP3	CHEK1
CASP3	ESR1
CASP3	AR
CASP3	GSK3B
CASP3	PTGS2
CASP3	CDK1
CASP3	VEGFA
CASP3	MMP9
CASP3	BCL2
CASP3	JUN

CASP3	PRKCA
CASP3	CASP8
CASP3	CASP9
CASP3	CDKN1A
CASP3	XIAP
CASP8	NFKBIA
CASP8	MMP2
CASP8	MAPK14
CASP8	CCNB1
CASP8	CDK2
CASP8	TP53
CASP8	CCNA2
CASP8	PPARG
CASP8	BAX
CASP8	BCL2L1
CASP8	CXCL8
CASP8	CASP3
CASP8	GSK3B
CASP8	CASP9
CASP8	ESR2
CASP8	CHEK1
CASP8	ESR1
CASP8	PTGS2
CASP8	CDK1
CASP8	MMP9
CASP8	VEGFA
CASP8	CDKN1A
CASP8	JUN
CASP8	BCL2
CASP8	RELA
CASP8	XIAP
CASP8	AR
CASP9	NFKBIA
CASP9	MMP2
CASP9	MAPK14
CASP9	CCNB1
CASP9	CDK2
CASP9	TP53
CASP9	CCNA2
CASP9	PPARG
CASP9	BAX
CASP9	BCL2L1
CASP9	CXCL8
CASP9	CASP3
CASP9	GSK3B
CASP9	RELA
CASP9	CHEK1
CASP9	ESR1
CASP9	PTGS2
CASP9	BCL2
CASP9	VEGFA
CASP9	CDKN1A
CASP9	MMP9
CASP9	CDK1
CASP9	CASP8
CASP9	JUN
CASP9	XIAP
CCNA2	CCNB1
CCNA2	CDK2
CCNA2	TP53
CCNA2	MMP9
CCNA2	VEGFA
CCNA2	CASP8

CCNA2	JUN
CCNA2	ESR1
CCNA2	AR
CCNA2	XIAP
CCNA2	CASP9
CCNA2	BCL2L1
CCNA2	CASP3
CCNA2	CDK12
CCNA2	CHEK1
CCNA2	CDK1
CCNA2	CDKN1A
CCNB1	NFKBIA
CCNB1	MMP2
CCNB1	AR
CCNB1	PTGS2
CCNB1	BAX
CCNB1	VEGFA
CCNB1	MMP9
CCNB1	ESR1
CCNB1	RELA
CCNB1	JUN
CCNB1	CASP8
CCNB1	XIAP
CCNB1	BCL2L1
CCNB1	CDK12
CCNB1	CASP9
CCNB1	CASP3
CCNB1	TP53
CCNB1	RASGRF1
CCNB1	CHEK1
CCNB1	CCNA2
CCNB1	CDKN1A
CCNB1	CDK2
CCNB1	CDK1
CDK1	CCNB1
CDK1	CDK2
CDK1	TP53
CDK1	CCNA2
CDK1	BCL2L1
CDK1	CASP3
CDK1	CASP9
CDK1	CASP8
CDK1	XIAP
CDK1	JUN
CDK1	AR
CDK1	VEGFA
CDK1	ESR1
CDK1	BCL2
CDK1	CDK12
CDK1	RXRA
CDK1	RASGRF1
CDK1	CHEK1
CDK1	CDKN1A
CDK12	CCNB1
CDK12	CDK2
CDK12	TP53
CDK12	CCNA2
CDK12	CDK1
CDK12	CDKN1A
CDK2	NFKBIA
CDK2	MMP2
CDK2	CCNB1
CDK2	BAX

CDK2	PTGS2
CDK2	PPARG
CDK2	BCL2
CDK2	MMP9
CDK2	AR
CDK2	XIAP
CDK2	VEGFA
CDK2	CASP8
CDK2	CASP9
CDK2	ESR1
CDK2	BCL2L1
CDK2	JUN
CDK2	CASP3
CDK2	CDK12
CDK2	CHEK1
CDK2	CDK1
CDK2	CDKN1A
CDK2	CCNA2
CDK2	TP53
CDKN1A	NFKBIA
CDKN1A	MMP2
CDKN1A	MAPK14
CDKN1A	CCNB1
CDKN1A	CDK2
CDKN1A	TP53
CDKN1A	CCNA2
CDKN1A	PPARG
CDKN1A	BAX
CDKN1A	BCL2L1
CDKN1A	CXCL8
CDKN1A	CASP3
CDKN1A	GSK3B
CDKN1A	NOS2
CDKN1A	CASP9
CDKN1A	CASP8
CDKN1A	PTGS2
CDKN1A	XIAP
CDKN1A	JUN
CDKN1A	MMP9
CDKN1A	AR
CDKN1A	CDK1
CDKN1A	RELA
CDKN1A	VEGFA
CDKN1A	CHEK1
CDKN1A	CDK12
CDKN1A	ESR1
CHEK1	CCNB1
CHEK1	CDK2
CHEK1	TP53
CHEK1	CCNA2
CHEK1	BCL2L1
CHEK1	CASP3
CHEK1	CASP9
CHEK1	CASP8
CHEK1	XIAP
CHEK1	JUN
CHEK1	CDK1
CHEK1	RELA
CHEK1	CDKN1A
CHEK1	VEGFA
CHEK1	ESR1
CHEK1	RASGRF1
CXCL8	NFKBIA

CXCL8	MMP2
CXCL8	MAPK14
CXCL8	KDR
CXCL8	TP53
CXCL8	PPARG
CXCL8	BCL2L1
CXCL8	AR
CXCL8	XIAP
CXCL8	CYP1A1
CXCL8	ADM
CXCL8	NR1I2
CXCL8	CDKN1A
CXCL8	CASP9
CXCL8	PTGS1
CXCL8	ESR1
CXCL8	CASP8
CXCL8	NOS2
CXCL8	CASP3
CXCL8	MMP9
CXCL8	VEGFA
CXCL8	PTGS2
CXCL8	JUN
CXCL8	RELA
CYP1A1	PON1
CYP1A1	TP53
CYP1A1	PPARG
CYP1A1	CXCL8
CYP1A1	GSTM1
CYP1A1	GSTA1
CYP1A1	NR1I2
CYP1A1	CYP1A2
CYP1A1	ESR2
CYP1A1	PTGS2
CYP1A1	AR
CYP1A1	ESR1
CYP1A1	GSTA2
CYP1A1	GSTP1
CYP1A1	RXRA
CYP1A2	PON1
CYP1A2	SLC6A4
CYP1A2	GSTM1
CYP1A2	GSTA1
CYP1A2	NR1I2
CYP1A2	ESR1
CYP1A2	GSTA2
CYP1A2	CYP1A1
CYP1A2	GSTP1
DPP4	MMP2
DPP4	TP53
DPP4	PPARG
DPP4	CASP3
DPP4	MMP9
DPP4	VEGFA
DPP4	IGF2
DRD2	SLC6A4
DRD2	KDR
DRD2	ACHE
DRD2	HTR3A
DRD2	RXRB
ESR1	NFKBIA
ESR1	MMP2
ESR1	MAPK14
ESR1	CCNB1

ESR1	SLC6A4
ESR1	KDR
ESR1	CDK2
ESR1	TP53
ESR1	CCNA2
ESR1	BCL2L1
ESR1	CXCL8
ESR1	CASP3
ESR1	GSTM1
ESR1	GSK3B
ESR1	CASP9
ESR1	CYP1A2
ESR1	ESR2
ESR1	CASP8
ESR1	PTGS2
ESR1	XIAP
ESR1	JUN
ESR1	MMP9
ESR1	AR
ESR1	CYP1A1
ESR1	CDK1
ESR1	BCL2
ESR1	GSTP1
ESR1	RELA
ESR1	CDKN1A
ESR1	CHEK1
ESR1	IGF2
ESR1	PRKCA
ESR1	VEGFA
ESR2	MAPK14
ESR2	TP53
ESR2	CASP3
ESR2	GSTP1
ESR2	MMP9
ESR2	CASP8
ESR2	PTGS2
ESR2	VEGFA
ESR2	CYP1A1
ESR2	JUN
ESR2	ESR1
GSK3B	NFKBIA
GSK3B	MMP2
GSK3B	TP53
GSK3B	PPARG
GSK3B	BAX
GSK3B	BCL2L1
GSK3B	ACHE
GSK3B	PRKCB
GSK3B	CASP3
GSK3B	PRKCA
GSK3B	CASP8
GSK3B	CDKN1A
GSK3B	PTGS2
GSK3B	VEGFA
GSK3B	RELA
GSK3B	MMP9
GSK3B	CASP9
GSK3B	ESR1
GSK3B	XIAP
GSK3B	AR
GSK3B	JUN
GSTA1	GSTM1
GSTA1	JUN

GSTA1	NR1I2
GSTA1	GSTP1
GSTA1	CYP1A2
GSTA1	CYP1A1
GSTA1	GSTA2
GSTA2	GSTM1
GSTA2	GSTA1
GSTA2	NR1I2
GSTA2	CYP1A2
GSTA2	JUN
GSTA2	CYP1A1
GSTA2	GSTP1
GSTM1	PON1
GSTM1	TP53
GSTM1	ESR1
GSTM1	GSTP1
GSTM1	GSTA2
GSTM1	GSTA1
GSTM1	CYP1A2
GSTM1	CYP1A1
GSTP1	NFKBIA
GSTP1	PON1
GSTP1	TP53
GSTP1	GSTM1
GSTP1	GSTA1
GSTP1	CYP1A2
GSTP1	ESR2
GSTP1	PTGS2
GSTP1	JUN
GSTP1	AR
GSTP1	CYP1A1
GSTP1	ESR1
GSTP1	GSTA2
HTR3A	SLC6A4
HTR3A	ADRA2A
HTR3A	OPRM1
HTR3A	DRD2
IGF2	MMP2
IGF2	KDR
IGF2	TP53
IGF2	PPARG
IGF2	CASP3
IGF2	DPP4
IGF2	JUN
IGF2	MMP9
IGF2	AR
IGF2	ESR1
IGF2	RASGRF1
IGF2	VEGFA
JUN	NFKBIA
JUN	MMP2
JUN	MAPK14
JUN	CCNB1
JUN	KDR
JUN	CDK2
JUN	TP53
JUN	CCNA2
JUN	PPARG
JUN	BAX
JUN	BCL2L1
JUN	CXCL8
JUN	LPL
JUN	CASP3

JUN	GSK3B
JUN	NOS2
JUN	CASP9
JUN	GSTA1
JUN	NR1I2
JUN	ESR2
JUN	CASP8
JUN	PTGS2
JUN	XIAP
JUN	IGF2
JUN	CHEK1
JUN	PRKCA
JUN	CDK1
JUN	MMP9
JUN	GSTA2
JUN	AR
JUN	CDKN1A
JUN	VEGFA
JUN	GSTP1
JUN	RELA
JUN	ESR1
KDR	MMP2
KDR	MAPK14
KDR	BCL2L1
KDR	DRD2
KDR	PPARG
KDR	ESR1
KDR	PTGS2
KDR	JUN
KDR	CASP3
KDR	CXCL8
KDR	TP53
KDR	IGF2
KDR	MMP9
KDR	VEGFA
LPL	PON1
LPL	PPARG
LPL	RELA
LPL	RXRA
LPL	JUN
MAPK14	NFKBIA
MAPK14	NOS2
MAPK14	CASP9
MAPK14	MMP9
MAPK14	CDKN1A
MAPK14	PTGS2
MAPK14	PPARG
MAPK14	BCL2L1
MAPK14	CASP8
MAPK14	ESR2
MAPK14	KDR
MAPK14	AR
MAPK14	VEGFA
MAPK14	CXCL8
MAPK14	RELA
MAPK14	ESR1
MAPK14	CASP3
MAPK14	JUN
MAPK14	TP53
MMP2	NFKBIA
MMP2	DPP4
MMP2	GSK3B
MMP2	AR

MMP2	CDKN1A
MMP2	CCNB1
MMP2	CDK2
MMP2	CASP8
MMP2	PPARG
MMP2	CASP9
MMP2	BCL2L1
MMP2	ESR1
MMP2	KDR
MMP2	JUN
MMP2	PTGS2
MMP2	TP53
MMP2	CASP3
MMP2	CXCL8
MMP2	MMP9
MMP2	IGF2
MMP2	VEGFA
MMP9	NFKBIA
MMP9	MMP2
MMP9	MAPK14
MMP9	CCNB1
MMP9	KDR
MMP9	CDK2
MMP9	TP53
MMP9	CCNA2
MMP9	PPARG
MMP9	BAX
MMP9	BCL2L1
MMP9	CXCL8
MMP9	PRSS1
MMP9	CASP3
MMP9	GSK3B
MMP9	NOS2
MMP9	CASP9
MMP9	ESR2
MMP9	CASP8
MMP9	DPP4
MMP9	PTGS2
MMP9	XIAP
MMP9	JUN
MMP9	IGF2
MMP9	CDKN1A
MMP9	PRKCA
MMP9	AR
MMP9	ESR1
MMP9	RELA
MMP9	VEGFA
NFKBIA	CCNB1
NFKBIA	CDK2
NFKBIA	CDKN1A
NFKBIA	ESR1
NFKBIA	GSK3B
NFKBIA	MMP2
NFKBIA	VEGFA
NFKBIA	PPARG
NFKBIA	CASP9
NFKBIA	XIAP
NFKBIA	BCL2L1
NFKBIA	MMP9
NFKBIA	GSTP1
NFKBIA	CASP3
NFKBIA	CASP8
NFKBIA	PTGS2

NFKBIA	CXCL8
NFKBIA	RXRA
NFKBIA	TP53
NFKBIA	PRKCB
NFKBIA	PRKCA
NFKBIA	NOS2
NFKBIA	MAPK14
NFKBIA	JUN
NFKBIA	RELA
NOS2	NFKBIA
NOS2	MAPK14
NOS2	TP53
NOS2	PPARG
NOS2	CXCL8
NOS2	CASP3
NOS2	OPRM1
NOS2	PTGS1
NOS2	VEGFA
NOS2	MMP9
NOS2	PTGS2
NOS2	RXRA
NOS2	CDKN1A
NOS2	JUN
NOS2	RELA
NR1I2	TP53
NR1I2	CXCL8
NR1I2	GSTA1
NR1I2	JUN
NR1I2	VEGFA
NR1I2	GSTA2
NR1I2	RXRB
NR1I2	CYP1A1
NR1I2	CYP1A2
NR1I2	RXRA
NR3C2	SLC6A4
NR3C2	AR
OPRM1	SLC6A4
OPRM1	NOS2
OPRM1	HTR3A
PON1	GSTP1
PON1	CYP1A1
PON1	CYP1A2
PON1	PPARG
PON1	GSTM1
PON1	ACHE
PON1	LPL
PPARG	NFKBIA
PPARG	MMP2
PPARG	PON1
PPARG	MAPK14
PPARG	KDR
PPARG	CDK2
PPARG	TP53
PPARG	CYP1A1
PPARG	CASP8
PPARG	CASP9
PPARG	IGF2
PPARG	BCL2L1
PPARG	CDKN1A
PPARG	PTGS1
PPARG	NOS2
PPARG	PRKCA
PPARG	DPP4

PPARG	GSK3B
PPARG	CXCL8
PPARG	CASP3
PPARG	MMP9
PPARG	VEGFA
PPARG	PTGS2
PPARG	RXRB
PPARG	LPL
PPARG	JUN
PPARG	RXRA
PPARG	RELA
PRKCA	NFKBIA
PRKCA	TP53
PRKCA	ADRA2A
PRKCA	PPARG
PRKCA	PRKCB
PRKCA	CASP3
PRKCA	GSK3B
PRKCA	CACNA1S
PRKCA	PTGS2
PRKCA	JUN
PRKCA	MMP9
PRKCA	RXRB
PRKCA	RELA
PRKCA	ESR1
PRKCA	VEGFA
PRKCA	RXRA
PRKCB	NFKBIA
PRKCB	ADRA2A
PRKCB	VEGFA
PRKCB	AR
PRKCB	CACNA1S
PRKCB	GSK3B
PRKCB	PRKCA
PRKCB	CASP3
PRKCB	RELA
PRSS1	MMP9
PTGS1	PPARG
PTGS1	CXCL8
PTGS1	NOS2
PTGS1	VEGFA
PTGS1	PTGS2
PTGS2	NFKBIA
PTGS2	MMP2
PTGS2	MAPK14
PTGS2	CCNB1
PTGS2	KDR
PTGS2	CDK2
PTGS2	TP53
PTGS2	PPARG
PTGS2	BAX
PTGS2	BCL2L1
PTGS2	ACHE
PTGS2	CXCL8
PTGS2	CASP3
PTGS2	GSK3B
PTGS2	NOS2
PTGS2	CASP9
PTGS2	ESR2
PTGS2	CASP8
PTGS2	PTGS1
PTGS2	PRKCA
PTGS2	AR

PTGS2	XIAP
PTGS2	CDKN1A
PTGS2	CYP1A1
PTGS2	GSTP1
PTGS2	RELA
PTGS2	ESR1
PTGS2	JUN
PTGS2	MMP9
PTGS2	VEGFA
RASGRF1	CCNB1
RASGRF1	CACNA1S
RASGRF1	CDK1
RASGRF1	CHEK1
RASGRF1	IGF2
RELA	NFKBIA
RELA	MAPK14
RELA	CCNB1
RELA	TP53
RELA	PPARG
RELA	BCL2L1
RELA	PRKCB
RELA	CXCL8
RELA	LPL
RELA	CASP3
RELA	GSK3B
RELA	NOS2
RELA	CASP9
RELA	CASP8
RELA	PTGS2
RELA	XIAP
RELA	JUN
RELA	MMP9
RELA	AR
RELA	CHEK1
RELA	VEGFA
RELA	CDKN1A
RELA	RXRA
RELA	PRKCA
RELA	ESR1
RXRA	NFKBIA
RXRA	PPARG
RXRA	LPL
RXRA	NOS2
RXRA	NR1I2
RXRA	RXRB
RXRA	CYP1A1
RXRA	CDK1
RXRA	BCL2
RXRA	RELA
RXRA	PRKCA
RXRB	PPARG
RXRB	NR1I2
RXRB	DRD2
RXRB	PRKCA
RXRB	BCL2
RXRB	RXRA
SLC6A4	NR3C2
SLC6A4	ESR1
SLC6A4	ACHE
SLC6A4	ADRA1B
SLC6A4	CYP1A2
SLC6A4	ADRA2A
SLC6A4	OPRM1

SLC6A4	HTR3A
SLC6A4	DRD2
TP53	NFKBIA
TP53	MMP2
TP53	MAPK14
TP53	CCNB1
TP53	KDR
TP53	CDK2
TP53	NOS2
TP53	CDK12
TP53	GSTM1
TP53	CYP1A1
TP53	PRKCA
TP53	IGF2
TP53	GSTP1
TP53	CXCL8
TP53	MMP9
TP53	XIAP
TP53	NR1I2
TP53	VEGFA
TP53	CASP9
TP53	AR
TP53	ESR2
TP53	PPARG
TP53	CASP3
TP53	DPP4
TP53	JUN
TP53	CDK1
TP53	CASP8
TP53	RELA
TP53	BAX
TP53	PTGS2
TP53	ESR1
TP53	BCL2
TP53	CCNA2
TP53	CHEK1
TP53	GSK3B
TP53	BCL2L1
TP53	CDKN1A
VEGFA	NFKBIA
VEGFA	MMP2
VEGFA	MAPK14
VEGFA	CCNB1
VEGFA	KDR
VEGFA	CDK2
VEGFA	TP53
VEGFA	CCNA2
VEGFA	PPARG
VEGFA	BAX
VEGFA	BCL2L1
VEGFA	PRKCB
VEGFA	CXCL8
VEGFA	CASP3
VEGFA	GSK3B
VEGFA	NOS2
VEGFA	CASP9
VEGFA	NR1I2
VEGFA	ESR2
VEGFA	CASP8
VEGFA	DPP4
VEGFA	PTGS1
VEGFA	PTGS2
VEGFA	XIAP

VEGFA	JUN
VEGFA	MMP9
VEGFA	AR
VEGFA	CDK1
VEGFA	RELA
VEGFA	CDKN1A
VEGFA	CHEK1
VEGFA	IGF2
VEGFA	ESR1
VEGFA	PRKCA
VEGFA	ADM
XIAP	NFKBIA
XIAP	CCNB1
XIAP	CDK2
XIAP	TP53
XIAP	CCNA2
XIAP	BAX
XIAP	BCL2L1
XIAP	CXCL8
XIAP	CASP3
XIAP	GSK3B
XIAP	CASP9
XIAP	CASP8
XIAP	PTGS2
XIAP	ESR1
XIAP	CDKN1A
XIAP	MMP9
XIAP	BCL2
XIAP	VEGFA
XIAP	JUN
XIAP	RELA
XIAP	CDK1
XIAP	CHEK1
