# Supplementary material for: Analysis of the Efficacy and Pharmacological Mechanisms of Action of Zhenren Yangzang Decoction on Ulcerative Colitis Using Meta-Analysis and Network Pharmacology
Source: Evid Based Complement Alternat Med. 2021 Dec 28;2021:4512755. doi: 10.1155/2021/4512755 (PMC8727130; doi:10.1155/2021/4512755)
Supplement: Supplementary Materials — Figure S1: Risk of bias graph. Figure S2: risk of bias summary. Figure S3: forest plot of comparison of serum cytokines. Figure S4: forest plot of comparison of the total syndrome score of TCM. Table S1: basic information on the active compounds in ZRYZD. Table S2: gene symbols and entrezID of active target genes. Table S3: compounds ranked by the degree in the network. Supplementary File 1: compounds of ZRYZD from TCMSP. Supplementary File 2: corresponding target genes of ZRYZD. Supplementary File 3: UC-related target genes. Supplementary File 4: GO functional enrichment analysis. Supplementary File 5: KEGG pathway enrichment analysis. Supplementary File 6: data of compound-target networks. Supplementary File 7: data of key compound-target networks. Supplementary File 8: data of PPI network. [file 4512755.f1.zip › 4512755.f1/Supplementary Tables.pdf]

Table S1. Basic information on the active compounds in ZRYZD.

Herb	Compound ID	Compound Chemical Name	OB (%)	DL
PP	MOL006980	Papaverine	64.04	0.38
PP	MOL006982	Codeine	45.48	0.56
PP	MOL000787	Fumarine	59.26	0.83
PP	MOL009324	Cryptogenin	35.11	0.81
PP	MOL009327	Noskapin	40.66	0.88
PP	MOL009328	5-[[(1S)-6,7-dimethoxy-2-methyl-3,4-dihydro-1H-isoquinolin-1-yl]methyl]-2-methoxyphenol	51.55	0.37
PP	MOL009329	Narcein	48.18	0.64
PP	MOL009330	Noscapine	53.29	0.88
PP	MOL009331	Palaudine	68.27	0.34
PP	MOL009335	Erythroculine	63.36	0.53
PP	MOL009338	Norswertianin	92.14	0.22
SM<br>RAS<br>RPA	MOL000358	Beta-sitosterol	36.91	0.75
SM	MOL007920	Meso-1,4-Bis-(4-hydroxy-3-methoxyphenyl)-2,3-dimethylbutane	31.32	0.26
SM	MOL009243	Isoguaiacin	48.78	0.31
SM	MOL009254	Galbacin	61.00	0.53
SM	MOL009255	5-[(2S,3S)-7-methoxy-3-methyl-5-[(E)-prop-1-enyl]-2,3-dihydrobenzofuran-2-yl]-1,3-benzodioxole	53.11	0.40
SM	MOL009259	Kudos	45.06	0.38

SM	MOL009263	Saucernetindiol	41.85	0.32
SM	MOL009264	Tetrahydrofuroguaiacin B	62.86	0.32
SM	MOL009265	Threo-austrobailignan-5	49.49	0.32
FC	MOL001002	Ellagic acid	43.06	0.43
FC	MOL002276	Sennoside E_qt	50.69	0.61
FC	MOL006376	7-Dehydrosigmasterol	37.42	0.75
FC	MOL006826	Chebulic acid	72.00	0.32
FC	MOL009135	Ellipticine	30.82	0.28
FC	MOL009136	Peraksine	82.58	0.78
FC	MOL009137	(R)-(6-methoxy-4-quinolyl)-[(2R,4R,5S)-5-vinylquinuclidin-2-yl]methanol	55.88	0.40
FC	MOL009149	Cheilanthifoline	46.51	0.72
CC	MOL000131	EIC	41.90	0.14
CC	MOL000208	()-Aromadendrene	55.74	0.10
CC	MOL000266	Beta-Cubebene	32.81	0.11
CC	MOL002697	Junipene	44.07	0.11
CC	MOL003522	()-Sativene	37.41	0.10
CC	MOL003538	()-Ledene	51.84	0.10
CC	MOL002003	(-)-Caryophyllene oxide	32.67	0.13
CC	MOL000057	DIBP	49.63	0.13
CC	MOL000612	(-)-alpha-cedrene	55.56	0.10
CC	MOL000675	Oleic acid	33.13	0.14
RC	MOL001006	Poriferasta-7,22E-dien-3beta-ol	42.98	0.76
RC	MOL002140	Perlolyrine	65.95	0.27
RC	MOL002879	Diop	43.59	0.39
RC	MOL003036	ZINC03978781	43.83	0.76
RC<br>RAS	MOL000449	Stigmasterol	43.83	0.76

RA<br>RC<br>RG	MOL003896	7-Methoxy-2-methyl isoflavone	42.56	0.20
RC	MOL004355	Spinasterol	42.98	0.76
RC	MOL004492	Chrysanthemaxanthin	38.72	0.58
RC	MOL005321	Frutinone A	65.90	0.34
RC	MOL000006	Luteolin	36.16	0.25
RC	MOL006554	Taraxerol	38.40	0.77
RC	MOL006774	Stigmast-7-enol	37.42	0.75
RC	MOL007059	3-beta-Hydroxymethyllenetanshiquinone	32.16	0.41
RC	MOL007514	Methyl icosa-11,14-dienoate	39.67	0.23
RC	MOL008391	5alpha-Stigmastan-3,6-dione	33.12	0.79
RC	MOL008393	7-(beta-Xylosyl)cephalomannine_qt	38.33	0.29
RC	MOL008397	Daturilin	50.37	0.77
RC	MOL008400	Glycitein	50.48	0.24
RC	MOL008406	Spinoside A	39.97	0.40
RC	MOL008407	(8S,9S,10R,13R,14S,17R)-17-[(E,2R,5S)-5-ethyl-6-methylhept-3-en-2-yl]-10,13-dimethyl-1,2,4,7,8,9,11,12,14,15,16,17-dodecahydrocyclopenta[a] phenanthren-3-one	45.40	0.76
RC	MOL008411	11-Hydroxyrankinidine	40.00	0.66
RAM	MOL000020	12-senecioyl-2E,8E,10E-atractylentriol	62.40	0.22
RAM	MOL000021	14-acetyl-12-senecioyl-2E,8E,10E-atractylentriol	60.31	0.31
RAM	MOL000022	14-acetyl-12-senecioyl-	63.37	0.30

		2E,8Z,10E-atractylentriol		
RAM	MOL000028	α-Amyrin	39.51	0.76
RAM	MOL000033	(3S,8S,9S,10R,13R,14S,17R)-10,13-dimethyl-17-[(2R,5S)-5-propan-2-yloctan-2-yl]-2,3,4,7,8,9,11,12,14,15,16,17-dodecahydro-1H-cyclopenta[a]phenanthren-3-ol	36.23	0.78
RAM	MOL000049	3β-acetoxyatractylone	54.07	0.22
RAM	MOL000072	8β-ethoxy atractylenolide III	35.95	0.21
RPA	MOL001910	11alpha,12alpha-epoxy-3beta-23-dihydroxy-30-norolean-20-en-28,12beta-olide	64.77	0.38
RPA	MOL001918	Paeoniflorgenone	87.59	0.37
RPA	MOL001919	(3S,5R,8R,9R,10S,14S)-3,17-dihydroxy-4,4,8,10,14-pentamethyl-2,3,5,6,7,9-hexahydro-1H-cyclopenta[a]phenanthrene-15,16-dione	43.56	0.53
RPA	MOL001921	Lactiflorin	49.12	0.80
RPA	MOL001924	Paeoniflorin	53.87	0.79
RPA	MOL001925	Paeoniflorin_qt	68.18	0.40
RPA	MOL001928	Albiflorin_qt	66.64	0.33
RPA	MOL001930	Benzoyl paeoniflorin	31.27	0.75
RPA<br>RA<br>RG	MOL000211	Mairin	55.38	0.78
RPA<br>RA	MOL000359	Sitosterol	36.91	0.75

RG				
RPA<br>RG	MOL000422	Kaempferol	41.88	0.24
RPA	MOL000492	(+)-catechin	54.83	0.24
RAM	MOL010813	Benzo[a]carbazole	35.22	0.22
RAM	MOL010828	Cynaropicrin	67.50	0.38
RAM	MOL010839	Lappadilactone	38.56	0.73
RG	MOL001484	Inermine	75.18	0.54
RG	MOL001792	DFV	32.76	0.18
RG	MOL002311	Glycyrol	90.78	0.67
RG	MOL000239	Jaranol	50.83	0.29
RG	MOL002565	Medicarpin	49.22	0.34
RG	MOL000354	Isorhamnetin	49.60	0.31
RG	MOL003656	Lupiwighteone	51.64	0.37
RG	MOL000392	Formononetin	69.67	0.21
RG	MOL000417	Calycosin	47.75	0.24
RG	MOL004328	Naringenin	59.29	0.21
RG	MOL004805	(2S)-2-[4-hydroxy-3-(3-methylbut-2-enyl)phenyl]-8,8-dimethyl-2,3-dihydropyrano[2,3-f]chromen-4-one	31.79	0.72
RG	MOL004806	Euchrenone	30.29	0.57
RG	MOL004808	Glyasperin B	65.22	0.44
RG	MOL004810	Glyasperin F	75.84	0.54
RG	MOL004811	Glyasperin C	45.56	0.40
RG	MOL004814	Isotrifoliol	31.94	0.42
RG	MOL004815	(E)-1-(2,4-dihydroxyphenyl)-3-(2,2-dimethylchromen-6-yl)prop-2-en-1-one	39.62	0.35

RG	MOL004820	Kanzonols W	50.48	0.52
RG	MOL004824	(2S)-6-(2,4-dihydroxyphenyl)-2-(2-hydroxypropan-2-yl)-4-methoxy-2,3-dihydrofuro[3,2-g]chromen-7-one	60.25	0.63
RG	MOL004827	Semilicoisoflavone B	48.78	0.55
RG	MOL004828	Glepidotin A	44.72	0.35
RG	MOL004829	Glepidotin B	64.46	0.34
RG	MOL004833	Phaseolinisoflavan	32.01	0.45
RG	MOL004835	Glypallichalcone	61.60	0.19
RG	MOL004838	8-(6-hydroxy-2-benzofuranyl)-2,2-dimethyl-5-chromenol	58.44	0.38
RG	MOL004841	Licochalcone B	76.76	0.19
RG	MOL004848	Licochalcone G	49.25	0.32
RG	MOL004849	3-(2,4-dihydroxyphenyl)-8-(1,1-dimethylprop-2-enyl)-7-hydroxy-5-methoxy-coumarin	59.62	0.43
RG	MOL004855	Licoricone	63.58	0.47
RG	MOL004856	Gancaonin A	51.08	0.40
RG	MOL004857	Gancaonin B	48.79	0.45
RG	MOL004860	Licorice glycoside E	32.89	0.27
RG	MOL004863	3-(3,4-dihydroxyphenyl)-5,7-dihydroxy-8-(3-methylbut-2-enyl)chromone	66.37	0.41
RG	MOL004864	5,7-dihydroxy-3-(4-methoxyphenyl)-8-(3-methylbut-2-enyl)chromone	30.49	0.41
RG	MOL004866	2-(3,4-dihydroxyphenyl)-5,7-dihydroxy-6-(3-methylbut-2-enyl)chromone	44.15	0.41

RG	MOL004879	Glycyrin	52.61	0.47
RG	MOL004882	Licocoumarone	33.21	0.36
RG	MOL004883	Licoisoflavone	41.61	0.42
RG	MOL004884	Licoisoflavone B	38.93	0.55
RG	MOL004885	Licoisoflavanone	52.47	0.54
RG	MOL004891	Shinpterocarpin	80.3	0.73
RG	MOL004898	(E)-3-[3,4-dihydroxy-5-(3-methylbut-2-enyl)phenyl]-1-(2,4-dihydroxyphenyl)prop-2-en-1-one	46.27	0.31
RG	MOL004903	Liquiritin	65.69	0.74
RG	MOL004904	Licopyranocoumarin	80.36	0.65
RG	MOL004905	3,22-Dihydroxy-11-oxo-delta(12)-oleanene-27-alpha-methoxycarbonyl-29-oic acid	34.32	0.55
RG	MOL004907	Glyzaglabrin	61.07	0.35
RG	MOL004908	Glabridin	53.25	0.47
RG	MOL004910	Glabranin	52.90	0.31
RG	MOL004911	Glabrene	46.27	0.44
RG	MOL004912	Glabrone	52.51	0.50
RG	MOL004913	1,3-dihydroxy-9-methoxy-6-benzofurano[3,2-c]chromenone	48.14	0.43
RG	MOL004914	1,3-dihydroxy-8,9-dimethoxy-6-benzofurano[3,2-c]chromenone	62.90	0.53
RG	MOL004915	Eurycarpin A	43.28	0.37
RG	MOL004917	Glycyroside	37.25	0.79
RG	MOL004924	(-)-Medicocarpin	40.99	0.95
RG	MOL004935	Sigmoidin-B	34.88	0.41
RG	MOL004941	(2R)-7-hydroxy-2-	71.12	0.18

		(4-hydroxyphenyl)chroman-4-one		
RG	MOL004945	(2S)-7-hydroxy-2-(4-hydroxyphenyl)-8-(3-methylbut-2-enyl)chroman-4-one	36.57	0.32
RG	MOL004948	Isoglycyrol	44.70	0.84
RG	MOL004949	Isolicoflavonol	45.17	0.42
RG	MOL004957	HMO	38.37	0.21
RG	MOL004959	1-Methoxyphaseollidin	69.98	0.64
RG	MOL004961	Quercetin der.	46.45	0.33
RG	MOL004966	3'-Hydroxy-4'-O-Methylglabridin	43.71	0.57
RG	MOL000497	Licochalcone a	40.79	0.29
RG	MOL004974	3'-Methoxyglabridin	46.16	0.57
RG	MOL004978	2-[(3R)-8,8-dimethyl-3,4-dihydro-2H-pyrano[6,5-f]chromen-3-yl]-5-methoxyphenol	36.21	0.52
RG	MOL004980	Inflacoumarin A	39.71	0.33
RG	MOL004985	Icos-5-enoic acid	30.70	0.20
RG	MOL004988	Kanzonol F	32.47	0.89
RG	MOL004989	6-prenylated eriodictyol	39.22	0.41
RG	MOL004990	7,2',4'-trihydroxy-5-methoxy-3-arylcoumarin	83.71	0.27
RG	MOL004991	7-Acetoxy-2-methylisoflavone	38.92	0.26
RG	MOL004993	8-prenylated eriodictyol	53.79	0.40
RG	MOL004996	Gadelaidic acid	30.70	0.20
RG	MOL000500	Vestitol	74.66	0.21
RG	MOL005000	Gancaonin G	60.44	0.39
RG	MOL005001	Gancaonin H	50.10	0.78
RG	MOL005003	Licoagrocarpin	58.81	0.58
RG	MOL005007	Glyasperins M	72.67	0.59
RG	MOL005008	Glycyrrhiza flavonol A	41.28	0.60

RG	MOL005012	Licoagroisoflavone	57.28	0.49
RG	MOL005013	18α-hydroxyglycyrrhetic acid	41.16	0.71
RG	MOL005016	Odoratin	49.95	0.30
RG	MOL005017	Phaseol	78.77	0.58
RG	MOL005018	Xambioona	54.85	0.87
RG	MOL005020	Dehydroglyasperins C	53.82	0.37
RG	MOL000098	Quercetin	46.43	0.28

ZRYZD: Zhenren Yangzang decoction, OB: oral bioavailability, DL: drug-likeness, PP: *Pericarpium Papaveris*, SM: *Semen Myristicae*, FC: *Fructus Chebulae*, CC: *Cortex Cinnamomi*, RC: *Radix Codonopsis*, RAM: *Rhizoma Atractylodis Macrocephalae*, RAS: *Radix Angelicae Sinensis*, RPA: *Radix Paeoniae Alba* , RA: *Radix Aucklandiaeand*, RG: *Radix Glycyrrhizae*

Table S2. Gene symbols and entrezID of active target genes.

Gene symbol	EntrezID	Gene symbol	EntrezID	Gene symbol	EntrezID
NR3C2	4306	CDKN1A	1026	CAV1	857
TNFAIP6	7130	MMP2	4313	MYC	4609
IL6R	3570	MMP9	4318	F3	2152
CD14	929	MAPK1	5594	GJA1	2697
LBP	3929	IL10RA	3587	IL1B	3553
PTGS1	5742	RB1	5925	CCL2	6347
PTGS2	5743	CDK4	1019	PTGER3	5733
ADRA1B	147	TP53	7157	CXCL8	3576
SLC6A4	6532	NFKBIA	4792	PRKCB	5579
OPRM1	4988	TOP1	7150	DUOX2	50506
BCL2	596	MDM2	4193	NOS3	4846
BAX	581	PCNA	5111	HSPB1	3315
CASP9	842	ERBB2	2064	THBD	7056
JUN	3725	CASP7	840	SERPINE1	5054
CASP3	836	MCL1	4170	COL1A1	1277
CASP8	841	BIRC5	332	IL1A	3552
PRKCA	5578	IL2RA	3559	MPO	4353
PON1	5444	CCNB1	891	ABCG2	9429
NOS2	4843	TYR	7299	NFE2L2	4780
AR	367	IFNG	3458	NQO1	1728
PPARG	5468	IL4	3565	PARP1	142
DPP4	1803	TOP2A	7153	CXCL11	6373
PRSS1	5644	XIAP	331	CXCL2	2920
ACHE	43	CD40LG	959	CHEK2	11200
RELA	5970	PTGES	9536	CLDN4	1364
IKBKB	3551	MET	4233	CXCL10	3627
AKT1	207	CA2	760	CHUK	1147

MAPK8	5599	NR3C1	2908	SPP1	6696
MMP1	4312	MMP13	4322	RUNX2	860
STAT1	6772	MMP8	4317	RASSF1	11186
CDK1	983	HTR3A	3359	E2F1	1869
HMOX1	3162	KDR	3791	CTSD	1509
CYP3A4	1576	MAPK10	5602	IGFBP3	3486
CYP1A2	1544	PPARD	5467	IGF2	3481
CYP1A1	1543	NCF1	653361	IRF1	3659
ICAM1	3383	MAPK3	5595	ERBB3	2065
SELE	6401	FASN	2194	PCOLCE	5118
VCAM1	7412	BAD	572	NPEPPS	9520
NR1I2	8856	SOD1	6647	HK2	3099
CYP1B1	1545	APOB	338	GSTA1	2938
ALOX5	240	PLB1	151056	GSTA2	2939
GSTP1	2950	HMGCR	3156	RASGRF1	5923
AHR	196	CYP19A1	1588	CDK12	51755
GSTM1	2944	UGT1A1	54658	DRD2	1813
AKR1C3	8644	PPARA	5465	TRPV1	7442
SLPI	6590	GSR	2936	ADH1B	125
ESR1	2099	ABCC1	4363	TEP1	7011
DPEP1	1800	AKR1C1	1645	EDN1	1906
RXRA	6256	GOT1	2805	LPL	4023
CAT	847	CES1	1066	BDNF	627
ADRA2A	150	SOAT1	6646	INS	3630
AKR1B1	231	RXRB	6257	SERPINB2	5055
PLAU	5328	STAT3	6774	FABP1	2168
ESR2	2100	FOSL2	2355	GCG	2641
MAPK14	1432	MMP3	4314	ENPEP	2028
GSK3B	2932	FOS	2353	UCP2	7351

CDK2	1017	EGF	1950	SCD	6319
CHEK1	1111	ODC1	4953	PYY	5697
CCNA2	890	RAF1	5894	ADM	133
EGFR	1956	HIF1A	3091	HRH1	3269
VEGFA	7422	RUNX1T1	862	CACNA1S	779
CCND1	595	HSPA5	3309	ACACA	31
BCL2L1	598				

Table S3. Compounds ranked by degree in the network

Compound ID	Degree	Drug	Compound ID	Degree	Drug
MOL000098	110	RG	MOL002311	9	RG
MOL000006	47	RC	MOL000787	8	PP
MOL000422	36	RAM<br>RG	MOL006982	8	PP
MOL000675	28	CC	MOL006980	8	PP
MOL004328	28	RG	MOL004913	8	RG
MOL000497	22	RG	MOL004898	8	RG
MOL000392	20	RG	MOL004866	8	RG
MOL000354	19	RG	MOL004855	8	RG
MOL003896	19	RC<br>RG	MOL004805	8	RG
MOL009135	18	FC	MOL000449	8	RAS<br>RC<br>RA
MOL000500	17	RG	MOL009149	7	FC
MOL004978	17	RG	MOL004980	7	RG
MOL004891	17	RG	MOL004914	7	RG
MOL001002	16	FC	MOL001484	7	RG
MOL005003	16	RG	MOL007059	7	RC
MOL004974	16	RG	MOL005321	7	RC
MOL004957	16	RG	MOL009330	6	PP
MOL004912	16	RG	MOL009136	6	FC
MOL004908	16	RG	MOL005001	6	RG
MOL008400	16	RC	MOL004948	6	RG
MOL005016	15	RG	MOL004945	6	RG
MOL004991	15	RG	MOL004941	6	RG
MOL004966	15	RG	MOL004882	6	RG

MOL004959	15	RG	MOL001792	6	RG
MOL004835	15	RG	MOL000049	6	RAM
MOL004833	15	RG	MOL000492	6	RPA
MOL004828	15	RG	MOL009327	5	PP
MOL004824	15	RG	MOL009263	5	SM
MOL004815	15	RG	MOL004829	5	RG
MOL004811	15	RG	MOL009335	4	PP
MOL000417	15	RG	MOL009331	4	PP
MOL009243	14	SM	MOL000057	4	CC
MOL005007	14	RG	MOL002003	4	CC
MOL004907	14	RG	MOL000131	4	CC
MOL004820	14	RG	MOL005018	4	RG
MOL002565	14	RG	MOL004988	4	RG
MOL009265	13	SM	MOL004910	4	RG
MOL005012	13	RG	MOL004838	4	RG
MOL004915	13	RG	MOL004806	4	RG
MOL004864	13	RG	MOL001924	4	RPA
MOL004857	13	RG	MOL009338	3	PP
MOL004849	13	RG	MOL000612	3	CC
MOL004841	13	RG	MOL009264	3	SM
MOL004808	13	RG	MOL004989	3	RG
MOL003656	13	RG	MOL004935	3	RG
MOL000358	13	RPA<br>RAS<br>SM	MOL004903	3	RG
MOL005000	12	RG	MOL008411	3	RC
MOL004990	12	RG	MOL009329	2	PP
MOL004961	12	RG	MOL003522	2	CC
MOL004911	12	RG	MOL009259	2	SM

MOL004885	12	RG	MOL009254	2	SM
MOL004884	12	RG	MOL010813	2	RA
MOL004883	12	RG	MOL004993	2	RG
MOL004856	12	RG	MOL004924	2	RG
MOL004810	12	RG	MOL002140	2	RC
MOL005020	11	RG	MOL009324	1	PP
MOL005008	11	RG	MOL002697	1	CC
MOL004863	11	RG	MOL000266	1	CC
MOL004848	11	RG	MOL010828	1	RA
MOL009328	10	PP	MOL006826	1	FC
MOL009255	10	SM	MOL008407	1	RC
MOL009137	10	FC	MOL008397	1	RC
MOL005017	10	RG	MOL004355	1	RC
MOL004904	10	RG	MOL003036	1	RC
MOL004879	10	RG	MOL001006	1	RC
MOL004827	10	RG	MOL000072	1	RAM
MOL004814	10	RG	MOL000022	1	RAM
MOL004949	9	RG	MOL000359	1	RPA<br>RG<br>RA
MOL000239	9	RG	MOL001919	1	RPA

PP: *Pericarpium Papaveris*, SM: *Semen Myristicae*, FC: *Fructus Chebulae*, CC: *Cortex Cinnamomi*, RC: *Radix Codonopsis*, RAM: *Rhizoma Atractylodis Macrocephalae*, RAS: *Radix Angelicae Sinensis*, RPA: *Radix Paeoniae Alba* , RA: *Radix Aucklandiaeand*, RG: *Radix Glycyrrhizae*
